# *In-Silico* Drug discovery approach targeting receptor tyrosine kinase-like orphan receptor 1 for cancer treatment

**DOI:** 10.1038/s41598-017-01254-w

**Published:** 2017-04-21

**Authors:** Onkar Nath, Archana Singh, Indrakant K. Singh

**Affiliations:** 1 Jawaharlal Nehru University, SCIS, New Delhi, 110067 India; 2 Department of Botany, Hans Raj College, University of Delhi, Delhi, 110007 India; 3 Molecular Biology Research Lab, Department of Zoology, Deshbandhu College, University of Delhi, Kalkaji, New Delhi 110019 India; 4 Department of Entomology, University of Kentucky, S-225 AG. Science - North, lexington, KY 40546-0091 United States

## Abstract

Receptor tyrosine kinases (RTK) are important cell signaling molecules that influence many cellular processes. Receptor tyrosine kinase such as orphan receptor 1 (Ror1), a surface antigen, is a member of the RTK family of Ror, which plays a crucial role in cancers that have high-grade histology. As Ror1 has been implicated to be a potential target for cancer therapy, we selected this protein for further investigation. The secondary and tertiary structure of this protein was determined, which revealed that this protein contained three β-sheets, seven α-helices, and coils. The prediction of the active site revealed its cage-like function that opens for ligand entry and then closes for interacting with the ligands. Optimized ligands from the database were virtually screened to obtain the most efficient and potent ones. The screened ligands were evaluated for their therapeutic usefulness. Furthermore, the ligands that passed the test were docked to the target protein resulting in a few ligands with high score, which were analyzed further. The highest scoring ligand, Beta-1, 2,3,4,6-Penta-O-Galloyl-D-Glucopyranose was reported to be a naturally occurring tannin. This *in silico* approach indicates the potential of this molecule for advancing a further step in cancer treatment.

## Introduction

Ror1, a member of RTK family, is an orphan-receptor tyrosine-kinase-like surface antigen, which is primarily expressed during the early stages of embryogenesis. Ror1 is evolutionarily conserved among different species^1–5^. Mutations in human Ror2 have been implicated in certain congenital skeletal defects including shortened or missing digits and a form of short-limbed dwarfism^6–8^ but Ror1 mutations have not been reported in any human disease. During mouse development, Ror1 protein is known to play an essential role^9^. Ror1 protein possess an extracellular immunoglobulin-like domain, a cysteine-rich Frizzled domain, and a membrane-proximal Kringle domain. In addition, Ror1 also possesses an intracellular portion with tyrosine kinase domain, two serine/threonine-rich domains and a proline-rich domain^1, 2, 10^. Notably, Ror1 lacks several key amino acids, shedding doubt on the actual enzymatic function of it^11^. Biochemical assays revealed that Ror1 is a pseudo-kinase that is devoid of catalytic activity^11^. Wnt5a acts as a potential ligand for Ror1 and Ror2^2, 12, 13^ and interaction between Ror1 and Ror2 is required for Wnt5a signaling, which promotes leukemia chemotaxis and proliferation^14^.

Although, Ror1 does not express itself virtually in all normal adult tissues, it re-expresses in many tissues during some B-cell malignancies, and various cancer cell lines^6, 8, 15, 16^. Ror1 was significantly more expressed in various tumours such as acute lymphocytic leukemia, renal carcinoma, breast cancer, lung cancer, adenocarcinoma and melanoma^16–21^. Ror1 has also been recognized as potential biomarker for lung adenocarcinoma^22^. Recent studies have reported the presence of natural humoral and cellular immunity against Ror1 in chronic lymphocytic leukemia (CLL) patients^23^ and expression of high levels of Ror1 may promote cancer cell activation and survival enhancing disease progression in patients suffering from CLL^24^. Moreover, Ror1 has also been suggested to be associated with epithelial-to-mesenchymal transition (EMT) during embryogenesis and in cancer metastasis, maintaining the undifferentiated features of stem cells^25, 26^. Some patients treated with vaccines of autologous leukemia cells genetically engineered to promote anti-leukemia immune responses generated auto-antibodies specific for Ror1 that did not react with non-tumor tissues showing that this receptor is specific to cancer cells^6^. These receptors may enhance chemoresistance, and its knockdown may sensitize these cells to cisplatin^27^. Primary cancers in which Ror1 is up-regulated, high levels of phosphorylated AKT/PKB (a serine/threonine-specific protein kinase B) (p-AKT) and phosphorylated cAMP response element binding-factor (p-CREB) are also expressed^28^. The association between Ror1 with activated AKT has also been reported^21^, which suggests that Ror1 could associate with epidermal growth factor receptor, thereby, enhancing signaling in response to relevant ligands. Ror1 could enhance the survival of tumor cells by either kinase-dependent or kinase-independent pathways.

Many studies support the notion that Ror1 plays a functional role in promoting tumor cell growth and suggest that it may be a potential target for diagnosis and development of therapies against a variety of different human cancers^10, 28^. In this study, we have investigated a truncated Ror1 (‘t-Ror1’), as not much information regarding this isoform of Ror1 (a 2373 bp transcript encoding 388 aa) is available. ‘t-Ror1’ is identical with the cytosolic, C-terminal region of Ror1 but lacks the transmembrane and the entire extracellular domain. It has been demonstrated that its mRNA level is up-regulated in fetal and adult human CNS, in human leukemia, lymphoma cell lines, and in a variety of human cancers derived from neuroectoderm^29^. Our study provides clues to a potential ligand, Beta-1,2,3,4,6-Penta-O-Galloyl-D-Glucopyranose, which is a naturally occurring tannin, and can inhibit the activity of Ror1. We also studied the dynamics of its structure, which helps in understanding the nature of the molecule. The major role of this protein is in cancer development and proliferation, and its role as a gateway for cancer indicates that the ligands can prove to be a remedy for the disease.

## Results

### Secondary and tertiary structure analysis

**Figure 1:**
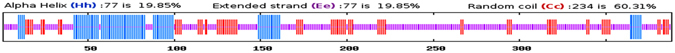
Secondary structure analysis of the Ror1 protein.

**Figure 2:**
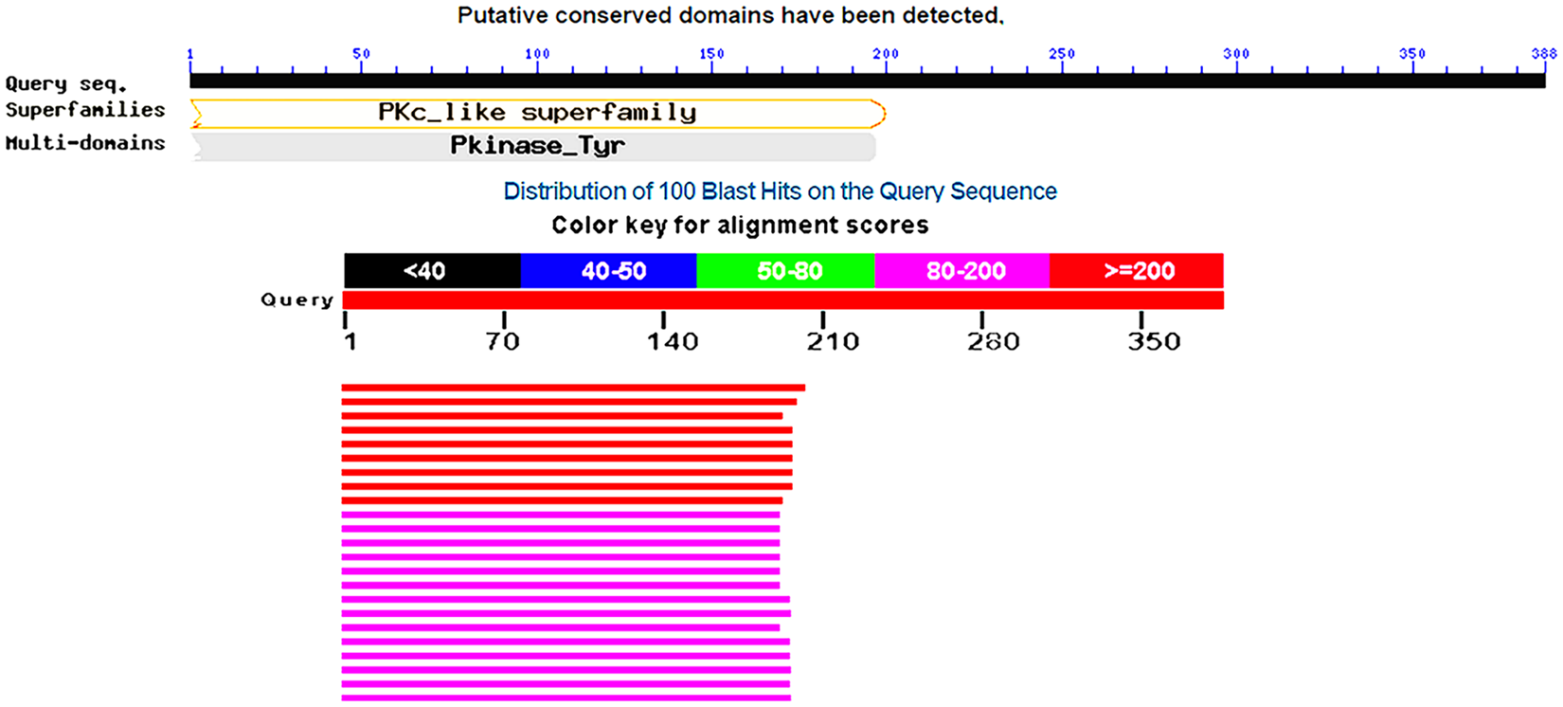
Template search using BLASTp tool.

The Ror1 protein of *Homo sapiens* is a 388 amino acid long sequence, which was retrieved from the NCBI protein database (https://www.ncbi.nlm.nih.gov/protein). The GenBank ID for Ror1 is AAC50714.1^29^ listed as tyrosine kinase t-Ror1. The secondary structure of the protein was analyzed by PROMOTIF tool^30^. The sequence was predicted to contain four strands, seven alpha helices and two 3_10_ helices. Disulphide bridges were found between cysteine 164 and cysteine 168. The secondary structure predicted by the GOR4^31^ method contained 77 amino acids in helix and 77 in sheet regions (as shown in Fig. 1); most of the regions are predicted to form coils. Presence of a higher percentage of coils in a protein indicates a flexible structure, which is key to molecular interactions with substrates and ligands. Due to unavailability of the 3D structure of the target protein on RCSB PDB^32^ and SCOP^33^ databases, the 3D structure of this protein was predicted from the primary sequence. In order to acquire a more accurate structure, different tools including CPHModel^34^, phyre2^35^, ps2v2^36^, RaptorX^37^, Modeller^38^ and I-Tasser^39^ were used for structure prediction^40^. A search for template (homologous protein sequences with known structures) resulted in sequences that covered only, approximately, 50% of initial region of the sequence (Fig. 2).

**Figure 3:**
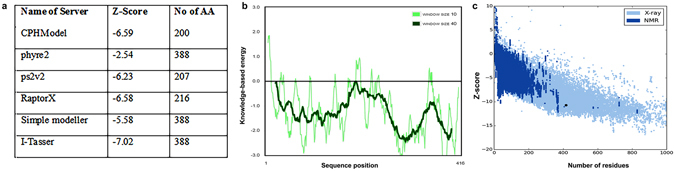
(**a**) Z-score and number of amino acids indicating accuracy and completeness of predicted structure (**b**) Z-Score plot of model predicted by I-Tasser with structures available in database (**c**), Energy plot for amino acid residues for the 3D-structure of I-Tasser.

Thus, it was necessary to check the accuracy of the predicted structures by comparing them on the basis of different parameters. Z-Score is considered as a standard parameter for quality assessment. To check the quality of the predicted structures, Prosa Server^41, 42^ was used (see Fig. 3a–c). As the structure predicted by I-Tasser^39, 43, 44^ had the lowest Z-score and it also modelled the most complete structure this was considered as the best predicted 3D-structure. The SAVES server^45^ was used to analyze other parameters of the protein 3D-Structure and predicted that 97.16% of the residues had an averaged 3D-1D score greater than or equal to 0.2 (For detail overview, see Supplementary Fig. 1a and b). As indicated in Supplementary Fig. S1b, Procheck^46^ analysis predicted 67.4% of the residues to be in the most favorable region whereas 27.3% were in additionally allowed, 3.8% were in generously allowed and 1.6% (i.e. 5 amino acid residues) were in disallowed regions. The RMSD value was predicted to be 1.5 angstroms from the native structure.

**Figure 4:**
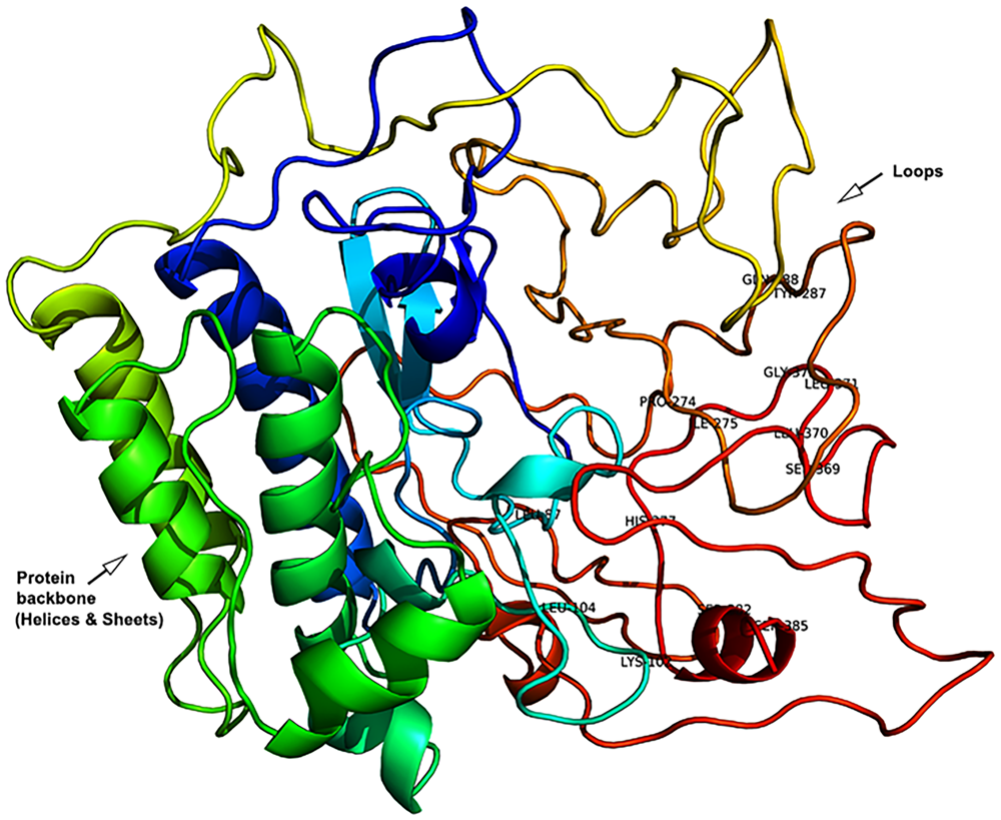
3D-Structure of Ror1 protein and its actives sites.

Before using this structure for further analysis, it was prepared and optimized using Schrodinger’s Protein Preparation wizard^47^ (shown in Fig. 4). The structure was found to be stable, and no unstable or sterically disallowed regions were noted except the steric clash between OD2 (ASP 66) and OH (TYR 96), which is due to their location in an intersecting field i.e. at a distance less than the allowed.

### Domain and Active Site Prediction

**Figure 5:**
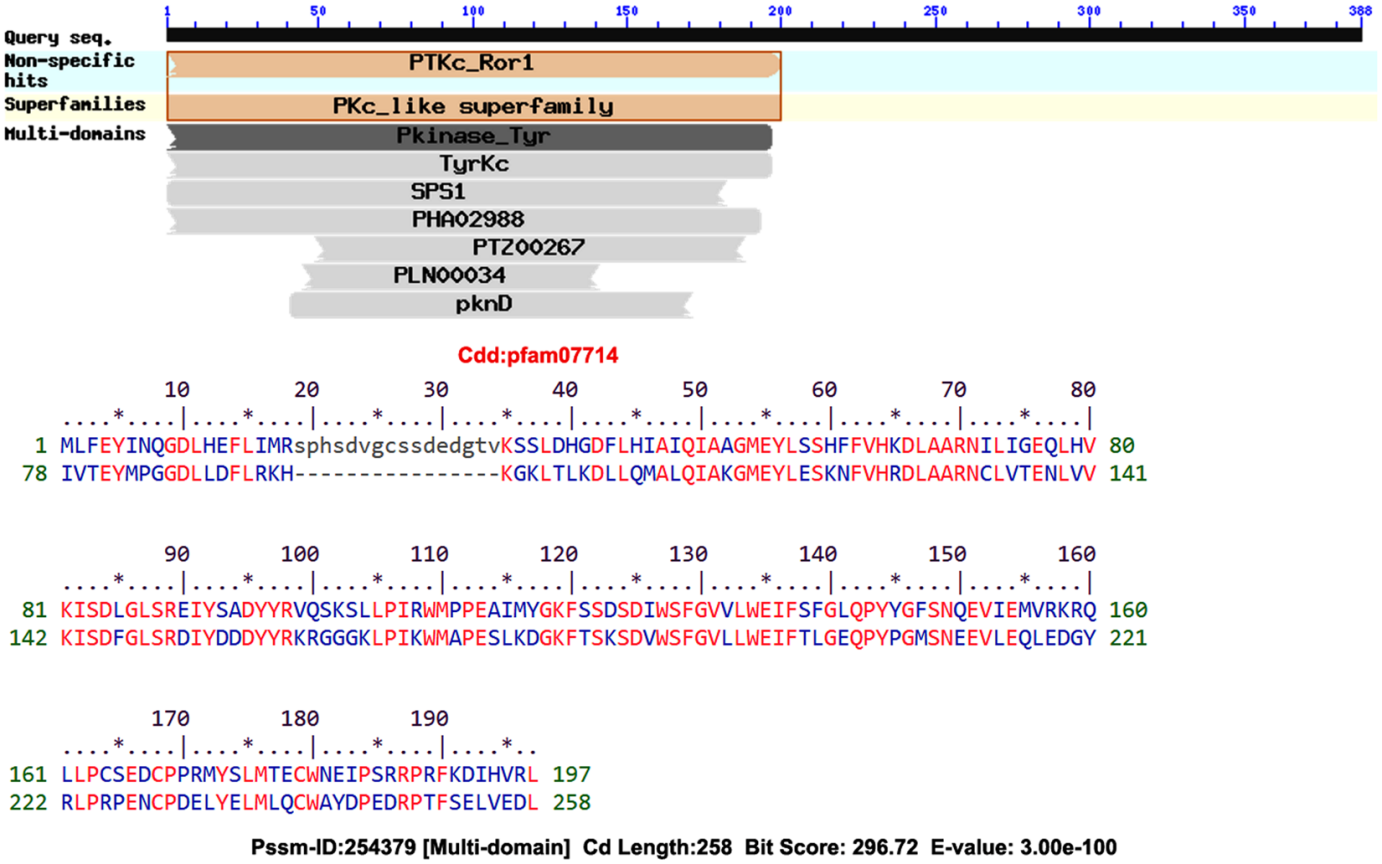
Domain Analysis by CD Search indicating similarity to tyrosine kinase domain.

Other researchers have also analyzed this protein and some of the domains of this protein have been deciphered. However, in order to find additional domains CDSearch was performed^48^. Domain analysis showed that the target protein belongs to a Pkc_like superfamily as shown in Fig. 5 It can be seen that only the N-terminal part of the sequence shows similarity with other conserved domains. The E-value of the domains indicated that only partial or modified regions of the domains are present. These results suggest that the domains present are modified and thus, may not play the expected functional role. The active site of the protein is not known thus it is necessary to predict the active sites of the protein for ligand docking.

The Active Sites of the protein were predicted using DogSiteScorer^49^ and Schrodinger’s siteprep^50, 51^. DogSiteScorer predicts the druggability of the active sites thus predicting confidence on sites. In this calculation, the largest site contained the first three active site residues/regions predicted by DogSiteScorer. Due to higher druggability scores of this site (shown in Table 1), it was definitely considered as the only active site. Thus, the total volume of the active site was predicted to be 2325 cubic angstroms and the surface area to be 2703 cubic angstroms.

**Table 1:** Active Site analysis of the Protein Ror1 using DogSiteScorer.

Name	Volume [Å³]	Surface [Å²]	Lipo surface [Å²]	Depth [Å]	Drug Score
P0	870.66	1010.30	701.62	23.83	0.84
P1	834.24	1092.71	801.51	16.42	0.80
P2	620.35	600.22	427.95	17.60	0.80
P3	584.77	570.70	329.67	14.97	0.74
P4	399.36	630.28	374.36	17.24	0.71
P5	225.86	381.32	263.09	8.65	0.37
P6	175.81	338.94	188.84	10.70	0.37
P7	140.54	371.06	253.04	6.81	0.19
P10	105.15	248.34	204.86	7.61	0.19
P8	127.81	192.05	125.80	6.07	0.15
P9	107.52	289.59	191.98	6.45	0.14

### Ligand Preparation

Prior to docking, a search for a molecule previously reported against Ror1 was carried out in the drug databases. No chemical molecules are known as its possible inhibitor, however, a natural molecule Wnt5a has been reported to be a possible inhibitor of Ror1 and Ror2. For docking, ligands were downloaded from the ZINC database^52^. To reduce the computational intensity and to maintain the accuracy of the prediction, the ligands were screened to obtain a single conformation for all ligands with the same scaffold. Then these ligands were prepared and optimized using LigPrep tool^53^. The optimized 3D structure of ligand molecules was obtained from LigPrep and 45,000 unique ligands were obtained.

### Protein-Ligand Docking

**Figure 6:**
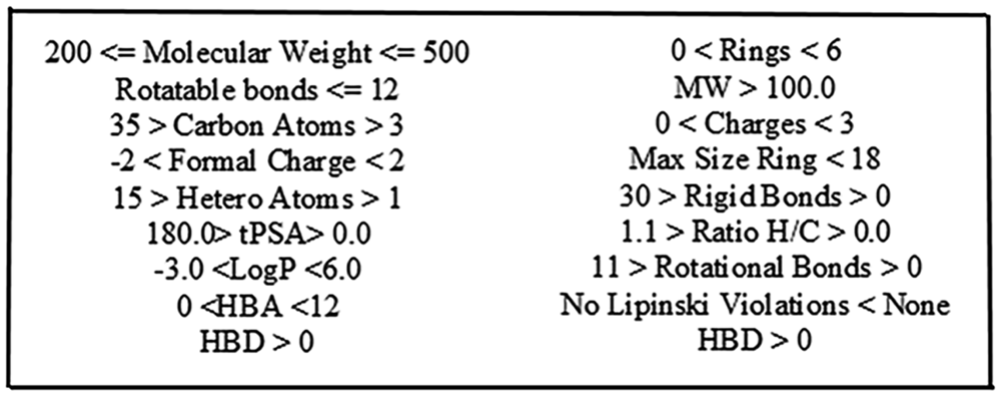
Parameters used to screen ligands based on drug likeliness.

It is computationally very intensive to dock a ligand library as large as ours and thus, before going for intensive docking the ligands were screened using high throughput technique to obtain the ligands that are best to carry forward. These ligands were initially screened using the High Throughput Virtual Screening (HTVS) module of Schrodinger^54^. The screening was conducted in flexible docking mode in order to analyze all the possible conformations of the ligands. This will remedy the initial cut down performed to reduce the database size. The results of this screening were obtained by selecting a cut off docking score value of −7 kcal/mol. These ligands were analyzed for druggability parameters (shown in Fig. 6) using Mobyl Server (FAF-Drugs3@rpbs a Free ADME/toxicity Filtering tool 3)^55, 56^.

A total of 7779 ligands were obtained after HTVS and absorption, distribution, metabolism and excretion/toxicity (ADMET) screening. These ligands were docked again using AutoDockVina^57^. This program keeps the ligand flexible while docking. Docking results obtained were consolidated and sorted in accordance with affinity score and root-mean-square deviations (RMSDs). Only ligands with an affinity value of 10.9 and above were considered. Duplicate ligand occurrences fulfilling the cutoff were removed i.e. only first occurrence was considered. All unique molecules fulfilling the criterion were considered for further analysis. These 300 ligands were then scored and screened using SP docking^58^. The top 50 ligands with highest glide score were taken for flexible XP (extra precision) docking^59^ to ensure a high accuracy of docking. In XP docking, flexibility of the interacting regions were considered, the ligand is kept flexible while some atoms of the protein that directly interact are also kept flexible. XP docking produced a high docking score and glide score (Shown in Table 2).

**Table 2:** Glide scores of the best 10 docked ligands.

Source File	Glide gscore (kcal/mol)	Glide energy (kcal/mol)	XP HBond	XP PoseRank
ligand_15	−20.6641	−84.4288	−8.16	6
ligand_302	−19.8955	−83.0535	−7.54205	8
ligand_1725	−19.5035	−74.0717	−7.23445	5
ligand_276	−18.8408	−91.4571	−7.96953	2
ligand_1259	−16.953	−65.4968	−1.57924	6
ligand_307	−16.2679	−69.7247	−1.88054	11
ligand_256	−15.8306	−66.6586	−2.22251	11
ligand_2126	−15.8234	−64.2842	−1.39871	9
ligand_682	−15.8049	−63.5301	−1.66	11
ligand_146	−15.7883	−85.6785	−1.66	11

**Figure 7:**
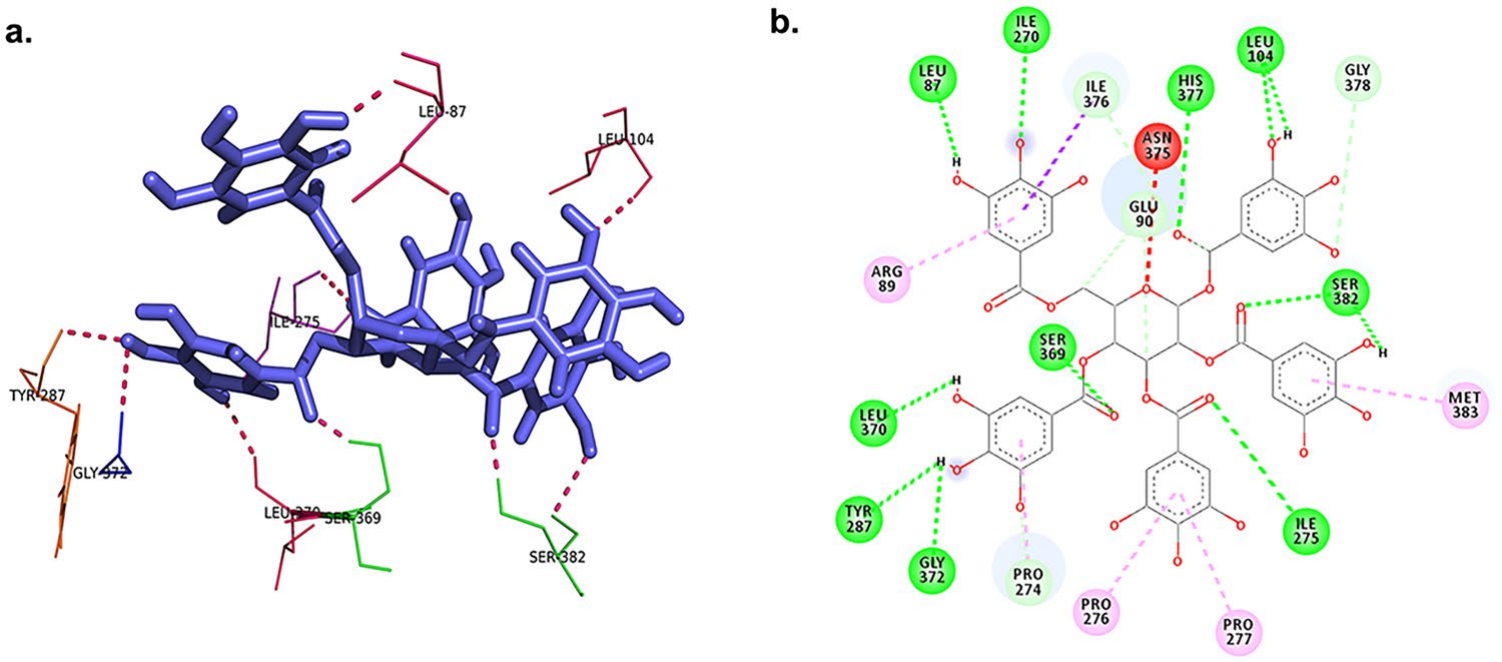
Hydrogen bond interactions within the Protein and ligand 15 complex (**a**) 3D-image (**b**) 2D-image

The best hit was Ligand 15 with a glide score of −20.66 kcal/mol (Fig. 7a and b). Ligand 15 is the ligand with DrugBank accession number DB03208. It is named as Beta-1,2,3,4,6-Penta-O-Galloyl-D-Glucopyranose. This compound belongs to the class of organic compounds known as tannins^60^. It is a naturally occurring polyphenol, aromatic heteromonocyclic compound^61^. It is a non-carcinogenic ligand with absorption in human intestine probability as −0.8347, Blood Brain Barrier value as 0.5216. Its water solubility is 0.679 mg/ml, logP 3.43 and potential energy 396.15 kcal/mol.

### Protein Simulation

The protein was simulated using Gromacs^62^ to check its conformational changes. The predicted structure of the protein was used as the initial structure for MD simulation analysis. Extended Simple Point Charge (SPC/E) water model and “AMBER99SB-ILDN protein” force-field was used to prepare the system.

The system consisted of a cubic water box containing a protein molecule at its center and Cl- and Na+ for charge stability. To keep the whole system neutral, the 3 Cl atoms were added. The protein was energetically minimized for 0.1 nanoseconds (ns) and for a maximum force greater than or equal to 10.0 kJ/mol. The production MD simulations were then carried out for 200 ns. The RMSD plot of the complete simulation indicates that the deviation from the initial structure continues to increase for the full simulation length. The deviation halted for short time intervals during the simulation but the plot direction faced upwards. The deviation was almost halted during 70 to 118 ns and then towards the end after 174 ns. The RMSF plot indicates that the initial region of the sequence has very less fluctuations whereas the later regions show higher fluctuations. Lower movement of helix and sheet region shows that loop regions provide flexibility to the protein structure. The residues that show maximum fluctuation are 210, 209, 212–215, 345, 344 and 256 respectively. The less-frequently fluctuated residues in the second half of the sequence indicate that these residues act as hinge points between the moving loop regions. The major hinge points in the sequence are residues 195, 221, 274, 310, 322, 332, and 361. The first half of the sequence showed lower fluctuations of around 0.1 nm to the minimum fluctuation. The area per atom plot indicates that the standard deviation of atoms in the second half of the protein was higher, in the first half of the sequence only a few atoms fluctuated more. The average accessible area plot showed that some residues were never accessible during the simulation at the same time some were less accessible and others were highly accessible. The high number of peaks in the second half of the sequence shows that the loop regions interacted the most with other molecules whereas there were some residues in the first that also showed high accessibility. The average displacement that protein has faced during the simulation is plotted smoothly thus there is no strong structural activity detected during the simulation. The protein slowly and gradually changes its structural conformation in response to its surrounding. The secondary structure plot indicates that the number of residues in coils and bend regions decreases whereas the number of residues in turn and 3-helix regions increased. This change was not very prominent because only around 10–15 residues changed their state. Other structures did not show any significant change during simulation. The lack of major changes indicates that they are very stable and conserved. Plotting Hydrogen bonds between protein with itself and with other residues indicated a very stable hydrogen bond count during the simulation. There were higher numbers of hydrogen bonds between the protein residues than with non-protein residues. This indicated that the protein is more internally stabilized than from outside forces.

### Protein-Ligand Complex Simulation

**Figure 8:**
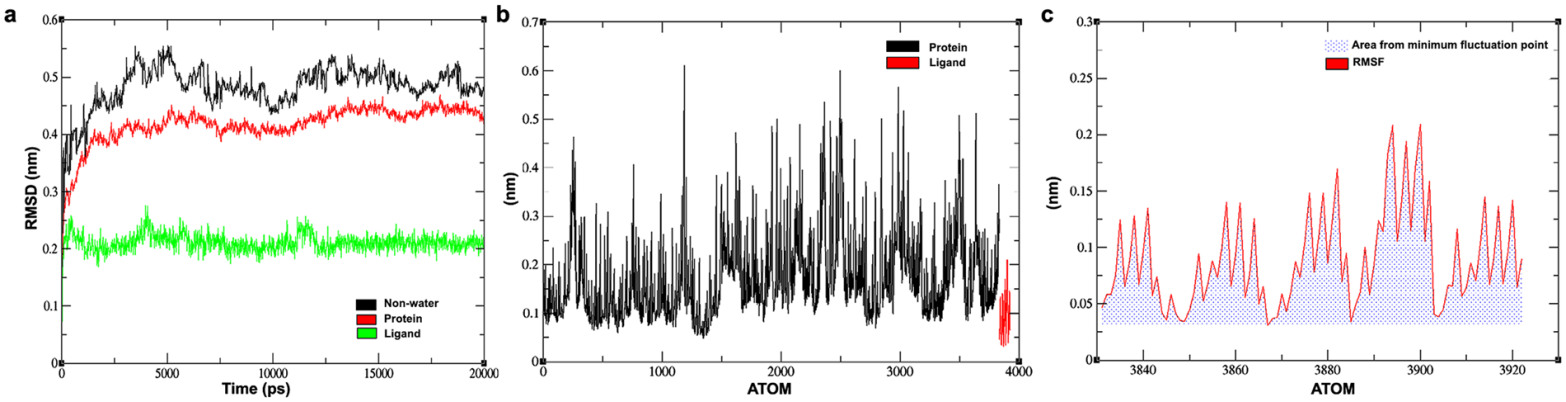
(**a**) RMSD plot of protein, ligand and complex (**b**), RMSF plot of protein and ligand (**c**), RMSF of ligand.

The protein-ligand complex simulation analysis shows that the complex is very stable (Fig. 8a). The protein became stable around 5 ns and the ligand around 2 ns. The protein was almost stable during the simulation, whereas, the ligand showed higher fluctuations at some points during the simulation. At the same time non-water molecules became stable around 6 ns. Nonetheless, the complex continued to show some fluctuations during the remaining 15 ns of simulations. The Root Mean Square Fluctuation (RMSF) analysis showed that the first half of the protein had lower fluctuations than the second half (Fig. 8b), which was comprised of loop regions. The ligand showed lower fluctuations. Most atoms had some fluctuations when they were analyzed together indicating that the protein show acceptability to other molecules and the loop region of the protein is flexible to accept ligands and adopt conformational changes based on interactions.

The hydrogen bond analysis confirmed that the high score of the complex is explainable and based on interactions. The ligand atoms that are towards the sheet region and forming a hydrogen bond with the protein showed higher fluctuations than other atoms. The atoms forming the central ring showed the least fluctuations or none at all (Fig. 8c) whereas the ‘-OH’ group attached to the outermost rings showed higher fluctuations than other atoms. The plot shows that during the simulation the movements of the outer rings are similar to one another. The amino acid residues interacting with the ligand were observed to have a bond length range of 1.5–2.8 Å.

### High-Throughput MM-PBSA Calculations by GMMPBSA

The top three hundred ligands were analyzed using the gmmpbsa method^63, 64^. This method produces a better score with respect to negating false positives i.e. top scoring ligands have higher chances to pass the experimental test. It aims to integrate high-throughput molecular dynamics (MD) simulations with binding energy calculations. The screening for 300 ligands selected after docking generated the top hundred ligands based on binding energy with energy in the range of 200–484 kcal/mol (for a detailed overview, see supplementary information: Tables S1 and S2).

## Discussion

In this work we have examined the target protein Ror1, whose expression changes during certain types of cancers. In a previous study, it was determined that when over-expression of Ror1 without HER2/neu, and hormone receptors on the cell surface were introduced in the breast cancer this protein served as an appropriate candidate for designing a cancer vaccine and it was concluded that Ror1 with an enterotoxin B could be a potent vaccine^65^. Recently, it was also reported that knockdown of Ror1 significantly inhibited cell migration and invasion and when bothRor1 and Ror2 were knocked down, the cells got significantly sensitized to cisplatin. However, Ror1 over expression in the parental cell line increased cell invasion, indicating that Ror1 and Ror2 have potential as novel drug targets in metastatic and recurrent ovarian cancer patients^29^. In a different report, it was shown that knockdown of Ror1 resulted in reduction of stemness and sphere formation capacity. Moreover, it was shown that down-regulation of Ror1 suppressed the expression of EMTrelated genes and the migratory and invasive abilities of the tumour. The results of this study indicated that targeting Ror1 could induce differentiation of cancer stem cells (CSCs) and inhibit metastasis in glioblastoma^15^. In a recent report, it was also shown that siRNAs targeting Ror1 in CLL induced apoptosis and a small molecule inhibitor, Ror1 tyrosine kinase inhibitor dephosphorylated Ror1, down-regulating the activated PI3K/AKT/mTOR signaling pathway and inducing specific apoptosis of CLL cells^66^. Therefore, we can conclude that Ror1 may be developed as a potential marker for several types of cancer and could be a potential target for stem cell therapy and drug discovery.

**Figure 9:**
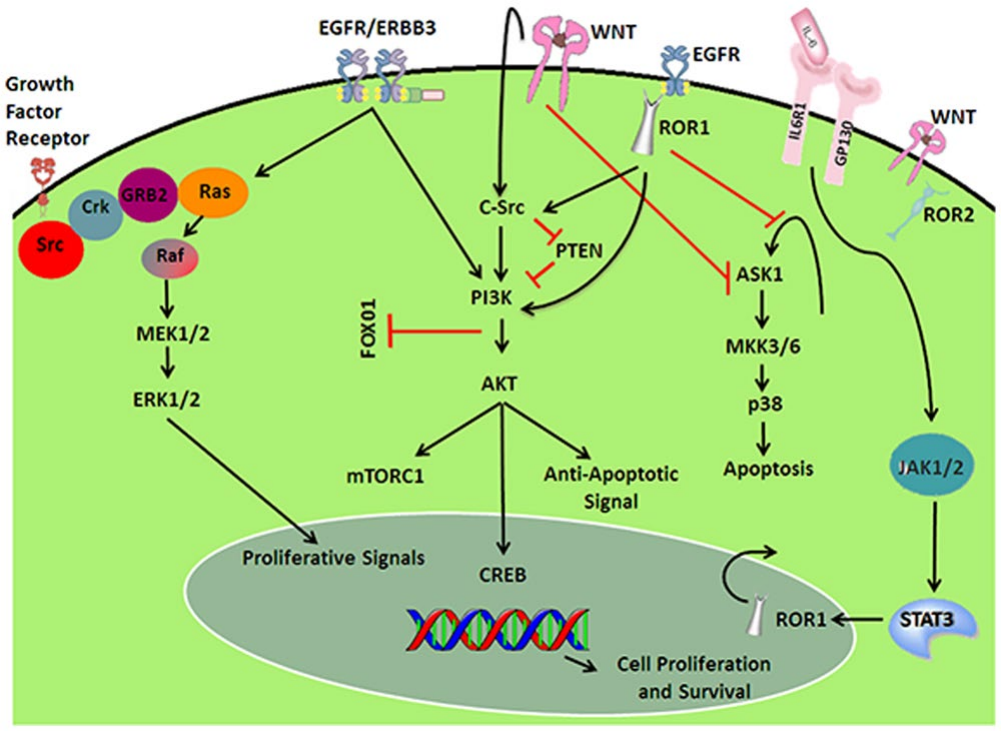
A pathway showing role of Ror1in promoting tumor-cell growth^16, 71^. During tumorous growth interleukin 6 (IL-6) binds with ILL6R1and GP130 and induces signal transducer activator of transcription 3 (STAT3) phosphorylation through the involvement of JAK proteins and upregulates Ror1 protein levels in a time- and dose-dependent manner by activating Ror1promoter activity. STAT3 also induces expression of Wnt5a that interacts with Ror1 to trigger PI3K/AKT through C-Src leading to activation of CREB that promotes tumor cell growth. Ror1 is synthesized in the nucleus and then it is transported to the cytoplasm where it inhibits the activity of ASK1, which follows a cascade that leads to apoptosis. At the same time, Ror1 activates S-src and PI3K, which in turn influences the CREB cycle to enhance expression of genes that enhance resistance to tumor cell apoptosis and/or promote tumor cell growth. Therefore, Ror1 functions to keep a balance between pro-survival PI3K-AKT and pro-apoptotic p38 signaling. [DNA Helix used in Fig. 9 is downloaded from https://pixabay.com/en/photos/?q=dna%20helix: All images on Pixabay website are released free of copyrights under Creative Commons CC0.]

Here, a detailed study of the nature of the receptor protein and scrutinizing of probable inhibitory small molecules has been performed. The target protein (Ror1) was selected on the basis of previous reports concluding that Ror1 can act as a novel target for designing an efficient drug for cancer treatment (Fig. 9). Under normal conditions this protein expresses itself during childhood to support fast growth in children and it is not expressed in normal adult cells. Thus, it is expected that targeting this molecule will control cells from becoming cancerous as well as remedying a cancerous cell conditions. Therefore, it can be used in early stage cancers as well as in late stage cancers. As this target is not expressed in normal cells and has a small role to play under normal conditions, targeting it will have minimal side effects.

In this study, the protein structure was modeled and found to be stable as analyzed by energy plot and other analyses (e.g. Ramachandran plot and Z-score). Considering an active site with high volume and high accessible surface area ensured the freedom of the ligand to find the best sites and positions to interact. The structure of this protein is very interesting as it can be divided into two halves of which the first half contains the helix and sheet regions whereas the other half contains the coil regions. Our predictions show that the active sites lie in the coil region. The helix and sheet region from its back and a coil lock from its front support the active site that we have predicted. The protein-ligand complex showed stability and minimal fluctuations during simulation; therefore, the complex is expected to be stable. Stepwise screening of a considerable number of ligands ensured that the best ligands from the database were obtained. Glide score for top hits reached a high score of −20 kcal/mol which indicates that the ligands interact with very high affinity towards the protein. Energy of the docked complexes was lower than the unbound protein and ligand, which increases the feasibility of their interaction. The best hit thus obtained is ligand 15 (DrugBank accession number DB03208), which is designated as Beta-1, 2,3,4,6-Penta-O-Galloyl-D-Glucopyranose, a naturally occurring organic compound (polyphenol) belonging to the class of tannins. Tannins are potent collagen cross-linking agent and found in many plants and plant products that are used as food. In recent reports, it was concluded that tannins are potential anticancer agents and apoptotic activity in breast cancer and prostate cancer cells is enhanced when they are exposed to tannin extracts^67–70^ indicating the role of tannins as prospective anti-cancer therapy. This discovery also supports our finding that since Beta-1,2,3,4,6-Penta-O-Galloyl-D-Glucopyranose interacts with Ror1 and inhibits its action, this compound could potentially serve as a potent drug for cancer treatment. This work can further be extended for wet lab experimentation and clinical trials. We also expect these molecules as the potent drug molecules in the market for both cancer inhibition and for decreasing chances of cancer.

## Methods

The sequence for Ror1 (ID AAC50714.1) was retrieved from NCBI protein database (https://www.ncbi.nlm.nih.gov/protein). Secondary structure predictions were performed by PROMOTIF^30^ and GOR4 tool^31^. Then the 3D-structure of the protein was predicted using following servers and tools CPHModel^34^ phyre2^35^, ps2v2^36^, RaptorX^37^, Modeller^38^ and I-Tasser^39^. The structures were analyzed and validated by Prosa^41, 42^ and SAVES^45^ server. The most correctly modelled structure was then optimized using Schrodinger’s Protein Preparation wizard^47^. Domains were searched using CDSearch^48^. Then the active site of the protein was predicted with help of DogSiteScorer^49^ and Schrodinger’s siteprep^50, 51^. The ligands were obtained from Zinc database^52^. Ligprep^53^ was used to prepare the ligands after having the ligands screened for multiple scaffolds. Then the ligands were screened using Schrodinger’s HTVS^54^. The ligands having docking score below −7 kcal/mol were analyzed on druggability parameters using Mobyl Server^55, 56^.

Then the ligands passing the druggability parameters were docked using AutoDockVina^57^. Again the screening was carried out at an affinity value 10.9 and above. The screened ligands were then screened for duplication and carried forward for SP docking^58^. Top 50 ligands obtained through screening with SP docking were docked again using XP (Extra precision) docking^59^. Then, the protein and protein-ligand15 complex were simulated using Gromacs^62^. Extended Simple Point Charge (SPC/E) water model and “AMBER99SB-ILDN protein” force-field was used to prepare the system. The system contained a cubic water box with protein, Cl- and Na+. The system was kept neutral by adding ions. The protein was energetically minimized for 0.1 ns before the production MD simulations of 200 ns. In similar fashion the protein-ligand complex was simulated for 20 ns. The top 300 ligands were analysed using g_mmpbsa^63, 64^ to obtain any probable false positives and to score better docked complexes with low energy scores.

## Electronic supplementary material

Supplementary Information
