# Supplementary material for: In-Silico Drug discovery approach targeting receptor tyrosine kinase-like orphan receptor 1 for cancer treatment
Source: Sci Rep. 2017 Apr 21;7:1029. doi: 10.1038/s41598-017-01254-w (PMC5430761; doi:10.1038/s41598-017-01254-w)
Supplement: Supplementary file 1 — Supplementary Information [file 41598_2017_1254_MOESM1_ESM.pdf]

In-Silico Drug discovery approach targeting receptor tyrosine kinase-like orphan receptor 1 for cancer treatment

Onkar Nath[1], Archana Singh[2] and Indrakant K Singh[3*]
[1]Jawaharlal Nehru University, SCIS, New Delhi - 110067, INDIA
[2]Department of Botany, Hans Raj College, University of Delhi, Delhi -110007, INDIA
[1,3]Mol Bio Res Lab, Deshbandhu College, University of Delhi, Kalkaji, New Delhi, 110019, INDIA
*Present Address: Department of Entomology, University of Kentucky, S-225 AG. Science - North, Lexington, KY 40546-0001

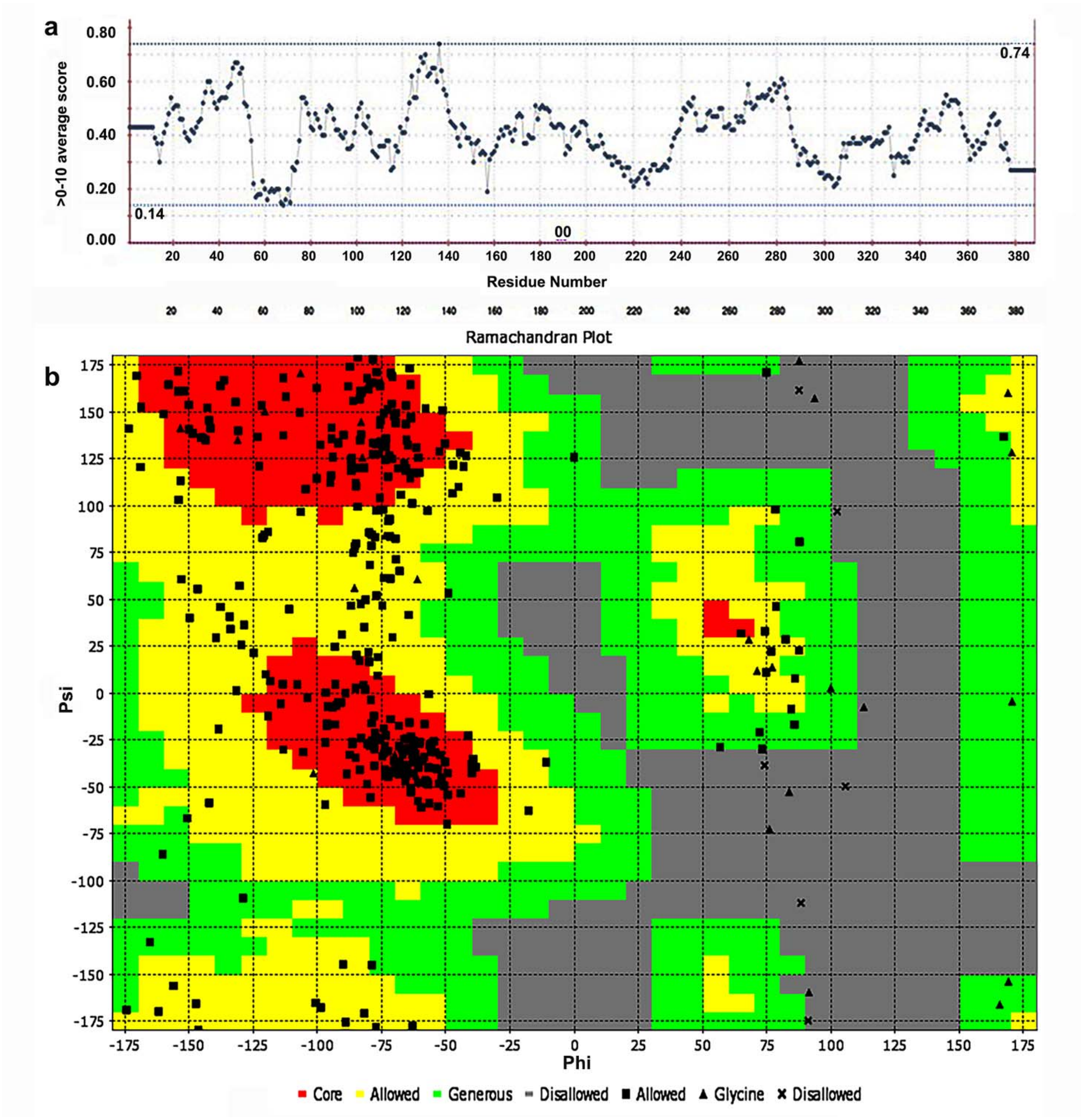

Supplementary Figure S1. (a) 3D-1D average score plot per residue, **(b)** Ramachandran plot analysis of I-Tasser model.

Supplimentary Table S1:
Ligand names and corresponding smile notation

Ligand Name	Smile
ligand_131	C1C[C@@H](CC[C@H]1C(=O)Nc1ccc(cc1)OC)CNC1=C(C(=O)C1=O)N1CCOCC1
ligand_53	C1=C(CCN(C1)CC(=O)Nc1ccc(cc1)OC)c1c[nH]c2ccc(cc12)OC
ligand_66	C1(=O)[C@H](SC(=O)N1c1ccc(cc1)O)CC(=O)Nc1ccc(c(c1)O)C(=O)O
ligand_42	c1cc(cc2c3c(CCN(C(=O)CCC(=O)NC[C@@H]4CCCO4)C3)[nH]c12)OC
ligand_43	c1cc(cc2c3c(CCN(C(=O)CCC(=O)NC[C@@H]4CCCO4)C3)[nH]c12)OC
ligand_44	C1CN(CCC1c1cc([nH]n1)CC(=O)O)c1cc(nc(n1)N)N1CCOCC1
ligand_7	N(c1cc(nc(N)n1)n1cccn1)CCC(=O)NCc1ccncc1
ligand_4	c1cc(cc2c1c(cc(=O)o2)CC(=O)NCC(=O)NCC(=O)O)O
ligand_49	O(C(=O)Cc1c2ccccc2c(=O)[nH]n1)Cc1c2ccc(c(c2oc(=O)c1)C)O
ligand_9	C(c1nc(on1)C(=O)NCCCO)Oc1ccc2c(c1)NC(=O)CC2
ligand_56	C(=O)(NCc1cccnc1N1CCN(CC1)C)Cc1ccc2c(c1)NC(=O)CO2
ligand_91	C(CC(=O)Nc1ccc(c(c1)Cl)OC)CC[C@@H]1[C@H]2[C@@H](CS1(=O)=O)NC(=O)N2
ligand_84	C1C[C@@H](CN(C1)CC(=O)Nc1ccc2OCOc2c1)C1=NN=C[C@H]1c1cc(C)no1
ligand_92	C(CC(=O)Nc1ccc(c(c1)Cl)OC)CC[C@@H]1[C@H]2[C@@H](CS1(=O)=O)NC(=O)N2
ligand_5	c1cc(cc2c1c(cc(=O)o2)CC(=O)NCC(=O)NCC(=O)O)O
ligand_60	c1c(nc(s1)NC(=O)C1=CCC(=O)N=C1)CC(=O)Nc1ccc(cc1)C(=O)N
ligand_73	c12c(CN(C(=O)c3ccc4c(cc[nH]4)c3)CC1)sc(n2)NC(=O)c1cnccn1
ligand_102	[C@H](C(=O)N1CCC[C@H]1C(=O)NCC(=O)O)(Cc1c[nH]cn1)NC(=O)[C@H]1CCC(=O)N1
ligand_36	[C@@H]1(C(=NC(=O)N[C@@H]1c1ccc(cc1)O)C)C(=O)NNCc1ccc(cc1O)O
ligand_177	C1C=C(CC2=C1[C@@H](C1=C(N(C=N[C@@H]1O2)CCN1CCOCC1)N)c1ccc(cc1)OC)O
ligand_65	C1(=O)[C@H](SC(=O)N1c1ccc(cc1)O)CC(=O)Nc1ccc(c(c1)O)C(=O)O
ligand_32	c1cc(cc(c1)OC(=O)CC1=[S]C(=O)NC1=O)CC1=[S]C(=O)N=C1N
ligand_25	C1CN(CCN1c1cc(=O)[nH]c(N)n1)C[C@@H]1[C@H](c2cc(ccc2N1)OC)C
ligand_132	C(C(=O)NNCc1cc(ccc1O)N(=O)=O)C(=O)NNCc1cc(ccc1O)N(=O)=O
ligand_8	C(#N)c1cnc(nc1N)SCc1c2ccc(c(c2oc(=O)c1)C)O
ligand_159	c1c(nc(s1)NC(=O)C1=CCC(=O)N=C1)CC(=O)Nc1ccc(cc1)S(=O)(=O)N
ligand_38	[C@H]1(C(=NC(=O)N[C@H]1c1ccc(cc1)O)C)C(=O)NNCc1ccc(cc1O)O
ligand_14	C(=O)(N1CCN(CC1)Cc1cccc(c1)NC(=O)C)c1ccc(cc1)O
ligand_30	C(#N)c1c(cc(nc1N)c1cccc(c1)NC(=O)C)c1ccc(O)c(c1)OC
ligand_116	c1cc2c(cc1C(=O)NCCCOC)[C@H]1CCC[C@H]1[C@@H](N2)c1cc(ccc1O)N(=O)=O
ligand_18	c1c2CN(CCn2nc1CNC(=O)N(C)C)c1c2c[nH]nc2nc(n1)N
ligand_134	C(C(=O)NNCc1cc(ccc1O)N(=O)=O)C(=O)NNCc1cc(ccc1O)N(=O)=O
ligand_79	c1(nc(no1)COc1ccc2CCC(=O)Nc2c1)C(=O)NCCc1ccc(cc1)O
ligand_22	C(#CCOc1cccc(c1)NC(=O)C)CNC(=O)c1ccc2cc[nH]c2c1
ligand_21	C(#N)C1=C([C@H](C(=O)NC1=O)/C=N/c1ccc(cc1)S(=O)(=O)CCO)C
ligand_74	c12c(CN(C(=O)c3ccc4c(c3)nc[nH]4)CC1)sc(n2)NC(=O)c1cnccn1
ligand_86	c1cc(cc(c1)OC1CCN(CC1)Cc1ccncc1)C(=O)N[C@H]1CC[C@H](CC1)O
ligand_35	c1cc(cc(c1)NC(=O)c1cccc(c1O)O)NC(=O)c1cccc(c1O)O
ligand_235	c1(nc(=O)c2c(CN(CC2)Cc2ccc(c(c2)CN2CCN(CC2)C)O)[nH]1)c1cccnc1
ligand_17	c1c2CN(CCn2nc1CNC(=O)N(C)C)c1c2c[nH]nc2nc(n1)N
ligand_103	[C@H](C(=O)N1CCC[C@H]1C(=O)NCC(=O)O)(Cc1c[nH]cn1)NC(=O)[C@H]1CCC(=O)N1
ligand_24	c1cc2c(c(c1)NC(=O)C)CCN(C2)Cc1ccc(cc1N(=O)=O)C(=O)N
ligand_1	C(#N)/C(=C(\C)/N)/C(=O)COC(=O)c1ccc2c(c1)NC(=O)CS2
ligand_19	c1cc(ccc1[C@H](O)CC1CCN(CC1)C)NC(=O)c1ccc(=O)[nH]n1

Supplimentary Table S1:
Ligand names and corresponding smile notation

Ligand Name	Smile
ligand_193	C(c1csc(n1)NC(=O)[C@@H]1C=CC=NC1=O)C(=O)Nc1cc(ccc1OC)NC(=O)C
ligand_40	C1=C(CCN(C1)CC(=O)Nc1sc(C)nn1)c1c[nH]c2ccc(cc12)OC
ligand_167	c1cc(ccc1CNNC(=O)c1ccc(cc1O)O)CNNC(=O)c1ccc(cc1O)O
ligand_109	c1cc(c(cc1CC1=[S]C(=NC1=O)N)OCC)OC(=O)C[C@@H]1C(=O)NC(=O)S1
ligand_29	C1CN(CCC1C(=O)Nc1cnn(c1)CC)Cc1cc(ccc1N(=O)=O)O
ligand_69	C1(=O)[C@@H](SC(=O)N1c1ccc(cc1)O)CC(=O)Nc1ccc(cc1O)N(=O)=O
ligand_67	C1(=O)[C@H](SC(=O)N1c1cccc(c1)O)CC(=O)Nc1ccc(c(c1)O)C(=O)O
ligand_71	c1c(nc(s1)NC(=O)C1=CCC(=O)N=C1)CCC(=O)N1CC[C@@H](CC1)C(=O)N
ligand_26	c1cc(ccc1[C@H](C)NC(=O)N[C@H](C)CCCO)NC(=O)c1ccncc1
ligand_285	c1c(c(n[nH]1)c1ccc(cc1)OC)/C=N/NC(=O)c1cc(ccc1O)O
ligand_88	S(c1[nH]c(nn1)N/N=C/c1cccnc1)CCC(=O)Nc1cccc(c1)N(=O)=O
ligand_209	c1cc(ccc1C(=O)NCCc1c[nH]c2ccccc12)OCC(=O)Nc1cccc(c1)OC
ligand_39	c1cc(ccc1CC(=O)N1CCC(CC1)O)NC(=O)Nc1ccc(cc1)OC
ligand_289	c1(c(c(oc1c1ccc(cc1)NC(=O)C)/C=N/N)N(=O)=O)c1ccc(cc1)NC(=O)C
ligand_45	C(#N)c1cccc(c1)NC(=O)CN1CC=C(CC1)c1c[nH]c2ccc(cc12)OC
ligand_99	C1=CC(=C[C@@H]2C1=CC(=C(NC1=C(CC(=CC1)OC)OC)O2)C(=O)Nc1ccncc1)O
ligand_158	C1C[C@@H](CC[C@H]1C(=O)Nc1sc(nn1)CC)CNC1=C(C(=O)C1=O)N1CCCCC1
ligand_54	C1=C(CCN(C1)CC(=O)Nc1cccc(c1)OC)c1c[nH]c2ccc(cc12)OC
ligand_28	C1CN(CCC1C(=O)Nc1cnn(c1)CC)Cc1cc(ccc1N(=O)=O)O
ligand_23	c1cc(ccc1/C(=N\NC(=O)c1[nH]cnn1)/C)/N=C/c1ccc(cc1O)O
ligand_239	c1cc(ccc1CNNC(=O)Cn1c(=S)[nH]c(C)n1)OC(=O)C[C@@H]1C(=O)NC(=O)S1
ligand_6	c1(nc(no1)COc1cccc(c1)NC(=O)C)C(=O)NCCCO
ligand_16	C(#N)c1cnc(nc1N)SCc1c2cc(c(cc2oc(=O)c1)O)CC
ligand_61	c1cc(ccc1CC(=O)N1CCC(CC1)O)NC(=O)Nc1ccc2OCOc2c1
ligand_139	C(#N)c1c(N)oc2c(c(C)nn2)c1c1ccc(cc1)OCC(=O)Nc1ccc(cc1)OC
ligand_70	C(C#CCOc1cccc(c1)NC(=O)C)NC(=O)C(=O)c1c2ccccc2[nH]c1C
ligand_217	c1cc(ccc1[C@H]1N(C(=O)CS1)c1ccc(cc1)O)OC(=O)C[C@H]1C(=O)NC(=O)S1
ligand_15	C(#N)c1cnc(nc1N)SCc1c2cc(c(cc2oc(=O)c1)O)CC
ligand_262	c1(nnc(s1)NC(=O)c1ccc(cc1)OC)CC(=O)NNCc1ccc(O)c(c1)O
ligand_297	C1CN(CCC1c1c[nH]c2ccc(cc12)OC)S(=O)(=O)c1ccc(cc1)NC(=O)N
ligand_291	c1cc(c(c2c1C(=O)/C(=C/c1ccc(cc1)N(=O)=O)/O2)CN1CCN(CC1)CCO)O
ligand_197	C(c1csc(n1)NC(=O)C1=CCC(=O)N=C1)C(=O)Nc1cc(ccc1OC)NC(=O)C
ligand_195	C(c1csc(n1)NC(=O)[C@@H]1C=CC=NC1=O)C(=O)Nc1cc(ccc1OC)NC(=O)C
ligand_173	C(C(=O)Nc1ccc(cc1)OC(F)(F)F)CCC[C@@H]1[C@H]2[C@@H](CS1(=O)=O)NC(=O)N2
ligand_154	C(#N)c1c(N)oc2c(c(C)nn2)c1c1ccc(cc1)OCC(=O)Nc1cccc(c1)OC
ligand_50	c1cc(cc2c3c(CCN(C(=O)CCC(=O)NCc4cccnc4)C3)[nH]c12)OC
ligand_133	C(C(=O)NNCc1cc(ccc1O)N(=O)=O)C(=O)NNCc1cc(ccc1O)N(=O)=O
ligand_82	c1cc(ccc1C(=O)Nc1ccncc1)NS(=O)(=O)c1ccc2c(c1)[nH]c(=O)[nH]2
ligand_124	C(=O)(N1CCC[C@@H](C1)c1ccc(cn1)Nc1cnccn1)CCc1c[nH]c2ccccc12
ligand_130	C1C[C@@H](CC[C@H]1C(=O)Nc1ccc(cc1)OC)CNC1=C(C(=O)C1=O)N1CCOCC1
ligand_48	C1=C(CCN(C(=O)Nc2ccc(cc2C)OC)C1)c1c[nH]c2ccc(cc12)OC
ligand_13	c1cc(c(c2c1c(cc(=O)o2)CC(=O)NCC(=O)NCC(=O)O)C)O
ligand_80	c1cc(ccc1C(=O)N1CC[C@H](CC1)C(=O)N)NC(=O)N[C@H]1CCS(=O)(=O)C1
ligand_81	c1cc(ccc1C(=O)N1CC[C@H](CC1)C(=O)N)NC(=O)N[C@H]1CCS(=O)(=O)C1

Supplimentary Table S1:
Ligand names and corresponding smile notation

Ligand Name	Smile
ligand_249	c1(nnc(s1)NC(=O)c1ccc(cc1)OC)CC(=O)NNCc1cccc(c1)O
ligand_166	c1cc(ccc1CNNC(=O)c1ccc(cc1O)O)CNNC(=O)c1ccc(cc1O)O
ligand_55	C1=C(CCN(C1)CC(=O)Nc1cccc(c1)OC)c1c[nH]c2ccc(cc12)OC
ligand_75	c1cc(c(cc1/C=C\1/C(=O)N=C(S1)N)OC)OC(=O)C[C@@H]1C(=O)NC(=O)S1
ligand_108	c1cc(c(cc1CC1=[S]C(=NC1=O)N)OCC)OC(=O)C[C@@H]1C(=O)NC(=O)S1
ligand_148	C(#N)c1c(N)oc2c(c(C)nn2)c1c1ccc(cc1)OCC(=O)Nc1cccc(c1)OC
ligand_12	c1cc(c(c2c1c(cc(=O)o2)CC(=O)NCC(=O)NCC(=O)O)C)O
ligand_113	c1cc(cc(c1)S(=O)(=O)N1CCN(CC1)CC)NC(=O)Cn1ccc(=O)[nH]c1=O
ligand_59	c1cc(ccc1C(=O)N1CCC(CC1)O)OC1CCN(CC1)Cc1ccccn1
ligand_243	C(CC(=O)NNCc1ccc(cc1)O)CC(=O)NNCc1ccc(cc1)O
ligand_215	c1cc(ccc1[C@@H]1N(C(=O)CS1)c1ccc(cc1)O)OC(=O)C[C@@H]1C(=O)NC(=O)S1
ligand_72	c12c(CN(C(=O)c3ccc4cc[nH]c4c3)CC1)sc(n2)NC(=O)c1cnccn1
ligand_155	C(#N)c1c(N)oc2c(c(C)nn2)c1c1ccc(cc1)OCC(=O)Nc1cccc(c1)OC
ligand_111	c12c(CN(C(=O)c3ccc(cc3)NC(=O)C)CC1)sc(n2)NC(=O)c1cnccn1
ligand_106	[C@H](C(=O)N1CCC[C@H]1C(=O)NCC(=O)O)(Cc1c[nH]cn1)NC(=O)[C@H]1CCC(=O)N1
ligand_181	c1cc(cc2c1C(=O)NC[C@]1(CCC(=O)N(CC1)CC(=O)NCc1ccncc1)O2)OC
ligand_182	C(=O)(N1CCC(CC1)C(=O)c1ccc2c(c1)NC(=O)CO2)c1ccc(o1)CN1CCCC1
ligand_214	c1cc(ccc1[C@@H]1N(C(=O)CS1)c1ccc(cc1)O)OC(=O)C[C@@H]1C(=O)NC(=O)S1
ligand_96	C1=CC(=C[C@@H]2C1=CC(=C(Nc1ccc(c(c1)OC)OC)O2)C(=O)Nc1ccncc1)O
ligand_57	c1cc(ccc1C(=O)N1CCC(CC1)O)OC1CCN(CC1)Cc1ccccn1
ligand_78	c1(nc(no1)COc1ccc2CCC(=O)Nc2c1)C(=O)NCCc1ccc(cc1)O
ligand_52	c1cc(cc2c3c(CCN(C(=O)CCC(=O)NCc4cccnc4)C3)[nH]c12)OC
ligand_95	C1=CC(=C[C@@H]2C1=CC(=C(Nc1ccc(c(c1)OC)OC)O2)C(=O)Nc1ccncc1)O
ligand_141	C(#N)c1c(N)oc2c(c(C)nn2)c1c1ccc(cc1)OCC(=O)Nc1ccc(cc1)OC
ligand_187	c1cc(ccc1NC(=O)N1CCC[C@@H](C1)C(=O)NCC)NC(=O)Nc1ccc(cc1)OC
ligand_231	C1C[C@@H](CC[C@H]1C(=O)Nc1sc(nn1)CC)CNC1=C(C(=O)C1=O)N1CC[C@H](CC1)C
ligand_275	C(#N)c1ccc(cc1)OCC(=O)N1CCC(CC1)C(=O)Nc1ccc(cc1)S(=O)(=O)N
ligand_272	c1cc(cc2c1c(cc(=O)o2)c1ccc(cc1)OC)OCC(=O)Nc1ccc2c(cc[nH]2)c1
ligand_191	C1C[C@@H](CC[C@H]1C(=O)Nc1ccc(cc1)OC)CNC1=C(C(=O)C1=O)N1CC[C@H](CC1)C
ligand_107	[C@H](C(=O)N1CCC[C@H]1C(=O)NCC(=O)O)(Cc1c[nH]cn1)NC(=O)[C@H]1CCC(=O)N1
ligand_246	c1cc(ccc1C(=O)NNCc1ccc(cc1)O)NNCc1ccc(cc1)O
ligand_255	C1CN(CCC1C(=O)NNCc1ccc(cc1)O)CC(=O)NNCc1ccc(cc1)O
ligand_149	C(#N)c1c(N)oc2c(c(C)nn2)c1c1ccc(cc1)OCC(=O)Nc1cccc(c1)OC
ligand_117	c1cc2c(cc1C(=O)NCCCOC)[C@H]1CCC[C@@H]1[C@@H](N2)c1cc(ccc1O)N(=O)=O
ligand_143	C(#N)c1c(N)oc2c(c(C)nn2)c1c1ccc(cc1)OCC(=O)Nc1ccc(cc1)OC
ligand_101	c1cc(c(cc1CNNC(=O)c1ccncc1)COc1ccc(cc1)NC(=O)C)OC
ligand_286	c1(nc2c(c(=O)[nH]1)CCN(C(=O)[C@@H](N)Cc1ccc(cc1)O)C2)c1ccncc1
ligand_100	c1cc(c(cc1CNNC(=O)c1ccncc1)COc1ccc(cc1)NC(=O)C)OC
ligand_150	C(#N)c1c(N)oc2c(c(C)nn2)c1c1ccc(cc1)OCC(=O)Nc1cccc(c1)OC
ligand_10	C(c1nc(on1)C(=O)NCCCO)Oc1ccc2c(c1)NC(=O)CC2
ligand_225	C(=O)(N1CC[C@@]2(CCCN(C2)Cc2cc(ccc2F)OC)C1)CCn1ccc(=O)[nH]c1=O
ligand_68	C1(=O)[C@H](SC(=O)N1c1cccc(c1)O)CC(=O)Nc1ccc(c(c1)O)C(=O)O
ligand_137	C(#N)c1c(N)oc2c(c(C)nn2)c1c1ccc(cc1)OCC(=O)Nc1ccc(cc1)OC
ligand_2	C(#N)CC(=O)NNCc1ccc(c(c1)N(=O)=O)N1CCN(CC1)C

Supplimentary Table S1:
Ligand names and corresponding smile notation

Ligand Name	Smile
ligand_27	c1[nH]c2c(CN(C(=O)COc3cccc(c3)NC(=O)CC)[C@H](C2)C(=O)O)n1
ligand_279	C(C(=O)NNCc1cccc(c1)O)CC(=O)NNCc1cccc(c1)O
ligand_123	C(=O)(N1CCC[C@H](C1)c1ccc(cn1)Nc1cnccn1)CCc1c[nH]c2ccccc12
ligand_3	c1cc(cc2c1c(cc(=O)o2)CC(=O)NCC(=O)NCC(=O)O)O
ligand_252	c1cc(oc1C(=O)N/N=C(\C)/c1ccc(cc1)O)C(=O)N/N=C(\C)/c1ccc(cc1)O
ligand_128	c12c(CN(C(=O)CCn3ccc(=O)[nH]c3=O)CC1)sc(n2)NC(=O)c1cnccn1
ligand_125	C(=O)(N1CCC[C@H](C1)c1ccc(cn1)Nc1cnccn1)CCc1c[nH]c2ccccc12
ligand_41	c1cc(cc2c3c(CCN(C(=O)CCC(=O)NC[C@H]4CCCO4)C3)[nH]c12)OC
ligand_256	C1CN(CCC1C(=O)NNCc1ccc(cc1)O)CC(=O)NNCc1ccc(cc1)O
ligand_206	C1C[C@@H](CC[C@H]1C(=O)Nc1ccc(cc1)OCC)CNC1=C(C(=O)C1=O)N1CCOCC1
ligand_207	C1C[C@@H](CC[C@H]1C(=O)NCc1ccccc1OC)CNC1=C(C(=O)C1=O)N1CCOCC1
ligand_183	C(=O)(NCCc1c2cc(ccc2[nH]c1)OC)[C@H]1CCN(CC1)[C@H]1Nc2cccnc2S1
ligand_299	C(#N)c1ccc(cc1)S(=O)(=O)N1CCN(CC1)Cc1ccc(cc1N(=O)=O)C(=O)N
ligand_162	c1cc(ccc1CNNC(=O)c1ccc(cc1O)O)CNNC(=O)c1ccc(cc1O)O
ligand_198	C(c1csc(n1)NC(=O)C1=CCC(=O)N=C1)C(=O)Nc1cc(ccc1OC)NC(=O)C
ligand_233	c1(nc(=O)c2c(CN(CC2)Cc2ccc(c(c2)CN2CCN(CC2)C)O)[nH]1)c1ccncc1
ligand_47	[C@@H]12C(=NCN(C2)CCCOc2cccc(c2)NC(=O)C)N(C(=O)N(C1=O)C)C
ligand_244	c1cc(cc(c1)NNCc1ccc(cc1)O)C(=O)NNCc1ccc(cc1)O
ligand_259	C1CN(CCC1C(=O)NNCc1ccc(cc1)O)CC(=O)NNCc1ccc(cc1)O
ligand_216	c1cc(ccc1[C@H]1N(C(=O)CS1)c1ccc(cc1)O)OC(=O)C[C@H]1C(=O)NC(=O)S1
ligand_126	C(=O)(N1CCC[C@@H](C1)c1ccc(cn1)Nc1cnccn1)CCc1c[nH]c2ccccc12
ligand_104	[C@@H](C(=O)N1CCC[C@H]1C(=O)NCC(=O)O)(Cc1c[nH]cn1)NC(=O)[C@H]1CCC(=O)N1
ligand_105	[C@@H](C(=O)N1CCC[C@H]1C(=O)NCC(=O)O)(Cc1c[nH]cn1)NC(=O)[C@H]1CCC(=O)N1
ligand_169	C(CCC(=O)Nc1cccc(c1)NC(=O)C)NC(=O)N1CC[C@H]2c3ccccc3N[C@H]2C1
ligand_122	C(=O)(N1CCC[C@@H](C1)c1ccc(cn1)Nc1cnccn1)CCc1c[nH]c2ccccc12
ligand_114	c1cc(cc(c1)S(=O)(=O)N1CCN(CC1)CC)NC(=O)Cn1ccc(=O)[nH]c1=O
ligand_223	c1c(cc(cc1C(=O)Nc1ccc(cc1)O)N1C(=O)CCC1=O)C(=O)Nc1ccc(cc1)O
ligand_250	c1(nnc(s1)NC(=O)c1ccc(cc1)OC)CC(=O)NNCc1ccc(cc1)O
ligand_51	c1cc(cc2c3c(CCN(C(=O)CCC(=O)NCc4cccnc4)C3)[nH]c12)OC
ligand_138	C(#N)c1c(N)oc2c(c(C)nn2)c1c1ccc(cc1)OCC(=O)Nc1ccc(cc1)OC
ligand_236	c1cc(ccc1CNNC(=O)Cn1c(=S)[nH]c(C)n1)OC(=O)C[C@@H]1C(=O)NC(=O)S1
ligand_120	c1cc(ccc1NC(=O)N1CCC[C@@H](C1)C(=O)NC)NC(=O)Nc1ccc(cc1)OC
ligand_121	c1(c(non1)C(=O)NNCc1ccc(cc1O)O)C(=O)NNCc1ccc(cc1O)O
ligand_119	C(C(=O)Nc1ccc2[nH]c(=O)[nH]c2c1)OC(=O)c1ccc(c(c1)S(=O)(=O)N)Cl
ligand_298	C1CN(CCC1c1c[nH]c2ccc(cc12)OC)S(=O)(=O)c1ccc(cc1)NC(=O)N
ligand_144	C(#N)c1c(N)oc2c(c(C)nn2)c1c1ccc(cc1)OCC(=O)Nc1ccc(cc1)OC
ligand_161	c1cc(ccc1CNNC(=O)c1ccc(cc1O)O)CNNC(=O)c1ccc(cc1O)O
ligand_226	C1C[C@@H](CC[C@H]1C(=O)Nc1ccc(cc1)O)CNS(=O)(=O)c1ccc(cc1)NC(=O)C
ligand_170	C(CCCNC(=O)N[C@H]1CCS(=O)(=O)C1)CCNC(=O)N[C@H]1CCS(=O)(=O)C1
ligand_280	C1CN(CCC1C(=O)NNCc1ccc(cc1)O)CC(=O)NNCc1ccc(cc1)O
ligand_37	[C@H]1(C(=NC(=O)N[C@H]1c1ccc(cc1)O)C)C(=O)NNCc1ccc(cc1O)O
ligand_188	c1cc(ccc1NC(=O)N1CCC[C@@H](C1)C(=O)NCC)NC(=O)Nc1ccc(cc1)OC
ligand_31	C(#N)c1c(cc(nc1N)c1cccc(c1)NC(=O)C)c1ccc(O)c(c1)OC
ligand_90	c1cc(ccc1CC(=O)N1CCC(CC1)O)NC(=O)Nc1cc(cc(c1)OC)OC

Supplimentary Table S1:
Ligand names and corresponding smile notation

Ligand Name	Smile
ligand_266	c1c(c(C)nc(c1C(=O)NNCc1ccc(cc1)O)C)C(=O)NNCc1ccc(cc1)O
ligand_160	c1cc(ccc1CNNC(=O)c1ccc(cc1O)O)CNNC(=O)c1ccc(cc1O)O
ligand_185	c1cc(ccc1C(=O)NCCN1CCOCC1)N1CCC(CC1)NCCc1ccncc1
ligand_213	c1cc(ccc1[C@H]1N(C(=O)CS1)c1ccc(cc1)O)OC(=O)C[C@@H]1C(=O)NC(=O)S1
ligand_63	c1cc(ccc1CC(=O)N1CCC(CC1)O)NC(=O)Nc1ccc(cc1)OCC
ligand_64	C1=C(CCN(C1)CC(=O)Nc1cccnc1Cl)c1c[nH]c2ccc(cc12)OC
ligand_151	C(#N)c1c(N)oc2c(c(C)nn2)c1c1ccc(cc1)OCC(=O)Nc1cccc(c1)OC
ligand_210	c1cc(ccc1C(=O)NCCc1c[nH]c2ccccc12)OCC(=O)Nc1cccc(c1)OC
ligand_145	C(#N)c1c(N)oc2c(c(C)nn2)c1c1ccc(cc1)OCC(=O)Nc1ccc(cc1)OC
ligand_83	c1cc(ccc1C(=O)Nc1ccncc1)NS(=O)(=O)c1ccc2c(c1)[nH]c(=O)[nH]2
ligand_175	c1cc(cc2c1[C@H](C1=C(N(C=N[C@H]1O2)CCN1CCOCC1)N)c1ccc(cc1)OC)O
ligand_240	c1cc(cc(c1)S(=O)(=O)N1CC(=O)NCC1)NCC(=O)Nc1ccc(cc1)C(=O)N
ligand_146	C(#N)c1c(N)oc2c(c(C)nn2)c1c1ccc(cc1)OCC(=O)Nc1cccc(c1)OC
ligand_245	c1cc(ccc1C(=O)NNCc1ccc(cc1)O)NNCc1ccc(cc1)O
ligand_110	C(=O)(NCCc1c[nH]c2ccc(cc12)OC)CCC(=O)Nc1ccc2[nH]c(=O)[nH]c2n1
ligand_267	c1c(c(C)nc(c1C(=O)NNCc1ccc(cc1)O)C)C(=O)NNCc1ccc(cc1)O
ligand_98	C1=CC(=C[C@@H]2C1=CC(=C(Nc1ccc(cc1OC)OC)O2)C(=O)Nc1ccncc1)O
ligand_196	C(c1csc(n1)NC(=O)[C@H]1C=CC=NC1=O)C(=O)Nc1cc(ccc1OC)NC(=O)C
ligand_228	C1C[C@@H](CC[C@H]1C(=O)Nc1cccc(c1)O)CNS(=O)(=O)c1ccc(cc1)NC(=O)C
ligand_211	c1cc(ccc1[C@H]1N(C(=O)CS1)c1ccc(cc1)O)OC(=O)C[C@@H]1C(=O)NC(=O)S1
ligand_261	c1(nnc(s1)NC(=O)c1ccc(cc1)OC)CC(=O)NNCc1ccc(O)c(c1)O
ligand_178	N(C(=O)NCC(=O)Nc1cccc2c1nsn2)[C@H](C(=O)O)Cc1c[nH]c2ccccc12
ligand_168	c1cc(cc2c3c(CCN(C(=O)CCC(=O)Nc4cccc(c4)NC(=O)C)C3)[nH]c12)OC
ligand_93	c1cc(ccc1C(=O)Nc1scnn1)NS(=O)(=O)c1ccc2c(c1)[nH]c(=O)[nH]2
ligand_77	c1cc(cc2c3c(CCN(C(=O)CCC(=O)Nc4ccccc4OC)C3)[nH]c12)OC
ligand_278	C(#N)c1ccc(cc1)OCC(=O)N1CCC(CC1)C(=O)Nc1ccc(cc1)S(=O)(=O)N
ligand_174	c1cc(ccc1Nc1cc(nc(n1)C)N1CCOCC1)NC(=O)Nc1ccccc1OC
ligand_165	c1cc(ccc1CNNC(=O)c1ccc(cc1O)O)CNNC(=O)c1ccc(cc1O)O
ligand_11	c1cc(c(c2c1c(cc(=O)o2)CC(=O)NCC(=O)NCC(=O)O)C)O
ligand_163	c1cc(ccc1CNNC(=O)c1ccc(cc1O)O)CNNC(=O)c1ccc(cc1O)O
ligand_194	C(c1csc(n1)NC(=O)[C@@H]1C=CC=NC1=O)C(=O)Nc1cc(ccc1OC)NC(=O)C
ligand_251	c1cc(oc1C(=O)N/N=C(\C)/c1ccc(cc1)O)C(=O)N/N=C(\C)/c1ccc(cc1)O
ligand_180	c1cc(cc2c1C(=O)NC[C@@]1(CCC(=O)N(CC1)CC(=O)NCc1ccncc1)O2)OC
ligand_115	c1cc2c(cc1C(=O)NCCCOC)[C@H]1CCC[C@H]1[C@H](N2)c1cc(ccc1O)N(=O)=O
ligand_295	C(=O)(Nc1cc(ccc1C(=O)O)C(=O)O)C[C@@H]1C(=O)N(C(=O)S1)c1cccc(c1)O
ligand_212	c1cc(ccc1[C@H]1N(C(=O)CS1)c1ccc(cc1)O)OC(=O)C[C@@H]1C(=O)NC(=O)S1
ligand_192	c1cc(ccc1C(=O)NCCN1CCC[C@H](C1)O)OC1CCN(CC1)Cc1ccccn1
ligand_230	c1cc(ccc1CC(=O)NCc1cccc(c1)OC)NC1=N[C@@H]2CS(=O)(=O)C[C@@H]2S1
ligand_20	c1cc(ccc1[C@@H](O)CC1CCN(CC1)C)NC(=O)c1ccc(=O)[nH]n1
ligand_221	c1cc(ccc1[C@@H]1N(C(=O)CS1)c1ccc(cc1)O)OC(=O)C[C@@H]1C(=O)NC(=O)S1
ligand_142	C(#N)c1c(N)oc2c(c(C)nn2)c1c1ccc(cc1)OCC(=O)Nc1ccc(cc1)OC
ligand_205	C1C[C@@H](CC[C@H]1C(=O)Nc1ccc(cc1)OCC)CNC1=C(C(=O)C1=O)N1CCOCC1
ligand_58	c1cc(ccc1C(=O)N1CCC(CC1)O)OC1CCN(CC1)Cc1ccccn1
ligand_292	c1cc(c(c2c1C(=O)/C(=C/c1ccc(cc1)N(=O)=O)/O2)CN1CCN(CC1)CCO)O

Supplimentary Table S1:
Ligand names and corresponding smile notation

Ligand Name	Smile
ligand_234	c1(nc(=O)c2c(CN(CC2)Cc2ccc(c(c2)CN2CCN(CC2)C)O)[nH]1)c1cccnc1
ligand_274	C(#N)c1ccc(cc1)OCC(=O)N1CCC(CC1)C(=O)Nc1ccc(cc1)S(=O)(=O)N
ligand_283	c1c(c(C)nc(c1C(=O)NNCc1ccc(cc1)O)C)C(=O)NNCc1ccc(cc1)O
ligand_186	C(=O)(N1CCN(CC1)c1cc(ncn1)Nc1ccccn1)c1ccc(cc1)S(=O)(=O)N
ligand_254	C1CN(CCC1C(=O)NNCc1ccc(cc1)O)CC(=O)NNCc1ccc(cc1)O
ligand_268	c1c(c(C)nc(c1C(=O)NNCc1ccc(cc1)O)C)C(=O)NNCc1ccc(cc1)O
ligand_199	[C@@H](c1ccc(cc1)OC)(c1c[nH]c2ccccc12)CNC(=O)c1ccc2c(c1)NC(=O)CO2
ligand_87	c1cc(ccc1[C@@H]1[C@H](N(C(=O)CO1)C1CCOCC1)CO)NC(=O)c1cccnc1
ligand_294	C(=O)(Nc1cc(ccc1C(=O)O)C(=O)O)C[C@@H]1C(=O)N(C(=O)S1)c1cccc(c1)O
ligand_282	c1c(c(C)nc(c1C(=O)NNCc1ccc(cc1)O)C)C(=O)NNCc1ccc(cc1)O
ligand_260	c1(nnc(s1)NC(=O)c1ccc(cc1)OC)CC(=O)NNCc1ccc(cc1O)O
ligand_46	C(#N)c1cccc(c1)NC(=O)CN1CC=C(CC1)c1c[nH]c2ccc(cc12)OC
ligand_284	c1c(c(C)nc(c1C(=O)NNCc1ccc(cc1)O)C)C(=O)NNCc1ccc(cc1)O
ligand_264	c1c(c(C)nc(c1C(=O)NNCc1ccc(cc1)O)C)C(=O)NNCc1ccc(cc1)O
ligand_281	C1CN(CCC1C(=O)NNCc1ccc(cc1)O)CC(=O)NNCc1ccc(cc1)O
ligand_171	C(CCCNC(=O)N[C@H]1CCS(=O)(=O)C1)CCNC(=O)N[C@H]1CCS(=O)(=O)C1
ligand_129	C1C[C@@H](CC[C@H]1C(=O)Nc1ccc(cc1)OC)CNC1=C(C(=O)C1=O)N1CCOCC1
ligand_208	C1C[C@@H](CC[C@H]1C(=O)NCc1ccccc1OC)CNC1=C(C(=O)C1=O)N1CCOCC1
ligand_152	C(#N)c1c(N)oc2c(c(C)nn2)c1c1ccc(cc1)OCC(=O)Nc1cccc(c1)OC
ligand_157	c1cc(cc(c1)S(=O)(=O)N1CC(=O)NCC1)NCC(=O)Nc1ccc(cc1)OCC
ligand_290	c1cc(c(c2c1C(=O)/C(=C/c1ccc(cc1)N(=O)=O)/O2)CN1CCN(CC1)CCO)O
ligand_224	c1cc(oc1C(=O)Nc1cccc(c1)NC(=O)C1CC1)COc1ccc2c(c1)CCC(=O)N2
ligand_189	C1C[C@@H](CC[C@H]1C(=O)Nc1cccc(c1)C(=O)C)CNC1=C(C(=O)C1=O)N1CCOCC1
ligand_258	C1CN(CCC1C(=O)NNCc1ccc(cc1)O)CC(=O)NNCc1ccc(cc1)O
ligand_276	C(#N)c1ccc(cc1)OCC(=O)N1CCC(CC1)C(=O)Nc1ccc(cc1)S(=O)(=O)N
ligand_253	c1cc(oc1C(=O)N/N=C(\C)/c1ccc(cc1)O)C(=O)N/N=C(\C)/c1ccc(cc1)O
ligand_269	c1c(c(C)nc(c1C(=O)NNCc1ccc(cc1)O)C)C(=O)NNCc1ccc(cc1)O
ligand_33	c1nc(c2c3c(CNCC3)sc2n1)N1CCC[C@H](C1)C(=O)NCCCO
ligand_34	c1cc(ccc1/C=C\1/C(=O)N=C(S1)N)OC(=O)C[C@@H]1C(=O)NC(=O)S1
ligand_287	c1(nc2c(c(=O)[nH]1)CCN(C(=O)[C@@H](N)Cc1ccc(cc1)O)C2)c1ccncc1
ligand_112	c12c(CN(C(=O)c3ccc(cc3)NC(=O)C)CC1)sc(n2)NC(=O)c1cnccn1
ligand_220	c1cc(ccc1[C@@H]1N(C(=O)CS1)c1ccc(cc1)O)OC(=O)C[C@@H]1C(=O)NC(=O)S1
ligand_229	C1C[C@@H](CC[C@H]1C(=O)Nc1cccc(c1)O)CNS(=O)(=O)c1ccc(cc1)NC(=O)C
ligand_300	C(#N)c1ccc(cc1)S(=O)(=O)N1CCN(CC1)Cc1ccc(cc1N(=O)=O)C(=O)N
ligand_97	C1=CC(=C[C@@H]2C1=CC(=C(Nc1ccc(c(c1)OC)OC)O2)C(=O)Nc1ccncc1)O
ligand_156	C(#N)c1c(N)oc2c(c(C)nn2)c1c1ccc(cc1)OCC(=O)Nc1cccc(c1)OC
ligand_135	C1CN(CCN1CC(=O)NC[C@@H]1CCCO1)[C@H](c1c[nH]c2ccc(cc12)OC)C(=O)O
ligand_202	C1C[C@@H](CC[C@H]1C(=O)Nc1ccc(cc1)OCC)CNC1=C(C(=O)C1=O)N1CCOCC1
ligand_247	c1cc(ccc1C(=O)NNCc1ccc(cc1)O)NNCc1ccc(cc1)O
ligand_204	C1C[C@@H](CC[C@H]1C(=O)Nc1ccc(cc1)OCC)CNC1=C(C(=O)C1=O)N1CCOCC1
ligand_153	C(#N)c1c(N)oc2c(c(C)nn2)c1c1ccc(cc1)OCC(=O)Nc1cccc(c1)OC
ligand_164	c1cc(ccc1CNNC(=O)c1ccc(cc1O)O)CNNC(=O)c1ccc(cc1O)O
ligand_179	C(#N)[C@H]1C(=C(C(=O)NC1=O)/C=N/c1ccc(cc1)S(=O)(=O)Nc1ccc(cc1)OC)C
ligand_238	c1cc(ccc1CNNC(=O)Cn1c(=S)[nH]c(C)n1)OC(=O)C[C@@H]1C(=O)NC(=O)S1

Supplimentary Table S1:
Ligand names and corresponding smile notation

Ligand Name	Smile
ligand_277	C(#N)c1ccc(cc1)OCC(=O)N1CCC(CC1)C(=O)Nc1ccc(cc1)S(=O)(=O)N
ligand_222	c1cc(ccc1[C@@H]1N(C(=O)CS1)c1ccc(cc1)O)OC(=O)C[C@H]1C(=O)NC(=O)S1
ligand_241	c1cc(cc(c1)S(=O)(=O)N1CC(=O)NCC1)NCC(=O)Nc1ccc(cc1)C(=O)N
ligand_242	C(CC(=O)NNCc1ccc(cc1)O)CC(=O)NNCc1ccc(cc1)O
ligand_184	C(=O)(NCCc1c2cc(ccc2[nH]c1)OC)[C@H]1CCN(CC1)[C@H]1Nc2cccnc2S1
ligand_296	C(=O)(Nc1cc(ccc1C(=O)O)C(=O)O)C[C@H]1C(=O)N(C(=O)S1)c1ccc(cc1)O
ligand_127	C(=O)(N1CCC[C@@H](C1)c1ccc(cn1)Nc1cnccn1)CCc1c[nH]c2ccccc12
ligand_270	c1c(c(C)nc(c1C(=O)NNCc1ccc(cc1)O)C)C(=O)NNCc1ccc(cc1)O
ligand_172	C(C(=O)Nc1ccc(cc1)OC(F)(F)F)CCC[C@@H]1[C@H]2[C@@H](CS1(=O)=O)NC(=O)N2
ligand_273	c1cc(cc2c1c(cc(=O)o2)c1ccc(cc1)OC)OCC(=O)Nc1ccc2c(cc[nH]2)c1
ligand_136	C1CN(CCN1CC(=O)NC[C@H]1CCCO1)[C@H](c1c[nH]c2ccc(cc12)OC)C(=O)O
ligand_271	C1=NN=C([C@@H]1CN1CC[C@@H](CC1)N1CCC[C@@H](C1)CO)c1ccc2cc(ccc2c1)OC
ligand_257	C1CN(CCC1C(=O)NNCc1ccc(cc1)O)CC(=O)NNCc1ccc(cc1)O
ligand_263	c1(nnc(s1)NC(=O)c1ccc(cc1)OC)CC(=O)NNCc1ccc(O)c(c1)O
ligand_293	c1cc(c(c2c1C(=O)/C(=C/c1cccc(c1)N(=O)=O)/O2)CN1CCN(CC1)CCO)O
ligand_265	c1c(c(C)nc(c1C(=O)NNCc1ccc(cc1)O)C)C(=O)NNCc1ccc(cc1)O
ligand_237	c1cc(ccc1CNNC(=O)Cn1c(=S)[nH]c(C)n1)OC(=O)C[C@@H]1C(=O)NC(=O)S1
ligand_203	C1C[C@@H](CC[C@H]1C(=O)Nc1ccc(cc1)OCC)CNC1=C(C(=O)C1=O)N1CCOCC1
ligand_201	[C@@H](c1ccc(cc1)OC)(c1c2ccccc2[nH]c1)CNC(=O)c1ccc2c(c1)NC(=O)CO2
ligand_200	[C@H](c1ccc(cc1)OC)(c1c2ccccc2[nH]c1)CNC(=O)c1ccc2c(c1)NC(=O)CO2
ligand_140	C(#N)c1c(N)oc2c(c(C)nn2)c1c1ccc(cc1)OCC(=O)Nc1ccc(cc1)OC
ligand_227	C1C[C@@H](CC[C@H]1C(=O)Nc1ccc(cc1)O)CNS(=O)(=O)c1ccc(cc1)NC(=O)C
ligand_89	c1cc(ccc1CNNC(=O)c1ccccc1O)OC(=O)C[C@@H]1C(=O)NC(=O)S1
ligand_288	c1c(c(nc2c1CN(C(=O)c1ccc(cc1)COC)CC2)N)C(=O)N1CCOCC1
ligand_232	c1(nc(=O)c2c(CN(CC2)Cc2ccc(c(c2)CN2CCN(CC2)C)O)[nH]1)c1cccnc1
ligand_147	C(#N)c1c(N)oc2c(c(C)nn2)c1c1ccc(cc1)OCC(=O)Nc1cccc(c1)OC
ligand_118	c1[nH]c(c(n1)C(=O)NNCc1ccc(c(c1)O)O)C(=O)NNCc1ccc(O)c(c1)O
ligand_76	c1cc(cc2c3c(CCN(C(=O)CCC(=O)Nc4ccccc4OC)C3)[nH]c12)OC
ligand_218	c1cc(ccc1[C@H]1N(C(=O)CS1)c1ccc(cc1)O)OC(=O)C[C@@H]1C(=O)NC(=O)S1
ligand_219	c1cc(ccc1[C@H]1N(C(=O)CS1)c1ccc(cc1)O)OC(=O)C[C@@H]1C(=O)NC(=O)S1
ligand_94	C1=CC(=C[C@@H]2C1=CC(=C(NC1=C(CC=C(C1)OC)OC)O2)C(=O)Nc1ccncc1)O
ligand_176	c1cc(cc2c1[C@H](C1=C(N(C=N[C@H]1O2)CCN1CCOCC1)N)c1ccc(cc1)OC)O
ligand_62	c1cc(ccc1CC(=O)N1CCC(CC1)O)NC(=O)Nc1ccc2OCOc2c1
ligand_190	C1C[C@@H](CC[C@H]1C(=O)Nc1ccc(cc1)OCC)CNC1=C(C(=O)C1=O)N1CCCCC1
ligand_248	c1cc(ccc1C(=O)NNCc1cccc(c1)O)NNCc1cccc(c1)O
ligand_85	C1C[C@H](CN(C1)CC(=O)Nc1ccc2OCOc2c1)C1=NN=C[C@@H]1c1cc(C)no1

Supplementary Table S2:
Detailed table of XP Docking using Schrodinger's Glide module

ZINC_ID	xLogP	ApolarDesolvation	PolarDesolvation	H Donors	H Acceptors	Charge	MolecularWeight	RotatableBonds	SMILES
ZINC18249417	4.49	12.17	-11.64	0	6	0	516.347	2	Cc1cccc(c1)[C@@H]2[C@@H]3[C@@H](C(=O)N(C3=O)c4ccc(cc4)Br)C5(O2)C(=O)c6ccccc6C5=O
ZINC20031600	2.96	5.41	-8.05	2	4	0	326.581	3	c1cc(cc(c1)Cl)NNC(=O)c2cc(cnc2)Br
ZINC09365179	3.58	0.63	-15.11	1	3	0	276.339	3	Cc1ccnc(c1)NC(=O)Cc2cccc3c2cccc3
ZINC19166762	2.91	9.65	-35.59	1	4	1	289.399	6	CC(=O)O[C@@H](CCN1CC2CC[NH+]1CC2)c3ccccc3
ZINC01700294	4.13	4.41	-31.48	1	2	1	279.407	5	C[C@@H](C[NH+](C)C)C(C#N)(c1ccccc1)c2ccccc2
ZINC12378847	2.77	6.66	-11.95	2	7	0	376.482	6	C/C(=N\N=C/1\NC(=O)[C@@H](S1)CC(=O)Nc2ccccc2OC)/C(C)(C)C
ZINC12378847	2.77	6.69	-11.16	2	7	0	376.482	6	C/C(=N\N=C\1/NC(=O)[C@@H](S1)CC(=O)Nc2ccccc2OC)/C(C)(C)C
ZINC03901268	2.67	5.71	-12.19	1	5	0	292.36	6	Cc1cc(=O)[nH]c(n1)SCCOc2ccccc2OC
ZINC03901268	3.13	3.72	-52.28	0	5	-1	291.352	6	Cc1cc(nc(n1)SCCOc2ccccc2OC)[O-]
ZINC19799526	1.71	8.15	-51.18	1	5	1	303.382	4	CCOc1ccc(cc1)N2C(=O)C[C@@H](C2=O)[NH+]3CCCCC3
ZINC19799526	1.71	5.89	-10.19	0	5	0	302.374	4	CCOc1ccc(cc1)N2C(=O)C[C@@H](C2=O)N3CCCCC3
ZINC00982962	1.92	-3.52	-14.66	1	5	0	336.388	6	CN(Cc1ccccc1)C(=O)CNS(=O)(=O)c2ccc(cc2)F
ZINC19989886	1.16	8.03	-54.39	3	7	1	377.49	8	CC[NH+](CC)CCN/C=C/1\C(=O)NC(=S)N(C1=O)c2ccccc2OC
ZINC08575396	2.27	6.7	-13.19	2	6	0	332.429	4	Cc1cc(cc(c1)NC(=O)C[C@H]2C(=O)N/C(=N/N=C(C)C)/S2)C
ZINC08575396	2.27	6.74	-12.42	2	6	0	332.429	4	Cc1cc(cc(c1)NC(=O)C[C@H]2C(=O)N/C(=N\N=C(C)C)/S2)C
ZINC19990070	0.73	1.75	-47.33	4	8	-1	372.308	4	c1cc(cc(c1)N2C(=O)/C(=C\NNC(=O)N)/C(=NC2=S)[O-])C(F)(F)F
ZINC05556455	1.9	4.5	-46.12	0	6	-1	248.262	4	CCc1c(nc2c(cnn2c1[O-])C(=O)OCC)C
ZINC19990034	4.72	9.58	-44.04	1	2	-1	284.404	3	CC(C)(C)c1ccc(cc1)C(=O)Nc2ccccc2[S-]
ZINC19990034	4.72	9.05	-6.7	1	2	0	285.412	3	CC(C)(C)c1ccc(cc1)C(=O)Nc2ccccc2S
ZINC01240782	2.25	4.48	-17.47	2	5	0	268.729	2	Cc1nnc(s1)NC(=O)Nc2ccc(cc2)Cl
ZINC01240782	1.88	1.84	-33.93	1	5	-1	267.721	3	Cc1nnc(s1)/N=C(/[N-]c2ccc(cc2)Cl)\O
ZINC19794473	3.01	7.51	-10.17	2	5	0	374.849	5	[H]/N=C/1\NC(=O)/C(=C/c2ccc(c(c2)OCc3ccccc3Cl)OC)/S1
ZINC05286115	3.33	10.24	-18.62	0	6	0	354.464	4	Cn1ccnc1Sc2c(nc3ccccc3n2)Sc4nccn4C
ZINC05286115	3.33	10.59	-32.99	1	6	1	355.472	4	Cn1cc[nH+]c1Sc2c(nc3ccccc3n2)Sc4nccn4C
ZINC05286115	3.33	10.96	-67.25	2	6	2	356.48	4	Cn1cc[nH+]c1Sc2c(nc3ccccc3n2)Sc4[nH+]ccn4C
ZINC00984053	2.52	-5.29	-7.54	1	4	0	263.728	1	c1ccc2c(c1)nc(c(n2)Cl)N3CCC[C@H](C3)O
ZINC05519407	2.9	-5.15	-8.88	1	4	0	277.755	2	c1ccc2c(c1)nc(c(n2)Cl)N3CCC[C@H](C3)CO
ZINC02758246	6.38	15.41	-19.05	1	5	0	458.602	7	CC[C@H](C)OC(=O)C1=C(NC2=C([C@@H]1c3ccc(cc3)N(C)C)C(=O)C[C@H](C2)c4ccccc4)C
ZINC02258599	1.28	-4.1	-47.85	4	6	1	341.453	9	CCOC(=O)c1c2c(sc1NC(=O)CC[NH2+]CCO)CCCC2
ZINC00841803	4.32	12.44	-18.66	2	6	0	453.589	8	CCOC(=O)CSC1=C([C@H](C(=C(N1)C)C(=O)Nc2ccccc2C)c3cccs3)C#N
ZINC06182368	1.21	-0.11	-8.68	1	5	0	267.306	4	COc1ccc2c(c1)NC(=O)[C@H](S2)CC(=O)OC
ZINC06219168	1.77	0.72	-8.86	0	4	0	262.334	3	Cn1c(=O)cc(n(c1=O)C)SCc2ccccc2
ZINC19318821	-3.35	-2.85	-23.39	4	10	0	206.114	2	[C@H]1([C@H](NC(=C([N+](=O)[O-])[N+](=O)[O-])[NH2+]1)O)[O-]
ZINC19318821	-3.35	-2.27	-47.96	3	10	-1	205.106	2	[C@H]1([C@H](NC(=C([N+](=O)[O-])[N+](=O)[O-])[NH2+]1)[O-])[O-]
ZINC19318821	-3.35	-3.36	-102.98	2	10	-2	204.098	2	[C@H]1([C@H](NC(=C([N+](=O)[O-])[N+](=O)[O-])N1)[O-])[O-]
ZINC02063189	5.08	11.41	-20.93	1	7	0	512.002	9	CC1=C([C@@H](C2=C(N1)C[C@@H](CC2=O)c3ccc(cc3)Cl)c4cc(ccc4OC)OC)C(=O)OCCOC
ZINC18141403	2.31	8.2	-14.32	2	5	0	339.42	4	Cc1cccc(c1)N2C(=O)C[C@H](C2=O)S/C(=N\c3ccccc3)/N
ZINC18141403	2.31	8.8	-13.98	2	5	0	339.42	4	Cc1cccc(c1)N2C(=O)C[C@@H](C2=O)S/C(=N/c3ccccc3)/N
ZINC18141403	2.31	8.2	-13.89	2	5	0	339.42	4	Cc1cccc(c1)N2C(=O)C[C@@H](C2=O)S/C(=N\c3ccccc3)/N
ZINC18141403	2.31	8.79	-13.99	2	5	0	339.42	4	Cc1cccc(c1)N2C(=O)C[C@H](C2=O)S/C(=N/c3ccccc3)/N
ZINC18141403	2.44	9.28	-13.93	2	5	0	339.42	5	[H]/N=C(/Nc1ccccc1)\S[C@H]2CC(=O)N(C2=O)c3cccc(c3)C
ZINC18141403	2.44	8.65	-13.03	2	5	0	339.42	5	[H]/N=C(\Nc1ccccc1)/S[C@H]2CC(=O)N(C2=O)c3cccc(c3)C
ZINC18141403	2.44	9.17	-13.4	2	5	0	339.42	5	[H]/N=C(/Nc1ccccc1)\S[C@@H]2CC(=O)N(C2=O)c3cccc(c3)C
ZINC18141403	2.44	8.64	-12.4	2	5	0	339.42	5	[H]/N=C(\Nc1ccccc1)/S[C@@H]2CC(=O)N(C2=O)c3cccc(c3)C
ZINC08442295	4.11	-4.47	-19.97	2	9	0	520.592	6	Cc1ccc(cc1C)N2C(=O)/C(=C\c3ccc(o3)c4ccc(cc4)S(=O)(=O)Nc5nccs5)/C(=O)N2
ZINC08442294	3.52	-4.76	-19.86	2	9	0	506.565	6	Cc1ccc(cc1)N2C(=O)/C(=C\c3ccc(o3)c4ccc(cc4)S(=O)(=O)Nc5nccs5)/C(=O)N2
ZINC08442293	3.42	-4.5	-21.41	2	7	0	430.917	6	Cc1cc(nc(n1)NS(=O)(=O)c2ccc(cc2)NC(=O)Cc3ccc(cc3)Cl)C
ZINC08442288	6.22	-5.13	-18.19	2	2	0	518.447	6	C/C(=N\NC(=O)c1c(c2ccc(cc2s1)Cl)Cl)/c3cccc(c3)NS(=O)(=O)c4ccccc4
ZINC00651582	6.03	8.28	-16.14	2	8	0	547.416	9	COc1ccccc1NS(=O)(=O)c2ccc(cc2)NC(=O)c3ccc(o3)COc4cc(ccc4Cl)Cl
ZINC08442281	6.12	-4.89	-21.07	2	9	0	621.513	8	CCOc1cccc(c1)c2cc(c3cc(ccc3n2)Br)C(=O)Nc4ccc(cc4)S(=O)(=O)Nc5c(c(no5)C)C
ZINC00730699	3.09	0.08	-55.73	2	10	-1	434.498	7	COc1cc(nc(n1)OC)[N-]S(=O)(=O)c2ccc(cc2)NC(=O)NC3CCCCC3
ZINC00730699	3.27	1.2	-17.92	3	10	0	435.506	6	COc1c/c(=N/S(=O)(=O)c2ccc(cc2)NC(=O)NC3CCCCC3)/[nH]c(n1)OC
ZINC08442279	1.84	-15.66	-20.82	4	11	0	510	7	c1cc(ccc1NS(=O)(=O)c2ccc(cc2)Cl)S(=O)(=O)Nc3nnc(s3)S(=O)(=O)N
ZINC08442278	4.74	-6.73	-18.53	2	6	0	430.551	6	Cc1ccc(cc1)S(=O)(=O)Nc2ccc(c(c2)NS(=O)(=O)c3ccc(cc3)C)C
ZINC08442277	3.78	-4.16	-25.05	2	8	0	462.531	7	Cc1cc(nc(n1)NS(=O)(=O)c2ccc(cc2)NC(=O)COc3cccc4c3cccc4)C
ZINC08442276	8.44	-3.97	-17.11	2	6	0	493.739	18	CCCCCCCCCCCCCCCC(=O)Nc1ccc(cc1)S(=O)(=O)Nc2nccs2
ZINC08442275	6.4	-11.57	-18.65	2	10	0	615.476	7	c1cc(ccc1S(=O)(=O)Nc2nc3ccc(cc3o2)c4ccc5c(c4)oc(n5)NS(=O)(=O)c6ccc(cc6)Cl)Cl
ZINC08442273	7.23	-9.65	-20.88	2	8	0	606.776	7	Cc1ccc(cc1)S(=O)(=O)Nc2nc3ccc(cc3s2)c4ccc5c(c4)sc(n5)NS(=O)(=O)c6ccc(cc6)C
ZINC08442272	4.24	-3.77	-15.93	2	7	0	446.912	7	COc1ccc(c(c1)OC)NC(=O)c2ccc(c(c2)S(=O)(=O)Nc3ccccc3)Cl
ZINC08442271	4.77	-5.8	-7.28	1	3	0	411.14	3	Cc1cc(ccc1Br)NS(=O)(=O)c2ccc(s2)Br
ZINC08442270	4.9	-1.25	-46.33	2	4	1	422.961	4	c1ccc(cc1)C[NH+]2CCc3c(sc4c3c(=O)[nH]c(n4)Cc5ccc(cc5)Cl)C2

Supplementary Table S2:
Detailed table of XP Docking using Schrodinger's Glide module

ZINC_ID	xLogP	ApolarDesolvation	PolarDesolvation	H Donors	H Acceptors	Charge	MolecularWeight	RotatableBonds	SMILES
ZINC08442268	3.63	-0.23	-17.15	1	7	0	444.487	8	COc1ccc2c(c1)cc(c(=O)[nH]2)CN(c3ccccc3OC)C(=O)COc4ccccc4
ZINC00634140	3.99	3.93	-19.64	0	6	0	447.531	10	C[C@H](C(=O)c1cc(c(c(c1)OC)OC)OC)C(=O)N(Cc2ccccc2)Cc3ccccc3
ZINC00634138	3.99	4.13	-19.82	0	6	0	447.531	10	C[C@@H](C(=O)c1cc(c(c(c1)OC)OC)OC)C(=O)N(Cc2ccccc2)Cc3ccccc3
ZINC01019824	4.14	2.47	-2.98	0	0	0	194.277	2	CC=C(c1ccccc1)c2ccccc2
ZINC08442267	7.14	2.42	-14.66	0	4	0	452.561	6	c1ccc(cc1)c2cc(n(n2)Cn3c(cc(n3)c4ccccc4)c5ccccc5)c6ccccc6
ZINC08442266	7.97	3.01	-9.06	0	5	0	519.689	5	C[C@H]1CC[C@H](c2c1c(n(n2)C(=O)[C@@H]3[C@@H](ON([C@@H]3c4ccccc4)c5ccccc5)C)c6ccccc6)C(C)C
ZINC08442265	7.97	2.18	-7.57	0	5	0	519.689	5	C[C@@H]1CC[C@H](c2c1c(n(n2)C(=O)[C@@H]3[C@@H](ON([C@@H]3c4ccccc4)c5ccccc5)C)c6ccccc6)C(C)C
ZINC08442264	7.97	2.96	-8.95	0	5	0	519.689	5	C[C@H]1CC[C@@H](c2c1c(n(n2)C(=O)[C@@H]3[C@@H](ON([C@@H]3c4ccccc4)c5ccccc5)C)c6ccccc6)C(C)C
ZINC08442263	7.97	2.97	-9.3	0	5	0	519.689	5	C[C@@H]1CC[C@@H](c2c1c(n(n2)C(=O)[C@@H]3[C@@H](ON([C@@H]3c4ccccc4)c5ccccc5)C)c6ccccc6)C(C)C
ZINC08442262	7.64	3.15	-7.96	0	3	0	434.583	4	C[C@H]1CC[C@H](c2c1c(nn2C(=O)/C=C/c3ccccc3)c4ccc5ccccc5c4)C(C)C
ZINC08442261	7.64	3.05	-7.78	0	3	0	434.583	4	C[C@@H]1CC[C@H](c2c1c(nn2C(=O)/C=C/c3ccccc3)c4ccc5ccccc5c4)C(C)C
ZINC08442260	7.64	3.06	-8.52	0	3	0	434.583	4	C[C@H]1CC[C@@H](c2c1c(nn2C(=O)/C=C/c3ccccc3)c4ccc5ccccc5c4)C(C)C
ZINC08442259	7.64	3.13	-8.47	0	3	0	434.583	4	C[C@@H]1CC[C@@H](c2c1c(nn2C(=O)/C=C/c3ccccc3)c4ccc5ccccc5c4)C(C)C
ZINC08442257	7.61	3.48	-9.57	0	3	0	434.583	4	C[C@H]1CC[C@@H](c2c1c(n(n2)C(=O)/C=C\c3ccccc3)c4cccc5c4cccc5)C(C)C
ZINC08442255	7.61	3.52	-9.52	0	3	0	434.583	4	C[C@@H]1CC[C@@H](c2c1c(n(n2)C(=O)/C=C\c3ccccc3)c4cccc5c4cccc5)C(C)C
ZINC08442254	7.6	2.64	-8.65	0	5	0	505.662	5	C[C@H]1CC[C@H](c2c1c(n(n2)C(=O)[C@@H]3CON([C@@H]3c4ccccc4)c5ccccc5)c6ccccc6)C(C)C
ZINC08442253	7.6	2.58	-8.54	0	5	0	505.662	5	C[C@@H]1CC[C@H](c2c1c(n(n2)C(=O)[C@@H]3CON([C@@H]3c4ccccc4)c5ccccc5)c6ccccc6)C(C)C
ZINC08442252	7.6	2.72	-7.77	0	5	0	505.662	5	C[C@H]1CC[C@@H](c2c1c(n(n2)C(=O)[C@@H]3CON([C@@H]3c4ccccc4)c5ccccc5)c6ccccc6)C(C)C
ZINC08442251	7.6	2.6	-9.12	0	5	0	505.662	5	C[C@@H]1CC[C@@H](c2c1c(n(n2)C(=O)[C@@H]3CON([C@@H]3c4ccccc4)c5ccccc5)c6ccccc6)C(C)C
ZINC08442250	7.61	3.11	-8.83	0	3	0	434.583	4	C[C@H]1CC[C@H](c2c1c(nn2C(=O)/C=C/c3ccccc3)c4cccc5c4cccc5)C(C)C
ZINC08442249	7.61	3.09	-8.94	0	3	0	434.583	4	C[C@@H]1CC[C@H](c2c1c(nn2C(=O)/C=C/c3ccccc3)c4cccc5c4cccc5)C(C)C
ZINC08442248	7.61	3.18	-9.09	0	3	0	434.583	4	C[C@H]1CC[C@@H](c2c1c(nn2C(=O)/C=C/c3ccccc3)c4cccc5c4cccc5)C(C)C
ZINC08442247	7.61	3.22	-9.13	0	3	0	434.583	4	C[C@@H]1CC[C@@H](c2c1c(nn2C(=O)/C=C/c3ccccc3)c4cccc5c4cccc5)C(C)C
ZINC08442246	9	-0.81	-13.67	1	6	0	645.869	10	Cc1ccc(cc1)S(=O)(=O)N[C@H](c2ccccc2)[C@H](Cc3ccccc3)C(=O)n4c(c5c(n4)[C@@H](CC[C@@H]5C)C(C)C)c6ccccc6
ZINC00667005	2.71	0.13	-17.64	1	10	0	445.501	8	CCn1c2c(nc1SCC(=O)Nc3ccc(cc3)C(=O)OCC)n(c(=O)n(c2=O)C)C
ZINC00667005	2.71	0.38	-53.97	2	10	1	446.509	8	CCn1c2c([nH+]c1SCC(=O)Nc3ccc(cc3)C(=O)OCC)n(c(=O)n(c2=O)C)C
ZINC08442245	9	-0.39	-12.63	1	6	0	645.869	10	Cc1ccc(cc1)S(=O)(=O)N[C@@H](c2ccccc2)[C@H](Cc3ccccc3)C(=O)n4c(c5c(n4)[C@@H](CC[C@@H]5C)C(C)C)c6ccccc6
ZINC08442244	6	-1.41	-12.13	3	8	0	547.457	5	c1ccc2c(c1)nc(n2CC(=O)Nc3cc(ccc3Br)C45CC6CC(C4)CC(C6)C5)c7c(non7)N
ZINC08442243	9	-0.49	-12.15	1	6	0	645.869	10	Cc1ccc(cc1)S(=O)(=O)N[C@H](c2ccccc2)[C@@H](Cc3ccccc3)C(=O)n4c(c5c(n4)[C@@H](CC[C@@H]5C)C(C)C)c6ccccc6
ZINC00666987	3.9	1.15	-10.92	0	9	0	420.425	6	Cn1c2c(c(=O)n(c1=O)C)n(c(n2)Oc3ccc(cc3)C(=O)OC)Cc4ccccc4
ZINC00666987	3.9	1.98	-38.13	1	9	1	421.433	6	Cn1c2c(c(=O)n(c1=O)C)n(c([nH+]2)Oc3ccc(cc3)C(=O)OC)Cc4ccccc4
ZINC08442242	9	-0.56	-13.73	1	6	0	645.869	10	Cc1ccc(cc1)S(=O)(=O)N[C@@H](c2ccccc2)[C@@H](Cc3ccccc3)C(=O)n4c(c5c(n4)[C@@H](CC[C@@H]5C)C(C)C)c6ccccc6
ZINC08442241	5.24	10.31	-13.12	1	6	0	439.581	8	CCC[C@H]([C@H](c1ccccc1)NS(=O)(=O)c2ccc(cc2)C)C(=O)n3c(cc(n3)C)C
ZINC08442240	5.24	10.06	-11.97	1	6	0	439.581	8	CCC[C@@H]([C@H](c1ccccc1)NS(=O)(=O)c2ccc(cc2)C)C(=O)n3c(cc(n3)C)C
ZINC08442239	6.8	-4.19	-15.29	1	6	0	494.638	5	CC(C)(C)[C@H]1CCc2c(sc3c2c(=O)[nH]c(n3)c4ccc(cc4)OS(=O)(=O)c5ccccc5)C1
ZINC08442238	5.24	10.17	-11.72	1	6	0	439.581	8	CCC[C@H]([C@@H](c1ccccc1)NS(=O)(=O)c2ccc(cc2)C)C(=O)n3c(cc(n3)C)C
ZINC08442237	6.8	-4.19	-15.25	1	6	0	494.638	5	CC(C)(C)[C@@H]1CCc2c(sc3c2c(=O)[nH]c(n3)c4ccc(cc4)OS(=O)(=O)c5ccccc5)C1
ZINC08442236	7.07	1.19	-13.19	1	6	0	462.571	5	CC(C)(C)[C@H]1CCc2c(sc3c2c(=O)[nH]c(n3)c4ccc(o4)c5ccc(cc5)C(=O)OC)C1
ZINC08442235	5.24	10.37	-12.2	1	6	0	439.581	8	CCC[C@@H]([C@@H](c1ccccc1)NS(=O)(=O)c2ccc(cc2)C)C(=O)n3c(cc(n3)C)C
ZINC08442234	7.07	1.18	-13.18	1	6	0	462.571	5	CC(C)(C)[C@@H]1CCc2c(sc3c2c(=O)[nH]c(n3)c4ccc(o4)c5ccc(cc5)C(=O)OC)C1
ZINC08442232	8.89	0.3	-11.34	1	6	0	621.243	9	CC(C)(C)[C@H]1CCc2c(sc3c2c(=O)[nH]c(n3)c4cc(c(c(c4)Cl)OCCOc5ccc(cc5)C6CCCCC6)OC)C1
ZINC08442231	8.89	0.3	-11.35	1	6	0	621.243	9	CC(C)(C)[C@@H]1CCc2c(sc3c2c(=O)[nH]c(n3)c4cc(c(c(c4)Cl)OCCOc5ccc(cc5)C6CCCCC6)OC)C1
ZINC08442230	8.13	3.77	-6.49	0	4	0	520.765	6	C[C@@H]1CC[C@H](c2c1c(nn2Cn3c4c(c(n3)c5ccccc5)[C@@H](CC[C@@H]4C(C)C)C)c6ccccc6)C(C)C
ZINC08442229	8.12	18.86	-12.51	0	4	0	520.765	6	C[C@@H]1CC[C@@H](c2c1c(nn2Cn3c4c(c(n3)c5ccccc5)[C@H](CC[C@@H]4C(C)C)C)c6ccccc6)C(C)C
ZINC08442228	5.9	-4.8	-16.15	1	6	0	466.584	4	Cc1ccc(cc1)S(=O)(=O)Oc2cccc(c2)c3[nH]c(=O)c4c5c(sc4n3)C[C@H](CC5)C
ZINC08442227	5.9	-4.8	-15.78	1	6	0	466.584	4	Cc1ccc(cc1)S(=O)(=O)Oc2cccc(c2)c3[nH]c(=O)c4c5c(sc4n3)C[C@@H](CC5)C
ZINC08442226	8.13	3.56	-8.4	0	4	0	520.765	6	C[C@@H]1CC[C@@H](c2c1c(nn2Cn3c4c(c(n3)c5ccccc5)[C@@H](CC[C@@H]4C(C)C)C)c6ccccc6)C(C)C
ZINC08442225	6.33	-3.6	-16.21	0	6	0	517.415	4	Cc1ccc(cc1)S(=O)(=O)Oc2c(cc(cc2Cl)Cl)/C=c\3/c(=O)n4c5ccccc5nc4s3
ZINC08442224	7.27	3.27	-7.34	0	3	0	446.66	6	C[C@H]1CC[C@H](c2c1c(n(n2)C(=O)[C@@H](C)[C@@H](C)Sc3ccccc3)c4ccccc4)C(C)C
ZINC02124724	7.24	-0.9	-12.09	1	5	0	503.023	5	COc1cc(cc(c1OCc2cccc3c2cccc3)Cl)c4[nH]c(=O)c5c6c(sc5n4)CCCC6
ZINC08442223	7.27	3.22	-7.21	0	3	0	446.66	6	C[C@@H]1CC[C@H](c2c1c(n(n2)C(=O)[C@@H](C)[C@@H](C)Sc3ccccc3)c4ccccc4)C(C)C
ZINC02124722	6.2	-0.23	-10.87	1	5	0	470.953	5	COc1cc(cc(c1OCc2ccccc2F)Cl)c3[nH]c(=O)c4c5c(sc4n3)CCCC5
ZINC02124713	5.28	12.49	-20.1	1	9	0	493.541	8	COc1ccccc1OCCOc2ccc(cc2c3[nH]c(=O)c4c5c(sc4n3)CCCC5)[N+](=O)[O-]
ZINC08442222	7.27	3.24	-7.27	0	3	0	446.66	6	C[C@H]1CC[C@@H](c2c1c(n(n2)C(=O)[C@@H](C)[C@@H](C)Sc3ccccc3)c4ccccc4)C(C)C
ZINC02124712	6.54	13.06	-11.09	1	4	0	485.378	4	c1ccc(c(c1)COc2ccc(cc2c3[nH]c(=O)c4c5c(sc4n3)CCCC5)Br)F
ZINC02124706	6.92	13.37	-10.95	1	4	0	457.382	4	c1ccc(c(c1)COc2ccc(cc2c3[nH]c(=O)c4c5c(sc4n3)CCCC5)Cl)Cl
ZINC08442221	7.27	3.21	-6.9	0	3	0	446.66	6	C[C@@H]1CC[C@@H](c2c1c(n(n2)C(=O)[C@@H](C)[C@@H](C)Sc3ccccc3)c4ccccc4)C(C)C
ZINC02124704	6.08	-1.05	-10.68	1	5	0	452.963	5	COc1cc(cc(c1OCc2ccccc2)Cl)c3[nH]c(=O)c4c5c(sc4n3)CCCC5
ZINC00666178	5.05	-0.53	-15.14	1	6	0	438.53	5	COc1cc(ccc1OC(=O)c2cccs2)c3[nH]c(=O)c4c5c(sc4n3)CCCC5
ZINC02124702	6.7	-0.81	-10.49	1	5	0	529.431	6	CCOc1cc(cc(c1OCc2ccccc2F)Br)c3[nH]c(=O)c4c5c(sc4n3)CCCC5
ZINC08442220	7.37	2.39	-14.31	0	8	0	572.665	10	COc1ccc(cc1)c2cc(n(n2)Cn3c(cc(n3)c4ccc(cc4)OC)c5ccc(cc5)OC)c6ccc(cc6)OC

Supplementary Table S2:
Detailed table of XP Docking using Schrodinger's Glide module

ZINC_ID	xLogP	ApolarDesolvation	PolarDesolvation	H Donors	H Acceptors	Charge	MolecularWeight	RotatableBonds	SMILES
ZINC02124700	7.02	-1.68	-14.01	1	6	0	517.434	7	COc1cccc(c1)OCCOc2c(cc(cc2Cl)c3[nH]c(=O)c4c5c(sc4n3)CCCC5)Cl
ZINC08442219	4.41	3.54	-4.45	0	2	0	222.291	2	Cc1ccc(cc1)N=C=Nc2ccc(cc2)C
ZINC02124697	6.59	12.81	-12.97	1	4	0	485.378	4	c1cc(ccc1COc2ccc(cc2c3[nH]c(=O)c4c5c(sc4n3)CCCC5)Br)F
ZINC08442218	3.26	-1.82	-23.14	2	7	0	423.494	6	CCOc1cc(ccc1OCC(=O)N)/C=c/2\c(=O)n3c4ccc(c(c4nc3s2)C)C
ZINC00634115	4.75	-1.49	-8	0	5	0	429.314	3	Cc1c2cc(ccc2oc1C(=O)N3CCN(CC3)c4ccc(cc4)OC)Br
ZINC08442217	3.64	-1.24	-12.36	0	9	0	451.593	10	CCCCCCCCn1c2c(nc1SCC(=O)N3CCOCC3)n(c(=O)n(c2=O)C)C
ZINC08442217	3.64	-0.99	-47.62	1	9	1	452.601	10	CCCCCCCCn1c2c([nH+]c1SCC(=O)N3CCOCC3)n(c(=O)n(c2=O)C)C
ZINC00634040	5.1	2.96	-13.91	0	6	0	467.521	8	CCOc1cc(ccc1OCc2ccccc2)[C@H]3c4c(=O)c5ccccc5oc4C(=O)N3CC=C
ZINC00634042	5.1	3	-13.9	0	6	0	467.521	8	CCOc1cc(ccc1OCc2ccccc2)[C@@H]3c4c(=O)c5ccccc5oc4C(=O)N3CC=C
ZINC00634032	5.15	3.27	-13.61	0	6	0	467.521	7	Cc1ccc2c(c1)c(=O)c3c(o2)C(=O)N([C@H]3c4ccc(c(c4)OC)OCc5ccccc5)CC=C
ZINC00634034	5.15	3.31	-13.72	0	6	0	467.521	7	Cc1ccc2c(c1)c(=O)c3c(o2)C(=O)N([C@@H]3c4ccc(c(c4)OC)OCc5ccccc5)CC=C
ZINC08442216	5.32	-1.56	-28.52	1	6	0	525.432	5	Cc1ccc(cc1Cl)NC(=O)COc2ccc(cc2[C@@H]3C4C(=NC5=C3C(=O)CCC5)CCCC4=O)Cl
ZINC08442216	5.48	-2.98	-24.74	1	6	0	525.432	5	Cc1ccc(cc1Cl)NC(=O)COc2ccc(cc2[C@H]3C4C(=NC5=CCCC(=O)C35)CCCC4=O)Cl
ZINC08442214	5.27	12.93	-58.27	1	6	-1	489.979	5	Cc1cccc(c1)NC(=O)COc2ccc(cc2[C@H]3C4=C(CCCC4=O)N=C5C3=C(CCC5)[O-])Cl
ZINC08442214	4.85	-2.87	-23.19	1	6	0	490.987	5	Cc1cccc(c1)NC(=O)COc2ccc(cc2[C@H]3C4C(=NC5=CCCC(=O)C35)CCCC4=O)Cl
ZINC00634014	3.51	-1	-19.81	2	7	0	437.569	9	CCn1c(nnc1SCC(=O)Nc2ccc(cc2)C)CCNC(=O)c3ccccc3C
ZINC08442211	5.45	-2.17	-28.37	1	6	0	569.883	5	Cc1ccc(cc1Cl)NC(=O)COc2ccc(cc2[C@@H]3C4C(=NC5=C3C(=O)CCC5)CCCC4=O)Br
ZINC08442211	5.61	-3.6	-24.69	1	6	0	569.883	5	Cc1ccc(cc1Cl)NC(=O)COc2ccc(cc2[C@H]3C4C(=NC5=CCCC(=O)C35)CCCC4=O)Br
ZINC00634012	3.53	-0.91	-20.78	2	7	0	437.569	9	CCn1c(nnc1SCC(=O)Nc2ccc(cc2)C)CCNC(=O)c3cccc(c3)C
ZINC08442210	2.83	-1.89	-73.24	0	6	-1	445.289	4	c1cc(c(cc1Br)[C@@H]2C3C(=NC4=C2C(=O)CCC4)CCCC3=O)OCC(=O)[O-]
ZINC08442210	3	-3.31	-66.73	0	6	-1	445.289	4	c1cc(c(cc1Br)[C@H]2C3C(=NC4=CCCC(=O)C24)CCCC3=O)OCC(=O)[O-]
ZINC00634008	3.56	-0.91	-20.56	2	7	0	437.569	9	CCn1c(nnc1SCC(=O)Nc2ccc(cc2)C)CCNC(=O)c3ccc(cc3)C
ZINC08442209	5.03	-1.98	-22.11	1	7	0	565.464	7	CCOc1cc(cc(c1OCC(=O)Nc2ccccc2)Br)[C@@H]3C4C(=NC5=C3C(=O)CCC5)CCCC4=O
ZINC08442209	5.19	-3.19	-19.62	1	7	0	565.464	7	CCOc1cc(cc(c1OCC(=O)Nc2ccccc2)Br)[C@H]3C4C(=NC5=CCCC(=O)C35)CCCC4=O
ZINC00634006	3.53	-0.93	-20.6	2	7	0	437.569	9	CCn1c(nnc1SCC(=O)Nc2cccc(c2)C)CCNC(=O)c3ccc(cc3)C
ZINC08442208	5.45	-1.66	-23.3	1	7	0	579.491	7	CCOc1cc(cc(c1OCC(=O)Nc2cccc(c2)C)Br)[C@@H]3C4C(=NC5=C3C(=O)CCC5)CCCC4=O
ZINC08442208	5.62	-2.87	-19.07	1	7	0	579.491	7	CCOc1cc(cc(c1OCC(=O)Nc2cccc(c2)C)Br)[C@H]3C4C(=NC5=CCCC(=O)C35)CCCC4=O
ZINC00634001	2.83	8.39	-15.12	2	7	0	435.962	8	CCn1c(nnc1SCC(=O)Nc2ccc(cc2)Cl)CNC(=O)c3cccs3
ZINC00633999	2.41	-2.79	-13.96	2	7	0	421.935	7	Cn1c(nnc1SCC(=O)Nc2ccccc2Cl)CNC(=O)c3cccs3
ZINC08442207	4.52	-1.4	-22.3	1	7	0	506.986	6	COc1cc(cc(c1OCC(=O)Nc2ccccc2)Cl)[C@@H]3C4C(=NC5=C3C(=O)CCC5)CCCC4=O
ZINC08442207	4.69	-2.61	-20.03	1	7	0	506.986	6	COc1cc(cc(c1OCC(=O)Nc2ccccc2)Cl)[C@H]3C4C(=NC5=CCCC(=O)C35)CCCC4=O
ZINC08442206	5.58	-1.23	-23.49	1	7	0	555.458	6	Cc1ccc(cc1Cl)NC(=O)COc2c(cc(cc2Cl)[C@@H]3C4C(=NC5=C3C(=O)CCC5)CCCC4=O)OC
ZINC08442206	5.74	-2.45	-21.37	1	7	0	555.458	6	Cc1ccc(cc1Cl)NC(=O)COc2c(cc(cc2Cl)[C@H]3C4C(=NC5=CCCC(=O)C35)CCCC4=O)OC
ZINC00633997	2.43	-2.94	-16.62	2	7	0	421.935	7	Cn1c(nnc1SCC(=O)Nc2cccc(c2)Cl)CNC(=O)c3cccs3
ZINC08442205	4.95	-1.08	-22.23	1	7	0	521.013	6	Cc1cccc(c1)NC(=O)COc2c(cc(cc2Cl)[C@@H]3C4C(=NC5=C3C(=O)CCC5)CCCC4=O)OC
ZINC08442205	5.11	-2.29	-19.9	1	7	0	521.013	6	Cc1cccc(c1)NC(=O)COc2c(cc(cc2Cl)[C@H]3C4C(=NC5=CCCC(=O)C35)CCCC4=O)OC
ZINC08442204	2.83	-2.36	-15.04	2	7	0	435.962	7	Cc1cc(ccc1NC(=O)CSc2nnc(n2C)CNC(=O)c3cccs3)Cl
ZINC08442203	5.71	-1.83	-23.28	1	7	0	599.909	6	Cc1ccc(cc1Cl)NC(=O)COc2c(cc(cc2Br)[C@@H]3C4C(=NC5=C3C(=O)CCC5)CCCC4=O)OC
ZINC08442203	5.87	-3.04	-21.17	1	7	0	599.909	6	Cc1ccc(cc1Cl)NC(=O)COc2c(cc(cc2Br)[C@H]3C4C(=NC5=CCCC(=O)C35)CCCC4=O)OC
ZINC00633992	2.46	7.48	-15.64	2	7	0	421.935	7	Cn1c(nnc1SCC(=O)Nc2ccc(cc2)Cl)CNC(=O)c3cccs3
ZINC08442202	4.87	-2.51	-23.13	1	6	0	521.411	5	c1ccc(cc1)NC(=O)COc2ccc(cc2Br)[C@@H]3C4C(=NC5=C3C(=O)CCC5)CCCC4=O
ZINC08442202	5.03	-3.59	-20.21	1	6	0	521.411	5	c1ccc(cc1)NC(=O)COc2ccc(cc2Br)[C@H]3C4C(=NC5=CCCC(=O)C35)CCCC4=O
ZINC08442201	1.88	-3.19	-22.5	2	8	0	434.572	10	C=CCn1c(nnc1SCC(=O)Nc2nccs2)CCNC(=O)c3cccs3
ZINC08442200	2.1	-3.18	-19.73	2	8	0	448.599	10	Cc1csc(n1)NC(=O)CSc2nnc(n2CC=C)CCNC(=O)c3cccs3
ZINC08442199	5.29	-2.2	-22.99	1	6	0	535.438	5	Cc1cccc(c1)NC(=O)COc2ccc(cc2Br)[C@@H]3C4C(=NC5=C3C(=O)CCC5)CCCC4=O
ZINC08442199	5.46	-3.28	-20.18	1	6	0	535.438	5	Cc1cccc(c1)NC(=O)COc2ccc(cc2Br)[C@H]3C4C(=NC5=CCCC(=O)C35)CCCC4=O
ZINC08442198	1.24	-3.8	-23.65	2	8	0	408.534	8	Cn1c(nnc1SCC(=O)Nc2nccs2)CCNC(=O)c3cccs3
ZINC08442197	5.98	-1.89	-22.79	1	7	0	646.909	6	Cc1ccc(cc1Cl)NC(=O)COc2c(cc(cc2I)[C@@H]3C4C(=NC5=C3C(=O)CCC5)CCCC4=O)OC
ZINC08442197	6.15	-3.1	-20.6	1	7	0	646.909	6	Cc1ccc(cc1Cl)NC(=O)COc2c(cc(cc2I)[C@H]3C4C(=NC5=CCCC(=O)C35)CCCC4=O)OC
ZINC08442196	1.46	6.59	-21.11	2	8	0	422.561	8	Cc1csc(n1)NC(=O)CSc2nnc(n2C)CCNC(=O)c3cccs3
ZINC08442195	3.57	-2.38	-72.88	0	6	-1	524.185	4	c1c(cc(c(c1[C@@H]2C3C(=NC4=C2C(=O)CCC4)CCCC3=O)OCC(=O)[O-])Br)Br
ZINC08442195	3.74	-3.84	-67.21	0	6	-1	524.185	4	c1c(cc(c(c1[C@H]2C3C(=NC4=CCCC(=O)C24)CCCC3=O)OCC(=O)[O-])Br)Br
ZINC08442194	1.83	-3.62	-19.97	2	8	0	436.588	9	CCn1c(nnc1SCC(=O)Nc2nc(cs2)C)CCNC(=O)c3cccs3
ZINC00633984	4.19	7.74	-17.78	2	6	0	437.294	6	COc1ccc(cc1)c2nc([nH]n2)SCC(=O)Nc3ccc(cc3F)Br
ZINC08442192	5.3	-2.04	-21.54	1	7	0	612.464	7	CCOc1cc(cc(c1OCC(=O)Nc2ccccc2)I)[C@@H]3C4C(=NC5=C3C(=O)CCC5)CCCC4=O
ZINC08442192	5.47	-3.25	-19.18	1	7	0	612.464	7	CCOc1cc(cc(c1OCC(=O)Nc2ccccc2)I)[C@H]3C4C(=NC5=CCCC(=O)C35)CCCC4=O
ZINC00633982	2.86	-2.92	-14.98	2	7	0	464.378	8	Cn1c(nnc1SCC(=O)NCc2ccccc2)CNC(=O)c3ccc(c(c3)Cl)Cl
ZINC08442191	6.39	-1.64	-23.86	1	6	0	559.877	5	Cc1ccc(cc1Cl)NC(=O)COc2c(cc(cc2Cl)[C@@H]3C4C(=NC5=C3C(=O)CCC5)CCCC4=O)Cl
ZINC08442191	6.56	-3.09	-20.35	1	6	0	559.877	5	Cc1ccc(cc1Cl)NC(=O)COc2c(cc(cc2Cl)[C@H]3C4C(=NC5=CCCC(=O)C35)CCCC4=O)Cl
ZINC00633978	2.45	-0.01	-33	2	10	0	469.523	10	Cn1c(nnc1SCC(=O)Nc2cccc(c2)C(=O)OC)CC(=O)Nc3cccc(c3)OC
ZINC08442190	4.14	0.03	-72.8	0	6	-1	501.397	4	CC1(CC2=NC3=C([C@H](C2C(=O)C1)c4cc(ccc4OCC(=O)[O-])Br)C(=O)CC(C3)(C)C)C

Supplementary Table S2:
Detailed table of XP Docking using Schrodinger's Glide module

ZINC_ID	xLogP	ApolarDesolvation	PolarDesolvation	H Donors	H Acceptors	Charge	MolecularWeight	RotatableBonds	SMILES
ZINC08442190	4.3	1.04	-79.63	0	6	-1	501.397	4	CC1(CC2=NC3=CC(CC(=O)C3[C@@H]([C@@H]2C(=O)C1)c4cc(ccc4OCC(=O)[O-])Br)(C)C)C
ZINC08442190	4.3	-0.17	-68.01	0	6	-1	501.397	4	CC1(CC2=NC3=CC(CC(=O)C3[C@@H](C2C(=O)C1)c4cc(ccc4OCC(=O)[O-])Br)(C)C)C
ZINC08442190	4.14	0.07	-71.9	0	6	-1	501.397	4	CC1(CC2=NC3=C([C@@H]([C@H]2C(=O)C1)c4cc(ccc4OCC(=O)[O-])Br)C(=O)CC(C3)(C)C)C
ZINC08442189	2.19	-1.56	-81.76	2	10	-1	454.488	9	Cn1c(nnc1SCC(=O)Nc2cccc(c2)C(=O)[O-])CC(=O)Nc3cccc(c3)OC
ZINC00633975	2.44	-2.18	-21.17	2	8	0	471.608	10	Cn1c(nnc1SCC(=O)Nc2cccc(c2)SC)CNC(=O)Cc3ccc(cc3)OC
ZINC08442188	5.57	0.18	-26.01	1	6	0	533.068	5	CC1(CC2=NC3=C([C@H](C2C(=O)C1)c4cc(ccc4OCC(=O)Nc5ccccc5)Cl)C(=O)CC(C3)(C)C)C
ZINC08442188	5.57	0.22	-25.33	1	6	0	533.068	5	CC1(CC2=NC3=C([C@@H]([C@H]2C(=O)C1)c4cc(ccc4OCC(=O)Nc5ccccc5)Cl)C(=O)CC(C3)(C)C)C
ZINC08442188	5.73	1.2	-30.62	1	6	0	533.068	5	CC1(CC2=NC3=CC(CC(=O)C3[C@@H]([C@@H]2C(=O)C1)c4cc(ccc4OCC(=O)Nc5ccccc5)Cl)(C)C)C
ZINC08442188	5.73	-0.02	-23	1	6	0	533.068	5	CC1(CC2=NC3=CC(CC(=O)C3[C@@H](C2C(=O)C1)c4cc(ccc4OCC(=O)Nc5ccccc5)Cl)(C)C)C
ZINC00633972	3.07	-0.6	-27.24	2	8	0	463.922	8	Cn1c(nnc1SCC(=O)Nc2ccc(c(c2)Cl)F)CC(=O)Nc3cccc(c3)OC
ZINC08442187	6.62	0.35	-27.17	1	6	0	581.54	5	Cc1ccc(cc1Cl)NC(=O)COc2ccc(cc2[C@@H]3C4C(=NC5=C3C(=O)CC(C5)(C)C)CC(CC4=O)(C)C)Cl
ZINC08442187	6.79	-0.6	-24.87	1	6	0	581.54	5	Cc1ccc(cc1Cl)NC(=O)COc2ccc(cc2[C@H]3C4C(=NC5=CC(CC(=O)C35)(C)C)CC(CC4=O)(C)C)Cl
ZINC00633970	4.11	0.67	-70.87	1	8	-1	453.544	10	Cc1cc(ccc1OC)CCCc2nnc(n2C)SCC(=O)Nc3cccc(c3)C(=O)[O-]
ZINC08442186	4.01	0.64	-72.78	0	6	-1	456.946	4	CC1(CC2=NC3=C([C@H](C2C(=O)C1)c4cc(ccc4OCC(=O)[O-])Cl)C(=O)CC(C3)(C)C)C
ZINC08442186	4.17	1.66	-79.54	0	6	-1	456.946	4	CC1(CC2=NC3=CC(CC(=O)C3[C@@H]([C@@H]2C(=O)C1)c4cc(ccc4OCC(=O)[O-])Cl)(C)C)C
ZINC08442186	4.17	0.43	-67.97	0	6	-1	456.946	4	CC1(CC2=NC3=CC(CC(=O)C3[C@@H](C2C(=O)C1)c4cc(ccc4OCC(=O)[O-])Cl)(C)C)C
ZINC08442186	4.01	0.68	-71.84	0	6	-1	456.946	4	CC1(CC2=NC3=C([C@@H]([C@H]2C(=O)C1)c4cc(ccc4OCC(=O)[O-])Cl)C(=O)CC(C3)(C)C)C
ZINC08442185	3.44	-1.52	-23.91	2	8	0	478.603	8	Cc1ccc(cc1)c2csc(n2)NC(=O)CSc3nnc(n3C)CC(=O)Nc4ccccc4
ZINC08442184	3.22	-1.96	-24.3	2	8	0	478.603	8	Cc1c(nc(s1)NC(=O)CSc2nnc(n2C)CC(=O)Nc3ccccc3)c4ccccc4
ZINC08442183	5.83	0.46	-22.34	1	7	0	563.094	6	CC1(CC2=NC3=C([C@H](C2C(=O)C1)c4cc(c(c(c4)Cl)OCC(=O)Nc5ccccc5)OC)C(=O)CC(C3)(C)C)C
ZINC08442183	5.83	0.5	-20.92	1	7	0	563.094	6	CC1(CC2=NC3=C([C@@H]([C@H]2C(=O)C1)c4cc(c(c(c4)Cl)OCC(=O)Nc5ccccc5)OC)C(=O)CC(C3)(C)C)C
ZINC08442183	5.99	1.47	-26.59	1	7	0	563.094	6	CC1(CC2=NC3=CC(CC(=O)C3[C@@H]([C@@H]2C(=O)C1)c4cc(c(c(c4)Cl)OCC(=O)Nc5ccccc5)OC)(C)C)C
ZINC08442183	5.99	0.26	-19.07	1	7	0	563.094	6	CC1(CC2=NC3=CC(CC(=O)C3[C@@H](C2C(=O)C1)c4cc(c(c(c4)Cl)OCC(=O)Nc5ccccc5)OC)(C)C)C
ZINC08442182	3	-1.84	-23.89	2	8	0	464.576	8	Cn1c(nnc1SCC(=O)Nc2nc(cs2)c3ccccc3)CC(=O)Nc4ccccc4
ZINC08442181	6.88	0.62	-22.35	1	7	0	611.566	6	Cc1ccc(cc1Cl)NC(=O)COc2c(cc(cc2Cl)[C@@H]3C4C(=NC5=C3C(=O)CC(C5)(C)C)CC(CC4=O)(C)C)OC
ZINC08442181	7.04	-0.33	-20.41	1	7	0	611.566	6	Cc1ccc(cc1Cl)NC(=O)COc2c(cc(cc2Cl)[C@H]3C4C(=NC5=CC(CC(=O)C35)(C)C)CC(CC4=O)(C)C)OC
ZINC08442180	3.44	-1.52	-23.91	2	8	0	478.603	8	Cc1ccc(cc1)NC(=O)Cc2nnc(n2C)SCC(=O)Nc3nc(cs3)c4ccccc4
ZINC08442179	6.25	0.78	-21.1	1	7	0	577.121	6	Cc1cccc(c1)NC(=O)COc2c(cc(cc2Cl)[C@@H]3C4C(=NC5=C3C(=O)CC(C5)(C)C)CC(CC4=O)(C)C)OC
ZINC08442179	6.42	-0.17	-18.99	1	7	0	577.121	6	Cc1cccc(c1)NC(=O)COc2c(cc(cc2Cl)[C@H]3C4C(=NC5=CC(CC(=O)C35)(C)C)CC(CC4=O)(C)C)OC
ZINC00633968	3.81	10.34	-23.98	2	7	0	478.405	8	C[C@H](c1ccccc1)NC(=O)CSc2nnc(n2C)CC(=O)Nc3ccc(c(c3)Cl)Cl
ZINC08442178	6.17	-0.46	-21.72	1	6	0	577.519	5	CC1(CC2=NC3=C([C@H](C2C(=O)C1)c4ccc(c(c4)Br)OCC(=O)Nc5ccccc5)C(=O)CC(C3)(C)C)C
ZINC08442178	6.33	-0.66	-19.57	1	6	0	577.519	5	CC1(CC2=NC3=CC(CC(=O)C3[C@@H](C2C(=O)C1)c4ccc(c(c4)Br)OCC(=O)Nc5ccccc5)(C)C)C
ZINC08442178	6.33	0.56	-25.55	1	6	0	577.519	5	CC1(CC2=NC3=CC(CC(=O)C3[C@@H]([C@@H]2C(=O)C1)c4ccc(c(c4)Br)OCC(=O)Nc5ccccc5)(C)C)C
ZINC08442178	6.17	-0.42	-21.53	1	6	0	577.519	5	CC1(CC2=NC3=C([C@@H]([C@H]2C(=O)C1)c4ccc(c(c4)Br)OCC(=O)Nc5ccccc5)C(=O)CC(C3)(C)C)C
ZINC00633966	3.81	10.34	-23.85	2	7	0	478.405	8	C[C@@H](c1ccccc1)NC(=O)CSc2nnc(n2C)CC(=O)Nc3ccc(c(c3)Cl)Cl
ZINC08442177	6.6	-0.15	-21.69	1	6	0	591.546	5	Cc1cccc(c1)NC(=O)COc2ccc(cc2Br)[C@@H]3C4C(=NC5=C3C(=O)CC(C5)(C)C)CC(CC4=O)(C)C
ZINC08442177	6.76	-1.1	-19.37	1	6	0	591.546	5	Cc1cccc(c1)NC(=O)COc2ccc(cc2Br)[C@H]3C4C(=NC5=CC(CC(=O)C35)(C)C)CC(CC4=O)(C)C
ZINC00633964	3.25	9.48	-24	2	7	0	464.378	8	Cn1c(nnc1SCC(=O)NCc2ccccc2)CC(=O)Nc3ccc(c(c3)Cl)Cl
ZINC08442176	6.47	0.44	-21.94	1	6	0	547.095	5	Cc1cccc(c1)NC(=O)COc2ccc(cc2Cl)[C@@H]3C4C(=NC5=C3C(=O)CC(C5)(C)C)CC(CC4=O)(C)C
ZINC08442176	6.63	-0.51	-19.68	1	6	0	547.095	5	Cc1cccc(c1)NC(=O)COc2ccc(cc2Cl)[C@H]3C4C(=NC5=CC(CC(=O)C35)(C)C)CC(CC4=O)(C)C
ZINC00633955	4	-1.26	-19.41	2	7	0	470.426	7	C[C@H]1CCCC[C@H]1NC(=O)CSc2nnc(n2C)CC(=O)Nc3ccc(c(c3)Cl)Cl
ZINC00633958	4	-1.77	-23.33	2	7	0	470.426	7	C[C@@H]1CCCC[C@H]1NC(=O)CSc2nnc(n2C)CC(=O)Nc3ccc(c(c3)Cl)Cl
ZINC08442175	6.34	-0.12	-20.91	1	7	0	621.572	7	CCOc1cc(cc(c1OCC(=O)Nc2ccccc2)Br)[C@@H]3C4C(=NC5=C3C(=O)CC(C5)(C)C)CC(CC4=O)(C)C
ZINC08442175	6.5	-1.07	-18.84	1	7	0	621.572	7	CCOc1cc(cc(c1OCC(=O)Nc2ccccc2)Br)[C@H]3C4C(=NC5=CC(CC(=O)C35)(C)C)CC(CC4=O)(C)C
ZINC08442174	4	-1.81	-19.89	2	7	0	470.426	7	C[C@H]1CCCC[C@@H]1NC(=O)CSc2nnc(n2C)CC(=O)Nc3ccc(c(c3)Cl)Cl
ZINC08442173	6.76	0.2	-22.08	1	7	0	635.599	7	CCOc1cc(cc(c1OCC(=O)Nc2cccc(c2)C)Br)[C@@H]3C4C(=NC5=C3C(=O)CC(C5)(C)C)CC(CC4=O)(C)C
ZINC08442173	6.92	-0.75	-18.3	1	7	0	635.599	7	CCOc1cc(cc(c1OCC(=O)Nc2cccc(c2)C)Br)[C@H]3C4C(=NC5=CC(CC(=O)C35)(C)C)CC(CC4=O)(C)C
ZINC00633962	4	-1.26	-19.42	2	7	0	470.426	7	C[C@@H]1CCCC[C@@H]1NC(=O)CSc2nnc(n2C)CC(=O)Nc3ccc(c(c3)Cl)Cl
ZINC00633953	3.76	-2.47	-23.58	2	7	0	456.399	7	Cn1c(nnc1SCC(=O)NC2CCCCC2)CC(=O)Nc3ccc(c(c3)Cl)Cl
ZINC08442172	6	13.52	-62.78	1	6	-1	497.615	5	CC1(CC2=C([C@H](C3=C(CC(CC3=N2)(C)C)[O-])c4cccc(c4)OCC(=O)Nc5ccccc5)C(=O)C1)C
ZINC08442172	5.57	1.23	-33.68	1	6	0	498.623	5	CC1(CC2=NC3=CC(CC(=O)C3[C@@H]([C@@H]2C(=O)C1)c4cccc(c4)OCC(=O)Nc5ccccc5)(C)C)C
ZINC08442172	5.57	0.02	-25.86	1	6	0	498.623	5	CC1(CC2=NC3=CC(CC(=O)C3[C@@H](C2C(=O)C1)c4cccc(c4)OCC(=O)Nc5ccccc5)(C)C)C
ZINC08442172	5.41	0.3	-28.8	1	6	0	498.623	5	CC1(CC2=NC3=C([C@@H]([C@H]2C(=O)C1)c4cccc(c4)OCC(=O)Nc5ccccc5)C(=O)CC(C3)(C)C)C
ZINC08442171	2.83	-2.51	-25	2	8	0	471.395	7	Cc1csc(n1)NC(=O)CSc2nnc(n2C)CC(=O)Nc3ccc(c(c3)Cl)Cl
ZINC00633951	4.12	1.22	-19.24	1	7	0	464.95	9	CCOc1cc(ccc1OCc2nnc(n2C)SCC(=O)Nc3ccc(c(c3)Cl)F)C
ZINC00633947	3.76	0.28	-19.75	1	7	0	458.609	10	CCOc1cc(ccc1OCc2nnc(n2C)SCC(=O)Nc3cccc(c3)SC)C
ZINC08442169	5.84	0.52	-29.46	1	6	0	512.65	5	Cc1cccc(c1)NC(=O)COc2cccc(c2)[C@@H]3C4C(=NC5=C3C(=O)CC(C5)(C)C)CC(CC4=O)(C)C
ZINC08442169	6	-0.46	-25.62	1	6	0	512.65	5	Cc1cccc(c1)NC(=O)COc2cccc(c2)[C@H]3C4C(=NC5=CC(CC(=O)C35)(C)C)CC(CC4=O)(C)C
ZINC08442168	5.4	0.47	-28.11	1	7	0	542.676	7	CCOc1cc(ccc1OCC(=O)Nc2ccccc2)[C@@H]3C4C(=NC5=C3C(=O)CC(C5)(C)C)CC(CC4=O)(C)C
ZINC08442168	5.56	-0.52	-24.51	1	7	0	542.676	7	CCOc1cc(ccc1OCC(=O)Nc2ccccc2)[C@H]3C4C(=NC5=CC(CC(=O)C35)(C)C)CC(CC4=O)(C)C
ZINC00633946	4.64	-0.91	-12.59	1	6	0	471.38	7	Cn1c(nnc1SCC(=O)NCCc2ccccc2)c3cc4cc(ccc4o3)Br

Supplementary Table S2:
Detailed table of XP Docking using Schrodinger's Glide module

ZINC_ID	xLogP	ApolarDesolvation	PolarDesolvation	H Donors	H Acceptors	Charge	MolecularWeight	RotatableBonds	SMILES
ZINC08442167	7.7	0.27	-22.57	1	6	0	615.985	5	Cc1ccc(cc1Cl)NC(=O)COc2c(cc(cc2Cl)[C@@H]3C4C(=NC5=C3C(=O)CC(C5)(C)C)CC(CC4=O)(C)C)Cl
ZINC08442167	7.86	-0.68	-18.6	1	6	0	615.985	5	Cc1ccc(cc1Cl)NC(=O)COc2c(cc(cc2Cl)[C@H]3C4C(=NC5=CC(CC(=O)C35)(C)C)CC(CC4=O)(C)C)Cl
ZINC00633944	4.79	-0.29	-11.16	1	6	0	471.38	6	C[C@H](c1ccccc1)NC(=O)CSc2nnc(n2C)c3cc4cc(ccc4o3)Br
ZINC08442166	5.28	0.09	-14.52	0	5	0	588.164	3	CC(=O)Oc1c(cc(cc1I)/C=c\2/c(=O)n3c4ccccc4nc3s2)I
ZINC08442165	4.17	2.88	-16.84	0	6	0	420.49	7	CCOc1cc(cc(c1OC(=O)C)CC=C)/C=c\2/c(=O)n3c4ccccc4nc3s2
ZINC08442164	6.1	2.53	-15.58	0	5	0	495.579	5	Cc1cc(c(n1c2ccc(cc2)OCc3ccccc3F)C)/C=c\4/c(=O)n5c6ccccc6nc5s4
ZINC00633938	3.54	1.33	-16.81	1	7	0	499.337	8	Cn1c(nnc1SCC(=O)Nc2c(cc(cc2Br)F)F)COc3ccccc3OC
ZINC00633936	3.98	-0.92	-16.25	0	7	0	465.623	9	Cn1c(nnc1SCC(=O)N2CCN(CC2)c3ccccc3)CCCc4ccc(cc4)OC
ZINC08442163	3.97	10	-10.73	1	5	0	465.097	5	c1cc(ccc1C(=O)COC(=O)c2cc(=O)[nH]c3c2cc(cc3)Br)Br
ZINC00977563	3.71	8.97	-26.36	3	6	0	452.337	5	c1ccc2c(c1)[nH]c(n2)c3ccc(cc3)NC(=S)NC(=O)c4cc(cnc4)Br
ZINC08442162	3.99	1.81	-23.44	1	8	0	454.552	11	Cn1c(nnc1SCC(=O)Nc2cccc(c2)C(=O)OC)CCCc3ccc(cc3)OC
ZINC08442161	5.97	1.17	-14.8	0	4	0	421.525	3	Cc1ccc(cc1)Cn2cc(c3c2cccc3)/C=c\4/c(=O)n5c6ccccc6nc5s4
ZINC00633932	4.51	0.67	-17.07	1	7	0	460.987	10	Cn1c(nnc1SCC(=O)Nc2cc(ccc2OC)Cl)CCCc3ccc(cc3)OC
ZINC08442160	6.49	14.52	-18.63	0	6	0	444.512	7	CCCCOC(=O)c1ccc(cc1)c2ccc(o2)/C=c\3/c(=O)n4c5ccccc5nc4s3
ZINC08442159	5.69	13.19	-12.94	0	5	0	493.382	5	COc1cc(c(cc1OCc2ccccc2)Br)/C=c/3\c(=O)n4c5ccccc5nc4s3
ZINC08442157	6.54	-0.25	-12.7	0	4	0	477.383	4	Cc1ccc(cc1)COc2ccc(cc2/C=c/3\c(=O)n4c5ccccc5nc4s3)Br
ZINC08442156	2.53	10.05	-21.48	1	9	0	495.605	8	Cc1cccc(c1C)OCc2nnc(n2C)SCC(=O)Nc3cccc(c3)C(=O)N4CCOCC4
ZINC00633925	4.73	1.64	-13.88	1	6	0	497.365	7	Cc1cccc(c1C)OCc2nnc(n2C)SCC(=O)Nc3c(cc(cc3Br)F)F
ZINC08442153	5.36	9.84	-19.63	0	12	0	573.635	6	COc1cc(ccc1Oc2nc(nc(n2)N3CCOCC3)N4CCOCC4)/C=c\5/c(=O)n6c7ccccc7nc6s5
ZINC08442152	2.82	-3.01	-19.85	1	8	0	478.622	8	Cc1ccccc1C(=O)NCCc2nnc(n2C)SCC(=O)N3CCN(CC3)c4ccccc4
ZINC08442149	3.41	11.57	-22.21	2	8	0	492.63	9	Cc1ccccc1C(=O)NCCc2nnc(n2C)SCC(=O)Nc3nc(cs3)c4ccccc4
ZINC00633923	3.35	-1.44	-20.64	2	8	0	473.986	9	Cc1ccccc1C(=O)NCCc2nnc(n2C)SCC(=O)Nc3cc(ccc3OC)Cl
ZINC00633922	2.85	-2.95	-21.18	1	8	0	478.622	8	Cc1cccc(c1)C(=O)NCCc2nnc(n2C)SCC(=O)N3CCN(CC3)c4ccccc4
ZINC08442142	2.6	10.03	-74.48	2	9	-1	452.516	9	Cc1cccc(c1)C(=O)NCCc2nnc(n2C)SCC(=O)Nc3cccc(c3)C(=O)[O-]
ZINC00633920	3.05	-3.39	-22.6	1	8	0	499.04	8	Cn1c(nnc1SCC(=O)N2CCN(CC2)c3ccccc3)CCNC(=O)c4ccccc4Cl
ZINC08442138	2.87	-2.97	-20.77	1	8	0	478.622	8	Cc1ccc(cc1)C(=O)NCCc2nnc(n2C)SCC(=O)N3CCN(CC3)c4ccccc4
ZINC08442137	7.38	2.04	-7.83	0	3	0	492.233	7	c1cc(ccc1C(c2ccc(cc2)Cl)O[C@H](Cn3ccnc3)c4ccc(cc4Cl)Cl)Cl
ZINC08442137	7.38	2.32	-38.45	1	3	1	493.241	7	c1cc(ccc1C(c2ccc(cc2)Cl)O[C@H](Cn3cc[nH+]c3)c4ccc(cc4Cl)Cl)Cl
ZINC08442136	7.38	1.96	-7.8	0	3	0	492.233	7	c1cc(ccc1C(c2ccc(cc2)Cl)O[C@@H](Cn3ccnc3)c4ccc(cc4Cl)Cl)Cl
ZINC08442136	7.38	2.24	-38.45	1	3	1	493.241	7	c1cc(ccc1C(c2ccc(cc2)Cl)O[C@@H](Cn3cc[nH+]c3)c4ccc(cc4Cl)Cl)Cl
ZINC08442135	3.46	11.58	-23.21	2	8	0	492.63	9	Cc1ccc(cc1)C(=O)NCCc2nnc(n2C)SCC(=O)Nc3nc(cs3)c4ccccc4
ZINC08442133	2.62	10.04	-74.22	2	9	-1	452.516	9	Cc1ccc(cc1)C(=O)NCCc2nnc(n2C)SCC(=O)Nc3cccc(c3)C(=O)[O-]
ZINC00633914	3.63	-1.14	-15.19	1	6	0	461.385	8	Cn1c(nnc1SCc2ccc(cc2)Br)CNC(=O)Cc3ccc(cc3)OC
ZINC00633912	4.1	-0.72	-14.98	1	6	0	451.379	8	Cn1c(nnc1SCc2ccc(cc2Cl)Cl)CNC(=O)Cc3ccc(cc3)OC
ZINC02851694	4	-1.02	-14.49	1	6	0	475.412	9	CCn1c(nnc1SCc2ccc(cc2)Br)CNC(=O)Cc3ccc(cc3)OC
ZINC00633907	3.25	-2.81	-20.19	1	8	0	492.649	9	CCn1c(nnc1SCC(=O)N2CCN(CC2)c3ccccc3)CCNC(=O)c4ccc(cc4)C
ZINC00633905	3.1	-3.42	-20.45	1	8	0	499.04	8	Cn1c(nnc1SCC(=O)N2CCN(CC2)c3ccccc3)CCNC(=O)c4ccc(cc4)Cl
ZINC00633902	4.17	-3.52	-12.04	0	7	0	466.607	8	Cc1ccc(c(c1)OCc2nnc(o2)SCC(=O)N3CCN(CC3)c4ccccc4)C(C)C
ZINC08442128	3.83	1.17	-15.71	0	7	0	428.492	5	Cn1c2c(c(=O)n(c1=O)C)n(cn2)CCCOC3c4ccccc4C=Cc5c3cccc5
ZINC08442128	3.83	1.45	-52.18	1	7	1	429.5	5	Cn1c2c(c(=O)n(c1=O)C)n(c[nH+]2)CCCOC3c4ccccc4C=Cc5c3cccc5
ZINC08442127	3.62	0.96	-14.07	0	7	0	430.508	5	Cn1c2c(c(=O)n(c1=O)C)n(cn2)CCCOC3c4ccccc4CCc5c3cccc5
ZINC08442127	3.62	1.24	-50.25	1	7	1	431.516	5	Cn1c2c(c(=O)n(c1=O)C)n(c[nH+]2)CCCOC3c4ccccc4CCc5c3cccc5
ZINC00633900	3.05	-2.64	-12.85	2	7	0	498.403	8	CCn1c(nnc1SCC(=O)Nc2ccc(cc2F)Br)CNC(=O)c3cccs3
ZINC08442126	2.71	-4.12	-17.69	2	8	0	458.594	8	CCn1c(nnc1SCC(=O)Nc2nc3ccccc3s2)CNC(=O)c4cccs4
ZINC00633899	2.68	-2.8	-13.33	2	7	0	484.376	7	Cn1c(nnc1SCC(=O)Nc2ccc(cc2F)Br)CNC(=O)c3cccs3
ZINC08442122	3.65	5.03	-11.99	4	7	0	449.923	3	Cc1ccc(cc1)SCc2c3c([nH]n2)OC(=C([C@]34c5cc(ccc5NC4=O)Cl)C#N)N
ZINC08442121	3.65	4.75	-13.09	4	7	0	449.923	3	Cc1ccc(cc1)SCc2c3c([nH]n2)OC(=C([C@@]34c5cc(ccc5NC4=O)Cl)C#N)N
ZINC08442120	3.89	8.12	-12.83	3	7	0	455.543	5	Cc1ccc(cc1)SCc2c3c([nH]n2)OC(=C([C@]34c5ccccc5N(C4=O)CC=C)C#N)N
ZINC08442119	3.89	7.95	-12.93	3	7	0	455.543	5	Cc1ccc(cc1)SCc2c3c([nH]n2)OC(=C([C@@]34c5ccccc5N(C4=O)CC=C)C#N)N
ZINC08442118	3.14	4.59	-12.4	4	7	0	433.468	3	Cc1ccc(cc1)SCc2c3c([nH]n2)OC(=C([C@]34c5cc(ccc5NC4=O)F)C#N)N
ZINC08442117	3.14	4.32	-13.57	4	7	0	433.468	3	Cc1ccc(cc1)SCc2c3c([nH]n2)OC(=C([C@@]34c5cc(ccc5NC4=O)F)C#N)N
ZINC08442116	2.89	-3.75	-17.15	2	8	0	458.594	7	C[C@H](c1nnc(n1C)SCC(=O)Nc2nc3ccccc3s2)NC(=O)c4cccs4
ZINC08442115	3.62	7.35	-13.2	3	7	0	443.532	4	CCN1c2ccccc2[C@]3(C1=O)c4c(n[nH]c4OC(=C3C#N)N)CSc5ccc(cc5)C
ZINC08442114	2.89	8.47	-17.14	2	8	0	458.594	7	C[C@@H](c1nnc(n1C)SCC(=O)Nc2nc3ccccc3s2)NC(=O)c4cccs4
ZINC08442112	3.62	7.57	-13.31	3	7	0	443.532	4	CCN1c2ccccc2[C@@]3(C1=O)c4c(n[nH]c4OC(=C3C#N)N)CSc5ccc(cc5)C
ZINC08442111	2.98	-1.85	-14.44	2	8	0	486.648	7	C[C@H](c1nnc(n1C)SCC(=O)Nc2c(c3c(s2)CCCC3)C#N)NC(=O)c4cccs4
ZINC01102227	4.45	-1.69	-15.72	3	7	0	496.523	3	COc1ccc(cc1)c2ccc3c4c(c5c(c(=O)[nH]4)[C@@H](C(=C(O5)N)C#N)c6ccc(cc6)F)sc3n2
ZINC08442110	4.26	12.54	-9.52	2	6	0	459.553	3	C[C@H]1CC=C2[C@H](C1)[C@@]3(c4ccccc4N(C3=O)CCc5ccccc5)C(C(=C2C#N)N)(C#N)C#N
ZINC08442109	4.26	12.51	-10.14	2	6	0	459.553	3	C[C@@H]1CC=C2[C@H](C1)[C@@]3(c4ccccc4N(C3=O)CCc5ccccc5)C(C(=C2C#N)N)(C#N)C#N
ZINC08442108	4.26	12.46	-10.64	2	6	0	459.553	3	C[C@H]1CC=C2[C@@H](C1)[C@@]3(c4ccccc4N(C3=O)CCc5ccccc5)C(C(=C2C#N)N)(C#N)C#N
ZINC08442108	4.34	2.7	-40.61	2	6	1	460.561	3	C[C@H]1CC=C2[C@@H](C1)[C@@]3(c4ccccc4N(C3=O)CCc5ccccc5)C(C(=[NH2+])C2C#N)(C#N)C#N

Supplementary Table S2:
Detailed table of XP Docking using Schrodinger's Glide module

ZINC_ID	xLogP	ApolarDesolvation	PolarDesolvation	H Donors	H Acceptors	Charge	MolecularWeight	RotatableBonds	SMILES
ZINC08442107	2.98	-1.87	-14.58	2	8	0	486.648	7	C[C@@H](c1nnc(n1C)SCC(=O)Nc2c(c3c(s2)CCCC3)C#N)NC(=O)c4cccs4
ZINC08442106	4.26	11.57	-10.66	2	6	0	459.553	3	C[C@@H]1CC=C2[C@@H](C1)[C@@]3(c4ccccc4N(C3=O)CCc5ccccc5)C(C(=C2C#N)N)(C#N)C#N
ZINC08442106	4.34	2.33	-41.22	2	6	1	460.561	3	C[C@@H]1CC=C2[C@@H](C1)[C@@]3(c4ccccc4N(C3=O)CCc5ccccc5)C(C(=[NH2+])C2C#N)(C#N)C#N
ZINC08442105	3.06	-1.77	-14.71	2	8	0	498.659	9	C=CCn1c(nnc1SCC(=O)Nc2c(c3c(s2)CCCC3)C#N)CNC(=O)c4cccs4
ZINC08442104	4.64	0.43	-11.71	1	6	0	491.997	8	CCOC(=O)C1[C@@H](C(=C(N=C1C)SCC(=O)Nc2ccc(cc2F)Cl)C#N)c3cccs3
ZINC08442104	5.21	-1.23	-10.26	1	6	0	491.997	8	CCOC(=O)C1[C@@H](C(C(=NC1=C)SCC(=O)Nc2ccc(cc2F)Cl)C#N)c3cccs3
ZINC08442104	5.03	-0.39	-9.9	1	6	0	491.997	8	CCOC(=O)C1=C(N=C(C([C@H]1c2cccs2)C#N)SCC(=O)Nc3ccc(cc3F)Cl)C
ZINC08442103	3.54	-3.15	-16.37	2	8	0	484.632	9	C[C@H](c1nnc(n1CC=C)SCC(=O)Nc2nc3ccccc3s2)NC(=O)c4cccs4
ZINC08442102	4.64	0.43	-11.87	1	6	0	491.997	8	CCOC(=O)C1[C@H](C(=C(N=C1C)SCC(=O)Nc2ccc(cc2F)Cl)C#N)c3cccs3
ZINC08442102	5.21	-1.42	-11.89	1	6	0	491.997	8	CCOC(=O)C1[C@H](C(C(=NC1=C)SCC(=O)Nc2ccc(cc2F)Cl)C#N)c3cccs3
ZINC08442102	5.03	-0.44	-10.41	1	6	0	491.997	8	CCOC(=O)C1=C(N=C(C([C@@H]1c2cccs2)C#N)SCC(=O)Nc3ccc(cc3F)Cl)C
ZINC08442101	3.54	-3.15	-16.42	2	8	0	484.632	9	C[C@@H](c1nnc(n1CC=C)SCC(=O)Nc2nc3ccccc3s2)NC(=O)c4cccs4
ZINC08442100	4.82	2.34	-12.41	1	5	0	414.412	5	Cc1ccc2c(c1)nc([nH]2)CSc3c(c(cc(n3)c4ccco4)C(F)(F)F)C#N
ZINC08442099	4.89	10.47	-16.2	1	6	0	470.476	7	COc1ccc(cc1OC)c2cc(c(c(n2)SCc3[nH]c4ccccc4n3)C#N)C(F)(F)F
ZINC08442098	3.27	-3.61	-16.75	2	8	0	472.621	8	CCn1c(nnc1SCC(=O)Nc2nc3ccccc3s2)[C@H](C)NC(=O)c4cccs4
ZINC08442097	3.94	7.17	-12.02	3	7	0	428.492	5	COc1cc(ccc1OC2CCCC2)[C@H]3c4c(n[nH]c4OC(=C3C#N)N)c5ccccc5
ZINC08442096	3.94	7.24	-11.78	3	7	0	428.492	5	COc1cc(ccc1OC2CCCC2)[C@@H]3c4c(n[nH]c4OC(=C3C#N)N)c5ccccc5
ZINC08442095	3.27	-3.59	-16.69	2	8	0	472.621	8	CCn1c(nnc1SCC(=O)Nc2nc3ccccc3s2)[C@@H](C)NC(=O)c4cccs4
ZINC00664784	4.81	6.27	-10.96	3	7	0	444.878	4	COc1ccc(cc1)c2c3c(n[nH]2)OC(=C([C@H]3c4ccc(o4)c5ccc(cc5)Cl)C#N)N
ZINC00664787	4.81	6.25	-11.12	3	7	0	444.878	4	COc1ccc(cc1)c2c3c(n[nH]2)OC(=C([C@@H]3c4ccc(o4)c5ccc(cc5)Cl)C#N)N
ZINC00664768	4.66	6.34	-10.02	3	6	0	465.332	3	c1cc(sc1)c2c3c(n[nH]2)OC(=C([C@@H]3c4ccc(o4)c5ccc(cc5)Br)C#N)N
ZINC08442094	3.64	-3.11	-16.5	2	8	0	486.648	8	CC(C)[C@H](c1nnc(n1C)SCC(=O)Nc2nc3ccccc3s2)NC(=O)c4cccs4
ZINC08670819	3.26	4.25	-12.69	3	7	0	417.856	2	CCN1c2ccccc2[C@]3(C1=O)c4c([nH]nc4OC(=C3C#N)N)c5ccc(cc5)Cl
ZINC08670819	3.26	4.97	-13.38	3	7	0	417.856	2	CCN1c2ccccc2[C@]3(C1=O)c4c(n[nH]c4OC(=C3C#N)N)c5ccc(cc5)Cl
ZINC08670818	3.26	4.82	-13.07	3	7	0	417.856	2	CCN1c2ccccc2[C@@]3(C1=O)c4c([nH]nc4OC(=C3C#N)N)c5ccc(cc5)Cl
ZINC08670818	3.26	5.6	-13.71	3	7	0	417.856	2	CCN1c2ccccc2[C@@]3(C1=O)c4c(n[nH]c4OC(=C3C#N)N)c5ccc(cc5)Cl
ZINC08442092	3.64	-3.61	-16.31	2	8	0	486.648	8	CC(C)[C@@H](c1nnc(n1C)SCC(=O)Nc2nc3ccccc3s2)NC(=O)c4cccs4
ZINC00664754	4.96	8.87	-12.33	3	7	0	478.552	6	Cc1ccc(cc1C)c2c3c(n[nH]2)OC(=C([C@H]3c4ccc(c(c4)OC)OCc5ccccc5)C#N)N
ZINC00664757	4.96	8.88	-12.27	3	7	0	478.552	6	Cc1ccc(cc1C)c2c3c(n[nH]2)OC(=C([C@@H]3c4ccc(c(c4)OC)OCc5ccccc5)C#N)N
ZINC08442091	3.09	10	-14.57	2	7	0	455.609	11	C[C@H](c1nnc(n1CC=C)SCC(=O)NCCc2ccccc2)NC(=O)c3cccs3
ZINC00664736	3.55	4.86	-10.58	3	8	0	432.48	5	Cc1ccc(cc1C)c2c3c(n[nH]2)OC(=C([C@H]3c4ccc(c(c4OC)OC)OC)C#N)N
ZINC00664738	3.55	4.53	-10.99	3	8	0	432.48	5	Cc1ccc(cc1C)c2c3c(n[nH]2)OC(=C([C@@H]3c4ccc(c(c4OC)OC)OC)C#N)N
ZINC08442090	3.09	9.99	-14.43	2	7	0	455.609	11	C[C@@H](c1nnc(n1CC=C)SCC(=O)NCCc2ccccc2)NC(=O)c3cccs3
ZINC08442089	4.25	6.6	-11.11	3	7	0	430.508	6	CCCOc1ccc(cc1OC)[C@H]2c3c([nH]nc3OC(=C2C#N)N)c4ccc(c(c4)C)C
ZINC08442088	4.25	6.62	-10.99	3	7	0	430.508	6	CCCOc1ccc(cc1OC)[C@@H]2c3c([nH]nc3OC(=C2C#N)N)c4ccc(c(c4)C)C
ZINC08442087	3.29	10.61	-22.1	2	8	0	498.659	10	CCn1c(nnc1SCC(=O)Nc2nc(cs2)c3ccccc3)CCNC(=O)c4cccs4
ZINC00664726	6	9.35	-9.55	3	6	0	482.971	5	Cc1ccc(cc1C)c2c3c(n[nH]2)OC(=C([C@H]3c4ccccc4OCc5ccc(cc5)Cl)C#N)N
ZINC00664729	6	9.31	-9.85	3	6	0	482.971	5	Cc1ccc(cc1C)c2c3c(n[nH]2)OC(=C([C@@H]3c4ccccc4OCc5ccc(cc5)Cl)C#N)N
ZINC00633895	3.19	-2.52	-18.3	2	7	0	449.989	9	CCn1c(nnc1SCC(=O)Nc2ccccc2Cl)CCNC(=O)c3cccs3
ZINC00664717	4.53	6.32	-7.67	3	5	0	421.298	2	Cc1ccc(cc1C)c2c3c(n[nH]2)OC(=C([C@H]3c4ccc(cc4)Br)C#N)N
ZINC00664719	4.53	6.34	-7.59	3	5	0	421.298	2	Cc1ccc(cc1C)c2c3c(n[nH]2)OC(=C([C@@H]3c4ccc(cc4)Br)C#N)N
ZINC00633893	3.84	-2.82	-20.68	2	7	0	484.434	9	CCn1c(nnc1SCC(=O)Nc2ccc(c(c2)Cl)Cl)CCNC(=O)c3cccs3
ZINC00664707	4.62	6.35	-8.3	3	5	0	439.288	2	Cc1ccc(cc1C)c2c3c(n[nH]2)OC(=C([C@H]3c4ccc(c(c4)Br)F)C#N)N
ZINC00664709	4.62	6.37	-8.17	3	5	0	439.288	2	Cc1ccc(cc1C)c2c3c(n[nH]2)OC(=C([C@@H]3c4ccc(c(c4)Br)F)C#N)N
ZINC00633892	3.84	-2.67	-17.13	2	7	0	484.434	9	CCn1c(nnc1SCC(=O)Nc2ccc(cc2Cl)Cl)CCNC(=O)c3cccs3
ZINC08442086	5.18	6.45	-6.3	3	5	0	451.368	2	c1cc(ccc1[C@H]2c3c([nH]nc3OC(=C2C#N)N)C45CC6CC(C4)CC(C6)C5)Br
ZINC08442085	5.18	6.43	-6.28	3	5	0	451.368	2	c1cc(ccc1[C@@H]2c3c([nH]nc3OC(=C2C#N)N)C45CC6CC(C4)CC(C6)C5)Br
ZINC08442084	4.41	4.43	-9.16	3	7	0	432.524	4	COc1cc(cc(c1)OC)[C@H]2c3c([nH]nc3OC(=C2C#N)N)C45CC6CC(C4)CC(C6)C5
ZINC00633890	3.21	-2.7	-20.68	2	7	0	449.989	9	CCn1c(nnc1SCC(=O)Nc2cccc(c2)Cl)CCNC(=O)c3cccs3
ZINC08442083	4.41	4.41	-9.23	3	7	0	432.524	4	COc1cc(cc(c1)OC)[C@@H]2c3c([nH]nc3OC(=C2C#N)N)C45CC6CC(C4)CC(C6)C5
ZINC08442082	4.2	4.78	-8.97	3	8	0	462.55	5	COc1ccc(c(c1OC)OC)[C@H]2c3c([nH]nc3OC(=C2C#N)N)C45CC6CC(C4)CC(C6)C5
ZINC08442081	4.2	4.95	-10.21	3	8	0	462.55	5	COc1ccc(c(c1OC)OC)[C@@H]2c3c([nH]nc3OC(=C2C#N)N)C45CC6CC(C4)CC(C6)C5
ZINC05921075	0.48	3.77	-58.32	4	10	1	440.48	7	CCOc1cc(ccc1OCC[NH+]2CCOCC2)[C@H]3c4c([nH]cnc4=O)OC(=C3C#N)N
ZINC05921073	0.48	3.77	-58.16	4	10	1	440.48	7	CCOc1cc(ccc1OCC[NH+]2CCOCC2)[C@@H]3c4c([nH]cnc4=O)OC(=C3C#N)N
ZINC00633888	3.61	-2.15	-19.01	2	7	0	464.016	9	CCn1c(nnc1SCC(=O)Nc2ccc(cc2C)Cl)CCNC(=O)c3cccs3
ZINC00664653	5.69	6.99	-11.69	3	7	0	499.398	6	CC(C)(C)c1c2c(n[nH]1)OC(=C([C@H]2c3ccc(c(c3)OC)OCc4ccc(c(c4)Cl)Cl)C#N)N
ZINC00633887	3.24	9.02	-20.1	2	7	0	449.989	9	CCn1c(nnc1SCC(=O)Nc2ccc(cc2)Cl)CCNC(=O)c3cccs3
ZINC00664656	5.69	6.99	-11.37	3	7	0	499.398	6	CC(C)(C)c1c2c(n[nH]1)OC(=C([C@@H]2c3ccc(c(c3)OC)OCc4ccc(c(c4)Cl)Cl)C#N)N
ZINC00664643	4.73	6.01	-13.91	3	7	0	493.954	3	Cc1ccc(cc1)c2c3c(n[nH]2)OC(=C([C@@]34c5ccccc5N(C4=O)Cc6ccc(cc6)Cl)C#N)N
ZINC08442078	3.13	10.46	-22.71	2	8	0	498.659	9	Cc1c(nc(s1)NC(=O)CSc2nnc(n2C)CCNC(=O)c3cccs3)c4ccccc4
ZINC00664621	3.96	5.22	-9.69	3	5	0	429.224	2	c1cc(ccc1c2c3c(n[nH]2)OC(=C([C@H]3c4ccc(c(c4)Br)F)C#N)N)F

Supplementary Table S2:
Detailed table of XP Docking using Schrodinger's Glide module

ZINC_ID	xLogP	ApolarDesolvation	PolarDesolvation	H Donors	H Acceptors	Charge	MolecularWeight	RotatableBonds	SMILES
ZINC00664623	3.96	5.2	-9.72	3	5	0	429.224	2	c1cc(ccc1c2c3c(n[nH]2)OC(=C([C@@H]3c4ccc(c(c4)Br)F)C#N)N)F
ZINC00633883	2.04	6.73	-14.29	2	8	0	468.308	7	Cn1c(nnc1SCC(=O)Nc2ccc(cc2F)Br)CNC(=O)c3ccco3
ZINC05918663	4.14	6.25	-11.92	3	7	0	434.471	7	CCCCOc1cc(ccc1OC)[C@H]2c3c([nH]nc3OC(=C2C#N)N)c4ccc(cc4)F
ZINC05918661	4.14	6.25	-11.89	3	7	0	434.471	7	CCCCOc1cc(ccc1OC)[C@@H]2c3c([nH]nc3OC(=C2C#N)N)c4ccc(cc4)F
ZINC00664588	4.37	4.98	-10.43	3	6	0	457.715	3	COc1ccc(cc1Br)[C@@H]2c3c([nH]nc3OC(=C2C#N)N)c4ccc(cc4)Cl
ZINC00664571	4.54	4.81	-8.54	3	6	0	432.789	4	c1cc(ccc1c2c3c(n[nH]2)OC(=C([C@H]3c4ccc(cc4)OC(F)(F)F)C#N)N)Cl
ZINC00633881	2.13	7.03	-15.59	2	8	0	486.298	7	Cn1c(nnc1SCC(=O)Nc2c(cc(cc2Br)F)F)CNC(=O)c3ccco3
ZINC00664574	4.54	4.79	-8.61	3	6	0	432.789	4	c1cc(ccc1c2c3c(n[nH]2)OC(=C([C@@H]3c4ccc(cc4)OC(F)(F)F)C#N)N)Cl
ZINC00664566	3.32	7.5	-20.58	3	9	0	507.978	8	CCN(CC)C(=O)COc1ccc(cc1OC)[C@H]2c3c([nH]nc3OC(=C2C#N)N)c4ccc(cc4)Cl
ZINC00664569	3.32	7.53	-20.5	3	9	0	507.978	8	CCN(CC)C(=O)COc1ccc(cc1OC)[C@@H]2c3c([nH]nc3OC(=C2C#N)N)c4ccc(cc4)Cl
ZINC00664546	3.44	4.35	-12.28	3	7	0	471.286	4	COc1ccc(cc1OC)c2c3c(n[nH]2)OC(=C([C@H]3c4ccc(c(c4)Br)F)C#N)N
ZINC08442077	1.78	-1.13	-16.31	2	9	0	456.553	7	Cn1c(nnc1SCC(=O)Nc2c(c3c(s2)CCCC3)C#N)CNC(=O)c4ccco4
ZINC00664549	3.44	4.36	-12.15	3	7	0	471.286	4	COc1ccc(cc1OC)c2c3c(n[nH]2)OC(=C([C@@H]3c4ccc(c(c4)Br)F)C#N)N
ZINC00633874	2.41	7.64	-13.71	2	8	0	482.335	8	CCn1c(nnc1SCC(=O)Nc2ccc(cc2F)Br)CNC(=O)c3ccco3
ZINC06303157	4.5	-1.89	-15.39	3	6	0	459.358	5	[H]/N=C\1/[C@H]([C@H](C(=C(S1)N)C#N)c2ccc(c(c2)OC)OCc3ccc(c(c3)Cl)Cl)C#N
ZINC06303157	4.96	-3.62	-15.05	2	6	0	459.358	5	[H]/N=C\1/C(C([C@H](/C(=N\[H])/S1)C#N)c2ccc(c(c2)OC)OCc3ccc(c(c3)Cl)Cl)C#N
ZINC08442075	2.15	-1	-15.67	2	9	0	470.58	8	CCn1c(nnc1SCC(=O)Nc2c(c3c(s2)CCCC3)C#N)CNC(=O)c4ccco4
ZINC08442074	3.57	-2.87	-19.87	2	8	0	482.587	8	CCOC(=O)NC(=O)CSC1=NC(=C([C@@H](C1C#N)c2cccs2)C(=O)Nc3ccccc3)C
ZINC08442074	3.19	-0.87	-20.54	2	8	0	482.587	8	CCOC(=O)NC(=O)CSC1=C([C@H]([C@H](C(=N1)C)C(=O)Nc2ccccc2)c3cccs3)C#N
ZINC08442074	3.19	-2.75	-21.54	2	8	0	482.587	8	CCOC(=O)NC(=O)CSC1=C([C@H](C(C(=N1)C)C(=O)Nc2ccccc2)c3cccs3)C#N
ZINC00633868	4.23	-0.06	-21.81	2	7	0	437.569	8	Cc1ccc(cc1)NC(=O)Cc2nnc(n2C)SCC(=O)Nc3ccc(cc3)C(C)C
ZINC04167844	3.47	3.72	-10.1	3	6	0	429.299	3	COc1ccc(cc1Br)[C@H]2c3c([nH]nc3OC(=C2C#N)N)c4cccs4
ZINC04167843	3.47	3.38	-10.19	3	6	0	429.299	3	COc1ccc(cc1Br)[C@@H]2c3c([nH]nc3OC(=C2C#N)N)c4cccs4
ZINC04166616	3.58	4.42	-8.38	3	5	0	417.263	2	c1cc(sc1)c2c3c(n[nH]2)OC(=C([C@@H]3c4ccc(c(c4)Br)F)C#N)N
ZINC00664421	5.07	6.73	-10.85	3	8	0	498.926	5	c1ccc(c(c1)[C@@H]2c3c([nH]nc3OC(=C2C#N)N)c4ccc5c(c4)OCO5)OCc6ccc(cc6)Cl
ZINC08442072	4.78	1.73	-16.42	1	9	0	480.546	10	CCCOC(=O)c1cccc(c1)NC(=O)CSc2nnc(n2C)c3cc4cccc(c4o3)OC
ZINC00664409	4.18	5.09	-7.88	3	5	0	413.3	2	Cc1ccc(cc1)c2c3c(n[nH]2)OC(=C([C@H]3c4ccc(s4)Br)C#N)N
ZINC00664411	4.18	5.08	-7.97	3	5	0	413.3	2	Cc1ccc(cc1)c2c3c(n[nH]2)OC(=C([C@@H]3c4ccc(s4)Br)C#N)N
ZINC04165398	3.09	7.67	-19.8	3	9	0	487.56	8	CCN(CC)C(=O)COc1ccc(cc1OC)[C@@H]2c3c([nH]nc3OC(=C2C#N)N)c4ccc(cc4)C
ZINC04167833	3.58	3.19	-10.53	3	8	0	467.279	3	COc1ccc(cc1[C@H]2c3c([nH]nc3OC(=C2C#N)N)c4ccc5c(c4)OCO5)Br
ZINC04167831	3.58	3.15	-10.51	3	8	0	467.279	3	COc1ccc(cc1[C@@H]2c3c([nH]nc3OC(=C2C#N)N)c4ccc5c(c4)OCO5)Br
ZINC05917163	5.46	3.74	-9.14	1	4	0	453.359	2	[H]/N=C\1/[C@@H]([C@H]([C@H]2C[C@H](CC=C2C1(C#N)C#N)C(C)(C)C)c3cc(ccc3F)Br)C#N
ZINC05917163	5.46	3.62	-37.53	2	4	1	454.367	2	CC(C)(C)[C@H]1CC=C2[C@H](C1)[C@@H]([C@H](C(=[NH2+])C2(C#N)C#N)C#N)c3cc(ccc3F)Br
ZINC05917152	5.46	3.56	-9.77	1	4	0	453.359	2	[H]/N=C\1/[C@H]([C@H]([C@H]2C[C@@H](CC=C2C1(C#N)C#N)C(C)(C)C)c3cc(ccc3F)Br)C#N
ZINC05917152	5.46	3.43	-40.17	2	4	1	454.367	2	CC(C)(C)[C@@H]1CC=C2[C@H](C1)[C@@H]([C@@H](C(=[NH2+])C2(C#N)C#N)C#N)c3cc(ccc3F)Br
ZINC13161990	5.46	10.56	-52.75	2	4	1	454.367	2	CC(C)(C)[C@@H]1CC=C2[C@@H](C1)[C@@H]([C@@H](C(=[NH2+])C2(C#N)C#N)C#N)c3cc(ccc3F)Br
ZINC05919492	3.25	2.79	-54.7	2	5	1	427.301	2	[H]/N=C/1\[C@@H]([C@H]([C@H]2C[NH+](CC=C2C1(C#N)C#N)CC)c3cc(ccc3F)Br)C#N
ZINC05919492	3.25	2.72	-105.28	3	5	2	428.309	2	CC[NH+]1CC=C2[C@H](C1)[C@@H]([C@H](C(=[NH2+])C2(C#N)C#N)C#N)c3cc(ccc3F)Br
ZINC08442070	3.29	8.52	-8.51	2	4	0	415.291	1	c1cc(c(cc1Br)[C@H]2[C@H]3CSCC=C3C(C(=C2C#N)N)(C#N)C#N)F
ZINC08442070	3.37	7.55	-40	2	4	1	416.299	1	c1cc(c(cc1Br)[C@H]2[C@H]3CSCC=C3C(C(=[NH2+])C2C#N)(C#N)C#N)F
ZINC08442069	3.29	8.81	-8.97	2	4	0	415.291	1	c1cc(c(cc1Br)[C@@H]2[C@H]3CSCC=C3C(C(=C2C#N)N)(C#N)C#N)F
ZINC08442069	3.37	7.89	-41.41	2	4	1	416.299	1	c1cc(c(cc1Br)[C@@H]2[C@H]3CSCC=C3C(C(=[NH2+])C2C#N)(C#N)C#N)F
ZINC08442067	3.29	8.83	-8.88	2	4	0	415.291	1	c1cc(c(cc1Br)[C@H]2[C@@H]3CSCC=C3C(C(=C2C#N)N)(C#N)C#N)F
ZINC08442067	3.37	7.83	-41.63	2	4	1	416.299	1	c1cc(c(cc1Br)[C@H]2[C@@H]3CSCC=C3C(C(=[NH2+])C2C#N)(C#N)C#N)F
ZINC08442066	3.29	8.54	-8.53	2	4	0	415.291	1	c1cc(c(cc1Br)[C@@H]2[C@@H]3CSCC=C3C(C(=C2C#N)N)(C#N)C#N)F
ZINC08442066	3.37	1.35	-41.96	2	4	1	416.299	1	c1cc(c(cc1Br)[C@@H]2[C@@H]3CSCC=C3C(C(=[NH2+])C2C#N)(C#N)C#N)F
ZINC05919718	3.13	2.24	-19.95	1	9	0	504.55	4	[H]/N=C/1\[C@@H]([C@@]2(c3ccccc3N(C2=O)Cc4ccccc4)[C@H]5CN(CC=C5C1(C#N)C#N)C(=O)OCC)C#N
ZINC05919718	3.13	2.16	-48.79	2	9	1	505.558	4	CCOC(=O)N1CC=C2[C@H](C1)[C@]3(c4ccccc4N(C3=O)Cc5ccccc5)[C@H](C(=[NH2+])C2(C#N)C#N)C#N
ZINC05919715	3.13	2.33	-12.8	1	9	0	504.55	4	[H]/N=C\1/[C@@H]([C@]2(c3ccccc3N(C2=O)Cc4ccccc4)[C@H]5CN(CC=C5C1(C#N)C#N)C(=O)OCC)C#N
ZINC05919715	3.13	2.2	-40.62	2	9	1	505.558	4	CCOC(=O)N1CC=C2[C@H](C1)[C@@]3(c4ccccc4N(C3=O)Cc5ccccc5)[C@H](C(=[NH2+])C2(C#N)C#N)C#N
ZINC05919712	3.13	2.28	-21	1	9	0	504.55	4	[H]/N=C\1/[C@H]([C@]2(c3ccccc3N(C2=O)Cc4ccccc4)[C@@H]5CN(CC=C5C1(C#N)C#N)C(=O)OCC)C#N
ZINC05919712	3.13	2.15	-47.26	2	9	1	505.558	4	CCOC(=O)N1CC=C2[C@@H](C1)[C@@]3(c4ccccc4N(C3=O)Cc5ccccc5)[C@@H](C(=[NH2+])C2(C#N)C#N)C#N
ZINC00633847	4.43	2.26	-15.38	1	9	0	466.519	9	CCn1c(nnc1SCC(=O)Nc2ccccc2C(=O)OC)c3cc4cccc(c4o3)OC
ZINC09210779	3.4	5.69	-14.55	3	7	0	431.883	3	CCc1c2c(n[nH]1)OC(=C([C@]23c4ccccc4N(C3=O)Cc5ccc(cc5)Cl)C#N)N
ZINC09210779	3.4	6.7	-13.93	3	7	0	431.883	3	CCc1c2c([nH]n1)OC(=C([C@]23c4ccccc4N(C3=O)Cc5ccc(cc5)Cl)C#N)N
ZINC09210009	3.4	5.68	-12.79	3	7	0	431.883	3	CCc1c2c(n[nH]1)OC(=C([C@@]23c4ccccc4N(C3=O)Cc5ccc(cc5)Cl)C#N)N
ZINC09210009	3.4	6.64	-13.58	3	7	0	431.883	3	CCc1c2c([nH]n1)OC(=C([C@@]23c4ccccc4N(C3=O)Cc5ccc(cc5)Cl)C#N)N
ZINC00633845	3.92	-0.81	-19.5	2	7	0	494.379	9	C=CCn1c(nnc1SCC(=O)Nc2ccc(c(c2)Cl)Cl)CNC(=O)c3ccccc3F
ZINC00633842	3.92	-0.86	-17.68	2	7	0	494.379	9	C=CCn1c(nnc1SCC(=O)Nc2cc(cc(c2)Cl)Cl)CNC(=O)c3ccccc3F
ZINC13161527	4.16	9.92	-9.81	1	6	0	442.906	4	[H]/N=C\1/[C@@H](C2=CCOC[C@@H]2[C@H](C1(C#N)C#N)c3ccccc3OCc4ccccc4Cl)C#N
ZINC00633838	4.99	1.56	-12.32	1	6	0	495.349	8	COc1ccc(cc1)c2nnc(n2CC=C)SCC(=O)Nc3c(cc(cc3Br)F)F

Supplementary Table S2:
Detailed table of XP Docking using Schrodinger's Glide module

ZINC_ID	xLogP	ApolarDesolvation	PolarDesolvation	H Donors	H Acceptors	Charge	MolecularWeight	RotatableBonds	SMILES
ZINC08442062	3.73	0.15	-16.93	0	7	0	454.529	7	CC1=NC(=C([C@@H](C1C(=O)OC)c2cccs2)C#N)SCC(=O)c3ccc4c(c3)OCO4
ZINC08442062	4.11	-0.4	-14.45	0	7	0	454.529	7	CC1=C([C@H]([C@H](C(=N1)SCC(=O)c2ccc3c(c2)OCO3)C#N)c4cccs4)C(=O)OC
ZINC08442062	4.29	1.56	-27.26	0	7	0	454.529	7	COC(=O)C1[C@H]([C@H](C(=NC1=C)SCC(=O)c2ccc3c(c2)OCO3)C#N)c4cccs4
ZINC08442062	4.29	-0.23	-15.85	0	7	0	454.529	7	COC(=O)C1[C@H](C(C(=NC1=C)SCC(=O)c2ccc3c(c2)OCO3)C#N)c4cccs4
ZINC00633836	5.36	1.94	-11.82	1	5	0	479.35	7	Cc1cccc(c1)c2nnc(n2CC=C)SCC(=O)Nc3c(cc(cc3Br)F)F
ZINC09281351	3.88	6.43	-12.5	3	7	0	445.91	3	CC(C)c1c2c(n[nH]1)OC(=C([C@@]23c4ccccc4N(C3=O)Cc5ccc(cc5)Cl)C#N)N
ZINC09281351	3.88	7.12	-12.66	3	7	0	445.91	3	CC(C)c1c2c([nH]n1)OC(=C([C@@]23c4ccccc4N(C3=O)Cc5ccc(cc5)Cl)C#N)N
ZINC00633833	5.09	1.48	-12.67	1	5	0	467.339	6	CCn1c(nnc1SCC(=O)Nc2c(cc(cc2Br)F)F)c3cccc(c3)C
ZINC08442061	2.8	4.36	-14.27	3	8	0	447.882	4	COCc1c2c(n[nH]1)OC(=C([C@]23c4ccccc4N(C3=O)Cc5ccc(cc5)Cl)C#N)N
ZINC00633832	4.71	1.22	-12.97	1	5	0	453.312	5	Cc1cccc(c1)c2nnc(n2C)SCC(=O)Nc3c(cc(cc3Br)F)F
ZINC08442060	2.8	3.94	-12.16	3	8	0	447.882	4	COCc1c2c(n[nH]1)OC(=C([C@@]23c4ccccc4N(C3=O)Cc5ccc(cc5)Cl)C#N)N
ZINC00633829	5.07	1.44	-12.03	1	5	0	467.339	6	CCn1c(nnc1SCC(=O)Nc2c(cc(cc2Br)F)F)c3ccccc3C
ZINC08442059	3.67	-0.12	-17	2	7	0	473.961	9	Cc1c(cccc1Cl)NC(=O)CSc2nnc(n2CC=C)CNC(=O)c3ccccc3F
ZINC08442058	5.4	13.91	-28.91	2	5	0	465.578	7	CC1=C([C@H](C(=C(N1)SCC(=O)c2ccccc2)C#N)c3ccccc3)C(=O)Nc4ccccc4
ZINC08442058	5.74	-0.66	-13.03	1	5	0	465.578	7	CC1=C([C@H]([C@H](C(=N1)SCC(=O)c2ccccc2)C#N)c3ccccc3)C(=O)Nc4ccccc4
ZINC08442058	5.92	1.11	-22.2	1	5	0	465.578	7	C=C1C([C@H]([C@H](C(=N1)SCC(=O)c2ccccc2)C#N)c3ccccc3)C(=O)Nc4ccccc4
ZINC08442058	5.92	-0.65	-13.73	1	5	0	465.578	7	C=C1C([C@H](C(C(=N1)SCC(=O)c2ccccc2)C#N)c3ccccc3)C(=O)Nc4ccccc4
ZINC08442057	3.69	-0.16	-18.84	2	7	0	473.961	9	Cc1ccc(cc1NC(=O)CSc2nnc(n2CC=C)CNC(=O)c3ccccc3F)Cl
ZINC04391381	5.4	13.87	-20.72	2	5	0	465.578	7	CC1=C([C@@H](C(=C(N1)SCC(=O)c2ccccc2)C#N)c3ccccc3)C(=O)Nc4ccccc4
ZINC04391381	5.92	12.3	-14.09	1	5	0	465.578	7	C=C1C([C@@H](C(C(=N1)SCC(=O)c2ccccc2)C#N)c3ccccc3)C(=O)Nc4ccccc4
ZINC04391381	5.92	13.43	-24.76	1	5	0	465.578	7	C=C1C([C@@H]([C@@H](C(=N1)SCC(=O)c2ccccc2)C#N)c3ccccc3)C(=O)Nc4ccccc4
ZINC13161526	4.61	11.51	-19.06	1	6	0	465.944	2	[H]/N=C\1/[C@H](C2=CCCC[C@@H]2[C@@]3(C1(C#N)C#N)c4ccccc4N(C3=O)Cc5ccc(cc5)Cl)C#N
ZINC00633816	3.29	-0.72	-19.23	2	7	0	459.934	9	C=CCn1c(nnc1SCC(=O)Nc2cccc(c2)Cl)CNC(=O)c3ccccc3F
ZINC13161525	4.61	11.72	-12.01	1	6	0	465.944	2	[H]/N=C\1/[C@H](C2=CCCC[C@@H]2[C@]3(C1(C#N)C#N)c4ccccc4N(C3=O)Cc5ccc(cc5)Cl)C#N
ZINC13161524	4.61	11.88	-12.19	1	6	0	465.944	2	[H]/N=C\1/[C@H](C2=CCCC[C@H]2[C@@]3(C1(C#N)C#N)c4ccccc4N(C3=O)Cc5ccc(cc5)Cl)C#N
ZINC00633814	4.29	1.23	-19.24	1	8	0	462.531	9	Cn1c(nnc1SCC(=O)Nc2ccc(cc2)C(=O)OC)COc3ccc4ccccc4c3
ZINC08442053	4.61	11.68	-12.62	1	6	0	465.944	2	[H]/N=C/1\[C@H](C2=CCCC[C@H]2[C@]3(C1(C#N)C#N)c4ccccc4N(C3=O)Cc5ccc(cc5)Cl)C#N
ZINC08442053	4.61	2.06	-40.32	2	6	1	466.952	2	c1ccc2c(c1)[C@@]3([C@@H]4CCCC=C4[C@@H](C(=[NH2+])C3(C#N)C#N)C#N)C(=O)N2Cc5ccc(cc5)Cl
ZINC00633812	3.79	0.96	-17.39	1	8	0	446.916	9	Cn1c(nnc1SCC(=O)Nc2ccc(cc2)C(=O)OC)COc3ccc(cc3)Cl
ZINC00633810	4.16	1.09	-16.64	1	8	0	460.943	10	CCn1c(nnc1SCC(=O)Nc2ccc(cc2)C(=O)OC)COc3ccc(cc3)Cl
ZINC00633806	4.26	-1.47	-16	0	7	0	473.602	7	Cn1c(nnc1SCC(=O)N2CCN(CC2)c3ccccc3)COc4ccc5ccccc5c4
ZINC00633803	3.75	-1.77	-13.99	0	7	0	457.987	7	Cn1c(nnc1SCC(=O)N2CCN(CC2)c3ccccc3)COc4ccc(cc4)Cl
ZINC04391378	3.9	1.02	-14.83	0	6	0	440.546	8	CC1=NC(=C([C@@H](C1C(=O)OC)c2cccs2)C#N)SCC(=O)c3ccc(cc3)OC
ZINC04391378	4.46	2.43	-24.82	0	6	0	440.546	8	COc1ccc(cc1)C(=O)CSC2=NC(=C)C([C@H]([C@H]2C#N)c3cccs3)C(=O)OC
ZINC04391378	4.46	0.64	-13.77	0	6	0	440.546	8	COc1ccc(cc1)C(=O)CSC2=NC(=C)C([C@H](C2C#N)c3cccs3)C(=O)OC
ZINC00633801	4.13	-1.61	-13.51	0	7	0	472.014	8	CCn1c(nnc1SCC(=O)N2CCN(CC2)c3ccccc3)COc4ccc(cc4)Cl
ZINC00633798	4.14	0.94	-16.15	1	8	0	460.943	10	CCOC(=O)c1cccc(c1)NC(=O)CSc2nnc(n2C)COc3ccc(cc3)Cl
ZINC08442050	3.9	1.04	-14.8	0	6	0	440.546	8	CC1=NC(=C([C@H](C1C(=O)OC)c2cccs2)C#N)SCC(=O)c3ccc(cc3)OC
ZINC08442050	4.46	0.64	-13.45	0	6	0	440.546	8	COc1ccc(cc1)C(=O)CSC2=NC(=C)C([C@@H](C2C#N)c3cccs3)C(=O)OC
ZINC08442050	4.28	0.53	-11.6	0	6	0	440.546	8	CC1=C([C@@H]([C@H](C(=N1)SCC(=O)c2ccc(cc2)OC)C#N)c3cccs3)C(=O)OC
ZINC08442050	4.46	2.19	-15.24	0	6	0	440.546	8	COc1ccc(cc1)C(=O)CSC2=NC(=C)C([C@@H]([C@@H]2C#N)c3cccs3)C(=O)OC
ZINC08442049	1.78	-3.08	-43.22	4	11	1	498.56	11	CCOc1cc(ccc1OCC[NH+]2CCOCC2)[C@H]3c4c([nH]nc4OC(=C3C#N)N)CC(=O)OCC
ZINC08442048	2.48	-0.48	-18.94	2	10	0	483.55	11	CCn1c(nnc1SCC(=O)Nc2ccc(cc2)C(=O)OC)CNC(=O)c3ccc(cc3)OC
ZINC08442047	1.78	-3.08	-47.2	4	11	1	498.56	11	CCOc1cc(ccc1OCC[NH+]2CCOCC2)[C@@H]3c4c([nH]nc4OC(=C3C#N)N)CC(=O)OCC
ZINC00803806	3.76	5.71	-11.64	3	7	0	434.521	6	Cc1ccc(cc1)SCc2c3c(n[nH]2)OC(=C([C@@H]3c4cccc(c4OC)OC)C#N)N
ZINC00633792	2.1	-0.67	-19.52	2	10	0	469.523	10	Cn1c(nnc1SCC(=O)Nc2ccc(cc2)C(=O)OC)CNC(=O)c3ccc(cc3)OC
ZINC00803800	3.83	5.17	-12.28	3	7	0	418.478	4	Cc1ccc(cc1)SCc2c3c(n[nH]2)OC(=C([C@@H]3c4ccc5c(c4)OCO5)C#N)N
ZINC00662497	4.57	6.27	-8.92	3	5	0	459.394	4	Cc1ccc(cc1)SCc2c3c(n[nH]2)OC(=C([C@H]3c4cc(cs4)Br)C#N)N
ZINC00633782	3.87	-1.38	-20.14	1	8	0	441.509	10	CC(C)OC(=O)c1cccc(c1)NC(=O)CSc2nnc(o2)Cc3ccc(cc3)OC
ZINC00662500	4.57	6.31	-9.64	3	5	0	459.394	4	Cc1ccc(cc1)SCc2c3c(n[nH]2)OC(=C([C@@H]3c4cc(cs4)Br)C#N)N
ZINC08442046	4.01	-1.56	-19.73	1	8	0	441.509	11	CCCOC(=O)c1cccc(c1)NC(=O)CSc2nnc(o2)Cc3ccc(cc3)OC
ZINC00803814	4.17	6.76	-12.62	3	6	0	425.517	4	Cc1ccc(cc1)SCc2c3c(n[nH]2)OC(=C([C@@H]3c4ccnc5c4cccc5)C#N)N
ZINC00803814	4.17	7.8	-36.61	4	6	1	426.525	4	Cc1ccc(cc1)SCc2c3c([nH]n2)OC(=C([C@@H]3c4cc[nH+]c5c4cccc5)C#N)N
ZINC08442045	2.79	-3.78	-41.54	4	9	1	488.568	8	CCOc1cc(ccc1OCC[NH+]2CCOCC2)[C@@H]3c4c([nH]nc4OC(=C3C#N)N)c5ccccc5
ZINC08442044	4.27	6.39	-68.66	1	8	-1	481.337	10	c1cc(cc(c1)NC(=O)CSc2nnc(o2)CCCOc3ccc(cc3Cl)Cl)C(=O)[O-]
ZINC08442043	3.07	-2.93	-40.56	4	9	1	468.578	8	CCOc1cc(ccc1OCC[NH+]2CCOCC2)[C@H]3c4c([nH]nc4OC(=C3C#N)N)C(C)(C)C
ZINC08442042	3.07	-2.96	-41.33	4	9	1	468.578	8	CCOc1cc(ccc1OCC[NH+]2CCOCC2)[C@@H]3c4c([nH]nc4OC(=C3C#N)N)C(C)(C)C
ZINC08442041	4.29	6.38	-61.09	1	8	-1	481.337	10	c1cc(ccc1C(=O)[O-])NC(=O)CSc2nnc(o2)CCCOc3ccc(cc3Cl)Cl
ZINC08442040	2.97	7.5	-12.01	2	9	0	461.522	5	CC(C)(C)OC(=O)N1CC=C2[C@H](C1)[C@H](C(C(=C2C#N)N)(C#N)C#N)c3cc(ccc3OC)OC
ZINC08442040	3.05	2.34	-43.39	2	9	1	462.53	5	CC(C)(C)OC(=O)N1CC=C2[C@H](C1)[C@H](C(C(=[NH2+])C2C#N)(C#N)C#N)c3cc(ccc3OC)OC
ZINC08442038	3.53	-1.64	-15.78	1	8	0	427.482	10	CCOC(=O)c1ccc(cc1)NC(=O)CSc2nnc(o2)Cc3ccc(cc3)OC

Supplementary Table S2:
Detailed table of XP Docking using Schrodinger's Glide module

ZINC_ID	xLogP	ApolarDesolvation	PolarDesolvation	H Donors	H Acceptors	Charge	MolecularWeight	RotatableBonds	SMILES
ZINC13161523	5.19	11.17	-19.87	1	7	0	457.578	4	[H]/N=C\1/[C@H](C2=CCN(C[C@@H]2[C@H](C1(C#N)C#N)c3ccc(cc3)C(C)(C)C(=O)OC(C)(C)C)C#N
ZINC08442036	3.7	9.39	-14.93	2	9	0	479.496	4	c1ccc(cc1)COC(=O)N2CC=C3[C@H](C2)[C@H](C(C(=C3C#N)N)(C#N)C#N)c4ccc5c(c4)OCO5
ZINC08442036	3.78	1.08	-51.89	2	9	1	480.504	4	c1ccc(cc1)COC(=O)N2CC=C3[C@H](C2)[C@H](C(C(=[NH2+])C3C#N)(C#N)C#N)c4ccc5c(c4)OCO5
ZINC08442035	2.86	9.8	-19.83	2	10	0	497.577	12	CCn1c(nnc1SCC(=O)Nc2ccc(cc2)C(=O)OCC)CNC(=O)c3ccc(cc3)OC
ZINC08442033	5.6	3.24	-13.25	1	7	0	491.595	5	[H]/N=C/1\[C@H](C2=CCN(C[C@@H]2[C@H](C1(C#N)C#N)c3ccc(cc3)C(C)(C)C(=O)OCc4ccccc4)C#N
ZINC08442033	5.6	3.07	-46.12	2	7	1	492.603	5	CC(C)(C)c1ccc(cc1)[C@@H]2[C@H]3CN(CC=C3[C@@H](C(=[NH2+])C2(C#N)C#N)C#N)C(=O)OCc4ccccc4
ZINC08442032	2.48	-0.7	-19.32	2	10	0	483.55	11	CCOC(=O)c1ccc(cc1)NC(=O)CSc2nnc(n2C)CNC(=O)c3ccc(cc3)OC
ZINC08442031	4.78	10.52	-12.87	2	8	0	519.483	6	c1ccc(cc1)COC(=O)N2CC=C3[C@H](C2)[C@H](C(C(=C3C#N)N)(C#N)C#N)c4ccc(cc4)OC(F)(F)F
ZINC08442031	4.86	9.61	-50.29	2	8	1	520.491	6	c1ccc(cc1)COC(=O)N2CC=C3[C@H](C2)[C@H](C(C(=[NH2+])C3C#N)(C#N)C#N)c4ccc(cc4)OC(F)(F)F
ZINC08442030	4.06	10.85	-14.46	2	7	0	471.467	4	c1ccc(cc1)COC(=O)N2CC=C3[C@H](C2)[C@H](C(C(=C3C#N)N)(C#N)C#N)c4ccc(c(c4)F)F
ZINC08442030	4.15	3.39	-52.78	2	7	1	472.475	4	c1ccc(cc1)COC(=O)N2CC=C3[C@H](C2)[C@H](C(C(=[NH2+])C3C#N)(C#N)C#N)c4ccc(c(c4)F)F
ZINC08442029	2.38	-1.59	-14.66	2	8	0	431.562	8	C[C@H]1CCCC[C@H]1NC(=O)CSc2nnc(n2C)CNC(=O)c3ccc(cc3)OC
ZINC08442028	3.59	10.87	-13.32	2	7	0	471.467	4	c1ccc(cc1)COC(=O)N2CC=C3[C@H](C2)[C@H](C(C(=C3C#N)N)(C#N)C#N)c4ccc(cc4F)F
ZINC08442028	3.67	3.36	-49.25	2	7	1	472.475	4	c1ccc(cc1)COC(=O)N2CC=C3[C@H](C2)[C@H](C(C(=[NH2+])C3C#N)(C#N)C#N)c4ccc(cc4F)F
ZINC08442027	2.98	7.67	-11.54	2	7	0	458.341	3	CCOC(=O)N1CC=C2[C@H](C1)[C@H](C(C(=C2C#N)N)(C#N)C#N)c3cc(cs3)Br
ZINC08442027	3.06	0.28	-47.2	2	7	1	459.349	3	CCOC(=O)N1CC=C2[C@H](C1)[C@H](C(C(=[NH2+])C2C#N)(C#N)C#N)c3cc(cs3)Br
ZINC00633764	2.38	-2.1	-15.21	2	8	0	431.562	8	C[C@@H]1CCCC[C@H]1NC(=O)CSc2nnc(n2C)CNC(=O)c3ccc(cc3)OC
ZINC08442026	2.69	6.62	-13.44	2	7	0	427.386	3	CC(=O)N1CC=C2[C@H](C1)[C@H](C(C(=C2C#N)N)(C#N)C#N)c3ccc(cc3)OC(F)(F)F
ZINC08442026	2.78	5.79	-51.39	2	7	1	428.394	3	CC(=O)N1CC=C2[C@H](C1)[C@H](C(C(=[NH2+])C2C#N)(C#N)C#N)c3ccc(cc3)OC(F)(F)F
ZINC08442025	3.74	1.88	-67.92	2	7	1	436.495	3	[H]/N=C/1\C(C2=CC[NH+](C[C@@H]2[C@H](C1(C#N)C#N)c3ccc4c(c3)OCO4)Cc5ccccc5)C#N
ZINC08442025	3.74	1.71	-125.63	3	7	2	437.503	3	c1ccc(cc1)C[NH+]2CC=C3[C@H](C2)[C@H](C(C(=[NH2+])C3C#N)(C#N)C#N)c4ccc5c(c4)OCO5
ZINC08442023	3.42	8.13	-11.26	1	7	0	451.53	5	[H]/N=C\1/[C@@H](C2=CCN(C[C@@H]2[C@H](C1(C#N)C#N)c3cc(ccc3OC)OC)Cc4ccccc4)C#N
ZINC08442023	3.42	2.44	-111.03	3	7	2	453.546	5	COc1ccc(c(c1)[C@@H]2[C@H]3C[NH+](CC=C3[C@H](C(=[NH2+])C2(C#N)C#N)C#N)Cc4ccccc4)OC
ZINC08442021	3.14	6.78	-11.82	2	9	0	516.646	9	CCCN1CC=C2[C@H](C1)[C@H](C(C(=C2C#N)N)(C#N)C#N)c3ccc(c(c3)OCC)OCCN4CCOCC4
ZINC08442021	3.23	1.75	-177.51	4	9	3	519.67	9	CCC[NH+]1CC=C2[C@H](C1)[C@H](C(C(=[NH2+])C2C#N)(C#N)C#N)c3ccc(c(c3)OCC)OCC[NH+]4CCOCC4
ZINC08442021	3.14	9.15	-43.15	3	9	1	517.654	9	CCCN1CC=C2[C@H](C1)[C@H](C(C(=C2C#N)N)(C#N)C#N)c3ccc(c(c3)OCC)OCC[NH+]4CCOCC4
ZINC00633767	2.38	-2.13	-15.09	2	8	0	431.562	8	C[C@H]1CCCC[C@@H]1NC(=O)CSc2nnc(n2C)CNC(=O)c3ccc(cc3)OC
ZINC08442020	3.2	6.65	-7.29	2	5	0	428.359	2	CC(C)N1CC=C2[C@H](C1)[C@H](C(C(=C2C#N)N)(C#N)C#N)c3cc(cs3)Br
ZINC08442020	3.28	0.97	-112.97	3	5	2	430.375	2	CC(C)[NH+]1CC=C2[C@H](C1)[C@H](C(C(=[NH2+])C2C#N)(C#N)C#N)c3cc(cs3)Br
ZINC08442019	5.04	9.4	-8.48	2	5	0	422.479	4	CC(C)(C)[C@H]1CC=C2[C@H](C1)[C@H](C(C(=C2C#N)N)(C#N)C#N)c3ccccc3OC(F)F
ZINC08442019	5.13	4.57	-38.78	2	5	1	423.487	4	CC(C)(C)[C@H]1CC=C2[C@H](C1)[C@H](C(C(=[NH2+])C2C#N)(C#N)C#N)c3ccccc3OC(F)F
ZINC08442018	2.38	-1.55	-14.69	2	8	0	431.562	8	C[C@@H]1CCCC[C@@H]1NC(=O)CSc2nnc(n2C)CNC(=O)c3ccc(cc3)OC
ZINC08442017	5.04	9.79	-9.18	2	5	0	422.479	4	CC(C)(C)[C@@H]1CC=C2[C@H](C1)[C@H](C(C(=C2C#N)N)(C#N)C#N)c3ccccc3OC(F)F
ZINC08442017	5.13	4.43	-41.59	2	5	1	423.487	4	CC(C)(C)[C@@H]1CC=C2[C@H](C1)[C@H](C(C(=[NH2+])C2C#N)(C#N)C#N)c3ccccc3OC(F)F
ZINC08442016	5.92	8.94	-7.73	2	5	0	440.469	4	CC(C)(C)[C@H]1CC=C2[C@H](C1)[C@H](C(C(=C2C#N)N)(C#N)C#N)c3ccc(cc3)OC(F)(F)F
ZINC08442016	6	4.46	-41.11	2	5	1	441.477	4	CC(C)(C)[C@H]1CC=C2[C@H](C1)[C@H](C(C(=[NH2+])C2C#N)(C#N)C#N)c3ccc(cc3)OC(F)(F)F
ZINC00633758	3.88	-0.75	-13.8	0	7	0	451.596	7	Cc1cccc(c1OCc2nnc(n2C)SCC(=O)N3CCN(CC3)c4ccccc4)C
ZINC08442015	5.92	9.89	-8.65	2	5	0	440.469	4	CC(C)(C)[C@@H]1CC=C2[C@H](C1)[C@H](C(C(=C2C#N)N)(C#N)C#N)c3ccc(cc3)OC(F)(F)F
ZINC08442015	6	4.83	-44.37	2	5	1	441.477	4	CC(C)(C)[C@@H]1CC=C2[C@H](C1)[C@H](C(C(=[NH2+])C2C#N)(C#N)C#N)c3ccc(cc3)OC(F)(F)F
ZINC08442014	2.35	5.36	-13.54	1	9	0	488.592	7	[H]/N=C\1/[C@@H](C2=CCN(C[C@@H]2[C@H](C1(C#N)C#N)c3ccc(c(c3)OCC)OCCN4CCOCC4)C)C#N
ZINC08442014	2.35	1.58	-172.38	4	9	3	491.616	7	CCOc1cc(ccc1OCC[NH+]2CCOCC2)[C@@H]3[C@H]4C[NH+](CC=C4[C@H](C(=[NH2+])C3(C#N)C#N)C#N)C
ZINC08442014	2.35	7.64	-44.57	2	9	1	489.6	7	[H]/N=C\1/[C@@H](C2=CCN(C[C@@H]2[C@H](C1(C#N)C#N)c3ccc(c(c3)OCC)OCC[NH+]4CCOCC4)C)C#N
ZINC17147048	5.53	8.16	-44.5	1	4	-1	419.448	2	c1c(c2c3c(c(cc(=O)[nH]3)[O-])sc2nc1C45CC6CC(C4)CC(C6)C5)C(F)(F)F
ZINC00633754	4.25	13.91	-14.07	0	7	0	465.623	8	CCn1c(nnc1SCC(=O)N2CCN(CC2)c3ccccc3)COc4c(cccc4C)C
ZINC08442012	4.06	3.22	-18.48	1	9	0	493.523	6	[H]/N=C/1\[C@H](C2=CCN(C[C@@H]2[C@H](C1(C#N)C#N)c3ccc(cc3)C(=O)OC)C(=O)OCc4ccccc4)C#N
ZINC08442012	4.06	3.04	-54.78	2	9	1	494.531	6	COC(=O)c1ccc(cc1)[C@@H]2[C@H]3CN(CC=C3[C@@H](C(=[NH2+])C2(C#N)C#N)C#N)C(=O)OCc4ccccc4
ZINC08442011	2.07	-3.36	-16.13	1	9	0	480.594	8	Cn1c(nnc1SCC(=O)N2CCN(CC2)c3ccccc3)CNC(=O)c4ccc(cc4)OC
ZINC08442010	4.1	8.42	-10.06	2	7	0	446.551	5	CC(C)(C)[C@H]1CC=C2[C@H](C1)[C@H](C(C(=C2C#N)N)(C#N)C#N)c3cc(c(cc3OC)OC)OC
ZINC08442010	4.19	3.86	-39.86	2	7	1	447.559	5	CC(C)(C)[C@H]1CC=C2[C@H](C1)[C@H](C(C(=[NH2+])C2C#N)(C#N)C#N)c3cc(c(cc3OC)OC)OC
ZINC08442009	4.1	8.78	-10.56	2	7	0	446.551	5	CC(C)(C)[C@@H]1CC=C2[C@H](C1)[C@H](C(C(=C2C#N)N)(C#N)C#N)c3cc(c(cc3OC)OC)OC
ZINC08442009	4.19	3.74	-42.84	2	7	1	447.559	5	CC(C)(C)[C@@H]1CC=C2[C@H](C1)[C@H](C(C(=[NH2+])C2C#N)(C#N)C#N)c3cc(c(cc3OC)OC)OC
ZINC08442007	3.74	6.53	-18.47	4	7	0	429.524	5	CC(C)(C)[C@H]1CC=C2[C@H](C1)[C@H](C(C(=C2C#N)N)(C#N)C#N)c3ccc(cc3)OCC(=O)N
ZINC08442007	3.82	0.3	-49.6	4	7	1	430.532	5	CC(C)(C)[C@H]1CC=C2[C@H](C1)[C@H](C(C(=[NH2+])C2C#N)(C#N)C#N)c3ccc(cc3)OCC(=O)N
ZINC08442006	3.74	7.48	-20.39	4	7	0	429.524	5	CC(C)(C)[C@@H]1CC=C2[C@H](C1)[C@H](C(C(=C2C#N)N)(C#N)C#N)c3ccc(cc3)OCC(=O)N
ZINC08442006	3.82	0.67	-52.53	4	7	1	430.532	5	CC(C)(C)[C@@H]1CC=C2[C@H](C1)[C@H](C(C(=[NH2+])C2C#N)(C#N)C#N)c3ccc(cc3)OCC(=O)N
ZINC08442005	0.75	-1.21	-52.15	2	9	1	433.558	8	CCn1c(nnc1SCC(=O)N2CC[NH+](CC2)C)CNC(=O)c3ccc(cc3)OC
ZINC08442004	4.02	3.6	-71.24	2	7	1	450.522	5	[H]/N=C/1\[C@H](C2=CC[NH+](C[C@@H]2[C@H](C1(C#N)C#N)c3ccc(cc3)C(=O)OC)Cc4ccccc4)C#N
ZINC08442004	4.02	3.43	-132.18	3	7	2	451.53	5	COC(=O)c1ccc(cc1)[C@@H]2[C@H]3C[NH+](CC=C3[C@@H](C(=[NH2+])C2(C#N)C#N)C#N)Cc4ccccc4
ZINC00633747	2.04	-2.46	-17.12	2	8	0	439.541	10	Cn1c(nnc1SCC(=O)NCCc2ccccc2)CNC(=O)c3ccc(cc3)OC
ZINC01107286	4.79	6.61	-10.71	3	7	0	444.535	6	Cc1c2c(n[nH]1)OC(=C([C@H]2c3ccc(c(c3)COc4ccc(cc4)C(C)(C)C)OC)C#N)N
ZINC01107287	4.79	6.7	-10.7	3	7	0	444.535	6	Cc1c2c(n[nH]1)OC(=C([C@@H]2c3ccc(c(c3)COc4ccc(cc4)C(C)(C)C)OC)C#N)N

Supplementary Table S2:
Detailed table of XP Docking using Schrodinger's Glide module

ZINC_ID	xLogP	ApolarDesolvation	PolarDesolvation	H Donors	H Acceptors	Charge	MolecularWeight	RotatableBonds	SMILES
ZINC09122187	4.75	7.04	-8.91	3	5	0	453.365	4	Cc1ccc(cc1)SCc2c3c(n[nH]2)OC(=C([C@H]3c4ccc(cc4)Br)C#N)N
ZINC00633743	2.53	10.07	-21.04	1	9	0	495.605	8	Cc1cccc(c1OCc2nnc(n2C)SCC(=O)Nc3cccc(c3)C(=O)N4CCOCC4)C
ZINC09122186	4.75	7.14	-9.7	3	5	0	453.365	4	Cc1ccc(cc1)SCc2c3c(n[nH]2)OC(=C([C@@H]3c4ccc(cc4)Br)C#N)N
ZINC08836444	4.72	7.04	-8.91	3	5	0	453.365	4	Cc1ccc(cc1)SCc2c3c(n[nH]2)OC(=C([C@H]3c4cccc(c4)Br)C#N)N
ZINC08836443	4.72	7.13	-9.26	3	5	0	453.365	4	Cc1ccc(cc1)SCc2c3c(n[nH]2)OC(=C([C@@H]3c4cccc(c4)Br)C#N)N
ZINC00633740	2.51	9.53	-20.62	1	8	0	451.552	6	Cc1cccc(c1)c2nnc(n2C)SCC(=O)Nc3cccc(c3)C(=O)N4CCOCC4
ZINC08442003	3.69	8.96	-20.84	3	9	0	533.654	10	CCN(CC)C(=O)COc1ccc(cc1OC)[C@@H]2c3c([nH]nc3OC(=C2C#N)N)CSc4ccc(cc4)C
ZINC00660155	4.32	7.17	-11.77	3	7	0	462.575	7	Cc1ccc(cc1)SCc2c3c(n[nH]2)OC(=C([C@H]3c4ccc(c(c4)OC)OC(C)C)C#N)N
ZINC00633738	2.53	9.54	-20.41	1	8	0	451.552	6	Cc1ccc(cc1)c2nnc(n2C)SCC(=O)Nc3cccc(c3)C(=O)N4CCOCC4
ZINC00660158	4.32	7.26	-12.86	3	7	0	462.575	7	Cc1ccc(cc1)SCc2c3c(n[nH]2)OC(=C([C@@H]3c4ccc(c(c4)OC)OC(C)C)C#N)N
ZINC07101975	6.07	12.59	-7.37	0	3	0	366.892	3	c1ccc(cc1)[C@H]2CN(N=C2c3ccc(cc3)Cl)C(=O)C4CCCCC4
ZINC07101974	6.07	12.59	-7.43	0	3	0	366.892	3	c1ccc(cc1)[C@@H]2CN(N=C2c3ccc(cc3)Cl)C(=O)C4CCCCC4
ZINC08442000	4.69	4.05	-17.32	0	7	0	456.564	10	CCOC(=O)C1[C@H](C(=C(N=C1C)SCC(=O)C(C)(C)C)C#N)c2ccc(cc2)C(=O)OC
ZINC08442000	5.25	2.47	-13.29	0	7	0	456.564	10	CCOC(=O)C1[C@H](C(C(=NC1=C)SCC(=O)C(C)(C)C)C#N)c2ccc(cc2)C(=O)OC
ZINC08442000	5.07	3.24	-13.6	0	7	0	456.564	10	CCOC(=O)C1=C(N=C(C([C@@H]1c2ccc(cc2)C(=O)OC)C#N)SCC(=O)C(C)(C)C)C
ZINC08441999	6.95	-0.73	-11.18	1	3	0	425.582	6	c1ccc(cc1)Cc2nc(cs2)c3ccc(cc3)Nc4nc(cs4)c5ccccc5
ZINC08441998	5.02	3.17	-18.15	0	8	0	506.58	11	CCOC(=O)C1[C@@H](C(=C(N=C1C)SCC(=O)c2ccc(cc2)OC)C#N)c3ccc(cc3)C(=O)OC
ZINC08441998	5.58	1.55	-16.31	0	8	0	506.58	11	CCOC(=O)C1[C@@H](C(C(=NC1=C)SCC(=O)c2ccc(cc2)OC)C#N)c3ccc(cc3)C(=O)OC
ZINC08441998	5.4	2.56	-15.01	0	8	0	506.58	11	CCOC(=O)C1=C(N=C(C([C@H]1c2ccc(cc2)C(=O)OC)C#N)SCC(=O)c3ccc(cc3)OC)C
ZINC08441997	8.67	-1.68	-9.53	1	3	0	524.896	5	c1cc(ccc1c2csc(n2)c3ccc(cc3)Cl)Nc4nc(cs4)c5ccc(cc5)Br
ZINC08441996	5.02	3.37	-20.02	0	8	0	506.58	11	CCOC(=O)C1[C@H](C(=C(N=C1C)SCC(=O)c2ccc(cc2)OC)C#N)c3ccc(cc3)C(=O)OC
ZINC08441996	5.4	2.57	-16.23	0	8	0	506.58	11	CCOC(=O)C1=C(N=C(C([C@@H]1c2ccc(cc2)C(=O)OC)C#N)SCC(=O)c3ccc(cc3)OC)C
ZINC08441996	5.58	1.79	-15.88	0	8	0	506.58	11	CCOC(=O)C1[C@H](C(C(=NC1=C)SCC(=O)c2ccc(cc2)OC)C#N)c3ccc(cc3)C(=O)OC
ZINC08441995	6.47	-2.37	-14.03	1	5	0	460.378	6	CCOC(=O)c1c(nc(s1)Nc2ccc(cc2)Br)c3nc4ccccc4s3
ZINC08441992	6.19	-1.38	-8.72	1	3	0	414.353	4	c1ccc(cc1)c2csc(n2)Nc3nc(cs3)c4ccc(cc4)Br
ZINC08441991	6.97	13.6	-63.26	2	4	0	425.557	4	C[NH+](C)c1ccc(cc1)/[NH+]=c\2/c(cc3ccccc3o2)c4nc(cs4)c5ccccc5
ZINC08441991	6.97	12.36	-53.72	2	4	0	425.557	4	C[NH+](C)c1ccc(cc1)/[NH+]=c/2\c(cc3ccccc3o2)c4nc(cs4)c5ccccc5
ZINC08441991	6.97	12.04	-23.28	1	4	1	424.549	4	CN(C)c1ccc(cc1)/[NH+]=c/2\c(cc3ccccc3o2)c4nc(cs4)c5ccccc5
ZINC08441991	6.97	12.85	-23.69	1	4	1	424.549	4	CN(C)c1ccc(cc1)/[NH+]=c\2/c(cc3ccccc3o2)c4nc(cs4)c5ccccc5
ZINC08441989	5.42	2.47	-16.2	1	6	0	444.579	5	[H]/N=C\1/[C@H](C2=CC[C@@H](C[C@@H]2[C@@H](C1(C#N)C#N)c3ccc(c(c3)OC)OC(C)C)C(C)(C)C)C#N
ZINC08441989	5.42	2.29	-44.5	2	6	1	445.587	5	CC(C)Oc1ccc(cc1OC)[C@H]2[C@H]3C[C@H](CC=C3[C@@H](C(=[NH2+])C2(C#N)C#N)C#N)C(C)(C)C
ZINC08441988	5.42	3.81	-11.79	1	6	0	444.579	5	[H]/N=C\1/[C@H](C2=CC[C@@H](C[C@@H]2[C@H](C1(C#N)C#N)c3ccc(c(c3)OC)OC(C)C)C(C)(C)C)C#N
ZINC08441988	5.42	3.63	-42.14	2	6	1	445.587	5	CC(C)Oc1ccc(cc1OC)[C@@H]2[C@H]3C[C@H](CC=C3[C@@H](C(=[NH2+])C2(C#N)C#N)C#N)C(C)(C)C
ZINC00633686	4.13	0.07	-30.68	0	6	0	440.278	1	c1ccc2c(c1)c(=O)n3c(n2)SCC(=N3)c4cc5cc(ccc5oc4=O)Br
ZINC08441987	5.42	4.12	-14.62	1	6	0	444.579	5	[H]/N=C/1\[C@@H](C2=CC[C@H](C[C@@H]2[C@@H](C1(C#N)C#N)c3ccc(c(c3)OC)OC(C)C)C(C)(C)C)C#N
ZINC08441987	5.42	3.96	-43.23	2	6	1	445.587	5	CC(C)Oc1ccc(cc1OC)[C@H]2[C@H]3C[C@@H](CC=C3[C@H](C(=[NH2+])C2(C#N)C#N)C#N)C(C)(C)C
ZINC08441986	6.18	10.5	-32.78	2	6	1	469.439	6	COc1ccc(cc1)NC(=O)c\2cc3cccc(c3o/c2=[NH+]\c4cccc(c4)C(F)(F)F)OC
ZINC08441986	6.18	8.94	-41.39	2	6	1	469.439	6	COc1ccc(cc1)NC(=O)c\2cc3cccc(c3o/c2=[NH+]/c4cccc(c4)C(F)(F)F)OC
ZINC00633661	6.13	11.11	-29.18	2	6	1	429.496	5	Cc1ccc(c(c1)/[NH+]=c\2/c(cc3cccc(c3o2)OC)C(=O)Nc4ccc(cc4)OC)C
ZINC00633661	6.13	8.89	-31.13	2	6	1	429.496	5	Cc1ccc(c(c1)/[NH+]=c/2\c(cc3cccc(c3o2)OC)C(=O)Nc4ccc(cc4)OC)C
ZINC06296296	6.17	10.69	-33.13	2	5	1	439.413	5	COc1ccc(cc1)NC(=O)c\2cc3ccccc3o/c2=[NH+]\c4cccc(c4)C(F)(F)F
ZINC06296296	6.17	9.08	-39.64	2	5	1	439.413	5	COc1ccc(cc1)NC(=O)c\2cc3ccccc3o/c2=[NH+]/c4cccc(c4)C(F)(F)F
ZINC08441985	5.42	4.12	-12.99	1	6	0	444.579	5	[H]/N=C\1/[C@H](C2=CC[C@H](C[C@@H]2[C@H](C1(C#N)C#N)c3ccc(c(c3)OC)OC(C)C)C(C)(C)C)C#N
ZINC08441985	5.42	3.94	-46.53	2	6	1	445.587	5	CC(C)Oc1ccc(cc1OC)[C@@H]2[C@H]3C[C@@H](CC=C3[C@@H](C(=[NH2+])C2(C#N)C#N)C#N)C(C)(C)C
ZINC08441984	4.62	11.35	-12.44	2	7	0	514.383	4	c1ccc(cc1)COC(=O)N2CC=C3[C@H](C2)[C@H](C(C(=C3C#N)N)(C#N)C#N)c4ccc(cc4)Br
ZINC08441984	4.7	1.15	-49.64	2	7	1	515.391	4	c1ccc(cc1)COC(=O)N2CC=C3[C@H](C2)[C@H](C(C(=[NH2+])C3C#N)(C#N)C#N)c4ccc(cc4)Br
ZINC08441983	2.07	0.29	-11	0	9	0	438.484	2	CC(C)c1c(nc(c(n1)C#N)C#N)c2ccc3c(c2)OCCOCCOCCOCCO3
ZINC08441982	4.62	11.69	-11.9	2	7	0	504.377	4	c1ccc(cc1)COC(=O)N2CC=C3[C@H](C2)[C@H](C(C(=C3C#N)N)(C#N)C#N)c4ccc(cc4Cl)Cl
ZINC08441982	4.7	1.65	-47.03	2	7	1	505.385	4	c1ccc(cc1)COC(=O)N2CC=C3[C@H](C2)[C@H](C(C(=[NH2+])C3C#N)(C#N)C#N)c4ccc(cc4Cl)Cl
ZINC08441981	0.58	4.28	-20.85	0	10	0	440.456	1	c1cc2c(cc1c3cnc(c(n3)C#N)C#N)OCCOCCOCCOCCO2
ZINC08441980	4.59	11.32	-11.32	2	7	0	504.377	4	c1ccc(cc1)COC(=O)N2CC=C3[C@H](C2)[C@H](C(C(=C3C#N)N)(C#N)C#N)c4c(cccc4Cl)Cl
ZINC08441980	4.68	1.55	-40.23	2	7	1	505.385	4	c1ccc(cc1)COC(=O)N2CC=C3[C@H](C2)[C@H](C(C(=[NH2+])C3C#N)(C#N)C#N)c4c(cccc4Cl)Cl
ZINC12416741	3.14	5.69	-9.89	2	3	0	212.252	2	c1ccc(cc1)NC(=O)Nc2ccccc2
ZINC12416741	2.77	3.39	-5.14	2	3	0	212.252	3	c1ccc(cc1)N/C(=N/c2ccccc2)/O
ZINC08441979	3.96	11.19	-12.01	2	7	0	469.932	4	c1ccc(cc1)COC(=O)N2CC=C3[C@H](C2)[C@H](C(C(=C3C#N)N)(C#N)C#N)c4ccccc4Cl
ZINC08441979	4.05	1.81	-44.87	2	7	1	470.94	4	c1ccc(cc1)COC(=O)N2CC=C3[C@H](C2)[C@H](C(C(=[NH2+])C3C#N)(C#N)C#N)c4ccccc4Cl
ZINC08441978	3.52	2.86	-18.95	1	10	0	525.565	7	[H]/N=C/1\[C@H](C2=CCN(C[C@@H]2[C@H](C1(C#N)C#N)c3cc(c(c(c3)OC)OC)OC)C(=O)OCc4ccccc4)C#N
ZINC08441978	3.52	2.69	-55.54	2	10	1	526.573	7	COc1cc(cc(c1OC)OC)[C@@H]2[C@H]3CN(CC=C3[C@@H](C(=[NH2+])C2(C#N)C#N)C#N)C(=O)OCc4ccccc4
ZINC08441977	5.35	1.93	-11.32	0	5	0	432.907	7	COc1ccc(cc1OC)/C=C/C(=O)c2nc3ccccc3n2Cc4ccccc4Cl
ZINC08441976	3.46	9.55	-25.13	1	9	0	495.539	6	[H]/N=C/1\[C@H](C2=CCN(C[C@@H]2[C@H](C1(C#N)C#N)c3cc(ccc3OC)OC)C(=O)OCc4ccccc4)C#N
ZINC08441976	3.46	2.06	-44.35	2	9	1	496.547	6	COc1ccc(c(c1)[C@@H]2[C@H]3CN(CC=C3[C@@H](C(=[NH2+])C2(C#N)C#N)C#N)C(=O)OCc4ccccc4)OC

Supplementary Table S2:
Detailed table of XP Docking using Schrodinger's Glide module

ZINC_ID	xLogP	ApolarDesolvation	PolarDesolvation	H Donors	H Acceptors	Charge	MolecularWeight	RotatableBonds	SMILES
ZINC08441975	6.14	12.25	-9.61	1	5	0	484.408	4	COc1ccc(cc1OC)[C@H]2CC(=Nc3ccccc3S2)c4[nH]c5cc(c(cc5n4)Cl)Cl
ZINC08441974	3.46	2.36	-15.45	1	9	0	495.539	6	[H]/N=C\1/[C@@H](C2=CCN(C[C@@H]2[C@H](C1(C#N)C#N)c3ccc(cc3OC)OC)C(=O)OCc4ccccc4)C#N
ZINC08441974	3.46	2.18	-46.82	2	9	1	496.547	6	COc1ccc(c(c1)OC)[C@@H]2[C@H]3CN(CC=C3[C@H](C(=[NH2+])C2(C#N)C#N)C#N)C(=O)OCc4ccccc4
ZINC08441973	3.54	2.44	-17.15	1	9	0	495.539	6	[H]/N=C\1/[C@@H](C2=CCN(C[C@@H]2[C@H](C1(C#N)C#N)c3ccc(c(c3)OC)OC)C(=O)OCc4ccccc4)C#N
ZINC08441973	3.54	2.26	-51.3	2	9	1	496.547	6	COc1ccc(cc1OC)[C@@H]2[C@H]3CN(CC=C3[C@H](C(=[NH2+])C2(C#N)C#N)C#N)C(=O)OCc4ccccc4
ZINC08441972	3.24	9.96	-19.19	1	9	0	495.539	6	[H]/N=C\1/[C@H](C2=CCN(C[C@@H]2[C@H](C1(C#N)C#N)c3cccc(c3OC)OC)C(=O)OCc4ccccc4)C#N
ZINC08441972	3.24	2.33	-48.45	2	9	1	496.547	6	COc1cccc(c1OC)[C@@H]2[C@H]3CN(CC=C3[C@@H](C(=[NH2+])C2(C#N)C#N)C#N)C(=O)OCc4ccccc4
ZINC06446605	2.28	8.91	-12.09	2	8	0	425.448	4	c1ccc(cc1)COC(=O)N2CC=C3[C@H](C2)[C@H](C(C(=C3C#N)N)(C#N)C#N)c4ccoc4
ZINC06446605	2.37	2.69	-46.29	2	8	1	426.456	4	c1ccc(cc1)COC(=O)N2CC=C3[C@H](C2)[C@H](C(C(=[NH2+])C3C#N)(C#N)C#N)c4ccoc4
ZINC08441971	6.14	12.25	-9.6	1	5	0	484.408	4	COc1ccc(cc1OC)[C@@H]2CC(=Nc3ccccc3S2)c4[nH]c5cc(c(cc5n4)Cl)Cl
ZINC08441970	3.57	10.12	-20.54	1	8	0	483.503	5	[H]/N=C\1/[C@H](C2=CCN(C[C@@H]2[C@H](C1(C#N)C#N)c3cc(ccc3F)OC)C(=O)OCc4ccccc4)C#N
ZINC08441970	3.57	2.64	-46.02	2	8	1	484.511	5	COc1ccc(c(c1)[C@@H]2[C@H]3CN(CC=C3[C@@H](C(=[NH2+])C2(C#N)C#N)C#N)C(=O)OCc4ccccc4)F
ZINC06446604	3.23	10.15	-11.52	2	7	0	441.516	4	c1ccc(cc1)COC(=O)N2CC=C3[C@H](C2)[C@H](C(C(=C3C#N)N)(C#N)C#N)c4cccs4
ZINC06446604	3.32	1.22	-45.46	2	7	1	442.524	4	c1ccc(cc1)COC(=O)N2CC=C3[C@H](C2)[C@H](C(C(=[NH2+])C3C#N)(C#N)C#N)c4cccs4
ZINC08441969	4.18	11.28	-13.05	2	7	0	503.484	5	c1ccc(cc1)COC(=O)N2CC=C3[C@H](C2)[C@H](C(C(=C3C#N)N)(C#N)C#N)c4ccccc4C(F)(F)F
ZINC08441969	4.27	3.58	-45.45	2	7	1	504.492	5	c1ccc(cc1)COC(=O)N2CC=C3[C@H](C2)[C@H](C(C(=[NH2+])C3C#N)(C#N)C#N)c4ccccc4C(F)(F)F
ZINC08441968	4.42	-1.68	-33.3	4	6	0	546.762	12	CCc1cccc(c1NC(=S)NC(=O)c2ccc(cc2)C(=O)NC(=S)Nc3c(cccc3CC)CC)CC
ZINC08441966	3.51	9.19	-13.62	2	8	0	453.502	4	CCOC(=O)N1CC=C2[C@H](C1)[C@H](C(C(=C2C#N)N)(C#N)C#N)c3ccc(c4c3cccc4)OC
ZINC08441966	3.59	2.01	-49.3	2	8	1	454.51	4	CCOC(=O)N1CC=C2[C@H](C1)[C@H](C(C(=[NH2+])C2C#N)(C#N)C#N)c3ccc(c4c3cccc4)OC
ZINC05316280	5.13	-1.25	-11.38	2	7	0	501.558	7	CC1=NC(=C([C@@H](C1C(=O)Nc2ccccc2OC)c3ccccc3F)C(=O)Nc4ccccc4OC)C
ZINC05316280	5.31	-1.65	-11.64	2	7	0	501.558	7	CC1=NC(=C)C([C@H](C1C(=O)Nc2ccccc2OC)c3ccccc3F)C(=O)Nc4ccccc4OC
ZINC05316280	5.31	0.17	-19.04	2	7	0	501.558	7	CC1=NC(=C)C([C@H]([C@H]1C(=O)Nc2ccccc2OC)c3ccccc3F)C(=O)Nc4ccccc4OC
ZINC05316280	5.13	-1.19	-11.97	2	7	0	501.558	7	CC1=NC(=C([C@H]([C@@H]1C(=O)Nc2ccccc2OC)c3ccccc3F)C(=O)Nc4ccccc4OC)C
ZINC08441965	4.54	2.97	-63.76	2	6	1	472.572	4	[H]/N=C/1\[C@H](C2=CC[NH+](C[C@@H]2[C@H](C1(C#N)C#N)c3ccc(c4c3cccc4)OC)Cc5ccccc5)C#N
ZINC08441965	4.54	2.8	-123.58	3	6	2	473.58	4	COc1ccc(c2c1cccc2)[C@@H]3[C@H]4C[NH+](CC=C4[C@@H](C(=[NH2+])C3(C#N)C#N)C#N)Cc5ccccc5
ZINC08441964	4.08	-1.21	-7.67	0	3	0	285.387	7	CCCCCCCC/N=C/1\CC(=O)C(=O)c2c1cccc2
ZINC08441962	5.31	12.54	-14.67	2	5	0	473.623	7	CC1=C([C@H](C(=C(N1)SCc2cccc(c2)OC)C#N)c3cccs3)C(=O)Nc4ccccc4
ZINC08441962	5.83	0.23	-19.16	1	5	0	473.623	7	COc1cccc(c1)CSC2=NC(=C)C([C@H]([C@H]2C#N)c3cccs3)C(=O)Nc4ccccc4
ZINC08441962	5.83	-1.72	-11.52	1	5	0	473.623	7	COc1cccc(c1)CSC2=NC(=C)C([C@H](C2C#N)c3cccs3)C(=O)Nc4ccccc4
ZINC08441962	5.64	-1.18	-10.61	1	5	0	473.623	7	CC1=C([C@H]([C@@H](C(=N1)SCc2cccc(c2)OC)C#N)c3cccs3)C(=O)Nc4ccccc4
ZINC08441961	7.75	12.32	-18.58	2	7	0	617.752	8	c1ccc(cc1)[C@](c2cc3ccccc3[nH]2)(c4cc5ccccc5n4S(=O)(=O)c6ccccc6)NS(=O)(=O)c7ccccc7
ZINC00061509	2.28	5.82	-6.96	0	4	0	194.186	4	COC(=O)c1ccc(cc1)C(=O)OC
ZINC03770286	6.95	-1.21	-12.88	1	6	0	505.599	3	CCOc1ccc(cc1)[C@H]2C3=C(c4ccccc4CC3)N=c5n2c(=O)/c(=C/6\c7ccccc7NC6=O)/s5
ZINC03770288	6.95	-1.22	-13.42	1	6	0	505.599	3	CCOc1ccc(cc1)[C@@H]2C3=C(c4ccccc4CC3)N=c5n2c(=O)/c(=C/6\c7ccccc7NC6=O)/s5
ZINC08441960	5.31	12.54	-14.67	2	5	0	473.623	7	CC1=C([C@@H](C(=C(N1)SCc2cccc(c2)OC)C#N)c3cccs3)C(=O)Nc4ccccc4
ZINC08441960	5.64	-1.3	-10.83	1	5	0	473.623	7	CC1=C([C@@H]([C@H](C(=N1)SCc2cccc(c2)OC)C#N)c3cccs3)C(=O)Nc4ccccc4
ZINC08441960	5.83	-1.62	-11.72	1	5	0	473.623	7	COc1cccc(c1)CSC2=NC(=C)C([C@@H](C2C#N)c3cccs3)C(=O)Nc4ccccc4
ZINC08441960	5.83	-0.07	-12.39	1	5	0	473.623	7	COc1cccc(c1)CSC2=NC(=C)C([C@@H]([C@@H]2C#N)c3cccs3)C(=O)Nc4ccccc4
ZINC08441959	6.41	-2.54	-13.12	1	5	0	467.575	1	c1ccc2c(c1)CCC3=C2N=c4n(c(=O)/c(=C/5\c6ccccc6NC5=O)/s4)[C@@H]3c7cccs7
ZINC08441958	4.59	11.36	-11.92	2	7	0	514.383	4	c1ccc(cc1)COC(=O)N2CC=C3[C@H](C2)[C@H](C(C(=C3C#N)N)(C#N)C#N)c4cccc(c4)Br
ZINC08441958	4.68	1.16	-47.39	2	7	1	515.391	4	c1ccc(cc1)COC(=O)N2CC=C3[C@H](C2)[C@H](C(C(=[NH2+])C3C#N)(C#N)C#N)c4cccc(c4)Br
ZINC08441957	7.95	2.32	-79.6	0	6	-1	547.587	4	c1ccc2c(c1)CCC3=C2N=c4n(c(=O)/c(=C\c5ccc(o5)c6cccc(c6)C(=O)[O-])/s4)[C@H]3c7cccc(c7)F
ZINC08441956	4.46	11.25	-12.04	2	7	0	469.932	4	c1ccc(cc1)COC(=O)N2CC=C3[C@H](C2)[C@H](C(C(=C3C#N)N)(C#N)C#N)c4cccc(c4)Cl
ZINC08441956	4.55	1.78	-47.48	2	7	1	470.94	4	c1ccc(cc1)COC(=O)N2CC=C3[C@H](C2)[C@H](C(C(=[NH2+])C3C#N)(C#N)C#N)c4cccc(c4)Cl
ZINC08441955	7.95	2.42	-79.06	0	6	-1	547.587	4	c1ccc2c(c1)CCC3=C2N=c4n(c(=O)/c(=C\c5ccc(o5)c6cccc(c6)C(=O)[O-])/s4)[C@@H]3c7cccc(c7)F
ZINC08441953	3.43	10.25	-21.76	1	8	0	465.513	5	[H]/N=C/1\[C@H](C2=CCN(C[C@@H]2[C@H](C1(C#N)C#N)c3ccccc3OC)C(=O)OCc4ccccc4)C#N
ZINC08441953	3.43	2.07	-44.01	2	8	1	466.521	5	COc1ccccc1[C@@H]2[C@H]3CN(CC=C3[C@@H](C(=[NH2+])C2(C#N)C#N)C#N)C(=O)OCc4ccccc4
ZINC08441952	9.77	1.92	-13.69	0	4	0	613.541	5	c1ccc2c(c1)CCC3=C2N=c4n(c(=O)/c(=C/c5ccccc5OCc6ccc(cc6Cl)Cl)/s4)[C@H]3c7cccc(c7)F
ZINC08441951	4.42	10.66	-16.91	1	9	0	523.593	8	[H]/N=C\1/[C@@H](C2=CCN(C[C@@H]2[C@H](C1(C#N)C#N)c3ccc(c(c3)OCCC)OC)C(=O)OCc4ccccc4)C#N
ZINC08441951	4.42	10.75	-51.39	2	9	1	524.601	8	CCCOc1cc(ccc1OC)[C@@H]2[C@H]3CN(CC=C3[C@H](C(=[NH2+])C2(C#N)C#N)C#N)C(=O)OCc4ccccc4
ZINC08441950	9.77	1.8	-11.2	0	4	0	613.541	5	c1ccc2c(c1)CCC3=C2N=c4n(c(=O)/c(=C/c5ccccc5OCc6ccc(cc6Cl)Cl)/s4)[C@@H]3c7cccc(c7)F
ZINC03770206	9.12	2.07	-14.2	0	4	0	579.096	5	c1ccc2c(c1)CCC3=C2N=c4n(c(=O)/c(=C/c5ccccc5OCc6ccccc6Cl)/s4)[C@H]3c7cccc(c7)F
ZINC08441949	3.57	7.69	-18.83	1	10	0	509.522	5	[H]/N=C\1/[C@H](C2=CCN(C[C@@H]2[C@H](C1(C#N)C#N)c3cc4c(c(c3)OC)OCO4)C(=O)OCc5ccccc5)C#N
ZINC08441949	3.57	7.77	-55.01	2	10	1	510.53	5	COc1cc(cc2c1OCO2)[C@@H]3[C@H]4CN(CC=C4[C@@H](C(=[NH2+])C3(C#N)C#N)C#N)C(=O)OCc5ccccc5
ZINC03770208	9.12	1.95	-11.47	0	4	0	579.096	5	c1ccc2c(c1)CCC3=C2N=c4n(c(=O)/c(=C/c5ccccc5OCc6ccccc6Cl)/s4)[C@@H]3c7cccc(c7)F
ZINC08441948	3.25	8.82	-16.76	1	10	0	525.565	7	[H]/N=C\1/[C@H](C2=CCN(C[C@@H]2[C@H](C1(C#N)C#N)c3ccc(c(c3OC)OC)OC)C(=O)OCc4ccccc4)C#N
ZINC08441948	3.25	8.91	-49.27	2	10	1	526.573	7	COc1ccc(c(c1OC)OC)[C@@H]2[C@H]3CN(CC=C3[C@@H](C(=[NH2+])C2(C#N)C#N)C#N)C(=O)OCc4ccccc4
ZINC03770229	8.49	2.01	-12.7	0	4	0	544.651	5	c1ccc(cc1)COc2ccccc2/C=c\3/c(=O)n4c(=NC5=C([C@H]4c6cccc(c6)F)CCc7c5cccc7)s3
ZINC08441947	3.82	2.92	-56.32	2	6	1	424.528	3	[H]/N=C/1\C(C2=CC[NH+](C[C@@H]2[C@H](C1(C#N)C#N)c3ccc(c4c3cccc4)OC)C(C)C)C#N
ZINC08441947	3.82	2.75	-114.58	3	6	2	425.536	3	CC(C)[NH+]1CC=C2[C@H](C1)[C@H](C(C(=[NH2+])C2C#N)(C#N)C#N)c3ccc(c4c3cccc4)OC
ZINC03770231	8.49	2.13	-15.44	0	4	0	544.651	5	c1ccc(cc1)COc2ccccc2/C=c\3/c(=O)n4c(=NC5=C([C@@H]4c6cccc(c6)F)CCc7c5cccc7)s3

Supplementary Table S2:
Detailed table of XP Docking using Schrodinger's Glide module

ZINC_ID	xLogP	ApolarDesolvation	PolarDesolvation	H Donors	H Acceptors	Charge	MolecularWeight	RotatableBonds	SMILES
ZINC03770224	8.06	0.88	-10.9	0	4	0	561.476	4	CCOc1ccc(cc1Br)/C=c/2\c(=O)n3c(=NC4=C([C@H]3c5cccc(c5)F)CCc6c4cccc6)s2
ZINC08441946	0.26	0.37	-23.57	5	11	0	427.417	9	CCOC(=O)Cc1c2c(n[nH]1)OC(=C([C@H]2c3ccc(c(c3)OC)OCC(=O)N)C#N)N
ZINC03770226	8.06	1.01	-13.37	0	4	0	561.476	4	CCOc1ccc(cc1Br)/C=c/2\c(=O)n3c(=NC4=C([C@@H]3c5cccc(c5)F)CCc6c4cccc6)s2
ZINC08441945	0.26	0.37	-23.87	5	11	0	427.417	9	CCOC(=O)Cc1c2c(n[nH]1)OC(=C([C@@H]2c3ccc(c(c3)OC)OCC(=O)N)C#N)N
ZINC08441944	8.61	2.73	-10.78	0	4	0	562.641	5	c1ccc2c(c1)CCC3=C2N=c4n(c(=O)/c(=C\c5ccccc5OCc6ccccc6F)/s4)[C@H]3c7cccc(c7)F
ZINC08441943	3.69	5.05	-10.58	3	6	0	423.27	3	COc1ccc(cc1Br)[C@H]2c3c(n[nH]c3OC(=C2C#N)N)c4ccccc4
ZINC08441942	8.61	2.71	-13.97	0	4	0	562.641	5	c1ccc2c(c1)CCC3=C2N=c4n(c(=O)/c(=C\c5ccccc5OCc6ccccc6F)/s4)[C@@H]3c7cccc(c7)F
ZINC08441941	3.69	5.12	-10.42	3	6	0	423.27	3	COc1ccc(cc1Br)[C@@H]2c3c(n[nH]c3OC(=C2C#N)N)c4ccccc4
ZINC08441940	9.25	2.42	-13.65	0	4	0	597.509	3	Cc1ccc(c(c1)Br)c2ccc(o2)/C=c/3\c(=O)n4c(=NC5=C([C@H]4c6cccc(c6)F)CCc7c5cccc7)s3
ZINC08441939	9.25	2.45	-16.67	0	4	0	597.509	3	Cc1ccc(c(c1)Br)c2ccc(o2)/C=c/3\c(=O)n4c(=NC5=C([C@@H]4c6cccc(c6)F)CCc7c5cccc7)s3
ZINC08441938	4.38	1.96	-15.58	0	5	0	442.537	8	CCOC(=O)C1[C@@H](C(=C(N=C1C)SCC(=O)c2ccc(cc2)F)C#N)c3cccs3
ZINC08441938	4.76	1.1	-12	0	5	0	442.537	8	CCOC(=O)C1=C(N=C(C([C@H]1c2cccs2)C#N)SCC(=O)c3ccc(cc3)F)C
ZINC08441938	4.94	0.17	-11.99	0	5	0	442.537	8	CCOC(=O)C1[C@@H](C(C(=NC1=C)SCC(=O)c2ccc(cc2)F)C#N)c3cccs3
ZINC08441937	7.46	-0.69	-15.32	1	5	0	610.448	3	COc1cc(cc(c1O)I)/C=c/2\c(=O)n3c(=NC4=C([C@@H]3c5cccc(c5)F)CCc6c4cccc6)s2
ZINC08441936	6.79	1.07	-9.92	0	3	0	444.556	2	c1ccc2c(c1)CCC3=C2N=c4n(c(=O)/c(=C\c5cccs5)/s4)[C@H]3c6cccc(c6)F
ZINC08441935	4.38	1.85	-13.3	0	5	0	442.537	8	CCOC(=O)C1[C@H](C(=C(N=C1C)SCC(=O)c2ccc(cc2)F)C#N)c3cccs3
ZINC08441935	4.94	0.14	-12.7	0	5	0	442.537	8	CCOC(=O)C1[C@H](C(C(=NC1=C)SCC(=O)c2ccc(cc2)F)C#N)c3cccs3
ZINC08441935	4.76	1.14	-10.81	0	5	0	442.537	8	CCOC(=O)C1=C(N=C(C([C@@H]1c2cccs2)C#N)SCC(=O)c3ccc(cc3)F)C
ZINC08441934	6.79	1.05	-10.12	0	3	0	444.556	2	c1ccc2c(c1)CCC3=C2N=c4n(c(=O)/c(=C\c5cccs5)/s4)[C@@H]3c6cccc(c6)F
ZINC02416783	6.53	1.98	-13.65	0	5	0	498.579	4	COc1ccc(cc1OC)/C=c/2\c(=O)n3c(=NC4=C([C@H]3c5cccc(c5)F)CCc6c4cccc6)s2
ZINC08441933	3.77	7.86	-10.38	2	7	0	431.54	7	CCCN1CC=C2[C@H](C1)[C@H](C(C(=C2C#N)N)(C#N)C#N)c3ccc(c(c3)OC)OCCC
ZINC08441933	3.85	2.81	-119.31	3	7	2	433.556	7	CCC[NH+]1CC=C2[C@H](C1)[C@H](C(C(=[NH2+])C2C#N)(C#N)C#N)c3ccc(c(c3)OC)OCCC
ZINC02416785	6.53	2	-15.77	0	5	0	498.579	4	COc1ccc(cc1OC)/C=c/2\c(=O)n3c(=NC4=C([C@@H]3c5cccc(c5)F)CCc6c4cccc6)s2
ZINC08441932	3.85	3.34	-63.37	2	7	1	432.548	7	[H]/N=C/1\C(C2=CC[NH+](C[C@H]2[C@H](C1(C#N)C#N)c3ccc(c(c3)OC)OCCC)CCC)C#N
ZINC08441932	3.85	3.17	-114.69	3	7	2	433.556	7	CCC[NH+]1CC=C2[C@@H](C1)[C@H](C(C(=[NH2+])C2C#N)(C#N)C#N)c3ccc(c(c3)OC)OCCC
ZINC03772287	7.48	2.02	-11.03	0	4	0	505.618	3	CCn1cc(c2c1cccc2)/C=c/3\c(=O)n4c(=NC5=C([C@H]4c6cccc(c6)F)CCc7c5cccc7)s3
ZINC08441931	4.04	3.33	-63.65	2	7	1	432.548	7	[H]/N=C/1\[C@@H](C2=CC[NH+](C[C@@H]2[C@@H](C1(C#N)C#N)c3ccc(c(c3)OC)OCCCCC)C)C#N
ZINC08441931	4.04	3.16	-113.57	3	7	2	433.556	7	CCCCCOc1ccc(cc1OC)[C@H]2[C@H]3C[NH+](CC=C3[C@H](C(=[NH2+])C2(C#N)C#N)C#N)C
ZINC03772289	7.48	2.14	-14.26	0	4	0	505.618	3	CCn1cc(c2c1cccc2)/C=c/3\c(=O)n4c(=NC5=C([C@@H]4c6cccc(c6)F)CCc7c5cccc7)s3
ZINC08441930	4.04	8.18	-12	1	7	0	431.54	7	[H]/N=C\1/[C@@H](C2=CCN(C[C@@H]2[C@H](C1(C#N)C#N)c3ccc(c(c3)OC)OCCCCC)C)C#N
ZINC08441930	4.04	2.98	-117.83	3	7	2	433.556	7	CCCCCOc1ccc(cc1OC)[C@@H]2[C@H]3C[NH+](CC=C3[C@H](C(=[NH2+])C2(C#N)C#N)C#N)C
ZINC08441929	9.38	2.43	-13.92	0	4	0	602.134	4	c1ccc2c(c1)CCC3=C2N=c4n(c(=O)/c(=C/c5cn(c6c5cccc6)Cc7ccc(cc7)Cl)/s4)[C@H]3c8cccc(c8)F
ZINC08441928	4.04	3.1	-63.62	2	7	1	432.548	7	[H]/N=C/1\[C@@H](C2=CC[NH+](C[C@H]2[C@@H](C1(C#N)C#N)c3ccc(c(c3)OC)OCCCCC)C)C#N
ZINC08441928	4.04	2.93	-119.74	3	7	2	433.556	7	CCCCCOc1ccc(cc1OC)[C@H]2[C@@H]3C[NH+](CC=C3[C@H](C(=[NH2+])C2(C#N)C#N)C#N)C
ZINC08441927	9.38	2.37	-16.28	0	4	0	602.134	4	c1ccc2c(c1)CCC3=C2N=c4n(c(=O)/c(=C/c5cn(c6c5cccc6)Cc7ccc(cc7)Cl)/s4)[C@@H]3c8cccc(c8)F
ZINC08441926	4.04	3.61	-63.5	2	7	1	432.548	7	[H]/N=C/1\[C@@H](C2=CC[NH+](C[C@H]2[C@H](C1(C#N)C#N)c3ccc(c(c3)OC)OCCCCC)C)C#N
ZINC08441926	4.04	3.44	-111.87	3	7	2	433.556	7	CCCCCOc1ccc(cc1OC)[C@@H]2[C@@H]3C[NH+](CC=C3[C@H](C(=[NH2+])C2(C#N)C#N)C#N)C
ZINC08441925	8.84	1.89	-13.36	0	5	0	559.735	7	CCCCn1cc(c2c1cccc2)/C=c\3/c(=O)n4c(=NC5=C([C@H]4c6ccc(cc6)OCC)CCc7c5cccc7)s3
ZINC08441924	4.84	10.32	-9.29	2	5	0	439.95	4	c1ccc(cc1)CCN2CC=C3[C@H](C2)[C@H](C(C(=C3C#N)N)(C#N)C#N)c4ccc(cc4)Cl
ZINC08441924	4.93	2.33	-127.32	3	5	2	441.966	4	c1ccc(cc1)CC[NH+]2CC=C3[C@H](C2)[C@H](C(C(=[NH2+])C3C#N)(C#N)C#N)c4ccc(cc4)Cl
ZINC06195024	2.85	6.32	-14.1	2	9	0	433.468	5	CCOC(=O)N1CC=C2[C@H](C1)[C@H](C(C(=C2C#N)N)(C#N)C#N)c3cc(cc(c3)OC)OC
ZINC06195024	2.94	1.82	-47.83	2	9	1	434.476	5	CCOC(=O)N1CC=C2[C@H](C1)[C@H](C(C(=[NH2+])C2C#N)(C#N)C#N)c3cc(cc(c3)OC)OC
ZINC08441923	8.84	1.92	-13.2	0	5	0	559.735	7	CCCCn1cc(c2c1cccc2)/C=c\3/c(=O)n4c(=NC5=C([C@@H]4c6ccc(cc6)OCC)CCc7c5cccc7)s3
ZINC08441922	4.28	2.73	-16.94	1	9	0	523.593	7	[H]/N=C\1/[C@@H](C2=CCN(C[C@@H]2[C@H](C1(C#N)C#N)c3ccc(c(c3)OC)OC(C)C)C(=O)OCc4ccccc4)C#N
ZINC08441922	4.28	2.55	-50.9	2	9	1	524.601	7	CC(C)Oc1ccc(cc1OC)[C@@H]2[C@H]3CN(CC=C3[C@H](C(=[NH2+])C2(C#N)C#N)C#N)C(=O)OCc4ccccc4
ZINC00631600	4.47	-1.91	-19.04	0	5	0	433.327	3	Cc1ccc(c(c1)Br)c2ccc(o2)/C=C/3\C(=O)N=C(S3)N4CCOCC4
ZINC08441919	7.45	3.9	-15.57	0	4	0	623.483	4	c1ccc2c(c1)CCC3=C2N=c4n(c(=O)/c(=C\c5ccc(o5)c6cccc(c6)C(F)(F)F)/s4)[C@H]3c7ccc(cc7Cl)Cl
ZINC08441920	3.51	9.83	-12.42	2	8	0	453.502	4	CCOC(=O)N1CC=C2[C@H](C1)[C@H](C(C(=C2C#N)N)(C#N)C#N)c3c4ccccc4ccc3OC
ZINC08441920	3.59	2.5	-43.38	2	8	1	454.51	4	CCOC(=O)N1CC=C2[C@H](C1)[C@H](C(C(=[NH2+])C2C#N)(C#N)C#N)c3c4ccccc4ccc3OC
ZINC08441918	7.45	3.77	-14.93	0	4	0	623.483	4	c1ccc2c(c1)CCC3=C2N=c4n(c(=O)/c(=C\c5ccc(o5)c6cccc(c6)C(F)(F)F)/s4)[C@@H]3c7ccc(cc7Cl)Cl
ZINC08441917	4.54	11.6	-9.84	1	6	0	471.564	4	[H]/N=C\1/[C@@H](C2=CCN(C[C@@H]2[C@H](C1(C#N)C#N)c3c4ccccc4ccc3OC)Cc5ccccc5)C#N
ZINC08441917	4.54	3.05	-112.04	3	6	2	473.58	4	COc1ccc2ccccc2c1[C@@H]3[C@H]4C[NH+](CC=C4[C@H](C(=[NH2+])C3(C#N)C#N)C#N)Cc5ccccc5
ZINC08441916	8.19	1.27	-11.17	0	3	0	507.417	2	c1ccc2c(c1)CCC3=C2N=c4n(c(=O)/c(=C\c5ccc(cc5)F)/s4)[C@H]3c6ccc(cc6Cl)Cl
ZINC08441915	3.94	9.06	-7.97	2	6	0	423.52	4	CCCN1CC=C2[C@H](C1)[C@H](C(C(=C2C#N)N)(C#N)C#N)c3c4ccccc4ccc3OC
ZINC08441915	4.02	3.11	-109.39	3	6	2	425.536	4	CCC[NH+]1CC=C2[C@H](C1)[C@H](C(C(=[NH2+])C2C#N)(C#N)C#N)c3c4ccccc4ccc3OC
ZINC08441914	8.19	1.25	-11.35	0	3	0	507.417	2	c1ccc2c(c1)CCC3=C2N=c4n(c(=O)/c(=C\c5ccc(cc5)F)/s4)[C@@H]3c6ccc(cc6Cl)Cl
ZINC08441913	3.73	8.82	-7.87	2	6	0	423.52	3	CC(C)N1CC=C2[C@H](C1)[C@H](C(C(=C2C#N)N)(C#N)C#N)c3c4ccccc4ccc3OC
ZINC08441913	3.82	10.14	-104.97	3	6	2	425.536	3	CC(C)[NH+]1CC=C2[C@H](C1)[C@H](C(C(=[NH2+])C2C#N)(C#N)C#N)c3c4ccccc4ccc3OC
ZINC06446299	2.1	8.97	-13.66	2	8	0	436.475	4	c1ccc(cc1)COC(=O)N2CC=C3[C@H](C2)[C@H](C(C(=C3C#N)N)(C#N)C#N)c4cccnc4
ZINC08441912	9.06	-0.14	-6.93	0	3	0	615.323	2	c1ccc2c(c1)CCC3=C2N=c4n(c(=O)/c(=C\c5ccccc5I)/s4)[C@H]3c6ccc(cc6Cl)Cl
ZINC08441911	4.49	11.25	-12.44	2	7	0	469.932	4	c1ccc(cc1)COC(=O)N2CC=C3[C@H](C2)[C@H](C(C(=C3C#N)N)(C#N)C#N)c4ccc(cc4)Cl

Supplementary Table S2:
Detailed table of XP Docking using Schrodinger's Glide module

ZINC_ID	xLogP	ApolarDesolvation	PolarDesolvation	H Donors	H Acceptors	Charge	MolecularWeight	RotatableBonds	SMILES
ZINC08441911	4.57	1.76	-49.47	2	7	1	470.94	4	c1ccc(cc1)COC(=O)N2CC=C3[C@H](C2)[C@H](C(C(=[NH2+])C3C#N)(C#N)C#N)c4ccc(cc4)Cl
ZINC08441910	9.06	-0.32	-8.52	0	3	0	615.323	2	c1ccc2c(c1)CCC3=C2N=c4n(c(=O)/c(=C\c5ccccc5I)/s4)[C@@H]3c6ccc(cc6Cl)Cl
ZINC08441909	3.95	10.11	-23.97	1	8	0	465.513	5	[H]/N=C\1/[C@H](C2=CCN(C[C@@H]2[C@H](C1(C#N)C#N)c3ccc(cc3)OC)C(=O)OCc4ccccc4)C#N
ZINC08441909	3.95	1.93	-49.12	2	8	1	466.521	5	COc1ccc(cc1)[C@@H]2[C@H]3CN(CC=C3[C@@H](C(=[NH2+])C2(C#N)C#N)C#N)C(=O)OCc4ccccc4
ZINC08441908	8.2	1.87	-13.73	0	5	0	547.463	4	COC(=O)c1ccc(cc1)/C=c\2/c(=O)n3c(=NC4=C([C@H]3c5ccc(cc5Cl)Cl)CCc6c4cccc6)s2
ZINC00882048	5.3	-1.83	-21.98	2	6	0	445.544	6	Cc1cc(c2c(c1)nc(o2)c3cccc(c3C)NC(=S)NC(=O)c4ccc(cc4)OC)C
ZINC00882039	5.17	-2.29	-28.86	2	5	0	433.558	6	Cc1ccc(cc1NC(=S)NC(=O)c2ccc(cc2)OC)c3nc4ccccc4s3
ZINC00882033	4.18	-3.07	-28.37	2	7	0	433.489	7	COc1ccc(cc1)c2nc3cc(ccc3o2)NC(=S)NC(=O)c4ccc(cc4)OC
ZINC08441906	8.2	1.9	-14.94	0	5	0	547.463	4	COC(=O)c1ccc(cc1)/C=c\2/c(=O)n3c(=NC4=C([C@@H]3c5ccc(cc5Cl)Cl)CCc6c4cccc6)s2
ZINC08441905	4.75	11.12	-16.79	1	7	0	493.611	8	[H]/N=C\1/[C@H](C2=CCN(C[C@@H]2[C@H](C1(C#N)C#N)c3ccc(c(c3)OCC)OCCC)Cc4ccccc4)C#N
ZINC08441905	4.75	2.99	-126.02	3	7	2	495.627	8	CCCOc1ccc(cc1OCC)[C@@H]2[C@H]3C[NH+](CC=C3[C@@H](C(=[NH2+])C2(C#N)C#N)C#N)Cc4ccccc4
ZINC00882026	4.61	-1.6	-16.56	2	5	0	448.367	6	COc1ccc(cc1)C(=O)NC(=S)Nc2nc(cs2)c3ccc(cc3)Br
ZINC02064406	4.15	-1.35	-32.37	2	9	0	466.45	7	COc1ccc(cc1[N+](=O)[O-])C(=O)NC(=S)Nc2ccc3c(c2)nc(o3)c4cccc(c4)F
ZINC02064405	4.64	-2.19	-33.83	2	9	0	482.905	7	COc1ccc(cc1[N+](=O)[O-])C(=O)NC(=S)Nc2ccc3c(c2)nc(o3)c4ccccc4Cl
ZINC02064404	4.13	-1.42	-40.52	2	9	0	466.45	7	COc1ccc(cc1[N+](=O)[O-])C(=O)NC(=S)Nc2ccc3c(c2)nc(o3)c4ccccc4F
ZINC08441904	4.25	10.32	-16.91	1	7	0	479.584	7	[H]/N=C\1/[C@H](C2=CCN(C[C@@H]2[C@H](C1(C#N)C#N)c3ccc(c(c3)OCC)OCC)Cc4ccccc4)C#N
ZINC08441904	4.25	2.92	-126.04	3	7	2	481.6	7	CCOc1ccc(cc1OCC)[C@@H]2[C@H]3C[NH+](CC=C3[C@@H](C(=[NH2+])C2(C#N)C#N)C#N)Cc4ccccc4
ZINC08441903	9.96	1.87	-13.23	0	4	0	636.579	4	c1ccc2c(c1)CCC3=C2N=c4n(c(=O)/c(=C\c5cn(c6c5cccc6)Cc7ccccc7F)/s4)[C@H]3c8ccc(cc8Cl)Cl
ZINC00881990	2.95	-0.44	-28.27	2	7	0	438.303	6	Cc1cc(cc(c1NC(=S)NC(=O)c2ccc(c(c2)[N+](=O)[O-])OC)C)Br
ZINC02064400	3.59	-0.82	-32.9	3	9	0	464.503	8	Cc1ccccc1C(=O)Nc2cccc(c2)NC(=S)NC(=O)c3ccc(c(c3)[N+](=O)[O-])OC
ZINC02064399	4.84	-1.49	-35.72	2	9	0	476.514	7	Cc1cc2c(cc1C)oc(n2)c3ccc(cc3)NC(=S)NC(=O)c4ccc(c(c4)[N+](=O)[O-])OC
ZINC08441902	4.02	8.82	-17.78	1	7	0	445.567	7	[H]/N=C/1\[C@H](C2=CCN(C[C@@H]2[C@H](C1(C#N)C#N)c3ccc(c(c3)OCC)OCCC)C(C)C)C#N
ZINC08441902	4.02	2.88	-118.65	3	7	2	447.583	7	CCCOc1ccc(cc1OCC)[C@@H]2[C@H]3C[NH+](CC=C3[C@@H](C(=[NH2+])C2(C#N)C#N)C#N)C(C)C
ZINC08441901	9.96	1.65	-12.45	0	4	0	636.579	4	c1ccc2c(c1)CCC3=C2N=c4n(c(=O)/c(=C\c5cn(c6c5cccc6)Cc7ccccc7F)/s4)[C@@H]3c8ccc(cc8Cl)Cl
ZINC02064398	4.93	-1.62	-35.24	2	9	0	476.514	8	CCc1ccc2c(c1)nc(o2)c3ccc(cc3)NC(=S)NC(=O)c4ccc(c(c4)[N+](=O)[O-])OC
ZINC02064397	4.46	-1.78	-35.57	2	9	0	462.487	7	Cc1ccc2c(c1)nc(o2)c3ccc(cc3)NC(=S)NC(=O)c4ccc(c(c4)[N+](=O)[O-])OC
ZINC02064396	4.04	-2.1	-37.08	2	9	0	448.46	7	COc1ccc(cc1[N+](=O)[O-])C(=O)NC(=S)Nc2ccc(cc2)c3nc4ccccc4o3
ZINC02064395	4.69	-2.25	-33.64	2	9	0	482.905	7	COc1ccc(cc1[N+](=O)[O-])C(=O)NC(=S)Nc2ccc(cc2)c3nc4cc(ccc4o3)Cl
ZINC08441900	4.09	3.3	-62.6	2	7	1	446.575	7	[H]/N=C/1\C(C2=CC[NH+](C[C@@H]2[C@H](C1(C#N)C#N)c3ccc(c(c3)OCC)OC(C)C)CCC)C#N
ZINC08441900	4.09	3.13	-119.92	3	7	2	447.583	7	CCC[NH+]1CC=C2[C@H](C1)[C@H](C(C(=[NH2+])C2C#N)(C#N)C#N)c3ccc(c(c3)OCC)OC(C)C
ZINC03770143	9.63	1.02	-13.19	0	4	0	595.551	5	c1ccc(cc1)COc2ccccc2/C=c\3/c(=O)n4c(=NC5=C([C@H]4c6ccc(cc6Cl)Cl)CCc7c5cccc7)s3
ZINC02064394	4.68	-1.81	-34.88	2	8	0	464.528	7	COc1ccc(cc1[N+](=O)[O-])C(=O)NC(=S)Nc2ccc(cc2)c3nc4ccccc4s3
ZINC01849163	5.19	-0.88	-32.46	2	9	0	490.541	7	Cc1cc(c2c(c1)nc(o2)c3cccc(c3C)NC(=S)NC(=O)c4ccc(c(c4)[N+](=O)[O-])OC)C
ZINC08441899	3.59	2.98	-61.66	2	7	1	432.548	6	[H]/N=C/1\C(C2=CC[NH+](C[C@@H]2[C@H](C1(C#N)C#N)c3ccc(c(c3)OCC)OC(C)C)CC)C#N
ZINC08441899	3.59	2.81	-118.19	3	7	2	433.556	6	CC[NH+]1CC=C2[C@H](C1)[C@H](C(C(=[NH2+])C2C#N)(C#N)C#N)c3ccc(c(c3)OCC)OC(C)C
ZINC03770145	9.63	1	-12.94	0	4	0	595.551	5	c1ccc(cc1)COc2ccccc2/C=c\3/c(=O)n4c(=NC5=C([C@@H]4c6ccc(cc6Cl)Cl)CCc7c5cccc7)s3
ZINC02064392	5.3	-1.09	-38.59	2	9	0	490.541	8	CCc1ccc2c(c1)nc(o2)c3ccc(c(c3)NC(=S)NC(=O)c4ccc(c(c4)[N+](=O)[O-])OC)C
ZINC02064391	4.9	-1.62	-39.02	2	9	0	476.514	8	CCc1ccc2c(c1)nc(o2)c3cccc(c3)NC(=S)NC(=O)c4ccc(c(c4)[N+](=O)[O-])OC
ZINC02064389	4.64	-2.12	-33.7	2	9	0	482.905	7	COc1ccc(cc1[N+](=O)[O-])C(=O)NC(=S)Nc2cc(ccc2Cl)c3nc4ccccc4o3
ZINC08441898	4.38	2.81	-15.29	1	9	0	503.603	8	[H]/N=C\1/[C@@H](C2=CCN(C[C@@H]2[C@H](C1(C#N)C#N)c3ccc(c(c3)OCC)OCCC)C(=O)OC(C)(C)C)C#N
ZINC08441898	4.38	2.63	-49.21	2	9	1	504.611	8	CCCOc1ccc(cc1OCC)[C@@H]2[C@H]3CN(CC=C3[C@H](C(=[NH2+])C2(C#N)C#N)C#N)C(=O)OC(C)(C)C
ZINC02064388	5.04	-2.03	-32.98	2	9	0	496.932	7	Cc1c(cccc1NC(=S)NC(=O)c2ccc(c(c2)[N+](=O)[O-])OC)c3nc4cc(ccc4o3)Cl
ZINC08441897	9.2	-0.16	-11.37	0	4	0	612.376	4	CCOc1ccc(cc1Br)/C=c/2\c(=O)n3c(=NC4=C([C@H]3c5ccc(cc5Cl)Cl)CCc6c4cccc6)s2
ZINC02064387	5.05	-1.31	-39.14	2	8	0	478.555	7	Cc1ccc(cc1NC(=S)NC(=O)c2ccc(c(c2)[N+](=O)[O-])OC)c3nc4ccccc4s3
ZINC02064386	4.65	-1.8	-39.86	2	8	0	464.528	7	COc1ccc(cc1[N+](=O)[O-])C(=O)NC(=S)Nc2cccc(c2)c3nc4ccccc4s3
ZINC08441896	3.8	9.76	-17.42	1	9	0	475.549	8	[H]/N=C/1\[C@@H](C2=CCN(C[C@@H]2[C@H](C1(C#N)C#N)c3ccc(c(c3)OCC)OCCC)C(=O)OCC)C#N
ZINC08441896	3.8	2.21	-50.43	2	9	1	476.557	8	CCCOc1ccc(cc1OCC)[C@@H]2[C@H]3CN(CC=C3[C@H](C(=[NH2+])C2(C#N)C#N)C#N)C(=O)OCC
ZINC08441895	9.2	0.04	-9.85	0	4	0	612.376	4	CCOc1ccc(cc1Br)/C=c/2\c(=O)n3c(=NC4=C([C@@H]3c5ccc(cc5Cl)Cl)CCc6c4cccc6)s2
ZINC08441894	3.49	-2.21	-35.34	2	10	0	463.475	7	Cc1c(cccc1NC(=S)NC(=O)c2ccc(c(c2)[N+](=O)[O-])OC)c3nc4c(o3)cccn4
ZINC08441893	3.51	-2.25	-45.39	2	10	0	463.475	7	Cc1ccc(cc1NC(=S)NC(=O)c2ccc(c(c2)[N+](=O)[O-])OC)c3nc4c(o3)cccn4
ZINC02064384	4.46	-1.79	-39.23	2	9	0	462.487	7	Cc1ccc(cc1)c2nc3cc(ccc3o2)NC(=S)NC(=O)c4ccc(c(c4)[N+](=O)[O-])OC
ZINC08441892	4.14	8.9	-10.18	2	7	0	445.567	8	CCCN1CC=C2[C@H](C1)[C@H](C(C(=C2C#N)N)(C#N)C#N)c3ccc(c(c3)OCC)OCCC
ZINC08441892	4.23	2.92	-119.63	3	7	2	447.583	8	CCC[NH+]1CC=C2[C@H](C1)[C@H](C(C(=[NH2+])C2C#N)(C#N)C#N)c3ccc(c(c3)OCC)OCCC
ZINC02064383	4.41	-2.09	-39.89	2	9	0	462.487	7	Cc1ccccc1c2nc3cc(ccc3o2)NC(=S)NC(=O)c4ccc(c(c4)[N+](=O)[O-])OC
ZINC08441891	9.02	-1.46	-10.4	1	4	0	574.316	2	c1ccc2c(c1)CCC3=C2N=c4n(c(=O)/c(=C\c5cc(cc(c5O)Cl)Cl)/s4)[C@H]3c6ccc(cc6Cl)Cl
ZINC02064382	4.18	-1.37	-31.61	2	9	0	466.45	7	COc1ccc(cc1[N+](=O)[O-])C(=O)NC(=S)Nc2ccc3c(c2)nc(o3)c4ccc(cc4)F
ZINC08441890	5.93	3.61	-11.93	1	6	0	458.606	7	[H]/N=C\1/[C@H](C2=CC[C@@H](C[C@@H]2[C@H](C1(C#N)C#N)c3ccc(c(c3)OCC)OCCC)C(C)(C)C)C#N
ZINC08441890	5.93	3.43	-42.34	2	6	1	459.614	7	CCCOc1ccc(cc1OCC)[C@@H]2[C@H]3C[C@H](CC=C3[C@@H](C(=[NH2+])C2(C#N)C#N)C#N)C(C)(C)C
ZINC02064381	3.69	10.98	-27.19	2	8	0	414.468	7	COc1ccc(cc1[N+](=O)[O-])C(=O)NC(=S)Nc2nc(cs2)c3ccccc3
ZINC02064380	4.5	11.6	-26.68	2	8	0	493.364	7	COc1ccc(cc1[N+](=O)[O-])C(=O)NC(=S)Nc2nc(cs2)c3ccc(cc3)Br
ZINC08441889	9.02	-1.62	-9.87	1	4	0	574.316	2	c1ccc2c(c1)CCC3=C2N=c4n(c(=O)/c(=C\c5cc(cc(c5O)Cl)Cl)/s4)[C@@H]3c6ccc(cc6Cl)Cl

Supplementary Table S2:
Detailed table of XP Docking using Schrodinger's Glide module

ZINC_ID	xLogP	ApolarDesolvation	PolarDesolvation	H Donors	H Acceptors	Charge	MolecularWeight	RotatableBonds	SMILES
ZINC01876223	3.43	0.37	-38.72	2	11	0	492.517	8	Cc1cc2c(cc1NC(=S)NC(=O)c3ccc(c(c3)[N+](=O)[O-])OC)nn(n2)c4ccc(cc4)OC
ZINC01860012	3.82	0.67	-38.74	2	10	0	476.518	7	Cc1ccc(cc1)n2nc3cc(c(cc3n2)NC(=S)NC(=O)c4ccc(c(c4)[N+](=O)[O-])OC)C
ZINC01824016	3.43	-0.13	-38.88	2	11	0	492.517	9	CCOc1ccc(cc1)n2nc3ccc(cc3n2)NC(=S)NC(=O)c4ccc(c(c4)[N+](=O)[O-])OC
ZINC08441888	8.06	-1.15	-11.76	0	5	0	547.724	5	CCOc1ccc(cc1)[C@H]2C3=C(c4ccccc4CC3)N=c5n2c(=O)/c(=C\c6ccc(cc6C)N7CCCC7)/s5
ZINC02064379	3.44	0.16	-39.58	2	10	0	462.491	7	Cc1ccc(cc1)n2nc3ccc(cc3n2)NC(=S)NC(=O)c4ccc(c(c4)[N+](=O)[O-])OC
ZINC13161518	5.93	11.39	-18.6	1	6	0	458.606	7	[H]/N=C/1\[C@H](C2=CC[C@H](C[C@@H]2[C@H](C1(C#N)C#N)c3ccc(c(c3)OCC)OCCC)C(C)(C)C)C#N
ZINC02064378	2.99	-0.17	-39.95	2	10	0	448.464	7	COc1ccc(cc1[N+](=O)[O-])C(=O)NC(=S)Nc2ccc3c(c2)nn(n3)c4ccccc4
ZINC00881982	3.04	-0.95	-32.57	2	8	0	411.237	6	COc1ccc(cc1[N+](=O)[O-])C(=O)NC(=S)Nc2ccc(cn2)Br
ZINC08441886	8.06	-1.16	-11.96	0	5	0	547.724	5	CCOc1ccc(cc1)[C@@H]2C3=C(c4ccccc4CC3)N=c5n2c(=O)/c(=C\c6ccc(cc6C)N7CCCC7)/s5
ZINC08441885	5.43	3.53	-11.65	1	6	0	444.579	6	[H]/N=C\1/[C@@H](C2=CC[C@@H](C[C@@H]2[C@H](C1(C#N)C#N)c3ccc(c(c3)OCC)OCC)C(C)(C)C)C#N
ZINC08441885	5.43	3.34	-42.75	2	6	1	445.587	6	CCOc1ccc(cc1OCC)[C@@H]2[C@H]3C[C@H](CC=C3[C@H](C(=[NH2+])C2(C#N)C#N)C#N)C(C)(C)C
ZINC00881954	4.31	-1.89	-24.98	3	6	0	453.951	7	Cc1ccccc1C(=O)Nc2cccc(c2)NC(=S)NC(=O)c3ccc(c(c3)Cl)OC
ZINC08441884	8.21	0.02	-9.2	0	4	0	590.486	4	CCOc1ccc(cc1)[C@H]2C3=C(c4ccccc4CC3)N=c5n2c(=O)/c(=C\c6ccccc6I)/s5
ZINC00881949	4.75	-3.18	-28.71	2	6	0	437.908	6	COc1ccc(cc1Cl)C(=O)NC(=S)Nc2ccc(cc2)c3nc4ccccc4o3
ZINC08441883	5.43	3.84	-13.15	1	6	0	444.579	6	[H]/N=C/1\[C@H](C2=CC[C@H](C[C@@H]2[C@H](C1(C#N)C#N)c3ccc(c(c3)OCC)OCC)C(C)(C)C)C#N
ZINC08441883	5.43	3.67	-47	2	6	1	445.587	6	CCOc1ccc(cc1OCC)[C@@H]2[C@H]3C[C@@H](CC=C3[C@@H](C(=[NH2+])C2(C#N)C#N)C#N)C(C)(C)C
ZINC08441882	8.21	0	-9.55	0	4	0	590.486	4	CCOc1ccc(cc1)[C@@H]2C3=C(c4ccccc4CC3)N=c5n2c(=O)/c(=C\c6ccccc6I)/s5
ZINC00881937	4.73	-3.18	-25.42	2	6	0	437.908	6	COc1ccc(cc1Cl)C(=O)NC(=S)Nc2cccc(c2)c3nc4ccccc4o3
ZINC00971829	4.23	-3.33	-37.12	2	7	0	452.923	6	Cc1ccc(cc1NC(=S)NC(=O)c2ccc(c(c2)Cl)OC)c3nc4c(o3)cccn4
ZINC02064376	4.22	-0.82	-23.88	2	6	0	451.342	9	CCCOC(=O)c1ccc(cc1)NC(=S)NC(=O)c2ccc(c(c2)Br)OC
ZINC00881919	3.72	-0.89	-24.14	2	6	0	437.315	8	CCOC(=O)c1ccc(cc1)NC(=S)NC(=O)c2ccc(c(c2)Br)OC
ZINC00881917	3.35	-0.86	-24.3	2	6	0	423.288	7	COc1ccc(cc1Br)C(=O)NC(=S)Nc2ccc(cc2)C(=O)OC
ZINC01126045	4.59	5.81	-8.44	3	6	0	446.816	5	c1ccc(c(c1)COc2ccccc2[C@H]3c4c([nH]nc4OC(=C3C#N)N)C(F)(F)F)Cl
ZINC08441880	4.81	-2.62	-6.03	2	4	0	458.164	4	[H]/N=C\1/NC(=O)/C(=C\c2ccc(c(c2)Br)OCc3ccc(cc3Cl)Cl)/S1
ZINC00881904	4.03	-1.47	-22.22	2	5	0	436.375	8	CCN(CC)c1ccc(cc1)NC(=S)NC(=O)c2ccc(c(c2)Br)OC
ZINC00881890	2.82	-1.66	-24.4	2	6	0	425.304	7	COc1ccc(cc1OC)NC(=S)NC(=O)c2ccc(c(c2)Br)OC
ZINC01126049	4.59	5.81	-8.41	3	6	0	446.816	5	c1ccc(c(c1)COc2ccccc2[C@@H]3c4c([nH]nc4OC(=C3C#N)N)C(F)(F)F)Cl
ZINC00881866	3.21	-3.02	-27.58	3	6	0	436.331	7	CCC(=O)Nc1cccc(c1)NC(=S)NC(=O)c2ccc(c(c2)Br)OC
ZINC02890864	2.83	3.54	-21.39	2	7	0	430.885	9	COc1ccc(cc1OC)CCNC(=O)CNS(=O)(=O)c2ccc(c(c2)Cl)F
ZINC00881863	4.04	-2.75	-25.76	3	6	0	484.375	7	COc1ccc(cc1Br)C(=O)NC(=S)Nc2cccc(c2)NC(=O)c3ccccc3
ZINC00631391	3.26	13.23	-21.46	0	7	0	461.522	4	c1ccc2c(c1)ccc3n2c(nn3)SCC(=O)N4CCC(CC4)c5c6cc(ccc6on5)F
ZINC09178123	2.92	5.36	-18.92	3	9	0	453.543	8	CCN(CC)C(=O)COc1ccc(cc1OC)[C@H]2c3c([nH]nc3OC(=C2C#N)N)C(C)(C)C
ZINC00881856	3.99	-4.45	-30.37	2	7	0	483.347	6	COc1ccc(cc1Br)C(=O)NC(=S)Nc2ccc(cc2)c3nc4c(o3)cccn4
ZINC00881853	5.24	-3.16	-24.46	2	6	0	496.386	6	Cc1c(cccc1NC(=S)NC(=O)c2ccc(c(c2)Br)OC)c3nc4ccccc4o3
ZINC00881851	4.86	-3.76	-25.41	2	6	0	482.359	6	COc1ccc(cc1Br)C(=O)NC(=S)Nc2cccc(c2)c3nc4ccccc4o3
ZINC09178122	2.92	5.34	-18.86	3	9	0	453.543	8	CCN(CC)C(=O)COc1ccc(cc1OC)[C@@H]2c3c([nH]nc3OC(=C2C#N)N)C(C)(C)C
ZINC01873774	5.22	3.49	-51.64	2	7	1	440.516	10	CCCOC(=O)c1ccc(cc1)Oc2c(oc3c(c2=O)ccc(c3C[NH+](CC)CC)O)C
ZINC00881847	4.34	-3.84	-27.07	2	7	0	497.374	6	Cc1c(cccc1NC(=S)NC(=O)c2ccc(c(c2)Br)OC)c3nc4c(o3)cccn4
ZINC00881842	4.36	-3.92	-37.1	2	7	0	497.374	6	Cc1ccc(cc1NC(=S)NC(=O)c2ccc(c(c2)Br)OC)c3nc4c(o3)cccn4
ZINC00881833	3.85	-2.06	-24.44	2	5	0	456.365	7	COc1ccc(cc1Br)C(=O)NC(=S)Nc2ccc(cc2)Cc3ccncc3
ZINC08441879	3.65	7.9	-17.86	1	7	0	431.54	6	[H]/N=C/1\[C@H](C2=CCN(C[C@@H]2[C@H](C1(C#N)C#N)c3ccc(c(c3)OC)OCCC)C(C)C)C#N
ZINC08441879	3.65	2.87	-118.27	3	7	2	433.556	6	CCCOc1ccc(cc1OC)[C@@H]2[C@H]3C[NH+](CC=C3[C@@H](C(=[NH2+])C2(C#N)C#N)C#N)C(C)C
ZINC00881828	4.54	-1.52	-19.43	2	5	0	448.367	6	COc1ccc(cc1Br)C(=O)NC(=S)Nc2nc(cs2)c3ccccc3
ZINC00881812	3.84	-1.58	-23.31	2	8	0	481.505	8	COc1cc(cc(c1OC)OC)C(=O)NC(=S)Nc2ccc3c(c2)nc(o3)c4cccc(c4)F
ZINC00881808	4.33	-2.43	-24.68	2	8	0	497.96	8	COc1cc(cc(c1OC)OC)C(=O)NC(=S)Nc2ccc3c(c2)nc(o3)c4ccccc4Cl
ZINC08441878	3.65	7.37	-13.08	1	7	0	431.54	6	[H]/N=C/1\[C@H](C2=CCN(C[C@H]2[C@H](C1(C#N)C#N)c3ccc(c(c3)OC)OCCC)C(C)C)C#N
ZINC08441878	3.65	3.33	-111.74	3	7	2	433.556	6	CCCOc1ccc(cc1OC)[C@@H]2[C@@H]3C[NH+](CC=C3[C@@H](C(=[NH2+])C2(C#N)C#N)C#N)C(C)C
ZINC00881806	3.7	-2.33	-23.5	2	8	0	463.515	8	COc1cc(cc(c1OC)OC)C(=O)NC(=S)Nc2ccc3c(c2)nc(o3)c4ccccc4
ZINC00881739	2.88	-1.3	-24.82	3	8	0	465.531	9	COc1cc(cc(c1OC)OC)C(=O)NC(=S)Nc2cccc(c2)NC(=O)c3ccccc3
ZINC08441877	2.33	1.97	-18.48	1	8	0	431.496	5	[H]/N=C\1/[C@@H](C2=CCN(C[C@@H]2[C@H](C1(C#N)C#N)c3ccc(c(c3)OC)OCCC)C(=O)C)C#N
ZINC08441877	2.33	1.79	-52.57	2	8	1	432.504	5	CCCOc1ccc(cc1OC)[C@@H]2[C@H]3CN(CC=C3[C@H](C(=[NH2+])C2(C#N)C#N)C#N)C(=O)C
ZINC00881738	3.43	5.16	-27.51	3	9	0	479.514	8	COc1cc(cc(c1OC)OC)C(=O)NC(=S)Nc2ccc(c(c2)O)c3nc4ccccc4o3
ZINC08441876	4.99	3.48	-58.16	2	7	1	460.506	8	Cc1c(c(=O)c2ccc(c(c2o1)C[NH+](C)Cc3ccccc3)O)Oc4ccc(cc4)C(=O)OC
ZINC00881734	4.53	-1.68	-26.37	2	8	0	491.569	8	Cc1cc(c2c(c1)nc(o2)c3ccc(cc3)NC(=S)NC(=O)c4cc(c(c(c4)OC)OC)OC)C
ZINC00881732	4.15	-2	-26.19	2	8	0	477.542	8	Cc1ccc2c(c1)oc(n2)c3ccc(cc3)NC(=S)NC(=O)c4cc(c(c(c4)OC)OC)OC
ZINC08441875	2.33	7.67	-17.95	1	8	0	431.496	5	[H]/N=C/1\[C@H](C2=CCN(C[C@H]2[C@H](C1(C#N)C#N)c3ccc(c(c3)OC)OCCC)C(=O)C)C#N
ZINC08441875	2.33	1.85	-47.19	2	8	1	432.504	5	CCCOc1ccc(cc1OC)[C@@H]2[C@@H]3CN(CC=C3[C@@H](C(=[NH2+])C2(C#N)C#N)C#N)C(=O)C
ZINC00881730	4.38	-2.47	-24.37	2	8	0	497.96	8	COc1cc(cc(c1OC)OC)C(=O)NC(=S)Nc2ccc(cc2)c3nc4cc(ccc4o3)Cl
ZINC00631361	4.18	3.07	-10.46	0	7	0	449.381	5	COc1ccc(cc1)c2c(=O)c3ccc(cc3oc2C(F)(F)F)OC(=O)N4CCOCC4
ZINC00881728	4.37	-2.03	-25.52	2	7	0	479.583	8	COc1cc(cc(c1OC)OC)C(=O)NC(=S)Nc2ccc(cc2)c3nc4ccccc4s3
ZINC00881726	3.71	-2.2	-27.48	2	9	0	493.541	9	COc1ccc(cc1NC(=S)NC(=O)c2cc(c(c(c2)OC)OC)OC)c3nc4ccccc4o3

Supplementary Table S2:
Detailed table of XP Docking using Schrodinger's Glide module

ZINC_ID	xLogP	ApolarDesolvation	PolarDesolvation	H Donors	H Acceptors	Charge	MolecularWeight	RotatableBonds	SMILES
ZINC08441874	4.49	9.98	-16.37	1	9	0	489.576	9	[H]/N=C/1\[C@@H](C2=CCN(C[C@@H]2[C@@H](C1(C#N)C#N)c3ccc(c(c3)OC)OCCCCC)C(=O)OCC)C#N
ZINC08441874	4.49	2.36	-47.04	2	9	1	490.584	9	CCCCCOc1ccc(cc1OC)[C@H]2[C@H]3CN(CC=C3[C@H](C(=[NH2+])C2(C#N)C#N)C#N)C(=O)OCC
ZINC00881724	4.1	-1.82	-29.5	2	8	0	477.542	8	Cc1ccc(cc1NC(=S)NC(=O)c2cc(c(c(c2)OC)OC)OC)c3nc4ccccc4o3
ZINC00881722	4.08	-1.79	-24.54	2	8	0	477.542	8	Cc1c(cccc1NC(=S)NC(=O)c2cc(c(c(c2)OC)OC)OC)c3nc4ccccc4o3
ZINC08441873	4.28	0.12	-18.33	1	9	0	498.634	12	CCOC(=O)c1ccc(cc1)NC(=O)CSc2nnc(n2CC=C)CSc3nc(cc(n3)C)C
ZINC00881721	4.5	-1.68	-31.43	2	8	0	491.569	8	Cc1cc(c2c(c1)nc(o2)c3cccc(c3)NC(=S)NC(=O)c4cc(c(c(c4)OC)OC)OC)C
ZINC00881706	4.74	-1.53	-29.7	2	7	0	493.61	8	Cc1ccc(cc1NC(=S)NC(=O)c2cc(c(c(c2)OC)OC)OC)c3nc4ccccc4s3
ZINC00387261	2.97	9.67	-16.39	1	6	0	332.363	2	Cc1cccn2c1nc3c(c2=O)cc(n3C)C(=O)Nc4ccccc4
ZINC08441872	4.49	10.4	-17.34	1	9	0	489.576	9	[H]/N=C/1\[C@@H](C2=CCN(C[C@@H]2[C@H](C1(C#N)C#N)c3ccc(c(c3)OC)OCCCCC)C(=O)OCC)C#N
ZINC08441872	4.49	2.44	-50.24	2	9	1	490.584	9	CCCCCOc1ccc(cc1OC)[C@@H]2[C@H]3CN(CC=C3[C@H](C(=[NH2+])C2(C#N)C#N)C#N)C(=O)OCC
ZINC00881705	4.34	-2.03	-23.12	2	7	0	479.583	8	COc1cc(cc(c1OC)OC)C(=O)NC(=S)Nc2cccc(c2)c3nc4ccccc4s3
ZINC00631218	3.59	11.4	-15.16	0	6	0	360.417	3	CCN(c1ccccc1)C(=O)c2cc3c(n2C)nc4c(cccn4c3=O)C
ZINC00881703	3.18	-2.79	-29.4	2	9	0	478.53	8	Cc1c(cccc1NC(=S)NC(=O)c2cc(c(c(c2)OC)OC)OC)c3nc4c(o3)cccn4
ZINC00881701	3.2	-2.48	-35.42	2	9	0	478.53	8	Cc1ccc(cc1NC(=S)NC(=O)c2cc(c(c(c2)OC)OC)OC)c3nc4c(o3)cccn4
ZINC08441871	4.49	2.61	-17	1	9	0	489.576	9	[H]/N=C/1\[C@@H](C2=CCN(C[C@H]2[C@@H](C1(C#N)C#N)c3ccc(c(c3)OC)OCCCCC)C(=O)OCC)C#N
ZINC08441871	4.49	2.44	-51.59	2	9	1	490.584	9	CCCCCOc1ccc(cc1OC)[C@H]2[C@@H]3CN(CC=C3[C@H](C(=[NH2+])C2(C#N)C#N)C#N)C(=O)OCC
ZINC00881699	2.8	-3.01	-26.99	2	9	0	464.503	8	COc1cc(cc(c1OC)OC)C(=O)NC(=S)Nc2cccc(c2)c3nc4c(o3)cccn4
ZINC00920168	3.63	-2.26	-22.63	2	7	0	463.987	8	COc1cc(cc(c1OC)OC)C(=O)NC(=S)Nc2ccc(c(c2)Cl)N3CCCCC3
ZINC08441870	3.75	-5.91	-19.39	2	7	0	453.545	6	Cc1nc2cc(ccc2s1)NC(=O)c3ccc(cc3)NS(=O)(=O)c4cccc(c4)OC
ZINC00881692	2.57	-3.67	-20.8	2	8	0	465.959	8	COc1cc(cc(c1OC)OC)C(=O)NC(=S)Nc2ccc(c(c2)Cl)N3CCOCC3
ZINC13161517	4.49	9.99	-15.97	1	9	0	489.576	9	[H]/N=C\1/[C@H](C2=CCN(C[C@H]2[C@H](C1(C#N)C#N)c3ccc(c(c3)OC)OCCCCC)C(=O)OCC)C#N
ZINC00881684	4.1	-2.31	-23.72	2	8	0	477.542	8	Cc1ccccc1c2nc3cc(ccc3o2)NC(=S)NC(=O)c4cc(c(c(c4)OC)OC)OC
ZINC08441867	1.17	3.31	-19.95	3	10	0	441.488	9	CCN(CC)C(=O)COc1ccc(cc1OC)[C@H]2c3c([nH]nc3OC(=C2C#N)N)COC
ZINC00881683	4.38	-2.48	-22.25	2	8	0	497.96	8	COc1cc(cc(c1OC)OC)C(=O)NC(=S)Nc2ccc3c(c2)nc(o3)c4ccc(cc4)Cl
ZINC00920166	3.51	0.45	-29.1	2	9	0	491.573	8	Cc1ccc(cc1)n2nc3cc(c(cc3n2)NC(=S)NC(=O)c4cc(c(c(c4)OC)OC)OC)C
ZINC08441866	4.38	-5.05	-16.79	2	6	0	444.94	8	CCOc1ccc(cc1)NC(=O)c2ccc(cc2)CNS(=O)(=O)c3cccc(c3)Cl
ZINC00881677	3.06	0.13	-29.44	2	9	0	477.546	8	Cc1cc2c(cc1NC(=S)NC(=O)c3cc(c(c(c3)OC)OC)OC)nn(n2)c4ccccc4
ZINC08441865	1.17	3.31	-19.99	3	10	0	441.488	9	CCN(CC)C(=O)COc1ccc(cc1OC)[C@@H]2c3c([nH]nc3OC(=C2C#N)N)COC
ZINC00881672	3.13	-0.07	-29.77	2	9	0	477.546	8	Cc1ccc(cc1)n2nc3ccc(cc3n2)NC(=S)NC(=O)c4cc(c(c(c4)OC)OC)OC
ZINC06143751	3.17	9.86	-17.8	2	9	0	461.532	6	c1ccc(cc1)c2nc(sn2)NC(=O)CSc3nc4c(cn[nH]4)c(=O)n3c5ccccc5
ZINC00881660	2.58	-0.18	-31.01	2	9	0	425.85	8	COc1cc(cc(c1OC)OC)C(=O)NC(=S)Nc2ccc(c(c2)[N+](=O)[O-])Cl
ZINC08441864	3.65	8.84	-55.82	2	7	1	432.548	7	[H]/N=C/1\C(C2=CC[NH+](C[C@@H]2[C@H](C1(C#N)C#N)c3ccc(cc3OCC)OCC)CCC)C#N
ZINC08441864	3.65	8.95	-109.68	3	7	2	433.556	7	CCC[NH+]1CC=C2[C@H](C1)[C@H](C(C(=[NH2+])C2C#N)(C#N)C#N)c3ccc(cc3OCC)OCC
ZINC00881656	4.37	-2.9	-23.68	2	7	0	467.934	7	COc1ccc(cc1OC)C(=O)NC(=S)Nc2ccc3c(c2)nc(o3)c4cccc(c4)Cl
ZINC00881655	3.85	-2.01	-24.15	2	7	0	451.479	7	COc1ccc(cc1OC)C(=O)NC(=S)Nc2ccc3c(c2)nc(o3)c4cccc(c4)F
ZINC00881652	4.34	-2.87	-30.75	2	7	0	467.934	7	COc1ccc(cc1OC)C(=O)NC(=S)Nc2ccc3c(c2)nc(o3)c4ccccc4Cl
ZINC02889263	4.12	-4.56	-15.18	1	6	0	425.564	5	c1ccc(cc1)c2nc(ns2)NC(=O)CSc3c4c5c(sc4ncn3)CCC5
ZINC00881648	3.71	-2.76	-24.39	2	7	0	433.489	7	COc1ccc(cc1OC)C(=O)NC(=S)Nc2ccc3c(c2)nc(o3)c4ccccc4
ZINC08441863	1.89	4.37	-12.3	2	9	0	474.565	6	CN1CC=C2[C@H](C1)[C@H](C(C(=C2C#N)N)(C#N)C#N)c3ccc(c(c3)OC)OCCN4CCOCC4
ZINC08441863	1.97	1.57	-174.92	4	9	3	477.589	6	C[NH+]1CC=C2[C@H](C1)[C@H](C(C(=[NH2+])C2C#N)(C#N)C#N)c3ccc(c(c3)OC)OCC[NH+]4CCOCC4
ZINC08441863	1.89	6.75	-43.51	3	9	1	475.573	6	CN1CC=C2[C@H](C1)[C@H](C(C(=C2C#N)N)(C#N)C#N)c3ccc(c(c3)OC)OCC[NH+]4CCOCC4
ZINC00881612	2.65	-1.09	-20.08	2	5	0	423.332	6	Cc1cc(cc(c1NC(=S)NC(=O)c2ccc(c(c2)OC)OC)C)Br
ZINC00881603	3.34	-1.41	-30.93	3	7	0	449.532	8	Cc1cccc(c1)C(=O)Nc2ccc(cc2)NC(=S)NC(=O)c3ccc(c(c3)OC)OC
ZINC00881599	3.3	-1.48	-25.48	3	7	0	449.532	8	Cc1ccccc1C(=O)Nc2cccc(c2)NC(=S)NC(=O)c3ccc(c(c3)OC)OC
ZINC08441862	3.9	9.31	-17.37	1	7	0	465.557	6	[H]/N=C\1/[C@H](C2=CCN(C[C@@H]2[C@H](C1(C#N)C#N)c3ccc(c(c3)OC)OC)CCc4ccccc4)C#N
ZINC08441862	3.9	2.83	-131.13	3	7	2	467.573	6	COc1ccc(cc1OC)[C@@H]2[C@H]3C[NH+](CC=C3[C@@H](C(=[NH2+])C2(C#N)C#N)C#N)CCc4ccccc4
ZINC01805638	4.23	0.41	-10.66	0	5	0	437.59	8	Cc1cc(c(n1CCOC)C)C(=O)CSc2c3c(csc3ncn2)c4ccccc4
ZINC00881590	4.54	-2.11	-28.47	2	7	0	461.543	7	Cc1cc(c2c(c1)nc(o2)c3ccc(cc3)NC(=S)NC(=O)c4ccc(c(c4)OC)OC)C
ZINC00630503	4.78	10.95	-14.88	1	6	0	455.539	8	COc1ccc(cc1)c2cc(nc(c2C#N)SCC(=O)NCc3ccco3)c4ccccc4
ZINC00630492	4.49	10.1	-14.69	1	6	0	459.571	8	COc1ccc(cc1)c2cc(nc(c2C#N)SCC(=O)NC[C@H]3CCCO3)c4ccccc4
ZINC00881588	4.54	-2.13	-27.26	2	7	0	461.543	7	Cc1cc2c(cc1C)oc(n2)c3ccc(cc3)NC(=S)NC(=O)c4ccc(c(c4)OC)OC
ZINC00630498	4.49	10.07	-14.97	1	6	0	459.571	8	COc1ccc(cc1)c2cc(nc(c2C#N)SCC(=O)NC[C@@H]3CCCO3)c4ccccc4
ZINC00881586	4.63	-2.28	-26.72	2	7	0	461.543	8	CCc1ccc2c(c1)nc(o2)c3ccc(cc3)NC(=S)NC(=O)c4ccc(c(c4)OC)OC
ZINC00630488	4.89	8.31	-11.08	3	6	0	459.571	6	COc1ccc(cc1)c2cc(nc3c2c(c(s3)C(=O)NC[C@@H]4CCCO4)N)c5ccccc5
ZINC00881582	4.16	-2.43	-27.07	2	7	0	447.516	7	Cc1ccc2c(c1)nc(o2)c3ccc(cc3)NC(=S)NC(=O)c4ccc(c(c4)OC)OC
ZINC01805608	2.5	-2.39	-54.7	2	7	1	439.561	8	c1ccc(cc1)n2c(=O)c3ccccc3nc2SCC(=O)NCCC[NH+]4CCOCC4
ZINC00881578	4.81	-2.14	-27.59	2	6	0	463.584	7	Cc1ccc2c(c1)sc(n2)c3ccc(cc3)NC(=S)NC(=O)c4ccc(c(c4)OC)OC
ZINC08441861	6.38	10.21	-52.98	0	6	-1	465.595	10	Cc1c(c(nc(n1)SCc2cc(ccc2OC)C(=O)C)[O-])Cc3ccc(cc3)OCC(C)C
ZINC08441861	5.92	11.07	-20.94	1	6	0	466.603	10	Cc1c(c(=O)[nH]c(n1)SCc2cc(ccc2OC)C(=O)C)Cc3ccc(cc3)OCC(C)C
ZINC00881571	2.84	-3.44	-32.96	2	8	0	434.477	7	COc1ccc(cc1OC)C(=O)NC(=S)Nc2ccc(cc2)c3nc4c(o3)cccn4
ZINC00630394	6.2	16.81	-16.27	0	5	0	481.408	5	Cc1cc(c(n1c2cc(ccc2Cl)Cl)C)C(=O)CSc3nnc4n3c5ccccc5cc4

Supplementary Table S2:
Detailed table of XP Docking using Schrodinger's Glide module

ZINC_ID	xLogP	ApolarDesolvation	PolarDesolvation	H Donors	H Acceptors	Charge	MolecularWeight	RotatableBonds	SMILES
ZINC00630390	5.17	15.79	-17.59	0	5	0	448.498	5	Cc1cc(c(n1c2cc(ccc2F)F)C)C(=O)CSc3nnc4n3c5ccccc5cc4
ZINC00881569	4.25	6.97	-34.57	3	8	0	477.542	7	Cc1cc2c(cc1C)oc(n2)c3cc(ccc3O)NC(=S)NC(=O)c4ccc(c(c4)OC)OC
ZINC00630388	4.65	15.65	-16.08	0	5	0	430.508	5	Cc1cc(c(n1c2ccc(cc2)F)C)C(=O)CSc3nnc4n3c5ccccc5cc4
ZINC00630364	5.04	11.8	-15.36	0	6	0	473.598	6	Cc1ccc(cc1C)c2cc(c(c(n2)SCC(=O)N3CCOCC3)C#N)c4ccc(cc4)OC
ZINC02117696	7.45	1.19	-8.96	1	5	0	488.653	7	CCOC(=O)[C@H](c1ccccc1)Sc2[nH]c(=O)c3c(n2)-c4ccccc4C[C@]3(C)C5CCCCC5
ZINC00881566	3.87	5.94	-33	3	8	0	463.515	7	Cc1ccc2c(c1)nc(o2)c3cc(ccc3O)NC(=S)NC(=O)c4ccc(c(c4)OC)OC
ZINC00881562	4.12	-2.25	-30.72	2	7	0	447.516	7	Cc1ccc(cc1NC(=S)NC(=O)c2ccc(c(c2)OC)OC)c3nc4ccccc4o3
ZINC00881560	4.09	-2.23	-25.4	2	7	0	447.516	7	Cc1c(cccc1NC(=S)NC(=O)c2ccc(c(c2)OC)OC)c3nc4ccccc4o3
ZINC00881558	4.89	-1.54	-24.49	2	7	0	475.57	7	Cc1cc(c2c(c1)nc(o2)c3cccc(c3C)NC(=S)NC(=O)c4ccc(c(c4)OC)OC)C
ZINC03646241	4.22	1.78	-16.95	0	6	0	436.533	5	CCc1c(c(nc2c1C(=O)CC(C2)(C)C)SCC(=O)c3ccc4c(c3)OCCO4)C#N
ZINC02117694	7.45	1.26	-9.71	1	5	0	488.653	7	CCOC(=O)[C@@H](c1ccccc1)Sc2[nH]c(=O)c3c(n2)-c4ccccc4C[C@]3(C)C5CCCCC5
ZINC00881554	5.01	-1.75	-30.09	2	7	0	475.57	8	CCc1ccc2c(c1)nc(o2)c3ccc(c(c3)NC(=S)NC(=O)c4ccc(c(c4)OC)OC)C
ZINC00881552	4.61	-2.28	-30.64	2	7	0	461.543	8	CCc1ccc2c(c1)nc(o2)c3cccc(c3)NC(=S)NC(=O)c4ccc(c(c4)OC)OC
ZINC02117701	7.45	1.08	-9.04	1	5	0	488.653	7	CCOC(=O)[C@H](c1ccccc1)Sc2[nH]c(=O)c3c(n2)-c4ccccc4C[C@@]3(C)C5CCCCC5
ZINC00630355	5.59	2.72	-15.31	0	4	0	426.541	5	CC1(Cc2c(c(c(c(n2)SCC(=O)c3ccccc3)C#N)c4ccccc4)C(=O)C1)C
ZINC00881550	3.71	-2.75	-25.34	2	7	0	433.489	7	COc1ccc(cc1OC)C(=O)NC(=S)Nc2cccc(c2)c3nc4ccccc4o3
ZINC00881548	4.77	-2.45	-25.53	2	7	0	481.961	7	Cc1ccc2c(c1)nc(o2)c3ccc(c(c3)NC(=S)NC(=O)c4ccc(c(c4)OC)OC)Cl
ZINC00630352	5.65	2.75	-16.62	0	5	0	456.567	6	CC1(Cc2c(c(c(c(n2)SCC(=O)c3ccccc3)C#N)c4ccc(cc4)OC)C(=O)C1)C
ZINC02117698	7.45	1.16	-9.91	1	5	0	488.653	7	CCOC(=O)[C@@H](c1ccccc1)Sc2[nH]c(=O)c3c(n2)-c4ccccc4C[C@@]3(C)C5CCCCC5
ZINC00630347	4.84	-1.2	-10.68	2	6	0	436.533	3	CCc1c2c(c(sc2nc3c1C(=O)CC(C3)(C)C)C(=O)c4ccc5c(c4)OCCO5)N
ZINC00630340	5.2	16.04	-17.06	0	5	0	462.575	6	Cc1cc(c(n1c2ccccc2F)C)C(=O)CSc3nnc4n3c(cs4)c5ccccc5
ZINC00630301	5.41	0.62	-19.82	1	6	0	499.636	8	CC1(Cc2c(c(c(c(n2)SCC(=O)NCCc3ccccc3)C#N)c4ccc(cc4)OC)C(=O)C1)C
ZINC00657651	5.87	1.33	-14.04	0	9	0	507.561	6	c1ccc(cc1)c2nccn2c3nc(nc(n3)n4ccnc4c5ccccc5)n6ccnc6c7ccccc7
ZINC00657651	5.87	2.08	-142.3	3	9	3	510.585	6	c1ccc(cc1)c2[nH+]ccn2c3nc(nc(n3)n4cc[nH+]c4c5ccccc5)n6cc[nH+]c6c7ccccc7
ZINC00657651	5.87	1.84	-82.34	2	9	2	509.577	6	c1ccc(cc1)c2[nH+]ccn2c3nc(nc(n3)n4ccnc4c5ccccc5)n6cc[nH+]c6c7ccccc7
ZINC00657651	5.87	1.59	-40.75	1	9	1	508.569	6	c1ccc(cc1)c2[nH+]ccn2c3nc(nc(n3)n4ccnc4c5ccccc5)n6ccnc6c7ccccc7
ZINC00630298	5.47	2.03	-19.6	1	6	0	489.572	6	CC1(Cc2c(c(c(c(n2)SCC(=O)Nc3ccc(cc3)F)C#N)c4ccc(cc4)OC)C(=O)C1)C
ZINC08441860	7.63	-0.21	-27.8	1	7	0	604.757	7	CC1(Cc2c(c(c(c(n2)SCC(=O)Nc3cccc(c3)c4nc5ccccc5s4)C#N)c6ccc(cc6)OC)C(=O)C1)C
ZINC00657136	5.54	15.7	-24.83	1	9	0	468.469	7	c1ccc(cc1)Cn\2c3ccccc3[nH]/c2=N\C(=O)c4ccc(o4)COc5ccccc5[N+](=O)[O-]
ZINC00630288	5.57	1.25	-14.9	1	6	0	485.609	7	CC1(Cc2c(c(c(c(n2)SCCC(=O)Nc3ccc(cc3)OC)C#N)c4ccccc4)C(=O)C1)C
ZINC00656852	5.15	0.04	-21.21	1	5	0	436.309	5	Cn1c2ccccc2nc1NC(=O)c3cccc(c3)COc4ccc(cc4)Br
ZINC00630284	6.12	3.39	-16.17	1	5	0	509.553	6	CC1(Cc2c(c(c(c(n2)SCC(=O)Nc3cccc(c3)C(F)(F)F)C#N)c4ccccc4)C(=O)C1)C
ZINC00630276	4.8	10.95	-12.6	1	5	0	425.529	6	CCc1c(c(nc2c1C(=O)CC(C2)(C)C)SCCC(=O)Nc3ccc(cc3)F)C#N
ZINC08441859	6.63	5.29	-8.59	2	0	0	565.535	6	CCOC(=O)C1[C@@H](C2=C(CC(CC2=O)(C)C)N=C1C)c3ccc(c(c3)COc4c(c(c(c(c4F)F)F)F)F)OC
ZINC08441859	6.63	5.21	-12.04	0	6	0	565.535	8	CCOC(=O)C1=C(N=C2CC(CC(=O)C2[C@H]1c3ccc(c(c3)COc4c(c(c(c(c4F)F)F)F)F)OC)(C)C)C
ZINC08441859	6.81	4.11	-9.11	0	6	0	565.535	8	CCOC(=O)C1[C@@H](C2C(=NC1=C)CC(CC2=O)(C)C)c3ccc(c(c3)COc4c(c(c(c(c4F)F)F)F)F)OC
ZINC08441857	6.63	5.48	-8.9	2	0	0	565.535	6	CCOC(=O)C1[C@H](C2=C(CC(CC2=O)(C)C)N=C1C)c3ccc(c(c3)COc4c(c(c(c(c4F)F)F)F)F)OC
ZINC08441857	6.63	5.12	-11.8	0	6	0	565.535	8	CCOC(=O)C1=C(N=C2CC(CC(=O)C2[C@@H]1c3ccc(c(c3)COc4c(c(c(c(c4F)F)F)F)F)OC)(C)C)C
ZINC08441857	6.81	4.2	-9.42	0	6	0	565.535	8	CCOC(=O)C1[C@H](C2C(=NC1=C)CC(CC2=O)(C)C)c3ccc(c(c3)COc4c(c(c(c(c4F)F)F)F)F)OC
ZINC08441856	6.27	-0.17	-21.45	2	1	0	567.686	8	Cc1ccc(nc1)NC(=O)C2[C@H](C3=C(CC(CC3=O)(C)C)N=C2C)c4ccc(c(c4)COc5ccc(cc5)OC)OC
ZINC08441856	6.45	-1.31	-21.19	1	8	0	567.686	8	Cc1ccc(nc1)NC(=O)C2[C@H](C3C(=NC2=C)CC(CC3=O)(C)C)c4ccc(c(c4)COc5ccc(cc5)OC)OC
ZINC08441856	6.27	-0.25	-23.84	1	8	0	567.686	8	Cc1ccc(nc1)NC(=O)C2=C(N=C3CC(CC(=O)C3[C@@H]2c4ccc(c(c4)COc5ccc(cc5)OC)OC)(C)C)C
ZINC08441855	3.96	-3.18	-11.28	0	2	0	437.285	6	c1cn(nc1NC(=O)c2c(c[nH]n2)I)C34CC5CC(C3)CC(C5)C4
ZINC00653533	5.53	-2.2	-11.29	1	2	0	442.357	5	CC(C)(C)c1cc(n[nH]1)CNC(=O)c2cccc(c2)COc3ccc(cc3)Br
ZINC08441853	6.81	-2.01	-15.82	2	2	0	552.059	7	CC1=C([C@H](n2c(=O)/c(=C\c3c[nH]nc3c4ccccc4)/sc2=N1)c5ccccc5Cl)C(=O)Nc6ccccc6
ZINC08441852	6.81	-2.36	-16.58	2	2	0	552.059	7	CC1=C([C@@H](n2c(=O)/c(=C\c3c[nH]nc3c4ccccc4)/sc2=N1)c5ccccc5Cl)C(=O)Nc6ccccc6
ZINC08441851	4.14	-3.56	-13.55	1	4	0	536.632	8	Cc1ccccc1NC(=O)[C@H]2[C@H]([C@@H]([C@@](CC2=O)(C)O)C(=O)Nc3ccccc3C)c4c[nH]nc4c5ccccc5
ZINC08441850	4.14	-2.55	-24.34	1	4	0	536.632	8	Cc1ccccc1NC(=O)[C@@H]2[C@H]([C@@H]([C@@](CC2=O)(C)O)C(=O)Nc3ccccc3C)c4c[nH]nc4c5ccccc5
ZINC08441849	4.14	-3.1	-14.53	1	4	0	536.632	8	Cc1ccccc1NC(=O)[C@H]2[C@@H]([C@@H]([C@@](CC2=O)(C)O)C(=O)Nc3ccccc3C)c4c[nH]nc4c5ccccc5
ZINC08441847	4.14	-2.7	-11.06	1	4	0	536.632	8	Cc1ccccc1NC(=O)[C@@H]2[C@@H]([C@@H]([C@@](CC2=O)(C)O)C(=O)Nc3ccccc3C)c4c[nH]nc4c5ccccc5
ZINC00652390	5.28	1.76	-12.64	1	2	0	463.403	5	CCOc1ccc2c(c1)nc([nH]2)SCC(=O)Nc3cc(cc(c3)C(F)(F)F)C(F)(F)F
ZINC08441846	3.4	0.94	-12.65	1	2	0	511.192	6	Cc1c(c(n(n1)Cc2c(c(c(c(c2F)F)F)F)F)C)NC(=O)c3c(c[nH]n3)I
ZINC08441844	5.03	12.98	-20.53	1	7	0	491.584	8	Cc1cc(c(cc1COc2c(cccc2OC)OC)[C@H]3C4=C(CCCC4=O)NC(=C3C(=O)OC)C)C
ZINC08441844	5.15	0.4	-16.22	0	7	0	491.584	8	Cc1cc(c(cc1COc2c(cccc2OC)OC)[C@H]3C4C(=O)CCC=C4N=C(C3C(=O)OC)C)C
ZINC08441843	5.03	12.99	-20.59	1	7	0	491.584	8	Cc1cc(c(cc1COc2c(cccc2OC)OC)[C@@H]3C4=C(CCCC4=O)NC(=C3C(=O)OC)C)C
ZINC08441843	5.15	0.61	-14.81	0	7	0	491.584	8	Cc1cc(c(cc1COc2c(cccc2OC)OC)[C@@H]3C4C(=O)CCC=C4N=C(C3C(=O)OC)C)C
ZINC08441842	6.08	13.44	-20.55	1	5	0	516.457	7	CCc1c(cc(s1)[C@H]2C3=C(CCCC3=O)NC(=C2C(=O)OC)C)COc4ccc(cc4)Br
ZINC08441842	6.2	-2.34	-9.87	0	5	0	516.457	7	CCc1c(cc(s1)[C@H]2C3C(=O)CCC=C3N=C(C2C(=O)OC)C)COc4ccc(cc4)Br
ZINC08441841	6.08	13.94	-27.61	1	5	0	516.457	7	CCc1c(cc(s1)[C@@H]2C3=C(CCCC3=O)NC(=C2C(=O)OC)C)COc4ccc(cc4)Br
ZINC08441841	6.2	-2.17	-12.75	0	5	0	516.457	7	CCc1c(cc(s1)[C@@H]2C3C(=O)CCC=C3N=C(C2C(=O)OC)C)COc4ccc(cc4)Br
ZINC08441840	7.11	15.72	-18.6	1	5	0	558.538	8	CCc1c(cc(s1)[C@H]2C3=C(CC(CC3=O)(C)C)NC(=C2C(=O)OCC)C)COc4ccc(cc4)Br

Supplementary Table S2:
Detailed table of XP Docking using Schrodinger's Glide module

ZINC_ID	xLogP	ApolarDesolvation	PolarDesolvation	H Donors	H Acceptors	Charge	MolecularWeight	RotatableBonds	SMILES
ZINC08441840	7.23	-1.25	-9.18	0	5	0	558.538	8	CCc1c(cc(s1)[C@H]2C3C(=O)CC(C=C3N=C(C2C(=O)OCC)C)(C)C)COc4ccc(cc4)Br
ZINC08441839	7.11	15.73	-18.66	1	5	0	558.538	8	CCc1c(cc(s1)[C@@H]2C3=C(CC(CC3=O)(C)C)NC(=C2C(=O)OCC)C)COc4ccc(cc4)Br
ZINC08441839	7.23	-1.38	-11.43	0	5	0	558.538	8	CCc1c(cc(s1)[C@@H]2C3C(=O)CC(C=C3N=C(C2C(=O)OCC)C)(C)C)COc4ccc(cc4)Br
ZINC08441838	6.74	14.81	-18.7	1	5	0	544.511	7	CCc1c(cc(s1)[C@H]2C3=C(CC(CC3=O)(C)C)NC(=C2C(=O)OC)C)COc4ccc(cc4)Br
ZINC08441838	6.85	-1.37	-9.68	0	5	0	544.511	7	CCc1c(cc(s1)[C@H]2C3C(=O)CC(C=C3N=C(C2C(=O)OC)C)(C)C)COc4ccc(cc4)Br
ZINC08441837	6.74	14.8	-18.69	1	5	0	544.511	7	CCc1c(cc(s1)[C@@H]2C3=C(CC(CC3=O)(C)C)NC(=C2C(=O)OC)C)COc4ccc(cc4)Br
ZINC08441837	6.85	-1.38	-9.85	0	5	0	544.511	7	CCc1c(cc(s1)[C@@H]2C3C(=O)CC(C=C3N=C(C2C(=O)OC)C)(C)C)COc4ccc(cc4)Br
ZINC08441836	6.86	-2.47	-13.35	2	2	0	552.059	7	CC1=C([C@H](n2c(=O)/c(=C\c3c[nH]nc3c4ccccc4)/sc2=N1)c5ccc(cc5)Cl)C(=O)Nc6ccccc6
ZINC08441835	6.86	-2.54	-13.45	2	2	0	552.059	7	CC1=C([C@@H](n2c(=O)/c(=C\c3c[nH]nc3c4ccccc4)/sc2=N1)c5ccc(cc5)Cl)C(=O)Nc6ccccc6
ZINC08441832	7.15	1.42	-9.64	2	0	0	552.509	5	CCOC(=O)C1[C@@H](C2=C(CC(CC2=O)(C)C)N=C1C)c3cc(c(cc3C)C)COc4ccccc4Br
ZINC08441832	7.33	0.23	-11.01	0	5	0	552.509	7	CCOC(=O)C1[C@@H](C2C(=NC1=C)CC(CC2=O)(C)C)c3cc(c(cc3C)C)COc4ccccc4Br
ZINC08441832	7.15	1.33	-12.55	0	5	0	552.509	7	CCOC(=O)C1=C(N=C2CC(CC(=O)C2[C@H]1c3cc(c(cc3C)C)COc4ccccc4Br)(C)C)C
ZINC08441831	7.15	1.38	-9.96	2	0	0	552.509	5	CCOC(=O)C1[C@H](C2=C(CC(CC2=O)(C)C)N=C1C)c3cc(c(cc3C)C)COc4ccccc4Br
ZINC08441831	7.33	-0.04	-11.74	0	5	0	552.509	7	CCOC(=O)C1[C@H](C2C(=NC1=C)CC(CC2=O)(C)C)c3cc(c(cc3C)C)COc4ccccc4Br
ZINC08441831	7.15	1.22	-13.32	0	5	0	552.509	7	CCOC(=O)C1=C(N=C2CC(CC(=O)C2[C@@H]1c3cc(c(cc3C)C)COc4ccccc4Br)(C)C)C
ZINC08441830	7.45	1.5	-7.52	2	0	0	524.126	4	CCOC(=O)C1[C@@H](C2=C(CC(CC2=O)(C)C)N=C1C)c3cc(c(cc3C)C)CSc4ccc(cc4)Cl
ZINC08441830	7.63	0.32	-8.34	0	4	0	524.126	7	CCOC(=O)C1[C@@H](C2C(=NC1=C)CC(CC2=O)(C)C)c3cc(c(cc3C)C)CSc4ccc(cc4)Cl
ZINC08441830	7.45	1.42	-10.02	0	4	0	524.126	7	CCOC(=O)C1=C(N=C2CC(CC(=O)C2[C@H]1c3cc(c(cc3C)C)CSc4ccc(cc4)Cl)(C)C)C
ZINC08441829	7.45	1.51	-7.72	2	0	0	524.126	4	CCOC(=O)C1[C@H](C2=C(CC(CC2=O)(C)C)N=C1C)c3cc(c(cc3C)C)CSc4ccc(cc4)Cl
ZINC08441829	7.45	1.35	-11.68	0	4	0	524.126	7	CCOC(=O)C1=C(N=C2CC(CC(=O)C2[C@@H]1c3cc(c(cc3C)C)CSc4ccc(cc4)Cl)(C)C)C
ZINC08441829	7.63	0.09	-9.56	0	4	0	524.126	7	CCOC(=O)C1[C@H](C2C(=NC1=C)CC(CC2=O)(C)C)c3cc(c(cc3C)C)CSc4ccc(cc4)Cl
ZINC08441828	6.41	14.93	-17.7	1	4	0	502.101	6	Cc1c(cc(s1)[C@H]2C3=C(CC(CC3=O)(C)C)NC(=C2C(=O)OC)C)CSc4ccc(cc4)Cl
ZINC08441828	6.53	-1.29	-8.61	0	4	0	502.101	6	Cc1c(cc(s1)[C@H]2C3C(=O)CC(C=C3N=C(C2C(=O)OC)C)(C)C)CSc4ccc(cc4)Cl
ZINC08441827	6.41	14.92	-18.06	1	4	0	502.101	6	Cc1c(cc(s1)[C@@H]2C3=C(CC(CC3=O)(C)C)NC(=C2C(=O)OC)C)CSc4ccc(cc4)Cl
ZINC08441827	6.53	-1.31	-9.07	0	4	0	502.101	6	Cc1c(cc(s1)[C@@H]2C3C(=O)CC(C=C3N=C(C2C(=O)OC)C)(C)C)CSc4ccc(cc4)Cl
ZINC08441826	6.18	-2.32	-14.63	2	2	0	517.614	7	CC1=C([C@H](n2c(=O)/c(=C\c3c[nH]nc3c4ccccc4)/sc2=N1)c5ccccc5)C(=O)Nc6ccccc6
ZINC08441825	6.18	-2.39	-15.12	2	2	0	517.614	7	CC1=C([C@@H](n2c(=O)/c(=C\c3c[nH]nc3c4ccccc4)/sc2=N1)c5ccccc5)C(=O)Nc6ccccc6
ZINC08441824	6.24	-2	-15.19	2	2	0	547.64	8	CC1=C([C@H](n2c(=O)/c(=C\c3c[nH]nc3c4ccccc4)/sc2=N1)c5ccc(cc5)OC)C(=O)Nc6ccccc6
ZINC08441823	6.24	-2.22	-16.21	2	2	0	547.64	8	CC1=C([C@@H](n2c(=O)/c(=C\c3c[nH]nc3c4ccccc4)/sc2=N1)c5ccc(cc5)OC)C(=O)Nc6ccccc6
ZINC01009265	5.66	-2.07	-15.88	2	2	0	521.602	8	Cc1ccc(o1)[C@H]2C(=C(N=c3n2c(=O)c(=Cc4c[nH]nc4c5ccccc5)s3)C)C(=O)Nc6ccccc6
ZINC01009267	5.66	-1.99	-16.03	2	2	0	521.602	8	Cc1ccc(o1)[C@@H]2C(=C(N=c3n2c(=O)c(=Cc4c[nH]nc4c5ccccc5)s3)C)C(=O)Nc6ccccc6
ZINC08441822	6.17	13.84	-14.57	1	5	0	510.428	6	Cc1cc(c(cc1COc2ccccc2Br)[C@H]3C4=C(CCCC4=O)NC(=C3C(=O)OC)C)C
ZINC08441822	6.29	-0.94	-11.24	0	5	0	510.428	6	Cc1cc(c(cc1COc2ccccc2Br)[C@H]3C4C(=O)CCC=C4N=C(C3C(=O)OC)C)C
ZINC08441821	6.17	13.86	-14.66	1	5	0	510.428	6	Cc1cc(c(cc1COc2ccccc2Br)[C@@H]3C4=C(CCCC4=O)NC(=C3C(=O)OC)C)C
ZINC08441821	6.29	-0.73	-10.34	0	5	0	510.428	6	Cc1cc(c(cc1COc2ccccc2Br)[C@@H]3C4C(=O)CCC=C4N=C(C3C(=O)OC)C)C
ZINC08441820	5.9	13.36	-23.98	1	5	0	472.006	7	CCc1c(cc(s1)[C@H]2C3=C(CCCC3=O)NC(=C2C(=O)OC)C)COc4ccccc4Cl
ZINC08441820	6.02	-1.62	-12.04	0	5	0	472.006	7	CCc1c(cc(s1)[C@H]2C3C(=O)CCC=C3N=C(C2C(=O)OC)C)COc4ccccc4Cl
ZINC08441819	6.62	-0.19	-12.62	1	7	0	622.497	7	c1ccc2c(c1)cc(o2)C(=O)C3=C(C(=O)N([C@H]3c4cccc(c4)Br)c5nnc(s5)SCc6ccc(cc6)F)O
ZINC08441819	6.04	-0.35	-13.59	0	7	0	622.497	7	c1ccc2c(c1)cc(o2)C(=O)[C@H]3[C@@H](N(C(=O)C3=O)c4nnc(s4)SCc5ccc(cc5)F)c6cccc(c6)Br
ZINC08441818	5.9	13.86	-31.82	1	5	0	472.006	7	CCc1c(cc(s1)[C@@H]2C3=C(CCCC3=O)NC(=C2C(=O)OC)C)COc4ccccc4Cl
ZINC08441818	6.02	-1.5	-15.65	0	5	0	472.006	7	CCc1c(cc(s1)[C@@H]2C3C(=O)CCC=C3N=C(C2C(=O)OC)C)COc4ccccc4Cl
ZINC08441817	6.62	-0.13	-13.61	1	7	0	622.497	7	c1ccc2c(c1)cc(o2)C(=O)C3=C(C(=O)N([C@@H]3c4cccc(c4)Br)c5nnc(s5)SCc6ccc(cc6)F)O
ZINC08441817	6.04	-0.31	-16.79	0	7	0	622.497	7	c1ccc2c(c1)cc(o2)C(=O)[C@H]3[C@H](N(C(=O)C3=O)c4nnc(s4)SCc5ccc(cc5)F)c6cccc(c6)Br
ZINC08441816	6.58	-1.72	-15.6	2	2	0	531.641	7	Cc1ccccc1NC(=O)C2=C(N=c3n(c(=O)/c(=C\c4c[nH]nc4c5ccccc5)/s3)[C@H]2c6ccccc6)C
ZINC08441815	4.31	0.94	-17.09	1	9	0	553.596	8	Cc1nnc(s1)N2[C@H](C(=C(C2=O)O)C(=O)c3cc4ccccc4o3)c5ccc(c(c5)OC)OCc6ccccc6
ZINC08441815	3.73	0.8	-18.96	0	9	0	553.596	8	Cc1nnc(s1)N2[C@H]([C@@H](C(=O)C2=O)C(=O)c3cc4ccccc4o3)c5ccc(c(c5)OC)OCc6ccccc6
ZINC08441814	6.58	-1.35	-14.06	2	2	0	531.641	7	Cc1ccccc1NC(=O)C2=C(N=c3n(c(=O)/c(=C\c4c[nH]nc4c5ccccc5)/s3)[C@@H]2c6ccccc6)C
ZINC00703153	4.31	0.88	-17.01	1	9	0	553.596	8	Cc1nnc(s1)N2[C@@H](C(=C(C2=O)O)C(=O)c3cc4ccccc4o3)c5ccc(c(c5)OC)OCc6ccccc6
ZINC00703153	3.73	0.66	-19.59	0	9	0	553.596	8	Cc1nnc(s1)N2[C@@H]([C@@H](C(=O)C2=O)C(=O)c3cc4ccccc4o3)c5ccc(c(c5)OC)OCc6ccccc6
ZINC08441813	6.79	0.9	-8.5	1	0	0	496.072	4	CCOC(=O)C1[C@@H](C2=C(CCCC2=O)N=C1C)c3cc(c(cc3C)C)CSc4ccc(cc4)Cl
ZINC08441813	6.97	-0.38	-10.13	0	4	0	496.072	7	CCOC(=O)C1[C@@H](C2C(=NC1=C)CCCC2=O)c3cc(c(cc3C)C)CSc4ccc(cc4)Cl
ZINC08441813	6.79	0.54	-11.55	0	4	0	496.072	7	CCOC(=O)C1=C(N=C2CCCC(=O)C2[C@H]1c3cc(c(cc3C)C)CSc4ccc(cc4)Cl)C
ZINC08441812	4.8	0.88	-14.81	1	8	0	509.543	6	Cc1nnc(s1)N2[C@H](C(=C(C2=O)O)C(=O)c3cc4ccccc4o3)c5cccc(c5)Oc6ccccc6
ZINC08441812	4.22	0.72	-15.01	0	8	0	509.543	6	Cc1nnc(s1)N2[C@H]([C@@H](C(=O)C2=O)C(=O)c3cc4ccccc4o3)c5cccc(c5)Oc6ccccc6
ZINC08441811	4.8	0.94	-14.93	1	8	0	509.543	6	Cc1nnc(s1)N2[C@@H](C(=C(C2=O)O)C(=O)c3cc4ccccc4o3)c5cccc(c5)Oc6ccccc6
ZINC08441811	4.22	0.77	-15.29	0	8	0	509.543	6	Cc1nnc(s1)N2[C@@H]([C@H](C(=O)C2=O)C(=O)c3cc4ccccc4o3)c5cccc(c5)Oc6ccccc6
ZINC08441810	6.79	0.97	-8.27	1	0	0	496.072	4	CCOC(=O)C1[C@H](C2=C(CCCC2=O)N=C1C)c3cc(c(cc3C)C)CSc4ccc(cc4)Cl
ZINC08441810	6.97	-0.09	-11.08	0	4	0	496.072	7	CCOC(=O)C1[C@H](C2C(=NC1=C)CCCC2=O)c3cc(c(cc3C)C)CSc4ccc(cc4)Cl
ZINC08441810	6.79	0.77	-12.44	0	4	0	496.072	7	CCOC(=O)C1=C(N=C2CCCC(=O)C2[C@@H]1c3cc(c(cc3C)C)CSc4ccc(cc4)Cl)C
ZINC00703148	4.35	0	-13.58	1	7	0	486.336	4	Cc1nnc(s1)N2[C@H](C(=C(C2=O)O)C(=O)c3cc4ccccc4o3)c5ccc(c(c5)Cl)Cl
ZINC00703148	3.77	-0.19	-16.62	0	7	0	486.336	4	Cc1nnc(s1)N2[C@H]([C@H](C(=O)C2=O)C(=O)c3cc4ccccc4o3)c5ccc(c(c5)Cl)Cl

Supplementary Table S2:
Detailed table of XP Docking using Schrodinger's Glide module

ZINC_ID	xLogP	ApolarDesolvation	PolarDesolvation	H Donors	H Acceptors	Charge	MolecularWeight	RotatableBonds	SMILES
ZINC08441809	6.69	2.91	-12.04	1	1	0	485.056	4	[H]/N=C/1\[C@H](C2=CCCC[C@@H]2[C@@H](C1(C#N)C#N)c3cc(c(cc3C)C)CSc4ccc(cc4)Cl)C#N
ZINC08441809	6.69	2.74	-36.92	2	4	1	486.064	4	Cc1cc(c(cc1CSc2ccc(cc2)Cl)[C@H]3[C@H]4CCCC=C4[C@@H](C(=[NH2+])C3(C#N)C#N)C#N)C
ZINC00703149	4.35	-0.01	-13.56	1	7	0	486.336	4	Cc1nnc(s1)N2[C@@H](C(=C(C2=O)O)C(=O)c3cc4ccccc4o3)c5ccc(c(c5)Cl)Cl
ZINC00703149	3.77	-0.2	-16.64	0	7	0	486.336	4	Cc1nnc(s1)N2[C@@H]([C@@H](C(=O)C2=O)C(=O)c3cc4ccccc4o3)c5ccc(c(c5)Cl)Cl
ZINC00703147	3.85	-0.48	-13.95	1	7	0	496.342	4	Cc1nnc(s1)N2[C@@H](C(=C(C2=O)O)C(=O)c3cc4ccccc4o3)c5cccc(c5)Br
ZINC00703147	3.27	-0.66	-16.78	0	7	0	496.342	4	Cc1nnc(s1)N2[C@@H]([C@@H](C(=O)C2=O)C(=O)c3cc4ccccc4o3)c5cccc(c5)Br
ZINC08441808	6.69	2.46	-10.16	1	1	0	485.056	4	[H]/N=C/1\[C@H](C2=CCCC[C@@H]2[C@H](C1(C#N)C#N)c3cc(c(cc3C)C)CSc4ccc(cc4)Cl)C#N
ZINC08441808	6.69	2.29	-41.57	2	4	1	486.064	4	Cc1cc(c(cc1CSc2ccc(cc2)Cl)[C@@H]3[C@H]4CCCC=C4[C@@H](C(=[NH2+])C3(C#N)C#N)C#N)C
ZINC08441807	6.69	3.03	-10.64	1	1	0	485.056	4	[H]/N=C/1\[C@@H](C2=CCCC[C@H]2[C@H](C1(C#N)C#N)c3cc(c(cc3C)C)CSc4ccc(cc4)Cl)C#N
ZINC08441807	6.69	2.86	-37.58	2	4	1	486.064	4	Cc1cc(c(cc1CSc2ccc(cc2)Cl)[C@@H]3[C@@H]4CCCC=C4[C@H](C(=[NH2+])C3(C#N)C#N)C#N)C
ZINC08441806	6.31	0.16	-15.57	1	8	0	579.659	7	Cc1ccc(o1)[C@H]2C(=C(C(=O)N2c3nnc(s3)SCc4cccc5c4cccc5)O)C(=O)c6cc7ccccc7o6
ZINC08441806	5.73	-0.03	-16.07	0	8	0	579.659	7	Cc1ccc(o1)[C@H]2[C@@H](C(=O)C(=O)N2c3nnc(s3)SCc4cccc5c4cccc5)C(=O)c6cc7ccccc7o6
ZINC08441805	5.26	4.19	-15.9	1	1	0	494.595	7	[H]/N=C/1\[C@H](C2=CCCC[C@@H]2[C@@H](C1(C#N)C#N)c3cc(c(cc3C)C)COc4c(cccc4OC)OC)C#N
ZINC08441805	5.26	4.03	-31.06	2	7	1	495.603	6	Cc1cc(c(cc1COc2c(cccc2OC)OC)[C@H]3[C@H]4CCCC=C4[C@@H](C(=[NH2+])C3(C#N)C#N)C#N)C
ZINC08441804	5.26	3.67	-15.86	1	1	0	494.595	7	[H]/N=C/1\[C@H](C2=CCCC[C@@H]2[C@H](C1(C#N)C#N)c3cc(c(cc3C)C)COc4c(cccc4OC)OC)C#N
ZINC08441804	5.26	3.5	-42.35	2	7	1	495.603	6	Cc1cc(c(cc1COc2c(cccc2OC)OC)[C@@H]3[C@H]4CCCC=C4[C@@H](C(=[NH2+])C3(C#N)C#N)C#N)C
ZINC08441803	6.31	0.16	-15.55	1	8	0	579.659	7	Cc1ccc(o1)[C@@H]2C(=C(C(=O)N2c3nnc(s3)SCc4cccc5c4cccc5)O)C(=O)c6cc7ccccc7o6
ZINC08441803	5.73	-0.01	-17.44	0	8	0	579.659	7	Cc1ccc(o1)[C@@H]2[C@@H](C(=O)C(=O)N2c3nnc(s3)SCc4cccc5c4cccc5)C(=O)c6cc7ccccc7o6
ZINC08441802	5.26	3.69	-15.6	1	1	0	494.595	7	[H]/N=C\1/[C@@H](C2=CCCC[C@H]2[C@@H](C1(C#N)C#N)c3cc(c(cc3C)C)COc4c(cccc4OC)OC)C#N
ZINC08441802	5.26	3.51	-41.94	2	7	1	495.603	6	Cc1cc(c(cc1COc2c(cccc2OC)OC)[C@H]3[C@@H]4CCCC=C4[C@H](C(=[NH2+])C3(C#N)C#N)C#N)C
ZINC08441801	6.95	0.73	-14.65	1	7	0	593.661	7	c1ccc2c(c1)cccc2CSc3nnc(s3)N4[C@H](C(=C(C4=O)O)C(=O)c5cc6ccccc6o5)c7ccccc7F
ZINC08441801	6.36	0.56	-14.66	0	7	0	593.661	7	c1ccc2c(c1)cccc2CSc3nnc(s3)N4[C@H]([C@@H](C(=O)C4=O)C(=O)c5cc6ccccc6o5)c7ccccc7F
ZINC08441800	5.26	4.3	-15.13	1	1	0	494.595	7	[H]/N=C/1\[C@@H](C2=CCCC[C@H]2[C@H](C1(C#N)C#N)c3cc(c(cc3C)C)COc4c(cccc4OC)OC)C#N
ZINC08441800	5.26	4.13	-31.23	2	7	1	495.603	6	Cc1cc(c(cc1COc2c(cccc2OC)OC)[C@@H]3[C@@H]4CCCC=C4[C@H](C(=[NH2+])C3(C#N)C#N)C#N)C
ZINC08441799	6.95	0.76	-14.67	1	7	0	593.661	7	c1ccc2c(c1)cccc2CSc3nnc(s3)N4[C@@H](C(=C(C4=O)O)C(=O)c5cc6ccccc6o5)c7ccccc7F
ZINC08441799	6.36	0.58	-14.6	0	7	0	593.661	7	c1ccc2c(c1)cccc2CSc3nnc(s3)N4[C@@H]([C@H](C(=O)C4=O)C(=O)c5cc6ccccc6o5)c7ccccc7F
ZINC08441798	6.64	-1.79	-15.77	2	2	0	561.667	8	Cc1ccccc1NC(=O)C2=C(N=c3n(c(=O)/c(=C\c4c[nH]nc4c5ccccc5)/s3)[C@H]2c6ccc(cc6)OC)C
ZINC08441797	6.93	0.01	-14.54	1	8	0	618.74	8	CN(C)c1ccc(cc1)[C@H]2C(=C(C(=O)N2c3nnc(s3)SCc4cccc5c4cccc5)O)C(=O)c6cc7ccccc7o6
ZINC08441796	6.64	-1.3	-14.78	2	2	0	561.667	8	Cc1ccccc1NC(=O)C2=C(N=c3n(c(=O)/c(=C\c4c[nH]nc4c5ccccc5)/s3)[C@@H]2c6ccc(cc6)OC)C
ZINC08441795	6.93	-0.04	-14.63	1	8	0	618.74	8	CN(C)c1ccc(cc1)[C@@H]2C(=C(C(=O)N2c3nnc(s3)SCc4cccc5c4cccc5)O)C(=O)c6cc7ccccc7o6
ZINC00650985	4.9	-2.18	-8.98	0	3	0	436.968	5	Cc1cc(c(cc1CSc2ccc(cc2)Cl)[C@H]3c4c(n[nH]c4OC(=C3C#N)N)C)C
ZINC08441794	8.34	0.79	-13.42	1	7	0	617.752	8	CC(C)c1ccc(cc1)[C@H]2C(=C(C(=O)N2c3nnc(s3)SCc4cccc5c4cccc5)O)C(=O)c6cc7ccccc7o6
ZINC08441794	7.76	0.62	-14.63	0	7	0	617.752	8	CC(C)c1ccc(cc1)[C@H]2[C@@H](C(=O)C(=O)N2c3nnc(s3)SCc4cccc5c4cccc5)C(=O)c6cc7ccccc7o6
ZINC00650986	4.9	-2.19	-8.77	0	3	0	436.968	5	Cc1cc(c(cc1CSc2ccc(cc2)Cl)[C@@H]3c4c(n[nH]c4OC(=C3C#N)N)C)C
ZINC08441793	8.34	0.7	-13.46	1	7	0	617.752	8	CC(C)c1ccc(cc1)[C@@H]2C(=C(C(=O)N2c3nnc(s3)SCc4cccc5c4cccc5)O)C(=O)c6cc7ccccc7o6
ZINC08441792	6.56	14.27	-23.21	1	5	0	500.06	7	CCc1c(cc(s1)[C@H]2C3=C(CC(CC3=O)(C)C)NC(=C2C(=O)OC)C)COc4ccccc4Cl
ZINC08441792	6.67	-0.56	-11.68	0	5	0	500.06	7	CCc1c(cc(s1)[C@H]2C3C(=O)CC(C=C3N=C(C2C(=O)OC)C)(C)C)COc4ccccc4Cl
ZINC08441791	7.53	0.47	-14.35	1	8	0	631.735	10	C=CCOc1ccc(cc1)[C@H]2C(=C(C(=O)N2c3nnc(s3)SCc4cccc5c4cccc5)O)C(=O)c6cc7ccccc7o6
ZINC08441791	6.95	0.31	-15.55	0	8	0	631.735	10	C=CCOc1ccc(cc1)[C@H]2[C@@H](C(=O)C(=O)N2c3nnc(s3)SCc4cccc5c4cccc5)C(=O)c6cc7ccccc7o6
ZINC08441790	6.56	14.27	-23.43	1	5	0	500.06	7	CCc1c(cc(s1)[C@@H]2C3=C(CC(CC3=O)(C)C)NC(=C2C(=O)OC)C)COc4ccccc4Cl
ZINC08441790	6.67	-0.71	-11.9	0	5	0	500.06	7	CCc1c(cc(s1)[C@@H]2C3C(=O)CC(C=C3N=C(C2C(=O)OC)C)(C)C)COc4ccccc4Cl
ZINC08441789	7.53	0.39	-14.33	1	8	0	631.735	10	C=CCOc1ccc(cc1)[C@@H]2C(=C(C(=O)N2c3nnc(s3)SCc4cccc5c4cccc5)O)C(=O)c6cc7ccccc7o6
ZINC08441789	6.95	0.21	-16.44	0	8	0	631.735	10	C=CCOc1ccc(cc1)[C@@H]2[C@@H](C(=O)C(=O)N2c3nnc(s3)SCc4cccc5c4cccc5)C(=O)c6cc7ccccc7o6
ZINC08441788	8.33	0.22	-14.04	1	8	0	647.778	11	CCCCOc1ccc(cc1)[C@H]2C(=C(C(=O)N2c3nnc(s3)SCc4cccc5c4cccc5)O)C(=O)c6cc7ccccc7o6
ZINC08441788	7.74	0.05	-15.17	0	8	0	647.778	11	CCCCOc1ccc(cc1)[C@H]2[C@@H](C(=O)C(=O)N2c3nnc(s3)SCc4cccc5c4cccc5)C(=O)c6cc7ccccc7o6
ZINC08441787	6.5	0.82	-10.55	2	0	0	524.455	5	CCOC(=O)C1[C@@H](C2=C(CCCC2=O)N=C1C)c3cc(c(cc3C)C)COc4ccccc4Br
ZINC08441787	6.5	0.45	-14.13	0	5	0	524.455	7	CCOC(=O)C1=C(N=C2CCCC(=O)C2[C@H]1c3cc(c(cc3C)C)COc4ccccc4Br)C
ZINC08441787	6.68	-0.46	-12.87	0	5	0	524.455	7	CCOC(=O)C1[C@@H](C2C(=NC1=C)CCCC2=O)c3cc(c(cc3C)C)COc4ccccc4Br
ZINC08441786	8.33	0.22	-14.02	1	8	0	647.778	11	CCCCOc1ccc(cc1)[C@@H]2C(=C(C(=O)N2c3nnc(s3)SCc4cccc5c4cccc5)O)C(=O)c6cc7ccccc7o6
ZINC08441786	7.74	0.06	-15.19	0	8	0	647.778	11	CCCCOc1ccc(cc1)[C@@H]2[C@H](C(=O)C(=O)N2c3nnc(s3)SCc4cccc5c4cccc5)C(=O)c6cc7ccccc7o6
ZINC08441785	6.55	-1.46	-15.63	2	9	0	635.723	9	CCOc1cc(ccc1O)[C@H]2C(=C(C(=O)N2c3nnc(s3)SCc4cccc5c4cccc5)O)C(=O)c6cc7ccccc7o6
ZINC08441785	5.96	-1.6	-17.44	1	9	0	635.723	9	CCOc1cc(ccc1O)[C@H]2[C@@H](C(=O)C(=O)N2c3nnc(s3)SCc4cccc5c4cccc5)C(=O)c6cc7ccccc7o6
ZINC08441784	6.5	0.84	-10.58	2	0	0	524.455	5	CCOC(=O)C1[C@H](C2=C(CCCC2=O)N=C1C)c3cc(c(cc3C)C)COc4ccccc4Br
ZINC08441784	6.68	-0.23	-13.04	0	5	0	524.455	7	CCOC(=O)C1[C@H](C2C(=NC1=C)CCCC2=O)c3cc(c(cc3C)C)COc4ccccc4Br
ZINC08441784	6.5	0.64	-13.83	0	5	0	524.455	7	CCOC(=O)C1=C(N=C2CCCC(=O)C2[C@@H]1c3cc(c(cc3C)C)COc4ccccc4Br)C
ZINC08441783	6.55	-1.48	-15.64	2	9	0	635.723	9	CCOc1cc(ccc1O)[C@@H]2C(=C(C(=O)N2c3nnc(s3)SCc4cccc5c4cccc5)O)C(=O)c6cc7ccccc7o6
ZINC08441783	5.96	-1.7	-18.02	1	9	0	635.723	9	CCOc1cc(ccc1O)[C@@H]2[C@@H](C(=O)C(=O)N2c3nnc(s3)SCc4cccc5c4cccc5)C(=O)c6cc7ccccc7o6
ZINC08441782	8.54	1.14	-13.4	1	7	0	631.779	8	CC(C)(C)c1ccc(cc1)[C@H]2C(=C(C(=O)N2c3nnc(s3)SCc4cccc5c4cccc5)O)C(=O)c6cc7ccccc7o6
ZINC08441782	7.95	0.97	-14.3	0	7	0	631.779	8	CC(C)(C)c1ccc(cc1)[C@H]2[C@@H](C(=O)C(=O)N2c3nnc(s3)SCc4cccc5c4cccc5)C(=O)c6cc7ccccc7o6
ZINC08441781	6.46	15.16	-15.95	1	4	0	482.045	6	Cc1cc(c(cc1CSc2ccc(cc2)Cl)[C@H]3C4=C(CCCC4=O)NC(=C3C(=O)OC)C)C
ZINC08441781	6.58	-0.81	-8.69	0	4	0	482.045	6	Cc1cc(c(cc1CSc2ccc(cc2)Cl)[C@H]3C4C(=O)CCC=C4N=C(C3C(=O)OC)C)C

Supplementary Table S2:
Detailed table of XP Docking using Schrodinger's Glide module

ZINC_ID	xLogP	ApolarDesolvation	PolarDesolvation	H Donors	H Acceptors	Charge	MolecularWeight	RotatableBonds	SMILES
ZINC08441780	8.54	1.05	-13.46	1	7	0	631.779	8	CC(C)(C)c1ccc(cc1)[C@@H]2C(=C(C(=O)N2c3nnc(s3)SCc4cccc5c4cccc5)O)C(=O)c6cc7ccccc7o6
ZINC08441780	7.95	0.88	-15.29	0	7	0	631.779	8	CC(C)(C)c1ccc(cc1)[C@@H]2[C@@H](C(=O)C(=O)N2c3nnc(s3)SCc4cccc5c4cccc5)C(=O)c6cc7ccccc7o6
ZINC08441779	6.46	15.16	-16.44	1	4	0	482.045	6	Cc1cc(c(cc1CSc2ccc(cc2)Cl)[C@@H]3C4=C(CCCC4=O)NC(=C3C(=O)OC)C)C
ZINC08441779	6.58	-0.6	-8.06	0	4	0	482.045	6	Cc1cc(c(cc1CSc2ccc(cc2)Cl)[C@@H]3C4C(=O)CCC=C4N=C(C3C(=O)OC)C)C
ZINC08441778	6.35	-2.07	-15	2	8	0	591.67	7	c1ccc2c(c1)cccc2CSc3nnc(s3)N4[C@@H](C(=C(C4=O)O)C(=O)c5cc6ccccc6o5)c7ccc(cc7)O
ZINC08441778	5.77	-2.24	-17.23	1	8	0	591.67	7	c1ccc2c(c1)cccc2CSc3nnc(s3)N4[C@@H]([C@@H](C(=O)C4=O)C(=O)c5cc6ccccc6o5)c7ccc(cc7)O
ZINC08441777	7.11	16.06	-15.25	1	4	0	510.099	6	Cc1cc(c(cc1CSc2ccc(cc2)Cl)[C@H]3C4=C(CC(CC4=O)(C)C)NC(=C3C(=O)OC)C)C
ZINC08441776	6.73	-1.25	-14.09	1	7	0	581.7	7	c1ccc2c(c1)cccc2CSc3nnc(s3)N4[C@H](C(=C(C4=O)O)C(=O)c5cc6ccccc6o5)c7cccs7
ZINC08441776	6.15	-1.44	-16.29	0	7	0	581.7	7	c1ccc2c(c1)cccc2CSc3nnc(s3)N4[C@H]([C@H](C(=O)C4=O)C(=O)c5cc6ccccc6o5)c7cccs7
ZINC08441775	6.73	-1.25	-14.2	1	7	0	581.7	7	c1ccc2c(c1)cccc2CSc3nnc(s3)N4[C@@H](C(=C(C4=O)O)C(=O)c5cc6ccccc6o5)c7cccs7
ZINC08441775	6.15	-1.42	-16.38	0	7	0	581.7	7	c1ccc2c(c1)cccc2CSc3nnc(s3)N4[C@@H]([C@@H](C(=O)C4=O)C(=O)c5cc6ccccc6o5)c7cccs7
ZINC08441774	7.11	16.07	-15.1	1	4	0	510.099	6	Cc1cc(c(cc1CSc2ccc(cc2)Cl)[C@@H]3C4=C(CC(CC4=O)(C)C)NC(=C3C(=O)OC)C)C
ZINC08441774	7.23	0.46	-7.48	0	4	0	510.099	6	Cc1cc(c(cc1CSc2ccc(cc2)Cl)[C@@H]3C4C(=O)CC(C=C4N=C(C3C(=O)OC)C)(C)C)C
ZINC08441772	6.93	15.19	-23.1	1	5	0	514.087	8	CCc1c(cc(s1)[C@H]2C3=C(CC(CC3=O)(C)C)NC(=C2C(=O)OCC)C)COc4ccccc4Cl
ZINC08441770	6.93	15.71	-31.54	1	5	0	514.087	8	CCc1c(cc(s1)[C@@H]2C3=C(CC(CC3=O)(C)C)NC(=C2C(=O)OCC)C)COc4ccccc4Cl
ZINC08441770	7.05	-0.71	-14.47	0	5	0	514.087	8	CCc1c(cc(s1)[C@@H]2C3C(=O)CC(C=C3N=C(C2C(=O)OCC)C)(C)C)COc4ccccc4Cl
ZINC08441769	7.28	0.31	-13.77	1	7	0	589.698	7	Cc1ccc(cc1)[C@H]2C(=C(C(=O)N2c3nnc(s3)SCc4cccc5c4cccc5)O)C(=O)c6cc7ccccc7o6
ZINC08441769	6.7	0.14	-14.92	0	7	0	589.698	7	Cc1ccc(cc1)[C@H]2[C@@H](C(=O)C(=O)N2c3nnc(s3)SCc4cccc5c4cccc5)C(=O)c6cc7ccccc7o6
ZINC08441768	5.3	0.03	-12.7	1	1	0	486.553	8	CC1=C([C@H](n2c(=O)/c(=C\c3c[nH]nc3c4ccccc4)/sc2=N1)c5ccc(cc5)OC)C(=O)OC
ZINC08441767	7.28	0.22	-13.76	1	7	0	589.698	7	Cc1ccc(cc1)[C@@H]2C(=C(C(=O)N2c3nnc(s3)SCc4cccc5c4cccc5)O)C(=O)c6cc7ccccc7o6
ZINC08441767	6.7	0.04	-15.73	0	7	0	589.698	7	Cc1ccc(cc1)[C@@H]2[C@@H](C(=O)C(=O)N2c3nnc(s3)SCc4cccc5c4cccc5)C(=O)c6cc7ccccc7o6
ZINC08441766	5.3	0.16	-12.12	1	1	0	486.553	8	CC1=C([C@@H](n2c(=O)/c(=C\c3c[nH]nc3c4ccccc4)/sc2=N1)c5ccc(cc5)OC)C(=O)OC
ZINC08441765	5.99	0.99	-13.43	1	6	0	515.565	8	c1ccc(cc1)CN2[C@H](C(=C(C2=O)O)C(=O)c3cc4ccccc4o3)c5ccc(cc5)OCc6ccccc6
ZINC08441765	5.41	0.8	-18.83	0	6	0	515.565	8	c1ccc(cc1)CN2[C@H]([C@H](C(=O)C2=O)C(=O)c3cc4ccccc4o3)c5ccc(cc5)OCc6ccccc6
ZINC08441764	5.78	0.87	-10.68	1	1	0	488.544	7	CCOC(=O)C1=C(N=c2n(c(=O)/c(=C\c3c[nH]nc3c4ccccc4)/s2)[C@H]1c5ccc(cc5)F)C
ZINC08441763	5.99	0.59	-14.1	1	6	0	515.565	8	c1ccc(cc1)CN2[C@@H](C(=C(C2=O)O)C(=O)c3cc4ccccc4o3)c5ccc(cc5)OCc6ccccc6
ZINC08441763	5.41	0.41	-20.98	0	6	0	515.565	8	c1ccc(cc1)CN2[C@@H]([C@@H](C(=O)C2=O)C(=O)c3cc4ccccc4o3)c5ccc(cc5)OCc6ccccc6
ZINC08441762	5.78	0.7	-11.32	1	1	0	488.544	7	CCOC(=O)C1=C(N=c2n(c(=O)/c(=C\c3c[nH]nc3c4ccccc4)/s2)[C@@H]1c5ccc(cc5)F)C
ZINC08441761	5.93	1.39	-14.58	1	7	0	525.601	11	CCCCCOc1ccc(cc1OC)[C@@H]2C(=C(C(=O)N2Cc3ccccc3)O)C(=O)c4cc5ccccc5o4
ZINC08441761	5.35	1.18	-22.02	0	7	0	525.601	11	CCCCCOc1ccc(cc1OC)[C@@H]2[C@@H](C(=O)C(=O)N2Cc3ccccc3)C(=O)c4cc5ccccc5o4
ZINC08441760	6.4	0.9	-14.01	1	6	0	529.592	9	c1ccc(cc1)CCN2[C@@H](C(=C(C2=O)O)C(=O)c3cc4ccccc4o3)c5ccc(cc5)OCc6ccccc6
ZINC08441760	5.81	0.74	-19.14	0	6	0	529.592	9	c1ccc(cc1)CCN2[C@@H]([C@H](C(=O)C2=O)C(=O)c3cc4ccccc4o3)c5ccc(cc5)OCc6ccccc6
ZINC08441759	5.36	2.11	-15.06	2	0	0	505.611	7	CCOC(=O)C1[C@@H](C2=C(CCCC2=O)N=C1C)c3cc(c(cc3C)C)COc4c(cccc4OC)OC
ZINC08441759	5.36	1.75	-19.36	0	7	0	505.611	9	CCOC(=O)C1=C(N=C2CCCC(=O)C2[C@H]1c3cc(c(cc3C)C)COc4c(cccc4OC)OC)C
ZINC08441759	5.54	0.83	-17.75	0	7	0	505.611	9	CCOC(=O)C1[C@@H](C2C(=NC1=C)CCCC2=O)c3cc(c(cc3C)C)COc4c(cccc4OC)OC
ZINC05917285	6.45	1.88	-11.27	1	5	0	479.576	7	CC(C)(C)c1ccc(cc1)[C@H]2C(=C(C(=O)N2CCc3ccccc3)O)C(=O)c4cc5ccccc5o4
ZINC05917285	5.87	1.7	-16.95	0	5	0	479.576	7	CC(C)(C)c1ccc(cc1)[C@H]2[C@H](C(=O)C(=O)N2CCc3ccccc3)C(=O)c4cc5ccccc5o4
ZINC08441758	5.36	2.18	-15.04	2	0	0	505.611	7	CCOC(=O)C1[C@H](C2=C(CCCC2=O)N=C1C)c3cc(c(cc3C)C)COc4c(cccc4OC)OC
ZINC08441758	5.36	1.98	-18.73	0	7	0	505.611	9	CCOC(=O)C1=C(N=C2CCCC(=O)C2[C@@H]1c3cc(c(cc3C)C)COc4c(cccc4OC)OC)C
ZINC08441758	5.54	1.11	-17.68	0	7	0	505.611	9	CCOC(=O)C1[C@H](C2C(=NC1=C)CCCC2=O)c3cc(c(cc3C)C)COc4c(cccc4OC)OC
ZINC05917235	6.45	1.86	-11.31	1	5	0	479.576	7	CC(C)(C)c1ccc(cc1)[C@@H]2C(=C(C(=O)N2CCc3ccccc3)O)C(=O)c4cc5ccccc5o4
ZINC05917235	5.87	1.67	-16.82	0	5	0	479.576	7	CC(C)(C)c1ccc(cc1)[C@@H]2[C@@H](C(=O)C(=O)N2CCc3ccccc3)C(=O)c4cc5ccccc5o4
ZINC08441757	6.29	-0.02	-10.29	2	1	0	504.999	7	CCOC(=O)C1=C(N=c2n(c(=O)/c(=C\c3c[nH]nc3c4ccccc4)/s2)[C@H]1c5ccc(cc5)Cl)C
ZINC08441756	7.33	-0.05	-14.77	1	8	0	588.641	8	CCOc1ccc2c(c1)sc(n2)N3[C@H](C(=C(C3=O)O)C(=O)c4cc5ccccc5o4)c6cccc(c6)Oc7ccccc7
ZINC08441756	6.75	-0.25	-16.19	0	8	0	588.641	8	CCOc1ccc2c(c1)sc(n2)N3[C@H]([C@H](C(=O)C3=O)C(=O)c4cc5ccccc5o4)c6cccc(c6)Oc7ccccc7
ZINC08441755	6.29	-0.19	-10.83	2	1	0	504.999	7	CCOC(=O)C1=C(N=c2n(c(=O)/c(=C\c3c[nH]nc3c4ccccc4)/s2)[C@@H]1c5ccc(cc5)Cl)C
ZINC08441754	7.33	-0.03	-14.64	1	8	0	588.641	8	CCOc1ccc2c(c1)sc(n2)N3[C@@H](C(=C(C3=O)O)C(=O)c4cc5ccccc5o4)c6cccc(c6)Oc7ccccc7
ZINC08441754	6.75	-0.21	-16.23	0	8	0	588.641	8	CCOc1ccc2c(c1)sc(n2)N3[C@@H]([C@@H](C(=O)C3=O)C(=O)c4cc5ccccc5o4)c6cccc(c6)Oc7ccccc7
ZINC00703142	5.25	-0.23	-16.66	1	9	0	556.596	8	CCOc1ccc2c(c1)sc(n2)N3[C@H](C(=C(C3=O)O)C(=O)c4cc5ccccc5o4)c6ccc(c(c6)OC)OC
ZINC00703142	4.67	-0.35	-19.45	0	9	0	556.596	8	CCOc1ccc2c(c1)sc(n2)N3[C@H]([C@@H](C(=O)C3=O)C(=O)c4cc5ccccc5o4)c6ccc(c(c6)OC)OC
ZINC00703143	5.25	-0.21	-16.64	1	9	0	556.596	8	CCOc1ccc2c(c1)sc(n2)N3[C@@H](C(=C(C3=O)O)C(=O)c4cc5ccccc5o4)c6ccc(c(c6)OC)OC
ZINC00703143	4.67	-0.34	-19.44	0	9	0	556.596	8	CCOc1ccc2c(c1)sc(n2)N3[C@@H]([C@H](C(=O)C3=O)C(=O)c4cc5ccccc5o4)c6ccc(c(c6)OC)OC
ZINC08441751	7.2	0.1	-16.17	1	9	0	612.704	12	CCCCCOc1ccc(cc1OC)[C@H]2C(=C(C(=O)N2c3nc4ccc(cc4s3)OCC)O)C(=O)c5cc6ccccc6o5
ZINC08441751	6.61	-0.02	-19.04	0	9	0	612.704	12	CCCCCOc1ccc(cc1OC)[C@H]2[C@@H](C(=O)C(=O)N2c3nc4ccc(cc4s3)OCC)C(=O)c5cc6ccccc6o5
ZINC08441750	6.82	15.13	-13.73	1	5	0	538.482	6	Cc1cc(c(cc1COc2ccccc2Br)[C@H]3C4=C(CC(CC4=O)(C)C)NC(=C3C(=O)OC)C)C
ZINC08441750	6.94	0.34	-9.91	0	5	0	538.482	6	Cc1cc(c(cc1COc2ccccc2Br)[C@H]3C4C(=O)CC(C=C4N=C(C3C(=O)OC)C)(C)C)C
ZINC08441749	7.2	0.12	-16.13	1	9	0	612.704	12	CCCCCOc1ccc(cc1OC)[C@@H]2C(=C(C(=O)N2c3nc4ccc(cc4s3)OCC)O)C(=O)c5cc6ccccc6o5
ZINC08441749	6.61	-0.01	-19.02	0	9	0	612.704	12	CCCCCOc1ccc(cc1OC)[C@@H]2[C@H](C(=O)C(=O)N2c3nc4ccc(cc4s3)OCC)C(=O)c5cc6ccccc6o5
ZINC00703140	5.13	-2.72	-14.71	2	8	0	512.543	6	CCOc1ccc2c(c1)sc(n2)N3[C@H](C(=C(C3=O)O)C(=O)c4cc5ccccc5o4)c6ccc(cc6)O
ZINC00703140	4.54	-2.87	-16.5	1	8	0	512.543	6	CCOc1ccc2c(c1)sc(n2)N3[C@H]([C@@H](C(=O)C3=O)C(=O)c4cc5ccccc5o4)c6ccc(cc6)O
ZINC00703141	5.13	-2.7	-14.56	2	8	0	512.543	6	CCOc1ccc2c(c1)sc(n2)N3[C@@H](C(=C(C3=O)O)C(=O)c4cc5ccccc5o4)c6ccc(cc6)O

Supplementary Table S2:
Detailed table of XP Docking using Schrodinger's Glide module

ZINC_ID	xLogP	ApolarDesolvation	PolarDesolvation	H Donors	H Acceptors	Charge	MolecularWeight	RotatableBonds	SMILES
ZINC00703141	4.54	-2.88	-17.32	1	8	0	512.543	6	CCOc1ccc2c(c1)sc(n2)N3[C@@H]([C@@H](C(=O)C3=O)C(=O)c4cc5ccccc5o4)c6ccc(cc6)O
ZINC08441748	6.82	15.15	-13.68	1	5	0	538.482	6	Cc1cc(c(cc1COc2ccccc2Br)[C@@H]3C4=C(CC(CC4=O)(C)C)NC(=C3C(=O)OC)C)C
ZINC08441748	6.94	0.32	-9.91	0	5	0	538.482	6	Cc1cc(c(cc1COc2ccccc2Br)[C@@H]3C4C(=O)CC(C=C4N=C(C3C(=O)OC)C)(C)C)C
ZINC08441747	5.5	-1.93	-13.89	1	7	0	502.573	6	CCOc1ccc2c(c1)sc(n2)N3[C@H](C(=C(C3=O)O)C(=O)c4cc5ccccc5o4)c6cccs6
ZINC08441747	4.92	-2.1	-16.64	0	7	0	502.573	6	CCOc1ccc2c(c1)sc(n2)N3[C@H]([C@H](C(=O)C3=O)C(=O)c4cc5ccccc5o4)c6cccs6
ZINC08441746	6.39	-4.09	-10.09	2	1	0	513.439	5	[H]/N=C/1\[C@H](C2=CCCC[C@@H]2[C@@H](C1(C#N)C#N)c3cc(c(cc3C)C)COc4ccccc4Br)C#N
ZINC08441746	6.39	-3.32	-35.96	2	5	1	514.447	4	Cc1cc(c(cc1COc2ccccc2Br)[C@H]3[C@H]4CCCC=C4[C@@H](C(=[NH2+])C3(C#N)C#N)C#N)C
ZINC08441745	6.39	2.33	-12.34	2	1	0	513.439	5	[H]/N=C/1\[C@H](C2=CCCC[C@@H]2[C@H](C1(C#N)C#N)c3cc(c(cc3C)C)COc4ccccc4Br)C#N
ZINC00703139	5.5	-1.93	-13.86	1	7	0	502.573	6	CCOc1ccc2c(c1)sc(n2)N3[C@@H](C(=C(C3=O)O)C(=O)c4cc5ccccc5o4)c6cccs6
ZINC00703139	4.92	-2.11	-15.71	0	7	0	502.573	6	CCOc1ccc2c(c1)sc(n2)N3[C@@H]([C@H](C(=O)C3=O)C(=O)c4cc5ccccc5o4)c6cccs6
ZINC08441744	6.39	2.29	-12.3	2	1	0	513.439	5	[H]/N=C/1\[C@@H](C2=CCCC[C@H]2[C@@H](C1(C#N)C#N)c3cc(c(cc3C)C)COc4ccccc4Br)C#N
ZINC08441744	6.39	2.12	-38.92	2	5	1	514.447	4	Cc1cc(c(cc1COc2ccccc2Br)[C@H]3[C@@H]4CCCC=C4[C@H](C(=[NH2+])C3(C#N)C#N)C#N)C
ZINC08441743	6.41	-1.51	-12.59	1	7	0	575.44	6	CCOc1ccc2c(c1)sc(n2)N3[C@H](C(=C(C3=O)O)C(=O)c4cc5ccccc5o4)c6ccc(cc6)Br
ZINC08441743	5.83	-1.7	-15.51	0	7	0	575.44	6	CCOc1ccc2c(c1)sc(n2)N3[C@H]([C@H](C(=O)C3=O)C(=O)c4cc5ccccc5o4)c6ccc(cc6)Br
ZINC08441742	6.41	-1.46	-12.61	1	7	0	575.44	6	CCOc1ccc2c(c1)sc(n2)N3[C@@H](C(=C(C3=O)O)C(=O)c4cc5ccccc5o4)c6ccc(cc6)Br
ZINC08441742	5.83	-1.63	-15.57	0	7	0	575.44	6	CCOc1ccc2c(c1)sc(n2)N3[C@@H]([C@@H](C(=O)C3=O)C(=O)c4cc5ccccc5o4)c6ccc(cc6)Br
ZINC08441741	6.39	-5.94	-9.94	2	1	0	513.439	5	[H]/N=C/1\[C@@H](C2=CCCC[C@H]2[C@H](C1(C#N)C#N)c3cc(c(cc3C)C)COc4ccccc4Br)C#N
ZINC08441741	6.39	-3.11	-36.22	2	5	1	514.447	4	Cc1cc(c(cc1COc2ccccc2Br)[C@@H]3[C@@H]4CCCC=C4[C@H](C(=[NH2+])C3(C#N)C#N)C#N)C
ZINC00703136	5.77	-0.01	-13.02	1	7	0	514.534	6	CCOc1ccc2c(c1)sc(n2)N3[C@H](C(=C(C3=O)O)C(=O)c4cc5ccccc5o4)c6ccc(cc6)F
ZINC00703136	5.18	-0.17	-15.1	0	7	0	514.534	6	CCOc1ccc2c(c1)sc(n2)N3[C@H]([C@@H](C(=O)C3=O)C(=O)c4cc5ccccc5o4)c6ccc(cc6)F
ZINC00703137	5.77	0.04	-13.09	1	7	0	514.534	6	CCOc1ccc2c(c1)sc(n2)N3[C@@H](C(=C(C3=O)O)C(=O)c4cc5ccccc5o4)c6ccc(cc6)F
ZINC00703137	5.18	-0.13	-16.18	0	7	0	514.534	6	CCOc1ccc2c(c1)sc(n2)N3[C@@H]([C@@H](C(=O)C3=O)C(=O)c4cc5ccccc5o4)c6ccc(cc6)F
ZINC08441740	7.11	15.29	-16.8	1	5	0	508.058	6	Cc1cc(c(cc1COc2ccc(cc2C)Cl)[C@H]3C4=C(CC(CC4=O)(C)C)NC(=C3C(=O)OC)C)C
ZINC08441740	7.23	1.27	-8.39	0	5	0	508.058	6	Cc1cc(c(cc1COc2ccc(cc2C)Cl)[C@H]3C4C(=O)CC(C=C4N=C(C3C(=O)OC)C)(C)C)C
ZINC00703134	6.05	-0.43	-13.47	1	7	0	510.571	6	CCOc1ccc2c(c1)sc(n2)N3[C@H](C(=C(C3=O)O)C(=O)c4cc5ccccc5o4)c6ccc(cc6)C
ZINC00703134	5.47	-0.59	-15	0	7	0	510.571	6	CCOc1ccc2c(c1)sc(n2)N3[C@H]([C@@H](C(=O)C3=O)C(=O)c4cc5ccccc5o4)c6ccc(cc6)C
ZINC08441739	7.11	15.31	-16.69	1	5	0	508.058	6	Cc1cc(c(cc1COc2ccc(cc2C)Cl)[C@@H]3C4=C(CC(CC4=O)(C)C)NC(=C3C(=O)OC)C)C
ZINC08441739	7.23	1.26	-8.35	0	5	0	508.058	6	Cc1cc(c(cc1COc2ccc(cc2C)Cl)[C@@H]3C4C(=O)CC(C=C4N=C(C3C(=O)OC)C)(C)C)C
ZINC00703135	6.05	-0.41	-13.53	1	7	0	510.571	6	CCOc1ccc2c(c1)sc(n2)N3[C@@H](C(=C(C3=O)O)C(=O)c4cc5ccccc5o4)c6ccc(cc6)C
ZINC00703135	5.47	-0.59	-16.06	0	7	0	510.571	6	CCOc1ccc2c(c1)sc(n2)N3[C@@H]([C@@H](C(=O)C3=O)C(=O)c4cc5ccccc5o4)c6ccc(cc6)C
ZINC08441738	6.26	-0.87	-12.4	1	7	0	530.989	6	CCOc1ccc2c(c1)sc(n2)N3[C@H](C(=C(C3=O)O)C(=O)c4cc5ccccc5o4)c6cccc(c6)Cl
ZINC08441738	5.68	-1.03	-13.73	0	7	0	530.989	6	CCOc1ccc2c(c1)sc(n2)N3[C@H]([C@@H](C(=O)C3=O)C(=O)c4cc5ccccc5o4)c6cccc(c6)Cl
ZINC08441737	6.26	-0.85	-14.14	1	7	0	530.989	6	CCOc1ccc2c(c1)sc(n2)N3[C@@H](C(=C(C3=O)O)C(=O)c4cc5ccccc5o4)c6cccc(c6)Cl
ZINC08441737	5.68	-1.02	-17.74	0	7	0	530.989	6	CCOc1ccc2c(c1)sc(n2)N3[C@@H]([C@@H](C(=O)C3=O)C(=O)c4cc5ccccc5o4)c6cccc(c6)Cl
ZINC08441736	6.18	13.79	-15.32	1	5	0	483.967	6	Cc1cc(c(cc1COc2ccc(cc2Cl)F)[C@H]3C4=C(CCCC4=O)NC(=C3C(=O)OC)C)C
ZINC08441736	6.29	0.38	-10.99	0	5	0	483.967	6	Cc1cc(c(cc1COc2ccc(cc2Cl)F)[C@H]3C4C(=O)CCC=C4N=C(C3C(=O)OC)C)C
ZINC08441735	6.85	-0.07	-17.42	1	9	0	632.694	10	CCOc1ccc2c(c1)sc(n2)N3[C@H](C(=C(C3=O)O)C(=O)c4cc5ccccc5o4)c6ccc(c(c6)OC)OCc7ccccc7
ZINC08441735	6.26	-0.19	-20.38	0	9	0	632.694	10	CCOc1ccc2c(c1)sc(n2)N3[C@H]([C@@H](C(=O)C3=O)C(=O)c4cc5ccccc5o4)c6ccc(c(c6)OC)OCc7ccccc7
ZINC08441734	6.18	13.79	-15.32	1	5	0	483.967	6	Cc1cc(c(cc1COc2ccc(cc2Cl)F)[C@@H]3C4=C(CCCC4=O)NC(=C3C(=O)OC)C)C
ZINC08441734	6.29	0.59	-10.38	0	5	0	483.967	6	Cc1cc(c(cc1COc2ccc(cc2Cl)F)[C@@H]3C4C(=O)CCC=C4N=C(C3C(=O)OC)C)C
ZINC08441733	6.85	-0.05	-17.41	1	9	0	632.694	10	CCOc1ccc2c(c1)sc(n2)N3[C@@H](C(=C(C3=O)O)C(=O)c4cc5ccccc5o4)c6ccc(c(c6)OC)OCc7ccccc7
ZINC08441733	6.26	-0.18	-20.38	0	9	0	632.694	10	CCOc1ccc2c(c1)sc(n2)N3[C@@H]([C@H](C(=O)C3=O)C(=O)c4cc5ccccc5o4)c6ccc(c(c6)OC)OCc7ccccc7
ZINC08441732	6.31	-0.26	-13.75	1	8	0	552.608	9	CCOc1ccc2c(c1)sc(n2)N3[C@H](C(=C(C3=O)O)C(=O)c4cc5ccccc5o4)c6ccc(cc6)OCC=C
ZINC08441732	5.72	-0.42	-15.36	0	8	0	552.608	9	CCOc1ccc2c(c1)sc(n2)N3[C@H]([C@@H](C(=O)C3=O)C(=O)c4cc5ccccc5o4)c6ccc(cc6)OCC=C
ZINC08441731	6.45	-0.14	-16.67	2	0	0	544.511	5	CCOC(=O)C1[C@@H](C2=C(CC(CC2=O)(C)C)N=C1C)c3cc(c(s3)C)COc4ccccc4Br
ZINC08441731	6.63	-1.03	-15.85	0	5	0	544.511	7	CCOC(=O)C1[C@@H](C2C(=NC1=C)CC(CC2=O)(C)C)c3cc(c(s3)C)COc4ccccc4Br
ZINC08441731	6.45	0.02	-18.9	0	5	0	544.511	7	CCOC(=O)C1=C(N=C2CC(CC(=O)C2[C@H]1c3cc(c(s3)C)COc4ccccc4Br)(C)C)C
ZINC08441730	6.31	-0.25	-13.79	1	8	0	552.608	9	CCOc1ccc2c(c1)sc(n2)N3[C@@H](C(=C(C3=O)O)C(=O)c4cc5ccccc5o4)c6ccc(cc6)OCC=C
ZINC08441730	5.72	-0.43	-16.41	0	8	0	552.608	9	CCOc1ccc2c(c1)sc(n2)N3[C@@H]([C@@H](C(=O)C3=O)C(=O)c4cc5ccccc5o4)c6ccc(cc6)OCC=C
ZINC08441729	6.1	0.16	-16.29	1	7	0	523.614	5	Cc1cc(c2c(c1)sc(n2)N3[C@H](C(=C(C3=O)O)C(=O)c4cc5ccccc5o4)c6ccc(cc6)N(C)C)C
ZINC08441728	6.45	0.06	-12.8	2	0	0	544.511	5	CCOC(=O)C1[C@H](C2=C(CC(CC2=O)(C)C)N=C1C)c3cc(c(s3)C)COc4ccccc4Br
ZINC08441728	6.45	0.13	-16.12	0	5	0	544.511	7	CCOC(=O)C1=C(N=C2CC(CC(=O)C2[C@@H]1c3cc(c(s3)C)COc4ccccc4Br)(C)C)C
ZINC08441728	6.63	-0.82	-13.74	0	5	0	544.511	7	CCOC(=O)C1[C@H](C2C(=NC1=C)CC(CC2=O)(C)C)c3cc(c(s3)C)COc4ccccc4Br
ZINC08441727	6.1	-0.06	-16.9	1	7	0	523.614	5	Cc1cc(c2c(c1)sc(n2)N3[C@@H](C(=C(C3=O)O)C(=O)c4cc5ccccc5o4)c6ccc(cc6)N(C)C)C
ZINC08441726	7.51	0.8	-15.65	1	6	0	522.626	5	Cc1cc(c2c(c1)sc(n2)N3[C@H](C(=C(C3=O)O)C(=O)c4cc5ccccc5o4)c6ccc(cc6)C(C)C)C
ZINC08441726	6.93	0.64	-18.77	0	6	0	522.626	5	Cc1cc(c2c(c1)sc(n2)N3[C@H]([C@@H](C(=O)C3=O)C(=O)c4cc5ccccc5o4)c6ccc(cc6)C(C)C)C
ZINC08441725	7.16	2.74	-9.51	2	0	0	526.048	5	CCOC(=O)C1[C@@H](C2=C(CC(CC2=O)(C)C)N=C1C)c3ccc(c(cc3C)C)COc4ccc(cc4Cl)F
ZINC08441725	7.34	1.56	-10.99	0	5	0	526.048	7	CCOC(=O)C1[C@@H](C2C(=NC1=C)CC(CC2=O)(C)C)c3cc(c(cc3C)C)COc4ccc(cc4Cl)F
ZINC08441725	7.16	2.66	-12.58	0	5	0	526.048	7	CCOC(=O)C1=C(N=C2CC(CC(=O)C2[C@H]1c3cc(c(cc3C)C)COc4ccc(cc4Cl)F)(C)C)C
ZINC08441724	7.51	0.87	-15.6	1	6	0	522.626	5	Cc1cc(c2c(c1)sc(n2)N3[C@@H](C(=C(C3=O)O)C(=O)c4cc5ccccc5o4)c6ccc(cc6)C(C)C)C
ZINC08441724	6.93	0.69	-19.74	0	6	0	522.626	5	Cc1cc(c2c(c1)sc(n2)N3[C@@H]([C@@H](C(=O)C3=O)C(=O)c4cc5ccccc5o4)c6ccc(cc6)C(C)C)C

Supplementary Table S2:
Detailed table of XP Docking using Schrodinger's Glide module

ZINC_ID	xLogP	ApolarDesolvation	PolarDesolvation	H Donors	H Acceptors	Charge	MolecularWeight	RotatableBonds	SMILES
ZINC08441723	7.24	0.65	-20.15	1	8	0	616.695	8	Cc1cc(c2c(c1)sc(n2)N3[C@H](C(=C(C3=O)O)C(=O)c4cc5ccccc5o4)c6ccc(c(c6)OC)OCc7ccccc7)C
ZINC08441723	6.66	0.43	-25.46	0	8	0	616.695	8	Cc1cc(c2c(c1)sc(n2)N3[C@H]([C@H](C(=O)C3=O)C(=O)c4cc5ccccc5o4)c6ccc(c(c6)OC)OCc7ccccc7)C
ZINC08441722	7.16	2.7	-9.73	2	0	0	526.048	5	CCOC(=O)C1[C@H](C2=C(CC(CC2=O)(C)C)N=C1C)c3cc(c(cc3C)C)COc4ccc(cc4Cl)F
ZINC08441722	7.34	1.29	-11.56	0	5	0	526.048	7	CCOC(=O)C1[C@H](C2C(=NC1=C)CC(CC2=O)(C)C)c3cc(c(cc3C)C)COc4ccc(cc4Cl)F
ZINC08441722	7.16	2.54	-13.14	0	5	0	526.048	7	CCOC(=O)C1=C(N=C2CC(CC(=O)C2[C@@H]1c3cc(c(cc3C)C)COc4ccc(cc4Cl)F)(C)C)C
ZINC08441721	7.24	0.66	-20.14	1	8	0	616.695	8	Cc1cc(c2c(c1)sc(n2)N3[C@@H](C(=C(C3=O)O)C(=O)c4cc5ccccc5o4)c6ccc(c(c6)OC)OCc7ccccc7)C
ZINC08441721	6.66	0.45	-25.41	0	8	0	616.695	8	Cc1cc(c2c(c1)sc(n2)N3[C@@H]([C@@H](C(=O)C3=O)C(=O)c4cc5ccccc5o4)c6ccc(c(c6)OC)OCc7ccccc7)C
ZINC08441720	7.08	0.7	-19	1	8	0	582.678	9	CCCCOc1ccc(cc1OC)[C@H]2C(=C(C(=O)N2c3nc4c(cc(cc4s3)C)C)O)C(=O)c5cc6ccccc6o5
ZINC08441720	6.5	0.48	-24.09	0	8	0	582.678	9	CCCCOc1ccc(cc1OC)[C@H]2[C@H](C(=O)C(=O)N2c3nc4c(cc(cc4s3)C)C)C(=O)c5cc6ccccc6o5
ZINC08441719	6.74	0.8	-13.4	2	0	0	514.087	5	CCOC(=O)C1[C@@H](C2=C(CC(CC2=O)(C)C)N=C1C)c3cc(c(s3)C)COc4ccc(cc4C)Cl
ZINC08441719	6.92	-0.1	-12.94	0	5	0	514.087	7	CCOC(=O)C1[C@@H](C2C(=NC1=C)CC(CC2=O)(C)C)c3cc(c(s3)C)COc4ccc(cc4C)Cl
ZINC08441719	6.74	0.96	-15.57	0	5	0	514.087	7	CCOC(=O)C1=C(N=C2CC(CC(=O)C2[C@H]1c3cc(c(s3)C)COc4ccc(cc4C)Cl)(C)C)C
ZINC08441718	7.08	0.71	-18.92	1	8	0	582.678	9	CCCCOc1ccc(cc1OC)[C@@H]2C(=C(C(=O)N2c3nc4c(cc(cc4s3)C)C)O)C(=O)c5cc6ccccc6o5
ZINC08441718	6.5	0.49	-24.1	0	8	0	582.678	9	CCCCOc1ccc(cc1OC)[C@@H]2[C@@H](C(=O)C(=O)N2c3nc4c(cc(cc4s3)C)C)C(=O)c5cc6ccccc6o5
ZINC08441717	6.74	1	-10.45	2	0	0	514.087	5	CCOC(=O)C1[C@H](C2=C(CC(CC2=O)(C)C)N=C1C)c3cc(c(s3)C)COc4ccc(cc4C)Cl
ZINC08441717	6.92	0.12	-11.54	0	5	0	514.087	7	CCOC(=O)C1[C@H](C2C(=NC1=C)CC(CC2=O)(C)C)c3cc(c(s3)C)COc4ccc(cc4C)Cl
ZINC08441717	6.74	1.07	-13.7	0	5	0	514.087	7	CCOC(=O)C1=C(N=C2CC(CC(=O)C2[C@@H]1c3cc(c(s3)C)COc4ccc(cc4C)Cl)(C)C)C
ZINC08441716	7.3	1.01	-18.88	1	8	0	596.705	9	Cc1cc(c2c(c1)sc(n2)N3[C@H](C(=C(C3=O)O)C(=O)c4cc5ccccc5o4)c6ccc(c(c6)OC)OCCC(C)C)C
ZINC08441716	6.71	0.79	-24.02	0	8	0	596.705	9	Cc1cc(c2c(c1)sc(n2)N3[C@H]([C@H](C(=O)C3=O)C(=O)c4cc5ccccc5o4)c6ccc(c(c6)OC)OCCC(C)C)C
ZINC08441715	7.3	1.03	-18.76	1	8	0	596.705	9	Cc1cc(c2c(c1)sc(n2)N3[C@@H](C(=C(C3=O)O)C(=O)c4cc5ccccc5o4)c6ccc(c(c6)OC)OCCC(C)C)C
ZINC08441715	6.71	0.81	-23.96	0	8	0	596.705	9	Cc1cc(c2c(c1)sc(n2)N3[C@@H]([C@@H](C(=O)C3=O)C(=O)c4cc5ccccc5o4)c6ccc(c(c6)OC)OCCC(C)C)C
ZINC08441714	5.46	12.69	-23.99	1	5	0	502.43	6	Cc1c(cc(s1)[C@H]2C3=C(CCCC3=O)NC(=C2C(=O)OC)C)COc4ccccc4Br
ZINC08441714	5.58	-2.33	-12.3	0	5	0	502.43	6	Cc1c(cc(s1)[C@H]2C3C(=O)CCC=C3N=C(C2C(=O)OC)C)COc4ccccc4Br
ZINC08441713	7.65	0.18	-17.09	1	7	0	586.669	7	Cc1cc(c2c(c1)sc(n2)N3[C@H](C(=C(C3=O)O)C(=O)c4cc5ccccc5o4)c6ccc(cc6)OCc7ccccc7)C
ZINC08441713	7.07	0.02	-20.19	0	7	0	586.669	7	Cc1cc(c2c(c1)sc(n2)N3[C@H]([C@@H](C(=O)C3=O)C(=O)c4cc5ccccc5o4)c6ccc(cc6)OCc7ccccc7)C
ZINC08441712	7.65	0.24	-18.04	1	7	0	586.669	7	Cc1cc(c2c(c1)sc(n2)N3[C@@H](C(=C(C3=O)O)C(=O)c4cc5ccccc5o4)c6ccc(cc6)OCc7ccccc7)C
ZINC08441712	7.07	0.07	-22.7	0	7	0	586.669	7	Cc1cc(c2c(c1)sc(n2)N3[C@@H]([C@@H](C(=O)C3=O)C(=O)c4cc5ccccc5o4)c6ccc(cc6)OCc7ccccc7)C
ZINC08441711	5.46	13.21	-31.81	1	5	0	502.43	6	Cc1c(cc(s1)[C@@H]2C3=C(CCCC3=O)NC(=C2C(=O)OC)C)COc4ccccc4Br
ZINC08441711	5.58	-2.43	-15.5	0	5	0	502.43	6	Cc1c(cc(s1)[C@@H]2C3C(=O)CCC=C3N=C(C2C(=O)OC)C)COc4ccccc4Br
ZINC08441710	6.7	0.48	-16.19	1	7	0	536.609	7	Cc1cc(c2c(c1)sc(n2)N3[C@H](C(=C(C3=O)O)C(=O)c4cc5ccccc5o4)c6ccc(cc6)OCC=C)C
ZINC08441710	6.12	0.32	-19.2	0	7	0	536.609	7	Cc1cc(c2c(c1)sc(n2)N3[C@H]([C@@H](C(=O)C3=O)C(=O)c4cc5ccccc5o4)c6ccc(cc6)OCC=C)C
ZINC08441709	6.7	0.56	-16.09	1	7	0	536.609	7	Cc1cc(c2c(c1)sc(n2)N3[C@@H](C(=C(C3=O)O)C(=O)c4cc5ccccc5o4)c6ccc(cc6)OCC=C)C
ZINC08441709	6.12	0.37	-20.13	0	7	0	536.609	7	Cc1cc(c2c(c1)sc(n2)N3[C@@H]([C@@H](C(=O)C3=O)C(=O)c4cc5ccccc5o4)c6ccc(cc6)OCC=C)C
ZINC08441708	5.47	12.64	-23.04	1	5	0	475.969	6	Cc1c(cc(s1)[C@H]2C3=C(CCCC3=O)NC(=C2C(=O)OC)C)COc4ccc(cc4Cl)F
ZINC08441708	5.59	-1	-12.11	0	5	0	475.969	6	Cc1c(cc(s1)[C@H]2C3C(=O)CCC=C3N=C(C2C(=O)OC)C)COc4ccc(cc4Cl)F
ZINC08441707	7.49	0.22	-15.91	1	7	0	552.652	8	CCCCOc1ccc(cc1)[C@H]2C(=C(C(=O)N2c3nc4c(cc(cc4s3)C)C)O)C(=O)c5cc6ccccc6o5
ZINC08441707	6.91	0.06	-18.89	0	7	0	552.652	8	CCCCOc1ccc(cc1)[C@H]2[C@@H](C(=O)C(=O)N2c3nc4c(cc(cc4s3)C)C)C(=O)c5cc6ccccc6o5
ZINC08441706	7.49	0.3	-15.82	1	7	0	552.652	8	CCCCOc1ccc(cc1)[C@@H]2C(=C(C(=O)N2c3nc4c(cc(cc4s3)C)C)O)C(=O)c5cc6ccccc6o5
ZINC08441706	6.91	0.12	-19.83	0	7	0	552.652	8	CCCCOc1ccc(cc1)[C@@H]2[C@@H](C(=O)C(=O)N2c3nc4c(cc(cc4s3)C)C)C(=O)c5cc6ccccc6o5
ZINC08441705	5.47	13.16	-30.56	1	5	0	475.969	6	Cc1c(cc(s1)[C@@H]2C3=C(CCCC3=O)NC(=C2C(=O)OC)C)COc4ccc(cc4Cl)F
ZINC08441705	5.59	-1.12	-15.03	0	5	0	475.969	6	Cc1c(cc(s1)[C@@H]2C3C(=O)CCC=C3N=C(C2C(=O)OC)C)COc4ccc(cc4Cl)F
ZINC00703132	5.63	12.07	-19.17	1	9	0	570.623	7	Cc1cc(c2c(c1)sc(n2)N3[C@H](C(=C(C3=O)O)C(=O)c4cc5ccccc5o4)c6cc(c(c(c6)OC)OC)OC)C
ZINC00703132	5.05	12.84	-22.96	0	9	0	570.623	7	Cc1cc(c2c(c1)sc(n2)N3[C@H]([C@@H](C(=O)C3=O)C(=O)c4cc5ccccc5o4)c6cc(c(c(c6)OC)OC)OC)C
ZINC00703133	5.63	12.09	-19.12	1	9	0	570.623	7	Cc1cc(c2c(c1)sc(n2)N3[C@@H](C(=C(C3=O)O)C(=O)c4cc5ccccc5o4)c6cc(c(c(c6)OC)OC)OC)C
ZINC00703133	5.05	12.92	-23.5	0	9	0	570.623	7	Cc1cc(c2c(c1)sc(n2)N3[C@@H]([C@@H](C(=O)C3=O)C(=O)c4cc5ccccc5o4)c6cc(c(c(c6)OC)OC)OC)C
ZINC08441704	6.83	14.69	-14.49	1	5	0	512.021	6	Cc1cc(c(cc1COc2ccc(cc2Cl)F)[C@H]3C4=C(CC(CC4=O)(C)C)NC(=C3C(=O)OC)C)C
ZINC08441704	6.95	1.66	-10.01	0	5	0	512.021	6	Cc1cc(c(cc1COc2ccc(cc2Cl)F)[C@H]3C4C(=O)CC(C=C4N=C(C3C(=O)OC)C)(C)C)C
ZINC08441703	6.91	0.47	-15.81	1	6	0	508.599	5	CCc1ccc(cc1)[C@H]2C(=C(C(=O)N2c3nc4c(cc(cc4s3)C)C)O)C(=O)c5cc6ccccc6o5
ZINC08441703	6.33	0.32	-18.97	0	6	0	508.599	5	CCc1ccc(cc1)[C@H]2[C@@H](C(=O)C(=O)N2c3nc4c(cc(cc4s3)C)C)C(=O)c5cc6ccccc6o5
ZINC08441702	6.91	0.55	-15.74	1	6	0	508.599	5	CCc1ccc(cc1)[C@@H]2C(=C(C(=O)N2c3nc4c(cc(cc4s3)C)C)O)C(=O)c5cc6ccccc6o5
ZINC08441702	6.33	0.37	-19.84	0	6	0	508.599	5	CCc1ccc(cc1)[C@@H]2[C@@H](C(=O)C(=O)N2c3nc4c(cc(cc4s3)C)C)C(=O)c5cc6ccccc6o5
ZINC08441701	6.83	14.69	-14.4	1	5	0	512.021	6	Cc1cc(c(cc1COc2ccc(cc2Cl)F)[C@@H]3C4=C(CC(CC4=O)(C)C)NC(=C3C(=O)OC)C)C
ZINC00650712	4.62	-0.99	-10.38	0	3	0	438.89	6	Cc1cc(c(cc1COc2ccc(cc2Cl)F)[C@H]3c4c(n[nH]c4OC(=C3C#N)N)C)C
ZINC00703130	5.71	11.39	-18.13	2	8	0	540.597	6	CCOc1cc(ccc1O)[C@H]2C(=C(C(=O)N2c3nc4c(cc(cc4s3)C)C)O)C(=O)c5cc6ccccc6o5
ZINC00703130	5.13	12.22	-23.15	1	8	0	540.597	6	CCOc1cc(ccc1O)[C@H]2[C@H](C(=O)C(=O)N2c3nc4c(cc(cc4s3)C)C)C(=O)c5cc6ccccc6o5
ZINC00650714	4.62	-0.99	-10.34	0	3	0	438.89	6	Cc1cc(c(cc1COc2ccc(cc2Cl)F)[C@@H]3c4c(n[nH]c4OC(=C3C#N)N)C)C
ZINC00703131	5.71	11.4	-18.34	2	8	0	540.597	6	CCOc1cc(ccc1O)[C@@H]2C(=C(C(=O)N2c3nc4c(cc(cc4s3)C)C)O)C(=O)c5cc6ccccc6o5
ZINC00703131	5.13	12.22	-23.4	1	8	0	540.597	6	CCOc1cc(ccc1O)[C@@H]2[C@@H](C(=O)C(=O)N2c3nc4c(cc(cc4s3)C)C)C(=O)c5cc6ccccc6o5
ZINC08441700	7.73	0.71	-16.08	1	7	0	572.642	6	Cc1cc(c2c(c1)sc(n2)N3[C@H](C(=C(C3=O)O)C(=O)c4cc5ccccc5o4)c6cccc(c6)Oc7ccccc7)C
ZINC08441700	7.14	0.52	-19.02	0	7	0	572.642	6	Cc1cc(c2c(c1)sc(n2)N3[C@H]([C@H](C(=O)C3=O)C(=O)c4cc5ccccc5o4)c6cccc(c6)Oc7ccccc7)C
ZINC08441699	7.73	0.63	-16.24	1	7	0	572.642	6	Cc1cc(c2c(c1)sc(n2)N3[C@@H](C(=C(C3=O)O)C(=O)c4cc5ccccc5o4)c6cccc(c6)Oc7ccccc7)C

Supplementary Table S2:
Detailed table of XP Docking using Schrodinger's Glide module

ZINC_ID	xLogP	ApolarDesolvation	PolarDesolvation	H Donors	H Acceptors	Charge	MolecularWeight	RotatableBonds	SMILES
ZINC08441699	7.14	0.47	-18.68	0	7	0	572.642	6	Cc1cc(c2c(c1)sc(n2)N3[C@@H]([C@H](C(=O)C3=O)C(=O)c4cc5ccccc5o4)c6cccc(c6)Oc7ccccc7)C
ZINC08441698	6.46	14.39	-17.45	1	5	0	480.004	6	Cc1cc(c(cc1COc2ccc(cc2C)Cl)[C@H]3C4=C(CCCC4=O)NC(=C3C(=O)OC)C)C
ZINC08441698	6.58	-0.01	-9.68	0	5	0	480.004	6	Cc1cc(c(cc1COc2ccc(cc2C)Cl)[C@H]3C4C(=O)CCC=C4N=C(C3C(=O)OC)C)C
ZINC08441697	6.02	0.5	-19.26	1	8	0	554.624	7	CCOc1ccc(cc1OC)[C@H]2C(=C(C(=O)N2c3nc4c(cc(cc4s3)C)C)O)C(=O)c5cc6ccccc6o5
ZINC08441697	5.44	0.28	-24.34	0	8	0	554.624	7	CCOc1ccc(cc1OC)[C@H]2[C@H](C(=O)C(=O)N2c3nc4c(cc(cc4s3)C)C)C(=O)c5cc6ccccc6o5
ZINC08441696	6.02	0.51	-19.14	1	8	0	554.624	7	CCOc1ccc(cc1OC)[C@@H]2C(=C(C(=O)N2c3nc4c(cc(cc4s3)C)C)O)C(=O)c5cc6ccccc6o5
ZINC08441696	5.44	0.3	-24.38	0	8	0	554.624	7	CCOc1ccc(cc1OC)[C@@H]2[C@@H](C(=O)C(=O)N2c3nc4c(cc(cc4s3)C)C)C(=O)c5cc6ccccc6o5
ZINC08441695	6.46	14.4	-17.5	1	5	0	480.004	6	Cc1cc(c(cc1COc2ccc(cc2C)Cl)[C@@H]3C4=C(CCCC4=O)NC(=C3C(=O)OC)C)C
ZINC08441695	6.58	0.2	-8.78	0	5	0	480.004	6	Cc1cc(c(cc1COc2ccc(cc2C)Cl)[C@@H]3C4C(=O)CCC=C4N=C(C3C(=O)OC)C)C
ZINC00703128	5.52	-1.97	-17.34	2	7	0	496.544	4	Cc1cc(c2c(c1)sc(n2)N3[C@H](C(=C(C3=O)O)C(=O)c4cc5ccccc5o4)c6ccc(cc6)O)C
ZINC00703128	4.93	-2.13	-20.66	1	7	0	496.544	4	Cc1cc(c2c(c1)sc(n2)N3[C@H]([C@@H](C(=O)C3=O)C(=O)c4cc5ccccc5o4)c6ccc(cc6)O)C
ZINC00703129	5.52	-1.9	-16.96	2	7	0	496.544	4	Cc1cc(c2c(c1)sc(n2)N3[C@@H](C(=C(C3=O)O)C(=O)c4cc5ccccc5o4)c6ccc(cc6)O)C
ZINC00703129	4.93	-2.08	-21.17	1	7	0	496.544	4	Cc1cc(c2c(c1)sc(n2)N3[C@@H]([C@@H](C(=O)C3=O)C(=O)c4cc5ccccc5o4)c6ccc(cc6)O)C
ZINC08441694	5.76	13.26	-20.6	1	5	0	472.006	6	Cc1cc(ccc1OCc2cc(sc2C)[C@H]3C4=C(CCCC4=O)NC(=C3C(=O)OC)C)Cl
ZINC08441694	5.88	-1.39	-10.23	0	5	0	472.006	6	Cc1cc(ccc1OCc2cc(sc2C)[C@H]3C4C(=O)CCC=C4N=C(C3C(=O)OC)C)Cl
ZINC00703126	5.9	-1.2	-16.46	1	6	0	486.574	4	Cc1cc(c2c(c1)sc(n2)N3[C@H](C(=C(C3=O)O)C(=O)c4cc5ccccc5o4)c6cccs6)C
ZINC00703126	5.31	-1.39	-19.76	0	6	0	486.574	4	Cc1cc(c2c(c1)sc(n2)N3[C@H]([C@@H](C(=O)C3=O)C(=O)c4cc5ccccc5o4)c6cccs6)C
ZINC00703127	5.9	-1.2	-16.57	1	6	0	486.574	4	Cc1cc(c2c(c1)sc(n2)N3[C@@H](C(=C(C3=O)O)C(=O)c4cc5ccccc5o4)c6cccs6)C
ZINC00703127	5.31	-1.38	-20.98	0	6	0	486.574	4	Cc1cc(c2c(c1)sc(n2)N3[C@@H]([C@@H](C(=O)C3=O)C(=O)c4cc5ccccc5o4)c6cccs6)C
ZINC08441693	5.76	13.78	-27.65	1	5	0	472.006	6	Cc1cc(ccc1OCc2cc(sc2C)[C@@H]3C4=C(CCCC4=O)NC(=C3C(=O)OC)C)Cl
ZINC08441693	5.88	-1.51	-12.7	0	5	0	472.006	6	Cc1cc(ccc1OCc2cc(sc2C)[C@@H]3C4C(=O)CCC=C4N=C(C3C(=O)OC)C)Cl
ZINC08441692	7.28	-0.3	-16.58	1	6	0	549.435	4	Cc1cc(c2c(c1)sc(n2)N3[C@H](C(=C(C3=O)O)C(=O)c4cc5ccccc5o4)c6ccc(c(c6)Cl)Cl)C
ZINC08441692	6.7	-0.49	-21.96	0	6	0	549.435	4	Cc1cc(c2c(c1)sc(n2)N3[C@H]([C@H](C(=O)C3=O)C(=O)c4cc5ccccc5o4)c6ccc(c(c6)Cl)Cl)C
ZINC08441691	6.69	-3.14	-11.62	1	1	0	483.015	5	[H]/N=C/1\[C@H](C2=CCCC[C@@H]2[C@@H](C1(C#N)C#N)c3cc(c(cc3C)C)COc4ccc(cc4C)Cl)C#N
ZINC08441691	6.69	-2.37	-38.16	2	5	1	484.023	4	Cc1cc(c(cc1COc2ccc(cc2C)Cl)[C@H]3[C@H]4CCCC=C4[C@@H](C(=[NH2+])C3(C#N)C#N)C#N)C
ZINC08441690	7.28	-0.21	-16.56	1	6	0	549.435	4	Cc1cc(c2c(c1)sc(n2)N3[C@@H](C(=C(C3=O)O)C(=O)c4cc5ccccc5o4)c6ccc(c(c6)Cl)Cl)C
ZINC08441690	6.7	-0.39	-21.98	0	6	0	549.435	4	Cc1cc(c2c(c1)sc(n2)N3[C@@H]([C@@H](C(=O)C3=O)C(=O)c4cc5ccccc5o4)c6ccc(c(c6)Cl)Cl)C
ZINC08441689	6.69	3.25	-11	1	1	0	483.015	5	[H]/N=C/1\[C@H](C2=CCCC[C@@H]2[C@H](C1(C#N)C#N)c3cc(c(cc3C)C)COc4ccc(cc4C)Cl)C#N
ZINC08441689	6.69	3.08	-41.06	2	5	1	484.023	4	Cc1cc(c(cc1COc2ccc(cc2C)Cl)[C@@H]3[C@H]4CCCC=C4[C@@H](C(=[NH2+])C3(C#N)C#N)C#N)C
ZINC00703124	6.16	0.74	-15.61	1	6	0	498.535	4	Cc1cc(c2c(c1)sc(n2)N3[C@H](C(=C(C3=O)O)C(=O)c4cc5ccccc5o4)c6ccc(cc6)F)C
ZINC00703124	5.58	0.58	-19.18	0	6	0	498.535	4	Cc1cc(c2c(c1)sc(n2)N3[C@H]([C@@H](C(=O)C3=O)C(=O)c4cc5ccccc5o4)c6ccc(cc6)F)C
ZINC08441688	6.69	3.2	-10.91	1	1	0	483.015	5	[H]/N=C/1\[C@@H](C2=CCCC[C@H]2[C@@H](C1(C#N)C#N)c3cc(c(cc3C)C)COc4ccc(cc4C)Cl)C#N
ZINC08441688	6.69	3.03	-40.88	2	5	1	484.023	4	Cc1cc(c(cc1COc2ccc(cc2C)Cl)[C@H]3[C@@H]4CCCC=C4[C@H](C(=[NH2+])C3(C#N)C#N)C#N)C
ZINC00703125	6.16	0.81	-15.51	1	6	0	498.535	4	Cc1cc(c2c(c1)sc(n2)N3[C@@H](C(=C(C3=O)O)C(=O)c4cc5ccccc5o4)c6ccc(cc6)F)C
ZINC00703125	5.58	0.63	-20.07	0	6	0	498.535	4	Cc1cc(c2c(c1)sc(n2)N3[C@@H]([C@@H](C(=O)C3=O)C(=O)c4cc5ccccc5o4)c6ccc(cc6)F)C
ZINC08441687	6.69	3.89	-11.8	1	1	0	483.015	5	[H]/N=C/1\[C@@H](C2=CCCC[C@H]2[C@H](C1(C#N)C#N)c3cc(c(cc3C)C)COc4ccc(cc4C)Cl)C#N
ZINC08441687	6.69	3.69	-32.76	2	5	1	484.023	4	Cc1cc(c(cc1COc2ccc(cc2C)Cl)[C@@H]3[C@@H]4CCCC=C4[C@H](C(=[NH2+])C3(C#N)C#N)C#N)C
ZINC08441686	6.78	-0.7	-16.99	1	6	0	559.441	4	Cc1cc(c2c(c1)sc(n2)N3[C@H](C(=C(C3=O)O)C(=O)c4cc5ccccc5o4)c6cccc(c6)Br)C
ZINC08441686	6.2	-0.9	-22.03	0	6	0	559.441	4	Cc1cc(c2c(c1)sc(n2)N3[C@H]([C@H](C(=O)C3=O)C(=O)c4cc5ccccc5o4)c6cccc(c6)Br)C
ZINC08441685	6.78	-0.78	-14.56	1	6	0	559.441	4	Cc1cc(c2c(c1)sc(n2)N3[C@@H](C(=C(C3=O)O)C(=O)c4cc5ccccc5o4)c6cccc(c6)Br)C
ZINC08441685	6.2	-0.95	-18.15	0	6	0	559.441	4	Cc1cc(c2c(c1)sc(n2)N3[C@@H]([C@@H](C(=O)C3=O)C(=O)c4cc5ccccc5o4)c6cccc(c6)Br)C
ZINC08441684	6.12	14.09	-22.01	1	5	0	530.484	6	Cc1c(cc(s1)[C@H]2C3=C(CC(CC3=O)(C)C)NC(=C2C(=O)OC)C)COc4ccccc4Br
ZINC08441684	6.23	-1.4	-11.96	0	5	0	530.484	6	Cc1c(cc(s1)[C@H]2C3C(=O)CC(C=C3N=C(C2C(=O)OC)C)(C)C)COc4ccccc4Br
ZINC08441683	6.45	0.32	-15.99	1	6	0	494.572	4	Cc1ccc(cc1)[C@H]2C(=C(C(=O)N2c3nc4c(cc(cc4s3)C)C)O)C(=O)c5cc6ccccc6o5
ZINC08441683	5.86	0.16	-19.05	0	6	0	494.572	4	Cc1ccc(cc1)[C@H]2[C@@H](C(=O)C(=O)N2c3nc4c(cc(cc4s3)C)C)C(=O)c5cc6ccccc6o5
ZINC08441682	6.45	0.39	-15.94	1	6	0	494.572	4	Cc1ccc(cc1)[C@@H]2C(=C(C(=O)N2c3nc4c(cc(cc4s3)C)C)O)C(=O)c5cc6ccccc6o5
ZINC08441682	5.86	0.21	-19.97	0	6	0	494.572	4	Cc1ccc(cc1)[C@@H]2[C@@H](C(=O)C(=O)N2c3nc4c(cc(cc4s3)C)C)C(=O)c5cc6ccccc6o5
ZINC08441681	6.12	14.08	-21.99	1	5	0	530.484	6	Cc1c(cc(s1)[C@@H]2C3=C(CC(CC3=O)(C)C)NC(=C2C(=O)OC)C)COc4ccccc4Br
ZINC08441681	6.23	-1.56	-11.35	0	5	0	530.484	6	Cc1c(cc(s1)[C@@H]2C3C(=O)CC(C=C3N=C(C2C(=O)OC)C)(C)C)COc4ccccc4Br
ZINC08441680	6.68	-0.15	-15.25	1	6	0	514.99	4	Cc1cc(c2c(c1)sc(n2)N3[C@H](C(=C(C3=O)O)C(=O)c4cc5ccccc5o4)c6ccc(cc6)Cl)C
ZINC08441680	6.09	-0.31	-18.75	0	6	0	514.99	4	Cc1cc(c2c(c1)sc(n2)N3[C@H]([C@@H](C(=O)C3=O)C(=O)c4cc5ccccc5o4)c6ccc(cc6)Cl)C
ZINC08441679	6.68	-0.08	-15.14	1	6	0	514.99	4	Cc1cc(c2c(c1)sc(n2)N3[C@@H](C(=C(C3=O)O)C(=O)c4cc5ccccc5o4)c6ccc(cc6)Cl)C
ZINC08441679	6.09	-0.26	-19.61	0	6	0	514.99	4	Cc1cc(c2c(c1)sc(n2)N3[C@@H]([C@@H](C(=O)C3=O)C(=O)c4cc5ccccc5o4)c6ccc(cc6)Cl)C
ZINC00703122	6	0.08	-16.06	1	6	0	480.545	4	Cc1cc(c2c(c1)sc(n2)N3[C@H](C(=C(C3=O)O)C(=O)c4cc5ccccc5o4)c6ccccc6)C
ZINC00703122	5.41	-0.09	-19.28	0	6	0	480.545	4	Cc1cc(c2c(c1)sc(n2)N3[C@H]([C@@H](C(=O)C3=O)C(=O)c4cc5ccccc5o4)c6ccccc6)C
ZINC08441678	6.09	0.21	-10.92	1	0	0	486.033	5	CCOC(=O)C1[C@@H](C2=C(CCCC2=O)N=C1C)c3cc(c(s3)C)COc4ccc(cc4C)Cl
ZINC08441678	6.27	-1	-11.88	0	5	0	486.033	7	CCOC(=O)C1[C@@H](C2C(=NC1=C)CCCC2=O)c3cc(c(s3)C)COc4ccc(cc4C)Cl
ZINC08441678	6.09	-0.07	-14.36	0	5	0	486.033	7	CCOC(=O)C1=C(N=C2CCCC(=O)C2[C@H]1c3cc(c(s3)C)COc4ccc(cc4C)Cl)C
ZINC00703123	6	-0.02	-16.14	1	6	0	480.545	4	Cc1cc(c2c(c1)sc(n2)N3[C@@H](C(=C(C3=O)O)C(=O)c4cc5ccccc5o4)c6ccccc6)C
ZINC00703123	5.41	-0.19	-20.26	0	6	0	480.545	4	Cc1cc(c2c(c1)sc(n2)N3[C@@H]([C@@H](C(=O)C3=O)C(=O)c4cc5ccccc5o4)c6ccccc6)C
ZINC08441677	6.09	0.2	-10.79	1	0	0	486.033	5	CCOC(=O)C1[C@H](C2=C(CCCC2=O)N=C1C)c3cc(c(s3)C)COc4ccc(cc4C)Cl

Supplementary Table S2:
Detailed table of XP Docking using Schrodinger's Glide module

ZINC_ID	xLogP	ApolarDesolvation	PolarDesolvation	H Donors	H Acceptors	Charge	MolecularWeight	RotatableBonds	SMILES
ZINC08441677	6.09	-0.07	-14.34	0	5	0	486.033	7	CCOC(=O)C1=C(N=C2CCCC(=O)C2[C@@H]1c3cc(c(s3)C)COc4ccc(cc4C)Cl)C
ZINC08441677	6.27	-1.03	-12.17	0	5	0	486.033	7	CCOC(=O)C1[C@H](C2C(=NC1=C)CCCC2=O)c3cc(c(s3)C)COc4ccc(cc4C)Cl
ZINC00703120	5.12	-2.43	-14.42	2	7	0	482.517	4	Cc1ccc2c(c1)sc(n2)N3[C@H](C(=C(C3=O)O)C(=O)c4cc5ccccc5o4)c6cccc(c6)O
ZINC00703120	4.53	-2.62	-16.2	1	7	0	482.517	4	Cc1ccc2c(c1)sc(n2)N3[C@H]([C@H](C(=O)C3=O)C(=O)c4cc5ccccc5o4)c6cccc(c6)O
ZINC00703121	5.12	-2.35	-14.49	2	7	0	482.517	4	Cc1ccc2c(c1)sc(n2)N3[C@@H](C(=C(C3=O)O)C(=O)c4cc5ccccc5o4)c6cccc(c6)O
ZINC00703121	4.53	-2.53	-16.32	1	7	0	482.517	4	Cc1ccc2c(c1)sc(n2)N3[C@@H]([C@@H](C(=O)C3=O)C(=O)c4cc5ccccc5o4)c6cccc(c6)O
ZINC00703118	5.74	0.38	-14.12	1	6	0	484.508	4	Cc1ccc2c(c1)sc(n2)N3[C@H](C(=C(C3=O)O)C(=O)c4cc5ccccc5o4)c6ccccc6F
ZINC00703118	5.15	0.18	-14.58	0	6	0	484.508	4	Cc1ccc2c(c1)sc(n2)N3[C@H]([C@H](C(=O)C3=O)C(=O)c4cc5ccccc5o4)c6ccccc6F
ZINC00703119	5.74	0.35	-14.09	1	6	0	484.508	4	Cc1ccc2c(c1)sc(n2)N3[C@@H](C(=C(C3=O)O)C(=O)c4cc5ccccc5o4)c6ccccc6F
ZINC00703119	5.15	0.17	-14.26	0	6	0	484.508	4	Cc1ccc2c(c1)sc(n2)N3[C@@H]([C@H](C(=O)C3=O)C(=O)c4cc5ccccc5o4)c6ccccc6F
ZINC00703116	5.68	-0.42	-13.87	1	7	0	496.544	5	Cc1ccc2c(c1)sc(n2)N3[C@H](C(=C(C3=O)O)C(=O)c4cc5ccccc5o4)c6ccc(cc6)OC
ZINC00703116	5.09	-0.58	-15.15	0	7	0	496.544	5	Cc1ccc2c(c1)sc(n2)N3[C@H]([C@@H](C(=O)C3=O)C(=O)c4cc5ccccc5o4)c6ccc(cc6)OC
ZINC08441676	6.79	1.71	-9.33	1	0	0	494.031	5	CCOC(=O)C1[C@@H](C2=C(CCCC2=O)N=C1C)c3cc(c(cc3C)C)COc4ccc(cc4C)Cl
ZINC08441676	6.97	0.43	-11.16	0	5	0	494.031	7	CCOC(=O)C1[C@@H](C2C(=NC1=C)CCCC2=O)c3cc(c(cc3C)C)COc4ccc(cc4C)Cl
ZINC08441676	6.79	1.34	-12.97	0	5	0	494.031	7	CCOC(=O)C1=C(N=C2CCCC(=O)C2[C@H]1c3cc(c(cc3C)C)COc4ccc(cc4C)Cl)C
ZINC00703117	5.68	-0.37	-13.9	1	7	0	496.544	5	Cc1ccc2c(c1)sc(n2)N3[C@@H](C(=C(C3=O)O)C(=O)c4cc5ccccc5o4)c6ccc(cc6)OC
ZINC00703117	5.09	-0.54	-16.16	0	7	0	496.544	5	Cc1ccc2c(c1)sc(n2)N3[C@@H]([C@@H](C(=O)C3=O)C(=O)c4cc5ccccc5o4)c6ccc(cc6)OC
ZINC08441675	6.86	0.24	-17.01	1	8	0	602.668	8	Cc1ccc2c(c1)sc(n2)N3[C@H](C(=C(C3=O)O)C(=O)c4cc5ccccc5o4)c6ccc(c(c6)OC)OCc7ccccc7
ZINC08441675	6.28	0.11	-19.53	0	8	0	602.668	8	Cc1ccc2c(c1)sc(n2)N3[C@H]([C@@H](C(=O)C3=O)C(=O)c4cc5ccccc5o4)c6ccc(c(c6)OC)OCc7ccccc7
ZINC08441674	6.79	1.78	-9.38	1	0	0	494.031	5	CCOC(=O)C1[C@H](C2=C(CCCC2=O)N=C1C)c3cc(c(cc3C)C)COc4ccc(cc4C)Cl
ZINC08441674	6.79	1.58	-12.74	0	5	0	494.031	7	CCOC(=O)C1=C(N=C2CCCC(=O)C2[C@@H]1c3cc(c(cc3C)C)COc4ccc(cc4C)Cl)C
ZINC08441674	6.97	0.71	-11.45	0	5	0	494.031	7	CCOC(=O)C1[C@H](C2C(=NC1=C)CCCC2=O)c3cc(c(cc3C)C)COc4ccc(cc4C)Cl
ZINC08441673	6.86	0.25	-17	1	8	0	602.668	8	Cc1ccc2c(c1)sc(n2)N3[C@@H](C(=C(C3=O)O)C(=O)c4cc5ccccc5o4)c6ccc(c(c6)OC)OCc7ccccc7
ZINC08441673	6.28	0.12	-19.51	0	8	0	602.668	8	Cc1ccc2c(c1)sc(n2)N3[C@@H]([C@H](C(=O)C3=O)C(=O)c4cc5ccccc5o4)c6ccc(c(c6)OC)OCc7ccccc7
ZINC08441672	6.51	2.14	-10.57	1	0	0	497.994	5	CCOC(=O)C1[C@@H](C2=C(CCCC2=O)N=C1C)c3cc(c(cc3C)C)COc4ccc(cc4Cl)F
ZINC08441672	6.51	1.77	-14.03	0	5	0	497.994	7	CCOC(=O)C1=C(N=C2CCCC(=O)C2[C@H]1c3cc(c(cc3C)C)COc4ccc(cc4Cl)F)C
ZINC08441671	6.92	0.6	-15.68	1	8	0	582.678	9	Cc1ccc2c(c1)sc(n2)N3[C@H](C(=C(C3=O)O)C(=O)c4cc5ccccc5o4)c6ccc(c(c6)OC)OCCC(C)C
ZINC08441671	6.34	0.47	-18.16	0	8	0	582.678	9	Cc1ccc2c(c1)sc(n2)N3[C@H]([C@@H](C(=O)C3=O)C(=O)c4cc5ccccc5o4)c6ccc(c(c6)OC)OCCC(C)C
ZINC08441670	6.92	0.62	-15.73	1	8	0	582.678	9	Cc1ccc2c(c1)sc(n2)N3[C@@H](C(=C(C3=O)O)C(=O)c4cc5ccccc5o4)c6ccc(c(c6)OC)OCCC(C)C
ZINC08441670	6.34	0.48	-18.15	0	8	0	582.678	9	Cc1ccc2c(c1)sc(n2)N3[C@@H]([C@H](C(=O)C3=O)C(=O)c4cc5ccccc5o4)c6ccc(c(c6)OC)OCCC(C)C
ZINC08441669	6.51	2.16	-10.61	1	0	0	497.994	5	CCOC(=O)C1[C@H](C2=C(CCCC2=O)N=C1C)c3cc(c(cc3C)C)COc4ccc(cc4Cl)F
ZINC08441669	6.51	1.97	-13.84	0	5	0	497.994	7	CCOC(=O)C1=C(N=C2CCCC(=O)C2[C@@H]1c3cc(c(cc3C)C)COc4ccc(cc4Cl)F)C
ZINC08441669	6.69	1.1	-13.06	0	5	0	497.994	7	CCOC(=O)C1[C@H](C2C(=NC1=C)CCCC2=O)c3cc(c(cc3C)C)COc4ccc(cc4Cl)F
ZINC08441668	6.56	-0.33	-13.46	1	7	0	524.598	7	CCCOc1ccc(cc1)[C@H]2C(=C(C(=O)N2c3nc4ccc(cc4s3)C)O)C(=O)c5cc6ccccc6o5
ZINC08441668	5.97	-0.49	-14.68	0	7	0	524.598	7	CCCOc1ccc(cc1)[C@H]2[C@@H](C(=O)C(=O)N2c3nc4ccc(cc4s3)C)C(=O)c5cc6ccccc6o5
ZINC00881545	4.75	8.67	-24.59	2	7	0	481.961	7	Cc1c(cccc1NC(=S)NC(=O)c2ccc(c(c2)OC)OC)c3nc4cc(ccc4o3)Cl
ZINC08441667	6.56	-0.25	-13.42	1	7	0	524.598	7	CCCOc1ccc(cc1)[C@@H]2C(=C(C(=O)N2c3nc4ccc(cc4s3)C)O)C(=O)c5cc6ccccc6o5
ZINC08441667	5.97	-0.44	-15.68	0	7	0	524.598	7	CCCOc1ccc(cc1)[C@@H]2[C@@H](C(=O)C(=O)N2c3nc4ccc(cc4s3)C)C(=O)c5cc6ccccc6o5
ZINC08441666	6.41	14.19	-19.89	1	5	0	500.06	6	Cc1cc(ccc1OCc2cc(sc2C)[C@H]3C4=C(CC(CC4=O)(C)C)NC(=C3C(=O)OC)C)Cl
ZINC08441666	6.53	-0.46	-9.96	0	5	0	500.06	6	Cc1cc(ccc1OCc2cc(sc2C)[C@H]3C4C(=O)CC(C=C4N=C(C3C(=O)OC)C)(C)C)Cl
ZINC00703115	5.34	10.63	-15.3	2	8	0	526.57	6	CCOc1cc(ccc1O)[C@@H]2C(=C(C(=O)N2c3nc4ccc(cc4s3)C)O)C(=O)c5cc6ccccc6o5
ZINC00703115	4.75	11.43	-17.51	1	8	0	526.57	6	CCOc1cc(ccc1O)[C@@H]2[C@H](C(=O)C(=O)N2c3nc4ccc(cc4s3)C)C(=O)c5cc6ccccc6o5
ZINC00881543	4.37	-2.9	-30.52	2	7	0	467.934	7	COc1ccc(cc1OC)C(=O)NC(=S)Nc2cccc(c2)c3nc4cc(ccc4o3)Cl
ZINC08441665	6.41	14.19	-20	1	5	0	500.06	6	Cc1cc(ccc1OCc2cc(sc2C)[C@@H]3C4=C(CC(CC4=O)(C)C)NC(=C3C(=O)OC)C)Cl
ZINC08441665	6.53	-0.48	-10.07	0	5	0	500.06	6	Cc1cc(ccc1OCc2cc(sc2C)[C@@H]3C4C(=O)CC(C=C4N=C(C3C(=O)OC)C)(C)C)Cl
ZINC00881541	4.73	-1.89	-25.63	2	6	0	463.584	7	Cc1c(cccc1NC(=S)NC(=O)c2ccc(c(c2)OC)OC)c3nc4ccccc4s3
ZINC08441664	7.35	0.34	-14.25	1	7	0	558.615	6	Cc1ccc2c(c1)sc(n2)N3[C@H](C(=C(C3=O)O)C(=O)c4cc5ccccc5o4)c6cccc(c6)Oc7ccccc7
ZINC08441664	6.77	0.11	-15.37	0	7	0	558.615	6	Cc1ccc2c(c1)sc(n2)N3[C@H]([C@H](C(=O)C3=O)C(=O)c4cc5ccccc5o4)c6cccc(c6)Oc7ccccc7
ZINC00881538	4.36	-2.46	-24.04	2	6	0	449.557	7	COc1ccc(cc1OC)C(=O)NC(=S)Nc2cccc(c2)c3nc4ccccc4s3
ZINC08441663	7.35	0.28	-14.47	1	7	0	558.615	6	Cc1ccc2c(c1)sc(n2)N3[C@@H](C(=C(C3=O)O)C(=O)c4cc5ccccc5o4)c6cccc(c6)Oc7ccccc7
ZINC08441663	6.77	0.1	-15.84	0	7	0	558.615	6	Cc1ccc2c(c1)sc(n2)N3[C@@H]([C@@H](C(=O)C3=O)C(=O)c4cc5ccccc5o4)c6cccc(c6)Oc7ccccc7
ZINC00971827	3.45	-3.53	-37.16	2	8	0	468.922	7	COc1ccc(cc1OC)C(=O)NC(=S)Nc2ccc(c(c2)c3nc4c(o3)cccn4)Cl
ZINC00881536	3.19	-2.86	-27.5	2	8	0	448.504	7	Cc1c(cccc1NC(=S)NC(=O)c2ccc(c(c2)OC)OC)c3nc4c(o3)cccn4
ZINC08441662	7.45	2.31	-8.43	2	0	0	522.085	5	CCOC(=O)C1[C@@H](C2=C(CC(CC2=O)(C)C)N=C1C)c3cc(c(cc3C)C)COc4ccc(cc4C)Cl
ZINC08441662	7.45	2.22	-11.46	0	5	0	522.085	7	CCOC(=O)C1=C(N=C2CC(CC(=O)C2[C@H]1c3cc(c(cc3C)C)COc4ccc(cc4C)Cl)(C)C)C
ZINC08441662	7.63	1.12	-9.58	0	5	0	522.085	7	CCOC(=O)C1[C@@H](C2C(=NC1=C)CC(CC2=O)(C)C)c3cc(c(cc3C)C)COc4ccc(cc4C)Cl
ZINC08441661	7.21	0.4	-15.75	1	8	0	582.678	10	CCCCCOc1ccc(cc1OC)[C@H]2C(=C(C(=O)N2c3nc4ccc(cc4s3)C)O)C(=O)c5cc6ccccc6o5
ZINC08441661	6.63	0.28	-18.19	0	8	0	582.678	10	CCCCCOc1ccc(cc1OC)[C@H]2[C@@H](C(=O)C(=O)N2c3nc4ccc(cc4s3)C)C(=O)c5cc6ccccc6o5
ZINC00881534	3.22	-2.91	-36.86	2	8	0	448.504	7	Cc1ccc(cc1NC(=S)NC(=O)c2ccc(c(c2)OC)OC)c3nc4c(o3)cccn4
ZINC00881528	3.65	-2.7	-23.6	2	6	0	433.961	7	COc1ccc(cc1OC)C(=O)NC(=S)Nc2ccc(c(c2)Cl)N3CCCCC3
ZINC08441660	7.21	0.42	-15.79	1	8	0	582.678	10	CCCCCOc1ccc(cc1OC)[C@@H]2C(=C(C(=O)N2c3nc4ccc(cc4s3)C)O)C(=O)c5cc6ccccc6o5

Supplementary Table S2:
Detailed table of XP Docking using Schrodinger's Glide module

ZINC_ID	xLogP	ApolarDesolvation	PolarDesolvation	H Donors	H Acceptors	Charge	MolecularWeight	RotatableBonds	SMILES
ZINC08441660	6.63	0.29	-18.19	0	8	0	582.678	10	CCCCCOc1ccc(cc1OC)[C@@H]2[C@H](C(=O)C(=O)N2c3nc4ccc(cc4s3)C)C(=O)c5cc6ccccc6o5
ZINC00881526	2.58	-4.1	-21.64	2	7	0	435.933	7	COc1ccc(cc1OC)C(=O)NC(=S)Nc2ccc(c(c2)Cl)N3CCOCC3
ZINC00881519	3.75	-2.73	-25.71	2	8	0	463.515	8	COc1cccc(c1)c2nc3cc(ccc3o2)NC(=S)NC(=O)c4ccc(c(c4)OC)OC
ZINC00881512	4.54	-2.43	-31.11	2	7	0	461.543	7	Cc1ccc(c(c1)C)c2nc3cc(ccc3o2)NC(=S)NC(=O)c4ccc(c(c4)OC)OC
ZINC00703112	5.52	11.73	-58.51	0	6	-1	471.539	4	Cc1ccc2c(c1)sc(n2)N3[C@H](C(=C(C3=O)[O-])C(=O)c4cc5ccccc5o4)c6cccs6
ZINC08441659	7.45	2.32	-8.73	2	0	0	522.085	5	CCOC(=O)C1[C@H](C2=C(CC(CC2=O)(C)C)N=C1C)c3cc(c(cc3C)C)COc4ccc(cc4C)Cl
ZINC08441659	7.63	0.9	-10.05	0	5	0	522.085	7	CCOC(=O)C1[C@H](C2C(=NC1=C)CC(CC2=O)(C)C)c3cc(c(cc3C)C)COc4ccc(cc4C)Cl
ZINC08441659	7.45	2.15	-12.06	0	5	0	522.085	7	CCOC(=O)C1=C(N=C2CC(CC(=O)C2[C@@H]1c3cc(c(cc3C)C)COc4ccc(cc4C)Cl)(C)C)C
ZINC00881510	3.77	-2.72	-24.5	2	8	0	463.515	8	COc1ccc(cc1)c2nc3cc(ccc3o2)NC(=S)NC(=O)c4ccc(c(c4)OC)OC
ZINC00881507	4.16	-2.43	-24.04	2	7	0	447.516	7	Cc1ccc(cc1)c2nc3cc(ccc3o2)NC(=S)NC(=O)c4ccc(c(c4)OC)OC
ZINC00703113	5.52	11.74	-58.77	0	6	-1	471.539	4	Cc1ccc2c(c1)sc(n2)N3[C@@H](C(=C(C3=O)[O-])C(=O)c4cc5ccccc5o4)c6cccs6
ZINC00881505	4.14	-2.44	-30.73	2	7	0	447.516	7	Cc1cccc(c1)c2nc3cc(ccc3o2)NC(=S)NC(=O)c4ccc(c(c4)OC)OC
ZINC00881503	4.12	-2.75	-31.36	2	7	0	447.516	7	Cc1ccccc1c2nc3cc(ccc3o2)NC(=S)NC(=O)c4ccc(c(c4)OC)OC
ZINC08441658	6.12	13.57	-22.37	1	5	0	504.023	6	Cc1c(cc(s1)[C@H]2C3=C(CC(CC3=O)(C)C)NC(=C2C(=O)OC)C)COc4ccc(cc4Cl)F
ZINC08441658	6.24	-0.08	-11.7	0	5	0	504.023	6	Cc1c(cc(s1)[C@H]2C3C(=O)CC(C=C3N=C(C2C(=O)OC)C)(C)C)COc4ccc(cc4Cl)F
ZINC00881501	4.39	-2.91	-23	2	7	0	467.934	7	COc1ccc(cc1OC)C(=O)NC(=S)Nc2ccc3c(c2)nc(o3)c4ccc(cc4)Cl
ZINC08441657	6.43	-1.2	-12.38	1	6	0	545.414	4	Cc1ccc2c(c1)sc(n2)N3[C@H](C(=C(C3=O)O)C(=O)c4cc5ccccc5o4)c6ccc(cc6)Br
ZINC08441657	5.85	-1.36	-14	0	6	0	545.414	4	Cc1ccc2c(c1)sc(n2)N3[C@H]([C@@H](C(=O)C3=O)C(=O)c4cc5ccccc5o4)c6ccc(cc6)Br
ZINC00881495	3.13	-0.28	-30.08	2	9	0	477.546	8	Cc1cc2c(cc1NC(=S)NC(=O)c3ccc(c(c3)OC)OC)nn(n2)c4ccc(cc4)OC
ZINC00881493	3.52	0.02	-30.11	2	8	0	461.547	7	Cc1ccc(cc1)n2nc3cc(c(cc3n2)NC(=S)NC(=O)c4ccc(c(c4)OC)OC)C
ZINC08441656	6.43	-1.15	-12.45	1	6	0	545.414	4	Cc1ccc2c(c1)sc(n2)N3[C@@H](C(=C(C3=O)O)C(=O)c4cc5ccccc5o4)c6ccc(cc6)Br
ZINC08441656	5.85	-1.32	-15.07	0	6	0	545.414	4	Cc1ccc2c(c1)sc(n2)N3[C@@H]([C@@H](C(=O)C3=O)C(=O)c4cc5ccccc5o4)c6ccc(cc6)Br
ZINC00881491	3.13	-0.79	-30.25	2	9	0	477.546	9	CCOc1ccc(cc1)n2nc3ccc(cc3n2)NC(=S)NC(=O)c4ccc(c(c4)OC)OC
ZINC08441655	6.12	13.56	-22.46	1	5	0	504.023	6	Cc1c(cc(s1)[C@@H]2C3=C(CC(CC3=O)(C)C)NC(=C2C(=O)OC)C)COc4ccc(cc4Cl)F
ZINC08441655	6.24	-0.09	-11.8	0	5	0	504.023	6	Cc1c(cc(s1)[C@@H]2C3C(=O)CC(C=C3N=C(C2C(=O)OC)C)(C)C)COc4ccc(cc4Cl)F
ZINC08441654	6.91	-0.66	-13.29	1	6	0	535.408	4	Cc1ccc2c(c1)sc(n2)N3[C@H](C(=C(C3=O)O)C(=O)c4cc5ccccc5o4)c6ccc(c(c6)Cl)Cl
ZINC08441654	6.32	-0.83	-15.6	0	6	0	535.408	4	Cc1ccc2c(c1)sc(n2)N3[C@H]([C@@H](C(=O)C3=O)C(=O)c4cc5ccccc5o4)c6ccc(c(c6)Cl)Cl
ZINC00881486	3.15	-0.5	-30.89	2	8	0	447.52	7	Cc1ccc(cc1)n2nc3ccc(cc3n2)NC(=S)NC(=O)c4ccc(c(c4)OC)OC
ZINC00881484	2.7	-0.82	-31.33	2	8	0	433.493	7	COc1ccc(cc1OC)C(=O)NC(=S)Nc2ccc3c(c2)nn(n3)c4ccccc4
ZINC00328721	4.03	-0.13	-18.58	0	2	0	312.438	3	Cc1ccc(cc1C)C(=O)NC(=S)Nc2ccc(c(c2)C)C
ZINC08441653	6.01	10.13	-16	2	5	0	360.889	10	CCCCCCCCC(=O)N/N=C/c1c[nH]nc1c2ccc(cc2)Cl
ZINC08441652	6.91	-0.71	-13.4	1	6	0	535.408	4	Cc1ccc2c(c1)sc(n2)N3[C@@H](C(=C(C3=O)O)C(=O)c4cc5ccccc5o4)c6ccc(c(c6)Cl)Cl
ZINC08441652	6.32	-0.87	-15.78	0	6	0	535.408	4	Cc1ccc2c(c1)sc(n2)N3[C@@H]([C@H](C(=O)C3=O)C(=O)c4cc5ccccc5o4)c6ccc(c(c6)Cl)Cl
ZINC00881458	4.7	-1.61	-28.87	3	5	0	437.952	6	Cc1ccc(cc1C)C(=O)NC(=S)Nc2cccc(c2)NC(=O)c3ccccc3Cl
ZINC00881427	3.57	-2.12	-18.34	2	4	0	425.295	5	CN(C)c1ccc(cc1)NC(=S)NC(=O)c2ccc(cc2)I
ZINC08441651	7.37	-0.21	-17.41	1	2	0	386.515	5	CCCCCCCCCCCC(=O)N/N=C/c1c(c[nH]n1)c2ccc(cc2)F
ZINC00703110	5.62	-0.44	-13.37	1	6	0	466.518	4	Cc1ccc2c(c1)sc(n2)N3[C@H](C(=C(C3=O)O)C(=O)c4cc5ccccc5o4)c6ccccc6
ZINC00703110	5.04	-0.6	-14.6	0	6	0	466.518	4	Cc1ccc2c(c1)sc(n2)N3[C@H]([C@@H](C(=O)C3=O)C(=O)c4cc5ccccc5o4)c6ccccc6
ZINC08441650	3.42	-1.66	-21.66	2	6	0	427.223	5	c1cc(ccc1C(=O)NC(=S)Nc2ccc(cc2)[N+](=O)[O-])I
ZINC06194338	3.8	-1.13	-20.76	2	6	0	441.25	5	Cc1cc(ccc1NC(=S)NC(=O)c2ccc(cc2)I)[N+](=O)[O-]
ZINC00881405	2.89	-1.41	-18.38	3	9	0	463.49	8	Cc1ccc(cc1)C(=O)Nc2ccc(cc2)C(=O)NNC(=O)c3cc(c(c(c3)OC)OC)OC
ZINC07077628	3.24	11.18	-21.99	1	5	0	360.442	4	C/C(=N\NC(=O)c1csc2c1cccc2)/c3ccc(cc3)n4ccnc4
ZINC07077628	3.24	11.66	-51.21	2	5	1	361.45	4	C/C(=N\NC(=O)c1csc2c1cccc2)/c3ccc(cc3)n4cc[nH+]c4
ZINC00703111	5.62	-0.37	-13.3	1	6	0	466.518	4	Cc1ccc2c(c1)sc(n2)N3[C@@H](C(=C(C3=O)O)C(=O)c4cc5ccccc5o4)c6ccccc6
ZINC00703111	5.04	-0.55	-15.49	0	6	0	466.518	4	Cc1ccc2c(c1)sc(n2)N3[C@@H]([C@@H](C(=O)C3=O)C(=O)c4cc5ccccc5o4)c6ccccc6
ZINC08441648	2.58	-1.74	-51.53	1	10	1	463.536	7	Cc1ccc(cc1[N+](=O)[O-])S(=O)(=O)N(CC(=O)N2CC[NH+](CC2)C)c3ccc(cc3)OC
ZINC15952846	3.57	9.76	-60.31	0	7	-1	462.522	7	CCOc1ccc(cc1)[C@H]2C(=C(C(=O)N2C[C@H]3CCCO3)[O-])C(=O)c4ccc5c(c4)C[C@@H](O5)C
ZINC08441645	4.91	-1.59	-18.81	1	9	0	469.519	8	Cc1ccccc1NC(=O)CN(c2ccc(cc2)OC)S(=O)(=O)c3ccc(c(c3)[N+](=O)[O-])C
ZINC00878804	4.44	3	-22.45	1	8	0	418.405	8	C[C@H](C(=O)c1ccccc1)OC(=O)c2ccc(cc2)NC(=O)c3cccc(c3)[N+](=O)[O-]
ZINC15952847	3.57	9.72	-62.76	0	7	-1	462.522	7	CCOc1ccc(cc1)[C@@H]2C(=C(C(=O)N2C[C@H]3CCCO3)[O-])C(=O)c4ccc5c(c4)C[C@@H](O5)C
ZINC00878803	4.44	3	-22.78	1	8	0	418.405	8	C[C@@H](C(=O)c1ccccc1)OC(=O)c2ccc(cc2)NC(=O)c3cccc(c3)[N+](=O)[O-]
ZINC00878801	4.47	2.99	-18.29	1	8	0	418.405	8	C[C@H](C(=O)c1ccccc1)OC(=O)c2ccc(cc2)NC(=O)c3ccc(cc3)[N+](=O)[O-]
ZINC15952844	3.57	9.69	-64.65	0	7	-1	462.522	7	CCOc1ccc(cc1)[C@H]2C(=C(C(=O)N2C[C@@H]3CCCO3)[O-])C(=O)c4ccc5c(c4)C[C@@H](O5)C
ZINC00878799	4.47	2.99	-18.21	1	8	0	418.405	8	C[C@@H](C(=O)c1ccccc1)OC(=O)c2ccc(cc2)NC(=O)c3ccc(cc3)[N+](=O)[O-]
ZINC08441642	4.42	3.95	-12.42	0	9	0	444.399	7	C[C@H](C(=O)c1ccccc1)OC(=O)c2ccc3c(c2)C(=O)N(C3=O)c4ccc(cc4)[N+](=O)[O-]
ZINC08441641	4.42	3.95	-12.5	0	9	0	444.399	7	C[C@@H](C(=O)c1ccccc1)OC(=O)c2ccc3c(c2)C(=O)N(C3=O)c4ccc(cc4)[N+](=O)[O-]
ZINC00971825	4.26	4.3	-20.74	0	9	0	444.399	7	C[C@H](C(=O)c1ccccc1)OC(=O)c2cccc(c2)N3C(=O)c4cccc(c4C3=O)[N+](=O)[O-]
ZINC15952845	3.57	9.25	-66.48	0	7	-1	462.522	7	CCOc1ccc(cc1)[C@@H]2C(=C(C(=O)N2C[C@@H]3CCCO3)[O-])C(=O)c4ccc5c(c4)C[C@@H](O5)C
ZINC00971824	4.26	4.3	-20.91	0	9	0	444.399	7	C[C@@H](C(=O)c1ccccc1)OC(=O)c2cccc(c2)N3C(=O)c4cccc(c4C3=O)[N+](=O)[O-]
ZINC00971823	4.6	3.95	-14.94	0	9	0	444.399	7	C[C@H](C(=O)c1ccccc1)OC(=O)c2ccc3c(c2)C(=O)N(C3=O)c4cccc(c4)[N+](=O)[O-]
ZINC08441639	6.14	3.39	-15.09	2	0	0	538.572	9	CCOC(=O)C1[C@@H](C2=C(CC(CC2=O)(C)C)N=C1C)c3ccc(c(c3)COc4cc(ccc4[N+](=O)[O-])F)OC

Supplementary Table S2:
Detailed table of XP Docking using Schrodinger's Glide module

ZINC_ID	xLogP	ApolarDesolvation	PolarDesolvation	H Donors	H Acceptors	Charge	MolecularWeight	RotatableBonds	SMILES
ZINC08441639	6.14	3.37	-17.75	0	9	0	538.572	9	CCOC(=O)C1=C(N=C2CC(CC(=O)C2[C@H]1c3ccc(c(c3)COc4cc(ccc4[N+](=O)[O-])F)OC)(C)C)C
ZINC08441639	6.33	2.45	-15.42	0	9	0	538.572	9	CCOC(=O)C1[C@@H](C2C(=NC1=C)CC(CC2=O)(C)C)c3ccc(c(c3)COc4cc(ccc4[N+](=O)[O-])F)OC
ZINC00971822	4.6	3.95	-15	0	9	0	444.399	7	C[C@@H](C(=O)c1ccccc1)OC(=O)c2ccc3c(c2)C(=O)N(C3=O)c4cccc(c4)[N+](=O)[O-]
ZINC08441638	3.85	-1.01	-15.94	1	9	0	537.609	11	CCCCCOc1ccc(cc1OC)[C@H]2C(=C(C(=O)N2C[C@H]3CCCO3)O)C(=O)c4ccc5c(c4)OCCO5
ZINC08441638	3.27	-1.17	-19.82	0	9	0	537.609	11	CCCCCOc1ccc(cc1OC)[C@H]2[C@@H](C(=O)C(=O)N2C[C@H]3CCCO3)C(=O)c4ccc5c(c4)OCCO5
ZINC00971821	4.08	4.3	-20.35	0	9	0	444.399	7	C[C@H](C(=O)c1ccccc1)OC(=O)c2ccc(cc2)N3C(=O)c4cccc(c4C3=O)[N+](=O)[O-]
ZINC02998601	4.08	4.3	-20.45	0	9	0	444.399	7	C[C@@H](C(=O)c1ccccc1)OC(=O)c2ccc(cc2)N3C(=O)c4cccc(c4C3=O)[N+](=O)[O-]
ZINC00971818	3.98	4.72	-21.89	0	10	0	488.452	10	COc1ccc(cc1)C(=O)COC(=O)[C@H](Cc2ccccc2)N3C(=O)c4cccc(c4C3=O)[N+](=O)[O-]
ZINC08441637	3.85	-0.83	-17.97	1	9	0	537.609	11	CCCCCOc1ccc(cc1OC)[C@@H]2C(=C(C(=O)N2C[C@H]3CCCO3)O)C(=O)c4ccc5c(c4)OCCO5
ZINC08441637	3.27	-0.93	-23.6	0	9	0	537.609	11	CCCCCOc1ccc(cc1OC)[C@@H]2[C@H](C(=O)C(=O)N2C[C@H]3CCCO3)C(=O)c4ccc5c(c4)OCCO5
ZINC08441636	6.14	3.41	-14.96	2	0	0	538.572	9	CCOC(=O)C1[C@H](C2=C(CC(CC2=O)(C)C)N=C1C)c3ccc(c(c3)COc4cc(ccc4[N+](=O)[O-])F)OC
ZINC08441636	6.33	2.14	-15.2	0	9	0	538.572	9	CCOC(=O)C1[C@H](C2C(=NC1=C)CC(CC2=O)(C)C)c3ccc(c(c3)COc4cc(ccc4[N+](=O)[O-])F)OC
ZINC08441636	6.14	3.04	-17.51	0	9	0	538.572	9	CCOC(=O)C1=C(N=C2CC(CC(=O)C2[C@@H]1c3ccc(c(c3)COc4cc(ccc4[N+](=O)[O-])F)OC)(C)C)C
ZINC00971817	3.98	4.71	-21.85	0	10	0	488.452	10	COc1ccc(cc1)C(=O)COC(=O)[C@@H](Cc2ccccc2)N3C(=O)c4cccc(c4C3=O)[N+](=O)[O-]
ZINC08441635	3.85	-1.04	-19.56	1	9	0	537.609	11	CCCCCOc1ccc(cc1OC)[C@H]2C(=C(C(=O)N2C[C@@H]3CCCO3)O)C(=O)c4ccc5c(c4)OCCO5
ZINC08441635	3.27	-1.21	-25.75	0	9	0	537.609	11	CCCCCOc1ccc(cc1OC)[C@H]2[C@@H](C(=O)C(=O)N2C[C@@H]3CCCO3)C(=O)c4ccc5c(c4)OCCO5
ZINC00971816	4.37	5.01	-20.97	0	9	0	472.453	9	Cc1ccc(cc1)C(=O)COC(=O)[C@H](Cc2ccccc2)N3C(=O)c4cccc(c4C3=O)[N+](=O)[O-]
ZINC00971815	4.37	4.99	-20.91	0	9	0	472.453	9	Cc1ccc(cc1)C(=O)COC(=O)[C@@H](Cc2ccccc2)N3C(=O)c4cccc(c4C3=O)[N+](=O)[O-]
ZINC00971812	3.92	4.67	-21.07	0	9	0	458.426	9	c1ccc(cc1)C[C@H](C(=O)OCC(=O)c2ccccc2)N3C(=O)c4cccc(c4C3=O)[N+](=O)[O-]
ZINC08441634	3.85	-1.35	-20.05	1	9	0	537.609	11	CCCCCOc1ccc(cc1OC)[C@@H]2C(=C(C(=O)N2C[C@@H]3CCCO3)O)C(=O)c4ccc5c(c4)OCCO5
ZINC08441634	3.27	-1.43	-28.34	0	9	0	537.609	11	CCCCCOc1ccc(cc1OC)[C@@H]2[C@@H](C(=O)C(=O)N2C[C@@H]3CCCO3)C(=O)c4ccc5c(c4)OCCO5
ZINC08441633	5.77	3.02	-14.78	2	0	0	524.545	9	CC1=NC2=C([C@@H](C1C(=O)OC)c3ccc(c(c3)COc4cc(ccc4[N+](=O)[O-])F)OC)C(=O)CC(C2)(C)C
ZINC08441633	5.77	3.15	-18.27	0	9	0	524.545	8	CC1=C([C@H]([C@H]2C(=N1)CC(CC2=O)(C)C)c3ccc(c(c3)COc4cc(ccc4[N+](=O)[O-])F)OC)C(=O)OC
ZINC08441633	5.95	3.11	-15.58	0	9	0	524.545	8	CC1(CC2=NC(=C)C([C@H](C2C(=O)C1)c3ccc(c(c3)COc4cc(ccc4[N+](=O)[O-])F)OC)C(=O)OC)C
ZINC08441633	5.95	4.18	-15.27	0	9	0	524.545	8	CC1(CC2=NC(=C)C([C@H]([C@H]2C(=O)C1)c3ccc(c(c3)COc4cc(ccc4[N+](=O)[O-])F)OC)C(=O)OC)C
ZINC00971811	3.92	4.68	-21.09	0	9	0	458.426	9	c1ccc(cc1)C[C@@H](C(=O)OCC(=O)c2ccccc2)N3C(=O)c4cccc(c4C3=O)[N+](=O)[O-]
ZINC00703105	2.16	7.84	-58.06	0	7	-1	426.47	5	c1cc(sc1)[C@H]2C(=C(C(=O)N2C[C@H]3CCCO3)[O-])C(=O)c4ccc5c(c4)OCCO5
ZINC00703105	2.6	-3.4	-25.85	1	7	0	427.478	4	c1cc(sc1)[C@H]2C(=C(c3ccc4c(c3)OCCO4)O)C(=O)C(=O)N2C[C@H]5CCCO5
ZINC00703105	1.58	-3.11	-19.28	0	7	0	427.478	5	c1cc(sc1)[C@H]2[C@H](C(=O)C(=O)N2C[C@H]3CCCO3)C(=O)c4ccc5c(c4)OCCO5
ZINC00878068	4.48	-2.28	-26.76	2	8	0	432.461	6	Cc1ccc2c(c1)nc(o2)c3ccc(cc3)NC(=S)NC(=O)c4ccc(cc4)[N+](=O)[O-]
ZINC02064361	3.76	6.76	-32.42	3	9	0	434.433	6	c1ccc2c(c1)nc(o2)c3cc(ccc3O)NC(=S)NC(=O)c4ccc(cc4)[N+](=O)[O-]
ZINC00878066	4.43	-2.07	-29.99	2	8	0	432.461	6	Cc1ccc(cc1NC(=S)NC(=O)c2ccc(cc2)[N+](=O)[O-])c3nc4ccccc4o3
ZINC00878064	4.83	-1.7	-23.93	2	8	0	446.488	6	Cc1ccc2c(c1)nc(o2)c3cccc(c3C)NC(=S)NC(=O)c4ccc(cc4)[N+](=O)[O-]
ZINC00703106	2.16	7.71	-62.02	0	7	-1	426.47	5	c1cc(sc1)[C@@H]2C(=C(C(=O)N2C[C@H]3CCCO3)[O-])C(=O)c4ccc5c(c4)OCCO5
ZINC00703106	2.6	-2.74	-26.69	1	7	0	427.478	4	c1cc(sc1)[C@@H]2C(=C(c3ccc4c(c3)OCCO4)O)C(=O)C(=O)N2C[C@H]5CCCO5
ZINC00703106	1.58	-2.47	-23.15	0	7	0	427.478	5	c1cc(sc1)[C@@H]2[C@@H](C(=O)C(=O)N2C[C@H]3CCCO3)C(=O)c4ccc5c(c4)OCCO5
ZINC08441632	3.5	-2.71	-27.02	2	9	0	433.449	6	Cc1c(cccc1NC(=S)NC(=O)c2ccc(cc2)[N+](=O)[O-])c3nc4c(o3)cccn4
ZINC00703107	2.16	8.33	-57.27	0	7	-1	426.47	5	c1cc(sc1)[C@H]2C(=C(C(=O)N2C[C@@H]3CCCO3)[O-])C(=O)c4ccc5c(c4)OCCO5
ZINC00703107	2.6	-3.54	-30.49	1	7	0	427.478	4	c1cc(sc1)[C@H]2C(=C(c3ccc4c(c3)OCCO4)O)C(=O)C(=O)N2C[C@@H]5CCCO5
ZINC00703107	1.58	-3.34	-23.22	0	7	0	427.478	5	c1cc(sc1)[C@H]2[C@@H](C(=O)C(=O)N2C[C@@H]3CCCO3)C(=O)c4ccc5c(c4)OCCO5
ZINC00878058	3.83	0.17	-29.51	2	9	0	446.492	6	Cc1ccc(cc1)n2nc3cc(c(cc3n2)NC(=S)NC(=O)c4ccc(cc4)[N+](=O)[O-])C
ZINC00878056	3.39	-0.15	-29.91	2	9	0	432.465	6	Cc1cc2c(cc1NC(=S)NC(=O)c3ccc(cc3)[N+](=O)[O-])nn(n2)c4ccccc4
ZINC00878053	3.01	-0.67	-30.7	2	9	0	418.438	6	c1ccc(cc1)n2nc3ccc(cc3n2)NC(=S)NC(=O)c4ccc(cc4)[N+](=O)[O-]
ZINC02064359	4.06	-2.59	-30.62	2	9	0	448.46	7	COc1cccc(c1)c2nc3cc(ccc3o2)NC(=S)NC(=O)c4ccc(cc4)[N+](=O)[O-]
ZINC00878051	4.19	-1.88	-23.08	2	8	0	436.424	6	c1cc(ccc1c2nc3cc(ccc3o2)NC(=S)NC(=O)c4ccc(cc4)[N+](=O)[O-])F
ZINC08441631	5.77	3.13	-14.88	2	0	0	524.545	9	CC1=NC2=C([C@H](C1C(=O)OC)c3ccc(c(c3)COc4cc(ccc4[N+](=O)[O-])F)OC)C(=O)CC(C2)(C)C
ZINC08441631	5.95	3.19	-15.16	0	9	0	524.545	8	CC1(CC2=NC(=C)C([C@@H](C2C(=O)C1)c3ccc(c(c3)COc4cc(ccc4[N+](=O)[O-])F)OC)C(=O)OC)C
ZINC08441631	5.95	4.35	-17.15	0	9	0	524.545	8	CC1(CC2=NC(=C)C([C@@H]([C@@H]2C(=O)C1)c3ccc(c(c3)COc4cc(ccc4[N+](=O)[O-])F)OC)C(=O)OC)C
ZINC08441631	5.77	3.08	-17.6	0	9	0	524.545	8	CC1=C([C@@H]([C@H]2C(=N1)CC(CC2=O)(C)C)c3ccc(c(c3)COc4cc(ccc4[N+](=O)[O-])F)OC)C(=O)OC
ZINC00703108	2.16	7.45	-59.67	0	7	-1	426.47	5	c1cc(sc1)[C@@H]2C(=C(C(=O)N2C[C@@H]3CCCO3)[O-])C(=O)c4ccc5c(c4)OCCO5
ZINC00703108	2.6	-3.57	-25.94	1	7	0	427.478	4	c1cc(sc1)[C@@H]2C(=C(c3ccc4c(c3)OCCO4)O)C(=O)C(=O)N2C[C@@H]5CCCO5
ZINC00703108	1.58	-3.36	-17.58	0	7	0	427.478	5	c1cc(sc1)[C@@H]2[C@H](C(=O)C(=O)N2C[C@@H]3CCCO3)C(=O)c4ccc5c(c4)OCCO5
ZINC00878047	4.03	-2.61	-24.13	2	8	0	418.434	6	c1ccc(cc1)c2nc3cc(ccc3o2)NC(=S)NC(=O)c4ccc(cc4)[N+](=O)[O-]
ZINC00650002	4.5	-2.97	-9.91	1	3	0	501.768	7	Cc1c2c([nH]n1)OC(=C([C@H]2c3ccc(c(c3)COc4ccc(cc4Cl)Br)OC)C#N)N
ZINC06474972	4.05	-0.66	-24.16	2	8	0	431.429	5	c1ccc2c(c1)C(=O)c3ccc(cc3C2=O)NC(=S)NC(=O)c4ccc(cc4)[N+](=O)[O-]
ZINC08441630	3.04	8.13	-63.76	0	6	-1	424.498	5	C[C@H]1Cc2cc(ccc2O1)C(=O)C3=C(C(=O)N([C@H]3c4cccs4)C[C@@H]5CCCO5)[O-]
ZINC08441630	3.49	-3.23	-30.63	1	6	0	425.506	4	C[C@H]1Cc2cc(ccc2O1)C(=C3[C@@H](N(C(=O)C3=O)C[C@@H]4CCCO4)c5cccs5)O
ZINC08441630	2.46	-3.03	-21.45	0	6	0	425.506	5	C[C@H]1Cc2cc(ccc2O1)C(=O)[C@H]3[C@@H](N(C(=O)C3=O)C[C@@H]4CCCO4)c5cccs5
ZINC00877702	5.06	0.78	-14.21	1	9	0	491.525	8	COc1cc(cc(c1OC)OC)C(=O)Nc2nc(c(s2)c3ccccc3)c4ccc(cc4)[N+](=O)[O-]
ZINC00650005	4.5	-2.98	-9.95	1	3	0	501.768	7	Cc1c2c([nH]n1)OC(=C([C@@H]2c3ccc(c(c3)COc4ccc(cc4Cl)Br)OC)C#N)N
ZINC04089482	2.17	-5.61	-17.06	0	10	0	494.957	5	c1ccc(c(c1)C(=O)N2CCN(CC2)c3ccc(cc3S(=O)(=O)N4CCOCC4)[N+](=O)[O-])Cl

Supplementary Table S2:
Detailed table of XP Docking using Schrodinger's Glide module

ZINC_ID	xLogP	ApolarDesolvation	PolarDesolvation	H Donors	H Acceptors	Charge	MolecularWeight	RotatableBonds	SMILES
ZINC08441629	3.04	8.17	-63.51	0	6	-1	424.498	5	C[C@@H]1Cc2cc(ccc2O1)C(=O)C3=C(C(=O)N([C@H]3c4cccs4)C[C@@H]5CCCO5)[O-]
ZINC08441629	3.49	-2.36	-24.77	1	6	0	425.506	4	C[C@@H]1Cc2cc(ccc2O1)C(=C3[C@@H](N(C(=O)C3=O)C[C@@H]4CCCO4)c5cccs5)O
ZINC08441629	2.46	-2.1	-20.47	0	6	0	425.506	5	C[C@@H]1Cc2cc(ccc2O1)C(=O)[C@@H]3[C@@H](N(C(=O)C3=O)C[C@@H]4CCCO4)c5cccs5
ZINC01019940	3.04	8.58	-44.08	0	6	-1	424.498	5	C[C@H]1Cc2cc(ccc2O1)C(=O)C3=C(C(=O)N([C@@H]3c4cccs4)C[C@@H]5CCCO5)[O-]
ZINC01019940	3.49	-3.1	-30.59	1	6	0	425.506	4	C[C@H]1Cc2cc(ccc2O1)C(=C3[C@H](N(C(=O)C3=O)C[C@@H]4CCCO4)c5cccs5)O
ZINC01019940	2.46	-2.82	-24.58	0	6	0	425.506	5	C[C@H]1Cc2cc(ccc2O1)C(=O)[C@H]3[C@H](N(C(=O)C3=O)C[C@@H]4CCCO4)c5cccs5
ZINC08441627	3.41	-3.13	-13.44	1	7	0	445.317	5	c1cc(ccc1C(=O)N2CCN(CC2)c3ccc(c(c3)NC4CC4)[N+](=O)[O-])Br
ZINC08441626	3.69	-5.09	-20.34	0	8	0	432.889	4	c1cc2c(ccc(c2nc1)[N+](=O)[O-])N3CCN(CC3)S(=O)(=O)c4ccc(cc4)Cl
ZINC08441625	3.04	8.28	-62.92	0	6	-1	424.498	5	C[C@@H]1Cc2cc(ccc2O1)C(=O)C3=C(C(=O)N([C@@H]3c4cccs4)C[C@@H]5CCCO5)[O-]
ZINC08441625	3.49	-3.26	-25.41	1	6	0	425.506	4	C[C@@H]1Cc2cc(ccc2O1)C(=C3[C@H](N(C(=O)C3=O)C[C@@H]4CCCO4)c5cccs5)O
ZINC08441625	2.46	-3.05	-15.99	0	6	0	425.506	5	C[C@@H]1Cc2cc(ccc2O1)C(=O)[C@@H]3[C@H](N(C(=O)C3=O)C[C@@H]4CCCO4)c5cccs5
ZINC00877571	3.38	11.87	-22.84	2	8	0	446.488	7	c1ccc(cc1)[C@H](CC(=O)Nc2nc3ccc(cc3s2)[N+](=O)[O-])NC(=O)c4ccccc4
ZINC08441624	6.31	3.1	-11.69	2	0	0	515.528	6	CCOC(=O)C1[C@@H](C2=C(CCCC2=O)N=C1C)c3ccc(c(c3)COc4cccc(c4)C(F)(F)F)OC
ZINC08441624	6.31	2.9	-15.1	0	6	0	515.528	9	CCOC(=O)C1=C(N=C2CCCC(=O)C2[C@H]1c3ccc(c(c3)COc4cccc(c4)C(F)(F)F)OC)C
ZINC08441624	6.5	1.99	-12.99	0	6	0	515.528	9	CCOC(=O)C1[C@@H](C2C(=NC1=C)CCCC2=O)c3ccc(c(c3)COc4cccc(c4)C(F)(F)F)OC
ZINC08441623	2.3	6.82	-63.21	0	9	-1	480.493	7	COc1ccc(c(c1)OC)[C@H]2C(=C(C(=O)N2C[C@H]3CCCO3)[O-])C(=O)c4ccc5c(c4)OCCO5
ZINC08441623	2.75	-2.29	-31.36	1	9	0	481.501	6	COc1ccc(c(c1)OC)[C@H]2C(=C(c3ccc4c(c3)OCCO4)O)C(=O)C(=O)N2C[C@H]5CCCO5
ZINC08441623	1.72	-2.13	-24.13	0	9	0	481.501	7	COc1ccc(c(c1)OC)[C@H]2[C@H](C(=O)C(=O)N2C[C@H]3CCCO3)C(=O)c4ccc5c(c4)OCCO5
ZINC00877569	3.38	11.87	-22.91	2	8	0	446.488	7	c1ccc(cc1)[C@@H](CC(=O)Nc2nc3ccc(cc3s2)[N+](=O)[O-])NC(=O)c4ccccc4
ZINC02064354	4.68	12.16	-19.43	1	10	0	479.547	7	CCCn1c2ccccc2c3c1nc(nn3)SCC(=O)Nc4nc5ccc(cc5s4)[N+](=O)[O-]
ZINC08441622	4.15	-0.56	-18.08	1	9	0	422.47	7	CCCn1c2ccccc2c3c1nc(nn3)SCC(=O)Nc4ccc(cc4)[N+](=O)[O-]
ZINC08441621	2.3	6.58	-63.86	0	9	-1	480.493	7	COc1ccc(c(c1)OC)[C@@H]2C(=C(C(=O)N2C[C@H]3CCCO3)[O-])C(=O)c4ccc5c(c4)OCCO5
ZINC08441621	2.75	-1.69	-25.28	1	9	0	481.501	6	COc1ccc(c(c1)OC)[C@@H]2C(=C(c3ccc4c(c3)OCCO4)O)C(=O)C(=O)N2C[C@H]5CCCO5
ZINC08441621	1.72	-1.43	-20.54	0	9	0	481.501	7	COc1ccc(c(c1)OC)[C@@H]2[C@@H](C(=O)C(=O)N2C[C@H]3CCCO3)C(=O)c4ccc5c(c4)OCCO5
ZINC08441618	4.77	-1.31	-25.71	2	5	0	420.877	4	c1ccc2c(c1)C(=O)c3ccc(cc3C2=O)NC(=S)NC(=O)c4ccc(cc4)Cl
ZINC08441617	6.31	3.1	-11.74	2	0	0	515.528	6	CCOC(=O)C1[C@H](C2=C(CCCC2=O)N=C1C)c3ccc(c(c3)COc4cccc(c4)C(F)(F)F)OC
ZINC08441617	6.31	2.74	-15.24	0	6	0	515.528	9	CCOC(=O)C1=C(N=C2CCCC(=O)C2[C@@H]1c3ccc(c(c3)COc4cccc(c4)C(F)(F)F)OC)C
ZINC08441617	6.5	1.82	-12.84	0	6	0	515.528	9	CCOC(=O)C1[C@H](C2C(=NC1=C)CCCC2=O)c3ccc(c(c3)COc4cccc(c4)C(F)(F)F)OC
ZINC08441616	2.3	6.75	-66.29	0	9	-1	480.493	7	COc1ccc(c(c1)OC)[C@H]2C(=C(C(=O)N2C[C@@H]3CCCO3)[O-])C(=O)c4ccc5c(c4)OCCO5
ZINC08441616	2.75	-1.81	-29.93	1	9	0	481.501	6	COc1ccc(c(c1)OC)[C@H]2C(=C(c3ccc4c(c3)OCCO4)O)C(=O)C(=O)N2C[C@@H]5CCCO5
ZINC08441616	1.72	-1.61	-21.72	0	9	0	481.501	7	COc1ccc(c(c1)OC)[C@H]2[C@@H](C(=O)C(=O)N2C[C@@H]3CCCO3)C(=O)c4ccc5c(c4)OCCO5
ZINC00331109	3.67	2.43	-15.73	1	6	0	318.304	5	Cc1ccc(c(c1)C)OCC(=O)Nc2cc(ccc2F)[N+](=O)[O-]
ZINC02973109	4.35	1.97	-14.31	0	1	0	332.331	6	CCCCOc1cccc(c1)C(=O)Nc2cc(ccc2F)[N+](=O)[O-]
ZINC08441615	4.36	-2.9	-18.03	1	10	0	499.545	10	Cc1ccc(cc1[N+](=O)[O-])S(=O)(=O)N(CC(=O)NCc2ccccc2OC)c3ccc(cc3)OC
ZINC02064327	4.04	11.81	-34.38	1	8	0	420.45	8	CCOC(=O)c1ccccc1N/C=C(\C#N)/c2nc(cs2)c3cccc(c3)[N+](=O)[O-]
ZINC02064327	4.04	11.81	-25.94	1	8	0	420.45	8	CCOC(=O)c1ccccc1N/C=C(/C#N)\c2nc(cs2)c3cccc(c3)[N+](=O)[O-]
ZINC08441614	2.3	6.09	-68.37	0	9	-1	480.493	7	COc1ccc(c(c1)OC)[C@@H]2C(=C(C(=O)N2C[C@@H]3CCCO3)[O-])C(=O)c4ccc5c(c4)OCCO5
ZINC08441614	2.75	-2.08	-31.52	1	9	0	481.501	6	COc1ccc(c(c1)OC)[C@@H]2C(=C(c3ccc4c(c3)OCCO4)O)C(=O)C(=O)N2C[C@@H]5CCCO5
ZINC08441614	1.72	-1.94	-24.1	0	9	0	481.501	7	COc1ccc(c(c1)OC)[C@@H]2[C@@H](C(=O)C(=O)N2C[C@@H]3CCCO3)C(=O)c4ccc5c(c4)OCCO5
ZINC02064325	4.07	11.81	-26.74	1	8	0	420.45	8	CCOC(=O)c1ccccc1N/C=C(\C#N)/c2nc(cs2)c3ccc(cc3)[N+](=O)[O-]
ZINC02064325	4.07	11.81	-19.3	1	8	0	420.45	8	CCOC(=O)c1ccccc1N/C=C(/C#N)\c2nc(cs2)c3ccc(cc3)[N+](=O)[O-]
ZINC08441613	1.75	15.9	-36.11	0	4	1	438.613	9	Cc1c(sc[n+]1CC(=O)c2ccccc2)CCOC(=O)CC34CC5CC(C3)CC(C5)C4
ZINC15880061	2.71	8.74	-62.23	0	7	-1	434.468	5	Cc1ccc(cc1)[C@H]2C(=C(C(=O)N2C[C@H]3CCCO3)[O-])C(=O)c4ccc5c(c4)OCCO5
ZINC15880062	2.71	8.7	-63.74	0	7	-1	434.468	5	Cc1ccc(cc1)[C@@H]2C(=C(C(=O)N2C[C@H]3CCCO3)[O-])C(=O)c4ccc5c(c4)OCCO5
ZINC08441610	7.13	0.5	-10.38	2	0	0	574.899	6	CC1=NC2=C([C@@H](C1C(=O)OC)c3ccc(c(c3)COc4ccc(cc4Cl)Br)OC)C(=O)CC(C2)(C)C
ZINC08441610	7.32	0.59	-11.07	0	6	0	574.899	7	CC1(CC2=NC(=C)C([C@H](C2C(=O)C1)c3ccc(c(c3)COc4ccc(cc4Cl)Br)OC)C(=O)OC)C
ZINC08441610	7.32	1.81	-10.98	0	6	0	574.899	7	CC1(CC2=NC(=C)C([C@H]([C@H]2C(=O)C1)c3ccc(c(c3)COc4ccc(cc4Cl)Br)OC)C(=O)OC)C
ZINC08441610	7.13	0.71	-13.36	0	6	0	574.899	7	CC1=C([C@H]([C@H]2C(=N1)CC(CC2=O)(C)C)c3ccc(c(c3)COc4ccc(cc4Cl)Br)OC)C(=O)OC
ZINC02064317	1.59	14.02	-40.28	0	4	1	445.358	8	Cc1c(sc[n+]1CC(=O)c2ccc(cc2)Br)CCOC(=O)c3ccccc3
ZINC15880059	2.71	8.67	-66.44	0	7	-1	434.468	5	Cc1ccc(cc1)[C@H]2C(=C(C(=O)N2C[C@@H]3CCCO3)[O-])C(=O)c4ccc5c(c4)OCCO5
ZINC15880060	2.71	8.22	-67.62	0	7	-1	434.468	5	Cc1ccc(cc1)[C@@H]2C(=C(C(=O)N2C[C@@H]3CCCO3)[O-])C(=O)c4ccc5c(c4)OCCO5
ZINC08441605	7.13	0.59	-10.44	2	0	0	574.899	6	CC1=NC2=C([C@H](C1C(=O)OC)c3ccc(c(c3)COc4ccc(cc4Cl)Br)OC)C(=O)CC(C2)(C)C
ZINC08441605	7.32	0.65	-10.91	0	6	0	574.899	7	CC1(CC2=NC(=C)C([C@@H](C2C(=O)C1)c3ccc(c(c3)COc4ccc(cc4Cl)Br)OC)C(=O)OC)C
ZINC08441605	7.32	1.96	-12.23	0	6	0	574.899	7	CC1(CC2=NC(=C)C([C@@H]([C@@H]2C(=O)C1)c3ccc(c(c3)COc4ccc(cc4Cl)Br)OC)C(=O)OC)C
ZINC08441605	7.13	0.53	-13.48	0	6	0	574.899	7	CC1=C([C@@H]([C@H]2C(=N1)CC(CC2=O)(C)C)c3ccc(c(c3)COc4ccc(cc4Cl)Br)OC)C(=O)OC
ZINC08441604	2.99	-5.58	-32.42	0	12	0	498.539	6	Cc1ccc(cc1S(=O)(=O)N2CCCN(CC2)S(=O)(=O)c3cc(ccc3C)[N+](=O)[O-])[N+](=O)[O-]
ZINC08441603	4.54	11.42	-60.24	0	8	-1	502.543	10	CCCCCOc1ccc(cc1)[C@H]2C(=C(C(=O)N2Cc3ccco3)[O-])C(=O)c4ccc5c(c4)OCCO5
ZINC08441603	4.99	0.33	-27.25	1	8	0	503.551	9	CCCCCOc1ccc(cc1)[C@H]2C(=C(c3ccc4c(c3)OCCO4)O)C(=O)C(=O)N2Cc5ccco5
ZINC08441603	3.96	0.61	-21.24	0	8	0	503.551	10	CCCCCOc1ccc(cc1)[C@H]2[C@H](C(=O)C(=O)N2Cc3ccco3)C(=O)c4ccc5c(c4)OCCO5
ZINC08441602	3.13	10.63	-54.84	0	7	-1	460.304	7	COc1ccc(cc1OC)[C@H]2CC(=NN2C(=O)CCC(=O)[O-])c3ccc(cc3)Br
ZINC08441601	4.54	0.65	-14.73	0	0	0	437.492	7	CCOC(=O)C1[C@@H](C2=C(CCCC2=O)N=C1C)c3ccc(o3)COc4ccc(cc4)OC

Supplementary Table S2:
Detailed table of XP Docking using Schrodinger's Glide module

ZINC_ID	xLogP	ApolarDesolvation	PolarDesolvation	H Donors	H Acceptors	Charge	MolecularWeight	RotatableBonds	SMILES
ZINC08441601	4.72	-0.56	-14.15	0	7	0	437.492	8	CCOC(=O)C1[C@@H](C2C(=NC1=C)CCCC2=O)c3ccc(o3)COc4ccc(cc4)OC
ZINC08441601	4.54	0.51	-16.35	0	7	0	437.492	8	CCOC(=O)C1=C(N=C2CCCC(=O)C2[C@H]1c3ccc(o3)COc4ccc(cc4)OC)C
ZINC08441600	3.13	10.63	-54.81	0	7	-1	460.304	7	COc1ccc(cc1OC)[C@@H]2CC(=NN2C(=O)CCC(=O)[O-])c3ccc(cc3)Br
ZINC08441599	4.54	11.05	-61.45	0	8	-1	502.543	10	CCCCCOc1ccc(cc1)[C@@H]2C(=C(C(=O)N2Cc3ccco3)[O-])C(=O)c4ccc5c(c4)OCCO5
ZINC08441599	4.99	0.43	-31.06	1	8	0	503.551	9	CCCCCOc1ccc(cc1)[C@@H]2C(=C(c3ccc4c(c3)OCCO4)O)C(=O)C(=O)N2Cc5ccco5
ZINC08441599	3.96	0.65	-22.59	0	8	0	503.551	10	CCCCCOc1ccc(cc1)[C@@H]2[C@H](C(=O)C(=O)N2Cc3ccco3)C(=O)c4ccc5c(c4)OCCO5
ZINC08441598	4.95	0.59	-13.24	0	6	0	490.357	5	COc1ccccc1[C@H]2CC(=NN2CN3c4ccccc4C(=O)C3=O)c5ccc(cc5)Br
ZINC05918601	5.42	12.87	-58.75	0	7	-1	500.571	10	CCCCCOc1ccc(cc1)[C@H]2C(=C(C(=O)N2Cc3ccco3)[O-])C(=O)c4ccc5c(c4)C[C@H](O5)C
ZINC05918601	5.87	0.23	-29.44	1	7	0	501.579	9	CCCCCOc1ccc(cc1)[C@H]2C(=C(c3ccc4c(c3)C[C@H](O4)C)O)C(=O)C(=O)N2Cc5ccco5
ZINC05918601	4.84	0.48	-24.88	0	7	0	501.579	10	CCCCCOc1ccc(cc1)[C@H]2[C@H](C(=O)C(=O)N2Cc3ccco3)C(=O)c4ccc5c(c4)C[C@H](O5)C
ZINC08441597	4.95	0.5	-13.33	0	6	0	490.357	5	COc1ccccc1[C@@H]2CC(=NN2CN3c4ccccc4C(=O)C3=O)c5ccc(cc5)Br
ZINC08441596	5.11	1.03	-13.72	0	5	0	478.321	4	c1ccc2c(c1)C(=O)C(=O)N2CN3[C@H](CC(=N3)c4ccc(cc4)Br)c5ccc(cc5)F
ZINC08441595	4.54	0.79	-11.94	0	0	0	437.492	7	CCOC(=O)C1[C@H](C2=C(CCCC2=O)N=C1C)c3ccc(o3)COc4ccc(cc4)OC
ZINC08441595	4.54	0.57	-16.72	0	7	0	437.492	8	CCOC(=O)C1=C(N=C2CCCC(=O)C2[C@@H]1c3ccc(o3)COc4ccc(cc4)OC)C
ZINC08441595	4.72	-0.4	-14.65	0	7	0	437.492	8	CCOC(=O)C1[C@H](C2C(=NC1=C)CCCC2=O)c3ccc(o3)COc4ccc(cc4)OC
ZINC05918582	5.42	12.5	-60.12	0	7	-1	500.571	10	CCCCCOc1ccc(cc1)[C@@H]2C(=C(C(=O)N2Cc3ccco3)[O-])C(=O)c4ccc5c(c4)C[C@H](O5)C
ZINC05918582	5.87	0.33	-27.8	1	7	0	501.579	9	CCCCCOc1ccc(cc1)[C@@H]2C(=C(c3ccc4c(c3)C[C@H](O4)C)O)C(=O)C(=O)N2Cc5ccco5
ZINC05918582	4.84	0.55	-20.46	0	7	0	501.579	10	CCCCCOc1ccc(cc1)[C@@H]2[C@H](C(=O)C(=O)N2Cc3ccco3)C(=O)c4ccc5c(c4)C[C@H](O5)C
ZINC08441594	5.11	1.25	-17.71	0	5	0	478.321	4	c1ccc2c(c1)C(=O)C(=O)N2CN3[C@@H](CC(=N3)c4ccc(cc4)Br)c5ccc(cc5)F
ZINC05918598	5.42	12.88	-58.26	0	7	-1	500.571	10	CCCCCOc1ccc(cc1)[C@H]2C(=C(C(=O)N2Cc3ccco3)[O-])C(=O)c4ccc5c(c4)C[C@@H](O5)C
ZINC05918598	5.87	0.7	-26.98	1	7	0	501.579	9	CCCCCOc1ccc(cc1)[C@H]2C(=C(c3ccc4c(c3)C[C@@H](O4)C)O)C(=O)C(=O)N2Cc5ccco5
ZINC05918598	4.84	0.91	-17.91	0	7	0	501.579	10	CCCCCOc1ccc(cc1)[C@H]2[C@@H](C(=O)C(=O)N2Cc3ccco3)C(=O)c4ccc5c(c4)C[C@@H](O5)C
ZINC08441592	5.89	0.12	-14.57	0	5	0	557.217	4	c1cc(ccc1[C@H]2CC(=NN2CN3c4ccc(cc4C(=O)C3=O)Br)c5ccc(cc5)Br)F
ZINC08441591	5.89	0.49	-10.17	0	5	0	557.217	4	c1cc(ccc1[C@@H]2CC(=NN2CN3c4ccc(cc4C(=O)C3=O)Br)c5ccc(cc5)Br)F
ZINC08441590	6.23	0.16	-10.48	2	0	0	526.427	6	CCOC(=O)C1[C@@H](C2=C(CCCC2=O)N=C1C)c3ccc(c(c3)COc4cccc(c4)Br)OC
ZINC08441590	6.23	-0.03	-14.1	0	6	0	526.427	8	CCOC(=O)C1=C(N=C2CCCC(=O)C2[C@H]1c3ccc(c(c3)COc4cccc(c4)Br)OC)C
ZINC08441590	6.41	-0.95	-11.86	0	6	0	526.427	8	CCOC(=O)C1[C@@H](C2C(=NC1=C)CCCC2=O)c3ccc(c(c3)COc4cccc(c4)Br)OC
ZINC05918578	5.42	12.52	-59.81	0	7	-1	500.571	10	CCCCCOc1ccc(cc1)[C@@H]2C(=C(C(=O)N2Cc3ccco3)[O-])C(=O)c4ccc5c(c4)C[C@@H](O5)C
ZINC05918578	5.87	0.3	-27.53	1	7	0	501.579	9	CCCCCOc1ccc(cc1)[C@@H]2C(=C(c3ccc4c(c3)C[C@@H](O4)C)O)C(=O)C(=O)N2Cc5ccco5
ZINC05918578	4.84	0.59	-22.31	0	7	0	501.579	10	CCCCCOc1ccc(cc1)[C@@H]2[C@@H](C(=O)C(=O)N2Cc3ccco3)C(=O)c4ccc5c(c4)C[C@@H](O5)C
ZINC00971750	5.63	15.51	-11.92	1	5	0	519	4	c1ccc(cc1)CC(=O)Nc2ccc(cc2Cl)N3C(=O)[C@H]4[C@H](C3=O)C5c6ccccc6C4c7c5cccc7
ZINC00971749	5.63	15.51	-11.88	1	5	0	519	4	c1ccc(cc1)CC(=O)Nc2ccc(cc2Cl)N3C(=O)[C@@H]4[C@@H](C3=O)C5c6ccccc6C4c7c5cccc7
ZINC00862927	4.16	1.48	-17.25	1	8	0	433.251	6	Cc1c(c(nn1CC(=O)Nc2ccc(cc2C(=O)c3ccccc3)Cl)[N+](=O)[O-])Cl
ZINC00703094	3.78	0.98	-20.57	1	9	0	553.567	9	COc1cc(ccc1OCc2ccccc2)[C@@H]3C(=C(C(=O)N3Cc4ccco4)O)C(=O)c5ccc6c(c5)OCCO6
ZINC00703094	3.2	0.8	-26	0	9	0	553.567	9	COc1cc(ccc1OCc2ccccc2)[C@@H]3[C@H](C(=O)C(=O)N3Cc4ccco4)C(=O)c5ccc6c(c5)OCCO6
ZINC00237625	4.35	8.56	-12.61	1	3	0	289.419	6	Cc1cc(ccc1OC)CCCC(=O)NC2CCCCC2
ZINC00703090	4.66	1.25	-16.54	1	8	0	551.595	9	C[C@@H]1Cc2cc(ccc2O1)C(=O)C3=C(C(=O)N([C@H]3c4ccc(c(c4)OC)OCc5ccccc5)Cc6ccco6)O
ZINC00703090	4.08	1.08	-21.26	0	8	0	551.595	9	C[C@@H]1Cc2cc(ccc2O1)C(=O)[C@H]3[C@@H](N(C(=O)C3=O)Cc4ccco4)c5ccc(c(c5)OC)OCc6ccccc6
ZINC08441586	7.62	-0.97	-13.42	2	5	0	509.407	5	Cc1ccc(cc1)NC(=O)c2cccc(c2)Nc3nc4ccc(cc4c(n3)c5ccccc5)Br
ZINC08441585	7.72	-0.83	-13.09	2	5	0	485.374	5	c1ccc(cc1)c2c3cc(ccc3nc(n2)Nc4cccc(c4)C(=O)Nc5ccc(cc5)Cl)Cl
ZINC06443438	7.06	-0.51	-11.5	2	5	0	523.434	5	Cc1cccc(c1NC(=O)c2ccc(cc2)Nc3nc4ccc(cc4c(n3)c5ccccc5)Br)C
ZINC00703091	4.66	1.35	-18.37	1	8	0	551.595	9	C[C@H]1Cc2cc(ccc2O1)C(=O)C3=C(C(=O)N([C@@H]3c4ccc(c(c4)OC)OCc5ccccc5)Cc6ccco6)O
ZINC00703091	4.08	1.18	-23.73	0	8	0	551.595	9	C[C@H]1Cc2cc(ccc2O1)C(=O)[C@@H]3[C@H](N(C(=O)C3=O)Cc4ccco4)c5ccc(c(c5)OC)OCc6ccccc6
ZINC06195326	8.02	-0.32	-12.08	2	5	0	523.434	5	Cc1ccc(c(c1)C)NC(=O)c2ccc(cc2)Nc3nc4ccc(cc4c(n3)c5ccccc5)Br
ZINC08441584	7.01	0.6	-51	0	6	-1	565.864	6	Cc1c(c(c2cc(ccc2n1)Cl)c3ccccc3)C4=NN([C@H](C4)c5ccc(cc5Cl)Cl)C(=O)CCC(=O)[O-]
ZINC08441583	7.01	0.54	-50.86	0	6	-1	565.864	6	Cc1c(c(c2cc(ccc2n1)Cl)c3ccccc3)C4=NN([C@@H](C4)c5ccc(cc5Cl)Cl)C(=O)CCC(=O)[O-]
ZINC00703092	4.66	0.86	-18.27	1	8	0	551.595	9	C[C@@H]1Cc2cc(ccc2O1)C(=O)C3=C(C(=O)N([C@@H]3c4ccc(c(c4)OC)OCc5ccccc5)Cc6ccco6)O
ZINC00703092	4.08	0.75	-25.91	0	8	0	551.595	9	C[C@@H]1Cc2cc(ccc2O1)C(=O)[C@H]3[C@H](N(C(=O)C3=O)Cc4ccco4)c5ccc(c(c5)OC)OCc6ccccc6
ZINC00857217	2.65	7.53	-15.57	2	6	0	469.666	4	c1cc(cc(c1)I)C(=O)NN[C@@H]2CC(=O)N(C2=O)c3ccc(cc3)Cl
ZINC00857217	2.65	7.71	-13.91	2	6	0	469.666	4	c1cc(cc(c1)I)C(=O)NN[C@H]2CC(=O)N(C2=O)c3ccc(cc3)Cl
ZINC00857215	2.63	7.51	-15.48	2	6	0	469.666	4	c1ccc(c(c1)C(=O)NN[C@@H]2CC(=O)N(C2=O)c3ccc(cc3)Cl)I
ZINC00857215	2.63	7.68	-13.31	2	6	0	469.666	4	c1ccc(c(c1)C(=O)NN[C@H]2CC(=O)N(C2=O)c3ccc(cc3)Cl)I
ZINC05918866	4.51	12.05	-59.04	0	8	-1	516.57	10	CCCCOc1ccc(cc1OC)[C@H]2C(=C(C(=O)N2Cc3ccco3)[O-])C(=O)c4ccc5c(c4)C[C@H](O5)C
ZINC05918866	4.95	0.92	-28.76	1	8	0	517.578	9	CCCCOc1ccc(cc1OC)[C@H]2C(=C(c3ccc4c(c3)C[C@H](O4)C)O)C(=O)C(=O)N2Cc5ccco5
ZINC05918866	3.92	1.13	-19.74	0	8	0	517.578	10	CCCCOc1ccc(cc1OC)[C@H]2[C@@H](C(=O)C(=O)N2Cc3ccco3)C(=O)c4ccc5c(c4)C[C@H](O5)C
ZINC08441582	5.85	-0.11	-10.53	2	0	0	512.4	6	CC1=NC2=C([C@@H](C1C(=O)OC)c3ccc(c(c3)COc4cccc(c4)Br)OC)C(=O)CCC2
ZINC08441582	6.03	1.34	-18.39	0	6	0	512.4	7	COc1ccc(cc1COc2cccc(c2)Br)[C@H]3[C@@H]4C(=NC(=C)C3C(=O)OC)CCCC4=O
ZINC08441582	5.85	-0.07	-14.13	0	6	0	512.4	7	CC1=C([C@H]([C@H]2C(=N1)CCCC2=O)c3ccc(c(c3)COc4cccc(c4)Br)OC)C(=O)OC
ZINC08441582	6.03	0.05	-11.5	0	6	0	512.4	7	COc1ccc(cc1COc2cccc(c2)Br)[C@H]3C4C(=NC(=C)C3C(=O)OC)CCCC4=O
ZINC05918812	4.51	11.6	-60.23	0	8	-1	516.57	10	CCCCOc1ccc(cc1OC)[C@@H]2C(=C(C(=O)N2Cc3ccco3)[O-])C(=O)c4ccc5c(c4)C[C@H](O5)C
ZINC05918812	4.95	0.53	-29.31	1	8	0	517.578	9	CCCCOc1ccc(cc1OC)[C@@H]2C(=C(c3ccc4c(c3)C[C@H](O4)C)O)C(=O)C(=O)N2Cc5ccco5

Supplementary Table S2:
Detailed table of XP Docking using Schrodinger's Glide module

ZINC_ID	xLogP	ApolarDesolvation	PolarDesolvation	H Donors	H Acceptors	Charge	MolecularWeight	RotatableBonds	SMILES
ZINC05918812	3.92	0.79	-24.4	0	8	0	517.578	10	CCCCOc1ccc(cc1OC)[C@@H]2[C@@H](C(=O)C(=O)N2Cc3ccco3)C(=O)c4ccc5c(c4)C[C@H](O5)C
ZINC00857211	2.38	7.06	-15.89	2	6	0	422.666	4	c1cc(cc(c1)Br)C(=O)NN[C@@H]2CC(=O)N(C2=O)c3ccc(cc3)Cl
ZINC00857211	2.38	7.24	-14.16	2	6	0	422.666	4	c1cc(cc(c1)Br)C(=O)NN[C@H]2CC(=O)N(C2=O)c3ccc(cc3)Cl
ZINC00971723	2.36	7.06	-16.5	2	6	0	422.666	4	c1ccc(c(c1)C(=O)NN[C@@H]2CC(=O)N(C2=O)c3ccc(cc3)Cl)Br
ZINC05918863	4.51	12.06	-58.49	0	8	-1	516.57	10	CCCCOc1ccc(cc1OC)[C@H]2C(=C(C(=O)N2Cc3ccco3)[O-])C(=O)c4ccc5c(c4)C[C@@H](O5)C
ZINC05918863	4.95	0.99	-28.76	1	8	0	517.578	9	CCCCOc1ccc(cc1OC)[C@H]2C(=C(c3ccc4c(c3)C[C@@H](O4)C)O)C(=O)C(=O)N2Cc5ccco5
ZINC05918863	3.92	1.26	-21.03	0	8	0	517.578	10	CCCCOc1ccc(cc1OC)[C@H]2[C@H](C(=O)C(=O)N2Cc3ccco3)C(=O)c4ccc5c(c4)C[C@@H](O5)C
ZINC00857173	3.2	10.49	-18.57	2	6	0	478.346	6	c1ccc(cc1)C(c2ccccc2)C(=O)NN[C@@H]3CC(=O)N(C3=O)c4ccc(cc4)Br
ZINC00857173	3.2	10.67	-16.72	2	6	0	478.346	6	c1ccc(cc1)C(c2ccccc2)C(=O)NN[C@H]3CC(=O)N(C3=O)c4ccc(cc4)Br
ZINC05918806	4.51	11.61	-60.08	0	8	-1	516.57	10	CCCCOc1ccc(cc1OC)[C@@H]2C(=C(C(=O)N2Cc3ccco3)[O-])C(=O)c4ccc5c(c4)C[C@@H](O5)C
ZINC05918806	4.95	1.01	-31.68	1	8	0	517.578	9	CCCCOc1ccc(cc1OC)[C@@H]2C(=C(c3ccc4c(c3)C[C@@H](O4)C)O)C(=O)C(=O)N2Cc5ccco5
ZINC05918806	3.92	1.23	-22.27	0	8	0	517.578	10	CCCCOc1ccc(cc1OC)[C@@H]2[C@H](C(=O)C(=O)N2Cc3ccco3)C(=O)c4ccc5c(c4)C[C@@H](O5)C
ZINC08441581	5.85	-0.1	-11.11	2	0	0	512.4	6	CC1=NC2=C([C@H](C1C(=O)OC)c3ccc(c(c3)COc4cccc(c4)Br)OC)C(=O)CCC2
ZINC08441581	5.85	-0.06	-14.68	0	6	0	512.4	7	CC1=C([C@@H]([C@@H]2C(=N1)CCCC2=O)c3ccc(c(c3)COc4cccc(c4)Br)OC)C(=O)OC
ZINC08441581	6.03	1.11	-11.68	0	6	0	512.4	7	COc1ccc(cc1COc2cccc(c2)Br)[C@@H]3[C@H]4C(=NC(=C)C3C(=O)OC)CCCC4=O
ZINC08441581	6.03	0.05	-12.25	0	6	0	512.4	7	COc1ccc(cc1COc2cccc(c2)Br)[C@@H]3C4C(=NC(=C)C3C(=O)OC)CCCC4=O
ZINC00971719	2.76	7.79	-13.28	2	6	0	514.117	4	c1ccc(c(c1)C(=O)NN[C@H]2CC(=O)N(C2=O)c3ccc(cc3)Br)I
ZINC00703087	3.84	1.16	-17.21	1	9	0	533.577	10	CC(C)CCOc1ccc(cc1OC)[C@H]2C(=C(C(=O)N2Cc3ccco3)O)C(=O)c4ccc5c(c4)OCCO5
ZINC00703087	3.26	1	-21.57	0	9	0	533.577	10	CC(C)CCOc1ccc(cc1OC)[C@H]2[C@@H](C(=O)C(=O)N2Cc3ccco3)C(=O)c4ccc5c(c4)OCCO5
ZINC00857151	2.51	7.34	-14.18	2	6	0	467.117	4	c1cc(cc(c1)Br)C(=O)NN[C@H]2CC(=O)N(C2=O)c3ccc(cc3)Br
ZINC00857151	2.51	7.16	-15.86	2	6	0	467.117	4	c1cc(cc(c1)Br)C(=O)NN[C@@H]2CC(=O)N(C2=O)c3ccc(cc3)Br
ZINC00857149	2.49	7.16	-16.51	2	6	0	467.117	4	c1ccc(c(c1)C(=O)NN[C@@H]2CC(=O)N(C2=O)c3ccc(cc3)Br)Br
ZINC08441580	3.84	1.34	-19.02	1	9	0	533.577	10	CC(C)CCOc1ccc(cc1OC)[C@@H]2C(=C(C(=O)N2Cc3ccco3)O)C(=O)c4ccc5c(c4)OCCO5
ZINC08441580	3.26	1.16	-24.45	0	9	0	533.577	10	CC(C)CCOc1ccc(cc1OC)[C@@H]2[C@H](C(=O)C(=O)N2Cc3ccco3)C(=O)c4ccc5c(c4)OCCO5
ZINC00971717	2.38	7.06	-15.94	2	6	0	422.666	4	c1cc(cc(c1)Cl)C(=O)NN[C@@H]2CC(=O)N(C2=O)c3ccc(cc3)Br
ZINC00857147	2.36	7.05	-16.85	2	6	0	422.666	4	c1ccc(c(c1)C(=O)NN[C@@H]2CC(=O)N(C2=O)c3ccc(cc3)Br)Cl
ZINC00857147	2.36	7.22	-14.49	2	6	0	422.666	4	c1ccc(c(c1)C(=O)NN[C@H]2CC(=O)N(C2=O)c3ccc(cc3)Br)Cl
ZINC08441579	6.48	-0.17	-11	2	0	0	546.845	6	CC1=NC2=C([C@@H](C1C(=O)OC)c3ccc(c(c3)COc4ccc(cc4Cl)Br)OC)C(=O)CCC2
ZINC08441579	6.66	0.09	-12.31	0	6	0	546.845	7	COc1ccc(cc1COc2ccc(cc2Cl)Br)[C@H]3C4C(=NC(=C)C3C(=O)OC)CCCC4=O
ZINC08441579	6.48	-0.07	-14.51	0	6	0	546.845	7	CC1=C([C@H]([C@H]2C(=N1)CCCC2=O)c3ccc(c(c3)COc4ccc(cc4Cl)Br)OC)C(=O)OC
ZINC08441579	6.66	1.14	-12.57	0	6	0	546.845	7	COc1ccc(cc1COc2ccc(cc2Cl)Br)[C@H]3[C@@H]4C(=NC(=C)C3C(=O)OC)CCCC4=O
ZINC00703083	4.72	12.42	-58.83	0	8	-1	530.597	10	C[C@H]1Cc2cc(ccc2O1)C(=O)C3=C(C(=O)N([C@H]3c4ccc(c(c4)OC)OCCC(C)C)Cc5ccco5)[O-]
ZINC00703083	5.17	1.3	-28.66	1	8	0	531.605	9	C[C@H]1Cc2cc(ccc2O1)C(=C3[C@@H](N(C(=O)C3=O)Cc4ccco4)c5ccc(c(c5)OC)OCCC(C)C)O
ZINC00703083	4.14	1.57	-20.88	0	8	0	531.605	10	C[C@H]1Cc2cc(ccc2O1)C(=O)[C@@H]3[C@@H](N(C(=O)C3=O)Cc4ccco4)c5ccc(c(c5)OC)OCCC(C)C
ZINC00241845	2.3	-4.1	-11.68	1	5	0	299.373	5	COc1ccc(cc1OC)NS(=O)(=O)c2cccs2
ZINC08441578	6.01	1.57	-28.11	2	10	0	606.631	12	COc1cc(ccc1N/C=C\C(=O)c2ccc3c(c2)OCO3)Cc4ccc(c(c4)OC)N/C=C\C(=O)c5ccc6c(c5)OCO6
ZINC00703084	4.72	12.45	-58.72	0	8	-1	530.597	10	C[C@@H]1Cc2cc(ccc2O1)C(=O)C3=C(C(=O)N([C@H]3c4ccc(c(c4)OC)OCCC(C)C)Cc5ccco5)[O-]
ZINC00703084	5.17	1.24	-28.61	1	8	0	531.605	9	C[C@@H]1Cc2cc(ccc2O1)C(=C3[C@@H](N(C(=O)C3=O)Cc4ccco4)c5ccc(c(c5)OC)OCCC(C)C)O
ZINC00703084	4.14	1.45	-19.66	0	8	0	531.605	10	C[C@@H]1Cc2cc(ccc2O1)C(=O)[C@H]3[C@@H](N(C(=O)C3=O)Cc4ccco4)c5ccc(c(c5)OC)OCCC(C)C
ZINC08441577	6.39	1.5	-51.03	2	3	1	435.591	6	c1ccc(cc1)CC2CC[NH+](CC2)Cc3ccc(cc3)C(=O)Nc4ccc5ccccc5c4
ZINC00703085	4.72	12.05	-60.1	0	8	-1	530.597	10	C[C@H]1Cc2cc(ccc2O1)C(=O)C3=C(C(=O)N([C@@H]3c4ccc(c(c4)OC)OCCC(C)C)Cc5ccco5)[O-]
ZINC00703085	5.17	1.33	-31.55	1	8	0	531.605	9	C[C@H]1Cc2cc(ccc2O1)C(=C3[C@H](N(C(=O)C3=O)Cc4ccco4)c5ccc(c(c5)OC)OCCC(C)C)O
ZINC00703085	4.14	1.54	-22.17	0	8	0	531.605	10	C[C@H]1Cc2cc(ccc2O1)C(=O)[C@@H]3[C@H](N(C(=O)C3=O)Cc4ccco4)c5ccc(c(c5)OC)OCCC(C)C
ZINC08441576	6.48	-0.2	-11.04	2	0	0	546.845	6	CC1=NC2=C([C@H](C1C(=O)OC)c3ccc(c(c3)COc4ccc(cc4Cl)Br)OC)C(=O)CCC2
ZINC08441576	6.66	-0.14	-11.93	0	6	0	546.845	7	COc1ccc(cc1COc2ccc(cc2Cl)Br)[C@@H]3C4C(=NC(=C)C3C(=O)OC)CCCC4=O
ZINC08441576	6.48	-0.27	-14.55	0	6	0	546.845	7	CC1=C([C@@H]([C@H]2C(=N1)CCCC2=O)c3ccc(c(c3)COc4ccc(cc4Cl)Br)OC)C(=O)OC
ZINC08441576	6.66	1.28	-17.68	0	6	0	546.845	7	COc1ccc(cc1COc2ccc(cc2Cl)Br)[C@@H]3[C@H]4C(=NC(=C)C3C(=O)OC)CCCC4=O
ZINC02064253	5.82	1.76	-15.62	0	6	0	472.548	6	COc1cc2c(cc1OC)c(nc3c2c(nn3c4ccccc4)Cc5ccccc5)c6cccnc6
ZINC02064252	6.33	2.92	-16.09	0	8	0	502.53	6	COc1cc2c(cc1OC)c(nc3c2c(nn3c4ccccc4)c5ccccc5)c6ccc(cc6)[N+](=O)[O-]
ZINC00703086	4.72	12.08	-59.64	0	8	-1	530.597	10	C[C@@H]1Cc2cc(ccc2O1)C(=O)C3=C(C(=O)N([C@@H]3c4ccc(c(c4)OC)OCCC(C)C)Cc5ccco5)[O-]
ZINC00703086	5.17	0.84	-29.21	1	8	0	531.605	9	C[C@@H]1Cc2cc(ccc2O1)C(=C3[C@H](N(C(=O)C3=O)Cc4ccco4)c5ccc(c(c5)OC)OCCC(C)C)O
ZINC00703086	4.14	1.1	-24.32	0	8	0	531.605	10	C[C@@H]1Cc2cc(ccc2O1)C(=O)[C@H]3[C@H](N(C(=O)C3=O)Cc4ccco4)c5ccc(c(c5)OC)OCCC(C)C
ZINC00861338	4.77	0.96	-10.97	2	6	0	440.496	7	CCOC(=O)c1c(c(sc1NC(=O)c2ccccc2C)C(=O)Nc3ccccc3F)C
ZINC08441575	4.19	12.37	-61.67	0	8	-1	522.533	8	c1ccc(cc1)COc2ccc(cc2)[C@H]3C(=C(C(=O)N3Cc4ccco4)[O-])C(=O)c5ccc6c(c5)OCCO6
ZINC08441575	4.64	0.16	-29.45	1	8	0	523.541	7	c1ccc(cc1)COc2ccc(cc2)[C@H]3C(=C(c4ccc5c(c4)OCCO5)O)C(=O)C(=O)N3Cc6ccco6
ZINC08441575	3.61	0.45	-23.39	0	8	0	523.541	8	c1ccc(cc1)COc2ccc(cc2)[C@H]3[C@H](C(=O)C(=O)N3Cc4ccco4)C(=O)c5ccc6c(c5)OCCO6
ZINC00181717	2.92	0.29	-12.4	0	5	0	324.38	3	CC1=NC2=C([C@@H](C1C#N)c3ccc(cc3OC)OC)C(=O)CCC2
ZINC00181717	3.1	0.49	-12.46	0	5	0	324.38	3	COc1ccc(c(c1)OC)[C@@H]2C(C(=C)N=C3C2C(=O)CCC3)C#N
ZINC08441574	6.88	0.96	-9.9	2	0	0	554.481	6	CCOC(=O)C1[C@@H](C2=C(CC(CC2=O)(C)C)N=C1C)c3ccc(c(c3)COc4cccc(c4)Br)OC
ZINC08441574	7.06	0.02	-10.82	0	6	0	554.481	8	CCOC(=O)C1[C@@H](C2C(=NC1=C)CC(CC2=O)(C)C)c3ccc(c(c3)COc4cccc(c4)Br)OC
ZINC08441574	6.88	0.94	-12.9	0	6	0	554.481	8	CCOC(=O)C1=C(N=C2CC(CC(=O)C2[C@H]1c3ccc(c(c3)COc4cccc(c4)Br)OC)(C)C)C

Supplementary Table S2:
Detailed table of XP Docking using Schrodinger's Glide module

ZINC_ID	xLogP	ApolarDesolvation	PolarDesolvation	H Donors	H Acceptors	Charge	MolecularWeight	RotatableBonds	SMILES
ZINC04112600	5.03	2.12	-13.36	0	8	0	521.61	10	CC1=NC2=C([C@@H](C1C(=O)OCCOc3ccccc3)c4ccc(c(c4OC)OC)OC)C(=O)CC(C2)(C)C
ZINC04112600	5.21	2.51	-14.1	0	8	0	521.61	10	CC1(CC2=NC(=C)C([C@H](C2C(=O)C1)c3ccc(c(c3OC)OC)OC)C(=O)OCCOc4ccccc4)C
ZINC04112600	5.21	3.4	-15.23	0	8	0	521.61	10	CC1(CC2=NC(=C)C([C@H]([C@H]2C(=O)C1)c3ccc(c(c3OC)OC)OC)C(=O)OCCOc4ccccc4)C
ZINC04112600	5.03	2.39	-16.31	0	8	0	521.61	10	CC1=C([C@H]([C@@H]2C(=N1)CC(CC2=O)(C)C)c3ccc(c(c3OC)OC)OC)C(=O)OCCOc4ccccc4
ZINC08441573	4.04	10.89	-60.38	0	8	-1	488.516	9	CCCCOc1ccc(cc1)[C@H]2C(=C(C(=O)N2Cc3ccco3)[O-])C(=O)c4ccc5c(c4)OCCO5
ZINC08441573	4.48	0.12	-27.63	1	8	0	489.524	8	CCCCOc1ccc(cc1)[C@H]2C(=C(c3ccc4c(c3)OCCO4)O)C(=O)C(=O)N2Cc5ccco5
ZINC08441573	3.45	0.35	-19.94	0	8	0	489.524	9	CCCCOc1ccc(cc1)[C@H]2[C@@H](C(=O)C(=O)N2Cc3ccco3)C(=O)c4ccc5c(c4)OCCO5
ZINC04112599	5.03	2.14	-11.67	0	8	0	521.61	10	CC1=NC2=C([C@H](C1C(=O)OCCOc3ccccc3)c4ccc(c(c4OC)OC)OC)C(=O)CC(C2)(C)C
ZINC04112599	5.21	2.21	-13.2	0	8	0	521.61	10	CC1(CC2=NC(=C)C([C@@H](C2C(=O)C1)c3ccc(c(c3OC)OC)OC)C(=O)OCCOc4ccccc4)C
ZINC04112599	5.21	3.44	-19.82	0	8	0	521.61	10	CC1(CC2=NC(=C)C([C@@H]([C@@H]2C(=O)C1)c3ccc(c(c3OC)OC)OC)C(=O)OCCOc4ccccc4)C
ZINC04112599	5.03	2.14	-15.83	0	8	0	521.61	10	CC1=C([C@@H]([C@H]2C(=N1)CC(CC2=O)(C)C)c3ccc(c(c3OC)OC)OC)C(=O)OCCOc4ccccc4
ZINC00181552	3	0.46	-12.61	0	5	0	324.38	3	CC1=NC2=C([C@@H](C1C#N)c3ccc(c(c3)OC)OC)C(=O)CCC2
ZINC00181552	3.18	0.64	-12.71	0	5	0	324.38	3	COc1ccc(cc1OC)[C@@H]2C(C(=C)N=C3C2C(=O)CCC3)C#N
ZINC00181552	3.18	1.9	-15.28	0	5	0	324.38	3	COc1ccc(cc1OC)[C@@H]2C(C(=C)N=C3[C@@H]2C(=O)CCC3)C#N
ZINC08441572	4.04	11.08	-57.71	0	8	-1	488.516	9	CCCCOc1ccc(cc1)[C@@H]2C(=C(C(=O)N2Cc3ccco3)[O-])C(=O)c4ccc5c(c4)OCCO5
ZINC08441572	4.48	-0.17	-28.23	1	8	0	489.524	8	CCCCOc1ccc(cc1)[C@@H]2C(=C(c3ccc4c(c3)OCCO4)O)C(=O)C(=O)N2Cc5ccco5
ZINC08441572	3.45	0.12	-24.81	0	8	0	489.524	9	CCCCOc1ccc(cc1)[C@@H]2[C@@H](C(=O)C(=O)N2Cc3ccco3)C(=O)c4ccc5c(c4)OCCO5
ZINC08441571	5.6	0.28	-10.29	0	5	0	483.337	4	COc1ccc(cc1OC)[C@H]2N3[C@@H](CC(=N3)c4ccc(cc4)F)c5cc(ccc5O2)Br
ZINC08441570	5.6	0.74	-9.14	0	5	0	483.337	4	COc1ccc(cc1OC)[C@@H]2N3[C@@H](CC(=N3)c4ccc(cc4)F)c5cc(ccc5O2)Br
ZINC08441569	6.88	0.98	-10.46	2	0	0	554.481	6	CCOC(=O)C1[C@H](C2=C(CC(CC2=O)(C)C)N=C1C)c3ccc(c(c3)COc4cccc(c4)Br)OC
ZINC08441569	7.06	-0.29	-11.11	0	6	0	554.481	8	CCOC(=O)C1[C@H](C2C(=NC1=C)CC(CC2=O)(C)C)c3ccc(c(c3)COc4cccc(c4)Br)OC
ZINC08441569	6.88	0.61	-13.29	0	6	0	554.481	8	CCOC(=O)C1=C(N=C2CC(CC(=O)C2[C@@H]1c3ccc(c(c3)COc4cccc(c4)Br)OC)(C)C)C
ZINC07060061	6.01	-0.04	-8.2	0	4	0	453.311	3	COc1ccc(cc1)[C@H]2N3[C@@H](CC(=N3)c4ccc(cc4)F)c5cc(ccc5O2)Br
ZINC15885016	2.17	7.86	-61.98	0	10	-1	506.487	8	COc1cc(cc(c1OC)OC)[C@H]2C(=C(C(=O)N2Cc3ccco3)[O-])C(=O)c4ccc5c(c4)OCCO5
ZINC15885018	2.17	7.63	-60.56	0	10	-1	506.487	8	COc1cc(cc(c1OC)OC)[C@@H]2C(=C(C(=O)N2Cc3ccco3)[O-])C(=O)c4ccc5c(c4)OCCO5
ZINC08441566	6.11	0.67	-7.46	0	3	0	441.275	2	c1cc(ccc1[C@H]2N3[C@@H](CC(=N3)c4ccc(cc4)F)c5cc(ccc5O2)Br)F
ZINC08441565	6.11	1.13	-6	0	3	0	441.275	2	c1cc(ccc1[C@@H]2N3[C@@H](CC(=N3)c4ccc(cc4)F)c5cc(ccc5O2)Br)F
ZINC08441564	6.86	0.07	-10.96	2	0	0	560.872	6	CCOC(=O)C1[C@@H](C2=C(CCCC2=O)N=C1C)c3ccc(c(c3)COc4ccc(cc4Cl)Br)OC
ZINC08441564	6.86	-0.12	-14.32	0	6	0	560.872	8	CCOC(=O)C1=C(N=C2CCCC(=O)C2[C@H]1c3ccc(c(c3)COc4ccc(cc4Cl)Br)OC)C
ZINC08441564	7.04	-1.04	-12.23	0	6	0	560.872	8	CCOC(=O)C1[C@@H](C2C(=NC1=C)CCCC2=O)c3ccc(c(c3)COc4ccc(cc4Cl)Br)OC
ZINC08441563	7.1	0.23	-6.12	0	3	0	447.724	2	c1cc(ccc1C2=NN3[C@H](C2)c4cc(ccc4O[C@@H]3c5ccc(cc5Cl)Cl)Cl)F
ZINC00703077	3.05	10.05	-55.55	0	9	-1	504.515	8	C[C@H]1Cc2cc(ccc2O1)C(=O)C3=C(C(=O)N([C@H]3c4cc(c(c(c4)OC)OC)OC)Cc5ccco5)[O-]
ZINC00703077	3.5	0.6	-32.28	1	9	0	505.523	7	C[C@H]1Cc2cc(ccc2O1)C(=C3[C@@H](N(C(=O)C3=O)Cc4ccco4)c5cc(c(c(c5)OC)OC)OC)O
ZINC00703077	2.47	0.84	-26.81	0	9	0	505.523	8	C[C@H]1Cc2cc(ccc2O1)C(=O)[C@H]3[C@@H](N(C(=O)C3=O)Cc4ccco4)c5cc(c(c(c5)OC)OC)OC
ZINC08441562	7.1	0.1	-5.84	0	3	0	447.724	2	c1cc(ccc1C2=NN3[C@@H](C2)c4cc(ccc4O[C@@H]3c5ccc(cc5Cl)Cl)Cl)F
ZINC08441561	7.34	-0.97	-9.69	0	3	0	451.378	2	c1ccc2cc(ccc2c1)C3=NN4[C@H](C3)c5cc(cc(c5O[C@@H]4c6cccs6)Cl)Cl
ZINC00703078	3.05	10.06	-58.07	0	9	-1	504.515	8	C[C@@H]1Cc2cc(ccc2O1)C(=O)C3=C(C(=O)N([C@H]3c4cc(c(c(c4)OC)OC)OC)Cc5ccco5)[O-]
ZINC00703078	3.5	1.06	-29.62	1	9	0	505.523	7	C[C@@H]1Cc2cc(ccc2O1)C(=C3[C@@H](N(C(=O)C3=O)Cc4ccco4)c5cc(c(c(c5)OC)OC)OC)O
ZINC00703078	2.47	1.29	-21.83	0	9	0	505.523	8	C[C@@H]1Cc2cc(ccc2O1)C(=O)[C@H]3[C@@H](N(C(=O)C3=O)Cc4ccco4)c5cc(c(c(c5)OC)OC)OC
ZINC08441560	7.34	-0.91	-8.76	0	3	0	451.378	2	c1ccc2cc(ccc2c1)C3=NN4[C@@H](C3)c5cc(cc(c5O[C@@H]4c6cccs6)Cl)Cl
ZINC08441559	6.86	0.08	-10.96	2	0	0	560.872	6	CCOC(=O)C1[C@H](C2=C(CCCC2=O)N=C1C)c3ccc(c(c3)COc4ccc(cc4Cl)Br)OC
ZINC08441559	6.86	-0.12	-14.45	0	6	0	560.872	8	CCOC(=O)C1=C(N=C2CCCC(=O)C2[C@@H]1c3ccc(c(c3)COc4ccc(cc4Cl)Br)OC)C
ZINC08441559	7.04	-1.03	-12.32	0	6	0	560.872	8	CCOC(=O)C1[C@H](C2C(=NC1=C)CCCC2=O)c3ccc(c(c3)COc4ccc(cc4Cl)Br)OC
ZINC08441558	6.16	-0.94	-10.21	0	4	0	446.337	2	c1ccc2cc(ccc2c1)C3=NN4[C@H](C3)c5cc(cc(c5O[C@@H]4c6ccncc6)Cl)Cl
ZINC00703079	3.05	9.98	-54.5	0	9	-1	504.515	8	C[C@H]1Cc2cc(ccc2O1)C(=O)C3=C(C(=O)N([C@@H]3c4cc(c(c(c4)OC)OC)OC)Cc5ccco5)[O-]
ZINC00703079	3.5	0.69	-29.01	1	9	0	505.523	7	C[C@H]1Cc2cc(ccc2O1)C(=C3[C@H](N(C(=O)C3=O)Cc4ccco4)c5cc(c(c(c5)OC)OC)OC)O
ZINC00703079	2.47	0.93	-22.88	0	9	0	505.523	8	C[C@H]1Cc2cc(ccc2O1)C(=O)[C@@H]3[C@H](N(C(=O)C3=O)Cc4ccco4)c5cc(c(c(c5)OC)OC)OC
ZINC08441557	6.16	-0.52	-8.19	0	4	0	446.337	2	c1ccc2cc(ccc2c1)C3=NN4[C@@H](C3)c5cc(cc(c5O[C@@H]4c6ccncc6)Cl)Cl
ZINC06475516	5.68	11.98	-9.32	0	4	0	456.343	2	c1ccc2cc(ccc2c1)C3=NN4[C@H](C3)c5cc(ccc5O[C@@H]4c6ccncc6)Br
ZINC06475513	5.68	12.18	-7.92	0	4	0	456.343	2	c1ccc2cc(ccc2c1)C3=NN4[C@@H](C3)c5cc(ccc5O[C@@H]4c6ccncc6)Br
ZINC08441556	8.22	-0.48	-6.55	0	3	0	548.278	2	C[C@]1(N2[C@@H](CC(=N2)c3ccc4ccccc4c3)c5cc(ccc5O1)Br)c6ccc(cc6)Br
ZINC08441555	8.22	-0.82	-7.11	0	3	0	548.278	2	C[C@@]1(N2[C@@H](CC(=N2)c3ccc4ccccc4c3)c5cc(ccc5O1)Br)c6ccc(cc6)Br
ZINC00703080	3.05	10.02	-58.5	0	9	-1	504.515	8	C[C@@H]1Cc2cc(ccc2O1)C(=O)C3=C(C(=O)N([C@@H]3c4cc(c(c(c4)OC)OC)OC)Cc5ccco5)[O-]
ZINC00703080	3.5	1.18	-32.94	1	9	0	505.523	7	C[C@@H]1Cc2cc(ccc2O1)C(=C3[C@H](N(C(=O)C3=O)Cc4ccco4)c5cc(c(c(c5)OC)OC)OC)O
ZINC00703080	2.47	1.4	-23.73	0	9	0	505.523	8	C[C@@H]1Cc2cc(ccc2O1)C(=O)[C@@H]3[C@H](N(C(=O)C3=O)Cc4ccco4)c5cc(c(c(c5)OC)OC)OC
ZINC08441554	6.5	0.59	-10.45	2	0	0	540.454	6	CC1=NC2=C([C@@H](C1C(=O)OC)c3ccc(c(c3)COc4cccc(c4)Br)OC)C(=O)CC(C2)(C)C
ZINC08441554	6.69	1.75	-10.92	0	6	0	540.454	7	CC1(CC2=NC(=C)C([C@H]([C@H]2C(=O)C1)c3ccc(c(c3)COc4cccc(c4)Br)OC)C(=O)OC)C
ZINC08441554	6.69	0.68	-11.28	0	6	0	540.454	7	CC1(CC2=NC(=C)C([C@H](C2C(=O)C1)c3ccc(c(c3)COc4cccc(c4)Br)OC)C(=O)OC)C
ZINC08441554	6.5	0.72	-13.7	0	6	0	540.454	7	CC1=C([C@H]([C@H]2C(=N1)CC(CC2=O)(C)C)c3ccc(c(c3)COc4cccc(c4)Br)OC)C(=O)OC
ZINC15885021	1.88	5.87	-61.77	1	9	-1	462.434	6	COc1cc(ccc1O)[C@H]2C(=C(C(=O)N2Cc3ccco3)[O-])C(=O)c4ccc5c(c4)OCCO5
ZINC08441553	4.45	1.38	-23	0	7	0	514.401	6	CC\1=NN(C(=O)/C1=C\c2cc(c(c(c2)OC)OC)OC)c3nc(cs3)c4ccc(cc4)Br

Supplementary Table S2:
Detailed table of XP Docking using Schrodinger's Glide module

ZINC_ID	xLogP	ApolarDesolvation	PolarDesolvation	H Donors	H Acceptors	Charge	MolecularWeight	RotatableBonds	SMILES
ZINC15885023	1.88	5.56	-60.77	1	9	-1	462.434	6	COc1cc(ccc1O)[C@@H]2C(=C(C(=O)N2Cc3ccco3)[O-])C(=O)c4ccc5c(c4)OCCO5
ZINC00863328	3.75	1.16	-17.9	0	5	0	459.365	3	CN(C)c1ccc(cc1)[C@H]2N(CCS2)C(=O)c3cc4cc(ccc4oc3=O)Br
ZINC00863329	3.75	1.06	-17.75	0	5	0	459.365	3	CN(C)c1ccc(cc1)[C@@H]2N(CCS2)C(=O)c3cc4cc(ccc4oc3=O)Br
ZINC00971705	4.62	0.34	-20.32	1	4	0	451.114	3	C[C@H](c1ccccc1)NC(=O)c2cc3cc(cc(c3oc2=O)Br)Br
ZINC13123302	2.76	7.36	-57.93	1	8	-1	460.462	6	C[C@@H]1Cc2cc(ccc2O1)C(=O)C3=C(C(=O)N([C@H]3c4ccc(c(c4)OC)O)Cc5ccco5)[O-]
ZINC00971704	4.62	0.34	-20.34	1	4	0	451.114	3	C[C@@H](c1ccccc1)NC(=O)c2cc3cc(cc(c3oc2=O)Br)Br
ZINC13123301	2.76	6.97	-61.33	1	8	-1	460.462	6	C[C@H]1Cc2cc(ccc2O1)C(=O)C3=C(C(=O)N([C@@H]3c4ccc(c(c4)OC)O)Cc5ccco5)[O-]
ZINC08441551	6.5	0.7	-10.07	2	0	0	540.454	6	CC1=NC2=C([C@H](C1C(=O)OC)c3ccc(c(c3)COc4cccc(c4)Br)OC)C(=O)CC(C2)(C)C
ZINC08441551	6.5	0.65	-13.06	0	6	0	540.454	7	CC1=C([C@@H]([C@H]2C(=N1)CC(CC2=O)(C)C)c3ccc(c(c3)COc4cccc(c4)Br)OC)C(=O)OC
ZINC08441551	6.69	0.76	-10.66	0	6	0	540.454	7	CC1(CC2=NC(=C)C([C@@H](C2C(=O)C1)c3ccc(c(c3)COc4cccc(c4)Br)OC)C(=O)OC)C
ZINC08441551	6.69	1.92	-12.84	0	6	0	540.454	7	CC1(CC2=NC(=C)C([C@@H]([C@@H]2C(=O)C1)c3ccc(c(c3)COc4cccc(c4)Br)OC)C(=O)OC)C
ZINC06444168	5.48	13.24	-16.12	1	6	0	513.022	7	Cc1ccccc1NC(=O)c2c(nc(c(c2c3cccnc3)C#N)SCC(=O)c4ccc(cc4)Cl)C
ZINC13123303	2.76	7	-60.87	1	8	-1	460.462	6	C[C@@H]1Cc2cc(ccc2O1)C(=O)C3=C(C(=O)N([C@@H]3c4ccc(c(c4)OC)O)Cc5ccco5)[O-]
ZINC00703067	5.15	13.53	-46.78	0	7	-1	506.534	7	C[C@H]1Cc2cc(ccc2O1)C(=O)C3=C(C(=O)N([C@H]3c4cccc(c4)Oc5ccccc5)Cc6ccco6)[O-]
ZINC00703067	5.6	0.48	-29.14	1	7	0	507.542	6	C[C@H]1Cc2cc(ccc2O1)C(=C3[C@@H](N(C(=O)C3=O)Cc4ccco4)c5cccc(c5)Oc6ccccc6)O
ZINC00703067	4.57	0.75	-23.14	0	7	0	507.542	7	C[C@H]1Cc2cc(ccc2O1)C(=O)[C@@H]3[C@@H](N(C(=O)C3=O)Cc4ccco4)c5cccc(c5)Oc6ccccc6
ZINC08441547	6.59	3.51	-11.08	2	0	0	529.555	6	CC1=NC2=C([C@@H](C1C(=O)OC)c3ccc(c(c3)COc4cccc(c4)C(F)(F)F)OC)C(=O)CC(C2)(C)C
ZINC08441547	6.77	3.6	-11.93	0	6	0	529.555	8	CC1(CC2=NC(=C)C([C@H](C2C(=O)C1)c3ccc(c(c3)COc4cccc(c4)C(F)(F)F)OC)C(=O)OC)C
ZINC08441547	6.77	4.67	-11.56	0	6	0	529.555	8	CC1(CC2=NC(=C)C([C@H]([C@H]2C(=O)C1)c3ccc(c(c3)COc4cccc(c4)C(F)(F)F)OC)C(=O)OC)C
ZINC08441547	6.59	3.65	-14.32	0	6	0	529.555	8	CC1=C([C@H]([C@H]2C(=N1)CC(CC2=O)(C)C)c3ccc(c(c3)COc4cccc(c4)C(F)(F)F)OC)C(=O)OC
ZINC00860537	4.82	-0.84	-14.44	1	6	0	428.517	7	COc1ccc(cc1)NC(=O)CSc2nc(c(nn2)c3ccccc3)c4ccccc4
ZINC00703068	5.15	13.56	-46.19	0	7	-1	506.534	7	C[C@@H]1Cc2cc(ccc2O1)C(=O)C3=C(C(=O)N([C@H]3c4cccc(c4)Oc5ccccc5)Cc6ccco6)[O-]
ZINC00703068	5.6	0.92	-26.96	1	7	0	507.542	6	C[C@@H]1Cc2cc(ccc2O1)C(=C3[C@@H](N(C(=O)C3=O)Cc4ccco4)c5cccc(c5)Oc6ccccc6)O
ZINC00703068	4.57	1.15	-18.04	0	7	0	507.542	7	C[C@@H]1Cc2cc(ccc2O1)C(=O)[C@H]3[C@@H](N(C(=O)C3=O)Cc4ccco4)c5cccc(c5)Oc6ccccc6
ZINC08974498	0.79	10.53	-24.14	1	9	0	509.563	5	CCn1cc(c(=O)c2c1cc(c(c2)F)N3CCN(CC3)C(=O)c4ccco4)C(=O)Nc5nc(cs5)C
ZINC00703069	5.15	13.17	-45.07	0	7	-1	506.534	7	C[C@H]1Cc2cc(ccc2O1)C(=O)C3=C(C(=O)N([C@@H]3c4cccc(c4)Oc5ccccc5)Cc6ccco6)[O-]
ZINC00703069	5.6	0.63	-27.89	1	7	0	507.542	6	C[C@H]1Cc2cc(ccc2O1)C(=C3[C@H](N(C(=O)C3=O)Cc4ccco4)c5cccc(c5)Oc6ccccc6)O
ZINC00703069	4.57	0.89	-22.24	0	7	0	507.542	7	C[C@H]1Cc2cc(ccc2O1)C(=O)[C@H]3[C@H](N(C(=O)C3=O)Cc4ccco4)c5cccc(c5)Oc6ccccc6
ZINC08441546	3.71	-0.81	-29.25	1	9	0	629.758	6	CCn1cc(c(=O)c2c1cc(c(c2)F)N3CCN(CC3)C(=O)c4ccco4)C(=O)Nc5nc(cs5)C67CC8CC(C6)CC(C8)C7
ZINC00703070	5.15	13.2	-44.8	0	7	-1	506.534	7	C[C@@H]1Cc2cc(ccc2O1)C(=O)C3=C(C(=O)N([C@@H]3c4cccc(c4)Oc5ccccc5)Cc6ccco6)[O-]
ZINC00703070	5.6	0.6	-27.32	1	7	0	507.542	6	C[C@@H]1Cc2cc(ccc2O1)C(=C3[C@H](N(C(=O)C3=O)Cc4ccco4)c5cccc(c5)Oc6ccccc6)O
ZINC00703070	4.57	0.89	-21.82	0	7	0	507.542	7	C[C@@H]1Cc2cc(ccc2O1)C(=O)[C@H]3[C@H](N(C(=O)C3=O)Cc4ccco4)c5cccc(c5)Oc6ccccc6
ZINC08441545	5.95	-0.5	-27.17	1	9	0	663.86	11	CCCCCCn1cc(c(=O)c2c1cc(c(c2)F)N3CCN(CC3)C(=O)OCC)C(=O)Nc4nc(cs4)C56CC7CC(C5)CC(C7)C6
ZINC08441543	4.13	0.96	-17.23	1	9	0	533.577	11	CCCCCOc1ccc(cc1OC)[C@H]2C(=C(C(=O)N2Cc3ccco3)O)C(=O)c4ccc5c(c4)OCCO5
ZINC08441543	3.55	0.8	-21.62	0	9	0	533.577	11	CCCCCOc1ccc(cc1OC)[C@H]2[C@@H](C(=O)C(=O)N2Cc3ccco3)C(=O)c4ccc5c(c4)OCCO5
ZINC08441542	6.59	3.63	-11.16	2	0	0	529.555	6	CC1=NC2=C([C@H](C1C(=O)OC)c3ccc(c(c3)COc4cccc(c4)C(F)(F)F)OC)C(=O)CC(C2)(C)C
ZINC08441542	6.77	3.69	-11.64	0	6	0	529.555	8	CC1(CC2=NC(=C)C([C@@H](C2C(=O)C1)c3ccc(c(c3)COc4cccc(c4)C(F)(F)F)OC)C(=O)OC)C
ZINC08441542	6.77	4.86	-13.63	0	6	0	529.555	8	CC1(CC2=NC(=C)C([C@@H]([C@@H]2C(=O)C1)c3ccc(c(c3)COc4cccc(c4)C(F)(F)F)OC)C(=O)OC)C
ZINC08441542	6.59	3.58	-13.97	0	6	0	529.555	8	CC1=C([C@@H]([C@H]2C(=N1)CC(CC2=O)(C)C)c3ccc(c(c3)COc4cccc(c4)C(F)(F)F)OC)C(=O)OC
ZINC01261828	1.93	14.35	-26.24	1	9	0	595.1	7	CCCc1nnc(s1)NC(=O)c2cn(c3cc(c(cc3c2=O)F)N4CCN(CC4)C(=O)c5cccc(c5)Cl)C6CC6
ZINC08441540	4.13	1.14	-19.07	1	9	0	533.577	11	CCCCCOc1ccc(cc1OC)[C@@H]2C(=C(C(=O)N2Cc3ccco3)O)C(=O)c4ccc5c(c4)OCCO5
ZINC08441540	3.55	0.97	-24.48	0	9	0	533.577	11	CCCCCOc1ccc(cc1OC)[C@@H]2[C@H](C(=O)C(=O)N2Cc3ccco3)C(=O)c4ccc5c(c4)OCCO5
ZINC00859989	3.45	0.9	-19.34	1	9	0	431.832	6	CCOc1c(cc(cc1OC)/C=C\2/C(=O)NN(C2=O)c3ccc(c(c3)Cl)C)[N+](=O)[O-]
ZINC13123054	2.58	7.59	-61.24	0	9	-1	476.461	7	COc1ccc(c(c1)OC)[C@H]2C(=C(C(=O)N2Cc3ccco3)[O-])C(=O)c4ccc5c(c4)OCCO5
ZINC08441539	6.97	3.89	-11.13	2	0	0	543.582	6	CCOC(=O)C1[C@@H](C2=C(CC(CC2=O)(C)C)N=C1C)c3ccc(c(c3)COc4cccc(c4)C(F)(F)F)OC
ZINC08441539	7.15	2.95	-11.86	0	6	0	543.582	9	CCOC(=O)C1[C@@H](C2C(=NC1=C)CC(CC2=O)(C)C)c3ccc(c(c3)COc4cccc(c4)C(F)(F)F)OC
ZINC08441539	6.97	3.87	-13.98	0	6	0	543.582	9	CCOC(=O)C1=C(N=C2CC(CC(=O)C2[C@H]1c3ccc(c(c3)COc4cccc(c4)C(F)(F)F)OC)(C)C)C
ZINC08441538	3.17	2.22	-10.45	0	6	0	417.259	2	Cc1cc(c(cc1C)Br)c2ccc(o2)C=C3C(=O)N(C(=O)N(C3=O)C)C
ZINC13123055	2.58	6.88	-62.07	0	9	-1	476.461	7	COc1ccc(c(c1)OC)[C@@H]2C(=C(C(=O)N2Cc3ccco3)[O-])C(=O)c4ccc5c(c4)OCCO5
ZINC08441537	7.82	2.11	-9	1	4	0	479.649	4	Cc1ccc2c(c1)c(cc(n2)c3ccccc3)C(=O)Nc4c(c5c(s4)C[C@H](CC5)C(C)(C)C)C#N
ZINC08441536	7.82	2.11	-9.06	1	4	0	479.649	4	Cc1ccc2c(c1)c(cc(n2)c3ccccc3)C(=O)Nc4c(c5c(s4)C[C@@H](CC5)C(C)(C)C)C#N
ZINC08441535	6.97	3.9	-11.07	2	0	0	543.582	6	CCOC(=O)C1[C@H](C2=C(CC(CC2=O)(C)C)N=C1C)c3ccc(c(c3)COc4cccc(c4)C(F)(F)F)OC
ZINC08441535	6.97	3.54	-13.88	0	6	0	543.582	9	CCOC(=O)C1=C(N=C2CC(CC(=O)C2[C@@H]1c3ccc(c(c3)COc4cccc(c4)C(F)(F)F)OC)(C)C)C
ZINC08441535	7.15	2.63	-11.75	0	6	0	543.582	9	CCOC(=O)C1[C@H](C2C(=NC1=C)CC(CC2=O)(C)C)c3ccc(c(c3)COc4cccc(c4)C(F)(F)F)OC
ZINC02064202	6.28	2.06	-9.73	0	4	0	460.327	6	Cc1ccc(cc1)c2cc(c3cc(ccc3n2)Br)C(=O)OCC(=O)c4ccccc4
ZINC08441534	4.26	10.67	-61.57	0	8	-1	506.575	10	CCCCCOc1ccc(cc1)[C@H]2C(=C(C(=O)N2C[C@H]3CCCO3)[O-])C(=O)c4ccc5c(c4)OCCO5
ZINC08441534	4.71	-1.7	-26.48	1	8	0	507.583	9	CCCCCOc1ccc(cc1)[C@H]2C(=C(c3ccc4c(c3)OCCO4)O)C(=O)C(=O)N2C[C@H]5CCCO5
ZINC08441534	3.68	-1.47	-18.27	0	8	0	507.583	10	CCCCCOc1ccc(cc1)[C@H]2[C@@H](C(=O)C(=O)N2C[C@H]3CCCO3)C(=O)c4ccc5c(c4)OCCO5
ZINC02064200	6	-2.15	-19.24	3	8	0	567.667	11	CCCCOc1ccc(cc1)c2c(c(nc(c2C(=O)Nc3ccccc3)C)SCC(=O)c4ccc(c(c4)O)O)C#N
ZINC08441533	4.26	10.62	-64.01	0	8	-1	506.575	10	CCCCCOc1ccc(cc1)[C@@H]2C(=C(C(=O)N2C[C@H]3CCCO3)[O-])C(=O)c4ccc5c(c4)OCCO5
ZINC08441533	4.71	-1.51	-27.54	1	8	0	507.583	9	CCCCCOc1ccc(cc1)[C@@H]2C(=C(c3ccc4c(c3)OCCO4)O)C(=O)C(=O)N2C[C@H]5CCCO5

Supplementary Table S2:
Detailed table of XP Docking using Schrodinger's Glide module

ZINC_ID	xLogP	ApolarDesolvation	PolarDesolvation	H Donors	H Acceptors	Charge	MolecularWeight	RotatableBonds	SMILES
ZINC08441533	3.68	-1.24	-23.99	0	8	0	507.583	10	CCCCCOc1ccc(cc1)[C@@H]2[C@@H](C(=O)C(=O)N2C[C@H]3CCCO3)C(=O)c4ccc5c(c4)OCCO5
ZINC08441532	6.21	4.78	-11.75	2	1	0	504.512	6	[H]/N=C/1\[C@@H](C2=CCCC[C@@H]2[C@@H](C1(C#N)C#N)c3ccc(c(c3)COc4cccc(c4)C(F)(F)F)OC)C#N
ZINC08441532	6.21	4.62	-41.36	2	6	1	505.52	6	COc1ccc(cc1COc2cccc(c2)C(F)(F)F)[C@H]3[C@H]4CCCC=C4[C@H](C(=[NH2+])C3(C#N)C#N)C#N
ZINC00858890	2.86	-1.32	-15.48	2	6	0	423.472	6	c1ccc(cc1)C(c2ccccc2)(C(=O)NN(c3ccccc3)C(=O)c4ccncc4)O
ZINC08441530	4.26	10.59	-66.17	0	8	-1	506.575	10	CCCCCOc1ccc(cc1)[C@H]2C(=C(C(=O)N2C[C@@H]3CCCO3)[O-])C(=O)c4ccc5c(c4)OCCO5
ZINC08441530	4.71	-1.48	-27.96	1	8	0	507.583	9	CCCCCOc1ccc(cc1)[C@H]2C(=C(c3ccc4c(c3)OCCO4)O)C(=O)C(=O)N2C[C@@H]5CCCO5
ZINC08441530	3.68	-1.22	-25.66	0	8	0	507.583	10	CCCCCOc1ccc(cc1)[C@H]2[C@H](C(=O)C(=O)N2C[C@@H]3CCCO3)C(=O)c4ccc5c(c4)OCCO5
ZINC08441529	6.21	4.73	-12.34	2	1	0	504.512	6	[H]/N=C/1\[C@H](C2=CCCC[C@@H]2[C@H](C1(C#N)C#N)c3ccc(c(c3)COc4cccc(c4)C(F)(F)F)OC)C#N
ZINC08441529	6.21	4.56	-42.27	2	6	1	505.52	6	COc1ccc(cc1COc2cccc(c2)C(F)(F)F)[C@@H]3[C@H]4CCCC=C4[C@@H](C(=[NH2+])C3(C#N)C#N)C#N
ZINC08441528	6.8	2.48	-15.92	1	4	0	467.569	6	Cc1ccc(cc1)c2cc(nc(c2C#N)SCC(=O)Nc3cccc(c3)C)c4ccc(cc4)F
ZINC08441527	7.24	2.93	-15.38	1	4	0	481.596	7	CCc1ccccc1NC(=O)CSc2c(c(cc(n2)c3ccc(cc3)F)c4ccc(cc4)C)C#N
ZINC08441526	4.26	10.14	-67.6	0	8	-1	506.575	10	CCCCCOc1ccc(cc1)[C@@H]2C(=C(C(=O)N2C[C@@H]3CCCO3)[O-])C(=O)c4ccc5c(c4)OCCO5
ZINC08441526	4.71	-1.72	-26.38	1	8	0	507.583	9	CCCCCOc1ccc(cc1)[C@@H]2C(=C(c3ccc4c(c3)OCCO4)O)C(=O)C(=O)N2C[C@@H]5CCCO5
ZINC08441526	3.68	-1.5	-18.04	0	8	0	507.583	10	CCCCCOc1ccc(cc1)[C@@H]2[C@H](C(=O)C(=O)N2C[C@@H]3CCCO3)C(=O)c4ccc5c(c4)OCCO5
ZINC13161513	6.21	12.1	-11.83	1	6	0	504.512	6	[H]/N=C/1\[C@H](C2=CCCC[C@H]2[C@@H](C1(C#N)C#N)c3ccc(c(c3)COc4cccc(c4)C(F)(F)F)OC)C#N
ZINC08441524	6.37	2.17	-16	1	4	0	453.542	6	Cc1ccc(cc1)c2cc(nc(c2C#N)SCC(=O)Nc3ccccc3)c4ccc(cc4)F
ZINC08441523	6.21	5.05	-16.78	2	1	0	504.512	6	[H]/N=C/1\[C@@H](C2=CCCC[C@H]2[C@H](C1(C#N)C#N)c3ccc(c(c3)COc4cccc(c4)C(F)(F)F)OC)C#N
ZINC08441523	6.21	4.88	-35.99	2	6	1	505.52	6	COc1ccc(cc1COc2cccc(c2)C(F)(F)F)[C@@H]3[C@@H]4CCCC=C4[C@H](C(=[NH2+])C3(C#N)C#N)C#N
ZINC08441522	5.14	12.12	-59.85	0	7	-1	504.603	10	CCCCCOc1ccc(cc1)[C@H]2C(=C(C(=O)N2C[C@H]3CCCO3)[O-])C(=O)c4ccc5c(c4)C[C@@H](O5)C
ZINC08441522	5.59	-1.65	-30.38	1	7	0	505.611	9	CCCCCOc1ccc(cc1)[C@H]2C(=C(c3ccc4c(c3)C[C@@H](O4)C)O)C(=O)C(=O)N2C[C@H]5CCCO5
ZINC08441522	4.56	-1.38	-25.26	0	7	0	505.611	10	CCCCCOc1ccc(cc1)[C@H]2[C@H](C(=O)C(=O)N2C[C@H]3CCCO3)C(=O)c4ccc5c(c4)C[C@@H](O5)C
ZINC08441521	6.31	1.19	-18.6	1	7	0	525.63	10	CCOc1ccc(cc1)NC(=O)CSc2c(c(cc(n2)c3ccc(cc3)OC)c4ccc(cc4)OC)C#N
ZINC08441520	2.99	8.59	-12.3	3	8	0	445.57	10	CCOC(=O)c1c(c(sc1NC(=O)CSc2[nH]nc(n2)CNc3ccccc3)C)C
ZINC08441519	2.11	16.82	-40.2	2	4	1	425.339	5	Cc1ccc(cc1)C[n+]2c3ccccc3n(c2N)CC(=O)c4ccc(c(c4)Cl)Cl
ZINC08441518	5.14	12.08	-62.21	0	7	-1	504.603	10	CCCCCOc1ccc(cc1)[C@@H]2C(=C(C(=O)N2C[C@H]3CCCO3)[O-])C(=O)c4ccc5c(c4)C[C@@H](O5)C
ZINC08441518	5.59	-1.5	-30.92	1	7	0	505.611	9	CCCCCOc1ccc(cc1)[C@@H]2C(=C(c3ccc4c(c3)C[C@@H](O4)C)O)C(=O)C(=O)N2C[C@H]5CCCO5
ZINC08441518	4.56	-1.28	-21.44	0	7	0	505.611	10	CCCCCOc1ccc(cc1)[C@@H]2[C@H](C(=O)C(=O)N2C[C@H]3CCCO3)C(=O)c4ccc5c(c4)C[C@@H](O5)C
ZINC08441517	5.94	2.85	-11.82	2	0	0	501.501	6	CC1=NC2=C([C@@H](C1C(=O)OC)c3ccc(c(c3)COc4cccc(c4)C(F)(F)F)OC)C(=O)CCC2
ZINC08441517	6.12	3.1	-13.12	0	6	0	501.501	8	COc1ccc(cc1COc2cccc(c2)C(F)(F)F)[C@H]3C4C(=NC(=C)C3C(=O)OC)CCCC4=O
ZINC08441517	6.12	4.16	-13.33	0	6	0	501.501	8	COc1ccc(cc1COc2cccc(c2)C(F)(F)F)[C@H]3[C@@H]4C(=NC(=C)C3C(=O)OC)CCCC4=O
ZINC08441517	5.94	2.94	-15.25	0	6	0	501.501	8	CC1=C([C@H]([C@H]2C(=N1)CCCC2=O)c3ccc(c(c3)COc4cccc(c4)C(F)(F)F)OC)C(=O)OC
ZINC08441516	5.14	12.05	-64.21	0	7	-1	504.603	10	CCCCCOc1ccc(cc1)[C@H]2C(=C(C(=O)N2C[C@@H]3CCCO3)[O-])C(=O)c4ccc5c(c4)C[C@@H](O5)C
ZINC08441516	5.59	-1.46	-30.96	1	7	0	505.611	9	CCCCCOc1ccc(cc1)[C@H]2C(=C(c3ccc4c(c3)C[C@@H](O4)C)O)C(=O)C(=O)N2C[C@@H]5CCCO5
ZINC08441516	4.56	-1.23	-21.48	0	7	0	505.611	10	CCCCCOc1ccc(cc1)[C@H]2[C@@H](C(=O)C(=O)N2C[C@@H]3CCCO3)C(=O)c4ccc5c(c4)C[C@@H](O5)C
ZINC08441515	5.14	11.61	-66	0	7	-1	504.603	10	CCCCCOc1ccc(cc1)[C@@H]2C(=C(C(=O)N2C[C@@H]3CCCO3)[O-])C(=O)c4ccc5c(c4)C[C@@H](O5)C
ZINC08441515	5.59	-1.7	-30.97	1	7	0	505.611	9	CCCCCOc1ccc(cc1)[C@@H]2C(=C(c3ccc4c(c3)C[C@@H](O4)C)O)C(=O)C(=O)N2C[C@@H]5CCCO5
ZINC08441515	4.56	-1.44	-25.09	0	7	0	505.611	10	CCCCCOc1ccc(cc1)[C@@H]2[C@@H](C(=O)C(=O)N2C[C@@H]3CCCO3)C(=O)c4ccc5c(c4)C[C@@H](O5)C
ZINC08441514	4.38	-0.69	-17.75	1	8	0	555.627	9	C[C@H]1Cc2cc(ccc2O1)C(=O)C3=C(C(=O)N([C@H]3c4ccc(c(c4)OC)OCc5ccccc5)C[C@@H]6CCCO6)O
ZINC08441514	3.8	-0.8	-23.83	0	8	0	555.627	9	C[C@H]1Cc2cc(ccc2O1)C(=O)[C@H]3[C@@H](N(C(=O)C3=O)C[C@@H]4CCCO4)c5ccc(c(c5)OC)OCc6ccccc6
ZINC08441513	5.94	2.83	-11.84	2	0	0	501.501	6	CC1=NC2=C([C@H](C1C(=O)OC)c3ccc(c(c3)COc4cccc(c4)C(F)(F)F)OC)C(=O)CCC2
ZINC08441513	6.12	2.89	-12.64	0	6	0	501.501	8	COc1ccc(cc1COc2cccc(c2)C(F)(F)F)[C@@H]3C4C(=NC(=C)C3C(=O)OC)CCCC4=O
ZINC08441513	6.12	4.31	-18.53	0	6	0	501.501	8	COc1ccc(cc1COc2cccc(c2)C(F)(F)F)[C@@H]3[C@H]4C(=NC(=C)C3C(=O)OC)CCCC4=O
ZINC08441513	5.94	2.77	-15.33	0	6	0	501.501	8	CC1=C([C@@H]([C@H]2C(=N1)CCCC2=O)c3ccc(c(c3)COc4cccc(c4)C(F)(F)F)OC)C(=O)OC
ZINC00703060	4.38	-0.99	-18.45	1	8	0	555.627	9	C[C@@H]1Cc2cc(ccc2O1)C(=O)C3=C(C(=O)N([C@H]3c4ccc(c(c4)OC)OCc5ccccc5)C[C@@H]6CCCO6)O
ZINC00703060	3.8	-1.15	-24.52	0	8	0	555.627	9	C[C@@H]1Cc2cc(ccc2O1)C(=O)[C@H]3[C@@H](N(C(=O)C3=O)C[C@@H]4CCCO4)c5ccc(c(c5)OC)OCc6ccccc6
ZINC01798315	0.99	14.26	-29.73	2	5	1	340.447	9	CCCCCCC[n+]1c2ccccc2n(c1N)CC(=O)c3ccco3
ZINC08441512	4.38	-1.16	-19.01	1	8	0	555.627	9	C[C@H]1Cc2cc(ccc2O1)C(=O)C3=C(C(=O)N([C@@H]3c4ccc(c(c4)OC)OCc5ccccc5)C[C@@H]6CCCO6)O
ZINC08441512	3.8	-1.29	-27.42	0	8	0	555.627	9	C[C@H]1Cc2cc(ccc2O1)C(=O)[C@H]3[C@H](N(C(=O)C3=O)C[C@@H]4CCCO4)c5ccc(c(c5)OC)OCc6ccccc6
ZINC00241369	-1.59	10.38	-29.19	2	5	1	270.312	4	CC[n+]1c2ccccc2n(c1N)CC(=O)c3ccco3
ZINC08441511	7.51	0.68	-9.9	2	0	0	588.926	6	CCOC(=O)C1[C@@H](C2=C(CC(CC2=O)(C)C)N=C1C)c3ccc(c(c3)COc4ccc(cc4Cl)Br)OC
ZINC08441511	7.51	0.6	-13.45	0	6	0	588.926	8	CCOC(=O)C1=C(N=C2CC(CC(=O)C2[C@H]1c3ccc(c(c3)COc4ccc(cc4Cl)Br)OC)(C)C)C
ZINC08441511	7.69	-0.5	-10.32	0	6	0	588.926	8	CCOC(=O)C1[C@@H](C2C(=NC1=C)CC(CC2=O)(C)C)c3ccc(c(c3)COc4ccc(cc4Cl)Br)OC
ZINC08441510	4.38	-1.14	-19.16	1	8	0	555.627	9	C[C@@H]1Cc2cc(ccc2O1)C(=O)C3=C(C(=O)N([C@@H]3c4ccc(c(c4)OC)OCc5ccccc5)C[C@@H]6CCCO6)O
ZINC08441510	3.8	-1.24	-27.54	0	8	0	555.627	9	C[C@@H]1Cc2cc(ccc2O1)C(=O)[C@H]3[C@H](N(C(=O)C3=O)C[C@@H]4CCCO4)c5ccc(c(c5)OC)OCc6ccccc6
ZINC06406305	4.85	9.05	-59.76	1	5	-1	463.307	4	Cc1ccc(cc1)C(=O)C2=C(C(=O)N([C@H]2c3ccc(cc3)Br)c4ccc(cc4)O)[O-]
ZINC06406732	4.85	8.99	-59.8	1	5	-1	463.307	4	Cc1ccc(cc1)C(=O)C2=C(C(=O)N([C@@H]2c3ccc(cc3)Br)c4ccc(cc4)O)[O-]
ZINC06406308	4.72	8.94	-59.88	1	5	-1	418.856	4	Cc1ccc(cc1)C(=O)C2=C(C(=O)N([C@H]2c3ccc(cc3)Cl)c4ccc(cc4)O)[O-]
ZINC06406737	4.72	8.89	-59.95	1	5	-1	418.856	4	Cc1ccc(cc1)C(=O)C2=C(C(=O)N([C@@H]2c3ccc(cc3)Cl)c4ccc(cc4)O)[O-]
ZINC08441509	4.22	-0.8	-13.97	1	8	0	521.61	10	CCCCOc1ccc(cc1OC)[C@H]2C(=C(C(=O)N2C[C@H]3CCCO3)O)C(=O)c4ccc5c(c4)C[C@@H](O5)C
ZINC08441509	3.64	-0.97	-17.9	0	8	0	521.61	10	CCCCOc1ccc(cc1OC)[C@H]2[C@@H](C(=O)C(=O)N2C[C@H]3CCCO3)C(=O)c4ccc5c(c4)C[C@@H](O5)C
ZINC08441508	7.51	0.86	-10.28	2	0	0	588.926	6	CCOC(=O)C1[C@H](C2=C(CC(CC2=O)(C)C)N=C1C)c3ccc(c(c3)COc4ccc(cc4Cl)Br)OC

Supplementary Table S2:
Detailed table of XP Docking using Schrodinger's Glide module

ZINC_ID	xLogP	ApolarDesolvation	PolarDesolvation	H Donors	H Acceptors	Charge	MolecularWeight	RotatableBonds	SMILES
ZINC08441508	7.51	0.5	-12.97	0	6	0	588.926	8	CCOC(=O)C1=C(N=C2CC(CC(=O)C2[C@@H]1c3ccc(c(c3)COc4ccc(cc4Cl)Br)OC)(C)C)C
ZINC08441508	7.69	-0.41	-10.59	0	6	0	588.926	8	CCOC(=O)C1[C@H](C2C(=NC1=C)CC(CC2=O)(C)C)c3ccc(c(c3)COc4ccc(cc4Cl)Br)OC
ZINC08441505	4.22	-0.77	-17.06	1	8	0	521.61	10	CCCCOc1ccc(cc1OC)[C@@H]2C(=C(C(=O)N2C[C@H]3CCCO3)O)C(=O)c4ccc5c(c4)C[C@@H](O5)C
ZINC08441505	3.64	-0.85	-24.6	0	8	0	521.61	10	CCCCOc1ccc(cc1OC)[C@@H]2[C@@H](C(=O)C(=O)N2C[C@H]3CCCO3)C(=O)c4ccc5c(c4)C[C@@H](O5)C
ZINC08441504	4.22	11.24	-63.97	0	8	-1	520.602	10	CCCCOc1ccc(cc1OC)[C@H]2C(=C(C(=O)N2C[C@@H]3CCCO3)[O-])C(=O)c4ccc5c(c4)C[C@@H](O5)C
ZINC08441504	4.67	-1.02	-27.28	1	8	0	521.61	9	CCCCOc1ccc(cc1OC)[C@H]2C(=C(c3ccc4c(c3)C[C@@H](O4)C)O)C(=O)C(=O)N2C[C@@H]5CCCO5
ZINC08441504	3.64	-0.75	-22.45	0	8	0	521.61	10	CCCCOc1ccc(cc1OC)[C@H]2[C@@H](C(=O)C(=O)N2C[C@@H]3CCCO3)C(=O)c4ccc5c(c4)C[C@@H](O5)C
ZINC08997319	4.16	12.74	-53.96	0	7	-1	472.546	5	CCc1ccc(cc1)N2C(=O)/C(=C/c3cc(n(c3C)c4ccc(cc4)C(=O)[O-])C)/C(=O)N=C2S
ZINC08914574	4.61	14.13	-50.36	0	5	-1	442.564	4	CCc1ccc(cc1)N2C(=O)/C(=C/c3cc(n(c3C)c4cccc(c4)C)C)/C(=NC2=S)[O-]
ZINC00858028	4.52	14.49	-50.75	0	7	-1	500.6	7	CCc1ccc(cc1)N2C(=O)/C(=C/c3cc(n(c3C)c4ccc(cc4)C(=O)OCC)C)/C(=NC2=S)[O-]
ZINC08441503	5.9	2.21	-11.83	1	0	0	487.955	6	CCOC(=O)C1[C@@H](C2=C(CC(CC2=O)(C)C)N=C1C)c3ccc(o3)COc4ccc(cc4Cl)F
ZINC08441503	6.08	1.25	-12.29	0	6	0	487.955	7	CCOC(=O)C1[C@@H](C2C(=NC1=C)CC(CC2=O)(C)C)c3ccc(o3)COc4ccc(cc4Cl)F
ZINC08441503	5.9	2.27	-13.81	0	6	0	487.955	7	CCOC(=O)C1=C(N=C2CC(CC(=O)C2[C@H]1c3ccc(o3)COc4ccc(cc4Cl)F)(C)C)C
ZINC08441502	4.22	10.66	-65.37	0	8	-1	520.602	10	CCCCOc1ccc(cc1OC)[C@@H]2C(=C(C(=O)N2C[C@@H]3CCCO3)[O-])C(=O)c4ccc5c(c4)C[C@@H](O5)C
ZINC08441502	4.67	-1.49	-30.55	1	8	0	521.61	9	CCCCOc1ccc(cc1OC)[C@@H]2C(=C(c3ccc4c(c3)C[C@@H](O4)C)O)C(=O)C(=O)N2C[C@@H]5CCCO5
ZINC08441502	3.64	-1.24	-26.03	0	8	0	521.61	10	CCCCOc1ccc(cc1OC)[C@@H]2[C@@H](C(=O)C(=O)N2C[C@@H]3CCCO3)C(=O)c4ccc5c(c4)C[C@@H](O5)C
ZINC00858026	4.61	12.57	-52.44	0	5	-1	434.585	4	CCc1ccc(cc1)N2C(=O)/C(=C/c3cc(n(c3C)C4CCCCC4)C)/C(=NC2=S)[O-]
ZINC00858024	5.23	14.25	-51.42	0	5	-1	456.591	4	CCc1ccc(cc1)N2C(=O)/C(=C/c3cc(n(c3C)c4cc(cc(c4)C)C)C)/C(=NC2=S)[O-]
ZINC08441501	3.56	-0.82	-15.84	1	9	0	537.609	10	CC(C)CCOc1ccc(cc1OC)[C@H]2C(=C(C(=O)N2C[C@H]3CCCO3)O)C(=O)c4ccc5c(c4)OCCO5
ZINC08441501	2.98	-0.98	-19.82	0	9	0	537.609	10	CC(C)CCOc1ccc(cc1OC)[C@H]2[C@@H](C(=O)C(=O)N2C[C@H]3CCCO3)C(=O)c4ccc5c(c4)OCCO5
ZINC08441500	2.78	6.31	-56.51	1	9	-1	457.513	8	CCc1nnc(s1)[N-]S(=O)(=O)c2ccc(cc2)N/C=C\C(=O)c3ccc4c(c3)OCO4
ZINC08441500	2.78	5.92	-57.46	1	9	-1	457.513	8	CCc1nnc(s1)[N-]S(=O)(=O)c2ccc(cc2)N/C=C/C(=O)c3ccc4c(c3)OCO4
ZINC08441499	3.56	-0.63	-17.94	1	9	0	537.609	10	CC(C)CCOc1ccc(cc1OC)[C@@H]2C(=C(C(=O)N2C[C@H]3CCCO3)O)C(=O)c4ccc5c(c4)OCCO5
ZINC08441499	2.98	-0.74	-23.58	0	9	0	537.609	10	CC(C)CCOc1ccc(cc1OC)[C@@H]2[C@H](C(=O)C(=O)N2C[C@H]3CCCO3)C(=O)c4ccc5c(c4)OCCO5
ZINC08441497	5.9	2.21	-11.23	1	0	0	487.955	6	CCOC(=O)C1[C@H](C2=C(CC(CC2=O)(C)C)N=C1C)c3ccc(o3)COc4ccc(cc4Cl)F
ZINC08441497	5.9	1.94	-16.39	0	6	0	487.955	7	CCOC(=O)C1=C(N=C2CC(CC(=O)C2[C@@H]1c3ccc(o3)COc4ccc(cc4Cl)F)(C)C)C
ZINC08441497	6.08	0.92	-13.85	0	6	0	487.955	7	CCOC(=O)C1[C@H](C2C(=NC1=C)CC(CC2=O)(C)C)c3ccc(o3)COc4ccc(cc4Cl)F
ZINC08441496	3.56	-0.85	-19.61	1	9	0	537.609	10	CC(C)CCOc1ccc(cc1OC)[C@H]2C(=C(C(=O)N2C[C@@H]3CCCO3)O)C(=O)c4ccc5c(c4)OCCO5
ZINC08441496	2.98	-1.02	-25.79	0	9	0	537.609	10	CC(C)CCOc1ccc(cc1OC)[C@H]2[C@@H](C(=O)C(=O)N2C[C@@H]3CCCO3)C(=O)c4ccc5c(c4)OCCO5
ZINC08441495	4.12	11.74	-59.9	1	7	-1	457.535	7	Cc1cc(nc(n1)[N-]S(=O)(=O)c2ccc(cc2)N/C=C\C(=O)c3ccc4ccccc4c3)C
ZINC08441495	4.12	11.35	-60.84	1	7	-1	457.535	7	Cc1cc(nc(n1)[N-]S(=O)(=O)c2ccc(cc2)N/C=C/C(=O)c3ccc4ccccc4c3)C
ZINC08441494	5.24	0.28	-12.81	1	7	0	456.527	5	CC1=C([C@H](n2c(=O)/c(=C/c3c[nH]nc3c4ccccc4)/sc2=N1)c5ccccc5)C(=O)OC
ZINC08441493	3.02	3.25	-52.77	1	9	-1	457.463	9	COc1cc(nc(n1)OC)[N-]S(=O)(=O)c2ccc(cc2)N/C=C\C(=O)c3ccc(cc3)F
ZINC08441493	3.02	2.86	-53.66	1	9	-1	457.463	9	COc1cc(nc(n1)OC)[N-]S(=O)(=O)c2ccc(cc2)N/C=C/C(=O)c3ccc(cc3)F
ZINC08441492	3.56	-1.16	-19.88	1	9	0	537.609	10	CC(C)CCOc1ccc(cc1OC)[C@@H]2C(=C(C(=O)N2C[C@@H]3CCCO3)O)C(=O)c4ccc5c(c4)OCCO5
ZINC08441492	2.98	-1.24	-28.31	0	9	0	537.609	10	CC(C)CCOc1ccc(cc1OC)[C@@H]2[C@@H](C(=O)C(=O)N2C[C@@H]3CCCO3)C(=O)c4ccc5c(c4)OCCO5
ZINC00857663	5.59	13.57	-15.29	0	6	0	452.558	6	CCc1c2c3cc(c(cc3c(nc2n(n1)c4ccccc4)c5ccc(cc5)N(C)C)OC)OC
ZINC02064139	7	2.52	-15.07	0	6	0	514.629	7	CN(C)c1ccc(cc1)c2c3cc(c(cc3c4c(nn(c4n2)c5ccccc5)Cc6ccccc6)OC)OC
ZINC00857662	5.92	13.41	-14.43	0	6	0	453.542	7	CCc1c2c3cc(c(cc3c(nc2n(n1)c4ccccc4)c5ccc(cc5)OCC)OC)OC
ZINC02064138	5.89	2.69	-16.77	0	7	0	497.595	9	CCc1c2c3cc(c(cc3c(nc2n(n1)c4ccccc4)c5ccc(c(c5)OCC)OCC)OC)OC
ZINC08441491	5.24	0.45	-12.95	1	7	0	456.527	5	CC1=C([C@@H](n2c(=O)/c(=C/c3c[nH]nc3c4ccccc4)/sc2=N1)c5ccccc5)C(=O)OC
ZINC00857661	5.2	11.26	-16.14	1	7	0	469.541	7	CCc1c2c3cc(c(cc3c(nc2n(n1)c4ccccc4)c5ccc(c(c5)OCC)O)OC)OC
ZINC00857661	5.2	11.57	-29.83	2	7	1	470.549	7	CCc1c2c3cc(c(cc3c([nH+]c2n(n1)c4ccccc4)c5ccc(c(c5)OCC)O)OC)OC
ZINC08441490	4.44	11.59	-64.45	0	8	-1	534.629	10	C[C@H]1Cc2cc(ccc2O1)C(=O)C3=C(C(=O)N([C@H]3c4ccc(c(c4)OC)OCCC(C)C)C[C@@H]5CCCO5)[O-]
ZINC08441490	4.89	-0.71	-27.23	1	8	0	535.637	9	C[C@H]1Cc2cc(ccc2O1)C(=C3[C@@H](N(C(=O)C3=O)C[C@@H]4CCCO4)c5ccc(c(c5)OC)OCCC(C)C)O
ZINC08441490	3.86	-0.43	-22.3	0	8	0	535.637	10	C[C@H]1Cc2cc(ccc2O1)C(=O)[C@H]3[C@@H](N(C(=O)C3=O)C[C@@H]4CCCO4)c5ccc(c(c5)OC)OCCC(C)C
ZINC08441489	5.67	0.15	-11.89	1	8	0	500.58	7	CCOC(=O)C1=C(N=c2n(c(=O)/c(=C\c3c[nH]nc3c4ccccc4)/s2)[C@H]1c5ccc(cc5)OC)C
ZINC00857660	5.35	2.25	-15.21	0	6	0	439.515	6	CCOc1ccc(cc1)c2c3cc(c(cc3c4c(nn(c4n2)c5ccccc5)C)OC)OC
ZINC08441488	4.44	11.62	-64.22	0	8	-1	534.629	10	C[C@@H]1Cc2cc(ccc2O1)C(=O)C3=C(C(=O)N([C@H]3c4ccc(c(c4)OC)OCCC(C)C)C[C@@H]5CCCO5)[O-]
ZINC08441488	4.89	-1.02	-32.1	1	8	0	535.637	9	C[C@@H]1Cc2cc(ccc2O1)C(=C3[C@@H](N(C(=O)C3=O)C[C@@H]4CCCO4)c5ccc(c(c5)OC)OCCC(C)C)O
ZINC08441488	3.86	-0.79	-22.93	0	8	0	535.637	10	C[C@@H]1Cc2cc(ccc2O1)C(=O)[C@H]3[C@@H](N(C(=O)C3=O)C[C@@H]4CCCO4)c5ccc(c(c5)OC)OCCC(C)C
ZINC04180918	4.74	11.22	-20.16	1	4	0	439.353	1	CC(=O)N1c2ccccc2NC3=C([C@H]1c4cccc(c4)Br)C(=O)CC(C3)(C)C
ZINC08441487	5.67	-0.02	-12.35	1	8	0	500.58	7	CCOC(=O)C1=C(N=c2n(c(=O)/c(=C\c3c[nH]nc3c4ccccc4)/s2)[C@@H]1c5ccc(cc5)OC)C
ZINC00703059	4.44	11.35	-64.61	0	8	-1	534.629	10	C[C@H]1Cc2cc(ccc2O1)C(=O)C3=C(C(=O)N([C@@H]3c4ccc(c(c4)OC)OCCC(C)C)C[C@@H]5CCCO5)[O-]
ZINC00703059	4.89	-1.18	-30.44	1	8	0	535.637	9	C[C@H]1Cc2cc(ccc2O1)C(=C3[C@H](N(C(=O)C3=O)C[C@@H]4CCCO4)c5ccc(c(c5)OC)OCCC(C)C)O
ZINC00703059	3.86	-0.93	-25.95	0	8	0	535.637	10	C[C@H]1Cc2cc(ccc2O1)C(=O)[C@H]3[C@H](N(C(=O)C3=O)C[C@@H]4CCCO4)c5ccc(c(c5)OC)OCCC(C)C
ZINC04180917	4.74	11.2	-20.53	1	4	0	439.353	1	CC(=O)N1c2ccccc2NC3=C([C@@H]1c4cccc(c4)Br)C(=O)CC(C3)(C)C
ZINC09334009	5.42	11.38	-17.88	2	6	0	482.58	5	CC1(CC2=C([C@H](Nc3ccc(cc3N2)C(=O)c4ccccc4)c5ccc(c(c5)OC)OC)C(=O)C1)C
ZINC08441484	5.72	0.39	-13.7	1	8	0	513.623	7	CCOC(=O)C1=C(N=c2n(c(=O)/c(=C/c3c[nH]nc3c4ccccc4)/s2)[C@H]1c5ccc(cc5)N(C)C)C
ZINC08441483	4.44	11.39	-64.12	0	8	-1	534.629	10	C[C@@H]1Cc2cc(ccc2O1)C(=O)C3=C(C(=O)N([C@@H]3c4ccc(c(c4)OC)OCCC(C)C)C[C@@H]5CCCO5)[O-]
ZINC08441483	4.89	-1.2	-31.64	1	8	0	535.637	9	C[C@@H]1Cc2cc(ccc2O1)C(=C3[C@H](N(C(=O)C3=O)C[C@@H]4CCCO4)c5ccc(c(c5)OC)OCCC(C)C)O

Supplementary Table S2:
Detailed table of XP Docking using Schrodinger's Glide module

ZINC_ID	xLogP	ApolarDesolvation	PolarDesolvation	H Donors	H Acceptors	Charge	MolecularWeight	RotatableBonds	SMILES
ZINC08441483	3.86	-0.97	-24.02	0	8	0	535.637	10	C[C@@H]1Cc2cc(ccc2O1)C(=O)[C@@H]3[C@H](N(C(=O)C3=O)C[C@@H]4CCCO4)c5ccc(c(c5)OC)OCCC(C)C
ZINC09334008	5.42	11.38	-18.46	2	6	0	482.58	5	CC1(CC2=C([C@@H](Nc3ccc(cc3N2)C(=O)c4ccccc4)c5ccc(c(c5)OC)OC)C(=O)C1)C
ZINC08441482	6.45	-0.08	-11.47	2	4	0	456.973	3	CC1(CC2=C([C@H](Nc3ccc(cc3N2)C(=O)c4ccccc4)c5ccc(cc5)Cl)C(=O)C1)C
ZINC08441481	5.72	0.23	-13.52	1	8	0	513.623	7	CCOC(=O)C1=C(N=c2n(c(=O)/c(=C/c3c[nH]nc3c4ccccc4)/s2)[C@@H]1c5ccc(cc5)N(C)C)C
ZINC08441480	4.79	12.64	-66.77	0	7	-1	524.593	8	C[C@H]1Cc2cc(ccc2O1)C(=O)C3=C(C(=O)N([C@H]3c4ccc(cc4)OCc5ccccc5)C[C@@H]6CCCO6)[O-]
ZINC08441480	5.24	-1.62	-32.27	1	7	0	525.601	7	C[C@H]1Cc2cc(ccc2O1)C(=C3[C@@H](N(C(=O)C3=O)C[C@@H]4CCCO4)c5ccc(cc5)OCc6ccccc6)O
ZINC08441480	4.21	-1.39	-22.85	0	7	0	525.601	8	C[C@H]1Cc2cc(ccc2O1)C(=O)[C@H]3[C@@H](N(C(=O)C3=O)C[C@@H]4CCCO4)c5ccc(cc5)OCc6ccccc6
ZINC08441479	6.45	-0.09	-10.01	2	4	0	456.973	3	CC1(CC2=C([C@@H](Nc3ccc(cc3N2)C(=O)c4ccccc4)c5ccc(cc5)Cl)C(=O)C1)C
ZINC00857639	5.55	2.42	-21.59	1	5	0	466.581	4	CC(=O)N1c2ccccc2NC3=C([C@H]1c4ccccc4OCc5ccccc5)C(=O)CC(C3)(C)C
ZINC08441478	4.79	12.67	-66.54	0	7	-1	524.593	8	C[C@@H]1Cc2cc(ccc2O1)C(=O)C3=C(C(=O)N([C@H]3c4ccc(cc4)OCc5ccccc5)C[C@@H]6CCCO6)[O-]
ZINC08441478	5.24	-1.27	-28.56	1	7	0	525.601	7	C[C@@H]1Cc2cc(ccc2O1)C(=C3[C@@H](N(C(=O)C3=O)C[C@@H]4CCCO4)c5ccc(cc5)OCc6ccccc6)O
ZINC08441478	4.21	-1.01	-24.58	0	7	0	525.601	8	C[C@@H]1Cc2cc(ccc2O1)C(=O)[C@@H]3[C@@H](N(C(=O)C3=O)C[C@@H]4CCCO4)c5ccc(cc5)OCc6ccccc6
ZINC08441477	5.62	0.26	-12.62	1	7	0	470.554	6	CCOC(=O)C1=C(N=c2n(c(=O)/c(=C/c3c[nH]nc3c4ccccc4)/s2)[C@@H]1c5ccccc5)C
ZINC00857640	5.55	2.41	-21.71	1	5	0	466.581	4	CC(=O)N1c2ccccc2NC3=C([C@@H]1c4ccccc4OCc5ccccc5)C(=O)CC(C3)(C)C
ZINC00857561	5.91	2.49	-13	0	7	0	487.581	6	CCOC(=O)C1=C(N=c2n(c(=O)/c(=C/c3cn(c4c3cccc4)C)/s2)[C@@H]1c5ccc(cc5)OC)C
ZINC08441476	4.79	12.39	-65.84	0	7	-1	524.593	8	C[C@H]1Cc2cc(ccc2O1)C(=O)C3=C(C(=O)N([C@@H]3c4ccc(cc4)OCc5ccccc5)C[C@@H]6CCCO6)[O-]
ZINC08441476	5.24	-1.83	-32.2	1	7	0	525.601	7	C[C@H]1Cc2cc(ccc2O1)C(=C3[C@H](N(C(=O)C3=O)C[C@@H]4CCCO4)c5ccc(cc5)OCc6ccccc6)O
ZINC08441476	4.21	-1.54	-25.54	0	7	0	525.601	8	C[C@H]1Cc2cc(ccc2O1)C(=O)[C@H]3[C@H](N(C(=O)C3=O)C[C@@H]4CCCO4)c5ccc(cc5)OCc6ccccc6
ZINC08441475	6.54	0.08	-14.45	1	6	0	519.626	4	COc1ccc(cc1OC)[C@H]2C3=C(c4ccccc4CC3)N=c5n2c(=O)/c(=C\c6c[nH]c7c6cccc7)/s5
ZINC08441474	6.54	-0.04	-13.57	1	6	0	519.626	4	COc1ccc(cc1OC)[C@@H]2C3=C(c4ccccc4CC3)N=c5n2c(=O)/c(=C\c6c[nH]c7c6cccc7)/s5
ZINC02064097	6.42	2.54	-12.56	0	6	0	458.51	6	CCOc1cc(ccc1OCc2cccc3c2cccc3)C=C4C(=O)OC5(CCCC5)OC4=O
ZINC02064096	6.73	2.61	-12.55	0	5	0	428.484	4	c1ccc(cc1)COc2ccc3ccccc3c2C=C4C(=O)OC5(CCCCC5)OC4=O
ZINC08441473	4.79	12.42	-65.33	0	7	-1	524.593	8	C[C@@H]1Cc2cc(ccc2O1)C(=O)C3=C(C(=O)N([C@@H]3c4ccc(cc4)OCc5ccccc5)C[C@@H]6CCCO6)[O-]
ZINC08441473	5.24	-1.83	-31.99	1	7	0	525.601	7	C[C@@H]1Cc2cc(ccc2O1)C(=C3[C@H](N(C(=O)C3=O)C[C@@H]4CCCO4)c5ccc(cc5)OCc6ccccc6)O
ZINC08441473	4.21	-1.53	-25.49	0	7	0	525.601	8	C[C@@H]1Cc2cc(ccc2O1)C(=O)[C@H]3[C@H](N(C(=O)C3=O)C[C@@H]4CCCO4)c5ccc(cc5)OCc6ccccc6
ZINC00648858	3.11	12.88	-36.26	2	6	1	461.283	4	CCn1c2ccccc2[nH+]c1N[C@H]3CC(=O)N(C3=O)c4ccccc4I
ZINC00648858	3.11	12.88	-36.35	2	6	1	461.283	4	CCn1c2ccccc2[nH+]c1N[C@@H]3CC(=O)N(C3=O)c4ccccc4I
ZINC00648858	3.11	12.47	-18.35	1	6	0	460.275	4	CCn1c2ccccc2nc1N[C@H]3CC(=O)N(C3=O)c4ccccc4I
ZINC00648858	3.11	12.47	-17.87	1	6	0	460.275	4	CCn1c2ccccc2nc1N[C@@H]3CC(=O)N(C3=O)c4ccccc4I
ZINC02064095	5.51	3.16	-11.58	0	6	0	426.44	5	COc1cc(ccc1OCc2ccccc2F)C=C3C(=O)OC4(CCCCC4)OC3=O
ZINC02064093	6.41	2.19	-11.93	0	5	0	435.907	3	c1ccc(c(c1)Cn2cc(c3c2cccc3)C=C4C(=O)OC5(CCCCC5)OC4=O)Cl
ZINC08441472	3.2	9.1	-61.8	0	8	-1	478.521	8	CCCOc1ccc(cc1)[C@H]2C(=C(C(=O)N2C[C@H]3CCCO3)[O-])C(=O)c4ccc5c(c4)OCCO5
ZINC08441472	3.64	-1.94	-26.54	1	8	0	479.529	7	CCCOc1ccc(cc1)[C@H]2C(=C(c3ccc4c(c3)OCCO4)O)C(=O)C(=O)N2C[C@H]5CCCO5
ZINC08441472	2.61	-1.71	-18.41	0	8	0	479.529	8	CCCOc1ccc(cc1)[C@H]2[C@@H](C(=O)C(=O)N2C[C@H]3CCCO3)C(=O)c4ccc5c(c4)OCCO5
ZINC00857521	5.58	3.82	-17.86	0	7	0	430.84	4	COC(=O)c1cc(ccc1Cl)c2ccc(o2)C=C3C(=O)OC4(CCCCC4)OC3=O
ZINC00857497	6.01	2.28	-18.12	0	6	0	457.574	10	CCc1cccc(c1N(COC)C(=O)Cn2c3ccccc3nc2COc4ccccc4)CC
ZINC00857496	6.32	2.72	-12.83	0	5	0	441.575	9	CCc1cccc(c1N(COC)C(=O)Cn2c3ccccc3nc2Cc4ccccc4)CC
ZINC08441471	3.2	9.06	-64.16	0	8	-1	478.521	8	CCCOc1ccc(cc1)[C@@H]2C(=C(C(=O)N2C[C@H]3CCCO3)[O-])C(=O)c4ccc5c(c4)OCCO5
ZINC08441471	3.64	-1.75	-27.71	1	8	0	479.529	7	CCCOc1ccc(cc1)[C@@H]2C(=C(c3ccc4c(c3)OCCO4)O)C(=O)C(=O)N2C[C@H]5CCCO5
ZINC08441471	2.61	-1.48	-24.13	0	8	0	479.529	8	CCCOc1ccc(cc1)[C@@H]2[C@@H](C(=O)C(=O)N2C[C@H]3CCCO3)C(=O)c4ccc5c(c4)OCCO5
ZINC00857459	4.86	-0.05	-62.82	0	6	-1	499.772	5	Cc1ccc2cc(c(nc2c1)Cl)C3=NN([C@H](C3)c4ccc(cc4)Br)C(=O)CCC(=O)[O-]
ZINC00857460	4.86	-0.05	-62.83	0	6	-1	499.772	5	Cc1ccc2cc(c(nc2c1)Cl)C3=NN([C@@H](C3)c4ccc(cc4)Br)C(=O)CCC(=O)[O-]
ZINC08441470	3.2	9.03	-66.33	0	8	-1	478.521	8	CCCOc1ccc(cc1)[C@H]2C(=C(C(=O)N2C[C@@H]3CCCO3)[O-])C(=O)c4ccc5c(c4)OCCO5
ZINC08441470	3.64	-1.72	-28.05	1	8	0	479.529	7	CCCOc1ccc(cc1)[C@H]2C(=C(c3ccc4c(c3)OCCO4)O)C(=O)C(=O)N2C[C@@H]5CCCO5
ZINC08441470	2.61	-1.46	-25.7	0	8	0	479.529	8	CCCOc1ccc(cc1)[C@H]2[C@H](C(=O)C(=O)N2C[C@@H]3CCCO3)C(=O)c4ccc5c(c4)OCCO5
ZINC08441469	5.83	1.26	-11.06	0	6	0	485.939	7	CC1=NC2=C([C@@H](C1C(=O)OC)c3ccc(c(c3)COc4ccc(c(c4)Cl)F)OC)C(=O)CCC2
ZINC08441469	6.02	1.51	-12.51	0	6	0	485.939	7	COc1ccc(cc1COc2ccc(c(c2)Cl)F)[C@H]3C4C(=NC(=C)C3C(=O)OC)CCCC4=O
ZINC08441469	6.02	2.56	-12.55	0	6	0	485.939	7	COc1ccc(cc1COc2ccc(c(c2)Cl)F)[C@H]3[C@@H]4C(=NC(=C)C3C(=O)OC)CCCC4=O
ZINC08441469	5.83	1.35	-14.51	0	6	0	485.939	7	CC1=C([C@H]([C@H]2C(=N1)CCCC2=O)c3ccc(c(c3)COc4ccc(c(c4)Cl)F)OC)C(=O)OC
ZINC08441468	6.57	1.81	-18.38	1	6	0	531.637	8	COc1ccc(cc1OC)c2cc(nc(c2C#N)SCC(=O)Nc3cccc4c3cccc4)c5ccccc5
ZINC08441467	3.2	8.58	-67.7	0	8	-1	478.521	8	CCCOc1ccc(cc1)[C@@H]2C(=C(C(=O)N2C[C@@H]3CCCO3)[O-])C(=O)c4ccc5c(c4)OCCO5
ZINC08441467	3.64	-2.43	-31.39	1	8	0	479.529	7	CCCOc1ccc(cc1)[C@@H]2C(=C(c3ccc4c(c3)OCCO4)O)C(=O)C(=O)N2C[C@@H]5CCCO5
ZINC08441467	2.61	-2.2	-25.21	0	8	0	479.529	8	CCCOc1ccc(cc1)[C@@H]2[C@H](C(=O)C(=O)N2C[C@@H]3CCCO3)C(=O)c4ccc5c(c4)OCCO5
ZINC08441466	6.27	2.23	-16.99	1	6	0	509.631	9	CCc1ccccc1NC(=O)CSc2c(c(cc(n2)c3ccccc3)c4ccc(c(c4)OC)OC)C#N
ZINC08441465	4.08	10.56	-59.97	0	7	-1	476.549	8	CCCOc1ccc(cc1)[C@H]2C(=C(C(=O)N2C[C@H]3CCCO3)[O-])C(=O)c4ccc5c(c4)C[C@@H](O5)C
ZINC08441465	4.52	-1.89	-30.48	1	7	0	477.557	7	CCCOc1ccc(cc1)[C@H]2C(=C(c3ccc4c(c3)C[C@@H](O4)C)O)C(=O)C(=O)N2C[C@H]5CCCO5
ZINC08441465	3.49	-1.63	-25.35	0	7	0	477.557	8	CCCOc1ccc(cc1)[C@H]2[C@H](C(=O)C(=O)N2C[C@H]3CCCO3)C(=O)c4ccc5c(c4)C[C@@H](O5)C
ZINC08441464	3.72	-2.05	-15.46	2	9	0	474.564	10	CCOC(=O)c1c(c(sc1NC(=O)CSc2[nH]nc(n2)c3ccc(cc3)OC)C(=O)C)C
ZINC08441463	6.67	-1.51	-11.19	3	6	0	495.604	6	Cc1ccc(cc1)c2cc(c3c(c(sc3n2)C(=O)Nc4ccccc4OC)N)c5ccc(cc5)OC
ZINC08441462	4.08	10.52	-62.38	0	7	-1	476.549	8	CCCOc1ccc(cc1)[C@@H]2C(=C(C(=O)N2C[C@H]3CCCO3)[O-])C(=O)c4ccc5c(c4)C[C@@H](O5)C
ZINC08441462	4.52	-1.38	-27.19	1	7	0	477.557	7	CCCOc1ccc(cc1)[C@@H]2C(=C(c3ccc4c(c3)C[C@@H](O4)C)O)C(=O)C(=O)N2C[C@H]5CCCO5

Supplementary Table S2:
Detailed table of XP Docking using Schrodinger's Glide module

ZINC_ID	xLogP	ApolarDesolvation	PolarDesolvation	H Donors	H Acceptors	Charge	MolecularWeight	RotatableBonds	SMILES
ZINC08441462	3.49	-1.08	-22.48	0	7	0	477.557	8	CCCOc1ccc(cc1)[C@@H]2[C@@H](C(=O)C(=O)N2C[C@H]3CCCO3)C(=O)c4ccc5c(c4)C[C@@H](O5)C
ZINC08441461	6.7	1.48	-17.12	1	6	0	509.631	9	CCOc1ccc(cc1)NC(=O)CSc2c(c(cc(n2)c3ccc(cc3)C)c4ccc(cc4)OC)C#N
ZINC08441460	5.83	1.23	-11.06	0	6	0	485.939	7	CC1=NC2=C([C@H](C1C(=O)OC)c3ccc(c(c3)COc4ccc(c(c4)Cl)F)OC)C(=O)CCC2
ZINC08441460	6.02	1.28	-12.14	0	6	0	485.939	7	COc1ccc(cc1COc2ccc(c(c2)Cl)F)[C@@H]3C4C(=NC(=C)C3C(=O)OC)CCCC4=O
ZINC08441460	6.02	2.7	-17.53	0	6	0	485.939	7	COc1ccc(cc1COc2ccc(c(c2)Cl)F)[C@@H]3[C@H]4C(=NC(=C)C3C(=O)OC)CCCC4=O
ZINC08441460	5.83	1.16	-14.59	0	6	0	485.939	7	CC1=C([C@@H]([C@H]2C(=N1)CCCC2=O)c3ccc(c(c3)COc4ccc(c(c4)Cl)F)OC)C(=O)OC
ZINC00857407	6.27	1.59	-15.05	1	6	0	495.604	8	Cc1ccc(cc1)c2cc(c(c(n2)SCC(=O)Nc3ccccc3OC)C#N)c4ccc(cc4)OC
ZINC08441459	4.08	11.27	-58.87	0	7	-1	476.549	8	CCCOc1ccc(cc1)[C@H]2C(=C(C(=O)N2C[C@@H]3CCCO3)[O-])C(=O)c4ccc5c(c4)C[C@@H](O5)C
ZINC08441459	4.52	-1.7	-31.06	1	7	0	477.557	7	CCCOc1ccc(cc1)[C@H]2C(=C(c3ccc4c(c3)C[C@@H](O4)C)O)C(=O)C(=O)N2C[C@@H]5CCCO5
ZINC08441459	3.49	-1.47	-21.62	0	7	0	477.557	8	CCCOc1ccc(cc1)[C@H]2[C@@H](C(=O)C(=O)N2C[C@@H]3CCCO3)C(=O)c4ccc5c(c4)C[C@@H](O5)C
ZINC06245927	6.69	14.05	-15.53	1	5	0	479.605	7	Cc1ccc(cc1)c2cc(c(c(n2)SCC(=O)Nc3cccc(c3)C)C#N)c4ccc(cc4)OC
ZINC08441455	4.08	10.81	-59.79	0	7	-1	476.549	8	CCCOc1ccc(cc1)[C@@H]2C(=C(C(=O)N2C[C@@H]3CCCO3)[O-])C(=O)c4ccc5c(c4)C[C@@H](O5)C
ZINC08441455	4.52	-1.91	-30.92	1	7	0	477.557	7	CCCOc1ccc(cc1)[C@@H]2C(=C(c3ccc4c(c3)C[C@@H](O4)C)O)C(=O)C(=O)N2C[C@@H]5CCCO5
ZINC08441455	3.49	-1.68	-22.82	0	7	0	477.557	8	CCCOc1ccc(cc1)[C@@H]2[C@H](C(=O)C(=O)N2C[C@@H]3CCCO3)C(=O)c4ccc5c(c4)C[C@@H](O5)C
ZINC08441454	7.38	2.03	-17.15	1	5	0	515.638	7	Cc1ccc(cc1)c2cc(c(c(n2)SCC(=O)Nc3cccc4c3cccc4)C#N)c5ccccc5OC
ZINC08441453	6.26	2.32	-17.42	1	6	0	509.631	8	Cc1ccc(cc1)c2cc(c(c(n2)SCC(=O)Nc3ccccc3C)C#N)c4ccc(c(c4)OC)OC
ZINC15884982	2.29	7.38	-61.9	0	8	-1	450.467	6	COc1cccc(c1)[C@H]2C(=C(C(=O)N2C[C@H]3CCCO3)[O-])C(=O)c4ccc5c(c4)OCCO5
ZINC08441452	7.09	-1.33	-10.19	3	5	0	479.605	5	Cc1ccc(cc1)c2cc(c3c(c(sc3n2)C(=O)Nc4cccc(c4)C)N)c5ccc(cc5)OC
ZINC08441451	6.48	-0.15	-10.9	0	6	0	546.845	7	CC1=NC2=C([C@@H](C1C(=O)OC)c3ccc(c(c3)COc4ccc(cc4Br)Cl)OC)C(=O)CCC2
ZINC08441451	6.66	0.11	-12.19	0	6	0	546.845	7	COc1ccc(cc1COc2ccc(cc2Br)Cl)[C@H]3C4C(=NC(=C)C3C(=O)OC)CCCC4=O
ZINC08441451	6.66	1.16	-12.4	0	6	0	546.845	7	COc1ccc(cc1COc2ccc(cc2Br)Cl)[C@H]3[C@@H]4C(=NC(=C)C3C(=O)OC)CCCC4=O
ZINC08441451	6.48	-0.05	-14.36	0	6	0	546.845	7	CC1=C([C@H]([C@H]2C(=N1)CCCC2=O)c3ccc(c(c3)COc4ccc(cc4Br)Cl)OC)C(=O)OC
ZINC08441450	7.13	2.22	-15.05	1	5	0	493.632	8	CCc1ccccc1NC(=O)CSc2c(c(cc(n2)c3ccc(cc3)C)c4ccc(cc4)OC)C#N
ZINC08441449	7.78	-1.09	-12.68	3	5	0	515.638	5	Cc1ccc(cc1)c2cc(c3c(c(sc3n2)C(=O)Nc4cccc5c4cccc5)N)c6ccccc6OC
ZINC08441448	7.09	2.09	-15.69	1	5	0	493.632	7	Cc1ccc(cc1)c2cc(c(c(n2)SCC(=O)Nc3cc(cc(c3)C)C)C#N)c4ccc(cc4)OC
ZINC08441447	6.68	2.42	-17.5	1	6	0	523.658	8	Cc1ccc(cc1)c2cc(c(c(n2)SCC(=O)Nc3cc(cc(c3)C)C)C#N)c4ccc(c(c4)OC)OC
ZINC15884984	2.29	7.33	-62	0	8	-1	450.467	6	COc1cccc(c1)[C@@H]2C(=C(C(=O)N2C[C@H]3CCCO3)[O-])C(=O)c4ccc5c(c4)OCCO5
ZINC08441446	7.42	1.8	-16.33	1	5	0	515.638	7	Cc1ccc(cc1)c2cc(c(c(n2)SCC(=O)Nc3cccc4c3cccc4)C#N)c5ccc(cc5)OC
ZINC15884978	2.29	7.3	-65.65	0	8	-1	450.467	6	COc1cccc(c1)[C@H]2C(=C(C(=O)N2C[C@@H]3CCCO3)[O-])C(=O)c4ccc5c(c4)OCCO5
ZINC08441445	9.39	3.49	-16.64	0	5	0	591.736	8	Cc1ccc(cc1)c2cc(c(c(n2)SCC(=O)N(c3ccccc3)c4ccc5ccccc5c4)C#N)c6ccc(cc6)OC
ZINC15884980	2.29	6.85	-66.04	0	8	-1	450.467	6	COc1cccc(c1)[C@@H]2C(=C(C(=O)N2C[C@@H]3CCCO3)[O-])C(=O)c4ccc5c(c4)OCCO5
ZINC08441444	6.48	-0.18	-10.84	0	6	0	546.845	7	CC1=NC2=C([C@H](C1C(=O)OC)c3ccc(c(c3)COc4ccc(cc4Br)Cl)OC)C(=O)CCC2
ZINC08441444	6.66	-0.12	-11.8	0	6	0	546.845	7	COc1ccc(cc1COc2ccc(cc2Br)Cl)[C@@H]3C4C(=NC(=C)C3C(=O)OC)CCCC4=O
ZINC08441444	6.66	1.3	-17.53	0	6	0	546.845	7	COc1ccc(cc1COc2ccc(cc2Br)Cl)[C@@H]3[C@H]4C(=NC(=C)C3C(=O)OC)CCCC4=O
ZINC08441444	6.48	-0.24	-14.35	0	6	0	546.845	7	CC1=C([C@@H]([C@H]2C(=N1)CCCC2=O)c3ccc(c(c3)COc4ccc(cc4Br)Cl)OC)C(=O)OC
ZINC08441443	9.33	3.46	-15.74	0	4	0	561.71	7	Cc1ccc(cc1)c2cc(c(c(n2)SCC(=O)N(c3ccccc3)c4ccc5ccccc5c4)C#N)c6ccccc6
ZINC15952848	3.17	8.65	-64.39	0	7	-1	448.495	6	C[C@H]1Cc2cc(ccc2O1)C(=O)C3=C(C(=O)N([C@H]3c4cccc(c4)OC)C[C@@H]5CCCO5)[O-]
ZINC00857358	2.95	4.08	-22.68	0	8	0	426.469	7	CC(=O)N(CC1(CCCC1)c2ccc(c(c2)OC)OC)C(=O)c3ccc(cc3)[N+](=O)[O-]
ZINC08441440	5.39	3.04	-12.25	0	6	0	471.5	7	CCOC(=O)C1[C@H](C2=C(CC(CC2=O)(C)C)N=C1C)c3ccc(o3)COc4ccc(cc4F)F
ZINC08441440	5.39	2.78	-17.57	0	6	0	471.5	7	CCOC(=O)C1=C(N=C2CC(CC(=O)C2[C@@H]1c3ccc(o3)COc4ccc(cc4F)F)(C)C)C
ZINC08441440	5.57	1.75	-15.02	0	6	0	471.5	7	CCOC(=O)C1[C@H](C2C(=NC1=C)CC(CC2=O)(C)C)c3ccc(o3)COc4ccc(cc4F)F
ZINC01019938	3.17	8.72	-60.93	0	7	-1	448.495	6	C[C@@H]1Cc2cc(ccc2O1)C(=O)C3=C(C(=O)N([C@H]3c4cccc(c4)OC)C[C@@H]5CCCO5)[O-]
ZINC00857096	5.3	10.97	-11.5	1	5	0	472.155	6	c1cc(oc1)CN(CC(=O)Nc2c(cc(cc2Cl)Cl)Cl)C(=O)c3ccc(cc3)Cl
ZINC08441439	7.83	1.96	-18.5	1	6	0	585.729	11	CCOc1cc(ccc1OCc2ccccc2)c3cc(nc(c3C#N)SCC(=O)Nc4ccc(cc4)C)c5ccccc5
ZINC06444931	6.76	-3.85	-9.86	2	3	0	511.19	5	c1cc(ccc1NC[C@H](Cn2c3ccc(cc3c4c2ccc(c4)Cl)Cl)O)I
ZINC06444928	6.76	-3.78	-9.79	2	3	0	511.19	5	c1cc(ccc1NC[C@@H](Cn2c3ccc(cc3c4c2ccc(c4)Cl)Cl)O)I
ZINC15952849	3.17	8.41	-69.92	0	7	-1	448.495	6	C[C@H]1Cc2cc(ccc2O1)C(=O)C3=C(C(=O)N([C@@H]3c4cccc(c4)OC)C[C@@H]5CCCO5)[O-]
ZINC00856957	6.34	-3.02	-10.26	2	4	0	449.765	6	COc1ccc(cc1NC[C@H](Cn2c3ccc(cc3c4c2ccc(c4)Cl)Cl)O)Cl
ZINC00856956	6.34	9.64	-10.2	2	4	0	449.765	6	COc1ccc(cc1NC[C@@H](Cn2c3ccc(cc3c4c2ccc(c4)Cl)Cl)O)Cl
ZINC00862589	3.95	-3.35	-14.89	3	7	0	427.526	7	CCOc1ccc(cc1)NC(=O)C2=C(NC(=S)N[C@H]2c3ccc(cc3OC)OC)C
ZINC00862590	3.95	7.58	-15.45	3	7	0	427.526	7	CCOc1ccc(cc1)NC(=O)C2=C(NC(=S)N[C@@H]2c3ccc(cc3OC)OC)C
ZINC00703053	3.17	8.83	-60.51	0	7	-1	448.495	6	C[C@@H]1Cc2cc(ccc2O1)C(=O)C3=C(C(=O)N([C@@H]3c4cccc(c4)OC)C[C@@H]5CCCO5)[O-]
ZINC00862489	3.12	-2.38	-16.36	3	8	0	443.525	7	CC1=C([C@H](NC(=S)N1)c2cc(c(c(c2)OC)OC)OC)C(=O)Nc3ccccc3OC
ZINC00862490	3.12	-2.6	-14.91	3	8	0	443.525	7	CC1=C([C@@H](NC(=S)N1)c2cc(c(c(c2)OC)OC)OC)C(=O)Nc3ccccc3OC
ZINC00703050	1.02	7.18	-62.13	0	8	-1	421.429	5	c1cc(cnc1)[C@@H]2C(=C(C(=O)N2C[C@H]3CCCO3)[O-])C(=O)c4ccc5c(c4)OCCO5
ZINC00703050	1.47	-2.95	-27.17	1	8	0	422.437	4	c1cc(cnc1)[C@@H]2C(=C(c3ccc4c(c3)OCCO4)O)C(=O)C(=O)N2C[C@H]5CCCO5
ZINC00703050	0.44	-2.72	-24.47	0	8	0	422.437	5	c1cc(cnc1)[C@@H]2[C@H](C(=O)C(=O)N2C[C@H]3CCCO3)C(=O)c4ccc5c(c4)OCCO5
ZINC08441437	7.13	0.52	-10.24	0	6	0	574.899	7	CC1=NC2=C([C@@H](C1C(=O)OC)c3ccc(c(c3)COc4ccc(cc4Br)Cl)OC)C(=O)CC(C2)(C)C
ZINC08441437	7.32	0.61	-11.02	0	6	0	574.899	7	CC1(CC2=NC(=C)C([C@H](C2C(=O)C1)c3ccc(c(c3)COc4ccc(cc4Br)Cl)OC)C(=O)OC)C
ZINC08441437	7.32	1.83	-10.89	0	6	0	574.899	7	CC1(CC2=NC(=C)C([C@H]([C@H]2C(=O)C1)c3ccc(c(c3)COc4ccc(cc4Br)Cl)OC)C(=O)OC)C
ZINC08441437	7.13	0.73	-13.21	0	6	0	574.899	7	CC1=C([C@H]([C@H]2C(=N1)CC(CC2=O)(C)C)c3ccc(c(c3)COc4ccc(cc4Br)Cl)OC)C(=O)OC

Supplementary Table S2:
Detailed table of XP Docking using Schrodinger's Glide module

ZINC_ID	xLogP	ApolarDesolvation	PolarDesolvation	H Donors	H Acceptors	Charge	MolecularWeight	RotatableBonds	SMILES
ZINC00865273	5.24	9.97	-51.48	2	6	1	436.528	10	CCOc1ccc(cc1)C[NH+](CCc2ccc3c(c2)OCO3)Cc4cc(ccc4O)OC
ZINC00865273	5.24	7.68	-10.93	1	6	0	435.52	10	CCOc1ccc(cc1)CN(CCc2ccc3c(c2)OCO3)Cc4cc(ccc4O)OC
ZINC08441436	6.06	13.31	-56.03	1	5	1	499.425	10	CCOc1ccc(cc1)C[NH+](CCc2ccc3c(c2)OCO3)Cc4ccc(c(c4)Br)OC
ZINC08441436	6.06	11.18	-10.15	0	5	0	498.417	10	CCOc1ccc(cc1)CN(CCc2ccc3c(c2)OCO3)Cc4ccc(c(c4)Br)OC
ZINC02753344	4.9	11.98	-50.94	1	7	1	480.581	12	CCOc1ccc(cc1)C[NH+](CCc2ccc3c(c2)OCO3)Cc4cc(c(cc4OC)OC)OC
ZINC02753344	4.9	9.77	-11.8	0	7	0	479.573	12	CCOc1ccc(cc1)CN(CCc2ccc3c(c2)OCO3)Cc4cc(c(cc4OC)OC)OC
ZINC00703051	1.02	6.9	-64.16	0	8	-1	421.429	5	c1cc(cnc1)[C@H]2C(=C(C(=O)N2C[C@@H]3CCCO3)[O-])C(=O)c4ccc5c(c4)OCCO5
ZINC00703051	1.47	-3.03	-33.43	1	8	0	422.437	4	c1cc(cnc1)[C@H]2C(=C(c3ccc4c(c3)OCCO4)O)C(=O)C(=O)N2C[C@@H]5CCCO5
ZINC00703051	0.44	-2.81	-26.21	0	8	0	422.437	5	c1cc(cnc1)[C@H]2[C@@H](C(=O)C(=O)N2C[C@@H]3CCCO3)C(=O)c4ccc5c(c4)OCCO5
ZINC00703052	1.02	6.8	-60.58	0	8	-1	421.429	5	c1cc(cnc1)[C@@H]2C(=C(C(=O)N2C[C@@H]3CCCO3)[O-])C(=O)c4ccc5c(c4)OCCO5
ZINC00703052	1.47	-3.05	-28.2	1	8	0	422.437	4	c1cc(cnc1)[C@@H]2C(=C(c3ccc4c(c3)OCCO4)O)C(=O)C(=O)N2C[C@@H]5CCCO5
ZINC00703052	0.44	-2.77	-22.19	0	8	0	422.437	5	c1cc(cnc1)[C@@H]2[C@@H](C(=O)C(=O)N2C[C@@H]3CCCO3)C(=O)c4ccc5c(c4)OCCO5
ZINC08441435	1.89	7.11	-63.14	0	10	-1	510.519	8	COc1cc(cc(c1OC)OC)[C@H]2C(=C(C(=O)N2C[C@H]3CCCO3)[O-])C(=O)c4ccc5c(c4)OCCO5
ZINC08441435	2.34	-1.63	-33.45	1	10	0	511.527	7	COc1cc(cc(c1OC)OC)[C@H]2C(=C(c3ccc4c(c3)OCCO4)O)C(=O)C(=O)N2C[C@H]5CCCO5
ZINC08441435	1.31	-1.4	-29.32	0	10	0	511.527	8	COc1cc(cc(c1OC)OC)[C@H]2[C@@H](C(=O)C(=O)N2C[C@H]3CCCO3)C(=O)c4ccc5c(c4)OCCO5
ZINC08441434	7.13	0.61	-10.26	0	6	0	574.899	7	CC1=NC2=C([C@H](C1C(=O)OC)c3ccc(c(c3)COc4ccc(cc4Br)Cl)OC)C(=O)CC(C2)(C)C
ZINC08441434	7.13	0.55	-13.34	0	6	0	574.899	7	CC1=C([C@@H]([C@H]2C(=N1)CC(CC2=O)(C)C)c3ccc(c(c3)COc4ccc(cc4Br)Cl)OC)C(=O)OC
ZINC08441434	7.32	1.99	-12.05	0	6	0	574.899	7	CC1(CC2=NC(=C)C([C@@H]([C@@H]2C(=O)C1)c3ccc(c(c3)COc4ccc(cc4Br)Cl)OC)C(=O)OC)C
ZINC08441434	7.32	0.67	-10.76	0	6	0	574.899	7	CC1(CC2=NC(=C)C([C@@H](C2C(=O)C1)c3ccc(c(c3)COc4ccc(cc4Br)Cl)OC)C(=O)OC)C
ZINC08441430	5.15	3.38	-15.82	1	6	0	448.885	4	[H]/N=C\1/[C@@H](C2=CCCC[C@@H]2[C@@H](C1(C#N)C#N)c3ccc(o3)COc4ccc(cc4Cl)F)C#N
ZINC08441430	5.15	3.2	-40.16	2	6	1	449.893	4	c1cc(c(cc1F)Cl)OCc2ccc(o2)[C@H]3[C@H]4CCCC=C4[C@H](C(=[NH2+])C3(C#N)C#N)C#N
ZINC08441429	1.89	7.14	-62.94	0	10	-1	510.519	8	COc1cc(cc(c1OC)OC)[C@@H]2C(=C(C(=O)N2C[C@H]3CCCO3)[O-])C(=O)c4ccc5c(c4)OCCO5
ZINC08441429	2.34	-1.12	-29.11	1	10	0	511.527	7	COc1cc(cc(c1OC)OC)[C@@H]2C(=C(c3ccc4c(c3)OCCO4)O)C(=O)C(=O)N2C[C@H]5CCCO5
ZINC08441429	1.31	-0.84	-25.82	0	10	0	511.527	8	COc1cc(cc(c1OC)OC)[C@@H]2[C@H](C(=O)C(=O)N2C[C@H]3CCCO3)C(=O)c4ccc5c(c4)OCCO5
ZINC08441424	5.15	3.22	-13.32	1	6	0	448.885	4	[H]/N=C/1\[C@H](C2=CCCC[C@@H]2[C@H](C1(C#N)C#N)c3ccc(o3)COc4ccc(cc4Cl)F)C#N
ZINC08441424	5.15	3.04	-44.96	2	6	1	449.893	4	c1cc(c(cc1F)Cl)OCc2ccc(o2)[C@@H]3[C@H]4CCCC=C4[C@@H](C(=[NH2+])C3(C#N)C#N)C#N
ZINC08441423	1.89	7.03	-67.84	0	10	-1	510.519	8	COc1cc(cc(c1OC)OC)[C@H]2C(=C(C(=O)N2C[C@@H]3CCCO3)[O-])C(=O)c4ccc5c(c4)OCCO5
ZINC08441423	2.34	-1.14	-28.82	1	10	0	511.527	7	COc1cc(cc(c1OC)OC)[C@H]2C(=C(c3ccc4c(c3)OCCO4)O)C(=O)C(=O)N2C[C@@H]5CCCO5
ZINC08441423	1.31	-0.88	-26.88	0	10	0	511.527	8	COc1cc(cc(c1OC)OC)[C@H]2[C@H](C(=O)C(=O)N2C[C@@H]3CCCO3)C(=O)c4ccc5c(c4)OCCO5
ZINC08441421	5.15	3.4	-12.96	1	6	0	448.885	4	[H]/N=C/1\[C@H](C2=CCCC[C@H]2[C@H](C1(C#N)C#N)c3ccc(o3)COc4ccc(cc4Cl)F)C#N
ZINC08441421	5.15	3.23	-41.55	2	6	1	449.893	4	c1cc(c(cc1F)Cl)OCc2ccc(o2)[C@@H]3[C@@H]4CCCC=C4[C@@H](C(=[NH2+])C3(C#N)C#N)C#N
ZINC08441420	1.89	6.64	-66.22	0	10	-1	510.519	8	COc1cc(cc(c1OC)OC)[C@@H]2C(=C(C(=O)N2C[C@@H]3CCCO3)[O-])C(=O)c4ccc5c(c4)OCCO5
ZINC08441420	2.34	-1.61	-33.42	1	10	0	511.527	7	COc1cc(cc(c1OC)OC)[C@@H]2C(=C(c3ccc4c(c3)OCCO4)O)C(=O)C(=O)N2C[C@@H]5CCCO5
ZINC08441420	1.31	-1.34	-30.23	0	10	0	511.527	8	COc1cc(cc(c1OC)OC)[C@@H]2[C@@H](C(=O)C(=O)N2C[C@@H]3CCCO3)C(=O)c4ccc5c(c4)OCCO5
ZINC15880022	2.77	8.48	-64.06	0	9	-1	508.547	8	C[C@H]1Cc2cc(ccc2O1)C(=O)C3=C(C(=O)N([C@H]3c4cc(c(c(c4)OC)OC)OC)C[C@@H]5CCCO5)[O-]
ZINC08441415	3.74	5.11	-20	0	10	0	619.739	8	Cc1cc(c2c(c1)N(C(C3=C2C4(C(=C(S3)C(=O)OC)C(=O)OC)SC(=C(S4)C(=O)OC)C(=O)OC)(C)C)C(=O)C)C
ZINC08441414	5.89	1.06	-11.14	0	6	0	461.558	8	CCOC(=O)C1[C@@H](C2=C(CCCC2=O)N=C1C)c3ccc(c(c3)COc4ccc(cc4)C)OC
ZINC08441414	5.89	0.95	-15.2	0	6	0	461.558	8	CCOC(=O)C1=C(N=C2CCCC(=O)C2[C@H]1c3ccc(c(c3)COc4ccc(cc4)C)OC)C
ZINC08441414	6.07	-0.13	-11.7	0	6	0	461.558	8	CCOC(=O)C1[C@@H](C2C(=NC1=C)CCCC2=O)c3ccc(c(c3)COc4ccc(cc4)C)OC
ZINC08441413	2.77	8.55	-61.76	0	9	-1	508.547	8	C[C@@H]1Cc2cc(ccc2O1)C(=O)C3=C(C(=O)N([C@H]3c4cc(c(c(c4)OC)OC)OC)C[C@@H]5CCCO5)[O-]
ZINC08441412	6.23	2.1	-17.67	1	6	0	509.631	8	Cc1cc(cc(c1)NC(=O)CSc2c(c(cc(n2)c3ccccc3)c4ccc(c(c4)OC)OC)C#N)C
ZINC08441411	7.35	3.4	-16.71	0	6	0	557.675	9	COc1ccc(cc1OC)c2cc(nc(c2C#N)SCC(=O)N(c3ccccc3)c4ccccc4)c5ccccc5
ZINC08441410	4.48	-2.58	-11.27	1	8	0	449.286	6	Cc1ccc(cc1)c2nnc(o2)SCC(=O)Nc3ccc(cc3Br)[N+](=O)[O-]
ZINC15880023	2.77	8.37	-63.63	0	9	-1	508.547	8	C[C@H]1Cc2cc(ccc2O1)C(=O)C3=C(C(=O)N([C@@H]3c4cc(c(c(c4)OC)OC)OC)C[C@@H]5CCCO5)[O-]
ZINC08441408	6.52	0	-8.4	2	4	0	422.937	5	CC(C)OC(=O)c1c(c2c(cc(nc2s1)c3ccccc3)c4ccc(cc4)Cl)N
ZINC08441407	5.89	1.24	-11.28	0	6	0	461.558	8	CCOC(=O)C1[C@H](C2=C(CCCC2=O)N=C1C)c3ccc(c(c3)COc4ccc(cc4)C)OC
ZINC08441407	5.89	0.98	-14.8	0	6	0	461.558	8	CCOC(=O)C1=C(N=C2CCCC(=O)C2[C@@H]1c3ccc(c(c3)COc4ccc(cc4)C)OC)C
ZINC08441407	6.07	0.06	-12.11	0	6	0	461.558	8	CCOC(=O)C1[C@H](C2C(=NC1=C)CCCC2=O)c3ccc(c(c3)COc4ccc(cc4)C)OC
ZINC08441406	6.87	1.85	-16.68	1	7	0	590.126	10	CCOC(=O)c1c(c(sc1NC(=O)CSc2c(c(cc(n2)c3ccccc3)c4ccc(cc4)Cl)C#N)C(=O)C)C
ZINC08441405	2.77	8.66	-60.91	0	9	-1	508.547	8	C[C@@H]1Cc2cc(ccc2O1)C(=O)C3=C(C(=O)N([C@@H]3c4cc(c(c(c4)OC)OC)OC)C[C@@H]5CCCO5)[O-]
ZINC00856396	4.15	-3.71	-10.71	1	6	0	415.689	5	c1cc(cnc1)c2nnc(o2)SCC(=O)Nc3c(cc(cc3Cl)Cl)Cl
ZINC08441404	7.85	-0.13	-7.89	2	3	0	454.982	4	Cc1ccc(cc1)c2cc(nc3c2c(c(s3)C(=O)c4ccc(cc4)Cl)N)c5ccccc5
ZINC08441403	2.48	7.52	-60.53	1	8	-1	464.494	6	C[C@H]1Cc2cc(ccc2O1)C(=O)C3=C(C(=O)N([C@H]3c4ccc(c(c4)OC)O)C[C@@H]5CCCO5)[O-]
ZINC08441403	2.93	-3.18	-27.61	2	8	0	465.502	5	C[C@H]1Cc2cc(ccc2O1)C(=C3[C@@H](N(C(=O)C3=O)C[C@@H]4CCCO4)c5ccc(c(c5)OC)O)O
ZINC08441403	1.9	-2.91	-21.74	1	8	0	465.502	6	C[C@H]1Cc2cc(ccc2O1)C(=O)[C@H]3[C@@H](N(C(=O)C3=O)C[C@@H]4CCCO4)c5ccc(c(c5)OC)O
ZINC08441401	6.21	1.31	-10.54	0	6	0	499.966	8	CCOC(=O)C1[C@@H](C2=C(CCCC2=O)N=C1C)c3ccc(c(c3)COc4ccc(c(c4)Cl)F)OC
ZINC08441401	6.21	1.2	-14.57	0	6	0	499.966	8	CCOC(=O)C1=C(N=C2CCCC(=O)C2[C@H]1c3ccc(c(c3)COc4ccc(c(c4)Cl)F)OC)C
ZINC08441401	6.39	0.12	-11.34	0	6	0	499.966	8	CCOC(=O)C1[C@@H](C2C(=NC1=C)CCCC2=O)c3ccc(c(c3)COc4ccc(c(c4)Cl)F)OC
ZINC00617102	1.12	5.55	-51.2	2	6	-1	249.275	4	c1ccc(cc1)c2nnc(n2N)SCC(=O)[O-]
ZINC08441400	2.48	6.53	-63.44	1	8	-1	464.494	6	C[C@@H]1Cc2cc(ccc2O1)C(=O)C3=C(C(=O)N([C@H]3c4ccc(c(c4)OC)O)C[C@@H]5CCCO5)[O-]
ZINC08441400	2.93	-3.49	-32.72	2	8	0	465.502	5	C[C@@H]1Cc2cc(ccc2O1)C(=C3[C@@H](N(C(=O)C3=O)C[C@@H]4CCCO4)c5ccc(c(c5)OC)O)O

Supplementary Table S2:

Detailed table of XP Docking using Schrodinger's Glide module

ZINC_ID	xLogP	ApolarDesolvation	PolarDesolvation	H Donors	H Acceptors	Charge	MolecularWeight	RotatableBonds	SMILES
ZINC08441400	1.9	-3.26	-24.05	1	8	0	465.502	6	C[C@@H]1Cc2cc(ccc2O1)C(=O)[C@H]3[C@@H](N(C(=O)C3=O)C[C@@H]4CCCO4)c5ccc(c(c5)OC)O
ZINC08441398	2.48	7.15	-61.1	1	8	-1	464.494	6	C[C@H]1Cc2cc(ccc2O1)C(=O)C3=C(C(=O)N([C@@H]3c4ccc(c(c4)OC)O)C[C@@H]5CCCO5)[O-]
ZINC08441398	2.93	-3.65	-31.46	2	8	0	465.502	5	C[C@H]1Cc2cc(ccc2O1)C(=C3[C@H](N(C(=O)C3=O)C[C@@H]4CCCO4)c5ccc(c(c5)OC)O)O
ZINC08441398	1.9	-3.4	-26.29	1	8	0	465.502	6	C[C@H]1Cc2cc(ccc2O1)C(=O)[C@H]3[C@H](N(C(=O)C3=O)C[C@@H]4CCCO4)c5ccc(c(c5)OC)O
ZINC08441397	5.24	11.41	-43.41	2	4	1	436.757	5	c1ccc(c(c1)C(=O)c2cc(ccc2NC(=O)C[NH+]3CCCCC3)Br)Cl
ZINC08441396	6.21	1.5	-10.92	0	6	0	499.966	8	CCOC(=O)C1[C@H](C2=C(CCCC2=O)N=C1C)c3ccc(c(c3)COc4ccc(c(c4)Cl)F)OC
ZINC08441396	6.21	1.23	-14.43	0	6	0	499.966	8	CCOC(=O)C1=C(N=C2CCCC(=O)C2[C@@H]1c3ccc(c(c3)COc4ccc(c(c4)Cl)F)OC)C
ZINC08441396	6.39	0.32	-12.09	0	6	0	499.966	8	CCOC(=O)C1[C@H](C2C(=NC1=C)CCCC2=O)c3ccc(c(c3)COc4ccc(c(c4)Cl)F)OC
ZINC08441395	5.06	0.27	-41.87	2	9	0	528.359	7	COc1cc(c(c(c1)[N+](=O)[O-])[O-])[C@H]2Nc3ccc(cc3[C@H]([NH+]2CC(=O)OC)c4ccccc4)Br
ZINC01019935	2.48	7.46	-57.75	1	8	-1	464.494	6	C[C@@H]1Cc2cc(ccc2O1)C(=O)C3=C(C(=O)N([C@@H]3c4ccc(c(c4)OC)O)C[C@@H]5CCCO5)[O-]
ZINC01019935	2.93	-3.62	-32.59	2	8	0	465.502	5	C[C@@H]1Cc2cc(ccc2O1)C(=C3[C@H](N(C(=O)C3=O)C[C@@H]4CCCO4)c5ccc(c(c5)OC)O)O
ZINC01019935	1.9	-3.36	-27.48	1	8	0	465.502	6	C[C@@H]1Cc2cc(ccc2O1)C(=O)[C@H]3[C@H](N(C(=O)C3=O)C[C@@H]4CCCO4)c5ccc(c(c5)OC)O
ZINC01797879	5.06	0.52	-27.03	2	9	0	528.359	7	COc1cc(c(c(c1)[N+](=O)[O-])[O-])[C@H]2Nc3ccc(cc3[C@@H]([NH+]2CC(=O)OC)c4ccccc4)Br
ZINC08441394	4.5	2.62	-70.69	1	9	1	451.455	5	Cc1cc(=O)oc2c1ccc(c2)OC/C=C/3\[C@H](c4cc(ccc4O3)[N+](=O)[O-])[NH+]5CCOCC5
ZINC00703043	2.69	9.01	-59.77	0	8	-1	464.494	7	CCOc1ccc(cc1)[C@@H]2C(=C(C(=O)N2C[C@H]3CCCO3)[O-])C(=O)c4ccc5c(c4)OCCO5
ZINC00703043	3.14	-1.83	-27.9	1	8	0	465.502	6	CCOc1ccc(cc1)[C@@H]2C(=C(c3ccc4c(c3)OCCO4)O)C(=O)C(=O)N2C[C@H]5CCCO5
ZINC00703043	2.11	-1.56	-24.34	0	8	0	465.502	7	CCOc1ccc(cc1)[C@@H]2[C@@H](C(=O)C(=O)N2C[C@H]3CCCO3)C(=O)c4ccc5c(c4)OCCO5
ZINC08441393	4.5	2.82	-65.05	1	9	1	451.455	5	Cc1cc(=O)oc2c1ccc(c2)OC/C=C/3\[C@@H](c4cc(ccc4O3)[N+](=O)[O-])[NH+]5CCOCC5
ZINC08441392	6.19	1.73	-10.35	0	6	0	483.992	7	CCOC(=O)C1[C@@H](C2=C(CC(CC2=O)(C)C)N=C1C)c3ccc(o3)COc4ccc(c(c4)C)Cl
ZINC08441392	6.37	0.77	-11.35	0	6	0	483.992	7	CCOC(=O)C1[C@@H](C2C(=NC1=C)CC(CC2=O)(C)C)c3ccc(o3)COc4ccc(c(c4)C)Cl
ZINC08441392	6.19	1.8	-13.07	0	6	0	483.992	7	CCOC(=O)C1=C(N=C2CC(CC(=O)C2[C@H]1c3ccc(o3)COc4ccc(c(c4)C)Cl)(C)C)C
ZINC00703044	2.69	9.36	-60.25	0	8	-1	464.494	7	CCOc1ccc(cc1)[C@H]2C(=C(C(=O)N2C[C@@H]3CCCO3)[O-])C(=O)c4ccc5c(c4)OCCO5
ZINC00703044	3.14	-1.79	-28.29	1	8	0	465.502	6	CCOc1ccc(cc1)[C@H]2C(=C(c3ccc4c(c3)OCCO4)O)C(=O)C(=O)N2C[C@@H]5CCCO5
ZINC00703044	2.11	-1.53	-26.03	0	8	0	465.502	7	CCOc1ccc(cc1)[C@H]2[C@H](C(=O)C(=O)N2C[C@@H]3CCCO3)C(=O)c4ccc5c(c4)OCCO5
ZINC00703045	2.69	9	-58.87	0	8	-1	464.494	7	CCOc1ccc(cc1)[C@@H]2C(=C(C(=O)N2C[C@@H]3CCCO3)[O-])C(=O)c4ccc5c(c4)OCCO5
ZINC00703045	3.14	-2.04	-26.65	1	8	0	465.502	6	CCOc1ccc(cc1)[C@@H]2C(=C(c3ccc4c(c3)OCCO4)O)C(=O)C(=O)N2C[C@@H]5CCCO5
ZINC00703045	2.11	-1.82	-18.4	0	8	0	465.502	7	CCOc1ccc(cc1)[C@@H]2[C@H](C(=O)C(=O)N2C[C@@H]3CCCO3)C(=O)c4ccc5c(c4)OCCO5
ZINC08441388	6.19	1.73	-10.01	0	6	0	483.992	7	CCOC(=O)C1[C@H](C2=C(CC(CC2=O)(C)C)N=C1C)c3ccc(o3)COc4ccc(c(c4)C)Cl
ZINC08441388	6.19	1.47	-14.75	0	6	0	483.992	7	CCOC(=O)C1=C(N=C2CC(CC(=O)C2[C@@H]1c3ccc(o3)COc4ccc(c(c4)C)Cl)(C)C)C
ZINC08441388	6.37	0.44	-12.38	0	6	0	483.992	7	CCOC(=O)C1[C@H](C2C(=NC1=C)CC(CC2=O)(C)C)c3ccc(o3)COc4ccc(c(c4)C)Cl
ZINC00856090	4.42	2.76	-13.46	0	5	0	467.85	4	Cc1c(c(no1)c2c(cccc2Cl)F)C(=O)N3CCN(CC3)c4cccc(c4)C(F)(F)F
ZINC00703040	5.05	1.07	-25.85	0	8	0	526.618	7	CCOC(=O)C1CCN(CC1)C2=NC(=O)/C(=C/c3cn(nc3c4cc5ccccc5o4)c6ccccc6)/S2
ZINC03201398	6.32	2.56	-9.47	0	3	0	328.843	8	CCCCCCCOc1ccc(cc1)c2ccc(c(n2)Cl)C#N
ZINC08441387	4.25	-0.41	-13.19	1	9	0	442.888	6	CCn1c2ccccc2c3c1nc(nn3)SCC(=O)Nc4ccc(cc4[N+](=O)[O-])Cl
ZINC08441386	3.19	-2.42	-16.91	0	8	0	424.5	6	Cc1ccc(cc1[N+](=O)[O-])S(=O)(=O)N2CCS[C@H]2c3ccc(c(c3)OC)OC
ZINC08441384	6.21	12.98	-21.97	1	6	0	475.585	7	Cc1ccc(cc1)OCc2cc(ccc2OC)[C@H]3C4=C(CC(CC4=O)(C)C)NC(=C3C(=O)OC)C
ZINC08441384	6.33	0.5	-9.21	0	6	0	475.585	7	Cc1ccc(cc1)OCc2cc(ccc2OC)[C@H]3C4C(=O)CC(C=C4N=C(C3C(=O)OC)C)(C)C
ZINC08441383	3.19	-2.38	-16.87	0	8	0	424.5	6	Cc1ccc(cc1[N+](=O)[O-])S(=O)(=O)N2CCS[C@@H]2c3ccc(c(c3)OC)OC
ZINC08441382	5.8	-3.6	-14.14	0	4	0	490.444	5	COc1ccc(cc1Br)[C@H]2N(CCS2)S(=O)(=O)c3ccc(cc3)c4ccccc4
ZINC01019934	4.61	-0.46	-20.13	0	7	0	484.581	4	C[C@@H]1CN(C[C@H](O1)C)C2=NC(=O)/C(=C/c3cn(nc3c4cc5ccccc5o4)c6ccccc6)/S2
ZINC08441381	5.8	-3.89	-10.67	0	4	0	490.444	5	COc1ccc(cc1Br)[C@@H]2N(CCS2)S(=O)(=O)c3ccc(cc3)c4ccccc4
ZINC08441380	6.21	12.96	-21.92	1	6	0	475.585	7	Cc1ccc(cc1)OCc2cc(ccc2OC)[C@@H]3C4=C(CC(CC4=O)(C)C)NC(=C3C(=O)OC)C
ZINC08441380	6.33	0.76	-9.63	0	6	0	475.585	7	Cc1ccc(cc1)OCc2cc(ccc2OC)[C@@H]3C4C(=O)CC(C=C4N=C(C3C(=O)OC)C)(C)C
ZINC08441378	4.61	-0.45	-19.74	0	7	0	484.581	4	C[C@H]1CN(C[C@@H](O1)C)C2=NC(=O)/C(=C/c3cn(nc3c4cc5ccccc5o4)c6ccccc6)/S2
ZINC03584707	5.78	0.8	-5.11	0	4	0	302.418	10	CCCCCCCOc1ccc(cc1)c2nc(no2)CCC
ZINC08441375	6.07	0.83	-12.49	0	6	0	481.976	8	CCOC(=O)C1[C@@H](C2=C(CCCC2=O)N=C1C)c3ccc(c(c3)COc4ccccc4Cl)OC
ZINC08441375	6.07	0.64	-15.94	0	6	0	481.976	8	CCOC(=O)C1=C(N=C2CCCC(=O)C2[C@H]1c3ccc(c(c3)COc4ccccc4Cl)OC)C
ZINC08441375	6.25	-0.28	-13.52	0	6	0	481.976	8	CCOC(=O)C1[C@@H](C2C(=NC1=C)CCCC2=O)c3ccc(c(c3)COc4ccccc4Cl)OC
ZINC08441374	7.49	1.08	-16.88	1	6	0	588.154	9	CCOC(=O)c1c2c(sc1NC(=O)CSc3c(c(cc(n3)c4ccccc4)c5ccccc5Cl)C#N)CCCC2
ZINC00703038	3.88	-1.2	-20.67	0	7	0	456.527	4	c1ccc(cc1)n2cc(c(n2)c3cc4ccccc4o3)/C=C\5/C(=O)N=C(S5)N6CCOCC6
ZINC08441373	7.14	1.85	-17.66	0	4	0	496.035	5	c1ccc(cc1)c2cc(c(c(n2)SCC(=O)N3CCCc4c3cccc4)C#N)c5ccccc5Cl
ZINC08441372	4.12	0.21	-57.7	2	9	1	549.644	11	CC(C)CCOc1ccc(cc1OC)[C@H]2C(=C(C(=O)N2CC[NH+]3CCOCC3)O)C(=O)c4cc5ccccc5o4
ZINC08441372	3.53	0.12	-67.81	1	9	1	549.644	11	CC(C)CCOc1ccc(cc1OC)[C@H]2[C@@H](C(=O)C(=O)N2CC[NH+]3CCOCC3)C(=O)c4cc5ccccc5o4
ZINC08441371	6.07	0.84	-12.57	0	6	0	481.976	8	CCOC(=O)C1[C@H](C2=C(CCCC2=O)N=C1C)c3ccc(c(c3)COc4ccccc4Cl)OC
ZINC08441371	6.07	0.65	-16.02	0	6	0	481.976	8	CCOC(=O)C1=C(N=C2CCCC(=O)C2[C@@H]1c3ccc(c(c3)COc4ccccc4Cl)OC)C
ZINC08441371	6.25	-0.27	-13.62	0	6	0	481.976	8	CCOC(=O)C1[C@H](C2C(=NC1=C)CCCC2=O)c3ccc(c(c3)COc4ccccc4Cl)OC
ZINC08441370	7.03	1.43	-14.44	1	5	0	541.101	6	c1ccc(cc1)c2cc(c(c(n2)SCC(=O)Nc3c(c4c(s3)CCCC4)C#N)C#N)c5ccccc5Cl
ZINC08441369	8.63	2.61	-15.44	1	4	0	527.693	7	CC(C)(C)c1ccc(cc1)c2cc(nc(c2C#N)SCC(=O)Nc3cccc4c3cccc4)c5ccccc5
ZINC08441368	6.56	2.14	-18.88	1	6	0	514.006	8	COC(=O)c1ccc(cc1)NC(=O)CSc2c(c(cc(n2)c3ccccc3)c4ccccc4Cl)C#N
ZINC08441367	4.12	0.07	-51.12	2	9	1	549.644	11	CC(C)CCOc1ccc(cc1OC)[C@@H]2C(=C(C(=O)N2CC[NH+]3CCOCC3)O)C(=O)c4cc5ccccc5o4
ZINC08441367	3.53	-0.15	-60.18	1	9	1	549.644	11	CC(C)CCOc1ccc(cc1OC)[C@@H]2[C@@H](C(=O)C(=O)N2CC[NH+]3CCOCC3)C(=O)c4cc5ccccc5o4

Supplementary Table S2:
Detailed table of XP Docking using Schrodinger's Glide module

ZINC_ID	xLogP	ApolarDesolvation	PolarDesolvation	H Donors	H Acceptors	Charge	MolecularWeight	RotatableBonds	SMILES
ZINC00853651	4.95	-4.4	-9.92	1	5	0	469.158	5	c1cc(ccc1c2nnc(o2)SCC(=O)Nc3ccc(cc3)Br)Br
ZINC08441366	6.72	1.44	-11.57	0	6	0	510.03	8	CCOC(=O)C1[C@@H](C2=C(CC(CC2=O)(C)C)N=C1C)c3ccc(c(c3)COc4ccccc4Cl)OC
ZINC08441366	6.72	1.36	-15.03	0	6	0	510.03	8	CCOC(=O)C1=C(N=C2CC(CC(=O)C2[C@H]1c3ccc(c(c3)COc4ccccc4Cl)OC)(C)C)C
ZINC08441366	6.91	0.26	-11.7	0	6	0	510.03	8	CCOC(=O)C1[C@@H](C2C(=NC1=C)CC(CC2=O)(C)C)c3ccc(c(c3)COc4ccccc4Cl)OC
ZINC08441365	4.47	-0.7	-49.09	2	8	1	539.608	9	c1ccc(cc1)COc2ccc(cc2)[C@H]3C(=C(C(=O)N3CC[NH+]4CCOCC4)O)C(=O)c5cc6ccccc6o5
ZINC08441365	3.89	-0.86	-56.6	1	8	1	539.608	9	c1ccc(cc1)COc2ccc(cc2)[C@H]3[C@@H](C(=O)C(=O)N3CC[NH+]4CCOCC4)C(=O)c5cc6ccccc6o5
ZINC02064038	6.36	-1.63	-8.25	0	4	0	443.682	5	c1nc2c(c3c(s2)CCCC3)c(n1)SCC(=O)N(C4CCCCC4)C5CCCCC5
ZINC08441364	4.48	-2.58	-13.64	1	8	0	449.286	6	Cc1cc(ccc1NC(=O)CSc2nnc(o2)c3ccc(cc3)Br)[N+](=O)[O-]
ZINC08441363	4.7	-1.02	-12.21	1	7	0	428.539	5	Cc1ccc(cc1[N+](=O)[O-])NC(=O)[C@H](C)Sc2c3c4c(sc3ncn2)CCCC4
ZINC08441362	4.7	-1.01	-18.53	1	7	0	428.539	5	Cc1ccc(cc1[N+](=O)[O-])NC(=O)[C@@H](C)Sc2c3c4c(sc3ncn2)CCCC4
ZINC08441361	4.47	-0.69	-48.94	2	8	1	539.608	9	c1ccc(cc1)COc2ccc(cc2)[C@@H]3C(=C(C(=O)N3CC[NH+]4CCOCC4)O)C(=O)c5cc6ccccc6o5
ZINC08441361	3.89	-0.85	-56.86	1	8	1	539.608	9	c1ccc(cc1)COc2ccc(cc2)[C@@H]3[C@H](C(=O)C(=O)N3CC[NH+]4CCOCC4)C(=O)c5cc6ccccc6o5
ZINC00853635	4.9	-4.17	-8.32	1	5	0	469.158	5	c1ccc(c(c1)NC(=O)CSc2nnc(o2)c3ccc(cc3)Br)Br
ZINC09070026	3.3	-0.1	-50.09	2	8	1	463.51	6	COc1cccc(c1)[C@H]2/C(=C(/c3cc4ccccc4o3)\O)/C(=O)C(=O)N2CC[NH+]5CCOCC5
ZINC08441359	6.72	1.62	-11.84	0	6	0	510.03	8	CCOC(=O)C1[C@H](C2=C(CC(CC2=O)(C)C)N=C1C)c3ccc(c(c3)COc4ccccc4Cl)OC
ZINC08441359	6.91	0.35	-11.91	0	6	0	510.03	8	CCOC(=O)C1[C@H](C2C(=NC1=C)CC(CC2=O)(C)C)c3ccc(c(c3)COc4ccccc4Cl)OC
ZINC08441359	6.72	1.26	-14.63	0	6	0	510.03	8	CCOC(=O)C1=C(N=C2CC(CC(=O)C2[C@@H]1c3ccc(c(c3)COc4ccccc4Cl)OC)(C)C)C
ZINC00853634	4.48	-2.93	-18.23	1	8	0	449.286	6	Cc1ccc(cc1[N+](=O)[O-])NC(=O)CSc2nnc(o2)c3ccc(cc3)Br
ZINC09070021	3.3	-0.1	-50.66	2	8	1	463.51	6	COc1cccc(c1)[C@@H]2/C(=C(/c3cc4ccccc4o3)\O)/C(=O)C(=O)N2CC[NH+]5CCOCC5
ZINC08441357	5.94	-0.18	-10.88	0	4	0	431.586	5	c1ccc(cc1)N(c2ccccc2)C(=O)CSc3c4c5c(sc4ncn3)CCCC5
ZINC09274085	2.9	9.7	-62.05	2	10	1	523.562	8	COc1cc(cc(c1OC)OC)[C@H]2/C(=C(/c3cc4ccccc4o3)\O)/C(=O)C(=O)N2CC[NH+]5CCOCC5
ZINC09274085	1.87	10.39	-59.5	1	10	1	523.562	9	COc1cc(cc(c1OC)OC)[C@H]2[C@@H](C(=O)C(=O)N2CC[NH+]3CCOCC3)C(=O)c4cc5ccccc5o4
ZINC08441355	4.7	-0.66	-13.65	1	7	0	428.539	5	Cc1cc(ccc1NC(=O)[C@H](C)Sc2c3c4c(sc3ncn2)CCCC4)[N+](=O)[O-]
ZINC08441354	4.7	-0.78	-13.71	1	7	0	428.539	5	Cc1cc(ccc1NC(=O)[C@@H](C)Sc2c3c4c(sc3ncn2)CCCC4)[N+](=O)[O-]
ZINC00853619	4.45	-2.57	-13.67	1	8	0	449.286	6	Cc1ccc(c(c1)Br)NC(=O)CSc2nnc(o2)c3cccc(c3)[N+](=O)[O-]
ZINC08441353	8.94	3.51	-8.05	0	2	0	469.053	6	CC(C)(C)c1ccc(cc1)CSc2c(c(cc(n2)c3ccccc3)c4ccc(cc4)Cl)C#N
ZINC08441352	6.49	1.93	-10.4	0	6	0	513.993	7	CC1=NC2=C([C@@H](C1C(=O)OC)c3ccc(c(c3)COc4ccc(c(c4)Cl)F)OC)C(=O)CC(C2)(C)C
ZINC08441352	6.49	2.14	-13.29	0	6	0	513.993	7	CC1=C([C@H]([C@H]2C(=N1)CC(CC2=O)(C)C)c3ccc(c(c3)COc4ccc(c(c4)Cl)F)OC)C(=O)OC
ZINC08441352	6.67	3.23	-10.92	0	6	0	513.993	7	CC1(CC2=NC(=C)C([C@H]([C@H]2C(=O)C1)c3ccc(c(c3)COc4ccc(c(c4)Cl)F)OC)C(=O)OC)C
ZINC08441352	6.67	2.02	-11.24	0	6	0	513.993	7	CC1(CC2=NC(=C)C([C@H](C2C(=O)C1)c3ccc(c(c3)COc4ccc(c(c4)Cl)F)OC)C(=O)OC)C
ZINC09232515	2.9	9.66	-59.52	2	10	1	523.562	8	COc1cc(cc(c1OC)OC)[C@@H]2/C(=C(/c3cc4ccccc4o3)\O)/C(=O)C(=O)N2CC[NH+]5CCOCC5
ZINC09232515	1.87	10.37	-58.7	1	10	1	523.562	9	COc1cc(cc(c1OC)OC)[C@@H]2[C@@H](C(=O)C(=O)N2CC[NH+]3CCOCC3)C(=O)c4cc5ccccc5o4
ZINC06076996	6.24	3.28	-10.92	0	4	0	422.937	7	CC(C)OC(=O)CSc1c(cc(n1)c2ccccc2)c3ccc(cc3)Cl)C#N
ZINC08441350	2.84	-0.3	-57.74	2	9	1	507.563	9	CCOc1ccc(cc1OC)[C@H]2C(=C(C(=O)N2CC[NH+]3CCOCC3)O)C(=O)c4cc5ccccc5o4
ZINC08441350	2.26	-0.39	-67.78	1	9	1	507.563	9	CCOc1ccc(cc1OC)[C@H]2[C@@H](C(=O)C(=O)N2CC[NH+]3CCOCC3)C(=O)c4cc5ccccc5o4
ZINC08441349	5.98	-1.16	-14.96	2	6	0	513.022	7	Cc1ccc(cc1)C(=O)NNC(=O)CSc2c(c(cc(n2)c3ccccc3)c4ccc(cc4)Cl)C#N
ZINC08441348	8.08	-0.6	-7.77	2	3	0	475.4	4	c1ccc(cc1)c2cc(c3c(c(sc3n2)C(=O)c4ccc(cc4)Cl)N)c5ccc(cc5)Cl
ZINC08441347	2.84	-0.44	-51.08	2	9	1	507.563	9	CCOc1ccc(cc1OC)[C@@H]2C(=C(C(=O)N2CC[NH+]3CCOCC3)O)C(=O)c4cc5ccccc5o4
ZINC08441347	2.26	-0.66	-60.03	1	9	1	507.563	9	CCOc1ccc(cc1OC)[C@@H]2[C@@H](C(=O)C(=O)N2CC[NH+]3CCOCC3)C(=O)c4cc5ccccc5o4
ZINC00853572	5.17	-3.77	-10.04	2	6	0	507.211	4	Cc1ccccc1NC(=O)CSc2nc3c(c4cc(cc(c4[nH]3)Br)Br)nn2
ZINC08441346	6.33	-1.92	-11.62	3	7	0	511.603	7	COc1ccc(cc1)c2cc(nc3c2c(c(s3)C(=O)Nc4ccc(cc4)OC)N)c5ccc(cc5)OC
ZINC08441345	6.49	2.02	-10.54	0	6	0	513.993	7	CC1=NC2=C([C@H](C1C(=O)OC)c3ccc(c(c3)COc4ccc(c(c4)Cl)F)OC)C(=O)CC(C2)(C)C
ZINC08441345	6.67	3.39	-12.19	0	6	0	513.993	7	CC1(CC2=NC(=C)C([C@@H]([C@@H]2C(=O)C1)c3ccc(c(c3)COc4ccc(c(c4)Cl)F)OC)C(=O)OC)C
ZINC08441345	6.49	1.95	-13.53	0	6	0	513.993	7	CC1=C([C@@H]([C@H]2C(=N1)CC(CC2=O)(C)C)c3ccc(c(c3)COc4ccc(c(c4)Cl)F)OC)C(=O)OC
ZINC08441345	6.67	2.08	-11.09	0	6	0	513.993	7	CC1(CC2=NC(=C)C([C@@H](C2C(=O)C1)c3ccc(c(c3)COc4ccc(c(c4)Cl)F)OC)C(=O)OC)C
ZINC08441344	4.17	0.03	-50.09	2	9	1	549.644	12	CCCCOc1ccc(cc1OC)[C@H]2C(=C(C(=O)N2CCC[NH+]3CCOCC3)O)C(=O)c4cc5ccccc5o4
ZINC08441344	3.59	-0.09	-58.72	1	9	1	549.644	12	CCCCOc1ccc(cc1OC)[C@H]2[C@@H](C(=O)C(=O)N2CCC[NH+]3CCOCC3)C(=O)c4cc5ccccc5o4
ZINC08441343	7.1	-1.31	-11.16	3	6	0	509.631	6	Cc1ccc(cc1C)NC(=O)c2c(c3c(cc(nc3s2)c4ccc(cc4)OC)c5ccc(cc5)OC)N
ZINC08441342	6.39	1.93	-17.95	1	8	0	587.723	11	CCOC(=O)c1c(c(sc1NC(=O)CSc2c(c(cc(n2)c3ccc(cc3)OC)c4ccc(cc4)OC)C#N)C)C
ZINC08441341	6.7	1.76	-17.28	1	6	0	509.631	8	Cc1ccc(cc1C)NC(=O)CSc2c(c(cc(n2)c3ccc(cc3)OC)c4ccc(cc4)OC)C#N
ZINC08441340	4.17	0.05	-56.67	2	9	1	549.644	12	CCCCOc1ccc(cc1OC)[C@@H]2C(=C(C(=O)N2CCC[NH+]3CCOCC3)O)C(=O)c4cc5ccccc5o4
ZINC08441340	3.59	-0.06	-66.08	1	9	1	549.644	12	CCCCOc1ccc(cc1OC)[C@@H]2[C@H](C(=O)C(=O)N2CCC[NH+]3CCOCC3)C(=O)c4cc5ccccc5o4
ZINC08441339	7.14	-1.16	-11.84	3	6	0	509.631	7	CCc1ccccc1NC(=O)c2c(c3c(cc(nc3s2)c4ccc(cc4)OC)c5ccc(cc5)OC)N
ZINC08441338	7.12	0.5	-11.09	3	6	0	549.574	7	COc1ccc(cc1)c2cc(nc3c2c(c(s3)C(=O)Nc4ccccc4C(F)(F)F)N)c5ccc(cc5)OC
ZINC08441337	6.74	1.93	-16.5	1	6	0	509.631	9	CCc1ccccc1NC(=O)CSc2c(c(cc(n2)c3ccc(cc3)OC)c4ccc(cc4)OC)C#N
ZINC08441336	6.66	3.55	-13.85	1	5	0	519.548	8	COc1ccc(cc1)c2cc(c(c(n2)SCC(=O)Nc3ccccc3C(F)(F)F)C#N)c4ccccc4
ZINC08441335	6.86	2.11	-9.95	0	6	0	528.02	8	CCOC(=O)C1[C@@H](C2=C(CC(CC2=O)(C)C)N=C1C)c3ccc(c(c3)COc4ccc(c(c4)Cl)F)OC
ZINC08441335	7.05	0.93	-10.5	0	6	0	528.02	8	CCOC(=O)C1[C@@H](C2C(=NC1=C)CC(CC2=O)(C)C)c3ccc(c(c3)COc4ccc(c(c4)Cl)F)OC
ZINC08441335	6.86	2.03	-13.49	0	6	0	528.02	8	CCOC(=O)C1=C(N=C2CC(CC(=O)C2[C@H]1c3ccc(c(c3)COc4ccc(c(c4)Cl)F)OC)(C)C)C
ZINC08441334	4.74	-0.33	-51.42	2	8	1	553.635	10	c1ccc(cc1)COc2ccc(cc2)[C@@H]3C(=C(C(=O)N3CCC[NH+]4CCOCC4)O)C(=O)c5cc6ccccc6o5
ZINC08441334	4.16	-0.49	-59.11	1	8	1	553.635	10	c1ccc(cc1)COc2ccc(cc2)[C@@H]3[C@H](C(=O)C(=O)N3CCC[NH+]4CCOCC4)C(=O)c5cc6ccccc6o5

Supplementary Table S2:
Detailed table of XP Docking using Schrodinger's Glide module

ZINC_ID	xLogP	ApolarDesolvation	PolarDesolvation	H Donors	H Acceptors	Charge	MolecularWeight	RotatableBonds	SMILES
ZINC08441333	7.04	-1.25	-11.15	3	5	0	479.605	5	Cc1cc(cc(c1)NC(=O)c2c(c3c(cc(nc3s2)c4ccc(cc4)OC)c5ccccc5)N)C
ZINC08441332	7.1	-1.28	-11.17	3	6	0	509.631	6	Cc1cc(cc(c1)NC(=O)c2c(c3c(cc(nc3s2)c4ccc(cc4)OC)c5ccc(cc5)OC)N)C
ZINC08441331	3.79	-0.23	-48.03	2	8	1	503.575	10	C=CCOc1ccc(cc1)[C@H]2C(=C(C(=O)N2CCC[NH+]3CCOCC3)O)C(=O)c4cc5ccccc5o4
ZINC08441331	3.21	-0.41	-55.4	1	8	1	503.575	10	C=CCOc1ccc(cc1)[C@H]2[C@H](C(=O)C(=O)N2CCC[NH+]3CCOCC3)C(=O)c4cc5ccccc5o4
ZINC08441330	8.31	3.69	-9.53	0	3	0	464.634	7	CC(C)(C)c1ccc(cc1)CSc2c(c(cc(n2)c3ccc(cc3)OC)c4ccccc4)C#N
ZINC08441329	6.28	2.1	-17.47	1	6	0	509.631	8	Cc1ccc(cc1)c2cc(c(c(n2)SCC(=O)Nc3cccc(c3)C)C#N)c4ccc(c(c4)OC)OC
ZINC08441328	7.11	3.86	-13.01	1	5	0	533.575	8	Cc1ccc(cc1)c2cc(c(c(n2)SCC(=O)Nc3ccccc3C(F)(F)F)C#N)c4ccc(cc4)OC
ZINC08441327	6.86	2.29	-10.3	0	6	0	528.02	8	CCOC(=O)C1[C@H](C2=C(CC(CC2=O)(C)C)N=C1C)c3ccc(c(c3)COc4ccc(c(c4)Cl)F)OC
ZINC08441327	7.05	1.01	-10.8	0	6	0	528.02	8	CCOC(=O)C1[C@H](C2C(=NC1=C)CC(CC2=O)(C)C)c3ccc(c(c3)COc4ccc(c(c4)Cl)F)OC
ZINC08441327	6.86	1.93	-13.09	0	6	0	528.02	8	CCOC(=O)C1=C(N=C2CC(CC(=O)C2[C@@H]1c3ccc(c(c3)COc4ccc(c(c4)Cl)F)OC)(C)C)C
ZINC08441326	3.79	-0.24	-47.14	2	8	1	503.575	10	C=CCOc1ccc(cc1)[C@@H]2C(=C(C(=O)N2CCC[NH+]3CCOCC3)O)C(=O)c4cc5ccccc5o4
ZINC08441326	3.21	-0.42	-54.31	1	8	1	503.575	10	C=CCOc1ccc(cc1)[C@@H]2[C@@H](C(=O)C(=O)N2CCC[NH+]3CCOCC3)C(=O)c4cc5ccccc5o4
ZINC08441325	7.06	2.86	-9.12	0	3	0	422.553	6	Cc1ccc(cc1)c2cc(c(c(n2)SCc3ccccc3)C#N)c4ccc(cc4)OC
ZINC08441323	7.51	0.8	-10.38	3	5	0	533.575	6	Cc1ccc(cc1)c2cc(c3c(c(sc3n2)C(=O)Nc4ccccc4C(F)(F)F)N)c5ccc(cc5)OC
ZINC08441322	4.58	-0.48	-47.54	2	8	1	519.618	11	CCCCOc1ccc(cc1)[C@H]2C(=C(C(=O)N2CCC[NH+]3CCOCC3)O)C(=O)c4cc5ccccc5o4
ZINC08441322	4	-0.66	-54.91	1	8	1	519.618	11	CCCCOc1ccc(cc1)[C@H]2[C@H](C(=O)C(=O)N2CCC[NH+]3CCOCC3)C(=O)c4cc5ccccc5o4
ZINC08441321	7.77	2.12	-15.25	1	4	0	499.639	6	Cc1ccc(cc1)c2cc(c(c(n2)SCC(=O)Nc3c(ccc4c3cccc4)C)C#N)c5ccccc5
ZINC08441320	5.92	0.66	-10.7	0	6	0	514.416	7	CCOC(=O)C1[C@@H](C2=C(CC(CC2=O)(C)C)N=C1C)c3ccc(o3)COc4cccc(c4)Br
ZINC08441320	6.1	-0.15	-10.58	0	6	0	514.416	7	CCOC(=O)C1[C@@H](C2C(=NC1=C)CC(CC2=O)(C)C)c3ccc(o3)COc4cccc(c4)Br
ZINC08441320	5.92	0.87	-13.26	0	6	0	514.416	7	CCOC(=O)C1=C(N=C2CC(CC(=O)C2[C@H]1c3ccc(o3)COc4cccc(c4)Br)(C)C)C
ZINC08441319	4.58	-0.49	-47.11	2	8	1	519.618	11	CCCCOc1ccc(cc1)[C@@H]2C(=C(C(=O)N2CCC[NH+]3CCOCC3)O)C(=O)c4cc5ccccc5o4
ZINC08441319	4	-0.67	-54.33	1	8	1	519.618	11	CCCCOc1ccc(cc1)[C@@H]2[C@@H](C(=O)C(=O)N2CCC[NH+]3CCOCC3)C(=O)c4cc5ccccc5o4
ZINC08441318	6.26	-1.12	-13.14	3	7	0	525.63	7	Cc1ccc(cc1)c2cc(c3c(c(sc3n2)C(=O)Nc4ccccc4OC)N)c5ccc(c(c5)OC)OC
ZINC08441317	8.17	-1.16	-10.38	3	4	0	499.639	4	Cc1ccc(cc1)c2cc(c3c(c(sc3n2)C(=O)Nc4c(ccc5c4cccc5)C)N)c6ccccc6
ZINC08441316	1.85	-1.77	-45.62	2	8	1	448.499	7	c1ccc2c(c1)cc(o2)C(=O)C3=C(C(=O)N([C@@H]3c4cccnc4)CCC[NH+]5CCOCC5)O
ZINC08441316	1.27	-1.94	-53.04	1	8	1	448.499	7	c1ccc2c(c1)cc(o2)C(=O)[C@@H]3[C@H](N(C(=O)C3=O)CCC[NH+]4CCOCC4)c5cccnc5
ZINC08441315	7.03	2.06	-15	1	4	0	463.606	6	Cc1ccc(cc1)c2cc(c(c(n2)SCC(=O)Nc3cc(cc(c3)C)C)C#N)c4ccccc4
ZINC00853507	6.25	1.57	-17.19	1	6	0	495.604	8	Cc1ccc(cc1)c2cc(c(c(n2)SCC(=O)Nc3cc(ccc3OC)OC)C#N)c4ccccc4
ZINC08441314	6.11	1.9	-21.23	1	5	0	477.589	7	Cc1ccc(cc1)c2cc(c(c(n2)SCC(=O)Nc3ccc(cc3)C(=O)C)C#N)c4ccccc4
ZINC08441313	5.92	0.81	-10.74	0	6	0	514.416	7	CCOC(=O)C1[C@H](C2=C(CC(CC2=O)(C)C)N=C1C)c3ccc(o3)COc4cccc(c4)Br
ZINC08441313	6.1	-0.48	-13.3	0	6	0	514.416	7	CCOC(=O)C1[C@H](C2C(=NC1=C)CC(CC2=O)(C)C)c3ccc(o3)COc4cccc(c4)Br
ZINC08441313	5.92	0.54	-15.77	0	6	0	514.416	7	CCOC(=O)C1=C(N=C2CC(CC(=O)C2[C@@H]1c3ccc(o3)COc4cccc(c4)Br)(C)C)C
ZINC08441312	3.11	-0.2	-56.77	2	9	1	521.59	10	CCOc1ccc(cc1OC)[C@H]2C(=C(C(=O)N2CCC[NH+]3CCOCC3)O)C(=O)c4cc5ccccc5o4
ZINC08441312	2.53	-0.29	-66.02	1	9	1	521.59	10	CCOc1ccc(cc1OC)[C@H]2[C@@H](C(=O)C(=O)N2CCC[NH+]3CCOCC3)C(=O)c4cc5ccccc5o4
ZINC00853504	4.76	-3.11	-12.79	1	6	0	454.733	6	COc1ccc(cc1NC(=O)CSc2nnc(o2)c3ccccc3Br)Cl
ZINC08441311	4	-2.2	-14.11	1	8	0	435.259	6	c1ccc(c(c1)c2nnc(o2)SCC(=O)Nc3ccccc3[N+](=O)[O-])Br
ZINC08441310	3.11	-0.15	-56.73	2	9	1	521.59	10	CCOc1ccc(cc1OC)[C@@H]2C(=C(C(=O)N2CCC[NH+]3CCOCC3)O)C(=O)c4cc5ccccc5o4
ZINC08441310	2.53	-0.25	-66.08	1	9	1	521.59	10	CCOc1ccc(cc1OC)[C@@H]2[C@H](C(=O)C(=O)N2CCC[NH+]3CCOCC3)C(=O)c4cc5ccccc5o4
ZINC08441309	7.08	2.19	-14.33	1	4	0	463.606	7	CCc1ccccc1NC(=O)CSc2c(c(cc(n2)c3ccc(cc3)C)c4ccccc4)C#N
ZINC08441308	6.86	0.09	-10.84	0	6	0	560.872	8	CCOC(=O)C1[C@@H](C2=C(CCCC2=O)N=C1C)c3ccc(c(c3)COc4ccc(cc4Br)Cl)OC
ZINC08441308	7.04	-1.02	-12.19	0	6	0	560.872	8	CCOC(=O)C1[C@@H](C2C(=NC1=C)CCCC2=O)c3ccc(c(c3)COc4ccc(cc4Br)Cl)OC
ZINC08441308	6.86	-0.1	-14.21	0	6	0	560.872	8	CCOC(=O)C1=C(N=C2CCCC(=O)C2[C@H]1c3ccc(c(c3)COc4ccc(cc4Br)Cl)OC)C
ZINC08441307	7.06	3.84	-12.29	1	4	0	503.549	7	Cc1ccc(cc1)c2cc(c(c(n2)SCC(=O)Nc3ccccc3C(F)(F)F)C#N)c4ccccc4
ZINC00853499	4.96	-2.33	-11.88	1	5	0	418.316	6	CCc1ccccc1NC(=O)CSc2nnc(o2)c3ccccc3Br
ZINC00702997	4.84	1.89	-16.25	1	8	0	535.552	9	COc1cc(ccc1OCc2ccccc2)[C@H]3C(=C(C(=O)N3Cc4ccco4)O)C(=O)c5cc6ccccc6o5
ZINC00702997	4.26	1.67	-21.45	0	8	0	535.552	9	COc1cc(ccc1OCc2ccccc2)[C@H]3[C@H](C(=O)C(=O)N3Cc4ccco4)C(=O)c5cc6ccccc6o5
ZINC08441306	6.7	4.19	-14.88	1	6	0	563.601	9	Cc1ccc(cc1)c2cc(c(c(n2)SCC(=O)Nc3ccccc3C(F)(F)F)C#N)c4ccc(c(c4)OC)OC
ZINC00702998	4.84	1.78	-16.47	1	8	0	535.552	9	COc1cc(ccc1OCc2ccccc2)[C@@H]3C(=C(C(=O)N3Cc4ccco4)O)C(=O)c5cc6ccccc6o5
ZINC00702998	4.26	1.57	-23.2	0	8	0	535.552	9	COc1cc(ccc1OCc2ccccc2)[C@@H]3[C@@H](C(=O)C(=O)N3Cc4ccco4)C(=O)c5cc6ccccc6o5
ZINC06245730	8.45	18.71	-11.9	0	4	0	523.746	7	Cc1ccc(cc1)c2cc(c(c(n2)SCC(=O)N(C3CCCCC3)C4CCCCC4)C#N)c5ccccc5
ZINC08441305	6.86	0.1	-10.81	0	6	0	560.872	8	CCOC(=O)C1[C@H](C2=C(CCCC2=O)N=C1C)c3ccc(c(c3)COc4ccc(cc4Br)Cl)OC
ZINC08441305	7.04	-1.01	-12.3	0	6	0	560.872	8	CCOC(=O)C1[C@H](C2C(=NC1=C)CCCC2=O)c3ccc(c(c3)COc4ccc(cc4Br)Cl)OC
ZINC08441305	6.86	-0.09	-14.31	0	6	0	560.872	8	CCOC(=O)C1=C(N=C2CCCC(=O)C2[C@@H]1c3ccc(c(c3)COc4ccc(cc4Br)Cl)OC)C
ZINC00702989	5.33	1.59	-14.46	1	7	0	491.499	7	c1ccc(cc1)Oc2cccc(c2)[C@H]3C(=C(C(=O)N3Cc4ccco4)O)C(=O)c5cc6ccccc6o5
ZINC00702989	4.74	1.44	-18.74	0	7	0	491.499	7	c1ccc(cc1)Oc2cccc(c2)[C@H]3[C@@H](C(=O)C(=O)N3Cc4ccco4)C(=O)c5cc6ccccc6o5
ZINC08441304	7.37	1.78	-15.7	1	4	0	485.612	6	Cc1ccc(cc1)c2cc(c(c(n2)SCC(=O)Nc3cccc4c3cccc4)C#N)c5ccccc5
ZINC00702990	5.33	2.11	-14.93	1	7	0	491.499	7	c1ccc(cc1)Oc2cccc(c2)[C@@H]3C(=C(C(=O)N3Cc4ccco4)O)C(=O)c5cc6ccccc6o5
ZINC00702990	4.74	1.9	-19.32	0	7	0	491.499	7	c1ccc(cc1)Oc2cccc(c2)[C@@H]3[C@@H](C(=O)C(=O)N3Cc4ccco4)C(=O)c5cc6ccccc6o5
ZINC00702987	5.3	2.11	-13.5	1	6	0	455.51	6	CC(C)(C)c1ccc(cc1)[C@H]2C(=C(C(=O)N2Cc3ccco3)O)C(=O)c4cc5ccccc5o4
ZINC00702987	4.72	1.94	-19.98	0	6	0	455.51	6	CC(C)(C)c1ccc(cc1)[C@H]2[C@H](C(=O)C(=O)N2Cc3ccco3)C(=O)c4cc5ccccc5o4
ZINC08441303	6.54	2.04	-10.73	0	6	0	489.612	8	CCOC(=O)C1[C@@H](C2=C(CC(CC2=O)(C)C)N=C1C)c3ccc(c(c3)COc4ccc(cc4)C)OC

Supplementary Table S2:
Detailed table of XP Docking using Schrodinger's Glide module

ZINC_ID	xLogP	ApolarDesolvation	PolarDesolvation	H Donors	H Acceptors	Charge	MolecularWeight	RotatableBonds	SMILES
ZINC08441303	6.72	1.1	-11.41	0	6	0	489.612	8	CCOC(=O)C1[C@@H](C2C(=NC1=C)CC(CC2=O)(C)C)c3ccc(c(c3)COc4ccc(cc4)C)OC
ZINC08441303	6.54	2.02	-13.75	0	6	0	489.612	8	CCOC(=O)C1=C(N=C2CC(CC(=O)C2[C@H]1c3ccc(c(c3)COc4ccc(cc4)C)OC)(C)C)C
ZINC08441302	6.64	1.45	-16.41	1	5	0	479.605	8	CCOc1ccc(cc1)NC(=O)CSc2c(c(cc(n2)c3ccc(cc3)C)c4ccccc4)C#N
ZINC00853498	6.22	1.56	-14.35	1	5	0	465.578	7	Cc1ccc(cc1)c2cc(c(c(n2)SCC(=O)Nc3ccccc3OC)C#N)c4ccccc4
ZINC00853497	4.94	-0.67	-11.21	1	5	0	458.259	6	c1ccc(c(c1)c2nnc(o2)SCC(=O)Nc3ccccc3C(F)(F)F)Br
ZINC00702988	5.3	2.18	-12.15	1	6	0	455.51	6	CC(C)(C)c1ccc(cc1)[C@@H]2C(=C(C(=O)N2Cc3ccco3)O)C(=O)c4cc5ccccc5o4
ZINC00702988	4.72	2.02	-16.58	0	6	0	455.51	6	CC(C)(C)c1ccc(cc1)[C@@H]2[C@H](C(=O)C(=O)N2Cc3ccco3)C(=O)c4cc5ccccc5o4
ZINC00853495	4.52	-1.23	-13.48	2	6	0	449.985	8	CCOC(=O)c1c(c(sc1NC(=O)CSc2[nH]cc(n2)c3ccc(cc3)Cl)C)C
ZINC00853495	4.52	-0.99	-46.23	3	6	1	450.993	8	CCOC(=O)c1c(c(sc1NC(=O)CSc2[nH]cc([nH+]2)c3ccc(cc3)Cl)C)C
ZINC06445732	6.36	13.61	-7.44	1	4	0	432.033	6	c1cc(ccc1c2cnc([nH]2)SCC(=O)N(C3CCCCC3)C4CCCCC4)Cl
ZINC06445732	6.36	14.06	-42.35	2	4	1	433.041	6	c1cc(ccc1c2c[nH+]c([nH]2)SCC(=O)N(C3CCCCC3)C4CCCCC4)Cl
ZINC00853494	5.89	-2	-10.26	2	4	0	447.174	5	c1cc(ccc1c2c[nH]c(n2)SCC(=O)Nc3c(cc(cc3Cl)Cl)Cl)Cl
ZINC00853494	5.89	-1.76	-42.21	3	4	1	448.182	5	c1cc(ccc1c2c[nH]c([nH+]2)SCC(=O)Nc3c(cc(cc3Cl)Cl)Cl)Cl
ZINC06135667	4.78	11.22	-14.61	1	3	0	314.388	3	CCn1c2ccccc2c3c1ccc(c3)NC(=O)c4ccccc4
ZINC08441301	4.89	1.24	-17.5	1	8	0	513.594	12	CCCCCOc1ccc(cc1)[C@@H]2C(=C(C(=O)N2CCCn3ccnc3)O)C(=O)c4cc5ccccc5o4
ZINC08441301	4.31	1.06	-23.39	0	8	0	513.594	12	CCCCCOc1ccc(cc1)[C@@H]2[C@@H](C(=O)C(=O)N2CCCn3ccnc3)C(=O)c4cc5ccccc5o4
ZINC08441301	5.34	1.15	-59.14	2	8	1	514.602	11	CCCCCOc1ccc(cc1)[C@@H]2C(=C(c3cc4ccccc4o3)O)C(=O)C(=O)N2CCCn5cc[nH+]c5
ZINC08441301	4.31	1.35	-55.13	1	8	1	514.602	12	CCCCCOc1ccc(cc1)[C@@H]2[C@@H](C(=O)C(=O)N2CCCn3cc[nH+]c3)C(=O)c4cc5ccccc5o4
ZINC08441300	6.54	2.05	-10.76	0	6	0	489.612	8	CCOC(=O)C1[C@H](C2=C(CC(CC2=O)(C)C)N=C1C)c3ccc(c(c3)COc4ccc(cc4)C)OC
ZINC08441300	6.72	0.79	-11.34	0	6	0	489.612	8	CCOC(=O)C1[C@H](C2C(=NC1=C)CC(CC2=O)(C)C)c3ccc(c(c3)COc4ccc(cc4)C)OC
ZINC08441300	6.54	1.69	-13.73	0	6	0	489.612	8	CCOC(=O)C1=C(N=C2CC(CC(=O)C2[C@@H]1c3ccc(c(c3)COc4ccc(cc4)C)OC)(C)C)C
ZINC08997625	2.98	-1.03	-58.06	1	8	-1	446.414	4	Cc1cc(c(n1c2cccc(c2)C(=O)[O-])C)/C=C/3\C(=NC(=O)N(C3=O)c4ccc(cc4)F)O
ZINC08441299	4.14	1.49	-22.78	1	9	0	563.61	11	COc1cc(ccc1OCc2ccccc2)[C@@H]3C(=C(C(=O)N3CCCn4ccnc4)O)C(=O)c5cc6ccccc6o5
ZINC08441299	3.55	1.39	-29.71	0	9	0	563.61	11	COc1cc(ccc1OCc2ccccc2)[C@@H]3[C@H](C(=O)C(=O)N3CCCn4ccnc4)C(=O)c5cc6ccccc6o5
ZINC08441299	4.58	2.04	-74.96	2	9	1	564.618	10	COc1cc(ccc1OCc2ccccc2)[C@@H]3/C(=C(/c4cc5ccccc5o4)\O)/C(=O)C(=O)N3CCCn6cc[nH+]c6
ZINC08441299	3.55	1.67	-64.2	1	9	1	564.618	11	COc1cc(ccc1OCc2ccccc2)[C@@H]3[C@H](C(=O)C(=O)N3CCCn4cc[nH+]c4)C(=O)c5cc6ccccc6o5
ZINC13569448	3.92	10.25	-49.54	0	6	-1	430.459	3	Cc1ccc(c(c1)C)n2c(cc(c2C)/C=C/3\C(=NC(=O)N(C3=O)c4ccc(cc4)F)[O-])C
ZINC08914319	3.9	10.12	-49.6	0	6	-1	430.459	3	Cc1cccc(c1C)n2c(cc(c2C)/C=C/3\C(=NC(=O)N(C3=O)c4ccc(cc4)F)[O-])C
ZINC08441298	4.19	1.85	-21.35	1	9	0	543.62	12	CC(C)CCOc1ccc(cc1OC)[C@H]2C(=C(C(=O)N2CCCn3ccnc3)O)C(=O)c4cc5ccccc5o4
ZINC08441298	3.61	1.75	-28.14	0	9	0	543.62	12	CC(C)CCOc1ccc(cc1OC)[C@H]2[C@@H](C(=O)C(=O)N2CCCn3ccnc3)C(=O)c4cc5ccccc5o4
ZINC08441298	4.64	2.4	-73.39	2	9	1	544.628	11	CC(C)CCOc1ccc(cc1OC)[C@H]2/C(=C(/c3cc4ccccc4o3)\O)/C(=O)C(=O)N2CCCn5cc[nH+]c5
ZINC08441298	3.61	2.03	-62.62	1	9	1	544.628	12	CC(C)CCOc1ccc(cc1OC)[C@H]2[C@@H](C(=O)C(=O)N2CCCn3cc[nH+]c3)C(=O)c4cc5ccccc5o4
ZINC08441297	4.38	3.05	-13.44	0	6	0	521.642	6	CCOc1ccc(cc1)N2C(=O)/C(=C\c3cc(n(c3C)c4ccccc4)C)/C(=O)N(C2=S)c5ccccc5
ZINC08441296	3.6	1.25	-17.89	1	8	0	483.524	10	C=CCOc1ccc(cc1)[C@H]2C(=C(C(=O)N2CCCn3ccnc3)O)C(=O)c4cc5ccccc5o4
ZINC08441296	3.01	1.06	-24.67	0	8	0	483.524	10	C=CCOc1ccc(cc1)[C@H]2[C@H](C(=O)C(=O)N2CCCn3ccnc3)C(=O)c4cc5ccccc5o4
ZINC08441296	4.04	1.15	-56	2	8	1	484.532	9	C=CCOc1ccc(cc1)[C@H]2C(=C(c3cc4ccccc4o3)O)C(=O)C(=O)N2CCCn5cc[nH+]c5
ZINC08441296	3.01	1.34	-50.37	1	8	1	484.532	10	C=CCOc1ccc(cc1)[C@H]2[C@H](C(=O)C(=O)N2CCCn3cc[nH+]c3)C(=O)c4cc5ccccc5o4
ZINC08441295	3.8	0.71	-52.2	2	9	1	473.575	7	CCOC(=O)c1c2c(sc1NC(=O)c3cc(ccc3N4CC[NH+](CC4)C)[N+](=O)[O-])CCCC2
ZINC08441294	5.54	0.59	-11.2	0	6	0	500.389	6	CC1=NC2=C([C@@H](C1C(=O)OC)c3ccc(o3)COc4cccc(c4)Br)C(=O)CC(C2)(C)C
ZINC08441294	5.54	0.61	-15.91	0	6	0	500.389	6	CC1=C([C@H]([C@@H]2C(=N1)CC(CC2=O)(C)C)c3ccc(o3)COc4cccc(c4)Br)C(=O)OC
ZINC08441294	5.72	1.88	-14.62	0	6	0	500.389	6	CC1(CC2=NC(=C)C([C@H]([C@H]2C(=O)C1)c3ccc(o3)COc4cccc(c4)Br)C(=O)OC)C
ZINC08441294	5.72	0.64	-12.98	0	6	0	500.389	6	CC1(CC2=NC(=C)C([C@H](C2C(=O)C1)c3ccc(o3)COc4cccc(c4)Br)C(=O)OC)C
ZINC08441292	3.6	1.37	-17.82	1	8	0	483.524	10	C=CCOc1ccc(cc1)[C@@H]2C(=C(C(=O)N2CCCn3ccnc3)O)C(=O)c4cc5ccccc5o4
ZINC08441292	3.01	1.19	-23.77	0	8	0	483.524	10	C=CCOc1ccc(cc1)[C@@H]2[C@@H](C(=O)C(=O)N2CCCn3ccnc3)C(=O)c4cc5ccccc5o4
ZINC08441292	4.04	1.28	-59.54	2	8	1	484.532	9	C=CCOc1ccc(cc1)[C@@H]2C(=C(c3cc4ccccc4o3)O)C(=O)C(=O)N2CCCn5cc[nH+]c5
ZINC08441292	3.01	1.48	-55.48	1	8	1	484.532	10	C=CCOc1ccc(cc1)[C@@H]2[C@@H](C(=O)C(=O)N2CCCn3cc[nH+]c3)C(=O)c4cc5ccccc5o4
ZINC02063954	8.01	18.33	-11.61	0	6	0	525.674	8	CCOC(=O)C1=C(N=c2n(c(=O)/c(=C/c3cn(c4c3cccc4)CC=C)/s2)[C@@H]1c5ccc(cc5)C(C)C)C
ZINC00702965	4.62	1.65	-19.12	1	8	0	519.557	9	c1ccc(cc1)Oc2cccc(c2)[C@H]3C(=C(C(=O)N3CCCn4ccnc4)O)C(=O)c5cc6ccccc6o5
ZINC00702965	4.04	1.46	-24.15	0	8	0	519.557	9	c1ccc(cc1)Oc2cccc(c2)[C@H]3[C@H](C(=O)C(=O)N3CCCn4ccnc4)C(=O)c5cc6ccccc6o5
ZINC00702965	5.07	1.55	-60.29	2	8	1	520.565	8	c1ccc(cc1)Oc2cccc(c2)[C@H]3C(=C(c4cc5ccccc5o4)O)C(=O)C(=O)N3CCCn6cc[nH+]c6
ZINC00702965	4.04	1.74	-57.6	1	8	1	520.565	9	c1ccc(cc1)Oc2cccc(c2)[C@H]3[C@H](C(=O)C(=O)N3CCCn4cc[nH+]c4)C(=O)c5cc6ccccc6o5
ZINC00852701	5.51	-1.54	-9.79	2	4	0	425.724	4	c1cc(sc1)C(=O)Nc2ccc(c(c2)NC(=O)c3ccc(cc3Cl)Cl)Cl
ZINC16956755	5.5	11.99	-53.95	0	4	-1	465.298	4	Cc1ccc(cc1)C(=O)C2=C(C(=O)N([C@H]2c3ccc(cc3)Br)c4ccc(cc4)F)[O-]
ZINC16956755	4.91	12.23	-8.97	0	4	0	466.306	4	Cc1ccc(cc1)C(=O)[C@H]2[C@@H](N(C(=O)C2=O)c3ccc(cc3)F)c4ccc(cc4)Br
ZINC16956754	5.5	11.93	-53.96	0	4	-1	465.298	4	Cc1ccc(cc1)C(=O)C2=C(C(=O)N([C@@H]2c3ccc(cc3)Br)c4ccc(cc4)F)[O-]
ZINC16956754	4.91	12.3	-9.01	0	4	0	466.306	4	Cc1ccc(cc1)C(=O)[C@@H]2[C@H](N(C(=O)C2=O)c3ccc(cc3)F)c4ccc(cc4)Br
ZINC00702966	4.62	1.78	-18.59	1	8	0	519.557	9	c1ccc(cc1)Oc2cccc(c2)[C@@H]3C(=C(C(=O)N3CCCn4ccnc4)O)C(=O)c5cc6ccccc6o5
ZINC00702966	4.04	1.57	-23.46	0	8	0	519.557	9	c1ccc(cc1)Oc2cccc(c2)[C@@H]3[C@@H](C(=O)C(=O)N3CCCn4ccnc4)C(=O)c5cc6ccccc6o5
ZINC00702966	5.07	1.69	-55.79	2	8	1	520.565	8	c1ccc(cc1)Oc2cccc(c2)[C@@H]3C(=C(c4cc5ccccc5o4)O)C(=O)C(=O)N3CCCn6cc[nH+]c6
ZINC00702966	4.04	1.85	-54.55	1	8	1	520.565	9	c1ccc(cc1)Oc2cccc(c2)[C@@H]3[C@@H](C(=O)C(=O)N3CCCn4cc[nH+]c4)C(=O)c5cc6ccccc6o5
ZINC08441288	1.87	1.62	-53.17	1	8	1	431.509	8	COc1cc(cc(c1)OC)C[NH+]2CCN(CC2)C(=O)c3cc(c(c(c3)OC)OC)OC

Supplementary Table S2:
Detailed table of XP Docking using Schrodinger's Glide module

ZINC_ID	xLogP	ApolarDesolvation	PolarDesolvation	H Donors	H Acceptors	Charge	MolecularWeight	RotatableBonds	SMILES
ZINC08441287	4.48	1.66	-21.4	1	9	0	543.62	13	CCCCCOc1ccc(cc1OC)[C@H]2C(=C(C(=O)N2CCCn3ccnc3)O)C(=O)c4cc5ccccc5o4
ZINC08441287	3.9	1.55	-28.18	0	9	0	543.62	13	CCCCCOc1ccc(cc1OC)[C@H]2[C@@H](C(=O)C(=O)N2CCCn3ccnc3)C(=O)c4cc5ccccc5o4
ZINC08441287	4.93	2.21	-73.25	2	9	1	544.628	12	CCCCCOc1ccc(cc1OC)[C@H]2/C(=C(/c3cc4ccccc4o3)\O)/C(=O)C(=O)N2CCCn5cc[nH+]c5
ZINC08441287	3.9	1.83	-62.5	1	9	1	544.628	13	CCCCCOc1ccc(cc1OC)[C@H]2[C@@H](C(=O)C(=O)N2CCCn3cc[nH+]c3)C(=O)c4cc5ccccc5o4
ZINC08441285	5.54	0.58	-10.34	0	6	0	500.389	6	CC1=NC2=C([C@H](C1C(=O)OC)c3ccc(o3)COc4cccc(c4)Br)C(=O)CC(C2)(C)C
ZINC08441285	5.72	0.56	-11.17	0	6	0	500.389	6	CC1(CC2=NC(=C)C([C@@H](C2C(=O)C1)c3ccc(o3)COc4cccc(c4)Br)C(=O)OC)C
ZINC08441285	5.72	1.81	-10.77	0	6	0	500.389	6	CC1(CC2=NC(=C)C([C@@H]([C@@H]2C(=O)C1)c3ccc(o3)COc4cccc(c4)Br)C(=O)OC)C
ZINC08441285	5.54	0.62	-12.59	0	6	0	500.389	6	CC1=C([C@@H]([C@@H]2C(=N1)CC(CC2=O)(C)C)c3ccc(o3)COc4cccc(c4)Br)C(=O)OC
ZINC08441284	7.05	2.25	-9.08	0	6	0	492.399	8	CC1=NC(=C([C@@H](C1C(=O)OC(C)C)c2ccc(o2)c3cc(ccc3Cl)Cl)C(=O)OC(C)C)C
ZINC08441284	7.24	2.28	-9.36	0	6	0	492.399	8	CC1=NC(=C)C([C@H](C1C(=O)OC(C)C)c2ccc(o2)c3cc(ccc3Cl)Cl)C(=O)OC(C)C
ZINC08441284	7.24	3.7	-16.14	0	6	0	492.399	8	CC1=NC(=C)C([C@H]([C@H]1C(=O)OC(C)C)c2ccc(o2)c3cc(ccc3Cl)Cl)C(=O)OC(C)C
ZINC08441284	7.05	2.32	-10.18	0	6	0	492.399	8	CC1=NC(=C([C@H]([C@H]1C(=O)OC(C)C)c2ccc(o2)c3cc(ccc3Cl)Cl)C(=O)OC(C)C)C
ZINC08441283	4.48	1.64	-22.32	1	9	0	543.62	13	CCCCCOc1ccc(cc1OC)[C@@H]2C(=C(C(=O)N2CCCn3ccnc3)O)C(=O)c4cc5ccccc5o4
ZINC08441283	3.9	1.42	-30.29	0	9	0	543.62	13	CCCCCOc1ccc(cc1OC)[C@@H]2[C@@H](C(=O)C(=O)N2CCCn3ccnc3)C(=O)c4cc5ccccc5o4
ZINC08441283	4.93	2.2	-61.97	2	9	1	544.628	12	CCCCCOc1ccc(cc1OC)[C@@H]2/C(=C(/c3cc4ccccc4o3)\O)/C(=O)C(=O)N2CCCn5cc[nH+]c5
ZINC08441283	3.9	1.71	-53.96	1	9	1	544.628	13	CCCCCOc1ccc(cc1OC)[C@@H]2[C@@H](C(=O)C(=O)N2CCCn3cc[nH+]c3)C(=O)c4cc5ccccc5o4
ZINC08441282	4.66	0.13	-20.71	2	7	0	506.606	8	Cc1ccc(cc1)C(=O)N/C(=C\c2cn(nc2c3ccccc3)c4ccccc4)/C(=O)NC[C@H]5CCCO5
ZINC08897840	2.9	12.11	-63.9	0	7	-1	426.452	7	c1ccc(cc1)[C@H]2C(=C(C(=O)N2CCCn3ccnc3)[O-])C(=O)c4cc5ccccc5o4
ZINC08897840	2.31	13.89	-45.42	1	7	1	428.468	7	c1ccc(cc1)[C@H]2[C@@H](C(=O)C(=O)N2CCCn3cc[nH+]c3)C(=O)c4cc5ccccc5o4
ZINC08897840	3.34	12.73	-47.11	2	7	1	428.468	6	c1ccc(cc1)[C@H]2/C(=C(\c3cc4ccccc4o3)/O)/C(=O)C(=O)N2CCCn5cc[nH+]c5
ZINC08897840	2.31	13.37	-19.04	0	7	0	427.46	7	c1ccc(cc1)[C@H]2[C@@H](C(=O)C(=O)N2CCCn3ccnc3)C(=O)c4cc5ccccc5o4
ZINC08897840	3.34	12.21	-21.77	1	7	0	427.46	6	c1ccc(cc1)[C@H]2/C(=C(\c3cc4ccccc4o3)/O)/C(=O)C(=O)N2CCCn5ccnc5
ZINC08897840	2.9	12.59	-75.65	1	7	0	427.46	7	c1ccc(cc1)[C@H]2C(=C(C(=O)N2CCCn3cc[nH+]c3)[O-])C(=O)c4cc5ccccc5o4
ZINC08441281	4.66	-0.19	-17.23	2	7	0	506.606	8	Cc1ccc(cc1)C(=O)N/C(=C\c2cn(nc2c3ccccc3)c4ccccc4)/C(=O)NC[C@@H]5CCCO5
ZINC02063926	8.42	2.37	-10.21	0	7	0	543.689	10	CCCCCCCN1c2ccccc2/C(=c/3\c(=O)n4c(=NC(=C([C@H]4c5ccccc5)C(=O)OCC)C)s3)/C1=O
ZINC08897821	2.9	12.11	-64.01	0	7	-1	426.452	7	c1ccc(cc1)[C@@H]2C(=C(C(=O)N2CCCn3ccnc3)[O-])C(=O)c4cc5ccccc5o4
ZINC08897821	3.34	12.72	-47.09	2	7	1	428.468	6	c1ccc(cc1)[C@@H]2/C(=C(\c3cc4ccccc4o3)/O)/C(=O)C(=O)N2CCCn5cc[nH+]c5
ZINC08897821	3.34	12.21	-21.89	1	7	0	427.46	6	c1ccc(cc1)[C@@H]2/C(=C(\c3cc4ccccc4o3)/O)/C(=O)C(=O)N2CCCn5ccnc5
ZINC08897821	2.9	12.59	-75.7	1	7	0	427.46	7	c1ccc(cc1)[C@@H]2C(=C(C(=O)N2CCCn3cc[nH+]c3)[O-])C(=O)c4cc5ccccc5o4
ZINC02063928	8.42	2.49	-10.22	0	7	0	543.689	10	CCCCCCCN1c2ccccc2/C(=c/3\c(=O)n4c(=NC(=C([C@@H]4c5ccccc5)C(=O)OCC)C)s3)/C1=O
ZINC08441279	7.33	0.35	-18.8	2	5	0	497.422	6	Cc1ccc(cc1)C(=O)N/C(=C\c2ccc(o2)c3cc(ccc3Cl)Cl)/C(=O)NC4CCCCC4
ZINC08441278	6.12	0.22	-12.46	1	7	0	539.638	7	Cc1ccc(cc1)CSc2nnc(s2)N3[C@H](C(=C(C3=O)O)C(=O)c4cc5ccccc5o4)c6ccccc6
ZINC08441278	5.54	0.06	-13.49	0	7	0	539.638	7	Cc1ccc(cc1)CSc2nnc(s2)N3[C@H]([C@@H](C(=O)C3=O)C(=O)c4cc5ccccc5o4)c6ccccc6
ZINC08441277	6.46	-0.28	-13.13	2	6	0	523.427	7	COc1ccc(cc1)C(=O)N/C(=C\c2ccc(o2)c3ccc(cc3)Br)/C(=O)NC4CCCCC4
ZINC08441276	4.71	2.83	-14.63	0	7	0	454.529	6	CC1=C([C@H](n2c(=O)/c(=C\c3cccs3)/sc2=N1)c4ccc(cc4)OC(=O)C)C(=O)OC
ZINC08441272	6.12	0.14	-12.49	1	7	0	539.638	7	Cc1ccc(cc1)CSc2nnc(s2)N3[C@@H](C(=C(C3=O)O)C(=O)c4cc5ccccc5o4)c6ccccc6
ZINC08441272	5.54	-0.03	-14.45	0	7	0	539.638	7	Cc1ccc(cc1)CSc2nnc(s2)N3[C@@H]([C@@H](C(=O)C3=O)C(=O)c4cc5ccccc5o4)c6ccccc6
ZINC08441271	6.6	4.17	-12.84	0	6	0	450.56	6	CCOC(=O)C1=C(N=c2n(c(=O)/c(=C\c3ccco3)/s2)[C@H]1c4ccc(cc4)C(C)(C)C)C
ZINC08441270	5.79	0.64	-13.31	1	7	0	543.601	7	c1ccc(cc1)CSc2nnc(s2)N3[C@H](C(=C(C3=O)O)C(=O)c4cc5ccccc5o4)c6ccccc6F
ZINC08441270	5.2	0.46	-13.29	0	7	0	543.601	7	c1ccc(cc1)CSc2nnc(s2)N3[C@H]([C@@H](C(=O)C3=O)C(=O)c4cc5ccccc5o4)c6ccccc6F
ZINC08441269	6.6	4.16	-12.82	0	6	0	450.56	6	CCOC(=O)C1=C(N=c2n(c(=O)/c(=C\c3ccco3)/s2)[C@@H]1c4ccc(cc4)C(C)(C)C)C
ZINC08441268	5.89	0.74	-12.93	0	6	0	514.416	7	CCOC(=O)C1[C@@H](C2=C(CC(CC2=O)(C)C)N=C1C)c3ccc(o3)COc4ccccc4Br
ZINC08441268	6.08	-0.07	-12.27	0	6	0	514.416	7	CCOC(=O)C1[C@@H](C2C(=NC1=C)CC(CC2=O)(C)C)c3ccc(o3)COc4ccccc4Br
ZINC08441268	5.89	0.94	-14.42	0	6	0	514.416	7	CCOC(=O)C1=C(N=C2CC(CC(=O)C2[C@H]1c3ccc(o3)COc4ccccc4Br)(C)C)C
ZINC08441267	5	1.67	-34.5	1	8	0	459.502	6	CC1CCN(CC1)C(=O)/C(=C\c2ccc(o2)c3ccccc3[N+](=O)[O-])/NC(=O)c4ccccc4
ZINC08441266	4.76	1.46	-33.83	1	8	0	445.475	6	c1ccc(cc1)C(=O)N/C(=C/c2ccc(o2)c3ccccc3[N+](=O)[O-])/C(=O)N4CCCCC4
ZINC08441265	5.79	0.67	-13.35	1	7	0	543.601	7	c1ccc(cc1)CSc2nnc(s2)N3[C@@H](C(=C(C3=O)O)C(=O)c4cc5ccccc5o4)c6ccccc6F
ZINC08441265	5.2	0.49	-13.34	0	7	0	543.601	7	c1ccc(cc1)CSc2nnc(s2)N3[C@@H]([C@H](C(=O)C3=O)C(=O)c4cc5ccccc5o4)c6ccccc6F
ZINC08441264	5.43	0.44	-11.29	2	5	0	429.303	5	Cc1ccc(cc1)C(=O)N/C(=C\c2ccc(o2)c3cc(ccc3Cl)Cl)/C(=O)NC
ZINC08441263	7.23	1.01	-11.91	2	5	0	519.428	8	Cc1ccc(cc1)C(=O)N/C(=C\c2ccc(o2)c3cc(ccc3Cl)Cl)/C(=O)NCCc4ccccc4
ZINC08441262	5.89	0.91	-11.62	0	6	0	514.416	7	CCOC(=O)C1[C@H](C2=C(CC(CC2=O)(C)C)N=C1C)c3ccc(o3)COc4ccccc4Br
ZINC08441262	6.08	-0.34	-14.47	0	6	0	514.416	7	CCOC(=O)C1[C@H](C2C(=NC1=C)CC(CC2=O)(C)C)c3ccc(o3)COc4ccccc4Br
ZINC08441262	5.89	0.63	-16.82	0	6	0	514.416	7	CCOC(=O)C1=C(N=C2CC(CC(=O)C2[C@@H]1c3ccc(o3)COc4ccccc4Br)(C)C)C
ZINC08441261	5.77	-0.04	-13.33	1	8	0	568.68	8	CN(C)c1ccc(cc1)[C@H]2C(=C(C(=O)N2c3nnc(s3)SCc4ccccc4)O)C(=O)c5cc6ccccc6o5
ZINC08441260	5.63	1.05	-13.05	2	6	0	481.335	7	c1ccc(cc1)C(=O)N/C(=C\c2ccc(o2)c3ccc(cc3Cl)Cl)/C(=O)NCc4ccco4
ZINC08441259	4.25	7.4	-10.61	2	7	0	425.268	7	CC1=C([C@H](NC(=O)N1)c2ccc(o2)c3cc(ccc3Cl)Cl)C(=O)OCCOC
ZINC08441258	5.77	0.02	-13.36	1	8	0	568.68	8	CN(C)c1ccc(cc1)[C@@H]2C(=C(C(=O)N2c3nnc(s3)SCc4ccccc4)O)C(=O)c5cc6ccccc6o5
ZINC08441257	4.25	7.4	-10.77	2	7	0	425.268	7	CC1=C([C@@H](NC(=O)N1)c2ccc(o2)c3cc(ccc3Cl)Cl)C(=O)OCCOC
ZINC02063893	6.78	0.73	-13.72	2	5	0	505.401	8	c1ccc(cc1)CCNC(=O)/C(=C/c2ccc(o2)c3ccc(cc3Cl)Cl)/NC(=O)c4ccccc4
ZINC02063891	5.65	0.7	-13.04	2	5	0	443.33	6	CC(C)NC(=O)/C(=C/c1ccc(o1)c2ccc(cc2Cl)Cl)/NC(=O)c3ccccc3
ZINC08441255	6.97	0.89	-14.64	1	9	0	641.771	12	CC(C)CCOc1ccc(cc1OC)[C@H]2C(=C(C(=O)N2c3nnc(s3)SCc4ccccc4)O)C(=O)c5cc6ccccc6o5

Supplementary Table S2:
Detailed table of XP Docking using Schrodinger's Glide module

ZINC_ID	xLogP	ApolarDesolvation	PolarDesolvation	H Donors	H Acceptors	Charge	MolecularWeight	RotatableBonds	SMILES
ZINC08441255	6.39	0.67	-17.3	0	9	0	641.771	12	CC(C)CCOc1ccc(cc1OC)[C@H]2[C@H](C(=O)C(=O)N2c3nnc(s3)SCc4ccccc4)C(=O)c5cc6ccccc6o5
ZINC02677884	6.13	0.72	-13.01	2	5	0	470.956	8	c1ccc(cc1)CCNC(=O)/C(=C/c2ccc(o2)c3ccccc3Cl)/NC(=O)c4ccccc4
ZINC08441254	6.35	1.26	-11.9	0	6	0	496.003	7	CC1=NC2=C([C@@H](C1C(=O)OC)c3ccc(c(c3)COc4ccccc4Cl)OC)C(=O)CC(C2)(C)C
ZINC08441254	6.35	1.47	-14.88	0	6	0	496.003	7	CC1=C([C@H]([C@H]2C(=N1)CC(CC2=O)(C)C)c3ccc(c(c3)COc4ccccc4Cl)OC)C(=O)OC
ZINC08441254	6.53	2.57	-12.4	0	6	0	496.003	7	CC1(CC2=NC(=C)C([C@H]([C@H]2C(=O)C1)c3ccc(c(c3)COc4ccccc4Cl)OC)C(=O)OC)C
ZINC08441254	6.53	1.35	-12.58	0	6	0	496.003	7	CC1(CC2=NC(=C)C([C@H](C2C(=O)C1)c3ccc(c(c3)COc4ccccc4Cl)OC)C(=O)OC)C
ZINC08441253	6.97	0.86	-14.62	1	9	0	641.771	12	CC(C)CCOc1ccc(cc1OC)[C@@H]2C(=C(C(=O)N2c3nnc(s3)SCc4ccccc4)O)C(=O)c5cc6ccccc6o5
ZINC08441253	6.39	0.65	-17.37	0	9	0	641.771	12	CC(C)CCOc1ccc(cc1OC)[C@@H]2[C@@H](C(=O)C(=O)N2c3nnc(s3)SCc4ccccc4)C(=O)c5cc6ccccc6o5
ZINC08441252	5.72	0.49	-17.65	2	5	0	456.929	7	c1ccc(cc1)CNC(=O)/C(=C\c2ccc(o2)c3ccccc3Cl)/NC(=O)c4ccccc4
ZINC08441251	7.32	0.08	-14.03	1	8	0	631.735	10	c1ccc(cc1)COc2ccc(cc2)[C@H]3C(=C(C(=O)N3c4nnc(s4)SCc5ccccc5)O)C(=O)c6cc7ccccc7o6
ZINC08441251	6.74	-0.11	-16.03	0	8	0	631.735	10	c1ccc(cc1)COc2ccc(cc2)[C@H]3[C@H](C(=O)C(=O)N3c4nnc(s4)SCc5ccccc5)C(=O)c6cc7ccccc7o6
ZINC08441250	6.64	2.09	-16.23	0	5	0	450.947	5	CC1=C([C@H](n2c(=O)/c(=C\C=C\c3ccccc3)/sc2=N1)c4ccccc4Cl)C(=O)OC
ZINC02063871	6.53	2.14	-10.56	0	7	0	487.581	6	CCCCN1c2ccccc2/C(=c/3\c(=O)n4c(=NC(=C([C@H]4c5ccccc5)C(=O)OC)C)s3)/C1=O
ZINC08441248	7.32	0.08	-14.1	1	8	0	631.735	10	c1ccc(cc1)COc2ccc(cc2)[C@@H]3C(=C(C(=O)N3c4nnc(s4)SCc5ccccc5)O)C(=O)c6cc7ccccc7o6
ZINC08441248	6.74	-0.1	-16.18	0	8	0	631.735	10	c1ccc(cc1)COc2ccc(cc2)[C@@H]3[C@@H](C(=O)C(=O)N3c4nnc(s4)SCc5ccccc5)C(=O)c6cc7ccccc7o6
ZINC02063873	6.53	2.14	-10.51	0	7	0	487.581	6	CCCCN1c2ccccc2/C(=c/3\c(=O)n4c(=NC(=C([C@@H]4c5ccccc5)C(=O)OC)C)s3)/C1=O
ZINC08441247	6.37	0.38	-13.08	1	8	0	581.675	10	C=CCOc1ccc(cc1)[C@H]2C(=C(C(=O)N2c3nnc(s3)SCc4ccccc4)O)C(=O)c5cc6ccccc6o5
ZINC08441247	5.79	0.19	-15.06	0	8	0	581.675	10	C=CCOc1ccc(cc1)[C@H]2[C@H](C(=O)C(=O)N2c3nnc(s3)SCc4ccccc4)C(=O)c5cc6ccccc6o5
ZINC02278020	5.12	1.91	-13.53	0	9	0	519.579	6	CCOC(=O)C1=C(N=c2n(c(=O)/c(=C/3\c4ccccc4N(C3=O)C)/s2)[C@@H]1c5ccc(c(c5)OC)OC)C
ZINC08441246	6.35	1.35	-11.99	0	6	0	496.003	7	CC1=NC2=C([C@H](C1C(=O)OC)c3ccc(c(c3)COc4ccccc4Cl)OC)C(=O)CC(C2)(C)C
ZINC08441246	6.53	1.41	-12.39	0	6	0	496.003	7	CC1(CC2=NC(=C)C([C@@H](C2C(=O)C1)c3ccc(c(c3)COc4ccccc4Cl)OC)C(=O)OC)C
ZINC08441246	6.35	1.29	-15.06	0	6	0	496.003	7	CC1=C([C@@H]([C@H]2C(=N1)CC(CC2=O)(C)C)c3ccc(c(c3)COc4ccccc4Cl)OC)C(=O)OC
ZINC08441246	6.53	2.73	-13.83	0	6	0	496.003	7	CC1(CC2=NC(=C)C([C@@H]([C@@H]2C(=O)C1)c3ccc(c(c3)COc4ccccc4Cl)OC)C(=O)OC)C
ZINC02282020	4.2	-0.97	-18.27	2	9	0	502.552	6	CCOC(=O)C1=C(N=c2n(c(=O)/c(=C/3\c4ccccc4N(C3=O)CC(=O)N)/s2)[C@H]1c5ccccc5)C
ZINC02282022	4.2	-1.09	-18.35	2	9	0	502.552	6	CCOC(=O)C1=C(N=c2n(c(=O)/c(=C/3\c4ccccc4N(C3=O)CC(=O)N)/s2)[C@@H]1c5ccccc5)C
ZINC08441245	7.25	2.57	-15.21	0	5	0	532.665	3	CCn1c(=O)/c(=C\C=C\2/C(c3cc(ccc3N2C)c4ccccc4)(C)C)/sc1=C5C(=O)c6ccccc6C5=O
ZINC08441244	6.37	0.36	-13.11	1	8	0	581.675	10	C=CCOc1ccc(cc1)[C@@H]2C(=C(C(=O)N2c3nnc(s3)SCc4ccccc4)O)C(=O)c5cc6ccccc6o5
ZINC08441244	5.79	0.17	-15.04	0	8	0	581.675	10	C=CCOc1ccc(cc1)[C@@H]2[C@@H](C(=O)C(=O)N2c3nnc(s3)SCc4ccccc4)C(=O)c5cc6ccccc6o5
ZINC06474045	6.18	-1.4	-11.36	1	5	0	487.401	4	c1ccc(cc1)c2c3cc(ccc3nc(n2)Nc4ccc(cc4)C(=O)N5CCCCC5)Br
ZINC08441243	5.3	0.82	-14.78	1	10	0	615.689	10	COc1cc(cc(c1OC)OC)[C@H]2C(=C(C(=O)N2c3nnc(s3)SCc4ccccc4)O)C(=O)c5cc6ccccc6o5
ZINC08441243	4.72	0.85	-15.92	0	10	0	615.689	10	COc1cc(cc(c1OC)OC)[C@H]2[C@@H](C(=O)C(=O)N2c3nnc(s3)SCc4ccccc4)C(=O)c5cc6ccccc6o5
ZINC08441242	7.51	0.7	-9.77	0	6	0	588.926	8	CCOC(=O)C1[C@@H](C2=C(CC(CC2=O)(C)C)N=C1C)c3ccc(c(c3)COc4ccc(cc4Br)Cl)OC
ZINC08441242	7.69	-0.48	-10.21	0	6	0	588.926	8	CCOC(=O)C1[C@@H](C2C(=NC1=C)CC(CC2=O)(C)C)c3ccc(c(c3)COc4ccc(cc4Br)Cl)OC
ZINC08441242	7.51	0.62	-13.33	0	6	0	588.926	8	CCOC(=O)C1=C(N=C2CC(CC(=O)C2[C@H]1c3ccc(c(c3)COc4ccc(cc4Br)Cl)OC)(C)C)C
ZINC08441241	5.3	0.71	-15.82	1	10	0	615.689	10	COc1cc(cc(c1OC)OC)[C@@H]2C(=C(C(=O)N2c3nnc(s3)SCc4ccccc4)O)C(=O)c5cc6ccccc6o5
ZINC08441241	4.72	0.5	-17.91	0	10	0	615.689	10	COc1cc(cc(c1OC)OC)[C@@H]2[C@@H](C(=O)C(=O)N2c3nnc(s3)SCc4ccccc4)C(=O)c5cc6ccccc6o5
ZINC00851613	4.79	0.65	-10.16	0	8	0	465.872	4	Cn1c2c(nc1Oc3ccc(c4c3nccc4)Cl)n(c(=O)n(c2=O)Cc5ccc(cc5)F)C
ZINC00851613	4.79	1.02	-35.34	1	8	1	466.88	4	Cn1c2c([nH+]c1Oc3ccc(c4c3nccc4)Cl)n(c(=O)n(c2=O)Cc5ccc(cc5)F)C
ZINC00851292	5.39	0.26	-10.36	0	6	0	447.347	4	Cn1c2c(c(=O)n(c1=O)C)n(c(n2)Sc3ccccc3)Cc4ccc(c(c4)Cl)Cl
ZINC00851292	5.39	0.49	-36.02	1	6	1	448.355	4	Cn1c2c(c(=O)n(c1=O)C)n(c([nH+]2)Sc3ccccc3)Cc4ccc(c(c4)Cl)Cl
ZINC00851289	4.91	-1.12	-8.83	0	9	0	475.288	4	Cn1c2c(c(=O)n(c1=O)C)n(c(n2)Oc3ccc4c(c3)OCO4)Cc5ccc(cc5Cl)Cl
ZINC00851289	4.91	-0.26	-34.02	1	9	1	476.296	4	Cn1c2c(c(=O)n(c1=O)C)n(c([nH+]2)Oc3ccc4c(c3)OCO4)Cc5ccc(cc5Cl)Cl
ZINC08441240	6.59	0.3	-12.25	1	7	0	553.665	8	CCc1ccc(cc1)[C@H]2C(=C(C(=O)N2c3nnc(s3)SCc4ccccc4)O)C(=O)c5cc6ccccc6o5
ZINC08441240	6	0.14	-13.29	0	7	0	553.665	8	CCc1ccc(cc1)[C@H]2[C@@H](C(=O)C(=O)N2c3nnc(s3)SCc4ccccc4)C(=O)c5cc6ccccc6o5
ZINC08441239	4.05	-2.58	-16.54	1	8	0	435.259	6	c1ccc(c(c1)c2nnc(o2)SCC(=O)Nc3ccc(cc3)[N+](=O)[O-])Br
ZINC08441238	7.51	0.89	-10.18	0	6	0	588.926	8	CCOC(=O)C1[C@H](C2=C(CC(CC2=O)(C)C)N=C1C)c3ccc(c(c3)COc4ccc(cc4Br)Cl)OC
ZINC08441238	7.51	0.53	-12.86	0	6	0	588.926	8	CCOC(=O)C1=C(N=C2CC(CC(=O)C2[C@@H]1c3ccc(c(c3)COc4ccc(cc4Br)Cl)OC)(C)C)C
ZINC08441238	7.69	-0.39	-10.43	0	6	0	588.926	8	CCOC(=O)C1[C@H](C2C(=NC1=C)CC(CC2=O)(C)C)c3ccc(c(c3)COc4ccc(cc4Br)Cl)OC
ZINC00851197	4.03	-2.59	-20.36	1	8	0	435.259	6	c1ccc(c(c1)c2nnc(o2)SCC(=O)Nc3cccc(c3)[N+](=O)[O-])Br
ZINC08441237	6.76	-2.08	-17.99	0	5	0	498.435	4	Cc1ccc(cc1)N2CCN(CC2)C3=NC(=O)/C(=C\c4ccc(o4)c5cc(ccc5Cl)Cl)/S3
ZINC08441236	6.59	0.36	-12.28	1	7	0	553.665	8	CCc1ccc(cc1)[C@@H]2C(=C(C(=O)N2c3nnc(s3)SCc4ccccc4)O)C(=O)c5cc6ccccc6o5
ZINC08441236	6	0.17	-14.16	0	7	0	553.665	8	CCc1ccc(cc1)[C@@H]2[C@@H](C(=O)C(=O)N2c3nnc(s3)SCc4ccccc4)C(=O)c5cc6ccccc6o5
ZINC08441234	4.69	0.19	-64.15	1	8	1	475.55	6	c1ccc(cc1)C[NH+]2CCN(CC2)C3=NC(=O)/C(=C\c4ccc(o4)c5ccc(cc5)[N+](=O)[O-])/S3
ZINC08441232	5.43	-1.76	-22.99	0	8	0	474.542	5	Cc1ccc(cc1)N2CCN(CC2)C3=NC(=O)/C(=C\c4ccc(o4)c5ccc(cc5)[N+](=O)[O-])/S3
ZINC08441231	6	3.75	-12.03	0	6	0	503.517	8	CCOC(=O)C1[C@@H](C2=C(CC(CC2=O)(C)C)N=C1C)c3ccc(o3)COc4cccc(c4)C(F)(F)F
ZINC08441231	6.19	2.79	-12.53	0	6	0	503.517	8	CCOC(=O)C1[C@@H](C2C(=NC1=C)CC(CC2=O)(C)C)c3ccc(o3)COc4cccc(c4)C(F)(F)F
ZINC08441231	6	3.82	-14.1	0	6	0	503.517	8	CCOC(=O)C1=C(N=C2CC(CC(=O)C2[C@H]1c3ccc(o3)COc4cccc(c4)C(F)(F)F)(C)C)C
ZINC08441230	5.39	-1.58	-14.41	2	9	0	585.663	9	CCOc1cc(ccc1O)[C@H]2C(=C(C(=O)N2c3nnc(s3)SCc4ccccc4)O)C(=O)c5cc6ccccc6o5
ZINC08441230	4.8	-1.79	-16.94	1	9	0	585.663	9	CCOc1cc(ccc1O)[C@H]2[C@H](C(=O)C(=O)N2c3nnc(s3)SCc4ccccc4)C(=O)c5cc6ccccc6o5
ZINC08441229	5.41	-1.75	-22.95	0	8	0	474.542	5	Cc1cccc(c1)N2CCN(CC2)C3=NC(=O)/C(=C\c4ccc(o4)c5ccc(cc5)[N+](=O)[O-])/S3
ZINC05013440	3.05	0.08	-44.79	2	3	1	250.362	5	COc1ccc(cc1OC)C[NH2+]C2CCCCC2

Supplementary Table S2:
Detailed table of XP Docking using Schrodinger's Glide module

ZINC_ID	xLogP	ApolarDesolvation	PolarDesolvation	H Donors	H Acceptors	Charge	MolecularWeight	RotatableBonds	SMILES
ZINC08441228	5.39	-1.57	-14.42	2	9	0	585.663	9	CCOc1cc(ccc1O)[C@@H]2C(=C(C(=O)N2c3nnc(s3)SCc4ccccc4)O)C(=O)c5cc6ccccc6o5
ZINC08441228	4.8	-1.79	-16.83	1	9	0	585.663	9	CCOc1cc(ccc1O)[C@@H]2[C@@H](C(=O)C(=O)N2c3nnc(s3)SCc4ccccc4)C(=O)c5cc6ccccc6o5
ZINC08441227	6	3.73	-11.59	0	6	0	503.517	8	CCOC(=O)C1[C@H](C2=C(CC(CC2=O)(C)C)N=C1C)c3ccc(o3)COc4cccc(c4)C(F)(F)F
ZINC08441227	6.19	2.44	-14.13	0	6	0	503.517	8	CCOC(=O)C1[C@H](C2C(=NC1=C)CC(CC2=O)(C)C)c3ccc(o3)COc4cccc(c4)C(F)(F)F
ZINC08441227	6	3.46	-16.65	0	6	0	503.517	8	CCOC(=O)C1=C(N=C2CC(CC(=O)C2[C@@H]1c3ccc(o3)COc4cccc(c4)C(F)(F)F)(C)C)C
ZINC02380267	1.71	-0.13	-54.29	2	4	1	273.356	7	COc1ccc(cc1OC)CC[NH2+]Cc2cccnc2
ZINC05313299	1.21	6.68	-53.49	3	10	1	494.37	6	Cn1c2c(c(=O)[nH]c1=O)n(c(n2)N3CC[NH+](CC3)C)C[C@H](COc4ccc(cc4)Br)O
ZINC05313299	1.21	7.12	-116.06	4	10	2	495.378	6	Cn1c2c(c(=O)[nH]c1=O)n(c([nH+]2)N3CC[NH+](CC3)C)C[C@H](COc4ccc(cc4)Br)O
ZINC05313297	1.21	6.64	-56.35	3	10	1	494.37	6	Cn1c2c(c(=O)[nH]c1=O)n(c(n2)N3CC[NH+](CC3)C)C[C@@H](COc4ccc(cc4)Br)O
ZINC05313297	1.21	7.09	-118.02	4	10	2	495.378	6	Cn1c2c(c(=O)[nH]c1=O)n(c([nH+]2)N3CC[NH+](CC3)C)C[C@@H](COc4ccc(cc4)Br)O
ZINC05313283	1.32	9.09	-53.1	2	9	1	462.328	4	Cn1c2c(c(=O)[nH]c1=O)n(c(n2)N3CC[NH+](CC3)C)CC(=O)c4ccc(cc4)Br
ZINC05313283	1.32	9.57	-114.72	3	9	2	463.336	4	Cn1c2c(c(=O)[nH]c1=O)n(c([nH+]2)N3CC[NH+](CC3)C)CC(=O)c4ccc(cc4)Br
ZINC08441224	6.86	0.85	-11.27	0	6	0	554.481	8	CCOC(=O)C1[C@@H](C2=C(CC(CC2=O)(C)C)N=C1C)c3ccc(c(c3)COc4ccccc4Br)OC
ZINC08441224	6.86	0.77	-14.74	0	6	0	554.481	8	CCOC(=O)C1=C(N=C2CC(CC(=O)C2[C@H]1c3ccc(c(c3)COc4ccccc4Br)OC)(C)C)C
ZINC08441224	7.04	-0.33	-11.45	0	6	0	554.481	8	CCOC(=O)C1[C@@H](C2C(=NC1=C)CC(CC2=O)(C)C)c3ccc(c(c3)COc4ccccc4Br)OC
ZINC05313280	0.92	10.57	-62.12	2	11	1	400.419	4	Cn1c2c(c(=O)[nH]c1=O)n(c(n2)N3CC[NH+](CC3)C)Cc4ccc(cc4)[N+](=O)[O-]
ZINC05313280	0.92	8.19	-12.35	1	11	0	399.411	4	Cn1c2c(c(=O)[nH]c1=O)n(c(n2)N3CCN(CC3)C)Cc4ccc(cc4)[N+](=O)[O-]
ZINC08441223	4.1	0.51	-56.08	0	7	-1	450.902	6	Cc1ccc2cc(c(nc2c1)Cl)[C@H]3CC(=NN3C(=O)CCC(=O)[O-])c4ccc(cc4)OC
ZINC08441222	7.26	0.69	-14.78	1	9	0	641.771	13	CCCCCOc1ccc(cc1OC)[C@H]2C(=C(C(=O)N2c3nnc(s3)SCc4ccccc4)O)C(=O)c5cc6ccccc6o5
ZINC08441222	6.68	0.48	-17.41	0	9	0	641.771	13	CCCCCOc1ccc(cc1OC)[C@H]2[C@H](C(=O)C(=O)N2c3nnc(s3)SCc4ccccc4)C(=O)c5cc6ccccc6o5
ZINC08441221	4.1	0.44	-55.84	0	7	-1	450.902	6	Cc1ccc2cc(c(nc2c1)Cl)[C@@H]3CC(=NN3C(=O)CCC(=O)[O-])c4ccc(cc4)OC
ZINC00970966	3.44	2.12	-14.26	0	8	0	470.476	9	Cn1c2c(c(=O)n(c1=O)C)n(cn2)C[C@H](COCc3ccccc3F)OCc4ccccc4F
ZINC00970966	3.44	2.41	-47.02	1	8	1	471.484	9	Cn1c2c(c(=O)n(c1=O)C)n(c[nH+]2)C[C@H](COCc3ccccc3F)OCc4ccccc4F
ZINC00970965	3.44	3.02	-16.08	0	8	0	470.476	9	Cn1c2c(c(=O)n(c1=O)C)n(cn2)C[C@@H](COCc3ccccc3F)OCc4ccccc4F
ZINC00970965	3.44	3.3	-54.62	1	8	1	471.484	9	Cn1c2c(c(=O)n(c1=O)C)n(c[nH+]2)C[C@@H](COCc3ccccc3F)OCc4ccccc4F
ZINC08441220	6.86	1.03	-11.59	0	6	0	554.481	8	CCOC(=O)C1[C@H](C2=C(CC(CC2=O)(C)C)N=C1C)c3ccc(c(c3)COc4ccccc4Br)OC
ZINC08441220	6.86	0.68	-14.34	0	6	0	554.481	8	CCOC(=O)C1=C(N=C2CC(CC(=O)C2[C@@H]1c3ccc(c(c3)COc4ccccc4Br)OC)(C)C)C
ZINC08441220	7.04	-0.24	-11.72	0	6	0	554.481	8	CCOC(=O)C1[C@H](C2C(=NC1=C)CC(CC2=O)(C)C)c3ccc(c(c3)COc4ccccc4Br)OC
ZINC00970964	3.54	1.76	-14.61	0	8	0	470.476	9	Cn1c2c(c(=O)n(c1=O)C)n(cn2)C[C@H](COCc3ccc(cc3)F)OCc4ccc(cc4)F
ZINC00970964	3.54	2.01	-58.36	1	8	1	471.484	9	Cn1c2c(c(=O)n(c1=O)C)n(c[nH+]2)C[C@H](COCc3ccc(cc3)F)OCc4ccc(cc4)F
ZINC08441219	7.26	0.67	-14.73	1	9	0	641.771	13	CCCCCOc1ccc(cc1OC)[C@@H]2C(=C(C(=O)N2c3nnc(s3)SCc4ccccc4)O)C(=O)c5cc6ccccc6o5
ZINC08441219	6.68	0.45	-17.43	0	9	0	641.771	13	CCCCCOc1ccc(cc1OC)[C@@H]2[C@@H](C(=O)C(=O)N2c3nnc(s3)SCc4ccccc4)C(=O)c5cc6ccccc6o5
ZINC00970963	3.54	2.73	-17.58	0	8	0	470.476	9	Cn1c2c(c(=O)n(c1=O)C)n(cn2)C[C@@H](COCc3ccc(cc3)F)OCc4ccc(cc4)F
ZINC00970963	3.54	3.01	-59.66	1	8	1	471.484	9	Cn1c2c(c(=O)n(c1=O)C)n(c[nH+]2)C[C@@H](COCc3ccc(cc3)F)OCc4ccc(cc4)F
ZINC00850367	4.43	-2.4	-14.49	1	8	0	449.286	6	Cc1ccc(cc1[N+](=O)[O-])NC(=O)CSc2nnc(o2)c3ccccc3Br
ZINC08441218	5.19	-2.07	-13.67	2	8	0	541.61	7	c1ccc(cc1)CSc2nnc(s2)N3[C@H](C(=C(C3=O)O)C(=O)c4cc5ccccc5o4)c6ccc(cc6)O
ZINC08441218	4.61	-2.26	-15.86	1	8	0	541.61	7	c1ccc(cc1)CSc2nnc(s2)N3[C@H]([C@H](C(=O)C3=O)C(=O)c4cc5ccccc5o4)c6ccc(cc6)O
ZINC08441217	6.26	2.45	-9.15	0	6	0	493.575	8	CCOC(=O)C1[C@@H](C2=C(CC(CC2=O)(C)C)N=C1C)c3ccc(c(c3)OC)COc4ccc(cc4)F
ZINC08441217	6.44	1.51	-10.45	0	6	0	493.575	8	CCOC(=O)C1[C@@H](C2C(=NC1=C)CC(CC2=O)(C)C)c3ccc(c(c3)OC)COc4ccc(cc4)F
ZINC08441217	6.26	2.43	-12.36	0	6	0	493.575	8	CCOC(=O)C1=C(N=C2CC(CC(=O)C2[C@H]1c3ccc(c(c3)OC)COc4ccc(cc4)F)(C)C)C
ZINC02063825	7.14	-0.78	-9.05	1	5	0	429.907	3	c1ccc2c(c1)-c3c4c(c(cc(c4no3)N5CCCCC5)Nc6ccc(cc6)Cl)C2=O
ZINC08441216	5.19	-2.09	-13.71	2	8	0	541.61	7	c1ccc(cc1)CSc2nnc(s2)N3[C@@H](C(=C(C3=O)O)C(=O)c4cc5ccccc5o4)c6ccc(cc6)O
ZINC08441216	4.61	-2.28	-15.94	1	8	0	541.61	7	c1ccc(cc1)CSc2nnc(s2)N3[C@@H]([C@@H](C(=O)C3=O)C(=O)c4cc5ccccc5o4)c6ccc(cc6)O
ZINC13196185	8.45	12.15	-9.15	2	5	0	443.934	4	c1ccc2c(c1)-c3c4c(c(cc(c4no3)NC5CCCCC5)Nc6ccc(cc6)Cl)C2=O
ZINC08441215	5.73	10.9	-33.47	2	6	0	506.583	6	c1ccc(cc1)/C=C/C(=O)Nc2cc3c(cc2)-c4c(cc(cc4)NC(=O)/C=C/c5ccccc5)S3(=O)=O
ZINC08441212	4.62	-0.26	-7.01	1	6	0	364.515	10	CCCCCCCCSc1nc2c(n1CC(=C)C)c(=O)[nH]c(=O)n2C
ZINC08441212	4.62	-0.01	-35.72	2	6	1	365.523	10	CCCCCCCCSc1[nH+]c2c(n1CC(=C)C)c(=O)[nH]c(=O)n2C
ZINC08441211	6.26	2.46	-9.15	0	6	0	493.575	8	CCOC(=O)C1[C@H](C2=C(CC(CC2=O)(C)C)N=C1C)c3ccc(c(c3)OC)COc4ccc(cc4)F
ZINC08441211	6.44	1.28	-10.01	0	6	0	493.575	8	CCOC(=O)C1[C@H](C2C(=NC1=C)CC(CC2=O)(C)C)c3ccc(c(c3)OC)COc4ccc(cc4)F
ZINC08441211	6.26	2.19	-12.08	0	6	0	493.575	8	CCOC(=O)C1=C(N=C2CC(CC(=O)C2[C@@H]1c3ccc(c(c3)OC)COc4ccc(cc4)F)(C)C)C
ZINC08441210	4.11	-0.38	-7.02	1	6	0	350.488	9	CCCCCCCSc1nc2c(n1CC(=C)C)c(=O)[nH]c(=O)n2C
ZINC08441210	4.11	-0.13	-35.65	2	6	1	351.496	9	CCCCCCCSc1[nH+]c2c(n1CC(=C)C)c(=O)[nH]c(=O)n2C
ZINC08441209	6.46	-0.87	-12.84	1	7	0	604.507	7	c1ccc(cc1)CSc2nnc(s2)N3[C@H](C(=C(C3=O)O)C(=O)c4cc5ccccc5o4)c6cccc(c6)Br
ZINC08441209	5.87	-1.03	-14.63	0	7	0	604.507	7	c1ccc(cc1)CSc2nnc(s2)N3[C@H]([C@@H](C(=O)C3=O)C(=O)c4cc5ccccc5o4)c6cccc(c6)Br
ZINC02063810	3.88	20.93	-37.82	2	4	1	474.628	7	CC(C)(C)c1ccc(cc1)C[n+]2c3ccccc3n(c2N)CC(=O)c4ccc(cc4)c5ccccc5
ZINC08441208	6.46	-0.87	-12.92	1	7	0	604.507	7	c1ccc(cc1)CSc2nnc(s2)N3[C@@H](C(=C(C3=O)O)C(=O)c4cc5ccccc5o4)c6cccc(c6)Br
ZINC08441208	5.87	-1.05	-15.87	0	7	0	604.507	7	c1ccc(cc1)CSc2nnc(s2)N3[C@@H]([C@@H](C(=O)C3=O)C(=O)c4cc5ccccc5o4)c6cccc(c6)Br
ZINC08441207	5.5	0.92	-10.93	0	7	0	477.557	9	CCOC(=O)C1[C@@H](C2=C(CCCC2=O)N=C1C)c3ccc(c(c3)OC)COc4ccc(cc4)OC
ZINC08441207	5.68	-0.36	-12.34	0	7	0	477.557	9	CCOC(=O)C1[C@@H](C2C(=NC1=C)CCCC2=O)c3ccc(c(c3)OC)COc4ccc(cc4)OC
ZINC08441207	5.5	0.57	-15.3	0	7	0	477.557	9	CCOC(=O)C1=C(N=C2CCCC(=O)C2[C@H]1c3ccc(c(c3)OC)COc4ccc(cc4)OC)C
ZINC08441206	5.67	-0.1	-12.46	1	7	0	525.611	7	c1ccc(cc1)CSc2nnc(s2)N3[C@H](C(=C(C3=O)O)C(=O)c4cc5ccccc5o4)c6ccccc6

Supplementary Table S2:
Detailed table of XP Docking using Schrodinger's Glide module

ZINC_ID	xLogP	ApolarDesolvation	PolarDesolvation	H Donors	H Acceptors	Charge	MolecularWeight	RotatableBonds	SMILES
ZINC08441206	5.09	-0.29	-14.36	0	7	0	525.611	7	c1ccc(cc1)CSc2nnc(s2)N3[C@H]([C@H](C(=O)C3=O)C(=O)c4cc5ccccc5o4)c6ccccc6
ZINC02063808	3.9	0.48	-24.6	1	11	0	547.593	8	Cc1nc2c(c3c(s2)CCCC3)c(=O)n1/N=C/c4ccc(c(c4)OC)OCC(=O)Nc5cccc(c5)[N+](=O)[O-]
ZINC08441204	5.67	-0.12	-12.51	1	7	0	525.611	7	c1ccc(cc1)CSc2nnc(s2)N3[C@@H](C(=C(C3=O)O)C(=O)c4cc5ccccc5o4)c6ccccc6
ZINC08441204	5.09	-0.31	-14.35	0	7	0	525.611	7	c1ccc(cc1)CSc2nnc(s2)N3[C@@H]([C@@H](C(=O)C3=O)C(=O)c4cc5ccccc5o4)c6ccccc6
ZINC08441203	6.25	3.87	-14.98	1	6	0	549.574	9	COc1ccc(cc1OC)c2cc(nc(c2C#N)SCC(=O)Nc3ccccc3C(F)(F)F)c4ccccc4
ZINC08441202	5.5	0.93	-10.82	0	7	0	477.557	9	CCOC(=O)C1[C@H](C2=C(CCCC2=O)N=C1C)c3ccc(c(c3)OC)COc4ccc(cc4)OC
ZINC08441202	5.68	-0.34	-12.83	0	7	0	477.557	9	CCOC(=O)C1[C@H](C2C(=NC1=C)CCCC2=O)c3ccc(c(c3)OC)COc4ccc(cc4)OC
ZINC08441202	5.5	0.57	-15	0	7	0	477.557	9	CCOC(=O)C1=C(N=C2CCCC(=O)C2[C@@H]1c3ccc(c(c3)OC)COc4ccc(cc4)OC)C
ZINC00702947	4.71	-0.23	-11.46	1	7	0	492.94	5	Cc1c(sc(n1)N2[C@H](C(=C(C2=O)O)C(=O)c3cc4ccccc4o3)c5cccc(c5)Cl)C(=O)C
ZINC00702947	4.12	-0.39	-12.05	0	7	0	492.94	5	Cc1c(sc(n1)N2[C@H]([C@@H](C(=O)C2=O)C(=O)c3cc4ccccc4o3)c5cccc(c5)Cl)C(=O)C
ZINC08441201	6.23	2.32	-17.7	1	6	0	509.631	8	Cc1ccc(c(c1)NC(=O)CSc2c(c(cc(n2)c3ccccc3)c4ccc(c(c4)OC)OC)C#N)C
ZINC08441199	8.71	3.98	-8.28	0	2	0	448.635	6	Cc1ccc(cc1)CSc2c(c(cc(n2)c3ccccc3)c4ccc(cc4)C(C)(C)C)C#N
ZINC00702948	4.71	-0.15	-12.31	1	7	0	492.94	5	Cc1c(sc(n1)N2[C@@H](C(=C(C2=O)O)C(=O)c3cc4ccccc4o3)c5cccc(c5)Cl)C(=O)C
ZINC00702948	4.12	-0.33	-15	0	7	0	492.94	5	Cc1c(sc(n1)N2[C@@H]([C@@H](C(=O)C2=O)C(=O)c3cc4ccccc4o3)c5cccc(c5)Cl)C(=O)C
ZINC08441197	6.63	-0.7	-12.37	3	6	0	509.631	6	Cc1ccc(c(c1)NC(=O)c2c(c3c(cc(nc3s2)c4ccccc4)c5ccc(c(c5)OC)OC)N)C
ZINC00702945	4.16	-0.1	-12.63	1	8	0	501.564	6	Cc1c(sc(n1)N2[C@H](C(=C(C2=O)O)C(=O)c3cc4ccccc4o3)c5ccc(cc5)N(C)C)C(=O)C
ZINC08441196	7.81	3.74	-13.16	0	3	0	462.618	7	CC(C)(C)c1ccc(cc1)c2cc(nc(c2C#N)SCC(=O)c3ccccc3)c4ccccc4
ZINC00702946	4.16	-0.22	-12.75	1	8	0	501.564	6	Cc1c(sc(n1)N2[C@@H](C(=C(C2=O)O)C(=O)c3cc4ccccc4o3)c5ccc(cc5)N(C)C)C(=O)C
ZINC08441195	6.82	1.84	-18.06	1	7	0	590.126	10	CCOC(=O)c1c(c(sc1NC(=O)CSc2c(c(cc(n2)c3ccccc3)c4ccccc4Cl)C#N)C(=O)C)C
ZINC08441193	5.57	0.69	-11.38	1	7	0	500.576	6	Cc1c(sc(n1)N2[C@H](C(=C(C2=O)O)C(=O)c3cc4ccccc4o3)c5ccc(cc5)C(C)C)C(=O)C
ZINC08441193	4.98	0.54	-12.28	0	7	0	500.576	6	Cc1c(sc(n1)N2[C@H]([C@@H](C(=O)C2=O)C(=O)c3cc4ccccc4o3)c5ccc(cc5)C(C)C)C(=O)C
ZINC08441192	7.51	2.4	-17.05	1	6	0	537.685	9	CC(C)(C)c1ccc(cc1)c2cc(nc(c2C#N)SCC(=O)Nc3cc(ccc3OC)OC)c4ccccc4
ZINC08441191	8.29	2.89	-14.81	1	4	0	505.687	7	Cc1cc(cc(c1)NC(=O)CSc2c(c(cc(n2)c3ccccc3)c4ccc(cc4)C(C)(C)C)C#N)C
ZINC08441190	7.03	2.06	-15.14	1	4	0	463.606	6	Cc1ccc(cc1)c2cc(nc(c2C#N)SCC(=O)Nc3cc(cc(c3)C)C)c4ccccc4
ZINC08441189	7.77	2.12	-15.46	1	4	0	499.639	6	Cc1ccc(cc1)c2cc(nc(c2C#N)SCC(=O)Nc3c(ccc4c3cccc4)C)c5ccccc5
ZINC08441188	5.57	0.8	-11.41	1	7	0	500.576	6	Cc1c(sc(n1)N2[C@@H](C(=C(C2=O)O)C(=O)c3cc4ccccc4o3)c5ccc(cc5)C(C)C)C(=O)C
ZINC08441188	4.98	0.62	-13.2	0	7	0	500.576	6	Cc1c(sc(n1)N2[C@@H]([C@@H](C(=O)C2=O)C(=O)c3cc4ccccc4o3)c5ccc(cc5)C(C)C)C(=O)C
ZINC08441187	6.55	2.91	-13.47	0	3	0	420.537	6	Cc1ccc(cc1)c2cc(nc(c2C#N)SCC(=O)c3ccccc3)c4ccccc4
ZINC08441186	7.68	1.77	-7.13	0	1	0	426.17	2	c1cc(c(cc1Cl)Cl)/C=C/2\CCCC/C(=C/c3ccc(cc3Cl)Cl)/C2=O
ZINC08441184	5.61	1.65	-9.89	0	6	0	465.521	8	CCOC(=O)C1[C@@H](C2=C(CCCC2=O)N=C1C)c3ccc(c(c3)OC)COc4ccc(cc4)F
ZINC08441184	5.61	1.3	-14.03	0	6	0	465.521	8	CCOC(=O)C1=C(N=C2CCCC(=O)C2[C@H]1c3ccc(c(c3)OC)COc4ccc(cc4)F)C
ZINC08441184	5.79	0.38	-11.21	0	6	0	465.521	8	CCOC(=O)C1[C@@H](C2C(=NC1=C)CCCC2=O)c3ccc(c(c3)OC)COc4ccc(cc4)F
ZINC08441183	4.36	-1.97	-12.73	2	9	0	583.416	6	Cc1c(sc(n1)N2[C@H](C(=C(C2=O)O)C(=O)c3cc4ccccc4o3)c5cc(c(c(c5)Br)O)OC)C(=O)C
ZINC08441183	3.77	-2.19	-14.63	1	9	0	583.416	6	Cc1c(sc(n1)N2[C@H]([C@H](C(=O)C2=O)C(=O)c3cc4ccccc4o3)c5cc(c(c(c5)Br)O)OC)C(=O)C
ZINC08441181	4.36	-2.24	-13.25	2	9	0	583.416	6	Cc1c(sc(n1)N2[C@@H](C(=C(C2=O)O)C(=O)c3cc4ccccc4o3)c5cc(c(c(c5)Br)O)OC)C(=O)C
ZINC08441181	3.77	-2.46	-14.76	1	9	0	583.416	6	Cc1c(sc(n1)N2[C@@H]([C@@H](C(=O)C2=O)C(=O)c3cc4ccccc4o3)c5cc(c(c(c5)Br)O)OC)C(=O)C
ZINC08441179	5.61	1.67	-9.76	0	6	0	465.521	8	CCOC(=O)C1[C@H](C2=C(CCCC2=O)N=C1C)c3ccc(c(c3)OC)COc4ccc(cc4)F
ZINC08441179	5.61	1.31	-13.75	0	6	0	465.521	8	CCOC(=O)C1=C(N=C2CCCC(=O)C2[C@@H]1c3ccc(c(c3)OC)COc4ccc(cc4)F)C
ZINC08441179	5.79	0.39	-11.63	0	6	0	465.521	8	CCOC(=O)C1[C@H](C2C(=NC1=C)CCCC2=O)c3ccc(c(c3)OC)COc4ccc(cc4)F
ZINC08441178	7.7	-1.46	-9.25	3	4	0	484.024	5	CCc1ccccc1NC(=O)c2c(c3c(cc(nc3s2)c4ccccc4)c5ccc(cc5)Cl)N
ZINC00702941	5.29	13.9	-15.73	1	9	0	594.645	9	Cc1c(sc(n1)N2[C@H](C(=C(C2=O)O)C(=O)c3cc4ccccc4o3)c5ccc(c(c5)OC)OCc6ccccc6)C(=O)C
ZINC00702941	4.71	14.74	-18.31	0	9	0	594.645	9	Cc1c(sc(n1)N2[C@H]([C@H](C(=O)C2=O)C(=O)c3cc4ccccc4o3)c5ccc(c(c5)OC)OCc6ccccc6)C(=O)C
ZINC00852503	4.79	-4.68	-8.96	3	6	0	456.786	7	Cc1cccc(c1)NCc2nc([nH]n2)SCC(=O)Nc3c(cc(cc3Cl)Cl)Cl
ZINC00702942	5.29	13.87	-15.76	1	9	0	594.645	9	Cc1c(sc(n1)N2[C@@H](C(=C(C2=O)O)C(=O)c3cc4ccccc4o3)c5ccc(c(c5)OC)OCc6ccccc6)C(=O)C
ZINC00702942	4.71	14.71	-18.3	0	9	0	594.645	9	Cc1c(sc(n1)N2[C@@H]([C@@H](C(=O)C2=O)C(=O)c3cc4ccccc4o3)c5ccc(c(c5)OC)OCc6ccccc6)C(=O)C
ZINC08441177	6.93	-0.67	-17.86	1	5	0	491.44	6	COc1ccc(cc1NC(=O)CN(C2CCCCC2)C(=O)c3c(c4ccccc4s3)Cl)Cl
ZINC08441176	4.7	-3.08	-13.22	2	6	0	475.315	4	Cc1cc(ccc1NC(=O)CSc2nc3c(c4ccccc4[nH]3)nn2)I
ZINC08441175	6.15	1.72	-10.21	0	7	0	505.611	9	CCOC(=O)C1[C@@H](C2=C(CC(CC2=O)(C)C)N=C1C)c3ccc(c(c3)OC)COc4ccc(cc4)OC
ZINC08441175	6.33	0.77	-11.67	0	7	0	505.611	9	CCOC(=O)C1[C@@H](C2C(=NC1=C)CC(CC2=O)(C)C)c3ccc(c(c3)OC)COc4ccc(cc4)OC
ZINC08441175	6.15	1.7	-13.45	0	7	0	505.611	9	CCOC(=O)C1=C(N=C2CC(CC(=O)C2[C@H]1c3ccc(c(c3)OC)COc4ccc(cc4)OC)(C)C)C
ZINC00970955	5.09	1.02	-14.21	1	5	0	437.71	6	c1ccc(cc1)CN(CC(=O)Nc2c(cc(cc2Cl)Cl)Cl)C(=O)c3ccco3
ZINC00702939	5.7	0.08	-13.52	1	8	0	564.619	8	Cc1c(sc(n1)N2[C@H](C(=C(C2=O)O)C(=O)c3cc4ccccc4o3)c5ccc(cc5)OCc6ccccc6)C(=O)C
ZINC00702939	5.12	-0.08	-14.35	0	8	0	564.619	8	Cc1c(sc(n1)N2[C@H]([C@@H](C(=O)C2=O)C(=O)c3cc4ccccc4o3)c5ccc(cc5)OCc6ccccc6)C(=O)C
ZINC06193975	6.7	13.34	-17.2	1	6	0	509.631	8	Cc1cc(cc(c1)NC(=O)CSc2c(c(cc(n2)c3ccc(cc3)OC)c4ccc(cc4)OC)C#N)C
ZINC06193984	6.64	14.04	-15.8	1	5	0	479.605	7	Cc1cc(cc(c1)NC(=O)CSc2c(c(cc(n2)c3ccccc3)c4ccc(cc4)OC)C#N)C
ZINC08441174	5.7	0.1	-13.54	1	8	0	564.619	8	Cc1c(sc(n1)N2[C@@H](C(=C(C2=O)O)C(=O)c3cc4ccccc4o3)c5ccc(cc5)OCc6ccccc6)C(=O)C
ZINC08441174	5.12	-0.08	-15.27	0	8	0	564.619	8	Cc1c(sc(n1)N2[C@@H]([C@@H](C(=O)C2=O)C(=O)c3cc4ccccc4o3)c5ccc(cc5)OCc6ccccc6)C(=O)C
ZINC08441173	7.57	1.78	-17.87	1	6	0	543.648	9	COc1ccc(cc1)c2cc(nc(c2C#N)SCC(=O)Nc3ccc(cc3)Oc4ccccc4)c5ccccc5
ZINC08441172	6.15	1.73	-10.16	0	7	0	505.611	9	CCOC(=O)C1[C@H](C2=C(CC(CC2=O)(C)C)N=C1C)c3ccc(c(c3)OC)COc4ccc(cc4)OC
ZINC08441172	6.15	1.46	-13.14	0	7	0	505.611	9	CCOC(=O)C1=C(N=C2CC(CC(=O)C2[C@@H]1c3ccc(c(c3)OC)COc4ccc(cc4)OC)(C)C)C
ZINC08441172	6.33	0.55	-11.05	0	7	0	505.611	9	CCOC(=O)C1[C@H](C2C(=NC1=C)CC(CC2=O)(C)C)c3ccc(c(c3)OC)COc4ccc(cc4)OC

Supplementary Table S2:
Detailed table of XP Docking using Schrodinger's Glide module

ZINC_ID	xLogP	ApolarDesolvation	PolarDesolvation	H Donors	H Acceptors	Charge	MolecularWeight	RotatableBonds	SMILES
ZINC08441171	6.66	3.54	-13.13	1	5	0	519.548	8	COc1ccc(cc1)c2cc(nc(c2C#N)SCC(=O)Nc3ccccc3C(F)(F)F)c4ccccc4
ZINC08441170	7.04	-1.36	-10.35	3	5	0	479.605	5	Cc1cc(cc(c1)NC(=O)c2c(c3c(cc(nc3s2)c4ccccc4)c5ccc(cc5)OC)N)C
ZINC08441169	4.75	0.41	-12.53	1	8	0	514.559	8	Cc1c(sc(n1)N2[C@H](C(=C(C2=O)O)C(=O)c3cc4ccccc4o3)c5ccc(cc5)OCC=C)C(=O)C
ZINC08441169	4.17	0.25	-13.35	0	8	0	514.559	8	Cc1c(sc(n1)N2[C@H]([C@@H](C(=O)C2=O)C(=O)c3cc4ccccc4o3)c5ccc(cc5)OCC=C)C(=O)C
ZINC06193978	6.64	14.03	-16.41	1	5	0	479.605	7	Cc1cc(cc(c1)NC(=O)CSc2c(c(cc(n2)c3ccc(cc3)OC)c4ccccc4)C#N)C
ZINC08441168	6.78	2.87	-11.57	0	5	0	458.583	8	COc1ccc(cc1)c2cc(nc(c2C#N)SCC(=O)OC3CCCCC3)c4ccccc4
ZINC08441167	4.75	0.33	-12.58	1	8	0	514.559	8	Cc1c(sc(n1)N2[C@@H](C(=C(C2=O)O)C(=O)c3cc4ccccc4o3)c5ccc(cc5)OCC=C)C(=O)C
ZINC08441167	4.17	0.16	-14.35	0	8	0	514.559	8	Cc1c(sc(n1)N2[C@@H]([C@@H](C(=O)C2=O)C(=O)c3cc4ccccc4o3)c5ccc(cc5)OCC=C)C(=O)C
ZINC08441166	6.07	3.83	-16.92	0	7	0	512.587	10	COc1ccc(cc1)c2cc(nc(c2C#N)SCC(=O)Oc3cccc(c3)OC)c4ccc(cc4)OC
ZINC08441165	6.84	4.39	-15.14	0	6	0	510.615	9	Cc1cccc(c1OC(=O)CSc2c(c(cc(n2)c3ccc(cc3)OC)c4ccc(cc4)OC)C#N)C
ZINC08441164	5.53	4.52	-13.25	0	8	0	506.58	12	CCOC(=O)C(C(=O)OCC)Sc1c(c(cc(n1)c2ccc(cc2)OC)c3ccc(cc3)OC)C#N
ZINC08441163	8.37	3.72	-9.95	0	4	0	494.66	8	CC(C)(C)c1ccc(cc1)CSc2c(c(cc(n2)c3ccc(cc3)OC)c4ccc(cc4)OC)C#N
ZINC08441162	5.55	0.16	-12.19	1	8	0	530.602	9	CCCCOc1ccc(cc1)[C@H]2C(=C(C(=O)N2c3nc(c(s3)C(=O)C)C)O)C(=O)c4cc5ccccc5o4
ZINC08441162	4.96	0	-13.06	0	8	0	530.602	9	CCCCOc1ccc(cc1)[C@H]2[C@@H](C(=O)C(=O)N2c3nc(c(s3)C(=O)C)C)C(=O)c4cc5ccccc5o4
ZINC08441161	5.88	2.08	-9.88	0	6	0	479.548	7	CC1=NC2=C([C@@H](C1C(=O)OC)c3ccc(c(c3)OC)COc4ccc(cc4)F)C(=O)CC(C2)(C)C
ZINC08441161	6.06	2.53	-10.46	0	6	0	479.548	7	CC1(CC2=NC(=C)C([C@H](C2C(=O)C1)c3ccc(c(c3)OC)COc4ccc(cc4)F)C(=O)OC)C
ZINC08441161	5.88	2.62	-12.47	0	6	0	479.548	7	CC1=C([C@H]([C@H]2C(=N1)CC(CC2=O)(C)C)c3ccc(c(c3)OC)COc4ccc(cc4)F)C(=O)OC
ZINC08441161	6.06	3.66	-17.01	0	6	0	479.548	7	CC1(CC2=NC(=C)C([C@H]([C@H]2C(=O)C1)c3ccc(c(c3)OC)COc4ccc(cc4)F)C(=O)OC)C
ZINC08441160	7.11	2.89	-10.34	0	4	0	452.579	7	Cc1ccc(cc1)CSc2c(c(cc(n2)c3ccc(cc3)OC)c4ccc(cc4)OC)C#N
ZINC08441159	6.67	2.56	-10.67	0	4	0	438.552	7	COc1ccc(cc1)c2cc(nc(c2C#N)SCc3ccccc3)c4ccc(cc4)OC
ZINC08441158	7.08	-1.29	-10.13	3	5	0	479.605	6	CCc1ccccc1NC(=O)c2c(c3c(cc(nc3s2)c4ccccc4)c5ccc(cc5)OC)N
ZINC08441157	5.55	0.12	-12.24	1	8	0	530.602	9	CCCCOc1ccc(cc1)[C@@H]2C(=C(C(=O)N2c3nc(c(s3)C(=O)C)C)O)C(=O)c4cc5ccccc5o4
ZINC08441157	4.96	-0.06	-14.04	0	8	0	530.602	9	CCCCOc1ccc(cc1)[C@@H]2[C@@H](C(=O)C(=O)N2c3nc(c(s3)C(=O)C)C)C(=O)c4cc5ccccc5o4
ZINC08441156	7.38	-1.64	-11.23	3	5	0	501.611	5	COc1ccc(cc1)c2cc(nc3c2c(c(s3)C(=O)Nc4cccc5c4cccc5)N)c6ccccc6
ZINC08441154	6.63	1.17	-17.66	1	6	0	509.631	10	CCOc1ccc(cc1)c2cc(c(c(n2)SCC(=O)Nc3ccc(cc3)OCC)C#N)c4ccccc4
ZINC08441153	3.68	0.8	-15.82	1	10	0	548.573	8	Cc1c(sc(n1)N2[C@H](C(=C(C2=O)O)C(=O)c3cc4ccccc4o3)c5cc(c(c(c5)OC)OC)OC)C(=O)C
ZINC08441153	3.1	0.75	-16.98	0	10	0	548.573	8	Cc1c(sc(n1)N2[C@H]([C@@H](C(=O)C2=O)C(=O)c3cc4ccccc4o3)c5cc(c(c(c5)OC)OC)OC)C(=O)C
ZINC08441152	6	2.42	-11.16	0	4	0	453.361	7	CCOC(=O)CSc1c(c(cc(n1)c2ccc(cc2)Br)c3ccccc3)C#N
ZINC08441151	7.04	-1.03	-11.1	3	5	0	479.605	5	Cc1ccc(c(c1)NC(=O)c2c(c3c(cc(nc3s2)c4ccccc4)c5ccc(cc5)OC)N)C
ZINC08441150	5.88	2.08	-9.63	0	6	0	479.548	7	CC1=NC2=C([C@H](C1C(=O)OC)c3ccc(c(c3)OC)COc4ccc(cc4)F)C(=O)CC(C2)(C)C
ZINC08441150	6.06	2.42	-10.38	0	6	0	479.548	7	CC1(CC2=NC(=C)C([C@@H](C2C(=O)C1)c3ccc(c(c3)OC)COc4ccc(cc4)F)C(=O)OC)C
ZINC08441150	6.06	3.4	-11.44	0	6	0	479.548	7	CC1(CC2=NC(=C)C([C@@H]([C@@H]2C(=O)C1)c3ccc(c(c3)OC)COc4ccc(cc4)F)C(=O)OC)C
ZINC08441150	5.88	2.46	-12.62	0	6	0	479.548	7	CC1=C([C@@H]([C@H]2C(=N1)CC(CC2=O)(C)C)c3ccc(c(c3)OC)COc4ccc(cc4)F)C(=O)OC
ZINC08441149	3.68	0.8	-15.66	1	10	0	548.573	8	Cc1c(sc(n1)N2[C@@H](C(=C(C2=O)O)C(=O)c3cc4ccccc4o3)c5cc(c(c(c5)OC)OC)OC)C(=O)C
ZINC08441149	3.1	0.59	-17.44	0	10	0	548.573	8	Cc1c(sc(n1)N2[C@@H]([C@@H](C(=O)C2=O)C(=O)c3cc4ccccc4o3)c5cc(c(c(c5)OC)OC)OC)C(=O)C
ZINC08441148	6.64	2	-15.85	1	5	0	479.605	7	Cc1ccc(c(c1)NC(=O)CSc2c(c(cc(n2)c3ccccc3)c4ccc(cc4)OC)C#N)C
ZINC08441147	3.77	-1.53	-14.13	2	9	0	518.547	7	CCOc1cc(ccc1O)[C@H]2C(=C(C(=O)N2c3nc(c(s3)C(=O)C)C)O)C(=O)c4cc5ccccc5o4
ZINC08441147	3.18	-1.75	-16.47	1	9	0	518.547	7	CCOc1cc(ccc1O)[C@H]2[C@H](C(=O)C(=O)N2c3nc(c(s3)C(=O)C)C)C(=O)c4cc5ccccc5o4
ZINC08441146	5.48	3.35	-13.07	0	5	0	432.545	10	CCOC(=O)CCCSc1c(c(cc(n1)c2ccccc2)c3ccc(cc3)OC)C#N
ZINC08441145	3.77	-1.53	-14.19	2	9	0	518.547	7	CCOc1cc(ccc1O)[C@@H]2C(=C(C(=O)N2c3nc(c(s3)C(=O)C)C)O)C(=O)c4cc5ccccc5o4
ZINC08441145	3.18	-1.75	-16.45	1	9	0	518.547	7	CCOc1cc(ccc1O)[C@@H]2[C@@H](C(=O)C(=O)N2c3nc(c(s3)C(=O)C)C)C(=O)c4cc5ccccc5o4
ZINC00850368	5.38	-1.52	-17.79	2	7	0	522.63	10	CCOc1ccc(cc1)c2cc(c(c(n2)SCC(=O)NNC(=O)Cc3ccccc3)C#N)c4ccccc4
ZINC08441144	7.37	1.77	-15.79	1	4	0	485.612	6	Cc1ccc(cc1)c2cc(nc(c2C#N)SCC(=O)Nc3cccc4c3cccc4)c5ccccc5
ZINC08441142	6.48	0.67	-11.65	0	6	0	540.454	7	CC1=NC2=C([C@@H](C1C(=O)OC)c3ccc(c(c3)COc4ccccc4Br)OC)C(=O)CC(C2)(C)C
ZINC08441142	6.66	0.76	-12.34	0	6	0	540.454	7	CC1(CC2=NC(=C)C([C@H](C2C(=O)C1)c3ccc(c(c3)COc4ccccc4Br)OC)C(=O)OC)C
ZINC08441142	6.66	1.98	-12.18	0	6	0	540.454	7	CC1(CC2=NC(=C)C([C@H]([C@H]2C(=O)C1)c3ccc(c(c3)COc4ccccc4Br)OC)C(=O)OC)C
ZINC08441142	6.48	0.88	-14.52	0	6	0	540.454	7	CC1=C([C@H]([C@H]2C(=N1)CC(CC2=O)(C)C)c3ccc(c(c3)COc4ccccc4Br)OC)C(=O)OC
ZINC00702937	4.08	10.83	-14.87	1	9	0	532.574	8	CCOc1ccc(cc1OC)[C@H]2C(=C(C(=O)N2c3nc(c(s3)C(=O)C)C)O)C(=O)c4cc5ccccc5o4
ZINC00702937	3.49	11.66	-17.37	0	9	0	532.574	8	CCOc1ccc(cc1OC)[C@H]2[C@H](C(=O)C(=O)N2c3nc(c(s3)C(=O)C)C)C(=O)c4cc5ccccc5o4
ZINC08441140	4.08	0.41	-14.9	1	9	0	532.574	8	CCOc1ccc(cc1OC)[C@@H]2C(=C(C(=O)N2c3nc(c(s3)C(=O)C)C)O)C(=O)c4cc5ccccc5o4
ZINC08441140	3.49	0.2	-17.33	0	9	0	532.574	8	CCOc1ccc(cc1OC)[C@@H]2[C@@H](C(=O)C(=O)N2c3nc(c(s3)C(=O)C)C)C(=O)c4cc5ccccc5o4
ZINC08441139	6.73	2.19	-15.69	1	6	0	541.698	9	CCOC(=O)c1c(c(sc1NC(=O)CSc2c(c(cc(n2)c3ccccc3)c4ccc(cc4)C)C#N)C)C
ZINC08441138	8.14	18.75	-13.99	0	4	0	511.65	7	Cc1ccc(cc1)c2cc(nc(c2C#N)SCC(=O)N(c3ccccc3)c4ccccc4)c5ccccc5
ZINC08441137	5.64	0.74	-14.52	1	9	0	574.655	11	CCCCCOc1ccc(cc1OC)[C@H]2C(=C(C(=O)N2c3nc(c(s3)C(=O)C)C)O)C(=O)c4cc5ccccc5o4
ZINC08441137	5.06	0.52	-16.88	0	9	0	574.655	11	CCCCCOc1ccc(cc1OC)[C@H]2[C@H](C(=O)C(=O)N2c3nc(c(s3)C(=O)C)C)C(=O)c4cc5ccccc5o4
ZINC08441136	7.98	3.02	-15.36	1	6	0	583.779	10	CCOC(=O)c1c(c(sc1NC(=O)CSc2c(c(cc(n2)c3ccccc3)c4ccc(cc4)C(C)(C)C)C#N)C)C
ZINC00852498	4.69	-3.83	-11.67	2	5	0	423.723	5	c1cc(cc(c1)Br)c2nc([nH]n2)SCC(=O)Nc3ccc(cc3)Cl
ZINC08441135	7.47	2.4	-14.18	1	5	0	507.659	8	CC(C)(C)c1ccc(cc1)c2cc(nc(c2C#N)SCC(=O)Nc3ccccc3OC)c4ccccc4
ZINC08441134	7.52	2.27	-16.37	1	5	0	507.659	8	CC(C)(C)c1ccc(cc1)c2cc(nc(c2C#N)SCC(=O)Nc3ccc(cc3)OC)c4ccccc4
ZINC08441133	5.64	0.73	-14.53	1	9	0	574.655	11	CCCCCOc1ccc(cc1OC)[C@@H]2C(=C(C(=O)N2c3nc(c(s3)C(=O)C)C)O)C(=O)c4cc5ccccc5o4
ZINC08441133	5.06	0.51	-16.9	0	9	0	574.655	11	CCCCCOc1ccc(cc1OC)[C@@H]2[C@@H](C(=O)C(=O)N2c3nc(c(s3)C(=O)C)C)C(=O)c4cc5ccccc5o4

Supplementary Table S2:
Detailed table of XP Docking using Schrodinger's Glide module

ZINC_ID	xLogP	ApolarDesolvation	PolarDesolvation	H Donors	H Acceptors	Charge	MolecularWeight	RotatableBonds	SMILES
ZINC08441132	6.26	1.44	-16.67	1	5	0	465.578	7	Cc1ccc(cc1)c2cc(nc(c2C#N)SCC(=O)Nc3ccc(cc3)OC)c4ccccc4
ZINC08441131	6.48	0.76	-11.7	0	6	0	540.454	7	CC1=NC2=C([C@H](C1C(=O)OC)c3ccc(c(c3)COc4ccccc4Br)OC)C(=O)CC(C2)(C)C
ZINC08441131	6.48	0.7	-14.8	0	6	0	540.454	7	CC1=C([C@@H]([C@H]2C(=N1)CC(CC2=O)(C)C)c3ccc(c(c3)COc4ccccc4Br)OC)C(=O)OC
ZINC08441131	6.66	2.13	-13.57	0	6	0	540.454	7	CC1(CC2=NC(=C)C([C@@H]([C@@H]2C(=O)C1)c3ccc(c(c3)COc4ccccc4Br)OC)C(=O)OC)C
ZINC08441131	6.66	0.82	-12.18	0	6	0	540.454	7	CC1(CC2=NC(=C)C([C@@H](C2C(=O)C1)c3ccc(c(c3)COc4ccccc4Br)OC)C(=O)OC)C
ZINC00702935	3.57	-2.1	-13.19	2	8	0	474.494	5	Cc1c(sc(n1)N2[C@H](C(=C(C2=O)O)C(=O)c3cc4ccccc4o3)c5ccc(cc5)O)C(=O)C
ZINC00702935	2.99	-2.26	-14.25	1	8	0	474.494	5	Cc1c(sc(n1)N2[C@H]([C@@H](C(=O)C2=O)C(=O)c3cc4ccccc4o3)c5ccc(cc5)O)C(=O)C
ZINC08441130	6.64	2.25	-16.61	1	7	0	569.708	10	CCOC(=O)c1c(c(sc1NC(=O)CSc2c(c(cc(n2)c3ccccc3)c4ccc(cc4)C)C#N)C(=O)C)C
ZINC08441129	8.35	3.44	-14.35	0	3	0	496.635	7	Cc1ccc(cc1)c2cc(nc(c2C#N)SCC(=O)c3ccc(cc3)c4ccccc4)c5ccccc5
ZINC00702936	3.57	-2.06	-13.23	2	8	0	474.494	5	Cc1c(sc(n1)N2[C@@H](C(=C(C2=O)O)C(=O)c3cc4ccccc4o3)c5ccc(cc5)O)C(=O)C
ZINC00702936	2.99	-2.23	-15.25	1	8	0	474.494	5	Cc1c(sc(n1)N2[C@@H]([C@@H](C(=O)C2=O)C(=O)c3cc4ccccc4o3)c5ccc(cc5)O)C(=O)C
ZINC08441128	7.36	1.75	-15.65	1	5	0	514.05	8	CCc1ccccc1NC(=O)CSc2c(c(cc(n2)c3ccc(cc3)Cl)c4ccc(cc4)OC)C#N
ZINC08441127	3.95	-1.28	-12.2	1	7	0	464.524	5	Cc1c(sc(n1)N2[C@@H](C(=C(C2=O)O)C(=O)c3cc4ccccc4o3)c5cccs5)C(=O)C
ZINC08441127	3.37	-1.46	-14.11	0	7	0	464.524	5	Cc1c(sc(n1)N2[C@@H]([C@@H](C(=O)C2=O)C(=O)c3cc4ccccc4o3)c5cccs5)C(=O)C
ZINC08441126	7.08	2.19	-14.5	1	4	0	463.606	7	CCc1ccccc1NC(=O)CSc2c(c(cc(n2)c3ccccc3)c4ccc(cc4)C)C#N
ZINC08441125	8.29	3.12	-14.83	1	4	0	505.687	7	Cc1ccc(c(c1)NC(=O)CSc2c(c(cc(n2)c3ccccc3)c4ccc(cc4)C(C)(C)C)C#N)C
ZINC08441124	7.92	2.57	-14.82	1	4	0	491.66	7	Cc1ccc(cc1)NC(=O)CSc2c(c(cc(n2)c3ccccc3)c4ccc(cc4)C(C)(C)C)C#N
ZINC08441123	5.41	0	-12.07	0	6	0	532.438	6	CC1=NC2=C([C@@H](C1C(=O)OC)c3cn(nc3c4ccccc4)Cc5ccc(cc5)Br)C(=O)CCC2
ZINC08441123	5.59	1.31	-15.32	0	6	0	532.438	6	COC(=O)C1[C@H]([C@@H]2C(=NC1=C)CCCC2=O)c3cn(nc3c4ccccc4)Cc5ccc(cc5)Br
ZINC08441123	5.41	0.08	-16.48	0	6	0	532.438	6	CC1=C([C@H]([C@@H]2C(=N1)CCCC2=O)c3cn(nc3c4ccccc4)Cc5ccc(cc5)Br)C(=O)OC
ZINC08441123	5.59	0.19	-13.76	0	6	0	532.438	6	COC(=O)C1[C@H](C2C(=NC1=C)CCCC2=O)c3cn(nc3c4ccccc4)Cc5ccc(cc5)Br
ZINC00702933	4.86	-0.84	-11.45	1	7	0	537.391	5	Cc1c(sc(n1)N2[C@H](C(=C(C2=O)O)C(=O)c3cc4ccccc4o3)c5ccc(cc5)Br)C(=O)C
ZINC00702933	4.28	-1	-12.64	0	7	0	537.391	5	Cc1c(sc(n1)N2[C@H]([C@@H](C(=O)C2=O)C(=O)c3cc4ccccc4o3)c5ccc(cc5)Br)C(=O)C
ZINC08441122	7.87	2.8	-14.63	1	4	0	491.66	7	Cc1ccccc1NC(=O)CSc2c(c(cc(n2)c3ccccc3)c4ccc(cc4)C(C)(C)C)C#N
ZINC00702934	4.86	-0.76	-11.37	1	7	0	537.391	5	Cc1c(sc(n1)N2[C@@H](C(=C(C2=O)O)C(=O)c3cc4ccccc4o3)c5ccc(cc5)Br)C(=O)C
ZINC00702934	4.28	-0.94	-13.49	0	7	0	537.391	5	Cc1c(sc(n1)N2[C@@H]([C@@H](C(=O)C2=O)C(=O)c3cc4ccccc4o3)c5ccc(cc5)Br)C(=O)C
ZINC08441121	8.14	2.11	-14.52	1	4	0	512.078	7	CC(C)(C)c1ccc(cc1)c2cc(nc(c2C#N)SCC(=O)Nc3ccc(cc3)Cl)c4ccccc4
ZINC08441120	9.22	2.91	-16.82	1	5	0	569.73	9	CC(C)(C)c1ccc(cc1)c2cc(nc(c2C#N)SCC(=O)Nc3ccc(cc3)Oc4ccccc4)c5ccccc5
ZINC00702931	5.34	-0.32	-12.21	1	7	0	527.385	5	Cc1c(sc(n1)N2[C@H](C(=C(C2=O)O)C(=O)c3cc4ccccc4o3)c5ccc(c(c5)Cl)Cl)C(=O)C
ZINC00702931	4.75	-0.49	-14.06	0	7	0	527.385	5	Cc1c(sc(n1)N2[C@H]([C@@H](C(=O)C2=O)C(=O)c3cc4ccccc4o3)c5ccc(c(c5)Cl)Cl)C(=O)C
ZINC08441119	7.96	2.42	-13.85	1	4	0	511.65	7	Cc1ccc(cc1)c2cc(nc(c2C#N)SCC(=O)Nc3ccccc3c4ccccc4)c5ccccc5
ZINC08441118	8.27	3.03	-14.87	1	4	0	505.687	7	Cc1cccc(c1C)NC(=O)CSc2c(c(cc(n2)c3ccccc3)c4ccc(cc4)C(C)(C)C)C#N
ZINC00702932	5.34	-0.41	-12.34	1	7	0	527.385	5	Cc1c(sc(n1)N2[C@@H](C(=C(C2=O)O)C(=O)c3cc4ccccc4o3)c5ccc(c(c5)Cl)Cl)C(=O)C
ZINC00702932	4.75	-0.58	-15.27	0	7	0	527.385	5	Cc1c(sc(n1)N2[C@@H]([C@@H](C(=O)C2=O)C(=O)c3cc4ccccc4o3)c5ccc(c(c5)Cl)Cl)C(=O)C
ZINC08441117	7.03	2.29	-15.13	1	4	0	463.606	6	Cc1ccc(cc1)c2cc(nc(c2C#N)SCC(=O)Nc3cc(ccc3C)C)c4ccccc4
ZINC08441116	7.41	-0.82	-9.87	3	4	0	463.606	4	Cc1ccc(cc1)c2cc(nc3c2c(c(s3)C(=O)Nc4cccc(c4C)C)N)c5ccccc5
ZINC08441115	5.41	0.22	-12.88	0	6	0	532.438	6	CC1=NC2=C([C@H](C1C(=O)OC)c3cn(nc3c4ccccc4)Cc5ccc(cc5)Br)C(=O)CCC2
ZINC08441115	5.59	0.26	-13.02	0	6	0	532.438	6	COC(=O)C1[C@@H](C2C(=NC1=C)CCCC2=O)c3cn(nc3c4ccccc4)Cc5ccc(cc5)Br
ZINC08441115	5.41	0.18	-15.31	0	6	0	532.438	6	CC1=C([C@@H]([C@H]2C(=N1)CCCC2=O)c3cn(nc3c4ccccc4)Cc5ccc(cc5)Br)C(=O)OC
ZINC08441115	5.59	1.7	-18.9	0	6	0	532.438	6	COC(=O)C1[C@@H]([C@H]2C(=NC1=C)CCCC2=O)c3cn(nc3c4ccccc4)Cc5ccc(cc5)Br
ZINC08441114	4.22	0.66	-11.85	1	7	0	476.485	5	Cc1c(sc(n1)N2[C@H](C(=C(C2=O)O)C(=O)c3cc4ccccc4o3)c5ccc(cc5)F)C(=O)C
ZINC08441114	3.63	0.5	-13.18	0	7	0	476.485	5	Cc1c(sc(n1)N2[C@H]([C@@H](C(=O)C2=O)C(=O)c3cc4ccccc4o3)c5ccc(cc5)F)C(=O)C
ZINC08441113	7.89	2.58	-14.65	1	4	0	491.66	7	Cc1cccc(c1)NC(=O)CSc2c(c(cc(n2)c3ccccc3)c4ccc(cc4)C(C)(C)C)C#N
ZINC08441112	6.63	1.75	-14.97	1	4	0	449.579	6	Cc1ccc(cc1)c2cc(nc(c2C#N)SCC(=O)Nc3cccc(c3)C)c4ccccc4
ZINC00702930	4.22	0.71	-11.85	1	7	0	476.485	5	Cc1c(sc(n1)N2[C@@H](C(=C(C2=O)O)C(=O)c3cc4ccccc4o3)c5ccc(cc5)F)C(=O)C
ZINC00702930	3.63	0.53	-14.01	0	7	0	476.485	5	Cc1c(sc(n1)N2[C@@H]([C@@H](C(=O)C2=O)C(=O)c3cc4ccccc4o3)c5ccc(cc5)F)C(=O)C
ZINC08441111	7.06	3.84	-12.37	1	4	0	503.549	7	Cc1ccc(cc1)c2cc(nc(c2C#N)SCC(=O)Nc3ccccc3C(F)(F)F)c4ccccc4
ZINC08441110	7.01	2.2	-15.19	1	4	0	463.606	6	Cc1ccc(cc1)c2cc(nc(c2C#N)SCC(=O)Nc3cccc(c3C)C)c4ccccc4
ZINC00702927	4.5	0.25	-11.87	1	7	0	472.522	5	Cc1ccc(cc1)[C@H]2C(=C(C(=O)N2c3nc(c(s3)C(=O)C)C)O)C(=O)c4cc5ccccc5o4
ZINC00702927	3.92	0.09	-12.61	0	7	0	472.522	5	Cc1ccc(cc1)[C@H]2[C@@H](C(=O)C(=O)N2c3nc(c(s3)C(=O)C)C)C(=O)c4cc5ccccc5o4
ZINC08441108	5.74	0.5	-10.2	0	6	0	467.949	7	CC1=NC2=C([C@@H](C1C(=O)OC)c3ccc(c(c3)COc4ccc(cc4)Cl)OC)C(=O)CCC2
ZINC08441108	5.92	0.65	-11.27	0	6	0	467.949	7	COc1ccc(cc1COc2ccc(cc2)Cl)[C@H]3C4C(=NC(=C)C3C(=O)OC)CCCC4=O
ZINC08441108	5.74	0.53	-13.8	0	6	0	467.949	7	CC1=C([C@H]([C@H]2C(=N1)CCCC2=O)c3ccc(c(c3)COc4ccc(cc4)Cl)OC)C(=O)OC
ZINC08441108	5.92	1.95	-18.13	0	6	0	467.949	7	COc1ccc(cc1COc2ccc(cc2)Cl)[C@H]3[C@@H]4C(=NC(=C)C3C(=O)OC)CCCC4=O
ZINC00702928	4.5	0.2	-11.78	1	7	0	472.522	5	Cc1ccc(cc1)[C@@H]2C(=C(C(=O)N2c3nc(c(s3)C(=O)C)C)O)C(=O)c4cc5ccccc5o4
ZINC00702928	3.92	0.03	-13.55	0	7	0	472.522	5	Cc1ccc(cc1)[C@@H]2[C@@H](C(=O)C(=O)N2c3nc(c(s3)C(=O)C)C)C(=O)c4cc5ccccc5o4
ZINC08441106	7.03	2.03	-15.2	1	4	0	463.606	6	Cc1ccc(cc1)c2cc(nc(c2C#N)SCC(=O)Nc3ccc(c(c3)C)C)c4ccccc4
ZINC00702925	4.05	-0.06	-11.93	1	7	0	458.495	5	Cc1c(sc(n1)N2[C@H](C(=C(C2=O)O)C(=O)c3cc4ccccc4o3)c5ccccc5)C(=O)C
ZINC00702925	3.47	-0.23	-12.84	0	7	0	458.495	5	Cc1c(sc(n1)N2[C@H]([C@@H](C(=O)C2=O)C(=O)c3cc4ccccc4o3)c5ccccc5)C(=O)C
ZINC08441105	6.61	1.97	-14.99	1	4	0	449.579	6	Cc1ccc(cc1)c2cc(nc(c2C#N)SCC(=O)Nc3ccccc3C)c4ccccc4
ZINC08441104	6.22	1.56	-14.42	1	5	0	465.578	7	Cc1ccc(cc1)c2cc(nc(c2C#N)SCC(=O)Nc3ccccc3OC)c4ccccc4

Supplementary Table S2:
Detailed table of XP Docking using Schrodinger's Glide module

ZINC_ID	xLogP	ApolarDesolvation	PolarDesolvation	H Donors	H Acceptors	Charge	MolecularWeight	RotatableBonds	SMILES
ZINC08441103	4.05	-0.05	-11.85	1	7	0	458.495	5	Cc1c(sc(n1)N2[C@@H](C(=C(C2=O)O)C(=O)c3cc4ccccc4o3)c5ccccc5)C(=O)C
ZINC08441103	3.47	-0.23	-13.54	0	7	0	458.495	5	Cc1c(sc(n1)N2[C@@H]([C@@H](C(=O)C2=O)C(=O)c3cc4ccccc4o3)c5ccccc5)C(=O)C
ZINC00851206	4.77	-3.25	-12.41	1	5	0	424.707	5	c1ccc(c(c1)c2nnc(o2)SCC(=O)Nc3ccc(cc3)Cl)Br
ZINC13548583	6.71	14.34	-59.79	0	6	-1	499.612	6	CCc1ccc2c(c1)sc(n2)N3[C@H](C(=C(C3=O)[O-])C(=O)c4ccc(o4)C)c5ccc(cc5)C(C)(C)C
ZINC13548583	7.15	13.98	-20.61	1	6	0	500.62	5	CCc1ccc2c(c1)sc(n2)N3[C@H](/C(=C(\c4ccc(o4)C)/O)/C(=O)C3=O)c5ccc(cc5)C(C)(C)C
ZINC13548583	6.12	15.69	-11.39	0	6	0	500.62	6	CCc1ccc2c(c1)sc(n2)N3[C@H]([C@H](C(=O)C3=O)C(=O)c4ccc(o4)C)c5ccc(cc5)C(C)(C)C
ZINC08441101	7.49	-0.75	-10.27	3	5	0	493.632	5	Cc1ccc(cc1)c2cc(c3c(c(sc3n2)C(=O)Nc4ccc(cc4C)C)N)c5ccc(cc5)OC
ZINC08441100	5.74	0.44	-13.75	0	6	0	467.949	7	CC1=NC2=C([C@H](C1C(=O)OC)c3ccc(c(c3)COc4ccc(cc4)Cl)OC)C(=O)CCC2
ZINC08441100	5.92	1.71	-14.28	0	6	0	467.949	7	COc1ccc(cc1COc2ccc(cc2)Cl)[C@@H]3[C@H]4C(=NC(=C)C3C(=O)OC)CCCC4=O
ZINC08441100	5.92	0.55	-13.36	0	6	0	467.949	7	COc1ccc(cc1COc2ccc(cc2)Cl)[C@@H]3C4C(=NC(=C)C3C(=O)OC)CCCC4=O
ZINC08441100	5.74	0.47	-15.84	0	6	0	467.949	7	CC1=C([C@@H]([C@H]2C(=N1)CCCC2=O)c3ccc(c(c3)COc4ccc(cc4)Cl)OC)C(=O)OC
ZINC08441098	7.01	-1.12	-9.57	3	4	0	449.579	4	Cc1ccc(cc1)c2cc(c3c(c(sc3n2)C(=O)Nc4ccccc4C)N)c5ccccc5
ZINC13548585	6.71	14.41	-59.82	0	6	-1	499.612	6	CCc1ccc2c(c1)sc(n2)N3[C@@H](C(=C(C3=O)[O-])C(=O)c4ccc(o4)C)c5ccc(cc5)C(C)(C)C
ZINC13548585	7.15	14.2	-20.04	1	6	0	500.62	5	CCc1ccc2c(c1)sc(n2)N3[C@@H](/C(=C(\c4ccc(o4)C)/O)/C(=O)C3=O)c5ccc(cc5)C(C)(C)C
ZINC13548585	6.12	15.77	-11.3	0	6	0	500.62	6	CCc1ccc2c(c1)sc(n2)N3[C@@H]([C@@H](C(=O)C3=O)C(=O)c4ccc(o4)C)c5ccc(cc5)C(C)(C)C
ZINC00851196	4.96	-0.93	-12.11	1	5	0	458.259	6	c1ccc(c(c1)c2nnc(o2)SCC(=O)Nc3cccc(c3)C(F)(F)F)Br
ZINC06246284	7.08	13.11	-12.22	3	6	0	523.658	6	Cc1ccc(cc1)c2cc(c3c(c(sc3n2)C(=O)Nc4cc(ccc4C)C)N)c5ccc(c(c5)OC)OC
ZINC08741004	5.59	11.81	-54.95	0	6	-1	508.373	5	CCc1ccc2c(c1)sc(n2)N3[C@H](C(=C(C3=O)[O-])C(=O)c4ccco4)c5ccc(cc5)Br
ZINC08741004	6.04	12.01	-19	1	6	0	509.381	4	CCc1ccc2c(c1)sc(n2)N3[C@H](/C(=C(\c4ccco4)/O)/C(=O)C3=O)c5ccc(cc5)Br
ZINC08441096	6.2	0.24	-12.29	0	6	0	526.427	8	CCOC(=O)C1[C@@H](C2=C(CCCC2=O)N=C1C)c3ccc(c(c3)COc4ccccc4Br)OC
ZINC08441096	6.38	-0.87	-13.37	0	6	0	526.427	8	CCOC(=O)C1[C@@H](C2C(=NC1=C)CCCC2=O)c3ccc(c(c3)COc4ccccc4Br)OC
ZINC08441096	6.2	0.05	-15.65	0	6	0	526.427	8	CCOC(=O)C1=C(N=C2CCCC(=O)C2[C@H]1c3ccc(c(c3)COc4ccccc4Br)OC)C
ZINC08441095	7.03	2.29	-15.03	1	4	0	463.606	6	Cc1ccc(cc1)c2cc(c(c(n2)SCC(=O)Nc3cc(ccc3C)C)C#N)c4ccccc4
ZINC08741003	5.59	11.89	-54.81	0	6	-1	508.373	5	CCc1ccc2c(c1)sc(n2)N3[C@@H](C(=C(C3=O)[O-])C(=O)c4ccco4)c5ccc(cc5)Br
ZINC08741003	6.04	11.92	-19.83	1	6	0	509.381	4	CCc1ccc2c(c1)sc(n2)N3[C@@H](/C(=C(\c4ccco4)/O)/C(=O)C3=O)c5ccc(cc5)Br
ZINC08441094	4.85	-3.64	-10.96	1	5	0	469.158	5	c1ccc(c(c1)c2nnc(o2)SCC(=O)Nc3ccccc3Br)Br
ZINC08441093	6.2	0.25	-12.17	0	6	0	526.427	8	CCOC(=O)C1[C@H](C2=C(CCCC2=O)N=C1C)c3ccc(c(c3)COc4ccccc4Br)OC
ZINC08441093	6.2	0.06	-15.8	0	6	0	526.427	8	CCOC(=O)C1=C(N=C2CCCC(=O)C2[C@@H]1c3ccc(c(c3)COc4ccccc4Br)OC)C
ZINC08441093	6.38	-0.86	-13.38	0	6	0	526.427	8	CCOC(=O)C1[C@H](C2C(=NC1=C)CCCC2=O)c3ccc(c(c3)COc4ccccc4Br)OC
ZINC00702909	4.23	10.87	-15.51	0	7	0	431.901	8	CCn1c(nnc1SCC(=O)c2ccc(cc2)Cl)COc3ccc4c(c3)OCO4
ZINC08441091	6.66	2.55	-17.58	1	6	0	523.658	8	Cc1ccc(cc1)c2cc(c(c(n2)SCC(=O)Nc3cccc(c3C)C)C#N)c4ccc(c(c4)OC)OC
ZINC08441090	5.72	2.62	-16.92	1	6	0	523.658	8	Cc1ccc(cc1)c2cc(c(c(n2)SCC(=O)Nc3c(cccc3C)C)C#N)c4ccc(c(c4)OC)OC
ZINC08441089	6.68	2.67	-17.63	1	6	0	523.658	8	Cc1ccc(cc1)c2cc(c(c(n2)SCC(=O)Nc3ccc(cc3C)C)C#N)c4ccc(c(c4)OC)OC
ZINC08441088	7.49	-0.78	-10.36	3	5	0	493.632	5	Cc1ccc(cc1)c2cc(c3c(c(sc3n2)C(=O)Nc4cc(ccc4C)C)N)c5ccc(cc5)OC
ZINC08441087	5.5	3.62	-14.28	1	6	0	454.505	5	[H]/N=C/1\[C@H](C2=CCCC[C@@H]2[C@@H](C1(C#N)C#N)c3ccc(c(c3)OC)COc4ccc(cc4)F)C#N
ZINC08441087	5.5	3.45	-40.85	2	6	1	455.513	5	COc1cc(ccc1COc2ccc(cc2)F)[C@H]3[C@H]4CCCC=C4[C@@H](C(=[NH2+])C3(C#N)C#N)C#N
ZINC09185694	7.39	15.34	-54.35	0	5	-1	513.614	6	CCc1ccc2c(c1)sc(n2)N3[C@H](C(=C(C3=O)[O-])C(=O)c4ccc(cc4)F)c5ccc(cc5)C(C)(C)C
ZINC09185694	7.84	2.07	-18.72	1	5	0	514.622	5	CCc1ccc2c(c1)sc(n2)N3[C@H](/C(=C(\c4ccc(cc4)F)/O)/C(=O)C3=O)c5ccc(cc5)C(C)(C)C
ZINC08441085	6.68	2.65	-17.55	1	6	0	523.658	8	Cc1ccc(cc1)c2cc(c(c(n2)SCC(=O)Nc3cc(ccc3C)C)C#N)c4ccc(c(c4)OC)OC
ZINC08441084	5.5	3.29	-12.5	1	6	0	454.505	5	[H]/N=C/1\[C@H](C2=CCCC[C@@H]2[C@H](C1(C#N)C#N)c3ccc(c(c3)OC)COc4ccc(cc4)F)C#N
ZINC08441084	5.5	3.12	-45.3	2	6	1	455.513	5	COc1cc(ccc1COc2ccc(cc2)F)[C@@H]3[C@H]4CCCC=C4[C@@H](C(=[NH2+])C3(C#N)C#N)C#N
ZINC09185693	7.39	15.39	-54.25	0	5	-1	513.614	6	CCc1ccc2c(c1)sc(n2)N3[C@@H](C(=C(C3=O)[O-])C(=O)c4ccc(cc4)F)c5ccc(cc5)C(C)(C)C
ZINC09185693	7.84	1.77	-18.06	1	5	0	514.622	5	CCc1ccc2c(c1)sc(n2)N3[C@@H](/C(=C(\c4ccc(cc4)F)/O)/C(=O)C3=O)c5ccc(cc5)C(C)(C)C
ZINC08441082	5.66	3.53	-11.01	0	5	0	432.545	9	CCOC(=O)CCSc1c(c(cc(n1)c2ccc(cc2)C)c3ccc(cc3)OC)C#N
ZINC06474387	7.32	14.03	-10.7	3	4	0	471.585	4	c1ccc(cc1)c2cc(nc3c2c(c(s3)C(=O)Nc4cccc5c4cccc5)N)c6ccccc6
ZINC08441081	5.5	3.29	-12.46	1	6	0	454.505	5	[H]/N=C/1\[C@@H](C2=CCCC[C@H]2[C@@H](C1(C#N)C#N)c3ccc(c(c3)OC)COc4ccc(cc4)F)C#N
ZINC08441081	5.5	3.12	-45.26	2	6	1	455.513	5	COc1cc(ccc1COc2ccc(cc2)F)[C@H]3[C@@H]4CCCC=C4[C@H](C(=[NH2+])C3(C#N)C#N)C#N
ZINC08441080	6.33	13.62	-55.05	0	5	-1	518.412	5	CCc1ccc2c(c1)sc(n2)N3[C@H](C(=C(C3=O)[O-])C(=O)c4ccccc4)c5ccc(cc5)Br
ZINC08441080	5.75	-1.36	-15.47	0	5	0	519.42	5	CCc1ccc2c(c1)sc(n2)N3[C@H]([C@@H](C(=O)C3=O)C(=O)c4ccccc4)c5ccc(cc5)Br
ZINC05520252	3.76	6.62	-13.46	3	6	0	354.435	5	Cc1cccc(c1C)NC(=O)CSc2[nH]nc(n2)c3ccccc3O
ZINC06556763	3.41	5.89	-13.48	3	6	0	340.408	5	Cc1ccc(cc1)NC(=O)CSc2[nH]nc(n2)c3ccccc3O
ZINC08441079	6.56	1.87	-15.14	1	4	0	449.579	6	Cc1cccc(c1C)NC(=O)CSc2c(c(cc(n2)c3ccccc3)c4ccccc4)C#N
ZINC08441078	5.5	3.63	-14.29	1	6	0	454.505	5	[H]/N=C/1\[C@@H](C2=CCCC[C@H]2[C@H](C1(C#N)C#N)c3ccc(c(c3)OC)COc4ccc(cc4)F)C#N
ZINC08441078	5.5	3.46	-40.93	2	6	1	455.513	5	COc1cc(ccc1COc2ccc(cc2)F)[C@@H]3[C@@H]4CCCC=C4[C@H](C(=[NH2+])C3(C#N)C#N)C#N
ZINC08441077	6.33	13.6	-54.88	0	5	-1	518.412	5	CCc1ccc2c(c1)sc(n2)N3[C@@H](C(=C(C3=O)[O-])C(=O)c4ccccc4)c5ccc(cc5)Br
ZINC08441077	5.75	-1.29	-17.14	0	5	0	519.42	5	CCc1ccc2c(c1)sc(n2)N3[C@@H]([C@@H](C(=O)C3=O)C(=O)c4ccccc4)c5ccc(cc5)Br
ZINC00850364	4.77	-3.38	-12.54	1	5	0	424.707	5	c1ccc(c(c1)c2nnc(o2)SCC(=O)Nc3ccc(cc3)Br)Cl
ZINC08441076	4.25	-4.7	-11.4	2	6	0	420.288	5	Cc1ccc(c(c1)Br)NC(=O)CSc2nnc(o2)c3ccccc3O
ZINC06445734	4.64	10.7	-10.06	2	5	0	428.97	5	c1cc(ccc1c2cnc([nH]2)SCC(=O)Nc3c(c4c(s3)CCCC4)C#N)Cl
ZINC06445734	4.64	11.15	-46.04	3	5	1	429.978	5	c1cc(ccc1c2c[nH+]c([nH]2)SCC(=O)Nc3c(c4c(s3)CCCC4)C#N)Cl
ZINC08441075	6.99	-1.34	-9.91	3	4	0	449.579	4	Cc1cc(cc(c1)NC(=O)c2c(c3c(cc(nc3s2)c4ccccc4)c5ccccc5)N)C

Supplementary Table S2:
Detailed table of XP Docking using Schrodinger's Glide module

ZINC_ID	xLogP	ApolarDesolvation	PolarDesolvation	H Donors	H Acceptors	Charge	MolecularWeight	RotatableBonds	SMILES
ZINC08441074	5.57	0.49	-9.92	0	6	0	500.389	6	CC1=NC2=C([C@@H](C1C(=O)OC)c3ccc(o3)COc4ccc(cc4)Br)C(=O)CC(C2)(C)C
ZINC08441074	5.75	0.5	-11.71	0	6	0	500.389	6	CC1(CC2=NC(=C)C([C@H](C2C(=O)C1)c3ccc(o3)COc4ccc(cc4)Br)C(=O)OC)C
ZINC08441074	5.75	1.67	-10.35	0	6	0	500.389	6	CC1(CC2=NC(=C)C([C@H]([C@@H]2C(=O)C1)c3ccc(o3)COc4ccc(cc4)Br)C(=O)OC)C
ZINC08441074	5.57	0.53	-14.09	0	6	0	500.389	6	CC1=C([C@H]([C@@H]2C(=N1)CC(CC2=O)(C)C)c3ccc(o3)COc4ccc(cc4)Br)C(=O)OC
ZINC08771456	7.13	14.54	-58.55	0	5	-1	501.653	6	CCc1ccc2c(c1)sc(n2)N3[C@H](C(=C(C3=O)[O-])C(=O)c4cccs4)c5ccc(cc5)C(C)(C)C
ZINC08915059	7.13	14.57	-58.33	0	5	-1	501.653	6	CCc1ccc2c(c1)sc(n2)N3[C@@H](C(=C(C3=O)[O-])C(=O)c4cccs4)c5ccc(cc5)C(C)(C)C
ZINC08915059	7.57	13.75	-17.74	1	5	0	502.661	5	CCc1ccc2c(c1)sc(n2)N3[C@@H](/C(=C(\c4cccs4)/O)/C(=O)C3=O)c5ccc(cc5)C(C)(C)C
ZINC08441071	7.32	1.8	-15.43	1	4	0	485.612	6	Cc1ccc2ccccc2c1NC(=O)CSc3c(c(cc(n3)c4ccccc4)c5ccccc5)C#N
ZINC08441070	6.58	1.74	-15.12	1	4	0	449.579	6	Cc1cc(cc(c1)NC(=O)CSc2c(c(cc(n2)c3ccccc3)c4ccccc4)C#N)C
ZINC08441069	6.5	13.69	-50.28	0	5	-1	536.402	5	CCc1ccc2c(c1)sc(n2)N3[C@H](C(=C(C3=O)[O-])C(=O)c4ccc(cc4)F)c5ccc(cc5)Br
ZINC08441069	5.91	-0.62	-15.21	0	5	0	537.41	5	CCc1ccc2c(c1)sc(n2)N3[C@H]([C@@H](C(=O)C3=O)C(=O)c4ccc(cc4)F)c5ccc(cc5)Br
ZINC08441066	4.91	0.73	-19.13	1	6	0	535.407	6	CCOC(=O)c1c2c(sc1N/C=C\3/C(=NN(C3=O)c4ccc(cc4)I)C)CCCC2
ZINC08441065	5.57	0.51	-10.65	0	6	0	500.389	6	CC1=NC2=C([C@H](C1C(=O)OC)c3ccc(o3)COc4ccc(cc4)Br)C(=O)CC(C2)(C)C
ZINC08441065	5.75	1.97	-12.37	0	6	0	500.389	6	CC1(CC2=NC(=C)C([C@@H]([C@H]2C(=O)C1)c3ccc(o3)COc4ccc(cc4)Br)C(=O)OC)C
ZINC08441065	5.75	0.52	-10.48	0	6	0	500.389	6	CC1(CC2=NC(=C)C([C@@H](C2C(=O)C1)c3ccc(o3)COc4ccc(cc4)Br)C(=O)OC)C
ZINC08441065	5.57	0.58	-12	0	6	0	500.389	6	CC1=C([C@@H]([C@@H]2C(=N1)CC(CC2=O)(C)C)c3ccc(o3)COc4ccc(cc4)Br)C(=O)OC
ZINC08441064	6.5	13.66	-50.16	0	5	-1	536.402	5	CCc1ccc2c(c1)sc(n2)N3[C@@H](C(=C(C3=O)[O-])C(=O)c4ccc(cc4)F)c5ccc(cc5)Br
ZINC08441064	5.91	-0.61	-15.17	0	5	0	537.41	5	CCc1ccc2c(c1)sc(n2)N3[C@@H]([C@H](C(=O)C3=O)C(=O)c4ccc(cc4)F)c5ccc(cc5)Br
ZINC02063637	6.32	2.37	-10.87	1	4	0	467.266	4	c1cc(cc(c1)Nc2nc(cs2)c3cc4cc(ccc4oc3=O)Br)C(F)(F)F
ZINC00856352	5.57	10.65	-12.51	1	5	0	418.396	5	COc1ccc2c(c1)cc(c(=O)o2)c3csc(n3)Nc4cccc(c4)C(F)(F)F
ZINC08441063	7.01	14.15	-50.33	0	5	-1	552.857	5	CCc1ccc2c(c1)sc(n2)N3[C@H](C(=C(C3=O)[O-])C(=O)c4ccc(cc4)Cl)c5ccc(cc5)Br
ZINC08441063	7.46	-1.74	-25.78	1	5	0	553.865	4	CCc1ccc2c(c1)sc(n2)N3[C@H](C(=C(c4ccc(cc4)Cl)O)C(=O)C3=O)c5ccc(cc5)Br
ZINC08441063	6.43	-1.51	-14.85	0	5	0	553.865	5	CCc1ccc2c(c1)sc(n2)N3[C@H]([C@@H](C(=O)C3=O)C(=O)c4ccc(cc4)Cl)c5ccc(cc5)Br
ZINC02063636	7.97	1.12	-13.18	2	7	0	492.597	11	CCCCCCc1cc2cc(c(=O)oc2cc1O)c3csc(n3)Nc4ccc(cc4)C(=O)OCC
ZINC08441062	7.01	14.11	-50.19	0	5	-1	552.857	5	CCc1ccc2c(c1)sc(n2)N3[C@@H](C(=C(C3=O)[O-])C(=O)c4ccc(cc4)Cl)c5ccc(cc5)Br
ZINC08441062	7.46	-1.72	-25.28	1	5	0	553.865	4	CCc1ccc2c(c1)sc(n2)N3[C@@H](C(=C(c4ccc(cc4)Cl)O)C(=O)C3=O)c5ccc(cc5)Br
ZINC08441062	6.43	-1.5	-14.64	0	5	0	553.865	5	CCc1ccc2c(c1)sc(n2)N3[C@@H]([C@H](C(=O)C3=O)C(=O)c4ccc(cc4)Cl)c5ccc(cc5)Br
ZINC00856338	5.86	12.45	-10.93	1	6	0	426.881	6	CCOC(=O)c1ccc(cc1)Nc2nc(cs2)c3cc4cc(ccc4oc3=O)Cl
ZINC00856337	5.22	2.26	-16.36	1	7	0	422.462	7	CCOC(=O)c1ccc(cc1)Nc2nc(cs2)c3cc4cccc(c4oc3=O)OC
ZINC02063635	6.37	2.14	-13.57	1	6	0	442.496	6	CCOC(=O)c1ccc(cc1)Nc2nc(cs2)c3cc4c5ccccc5ccc4oc3=O
ZINC08441061	6.06	0.79	-11.34	0	6	0	560.492	6	CC1=NC2=C([C@@H](C1C(=O)OC)c3cn(nc3c4ccccc4)Cc5ccc(cc5)Br)C(=O)CC(C2)(C)C
ZINC08441061	6.25	1.19	-12.94	0	6	0	560.492	6	CC1(CC2=NC(=C)C([C@H](C2C(=O)C1)c3cn(nc3c4ccccc4)Cc5ccc(cc5)Br)C(=O)OC)C
ZINC08441061	6.06	1.27	-15.43	0	6	0	560.492	6	CC1=C([C@H]([C@H]2C(=N1)CC(CC2=O)(C)C)c3cn(nc3c4ccccc4)Cc5ccc(cc5)Br)C(=O)OC
ZINC08441061	6.25	2.1	-11.74	0	6	0	560.492	6	CC1(CC2=NC(=C)C([C@H]([C@H]2C(=O)C1)c3cn(nc3c4ccccc4)Cc5ccc(cc5)Br)C(=O)OC)C
ZINC08441060	7.91	15.84	-54.29	0	5	-1	530.069	6	CCc1ccc2c(c1)sc(n2)N3[C@H](C(=C(C3=O)[O-])C(=O)c4ccc(cc4)Cl)c5ccc(cc5)C(C)(C)C
ZINC08441060	7.32	0.41	-14.37	0	5	0	531.077	6	CCc1ccc2c(c1)sc(n2)N3[C@H]([C@@H](C(=O)C3=O)C(=O)c4ccc(cc4)Cl)c5ccc(cc5)C(C)(C)C
ZINC00856322	5.03	0.83	-10.48	0	5	0	427.279	2	COc1ccc2c(c1)sc3n2cc(n3)c4cc5cc(ccc5oc4=O)Br
ZINC00856315	5.77	1.68	-13.87	1	7	0	431.473	5	COc1ccc(c(c1)OC)Nc2nnc(s2)c3cc4c5ccccc5ccc4oc3=O
ZINC00856312	5.49	1.35	-13.21	1	5	0	418.247	3	c1cc(cc(c1)F)Nc2nnc(s2)c3cc4cc(ccc4oc3=O)Br
ZINC08441059	7.91	15.81	-54.11	0	5	-1	530.069	6	CCc1ccc2c(c1)sc(n2)N3[C@@H](C(=C(C3=O)[O-])C(=O)c4ccc(cc4)Cl)c5ccc(cc5)C(C)(C)C
ZINC08441059	7.32	0.42	-14.34	0	5	0	531.077	6	CCc1ccc2c(c1)sc(n2)N3[C@@H]([C@H](C(=O)C3=O)C(=O)c4ccc(cc4)Cl)c5ccc(cc5)C(C)(C)C
ZINC02063632	6.09	-0.14	-11.19	1	5	0	479.153	3	c1ccc(cc1)Nc2nnc(s2)c3cc4cc(cc(c4oc3=O)Br)Br
ZINC09357898	2.52	11.62	-70.7	1	8	0	479.92	7	c1cc(ccc1[C@@H]2C(=C(C(=O)N2CCCn3cc[nH+]c3)[O-])C(=O)c4ccc5c(c4)OCCO5)Cl
ZINC09357898	2.52	11.11	-63.77	0	8	-1	478.912	7	c1cc(ccc1[C@@H]2C(=C(C(=O)N2CCCn3ccnc3)[O-])C(=O)c4ccc5c(c4)OCCO5)Cl
ZINC02063630	6.56	2.23	-14.88	1	6	0	442.544	6	CCN(CC)c1ccc2cc(c(=O)oc2c1)c3nnc(s3)Nc4cccc5c4cccc5
ZINC02063629	6.24	1.52	-13.71	1	6	0	434.304	5	CCOc1cccc2c1oc(=O)c(c2)c3nnc(s3)Nc4ccc(cc4Cl)Cl
ZINC02063628	6.54	0.7	-12.75	1	5	0	450.317	3	c1ccc2c(c1)ccc3c2cc(c(=O)o3)c4nnc(s4)Nc5ccc(cc5)Br
ZINC00856288	5.15	2.47	-15.71	1	8	0	423.45	7	CCOC(=O)c1ccc(cc1)Nc2nnc(s2)c3cc4cc(ccc4oc3=O)OC
ZINC08996455	2.4	7.58	-49.17	2	9	1	469.514	7	C[NH+](C)CCN1[C@H](/C(=C(/c2ccc3c(c2)OCCO3)\O)/C(=O)C1=O)c4cc(ccc4OC)OC
ZINC08996455	1.37	8.24	-48.96	1	9	1	469.514	8	C[NH+](C)CCN1[C@H]([C@@H](C(=O)C1=O)C(=O)c2ccc3c(c2)OCCO3)c4cc(ccc4OC)OC
ZINC08441056	6.06	0.93	-12.26	0	6	0	560.492	6	CC1=NC2=C([C@H](C1C(=O)OC)c3cn(nc3c4ccccc4)Cc5ccc(cc5)Br)C(=O)CC(C2)(C)C
ZINC08441056	6.25	2.24	-12.59	0	6	0	560.492	6	CC1(CC2=NC(=C)C([C@@H]([C@@H]2C(=O)C1)c3cn(nc3c4ccccc4)Cc5ccc(cc5)Br)C(=O)OC)C
ZINC08441056	6.06	1.4	-14.16	0	6	0	560.492	6	CC1=C([C@@H]([C@@H]2C(=N1)CC(CC2=O)(C)C)c3cn(nc3c4ccccc4)Cc5ccc(cc5)Br)C(=O)OC
ZINC08441056	6.25	1.32	-12.59	0	6	0	560.492	6	CC1(CC2=NC(=C)C([C@@H](C2C(=O)C1)c3cn(nc3c4ccccc4)Cc5ccc(cc5)Br)C(=O)OC)C
ZINC00856284	5.47	3.56	-11.7	1	6	0	419.384	5	COc1ccc2c(c1)cc(c(=O)o2)c3nnc(s3)Nc4cccc(c4)C(F)(F)F
ZINC02063627	6.51	1	-11.71	1	5	0	450.317	3	c1ccc2c(c1)cccc2Nc3nnc(s3)c4cc5cc(ccc5oc4=O)Br
ZINC08996454	2.4	6.67	-50.62	2	9	1	469.514	7	C[NH+](C)CCN1[C@@H](/C(=C(/c2ccc3c(c2)OCCO3)\O)/C(=O)C1=O)c4cc(ccc4OC)OC
ZINC08441053	6.28	12.89	-54.04	0	6	-1	548.438	5	Cc1ccc2c(c1)sc(n2)N3[C@H](C(=C(C3=O)[O-])C(=O)c4ccc(c(c4)C)OC)c5cccc(c5)Br
ZINC08441053	5.69	-1.07	-14.87	0	6	0	549.446	5	Cc1ccc2c(c1)sc(n2)N3[C@H]([C@@H](C(=O)C3=O)C(=O)c4ccc(c(c4)C)OC)c5cccc(c5)Br
ZINC00856250	4.52	1.25	-15.8	0	8	0	430.416	5	COc1cc(cc(c1OC)OC)c2nnc(o2)c3cc4c5ccccc5ccc4oc3=O
ZINC08441052	6.09	8.55	-28.88	2	4	1	414.304	2	Cc1ccc(cc1)c2csc(n2)c\3cc4cc(ccc4o/c3=[NH+]\O)Br

Supplementary Table S2:
Detailed table of XP Docking using Schrodinger's Glide module

ZINC_ID	xLogP	ApolarDesolvation	PolarDesolvation	H Donors	H Acceptors	Charge	MolecularWeight	RotatableBonds	SMILES
ZINC08441052	6.09	8.25	-30.56	2	4	1	414.304	2	Cc1ccc(cc1)c2csc(n2)c\3cc4cc(ccc4o/c3=[NH+]/O)Br
ZINC08441051	5.83	0	-12.25	0	6	0	512.4	7	CC1=NC2=C([C@@H](C1C(=O)OC)c3ccc(c(c3)COc4ccccc4Br)OC)C(=O)CCC2
ZINC08441051	6.01	0.26	-13.48	0	6	0	512.4	7	COc1ccc(cc1COc2ccccc2Br)[C@H]3C4C(=NC(=C)C3C(=O)OC)CCCC4=O
ZINC08441051	6.01	1.31	-13.81	0	6	0	512.4	7	COc1ccc(cc1COc2ccccc2Br)[C@H]3[C@@H]4C(=NC(=C)C3C(=O)OC)CCCC4=O
ZINC08441051	5.83	0.1	-15.75	0	6	0	512.4	7	CC1=C([C@H]([C@H]2C(=N1)CCCC2=O)c3ccc(c(c3)COc4ccccc4Br)OC)C(=O)OC
ZINC08441050	6.28	12.86	-53.92	0	6	-1	548.438	5	Cc1ccc2c(c1)sc(n2)N3[C@@H](C(=C(C3=O)[O-])C(=O)c4ccc(c(c4)C)OC)c5cccc(c5)Br
ZINC08441050	5.69	-1.06	-18.06	0	6	0	549.446	5	Cc1ccc2c(c1)sc(n2)N3[C@@H]([C@H](C(=O)C3=O)C(=O)c4ccc(c(c4)C)OC)c5cccc(c5)Br
ZINC08441046	4.82	-5.13	-16.06	0	5	0	476.179	2	c1cc(ccc1N2[C@@H]3CS(=O)(=O)C[C@H]3N(C2=O)c4ccc(c(c4)Cl)Cl)Br
ZINC08441045	7.02	12.61	-12.45	1	6	0	448.544	7	CCCCCCc1cc2cc(c(=O)oc2cc1O)c3cn4c5ccc(cc5sc4n3)OC
ZINC00970921	5.43	1.14	-10.9	0	4	0	411.28	1	Cc1ccc2c(c1)sc3n2cc(n3)c4cc5cc(ccc5oc4=O)Br
ZINC08441044	4.82	-4.7	-25.11	0	5	0	476.179	2	c1cc(ccc1N2[C@@H]3CS(=O)(=O)C[C@@H]3N(C2=O)c4ccc(c(c4)Cl)Cl)Br
ZINC02063584	6.6	12.94	-12.11	1	5	0	439.418	4	c1ccc2c(c1)ccc3c2cc(c(=O)o3)c4nnc(s4)Nc5cccc(c5)C(F)(F)F
ZINC08441042	6.98	2.23	-12.38	0	4	0	435.523	6	COc1ccc(cc1)c2cc3cc(c(cc3c(n2)Cc4cccc5c4cccc5)OC)OC
ZINC08441041	4.19	-5	-19.44	0	5	0	441.734	2	c1cc(cc(c1)Cl)N2[C@@H]3CS(=O)(=O)C[C@H]3N(C2=O)c4ccc(cc4)Br
ZINC08441040	5.83	-0.03	-12.29	0	6	0	512.4	7	CC1=NC2=C([C@H](C1C(=O)OC)c3ccc(c(c3)COc4ccccc4Br)OC)C(=O)CCC2
ZINC08441040	6.01	1.45	-19.17	0	6	0	512.4	7	COc1ccc(cc1COc2ccccc2Br)[C@@H]3[C@H]4C(=NC(=C)C3C(=O)OC)CCCC4=O
ZINC08441040	6.01	0.03	-13.07	0	6	0	512.4	7	COc1ccc(cc1COc2ccccc2Br)[C@@H]3C4C(=NC(=C)C3C(=O)OC)CCCC4=O
ZINC08441040	5.83	-0.09	-15.79	0	6	0	512.4	7	CC1=C([C@@H]([C@H]2C(=N1)CCCC2=O)c3ccc(c(c3)COc4ccccc4Br)OC)C(=O)OC
ZINC02063579	5.45	2.91	-18.99	2	4	0	505.267	8	c1cc(c(cc1C(F)(F)F)NC(=O)CSCC(=O)Nc2cc(ccc2Cl)C(F)(F)F)Cl
ZINC08441039	4.19	-4.53	-21.12	0	5	0	441.734	2	c1cc(cc(c1)Cl)N2[C@@H]3CS(=O)(=O)C[C@@H]3N(C2=O)c4ccc(cc4)Br
ZINC00849797	4.79	-3.8	-9.22	1	5	0	424.707	5	c1cc(cc(c1)Cl)NC(=O)CSc2nnc(o2)c3ccc(cc3)Br
ZINC00849793	5.01	-1.47	-9.69	1	5	0	458.259	6	c1cc(cc(c1)NC(=O)CSc2nnc(o2)c3ccc(cc3)Br)C(F)(F)F
ZINC06144687	4.08	-5.4	-18.59	0	4	0	423.357	2	c1ccc(cc1)N2[C@@H]3CS(=O)(=O)C[C@H]3N(C2=S)c4ccc(cc4)Br
ZINC06144685	4.08	-5.02	-20.85	0	4	0	423.357	2	c1ccc(cc1)N2[C@@H]3CS(=O)(=O)C[C@@H]3N(C2=S)c4ccc(cc4)Br
ZINC08441038	6.58	1.97	-15.07	1	4	0	449.579	6	Cc1ccc(c(c1)NC(=O)CSc2c(c(cc(n2)c3ccccc3)c4ccccc4)C#N)C
ZINC06474284	5.97	13.2	-14.06	1	4	0	427.573	6	c1ccc(cc1)c2cc(nc(c2C#N)SCC(=O)NC3CCCCC3)c4ccccc4
ZINC00850105	4.78	11.5	-20.9	3	7	0	491.547	6	Cc1cccc(c1)NC(=O)COc2ccc(cc2OC)[C@@H]3c4ccc5ccccc5c4OC(=C3C#N)N
ZINC08441037	5.15	-1.79	-8.55	0	3	0	567.135	3	CC1([C@@]2(CC[C@]1([C@@H]2Br)C(=O)N3CCN(CC3)c4ccc(cc4)F)C(Br)Br)C
ZINC08441036	4.91	-0.22	-10.83	0	6	0	472.335	6	CC1=NC2=C([C@@H](C1C(=O)OC)c3ccc(o3)COc4ccc(cc4)Br)C(=O)CCC2
ZINC08441036	4.91	-0.23	-15.88	0	6	0	472.335	6	CC1=C([C@H]([C@@H]2C(=N1)CCCC2=O)c3ccc(o3)COc4ccc(cc4)Br)C(=O)OC
ZINC08441036	5.09	1.18	-13.22	0	6	0	472.335	6	COC(=O)C1[C@H]([C@@H]2C(=NC1=C)CCCC2=O)c3ccc(o3)COc4ccc(cc4)Br
ZINC08441036	5.09	-0.16	-12.83	0	6	0	472.335	6	COC(=O)C1[C@H](C2C(=NC1=C)CCCC2=O)c3ccc(o3)COc4ccc(cc4)Br
ZINC00850102	3.96	1.09	-28.63	3	8	0	487.556	6	Cc1cccc(c1)NC(=O)COc2ccc(cc2OC)[C@H]3C(=C(OC4=C3C(=O)CC(C4)(C)C)N)C#N
ZINC08441035	5.15	-1.79	-7.19	0	3	0	567.135	3	CC1([C@]2(CC[C@@]1([C@@H]2Br)C(=O)N3CCN(CC3)c4ccc(cc4)F)C(Br)Br)C
ZINC00850103	3.96	1.08	-28.7	3	8	0	487.556	6	Cc1cccc(c1)NC(=O)COc2ccc(cc2OC)[C@@H]3C(=C(OC4=C3C(=O)CC(C4)(C)C)N)C#N
ZINC08441034	5.35	14.56	-17.57	2	6	0	554.731	6	C[C@]12CC[C@](C1(C)C)(CC2=O)C(=O)Nc3ccc(cc3)Cc4ccc(cc4)NC(=O)[C@@]56CC[C@@](C5(C)C)(C(=O)C6)C
ZINC00850099	5.25	-2.92	-12.11	1	5	0	414.701	5	c1ccc(c(c1)c2nnc(o2)SCC(=O)Nc3cc(cc(c3)Cl)Cl)Cl
ZINC08441033	5.35	14.54	-16.69	2	6	0	554.731	6	C[C@]12CC[C@](C1(C)C)(CC2=O)C(=O)Nc3ccc(cc3)Cc4ccc(cc4)NC(=O)[C@]56CC[C@](C5(C)C)(C(=O)C6)C
ZINC08441032	4.91	-0.23	-13.68	0	6	0	472.335	6	CC1=NC2=C([C@H](C1C(=O)OC)c3ccc(o3)COc4ccc(cc4)Br)C(=O)CCC2
ZINC08441032	4.91	-0.2	-15.24	0	6	0	472.335	6	CC1=C([C@@H]([C@H]2C(=N1)CCCC2=O)c3ccc(o3)COc4ccc(cc4)Br)C(=O)OC
ZINC08441032	5.09	-0.11	-13.27	0	6	0	472.335	6	COC(=O)C1[C@@H](C2C(=NC1=C)CCCC2=O)c3ccc(o3)COc4ccc(cc4)Br
ZINC08441032	5.09	1.06	-14.41	0	6	0	472.335	6	COC(=O)C1[C@@H]([C@H]2C(=NC1=C)CCCC2=O)c3ccc(o3)COc4ccc(cc4)Br
ZINC08441031	6.72	4.33	-12.87	0	4	0	450.563	7	Cc1cccc(c1OC(=O)CSc2c(c(cc(n2)c3ccccc3)c4ccccc4)C#N)C
ZINC08441030	7.7	18.09	-14.03	0	4	0	497.623	7	c1ccc(cc1)c2cc(nc(c2C#N)SCC(=O)N(c3ccccc3)c4ccccc4)c5ccccc5
ZINC02063567	5.74	15.08	-14.38	1	4	0	443.93	7	CC(=O)Oc1ccc2ccccc2c1[C@H](c3ccc(cc3)Cl)NC(=O)Cc4ccccc4
ZINC01889816	5.11	11.19	-13.11	1	5	0	480.193	5	c1cc(ccc1C2=Nc3nc(nn3[C@@H](C2)c4ccc(cc4Cl)Cl)CCCO)Br
ZINC01889816	5.11	11.66	-32.79	2	5	1	481.201	5	c1cc(ccc1C2=Nc3[nH+]c(nn3[C@@H](C2)c4ccc(cc4Cl)Cl)CCCO)Br
ZINC02063566	5.74	15.09	-13.96	1	4	0	443.93	7	CC(=O)Oc1ccc2ccccc2c1[C@@H](c3ccc(cc3)Cl)NC(=O)Cc4ccccc4
ZINC08441029	5.94	0.49	-12.79	0	6	0	514.416	7	CCOC(=O)C1[C@@H](C2=C(CC(CC2=O)(C)C)N=C1C)c3ccc(o3)COc4ccc(cc4)Br
ZINC08441029	5.94	0.34	-14.11	0	6	0	514.416	7	CCOC(=O)C1=C(N=C2CC(CC(=O)C2[C@H]1c3ccc(o3)COc4ccc(cc4)Br)(C)C)C
ZINC08441029	6.12	-0.75	-12.05	0	6	0	514.416	7	CCOC(=O)C1[C@@H](C2C(=NC1=C)CC(CC2=O)(C)C)c3ccc(o3)COc4ccc(cc4)Br
ZINC01889820	5.11	11.67	-13.27	1	5	0	480.193	5	c1cc(ccc1C2=Nc3nc(nn3[C@H](C2)c4ccc(cc4Cl)Cl)CCCO)Br
ZINC01889820	5.11	12.14	-33.36	2	5	1	481.201	5	c1cc(ccc1C2=Nc3[nH+]c(nn3[C@H](C2)c4ccc(cc4Cl)Cl)CCCO)Br
ZINC08441028	5.56	0.81	-21.77	1	7	0	512.653	10	CCOC(=O)c1c2c(sc1N(Cc3ccccc3O)C(=O)CO/N=C(\C)/c4cccs4)CCCC2
ZINC08441027	4.26	-0.53	-40.7	2	4	1	450.422	4	CC(=O)Nc1c(c2c(s1)CCCC2)[C@H](c3cccc(c3)Br)[NH+]4CCOCC4
ZINC08441026	4.26	-0.51	-40.87	2	4	1	450.422	4	CC(=O)Nc1c(c2c(s1)CCCC2)[C@@H](c3cccc(c3)Br)[NH+]4CCOCC4
ZINC08441025	5.94	0.65	-9.66	0	6	0	514.416	7	CCOC(=O)C1[C@H](C2=C(CC(CC2=O)(C)C)N=C1C)c3ccc(o3)COc4ccc(cc4)Br
ZINC08441025	5.94	0.85	-13.45	0	6	0	514.416	7	CCOC(=O)C1=C(N=C2CC(CC(=O)C2[C@@H]1c3ccc(o3)COc4ccc(cc4)Br)(C)C)C
ZINC08441025	6.12	-0.31	-11.29	0	6	0	514.416	7	CCOC(=O)C1[C@H](C2C(=NC1=C)CC(CC2=O)(C)C)c3ccc(o3)COc4ccc(cc4)Br
ZINC08441024	5.52	0.08	-8.54	1	4	0	421.257	7	CCCCCCC1=NN([C@](C1)(C(F)(F)F)O)C(=O)c2ccc(cc2)Br
ZINC08441023	4.37	2.41	-44.27	2	4	1	439.523	5	CC(=O)Nc1c(c2c(s1)CCCC2)[C@H](c3ccc(cc3)C(F)(F)F)[NH+]4CCOCC4

Supplementary Table S2:
Detailed table of XP Docking using Schrodinger's Glide module

ZINC_ID	xLogP	ApolarDesolvation	PolarDesolvation	H Donors	H Acceptors	Charge	MolecularWeight	RotatableBonds	SMILES
ZINC08441022	5.52	0.09	-8.44	1	4	0	421.257	7	CCCCCCC1=NN([C@@](C1)(C(F)(F)F)O)C(=O)c2ccc(cc2)Br
ZINC08441021	4.08	1.25	-41.64	2	5	1	437.532	6	CC(=O)Nc1c(c2c(s1)CCCC2)[C@H](c3ccc(cc3)OC(F)F)[NH+]4CCOCC4
ZINC02063547	8.93	1.29	-11.72	1	4	0	486.681	7	CCOc1ccc2c(c1)sc(n2)c3ccc(cc3)NC(=O)c4cc(cc(c4)C(C)(C)C)C(C)(C)C
ZINC08441020	6.12	0.59	-9.93	0	6	0	481.976	8	CCOC(=O)C1[C@@H](C2=C(CCCC2=O)N=C1C)c3ccc(c(c3)COc4ccc(cc4)Cl)OC
ZINC08441020	6.3	-0.6	-10.62	0	6	0	481.976	8	CCOC(=O)C1[C@@H](C2C(=NC1=C)CCCC2=O)c3ccc(c(c3)COc4ccc(cc4)Cl)OC
ZINC08441020	6.12	0.48	-13.9	0	6	0	481.976	8	CCOC(=O)C1=C(N=C2CCCC(=O)C2[C@H]1c3ccc(c(c3)COc4ccc(cc4)Cl)OC)C
ZINC08441019	3.8	-2.81	-23.29	1	7	0	421.43	4	CC(=O)Nc1ccc(cc1)OS(=O)(=O)c2cccc3c2C(=O)c4ccccc4C3=O
ZINC08441018	4.08	1.32	-41.47	2	5	1	437.532	6	CC(=O)Nc1c(c2c(s1)CCCC2)[C@@H](c3ccc(cc3)OC(F)F)[NH+]4CCOCC4
ZINC06446915	7.96	14.75	-12.56	2	4	0	438.666	13	c1ccc2c(c1)[nH]c(n2)SCCCCCCCCCSc3[nH]c4ccccc4n3
ZINC02355111	7.63	3.88	-11.77	0	6	0	511.647	7	CC1=C([C@H](n2c(=O)/c(=C/c3cn(c4c3cccc4)CC=C)/sc2=N1)c5ccc(cc5)C(C)C)C(=O)OC
ZINC08441017	4.61	0.78	-36.95	2	5	1	476.416	5	Cc1c(sc(c1[C@H](c2cccc(c2)Br)[NH+]3CCOCC3)NC(=O)c4ccco4)C
ZINC00970906	7.14	1.86	-11.58	1	6	0	485.609	5	Cc1c(c2ccccc2[nH]1)/C=c\3/c(=O)n4c(=NC(=C([C@H]4c5ccc(cc5)C(C)C)C(=O)OC)C)s3
ZINC00970908	7.14	1.94	-11.73	1	6	0	485.609	5	Cc1c(c2ccccc2[nH]1)/C=c\3/c(=O)n4c(=NC(=C([C@@H]4c5ccc(cc5)C(C)C)C(=O)OC)C)s3
ZINC08441016	4.31	11.34	-54.32	0	6	-1	450.584	5	CCOc1cccc(c1)N2C(=O)/C(=C/c3cc(n(c3C)C4CCCCC4)C)/C(=NC2=S)[O-]
ZINC00859938	3.79	12.13	-54.12	0	7	-1	487.605	6	CCOc1cccc(c1)N2C(=O)/C(=C/c3cc(n(c3C)c4ccc(cc4)N(C)C)C)/C(=NC2=S)[O-]
ZINC08441015	6.12	0.77	-10.15	0	6	0	481.976	8	CCOC(=O)C1[C@H](C2=C(CCCC2=O)N=C1C)c3ccc(c(c3)COc4ccc(cc4)Cl)OC
ZINC08441015	6.12	0.48	-13.69	0	6	0	481.976	8	CCOC(=O)C1=C(N=C2CCCC(=O)C2[C@@H]1c3ccc(c(c3)COc4ccc(cc4)Cl)OC)C
ZINC08441015	6.3	-0.43	-10.96	0	6	0	481.976	8	CCOC(=O)C1[C@H](C2C(=NC1=C)CCCC2=O)c3ccc(c(c3)COc4ccc(cc4)Cl)OC
ZINC08441014	4.61	0.81	-37.08	2	5	1	476.416	5	Cc1c(sc(c1[C@@H](c2cccc(c2)Br)[NH+]3CCOCC3)NC(=O)c4ccco4)C
ZINC08441013	4.72	12.92	-53.37	0	6	-1	472.59	5	CCOc1cccc(c1)N2C(=O)/C(=C/c3cc(n(c3C)c4ccc(c(c4)C)C)C)/C(=NC2=S)[O-]
ZINC00852798	3.07	0.54	-9.52	1	6	0	464.319	3	Cc1cc(c(n1c2ccccc2)C)/C=C\3/C(=O)NC(=O)N(C3=O)c4ccc(cc4)Br
ZINC08441012	4.48	1.39	-36.95	2	5	1	431.965	5	Cc1c(sc(c1[C@H](c2cccc(c2)Cl)[NH+]3CCOCC3)NC(=O)c4ccco4)C
ZINC00970877	3.89	-3.42	-10.21	2	5	0	496.18	2	Cc1cc(ccc1N2C(=O)/C(=C/c3ccc(c(c3)Br)O)/C(=O)NC2=S)Br
ZINC08441011	3.41	-5.97	-10.72	3	6	0	498.152	2	c1cc(cc(c1)Br)N2C(=O)/C(=C\c3cc(c(cc3O)O)Br)/C(=O)NC2=S
ZINC08441010	4.48	1.42	-37.05	2	5	1	431.965	5	Cc1c(sc(c1[C@@H](c2cccc(c2)Cl)[NH+]3CCOCC3)NC(=O)c4ccco4)C
ZINC08441009	7.8	2.84	-17.88	0	7	0	632.811	5	Cc1cc(c(n1c2c(c3c(s2)CCCC3)C#N)C)/C=c\4/c(=O)n5c(=NC(=C([C@H]5c6c7ccccc7ccc6OC)C(=O)C)C)s4
ZINC08441008	7.8	2.71	-17.81	0	7	0	632.811	5	Cc1cc(c(n1c2c(c3c(s2)CCCC3)C#N)C)/C=c\4/c(=O)n5c(=NC(=C([C@@H]5c6c7ccccc7ccc6OC)C(=O)C)C)s4
ZINC00970872	6.48	0.88	-17.36	1	6	0	507.615	4	Cc1c(c2ccccc2[nH]1)/C=c\3/c(=O)n4c(=NC(=C([C@H]4c5c6ccccc6ccc5OC)C(=O)C)C)s3
ZINC00970874	6.48	0.92	-17.28	1	6	0	507.615	4	Cc1c(c2ccccc2[nH]1)/C=c\3/c(=O)n4c(=NC(=C([C@@H]4c5c6ccccc6ccc5OC)C(=O)C)C)s3
ZINC08441007	6.4	1.33	-9.68	0	6	0	496.003	7	CC1=NC2=C([C@@H](C1C(=O)OC)c3ccc(c(c3)COc4ccc(cc4)Cl)OC)C(=O)CC(C2)(C)C
ZINC08441007	6.58	2.77	-17.73	0	6	0	496.003	7	CC1(CC2=NC(=C)C([C@H]([C@H]2C(=O)C1)c3ccc(c(c3)COc4ccc(cc4)Cl)OC)C(=O)OC)C
ZINC08441007	6.58	1.47	-10.43	0	6	0	496.003	7	CC1(CC2=NC(=C)C([C@H](C2C(=O)C1)c3ccc(c(c3)COc4ccc(cc4)Cl)OC)C(=O)OC)C
ZINC08441007	6.4	1.35	-12.53	0	6	0	496.003	7	CC1=C([C@H]([C@H]2C(=N1)CC(CC2=O)(C)C)c3ccc(c(c3)COc4ccc(cc4)Cl)OC)C(=O)OC
ZINC08441005	5.26	-1.23	-6.8	1	4	0	504.62	2	Cc1cc(ccc1N2C(=O)/C(=C\c3cc(cc(c3Cl)Cl)Cl)/C(=O)NC2=S)Br
ZINC08441004	6.5	-1.14	-11.49	1	6	0	697.776	6	Cc1cc(ccc1N2C(=O)/C(=C\c3cc(c(c(c3)I)OCc4ccc(cc4)Cl)OC)/C(=O)NC2=S)Br
ZINC08441002	4.5	-4.84	-8.95	2	5	0	608.049	2	c1cc(cc(c1)Br)N2C(=O)/C(=C\c3cc(cc(c3O)I)Br)/C(=O)NC2=S
ZINC08441000	4.5	1.36	-38.13	2	5	1	431.965	5	Cc1c(sc(c1[C@H](c2ccc(cc2)Cl)[NH+]3CCOCC3)NC(=O)c4ccco4)C
ZINC00852837	4.69	7.38	-90.53	0	5	-2	559.033	2	c1cc(cc(c1)Br)N2C(=O)/C(=C/c3cc(c(c(c3)Br)[O-])Br)/C(=NC2=S)[O-]
ZINC00852837	4.69	6.6	-40.57	1	5	-1	560.041	2	c1cc(cc(c1)Br)N2C(=O)/C(=C/c3cc(c(c(c3)Br)O)Br)/C(=NC2=S)[O-]
ZINC02063498	6.13	0.21	-13.26	0	6	0	436.198	5	c1cc(c(cc1C(F)(F)F)[N+](=O)[O-])Sc2nnc(o2)c3ccc(cc3Cl)Cl
ZINC08440999	4.5	1.35	-38.26	2	5	1	431.965	5	Cc1c(sc(c1[C@@H](c2ccc(cc2)Cl)[NH+]3CCOCC3)NC(=O)c4ccco4)C
ZINC08440998	6.4	1.31	-9.72	0	6	0	496.003	7	CC1=NC2=C([C@H](C1C(=O)OC)c3ccc(c(c3)COc4ccc(cc4)Cl)OC)C(=O)CC(C2)(C)C
ZINC08440998	6.58	1.36	-10.22	0	6	0	496.003	7	CC1(CC2=NC(=C)C([C@@H](C2C(=O)C1)c3ccc(c(c3)COc4ccc(cc4)Cl)OC)C(=O)OC)C
ZINC08440998	6.4	1.26	-12.66	0	6	0	496.003	7	CC1=C([C@@H]([C@H]2C(=N1)CC(CC2=O)(C)C)c3ccc(c(c3)COc4ccc(cc4)Cl)OC)C(=O)OC
ZINC08440998	6.58	2.53	-12.23	0	6	0	496.003	7	CC1(CC2=NC(=C)C([C@@H]([C@@H]2C(=O)C1)c3ccc(c(c3)COc4ccc(cc4)Cl)OC)C(=O)OC)C
ZINC02063496	5.79	1.26	-26.05	2	5	1	482.398	7	CCOC(=O)C[NH+]1[C@H](c2cc(ccc2N[C@@H]1c3ccccc3OC)Br)c4ccccc4
ZINC08440997	5.79	0.9	-30.75	2	5	1	482.398	7	CCOC(=O)C[NH+]1[C@@H](c2cc(ccc2N[C@@H]1c3ccccc3OC)Br)c4ccccc4
ZINC08440996	5.86	-0.54	-12.65	1	6	0	585.296	7	COc1ccccc1NC(=O)CCC(=O)N2[C@H](CC(=N2)c3ccc(cc3)Br)c4ccc(cc4)Br
ZINC08440995	5.86	-0.58	-12.49	1	6	0	585.296	7	COc1ccccc1NC(=O)CCC(=O)N2[C@@H](CC(=N2)c3ccc(cc3)Br)c4ccc(cc4)Br
ZINC08440994	4.44	2.52	-38.76	2	6	1	463.526	7	Cc1c(sc(c1[C@H](c2ccc(cc2)OC(F)F)[NH+]3CCOCC3)NC(=O)c4ccco4)C
ZINC08440992	6.44	0.85	-14.86	0	6	0	574.519	7	CCOC(=O)C1[C@@H](C2=C(CC(CC2=O)(C)C)N=C1C)c3cn(nc3c4ccccc4)Cc5ccc(cc5)Br
ZINC08440992	6.62	-0.01	-14.34	0	6	0	574.519	7	CCOC(=O)C1[C@@H](C2C(=NC1=C)CC(CC2=O)(C)C)c3cn(nc3c4ccccc4)Cc5ccc(cc5)Br
ZINC08440992	6.44	1.07	-16.79	0	6	0	574.519	7	CCOC(=O)C1=C(N=C2CC(CC(=O)C2[C@H]1c3cn(nc3c4ccccc4)Cc5ccc(cc5)Br)(C)C)C
ZINC08440990	3.43	-0.39	-98.7	3	7	2	475.618	6	Cc1c(sc(c1[C@H](c2cccnc2)[NH+]3CCN(CC3)c4cccc[nH+]4)NC(=O)c5ccco5)C
ZINC08440987	3.43	-0.45	-96.78	3	7	2	475.618	6	Cc1c(sc(c1[C@@H](c2cccnc2)[NH+]3CCN(CC3)c4cccc[nH+]4)NC(=O)c5ccco5)C
ZINC08440986	6.44	0.94	-11.19	0	6	0	574.519	7	CCOC(=O)C1[C@H](C2=C(CC(CC2=O)(C)C)N=C1C)c3cn(nc3c4ccccc4)Cc5ccc(cc5)Br
ZINC08440986	6.62	0.05	-13.13	0	6	0	574.519	7	CCOC(=O)C1[C@H](C2C(=NC1=C)CC(CC2=O)(C)C)c3cn(nc3c4ccccc4)Cc5ccc(cc5)Br
ZINC04182261	6.54	-3.3	-14.37	0	4	0	534.273	4	Cc1ccc(cc1)S(=O)(=O)N2[C@H](CC(=N2)c3ccc(cc3)Br)c4ccc(cc4)Br
ZINC04182260	6.54	-3.69	-11.84	0	4	0	534.273	4	Cc1ccc(cc1)S(=O)(=O)N2[C@@H](CC(=N2)c3ccc(cc3)Br)c4ccc(cc4)Br
ZINC08440985	6.84	0.49	-11.41	0	3	0	518.636	3	c1ccc(c(c1)C(=O)N2[C@H](CC(=N2)c3ccc(cc3)Br)c4ccc(cc4)Br)Cl
ZINC08440984	6.84	0.49	-11.43	0	3	0	518.636	3	c1ccc(c(c1)C(=O)N2[C@@H](CC(=N2)c3ccc(cc3)Br)c4ccc(cc4)Br)Cl

Supplementary Table S2:
Detailed table of XP Docking using Schrodinger's Glide module

ZINC_ID	xLogP	ApolarDesolvation	PolarDesolvation	H Donors	H Acceptors	Charge	MolecularWeight	RotatableBonds	SMILES
ZINC02063489	8.37	0.13	-8.01	1	4	0	514.81	5	c1ccc(cc1)c2c3cc(ccc3nc(n2)Nc4ccc(cc4C(=O)c5ccccc5)Cl)Br
ZINC02063488	7.71	0.39	-9.81	1	4	0	480.365	5	c1ccc(cc1)c2c3cc(ccc3nc(n2)Nc4ccccc4C(=O)c5ccccc5)Br
ZINC01927667	7.25	1.79	-15.84	1	5	0	586.06	7	Cc1c(c2c(s1)nc(n(c2=O)c3ccccc3)SCC(=O)Nc4cc(ccc4Cl)C(F)(F)F)c5ccccc5
ZINC08440983	6.77	1.57	-9.55	0	6	0	510.03	8	CCOC(=O)C1[C@@H](C2=C(CC(CC2=O)(C)C)N=C1C)c3ccc(c(c3)COc4ccc(cc4)Cl)OC
ZINC08440983	6.96	0.63	-10.58	0	6	0	510.03	8	CCOC(=O)C1[C@@H](C2C(=NC1=C)CC(CC2=O)(C)C)c3ccc(c(c3)COc4ccc(cc4)Cl)OC
ZINC08440983	6.77	1.55	-12.58	0	6	0	510.03	8	CCOC(=O)C1=C(N=C2CC(CC(=O)C2[C@H]1c3ccc(c(c3)COc4ccc(cc4)Cl)OC)(C)C)C
ZINC02063487	7.78	1.68	-11.56	1	4	0	429.523	5	Cc1ccc2c(c1)c(nc(n2)Nc3ccc(cc3C(=O)c4ccccc4)C)c5ccccc5
ZINC08440982	7.81	4.65	-16.37	0	5	0	629.729	8	Cc1cc(c(n1c2cccc(c2)C(F)(F)F)C)C(=O)CSc3nc4c(c(s4)C)c5ccccc5)c(=O)n3c6ccccc6
ZINC08440981	5.77	-0.09	-13.18	1	6	0	540.845	7	COc1ccc(cc1)[C@H]2CC(=NN2C(=O)CCC(=O)Nc3ccc(cc3)Cl)c4ccc(cc4)Br
ZINC08440980	5.77	-0.1	-13.15	1	6	0	540.845	7	COc1ccc(cc1)[C@@H]2CC(=NN2C(=O)CCC(=O)Nc3ccc(cc3)Cl)c4ccc(cc4)Br
ZINC08440979	6.77	1.59	-9.61	0	6	0	510.03	8	CCOC(=O)C1[C@H](C2=C(CC(CC2=O)(C)C)N=C1C)c3ccc(c(c3)COc4ccc(cc4)Cl)OC
ZINC08440979	6.96	0.32	-10.42	0	6	0	510.03	8	CCOC(=O)C1[C@H](C2C(=NC1=C)CC(CC2=O)(C)C)c3ccc(c(c3)COc4ccc(cc4)Cl)OC
ZINC08440979	6.77	1.22	-12.63	0	6	0	510.03	8	CCOC(=O)C1=C(N=C2CC(CC(=O)C2[C@@H]1c3ccc(c(c3)COc4ccc(cc4)Cl)OC)(C)C)C
ZINC01927657	6.62	1.74	-16.26	1	5	0	551.615	7	Cc1c(c2c(s1)nc(n(c2=O)c3ccccc3)SCC(=O)Nc4cccc(c4)C(F)(F)F)c5ccccc5
ZINC06284383	6.37	15.65	-14.23	0	6	0	471.601	10	CCc1cccc(c1N(COCC)C(=O)Cn2c3ccccc3nc2COc4ccc(cc4)C)C
ZINC08440978	6.97	-0.06	-11.02	0	3	0	563.087	3	c1ccc(c(c1)C(=O)N2[C@H](CC(=N2)c3ccc(cc3)Br)c4ccc(cc4)Br)Br
ZINC08440977	6.97	-0.06	-10.94	0	3	0	563.087	3	c1ccc(c(c1)C(=O)N2[C@@H](CC(=N2)c3ccc(cc3)Br)c4ccc(cc4)Br)Br
ZINC08440976	3.79	-1.48	-16.35	2	7	0	531.764	7	c1cc(ccc1[C@H]2C[C@H](n3c(c(c(n3)C(=O)NCCCn4ccnc4)Cl)N2)C(F)(F)F)Br
ZINC08440976	3.79	-1.2	-42.66	3	7	1	532.772	7	c1cc(ccc1[C@H]2C[C@H](n3c(c(c(n3)C(=O)NCCCn4cc[nH+]c4)Cl)N2)C(F)(F)F)Br
ZINC08440975	3.5	-1.19	-17.75	3	10	0	558.616	8	CCOC(=O)c1c2c(sc1NC(=O)c3ccc(cc3)N/C=C\4/C(=O)NC(=O)N(C4=O)c5ccccc5)CCCC2
ZINC00970849	4.95	11.22	-38.51	0	6	-1	460.728	3	Cc1c(c(n(n1)c2c(cc(cc2Cl)Cl)Cl)[O-])/C=C/3\C(=NN(C3=O)c4ccccc4)C
ZINC08440974	3.55	4.25	-55.73	1	11	-1	619.421	8	CC\1=NN(C(=O)/C1=C\Nc2ccc(cc2)S(=O)(=O)[N-]c3cc(nc(n3)OC)OC)c4ccc(cc4)I
ZINC08440974	3.73	5.38	-22.79	2	11	0	620.429	7	CC\1=NN(C(=O)/C1=C\Nc2ccc(cc2)S(=O)(=O)/N=c\3/cc(nc([nH]3)OC)OC)c4ccc(cc4)I
ZINC08440973	3.79	-1.82	-18.26	2	7	0	531.764	7	c1cc(ccc1[C@@H]2C[C@H](n3c(c(c(n3)C(=O)NCCCn4ccnc4)Cl)N2)C(F)(F)F)Br
ZINC08440973	3.79	-1.54	-43.12	3	7	1	532.772	7	c1cc(ccc1[C@@H]2C[C@H](n3c(c(c(n3)C(=O)NCCCn4cc[nH+]c4)Cl)N2)C(F)(F)F)Br
ZINC08440972	5.67	1.92	-73.55	1	5	1	444.99	4	CC(C)c1cc(n2c3ccccc3nc2c1C#N)N4CC[NH+](CC4)Cc5ccc(cc5)Cl
ZINC08440969	5.4	0.93	-6.51	0	5	0	455.292	7	CCOC(=O)C1[C@H](C(=C(N=C1C)C)C(=O)OCC)c2cccc(c2)I
ZINC08440969	5.4	0.78	-7.61	0	5	0	455.292	7	CCOC(=O)C1[C@@H](C(=C(N=C1C)C)C(=O)OCC)c2cccc(c2)I
ZINC08440969	5.58	-0.18	-6.75	0	5	0	455.292	7	CCOC(=O)C1[C@@H](C(C(=C)N=C1C)C(=O)OCC)c2cccc(c2)I
ZINC08440968	3.79	-1.87	-17.48	2	7	0	531.764	7	c1cc(ccc1[C@H]2C[C@@H](n3c(c(c(n3)C(=O)NCCCn4ccnc4)Cl)N2)C(F)(F)F)Br
ZINC08440968	3.79	-1.59	-42.33	3	7	1	532.772	7	c1cc(ccc1[C@H]2C[C@@H](n3c(c(c(n3)C(=O)NCCCn4cc[nH+]c4)Cl)N2)C(F)(F)F)Br
ZINC08440967	4.84	-1.47	-51.22	1	7	1	484.411	7	c1cc(ccc1Cn2c3c(cn2)c(ncn3)N4CC[NH+](CC4)CCOc5ccc(cc5)Cl)Cl
ZINC08440966	4.54	2.63	-12.02	2	13	0	394.307	6	c1ccc(c(c1)Nc2c(nc3c(n2)non3)Nc4ccccc4[N+](=O)[O-])[N+](=O)[O-]
ZINC08440965	4.55	-1.31	-52.07	1	6	1	454.385	5	c1cc(ccc1Cn2c3c(cn2)c(ncn3)N4CC[NH+](CC4)Cc5ccc(cc5)Cl)Cl
ZINC08440964	3.79	-1.42	-16.61	2	7	0	531.764	7	c1cc(ccc1[C@@H]2C[C@@H](n3c(c(c(n3)C(=O)NCCCn4ccnc4)Cl)N2)C(F)(F)F)Br
ZINC08440964	3.79	-1.14	-43.1	3	7	1	532.772	7	c1cc(ccc1[C@@H]2C[C@@H](n3c(c(c(n3)C(=O)NCCCn4cc[nH+]c4)Cl)N2)C(F)(F)F)Br
ZINC08440963	3.77	3.38	-8.1	0	19	0	484.301	8	c1cc(ccc1N(c2c(nc3c(n2)non3)N(c4ccc(cc4)[N+](=O)[O-])[N+](=O)[O-])[N+](=O)[O-])[N+](=O)[O-]
ZINC08440962	5.42	0.48	-43.85	1	6	1	475.62	6	Cc1ccc(cc1)Cn2c3c(cn2)c(ncn3)N4CC[NH+](CC4)C(c5ccccc5)c6ccccc6
ZINC00648070	4.66	-2.39	-9.68	3	6	0	465.351	4	Cc1cc(c(cc1COc2ccc(cc2)Br)[C@H]3c4c(n[nH]c4OC(=C3C#N)N)C)C
ZINC08440958	7.18	2.13	-16.59	1	4	0	362.521	10	CCCCCCCCNc1cc(c(c2n1c3ccccc3n2)C#N)CCC
ZINC00648073	4.66	-2.4	-9.58	3	6	0	465.351	4	Cc1cc(c(cc1COc2ccc(cc2)Br)[C@@H]3c4c(n[nH]c4OC(=C3C#N)N)C)C
ZINC06300227	3.89	7.26	-12.37	3	7	0	430.508	5	Cc1cc(c(c(c1)C)COc2ccc(cc2OC)[C@H]3c4c(n[nH]c4OC(=C3C#N)N)C)C
ZINC00702815	4.45	1.2	-14.92	0	7	0	445.5	8	c1ccc(cc1)C(=O)CSc2nnc(n2c3ccccc3)COc4ccc5c(c4)OCO5
ZINC06300225	3.89	7.19	-12.32	3	7	0	430.508	5	Cc1cc(c(c(c1)C)COc2ccc(cc2OC)[C@@H]3c4c(n[nH]c4OC(=C3C#N)N)C)C
ZINC00702814	5.12	1.03	-15.08	0	7	0	479.945	8	c1ccc(cc1)n2c(nnc2SCC(=O)c3ccc(cc3)Cl)COc4ccc5c(c4)OCO5
ZINC00846261	4.59	8.94	-17.91	4	8	0	523.589	9	Cc1ccc(cc1)NC(=O)[C@H](Cc2ccc(cc2)O)NC(=O)c3ccccc3NC(=O)c4ccc(cc4)OC
ZINC03190354	4.59	-2.76	-17.9	4	8	0	523.589	9	Cc1ccc(cc1)NC(=O)[C@@H](Cc2ccc(cc2)O)NC(=O)c3ccccc3NC(=O)c4ccc(cc4)OC
ZINC04391415	5.88	0.51	-10.84	0	6	0	522.432	6	CC1=NC2=C([C@@H](C1C(=O)OC)c3cn(nc3c4ccccc4)Cc5ccc(cc5Cl)Cl)C(=O)CCC2
ZINC04391415	6.07	0.64	-11.63	0	6	0	522.432	6	COC(=O)C1[C@H](C2C(=NC1=C)CCCC2=O)c3cn(nc3c4ccccc4)Cc5ccc(cc5Cl)Cl
ZINC04391415	6.07	1.92	-12.65	0	6	0	522.432	6	COC(=O)C1[C@H]([C@@H]2C(=NC1=C)CCCC2=O)c3cn(nc3c4ccccc4)Cc5ccc(cc5Cl)Cl
ZINC08440954	3.28	11	-17.84	0	7	0	443.503	5	COc1ccccc1N2CCN(CC2)C(=O)CCN3C(=O)c4cccc5c4c(ccc5)C3=O
ZINC08440953	5.4	-3.48	-16.05	4	8	0	531.653	14	CCCCCCCNC(=O)[C@H](Cc1ccc(cc1)O)NC(=O)c2ccccc2NC(=O)c3ccc(cc3)OC
ZINC08440951	5.4	-3.42	-16.46	4	8	0	531.653	14	CCCCCCCNC(=O)[C@@H](Cc1ccc(cc1)O)NC(=O)c2ccccc2NC(=O)c3ccc(cc3)OC
ZINC08440950	3.87	-0.98	-11.56	3	7	0	439.556	10	CC[C@H](C)[C@H](C(=O)NCC(C)C)NC(=O)c1ccccc1NC(=O)c2ccc(cc2)OC
ZINC08440949	3.87	-0.79	-11.6	3	7	0	439.556	10	CC[C@@H](C)[C@H](C(=O)NCC(C)C)NC(=O)c1ccccc1NC(=O)c2ccc(cc2)OC
ZINC08440948	3.87	-0.6	-12.36	3	7	0	439.556	10	CC[C@H](C)[C@@H](C(=O)NCC(C)C)NC(=O)c1ccccc1NC(=O)c2ccc(cc2)OC
ZINC02129169	4.67	15.15	-12.73	0	6	0	447.589	9	C=CCn1c(=O)c2ccccc2nc1SCc3nnc(n3c4ccccc4)SCC=C
ZINC08440946	3.87	-0.96	-11.55	3	7	0	439.556	10	CC[C@@H](C)[C@@H](C(=O)NCC(C)C)NC(=O)c1ccccc1NC(=O)c2ccc(cc2)OC
ZINC08440945	3.46	-3.07	-18.46	4	10	0	532.597	11	Cc1ccc(cc1)OCC(=O)NNC(=O)[C@H](C(C)C)NC(=O)c2ccccc2NC(=O)c3ccc(cc3)OC
ZINC08440944	5.88	0.56	-17.95	0	6	0	522.432	6	CC1=NC2=C([C@H](C1C(=O)OC)c3cn(nc3c4ccccc4)Cc5ccc(cc5Cl)Cl)C(=O)CCC2
ZINC08440944	6.07	1.93	-20.38	0	6	0	522.432	6	COC(=O)C1[C@@H]([C@H]2C(=NC1=C)CCCC2=O)c3cn(nc3c4ccccc4)Cc5ccc(cc5Cl)Cl

Supplementary Table S2:
Detailed table of XP Docking using Schrodinger's Glide module

ZINC_ID	xLogP	ApolarDesolvation	PolarDesolvation	H Donors	H Acceptors	Charge	MolecularWeight	RotatableBonds	SMILES
ZINC08440944	5.88	0.55	-18.14	0	6	0	522.432	6	CC1=C([C@@H]([C@@H]2C(=N1)CCCC2=O)c3cn(nc3c4ccccc4)Cc5ccc(cc5Cl)Cl)C(=O)OC
ZINC08440944	6.07	0.67	-16.2	0	6	0	522.432	6	COC(=O)C1[C@@H](C2C(=NC1=C)CCCC2=O)c3cn(nc3c4ccccc4)Cc5ccc(cc5Cl)Cl
ZINC06142963	3.45	-9.69	-22.95	2	12	0	602.72	9	Cn1c(=O)c2c3c(sc2nc1SCC(=O)Nc4ccc(cc4)S(=O)(=O)Nc5cc(nc(n5)OC)OC)CCCC3
ZINC08440943	3.46	-2.88	-18.58	4	10	0	532.597	11	Cc1ccc(cc1)OCC(=O)NNC(=O)[C@@H](C(C)C)NC(=O)c2ccccc2NC(=O)c3ccc(cc3)OC
ZINC00702801	4.59	0.46	-20.15	1	7	0	471.604	10	CCOC(=O)c1ccc(cc1)NC(=O)CCSc2nc3c(c(s3)C)c(=O)n2CC=C
ZINC08440940	4.25	0.74	-13.49	3	9	0	503.555	10	CC(C)[C@H](C(=O)Nc1ccccc1C(=O)OC)NC(=O)c2ccccc2NC(=O)c3ccc(cc3)OC
ZINC08440939	4.25	0.78	-13.74	3	9	0	503.555	10	CC(C)[C@@H](C(=O)Nc1ccccc1C(=O)OC)NC(=O)c2ccccc2NC(=O)c3ccc(cc3)OC
ZINC02129165	4.4	11.7	-21.29	1	7	0	484.434	11	C=CCn1c(nnc1SCC(=O)Nc2nccs2)CCCOc3ccc(cc3Cl)Cl
ZINC08440938	6.26	0.56	-16.61	0	6	0	536.459	7	CCOC(=O)C1[C@@H](C2=C(CCCC2=O)N=C1C)c3cn(nc3c4ccccc4)Cc5ccc(cc5Cl)Cl
ZINC08440938	6.44	-0.6	-15.29	0	6	0	536.459	7	CCOC(=O)C1[C@@H](C2C(=NC1=C)CCCC2=O)c3cn(nc3c4ccccc4)Cc5ccc(cc5Cl)Cl
ZINC08440938	6.26	0.44	-17.85	0	6	0	536.459	7	CCOC(=O)C1=C(N=C2CCCC(=O)C2[C@H]1c3cn(nc3c4ccccc4)Cc5ccc(cc5Cl)Cl)C
ZINC00846215	5.13	-0.51	-14.04	3	7	0	495.579	8	CC(C)[C@H](C(=O)Nc1ccc2ccccc2c1)NC(=O)c3ccccc3NC(=O)c4ccc(cc4)OC
ZINC00846213	4.77	0.03	-13.21	3	7	0	473.573	8	Cc1ccc(c(c1)C)NC(=O)[C@H](C(C)C)NC(=O)c2ccccc2NC(=O)c3ccc(cc3)OC
ZINC00846212	4.77	0.15	-13.08	3	7	0	473.573	8	Cc1ccc(c(c1)C)NC(=O)[C@@H](C(C)C)NC(=O)c2ccccc2NC(=O)c3ccc(cc3)OC
ZINC00846210	4.7	-1.24	-11.76	3	7	0	524.415	8	CC(C)[C@@H](C(=O)Nc1ccccc1Br)NC(=O)c2ccccc2NC(=O)c3ccc(cc3)OC
ZINC08440936	6.26	0.5	-10.54	0	6	0	536.459	7	CCOC(=O)C1[C@H](C2=C(CCCC2=O)N=C1C)c3cn(nc3c4ccccc4)Cc5ccc(cc5Cl)Cl
ZINC08440936	6.26	0.47	-14.16	0	6	0	536.459	7	CCOC(=O)C1=C(N=C2CCCC(=O)C2[C@@H]1c3cn(nc3c4ccccc4)Cc5ccc(cc5Cl)Cl)C
ZINC08440936	6.44	-0.56	-12.26	0	6	0	536.459	7	CCOC(=O)C1[C@H](C2C(=NC1=C)CCCC2=O)c3cn(nc3c4ccccc4)Cc5ccc(cc5Cl)Cl
ZINC00970838	4	-0.92	-14.21	3	8	0	475.545	9	CC(C)[C@H](C(=O)Nc1ccc(cc1)OC)NC(=O)c2ccccc2NC(=O)c3ccc(cc3)OC
ZINC08440935	4.99	-1.22	-55.49	2	7	1	592.51	10	c1ccc(cc1)COc2ccc(cc2)[C@H]3C(=C(C(=O)N3CCC[NH+]4CCOCC4)O)C(=O)c5ccc(cc5)Br
ZINC08440935	4.4	-1.31	-66.97	1	7	1	592.51	10	c1ccc(cc1)COc2ccc(cc2)[C@H]3[C@H](C(=O)C(=O)N3CCC[NH+]4CCOCC4)C(=O)c5ccc(cc5)Br
ZINC00970837	4	-0.51	-14.32	3	8	0	475.545	9	CC(C)[C@@H](C(=O)Nc1ccc(cc1)OC)NC(=O)c2ccccc2NC(=O)c3ccc(cc3)OC
ZINC00970834	3.94	9.31	-15.25	3	7	0	445.519	8	CC(C)[C@H](C(=O)Nc1ccccc1)NC(=O)c2ccccc2NC(=O)c3ccc(cc3)OC
ZINC00970833	3.94	9.1	-15.33	3	7	0	445.519	8	CC(C)[C@@H](C(=O)Nc1ccccc1)NC(=O)c2ccccc2NC(=O)c3ccc(cc3)OC
ZINC08440934	4.99	-1.55	-50.51	2	7	1	592.51	10	c1ccc(cc1)COc2ccc(cc2)[C@@H]3C(=C(C(=O)N3CCC[NH+]4CCOCC4)O)C(=O)c5ccc(cc5)Br
ZINC08440934	4.4	-1.63	-60.18	1	7	1	592.51	10	c1ccc(cc1)COc2ccc(cc2)[C@@H]3[C@@H](C(=O)C(=O)N3CCC[NH+]4CCOCC4)C(=O)c5ccc(cc5)Br
ZINC08440933	4.5	-0.01	-57.76	2	8	1	558.095	12	CC(C)CCOc1ccc(cc1OC)[C@H]2C(=C(C(=O)N2CCC[NH+]3CCOCC3)O)C(=O)c4ccc(cc4)Cl
ZINC08440933	3.92	-0.12	-69.32	1	8	1	558.095	12	CC(C)CCOc1ccc(cc1OC)[C@H]2[C@H](C(=O)C(=O)N2CCC[NH+]3CCOCC3)C(=O)c4ccc(cc4)Cl
ZINC08440932	4.25	0.68	-11.51	2	7	0	453.583	11	CCCN(CCC)C(=O)[C@H](C(C)C)NC(=O)c1ccccc1NC(=O)c2ccc(cc2)OC
ZINC08440931	4.25	0.45	-12.32	2	7	0	453.583	11	CCCN(CCC)C(=O)[C@@H](C(C)C)NC(=O)c1ccccc1NC(=O)c2ccc(cc2)OC
ZINC08440930	4.5	-0.22	-51.4	2	8	1	558.095	12	CC(C)CCOc1ccc(cc1OC)[C@@H]2C(=C(C(=O)N2CCC[NH+]3CCOCC3)O)C(=O)c4ccc(cc4)Cl
ZINC08440930	3.92	-0.3	-60.72	1	8	1	558.095	12	CC(C)CCOc1ccc(cc1OC)[C@@H]2[C@@H](C(=O)C(=O)N2CCC[NH+]3CCOCC3)C(=O)c4ccc(cc4)Cl
ZINC08440929	3.75	12.09	-24.61	2	8	0	494.551	7	Cc1c(c(=O)n([nH]1)c2ccccc2)[C@@H](c3ccc(cc3)OC(=O)C)c4c(nn(c4O)c5ccccc5)C
ZINC08440929	4.2	11.9	-148.73	0	8	-2	492.535	7	Cc1c(c(n(n1)c2ccccc2)[O-])C(c3ccc(cc3)OC(=O)C)c4c(nn(c4[O-])c5ccccc5)C
ZINC06444396	6.68	15.23	-10.37	0	5	0	444.336	3	Cn1c2ccccc2c(c1c3ccccc3)/N=N\c4nc5ccc(cc5n4C)Br
ZINC08440926	4.27	-1.29	-54.14	2	7	1	544.466	10	CCCOc1ccc(cc1)[C@H]2C(=C(C(=O)N2CCC[NH+]3CCOCC3)O)C(=O)c4ccc(cc4)Br
ZINC08440926	3.69	-1.38	-65.4	1	7	1	544.466	10	CCCOc1ccc(cc1)[C@H]2[C@H](C(=O)C(=O)N2CCC[NH+]3CCOCC3)C(=O)c4ccc(cc4)Br
ZINC08440925	4.27	-1.58	-48.68	2	7	1	544.466	10	CCCOc1ccc(cc1)[C@@H]2C(=C(C(=O)N2CCC[NH+]3CCOCC3)O)C(=O)c4ccc(cc4)Br
ZINC08440925	3.69	-1.69	-58.37	1	7	1	544.466	10	CCCOc1ccc(cc1)[C@@H]2[C@@H](C(=O)C(=O)N2CCC[NH+]3CCOCC3)C(=O)c4ccc(cc4)Br
ZINC08904385	3.81	9.46	-47.91	2	7	1	471.599	9	CCCOc1ccc(cc1)[C@H]2/C(=C(/c3cccs3)\O)/C(=O)C(=O)N2CCC[NH+]4CCOCC4
ZINC08838065	3.81	-1.19	-49.44	2	7	1	471.599	9	CCCOc1ccc(cc1)[C@@H]2/C(=C(/c3cccs3)\O)/C(=O)C(=O)N2CCC[NH+]4CCOCC4
ZINC08440921	5.92	13.59	-22.17	1	6	0	475.585	7	Cc1cc(c(c(c1)C)COc2ccc(cc2OC)[C@H]3C4=C(CCCC4=O)NC(=C3C(=O)OC)C)C
ZINC08440921	6.04	0.3	-11.1	0	6	0	475.585	7	Cc1cc(c(c(c1)C)COc2ccc(cc2OC)[C@H]3C4C(=O)CCC=C4N=C(C3C(=O)OC)C)C
ZINC08440920	3.39	-0.59	-52.44	2	8	1	493.58	11	CCOc1ccc(cc1)C(=O)C2=C(C(=O)N([C@H]2c3ccc(cc3)OCC=C)CC[NH+]4CCOCC4)O
ZINC08440920	2.8	-0.74	-61.77	1	8	1	493.58	11	CCOc1ccc(cc1)C(=O)[C@H]2[C@@H](N(C(=O)C2=O)CC[NH+]3CCOCC3)c4ccc(cc4)OCC=C
ZINC08440919	6.22	-6.9	-14.35	1	6	0	636.406	8	Cc1ccc(cc1)S(=O)(=O)N(C[C@H]2CCCO2)C[C@@H](Cn3c4ccc(cc4c5c3ccc(c5)Br)Br)O
ZINC08440918	3.39	-0.38	-56.23	2	8	1	493.58	11	CCOc1ccc(cc1)C(=O)C2=C(C(=O)N([C@@H]2c3ccc(cc3)OCC=C)CC[NH+]4CCOCC4)O
ZINC08440918	2.8	-0.46	-67.88	1	8	1	493.58	11	CCOc1ccc(cc1)C(=O)[C@H]2[C@H](N(C(=O)C2=O)CC[NH+]3CCOCC3)c4ccc(cc4)OCC=C
ZINC08440917	6.22	-6.53	-18.97	1	6	0	636.406	8	Cc1ccc(cc1)S(=O)(=O)N(C[C@@H]2CCCO2)C[C@@H](Cn3c4ccc(cc4c5c3ccc(c5)Br)Br)O
ZINC08440916	6.76	-5.65	-27.07	2	7	0	685.438	8	Cc1ccc(cc1)S(=O)(=O)N(C[C@H](Cn2c3ccc(cc3c4c2ccc(c4)Br)Br)O)c5ccc(cc5)NC(=O)C
ZINC08440915	3.03	0.5	-58.81	2	9	1	513.611	11	Cc1ccc(o1)C(=O)C2=C(C(=O)N([C@H]2c3ccc(c(c3)OC)OCCC(C)C)CC[NH+]4CCOCC4)O
ZINC08440915	3.48	0.77	-71.43	2	9	1	513.611	10	Cc1ccc(o1)/C(=C\2/[C@@H](N(C(=O)C2=O)CC[NH+]3CCOCC3)c4ccc(c(c4)OC)OCCC(C)C)/O
ZINC08440915	2.45	0.28	-70.5	1	9	1	513.611	11	Cc1ccc(o1)C(=O)[C@@H]2[C@@H](N(C(=O)C2=O)CC[NH+]3CCOCC3)c4ccc(c(c4)OC)OCCC(C)C
ZINC08440914	5.92	14.12	-28.62	1	6	0	475.585	7	Cc1cc(c(c(c1)C)COc2ccc(cc2OC)[C@@H]3C4=C(CCCC4=O)NC(=C3C(=O)OC)C)C
ZINC08440914	6.04	0.47	-13.05	0	6	0	475.585	7	Cc1cc(c(c(c1)C)COc2ccc(cc2OC)[C@@H]3C4C(=O)CCC=C4N=C(C3C(=O)OC)C)C
ZINC08440913	6.76	-6.6	-21.07	2	7	0	685.438	8	Cc1ccc(cc1)S(=O)(=O)N(C[C@@H](Cn2c3ccc(cc3c4c2ccc(c4)Br)Br)O)c5ccc(cc5)NC(=O)C
ZINC08440912	3.03	0.1	-50.13	2	9	1	513.611	11	Cc1ccc(o1)C(=O)C2=C(C(=O)N([C@@H]2c3ccc(c(c3)OC)OCCC(C)C)CC[NH+]4CCOCC4)O
ZINC08440912	2.45	-0.08	-58.39	1	9	1	513.611	11	Cc1ccc(o1)C(=O)[C@H]2[C@H](N(C(=O)C2=O)CC[NH+]3CCOCC3)c4ccc(c(c4)OC)OCCC(C)C
ZINC08440911	7.99	-4.34	-20.02	0	7	0	670.812	10	c1ccc(cc1)CN(c2ccccc2)S(=O)(=O)c3ccc-4c(c3)C(=O)c5c4ccc(c5)S(=O)(=O)N(Cc6ccccc6)c7ccccc7
ZINC08440910	8.6	-4.47	-17.22	0	6	0	656.829	10	c1ccc(cc1)CN(c2ccccc2)S(=O)(=O)c3ccc-4c(c3)Cc5c4ccc(c5)S(=O)(=O)N(Cc6ccccc6)c7ccccc7
ZINC08440909	3.45	-0.65	-64.25	2	8	1	515.652	11	CC(C)CCOc1ccc(cc1OC)[C@H]2C(=C(C(=O)N2CC[NH+]3CCOCC3)O)C(=O)c4cccs4

Supplementary Table S2:
Detailed table of XP Docking using Schrodinger's Glide module

ZINC_ID	xLogP	ApolarDesolvation	PolarDesolvation	H Donors	H Acceptors	Charge	MolecularWeight	RotatableBonds	SMILES
ZINC08440909	2.87	-0.87	-76.82	1	8	1	515.652	11	CC(C)CCOc1ccc(cc1OC)[C@H]2[C@H](C(=O)C(=O)N2CC[NH+]3CCOCC3)C(=O)c4cccs4
ZINC08440908	6.74	14.58	-16.53	1	5	0	494.031	6	Cc1cc(c(cc1COc2ccc(cc2)Cl)[C@H]3C4=C(CC(CC4=O)(C)C)NC(=C3C(=O)OC)C)C
ZINC08440908	6.85	0.87	-8.78	0	5	0	494.031	6	Cc1cc(c(cc1COc2ccc(cc2)Cl)[C@H]3C4C(=O)CC(C=C4N=C(C3C(=O)OC)C)(C)C)C
ZINC08440907	3.45	-0.71	-53.61	2	8	1	515.652	11	CC(C)CCOc1ccc(cc1OC)[C@@H]2C(=C(C(=O)N2CC[NH+]3CCOCC3)O)C(=O)c4cccs4
ZINC08440907	2.87	-0.93	-63.4	1	8	1	515.652	11	CC(C)CCOc1ccc(cc1OC)[C@@H]2[C@@H](C(=O)C(=O)N2CC[NH+]3CCOCC3)C(=O)c4cccs4
ZINC08440906	6.42	2.98	-14.07	1	12	0	505.439	9	CCCCOC(=O)c1cc(cc2c1-c3c(cc(cc3[N+](=O)[O-])[N+](=O)[O-])C2=Cc4ccccc4O)[N+](=O)[O-]
ZINC08440905	5.74	16.74	-15.39	0	12	0	479.401	9	CCCCOC(=O)c1cc(cc\2c1-c3c(cc(cc3[N+](=O)[O-])[N+](=O)[O-])/C2=C\c4ccco4)[N+](=O)[O-]
ZINC09046769	4.08	-0.37	-46.31	2	7	1	465.57	8	CCCOc1ccc(cc1)[C@H]2/C(=C(/c3ccc(cc3)C)\O)/C(=O)C(=O)N2CC[NH+]4CCOCC4
ZINC09046769	3.05	-0.55	-48.83	1	7	1	465.57	9	CCCOc1ccc(cc1)[C@H]2[C@H](C(=O)C(=O)N2CC[NH+]3CCOCC3)C(=O)c4ccc(cc4)C
ZINC08440903	6.74	14.6	-16.38	1	5	0	494.031	6	Cc1cc(c(cc1COc2ccc(cc2)Cl)[C@@H]3C4=C(CC(CC4=O)(C)C)NC(=C3C(=O)OC)C)C
ZINC08440903	6.85	0.86	-8.71	0	5	0	494.031	6	Cc1cc(c(cc1COc2ccc(cc2)Cl)[C@@H]3C4C(=O)CC(C=C4N=C(C3C(=O)OC)C)(C)C)C
ZINC02063453	6.91	3.64	-19.46	0	3	0	445.562	2	Cc1cccc(c1)N2C3=C([C@H](C4=C2c5ccccc5C4=O)c6ccccc6)C(=O)CC(C3)(C)C
ZINC02063454	6.91	3.63	-19.75	0	3	0	445.562	2	Cc1cccc(c1)N2C3=C([C@@H](C4=C2c5ccccc5C4=O)c6ccccc6)C(=O)CC(C3)(C)C
ZINC02063452	8.55	2.42	-8.58	0	1	0	456.61	4	Cc1ccc(cc1)Sc2ccc(cc2)[C@@]3(C=Cc4c5ccccc5ccc4O3)c6ccccc6
ZINC06445340	6.5	-0.43	-3.21	1	1	0	442.127	1	c1cc2c(c(c1)I)N[C@H]([C@H]3[C@@H]2C=CC3)c4ccc(cc4Cl)Cl
ZINC09046768	4.08	-0.13	-48.28	2	7	1	465.57	8	CCCOc1ccc(cc1)[C@@H]2/C(=C(/c3ccc(cc3)C)\O)/C(=O)C(=O)N2CC[NH+]4CCOCC4
ZINC02063439	6.5	-1.51	-1.84	1	1	0	442.127	1	c1cc2c(c(c1)I)N[C@@H]([C@H]3[C@@H]2C=CC3)c4ccc(cc4Cl)Cl
ZINC06445339	6.5	-1.03	-1.85	1	1	0	442.127	1	c1cc2c(c(c1)I)N[C@H]([C@@H]3[C@@H]2C=CC3)c4ccc(cc4Cl)Cl
ZINC08440901	4.58	-0.72	-56.66	2	7	1	534.032	9	c1ccc(cc1)COc2ccc(cc2)[C@H]3C(=C(C(=O)N3CC[NH+]4CCOCC4)O)C(=O)c5ccc(cc5)Cl
ZINC08440901	4	-0.88	-66.59	1	7	1	534.032	9	c1ccc(cc1)COc2ccc(cc2)[C@H]3[C@@H](C(=O)C(=O)N3CC[NH+]4CCOCC4)C(=O)c5ccc(cc5)Cl
ZINC08440900	6.57	14.51	-21.47	1	6	0	503.639	7	Cc1cc(c(c(c1)C)COc2ccc(cc2OC)[C@H]3C4=C(CC(CC4=O)(C)C)NC(=C3C(=O)OC)C)C
ZINC08440900	6.69	1.41	-10.45	0	6	0	503.639	7	Cc1cc(c(c(c1)C)COc2ccc(cc2OC)[C@H]3C4C(=O)CC(C=C4N=C(C3C(=O)OC)C)(C)C)C
ZINC06445337	6.5	-1.04	-3.21	1	1	0	442.127	1	c1cc2c(c(c1)I)N[C@@H]([C@@H]3[C@@H]2C=CC3)c4ccc(cc4Cl)Cl
ZINC08440899	4.58	-0.6	-55.95	2	7	1	534.032	9	c1ccc(cc1)COc2ccc(cc2)[C@@H]3C(=C(C(=O)N3CC[NH+]4CCOCC4)O)C(=O)c5ccc(cc5)Cl
ZINC08440899	4	-0.77	-65.49	1	7	1	534.032	9	c1ccc(cc1)COc2ccc(cc2)[C@@H]3[C@H](C(=O)C(=O)N3CC[NH+]4CCOCC4)C(=O)c5ccc(cc5)Cl
ZINC06445680	5.23	0.63	-67.1	1	9	-1	524.962	6	c1ccc(c(c1)[C@H]2[C@H]3C[C@@H]([C@@H]([C@@H]3c4cccc(c4N2)C(=O)[O-])Cl)Sc5ccccc5[N+](=O)[O-])[N+](=O)[O-]
ZINC06445651	5.23	0.86	-72.86	1	9	-1	524.962	6	c1ccc(c(c1)[C@@H]2[C@H]3C[C@@H]([C@@H]([C@@H]3c4cccc(c4N2)C(=O)[O-])Cl)Sc5ccccc5[N+](=O)[O-])[N+](=O)[O-]
ZINC08440898	3.38	-0.15	-52.79	2	8	1	503.575	9	Cc1ccc(o1)C(=O)C2=C(C(=O)N([C@H]2c3ccc(cc3)OCc4ccccc4)CC[NH+]5CCOCC5)O
ZINC08440898	2.8	-0.31	-61.47	1	8	1	503.575	9	Cc1ccc(o1)C(=O)[C@H]2[C@@H](N(C(=O)C2=O)CC[NH+]3CCOCC3)c4ccc(cc4)OCc5ccccc5
ZINC06445669	5.23	0.57	-89.03	1	9	-1	524.962	6	c1ccc(c(c1)[C@H]2[C@@H]3C[C@@H]([C@@H]([C@@H]3c4cccc(c4N2)C(=O)[O-])Cl)Sc5ccccc5[N+](=O)[O-])[N+](=O)[O-]
ZINC06445639	5.23	1.03	-67.41	1	9	-1	524.962	6	c1ccc(c(c1)[C@@H]2[C@@H]3C[C@@H]([C@@H]([C@@H]3c4cccc(c4N2)C(=O)[O-])Cl)Sc5ccccc5[N+](=O)[O-])[N+](=O)[O-]
ZINC08440897	6.57	14.52	-21.48	1	6	0	503.639	7	Cc1cc(c(c(c1)C)COc2ccc(cc2OC)[C@@H]3C4=C(CC(CC4=O)(C)C)NC(=C3C(=O)OC)C)C
ZINC08440897	6.69	1.24	-11.04	0	6	0	503.639	7	Cc1cc(c(c(c1)C)COc2ccc(cc2OC)[C@@H]3C4C(=O)CC(C=C4N=C(C3C(=O)OC)C)(C)C)C
ZINC06445812	3.65	-0.21	-49.93	1	9	-1	448.864	5	c1ccc(c(c1)[N+](=O)[O-])S[C@H]2C[C@H]3CNc4c(ccc(c4[C@@H]3[C@@H]2Cl)[N+](=O)[O-])C(=O)[O-]
ZINC08440896	3.38	-0.39	-49.53	2	8	1	503.575	9	Cc1ccc(o1)C(=O)C2=C(C(=O)N([C@@H]2c3ccc(cc3)OCc4ccccc4)CC[NH+]5CCOCC5)O
ZINC08440896	2.8	-0.55	-57.76	1	8	1	503.575	9	Cc1ccc(o1)C(=O)[C@@H]2[C@H](N(C(=O)C2=O)CC[NH+]3CCOCC3)c4ccc(cc4)OCc5ccccc5
ZINC06445743	3.65	0.01	-54.62	1	9	-1	448.864	5	c1ccc(c(c1)[N+](=O)[O-])S[C@@H]2C[C@H]3CNc4c(ccc(c4[C@@H]3[C@@H]2Cl)[N+](=O)[O-])C(=O)[O-]
ZINC06445778	3.65	-0.54	-48.81	1	9	-1	448.864	5	c1ccc(c(c1)[N+](=O)[O-])S[C@H]2C[C@@H]3CNc4c(ccc(c4[C@@H]3[C@@H]2Cl)[N+](=O)[O-])C(=O)[O-]
ZINC06445684	3.65	-0.08	-56.85	1	9	-1	448.864	5	c1ccc(c(c1)[N+](=O)[O-])S[C@@H]2C[C@@H]3CNc4c(ccc(c4[C@@H]3[C@@H]2Cl)[N+](=O)[O-])C(=O)[O-]
ZINC08440895	5.19	1.24	-49.43	1	12	-1	569.959	7	c1ccc(c(c1)[N+](=O)[O-])S[C@H]2C[C@H]3[C@@H](Nc4c(ccc(c4[C@@H]3[C@@H]2Cl)[N+](=O)[O-])C(=O)[O-])c5ccc(cc5)[N+](=O)[O-]
ZINC08440894	3.8	-1.47	-54.42	2	7	1	505.616	9	c1ccc(cc1)COc2ccc(cc2)[C@H]3C(=C(C(=O)N3CC[NH+]4CCOCC4)O)C(=O)c5cccs5
ZINC08440894	3.22	-1.63	-63.13	1	7	1	505.616	9	c1ccc(cc1)COc2ccc(cc2)[C@H]3[C@@H](C(=O)C(=O)N3CC[NH+]4CCOCC4)C(=O)c5cccs5
ZINC08440893	5.19	1.38	-56.74	1	12	-1	569.959	7	c1ccc(c(c1)[N+](=O)[O-])S[C@@H]2C[C@H]3[C@@H](Nc4c(ccc(c4[C@@H]3[C@@H]2Cl)[N+](=O)[O-])C(=O)[O-])c5ccc(cc5)[N+](=O)[O-]
ZINC08440892	6.9	2.69	-11.16	0	6	0	517.666	8	CCOC(=O)C1[C@@H](C2=C(CC(CC2=O)(C)C)N=C1C)c3ccc(c(c3)OC)OCc4c(cc(cc4C)C)C
ZINC08440892	6.9	2.68	-14.48	0	6	0	517.666	8	CCOC(=O)C1=C(N=C2CC(CC(=O)C2[C@H]1c3ccc(c(c3)OC)OCc4c(cc(cc4C)C)C)(C)C)C
ZINC08440892	7.08	1.75	-12.24	0	6	0	517.666	8	CCOC(=O)C1[C@@H](C2C(=NC1=C)CC(CC2=O)(C)C)c3ccc(c(c3)OC)OCc4c(cc(cc4C)C)C
ZINC08440891	5.19	1.44	-58.11	1	12	-1	569.959	7	c1ccc(c(c1)[N+](=O)[O-])S[C@H]2C[C@@H]3[C@@H](Nc4c(ccc(c4[C@@H]3[C@@H]2Cl)[N+](=O)[O-])C(=O)[O-])c5ccc(cc5)[N+](=O)[O-]
ZINC08440890	5.19	1.36	-61.53	1	12	-1	569.959	7	c1ccc(c(c1)[N+](=O)[O-])S[C@@H]2C[C@@H]3[C@@H](Nc4c(ccc(c4[C@@H]3[C@@H]2Cl)[N+](=O)[O-])C(=O)[O-])c5ccc(cc5)[N+](=O)[O-]
ZINC08440889	3.8	-1.75	-50.92	2	7	1	505.616	9	c1ccc(cc1)COc2ccc(cc2)[C@@H]3C(=C(C(=O)N3CC[NH+]4CCOCC4)O)C(=O)c5cccs5
ZINC08440889	3.22	-1.9	-60.21	1	7	1	505.616	9	c1ccc(cc1)COc2ccc(cc2)[C@@H]3[C@@H](C(=O)C(=O)N3CC[NH+]4CCOCC4)C(=O)c5cccs5
ZINC06445822	6.06	3.15	-13.9	1	7	0	549.958	6	c1ccc(c(c1)[N+](=O)[O-])S[C@H]2C[C@H]3[C@@H](Nc4c(cccc4C(F)(F)F)[C@@H]3[C@@H]2Cl)c5cccc(c5)[N+](=O)[O-]
ZINC06445750	6.06	3.22	-19.38	1	7	0	549.958	6	c1ccc(c(c1)[N+](=O)[O-])S[C@@H]2C[C@H]3[C@@H](Nc4c(cccc4C(F)(F)F)[C@@H]3[C@@H]2Cl)c5cccc(c5)[N+](=O)[O-]
ZINC06445785	6.06	3.34	-14.06	1	7	0	549.958	6	c1ccc(c(c1)[N+](=O)[O-])S[C@H]2C[C@@H]3[C@@H](Nc4c(cccc4C(F)(F)F)[C@@H]3[C@@H]2Cl)c5cccc(c5)[N+](=O)[O-]
ZINC06445695	6.06	3.41	-19.5	1	7	0	549.958	6	c1ccc(c(c1)[N+](=O)[O-])S[C@@H]2C[C@@H]3[C@@H](Nc4c(cccc4C(F)(F)F)[C@@H]3[C@@H]2Cl)c5cccc(c5)[N+](=O)[O-]
ZINC08440888	5.14	2.14	-55.34	2	7	1	527.641	12	Cc1cc(ccc1OCC=C)C(=O)C2=C(C(=O)N([C@H]2c3ccc(cc3)OCc4ccccc4)CC[NH+](C)C)O
ZINC08440888	4.55	2.05	-66.63	1	7	1	527.641	12	Cc1cc(ccc1OCC=C)C(=O)[C@@H]2[C@@H](N(C(=O)C2=O)CC[NH+](C)C)c3ccc(cc3)OCc4ccccc4
ZINC08440887	6.9	2.7	-11.19	0	6	0	517.666	8	CCOC(=O)C1[C@H](C2=C(CC(CC2=O)(C)C)N=C1C)c3ccc(c(c3)OC)OCc4c(cc(cc4C)C)C
ZINC08440887	7.08	1.53	-11.83	0	6	0	517.666	8	CCOC(=O)C1[C@H](C2C(=NC1=C)CC(CC2=O)(C)C)c3ccc(c(c3)OC)OCc4c(cc(cc4C)C)C
ZINC08440887	6.9	2.44	-14.11	0	6	0	517.666	8	CCOC(=O)C1=C(N=C2CC(CC(=O)C2[C@@H]1c3ccc(c(c3)OC)OCc4c(cc(cc4C)C)C)(C)C)C
ZINC06445857	2.98	0.34	-59.25	1	9	-1	448.864	5	c1ccc(c(c1)[N+](=O)[O-])S[C@H]2C[C@H]3[C@@H]([C@@H]2Cl)c4cccc(c4N[C@@H]3C(=O)[O-])[N+](=O)[O-]
ZINC06445775	2.98	0.45	-79.42	1	9	-1	448.864	5	c1ccc(c(c1)[N+](=O)[O-])S[C@@H]2C[C@H]3[C@@H]([C@@H]2Cl)c4cccc(c4N[C@@H]3C(=O)[O-])[N+](=O)[O-]

Supplementary Table S2:
Detailed table of XP Docking using Schrodinger's Glide module

ZINC_ID	xLogP	ApolarDesolvation	PolarDesolvation	H Donors	H Acceptors	Charge	MolecularWeight	RotatableBonds	SMILES
ZINC08440886	5.14	2.08	-56.24	2	7	1	527.641	12	Cc1cc(ccc1OCC=C)C(=O)C2=C(C(=O)N([C@@H]2c3ccc(cc3)OCc4ccccc4)CC[NH+](C)C)O
ZINC08440886	4.55	1.91	-65.77	1	7	1	527.641	12	Cc1cc(ccc1OCC=C)C(=O)[C@@H]2[C@H](N(C(=O)C2=O)CC[NH+](C)C)c3ccc(cc3)OCc4ccccc4
ZINC06445809	2.98	0.02	-58.75	1	9	-1	448.864	5	c1ccc(c(c1)[N+](=O)[O-])S[C@H]2C[C@@H]3[C@@H]([C@@H]2Cl)c4cccc(c4N[C@@H]3C(=O)[O-])[N+](=O)[O-]
ZINC06445739	2.98	0.16	-54.2	1	9	-1	448.864	5	c1ccc(c(c1)[N+](=O)[O-])S[C@@H]2C[C@@H]3[C@@H]([C@@H]2Cl)c4cccc(c4N[C@@H]3C(=O)[O-])[N+](=O)[O-]
ZINC08440885	6.87	15.08	-15.42	1	5	0	538.482	6	Cc1cc(c(cc1COc2ccc(cc2)Br)[C@H]3C4=C(CC(CC4=O)(C)C)NC(=C3C(=O)OC)C)C
ZINC08440885	6.99	0.26	-8.73	0	5	0	538.482	6	Cc1cc(c(cc1COc2ccc(cc2)Br)[C@H]3C4C(=O)CC(C=C4N=C(C3C(=O)OC)C)(C)C)C
ZINC08919518	4.44	0.71	-42.81	2	6	1	443.589	10	CCCOc1ccc(cc1)[C@H]2/C(=C(/c3cccs3)\O)/C(=O)C(=O)N2CC[NH+](CC)CC
ZINC08440883	7.63	-0.56	-12.18	1	4	0	550.305	4	c1ccc(c(c1)[C@H]2[C@H]3C[C@@H]([C@@H]([C@@H]3c4cc(ccc4N2)Cl)Cl)Sc5ccccc5[N+](=O)[O-])Br
ZINC08440882	7.63	-0.76	-12.74	1	4	0	550.305	4	c1ccc(c(c1)[C@@H]2[C@H]3C[C@@H]([C@@H]([C@@H]3c4cc(ccc4N2)Cl)Cl)Sc5ccccc5[N+](=O)[O-])Br
ZINC08919514	4.44	0.52	-43.16	2	6	1	443.589	10	CCCOc1ccc(cc1)[C@@H]2/C(=C(/c3cccs3)\O)/C(=O)C(=O)N2CC[NH+](CC)CC
ZINC08440880	7.63	-0.51	-13.66	1	4	0	550.305	4	c1ccc(c(c1)[C@H]2[C@@H]3C[C@@H]([C@@H]([C@@H]3c4cc(ccc4N2)Cl)Cl)Sc5ccccc5[N+](=O)[O-])Br
ZINC08440879	7.63	-0.27	-13.92	1	4	0	550.305	4	c1ccc(c(c1)[C@@H]2[C@@H]3C[C@@H]([C@@H]([C@@H]3c4cc(ccc4N2)Cl)Cl)Sc5ccccc5[N+](=O)[O-])Br
ZINC00850230	5.04	-1	-9.31	0	6	0	427.309	7	COc1cc(cc(c1OC)OC)c2nnc(o2)SCc3ccc(cc3Cl)Cl
ZINC08440878	6.87	15.1	-15.55	1	5	0	538.482	6	Cc1cc(c(cc1COc2ccc(cc2)Br)[C@@H]3C4=C(CC(CC4=O)(C)C)NC(=C3C(=O)OC)C)C
ZINC08440878	6.99	0.25	-8.63	0	5	0	538.482	6	Cc1cc(c(cc1COc2ccc(cc2)Br)[C@@H]3C4C(=O)CC(C=C4N=C(C3C(=O)OC)C)(C)C)C
ZINC08440877	4.42	12.15	-55.61	0	6	-1	430.48	8	CCCOc1ccc(cc1)[C@H]2C(=C(C(=O)N2Cc3ccco3)[O-])C(=O)c4ccc(cc4)C
ZINC08440877	4.86	1.17	-26.68	1	6	0	431.488	7	CCCOc1ccc(cc1)[C@H]2C(=C(c3ccc(cc3)C)O)C(=O)C(=O)N2Cc4ccco4
ZINC08440877	3.83	1.4	-17.66	0	6	0	431.488	8	CCCOc1ccc(cc1)[C@H]2[C@@H](C(=O)C(=O)N2Cc3ccco3)C(=O)c4ccc(cc4)C
ZINC08440876	3	-1.43	-29.12	3	10	0	587.702	11	Cc1c(c(=O)n(n1C)c2ccccc2)NC(=O)[C@H](CCSC)NC(=O)c3ccccc3NC(=O)c4ccc(cc4)OC
ZINC08440875	3	-1.2	-29.19	3	10	0	587.702	11	Cc1c(c(=O)n(n1C)c2ccccc2)NC(=O)[C@@H](CCSC)NC(=O)c3ccccc3NC(=O)c4ccc(cc4)OC
ZINC08440874	4.42	12.1	-58.63	0	6	-1	430.48	8	CCCOc1ccc(cc1)[C@@H]2C(=C(C(=O)N2Cc3ccco3)[O-])C(=O)c4ccc(cc4)C
ZINC08440874	4.86	1.37	-29.72	1	6	0	431.488	7	CCCOc1ccc(cc1)[C@@H]2C(=C(c3ccc(cc3)C)O)C(=O)C(=O)N2Cc4ccco4
ZINC08440874	3.83	1.58	-20.02	0	6	0	431.488	8	CCCOc1ccc(cc1)[C@@H]2[C@H](C(=O)C(=O)N2Cc3ccco3)C(=O)c4ccc(cc4)C
ZINC08440873	7.12	-7.92	-15.6	1	7	0	564.814	16	CCCCN(CCCC)S(=O)(=O)c1ccc-2c(c1)C(c3c2ccc(c3)S(=O)(=O)N(CCCC)CCCC)O
ZINC00970803	1.2	8.92	-29.3	1	11	0	372.341	4	Cc1ccc(cc1NC(=O)Cn2cnc3c2c(=O)n(c(=O)n3C)C)[N+](=O)[O-]
ZINC00970803	1.2	1.38	-62.03	2	11	1	373.349	4	Cc1ccc(cc1NC(=O)Cn2c[nH+]c3c2c(=O)n(c(=O)n3C)C)[N+](=O)[O-]
ZINC00851794	1.9	-0.53	-17.17	1	8	0	439.213	3	Cn1c2c(c(=O)n(c1=O)C)n(cn2)CC(=O)Nc3ccccc3I
ZINC00851794	1.9	-0.25	-53.69	2	8	1	440.221	3	Cn1c2c(c(=O)n(c1=O)C)n(c[nH+]2)CC(=O)Nc3ccccc3I
ZINC00702763	5.17	12.61	-42.59	0	6	-1	458.534	8	CCOc1ccc(cc1)C(=O)C2=C(C(=O)N([C@H]2c3ccc(cc3)C(C)(C)C)Cc4ccco4)[O-]
ZINC00702763	5.62	1.94	-26.67	1	6	0	459.542	7	CCOc1ccc(cc1)C(=C2[C@@H](N(C(=O)C2=O)Cc3ccco3)c4ccc(cc4)C(C)(C)C)O
ZINC00702763	4.59	2.17	-17.45	0	6	0	459.542	8	CCOc1ccc(cc1)C(=O)[C@H]2[C@@H](N(C(=O)C2=O)Cc3ccco3)c4ccc(cc4)C(C)(C)C
ZINC08440872	5.85	1.28	-14.26	0	5	0	483.605	7	CCOC(=O)C1[C@@H](C2=C(CC(CC2=O)(C)C)N=C1C)c3cc(c(s3)C)COc4ccc(cc4)F
ZINC08440872	6.03	0.38	-13.67	0	5	0	483.605	7	CCOC(=O)C1[C@@H](C2C(=NC1=C)CC(CC2=O)(C)C)c3cc(c(s3)C)COc4ccc(cc4)F
ZINC08440872	5.85	1.43	-16.3	0	5	0	483.605	7	CCOC(=O)C1=C(N=C2CC(CC(=O)C2[C@H]1c3cc(c(s3)C)COc4ccc(cc4)F)(C)C)C
ZINC02063421	4.87	0.24	-9.98	2	7	0	430.247	5	c1ccc(c(c1)NC(=O)c2cc(cc(c2)[N+](=O)[O-])C(=O)Nc3ccccc3Cl)Cl
ZINC02063420	5.65	4.76	-9.07	0	7	0	445.519	7	CCN(c1ccccc1C)C(=O)c2cc(cc(c2)[N+](=O)[O-])C(=O)N(CC)c3ccccc3C
ZINC02063419	-1.1	6.58	-28.42	0	11	0	365.309	7	c1c(cc(cc1C(=O)N(CC#N)CC#N)[N+](=O)[O-])C(=O)N(CC#N)CC#N
ZINC00702764	5.17	12.63	-42.97	0	6	-1	458.534	8	CCOc1ccc(cc1)C(=O)C2=C(C(=O)N([C@@H]2c3ccc(cc3)C(C)(C)C)Cc4ccco4)[O-]
ZINC00702764	5.62	1.63	-26.85	1	6	0	459.542	7	CCOc1ccc(cc1)C(=C2[C@H](N(C(=O)C2=O)Cc3ccco3)c4ccc(cc4)C(C)(C)C)O
ZINC00702764	4.59	1.93	-21.91	0	6	0	459.542	8	CCOc1ccc(cc1)C(=O)[C@H]2[C@H](N(C(=O)C2=O)Cc3ccco3)c4ccc(cc4)C(C)(C)C
ZINC08440871	3.49	2.04	-14.35	2	15	0	511.403	9	COc1ccc(c(c1)[N+](=O)[O-])NC(=O)c2cc(cc(c2)[N+](=O)[O-])C(=O)Nc3ccc(cc3[N+](=O)[O-])OC
ZINC08440870	4.74	1.26	-19.68	2	13	0	520.241	7	c1cc(c(cc1[N+](=O)[O-])NC(=O)c2cc(cc(c2)[N+](=O)[O-])C(=O)Nc3cc(ccc3Cl)[N+](=O)[O-])Cl
ZINC00702759	3.86	10.02	-40.87	0	6	-1	440.903	7	CCOc1ccc(cc1)C(=O)C2=C(C(=O)N([C@H]2c3ccc(cc3)Cl)C[C@H]4CCCO4)[O-]
ZINC00702759	4.31	-1.29	-25.18	1	6	0	441.911	6	CCOc1ccc(cc1)C(=C2[C@@H](N(C(=O)C2=O)C[C@H]3CCCO3)c4ccc(cc4)Cl)O
ZINC00702759	3.28	-1	-17.62	0	6	0	441.911	7	CCOc1ccc(cc1)C(=O)[C@@H]2[C@@H](N(C(=O)C2=O)C[C@H]3CCCO3)c4ccc(cc4)Cl
ZINC08440869	5.85	1.47	-11.15	0	5	0	483.605	7	CCOC(=O)C1[C@H](C2=C(CC(CC2=O)(C)C)N=C1C)c3cc(c(s3)C)COc4ccc(cc4)F
ZINC08440869	5.85	1.54	-14.34	0	5	0	483.605	7	CCOC(=O)C1=C(N=C2CC(CC(=O)C2[C@@H]1c3cc(c(s3)C)COc4ccc(cc4)F)(C)C)C
ZINC08440869	6.03	0.59	-12.35	0	5	0	483.605	7	CCOC(=O)C1[C@H](C2C(=NC1=C)CC(CC2=O)(C)C)c3cc(c(s3)C)COc4ccc(cc4)F
ZINC06300109	5.52	13.01	-20.73	1	5	0	469.578	6	Cc1c(cc(s1)[C@H]2C3=C(CC(CC3=O)(C)C)NC(=C2C(=O)OC)C)COc4ccc(cc4)F
ZINC06300109	5.63	0.02	-10.69	0	5	0	469.578	6	Cc1c(cc(s1)[C@H]2C3C(=O)CC(C=C3N=C(C2C(=O)OC)C)(C)C)COc4ccc(cc4)F
ZINC00851625	3.69	6.64	-13.7	2	11	0	481.461	9	COc1ccc(c(c1)OC)NC(=O)c2cc(cc(c2)[N+](=O)[O-])C(=O)Nc3ccc(cc3OC)OC
ZINC00970788	4.38	-0.08	-24.36	1	7	0	422.444	3	c1ccc2c(c1)cccc2n3nc4ccc(cc4n3)NC(=O)c5ccc6c(c5)OCCO6
ZINC00702760	3.86	9.82	-42.09	0	6	-1	440.903	7	CCOc1ccc(cc1)C(=O)C2=C(C(=O)N([C@@H]2c3ccc(cc3)Cl)C[C@H]4CCCO4)[O-]
ZINC00702760	4.31	-1.5	-30.41	1	6	0	441.911	6	CCOc1ccc(cc1)C(=C2[C@H](N(C(=O)C2=O)C[C@H]3CCCO3)c4ccc(cc4)Cl)O
ZINC00702760	3.28	-1.21	-23.24	0	6	0	441.911	7	CCOc1ccc(cc1)C(=O)[C@H]2[C@H](N(C(=O)C2=O)C[C@H]3CCCO3)c4ccc(cc4)Cl
ZINC06300107	5.52	13	-20.81	1	5	0	469.578	6	Cc1c(cc(s1)[C@@H]2C3=C(CC(CC3=O)(C)C)NC(=C2C(=O)OC)C)COc4ccc(cc4)F
ZINC06300107	5.63	0	-10.79	0	5	0	469.578	6	Cc1c(cc(s1)[C@@H]2C3C(=O)CC(C=C3N=C(C2C(=O)OC)C)(C)C)COc4ccc(cc4)F
ZINC02063410	6.49	-1.4	-14.57	1	5	0	457.711	4	COc1ccc(cc1C(=O)Nc2cc(ccc2Cl)c3nc4ccccc4o3)Br
ZINC00702761	3.86	9.76	-41.76	0	6	-1	440.903	7	CCOc1ccc(cc1)C(=O)C2=C(C(=O)N([C@H]2c3ccc(cc3)Cl)C[C@@H]4CCCO4)[O-]
ZINC00702761	4.31	-1.47	-30.19	1	6	0	441.911	6	CCOc1ccc(cc1)C(=C2[C@@H](N(C(=O)C2=O)C[C@@H]3CCCO3)c4ccc(cc4)Cl)O
ZINC00702761	3.28	-1.18	-23.53	0	6	0	441.911	7	CCOc1ccc(cc1)C(=O)[C@@H]2[C@@H](N(C(=O)C2=O)C[C@@H]3CCCO3)c4ccc(cc4)Cl

Supplementary Table S2:
Detailed table of XP Docking using Schrodinger's Glide module

ZINC_ID	xLogP	ApolarDesolvation	PolarDesolvation	H Donors	H Acceptors	Charge	MolecularWeight	RotatableBonds	SMILES
ZINC00970786	5.77	-0.78	-19.31	1	7	0	419.824	5	c1cc(cc(c1)[N+](=O)[O-])/C=C/C(=O)Nc2ccc(cc2)c3nc4cc(ccc4o3)Cl
ZINC02063408	6.66	-0.88	-18.43	1	5	0	451.32	4	Cc1cc2c(cc1C)oc(n2)c3cccc(c3)NC(=O)c4cc(ccc4OC)Br
ZINC08440868	6.44	2.92	-11.39	1	5	0	513.439	4	[H]/N=C/1\[C@H](C2=CCCC[C@@H]2[C@@H](C1(C#N)C#N)c3cc(c(cc3C)C)COc4ccc(cc4)Br)C#N
ZINC08440868	6.44	2.75	-33.72	2	5	1	514.447	4	Cc1cc(c(cc1COc2ccc(cc2)Br)[C@H]3[C@H]4CCCC=C4[C@@H](C(=[NH2+])C3(C#N)C#N)C#N)C
ZINC02063407	6.66	-0.77	-19.9	1	5	0	451.32	4	Cc1cc(c2c(c1)nc(o2)c3cccc(c3)NC(=O)c4cc(ccc4OC)Br)C
ZINC00702762	3.86	9.59	-41.12	0	6	-1	440.903	7	CCOc1ccc(cc1)C(=O)C2=C(C(=O)N([C@@H]2c3ccc(cc3)Cl)C[C@@H]4CCCO4)[O-]
ZINC00702762	4.31	-1.74	-30.82	1	6	0	441.911	6	CCOc1ccc(cc1)C(=C2[C@H](N(C(=O)C2=O)C[C@@H]3CCCO3)c4ccc(cc4)Cl)O
ZINC00702762	3.28	-1.45	-25.3	0	6	0	441.911	7	CCOc1ccc(cc1)C(=O)[C@H]2[C@H](N(C(=O)C2=O)C[C@@H]3CCCO3)c4ccc(cc4)Cl
ZINC00850292	6.28	-1.1	-19.26	1	5	0	437.293	4	Cc1ccc2c(c1)oc(n2)c3cccc(c3)NC(=O)c4cc(ccc4OC)Br
ZINC00850290	6.28	-1.1	-18.19	1	5	0	437.293	4	Cc1ccc2c(c1)nc(o2)c3cccc(c3)NC(=O)c4cc(ccc4OC)Br
ZINC08440867	6.44	2.25	-11.11	1	5	0	513.439	4	[H]/N=C/1\[C@H](C2=CCCC[C@@H]2[C@H](C1(C#N)C#N)c3cc(c(cc3C)C)COc4ccc(cc4)Br)C#N
ZINC08440867	6.44	2.08	-40.63	2	5	1	514.447	4	Cc1cc(c(cc1COc2ccc(cc2)Br)[C@@H]3[C@H]4CCCC=C4[C@@H](C(=[NH2+])C3(C#N)C#N)C#N)C
ZINC00850286	5.86	-1.42	-18.58	1	5	0	423.266	4	COc1ccc(cc1C(=O)Nc2cccc(c2)c3nc4ccccc4o3)Br
ZINC08440866	5.15	2.34	-7.16	2	6	0	498.391	5	CCOC(=O)C1=c2n(c(=O)/c(=C\c3ccccc3Cl)/s2)C(=C([C@H]1c4cccc(c4)Cl)C#N)N
ZINC00702749	4.57	13.1	-53.3	0	6	-1	486.519	8	c1ccc(cc1)COc2ccc(cc2)[C@H]3C(=C(C(=O)N3C[C@H]4CCCO4)[O-])C(=O)c5ccc(cc5)F
ZINC00702749	5.01	-0.6	-31.33	1	6	0	487.527	7	c1ccc(cc1)COc2ccc(cc2)[C@H]3C(=C(c4ccc(cc4)F)O)C(=O)C(=O)N3C[C@H]5CCCO5
ZINC00702749	3.98	-0.34	-25.91	0	6	0	487.527	8	c1ccc(cc1)COc2ccc(cc2)[C@H]3[C@H](C(=O)C(=O)N3C[C@H]4CCCO4)C(=O)c5ccc(cc5)F
ZINC08440865	6.44	2.26	-11.1	1	5	0	513.439	4	[H]/N=C/1\[C@@H](C2=CCCC[C@H]2[C@@H](C1(C#N)C#N)c3cc(c(cc3C)C)COc4ccc(cc4)Br)C#N
ZINC08440865	6.44	2.09	-40.72	2	5	1	514.447	4	Cc1cc(c(cc1COc2ccc(cc2)Br)[C@H]3[C@@H]4CCCC=C4[C@H](C(=[NH2+])C3(C#N)C#N)C#N)C
ZINC08440864	5.15	2.36	-7.14	2	6	0	498.391	5	CCOC(=O)C1=c2n(c(=O)/c(=C\c3ccccc3Cl)/s2)C(=C([C@@H]1c4cccc(c4)Cl)C#N)N
ZINC02063399	5.15	7.18	-11.37	2	5	0	518.442	4	c1cc(cc(c1)C(F)(F)F)/C=c/2\c(=O)n3c(=C([C@H](C(=C3N)C#N)c4cccc(c4)C(F)(F)F)C#N)s2
ZINC02063401	5.15	7.16	-11.26	2	5	0	518.442	4	c1cc(cc(c1)C(F)(F)F)/C=c/2\c(=O)n3c(=C([C@@H](C(=C3N)C#N)c4cccc(c4)C(F)(F)F)C#N)s2
ZINC00702750	4.57	13.32	-54.22	0	6	-1	486.519	8	c1ccc(cc1)COc2ccc(cc2)[C@@H]3C(=C(C(=O)N3C[C@H]4CCCO4)[O-])C(=O)c5ccc(cc5)F
ZINC00702750	5.01	-0.48	-31.88	1	6	0	487.527	7	c1ccc(cc1)COc2ccc(cc2)[C@@H]3C(=C(c4ccc(cc4)F)O)C(=O)C(=O)N3C[C@H]5CCCO5
ZINC00702750	3.98	-0.19	-23.57	0	6	0	487.527	8	c1ccc(cc1)COc2ccc(cc2)[C@@H]3[C@@H](C(=O)C(=O)N3C[C@H]4CCCO4)C(=O)c5ccc(cc5)F
ZINC08440863	6.44	2.88	-11.36	1	5	0	513.439	4	[H]/N=C/1\[C@@H](C2=CCCC[C@H]2[C@H](C1(C#N)C#N)c3cc(c(cc3C)C)COc4ccc(cc4)Br)C#N
ZINC08440863	6.44	2.71	-33.84	2	5	1	514.447	4	Cc1cc(c(cc1COc2ccc(cc2)Br)[C@@H]3[C@@H]4CCCC=C4[C@H](C(=[NH2+])C3(C#N)C#N)C#N)C
ZINC00702751	4.57	12.98	-58.4	0	6	-1	486.519	8	c1ccc(cc1)COc2ccc(cc2)[C@H]3C(=C(C(=O)N3C[C@@H]4CCCO4)[O-])C(=O)c5ccc(cc5)F
ZINC00702751	5.01	-0.41	-31.51	1	6	0	487.527	7	c1ccc(cc1)COc2ccc(cc2)[C@H]3C(=C(c4ccc(cc4)F)O)C(=O)C(=O)N3C[C@@H]5CCCO5
ZINC00702751	3.98	-0.12	-23.72	0	6	0	487.527	8	c1ccc(cc1)COc2ccc(cc2)[C@H]3[C@H](C(=O)C(=O)N3C[C@@H]4CCCO4)C(=O)c5ccc(cc5)F
ZINC00702752	4.57	12.6	-57.82	0	6	-1	486.519	8	c1ccc(cc1)COc2ccc(cc2)[C@@H]3C(=C(C(=O)N3C[C@@H]4CCCO4)[O-])C(=O)c5ccc(cc5)F
ZINC00702752	5.01	-0.62	-32.13	1	6	0	487.527	7	c1ccc(cc1)COc2ccc(cc2)[C@@H]3C(=C(c4ccc(cc4)F)O)C(=O)C(=O)N3C[C@@H]5CCCO5
ZINC00702752	3.98	-0.34	-25.47	0	6	0	487.527	8	c1ccc(cc1)COc2ccc(cc2)[C@@H]3[C@@H](C(=O)C(=O)N3C[C@@H]4CCCO4)C(=O)c5ccc(cc5)F
ZINC08440862	5.2	0.66	-15.75	0	5	0	455.551	7	CCOC(=O)C1[C@@H](C2=C(CCCC2=O)N=C1C)c3cc(c(s3)C)COc4ccc(cc4)F
ZINC08440862	5.2	0.36	-17.34	0	5	0	455.551	7	CCOC(=O)C1=C(N=C2CCCC(=O)C2[C@H]1c3cc(c(s3)C)COc4ccc(cc4)F)C
ZINC08440862	5.38	-0.52	-15.77	0	5	0	455.551	7	CCOC(=O)C1[C@@H](C2C(=NC1=C)CCCC2=O)c3cc(c(s3)C)COc4ccc(cc4)F
ZINC08440861	1.01	0.98	-20.41	0	9	0	444.484	7	COc1cccc(c1C(=O)N2CCN(CC2)C(=O)c3cc(c(c(c3)OC)OC)OC)OC
ZINC00702745	4.84	12.83	-45.48	0	7	-1	512.582	10	CCOc1ccc(cc1)C(=O)C2=C(C(=O)N([C@H]2c3ccc(cc3)OCc4ccccc4)C[C@H]5CCCO5)[O-]
ZINC00702745	5.28	-1.32	-32.21	1	7	0	513.59	9	CCOc1ccc(cc1)C(=C2[C@@H](N(C(=O)C2=O)C[C@H]3CCCO3)c4ccc(cc4)OCc5ccccc5)O
ZINC00702745	4.25	-1.08	-25.23	0	7	0	513.59	10	CCOc1ccc(cc1)C(=O)[C@H]2[C@@H](N(C(=O)C2=O)C[C@H]3CCCO3)c4ccc(cc4)OCc5ccccc5
ZINC08440859	7.51	1.18	-13.17	0	4	0	499.394	4	Cc1ccc2c(c1C)nc3n2c(=O)/c(=C\c4cc(ccc4OCc5c(cccc5Cl)F)Cl)/s3
ZINC02063383	5.54	1.3	-11.73	1	4	0	439.555	5	CC1(CC2=C([C@H](Nc3c2c4ccccc4cc3)c5ccc(c(c5)OC)OCC=C)C(=O)C1)C
ZINC00702746	4.84	12.78	-46.28	0	7	-1	512.582	10	CCOc1ccc(cc1)C(=O)C2=C(C(=O)N([C@@H]2c3ccc(cc3)OCc4ccccc4)C[C@H]5CCCO5)[O-]
ZINC00702746	5.28	-1.17	-31.87	1	7	0	513.59	9	CCOc1ccc(cc1)C(=C2[C@H](N(C(=O)C2=O)C[C@H]3CCCO3)c4ccc(cc4)OCc5ccccc5)O
ZINC00702746	4.25	-0.87	-24.51	0	7	0	513.59	10	CCOc1ccc(cc1)C(=O)[C@H]2[C@H](N(C(=O)C2=O)C[C@H]3CCCO3)c4ccc(cc4)OCc5ccccc5
ZINC02063384	5.54	1.27	-11.7	1	4	0	439.555	5	CC1(CC2=C([C@@H](Nc3c2c4ccccc4cc3)c5ccc(c(c5)OC)OCC=C)C(=O)C1)C
ZINC08440858	5.2	0.68	-11.5	0	5	0	455.551	7	CCOC(=O)C1[C@H](C2=C(CCCC2=O)N=C1C)c3cc(c(s3)C)COc4ccc(cc4)F
ZINC08440858	5.2	0.41	-15.07	0	5	0	455.551	7	CCOC(=O)C1=C(N=C2CCCC(=O)C2[C@@H]1c3cc(c(s3)C)COc4ccc(cc4)F)C
ZINC08440858	5.38	-0.55	-12.78	0	5	0	455.551	7	CCOC(=O)C1[C@H](C2C(=NC1=C)CCCC2=O)c3cc(c(s3)C)COc4ccc(cc4)F
ZINC00702747	4.84	12.69	-46.07	0	7	-1	512.582	10	CCOc1ccc(cc1)C(=O)C2=C(C(=O)N([C@H]2c3ccc(cc3)OCc4ccccc4)C[C@@H]5CCCO5)[O-]
ZINC00702747	5.28	-1.09	-31.65	1	7	0	513.59	9	CCOc1ccc(cc1)C(=C2[C@@H](N(C(=O)C2=O)C[C@@H]3CCCO3)c4ccc(cc4)OCc5ccccc5)O
ZINC00702747	4.25	-0.87	-23.05	0	7	0	513.59	10	CCOc1ccc(cc1)C(=O)[C@H]2[C@@H](N(C(=O)C2=O)C[C@@H]3CCCO3)c4ccc(cc4)OCc5ccccc5
ZINC00702748	4.84	12.36	-45.54	0	7	-1	512.582	10	CCOc1ccc(cc1)C(=O)C2=C(C(=O)N([C@@H]2c3ccc(cc3)OCc4ccccc4)C[C@@H]5CCCO5)[O-]
ZINC00702748	5.28	-1.3	-32.28	1	7	0	513.59	9	CCOc1ccc(cc1)C(=C2[C@H](N(C(=O)C2=O)C[C@@H]3CCCO3)c4ccc(cc4)OCc5ccccc5)O
ZINC00702748	4.25	-1.03	-26.53	0	7	0	513.59	10	CCOc1ccc(cc1)C(=O)[C@H]2[C@H](N(C(=O)C2=O)C[C@@H]3CCCO3)c4ccc(cc4)OCc5ccccc5
ZINC08440856	3.58	1.27	-14.72	0	6	0	445.294	4	CC(=O)Oc1cc(c(cc1OC)/C=c\2/c(=O)n3c4ccccc4nc3s2)Br
ZINC08440855	7.97	-0.38	-7.85	1	3	0	661.321	4	CC1(CC2=C([C@H](Nc3c2c4ccccc4cc3)c5cc(cc(c5OCC=C)I)I)C(=O)C1)C
ZINC08440854	6.25	1.89	-11.86	0	6	0	489.612	8	CCOC(=O)C1[C@@H](C2=C(CCCC2=O)N=C1C)c3ccc(c(c3)OC)OCc4c(cc(cc4C)C)C
ZINC08440854	6.25	1.54	-16.15	0	6	0	489.612	8	CCOC(=O)C1=C(N=C2CCCC(=O)C2[C@H]1c3ccc(c(c3)OC)OCc4c(cc(cc4C)C)C)C
ZINC08440854	6.43	0.62	-12.97	0	6	0	489.612	8	CCOC(=O)C1[C@@H](C2C(=NC1=C)CCCC2=O)c3ccc(c(c3)OC)OCc4c(cc(cc4C)C)C
ZINC08440853	7.97	0.22	-7.26	1	3	0	661.321	4	CC1(CC2=C([C@@H](Nc3c2c4ccccc4cc3)c5cc(cc(c5OCC=C)I)I)C(=O)C1)C

Supplementary Table S2:
Detailed table of XP Docking using Schrodinger's Glide module

ZINC_ID	xLogP	ApolarDesolvation	PolarDesolvation	H Donors	H Acceptors	Charge	MolecularWeight	RotatableBonds	SMILES
ZINC08440852	5.21	-1.73	-16.09	1	6	0	548.433	8	c1ccc(cc1)COc2ccc(cc2)[C@H]3C(=C(C(=O)N3C[C@H]4CCCO4)O)C(=O)c5ccc(cc5)Br
ZINC08440852	4.63	-1.84	-25.25	0	6	0	548.433	8	c1ccc(cc1)COc2ccc(cc2)[C@H]3[C@H](C(=O)C(=O)N3C[C@H]4CCCO4)C(=O)c5ccc(cc5)Br
ZINC02063377	6.85	1.84	-13.79	1	6	0	468.944	5	Cc1ccc(c(c1)C)n2nc3ccc(cc3n2)NC(=O)/C=C/c4ccc(o4)c5ccc(cc5)Cl
ZINC00850295	6.26	1.57	-13.39	1	6	0	454.917	5	Cc1ccc(cc1)n2nc3ccc(cc3n2)NC(=O)/C=C/c4ccc(o4)c5ccc(cc5)Cl
ZINC02063375	6.94	-0.29	-18.86	1	6	0	464.521	7	Cc1cc(c2c(c1)nc(o2)c3cccc(c3)NC(=O)C(Oc4ccccc4)Oc5ccccc5)C
ZINC08440851	5.21	-1.6	-14.67	1	6	0	548.433	8	c1ccc(cc1)COc2ccc(cc2)[C@@H]3C(=C(C(=O)N3C[C@H]4CCCO4)O)C(=O)c5ccc(cc5)Br
ZINC08440851	4.63	-1.76	-21.33	0	6	0	548.433	8	c1ccc(cc1)COc2ccc(cc2)[C@@H]3[C@H](C(=O)C(=O)N3C[C@H]4CCCO4)C(=O)c5ccc(cc5)Br
ZINC02063374	6.9	-0.56	-18.69	1	4	0	453.361	4	Cc1ccc(cc1NC(=O)c2cc(ccc2OC)Br)c3nc4ccccc4s3
ZINC02063373	6.81	-0.64	-13.48	1	5	0	452.535	7	c1ccc(cc1)OC(C(=O)Nc2ccc(cc2)c3nc4ccccc4s3)Oc5ccccc5
ZINC08440850	6.25	1.93	-11.74	0	6	0	489.612	8	CCOC(=O)C1[C@H](C2=C(CCCC2=O)N=C1C)c3ccc(c(c3)OC)OCc4c(cc(cc4C)C)C
ZINC08440850	6.25	1.73	-15.28	0	6	0	489.612	8	CCOC(=O)C1=C(N=C2CCCC(=O)C2[C@@H]1c3ccc(c(c3)OC)OCc4c(cc(cc4C)C)C)C
ZINC08440850	6.43	0.87	-13.32	0	6	0	489.612	8	CCOC(=O)C1[C@H](C2C(=NC1=C)CCCC2=O)c3ccc(c(c3)OC)OCc4c(cc(cc4C)C)C
ZINC08440849	3.86	-1.76	-12.96	1	6	0	447.31	4	COc1ccc(cc1Br)c2csc(n2)NC(=O)c3ccc4c(c3)OCCO4
ZINC08440848	5.21	-1.52	-14.83	1	6	0	548.433	8	c1ccc(cc1)COc2ccc(cc2)[C@H]3C(=C(C(=O)N3C[C@@H]4CCCO4)O)C(=O)c5ccc(cc5)Br
ZINC08440848	4.63	-1.68	-21.43	0	6	0	548.433	8	c1ccc(cc1)COc2ccc(cc2)[C@H]3[C@@H](C(=O)C(=O)N3C[C@@H]4CCCO4)C(=O)c5ccc(cc5)Br
ZINC08440847	7.14	-3.68	-12.6	2	5	0	610.189	3	Cc1cc(c2c(c1)nc(o2)c3ccc(cc3O)NC(=O)c4cc(ccc4I)I)C
ZINC02063370	6.13	7.32	-14.07	2	5	0	484.293	3	Cc1cc2c(cc1C)oc(n2)c3ccc(cc3O)NC(=O)c4ccc(cc4)I
ZINC08440846	5.12	10.83	-9.92	3	6	0	524.422	3	c1ccc(cc1)CC[C@H]2CC=C3[C@H](C2)[C@@]4(c5cc(ccc5NC4=O)Br)C(C(=C3C#N)N)(C#N)C#N
ZINC08440846	5.21	0.26	-34.44	3	6	1	525.43	3	c1ccc(cc1)CC[C@H]2CC=C3[C@H](C2)[C@@]4(c5cc(ccc5NC4=O)Br)C(C(=[NH2+])C3C#N)(C#N)C#N
ZINC08440845	5.21	-1.75	-15.78	1	6	0	548.433	8	c1ccc(cc1)COc2ccc(cc2)[C@@H]3C(=C(C(=O)N3C[C@@H]4CCCO4)O)C(=O)c5ccc(cc5)Br
ZINC08440845	4.63	-1.85	-24.84	0	6	0	548.433	8	c1ccc(cc1)COc2ccc(cc2)[C@@H]3[C@@H](C(=O)C(=O)N3C[C@@H]4CCCO4)C(=O)c5ccc(cc5)Br
ZINC08440844	5.12	10.88	-10.31	3	6	0	524.422	3	c1ccc(cc1)CC[C@@H]2CC=C3[C@H](C2)[C@@]4(c5cc(ccc5NC4=O)Br)C(C(=C3C#N)N)(C#N)C#N
ZINC08440844	5.21	0.03	-37.84	3	6	1	525.43	3	c1ccc(cc1)CC[C@@H]2CC=C3[C@H](C2)[C@@]4(c5cc(ccc5NC4=O)Br)C(C(=[NH2+])C3C#N)(C#N)C#N
ZINC00702741	5.08	13.56	-53.6	0	6	-1	502.974	8	c1ccc(cc1)COc2ccc(cc2)[C@H]3C(=C(C(=O)N3C[C@H]4CCCO4)[O-])C(=O)c5ccc(cc5)Cl
ZINC00702741	5.53	-1.49	-30.86	1	6	0	503.982	7	c1ccc(cc1)COc2ccc(cc2)[C@H]3C(=C(c4ccc(cc4)Cl)O)C(=O)C(=O)N3C[C@H]5CCCO5
ZINC00702741	4.5	-1.23	-25.46	0	6	0	503.982	8	c1ccc(cc1)COc2ccc(cc2)[C@H]3[C@H](C(=O)C(=O)N3C[C@H]4CCCO4)C(=O)c5ccc(cc5)Cl
ZINC08440843	5.12	10.79	-10.78	3	6	0	524.422	3	c1ccc(cc1)CC[C@H]2CC=C3[C@@H](C2)[C@@]4(c5cc(ccc5NC4=O)Br)C(C(=C3C#N)N)(C#N)C#N
ZINC08440843	5.21	0.12	-41.91	3	6	1	525.43	3	c1ccc(cc1)CC[C@H]2CC=C3[C@@H](C2)[C@@]4(c5cc(ccc5NC4=O)Br)C(C(=[NH2+])C3C#N)(C#N)C#N
ZINC08440842	6.21	13.78	-17.12	1	5	0	510.428	6	Cc1cc(c(cc1COc2ccc(cc2)Br)[C@H]3C4=C(CCCC4=O)NC(=C3C(=O)OC)C)C
ZINC08440842	6.33	-1.02	-9.95	0	5	0	510.428	6	Cc1cc(c(cc1COc2ccc(cc2)Br)[C@H]3C4C(=O)CCC=C4N=C(C3C(=O)OC)C)C
ZINC08440841	5.12	8.48	-9.01	3	6	0	524.422	3	c1ccc(cc1)CC[C@@H]2CC=C3[C@@H](C2)[C@@]4(c5cc(ccc5NC4=O)Br)C(C(=C3C#N)N)(C#N)C#N
ZINC08440841	5.21	-0.44	-39.49	3	6	1	525.43	3	c1ccc(cc1)CC[C@@H]2CC=C3[C@@H](C2)[C@@]4(c5cc(ccc5NC4=O)Br)C(C(=[NH2+])C3C#N)(C#N)C#N
ZINC08440840	2.38	6.93	-12.18	2	8	0	428.857	5	COc1cc(ccc1N/C=C/2\C(=O)NC(=O)N(C2=O)c3ccc(cc3)Cl)SC#N
ZINC08440840	2.38	6.93	-12.15	2	8	0	428.857	5	COc1cc(ccc1N/C=C\2/C(=O)NC(=O)N(C2=O)c3ccc(cc3)Cl)SC#N
ZINC00702742	5.08	13.78	-54.41	0	6	-1	502.974	8	c1ccc(cc1)COc2ccc(cc2)[C@@H]3C(=C(C(=O)N3C[C@H]4CCCO4)[O-])C(=O)c5ccc(cc5)Cl
ZINC00702742	5.53	-1.37	-31.26	1	6	0	503.982	7	c1ccc(cc1)COc2ccc(cc2)[C@@H]3C(=C(c4ccc(cc4)Cl)O)C(=O)C(=O)N3C[C@H]5CCCO5
ZINC00702742	4.5	-1.14	-21.52	0	6	0	503.982	8	c1ccc(cc1)COc2ccc(cc2)[C@@H]3[C@H](C(=O)C(=O)N3C[C@H]4CCCO4)C(=O)c5ccc(cc5)Cl
ZINC08440837	0.32	5.84	-15.99	2	9	0	420.425	4	c1ccc(cc1)N2C(=O)/C(=C/Nc3ccc(cc3)C(=O)N4CCOCC4)/C(=O)NC2=O
ZINC00070425	4.49	8.28	-9.71	2	4	0	334.504	4	C1CCC(CC1)C(=O)N[C@H]2CCCC[C@@H]2NC(=O)C3CCCCC3
ZINC08440836	6.21	13.79	-17.08	1	5	0	510.428	6	Cc1cc(c(cc1COc2ccc(cc2)Br)[C@@H]3C4=C(CCCC4=O)NC(=C3C(=O)OC)C)C
ZINC08440836	6.33	-0.81	-9.12	0	5	0	510.428	6	Cc1cc(c(cc1COc2ccc(cc2)Br)[C@@H]3C4C(=O)CCC=C4N=C(C3C(=O)OC)C)C
ZINC00702743	5.08	13.43	-58.57	0	6	-1	502.974	8	c1ccc(cc1)COc2ccc(cc2)[C@H]3C(=C(C(=O)N3C[C@@H]4CCCO4)[O-])C(=O)c5ccc(cc5)Cl
ZINC00702743	5.53	-1.29	-31	1	6	0	503.982	7	c1ccc(cc1)COc2ccc(cc2)[C@H]3C(=C(c4ccc(cc4)Cl)O)C(=O)C(=O)N3C[C@@H]5CCCO5
ZINC00702743	4.5	-1.01	-23.27	0	6	0	503.982	8	c1ccc(cc1)COc2ccc(cc2)[C@H]3[C@H](C(=O)C(=O)N3C[C@@H]4CCCO4)C(=O)c5ccc(cc5)Cl
ZINC08440835	4.02	0.98	-21.11	1	6	0	424.403	7	c1cc2cccc3c2c(c1)C(=O)N(C3=O)CCCC(=O)Nc4ccc(cc4)OC(F)F
ZINC00702744	5.08	13.05	-57.94	0	6	-1	502.974	8	c1ccc(cc1)COc2ccc(cc2)[C@@H]3C(=C(C(=O)N3C[C@@H]4CCCO4)[O-])C(=O)c5ccc(cc5)Cl
ZINC00702744	5.53	-1.5	-31.57	1	6	0	503.982	7	c1ccc(cc1)COc2ccc(cc2)[C@@H]3C(=C(c4ccc(cc4)Cl)O)C(=O)C(=O)N3C[C@@H]5CCCO5
ZINC00702744	4.5	-1.23	-25.04	0	6	0	503.982	8	c1ccc(cc1)COc2ccc(cc2)[C@@H]3[C@@H](C(=O)C(=O)N3C[C@@H]4CCCO4)C(=O)c5ccc(cc5)Cl
ZINC08440834	3.22	-2.92	-53.13	1	10	1	509.564	6	Cc1ccc(cc1[N+](=O)[O-])S(=O)(=O)N2CC[NH+](CC2)CCN3C(=O)c4cccc5c4c(ccc5)C3=O
ZINC08440833	4.8	-0.36	-17.92	1	6	0	610.193	3	Cc1c(c(n(n1)c2ccc(cc2)I)O)/C=C\3/C(=NN(C3=O)c4ccc(cc4)I)C
ZINC08440831	5.49	0.63	-12.89	1	6	0	544.401	8	c1ccc(cc1)COc2ccc(cc2)[C@H]3C(=C(C(=O)N3Cc4ccco4)O)C(=O)c5ccc(cc5)Br
ZINC08440831	4.91	0.46	-17.89	0	6	0	544.401	8	c1ccc(cc1)COc2ccc(cc2)[C@H]3[C@@H](C(=O)C(=O)N3Cc4ccco4)C(=O)c5ccc(cc5)Br
ZINC00848923	4.52	-0.29	-18.12	1	6	0	563.193	3	Cc1c(c(n(n1)c2ccc(cc2)Br)O)/C=C\3/C(=NN(C3=O)c4ccc(cc4)I)C
ZINC08440830	6.08	13.68	-17.18	1	5	0	465.977	6	Cc1cc(c(cc1COc2ccc(cc2)Cl)[C@H]3C4=C(CCCC4=O)NC(=C3C(=O)OC)C)C
ZINC08440830	6.2	-0.41	-10.04	0	5	0	465.977	6	Cc1cc(c(cc1COc2ccc(cc2)Cl)[C@H]3C4C(=O)CCC=C4N=C(C3C(=O)OC)C)C
ZINC08440829	6.21	1.19	-9.47	0	5	0	444.965	5	CCOC(=O)C1=C(N=c2n(c(=O)/c(=C\c3cccs3)/s2)[C@H]1c4ccc(cc4)Cl)C
ZINC08440828	6.21	1.08	-9.24	0	5	0	444.965	5	CCOC(=O)C1=C(N=c2n(c(=O)/c(=C\c3cccs3)/s2)[C@@H]1c4ccc(cc4)Cl)C
ZINC08440827	5.36	13.49	-56.67	0	6	-1	498.942	8	c1ccc(cc1)COc2ccc(cc2)[C@H]3C(=C(C(=O)N3Cc4ccco4)[O-])C(=O)c5ccc(cc5)Cl
ZINC08440827	5.81	0.86	-27.55	1	6	0	499.95	7	c1ccc(cc1)COc2ccc(cc2)[C@H]3C(=C(c4ccc(cc4)Cl)O)C(=O)C(=O)N3Cc5ccco5
ZINC08440827	4.78	1.15	-19.47	0	6	0	499.95	8	c1ccc(cc1)COc2ccc(cc2)[C@H]3[C@H](C(=O)C(=O)N3Cc4ccco4)C(=O)c5ccc(cc5)Cl
ZINC08440826	8.14	2.88	-15.6	0	6	0	539.44	6	CCOC(=O)C1=C(N=c2n(c(=O)/c(=C\c3ccc(o3)c4cccc(c4)Cl)/s2)[C@H]1c5ccc(cc5)Cl)C

Supplementary Table S2:
Detailed table of XP Docking using Schrodinger's Glide module

ZINC_ID	xLogP	ApolarDesolvation	PolarDesolvation	H Donors	H Acceptors	Charge	MolecularWeight	RotatableBonds	SMILES
ZINC08440825	8.14	2.89	-15.34	0	6	0	539.44	6	CCOC(=O)C1=C(N=c2n(c(=O)/c(=C\c3ccc(o3)c4cccc(c4)Cl)/s2)[C@@H]1c5ccc(cc5)Cl)C
ZINC08440824	8.77	2.72	-13.14	0	6	0	573.885	6	CCOC(=O)C1=C(N=c2n(c(=O)/c(=C\c3ccc(o3)c4cc(ccc4Cl)Cl)/s2)[C@H]1c5ccc(cc5)Cl)C
ZINC08440823	8.77	2.74	-13.37	0	6	0	573.885	6	CCOC(=O)C1=C(N=c2n(c(=O)/c(=C\c3ccc(o3)c4cc(ccc4Cl)Cl)/s2)[C@@H]1c5ccc(cc5)Cl)C
ZINC08440822	6.08	13.69	-17.21	1	5	0	465.977	6	Cc1cc(c(cc1COc2ccc(cc2)Cl)[C@@H]3C4=C(CCCC4=O)NC(=C3C(=O)OC)C)C
ZINC08440822	6.2	-0.2	-9.22	0	5	0	465.977	6	Cc1cc(c(cc1COc2ccc(cc2)Cl)[C@@H]3C4C(=O)CCC=C4N=C(C3C(=O)OC)C)C
ZINC00702738	5.36	14.22	-54.73	0	6	-1	498.942	8	c1ccc(cc1)COc2ccc(cc2)[C@@H]3C(=C(C(=O)N3Cc4ccco4)[O-])C(=O)c5ccc(cc5)Cl
ZINC00702738	5.81	0.46	-29.22	1	6	0	499.95	7	c1ccc(cc1)COc2ccc(cc2)[C@@H]3C(=C(c4ccc(cc4)Cl)O)C(=O)C(=O)N3Cc5ccco5
ZINC00702738	4.78	0.76	-23.97	0	6	0	499.95	8	c1ccc(cc1)COc2ccc(cc2)[C@@H]3[C@@H](C(=O)C(=O)N3Cc4ccco4)C(=O)c5ccc(cc5)Cl
ZINC02063313	7.52	3.15	-11.92	0	6	0	590.543	6	CCOC(=O)C1=C(N=c2n(c(=O)/c(=C/c3cc(n(c3C)c4ccc(cc4)Br)C)/s2)[C@H]1c5ccc(cc5)C)C
ZINC02063315	7.52	2.99	-11.85	0	6	0	590.543	6	CCOC(=O)C1=C(N=c2n(c(=O)/c(=C/c3cc(n(c3C)c4ccc(cc4)Br)C)/s2)[C@@H]1c5ccc(cc5)C)C
ZINC08440821	7.16	4.23	-11.95	0	6	0	525.674	6	CCOC(=O)C1=C(N=c2n(c(=O)/c(=C/c3cc(n(c3C)c4ccc(cc4)C)C)/s2)[C@H]1c5ccc(cc5)C)C
ZINC02063311	7.16	4.07	-11.85	0	6	0	525.674	6	CCOC(=O)C1=C(N=c2n(c(=O)/c(=C/c3cc(n(c3C)c4ccc(cc4)C)C)/s2)[C@@H]1c5ccc(cc5)C)C
ZINC00667142	4.61	0.63	-11.64	1	7	0	511.361	6	Cc1ccc(cc1)N2C(=O)/C(=C/c3ccc(c(c3)OC)OCc4ccc(cc4Cl)Cl)/C(=O)NC2=O
ZINC00647877	3.71	2.57	-22.86	3	6	0	475.474	4	c1ccc(cc1)c2c(c[nH]n2)[C@@H]3C(=C(N(C4=C3C(=O)CCC4)c5cccc(c5)C(F)(F)F)N)C#N
ZINC08440820	4.41	10.78	-56.37	0	6	-1	452.502	9	CCCCOc1ccc(cc1)[C@H]2C(=C(C(=O)N2C[C@H]3CCCO3)[O-])C(=O)c4ccc(cc4)F
ZINC08440820	4.86	-0.22	-25.47	1	6	0	453.51	8	CCCCOc1ccc(cc1)[C@H]2C(=C(c3ccc(cc3)F)O)C(=O)C(=O)N2C[C@H]4CCCO4
ZINC08440820	3.83	0	-15.37	0	6	0	453.51	9	CCCCOc1ccc(cc1)[C@H]2[C@@H](C(=O)C(=O)N2C[C@H]3CCCO3)C(=O)c4ccc(cc4)F
ZINC08440818	4.41	10.74	-58.46	0	6	-1	452.502	9	CCCCOc1ccc(cc1)[C@@H]2C(=C(C(=O)N2C[C@H]3CCCO3)[O-])C(=O)c4ccc(cc4)F
ZINC08440818	4.86	-0.44	-30.44	1	6	0	453.51	8	CCCCOc1ccc(cc1)[C@@H]2C(=C(c3ccc(cc3)F)O)C(=O)C(=O)N2C[C@H]4CCCO4
ZINC08440818	3.83	-0.14	-22.23	0	6	0	453.51	9	CCCCOc1ccc(cc1)[C@@H]2[C@@H](C(=O)C(=O)N2C[C@H]3CCCO3)C(=O)c4ccc(cc4)F
ZINC08440815	4.41	11.48	-55.71	0	6	-1	452.502	9	CCCCOc1ccc(cc1)[C@H]2C(=C(C(=O)N2C[C@@H]3CCCO3)[O-])C(=O)c4ccc(cc4)F
ZINC08440815	4.86	-0.36	-30.16	1	6	0	453.51	8	CCCCOc1ccc(cc1)[C@H]2C(=C(c3ccc(cc3)F)O)C(=O)C(=O)N2C[C@@H]4CCCO4
ZINC08440815	3.83	-0.07	-22.34	0	6	0	453.51	9	CCCCOc1ccc(cc1)[C@H]2[C@H](C(=O)C(=O)N2C[C@@H]3CCCO3)C(=O)c4ccc(cc4)F
ZINC08440813	4.3	11.93	-139.13	0	11	-2	499.387	4	c1ccc2c(c1)c(c(c(=O)o2)C(c3cc4c(cc3[N+](=O)[O-])OCO4)c5c(c6ccccc6oc5=O)[O-])[O-]
ZINC08440813	4.3	11.14	-63.38	1	11	-1	500.395	4	c1ccc2c(c1)c(c(c(=O)o2)[C@@H](c3cc4c(cc3[N+](=O)[O-])OCO4)c5c(c6ccccc6oc5=O)[O-])O
ZINC08440812	4.41	11.11	-56.4	0	6	-1	452.502	9	CCCCOc1ccc(cc1)[C@@H]2C(=C(C(=O)N2C[C@@H]3CCCO3)[O-])C(=O)c4ccc(cc4)F
ZINC08440812	4.86	-0.68	-31.75	1	6	0	453.51	8	CCCCOc1ccc(cc1)[C@@H]2C(=C(c3ccc(cc3)F)O)C(=O)C(=O)N2C[C@@H]4CCCO4
ZINC08440812	3.83	-0.39	-25.56	0	6	0	453.51	9	CCCCOc1ccc(cc1)[C@@H]2[C@@H](C(=O)C(=O)N2C[C@@H]3CCCO3)C(=O)c4ccc(cc4)F
ZINC02334044	5.11	-5.94	-15.71	3	9	0	457.362	11	CC(C)Nc1nc(nc(n1)OCCNC(=O)COc2ccc(cc2Cl)Cl)NC(C)C
ZINC08440810	5.21	12.18	-54.18	0	6	-1	464.925	9	CCCCOc1ccc(cc1)[C@H]2C(=C(C(=O)N2Cc3ccco3)[O-])C(=O)c4ccc(cc4)Cl
ZINC08440810	5.65	0.81	-26.76	1	6	0	465.933	8	CCCCOc1ccc(cc1)[C@H]2C(=C(c3ccc(cc3)Cl)O)C(=O)C(=O)N2Cc4ccco4
ZINC08440810	4.62	1.05	-16.91	0	6	0	465.933	9	CCCCOc1ccc(cc1)[C@H]2[C@@H](C(=O)C(=O)N2Cc3ccco3)C(=O)c4ccc(cc4)Cl
ZINC00667115	6.18	-0.16	-15.32	2	5	0	451.309	5	c1ccc(cc1)C(=O)Nc2ccc(cc2)NC(=O)c3ccc(o3)c4cc(ccc4Cl)Cl
ZINC00848995	6.01	10.6	-11.32	1	7	0	479.323	5	COc1ccc(cc1)n2nc3ccc(cc3n2)NC(=O)c4ccc(o4)c5cc(ccc5Cl)Cl
ZINC00848995	3.66	4.73	-18.97	3	7	0	403.438	5	COc1ccc(cc1)N2Cc3ccc(cc3C2)C(=O)N/N=C/c4ccc(cc4O)O
ZINC08440809	6.69	0.34	-13.33	1	5	0	483.015	4	[H]/N=C\1/[C@H](C2=CCCC[C@@H]2[C@@H](C1(C#N)C#N)c3c(cc(c(c3C)COc4ccc(cc4)Cl)C)C)C#N
ZINC08440809	6.69	0.17	-35.76	2	5	1	484.023	4	Cc1cc(c(c(c1COc2ccc(cc2)Cl)C)[C@H]3[C@H]4CCCC=C4[C@@H](C(=[NH2+])C3(C#N)C#N)C#N)C
ZINC00848993	6.08	9.23	-20.88	1	6	0	450.281	4	c1cc(cc(c1)NC(=O)c2ccc(o2)c3cc(ccc3Cl)Cl)c4nc5c(o4)cccn5
ZINC08440808	5.21	12.49	-51.45	0	6	-1	464.925	9	CCCCOc1ccc(cc1)[C@@H]2C(=C(C(=O)N2Cc3ccco3)[O-])C(=O)c4ccc(cc4)Cl
ZINC08440808	5.65	0.5	-26.97	1	6	0	465.933	8	CCCCOc1ccc(cc1)[C@@H]2C(=C(c3ccc(cc3)Cl)O)C(=O)C(=O)N2Cc4ccco4
ZINC08440808	4.62	0.8	-21.54	0	6	0	465.933	9	CCCCOc1ccc(cc1)[C@@H]2[C@@H](C(=O)C(=O)N2Cc3ccco3)C(=O)c4ccc(cc4)Cl
ZINC02063292	7.4	-0.86	-14.85	1	5	0	463.32	4	Cc1ccc2c(c1)nc(o2)c3cccc(c3)NC(=O)c4ccc(o4)c5cc(ccc5Cl)Cl
ZINC02063291	6.97	-1.18	-15.19	1	5	0	449.293	4	c1ccc2c(c1)nc(o2)c3cccc(c3)NC(=O)c4ccc(o4)c5cc(ccc5Cl)Cl
ZINC08440807	6.69	2.65	-12.29	1	5	0	483.015	4	[H]/N=C/1\[C@H](C2=CCCC[C@@H]2[C@H](C1(C#N)C#N)c3c(cc(c(c3C)COc4ccc(cc4)Cl)C)C)C#N
ZINC08440807	6.69	2.48	-42.55	2	5	1	484.023	4	Cc1cc(c(c(c1COc2ccc(cc2)Cl)C)[C@@H]3[C@H]4CCCC=C4[C@@H](C(=[NH2+])C3(C#N)C#N)C#N)C
ZINC08440806	4.01	10.43	-60.34	0	7	-1	434.468	9	CCCCOc1ccc(cc1)[C@H]2C(=C(C(=O)N2Cc3ccco3)[O-])C(=O)c4ccc(o4)C
ZINC08440806	4.01	9.68	-13.27	1	7	0	435.476	9	CCCCOc1ccc(cc1)[C@H]2C(=C(C(=O)N2Cc3ccco3)O)C(=O)c4ccc(o4)C
ZINC00848992	6.01	-0.09	-8.51	1	4	0	415.301	4	c1ccc(cc1)c2csc(n2)NC(=O)c3ccc(o3)c4cc(ccc4Cl)Cl
ZINC08440805	6.69	-0.94	-12.71	1	5	0	483.015	4	[H]/N=C\1/[C@H](C2=CCCC[C@H]2[C@@H](C1(C#N)C#N)c3c(cc(c(c3C)COc4ccc(cc4)Cl)C)C)C#N
ZINC08440805	6.69	-1.12	-43.49	2	5	1	484.023	4	Cc1cc(c(c(c1COc2ccc(cc2)Cl)C)[C@H]3[C@@H]4CCCC=C4[C@@H](C(=[NH2+])C3(C#N)C#N)C#N)C
ZINC08440803	4.01	10.16	-60.9	0	7	-1	434.468	9	CCCCOc1ccc(cc1)[C@@H]2C(=C(C(=O)N2Cc3ccco3)[O-])C(=O)c4ccc(o4)C
ZINC08440803	4.01	9.41	-13.93	1	7	0	435.476	9	CCCCOc1ccc(cc1)[C@@H]2C(=C(C(=O)N2Cc3ccco3)O)C(=O)c4ccc(o4)C
ZINC08440802	7.9	-2.38	-13.22	1	8	0	729.957	6	c1cc2c(cc1NC(=O)c3ccc(o3)Br)nc(o2)c4cc(c(c(c4)Br)OC(=O)c5ccc(o5)Br)Br
ZINC00848910	5.64	0.3	-15.15	1	5	0	424.891	3	c1cc(cc(c1)Cl)n2nc3ccc(cc3n2)NC(=O)c4ccc5c6c4cccc6CC5
ZINC00848909	5.21	0.47	-15.18	1	6	0	434.499	5	CCOc1ccc(cc1)n2nc3ccc(cc3n2)NC(=O)c4ccc5c6c4cccc6CC5
ZINC02063284	7.08	-1.09	-13.66	1	4	0	459.332	3	c1cc2c3c(ccc(c3c1)C(=O)Nc4ccc5c(c4)nc(o5)c6cc(ccc6Cl)Cl)CC2
ZINC08440801	4.43	0.53	-12.75	1	6	0	437.517	9	CCCCOc1ccc(cc1)[C@H]2C(=C(C(=O)N2Cc3ccco3)O)C(=O)c4cccs4
ZINC08440801	3.84	0.36	-16.97	0	6	0	437.517	9	CCCCOc1ccc(cc1)[C@H]2[C@@H](C(=O)C(=O)N2Cc3ccco3)C(=O)c4cccs4
ZINC08440799	6.69	-2.53	-14.3	1	5	0	483.015	4	[H]/N=C\1/[C@H](C2=CCCC[C@H]2[C@H](C1(C#N)C#N)c3c(cc(c(c3C)COc4ccc(cc4)Cl)C)C)C#N
ZINC08440799	6.69	-1.66	-40.39	2	5	1	484.023	4	Cc1cc(c(c(c1COc2ccc(cc2)Cl)C)[C@@H]3[C@@H]4CCCC=C4[C@@H](C(=[NH2+])C3(C#N)C#N)C#N)C
ZINC02063276	6.77	2.83	-15.28	0	5	0	471.56	5	Cc1ccccc1c2c3cc(c(cc3c4c(nn(c4n2)c5ccccc5)c6ccccc6)OC)OC

Supplementary Table S2:
Detailed table of XP Docking using Schrodinger's Glide module

ZINC_ID	xLogP	ApolarDesolvation	PolarDesolvation	H Donors	H Acceptors	Charge	MolecularWeight	RotatableBonds	SMILES
ZINC08440798	7.47	-1.18	-13.89	1	6	0	588.926	8	CCOc1cc(cc(c1O)Br)[C@H]2C3C(=NC(=C2C(=O)OCC(C)C)C)C[C@H](CC3=O)c4ccc(cc4)Cl
ZINC08440798	7.63	-2.41	-8.72	1	6	0	588.926	8	CCOc1cc(cc(c1O)Br)[C@H]2C3C(=O)C[C@@H](C=C3N=C(C2C(=O)OCC(C)C)C)c4ccc(cc4)Cl
ZINC08440797	4.43	0.16	-13.84	1	6	0	437.517	9	CCCCOc1ccc(cc1)[C@@H]2C(=C(C(=O)N2Cc3ccco3)O)C(=O)c4cccs4
ZINC08440797	3.84	0.03	-20.6	0	6	0	437.517	9	CCCCOc1ccc(cc1)[C@@H]2[C@@H](C(=O)C(=O)N2Cc3ccco3)C(=O)c4cccs4
ZINC08440796	7.47	-1.12	-13.22	1	6	0	588.926	8	CCOc1cc(cc(c1O)Br)[C@@H]2C3C(=NC(=C2C(=O)OCC(C)C)C)C[C@H](CC3=O)c4ccc(cc4)Cl
ZINC08440796	7.63	-2.31	-9.75	1	6	0	588.926	8	CCOc1cc(cc(c1O)Br)[C@@H]2C3C(=O)C[C@@H](C=C3N=C(C2C(=O)OCC(C)C)C)c4ccc(cc4)Cl
ZINC08440795	7.47	-1.09	-13.93	1	6	0	588.926	8	CCOc1cc(cc(c1O)Br)[C@H]2C3C(=NC(=C2C(=O)OCC(C)C)C)C[C@@H](CC3=O)c4ccc(cc4)Cl
ZINC08440795	7.63	-2.32	-9.58	1	6	0	588.926	8	CCOc1cc(cc(c1O)Br)[C@H]2C3C(=O)C[C@H](C=C3N=C(C2C(=O)OCC(C)C)C)c4ccc(cc4)Cl
ZINC08440794	7.47	-1.24	-14.53	1	6	0	588.926	8	CCOc1cc(cc(c1O)Br)[C@@H]2C3C(=NC(=C2C(=O)OCC(C)C)C)C[C@@H](CC3=O)c4ccc(cc4)Cl
ZINC08440794	7.63	-2.32	-9.64	1	6	0	588.926	8	CCOc1cc(cc(c1O)Br)[C@@H]2C3C(=O)C[C@H](C=C3N=C(C2C(=O)OCC(C)C)C)c4ccc(cc4)Cl
ZINC05921386	4.75	11.27	-37.37	0	6	-1	471.316	7	CCOc1ccc(cc1)C(=O)C2=C(C(=O)N([C@H]2c3ccc(c(c3)Cl)Cl)Cc4ccco4)[O-]
ZINC05921386	5.2	0.51	-28.52	1	6	0	472.324	6	CCOc1ccc(cc1)C(=C2[C@@H](N(C(=O)C2=O)Cc3ccco3)c4ccc(c(c4)Cl)Cl)O
ZINC05921386	4.17	0.74	-20.21	0	6	0	472.324	7	CCOc1ccc(cc1)C(=O)[C@H]2[C@@H](N(C(=O)C2=O)Cc3ccco3)c4ccc(c(c4)Cl)Cl
ZINC08440793	7.11	1.6	-13.69	0	6	0	537.656	10	CCCOc1ccc(cc1OC)[C@H]2C3C(=NC(=C2C(=O)OCc4ccccc4)C)C[C@H](CC3=O)c5ccccc5
ZINC08440793	7.27	0.21	-9.32	0	6	0	537.656	10	CCCOc1ccc(cc1OC)[C@H]2C3C(=O)C[C@@H](C=C3N=C(C2C(=O)OCc4ccccc4)C)c5ccccc5
ZINC08440792	7.11	1.53	-13.43	0	6	0	537.656	10	CCCOc1ccc(cc1OC)[C@@H]2C3C(=NC(=C2C(=O)OCc4ccccc4)C)C[C@H](CC3=O)c5ccccc5
ZINC08440792	7.27	0.14	-9.27	0	6	0	537.656	10	CCCOc1ccc(cc1OC)[C@@H]2C3C(=O)C[C@@H](C=C3N=C(C2C(=O)OCc4ccccc4)C)c5ccccc5
ZINC08440791	6.16	14.04	-19.13	1	5	0	530.484	6	Cc1c(cc(s1)[C@H]2C3=C(CC(CC3=O)(C)C)NC(=C2C(=O)OC)C)COc4ccc(cc4)Br
ZINC08440791	6.28	-1.48	-10.03	0	5	0	530.484	6	Cc1c(cc(s1)[C@H]2C3C(=O)CC(C=C3N=C(C2C(=O)OC)C)(C)C)COc4ccc(cc4)Br
ZINC00702733	3.63	9.68	-38.97	0	6	-1	420.416	7	CCOc1ccc(cc1)C(=O)C2=C(C(=O)N([C@H]2c3ccc(cc3)F)Cc4ccco4)[O-]
ZINC00702733	4.08	1.61	-27.08	1	6	0	421.424	6	CCOc1ccc(cc1)C(=C2[C@@H](N(C(=O)C2=O)Cc3ccco3)c4ccc(cc4)F)O
ZINC00702733	3.05	1.9	-19.99	0	6	0	421.424	7	CCOc1ccc(cc1)C(=O)[C@@H]2[C@@H](N(C(=O)C2=O)Cc3ccco3)c4ccc(cc4)F
ZINC08440790	7.11	1.54	-13.38	0	6	0	537.656	10	CCCOc1ccc(cc1OC)[C@H]2C3C(=NC(=C2C(=O)OCc4ccccc4)C)C[C@@H](CC3=O)c5ccccc5
ZINC08440790	7.27	0.01	-10.24	0	6	0	537.656	10	CCCOc1ccc(cc1OC)[C@H]2C3C(=O)C[C@H](C=C3N=C(C2C(=O)OCc4ccccc4)C)c5ccccc5
ZINC08440789	7.11	1.38	-15.42	0	6	0	537.656	10	CCCOc1ccc(cc1OC)[C@@H]2C3C(=NC(=C2C(=O)OCc4ccccc4)C)C[C@@H](CC3=O)c5ccccc5
ZINC08440789	7.27	0.3	-11.6	0	6	0	537.656	10	CCCOc1ccc(cc1OC)[C@@H]2C3C(=O)C[C@H](C=C3N=C(C2C(=O)OCc4ccccc4)C)c5ccccc5
ZINC00702734	3.63	9.98	-37.53	0	6	-1	420.416	7	CCOc1ccc(cc1)C(=O)C2=C(C(=O)N([C@@H]2c3ccc(cc3)F)Cc4ccco4)[O-]
ZINC00702734	4.08	1.24	-28.4	1	6	0	421.424	6	CCOc1ccc(cc1)C(=C2[C@H](N(C(=O)C2=O)Cc3ccco3)c4ccc(cc4)F)O
ZINC00702734	3.05	1.5	-24.15	0	6	0	421.424	7	CCOc1ccc(cc1)C(=O)[C@H]2[C@H](N(C(=O)C2=O)Cc3ccco3)c4ccc(cc4)F
ZINC04068118	8.04	0.79	-8.81	0	5	0	533.668	8	CC1=NC2=C([C@@H](C1C(=O)OC3CCCC3)c4ccc(cc4)OCc5ccccc5)C(=O)C[C@@H](C2)c6ccccc6
ZINC04068118	8.04	0.91	-11.75	0	5	0	533.668	8	CC1=C([C@H]([C@H]2C(=N1)C[C@H](CC2=O)c3ccccc3)c4ccc(cc4)OCc5ccccc5)C(=O)OC6CCCC6
ZINC04068118	8.22	2.24	-16.46	0	5	0	533.668	8	C=C1C([C@H]([C@@H]2C(=N1)C[C@H](CC2=O)c3ccccc3)c4ccc(cc4)OCc5ccccc5)C(=O)OC6CCCC6
ZINC04068118	8.22	1.02	-9.66	0	5	0	533.668	8	C=C1C([C@H](C2C(=N1)C[C@H](CC2=O)c3ccccc3)c4ccc(cc4)OCc5ccccc5)C(=O)OC6CCCC6
ZINC04068116	8.04	0.8	-8.69	0	5	0	533.668	8	CC1=NC2=C([C@@H](C1C(=O)OC3CCCC3)c4ccc(cc4)OCc5ccccc5)C(=O)C[C@H](C2)c6ccccc6
ZINC04068116	8.04	0.83	-11.87	0	5	0	533.668	8	CC1=C([C@H]([C@H]2C(=N1)C[C@@H](CC2=O)c3ccccc3)c4ccc(cc4)OCc5ccccc5)C(=O)OC6CCCC6
ZINC04068116	8.22	2.02	-9.23	0	5	0	533.668	8	C=C1C([C@H]([C@@H]2C(=N1)C[C@@H](CC2=O)c3ccccc3)c4ccc(cc4)OCc5ccccc5)C(=O)OC6CCCC6
ZINC04068116	8.22	0.95	-9.85	0	5	0	533.668	8	C=C1C([C@H](C2C(=N1)C[C@@H](CC2=O)c3ccccc3)c4ccc(cc4)OCc5ccccc5)C(=O)OC6CCCC6
ZINC00702729	3.99	10.13	-40.86	0	6	-1	485.354	7	CCOc1ccc(cc1)C(=O)C2=C(C(=O)N([C@H]2c3ccc(cc3)Br)C[C@H]4CCCO4)[O-]
ZINC00702729	4.44	-1.9	-25.06	1	6	0	486.362	6	CCOc1ccc(cc1)C(=C2[C@@H](N(C(=O)C2=O)C[C@H]3CCCO3)c4ccc(cc4)Br)O
ZINC00702729	3.41	-1.61	-17.52	0	6	0	486.362	7	CCOc1ccc(cc1)C(=O)[C@@H]2[C@@H](N(C(=O)C2=O)C[C@H]3CCCO3)c4ccc(cc4)Br
ZINC08440788	6.16	14.03	-19.11	1	5	0	530.484	6	Cc1c(cc(s1)[C@@H]2C3=C(CC(CC3=O)(C)C)NC(=C2C(=O)OC)C)COc4ccc(cc4)Br
ZINC08440788	6.28	-1.5	-10.17	0	5	0	530.484	6	Cc1c(cc(s1)[C@@H]2C3C(=O)CC(C=C3N=C(C2C(=O)OC)C)(C)C)COc4ccc(cc4)Br
ZINC04068115	8.04	0.78	-9.04	0	5	0	533.668	8	CC1=NC2=C([C@H](C1C(=O)OC3CCCC3)c4ccc(cc4)OCc5ccccc5)C(=O)C[C@H](C2)c6ccccc6
ZINC04068115	8.04	0.72	-12.46	0	5	0	533.668	8	CC1=C([C@@H]([C@H]2C(=N1)C[C@@H](CC2=O)c3ccccc3)c4ccc(cc4)OCc5ccccc5)C(=O)OC6CCCC6
ZINC04068115	8.22	2.26	-15.6	0	5	0	533.668	8	C=C1C([C@@H]([C@H]2C(=N1)C[C@@H](CC2=O)c3ccccc3)c4ccc(cc4)OCc5ccccc5)C(=O)OC6CCCC6
ZINC04068115	8.22	0.84	-10.28	0	5	0	533.668	8	C=C1C([C@@H](C2C(=N1)C[C@@H](CC2=O)c3ccccc3)c4ccc(cc4)OCc5ccccc5)C(=O)OC6CCCC6
ZINC08440787	6	0.04	-11.39	1	7	0	505.611	9	CC[C@H](C)OC(=O)C1[C@@H](C2=C(C[C@H](CC2=O)c3ccc(cc3)OC)N=C1C)c4ccc(c(c4)OCC)O
ZINC08440787	6	-0.24	-14.95	1	7	0	505.611	9	CC[C@H](C)OC(=O)C1=C(N=C2C[C@H](CC(=O)C2[C@H]1c3ccc(c(c3)OCC)O)c4ccc(cc4)OC)C
ZINC08440787	6.19	-1.29	-12.64	1	7	0	505.611	9	CC[C@H](C)OC(=O)C1[C@@H](C2C(=NC1=C)C[C@H](CC2=O)c3ccc(cc3)OC)c4ccc(c(c4)OCC)O
ZINC08440786	6	-0.01	-14.82	1	7	0	505.611	9	CC[C@@H](C)OC(=O)C1[C@@H](C2=C(C[C@H](CC2=O)c3ccc(cc3)OC)N=C1C)c4ccc(c(c4)OCC)O
ZINC08440786	6	-0.26	-16.14	1	7	0	505.611	9	CC[C@@H](C)OC(=O)C1=C(N=C2C[C@H](CC(=O)C2[C@H]1c3ccc(c(c3)OCC)O)c4ccc(cc4)OC)C
ZINC08440786	6.19	-1.13	-14.55	1	7	0	505.611	9	CC[C@@H](C)OC(=O)C1[C@@H](C2C(=NC1=C)C[C@H](CC2=O)c3ccc(cc3)OC)c4ccc(c(c4)OCC)O
ZINC08440785	6	0.04	-11.13	1	7	0	505.611	9	CC[C@H](C)OC(=O)C1[C@H](C2=C(C[C@H](CC2=O)c3ccc(cc3)OC)N=C1C)c4ccc(c(c4)OCC)O
ZINC08440785	6	-0.32	-15.39	1	7	0	505.611	9	CC[C@H](C)OC(=O)C1=C(N=C2C[C@H](CC(=O)C2[C@@H]1c3ccc(c(c3)OCC)O)c4ccc(cc4)OC)C
ZINC08440785	6.19	-1.38	-12.77	1	7	0	505.611	9	CC[C@H](C)OC(=O)C1[C@H](C2C(=NC1=C)C[C@H](CC2=O)c3ccc(cc3)OC)c4ccc(c(c4)OCC)O
ZINC00702730	3.99	9.93	-42.07	0	6	-1	485.354	7	CCOc1ccc(cc1)C(=O)C2=C(C(=O)N([C@@H]2c3ccc(cc3)Br)C[C@H]4CCCO4)[O-]
ZINC00702730	4.44	-2.11	-30.33	1	6	0	486.362	6	CCOc1ccc(cc1)C(=C2[C@H](N(C(=O)C2=O)C[C@H]3CCCO3)c4ccc(cc4)Br)O
ZINC00702730	3.41	-1.82	-23.18	0	6	0	486.362	7	CCOc1ccc(cc1)C(=O)[C@H]2[C@H](N(C(=O)C2=O)C[C@H]3CCCO3)c4ccc(cc4)Br
ZINC08440784	6.14	3.86	-14.92	1	6	0	478.596	5	[H]/N=C/1\[C@H](C2=CCCC[C@@H]2[C@@H](C1(C#N)C#N)c3ccc(c(c3)OC)OCc4c(cc(cc4C)C)C)C#N
ZINC08440784	6.14	3.69	-43.05	2	6	1	479.604	5	Cc1cc(c(c(c1)C)COc2ccc(cc2OC)[C@H]3[C@H]4CCCC=C4[C@@H](C(=[NH2+])C3(C#N)C#N)C#N)C
ZINC08440783	6	0.05	-11.22	1	7	0	505.611	9	CC[C@@H](C)OC(=O)C1[C@H](C2=C(C[C@H](CC2=O)c3ccc(cc3)OC)N=C1C)c4ccc(c(c4)OCC)O

Supplementary Table S2:
Detailed table of XP Docking using Schrodinger's Glide module

ZINC_ID	xLogP	ApolarDesolvation	PolarDesolvation	H Donors	H Acceptors	Charge	MolecularWeight	RotatableBonds	SMILES
ZINC08440783	6	-0.3	-15.47	1	7	0	505.611	9	CC[C@@H](C)OC(=O)C1=C(N=C2C[C@H](CC(=O)C2[C@@H]1c3ccc(c(c3)OCC)O)c4ccc(cc4)OC)C
ZINC08440783	6.19	-1.18	-13.52	1	7	0	505.611	9	CC[C@@H](C)OC(=O)C1[C@H](C2C(=NC1=C)C[C@H](CC2=O)c3ccc(cc3)OC)c4ccc(c(c4)OCC)O
ZINC08440782	6.82	2.02	-10.71	0	6	0	558.074	9	CC1=NC2=C([C@@H](C1C(=O)OCCc3ccccc3)c4cccc(c4OC)OC)C(=O)C[C@@H](C2)c5ccc(cc5)Cl
ZINC08440782	7	1.64	-11.1	0	6	0	558.074	9	COc1cccc(c1OC)[C@H]2C3C(=NC(=C)C2C(=O)OCCc4ccccc4)C[C@H](CC3=O)c5ccc(cc5)Cl
ZINC08440782	7	3.1	-16.72	0	6	0	558.074	9	COc1cccc(c1OC)[C@H]2[C@@H]3C(=NC(=C)C2C(=O)OCCc4ccccc4)C[C@H](CC3=O)c5ccc(cc5)Cl
ZINC08440782	6.82	1.7	-13.25	0	6	0	558.074	9	CC1=C([C@H]([C@H]2C(=N1)C[C@H](CC2=O)c3ccc(cc3)Cl)c4cccc(c4OC)OC)C(=O)OCCc5ccccc5
ZINC08440781	6.82	1.85	-10.3	0	6	0	558.074	9	CC1=NC2=C([C@H](C1C(=O)OCCc3ccccc3)c4cccc(c4OC)OC)C(=O)C[C@@H](C2)c5ccc(cc5)Cl
ZINC08440781	7	2	-12.18	0	6	0	558.074	9	COc1cccc(c1OC)[C@@H]2C3C(=NC(=C)C2C(=O)OCCc4ccccc4)C[C@H](CC3=O)c5ccc(cc5)Cl
ZINC08440781	7	3.28	-12.22	0	6	0	558.074	9	COc1cccc(c1OC)[C@@H]2[C@H]3C(=NC(=C)C2C(=O)OCCc4ccccc4)C[C@H](CC3=O)c5ccc(cc5)Cl
ZINC08440781	6.82	1.9	-15.7	0	6	0	558.074	9	CC1=C([C@@H]([C@H]2C(=N1)C[C@H](CC2=O)c3ccc(cc3)Cl)c4cccc(c4OC)OC)C(=O)OCCc5ccccc5
ZINC00702731	3.99	9.87	-41.71	0	6	-1	485.354	7	CCOc1ccc(cc1)C(=O)C2=C(C(=O)N([C@H]2c3ccc(cc3)Br)C[C@@H]4CCCO4)[O-]
ZINC00702731	4.44	-2.08	-30.15	1	6	0	486.362	6	CCOc1ccc(cc1)C(=C2[C@@H](N(C(=O)C2=O)C[C@@H]3CCCO3)c4ccc(cc4)Br)O
ZINC00702731	3.41	-1.79	-23.44	0	6	0	486.362	7	CCOc1ccc(cc1)C(=O)[C@@H]2[C@@H](N(C(=O)C2=O)C[C@@H]3CCCO3)c4ccc(cc4)Br
ZINC08440780	6.82	1.75	-16.71	0	6	0	558.074	9	CC1=NC2=C([C@@H](C1C(=O)OCCc3ccccc3)c4cccc(c4OC)OC)C(=O)C[C@H](C2)c5ccc(cc5)Cl
ZINC08440780	7	3.02	-19.44	0	6	0	558.074	9	COc1cccc(c1OC)[C@H]2[C@@H]3C(=NC(=C)C2C(=O)OCCc4ccccc4)C[C@@H](CC3=O)c5ccc(cc5)Cl
ZINC08440780	6.82	1.63	-18	0	6	0	558.074	9	CC1=C([C@H]([C@H]2C(=N1)C[C@@H](CC2=O)c3ccc(cc3)Cl)c4cccc(c4OC)OC)C(=O)OCCc5ccccc5
ZINC08440779	6.14	3.53	-14.68	1	6	0	478.596	5	[H]/N=C/1\[C@H](C2=CCCC[C@@H]2[C@H](C1(C#N)C#N)c3ccc(c(c3)OC)OCc4c(cc(cc4C)C)C)C#N
ZINC08440779	6.14	3.36	-47.14	2	6	1	479.604	5	Cc1cc(c(c(c1)C)COc2ccc(cc2OC)[C@@H]3[C@H]4CCCC=C4[C@@H](C(=[NH2+])C3(C#N)C#N)C#N)C
ZINC08440778	6.82	2.34	-10.72	0	6	0	558.074	9	CC1=NC2=C([C@H](C1C(=O)OCCc3ccccc3)c4cccc(c4OC)OC)C(=O)C[C@H](C2)c5ccc(cc5)Cl
ZINC08440778	7	2.29	-11.67	0	6	0	558.074	9	COc1cccc(c1OC)[C@@H]2C3C(=NC(=C)C2C(=O)OCCc4ccccc4)C[C@@H](CC3=O)c5ccc(cc5)Cl
ZINC08440778	6.82	2.15	-13.5	0	6	0	558.074	9	CC1=C([C@@H]([C@H]2C(=N1)C[C@@H](CC2=O)c3ccc(cc3)Cl)c4cccc(c4OC)OC)C(=O)OCCc5ccccc5
ZINC08440777	6.8	1.57	-11.9	0	4	0	465.593	6	CC1=NC2=C([C@@H](C1C(=O)OCC(C)C)c3cccc4c3cccc4)C(=O)C[C@@H](C2)c5ccccc5
ZINC08440777	6.98	1.82	-12.22	0	4	0	465.593	6	CC(C)COC(=O)C1[C@H](C2C(=NC1=C)C[C@H](CC2=O)c3ccccc3)c4cccc5c4cccc5
ZINC08440777	6.98	2.84	-14.09	0	4	0	465.593	6	CC(C)COC(=O)C1[C@H]([C@@H]2C(=NC1=C)C[C@H](CC2=O)c3ccccc3)c4cccc5c4cccc5
ZINC08440777	6.8	1.57	-13.34	0	4	0	465.593	6	CC1=C([C@H]([C@H]2C(=N1)C[C@H](CC2=O)c3ccccc3)c4cccc5c4cccc5)C(=O)OCC(C)C
ZINC00702732	3.99	9.69	-40.99	0	6	-1	485.354	7	CCOc1ccc(cc1)C(=O)C2=C(C(=O)N([C@@H]2c3ccc(cc3)Br)C[C@@H]4CCCO4)[O-]
ZINC00702732	4.44	-2.36	-30.67	1	6	0	486.362	6	CCOc1ccc(cc1)C(=C2[C@H](N(C(=O)C2=O)C[C@@H]3CCCO3)c4ccc(cc4)Br)O
ZINC00702732	3.41	-2.06	-25.18	0	6	0	486.362	7	CCOc1ccc(cc1)C(=O)[C@H]2[C@H](N(C(=O)C2=O)C[C@@H]3CCCO3)c4ccc(cc4)Br
ZINC08440776	6.14	3.53	-14.67	1	6	0	478.596	5	[H]/N=C/1\[C@@H](C2=CCCC[C@H]2[C@@H](C1(C#N)C#N)c3ccc(c(c3)OC)OCc4c(cc(cc4C)C)C)C#N
ZINC08440776	6.14	3.36	-47.17	2	6	1	479.604	5	Cc1cc(c(c(c1)C)COc2ccc(cc2OC)[C@H]3[C@@H]4CCCC=C4[C@H](C(=[NH2+])C3(C#N)C#N)C#N)C
ZINC08440775	6.8	1.4	-7.74	0	4	0	465.593	6	CC1=NC2=C([C@H](C1C(=O)OCC(C)C)c3cccc4c3cccc4)C(=O)C[C@@H](C2)c5ccccc5
ZINC08440775	6.98	1.51	-8.82	0	4	0	465.593	6	CC(C)COC(=O)C1[C@@H](C2C(=NC1=C)C[C@H](CC2=O)c3ccccc3)c4cccc5c4cccc5
ZINC08440775	6.98	2.85	-16.27	0	4	0	465.593	6	CC(C)COC(=O)C1[C@@H]([C@H]2C(=NC1=C)C[C@H](CC2=O)c3ccccc3)c4cccc5c4cccc5
ZINC08440775	6.8	1.37	-10.81	0	4	0	465.593	6	CC1=C([C@@H]([C@@H]2C(=N1)C[C@H](CC2=O)c3ccccc3)c4cccc5c4cccc5)C(=O)OCC(C)C
ZINC00702727	4.28	10.23	-38.95	0	6	-1	481.322	7	CCOc1ccc(cc1)C(=O)C2=C(C(=O)N([C@H]2c3ccc(cc3)Br)Cc4ccco4)[O-]
ZINC00702727	4.72	0.12	-26.35	1	6	0	482.33	6	CCOc1ccc(cc1)C(=C2[C@@H](N(C(=O)C2=O)Cc3ccco3)c4ccc(cc4)Br)O
ZINC00702727	3.69	0.4	-19.37	0	6	0	482.33	7	CCOc1ccc(cc1)C(=O)[C@@H]2[C@@H](N(C(=O)C2=O)Cc3ccco3)c4ccc(cc4)Br
ZINC08440774	6.8	1.26	-8	0	4	0	465.593	6	CC1=NC2=C([C@@H](C1C(=O)OCC(C)C)c3cccc4c3cccc4)C(=O)C[C@H](C2)c5ccccc5
ZINC08440774	6.98	1.29	-8.86	0	4	0	465.593	6	CC(C)COC(=O)C1[C@H](C2C(=NC1=C)C[C@@H](CC2=O)c3ccccc3)c4cccc5c4cccc5
ZINC08440774	6.98	2.22	-8.26	0	4	0	465.593	6	CC(C)COC(=O)C1[C@H]([C@@H]2C(=NC1=C)C[C@@H](CC2=O)c3ccccc3)c4cccc5c4cccc5
ZINC08440774	6.8	1.39	-11.18	0	4	0	465.593	6	CC1=C([C@H]([C@@H]2C(=N1)C[C@@H](CC2=O)c3ccccc3)c4cccc5c4cccc5)C(=O)OCC(C)C
ZINC08440773	6.14	3.67	-15	1	6	0	478.596	5	[H]/N=C\1/[C@H](C2=CCCC[C@H]2[C@H](C1(C#N)C#N)c3ccc(c(c3)OC)OCc4c(cc(cc4C)C)C)C#N
ZINC08440773	6.14	3.48	-44.82	2	6	1	479.604	5	Cc1cc(c(c(c1)C)COc2ccc(cc2OC)[C@@H]3[C@@H]4CCCC=C4[C@@H](C(=[NH2+])C3(C#N)C#N)C#N)C
ZINC08440772	4.28	10.54	-37.52	0	6	-1	481.322	7	CCOc1ccc(cc1)C(=O)C2=C(C(=O)N([C@@H]2c3ccc(cc3)Br)Cc4ccco4)[O-]
ZINC08440772	4.72	-0.26	-27.52	1	6	0	482.33	6	CCOc1ccc(cc1)C(=C2[C@H](N(C(=O)C2=O)Cc3ccco3)c4ccc(cc4)Br)O
ZINC08440772	3.69	0	-23.18	0	6	0	482.33	7	CCOc1ccc(cc1)C(=O)[C@H]2[C@H](N(C(=O)C2=O)Cc3ccco3)c4ccc(cc4)Br
ZINC08440771	6.8	1.4	-7.85	0	4	0	465.593	6	CC1=NC2=C([C@H](C1C(=O)OCC(C)C)c3cccc4c3cccc4)C(=O)C[C@H](C2)c5ccccc5
ZINC08440771	6.98	1.59	-8.94	0	4	0	465.593	6	CC(C)COC(=O)C1[C@@H](C2C(=NC1=C)C[C@@H](CC2=O)c3ccccc3)c4cccc5c4cccc5
ZINC08440771	6.98	2.6	-8.28	0	4	0	465.593	6	CC(C)COC(=O)C1[C@@H]([C@H]2C(=NC1=C)C[C@@H](CC2=O)c3ccccc3)c4cccc5c4cccc5
ZINC08440771	6.8	1.47	-10.64	0	4	0	465.593	6	CC1=C([C@@H]([C@@H]2C(=N1)C[C@@H](CC2=O)c3ccccc3)c4cccc5c4cccc5)C(=O)OCC(C)C
ZINC00848043	5.7	0.26	-13.08	0	7	0	531.649	9	CC1=NC2=C([C@@H](C1C(=O)OC[C@H]3CCCO3)c4ccc(c(c4)OC)OCc5ccccc5)C(=O)CC(C2)(C)C
ZINC00848043	5.88	0.6	-13.98	0	7	0	531.649	9	CC1(CC2=NC(=C)C([C@H](C2C(=O)C1)c3ccc(c(c3)OC)OCc4ccccc4)C(=O)OC[C@H]5CCCO5)C
ZINC00848043	5.88	1.72	-20.83	0	7	0	531.649	9	CC1(CC2=NC(=C)C([C@H]([C@H]2C(=O)C1)c3ccc(c(c3)OC)OCc4ccccc4)C(=O)OC[C@H]5CCCO5)C
ZINC00848043	5.7	0.65	-16.42	0	7	0	531.649	9	CC1=C([C@H]([C@H]2C(=N1)CC(CC2=O)(C)C)c3ccc(c(c3)OC)OCc4ccccc4)C(=O)OC[C@H]5CCCO5
ZINC00848044	5.7	-0.71	-18.9	0	7	0	531.649	9	CC1=NC2=C([C@H](C1C(=O)OC[C@H]3CCCO3)c4ccc(c(c4)OC)OCc5ccccc5)C(=O)CC(C2)(C)C
ZINC00848044	5.7	0.42	-21.4	0	7	0	531.649	9	CC1=C([C@@H]([C@@H]2C(=N1)CC(CC2=O)(C)C)c3ccc(c(c3)OC)OCc4ccccc4)C(=O)OC[C@H]5CCCO5
ZINC00848044	5.88	0.14	-18.76	0	7	0	531.649	9	CC1(CC2=NC(=C)C([C@@H](C2C(=O)C1)c3ccc(c(c3)OC)OCc4ccccc4)C(=O)OC[C@H]5CCCO5)C
ZINC00702724	3.02	-0.6	-14.74	1	6	0	446.297	5	Cc1ccc(o1)C(=O)C2=C(C(=O)N([C@@H]2c3cccc(c3)Br)C[C@H]4CCCO4)O
ZINC00702724	2.43	-0.83	-22.91	0	6	0	446.297	5	Cc1ccc(o1)C(=O)[C@H]2[C@H](N(C(=O)C2=O)C[C@H]3CCCO3)c4cccc(c4)Br
ZINC00848045	5.7	0.1	-12.85	0	7	0	531.649	9	CC1=NC2=C([C@@H](C1C(=O)OC[C@@H]3CCCO3)c4ccc(c(c4)OC)OCc5ccccc5)C(=O)CC(C2)(C)C
ZINC00848045	5.88	0.15	-13.27	0	7	0	531.649	9	CC1(CC2=NC(=C)C([C@H](C2C(=O)C1)c3ccc(c(c3)OC)OCc4ccccc4)C(=O)OC[C@@H]5CCCO5)C

Supplementary Table S2:
Detailed table of XP Docking using Schrodinger's Glide module

ZINC_ID	xLogP	ApolarDesolvation	PolarDesolvation	H Donors	H Acceptors	Charge	MolecularWeight	RotatableBonds	SMILES
ZINC00848045	5.88	1.55	-19.19	0	7	0	531.649	9	CC1(CC2=NC(=C)C([C@H]([C@H]2C(=O)C1)c3ccc(c(c3)OC)OCc4ccccc4)C(=O)OC[C@@H]5CCCO5)C
ZINC08440770	4.38	0.44	-12.67	0	7	0	477.557	9	CC1=NC2=C([C@@H](C1C(=O)OCCOC)c3ccccc3OC)C(=O)C[C@@H](C2)c4ccc(cc4)OC
ZINC08440770	4.56	0.68	-13.96	0	7	0	477.557	9	COCCOC(=O)C1[C@H](C2C(=NC1=C)C[C@H](CC2=O)c3ccc(cc3)OC)c4ccccc4OC
ZINC08440770	4.56	1.67	-13.35	0	7	0	477.557	9	COCCOC(=O)C1[C@H]([C@@H]2C(=NC1=C)C[C@H](CC2=O)c3ccc(cc3)OC)c4ccccc4OC
ZINC08440770	4.38	0.38	-15.82	0	7	0	477.557	9	CC1=C([C@H]([C@@H]2C(=N1)C[C@H](CC2=O)c3ccc(cc3)OC)c4ccccc4OC)C(=O)OCCOC
ZINC08440769	5.71	-0.23	-15.32	0	5	0	472.006	7	CCOC(=O)C1[C@@H](C2=C(CCCC2=O)N=C1C)c3cc(c(s3)C)COc4ccc(cc4)Cl
ZINC08440769	5.71	-0.52	-16.84	0	5	0	472.006	7	CCOC(=O)C1=C(N=C2CCCC(=O)C2[C@H]1c3cc(c(s3)C)COc4ccc(cc4)Cl)C
ZINC08440769	5.89	-1.4	-15.28	0	5	0	472.006	7	CCOC(=O)C1[C@@H](C2C(=NC1=C)CCCC2=O)c3cc(c(s3)C)COc4ccc(cc4)Cl
ZINC00702725	3.02	-1.2	-15.14	1	6	0	446.297	5	Cc1ccc(o1)C(=O)C2=C(C(=O)N([C@H]2c3cccc(c3)Br)C[C@@H]4CCCO4)O
ZINC00702725	2.43	-1.4	-22.73	0	6	0	446.297	5	Cc1ccc(o1)C(=O)[C@@H]2[C@@H](N(C(=O)C2=O)C[C@@H]3CCCO3)c4cccc(c4)Br
ZINC08440768	4.38	0.12	-12.16	0	7	0	477.557	9	CC1=NC2=C([C@H](C1C(=O)OCCOC)c3ccccc3OC)C(=O)C[C@@H](C2)c4ccc(cc4)OC
ZINC08440768	4.56	0.22	-13.32	0	7	0	477.557	9	COCCOC(=O)C1[C@@H](C2C(=NC1=C)C[C@H](CC2=O)c3ccc(cc3)OC)c4ccccc4OC
ZINC08440768	4.56	1.28	-12.51	0	7	0	477.557	9	COCCOC(=O)C1[C@@H]([C@H]2C(=NC1=C)C[C@H](CC2=O)c3ccc(cc3)OC)c4ccccc4OC
ZINC08440768	4.38	0.27	-15.6	0	7	0	477.557	9	CC1=C([C@@H]([C@@H]2C(=N1)C[C@H](CC2=O)c3ccc(cc3)OC)c4ccccc4OC)C(=O)OCCOC
ZINC08440767	4.38	0.34	-12.17	0	7	0	477.557	9	CC1=NC2=C([C@@H](C1C(=O)OCCOC)c3ccccc3OC)C(=O)C[C@H](C2)c4ccc(cc4)OC
ZINC08440767	4.56	0.44	-13.27	0	7	0	477.557	9	COCCOC(=O)C1[C@H](C2C(=NC1=C)C[C@@H](CC2=O)c3ccc(cc3)OC)c4ccccc4OC
ZINC08440767	4.56	1.51	-12.59	0	7	0	477.557	9	COCCOC(=O)C1[C@H]([C@@H]2C(=NC1=C)C[C@@H](CC2=O)c3ccc(cc3)OC)c4ccccc4OC
ZINC08440767	4.38	0.48	-15.67	0	7	0	477.557	9	CC1=C([C@H]([C@H]2C(=N1)C[C@@H](CC2=O)c3ccc(cc3)OC)c4ccccc4OC)C(=O)OCCOC
ZINC08440766	4.38	0.57	-15.19	0	7	0	477.557	9	CC1=NC2=C([C@H](C1C(=O)OCCOC)c3ccccc3OC)C(=O)C[C@H](C2)c4ccc(cc4)OC
ZINC08440766	4.56	0.61	-15.63	0	7	0	477.557	9	COCCOC(=O)C1[C@@H](C2C(=NC1=C)C[C@@H](CC2=O)c3ccc(cc3)OC)c4ccccc4OC
ZINC08440766	4.56	1.66	-14.99	0	7	0	477.557	9	COCCOC(=O)C1[C@@H]([C@H]2C(=NC1=C)C[C@@H](CC2=O)c3ccc(cc3)OC)c4ccccc4OC
ZINC08440766	4.38	0.36	-17.12	0	7	0	477.557	9	CC1=C([C@@H]([C@H]2C(=N1)C[C@@H](CC2=O)c3ccc(cc3)OC)c4ccccc4OC)C(=O)OCCOC
ZINC00702726	3.02	-1.37	-12	1	6	0	446.297	5	Cc1ccc(o1)C(=O)C2=C(C(=O)N([C@@H]2c3cccc(c3)Br)C[C@@H]4CCCO4)O
ZINC00702726	2.43	-1.56	-17.31	0	6	0	446.297	5	Cc1ccc(o1)C(=O)[C@H]2[C@H](N(C(=O)C2=O)C[C@@H]3CCCO3)c4cccc(c4)Br
ZINC08440765	6.05	1.08	-11.9	0	4	0	437.539	5	CCOC(=O)C1[C@@H](C2=C(C[C@@H](CC2=O)c3ccccc3)N=C1C)c4cccc5c4cccc5
ZINC08440765	6.05	1.11	-14	0	4	0	437.539	5	CCOC(=O)C1=C(N=C2C[C@@H](CC(=O)C2[C@H]1c3cccc4c3cccc4)c5ccccc5)C
ZINC08440765	6.24	0.05	-11.78	0	4	0	437.539	5	CCOC(=O)C1[C@@H](C2C(=NC1=C)C[C@@H](CC2=O)c3ccccc3)c4cccc5c4cccc5
ZINC08440764	6.05	1.12	-8.57	0	4	0	437.539	5	CCOC(=O)C1[C@H](C2=C(C[C@@H](CC2=O)c3ccccc3)N=C1C)c4cccc5c4cccc5
ZINC08440764	6.05	1.04	-11.46	0	4	0	437.539	5	CCOC(=O)C1=C(N=C2C[C@@H](CC(=O)C2[C@@H]1c3cccc4c3cccc4)c5ccccc5)C
ZINC08440764	6.24	-0.07	-9.49	0	4	0	437.539	5	CCOC(=O)C1[C@H](C2C(=NC1=C)C[C@@H](CC2=O)c3ccccc3)c4cccc5c4cccc5
ZINC08440763	5.71	-0.21	-11.05	0	5	0	472.006	7	CCOC(=O)C1[C@H](C2=C(CCCC2=O)N=C1C)c3cc(c(s3)C)COc4ccc(cc4)Cl
ZINC08440763	5.71	-0.48	-14.6	0	5	0	472.006	7	CCOC(=O)C1=C(N=C2CCCC(=O)C2[C@@H]1c3cc(c(s3)C)COc4ccc(cc4)Cl)C
ZINC08440763	5.89	-1.43	-12.29	0	5	0	472.006	7	CCOC(=O)C1[C@H](C2C(=NC1=C)CCCC2=O)c3cc(c(s3)C)COc4ccc(cc4)Cl
ZINC00702715	1.88	7.34	-70.41	0	8	-1	426.445	7	Cc1ccc(o1)C(=O)C2=C(C(=O)N([C@H]2c3ccc(c(c3)OC)OC)C[C@H]4CCCO4)[O-]
ZINC08440762	6.05	1.09	-11.81	0	4	0	437.539	5	CCOC(=O)C1[C@@H](C2=C(C[C@H](CC2=O)c3ccccc3)N=C1C)c4cccc5c4cccc5
ZINC08440762	6.05	0.99	-13.77	0	4	0	437.539	5	CCOC(=O)C1=C(N=C2C[C@H](CC(=O)C2[C@H]1c3cccc4c3cccc4)c5ccccc5)C
ZINC08440762	6.24	-0.05	-11.64	0	4	0	437.539	5	CCOC(=O)C1[C@@H](C2C(=NC1=C)C[C@H](CC2=O)c3ccccc3)c4cccc5c4cccc5
ZINC08440761	6.05	1.33	-8.82	0	4	0	437.539	5	CCOC(=O)C1[C@H](C2=C(C[C@H](CC2=O)c3ccccc3)N=C1C)c4cccc5c4cccc5
ZINC08440761	6.05	0.95	-11.96	0	4	0	437.539	5	CCOC(=O)C1=C(N=C2C[C@H](CC(=O)C2[C@@H]1c3cccc4c3cccc4)c5ccccc5)C
ZINC08440761	6.24	0.05	-9.74	0	4	0	437.539	5	CCOC(=O)C1[C@H](C2C(=NC1=C)C[C@H](CC2=O)c3ccccc3)c4cccc5c4cccc5
ZINC00702716	1.88	7.52	-70.22	0	8	-1	426.445	7	Cc1ccc(o1)C(=O)C2=C(C(=O)N([C@@H]2c3ccc(c(c3)OC)OC)C[C@H]4CCCO4)[O-]
ZINC08440760	6.85	1.51	-14.77	0	6	0	537.656	10	CC1=NC2=C([C@@H](C1C(=O)OCCOc3ccccc3)c4ccccc4OC(C)C)C(=O)C[C@@H](C2)c5ccccc5
ZINC08440760	7.04	1.51	-14.67	0	6	0	537.656	10	CC(C)Oc1ccccc1[C@H]2C3C(=NC(=C)C2C(=O)OCCOc4ccccc4)C[C@H](CC3=O)c5ccccc5
ZINC08440760	7.04	2.84	-18.29	0	6	0	537.656	10	CC(C)Oc1ccccc1[C@H]2[C@@H]3C(=NC(=C)C2C(=O)OCCOc4ccccc4)C[C@H](CC3=O)c5ccccc5
ZINC08440760	6.85	1.46	-16.54	0	6	0	537.656	10	CC1=C([C@H]([C@@H]2C(=N1)C[C@H](CC2=O)c3ccccc3)c4ccccc4OC(C)C)C(=O)OCCOc5ccccc5
ZINC08440759	6.85	1.21	-13.15	0	6	0	537.656	10	CC1=NC2=C([C@H](C1C(=O)OCCOc3ccccc3)c4ccccc4OC(C)C)C(=O)C[C@@H](C2)c5ccccc5
ZINC08440759	7.04	1.35	-13.4	0	6	0	537.656	10	CC(C)Oc1ccccc1[C@@H]2C3C(=NC(=C)C2C(=O)OCCOc4ccccc4)C[C@H](CC3=O)c5ccccc5
ZINC08440759	7.04	2.51	-13.72	0	6	0	537.656	10	CC(C)Oc1ccccc1[C@@H]2[C@H]3C(=NC(=C)C2C(=O)OCCOc4ccccc4)C[C@H](CC3=O)c5ccccc5
ZINC08440759	6.85	1.25	-15.48	0	6	0	537.656	10	CC1=C([C@@H]([C@H]2C(=N1)C[C@H](CC2=O)c3ccccc3)c4ccccc4OC(C)C)C(=O)OCCOc5ccccc5
ZINC08440758	6.85	1.44	-15.52	0	6	0	537.656	10	CC1=NC2=C([C@@H](C1C(=O)OCCOc3ccccc3)c4ccccc4OC(C)C)C(=O)C[C@H](C2)c5ccccc5
ZINC08440758	7.04	1.38	-14.54	0	6	0	537.656	10	CC(C)Oc1ccccc1[C@H]2C3C(=NC(=C)C2C(=O)OCCOc4ccccc4)C[C@@H](CC3=O)c5ccccc5
ZINC08440758	7.04	2.72	-18.85	0	6	0	537.656	10	CC(C)Oc1ccccc1[C@H]2[C@@H]3C(=NC(=C)C2C(=O)OCCOc4ccccc4)C[C@@H](CC3=O)c5ccccc5
ZINC08440758	6.85	1.34	-16.38	0	6	0	537.656	10	CC1=C([C@H]([C@H]2C(=N1)C[C@@H](CC2=O)c3ccccc3)c4ccccc4OC(C)C)C(=O)OCCOc5ccccc5
ZINC08440757	6.85	1.53	-14.84	0	6	0	537.656	10	CC1=NC2=C([C@H](C1C(=O)OCCOc3ccccc3)c4ccccc4OC(C)C)C(=O)C[C@H](C2)c5ccccc5
ZINC08440757	7.04	1.67	-14.76	0	6	0	537.656	10	CC(C)Oc1ccccc1[C@@H]2C3C(=NC(=C)C2C(=O)OCCOc4ccccc4)C[C@@H](CC3=O)c5ccccc5
ZINC08440757	7.04	2.67	-15.47	0	6	0	537.656	10	CC(C)Oc1ccccc1[C@@H]2[C@H]3C(=NC(=C)C2C(=O)OCCOc4ccccc4)C[C@@H](CC3=O)c5ccccc5
ZINC08440757	6.85	1.61	-16.44	0	6	0	537.656	10	CC1=C([C@@H]([C@@H]2C(=N1)C[C@@H](CC2=O)c3ccccc3)c4ccccc4OC(C)C)C(=O)OCCOc5ccccc5
ZINC08440756	6.36	0.39	-13.8	0	5	0	500.06	7	CCOC(=O)C1[C@@H](C2=C(CC(CC2=O)(C)C)N=C1C)c3cc(c(s3)C)COc4ccc(cc4)Cl
ZINC08440756	6.36	0.55	-15.9	0	5	0	500.06	7	CCOC(=O)C1=C(N=C2CC(CC(=O)C2[C@H]1c3cc(c(s3)C)COc4ccc(cc4)Cl)(C)C)C
ZINC08440756	6.54	-0.5	-13.16	0	5	0	500.06	7	CCOC(=O)C1[C@@H](C2C(=NC1=C)CC(CC2=O)(C)C)c3cc(c(s3)C)COc4ccc(cc4)Cl
ZINC00702717	1.88	7.83	-66.41	0	8	-1	426.445	7	Cc1ccc(o1)C(=O)C2=C(C(=O)N([C@H]2c3ccc(c(c3)OC)OC)C[C@@H]4CCCO4)[O-]

Supplementary Table S2:
Detailed table of XP Docking using Schrodinger's Glide module

ZINC_ID	xLogP	ApolarDesolvation	PolarDesolvation	H Donors	H Acceptors	Charge	MolecularWeight	RotatableBonds	SMILES
ZINC08440754	7.1	-1.29	-10.11	1	6	0	574.899	7	CC1=NC2=C([C@H](C1C(=O)OCC(C)C)c3cc(c(c(c3)Br)O)OC)C(=O)C[C@@H](C2)c4ccc(cc4)Cl
ZINC08440754	7.28	-1.22	-11.32	1	6	0	574.899	7	CC(C)COC(=O)C1[C@@H](C2C(=NC1=C)C[C@H](CC2=O)c3ccc(cc3)Cl)c4cc(c(c(c4)Br)O)OC
ZINC08440754	7.28	0.03	-12.04	1	6	0	574.899	7	CC(C)COC(=O)C1[C@@H]([C@H]2C(=NC1=C)C[C@H](CC2=O)c3ccc(cc3)Cl)c4cc(c(c(c4)Br)O)OC
ZINC08440754	7.1	-1.18	-13.83	1	6	0	574.899	7	CC1=C([C@@H]([C@H]2C(=N1)C[C@H](CC2=O)c3ccc(cc3)Cl)c4cc(c(c(c4)Br)O)OC)C(=O)OCC(C)C
ZINC08440753	7.1	-1.25	-9.68	1	6	0	574.899	7	CC1=NC2=C([C@@H](C1C(=O)OCC(C)C)c3cc(c(c(c3)Br)O)OC)C(=O)C[C@H](C2)c4ccc(cc4)Cl
ZINC08440753	7.28	-1.15	-11.25	1	6	0	574.899	7	CC(C)COC(=O)C1[C@H](C2C(=NC1=C)C[C@@H](CC2=O)c3ccc(cc3)Cl)c4cc(c(c(c4)Br)O)OC
ZINC08440753	7.28	0.08	-9.53	1	6	0	574.899	7	CC(C)COC(=O)C1[C@H]([C@@H]2C(=NC1=C)C[C@@H](CC2=O)c3ccc(cc3)Cl)c4cc(c(c(c4)Br)O)OC
ZINC00702718	1.88	7.51	-65.93	0	8	-1	426.445	7	Cc1ccc(o1)C(=O)C2=C(C(=O)N([C@@H]2c3ccc(c(c3)OC)OC)C[C@@H]4CCCO4)[O-]
ZINC08440751	7.54	17.24	-19.99	1	5	0	507.63	9	CCc1ccc(cc1)[C@H]2C3=C(C[C@H](CC3=O)c4ccccc4)NC(=C2C(=O)OCCOc5ccccc5)C
ZINC08440751	7.66	-0.01	-9.21	0	5	0	507.63	9	CCc1ccc(cc1)[C@H]2C3C(=O)C[C@@H](C=C3N=C(C2C(=O)OCCOc4ccccc4)C)c5ccccc5
ZINC08440750	7.54	17.23	-19.59	1	5	0	507.63	9	CCc1ccc(cc1)[C@@H]2C3=C(C[C@H](CC3=O)c4ccccc4)NC(=C2C(=O)OCCOc5ccccc5)C
ZINC08440750	7.66	-0.2	-9.66	0	5	0	507.63	9	CCc1ccc(cc1)[C@@H]2C3C(=O)C[C@@H](C=C3N=C(C2C(=O)OCCOc4ccccc4)C)c5ccccc5
ZINC08440749	6.36	0.59	-10.68	0	5	0	500.06	7	CCOC(=O)C1[C@H](C2=C(CC(CC2=O)(C)C)N=C1C)c3cc(c(s3)C)COc4ccc(cc4)Cl
ZINC08440749	6.54	-0.29	-11.78	0	5	0	500.06	7	CCOC(=O)C1[C@H](C2C(=NC1=C)CC(CC2=O)(C)C)c3cc(c(s3)C)COc4ccc(cc4)Cl
ZINC08440749	6.36	0.66	-13.99	0	5	0	500.06	7	CCOC(=O)C1=C(N=C2CC(CC(=O)C2[C@@H]1c3cc(c(s3)C)COc4ccc(cc4)Cl)(C)C)C
ZINC00702713	2.84	9.74	-53.25	0	7	-1	436.415	7	COc1ccc(cc1OC)[C@H]2C(=C(C(=O)N2Cc3ccco3)[O-])C(=O)c4ccc(cc4)F
ZINC00702713	3.29	1.82	-29.33	1	7	0	437.423	6	COc1ccc(cc1OC)[C@H]2C(=C(c3ccc(cc3)F)O)C(=O)C(=O)N2Cc4ccco4
ZINC00702713	2.26	2.09	-21.03	0	7	0	437.423	7	COc1ccc(cc1OC)[C@H]2[C@H](C(=O)C(=O)N2Cc3ccco3)C(=O)c4ccc(cc4)F
ZINC08440748	7.54	17.24	-20.29	1	5	0	507.63	9	CCc1ccc(cc1)[C@H]2C3=C(C[C@@H](CC3=O)c4ccccc4)NC(=C2C(=O)OCCOc5ccccc5)C
ZINC08440748	7.66	-0.17	-8.95	0	5	0	507.63	9	CCc1ccc(cc1)[C@H]2C3C(=O)C[C@H](C=C3N=C(C2C(=O)OCCOc4ccccc4)C)c5ccccc5
ZINC08440747	7.54	17.24	-19.85	1	5	0	507.63	9	CCc1ccc(cc1)[C@@H]2C3=C(C[C@@H](CC3=O)c4ccccc4)NC(=C2C(=O)OCCOc5ccccc5)C
ZINC08440747	7.66	0	-8.84	0	5	0	507.63	9	CCc1ccc(cc1)[C@@H]2C3C(=O)C[C@H](C=C3N=C(C2C(=O)OCCOc4ccccc4)C)c5ccccc5
ZINC08440746	6.06	1.79	-14.49	0	6	0	489.612	8	CC1=NC2=C([C@@H](C1C(=O)OC(C)C)c3ccccc3OC(C)C)C(=O)C[C@@H](C2)c4ccc(cc4)OC
ZINC08440746	6.24	2	-15.06	0	6	0	489.612	8	CC(C)Oc1ccccc1[C@H]2C3C(=NC(=C)C2C(=O)OC(C)C)C[C@H](CC3=O)c4ccc(cc4)OC
ZINC08440746	6.24	3.09	-18.11	0	6	0	489.612	8	CC(C)Oc1ccccc1[C@H]2[C@@H]3C(=NC(=C)C2C(=O)OC(C)C)C[C@H](CC3=O)c4ccc(cc4)OC
ZINC08440746	6.06	1.75	-16.18	0	6	0	489.612	8	CC1=C([C@H]([C@H]2C(=N1)C[C@H](CC2=O)c3ccc(cc3)OC)c4ccccc4OC(C)C)C(=O)OC(C)C
ZINC08440745	6.06	1.64	-10.25	0	6	0	489.612	8	CC1=NC2=C([C@H](C1C(=O)OC(C)C)c3ccccc3OC(C)C)C(=O)C[C@@H](C2)c4ccc(cc4)OC
ZINC08440745	6.24	1.75	-11.59	0	6	0	489.612	8	CC(C)Oc1ccccc1[C@@H]2C3C(=NC(=C)C2C(=O)OC(C)C)C[C@H](CC3=O)c4ccc(cc4)OC
ZINC08440745	6.24	2.83	-10.81	0	6	0	489.612	8	CC(C)Oc1ccccc1[C@@H]2[C@H]3C(=NC(=C)C2C(=O)OC(C)C)C[C@H](CC3=O)c4ccc(cc4)OC
ZINC08440745	6.06	1.62	-13.38	0	6	0	489.612	8	CC1=C([C@@H]([C@H]2C(=N1)C[C@H](CC2=O)c3ccc(cc3)OC)c4ccccc4OC(C)C)C(=O)OC(C)C
ZINC08440744	6.5	-0.22	-13.73	0	5	0	544.511	7	CCOC(=O)C1[C@@H](C2=C(CC(CC2=O)(C)C)N=C1C)c3cc(c(s3)C)COc4ccc(cc4)Br
ZINC08440744	6.68	-1.11	-13.14	0	5	0	544.511	7	CCOC(=O)C1[C@@H](C2C(=NC1=C)CC(CC2=O)(C)C)c3cc(c(s3)C)COc4ccc(cc4)Br
ZINC08440744	6.5	-0.06	-15.81	0	5	0	544.511	7	CCOC(=O)C1=C(N=C2CC(CC(=O)C2[C@H]1c3cc(c(s3)C)COc4ccc(cc4)Br)(C)C)C
ZINC00702714	2.84	9.44	-53.29	0	7	-1	436.415	7	COc1ccc(cc1OC)[C@@H]2C(=C(C(=O)N2Cc3ccco3)[O-])C(=O)c4ccc(cc4)F
ZINC00702714	3.29	1.53	-29.32	1	7	0	437.423	6	COc1ccc(cc1OC)[C@@H]2C(=C(c3ccc(cc3)F)O)C(=O)C(=O)N2Cc4ccco4
ZINC00702714	2.26	1.82	-24.21	0	7	0	437.423	7	COc1ccc(cc1OC)[C@@H]2[C@@H](C(=O)C(=O)N2Cc3ccco3)C(=O)c4ccc(cc4)F
ZINC08440743	6.06	1.63	-10.66	0	6	0	489.612	8	CC1=NC2=C([C@@H](C1C(=O)OC(C)C)c3ccccc3OC(C)C)C(=O)C[C@H](C2)c4ccc(cc4)OC
ZINC08440743	6.24	1.74	-11.86	0	6	0	489.612	8	CC(C)Oc1ccccc1[C@H]2C3C(=NC(=C)C2C(=O)OC(C)C)C[C@@H](CC3=O)c4ccc(cc4)OC
ZINC08440743	6.24	3.06	-18.21	0	6	0	489.612	8	CC(C)Oc1ccccc1[C@H]2[C@@H]3C(=NC(=C)C2C(=O)OC(C)C)C[C@@H](CC3=O)c4ccc(cc4)OC
ZINC08440743	6.06	1.63	-13.93	0	6	0	489.612	8	CC1=C([C@H]([C@H]2C(=N1)C[C@@H](CC2=O)c3ccc(cc3)OC)c4ccccc4OC(C)C)C(=O)OC(C)C
ZINC08440742	6.06	1.64	-10.22	0	6	0	489.612	8	CC1=NC2=C([C@H](C1C(=O)OC(C)C)c3ccccc3OC(C)C)C(=O)C[C@H](C2)c4ccc(cc4)OC
ZINC08440742	6.24	1.83	-11.68	0	6	0	489.612	8	CC(C)Oc1ccccc1[C@@H]2C3C(=NC(=C)C2C(=O)OC(C)C)C[C@@H](CC3=O)c4ccc(cc4)OC
ZINC08440742	6.24	2.84	-10.82	0	6	0	489.612	8	CC(C)Oc1ccccc1[C@@H]2[C@H]3C(=NC(=C)C2C(=O)OC(C)C)C[C@@H](CC3=O)c4ccc(cc4)OC
ZINC08440742	6.06	1.55	-13.4	0	6	0	489.612	8	CC1=C([C@@H]([C@H]2C(=N1)C[C@@H](CC2=O)c3ccc(cc3)OC)c4ccccc4OC(C)C)C(=O)OC(C)C
ZINC08440741	5.11	0.86	-12.36	0	5	0	469.94	7	CC1=NC2=C([C@@H](C1C(=O)OCCOC)c3ccccc3F)C(=O)C[C@@H](C2)c4ccc(cc4)Cl
ZINC08440741	5.29	1.13	-13.52	0	5	0	469.94	7	COCCOC(=O)C1[C@H](C2C(=NC1=C)C[C@H](CC2=O)c3ccc(cc3)Cl)c4ccccc4F
ZINC08440741	5.29	2.17	-14.03	0	5	0	469.94	7	COCCOC(=O)C1[C@H]([C@@H]2C(=NC1=C)C[C@H](CC2=O)c3ccc(cc3)Cl)c4ccccc4F
ZINC08440741	5.11	0.96	-15.85	0	5	0	469.94	7	CC1=C([C@H]([C@H]2C(=N1)C[C@H](CC2=O)c3ccc(cc3)Cl)c4ccccc4F)C(=O)OCCOC
ZINC00702711	3.11	9.56	-43.77	0	8	-1	462.478	9	CCOc1ccc(cc1)C(=O)C2=C(C(=O)N([C@H]2c3ccc(c(c3)OC)OC)Cc4ccco4)[O-]
ZINC00702711	3.56	1.15	-29.46	1	8	0	463.486	8	CCOc1ccc(cc1)C(=C2[C@@H](N(C(=O)C2=O)Cc3ccco3)c4ccc(c(c4)OC)OC)O
ZINC00702711	2.53	1.41	-22.14	0	8	0	463.486	9	CCOc1ccc(cc1)C(=O)[C@@H]2[C@@H](N(C(=O)C2=O)Cc3ccco3)c4ccc(c(c4)OC)OC
ZINC08440740	5.11	0.86	-11.53	0	5	0	469.94	7	CC1=NC2=C([C@H](C1C(=O)OCCOC)c3ccccc3F)C(=O)C[C@@H](C2)c4ccc(cc4)Cl
ZINC08440740	5.29	0.6	-11.91	0	5	0	469.94	7	COCCOC(=O)C1[C@@H](C2C(=NC1=C)C[C@H](CC2=O)c3ccc(cc3)Cl)c4ccccc4F
ZINC08440740	5.29	2.06	-19.54	0	5	0	469.94	7	COCCOC(=O)C1[C@@H]([C@H]2C(=NC1=C)C[C@H](CC2=O)c3ccc(cc3)Cl)c4ccccc4F
ZINC08440740	5.11	0.96	-15.21	0	5	0	469.94	7	CC1=C([C@@H]([C@@H]2C(=N1)C[C@H](CC2=O)c3ccc(cc3)Cl)c4ccccc4F)C(=O)OCCOC
ZINC08440739	6.5	-0.02	-10.61	0	5	0	544.511	7	CCOC(=O)C1[C@H](C2=C(CC(CC2=O)(C)C)N=C1C)c3cc(c(s3)C)COc4ccc(cc4)Br
ZINC08440739	6.68	-0.9	-11.76	0	5	0	544.511	7	CCOC(=O)C1[C@H](C2C(=NC1=C)CC(CC2=O)(C)C)c3cc(c(s3)C)COc4ccc(cc4)Br
ZINC08440739	6.5	0.05	-13.84	0	5	0	544.511	7	CCOC(=O)C1=C(N=C2CC(CC(=O)C2[C@@H]1c3cc(c(s3)C)COc4ccc(cc4)Br)(C)C)C
ZINC08440738	5.11	0.75	-11.93	0	5	0	469.94	7	CC1=NC2=C([C@@H](C1C(=O)OCCOC)c3ccccc3F)C(=O)C[C@H](C2)c4ccc(cc4)Cl
ZINC08440738	5.29	0.89	-13.08	0	5	0	469.94	7	COCCOC(=O)C1[C@H](C2C(=NC1=C)C[C@@H](CC2=O)c3ccc(cc3)Cl)c4ccccc4F
ZINC08440738	5.29	2	-13.46	0	5	0	469.94	7	COCCOC(=O)C1[C@H]([C@@H]2C(=NC1=C)C[C@@H](CC2=O)c3ccc(cc3)Cl)c4ccccc4F

Supplementary Table S2:
Detailed table of XP Docking using Schrodinger's Glide module

ZINC_ID	xLogP	ApolarDesolvation	PolarDesolvation	H Donors	H Acceptors	Charge	MolecularWeight	RotatableBonds	SMILES
ZINC08440738	5.11	0.89	-15.89	0	5	0	469.94	7	CC1=C([C@H]([C@H]2C(=N1)C[C@@H](CC2=O)c3ccc(cc3)Cl)c4ccccc4F)C(=O)OCCOC
ZINC08440737	5.11	0.83	-11.63	0	5	0	469.94	7	CC1=NC2=C([C@H](C1C(=O)OCCOC)c3ccccc3F)C(=O)C[C@H](C2)c4ccc(cc4)Cl
ZINC08440737	5.29	1.06	-12.9	0	5	0	469.94	7	COCCOC(=O)C1[C@@H](C2C(=NC1=C)C[C@@H](CC2=O)c3ccc(cc3)Cl)c4ccccc4F
ZINC08440737	5.29	2.05	-12.17	0	5	0	469.94	7	COCCOC(=O)C1[C@@H]([C@H]2C(=NC1=C)C[C@@H](CC2=O)c3ccc(cc3)Cl)c4ccccc4F
ZINC08440737	5.11	0.95	-15.44	0	5	0	469.94	7	CC1=C([C@@H]([C@@H]2C(=N1)C[C@@H](CC2=O)c3ccc(cc3)Cl)c4ccccc4F)C(=O)OCCOC
ZINC05921172	3.11	9.17	-42.6	0	8	-1	462.478	9	CCOc1ccc(cc1)C(=O)C2=C(C(=O)N([C@@H]2c3ccc(c(c3)OC)OC)Cc4ccco4)[O-]
ZINC05921172	3.56	0.85	-29.45	1	8	0	463.486	8	CCOc1ccc(cc1)C(=C2[C@H](N(C(=O)C2=O)Cc3ccco3)c4ccc(c(c4)OC)OC)O
ZINC05921172	2.53	1.08	-22.99	0	8	0	463.486	9	CCOc1ccc(cc1)C(=O)[C@@H]2[C@H](N(C(=O)C2=O)Cc3ccco3)c4ccc(c(c4)OC)OC
ZINC08440736	5.96	13.42	-19.88	1	5	0	465.977	7	Cc1ccc(cc1)[C@H]2C3=C(C[C@H](CC3=O)c4ccc(cc4)Cl)NC(=C2C(=O)OCCOC)C
ZINC08440736	6.08	-0.81	-8.45	0	5	0	465.977	7	Cc1ccc(cc1)[C@H]2C3C(=O)C[C@@H](C=C3N=C(C2C(=O)OCCOC)C)c4ccc(cc4)Cl
ZINC08440735	5.96	13.44	-19.83	1	5	0	465.977	7	Cc1ccc(cc1)[C@@H]2C3=C(C[C@H](CC3=O)c4ccc(cc4)Cl)NC(=C2C(=O)OCCOC)C
ZINC08440735	6.08	-1.01	-9.52	0	5	0	465.977	7	Cc1ccc(cc1)[C@@H]2C3C(=O)C[C@@H](C=C3N=C(C2C(=O)OCCOC)C)c4ccc(cc4)Cl
ZINC08440734	5.96	13.41	-19.87	1	5	0	465.977	7	Cc1ccc(cc1)[C@H]2C3=C(C[C@@H](CC3=O)c4ccc(cc4)Cl)NC(=C2C(=O)OCCOC)C
ZINC08440734	6.08	-0.89	-8.42	0	5	0	465.977	7	Cc1ccc(cc1)[C@H]2C3C(=O)C[C@H](C=C3N=C(C2C(=O)OCCOC)C)c4ccc(cc4)Cl
ZINC00702709	2.16	7.95	-66.68	0	8	-1	422.413	7	Cc1ccc(o1)C(=O)C2=C(C(=O)N([C@H]2c3ccc(c(c3)OC)OC)Cc4ccco4)[O-]
ZINC00702709	2.16	7.06	-19.39	1	8	0	423.421	7	Cc1ccc(o1)C(=O)C2=C(C(=O)N([C@H]2c3ccc(c(c3)OC)OC)Cc4ccco4)O
ZINC08440733	5.96	13.43	-19.84	1	5	0	465.977	7	Cc1ccc(cc1)[C@@H]2C3=C(C[C@@H](CC3=O)c4ccc(cc4)Cl)NC(=C2C(=O)OCCOC)C
ZINC08440733	5.91	0.43	-9.37	0	5	0	465.977	7	Cc1ccc(cc1)[C@H]2C(C(=NC3=C2C(=O)C[C@H](C3)c4ccc(cc4)Cl)C)C(=O)OCCOC
ZINC08440732	5.38	12.54	-21.1	1	5	0	457.979	6	Cc1c(cc(s1)[C@H]2C3=C(CCCC3=O)NC(=C2C(=O)OC)C)COc4ccc(cc4)Cl
ZINC08440732	5.5	-1.79	-10.43	0	5	0	457.979	6	Cc1c(cc(s1)[C@H]2C3C(=O)CCC=C3N=C(C2C(=O)OC)C)COc4ccc(cc4)Cl
ZINC08440731	5.53	11.65	-19.58	1	6	0	447.531	7	CCOc1ccc(cc1)[C@H]2C3=C(C[C@H](CC3=O)c4ccc(cc4)OC)NC(=C2C(=O)OC)C
ZINC08440731	5.64	-0.3	-9.03	0	6	0	447.531	7	CCOc1ccc(cc1)[C@H]2C3C(=O)C[C@@H](C=C3N=C(C2C(=O)OC)C)c4ccc(cc4)OC
ZINC08440730	5.53	12.16	-25.98	1	6	0	447.531	7	CCOc1ccc(cc1)[C@@H]2C3=C(C[C@H](CC3=O)c4ccc(cc4)OC)NC(=C2C(=O)OC)C
ZINC08440730	5.64	-0.26	-11.23	0	6	0	447.531	7	CCOc1ccc(cc1)[C@@H]2C3C(=O)C[C@@H](C=C3N=C(C2C(=O)OC)C)c4ccc(cc4)OC
ZINC00702710	2.16	8.32	-66.81	0	8	-1	422.413	7	Cc1ccc(o1)C(=O)C2=C(C(=O)N([C@@H]2c3ccc(c(c3)OC)OC)Cc4ccco4)[O-]
ZINC00702710	2.16	7.43	-19.07	1	8	0	423.421	7	Cc1ccc(o1)C(=O)C2=C(C(=O)N([C@@H]2c3ccc(c(c3)OC)OC)Cc4ccco4)O
ZINC08440729	5.53	11.65	-19.56	1	6	0	447.531	7	CCOc1ccc(cc1)[C@H]2C3=C(C[C@@H](CC3=O)c4ccc(cc4)OC)NC(=C2C(=O)OC)C
ZINC08440729	5.64	-0.5	-9.93	0	6	0	447.531	7	CCOc1ccc(cc1)[C@H]2C3C(=O)C[C@H](C=C3N=C(C2C(=O)OC)C)c4ccc(cc4)OC
ZINC08440728	5.53	11.64	-19.55	1	6	0	447.531	7	CCOc1ccc(cc1)[C@@H]2C3=C(C[C@@H](CC3=O)c4ccc(cc4)OC)NC(=C2C(=O)OC)C
ZINC08440728	5.64	-0.29	-8.97	0	6	0	447.531	7	CCOc1ccc(cc1)[C@@H]2C3C(=O)C[C@H](C=C3N=C(C2C(=O)OC)C)c4ccc(cc4)OC
ZINC08440727	5.02	1.69	-13.83	0	7	0	509.986	9	CC1=NC2=C([C@@H](C1C(=O)OCCOC)c3ccc(cc3)OC(=O)C)C(=O)C[C@@H](C2)c4ccc(cc4)Cl
ZINC08440727	5.2	3.07	-15.8	0	7	0	509.986	9	CC(=O)Oc1ccc(cc1)[C@H]2[C@@H]3C(=NC(=C)C2C(=O)OCCOC)C[C@H](CC3=O)c4ccc(cc4)Cl
ZINC08440727	5.02	1.62	-16.99	0	7	0	509.986	9	CC1=C([C@H]([C@@H]2C(=N1)C[C@H](CC2=O)c3ccc(cc3)Cl)c4ccc(cc4)OC(=O)C)C(=O)OCCOC
ZINC00702699	4.93	12.76	-48.41	0	6	-1	468.529	7	Cc1ccc(cc1)C(=O)C2=C(C(=O)N([C@H]2c3cccc(c3)Oc4ccccc4)C[C@H]5CCCO5)[O-]
ZINC00702699	5.38	-0.66	-28.59	1	6	0	469.537	6	Cc1ccc(cc1)C(=C2[C@@H](N(C(=O)C2=O)C[C@H]3CCCO3)c4cccc(c4)Oc5ccccc5)O
ZINC00702699	4.35	-0.38	-23.32	0	6	0	469.537	7	Cc1ccc(cc1)C(=O)[C@@H]2[C@@H](N(C(=O)C2=O)C[C@H]3CCCO3)c4cccc(c4)Oc5ccccc5
ZINC08440726	5.02	1.61	-14.58	0	7	0	509.986	9	CC1=NC2=C([C@H](C1C(=O)OCCOC)c3ccc(cc3)OC(=O)C)C(=O)C[C@@H](C2)c4ccc(cc4)Cl
ZINC08440726	5.2	1.67	-16.32	0	7	0	509.986	9	CC(=O)Oc1ccc(cc1)[C@@H]2C3C(=NC(=C)C2C(=O)OCCOC)C[C@H](CC3=O)c4ccc(cc4)Cl
ZINC08440726	5.2	2.91	-16.77	0	7	0	509.986	9	CC(=O)Oc1ccc(cc1)[C@@H]2[C@H]3C(=NC(=C)C2C(=O)OCCOC)C[C@H](CC3=O)c4ccc(cc4)Cl
ZINC08440726	5.02	1.71	-19.29	0	7	0	509.986	9	CC1=C([C@@H]([C@H]2C(=N1)C[C@H](CC2=O)c3ccc(cc3)Cl)c4ccc(cc4)OC(=O)C)C(=O)OCCOC
ZINC08440725	5.38	13.06	-28.14	1	5	0	457.979	6	Cc1c(cc(s1)[C@@H]2C3=C(CCCC3=O)NC(=C2C(=O)OC)C)COc4ccc(cc4)Cl
ZINC08440725	5.5	-1.91	-12.99	0	5	0	457.979	6	Cc1c(cc(s1)[C@@H]2C3C(=O)CCC=C3N=C(C2C(=O)OC)C)COc4ccc(cc4)Cl
ZINC08440724	5.02	1.6	-13.95	0	7	0	509.986	9	CC1=NC2=C([C@@H](C1C(=O)OCCOC)c3ccc(cc3)OC(=O)C)C(=O)C[C@H](C2)c4ccc(cc4)Cl
ZINC08440724	5.2	1.69	-14.55	0	7	0	509.986	9	CC(=O)Oc1ccc(cc1)[C@H]2C3C(=NC(=C)C2C(=O)OCCOC)C[C@@H](CC3=O)c4ccc(cc4)Cl
ZINC08440724	5.2	2.76	-14.56	0	7	0	509.986	9	CC(=O)Oc1ccc(cc1)[C@H]2[C@@H]3C(=NC(=C)C2C(=O)OCCOC)C[C@@H](CC3=O)c4ccc(cc4)Cl
ZINC08440724	5.02	1.74	-16.45	0	7	0	509.986	9	CC1=C([C@H]([C@H]2C(=N1)C[C@@H](CC2=O)c3ccc(cc3)Cl)c4ccc(cc4)OC(=O)C)C(=O)OCCOC
ZINC00702700	4.93	12.62	-48.84	0	6	-1	468.529	7	Cc1ccc(cc1)C(=O)C2=C(C(=O)N([C@@H]2c3cccc(c3)Oc4ccccc4)C[C@H]5CCCO5)[O-]
ZINC00702700	5.38	-0.05	-28.93	1	6	0	469.537	6	Cc1ccc(cc1)C(=C2[C@H](N(C(=O)C2=O)C[C@H]3CCCO3)c4cccc(c4)Oc5ccccc5)O
ZINC00702700	4.35	0.2	-26.62	0	6	0	469.537	7	Cc1ccc(cc1)C(=O)[C@H]2[C@H](N(C(=O)C2=O)C[C@H]3CCCO3)c4cccc(c4)Oc5ccccc5
ZINC08440723	5.02	1.71	-13.87	0	7	0	509.986	9	CC1=NC2=C([C@H](C1C(=O)OCCOC)c3ccc(cc3)OC(=O)C)C(=O)C[C@H](C2)c4ccc(cc4)Cl
ZINC08440723	5.2	2.94	-17.57	0	7	0	509.986	9	CC(=O)Oc1ccc(cc1)[C@@H]2[C@H]3C(=NC(=C)C2C(=O)OCCOC)C[C@@H](CC3=O)c4ccc(cc4)Cl
ZINC08440723	5.02	1.66	-19.46	0	7	0	509.986	9	CC1=C([C@@H]([C@H]2C(=N1)C[C@@H](CC2=O)c3ccc(cc3)Cl)c4ccc(cc4)OC(=O)C)C(=O)OCCOC
ZINC08440723	5.2	1.77	-15.76	0	7	0	509.986	9	CC(=O)Oc1ccc(cc1)[C@@H]2C3C(=NC(=C)C2C(=O)OCCOC)C[C@@H](CC3=O)c4ccc(cc4)Cl
ZINC08440722	5.36	13.19	-21.57	1	7	0	505.611	11	CCCOc1ccc(cc1OC)[C@H]2C3=C(C[C@H](CC3=O)c4ccccc4)NC(=C2C(=O)OCCOC)C
ZINC08440722	5.47	-0.55	-10.12	0	7	0	505.611	11	CCCOc1ccc(cc1OC)[C@H]2C3C(=O)C[C@@H](C=C3N=C(C2C(=O)OCCOC)C)c4ccccc4
ZINC08440721	5.36	13.7	-25.49	1	7	0	505.611	11	CCCOc1ccc(cc1OC)[C@@H]2C3=C(C[C@H](CC3=O)c4ccccc4)NC(=C2C(=O)OCCOC)C
ZINC08440721	5.47	-0.49	-11.52	0	7	0	505.611	11	CCCOc1ccc(cc1OC)[C@@H]2C3C(=O)C[C@@H](C=C3N=C(C2C(=O)OCCOC)C)c4ccccc4
ZINC00702701	4.93	12.73	-50.05	0	6	-1	468.529	7	Cc1ccc(cc1)C(=O)C2=C(C(=O)N([C@H]2c3cccc(c3)Oc4ccccc4)C[C@@H]5CCCO5)[O-]
ZINC00702701	5.38	-0.37	-27.97	1	6	0	469.537	6	Cc1ccc(cc1)C(=C2[C@@H](N(C(=O)C2=O)C[C@@H]3CCCO3)c4cccc(c4)Oc5ccccc5)O
ZINC00702701	4.35	-0.15	-20.8	0	6	0	469.537	7	Cc1ccc(cc1)C(=O)[C@H]2[C@@H](N(C(=O)C2=O)C[C@@H]3CCCO3)c4cccc(c4)Oc5ccccc5
ZINC08440720	5.36	13.19	-21.67	1	7	0	505.611	11	CCCOc1ccc(cc1OC)[C@H]2C3=C(C[C@@H](CC3=O)c4ccccc4)NC(=C2C(=O)OCCOC)C

Supplementary Table S2:
Detailed table of XP Docking using Schrodinger's Glide module

ZINC_ID	xLogP	ApolarDesolvation	PolarDesolvation	H Donors	H Acceptors	Charge	MolecularWeight	RotatableBonds	SMILES
ZINC08440720	5.47	-0.75	-10.66	0	7	0	505.611	11	CCCOc1ccc(cc1OC)[C@H]2C3C(=O)C[C@H](C=C3N=C(C2C(=O)OCCOC)C)c4ccccc4
ZINC08440719	5.51	13.16	-28.11	1	5	0	502.43	6	Cc1c(cc(s1)[C@@H]2C3=C(CCCC3=O)NC(=C2C(=O)OC)C)COc4ccc(cc4)Br
ZINC08440719	5.63	-2.53	-12.86	0	5	0	502.43	6	Cc1c(cc(s1)[C@@H]2C3C(=O)CCC=C3N=C(C2C(=O)OC)C)COc4ccc(cc4)Br
ZINC08440718	5.36	13.71	-25.6	1	7	0	505.611	11	CCCOc1ccc(cc1OC)[C@@H]2C3=C(C[C@@H](CC3=O)c4ccccc4)NC(=C2C(=O)OCCOC)C
ZINC08440718	5.47	-0.46	-11.59	0	7	0	505.611	11	CCCOc1ccc(cc1OC)[C@@H]2C3C(=O)C[C@H](C=C3N=C(C2C(=O)OCCOC)C)c4ccccc4
ZINC08440717	7.44	1.36	-10.71	0	6	0	537.656	9	CC1=NC2=C([C@@H](C1C(=O)OC(C)C)c3ccc(cc3)OCc4ccccc4)C(=O)C[C@@H](C2)c5ccc(cc5)OC
ZINC08440717	7.62	1.41	-11.82	0	6	0	537.656	9	CC(C)OC(=O)C1[C@H](C2C(=NC1=C)C[C@H](CC2=O)c3ccc(cc3)OC)c4ccc(cc4)OCc5ccccc5
ZINC08440717	7.44	1.29	-14.41	0	6	0	537.656	9	CC1=C([C@H]([C@@H]2C(=N1)C[C@H](CC2=O)c3ccc(cc3)OC)c4ccc(cc4)OCc5ccccc5)C(=O)OC(C)C
ZINC00702702	4.93	12.27	-47.96	0	6	-1	468.529	7	Cc1ccc(cc1)C(=O)C2=C(C(=O)N([C@@H]2c3cccc(c3)Oc4ccccc4)C[C@@H]5CCCO5)[O-]
ZINC00702702	5.38	-0.6	-29.18	1	6	0	469.537	6	Cc1ccc(cc1)C(=C2[C@H](N(C(=O)C2=O)C[C@@H]3CCCO3)c4cccc(c4)Oc5ccccc5)O
ZINC00702702	4.35	-0.34	-23.38	0	6	0	469.537	7	Cc1ccc(cc1)C(=O)[C@H]2[C@H](N(C(=O)C2=O)C[C@@H]3CCCO3)c4cccc(c4)Oc5ccccc5
ZINC08440716	7.44	1.25	-10.99	0	6	0	537.656	9	CC1=NC2=C([C@H](C1C(=O)OC(C)C)c3ccc(cc3)OCc4ccccc4)C(=O)C[C@@H](C2)c5ccc(cc5)OC
ZINC08440716	7.62	1.32	-12.47	0	6	0	537.656	9	CC(C)OC(=O)C1[C@@H](C2C(=NC1=C)C[C@H](CC2=O)c3ccc(cc3)OC)c4ccc(cc4)OCc5ccccc5
ZINC08440716	7.62	2.67	-18.42	0	6	0	537.656	9	CC(C)OC(=O)C1[C@@H]([C@H]2C(=NC1=C)C[C@H](CC2=O)c3ccc(cc3)OC)c4ccc(cc4)OCc5ccccc5
ZINC08440716	7.44	1.36	-14.53	0	6	0	537.656	9	CC1=C([C@@H]([C@H]2C(=N1)C[C@H](CC2=O)c3ccc(cc3)OC)c4ccc(cc4)OCc5ccccc5)C(=O)OC(C)C
ZINC08440715	7.44	1.28	-10.93	0	6	0	537.656	9	CC1=NC2=C([C@@H](C1C(=O)OC(C)C)c3ccc(cc3)OCc4ccccc4)C(=O)C[C@H](C2)c5ccc(cc5)OC
ZINC08440715	7.62	1.41	-12.21	0	6	0	537.656	9	CC(C)OC(=O)C1[C@H](C2C(=NC1=C)C[C@@H](CC2=O)c3ccc(cc3)OC)c4ccc(cc4)OCc5ccccc5
ZINC08440715	7.62	2.52	-12.22	0	6	0	537.656	9	CC(C)OC(=O)C1[C@H]([C@@H]2C(=NC1=C)C[C@@H](CC2=O)c3ccc(cc3)OC)c4ccc(cc4)OCc5ccccc5
ZINC08440715	7.44	1.42	-14.27	0	6	0	537.656	9	CC1=C([C@H]([C@H]2C(=N1)C[C@@H](CC2=O)c3ccc(cc3)OC)c4ccc(cc4)OCc5ccccc5)C(=O)OC(C)C
ZINC08440714	7.44	1.48	-13.98	0	6	0	537.656	9	CC1=NC2=C([C@H](C1C(=O)OC(C)C)c3ccc(cc3)OCc4ccccc4)C(=O)C[C@H](C2)c5ccc(cc5)OC
ZINC08440714	7.62	2.72	-14.59	0	6	0	537.656	9	CC(C)OC(=O)C1[C@@H]([C@H]2C(=NC1=C)C[C@@H](CC2=O)c3ccc(cc3)OC)c4ccc(cc4)OCc5ccccc5
ZINC08440714	7.44	1.26	-15.49	0	6	0	537.656	9	CC1=C([C@@H]([C@H]2C(=N1)C[C@@H](CC2=O)c3ccc(cc3)OC)c4ccc(cc4)OCc5ccccc5)C(=O)OC(C)C
ZINC00702695	3.96	0.02	-15.05	1	7	0	459.498	7	Cc1ccc(o1)C(=O)C2=C(C(=O)N([C@H]2c3cccc(c3)Oc4ccccc4)C[C@H]5CCCO5)O
ZINC00702695	3.38	-0.19	-21.62	0	7	0	459.498	7	Cc1ccc(o1)C(=O)[C@@H]2[C@@H](N(C(=O)C2=O)C[C@H]3CCCO3)c4cccc(c4)Oc5ccccc5
ZINC08440713	5.86	12.35	-19.06	1	6	0	469.965	6	Cc1cc(ccc1Cl)OCc2ccc(o2)[C@H]3C4=C(CC(CC4=O)(C)C)NC(=C3C(=O)OC)C
ZINC08440713	5.97	0.45	-10.01	0	6	0	469.965	6	Cc1cc(ccc1Cl)OCc2ccc(o2)[C@H]3C4C(=O)CC(C=C4N=C(C3C(=O)OC)C)(C)C
ZINC00702696	3.96	0.74	-17.32	1	7	0	459.498	7	Cc1ccc(o1)C(=O)C2=C(C(=O)N([C@@H]2c3cccc(c3)Oc4ccccc4)C[C@H]5CCCO5)O
ZINC00702696	3.38	0.68	-24.75	0	7	0	459.498	7	Cc1ccc(o1)C(=O)[C@@H]2[C@H](N(C(=O)C2=O)C[C@H]3CCCO3)c4cccc(c4)Oc5ccccc5
ZINC06148778	7.32	0.95	-9.9	0	5	0	514.021	7	CC1=NC2=C([C@@H](C1C(=O)OC)c3ccc(cc3)OCc4ccccc4)C(=O)C[C@@H](C2)c5ccc(cc5)Cl
ZINC06148778	7.5	1.19	-11.16	0	5	0	514.021	7	COC(=O)C1[C@H](C2C(=NC1=C)C[C@H](CC2=O)c3ccc(cc3)Cl)c4ccc(cc4)OCc5ccccc5
ZINC06148778	7.5	2.41	-17.11	0	5	0	514.021	7	COC(=O)C1[C@H]([C@@H]2C(=NC1=C)C[C@H](CC2=O)c3ccc(cc3)Cl)c4ccc(cc4)OCc5ccccc5
ZINC06148778	7.32	1.06	-13.64	0	5	0	514.021	7	CC1=C([C@H]([C@H]2C(=N1)C[C@H](CC2=O)c3ccc(cc3)Cl)c4ccc(cc4)OCc5ccccc5)C(=O)OC
ZINC00702697	3.96	0.28	-15.05	1	7	0	459.498	7	Cc1ccc(o1)C(=O)C2=C(C(=O)N([C@H]2c3cccc(c3)Oc4ccccc4)C[C@@H]5CCCO5)O
ZINC00702697	3.38	0.09	-20.83	0	7	0	459.498	7	Cc1ccc(o1)C(=O)[C@@H]2[C@@H](N(C(=O)C2=O)C[C@@H]3CCCO3)c4cccc(c4)Oc5ccccc5
ZINC08440712	4.76	-0.45	-14.46	1	7	0	463.53	7	CCOC(=O)C1[C@@H](C2=C(C[C@@H](CC2=O)c3ccc(cc3)OC)N=C1C)c4ccc(c(c4)OC)O
ZINC08440712	4.76	-0.81	-16.7	1	7	0	463.53	7	CCOC(=O)C1=C(N=C2C[C@@H](CC(=O)C2[C@H]1c3ccc(c(c3)OC)O)c4ccc(cc4)OC)C
ZINC08440712	4.95	-1.68	-14.8	1	7	0	463.53	7	CCOC(=O)C1[C@@H](C2C(=NC1=C)C[C@@H](CC2=O)c3ccc(cc3)OC)c4ccc(c(c4)OC)O
ZINC08440711	4.76	-0.56	-12	1	7	0	463.53	7	CCOC(=O)C1[C@H](C2=C(C[C@@H](CC2=O)c3ccc(cc3)OC)N=C1C)c4ccc(c(c4)OC)O
ZINC08440711	4.76	-0.64	-14.81	1	7	0	463.53	7	CCOC(=O)C1=C(N=C2C[C@@H](CC(=O)C2[C@@H]1c3ccc(c(c3)OC)O)c4ccc(cc4)OC)C
ZINC08440711	4.95	-1.5	-13.1	1	7	0	463.53	7	CCOC(=O)C1[C@H](C2C(=NC1=C)C[C@@H](CC2=O)c3ccc(cc3)OC)c4ccc(c(c4)OC)O
ZINC00702698	3.96	-0.02	-14.99	1	7	0	459.498	7	Cc1ccc(o1)C(=O)C2=C(C(=O)N([C@@H]2c3cccc(c3)Oc4ccccc4)C[C@@H]5CCCO5)O
ZINC00702698	3.38	-0.21	-21.37	0	7	0	459.498	7	Cc1ccc(o1)C(=O)[C@H]2[C@H](N(C(=O)C2=O)C[C@@H]3CCCO3)c4cccc(c4)Oc5ccccc5
ZINC08440710	5.86	12.36	-18.58	1	6	0	469.965	6	Cc1cc(ccc1Cl)OCc2ccc(o2)[C@@H]3C4=C(CC(CC4=O)(C)C)NC(=C3C(=O)OC)C
ZINC08440710	5.97	0.1	-9.62	0	6	0	469.965	6	Cc1cc(ccc1Cl)OCc2ccc(o2)[C@@H]3C4C(=O)CC(C=C4N=C(C3C(=O)OC)C)(C)C
ZINC08440709	4.76	-0.38	-11.36	1	7	0	463.53	7	CCOC(=O)C1[C@@H](C2=C(C[C@H](CC2=O)c3ccc(cc3)OC)N=C1C)c4ccc(c(c4)OC)O
ZINC08440709	4.76	-0.65	-15.2	1	7	0	463.53	7	CCOC(=O)C1=C(N=C2C[C@H](CC(=O)C2[C@H]1c3ccc(c(c3)OC)O)c4ccc(cc4)OC)C
ZINC08440709	4.95	-1.76	-12.99	1	7	0	463.53	7	CCOC(=O)C1[C@@H](C2C(=NC1=C)C[C@H](CC2=O)c3ccc(cc3)OC)c4ccc(c(c4)OC)O
ZINC08440708	4.76	-0.39	-11.59	1	7	0	463.53	7	CCOC(=O)C1[C@H](C2=C(C[C@H](CC2=O)c3ccc(cc3)OC)N=C1C)c4ccc(c(c4)OC)O
ZINC08440708	4.76	-0.59	-15.44	1	7	0	463.53	7	CCOC(=O)C1=C(N=C2C[C@H](CC(=O)C2[C@@H]1c3ccc(c(c3)OC)O)c4ccc(cc4)OC)C
ZINC08440708	4.95	-1.49	-12.95	1	7	0	463.53	7	CCOC(=O)C1[C@H](C2C(=NC1=C)C[C@H](CC2=O)c3ccc(cc3)OC)c4ccc(c(c4)OC)O
ZINC05918861	4.93	0.91	-13.85	1	7	0	540.41	10	CCCCOc1ccc(cc1OC)[C@H]2C(=C(C(=O)N2Cc3ccco3)O)C(=O)c4ccc(cc4)Br
ZINC05918861	4.34	0.8	-20.07	0	7	0	540.41	10	CCCCOc1ccc(cc1OC)[C@H]2[C@H](C(=O)C(=O)N2Cc3ccco3)C(=O)c4ccc(cc4)Br
ZINC08440707	7.68	0.34	-8.04	0	4	0	510.461	5	CC1=NC2=C([C@@H](C1C(=O)OC3CCCCC3)c4cccc(c4Cl)Cl)C(=O)C[C@@H](C2)c5ccccc5
ZINC08440707	7.87	0.57	-9.13	0	4	0	510.461	5	C=C1C([C@H](C2C(=N1)C[C@H](CC2=O)c3ccccc3)c4cccc(c4Cl)Cl)C(=O)OC5CCCCC5
ZINC08440707	7.87	1.56	-8.73	0	4	0	510.461	5	C=C1C([C@H]([C@@H]2C(=N1)C[C@H](CC2=O)c3ccccc3)c4cccc(c4Cl)Cl)C(=O)OC5CCCCC5
ZINC08440707	7.68	0.46	-11.31	0	4	0	510.461	5	CC1=C([C@H]([C@H]2C(=N1)C[C@H](CC2=O)c3ccccc3)c4cccc(c4Cl)Cl)C(=O)OC5CCCCC5
ZINC08440706	7.68	0.21	-8.03	0	4	0	510.461	5	CC1=NC2=C([C@H](C1C(=O)OC3CCCCC3)c4cccc(c4Cl)Cl)C(=O)C[C@@H](C2)c5ccccc5
ZINC08440706	7.87	0.3	-8.76	0	4	0	510.461	5	C=C1C([C@@H](C2C(=N1)C[C@H](CC2=O)c3ccccc3)c4cccc(c4Cl)Cl)C(=O)OC5CCCCC5
ZINC08440706	7.87	1.62	-16.48	0	4	0	510.461	5	C=C1C([C@@H]([C@H]2C(=N1)C[C@H](CC2=O)c3ccccc3)c4cccc(c4Cl)Cl)C(=O)OC5CCCCC5
ZINC08440706	7.68	0.35	-11.54	0	4	0	510.461	5	CC1=C([C@@H]([C@@H]2C(=N1)C[C@H](CC2=O)c3ccccc3)c4cccc(c4Cl)Cl)C(=O)OC5CCCCC5
ZINC08440705	7.68	0.37	-8.15	0	4	0	510.461	5	CC1=NC2=C([C@@H](C1C(=O)OC3CCCCC3)c4cccc(c4Cl)Cl)C(=O)C[C@H](C2)c5ccccc5

Supplementary Table S2:
Detailed table of XP Docking using Schrodinger's Glide module

ZINC_ID	xLogP	ApolarDesolvation	PolarDesolvation	H Donors	H Acceptors	Charge	MolecularWeight	RotatableBonds	SMILES
ZINC08440705	7.87	0.39	-8.95	0	4	0	510.461	5	C=C1C([C@H](C2C(=N1)C[C@@H](CC2=O)c3ccccc3)c4cccc(c4Cl)Cl)C(=O)OC5CCCCC5
ZINC08440705	7.87	1.67	-9.45	0	4	0	510.461	5	C=C1C([C@H]([C@@H]2C(=N1)C[C@@H](CC2=O)c3ccccc3)c4cccc(c4Cl)Cl)C(=O)OC5CCCCC5
ZINC08440705	7.68	0.43	-11.38	0	4	0	510.461	5	CC1=C([C@H]([C@H]2C(=N1)C[C@@H](CC2=O)c3ccccc3)c4cccc(c4Cl)Cl)C(=O)OC5CCCCC5
ZINC08440704	4.43	9.14	-42.87	0	5	-1	431.306	3	COc1cc(cc(c1OC)Br)[C@H]2C3=C(CCCC3=O)N=C4C2=C(CCC4)[O-]
ZINC08440704	4.01	-2.27	-12.29	0	5	0	432.314	3	COc1cc(cc(c1OC)Br)[C@H]2C3C(=NC4=CCCC(=O)C24)CCCC3=O
ZINC08440703	7.68	0.23	-7.76	0	4	0	510.461	5	CC1=NC2=C([C@H](C1C(=O)OC3CCCCC3)c4cccc(c4Cl)Cl)C(=O)C[C@H](C2)c5ccccc5
ZINC08440703	7.87	0.41	-8.8	0	4	0	510.461	5	C=C1C([C@@H](C2C(=N1)C[C@@H](CC2=O)c3ccccc3)c4cccc(c4Cl)Cl)C(=O)OC5CCCCC5
ZINC08440703	7.87	1.56	-8.38	0	4	0	510.461	5	C=C1C([C@@H]([C@H]2C(=N1)C[C@@H](CC2=O)c3ccccc3)c4cccc(c4Cl)Cl)C(=O)OC5CCCCC5
ZINC08440703	7.68	0.52	-10.84	0	4	0	510.461	5	CC1=C([C@@H]([C@@H]2C(=N1)C[C@@H](CC2=O)c3ccccc3)c4cccc(c4Cl)Cl)C(=O)OC5CCCCC5
ZINC02063157	8.19	11.96	-10.05	1	5	0	462.977	10	CCCCCCCOc1ccc(cc1)C(=O)Nc2ccc3c(c2)nc(o3)c4ccc(cc4)Cl
ZINC05918802	4.93	0.62	-15.29	1	7	0	540.41	10	CCCCOc1ccc(cc1OC)[C@@H]2C(=C(C(=O)N2Cc3ccco3)O)C(=O)c4ccc(cc4)Br
ZINC05918802	4.34	0.53	-23.03	0	7	0	540.41	10	CCCCOc1ccc(cc1OC)[C@@H]2[C@@H](C(=O)C(=O)N2Cc3ccco3)C(=O)c4ccc(cc4)Br
ZINC02063155	6.41	2.06	-8.02	0	3	0	439.627	3	Cc1ccc(cc1)/C=C/C(=O)N2c3cc(ccc3-c4c(ssc4=S)C2(C)C)OC
ZINC00702689	2.94	9.16	-56.74	0	7	-1	454.474	8	CCOc1ccc(cc1OC)[C@H]2C(=C(C(=O)N2C[C@H]3CCCO3)[O-])C(=O)c4ccc(cc4)F
ZINC00702689	2.36	8.95	-18.71	0	7	0	455.482	8	CCOc1ccc(cc1OC)[C@H]2[C@H](C(=O)C(=O)N2C[C@H]3CCCO3)C(=O)c4ccc(cc4)F
ZINC00702689	3.38	7.99	-27.02	1	7	0	455.482	7	CCOc1ccc(cc1OC)[C@H]2C(=C(c3ccc(cc3)F)O)C(=O)C(=O)N2C[C@H]4CCCO4
ZINC00702690	2.94	8.98	-58.25	0	7	-1	454.474	8	CCOc1ccc(cc1OC)[C@@H]2C(=C(C(=O)N2C[C@H]3CCCO3)[O-])C(=O)c4ccc(cc4)F
ZINC00702690	2.36	8.97	-22.64	0	7	0	455.482	8	CCOc1ccc(cc1OC)[C@@H]2[C@H](C(=O)C(=O)N2C[C@H]3CCCO3)C(=O)c4ccc(cc4)F
ZINC00702690	3.38	7.97	-32.2	1	7	0	455.482	7	CCOc1ccc(cc1OC)[C@@H]2C(=C(c3ccc(cc3)F)O)C(=O)C(=O)N2C[C@H]4CCCO4
ZINC08440702	6.88	4.64	-16.3	0	7	0	581.737	7	Cc1ccc(cc1)/C=C/C(=O)N2c3ccc(cc3C(=C4SC(=C(S4)C(=O)OC)C(=O)OC)C(=S)C2(C)C)OC
ZINC00702692	2.94	9.72	-53.65	0	7	-1	454.474	8	CCOc1ccc(cc1OC)[C@@H]2C(=C(C(=O)N2C[C@@H]3CCCO3)[O-])C(=O)c4ccc(cc4)F
ZINC00702692	3.38	7.47	-31.95	1	7	0	455.482	7	CCOc1ccc(cc1OC)[C@@H]2C(=C(c3ccc(cc3)F)O)C(=O)C(=O)N2C[C@@H]4CCCO4
ZINC00702692	2.36	8.46	-26.34	0	7	0	455.482	8	CCOc1ccc(cc1OC)[C@@H]2[C@@H](C(=O)C(=O)N2C[C@@H]3CCCO3)C(=O)c4ccc(cc4)F
ZINC02063153	6.41	2.07	-10.68	0	3	0	439.627	3	Cc1ccc(cc1)/C=C/C(=O)N2c3cc(cc3-c4c(ssc4=S)C2(C)C)OC
ZINC08440701	7.43	3.46	-16.45	0	7	0	624.589	7	CCOc1cccc2c1N(C(C(=S)C2=C3SC(=C(S3)C(=O)OC)C(=O)OC)(C)C)C(=O)c4ccc(cc4Cl)Cl
ZINC08440700	7.85	3.77	-15.05	0	6	0	608.59	5	Cc1cc2c(cc1C)N(C(C(=S)C2=C3SC(=C(S3)C(=O)OC)C(=O)OC)(C)C)C(=O)c4ccc(cc4Cl)Cl
ZINC08440699	3.34	-0.93	-64.19	0	7	-1	444.891	6	CCOc1cc(cc(c1OCC(=O)[O-])Cl)[C@@H]2C3C(=NC4=C2C(=O)CCC4)CCCC3=O
ZINC08440699	3.5	-2.14	-59.94	0	7	-1	444.891	6	CCOc1cc(cc(c1OCC(=O)[O-])Cl)[C@H]2C3C(=NC4=CCCC(=O)C24)CCCC3=O
ZINC00702685	4.48	11.77	-45.12	0	8	-1	522.618	12	CCOc1ccc(cc1)C(=O)C2=C(C(=O)N([C@H]2c3ccc(c(c3)OC)OCCC(C)C)C[C@H]4CCCO4)[O-]
ZINC00702685	4.93	-0.31	-26.57	1	8	0	523.626	11	CCOc1ccc(cc1)C(=C2[C@@H](N(C(=O)C2=O)C[C@H]3CCCO3)c4ccc(c(c4)OC)OCCC(C)C)O
ZINC00702685	3.9	-0.02	-19.45	0	8	0	523.626	12	CCOc1ccc(cc1)C(=O)[C@@H]2[C@@H](N(C(=O)C2=O)C[C@H]3CCCO3)c4ccc(c(c4)OC)OCCC(C)C
ZINC00666637	5.33	-0.12	-9.22	0	4	0	429.588	3	CC1(c2c(c(=S)ss2)-c3cccc(c3N1C(=O)c4cccc(c4)OC)OC)C
ZINC01810652	6.74	0.26	-20.26	0	3	0	462.061	3	Cc1cc-2c(cc1C)N(C(c3c2c(=S)ss3)(C)C)C(=O)COc4ccc(cc4)Cl
ZINC00702686	4.48	11.72	-46.58	0	8	-1	522.618	12	CCOc1ccc(cc1)C(=O)C2=C(C(=O)N([C@@H]2c3ccc(c(c3)OC)OCCC(C)C)C[C@H]4CCCO4)[O-]
ZINC00702686	4.93	10.35	-32.38	1	8	0	523.626	11	CCOc1ccc(cc1)C(=C2[C@H](N(C(=O)C2=O)C[C@H]3CCCO3)c4ccc(c(c4)OC)OCCC(C)C)O
ZINC00702686	3.9	11.39	-22.57	0	8	0	523.626	12	CCOc1ccc(cc1)C(=O)[C@@H]2[C@H](N(C(=O)C2=O)C[C@H]3CCCO3)c4ccc(c(c4)OC)OCCC(C)C
ZINC08440698	6.61	3.73	-7.13	0	2	0	451.56	2	Cc1ccc2c(c1)-c3c(ssc3=S)C(N2C(=O)c4ccc(cc4)C(F)(F)F)(C)C
ZINC02063152	7.47	0.47	-8.05	0	3	0	489.687	4	CCOc1cccc-2c1N(C(c3c2c(=S)ss3)(C)C)C(=O)c4ccc(cc4)c5ccccc5
ZINC00702687	4.48	11.43	-46.09	0	8	-1	522.618	12	CCOc1ccc(cc1)C(=O)C2=C(C(=O)N([C@H]2c3ccc(c(c3)OC)OCCC(C)C)C[C@@H]4CCCO4)[O-]
ZINC00702687	4.93	-0.03	-28.17	1	8	0	523.626	11	CCOc1ccc(cc1)C(=C2[C@@H](N(C(=O)C2=O)C[C@@H]3CCCO3)c4ccc(c(c4)OC)OCCC(C)C)O
ZINC00702687	3.9	0.24	-23.63	0	8	0	523.626	12	CCOc1ccc(cc1)C(=O)[C@@H]2[C@@H](N(C(=O)C2=O)C[C@@H]3CCCO3)c4ccc(c(c4)OC)OCCC(C)C
ZINC02063151	6.96	-0.33	-6.49	0	3	0	482.479	3	CCOc1cccc-2c1N(C(c3c2c(=S)ss3)(C)C)C(=O)c4ccc(cc4Cl)Cl
ZINC02063150	7.37	-0.13	-6.69	0	2	0	466.48	1	Cc1cc-2c(cc1C)N(C(c3c2c(=S)ss3)(C)C)C(=O)c4ccc(cc4Cl)Cl
ZINC02063149	6.57	-0.69	-6.29	0	2	0	438.426	1	CC1(c2c(c(=S)ss2)-c3ccccc3N1C(=O)c4ccc(cc4Cl)Cl)C
ZINC02063148	7.16	2.13	-18.62	0	3	0	500.761	3	Cc1cc(c2c(c1)-c3c(ssc3=S)C(N2C(=O)CSc4nc5ccccc5s4)(C)C)C
ZINC00702688	4.48	11.33	-45.27	0	8	-1	522.618	12	CCOc1ccc(cc1)C(=O)C2=C(C(=O)N([C@@H]2c3ccc(c(c3)OC)OCCC(C)C)C[C@@H]4CCCO4)[O-]
ZINC00702688	4.93	-0.67	-31.67	1	8	0	523.626	11	CCOc1ccc(cc1)C(=C2[C@H](N(C(=O)C2=O)C[C@@H]3CCCO3)c4ccc(c(c4)OC)OCCC(C)C)O
ZINC00702688	3.9	-0.41	-27.09	0	8	0	523.626	12	CCOc1ccc(cc1)C(=O)[C@H]2[C@H](N(C(=O)C2=O)C[C@@H]3CCCO3)c4ccc(c(c4)OC)OCCC(C)C
ZINC01808753	7.47	3.22	-12.96	0	4	0	523.745	6	CCCC(=O)N1c2cc(ccc2/C(=C/3\SC(=C(S3)C(=O)OCC)c4ccccc4)/C(=S)C1(C)C)C
ZINC02063145	9.74	4.99	-11.59	0	4	0	549.67	6	CC1=CC(N(c2c1cc(cc2)OC(=O)c3ccc(cc3)c4ccccc4)C(=O)c5ccc(cc5)c6ccccc6)(C)C
ZINC00970719	6.26	4	-11.35	0	6	0	457.526	6	CC1=CC(N(c2c1cc(cc2)OC(=O)c3ccc(cc3)OC)C(=O)c4ccc(cc4)OC)(C)C
ZINC08440694	6.82	3.45	-14.47	0	6	0	559.775	7	Cc1ccc2c(c1)N(C(C(=S)C2=C3SC(=C(S3)C(=O)OC)C(=O)OC)(C)C)C(=O)CCC4CCCCC4
ZINC08440693	6.38	16.04	-21.8	2	6	0	539.701	7	Cc1ccc(cc1)SCc2cc(ccc2OC)[C@H]3C4=C(CCCC4=O)NC(=C3C(=O)Nc5c(cccn5)C)C
ZINC08440693	6.5	-2.5	-14.24	1	6	0	539.701	7	Cc1ccc(cc1)SCc2cc(ccc2OC)[C@H]3C4C(=O)CCC=C4N=C(C3C(=O)Nc5c(cccn5)C)C
ZINC00702684	4.5	12.46	-52.59	0	7	-1	492.523	10	CC(C)CCOc1ccc(cc1OC)[C@@H]2C(=C(C(=O)N2Cc3ccco3)[O-])C(=O)c4ccc(cc4)F
ZINC00702684	4.94	2.05	-28.65	1	7	0	493.531	9	CC(C)CCOc1ccc(cc1OC)[C@@H]2C(=C(c3ccc(cc3)F)O)C(=O)C(=O)N2Cc4ccco4
ZINC00702684	3.91	2.34	-23.62	0	7	0	493.531	10	CC(C)CCOc1ccc(cc1OC)[C@@H]2[C@@H](C(=O)C(=O)N2Cc3ccco3)C(=O)c4ccc(cc4)F
ZINC08440692	7.2	4.14	-15.64	0	6	0	573.802	7	Cc1cc2c(cc1C)N(C(C(=S)C2=C3SC(=C(S3)C(=O)OC)C(=O)OC)(C)C)C(=O)CCC4CCCCC4
ZINC02063143	6.33	1.34	-9.94	0	3	0	447.691	5	CCOc1ccc2c(c1)-c3c(ssc3=S)C(N2C(=O)CCC4CCCCC4)(C)C
ZINC02063142	5.95	13.32	-10.04	0	3	0	433.664	4	CC1(c2c(c(=S)ss2)-c3cc(ccc3N1C(=O)CCC4CCCCC4)OC)C
ZINC02063141	6.72	0.29	-16.41	0	2	0	431.692	3	Cc1cc-2c(cc1C)N(C(c3c2c(=S)ss3)(C)C)C(=O)CCC4CCCCC4

Supplementary Table S2:
Detailed table of XP Docking using Schrodinger's Glide module

ZINC_ID	xLogP	ApolarDesolvation	PolarDesolvation	H Donors	H Acceptors	Charge	MolecularWeight	RotatableBonds	SMILES
ZINC00702681	4.77	12.57	-43.18	0	8	-1	518.586	12	CCOc1ccc(cc1)C(=O)C2=C(C(=O)N([C@H]2c3ccc(c(c3)OC)OCCC(C)C)Cc4ccco4)[O-]
ZINC00702681	5.21	1.67	-28.74	1	8	0	519.594	11	CCOc1ccc(cc1)C(=C2[C@@H](N(C(=O)C2=O)Cc3ccco3)c4ccc(c(c4)OC)OCCC(C)C)O
ZINC00702681	4.18	1.94	-21.62	0	8	0	519.594	12	CCOc1ccc(cc1)C(=O)[C@@H]2[C@@H](N(C(=O)C2=O)Cc3ccco3)c4ccc(c(c4)OC)OCCC(C)C
ZINC08440689	6.38	16.01	-20.87	2	6	0	539.701	7	Cc1ccc(cc1)SCc2cc(ccc2OC)[C@@H]3C4=C(CCCC4=O)NC(=C3C(=O)Nc5c(cccn5)C)C
ZINC08440689	6.5	-2.66	-18.79	1	6	0	539.701	7	Cc1ccc(cc1)SCc2cc(ccc2OC)[C@@H]3C4C(=O)CCC=C4N=C(C3C(=O)Nc5c(cccn5)C)C
ZINC05921188	4.77	11.72	-59.72	0	8	-1	518.586	12	CCOc1ccc(cc1)C(=O)C2=C(C(=O)N([C@@H]2c3ccc(c(c3)OC)OCCC(C)C)Cc4ccco4)[O-]
ZINC05921188	5.21	1.37	-28.75	1	8	0	519.594	11	CCOc1ccc(cc1)C(=C2[C@H](N(C(=O)C2=O)Cc3ccco3)c4ccc(c(c4)OC)OCCC(C)C)O
ZINC05921188	4.18	1.6	-22.42	0	8	0	519.594	12	CCOc1ccc(cc1)C(=O)[C@@H]2[C@H](N(C(=O)C2=O)Cc3ccco3)c4ccc(c(c4)OC)OCCC(C)C
ZINC01826409	6.32	2.54	-12.1	0	6	0	506.602	6	C[C@H]1C[C@H](c2ccccc2N1C(=O)c3ccc(cc3)OC)N(c4ccccc4)C(=O)c5ccc(cc5)OC
ZINC01826410	6.32	2.11	-14.23	0	6	0	506.602	6	C[C@@H]1C[C@H](c2ccccc2N1C(=O)c3ccc(cc3)OC)N(c4ccccc4)C(=O)c5ccc(cc5)OC
ZINC01826411	6.32	2.79	-19.15	0	6	0	506.602	6	C[C@H]1C[C@@H](c2ccccc2N1C(=O)c3ccc(cc3)OC)N(c4ccccc4)C(=O)c5ccc(cc5)OC
ZINC05918614	4.79	12.29	-55.15	0	7	-1	492.523	11	CCCCCOc1ccc(cc1OC)[C@H]2C(=C(C(=O)N2Cc3ccco3)[O-])C(=O)c4ccc(cc4)F
ZINC05918614	5.23	2.15	-28.65	1	7	0	493.531	10	CCCCCOc1ccc(cc1OC)[C@H]2C(=C(c3ccc(cc3)F)O)C(=O)C(=O)N2Cc4ccco4
ZINC05918614	4.2	2.42	-20.61	0	7	0	493.531	11	CCCCCOc1ccc(cc1OC)[C@H]2[C@H](C(=O)C(=O)N2Cc3ccco3)C(=O)c4ccc(cc4)F
ZINC01826412	6.32	2.59	-14.49	0	6	0	506.602	6	C[C@@H]1C[C@@H](c2ccccc2N1C(=O)c3ccc(cc3)OC)N(c4ccccc4)C(=O)c5ccc(cc5)OC
ZINC02254907	6.53	3.92	-8.85	0	4	0	482.53	4	C[C@H]1C[C@H](c2ccccc2N1C(=O)c3ccc(cc3)F)N(c4ccccc4)C(=O)c5ccc(cc5)F
ZINC02254908	6.53	3.64	-10.3	0	4	0	482.53	4	C[C@@H]1C[C@H](c2ccccc2N1C(=O)c3ccc(cc3)F)N(c4ccccc4)C(=O)c5ccc(cc5)F
ZINC05918609	4.79	11.83	-56.45	0	7	-1	492.523	11	CCCCCOc1ccc(cc1OC)[C@@H]2C(=C(C(=O)N2Cc3ccco3)[O-])C(=O)c4ccc(cc4)F
ZINC05918609	5.23	1.85	-28.71	1	7	0	493.531	10	CCCCCOc1ccc(cc1OC)[C@@H]2C(=C(c3ccc(cc3)F)O)C(=O)C(=O)N2Cc4ccco4
ZINC05918609	4.2	2.15	-23.6	0	7	0	493.531	11	CCCCCOc1ccc(cc1OC)[C@@H]2[C@@H](C(=O)C(=O)N2Cc3ccco3)C(=O)c4ccc(cc4)F
ZINC02254909	6.53	4.19	-15.05	0	4	0	482.53	4	C[C@H]1C[C@@H](c2ccccc2N1C(=O)c3ccc(cc3)F)N(c4ccccc4)C(=O)c5ccc(cc5)F
ZINC08440687	2.25	-3.39	-23.08	3	9	0	476.533	7	Cc1ccnc(c1)NC(=O)C2[C@H](C3=C(CCCC3=O)N=C2C)c4ccc(c(c4)OC)OCC(=O)N
ZINC08440687	2.43	-4.68	-25.36	3	9	0	476.533	7	Cc1ccnc(c1)NC(=O)C2[C@H](C3C(=NC2=C)CCCC3=O)c4ccc(c(c4)OC)OCC(=O)N
ZINC02254910	6.53	3.88	-9.34	0	4	0	482.53	4	C[C@@H]1C[C@@H](c2ccccc2N1C(=O)c3ccc(cc3)F)N(c4ccccc4)C(=O)c5ccc(cc5)F
ZINC05918671	4.56	0.96	-18.75	1	10	0	566.632	12	CCCCOc1ccc(cc1)C(=O)C2=C(C(=O)N([C@H]2c3ccc(c(c3)OC)OC)c4nc(c(s4)C(=O)OC)C)O
ZINC05918671	3.98	0.8	-23.2	0	10	0	566.632	12	CCCCOc1ccc(cc1)C(=O)[C@H]2[C@@H](N(C(=O)C2=O)c3nc(c(s3)C(=O)OC)C)c4ccc(c(c4)OC)OC
ZINC02063128	6.77	-1.71	-18.07	0	2	0	421.418	1	Cc1ccc2c(c1)-c3c(sn(c3=S)c4ccc(c(c4)Cl)Cl)C(N2C)(C)C
ZINC02063127	6.91	-1.44	-16.55	1	2	0	421.418	1	Cc1cc(c2c(c1)-c3c(sn(c3=S)c4ccc(c(c4)Cl)Cl)C(N2)(C)C)C
ZINC05918665	4.56	1.03	-18.4	1	10	0	566.632	12	CCCCOc1ccc(cc1)C(=O)C2=C(C(=O)N([C@@H]2c3ccc(c(c3)OC)OC)c4nc(c(s4)C(=O)OC)C)O
ZINC05918665	3.98	0.94	-24.57	0	10	0	566.632	12	CCCCOc1ccc(cc1)C(=O)[C@H]2[C@H](N(C(=O)C2=O)c3nc(c(s3)C(=O)OC)C)c4ccc(c(c4)OC)OC
ZINC00666632	5.95	-1.8	-16.57	1	5	0	449.304	5	COc1ccc(cc1Br)c2nc3cc(ccc3o2)NC(=O)/C=C/c4ccccc4
ZINC02063121	4.92	-0.88	-14.5	0	4	0	411.304	6	c1cc(ccc1N2CCN(CC2)C(=O)CCCOc3ccc(cc3Cl)Cl)F
ZINC05918675	4.16	10.93	-51.46	0	7	-1	466.514	8	CCCCOc1ccc(cc1)C(=O)C2=C(C(=O)N([C@H]2c3ccc(cc3)F)c4nnc(s4)C)[O-]
ZINC05918675	4.61	0.68	-25.65	1	7	0	467.522	7	CCCCOc1ccc(cc1)C(=C2[C@@H](N(C(=O)C2=O)c3nnc(s3)C)c4ccc(cc4)F)O
ZINC05918675	3.58	0.9	-16.71	0	7	0	467.522	8	CCCCOc1ccc(cc1)C(=O)[C@H]2[C@@H](N(C(=O)C2=O)c3nnc(s3)C)c4ccc(cc4)F
ZINC08440686	5.54	-2.37	-10.14	1	5	0	512.473	4	CC1=NC2=C([C@@H](C1C(=O)Nc3ccc(cn3)Br)c4ccc(cc4)SC)C(=O)CC(C2)(C)C
ZINC08440686	5.72	-2.38	-11.97	1	5	0	512.473	4	CC1(CC2=NC(=C)C([C@H](C2C(=O)C1)c3ccc(cc3)SC)C(=O)Nc4ccc(cn4)Br)C
ZINC08440686	5.72	-1.14	-10.5	1	5	0	512.473	4	CC1(CC2=NC(=C)C([C@H]([C@H]2C(=O)C1)c3ccc(cc3)SC)C(=O)Nc4ccc(cn4)Br)C
ZINC02063120	6.46	-0.38	-17.99	1	7	0	496.563	9	CCOc1cc(cc(c1OCC)OCC)C(=O)Nc2ccc(cc2)c3nc4c5ccccc5ccc4o3
ZINC00847195	4.55	-0.13	-15.15	1	7	0	430.526	10	CCOc1ccc2c(c1)sc(n2)NC(=O)c3cc(c(c(c3)OCC)OCC)OCC
ZINC00666601	5.8	1.25	-11.75	1	6	0	428.879	4	Cc1ccc(cc1)n2nc3ccc(cc3n2)NC(=O)c4ccc(o4)c5ccc(cc5)Cl
ZINC02063119	7.05	-1.17	-9.21	1	5	0	449.293	4	c1cc(ccc1c2ccc(o2)C(=O)Nc3ccc4c(c3)nc(o4)c5ccc(cc5)Cl)Cl
ZINC00666597	5.06	1.94	-17.94	1	9	0	439.431	5	Cc1ccc(cc1)n2nc3ccc(cc3n2)NC(=O)c4ccc(o4)c5cccc(c5)[N+](=O)[O-]
ZINC05918668	4.16	10.95	-51.53	0	7	-1	466.514	8	CCCCOc1ccc(cc1)C(=O)C2=C(C(=O)N([C@@H]2c3ccc(cc3)F)c4nnc(s4)C)[O-]
ZINC05918668	4.61	0.66	-25.99	1	7	0	467.522	7	CCCCOc1ccc(cc1)C(=C2[C@H](N(C(=O)C2=O)c3nnc(s3)C)c4ccc(cc4)F)O
ZINC05918668	3.58	0.96	-18.34	0	7	0	467.522	8	CCCCOc1ccc(cc1)C(=O)[C@H]2[C@H](N(C(=O)C2=O)c3nnc(s3)C)c4ccc(cc4)F
ZINC08440684	8.08	2.95	-11.37	1	6	0	501.671	7	CC(C)(C)c1cc(cc(c1O)C(C)(C)C)n2c3cc(c(cc3nc2c4ccc(cc4)N(C)C)OC)OC
ZINC08440683	9.14	1.88	-8.11	1	3	0	477.446	4	CC(C)(C)c1cc(cc(c1O)C(C)(C)C)n2c3ccccc3nc2c4cccc(c4)Br
ZINC00666595	5.29	-0.54	-16.42	1	5	0	461.612	5	Cc1ccc(c(c1)NC(=O)CSc2nc3c(c4c(s3)CCC4)c(=O)n2c5ccccc5)C
ZINC00632059	4.63	-0.69	-17.16	1	5	0	451.548	5	c1ccc(cc1)n2c(=O)c3c4c(sc3nc2SCC(=O)Nc5ccc(cc5)F)CCC4
ZINC02063118	5.27	-2.19	-16.5	1	5	0	512.454	5	c1ccc(cc1)n2c(=O)c3c4c(sc3nc2SCC(=O)Nc5ccc(cc5)Br)CCC4
ZINC08440682	5.54	12.36	-19.11	1	5	0	512.473	4	CC1=C([C@@H]([C@@H]2C(=N1)CC(CC2=O)(C)C)c3ccc(cc3)SC)C(=O)Nc4ccc(cn4)Br
ZINC08440682	5.54	-1.64	-13.91	1	5	0	512.473	4	CC1=C([C@@H]([C@H]2C(=N1)CC(CC2=O)(C)C)c3ccc(cc3)SC)C(=O)Nc4ccc(cn4)Br
ZINC08440682	5.72	-1.95	-11.58	1	5	0	512.473	4	CC1(CC2=NC(=C)C([C@@H](C2C(=O)C1)c3ccc(cc3)SC)C(=O)Nc4ccc(cn4)Br)C
ZINC08440682	5.72	-1.13	-12.08	1	5	0	512.473	4	CC1(CC2=NC(=C)C([C@@H]([C@@H]2C(=O)C1)c3ccc(cc3)SC)C(=O)Nc4ccc(cn4)Br)C
ZINC00702673	3.1	10.72	-37.76	0	8	-1	480.497	7	CCOC(=O)c1c(nc(s1)N2[C@H](C(=C(C2=O)[O-])C(=O)c3ccc(c(c3)F)C)c4cccnc4)C
ZINC00702673	3.55	0.19	-33.1	1	8	0	481.505	6	CCOC(=O)c1c(nc(s1)N2[C@H](C(=C(c3ccc(c(c3)F)C)O)C(=O)C2=O)c4cccnc4)C
ZINC00702673	2.52	0.39	-25.33	0	8	0	481.505	7	CCOC(=O)c1c(nc(s1)N2[C@H]([C@@H](C(=O)C2=O)C(=O)c3ccc(c(c3)F)C)c4cccnc4)C
ZINC08440681	4.91	-1.12	-16.63	1	5	0	447.585	5	Cc1ccc(cc1)NC(=O)CSc2nc3c(c4c(s3)CCC4)c(=O)n2c5ccccc5
ZINC08440680	7.89	-0.58	-14.67	0	4	0	557.549	5	c1ccc2c(c1)cccc2n3c(=O)c4c5c(sc4nc3SCC(=O)c6cc(sc6Cl)Cl)CCCC5
ZINC08440679	2.28	-1.58	-58.95	2	8	1	481.318	5	Cn1cc(c(n1)C(=O)Nc2nc3ccccc3n2CC[NH+]4CCOCC4)I

Supplementary Table S2:
Detailed table of XP Docking using Schrodinger's Glide module

ZINC_ID	xLogP	ApolarDesolvation	PolarDesolvation	H Donors	H Acceptors	Charge	MolecularWeight	RotatableBonds	SMILES
ZINC08440678	7.18	-0.05	-17.57	1	5	0	525.699	5	Cc1ccc(c(c1)NC(=O)CSc2nc3c(c4c(s3)CCCC4)c(=O)n2c5cccc6c5cccc6)C
ZINC00702674	3.1	10.22	-60.18	0	8	-1	480.497	7	CCOC(=O)c1c(nc(s1)N2[C@@H](C(=C(C2=O)[O-])C(=O)c3ccc(c(c3)F)C)c4cccnc4)C
ZINC02063116	7.16	-1.67	-17.36	1	5	0	576.541	5	c1ccc2c(c1)cccc2n3c(=O)c4c5c(sc4nc3SCC(=O)Nc6ccc(cc6)Br)CCCC5
ZINC08440677	3.9	0.21	-16.77	1	8	0	475.545	7	Cc1cccnc1NC(=O)C2[C@@H](C3=C(CCCC3=O)N=C2C)c4ccc(c(c4)COC(=O)C)OC
ZINC08440677	3.9	0.13	-21.68	1	8	0	475.545	7	Cc1cccnc1NC(=O)C2=C(N=C3CCCC(=O)C3[C@H]2c4ccc(c(c4)COC(=O)C)OC)C
ZINC08440677	4.08	-0.98	-19.12	1	8	0	475.545	7	Cc1cccnc1NC(=O)C2[C@@H](C3C(=NC2=C)CCCC3=O)c4ccc(c(c4)COC(=O)C)OC
ZINC08440676	3.5	9.69	-54.99	0	6	-1	424.448	10	COCCCN1[C@H](C(=C(C1=O)[O-])C(=O)c2ccc(cc2)OCC=C)c3ccc(cc3)F
ZINC08440676	3.94	0.77	-28.28	1	6	0	425.456	9	COCCCN1[C@H](C(=C(c2ccc(cc2)OCC=C)O)C(=O)C1=O)c3ccc(cc3)F
ZINC08440676	2.91	1.06	-22.17	0	6	0	425.456	10	COCCCN1[C@H]([C@H](C(=O)C1=O)C(=O)c2ccc(cc2)OCC=C)c3ccc(cc3)F
ZINC02063115	6.8	-0.59	-17.63	1	5	0	511.672	5	Cc1ccc(cc1)NC(=O)CSc2nc3c(c4c(s3)CCCC4)c(=O)n2c5cccc6c5cccc6
ZINC08440675	8.43	0.92	-15.95	1	4	0	559.713	8	Cc1ccc2c(c1)c(c(n2C[C@H](Cn3cnc(c3c4ccccc4)c5ccccc5)O)c6ccccc6)c7ccccc7
ZINC08440673	3.5	9.64	-55.16	0	6	-1	424.448	10	COCCCN1[C@@H](C(=C(C1=O)[O-])C(=O)c2ccc(cc2)OCC=C)c3ccc(cc3)F
ZINC08440673	3.94	0.49	-30.3	1	6	0	425.456	9	COCCCN1[C@@H](C(=C(c2ccc(cc2)OCC=C)O)C(=O)C1=O)c3ccc(cc3)F
ZINC08440673	2.91	0.75	-25.79	0	6	0	425.456	10	COCCCN1[C@@H]([C@@H](C(=O)C1=O)C(=O)c2ccc(cc2)OCC=C)c3ccc(cc3)F
ZINC02063114	6.91	2.15	-16.9	0	3	0	415.901	3	c1ccc(cc1)c2ccc(cc2)c3csc(n3)c4cc5cc(ccc5oc4=O)Cl
ZINC09057742	8.49	18.57	-13.32	1	3	0	476.579	6	c1ccc(cc1)c2ccc(cc2)c3[nH]c(c(n3)c4ccccc4)c5ccc(cc5)C(=O)c6ccccc6
ZINC09057742	8.49	18.58	-12.3	1	3	0	476.579	6	c1ccc(cc1)c2ccc(cc2)c3[nH]c(c(n3)c4ccc(cc4)C(=O)c5ccccc5)c6ccccc6
ZINC09057742	8.49	19	-37.99	2	3	1	477.587	6	c1ccc(cc1)c2ccc(cc2)c3[nH]c(c([nH+]3)c4ccccc4)c5ccc(cc5)C(=O)c6ccccc6
ZINC09057742	8.49	19.01	-38.22	2	3	1	477.587	6	c1ccc(cc1)c2ccc(cc2)c3[nH]c(c([nH+]3)c4ccc(cc4)C(=O)c5ccccc5)c6ccccc6
ZINC02063104	5.78	2.53	-14.68	0	5	0	519.409	4	c1ccc(cc1)N2C(=O)C(=Cc3ccc(o3)c4cc(ccc4Cl)Cl)C(=O)N(C2=S)c5ccccc5
ZINC08440666	2.8	-6.64	-22.92	2	7	0	462.593	6	Cc1ccccc1OCC(=O)Nc2c(c3c(s2)CCCC3)C(=O)N[C@H]4CCS(=O)(=O)C4
ZINC08440665	8.04	2.75	-20.27	0	8	0	623.525	8	CCOC(=O)C1=C(N=c2n(c(=O)/c(=C\c3ccc(o3)c4ccc(cc4Br)C)/s2)[C@H]1c5cc(ccc5OC)OC)C
ZINC08440664	8.04	2.99	-17.88	0	8	0	623.525	8	CCOC(=O)C1=C(N=c2n(c(=O)/c(=C\c3ccc(o3)c4ccc(cc4Br)C)/s2)[C@@H]1c5cc(ccc5OC)OC)C
ZINC08440661	2.8	-6.66	-22.71	2	7	0	462.593	6	Cc1ccccc1OCC(=O)Nc2c(c3c(s2)CCCC3)C(=O)N[C@@H]4CCS(=O)(=O)C4
ZINC08440659	6.72	2.8	-14.85	0	8	0	636.568	8	CCOC(=O)C1=C(N=c2n(c(=O)/c(=C\c3cc(n(c3C)c4ccc(cc4)Br)C)/s2)[C@H]1c5ccc(c(c5)OC)OC)C
ZINC08440658	6.72	2.8	-14.76	0	8	0	636.568	8	CCOC(=O)C1=C(N=c2n(c(=O)/c(=C\c3cc(n(c3C)c4ccc(cc4)Br)C)/s2)[C@@H]1c5ccc(c(c5)OC)OC)C
ZINC08440657	6.36	3.88	-15.44	0	8	0	571.699	8	CCOC(=O)C1=C(N=c2n(c(=O)/c(=C\c3cc(n(c3C)c4ccc(cc4)C)C)/s2)[C@H]1c5ccc(c(c5)OC)OC)C
ZINC08440656	3.71	-7.29	-19.26	2	6	0	487.43	4	c1cc(c(cc1Cl)Cl)C(=O)Nc2c(c3c(s2)CCCC3)C(=O)N[C@H]4CCS(=O)(=O)C4
ZINC08440655	6.36	3.88	-15.31	0	8	0	571.699	8	CCOC(=O)C1=C(N=c2n(c(=O)/c(=C\c3cc(n(c3C)c4ccc(cc4)C)C)/s2)[C@@H]1c5ccc(c(c5)OC)OC)C
ZINC08440654	3.71	-7.32	-19.39	2	6	0	487.43	4	c1cc(c(cc1Cl)Cl)C(=O)Nc2c(c3c(s2)CCCC3)C(=O)N[C@@H]4CCS(=O)(=O)C4
ZINC08440653	7.95	2.92	-14.66	0	8	0	617.727	7	CCOC(=O)C1=C(N=c2n(c(=O)/c(=C\c3cc(n(c3C)c4ccc(cc4)c5ccccc5)C)/s2)[C@H]1c6ccc7c(c6)OCO7)C
ZINC08440652	7.95	3.09	-14.48	0	8	0	617.727	7	CCOC(=O)C1=C(N=c2n(c(=O)/c(=C\c3cc(n(c3C)c4ccc(cc4)c5ccccc5)C)/s2)[C@@H]1c6ccc7c(c6)OCO7)C
ZINC08440651	5.68	16.37	-24.87	2	7	0	564.089	5	Cc1cccnc1NC(=O)C2=C(NC3=C([C@@H]2c4c(nn(c4Cl)c5ccccc5)C)C(=O)C[C@@H](C3)c6ccccc6)C
ZINC08440651	5.82	-0.25	-17	1	7	0	564.089	5	Cc1cccnc1NC(=O)C2[C@H](C3C(=NC2=C)C[C@H](CC3=O)c4ccccc4)c5c(nn(c5Cl)c6ccccc6)C
ZINC08440650	6.83	2.23	-13.3	0	8	0	576.074	6	CCOC(=O)C1=C(N=c2n(c(=O)/c(=C\c3cc(n(c3C)c4ccc(cc4)Cl)C)/s2)[C@H]1c5ccc6c(c5)OCO6)C
ZINC08440649	4.02	0.86	-22.98	1	8	0	535.568	12	COCCCN1[C@H](C(=C(C1=O)O)C(=O)c2ccc(c(c2)F)OC)c3ccc(c(c3)OC)OCc4ccccc4
ZINC08440649	3.44	0.71	-29.45	0	8	0	535.568	12	COCCCN1[C@H]([C@@H](C(=O)C1=O)C(=O)c2ccc(c(c2)F)OC)c3ccc(c(c3)OC)OCc4ccccc4
ZINC08440648	6.83	2.4	-13.15	0	8	0	576.074	6	CCOC(=O)C1=C(N=c2n(c(=O)/c(=C\c3cc(n(c3C)c4ccc(cc4)Cl)C)/s2)[C@@H]1c5ccc6c(c5)OCO6)C
ZINC08440647	7.17	2.7	-12.96	0	7	0	619.585	7	CCOC(=O)C1=C(N=c2n(c(=O)/c(=C\c3cc(n(c3C)c4ccc(cc4)Br)C)/s2)[C@H]1c5ccc(cc5)N(C)C)C
ZINC08440646	7.17	2.54	-12.83	0	7	0	619.585	7	CCOC(=O)C1=C(N=c2n(c(=O)/c(=C\c3cc(n(c3C)c4ccc(cc4)Br)C)/s2)[C@@H]1c5ccc(cc5)N(C)C)C
ZINC08440645	4.02	1.33	-20.71	1	8	0	535.568	12	COCCCN1[C@@H](C(=C(C1=O)O)C(=O)c2ccc(c(c2)F)OC)c3ccc(c(c3)OC)OCc4ccccc4
ZINC08440645	3.44	1.16	-26.58	0	8	0	535.568	12	COCCCN1[C@@H]([C@H](C(=O)C1=O)C(=O)c2ccc(c(c2)F)OC)c3ccc(c(c3)OC)OCc4ccccc4
ZINC08440644	6.53	4.12	-13.62	0	7	0	558.679	7	CCOC(=O)C1=C(N=c2n(c(=O)/c(=C\c3cc(n(c3C)c4ccc(cc4)F)C)/s2)[C@H]1c5ccc(cc5)N(C)C)C
ZINC08440643	4.11	0.28	-63.67	0	7	-1	545.45	6	CCOc1cc(c(cc1OCC(=O)[O-])Br)[C@@H]2C3C(=NC4=C2C(=O)CC(C4)(C)C)CC(CC3=O)(C)C
ZINC08440643	4.27	-0.67	-59.12	0	7	-1	545.45	6	CCOc1cc(c(cc1OCC(=O)[O-])Br)[C@H]2C3C(=NC4=CC(CC(=O)C24)(C)C)CC(CC3=O)(C)C
ZINC08440642	6.53	3.95	-13.34	0	7	0	558.679	7	CCOC(=O)C1=C(N=c2n(c(=O)/c(=C\c3cc(n(c3C)c4ccc(cc4)F)C)/s2)[C@@H]1c5ccc(cc5)N(C)C)C
ZINC08440641	7.04	3.31	-13.1	0	7	0	575.134	7	CCOC(=O)C1=C(N=c2n(c(=O)/c(=C\c3cc(n(c3C)c4ccc(cc4)Cl)C)/s2)[C@H]1c5ccc(cc5)N(C)C)C
ZINC08440640	2.63	-0.91	-63.66	3	9	1	550.426	10	C[NH+](C)CCCN1[C@H](C(=C(C1=O)O)C(=O)c2ccc(c(c2)OC)OC)c3cc(c(c(c3)Br)O)OC
ZINC08440640	2.05	-1.06	-73.58	2	9	1	550.426	10	C[NH+](C)CCCN1[C@H]([C@@H](C(=O)C1=O)C(=O)c2ccc(c(c2)OC)OC)c3cc(c(c(c3)Br)O)OC
ZINC08440639	7.04	3.14	-12.69	0	7	0	575.134	7	CCOC(=O)C1=C(N=c2n(c(=O)/c(=C\c3cc(n(c3C)c4ccc(cc4)Cl)C)/s2)[C@@H]1c5ccc(cc5)N(C)C)C
ZINC08440638	6.43	3.97	-12.33	0	6	0	515.61	6	CCOC(=O)C1=C(N=c2n(c(=O)/c(=C\c3cc(n(c3C)c4ccc(cc4)F)C)/s2)[C@H]1c5ccccc5)C
ZINC08440637	6.43	4.05	-12.48	0	6	0	515.61	6	CCOC(=O)C1=C(N=c2n(c(=O)/c(=C\c3cc(n(c3C)c4ccc(cc4)F)C)/s2)[C@@H]1c5ccccc5)C
ZINC08440636	7.73	3.19	-11.72	0	4	0	502.397	6	CCOc1ccc(cc1)N2C(=C/C(=C\c3ccc(o3)c4ccc(c(c4)Cl)Cl)/C2=O)c5ccccc5
ZINC08440635	8.16	3.02	-12.27	0	3	0	492.789	4	c1ccc(cc1)C2=C/C(=C\c3ccc(o3)c4ccc(c(c4)Cl)Cl)/C(=O)N2c5cccc(c5)Cl
ZINC08440634	2.63	-1.11	-50.69	3	9	1	550.426	10	C[NH+](C)CCCN1[C@@H](C(=C(C1=O)O)C(=O)c2ccc(c(c2)OC)OC)c3cc(c(c(c3)Br)O)OC
ZINC08440634	2.05	-1.26	-59.72	2	9	1	550.426	10	C[NH+](C)CCCN1[C@@H]([C@H](C(=O)C1=O)C(=O)c2ccc(c(c2)OC)OC)c3cc(c(c(c3)Br)O)OC
ZINC08440632	9.26	3.08	-18.64	0	7	0	619.526	7	CCOC(=O)C1=C(N=c2n(c(=O)/c(=C\c3ccc(o3)c4ccc(c(c4)Cl)Cl)/s2)[C@H]1c5c6ccccc6ccc5OC)C
ZINC08440631	9.26	3.09	-18.1	0	7	0	619.526	7	CCOC(=O)C1=C(N=c2n(c(=O)/c(=C\c3ccc(o3)c4ccc(c(c4)Cl)Cl)/s2)[C@@H]1c5c6ccccc6ccc5OC)C
ZINC05919169	3.12	10.01	-63.49	0	8	-1	487.532	10	CCCOc1ccc(cc1)[C@H]2C(=C(C(=O)N2Cc3cccnc3)[O-])C(=O)c4ccc(c(c4)OC)OC
ZINC05919169	3.57	-0.08	-28.13	1	8	0	488.54	9	CCCOc1ccc(cc1)[C@H]2C(=C(c3ccc(c(c3)OC)OC)O)C(=O)C(=O)N2Cc4cccnc4
ZINC05919169	2.54	0.14	-21.26	0	8	0	488.54	10	CCCOc1ccc(cc1)[C@H]2[C@@H](C(=O)C(=O)N2Cc3cccnc3)C(=O)c4ccc(c(c4)OC)OC

Supplementary Table S2:
Detailed table of XP Docking using Schrodinger's Glide module

ZINC_ID	xLogP	ApolarDesolvation	PolarDesolvation	H Donors	H Acceptors	Charge	MolecularWeight	RotatableBonds	SMILES
ZINC02313508	7.26	2.96	-18.76	0	8	0	548.642	9	CCOC(=O)c1ccc(cc1)c2ccc(o2)/C=c/3\c(=O)n4c(=NC(=C([C@H]4c5cccs5)C(=O)OCC)C)s3
ZINC02313510	7.26	2.84	-18.61	0	8	0	548.642	9	CCOC(=O)c1ccc(cc1)c2ccc(o2)/C=c/3\c(=O)n4c(=NC(=C([C@@H]4c5cccs5)C(=O)OCC)C)s3
ZINC08440630	7.01	-0.16	-21.23	1	6	0	552.678	4	Cc1ccnc(c1)NC(=O)C2[C@H](C3=C(C[C@@H](CC3=O)c4ccccc4)N=C2C)c5ccc6c(c5)c7ccccc7n6C
ZINC08440629	7.36	1.59	-14.13	0	6	0	511.024	6	CCOC(=O)C1=C(N=c2n(c(=O)/c(=C\c3ccc(o3)c4cccc(c4)Cl)/s2)[C@H]1c5cccs5)C
ZINC08440628	7.36	1.61	-16.28	0	6	0	511.024	6	CCOC(=O)C1=C(N=c2n(c(=O)/c(=C\c3ccc(o3)c4cccc(c4)Cl)/s2)[C@@H]1c5cccs5)C
ZINC05919133	3.12	9.16	-63.8	0	8	-1	487.532	10	CCCOc1ccc(cc1)[C@@H]2C(=C(C(=O)N2Cc3cccnc3)[O-])C(=O)c4ccc(c(c4)OC)OC
ZINC05919133	3.57	-0.47	-31.98	1	8	0	488.54	9	CCCOc1ccc(cc1)[C@@H]2C(=C(c3ccc(c(c3)OC)OC)O)C(=O)C(=O)N2Cc4cccnc4
ZINC05919133	2.54	-0.24	-26.41	0	8	0	488.54	10	CCCOc1ccc(cc1)[C@@H]2[C@H](C(=O)C(=O)N2Cc3cccnc3)C(=O)c4ccc(c(c4)OC)OC
ZINC08440627	7.72	5.35	-24.96	0	8	0	598.599	9	CCOC(=O)C1=C(N=c2n(c(=O)/c(=C\c3ccc(o3)c4cccc(c4)C(F)(F)F)/s2)[C@H]1c5cc(ccc5OC)OC)C
ZINC08440626	7.72	5.45	-21.69	0	8	0	598.599	9	CCOC(=O)C1=C(N=c2n(c(=O)/c(=C\c3ccc(o3)c4cccc(c4)C(F)(F)F)/s2)[C@@H]1c5cc(ccc5OC)OC)C
ZINC08440625	7.01	-0.74	-20.94	1	6	0	552.678	4	Cc1ccnc(c1)NC(=O)C2[C@@H](C3=C(C[C@H](CC3=O)c4ccccc4)N=C2C)c5ccc6c(c5)c7ccccc7n6C
ZINC08440624	4.13	1.76	-20.27	1	8	0	541.579	11	COc1ccc(cc1F)C(=O)C2=C(C(=O)N([C@H]2c3ccc(cc3)OCc4ccccc4)CCCn5ccnc5)O
ZINC08440624	3.55	1.59	-26.43	0	8	0	541.579	11	COc1ccc(cc1F)C(=O)[C@H]2[C@@H](N(C(=O)C2=O)CCCn3ccnc3)c4ccc(cc4)OCc5ccccc5
ZINC08440624	4.58	1.66	-57.89	2	8	1	542.587	10	COc1ccc(cc1F)C(=C2[C@@H](N(C(=O)C2=O)CCCn3cc[nH+]c3)c4ccc(cc4)OCc5ccccc5)O
ZINC08440624	3.55	1.88	-54.02	1	8	1	542.587	11	COc1ccc(cc1F)C(=O)[C@H]2[C@@H](N(C(=O)C2=O)CCCn3cc[nH+]c3)c4ccc(cc4)OCc5ccccc5
ZINC08440623	5.83	0.81	-16.17	1	8	0	503.58	7	CCOC(=O)C1=C(N=c2n(c(=O)/c(=C\c3c[nH]c4c3cccc4)/s2)[C@H]1c5cc(ccc5OC)OC)C
ZINC08440622	5.83	0.92	-13.03	1	8	0	503.58	7	CCOC(=O)C1=C(N=c2n(c(=O)/c(=C\c3c[nH]c4c3cccc4)/s2)[C@@H]1c5cc(ccc5OC)OC)C
ZINC08440621	8.36	5.21	-16.86	0	6	0	572.992	7	CCOC(=O)C1=C(N=c2n(c(=O)/c(=C\c3ccc(o3)c4cccc(c4)C(F)(F)F)/s2)[C@H]1c5ccc(cc5)Cl)C
ZINC08440620	3.37	1.91	-47.75	2	7	1	457.522	10	CC[NH+](CC)CCN1[C@H](C(=C(C1=O)O)C(=O)c2ccc(c(c2)F)OC)c3ccc(cc3)OC
ZINC08440620	2.78	1.75	-56.39	1	7	1	457.522	10	CC[NH+](CC)CCN1[C@H]([C@@H](C(=O)C1=O)C(=O)c2ccc(c(c2)F)OC)c3ccc(cc3)OC
ZINC08440619	8.36	5.2	-16.69	0	6	0	572.992	7	CCOC(=O)C1=C(N=c2n(c(=O)/c(=C\c3ccc(o3)c4cccc(c4)C(F)(F)F)/s2)[C@@H]1c5ccc(cc5)Cl)C
ZINC08440618	7.57	4.33	-20.03	0	8	0	582.556	7	CCOC(=O)C1=C(N=c2n(c(=O)/c(=C\c3ccc(o3)c4cccc(c4)C(F)(F)F)/s2)[C@H]1c5ccc6c(c5)OCO6)C
ZINC08440617	7.05	18.13	-31.14	2	6	0	552.678	4	Cc1ccnc(c1)NC(=O)C2=C(NC3=C([C@@H]2c4ccc5c(c4)c6ccccc6n5C)C(=O)C[C@@H](C3)c7ccccc7)C
ZINC08440615	3.37	1.66	-45.29	2	7	1	457.522	10	CC[NH+](CC)CCN1[C@@H](C(=C(C1=O)O)C(=O)c2ccc(c(c2)F)OC)c3ccc(cc3)OC
ZINC08440615	2.78	1.57	-54.46	1	7	1	457.522	10	CC[NH+](CC)CCN1[C@@H]([C@@H](C(=O)C1=O)C(=O)c2ccc(c(c2)F)OC)c3ccc(cc3)OC
ZINC08440616	7.57	4.34	-19.89	0	8	0	582.556	7	CCOC(=O)C1=C(N=c2n(c(=O)/c(=C\c3ccc(o3)c4cccc(c4)C(F)(F)F)/s2)[C@@H]1c5ccc6c(c5)OCO6)C
ZINC08440614	7.36	2.01	-14.83	0	8	0	549.004	6	CCOC(=O)C1=C(N=c2n(c(=O)/c(=C\c3ccc(o3)c4cccc(c4)Cl)/s2)[C@H]1c5ccc6c(c5)OCO6)C
ZINC08440613	7.36	2.1	-17.54	0	8	0	549.004	6	CCOC(=O)C1=C(N=c2n(c(=O)/c(=C\c3ccc(o3)c4cccc(c4)Cl)/s2)[C@@H]1c5ccc6c(c5)OCO6)C
ZINC08440612	7.33	5.3	-19.9	0	8	0	598.599	9	CCOC(=O)C1=C(N=c2n(c(=O)/c(=C\c3ccc(o3)c4cccc(c4)C(F)(F)F)/s2)[C@H]1c5ccc(c(c5)OC)OC)C
ZINC08440611	7.33	5.54	-20.98	0	8	0	598.599	9	CCOC(=O)C1=C(N=c2n(c(=O)/c(=C\c3ccc(o3)c4cccc(c4)C(F)(F)F)/s2)[C@@H]1c5ccc(c(c5)OC)OC)C
ZINC00702636	4.38	0.08	-23.58	0	7	0	492.626	7	CCOC(=O)C1CCN(CC1)C2=NC(=O)/C(=C/c3cn(nc3c4cccs4)c5ccccc5)/S2
ZINC08440609	7.11	3.07	-19.41	0	8	0	565.047	8	CCOC(=O)C1=C(N=c2n(c(=O)/c(=C\c3ccc(o3)c4cccc(c4)Cl)/s2)[C@H]1c5ccc(c(c5)OC)OC)C
ZINC08440608	3.39	8.88	-42.16	0	7	-1	436.484	11	COCCCN1[C@H](C(=C(C1=O)[O-])C(=O)c2ccc(cc2)OCC=C)c3ccc(cc3)OC
ZINC08440608	3.84	0.06	-28.17	1	7	0	437.492	10	COCCCN1[C@H](C(=C(c2ccc(cc2)OCC=C)O)C(=O)C1=O)c3ccc(cc3)OC
ZINC08440608	2.81	0.35	-21.76	0	7	0	437.492	11	COCCCN1[C@H]([C@H](C(=O)C1=O)C(=O)c2ccc(cc2)OCC=C)c3ccc(cc3)OC
ZINC08440607	7.11	3.23	-17.33	0	8	0	565.047	8	CCOC(=O)C1=C(N=c2n(c(=O)/c(=C\c3ccc(o3)c4cccc(c4)Cl)/s2)[C@@H]1c5ccc(c(c5)OC)OC)C
ZINC08440606	3.39	8.88	-42.19	0	7	-1	436.484	11	COCCCN1[C@@H](C(=C(C1=O)[O-])C(=O)c2ccc(cc2)OCC=C)c3ccc(cc3)OC
ZINC08440606	3.84	-0.23	-29.97	1	7	0	437.492	10	COCCCN1[C@@H](C(=C(c2ccc(cc2)OCC=C)O)C(=O)C1=O)c3ccc(cc3)OC
ZINC08440606	2.81	0.03	-25.01	0	7	0	437.492	11	COCCCN1[C@@H]([C@@H](C(=O)C1=O)C(=O)c2ccc(cc2)OCC=C)c3ccc(cc3)OC
ZINC08440605	7.68	5.34	-18.02	0	6	0	538.547	7	CCOC(=O)C1=C(N=c2n(c(=O)/c(=C\c3ccc(o3)c4cccc(c4)C(F)(F)F)/s2)[C@H]1c5ccccc5)C
ZINC08440604	7.68	5.28	-17.34	0	6	0	538.547	7	CCOC(=O)C1=C(N=c2n(c(=O)/c(=C\c3ccc(o3)c4cccc(c4)C(F)(F)F)/s2)[C@@H]1c5ccccc5)C
ZINC08440603	6.75	3.37	-19.91	0	10	0	545.573	8	CCOC(=O)C1=C(N=c2n(c(=O)/c(=C\c3ccc(o3)c4cc(ccc4OC)[N+](=O)[O-])/s2)[C@H]1c5ccccc5)C
ZINC08440601	6.75	3.36	-19.21	0	10	0	545.573	8	CCOC(=O)C1=C(N=c2n(c(=O)/c(=C\c3ccc(o3)c4cc(ccc4OC)[N+](=O)[O-])/s2)[C@@H]1c5ccccc5)C
ZINC08440600	5.04	11.5	-59.43	0	6	-1	462.566	11	CC(C)(C)c1ccc(cc1)[C@H]2C(=C(C(=O)N2CCCOC)[O-])C(=O)c3ccc(cc3)OCC=C
ZINC08440600	5.49	1.19	-27.01	1	6	0	463.574	10	CC(C)(C)c1ccc(cc1)[C@H]2C(=C(c3ccc(cc3)OCC=C)O)C(=O)C(=O)N2CCCOC
ZINC08440600	4.46	1.48	-20.33	0	6	0	463.574	11	CC(C)(C)c1ccc(cc1)[C@H]2[C@H](C(=O)C(=O)N2CCCOC)C(=O)c3ccc(cc3)OCC=C
ZINC00845776	4.22	-2.2	-18.93	2	6	0	478.144	4	Cn1c(cc(n1)C(=O)Nc2cccc(c2)Br)C(=O)Nc3cccc(c3)Br
ZINC08440599	5.04	11.07	-61.14	0	6	-1	462.566	11	CC(C)(C)c1ccc(cc1)[C@@H]2C(=C(C(=O)N2CCCOC)[O-])C(=O)c3ccc(cc3)OCC=C
ZINC08440599	5.49	0.9	-28.81	1	6	0	463.574	10	CC(C)(C)c1ccc(cc1)[C@@H]2C(=C(c3ccc(cc3)OCC=C)O)C(=O)C(=O)N2CCCOC
ZINC08440599	4.46	1.16	-23.63	0	6	0	463.574	11	CC(C)(C)c1ccc(cc1)[C@@H]2[C@@H](C(=O)C(=O)N2CCCOC)C(=O)c3ccc(cc3)OCC=C
ZINC08440598	5.99	0.89	-48.89	2	7	1	569.722	10	Cc1cc(ccc1C(=O)C2=C(C(=O)N([C@@H]2c3ccc(cc3)C(C)(C)C)CC[NH+]4CCOCC4)O)OCc5ccccc5
ZINC08440598	6.43	0.49	-60.16	2	7	1	569.722	9	Cc1cc(ccc1/C(=C\2/[C@H](N(C(=O)C2=O)CC[NH+]3CCOCC3)c4ccc(cc4)C(C)(C)C)/O)OCc5ccccc5
ZINC08440598	5.41	0.78	-59.3	1	7	1	569.722	10	Cc1cc(ccc1C(=O)[C@H]2[C@H](N(C(=O)C2=O)CC[NH+]3CCOCC3)c4ccc(cc4)C(C)(C)C)OCc5ccccc5
ZINC00845579	5.19	-3.32	-13.81	3	5	0	436.364	5	CCOc1ccc(cc1)NC(=O)C2=C(NC(=S)N[C@@H]2c3ccc(cc3Cl)Cl)C
ZINC08440596	3.46	-3.01	-10.96	1	6	0	467.367	3	CC1=NC2=C([C@@H](C1C(=O)Nc3ccc(cn3)Br)c4ccccn4)C(=O)CC(C2)(C)C
ZINC08440596	3.46	-2.4	-13.93	1	6	0	467.367	3	CC1=C([C@H]([C@H]2C(=N1)CC(CC2=O)(C)C)c3ccccn3)C(=O)Nc4ccc(cn4)Br
ZINC08440596	3.64	-2.89	-11.96	1	6	0	467.367	3	CC1(CC2=NC(=C)C([C@H](C2C(=O)C1)c3ccccn3)C(=O)Nc4ccc(cn4)Br)C
ZINC08440596	3.64	-1.86	-11.7	1	6	0	467.367	3	CC1(CC2=NC(=C)C([C@H]([C@H]2C(=O)C1)c3ccccn3)C(=O)Nc4ccc(cn4)Br)C
ZINC08440595	4	-1.78	-53.1	3	9	1	573.666	11	CCOc1cc(ccc1O)[C@H]2C(=C(C(=O)N2CC[NH+]3CCOCC3)O)C(=O)c4ccc(cc4C)OCc5ccccc5
ZINC08440595	3.41	-2.06	-61.05	2	9	1	573.666	11	CCOc1cc(ccc1O)[C@H]2[C@H](C(=O)C(=O)N2CC[NH+]3CCOCC3)C(=O)c4ccc(cc4C)OCc5ccccc5
ZINC08440589	4	-2.02	-47.59	3	9	1	573.666	11	CCOc1cc(ccc1O)[C@@H]2C(=C(C(=O)N2CC[NH+]3CCOCC3)O)C(=O)c4ccc(cc4C)OCc5ccccc5

Supplementary Table S2:
Detailed table of XP Docking using Schrodinger's Glide module

ZINC_ID	xLogP	ApolarDesolvation	PolarDesolvation	H Donors	H Acceptors	Charge	MolecularWeight	RotatableBonds	SMILES
ZINC08440589	3.41	-2.24	-53.59	2	9	1	573.666	11	CCOc1cc(ccc1O)[C@@H]2[C@H](C(=O)C(=O)N2CC[NH+]3CCOCC3)C(=O)c4ccc(cc4C)OCc5ccccc5
ZINC02063033	7.11	1.83	-12.13	1	7	0	583.419	7	CCOC(=O)c1c2c(sc1NC(=O)c3cc4nc(cc(n4n3)C(F)(F)F)c5ccc(c(c5)Cl)Cl)CCCC2
ZINC08440586	5.74	3.06	-15.62	1	5	0	471.504	8	CCOc1ccc(cc1)NC(=O)CSc2c(c(cc(n2)c3ccc(cc3)C)C(F)(F)F)C#N
ZINC08440585	7.29	2.67	-18.21	1	9	0	638.4	7	c1cc(ccc1c2cc(n3c(n2)c(cn3)C(=O)Nc4cc(cc(c4)Oc5ccc6c(c5)CCC6)[N+](=O)[O-])C(F)(F)F)Br
ZINC02742587	6.04	0.87	-15.34	1	5	0	540.137	4	c1cc(ccc1c2cc(n3c(n2)c(cn3)C(=O)Nc4ccc(cc4)Br)C(F)(F)F)Br
ZINC08440584	3.5	9.97	-17.43	2	6	0	467.367	3	CC1=C([C@@H](C2=C(N1)CC(CC2=O)(C)C)c3ccccn3)C(=O)Nc4ccc(cn4)Br
ZINC08440584	3.64	-2.14	-21.5	1	6	0	467.367	3	CC1(CC2=NC(=C)C([C@@H]([C@@H]2C(=O)C1)c3ccccn3)C(=O)Nc4ccc(cn4)Br)C
ZINC08440584	3.64	-2.72	-20.25	1	6	0	467.367	3	CC1(CC2=NC(=C)C([C@@H](C2C(=O)C1)c3ccccn3)C(=O)Nc4ccc(cn4)Br)C
ZINC08440584	3.46	-2.75	-20.97	1	6	0	467.367	3	CC1=C([C@@H]([C@H]2C(=N1)CC(CC2=O)(C)C)c3ccccn3)C(=O)Nc4ccc(cn4)Br
ZINC00666527	5.54	1.45	-11.6	1	6	0	491.267	5	COc1ccccc1NC(=O)c2cc3nc(cc(n3n2)C(F)(F)F)c4ccc(cc4)Br
ZINC02063028	3.54	-3.25	-12.49	0	4	0	319.769	3	c1cc2cccnc2c(c1)OS(=O)(=O)c3ccc(cc3)Cl
ZINC08440583	4.62	0.55	-13.83	0	7	0	423.494	6	CC(C)Oc1ccc(cc1)c2nc3n(n2)c(=O)/c(=C\c4ccc(cc4OC)OC)/s3
ZINC00666509	3.26	0.93	-14.72	1	10	0	469.494	8	COc1ccc(cc1)C(=O)N/C(=C/c2cc(c(cc2[N+](=O)[O-])OC)OC)/C(=O)N3CCCCC3
ZINC08440582	10.33	6.08	-7.5	0	2	0	629.819	6	Cc1ccc(cc1)C(=O)N2c3ccc(cc3[C@](CC2(C)C)(C)c4ccc(cc4)F)C(c5ccccc5)(c6ccccc6)c7ccccc7
ZINC08440581	10.33	6.14	-8.26	0	2	0	629.819	6	Cc1ccc(cc1)C(=O)N2c3ccc(cc3[C@@](CC2(C)C)(C)c4ccc(cc4)F)C(c5ccccc5)(c6ccccc6)c7ccccc7
ZINC00702625	0.11	10.89	-33.36	2	7	1	423.518	4	CCC[n+]1c(c(cc2c1nc3c(cccn3c2=O)C)S(=O)(=O)c4ccc(cc4)C)N
ZINC02063023	6.94	2.35	-10.43	0	5	0	511.021	5	C[C@H]1C[C@H](c2ccccc2N1C(=O)c3ccc(cc3)Cl)N(c4ccccc4)C(=O)c5ccc(cc5)OC
ZINC02063024	6.94	2.05	-11.51	0	5	0	511.021	5	C[C@@H]1C[C@H](c2ccccc2N1C(=O)c3ccc(cc3)Cl)N(c4ccccc4)C(=O)c5ccc(cc5)OC
ZINC08440580	6.97	14.83	-36.44	2	8	0	619.738	8	Cc1ccc(nc1)NC(=O)C2=C(NC3=C([C@H]2c4ccc(c(c4)CSc5nc(cc(n5)C(F)F)C)OC)C(=O)CC(C3)(C)C)C
ZINC08440580	7.11	-1.87	-23.2	1	8	0	619.738	8	Cc1ccc(nc1)NC(=O)C2[C@@H](C3C(=NC2=C)CC(CC3=O)(C)C)c4ccc(c(c4)CSc5nc(cc(n5)C(F)F)C)OC
ZINC02063025	6.94	2.61	-16.73	0	5	0	511.021	5	C[C@H]1C[C@@H](c2ccccc2N1C(=O)c3ccc(cc3)Cl)N(c4ccccc4)C(=O)c5ccc(cc5)OC
ZINC02063026	6.94	2.4	-12.41	0	5	0	511.021	5	C[C@@H]1C[C@@H](c2ccccc2N1C(=O)c3ccc(cc3)Cl)N(c4ccccc4)C(=O)c5ccc(cc5)OC
ZINC00702621	0.32	11.08	-34.91	2	8	1	481.941	4	Cc1cccn2c1nc3c(c2=O)cc(c([n+]3Cc4ccco4)N)S(=O)(=O)c5ccc(cc5)Cl
ZINC01823289	8.57	3.25	-11.68	0	4	0	516.685	8	CCCCCc1ccc(cc1)C(=O)N2c3ccccc3[C@H](C[C@H]2C)N(c4ccccc4)C(=O)c5ccccc5
ZINC01823291	8.57	2.91	-12.81	0	4	0	516.685	8	CCCCCc1ccc(cc1)C(=O)N2c3ccccc3[C@@H](C[C@H]2C)N(c4ccccc4)C(=O)c5ccccc5
ZINC01823290	8.57	3.56	-17.39	0	4	0	516.685	8	CCCCCc1ccc(cc1)C(=O)N2c3ccccc3[C@H](C[C@@H]2C)N(c4ccccc4)C(=O)c5ccccc5
ZINC01823292	8.57	3.32	-11.31	0	4	0	516.685	8	CCCCCc1ccc(cc1)C(=O)N2c3ccccc3[C@@H](C[C@@H]2C)N(c4ccccc4)C(=O)c5ccccc5
ZINC08440579	6.97	15	-35.1	2	8	0	619.738	8	Cc1ccc(nc1)NC(=O)C2=C(NC3=C([C@@H]2c4ccc(c(c4)CSc5nc(cc(n5)C(F)F)C)OC)C(=O)CC(C3)(C)C)C
ZINC08440579	7.11	-1.06	-18.04	1	8	0	619.738	8	Cc1ccc(nc1)NC(=O)C2[C@H](C3C(=NC2=C)CC(CC3=O)(C)C)c4ccc(c(c4)CSc5nc(cc(n5)C(F)F)C)OC
ZINC01746406	8.02	2.77	-13.36	0	5	0	573.761	6	CCOc1ccc2c(c1)/C(=C/3\SC(=C(S3)C(=O)OC)c4ccccc4)/C(=S)C(N2C(=O)c5ccccc5)(C)C
ZINC01761545	7.64	2.41	-13.58	0	5	0	559.734	5	CC1(C(=S)/C(=C\2/SC(=C(S2)C(=O)OC)c3ccccc3)/c4cc(ccc4N1C(=O)c5ccccc5)OC)C
ZINC00702618	4.1	0.9	-16.62	0	7	0	466.559	8	CCOc1ccc(cc1OC)[C@H]2C(=C(N=C3N2C(=O)CCS3)C)C(=O)OCc4ccccc4
ZINC02063018	8.04	3.07	-13.5	0	4	0	543.735	4	Cc1ccc2c(c1)/C(=C/3\SC(=C(S3)C(=O)OC)c4ccccc4)/C(=S)C(N2C(=O)c5ccccc5)(C)C
ZINC08440578	7.25	1.61	-13.03	0	4	0	478.617	9	COc1cccc(c1)OCCSc2nc(c(n2c3ccccc3)c4ccccc4)c5ccccc5
ZINC01812606	5.97	2.61	-14.2	0	5	0	497.663	4	CC(=O)N1c2ccc(cc2/C(=C/3\SC(=C(S3)C(=O)OC)c4ccccc4)/C(=S)C1(C)C)OC
ZINC00702619	4.1	0.93	-14.97	0	7	0	466.559	8	CCOc1ccc(cc1OC)[C@@H]2C(=C(N=C3N2C(=O)CCS3)C)C(=O)OCc4ccccc4
ZINC02063015	6.76	0.74	-9.16	1	3	0	439.627	3	Cc1ccc\2c(c1)NC(C(=S)/C2=C\3/SC(=C(S3)C(=O)OC)c4ccccc4)(C)C
ZINC02265970	6.74	0.78	-10.38	1	4	0	469.653	5	CCOc1ccc2c(c1)/C(=C/3\SC(=C(S3)C(=O)OC)c4ccccc4)/C(=S)C(N2)(C)C
ZINC08440577	6.46	3.25	-16.92	0	8	0	583.709	5	Cc1cc2c(cc1C)N(C(C(=S)C2=C3SC(=C(S3)C(=O)OC)C(=O)OC)(C)C)C(=O)c4ccc5c(c4)OCO5
ZINC01806052	5.97	-0.39	-12.97	0	4	0	464.033	4	CC1(c2c(c(=S)ss2)-c3cc(ccc3N1C(=O)COc4ccc(cc4)Cl)OC)C
ZINC08440576	6.81	14.57	-37.91	2	7	0	578.496	7	Cc1cccnc1NC(=O)C2=C(NC3=C([C@H]2c4ccc(c(c4)COc5cccc(c5Cl)Cl)OC)C(=O)CCC3)C
ZINC08440576	6.95	-1.97	-17.27	1	7	0	578.496	7	Cc1cccnc1NC(=O)C2[C@@H](C3C(=NC2=C)CCCC3=O)c4ccc(c(c4)COc5cccc(c5Cl)Cl)OC
ZINC08440575	4.12	0.91	-52.06	2	7	1	487.576	10	C[NH+](C)CCN1[C@H](C(=C(C1=O)O)C(=O)c2ccc(cc2)OC)c3ccc(cc3)OCc4ccccc4
ZINC08440575	3.53	0.76	-61.47	1	7	1	487.576	10	C[NH+](C)CCN1[C@H]([C@@H](C(=O)C1=O)C(=O)c2ccc(cc2)OC)c3ccc(cc3)OCc4ccccc4
ZINC08440574	6.84	3.61	-17.63	0	7	0	590.144	7	Cc1ccc2c(c1)C(=C3SC(=C(S3)C(=O)OC)C(=O)OC)C(=S)C(N2C(=O)COc4ccc(cc4)Cl)(C)C
ZINC02062574	5.94	-0.4	-10.36	0	3	0	434.007	3	CC1(c2c(c(=S)ss2)-c3ccccc3N1C(=O)COc4ccc(cc4)Cl)C
ZINC02062573	7.4	0.82	-6.55	0	2	0	439.671	2	Cc1cccc-2c1N(C(c3c2c(=S)ss3)(C)C)C(=O)c4ccc(cc4)C(C)(C)C
ZINC08440573	4.12	1.39	-56.34	2	7	1	487.576	10	C[NH+](C)CCN1[C@@H](C(=C(C1=O)O)C(=O)c2ccc(cc2)OC)c3ccc(cc3)OCc4ccccc4
ZINC08440573	3.53	1.22	-65.71	1	7	1	487.576	10	C[NH+](C)CCN1[C@@H]([C@H](C(=O)C1=O)C(=O)c2ccc(cc2)OC)c3ccc(cc3)OCc4ccccc4
ZINC08440571	5.05	-0.15	-56.15	3	7	1	529.657	12	CC[NH+](CC)CCCN1[C@H](C(=C(C1=O)O)C(=O)c2ccc(cc2)OCc3ccc(cc3)C)c4ccc(cc4)O
ZINC08440571	4.47	-0.32	-65.55	2	7	1	529.657	12	CC[NH+](CC)CCCN1[C@H]([C@@H](C(=O)C1=O)C(=O)c2ccc(cc2)OCc3ccc(cc3)C)c4ccc(cc4)O
ZINC08440570	6.81	14.71	-36.24	2	7	0	578.496	7	Cc1cccnc1NC(=O)C2=C(NC3=C([C@@H]2c4ccc(c(c4)COc5cccc(c5Cl)Cl)OC)C(=O)CCC3)C
ZINC08440570	6.95	-1.93	-17.11	1	7	0	578.496	7	Cc1cccnc1NC(=O)C2[C@H](C3C(=NC2=C)CCCC3=O)c4ccc(c(c4)COc5cccc(c5Cl)Cl)OC
ZINC08440566	5.05	-0.44	-46.69	3	7	1	529.657	12	CC[NH+](CC)CCCN1[C@@H](C(=C(C1=O)O)C(=O)c2ccc(cc2)OCc3ccc(cc3)C)c4ccc(cc4)O
ZINC08440566	4.47	-0.53	-56.54	2	7	1	529.657	12	CC[NH+](CC)CCCN1[C@@H]([C@@H](C(=O)C1=O)C(=O)c2ccc(cc2)OCc3ccc(cc3)C)c4ccc(cc4)O
ZINC00666458	5.14	11.14	-8.88	2	6	0	399.535	8	CCN(CC)c1ccc(cc1)[C@H]2C(=C(NC(=O)N2)C)C(=O)OCC3CCCCC3
ZINC08440561	4.13	0.48	-61.37	2	8	1	533.576	9	COc1ccc(cc1F)C(=O)C2=C(C(=O)N([C@H]2c3cccc(c3)Oc4ccccc4)CC[NH+]5CCOCC5)O
ZINC08440561	3.55	0.32	-70.39	1	8	1	533.576	9	COc1ccc(cc1F)C(=O)[C@H]2[C@@H](N(C(=O)C2=O)CC[NH+]3CCOCC3)c4cccc(c4)Oc5ccccc5
ZINC00666462	5.14	11.05	-10.01	2	6	0	399.535	8	CCN(CC)c1ccc(cc1)[C@@H]2C(=C(NC(=O)N2)C)C(=O)OCC3CCCCC3
ZINC08440560	5.94	14.38	-18.72	1	6	0	475.585	9	CCCOc1ccc(cc1OC)[C@H]2C3=C(C[C@H](CC3=O)c4ccccc4)NC(=C2C(=O)OCC)C
ZINC08440560	6.05	0.08	-9.01	0	6	0	475.585	9	CCCOc1ccc(cc1OC)[C@H]2C3C(=O)C[C@@H](C=C3N=C(C2C(=O)OCC)C)c4ccccc4

Supplementary Table S2:
Detailed table of XP Docking using Schrodinger's Glide module

ZINC_ID	xLogP	ApolarDesolvation	PolarDesolvation	H Donors	H Acceptors	Charge	MolecularWeight	RotatableBonds	SMILES
ZINC08440559	4.13	0.33	-60.52	2	8	1	533.576	9	COc1ccc(cc1F)C(=O)C2=C(C(=O)N([C@@H]2c3cccc(c3)Oc4ccccc4)CC[NH+]5CCOCC5)O
ZINC08440559	3.55	0.22	-71.17	1	8	1	533.576	9	COc1ccc(cc1F)C(=O)[C@H]2[C@H](N(C(=O)C2=O)CC[NH+]3CCOCC3)c4cccc(c4)Oc5ccccc5
ZINC08440558	5.94	14.38	-18.63	1	6	0	475.585	9	CCCOc1ccc(cc1OC)[C@@H]2C3=C(C[C@H](CC3=O)c4ccccc4)NC(=C2C(=O)OCC)C
ZINC08440556	2.78	6.98	-60.51	1	8	-1	452.483	11	CCOc1cc(ccc1O)[C@H]2C(=C(C(=O)N2CCOC)[O-])C(=O)c3ccc(cc3)OCC=C
ZINC08440556	3.22	-1.62	-31.99	2	8	0	453.491	10	CCOc1cc(ccc1O)[C@H]2C(=C(c3ccc(cc3)OCC=C)O)C(=O)C(=O)N2CCOC
ZINC08440556	2.19	-1.33	-26.11	1	8	0	453.491	11	CCOc1cc(ccc1O)[C@H]2[C@H](C(=O)C(=O)N2CCOC)C(=O)c3ccc(cc3)OCC=C
ZINC08440555	5.94	14.38	-18.64	1	6	0	475.585	9	CCCOc1ccc(cc1OC)[C@H]2C3=C(C[C@@H](CC3=O)c4ccccc4)NC(=C2C(=O)OCC)C
ZINC08440555	6.05	0.02	-8.96	0	6	0	475.585	9	CCCOc1ccc(cc1OC)[C@H]2C3C(=O)C[C@H](C=C3N=C(C2C(=O)OCC)C)c4ccccc4
ZINC08440554	5.94	14.38	-18.68	1	6	0	475.585	9	CCCOc1ccc(cc1OC)[C@@H]2C3=C(C[C@@H](CC3=O)c4ccccc4)NC(=C2C(=O)OCC)C
ZINC08440554	6.05	0.05	-8.98	0	6	0	475.585	9	CCCOc1ccc(cc1OC)[C@@H]2C3C(=O)C[C@H](C=C3N=C(C2C(=O)OCC)C)c4ccccc4
ZINC08440553	2.78	6.75	-60.61	1	8	-1	452.483	11	CCOc1cc(ccc1O)[C@@H]2C(=C(C(=O)N2CCOC)[O-])C(=O)c3ccc(cc3)OCC=C
ZINC08440553	3.22	-1.91	-31.81	2	8	0	453.491	10	CCOc1cc(ccc1O)[C@@H]2C(=C(c3ccc(cc3)OCC=C)O)C(=O)C(=O)N2CCOC
ZINC08440553	2.19	-1.68	-25.43	1	8	0	453.491	11	CCOc1cc(ccc1O)[C@@H]2[C@H](C(=O)C(=O)N2CCOC)C(=O)c3ccc(cc3)OCC=C
ZINC00844490	4.43	8.08	-8.61	2	6	0	431.286	6	CC1=C([C@H](NC(=O)N1)c2cccc(c2)Br)C(=O)OCc3ccc(cc3)OC
ZINC08440552	4.43	8.08	-8.33	2	6	0	431.286	6	CC1=C([C@@H](NC(=O)N1)c2cccc(c2)Br)C(=O)OCc3ccc(cc3)OC
ZINC00666441	3.52	-1.3	-15.3	2	9	0	426.425	7	CC1=C([C@H](NC(=O)N1)c2cc(ccc2OC)OC)C(=O)OCc3ccc4c(c3)OCO4
ZINC00666443	3.52	-1.35	-15.05	2	9	0	426.425	7	CC1=C([C@@H](NC(=O)N1)c2cc(ccc2OC)OC)C(=O)OCc3ccc4c(c3)OCO4
ZINC00666409	4.91	9.27	-10.55	2	5	0	362.857	5	CC1=C([C@H](NC(=O)N1)c2ccccc2Cl)C(=O)OCC3CCCCC3
ZINC08440550	4.35	9.08	-53.05	0	6	-1	461.321	9	COCCN1[C@H](C(=C(C1=O)[O-])C(=O)c2ccc(cc2)OCC=C)c3ccc(c(c3)Cl)Cl
ZINC08440550	4.79	-0.39	-31.96	1	6	0	462.329	8	COCCN1[C@H](C(=C(c2ccc(cc2)OCC=C)O)C(=O)C1=O)c3ccc(c(c3)Cl)Cl
ZINC08440550	3.76	-0.17	-24.93	0	6	0	462.329	9	COCCN1[C@H]([C@@H](C(=O)C1=O)C(=O)c2ccc(cc2)OCC=C)c3ccc(c(c3)Cl)Cl
ZINC00666412	4.91	9.24	-10.41	2	5	0	362.857	5	CC1=C([C@@H](NC(=O)N1)c2ccccc2Cl)C(=O)OCC3CCCCC3
ZINC00666403	5.03	9.9	-11.11	2	6	0	386.492	7	CC1=C([C@H](NC(=O)N1)c2ccccc2OC(C)C)C(=O)OCC3CCCCC3
ZINC00666406	5.03	9.86	-11.35	2	6	0	386.492	7	CC1=C([C@@H](NC(=O)N1)c2ccccc2OC(C)C)C(=O)OCC3CCCCC3
ZINC00759153	5.07	8.9	-8.76	2	5	0	407.308	5	CC1=C([C@H](NC(=O)N1)c2cccc(c2)Br)C(=O)OCC3CCCCC3
ZINC00759154	5.07	9.33	-6.57	2	5	0	407.308	5	CC1=C([C@@H](NC(=O)N1)c2cccc(c2)Br)C(=O)OCC3CCCCC3
ZINC08440549	4.35	9.34	-52.25	0	6	-1	461.321	9	COCCN1[C@@H](C(=C(C1=O)[O-])C(=O)c2ccc(cc2)OCC=C)c3ccc(c(c3)Cl)Cl
ZINC08440549	4.79	-0.6	-32	1	6	0	462.329	8	COCCN1[C@@H](C(=C(c2ccc(cc2)OCC=C)O)C(=O)C1=O)c3ccc(c(c3)Cl)Cl
ZINC08440549	3.76	-0.31	-27.96	0	6	0	462.329	9	COCCN1[C@@H]([C@@H](C(=O)C1=O)C(=O)c2ccc(cc2)OCC=C)c3ccc(c(c3)Cl)Cl
ZINC00666393	3.62	5.79	-12.14	3	7	0	374.437	6	CC1=C([C@H](NC(=O)N1)c2ccc(c(c2)OC)O)C(=O)OCC3CCCCC3
ZINC00666396	3.62	5.86	-11.86	3	7	0	374.437	6	CC1=C([C@@H](NC(=O)N1)c2ccc(c(c2)OC)O)C(=O)OCC3CCCCC3
ZINC00844382	3.23	3.42	-10.7	3	7	0	439.184	6	CC1=C([C@H](NC(=O)N1)c2cc(c(c(c2)Br)O)OC)C(=O)OCC(F)(F)F
ZINC00844383	3.23	-1.37	-10.8	3	7	0	439.184	6	CC1=C([C@@H](NC(=O)N1)c2cc(c(c(c2)Br)O)OC)C(=O)OCC(F)(F)F
ZINC08440547	5.49	0.95	-56.3	2	6	1	564.5	10	Cc1cc(ccc1OCc2ccccc2)C(=O)C3=C(C(=O)N([C@H]3c4cccc(c4)Br)CCC[NH+](C)C)O
ZINC08440547	4.91	0.86	-68.62	1	6	1	564.5	10	Cc1cc(ccc1OCc2ccccc2)C(=O)[C@@H]3[C@@H](N(C(=O)C3=O)CCC[NH+](C)C)c4cccc(c4)Br
ZINC00844300	4.71	-3.43	-9.28	3	7	0	453.333	5	CC1=C([C@H](NC(=O)N1)c2cc(c(c(c2)Br)O)OC)C(=O)OC3CCCCCC3
ZINC00844301	4.71	-3.47	-9.48	3	7	0	453.333	5	CC1=C([C@@H](NC(=O)N1)c2cc(c(c(c2)Br)O)OC)C(=O)OC3CCCCCC3
ZINC08440546	5.69	-0.43	-16.58	0	5	0	477.988	6	CCOc1cc(cc(c1OCc2ccccc2)Cl)[C@H]3C4C(=NC5=C3C(=O)CCC5)CCCC4=O
ZINC08440546	5.85	-1.78	-13.93	0	5	0	477.988	6	CCOc1cc(cc(c1OCc2ccccc2)Cl)[C@@H]3C4C(=NC5=CCCC(=O)C35)CCCC4=O
ZINC00844296	4.93	-0.97	-8.61	2	6	0	421.28	6	CC1=C([C@H](NC(=O)N1)c2ccc(c(c2)Cl)Cl)C(=O)OCc3ccc(cc3)OC
ZINC08440545	5.49	0.7	-48.72	2	6	1	564.5	10	Cc1cc(ccc1OCc2ccccc2)C(=O)C3=C(C(=O)N([C@@H]3c4cccc(c4)Br)CCC[NH+](C)C)O
ZINC08440545	4.91	0.58	-57.79	1	6	1	564.5	10	Cc1cc(ccc1OCc2ccccc2)C(=O)[C@H]3[C@H](N(C(=O)C3=O)CCC[NH+](C)C)c4cccc(c4)Br
ZINC00844297	4.93	-0.98	-10.58	2	6	0	421.28	6	CC1=C([C@@H](NC(=O)N1)c2ccc(c(c2)Cl)Cl)C(=O)OCc3ccc(cc3)OC
ZINC02062503	6.84	2.17	-10.03	0	3	0	422.434	4	c1ccc(cc1)C(=O)c2ccc3c(c2)nc(c(n3)c4ccc(cc4)F)c5ccc(cc5)F
ZINC02062499	7.04	16.18	-10.42	0	4	0	536.425	3	Cc1ccc(cc1)Oc2ccc(cc2)N3C(=O)[C@@H]4[C@H](C3=O)C5(c6ccccc6C4c7c5cccc7)Br
ZINC08440544	5.34	10.46	-13.69	2	6	0	429.501	7	c1ccc(cc1)c2cc(=O)[nH]c(n2)SCC(=O)Nc3ccccc3Oc4ccccc4
ZINC08440543	4.66	1.04	-45.52	2	6	1	516.456	9	Cc1cc(ccc1C(=O)C2=C(C(=O)N([C@H]2c3ccc(cc3)Br)CCC[NH+](C)C)O)OC(C)C
ZINC08440543	5.11	0.64	-57.94	2	6	1	516.456	8	Cc1cc(ccc1/C(=C\2/[C@@H](N(C(=O)C2=O)CCC[NH+](C)C)c3ccc(cc3)Br)/O)OC(C)C
ZINC08440543	4.08	0.88	-54.82	1	6	1	516.456	9	Cc1cc(ccc1C(=O)[C@@H]2[C@@H](N(C(=O)C2=O)CCC[NH+](C)C)c3ccc(cc3)Br)OC(C)C
ZINC02062501	7.04	16.16	-8.43	0	4	0	536.425	3	Cc1ccc(cc1)Oc2ccc(cc2)N3C(=O)[C@H]4[C@H](C3=O)C5(c6ccccc6C4c7c5cccc7)Br
ZINC02062500	7.04	16.15	-8.54	0	4	0	536.425	3	Cc1ccc(cc1)Oc2ccc(cc2)N3C(=O)[C@@H]4[C@@H](C3=O)C5(c6ccccc6C4c7c5cccc7)Br
ZINC02062502	7.04	16.18	-10.7	0	4	0	536.425	3	Cc1ccc(cc1)Oc2ccc(cc2)N3C(=O)[C@H]4[C@@H](C3=O)C5(c6ccccc6C4c7c5cccc7)Br
ZINC02062493	6.55	3.48	-18.54	0	4	0	440.446	3	Cc1cc2c(cc1C)n3c(=O)/c(=C/c4ccc(o4)c5cccc(c5)C(F)(F)F)/sc3n2
ZINC08440542	4.66	1.17	-48.69	2	6	1	516.456	9	Cc1cc(ccc1C(=O)C2=C(C(=O)N([C@@H]2c3ccc(cc3)Br)CCC[NH+](C)C)O)OC(C)C
ZINC08440542	5.11	0.75	-63.79	2	6	1	516.456	8	Cc1cc(ccc1/C(=C\2/[C@H](N(C(=O)C2=O)CCC[NH+](C)C)c3ccc(cc3)Br)/O)OC(C)C
ZINC08440542	4.08	1.13	-59.26	1	6	1	516.456	9	Cc1cc(ccc1C(=O)[C@H]2[C@H](N(C(=O)C2=O)CCC[NH+](C)C)c3ccc(cc3)Br)OC(C)C
ZINC02062487	7.49	3.94	-22.6	0	5	0	535.08	6	CCOc1cc(ccc1OCc2ccc(cc2)Cl)C3C4=C(CC(CC4=O)(C)C)OC5=C3C(=O)CC(C5)(C)C
ZINC02062485	6.94	1.29	-12.64	1	4	0	503.642	5	Cc1ccc(cc1)COc2ccc(cc2OC)[C@H]3C4=C(CC(CC4=O)(C)C)c5c6ccccc6ccc5N3
ZINC00702603	5.33	1.54	-13.59	0	6	0	450.56	7	CC1=C([C@H](N2C(=O)CCSC2=N1)c3cccc(c3)Oc4ccccc4)C(=O)OCC(C)C
ZINC08440541	6.49	14.79	-46.65	4	9	0	628.729	9	Cc1ccc(nc1)NC(=O)C2=C(NC3=C([C@H]2c4ccc(c(c4)COc5ccccc5C(=O)N)OC)C(=O)C[C@H](C3)c6ccccc6)C
ZINC08440541	6.62	-3.37	-21.31	3	9	0	628.729	9	Cc1ccc(nc1)NC(=O)C2[C@@H](C3C(=NC2=C)C[C@@H](CC3=O)c4ccccc4)c5ccc(c(c5)COc6ccccc6C(=O)N)OC

Supplementary Table S2:
Detailed table of XP Docking using Schrodinger's Glide module

ZINC_ID	xLogP	ApolarDesolvation	PolarDesolvation	H Donors	H Acceptors	Charge	MolecularWeight	RotatableBonds	SMILES
ZINC02062486	6.94	1.45	-13.02	1	4	0	503.642	5	Cc1ccc(cc1)COc2ccc(cc2OC)[C@@H]3C4=C(CC(CC4=O)(C)C)c5c6ccccc6ccc5N3
ZINC02062480	7.28	18.04	-52.49	0	5	-1	573.097	5	Cc1cccc(c1)N2C(=O)/C(=C/c3cc(n(c3c4ccccc4)c5ccc(cc5)Cl)c6ccccc6)/C(=NC2=S)[O-]
ZINC08440540	6.63	1.07	-10.28	1	5	0	574.105	5	Cc1ccc(cc1)N2C(=O)/C(=C\c3cc(n(c3c4ccccc4)c5ccc(cc5)Cl)c6ccccc6)/C(=O)NC2=S
ZINC00702604	5.33	1.53	-15.63	0	6	0	450.56	7	CC1=C([C@@H](N2C(=O)CCSC2=N1)c3cccc(c3)Oc4ccccc4)C(=O)OCC(C)C
ZINC08440539	7.53	3.13	-12.47	0	5	0	636.176	6	c1ccc(cc1)c2cc(c(n2c3ccc(cc3)Cl)c4ccccc4)C=C5C(=O)N(C(=S)N(C5=O)c6ccccc6)c7ccccc7
ZINC02062474	4.02	1.61	-13.76	1	7	0	507.999	6	CCOC(=O)c1ccc(cc1)n2c(cc(c2C)/C=C/3\C(=O)NC(=S)N(C3=O)c4cccc(c4)Cl)C
ZINC00702601	5.36	0.51	-13.79	0	5	0	461.37	5	CC1=C([C@H](N2C(=O)CCSC2=N1)c3ccc(cc3Cl)Cl)C(=O)OCc4ccccc4
ZINC08440537	6.49	14.94	-45.08	4	9	0	628.729	9	Cc1ccc(nc1)NC(=O)C2=C(NC3=C([C@@H]2c4ccc(c(c4)COc5ccccc5C(=O)N)OC)C(=O)C[C@H](C3)c6ccccc6)C
ZINC08440537	6.62	-3.43	-21.25	3	9	0	628.729	9	Cc1ccc(nc1)NC(=O)C2[C@H](C3C(=NC2=C)C[C@@H](CC3=O)c4ccccc4)c5ccc(c(c5)COc6ccccc6C(=O)N)OC
ZINC02062470	3.96	1.01	-12.93	1	7	0	552.45	6	CCOC(=O)c1ccc(cc1)n2c(cc(c2C)/C=C/3\C(=O)NC(=S)N(C3=O)c4ccc(cc4)Br)C
ZINC00702602	5.36	0.3	-14.01	0	5	0	461.37	5	CC1=C([C@@H](N2C(=O)CCSC2=N1)c3ccc(cc3Cl)Cl)C(=O)OCc4ccccc4
ZINC00666327	3.5	4.59	-17.39	0	7	0	535.625	6	Cc1cc(c(n1c2ccc(cc2)OC(=O)C)C)C=C3C(=O)N(C(=S)N(C3=O)c4ccccc4)c5ccccc5
ZINC00702595	4.88	0.75	-12.76	0	5	0	427.353	5	CC1=C([C@H](N2C(=O)CCSC2=N1)c3ccc(cc3Cl)Cl)C(=O)OCC(C)C
ZINC08440533	5.41	0.96	-11.87	1	7	0	525.388	6	Cc1cc(cc(c1)N2C(=O)/C(=C/c3ccc(c(c3)OC)OCc4ccc(cc4Cl)Cl)/C(=O)NC2=O)C
ZINC08440532	6.49	14.78	-46.99	4	9	0	628.729	9	Cc1ccc(nc1)NC(=O)C2=C(NC3=C([C@H]2c4ccc(c(c4)COc5ccccc5C(=O)N)OC)C(=O)C[C@@H](C3)c6ccccc6)C
ZINC08440532	6.62	-3.27	-21.86	3	9	0	628.729	9	Cc1ccc(nc1)NC(=O)C2[C@@H](C3C(=NC2=C)C[C@H](CC3=O)c4ccccc4)c5ccc(c(c5)COc6ccccc6C(=O)N)OC
ZINC00702596	4.88	0.54	-13.08	0	5	0	427.353	5	CC1=C([C@@H](N2C(=O)CCSC2=N1)c3ccc(cc3Cl)Cl)C(=O)OCC(C)C
ZINC08440531	9.2	22.98	-13.43	0	5	0	577.765	15	CCCCCCCCCCCCCOC(=O)c1ccccc1N2C(=O)[C@@H]3[C@H](C2=O)C4c5ccccc5C3c6c4cccc6
ZINC08440530	9.2	22.96	-13.21	0	5	0	577.765	15	CCCCCCCCCCCCCOC(=O)c1ccccc1N2C(=O)[C@H]3[C@H](C2=O)C4c5ccccc5C3c6c4cccc6
ZINC08440529	9.2	22.96	-12.16	0	5	0	577.765	15	CCCCCCCCCCCCCOC(=O)c1ccccc1N2C(=O)[C@@H]3[C@@H](C2=O)C4c5ccccc5C3c6c4cccc6
ZINC08440528	6.44	-2.04	-20.37	3	9	0	628.729	9	Cc1ccc(nc1)NC(=O)C2[C@H](C3=C(C[C@H](CC3=O)c4ccccc4)N=C2C)c5ccc(c(c5)COc6ccccc6C(=O)N)OC
ZINC08440528	6.44	-2.21	-24.18	3	9	0	628.729	9	Cc1ccc(nc1)NC(=O)C2=C(N=C3C[C@H](CC(=O)C3[C@@H]2c4ccc(c(c4)COc5ccccc5C(=O)N)OC)c6ccccc6)C
ZINC08440528	6.62	-3.13	-22.06	3	9	0	628.729	9	Cc1ccc(nc1)NC(=O)C2[C@H](C3C(=NC2=C)C[C@H](CC3=O)c4ccccc4)c5ccc(c(c5)COc6ccccc6C(=O)N)OC
ZINC00702587	4.59	0.24	-13.97	1	5	0	433.529	7	Cc1ccc(cc1)[C@H]2C(=C(C(=O)N2CCc3ccc(cc3)OC)O)C(=O)c4cccs4
ZINC00702587	4	0.07	-19.25	0	5	0	433.529	7	Cc1ccc(cc1)[C@H]2[C@@H](C(=O)C(=O)N2CCc3ccc(cc3)OC)C(=O)c4cccs4
ZINC02062436	7.8	19.07	-13.54	0	5	0	507.63	10	CCCCCCCCOC(=O)c1ccccc1N2C(=O)[C@@H]3[C@H](C2=O)C4c5ccccc5C3c6c4cccc6
ZINC02062438	7.8	19.06	-13.39	0	5	0	507.63	10	CCCCCCCCOC(=O)c1ccccc1N2C(=O)[C@H]3[C@H](C2=O)C4c5ccccc5C3c6c4cccc6
ZINC02062437	7.8	19.05	-12.34	0	5	0	507.63	10	CCCCCCCCOC(=O)c1ccccc1N2C(=O)[C@@H]3[C@@H](C2=O)C4c5ccccc5C3c6c4cccc6
ZINC00702588	4.59	0	-13.5	1	5	0	433.529	7	Cc1ccc(cc1)[C@@H]2C(=C(C(=O)N2CCc3ccc(cc3)OC)O)C(=O)c4cccs4
ZINC00702588	4	-0.15	-20.29	0	5	0	433.529	7	Cc1ccc(cc1)[C@@H]2[C@@H](C(=O)C(=O)N2CCc3ccc(cc3)OC)C(=O)c4cccs4
ZINC08440526	5.5	13.64	-42.6	0	6	-1	484.572	9	CCOc1ccc(cc1C)C(=O)C2=C(C(=O)N([C@H]2c3ccc(cc3)C)CCc4ccc(cc4)OC)[O-]
ZINC08440526	5.94	1.14	-28.42	1	6	0	485.58	8	CCOc1ccc(cc1C)C(=C2[C@@H](N(C(=O)C2=O)CCc3ccc(cc3)OC)c4ccc(cc4)C)O
ZINC08440526	4.91	1.37	-21.09	0	6	0	485.58	9	CCOc1ccc(cc1C)C(=O)[C@H]2[C@@H](N(C(=O)C2=O)CCc3ccc(cc3)OC)c4ccc(cc4)C
ZINC02062435	6.41	0.52	-9.19	0	6	0	505.016	6	COc1cc(c(cc1Cn2c(c(nc2Cl)Cl)Cl)OC)Cn3c(c(nc3Cl)Cl)Cl
ZINC02062434	6.92	2.43	-12.34	0	4	0	467.348	6	c1cc(ccc1COc2cc3c(c(c2)OCc4ccc(cc4)Cl)c5c(c(=O)o3)CCC5)Cl
ZINC01797481	8.2	3.25	-9.27	0	4	0	503.381	7	c1ccc(cc1)c2cc(=O)oc3c2c(cc(c3)OCc4ccccc4Cl)OCc5ccccc5Cl
ZINC00702586	5.5	13.23	-42.07	0	6	-1	484.572	9	CCOc1ccc(cc1C)C(=O)C2=C(C(=O)N([C@@H]2c3ccc(cc3)C)CCc4ccc(cc4)OC)[O-]
ZINC00702586	5.94	0.92	-27.68	1	6	0	485.58	8	CCOc1ccc(cc1C)C(=C2[C@H](N(C(=O)C2=O)CCc3ccc(cc3)OC)c4ccc(cc4)C)O
ZINC00702586	4.91	1.22	-21.94	0	6	0	485.58	9	CCOc1ccc(cc1C)C(=O)[C@H]2[C@H](N(C(=O)C2=O)CCc3ccc(cc3)OC)c4ccc(cc4)C
ZINC02062426	7.69	2.85	-11.05	0	4	0	477.343	6	c1ccc2c(c1)c3c(cc(cc3OCc4ccc(cc4)Cl)OCc5ccc(cc5)Cl)oc2=O
ZINC08440524	1.34	1.59	-66.13	0	8	-1	423.445	9	COc1ccc(cc1)CCN([C@H]2CC(=O)N(C2=O)c3ccccc3)C(=O)CCC(=O)[O-]
ZINC00702583	0.86	11.99	-29.94	2	7	1	469.974	3	Cc1cccn2c1nc3c(c2=O)cc(c([n+]3C4CCCC4)N)S(=O)(=O)c5ccc(cc5)Cl
ZINC08440522	6.3	-1.24	-9.84	1	4	0	458.414	6	CCCSC1=NC(=C([C@@H](C1C#N)c2ccccc2Cl)C(=O)Nc3ccc(cc3)Cl)C
ZINC08440522	5.92	-0.82	-14.9	1	4	0	458.414	6	CCCSC1=C([C@H](C(C(=N1)C)C(=O)Nc2ccc(cc2)Cl)c3ccccc3Cl)C#N
ZINC08440522	5.92	0.69	-10.24	1	4	0	458.414	6	CCCSC1=C([C@H]([C@H](C(=N1)C)C(=O)Nc2ccc(cc2)Cl)c3ccccc3Cl)C#N
ZINC08440521	6.3	-0.98	-9.99	1	4	0	458.414	6	CCCSC1=NC(=C([C@H](C1C#N)c2ccccc2Cl)C(=O)Nc3ccc(cc3)Cl)C
ZINC08440521	5.92	-0.91	-12.92	1	4	0	458.414	6	CCCSC1=C([C@@H](C(C(=N1)C)C(=O)Nc2ccc(cc2)Cl)c3ccccc3Cl)C#N
ZINC08440521	5.92	0.86	-9.8	1	4	0	458.414	6	CCCSC1=C([C@@H]([C@@H](C(=N1)C)C(=O)Nc2ccc(cc2)Cl)c3ccccc3Cl)C#N
ZINC08440520	6.37	-0.45	-9.18	3	5	0	491.922	6	CCOc1ccc(cc1)NC(=O)c2c(c3c(cc(nc3s2)c4ccc(cc4)Cl)C(F)(F)F)N
ZINC08440519	4.6	12.32	-17.84	2	5	0	433.577	4	CC1=C([C@@H](C2=C(N1)CC(CC2=O)(C)C)c3ccc(cc3)SC)C(=O)Nc4ccccn4
ZINC06444324	7.59	17.91	-24.79	2	5	0	567.78	8	Cc1ccccc1NC(=O)C2=C(NC(=C([C@H]2c3cccs3)C#N)SCC(=O)c4ccc(cc4)C5CCCCC5)C
ZINC06444324	7.93	-0.4	-12.25	1	5	0	567.78	8	Cc1ccccc1NC(=O)C2=C(N=C(C([C@H]2c3cccs3)C#N)SCC(=O)c4ccc(cc4)C5CCCCC5)C
ZINC06444324	8.11	-1.43	-14.32	1	5	0	567.78	8	Cc1ccccc1NC(=O)C2[C@@H](C(C(=NC2=C)SCC(=O)c3ccc(cc3)C4CCCCC4)C#N)c5cccs5
ZINC00702582	3.75	-3.29	-11.53	2	5	0	425.941	5	c1cc(cnc1)CNC(=O)c2c3c(sc2NC(=O)c4ccc(cc4)Cl)CCCC3
ZINC06444294	7.59	17.87	-22.84	2	5	0	567.78	8	Cc1ccccc1NC(=O)C2=C(NC(=C([C@@H]2c3cccs3)C#N)SCC(=O)c4ccc(cc4)C5CCCCC5)C
ZINC06444294	7.93	-0.4	-13.23	1	5	0	567.78	8	Cc1ccccc1NC(=O)C2=C(N=C(C([C@@H]2c3cccs3)C#N)SCC(=O)c4ccc(cc4)C5CCCCC5)C
ZINC06444294	8.11	-1.17	-13.18	1	5	0	567.78	8	Cc1ccccc1NC(=O)C2[C@H](C(C(=NC2=C)SCC(=O)c3ccc(cc3)C4CCCCC4)C#N)c5cccs5
ZINC08440518	7.04	-0.39	-12.37	1	6	0	557.741	11	CCCCc1ccc(cc1)C(=O)CSC2=NC(=C([C@@H](C2C#N)c3cccs3)C(=O)Nc4ccccc4OC)C
ZINC08440518	6.66	-0.35	-14.64	1	6	0	557.741	11	CCCCc1ccc(cc1)C(=O)CSC2=C([C@H](C(C(=N2)C)C(=O)Nc3ccccc3OC)c4cccs4)C#N
ZINC08440518	6.66	1.54	-13.42	1	6	0	557.741	11	CCCCc1ccc(cc1)C(=O)CSC2=C([C@H]([C@H](C(=N2)C)C(=O)Nc3ccccc3OC)c4cccs4)C#N
ZINC08440517	6.47	15.73	-25.54	2	10	0	633.499	8	Cc1ccc(c(c1)[N+](=O)[O-])OCc2cc(ccc2OC)[C@H]3C4=C(CCCC4=O)NC(=C3C(=O)Nc5ccc(cn5)Br)C

Supplementary Table S2:
Detailed table of XP Docking using Schrodinger's Glide module

ZINC_ID	xLogP	ApolarDesolvation	PolarDesolvation	H Donors	H Acceptors	Charge	MolecularWeight	RotatableBonds	SMILES
ZINC08440517	6.59	-2.47	-15.95	1	10	0	633.499	8	Cc1ccc(c(c1)[N+](=O)[O-])OCc2cc(ccc2OC)[C@H]3C4C(=O)CCC=C4N=C(C3C(=O)Nc5ccc(cn5)Br)C
ZINC08440516	4.81	-0.44	-13.52	1	7	0	528.428	8	CCCCOc1ccc(cc1)C(=O)C2=C(C(=O)N([C@H]2c3ccc(cc3)Br)c4nnc(s4)C)O
ZINC08440516	4.23	-0.6	-16.08	0	7	0	528.428	8	CCCCOc1ccc(cc1)C(=O)[C@H]2[C@@H](N(C(=O)C2=O)c3nnc(s3)C)c4ccc(cc4)Br
ZINC08440515	7.04	-0.39	-13.37	1	6	0	557.741	11	CCCCc1ccc(cc1)C(=O)CSC2=NC(=C([C@H](C2C#N)c3cccs3)C(=O)Nc4ccccc4OC)C
ZINC08440515	6.66	0.03	-17.17	1	6	0	557.741	11	CCCCc1ccc(cc1)C(=O)CSC2=C([C@@H](C(C(=N2)C)C(=O)Nc3ccccc3OC)c4cccs4)C#N
ZINC08440515	6.66	1.51	-14.21	1	6	0	557.741	11	CCCCc1ccc(cc1)C(=O)CSC2=C([C@@H]([C@@H](C(=N2)C)C(=O)Nc3ccccc3OC)c4cccs4)C#N
ZINC08440512	4.81	-0.46	-13.49	1	7	0	528.428	8	CCCCOc1ccc(cc1)C(=O)C2=C(C(=O)N([C@@H]2c3ccc(cc3)Br)c4nnc(s4)C)O
ZINC08440512	4.23	-0.54	-17.72	0	7	0	528.428	8	CCCCOc1ccc(cc1)C(=O)[C@H]2[C@H](N(C(=O)C2=O)c3nnc(s3)C)c4ccc(cc4)Br
ZINC00841851	5.65	2.26	-12.71	0	4	0	422.937	5	Cc1ccc(o1)c2c3c(nc(c2C#N)SCC(=O)c4ccc(cc4)Cl)CCCC3
ZINC06444297	5.76	13.49	-30.05	2	6	0	515.66	8	Cc1ccccc1NC(=O)C2=C(NC(=C([C@H]2c3ccc(cc3)OC)C#N)SCC(=O)c4cccs4)C
ZINC06444297	6.09	-0.94	-14.36	1	6	0	515.66	8	Cc1ccccc1NC(=O)C2=C(N=C(C([C@H]2c3ccc(cc3)OC)C#N)SCC(=O)c4cccs4)C
ZINC06444297	6.28	-1.9	-14.36	1	6	0	515.66	8	Cc1ccccc1NC(=O)C2[C@@H](C(C(=NC2=C)SCC(=O)c3cccs3)C#N)c4ccc(cc4)OC
ZINC00702574	3.98	1.75	-19.39	1	9	0	540.569	9	CCOC(=O)c1c(nc(s1)N2[C@H](C(=C(C2=O)O)C(=O)c3ccc(c(c3)F)C)c4ccc(c(c4)OC)OC)C
ZINC00702574	3.4	1.59	-23.67	0	9	0	540.569	9	CCOC(=O)c1c(nc(s1)N2[C@H]([C@@H](C(=O)C2=O)C(=O)c3ccc(c(c3)F)C)c4ccc(c(c4)OC)OC)C
ZINC08440511	6.47	15.71	-25.25	2	10	0	633.499	8	Cc1ccc(c(c1)[N+](=O)[O-])OCc2cc(ccc2OC)[C@@H]3C4=C(CCCC4=O)NC(=C3C(=O)Nc5ccc(cn5)Br)C
ZINC08440511	6.59	-2.5	-15.93	1	10	0	633.499	8	Cc1ccc(c(c1)[N+](=O)[O-])OCc2cc(ccc2OC)[C@@H]3C4C(=O)CCC=C4N=C(C3C(=O)Nc5ccc(cn5)Br)C
ZINC06444263	5.76	13.62	-21.98	2	6	0	515.66	8	Cc1ccccc1NC(=O)C2=C(NC(=C([C@@H]2c3ccc(cc3)OC)C#N)SCC(=O)c4cccs4)C
ZINC06444263	6.09	-0.42	-13.5	1	6	0	515.66	8	Cc1ccccc1NC(=O)C2=C(N=C(C([C@@H]2c3ccc(cc3)OC)C#N)SCC(=O)c4cccs4)C
ZINC06444263	6.28	-1.28	-14.59	1	6	0	515.66	8	Cc1ccccc1NC(=O)C2[C@H](C(C(=NC2=C)SCC(=O)c3cccs3)C#N)c4ccc(cc4)OC
ZINC08440510	4.42	10.18	-23.25	2	5	0	425.941	5	CC1=C([C@H](C(=C(N1)SC)C#N)c2ccccc2Cl)C(=O)Nc3ccccc3OC
ZINC08440510	4.94	-0.87	-10.41	1	5	0	425.941	5	COc1ccccc1NC(=O)C2[C@H](C(C(=NC2=C)SC)C#N)c3ccccc3Cl
ZINC08440510	4.94	0.94	-15.84	1	5	0	425.941	5	COc1ccccc1NC(=O)C2[C@H]([C@H](C(=NC2=C)SC)C#N)c3ccccc3Cl
ZINC08440510	4.76	-0.64	-11.56	1	5	0	425.941	5	CC1=C([C@H]([C@@H](C(=N1)SC)C#N)c2ccccc2Cl)C(=O)Nc3ccccc3OC
ZINC08440509	4.42	10.14	-15.39	2	5	0	425.941	5	CC1=C([C@@H](C(=C(N1)SC)C#N)c2ccccc2Cl)C(=O)Nc3ccccc3OC
ZINC08440509	4.94	-0.71	-8.91	1	5	0	425.941	5	COc1ccccc1NC(=O)C2[C@@H](C(C(=NC2=C)SC)C#N)c3ccccc3Cl
ZINC08440509	4.94	1.16	-16.36	1	5	0	425.941	5	COc1ccccc1NC(=O)C2[C@@H]([C@@H](C(=NC2=C)SC)C#N)c3ccccc3Cl
ZINC08440509	4.76	-0.4	-8.75	1	5	0	425.941	5	CC1=C([C@@H]([C@@H](C(=N1)SC)C#N)c2ccccc2Cl)C(=O)Nc3ccccc3OC
ZINC00702575	3.98	1.74	-19.63	1	9	0	540.569	9	CCOC(=O)c1c(nc(s1)N2[C@@H](C(=C(C2=O)O)C(=O)c3ccc(c(c3)F)C)c4ccc(c(c4)OC)OC)C
ZINC00702575	3.4	1.65	-26.06	0	9	0	540.569	9	CCOC(=O)c1c(nc(s1)N2[C@@H]([C@@H](C(=O)C2=O)C(=O)c3ccc(c(c3)F)C)c4ccc(c(c4)OC)OC)C
ZINC08440508	4.66	-0.07	-11.57	1	6	0	453.589	8	CCOC(=O)CSC1=NC(=C([C@@H](C1C#N)c2cccs2)C(=O)Nc3ccccc3C)C
ZINC08440508	4.28	0.09	-12.58	1	6	0	453.589	8	CCOC(=O)CSC1=C([C@H](C(C(=N1)C)C(=O)Nc2ccccc2C)c3cccs3)C#N
ZINC08440508	4.28	1.92	-12.59	1	6	0	453.589	8	CCOC(=O)CSC1=C([C@H]([C@H](C(=N1)C)C(=O)Nc2ccccc2C)c3cccs3)C#N
ZINC08440507	4.66	-0.07	-12.05	1	6	0	453.589	8	CCOC(=O)CSC1=NC(=C([C@H](C1C#N)c2cccs2)C(=O)Nc3ccccc3C)C
ZINC08440507	4.28	0.39	-15.72	1	6	0	453.589	8	CCOC(=O)CSC1=C([C@@H](C(C(=N1)C)C(=O)Nc2ccccc2C)c3cccs3)C#N
ZINC08440507	4.28	1.83	-12.7	1	6	0	453.589	8	CCOC(=O)CSC1=C([C@@H]([C@@H](C(=N1)C)C(=O)Nc2ccccc2C)c3cccs3)C#N
ZINC08440504	3.75	10.24	-52.42	2	6	1	443.589	9	Cc1cc(ccc1OC(C)C)C(=O)C2=C(C(=O)N([C@H]2c3cccs3)CCC[NH+](C)C)O
ZINC08440504	3.17	10.96	-60.85	1	6	1	443.589	9	Cc1cc(ccc1OC(C)C)C(=O)[C@H]2[C@@H](N(C(=O)C2=O)CCC[NH+](C)C)c3cccs3
ZINC08440503	5.39	14.47	-23.39	2	5	0	503.624	7	Cc1ccccc1NC(=O)C2=C(NC(=C([C@H]2c3cccs3)C#N)SCC(=O)c4ccc(cc4)F)C
ZINC08440503	5.73	0.05	-13.08	1	5	0	503.624	7	Cc1ccccc1NC(=O)C2=C(N=C(C([C@H]2c3cccs3)C#N)SCC(=O)c4ccc(cc4)F)C
ZINC08440503	5.91	-0.98	-14.61	1	5	0	503.624	7	Cc1ccccc1NC(=O)C2[C@@H](C(C(=NC2=C)SCC(=O)c3ccc(cc3)F)C#N)c4cccs4
ZINC08440502	3.75	10.04	-46.13	2	6	1	443.589	9	Cc1cc(ccc1OC(C)C)C(=O)C2=C(C(=O)N([C@@H]2c3cccs3)CCC[NH+](C)C)O
ZINC08440502	3.17	10.7	-55.4	1	6	1	443.589	9	Cc1cc(ccc1OC(C)C)C(=O)[C@H]2[C@H](N(C(=O)C2=O)CCC[NH+](C)C)c3cccs3
ZINC08440501	2.56	9.64	-55.78	2	7	1	438.548	9	Cc1cc(ccc1OC(C)C)C(=O)C2=C(C(=O)N([C@H]2c3ccncc3)CCC[NH+](C)C)O
ZINC08440501	1.98	10.34	-64.83	1	7	1	438.548	9	Cc1cc(ccc1OC(C)C)C(=O)[C@H]2[C@@H](N(C(=O)C2=O)CCC[NH+](C)C)c3ccncc3
ZINC08440500	6.47	16.95	-21.56	2	8	0	596.687	8	CC1=C([C@@H](C2=C(N1)C[C@H](CC2=O)c3ccccc3)c4ccc(c(c4)COc5ccc(cc5)C#N)OC)C(=O)Nc6ccccn6
ZINC08440500	6.61	1.53	-15.95	1	8	0	596.687	8	COc1ccc(cc1COc2ccc(cc2)C#N)[C@@H]3[C@H]4C(=NC(=C)C3C(=O)Nc5ccccn5)C[C@H](CC4=O)c6ccccc6
ZINC08440500	6.61	0.46	-18.12	1	8	0	596.687	8	COc1ccc(cc1COc2ccc(cc2)C#N)[C@@H]3C4C(=NC(=C)C3C(=O)Nc5ccccn5)C[C@H](CC4=O)c6ccccc6
ZINC08440499	2.56	9.29	-47.49	2	7	1	438.548	9	Cc1cc(ccc1OC(C)C)C(=O)C2=C(C(=O)N([C@@H]2c3ccncc3)CCC[NH+](C)C)O
ZINC08440499	1.98	9.95	-56.89	1	7	1	438.548	9	Cc1cc(ccc1OC(C)C)C(=O)[C@H]2[C@H](N(C(=O)C2=O)CCC[NH+](C)C)c3ccncc3
ZINC08440498	5.39	14.29	-31.81	2	5	0	503.624	7	Cc1ccccc1NC(=O)C2=C(NC(=C([C@@H]2c3cccs3)C#N)SCC(=O)c4ccc(cc4)F)C
ZINC08440498	5.73	0.06	-14.06	1	5	0	503.624	7	Cc1ccccc1NC(=O)C2=C(N=C(C([C@@H]2c3cccs3)C#N)SCC(=O)c4ccc(cc4)F)C
ZINC08440498	5.91	-0.72	-13.76	1	5	0	503.624	7	Cc1ccccc1NC(=O)C2[C@H](C(C(=NC2=C)SCC(=O)c3ccc(cc3)F)C#N)c4cccs4
ZINC08440495	2.18	1.01	-18.53	1	8	0	427.453	11	COCCCN1[C@H](C(=C(C1=O)O)C(=O)c2ccco2)c3ccc(c(c3)OC)OCC=C
ZINC08440495	1.6	0.91	-26.36	0	8	0	427.453	11	COCCCN1[C@H]([C@@H](C(=O)C1=O)C(=O)c2ccco2)c3ccc(c(c3)OC)OCC=C
ZINC08440494	9.12	2.84	-14.37	0	4	0	546.736	11	CCCCc1ccc(cc1)C(=O)CSc2c(c(c3c(n2)CCCC3)c4ccc(cc4)OCc5ccccc5)C#N
ZINC08440493	2.18	1.08	-18.35	1	8	0	427.453	11	COCCCN1[C@@H](C(=C(C1=O)O)C(=O)c2ccco2)c3ccc(c(c3)OC)OCC=C
ZINC08440493	1.6	0.94	-26.11	0	8	0	427.453	11	COCCCN1[C@@H]([C@H](C(=O)C1=O)C(=O)c2ccco2)c3ccc(c(c3)OC)OCC=C
ZINC08440492	7.57	2.86	-12.86	0	5	0	520.654	10	c1ccc(cc1)COc2ccc(cc2)c3c4c(nc(c3C#N)SCC(=O)OCc5ccccc5)CCCC4
ZINC02062389	6.58	-1.43	-10.71	1	4	0	461.29	3	c1cc(cc(c1)F)C(=O)Nc2ccc3c(c2)nc(o3)c4cccc5c4cccc5Br
ZINC08440491	6.47	16.96	-21.83	2	8	0	596.687	8	CC1=C([C@H](C2=C(N1)C[C@@H](CC2=O)c3ccccc3)c4ccc(c(c4)COc5ccc(cc5)C#N)OC)C(=O)Nc6ccccn6
ZINC08440491	6.61	0.4	-17.87	1	8	0	596.687	8	COc1ccc(cc1COc2ccc(cc2)C#N)[C@H]3C4C(=NC(=C)C3C(=O)Nc5ccccn5)C[C@@H](CC4=O)c6ccccc6

Supplementary Table S2:
Detailed table of XP Docking using Schrodinger's Glide module

ZINC_ID	xLogP	ApolarDesolvation	PolarDesolvation	H Donors	H Acceptors	Charge	MolecularWeight	RotatableBonds	SMILES
ZINC08440491	6.61	1.48	-15.89	1	8	0	596.687	8	COc1ccc(cc1COc2ccc(cc2)C#N)[C@H]3[C@@H]4C(=NC(=C)C3C(=O)Nc5ccccn5)C[C@@H](CC4=O)c6ccccc6
ZINC08440490	3.23	2.32	-56.55	2	8	1	471.574	12	CC(C)CCOc1ccc(cc1OC)[C@H]2C(=C(C(=O)N2CCC[NH+](C)C)O)C(=O)c3ccco3
ZINC08440490	2.65	2.23	-67.26	1	8	1	471.574	12	CC(C)CCOc1ccc(cc1OC)[C@H]2[C@@H](C(=O)C(=O)N2CCC[NH+](C)C)C(=O)c3ccco3
ZINC08440489	5.59	-1.22	-9.21	1	3	0	540.142	4	c1cc(ccc1Cc2ccncc2)NC(=O)c3cc(ccc3I)I
ZINC06444923	4.87	-4.39	-7.63	1	4	0	534.135	3	c1cc(ccc1NC(=O)c2cc(ccc2I)I)N3CCOCC3
ZINC06445251	3.85	6.93	-6.6	1	3	0	450.017	2	c1cc(cnc1)NC(=O)c2cc(ccc2I)I
ZINC02062388	6.74	-2.99	-13.94	1	4	0	506.581	3	c1cc(ccc1c2nc3cc(ccc3o2)NC(=O)c4cc(ccc4Cl)Br)Br
ZINC08440488	3.23	2.39	-55.45	2	8	1	471.574	12	CC(C)CCOc1ccc(cc1OC)[C@@H]2C(=C(C(=O)N2CCC[NH+](C)C)O)C(=O)c3ccco3
ZINC08440488	2.65	2.26	-65.88	1	8	1	471.574	12	CC(C)CCOc1ccc(cc1OC)[C@@H]2[C@H](C(=O)C(=O)N2CCC[NH+](C)C)C(=O)c3ccco3
ZINC02062387	6.09	-1.49	-14.29	1	4	0	445.675	3	c1cc(ccc1c2nc3cc(ccc3o2)NC(=O)c4cc(ccc4Cl)Br)F
ZINC02811423	6.04	-1.52	-13	1	4	0	445.675	3	c1ccc(c(c1)c2nc3cc(ccc3o2)NC(=O)c4cc(ccc4Cl)Br)F
ZINC08440486	4.53	1.85	-53.57	2	6	1	472.005	9	Cc1cc(ccc1OC(C)C)C(=O)C2=C(C(=O)N([C@H]2c3ccc(cc3)Cl)CCC[NH+](C)C)O
ZINC08440486	3.94	1.67	-62.59	1	6	1	472.005	9	Cc1cc(ccc1OC(C)C)C(=O)[C@H]2[C@@H](N(C(=O)C2=O)CCC[NH+](C)C)c3ccc(cc3)Cl
ZINC00841474	5.93	-2.23	-15.97	1	4	0	427.685	3	c1ccc(cc1)c2nc3cc(ccc3o2)NC(=O)c4cc(ccc4Cl)Br
ZINC02062386	6.97	-1.37	-16.32	1	3	0	457.78	3	Cc1ccc(cc1NC(=O)c2cc(ccc2Cl)Br)c3nc4ccccc4s3
ZINC08440485	6.42	-0.13	-21.78	1	8	0	596.687	8	CC1=NC2=C([C@H](C1C(=O)Nc3ccccn3)c4ccc(c(c4)COc5ccc(cc5)C#N)OC)C(=O)C[C@H](C2)c6ccccc6
ZINC08440485	6.42	0.26	-22.49	1	8	0	596.687	8	CC1=C([C@@H]([C@H]2C(=N1)C[C@@H](CC2=O)c3ccccc3)c4ccc(c(c4)COc5ccc(cc5)C#N)OC)C(=O)Nc6ccccn6
ZINC08440485	6.61	1.16	-21.6	1	8	0	596.687	8	COc1ccc(cc1COc2ccc(cc2)C#N)[C@@H]3[C@H]4C(=NC(=C)C3C(=O)Nc5ccccn5)C[C@@H](CC4=O)c6ccccc6
ZINC08440485	6.61	-0.07	-20.41	1	8	0	596.687	8	COc1ccc(cc1COc2ccc(cc2)C#N)[C@@H]3C4C(=NC(=C)C3C(=O)Nc5ccccn5)C[C@@H](CC4=O)c6ccccc6
ZINC08440484	4.53	1.8	-53.75	2	6	1	472.005	9	Cc1cc(ccc1OC(C)C)C(=O)C2=C(C(=O)N([C@@H]2c3ccc(cc3)Cl)CCC[NH+](C)C)O
ZINC08440484	3.94	1.63	-62.8	1	6	1	472.005	9	Cc1cc(ccc1OC(C)C)C(=O)[C@@H]2[C@H](N(C(=O)C2=O)CCC[NH+](C)C)c3ccc(cc3)Cl
ZINC00841471	5.34	-0.23	-11.66	1	6	0	471.742	5	CCOc1ccc(cc1)n2nc3ccc(cc3n2)NC(=O)c4cc(ccc4Cl)Br
ZINC00702563	5.75	-0.28	-10.4	3	6	0	487.503	7	CCOc1ccc(cc1)c2cc(c3c(c(sc3n2)C(=O)Nc4ccc(cc4)OC)N)C(F)(F)F
ZINC08440482	5.96	0.89	-7.26	1	4	0	489.291	4	c1ccc(cc1)c2ccc(cc2)C(=O)N3[C@](CC(=N3)C(F)(F)F)(c4ccc(cc4)Br)O
ZINC05920825	4.27	11.31	-43.22	0	6	-1	436.528	10	CCOCCCN1[C@H](C(=C(C1=O)[O-])C(=O)c2ccc(c(c2)C)OCC)c3ccc(cc3)C
ZINC05920825	4.71	0.3	-26.79	1	6	0	437.536	9	CCOCCCN1[C@H](C(=C(c2ccc(c(c2)C)OCC)O)C(=O)C1=O)c3ccc(cc3)C
ZINC05920825	3.68	0.51	-18.67	0	6	0	437.536	10	CCOCCCN1[C@H]([C@@H](C(=O)C1=O)C(=O)c2ccc(c(c2)C)OCC)c3ccc(cc3)C
ZINC08440481	5.96	0.89	-7.08	1	4	0	489.291	4	c1ccc(cc1)c2ccc(cc2)C(=O)N3[C@@](CC(=N3)C(F)(F)F)(c4ccc(cc4)Br)O
ZINC05920823	4.27	11.35	-42.77	0	6	-1	436.528	10	CCOCCCN1[C@@H](C(=C(C1=O)[O-])C(=O)c2ccc(c(c2)C)OCC)c3ccc(cc3)C
ZINC05920823	4.71	0.01	-28.01	1	6	0	437.536	9	CCOCCCN1[C@@H](C(=C(c2ccc(c(c2)C)OCC)O)C(=O)C1=O)c3ccc(cc3)C
ZINC05920823	3.68	0.31	-23.04	0	6	0	437.536	10	CCOCCCN1[C@@H]([C@@H](C(=O)C1=O)C(=O)c2ccc(c(c2)C)OCC)c3ccc(cc3)C
ZINC08440480	5.66	1.54	-17.34	0	5	0	451.607	6	CCCCOc1ccc(cc1OC)[C@@H]2C3C(=NC4=C2C(=O)CC(C4)(C)C)CC(CC3=O)(C)C
ZINC08440480	5.82	0.59	-13.71	0	5	0	451.607	6	CCCCOc1ccc(cc1OC)[C@H]2C3C(=NC4=CC(CC(=O)C24)(C)C)CC(CC3=O)(C)C
ZINC00702562	0.38	12.58	-34.86	2	7	1	457.535	4	Cc1cccn2c1nc3c(c2=O)cc(c([n+]3Cc4ccccc4)N)S(=O)(=O)c5ccccc5
ZINC00839402	6.18	13.37	-24.2	2	6	0	461.521	7	C[C@H](C(=O)Nc1cccc(c1)c2[nH]c3cc(ccc3n2)C(=O)c4ccccc4)Oc5ccccc5
ZINC00839398	6.18	13.4	-24.21	2	6	0	461.521	7	C[C@@H](C(=O)Nc1cccc(c1)c2[nH]c3cc(ccc3n2)C(=O)c4ccccc4)Oc5ccccc5
ZINC02062381	7.65	4	-9.21	0	3	0	472.584	7	CCOc1ccc(cc1)C(=O)/C=C/C2=C(Oc3ccccc3[C@H]2c4ccccc4C)c5ccccc5
ZINC02062383	7.65	4.01	-10.07	0	3	0	472.584	7	CCOc1ccc(cc1)C(=O)/C=C/C2=C(Oc3ccccc3[C@@H]2c4ccccc4C)c5ccccc5
ZINC02062377	6.51	4.7	-15.56	0	6	0	548.635	9	Cc1ccccc1[C@H]2c3ccccc3OC(=C2/C=C/C(=O)c4ccc(c(c4)OC)OC)c5ccc(c(c5)OC)OC
ZINC08440479	7.51	2.58	-23.05	1	7	0	657.664	8	Cc1ccnc(c1)NC(=O)C2[C@@H](C3=C(C[C@@H](CC3=O)c4ccccc4)N=C2C)c5ccc(c(c5)COc6c(c(cc(c6F)F)F)F)OC
ZINC08440479	7.51	2.19	-22.41	1	7	0	657.664	8	Cc1ccnc(c1)NC(=O)C2=C(N=C3C[C@@H](CC(=O)C3[C@H]2c4ccc(c(c4)COc5c(c(cc(c5F)F)F)F)OC)c6ccccc6)C
ZINC08440479	7.69	1.35	-21.05	1	7	0	657.664	8	Cc1ccnc(c1)NC(=O)C2[C@@H](C3C(=NC2=C)C[C@@H](CC3=O)c4ccccc4)c5ccc(c(c5)COc6c(c(cc(c6F)F)F)F)OC
ZINC00702561	0.69	12.07	-30	2	7	1	449.556	3	Cc1cccn2c1nc3c(c2=O)cc(c([n+]3C4CCCCC4)N)S(=O)(=O)c5ccccc5
ZINC02062379	6.51	4.71	-13.61	0	6	0	548.635	9	Cc1ccccc1[C@@H]2c3ccccc3OC(=C2/C=C/C(=O)c4ccc(c(c4)OC)OC)c5ccc(c(c5)OC)OC
ZINC08440476	6.92	14.07	-18.19	1	6	0	515.224	8	CCCCOC(=O)c1cc(ccc1Cl)N/C=C/2\C(=NN(C2=O)c3c(cc(cc3Cl)Cl)Cl)C
ZINC08440475	5.86	12.49	-18.43	1	6	0	487.17	6	CCOC(=O)c1cc(ccc1Cl)N/C=C/2\C(=NN(C2=O)c3c(cc(cc3Cl)Cl)Cl)C
ZINC08440474	5.48	11.54	-18.96	1	6	0	473.143	5	CC\1=NN(C(=O)/C1=C/Nc2ccc(c(c2)C(=O)OC)Cl)c3c(cc(cc3Cl)Cl)Cl
ZINC08440473	7.55	17.41	-31.84	2	7	0	657.664	8	Cc1ccnc(c1)NC(=O)C2=C(NC3=C([C@@H]2c4ccc(c(c4)COc5c(c(cc(c5F)F)F)F)OC)C(=O)C[C@H](C3)c6ccccc6)C
ZINC08440473	7.69	1.83	-15.31	1	7	0	657.664	8	Cc1ccnc(c1)NC(=O)C2[C@H](C3C(=NC2=C)C[C@@H](CC3=O)c4ccccc4)c5ccc(c(c5)COc6c(c(cc(c6F)F)F)F)OC
ZINC00702555	4.79	12.15	-17.13	1	9	0	584.65	10	Cc1c(sc(n1)N2[C@H](C(=C(C2=O)O)C(=O)c3ccc(cc3)OC)c4ccc(c(c4)OC)OCc5ccccc5)C(=O)C
ZINC00702555	4.2	12.82	-20.76	0	9	0	584.65	10	Cc1c(sc(n1)N2[C@H]([C@H](C(=O)C2=O)C(=O)c3ccc(cc3)OC)c4ccc(c(c4)OC)OCc5ccccc5)C(=O)C
ZINC00702556	4.79	0.37	-16.62	1	9	0	584.65	10	Cc1c(sc(n1)N2[C@@H](C(=C(C2=O)O)C(=O)c3ccc(cc3)OC)c4ccc(c(c4)OC)OCc5ccccc5)C(=O)C
ZINC00702556	4.2	0.2	-18.16	0	9	0	584.65	10	Cc1c(sc(n1)N2[C@@H]([C@H](C(=O)C2=O)C(=O)c3ccc(cc3)OC)c4ccc(c(c4)OC)OCc5ccccc5)C(=O)C
ZINC08440467	7.55	17.26	-33.55	2	7	0	657.664	8	Cc1ccnc(c1)NC(=O)C2=C(NC3=C([C@H]2c4ccc(c(c4)COc5c(c(cc(c5F)F)F)F)OC)C(=O)C[C@@H](C3)c6ccccc6)C
ZINC08440467	7.69	1.83	-15.77	1	7	0	657.664	8	Cc1ccnc(c1)NC(=O)C2[C@@H](C3C(=NC2=C)C[C@H](CC3=O)c4ccccc4)c5ccc(c(c5)COc6c(c(cc(c6F)F)F)F)OC
ZINC08440465	3.55	10.58	-46.83	0	7	-1	447.492	6	Cc1c(sc(n1)N2[C@H](C(=C(C2=O)[O-])C(=O)c3ccc(cc3)OC)c4ccccc4)C(=O)C
ZINC08440465	3.99	-0.59	-23.81	1	7	0	448.5	5	Cc1c(sc(n1)N2[C@H](C(=C(c3ccc(cc3)OC)O)C(=O)C2=O)c4ccccc4)C(=O)C
ZINC08440465	2.96	-0.36	-14.85	0	7	0	448.5	6	Cc1c(sc(n1)N2[C@H]([C@@H](C(=O)C2=O)C(=O)c3ccc(cc3)OC)c4ccccc4)C(=O)C
ZINC02062354	2.25	8.77	-14.05	2	9	0	438.465	6	CCOC(=O)c1c(c(c(s1)N/C=C/2\C(=O)NC(=O)N(C2=O)c3ccc(cc3)C)C#N)C
ZINC08440462	3.05	10.47	-13.82	2	8	0	453.52	6	CCOC(=O)c1c2c(sc1N/C=C/3\C(=O)NC(=O)N(C3=O)c4ccc(cc4)C)CCCC2
ZINC00702554	3.55	10.11	-37.15	0	7	-1	447.492	6	Cc1c(sc(n1)N2[C@@H](C(=C(C2=O)[O-])C(=O)c3ccc(cc3)OC)c4ccccc4)C(=O)C

Supplementary Table S2:
Detailed table of XP Docking using Schrodinger's Glide module

ZINC_ID	xLogP	ApolarDesolvation	PolarDesolvation	H Donors	H Acceptors	Charge	MolecularWeight	RotatableBonds	SMILES
ZINC00702554	3.99	-0.6	-23.25	1	7	0	448.5	5	Cc1c(sc(n1)N2[C@@H](C(=C(c3ccc(cc3)OC)O)C(=O)C2=O)c4ccccc4)C(=O)C
ZINC00702554	2.96	-0.37	-14.72	0	7	0	448.5	6	Cc1c(sc(n1)N2[C@@H]([C@H](C(=O)C2=O)C(=O)c3ccc(cc3)OC)c4ccccc4)C(=O)C
ZINC08440461	7.51	3.04	-13.76	1	7	0	657.664	8	Cc1ccnc(c1)NC(=O)C2[C@H](C3=C(C[C@H](CC3=O)c4ccccc4)N=C2C)c5ccc(c(c5)COc6c(c(cc(c6F)F)F)F)OC
ZINC08440461	7.69	1.75	-15.43	1	7	0	657.664	8	Cc1ccnc(c1)NC(=O)C2[C@H](C3C(=NC2=C)C[C@H](CC3=O)c4ccccc4)c5ccc(c(c5)COc6c(c(cc(c6F)F)F)F)OC
ZINC08440461	7.51	2.69	-17.78	1	7	0	657.664	8	Cc1ccnc(c1)NC(=O)C2=C(N=C3C[C@H](CC(=O)C3[C@@H]2c4ccc(c(c4)COc5c(c(cc(c5F)F)F)F)OC)c6ccccc6)C
ZINC08440459	3.53	4.57	-36.11	1	9	-1	413.222	5	COc1c(c([n-]n1)C(=O)Nc2nc(cs2)c3ccc(c(c3)Cl)Cl)[N+](=O)[O-]
ZINC08440458	2.57	6.92	-13.26	2	8	0	428.857	5	COc1cc(ccc1N/C=C/2\C(=O)NC(=O)N(C2=O)c3cccc(c3)Cl)SC#N
ZINC08440458	2.57	6.92	-13.38	2	8	0	428.857	5	COc1cc(ccc1N/C=C\2/C(=O)NC(=O)N(C2=O)c3cccc(c3)Cl)SC#N
ZINC08440456	6.56	-3.08	-11.97	0	5	0	455.649	6	CCCn\1c(cs/c1=N\c2ccccc2)c3ccc(cc3)S(=O)(=O)N4CCC(CC4)C
ZINC02062344	5.83	11.82	-10.71	2	4	0	438.98	5	c1ccc(cc1)CC(=O)Nc2c(c3c(s2)CCCCC3)C(=O)Nc4ccccc4Cl
ZINC08440450	6.82	-3.05	-12.34	0	5	0	455.649	6	CCCn\1c(cs/c1=N\c2ccccc2)c3ccc(cc3)S(=O)(=O)N4CCCCCC4
ZINC08440449	5.86	11.71	-12.61	2	4	0	438.98	5	c1ccc(cc1)CC(=O)Nc2c(c3c(s2)CCCCC3)C(=O)Nc4cccc(c4)Cl
ZINC08440448	3.59	-2.2	-74.94	2	9	1	555.501	6	c1ccc2c(c1)nc(n2CC[NH+]3CCOCC3)NC(=O)C45C[C@H]6C[C@@H](C4)CC(C6)(C5)n7cnc(n7)Br
ZINC08440447	5.35	11.27	-13.54	2	4	0	424.953	5	c1ccc(cc1)CC(=O)Nc2c(c3c(s2)CCCC3)C(=O)Nc4cccc(c4)Cl
ZINC08440446	5.26	-3.16	-12.96	0	6	0	445.61	9	CCN(CC)S(=O)(=O)c1ccc(cc1)c2cs/c(=N\c3ccccc3)/n2CCOC
ZINC00837684	3.97	-0.4	-13.55	1	7	0	481.368	7	CCOC(=O)c1c2c(sc1NC(=O)c3ccccc3Br)CN(CC2)C(=O)OCC
ZINC00666145	3.84	0.15	-13.69	1	7	0	436.917	7	CCOC(=O)c1c2c(sc1NC(=O)c3ccccc3Cl)CN(CC2)C(=O)OCC
ZINC02062340	4.99	10.81	-12.07	2	4	0	455.377	5	c1ccc(cc1)CC(=O)Nc2c(c3c(s2)CCC3)C(=O)Nc4ccc(cc4)Br
ZINC08440445	6.55	-2.09	-12.84	0	5	0	463.628	7	CCN(CC)S(=O)(=O)c1ccc(cc1)c2cs/c(=N\c3ccccc3)/n2c4ccccc4
ZINC08440444	9.38	22.85	-10.53	0	5	0	656.661	15	CCCCCCCCCCCCCOC(=O)c1ccc(cc1)N2C(=O)[C@@H]3[C@H](C2=O)C4(c5ccccc5C3c6c4cccc6)Br
ZINC08440443	4.84	-0.97	-16.59	0	6	0	600.247	6	CCOC(=O)COc1c(cc(cc1I)Br)[C@@H]2C3C(=NC4=C2C(=O)CCC4)CCCC3=O
ZINC08440443	5	-2.44	-14.6	0	6	0	600.247	6	CCOC(=O)COc1c(cc(cc1I)Br)[C@H]2C3C(=NC4=CCCC(=O)C24)CCCC3=O
ZINC08440442	9.38	22.75	-8.86	0	5	0	656.661	15	CCCCCCCCCCCCCOC(=O)c1ccc(cc1)N2C(=O)[C@H]3[C@H](C2=O)C4(c5ccccc5C3c6c4cccc6)Br
ZINC08440441	6.21	-3.54	-13.25	0	5	0	461.612	5	c1ccc(cc1)/N=c\2/n(c(cs2)c3ccc(cc3)S(=O)(=O)N4CCCC4)c5ccccc5
ZINC08440440	9.38	22.75	-9.04	0	5	0	656.661	15	CCCCCCCCCCCCCOC(=O)c1ccc(cc1)N2C(=O)[C@@H]3[C@@H](C2=O)C4(c5ccccc5C3c6c4cccc6)Br
ZINC08440439	6.71	-3.2	-12.52	0	5	0	475.639	5	c1ccc(cc1)/N=c\2/n(c(cs2)c3ccc(cc3)S(=O)(=O)N4CCCCC4)c5ccccc5
ZINC08440438	9.38	22.85	-11.08	0	5	0	656.661	15	CCCCCCCCCCCCCOC(=O)c1ccc(cc1)N2C(=O)[C@H]3[C@@H](C2=O)C4(c5ccccc5C3c6c4cccc6)Br
ZINC00666141	5.56	2.24	-11.9	1	5	0	428.271	7	c1ccc(cc1)C(=O)COC(=O)c2ccccc2NC(=O)c3ccc(cc3Cl)Cl
ZINC08440436	5.19	-4.38	-14.2	0	6	0	457.621	8	COCCCn\1c(cs/c1=N\c2ccccc2)c3ccc(cc3)S(=O)(=O)N4CCCC4
ZINC00666139	4.06	-0.88	-15.53	2	5	0	432.217	4	c1cc(oc1)C(=O)Nc2ccc(cc2)NC(=O)c3ccc(cc3)I
ZINC02062329	5.93	1.43	-18.5	0	3	0	445.606	4	CCOc1cccc-2c1N(C(c3c2c(=S)ss3)(C)C)C(=O)Cc4ccc(cc4)F
ZINC08440435	7.47	3.5	-15.41	0	6	0	594.563	5	Cc1ccc2c(c1)N(C(C(=S)C2=C3SC(=C(S3)C(=O)OC)C(=O)OC)(C)C)C(=O)c4ccc(cc4Cl)Cl
ZINC08440434	7.48	-2.77	-11.56	0	5	0	517.72	7	CC1CCN(CC1)S(=O)(=O)c2ccc(cc2)c3cs/c(=N\c4ccccc4)/n3CCc5ccccc5
ZINC08440433	7	-0.36	-6.43	0	2	0	452.453	1	Cc1ccc-2c(c1)N(C(c3c2c(=S)ss3)(C)C)C(=O)c4ccc(cc4Cl)Cl
ZINC00666138	5.46	0.6	-7.09	0	3	0	417.552	2	CC1(c2c(c(=S)ss2)-c3cccc(c3N1C(=O)c4ccc(cc4)F)OC)C
ZINC00666136	5.73	-0.05	-7.85	0	4	0	443.615	4	CCOc1cccc-2c1N(C(c3c2c(=S)ss3)(C)C)C(=O)c4ccc(cc4)OC
ZINC02062325	7.88	0.73	-8.07	0	2	0	473.688	2	Cc1cc-2c(cc1C)N(C(c3c2c(=S)ss3)(C)C)C(=O)c4ccc(cc4)c5ccccc5
ZINC08440432	6.12	-2.72	-12.57	0	5	0	449.601	6	CN(C)S(=O)(=O)c1ccc(cc1)c2cs/c(=N\c3ccccc3)/n2Cc4ccccc4
ZINC08440431	5.85	14.19	-24.19	2	5	0	517.551	5	Cc1ccnc(c1)NC(=O)C2=C(NC3=C([C@@H]2c4ccccc4C(F)(F)F)C(=O)C[C@H](C3)c5ccccc5)C
ZINC08440431	5.99	-0.53	-16.77	1	5	0	517.551	5	Cc1ccnc(c1)NC(=O)C2[C@H](C3C(=NC2=C)C[C@@H](CC3=O)c4ccccc4)c5ccccc5C(F)(F)F
ZINC08440430	7.54	-2.99	-12.75	0	5	0	503.693	6	c1ccc(cc1)Cn\2c(cs/c2=N\c3ccccc3)c4ccc(cc4)S(=O)(=O)N5CCCCCC5
ZINC08440428	7.22	-2.14	-11.73	0	5	0	457.665	10	CCCCCn\1c(cs/c1=N\c2ccccc2)c3ccc(cc3)S(=O)(=O)N(CC)CC
ZINC08440427	5.81	-0.35	-13.35	1	5	0	517.551	5	Cc1ccnc(c1)NC(=O)C2[C@@H](C3=C(C[C@H](CC3=O)c4ccccc4)N=C2C)c5ccccc5C(F)(F)F
ZINC08440427	5.99	-1.55	-17.4	1	5	0	517.551	5	Cc1ccnc(c1)NC(=O)C2[C@@H](C3C(=NC2=C)C[C@H](CC3=O)c4ccccc4)c5ccccc5C(F)(F)F
ZINC08440427	5.81	-0.71	-15.99	1	5	0	517.551	5	Cc1ccnc(c1)NC(=O)C2=C(N=C3C[C@H](CC(=O)C3[C@H]2c4ccccc4C(F)(F)F)c5ccccc5)C
ZINC08440426	7.09	1.9	-21.31	0	8	0	626.803	7	Cc1cc2c(cc1C)N(C(C(=S)C2=C3SC(=C(S3)C(=O)OC)C(=O)OC)(C)C)C(=O)COc4nc5ccccc5s4
ZINC08440425	6.87	-3.59	-12.04	0	5	0	455.649	8	CCCCCn\1c(cs/c1=N\c2ccccc2)c3ccc(cc3)S(=O)(=O)N4CCCC4
ZINC08440424	7.91	4.56	-11.49	0	4	0	551.734	6	Cc1cc(n(n1)CC(=O)N2c3ccc(cc3C(=CC2(C)C)C)C(c4ccccc4)(c5ccccc5)c6ccccc6)C
ZINC02261143	6.47	5.4	-8.38	0	4	0	433.454	4	CC1=CC(N(c2c1cc(cc2)OC(=O)c3ccc(cc3)F)C(=O)c4ccc(cc4)F)(C)C
ZINC08440422	6.73	4.51	-14.92	0	6	0	533.737	7	CCOC(=O)C1=C(SC(=C2c3cc(c(cc3N(C(C2=S)(C)C)C(=O)C(C)C)C)C)S1)C(=O)OCC
ZINC08440420	7.26	3.39	-20.6	0	7	0	628.844	7	Cc1ccc2c(c1)N(C(C(=S)C2=C3SC(=C(S3)C(=O)OC)C(=O)OC)(C)C)C(=O)CSc4nc5ccccc5s4
ZINC08440419	7.58	-3.1	-10.5	0	5	0	495.714	5	CC1CCN(CC1)S(=O)(=O)c2ccc(cc2)c3cs/c(=N\c4ccccc4)/n3C5CCCCC5
ZINC08440418	7.26	3.28	-18.96	0	7	0	628.844	7	Cc1ccc2c(c1)C(=C3SC(=C(S3)C(=O)OC)C(=O)OC)C(=S)C(N2C(=O)CSc4nc5ccccc5s4)(C)C
ZINC08440417	6.43	-2.62	-11.18	0	5	0	441.622	5	CN(C)S(=O)(=O)c1ccc(cc1)c2cs/c(=N\c3ccccc3)/n2C4CCCCC4
ZINC08440415	3.27	9.59	-17.43	2	7	0	484.398	3	Cc1c(cn(n1)C)[C@H]2C3=C(CC(CC3=O)(C)C)NC(=C2C(=O)Nc4ccc(cn4)Br)C
ZINC08440415	3.39	-3.19	-15.69	1	7	0	484.398	3	Cc1c(cn(n1)C)[C@H]2C3C(=O)CC(C=C3N=C(C2C(=O)Nc4ccc(cn4)Br)C)(C)C
ZINC08440414	6.09	-4.15	-43.66	1	7	1	541.763	8	c1ccc(cc1)/N=c\2/n(c(cs2)c3ccc(cc3)S(=O)(=O)N4CCCCCC4)CCC[NH+]5CCOCC5
ZINC08440411	5.55	-4.44	-52.49	1	7	1	527.736	7	CC1CCN(CC1)S(=O)(=O)c2ccc(cc2)c3cs/c(=N\c4ccccc4)/n3CC[NH+]5CCOCC5
ZINC08440410	3.27	10.19	-18.92	2	7	0	484.398	3	Cc1c(cn(n1)C)[C@@H]2C3=C(CC(CC3=O)(C)C)NC(=C2C(=O)Nc4ccc(cn4)Br)C
ZINC08440409	0.88	-0.83	-56.45	2	14	-1	448.397	8	COc1cc(ncn1)[N-]S(=O)(=O)c2ccc(cc2)NC(=O)c3c(c(n[nH]3)OC)[N+](=O)[O-]
ZINC08440407	4.4	-4.21	-44.65	1	7	1	473.644	7	CN(C)S(=O)(=O)c1ccc(cc1)c2cs/c(=N\c3ccccc3)/n2CC[NH+]4CCOCC4

Supplementary Table S2:
Detailed table of XP Docking using Schrodinger's Glide module

ZINC_ID	xLogP	ApolarDesolvation	PolarDesolvation	H Donors	H Acceptors	Charge	MolecularWeight	RotatableBonds	SMILES
ZINC08440404	4.81	9.16	-38	1	7	1	499.682	7	c1ccc(cc1)/[NH+]=c\2/n(c(cs2)c3ccc(cc3)S(=O)(=O)N4CCCC4)CCN5CCOCC5
ZINC08440404	4.81	11.5	-108.6	2	7	2	500.69	7	c1ccc(cc1)/[NH+]=c\2/n(c(cs2)c3ccc(cc3)S(=O)(=O)N4CCCC4)CC[NH+]5CCOCC5
ZINC08440402	6.86	-2.17	-11.27	0	5	0	469.676	5	CC1CCN(CC1)S(=O)(=O)c2ccc(cc2)c3cs/c(=N\c4ccccc4)/n3C(C)(C)C
ZINC08440401	5.31	12.87	-21.46	1	8	0	511.578	8	CCOC(=O)[C@H]1[C@H](CC2=C(C1=O)[C@@H](C(=C(N2)C)C(=O)OCC)c3cnc4ccccc4n3)c5ccccc5
ZINC08440401	5.43	-0.52	-13.31	0	8	0	511.578	8	CCOC(=O)[C@H]1[C@H](C=C2C(C1=O)[C@@H](C(C(=N2)C)C(=O)OCC)c3cnc4ccccc4n3)c5ccccc5
ZINC08440401	5.27	-0.4	-14.35	0	8	0	511.578	8	CCOC(=O)[C@H]1[C@H](CC2=C(C1=O)[C@@H]([C@@H](C(=N2)C)C(=O)OCC)c3cnc4ccccc4n3)c5ccccc5
ZINC08440395	7.13	-2.14	-11.34	0	5	0	469.676	5	CC(C)(C)n\1c(cs/c1=N\c2ccccc2)c3ccc(cc3)S(=O)(=O)N4CCCCCC4
ZINC08440394	5.31	13.77	-23.88	1	8	0	511.578	8	CCOC(=O)[C@@H]1[C@H](CC2=C(C1=O)[C@@H](C(=C(N2)C)C(=O)OCC)c3cnc4ccccc4n3)c5ccccc5
ZINC08440394	5.43	0.22	-12.51	0	8	0	511.578	8	CCOC(=O)[C@@H]1[C@H](C=C2C(C1=O)[C@@H](C(C(=N2)C)C(=O)OCC)c3cnc4ccccc4n3)c5ccccc5
ZINC08440394	5.27	0.34	-14.26	0	8	0	511.578	8	CCOC(=O)[C@@H]1[C@H](CC2=C(C1=O)[C@@H]([C@H](C(=N2)C)C(=O)OCC)c3cnc4ccccc4n3)c5ccccc5
ZINC08440394	5.43	1.58	-26.95	0	8	0	511.578	8	CCOC(=O)[C@@H]1[C@H](C=C2C(C1=O)[C@@H]([C@@H](C(=N2)C)C(=O)OCC)c3cnc4ccccc4n3)c5ccccc5
ZINC08440392	4.35	0.36	-13.71	0	6	0	438.568	10	CCCCOc1ccc(cc1)C(=O)N2CCN(CC2)C(=O)c3ccc(cc3)OCCCC
ZINC00702509	4.32	-3.39	-17.17	2	6	0	490.412	7	c1cc(cnc1)CNC(=O)c2c3c(sc2NC(=O)COc4ccc(cc4Cl)Cl)CCCC3
ZINC08440388	5.11	0.03	-51.93	2	3	1	469.346	4	c1ccc2c(c1)CC[NH+](C2)Cc3ccc(cc3)C(=O)Nc4ccc(cc4)I
ZINC08440387	5.31	13.77	-24.68	1	8	0	511.578	8	CCOC(=O)[C@H]1[C@@H](CC2=C(C1=O)[C@@H](C(=C(N2)C)C(=O)OCC)c3cnc4ccccc4n3)c5ccccc5
ZINC08440387	5.43	0.14	-13.58	0	8	0	511.578	8	CCOC(=O)[C@H]1[C@@H](C=C2C(C1=O)[C@@H](C(C(=N2)C)C(=O)OCC)c3cnc4ccccc4n3)c5ccccc5
ZINC08440387	5.27	0.33	-15.55	0	8	0	511.578	8	CCOC(=O)[C@H]1[C@@H](CC2=C(C1=O)[C@@H]([C@H](C(=N2)C)C(=O)OCC)c3cnc4ccccc4n3)c5ccccc5
ZINC08440387	5.43	1.47	-19.74	0	8	0	511.578	8	CCOC(=O)[C@H]1[C@@H](C=C2C(C1=O)[C@@H]([C@@H](C(=N2)C)C(=O)OCC)c3cnc4ccccc4n3)c5ccccc5
ZINC00702507	3.88	-3.9	-11.42	2	5	0	470.392	5	c1cc(cnc1)CNC(=O)c2c3c(sc2NC(=O)c4ccc(cc4)Br)CCCC3
ZINC05944355	4.78	10.72	-11.79	2	5	0	447.604	6	CC(C)(C)c1ccc(cc1)C(=O)Nc2c(c3c(s2)CCCC3)C(=O)NCc4cccnc4
ZINC02062318	6.6	9.87	-11.39	1	4	0	461.29	3	c1cc2c(cccc2Br)c(c1)c3nc4cc(ccc4o3)NC(=O)c5ccc(cc5)F
ZINC00702501	4.01	-0.79	-18.21	2	6	0	442.512	7	C[C@H](C(=O)Nc1c(c2c(s1)CCCC2)C(=O)NCc3ccco3)Oc4ccccc4F
ZINC08440386	5.31	13.06	-22.34	1	8	0	511.578	8	CCOC(=O)[C@@H]1[C@@H](CC2=C(C1=O)[C@@H](C(=C(N2)C)C(=O)OCC)c3cnc4ccccc4n3)c5ccccc5
ZINC08440386	5.43	-0.34	-12	0	8	0	511.578	8	CCOC(=O)[C@@H]1[C@@H](C=C2C(C1=O)[C@@H](C(C(=N2)C)C(=O)OCC)c3cnc4ccccc4n3)c5ccccc5
ZINC08440386	5.27	-0.12	-13.33	0	8	0	511.578	8	CCOC(=O)[C@@H]1[C@@H](CC2=C(C1=O)[C@@H]([C@H](C(=N2)C)C(=O)OCC)c3cnc4ccccc4n3)c5ccccc5
ZINC08440386	5.43	1.04	-16.73	0	8	0	511.578	8	CCOC(=O)[C@@H]1[C@@H](C=C2C(C1=O)[C@@H]([C@@H](C(=N2)C)C(=O)OCC)c3cnc4ccccc4n3)c5ccccc5
ZINC08440385	6.14	2.8	-13.6	0	8	0	535.666	10	CCN(CC)c1ccc(cc1)/C=c\2/c(=O)n3c(=NC(=C([C@H]3c4ccc(c(c4)OC)OC)C(=O)OCC)C)s2
ZINC02062317	6.14	3.03	-14.1	0	8	0	535.666	10	CCN(CC)c1ccc(cc1)/C=c\2/c(=O)n3c(=NC(=C([C@@H]3c4ccc(c(c4)OC)OC)C(=O)OCC)C)s2
ZINC00702500	4.01	-0.79	-18.21	2	6	0	442.512	7	C[C@@H](C(=O)Nc1c(c2c(s1)CCCC2)C(=O)NCc3ccco3)Oc4ccccc4F
ZINC05946151	5.58	11.79	-12.49	2	6	0	491.657	11	CCCCCCOc1ccc(cc1)C(=O)Nc2c(c3c(s2)CCCC3)C(=O)NCc4cccnc4
ZINC08440383	6.07	0.94	-7.06	1	4	0	420.299	5	c1ccc(cc1)COc2c(cc(cc2Cl)Cl)/C=C(\C#N)/c3[nH]c4ccccc4n3
ZINC08440382	7.38	0.43	-9.56	1	4	0	537.255	5	Cc1ccc(cc1)COc2c(cc(cc2Br)/C=C(\C#N)/c3[nH]c4ccc(cc4n3)C)Br
ZINC08440381	9.12	2.09	-13.57	0	6	0	595.12	8	CCOC(=O)C1=C(N=c2n(c(=O)/c(=C\c3ccc(cc3)OCc4cccc5c4cccc5)/s2)[C@H]1c6ccc(cc6)Cl)C
ZINC08440380	9.12	2.07	-12.69	0	6	0	595.12	8	CCOC(=O)C1=C(N=c2n(c(=O)/c(=C\c3ccc(cc3)OCc4cccc5c4cccc5)/s2)[C@@H]1c6ccc(cc6)Cl)C
ZINC08440379	7.38	2.01	-15.91	0	8	0	549.004	6	CCOC(=O)C1=C(N=c2n(c(=O)/c(=C\c3ccc(o3)c4ccc(cc4)Cl)/s2)[C@H]1c5ccc6c(c5)OCO6)C
ZINC08440378	6.15	-1.2	-14.49	1	5	0	532.413	4	CC1=NC2=C([C@@H](C1C(=O)Nc3ccc(cn3)Br)c4cccc(c4)F)C(=O)C[C@@H](C2)c5ccccc5
ZINC08440378	6.33	0.01	-16.53	1	5	0	532.413	4	C=C1C([C@H]([C@@H]2C(=N1)C[C@H](CC2=O)c3ccccc3)c4cccc(c4)F)C(=O)Nc5ccc(cn5)Br
ZINC02263440	6.42	3.33	-16.9	0	8	0	517.559	8	CCc1cc2c(cc1OC(=O)c3cc(c(c(c3)OC)OC)OC)occ(c2=O)c4nc5ccccc5s4
ZINC08440377	7.38	2.05	-16.16	0	8	0	549.004	6	CCOC(=O)C1=C(N=c2n(c(=O)/c(=C\c3ccc(o3)c4ccc(cc4)Cl)/s2)[C@@H]1c5ccc6c(c5)OCO6)C
ZINC08440376	8.14	2.66	-16.6	0	8	0	633.77	10	CCOC(=O)C1=C(N=c2n(c(=O)/c(=C\c3ccc(c(c3)OC)OCc4cccc5c4cccc5)/s2)[C@H]1c6ccc(cc6)N(C)C)C
ZINC08440375	8.14	2.64	-16.79	0	8	0	633.77	10	CCOC(=O)C1=C(N=c2n(c(=O)/c(=C\c3ccc(c(c3)OC)OCc4cccc5c4cccc5)/s2)[C@@H]1c6ccc(cc6)N(C)C)C
ZINC02148492	5.84	3.65	-15.07	0	6	0	481.298	6	COc1ccc(cc1OC)C(=O)Oc2ccc3c(c2)occ(c3=O)c4ccc(cc4)Br
ZINC08440374	7.48	3.01	-19.87	0	10	0	593.061	8	CCOC(=O)C1=C(N=c2n(c(=O)/c(=C\c3ccc(o3)c4ccc(cc4Cl)[N+](=O)[O-])/s2)[C@H]1c5ccc(cc5)N(C)C)C
ZINC08440373	7.48	3.07	-19.32	0	10	0	593.061	8	CCOC(=O)C1=C(N=c2n(c(=O)/c(=C\c3ccc(o3)c4ccc(cc4Cl)[N+](=O)[O-])/s2)[C@@H]1c5ccc(cc5)N(C)C)C
ZINC08440372	7.27	2.2	-15.98	0	8	0	609.498	8	CCOC(=O)C1=C(N=c2n(c(=O)/c(=C/c3ccc(o3)c4ccc(cc4)Br)/s2)[C@H]1c5ccc(c(c5)OC)OC)C
ZINC00702490	5.87	4.87	-15.06	0	8	0	476.481	10	CCOC(=O)c1c2cc(ccc2oc1c3ccc(cc3)OC)OC(=O)c4ccc(c(c4)OC)OC
ZINC08440371	7.27	2.16	-15.81	0	8	0	609.498	8	CCOC(=O)C1=C(N=c2n(c(=O)/c(=C/c3ccc(o3)c4ccc(cc4)Br)/s2)[C@@H]1c5ccc(c(c5)OC)OC)C
ZINC00659617	5.18	1.73	-13.76	0	7	0	470.572	7	CCOC(=O)C1=C(N=c2n(c(=O)/c(=C/c3cccs3)/s2)[C@H]1c4ccc(c(c4)OC)OC)C
ZINC02235961	5.88	4.04	-17.64	0	8	0	516.546	7	CCc1cc2c(cc1OC(=O)c3ccc(c(c3)OC)OC)oc(c(c2=O)c4ccc5c(c4)OCCCO5)C
ZINC00659622	5.18	1.74	-13.76	0	7	0	470.572	7	CCOC(=O)C1=C(N=c2n(c(=O)/c(=C/c3cccs3)/s2)[C@@H]1c4ccc(c(c4)OC)OC)C
ZINC06245601	4.98	8.84	-19.21	1	6	0	476.023	9	Cc1ccc(cc1)S(=O)(=O)N(CC(=O)NCCSc2ccccn2)c3cccc(c3)Cl
ZINC08440370	6.15	-1.59	-19.45	1	5	0	532.413	4	CC1=NC2=C([C@H](C1C(=O)Nc3ccc(cn3)Br)c4cccc(c4)F)C(=O)C[C@@H](C2)c5ccccc5
ZINC08440370	6.15	-1.46	-18.32	1	5	0	532.413	4	CC1=C([C@@H]([C@H]2C(=N1)C[C@H](CC2=O)c3ccccc3)c4cccc(c4)F)C(=O)Nc5ccc(cn5)Br
ZINC08440370	6.33	-1.5	-17.27	1	5	0	532.413	4	C=C1C([C@@H](C2C(=N1)C[C@H](CC2=O)c3ccccc3)c4cccc(c4)F)C(=O)Nc5ccc(cn5)Br
ZINC08440370	6.33	-0.63	-17.96	1	5	0	532.413	4	C=C1C([C@@H]([C@H]2C(=N1)C[C@H](CC2=O)c3ccccc3)c4cccc(c4)F)C(=O)Nc5ccc(cn5)Br
ZINC08440369	7.04	10.24	-8.15	2	4	0	445.733	5	c1cc(ccc1c2c[nH]nc2c3ccc(cc3O)OCc4ccc(cc4Cl)Cl)Cl
ZINC02062287	5.86	0.54	-16.56	0	3	0	432.353	3	c1cc(cc(c1)Cl)c2ccc(o2)/C=C/3\C(=O)N(C(=S)S3)c4cccc(c4)Cl
ZINC08440368	8.76	3.4	-13.96	0	5	0	643.95	10	c1ccc(c(c1)COc2ccc(cc2)c3coc4cc(cc(c4c3=O)OCc5ccccc5Cl)OCc6ccccc6Cl)Cl
ZINC08440366	5.78	4.13	-16.12	0	5	0	422.864	8	COc1ccc(cc1OC)C(=O)Oc2ccc(cc2)C(=O)/C=C/c3ccccc3Cl
ZINC00659595	4.78	0.04	-20.58	1	7	0	438.867	6	COc1cc(cc(c1OC)OC)C(=O)Nc2ccc(c(c2)c3nc4ccccc4o3)Cl
ZINC08440365	6.2	14.47	-16.85	2	5	0	532.413	4	CC1=C([C@H](C2=C(N1)C[C@@H](CC2=O)c3ccccc3)c4cccc(c4)F)C(=O)Nc5ccc(cn5)Br

Supplementary Table S2:
Detailed table of XP Docking using Schrodinger's Glide module

ZINC_ID	xLogP	ApolarDesolvation	PolarDesolvation	H Donors	H Acceptors	Charge	MolecularWeight	RotatableBonds	SMILES
ZINC08440365	6.33	-0.18	-12.51	1	5	0	532.413	4	C=C1C([C@H]([C@@H]2C(=N1)C[C@@H](CC2=O)c3ccccc3)c4cccc(c4)F)C(=O)Nc5ccc(cn5)Br
ZINC08440365	6.33	-1.43	-13.94	1	5	0	532.413	4	C=C1C([C@H](C2C(=N1)C[C@@H](CC2=O)c3ccccc3)c4cccc(c4)F)C(=O)Nc5ccc(cn5)Br
ZINC08440365	6.15	-0.82	-15.51	1	5	0	532.413	4	CC1=C([C@H]([C@H]2C(=N1)C[C@@H](CC2=O)c3ccccc3)c4cccc(c4)F)C(=O)Nc5ccc(cn5)Br
ZINC08440364	4.95	0	-20.29	1	7	0	432.476	6	Cc1cc2c(cc1C)oc(n2)c3cccc(c3)NC(=O)c4cc(c(c(c4)OC)OC)OC
ZINC06159425	4.48	10.54	-45.68	2	3	1	340.487	6	CC(C)(C)c1ccc(cc1)OC[C@H](C[NH+]2CCc3ccccc3C2)O
ZINC06159425	4.48	8.14	-6.53	1	3	0	339.479	6	CC(C)(C)c1ccc(cc1)OC[C@H](CN2CCc3ccccc3C2)O
ZINC00659594	5.11	1	-12.53	0	3	0	409.61	3	c1cc2cccnc2c(c1)OC(=O)c3cc(ccc3Cl)I
ZINC06159424	4.48	10.29	-46.03	2	3	1	340.487	6	CC(C)(C)c1ccc(cc1)OC[C@@H](C[NH+]2CCc3ccccc3C2)O
ZINC06159424	4.48	7.83	-6.51	1	3	0	339.479	6	CC(C)(C)c1ccc(cc1)OC[C@@H](CN2CCc3ccccc3C2)O
ZINC02062267	5.99	-1.5	-17.35	1	5	0	457.711	4	COc1ccc(cc1)C(=O)Nc2ccc3c(c2)nc(o3)c4cc(ccc4Cl)Br
ZINC02062266	7.21	-1.82	-17.07	1	4	0	496.575	3	c1cc(c(cc1C(=O)Nc2ccc3c(c2)nc(o3)c4cc(ccc4Cl)Br)Cl)Cl
ZINC08440363	6.35	-1.22	-16.52	1	4	0	441.712	3	Cc1cccc(c1)C(=O)Nc2ccc3c(c2)nc(o3)c4cc(ccc4Cl)Br
ZINC05917882	6.19	-1.64	-20.3	2	5	0	522.468	4	CC1(CC2=C([C@H](c3c(=O)[nH]c(nc3N2)SCc4ccccc4)c5ccc(cc5)Br)C(=O)C1)C
ZINC02062265	6.31	-2.08	-15.18	1	5	0	471.738	5	CCOc1ccc(cc1Br)C(=O)Nc2ccc3c(c2)nc(o3)c4ccccc4Cl
ZINC00659590	5.8	-1.29	-16.46	1	5	0	455.283	5	CCOc1ccc(cc1Br)C(=O)Nc2ccc3c(c2)nc(o3)c4ccccc4F
ZINC05917844	6.19	-1.64	-20.11	2	5	0	522.468	4	CC1(CC2=C([C@@H](c3c(=O)[nH]c(nc3N2)SCc4ccccc4)c5ccc(cc5)Br)C(=O)C1)C
ZINC08440361	6.15	-1.65	-18.96	1	5	0	532.413	4	CC1=NC2=C([C@H](C1C(=O)Nc3ccc(cn3)Br)c4cccc(c4)F)C(=O)C[C@H](C2)c5ccccc5
ZINC08440361	6.33	-1.45	-17.46	1	5	0	532.413	4	C=C1C([C@@H](C2C(=N1)C[C@@H](CC2=O)c3ccccc3)c4cccc(c4)F)C(=O)Nc5ccc(cn5)Br
ZINC08440361	6.33	-0.14	-33.22	1	5	0	532.413	4	C=C1C([C@@H]([C@H]2C(=N1)C[C@@H](CC2=O)c3ccccc3)c4cccc(c4)F)C(=O)Nc5ccc(cn5)Br
ZINC02225650	6.17	8.32	-16.64	2	4	0	415.301	3	c1ccc2c(c1)nc(s2)c3cc(ccc3O)NC(=O)c4cc(ccc4Cl)Cl
ZINC00702484	3.81	8.6	-35.77	2	7	0	441.553	5	CCSc1[nH]c2c(c(=O)n1)[C@@H](C3=C(N2)CC(CC3=O)(C)C)c4ccc(c(c4)OC)OC
ZINC00702484	4.26	7.23	-63.43	1	7	-1	440.545	5	CCSc1nc2c(c(n1)[O-])[C@@H](C3=C(N2)CC(CC3=O)(C)C)c4ccc(c(c4)OC)OC
ZINC02062260	7.17	-0.72	-20.54	1	5	0	436.511	7	CCCCOc1ccc(cc1)C(=O)Nc2ccc3c(c2)nc(o3)c4cccc5c4cccc5
ZINC02062259	7.49	-2.46	-11.83	1	4	0	569.196	3	c1ccc2c(c1)cccc2c3nc4cc(ccc4o3)NC(=O)c5cc(ccc5I)Br
ZINC00702480	5.07	-1.36	-17.99	2	5	0	436.364	2	CC1(CC2=C([C@H](c3c(=O)[nH]c(nc3N2)SC)c4ccc(c(c4)Cl)Cl)C(=O)C1)C
ZINC08440359	9.74	1.85	-13.06	1	3	0	535.046	5	c1ccc(cc1)c2c(nc([nH]2)c3c4ccccc4c(c5c3cccc5)Cl)c6ccc(cc6)C(=O)c7ccccc7
ZINC08440359	9.74	1.94	-34.41	2	3	1	536.054	5	c1ccc(cc1)c2c([nH+]c([nH]2)c3c4ccccc4c(c5c3cccc5)Cl)c6ccc(cc6)C(=O)c7ccccc7
ZINC08740856	7.82	15.26	-8.24	1	3	0	422.915	5	c1ccc(cc1)c2c(nc([nH]2)c3ccc(cc3)Cl)c4ccc(cc4)Oc5ccccc5
ZINC08740856	7.82	15.64	-32.91	2	3	1	423.923	5	c1ccc(cc1)c2c([nH]c([nH+]2)c3ccc(cc3)Cl)c4ccc(cc4)Oc5ccccc5
ZINC02062255	6.43	4.86	-9.06	0	6	0	448.563	9	CCOC(=O)C1=C(N(C(=C(C1c2ccc(cc2)N(C)C)C(=O)OCC)C)c3ccccc3)C
ZINC00702481	5.07	-1.36	-17.98	2	5	0	436.364	2	CC1(CC2=C([C@@H](c3c(=O)[nH]c(nc3N2)SC)c4ccc(c(c4)Cl)Cl)C(=O)C1)C
ZINC08440357	5.01	0.65	-14.68	0	6	0	450.56	11	COc1ccccc1OCCn2c3ccccc3nc2SCCOc4ccccc4OC
ZINC08440356	7.7	2.41	-13.97	0	6	0	610.961	6	CCOC(=O)C1=C(N=c2n(c(=O)/c(=C/c3cc(n(c3C)c4ccc(cc4)Br)C)/s2)[C@H]1c5ccccc5Cl)C
ZINC08440355	7.32	18.41	-19.74	1	7	0	562.666	8	CCOC(=O)[C@H]1[C@H](CC2=C(C1=O)[C@@H](C(=C(N2)C)C(=O)OCC)c3ccc4c(c3)c5ccccc5n4C)c6ccccc6
ZINC08440354	7.7	2.41	-14.77	0	6	0	610.961	6	CCOC(=O)C1=C(N=c2n(c(=O)/c(=C/c3cc(n(c3C)c4ccc(cc4)Br)C)/s2)[C@@H]1c5ccccc5Cl)C
ZINC08440353	9.5	2.6	-11.47	0	6	0	735.103	8	CCOC(=O)C1=C(N=c2n(c(=O)/c(=C/c3cc(n(c3c4ccccc4)c5ccc(cc5)Br)c6ccccc6)/s2)[C@H]1c7ccccc7Cl)C
ZINC00702477	4.64	10.83	-33.95	2	6	0	438.597	5	CCN(CC)c1ccc(cc1)[C@@H]2c3c([nH]c(nc3=O)SC)NC4=C2C(=O)CC(C4)(C)C
ZINC00702477	5.09	9.56	-63.99	2	6	0	438.597	5	CC[NH+](CC)c1ccc(cc1)[C@@H]2c3c(nc(nc3[O-])SC)NC4=C2C(=O)CC(C4)(C)C
ZINC00702477	5.09	9.45	-64.49	1	6	-1	437.589	5	CCN(CC)c1ccc(cc1)[C@@H]2c3c(nc(nc3[O-])SC)NC4=C2C(=O)CC(C4)(C)C
ZINC00702477	4.64	10.94	-64.89	3	6	0	439.605	5	CC[NH+](CC)c1ccc(cc1)[C@@H]2c3c([nH]c(nc3=O)SC)NC4=C2C(=O)CC(C4)(C)C
ZINC00702474	5.16	11.77	-33.77	2	6	0	459.571	6	CCSc1[nH]c2c(c(=O)n1)[C@@H](C3=C(N2)CCCC3=O)c4ccc(cc4)OCc5ccccc5
ZINC00702474	5.62	10.39	-63.18	1	6	-1	458.563	6	CCSc1nc2c(c(n1)[O-])[C@@H](C3=C(N2)CCCC3=O)c4ccc(cc4)OCc5ccccc5
ZINC08440352	9.5	2.64	-14.07	0	6	0	735.103	8	CCOC(=O)C1=C(N=c2n(c(=O)/c(=C/c3cc(n(c3c4ccccc4)c5ccc(cc5)Br)c6ccccc6)/s2)[C@@H]1c7ccccc7Cl)C
ZINC00702475	5.16	11.76	-33.85	2	6	0	459.571	6	CCSc1[nH]c2c(c(=O)n1)[C@H](C3=C(N2)CCCC3=O)c4ccc(cc4)OCc5ccccc5
ZINC00702475	5.62	10.38	-63.38	1	6	-1	458.563	6	CCSc1nc2c(c(n1)[O-])[C@H](C3=C(N2)CCCC3=O)c4ccc(cc4)OCc5ccccc5
ZINC00659579	6.46	1.22	-14.43	0	6	0	507.793	5	CCOC(=O)C1=C(N=c2n(c(=O)/c(=C/c3ccc(o3)Br)/s2)[C@H]1c4ccccc4Cl)C
ZINC00659581	6.46	1.23	-14.34	0	6	0	507.793	5	CCOC(=O)C1=C(N=c2n(c(=O)/c(=C/c3ccc(o3)Br)/s2)[C@@H]1c4ccccc4Cl)C
ZINC00823055	5.74	2.32	-13.78	0	6	0	442.924	5	CCOC(=O)C1=C(N=c2n(c(=O)/c(=C/c3ccc(o3)C)/s2)[C@H]1c4ccccc4Cl)C
ZINC08440351	6.43	-0.72	-22.68	2	5	0	492.044	4	Cc1cccc(c1)[C@H]2c3c(=O)[nH]c(nc3NC4=C2C(=O)CC(C4)(C)C)SCc5ccccc5Cl
ZINC00823057	5.74	2.31	-14.58	0	6	0	442.924	5	CCOC(=O)C1=C(N=c2n(c(=O)/c(=C/c3ccc(o3)C)/s2)[C@@H]1c4ccccc4Cl)C
ZINC02062206	7.38	2.8	-26.97	0	10	0	580.018	8	CCOC(=O)C1=C(N=c2n(c(=O)/c(=C/c3ccc(o3)c4ccc(cc4[N+](=O)[O-])OC)/s2)[C@H]1c5ccccc5Cl)C
ZINC02062208	7.38	2.84	-25.75	0	10	0	580.018	8	CCOC(=O)C1=C(N=c2n(c(=O)/c(=C/c3ccc(o3)c4ccc(cc4[N+](=O)[O-])OC)/s2)[C@@H]1c5ccccc5Cl)C
ZINC08440350	6.43	-0.72	-23.03	2	5	0	492.044	4	Cc1cccc(c1)[C@@H]2c3c(=O)[nH]c(nc3NC4=C2C(=O)CC(C4)(C)C)SCc5ccccc5Cl
ZINC08440349	7.32	19.33	-23.3	1	7	0	562.666	8	CCOC(=O)[C@@H]1[C@H](CC2=C(C1=O)[C@@H](C(=C(N2)C)C(=O)OCC)c3ccc4c(c3)c5ccccc5n4C)c6ccccc6
ZINC02062198	8.25	1.63	-16.07	0	6	0	583.891	6	CCOC(=O)C1=C(N=c2n(c(=O)/c(=C/c3ccc(o3)c4ccc(cc4)Br)/s2)[C@H]1c5ccccc5Cl)C
ZINC02062200	8.25	1.68	-15.91	0	6	0	583.891	6	CCOC(=O)C1=C(N=c2n(c(=O)/c(=C/c3ccc(o3)c4ccc(cc4)Br)/s2)[C@@H]1c5ccccc5Cl)C
ZINC02062194	7.26	14.18	-5.87	0	2	0	424.371	2	C[C@@]1(CC(N(c2c1cccc2)C(=O)c3ccc(cc3Cl)Cl)(C)C)c4ccccc4
ZINC02062195	7.26	14.16	-5.78	0	2	0	424.371	2	C[C@]1(CC(N(c2c1cccc2)C(=O)c3ccc(cc3Cl)Cl)(C)C)c4ccccc4
ZINC05917888	7.66	-0.85	-24.06	2	6	0	584.141	7	CC1(CC2=C([C@H](c3c(=O)[nH]c(nc3N2)SCc4ccccc4Cl)c5ccc(cc5)OCc6ccccc6)C(=O)C1)C
ZINC02062192	6.97	-1.55	-12.74	1	5	0	506.183	5	CCOc1ccc(cc1Br)C(=O)Nc2ccc3c(c2)nc(o3)c4ccc(cc4Cl)Cl
ZINC05917848	7.66	-0.85	-24.44	2	6	0	584.141	7	CC1(CC2=C([C@@H](c3c(=O)[nH]c(nc3N2)SCc4ccccc4Cl)c5ccc(cc5)OCc6ccccc6)C(=O)C1)C

Supplementary Table S2:

Detailed table of XP Docking using Schrodinger's Glide module

ZINC_ID	xLogP	ApolarDesolvation	PolarDesolvation	H Donors	H Acceptors	Charge	MolecularWeight	RotatableBonds	SMILES
ZINC08440347	7.32	19.34	-23.02	1	7	0	562.666	8	CCOC(=O)[C@H]1[C@@H](CC2=C(C1=O)[C@@H](C(=C(N2)C)C(=O)OCC)c3ccc4c(c3)c5ccccc5n4C)c6ccccc6
ZINC00659570	5.17	1.16	-12.95	0	3	0	423.637	3	Cc1ccc2cccc(c2n1)OC(=O)c3cc(ccc3Cl)I
ZINC02062185	6.65	-1.97	-13.13	1	4	0	488.712	3	Cc1ccc2c(c1)oc(n2)c3ccc(cc3)NC(=O)c4cc(ccc4Cl)I
ZINC00702472	5.92	0.14	-23.2	2	5	0	475.589	4	Cc1cccc(c1)[C@H]2c3c(=O)[nH]c(nc3NC4=C2C(=O)CC(C4)(C)C)SCc5ccccc5F
ZINC08440343	4.58	9.41	-7.58	3	4	0	256.419	10	CCCCCCCCCCSc1nc([nH]n1)N
ZINC00702473	5.92	0.13	-23.58	2	5	0	475.589	4	Cc1cccc(c1)[C@@H]2c3c(=O)[nH]c(nc3NC4=C2C(=O)CC(C4)(C)C)SCc5ccccc5F
ZINC08440341	6.32	3.83	-15.02	0	8	0	595.479	5	CCOC(=O)C1=C(C2[C@H]3[C@H](C1([C@@H]4[C@@H]2C(=O)N(C4=O)c5ccc(c(c5)Cl)C)C)C(=O)N(C3=O)c6ccc(c(c6)Cl)C)C
ZINC08440340	6.32	15.16	-15.42	0	8	0	595.479	5	CCOC(=O)C1=C(C2[C@@H]3[C@@H](C1([C@H]4[C@@H]2C(=O)N(C4=O)c5ccc(c(c5)Cl)C)C)C(=O)N(C3=O)c6ccc(c(c6)Cl)C)C
ZINC08440339	6.32	4.03	-15.51	0	8	0	595.479	5	CCOC(=O)C1=C(C2[C@H]3[C@@H](C1([C@@H]4[C@@H]2C(=O)N(C4=O)c5ccc(c(c5)Cl)C)C)C(=O)N(C3=O)c6ccc(c(c6)Cl)C)C
ZINC08440338	6.32	3.92	-11.57	0	8	0	595.479	5	CCOC(=O)C1=C(C2[C@@H]3[C@@H](C1([C@@H]4[C@@H]2C(=O)N(C4=O)c5ccc(c(c5)Cl)C)C)C(=O)N(C3=O)c6ccc(c(c6)Cl)C)C
ZINC08440337	7.32	18.62	-21.74	1	7	0	562.666	8	CCOC(=O)[C@@H]1[C@@H](CC2=C(C1=O)[C@@H](C(=C(N2)C)C(=O)OCC)c3ccc4c(c3)c5ccccc5n4C)c6ccccc6
ZINC00702470	5.31	0.12	-22.56	2	7	0	521.614	6	CC1(CC2=C([C@H](c3c(=O)[nH]c(nc3N2)SCc4ccccc4F)c5cccc(c5OC)OC)C(=O)C1)C
ZINC08440336	7.23	16.82	-14.24	0	8	0	582.697	7	CCOC(=O)C1=C(C2[C@@H]3[C@@H](C1([C@H]4[C@@H]2C(=O)N(C4=O)c5ccc(cc5)C(C)C)C)C(=O)N(C3=O)c6ccc(cc6)C(C)C)C
ZINC08440335	7.23	5.14	-17.98	0	8	0	582.697	7	CCOC(=O)C1=C(C2[C@H]3[C@@H](C1([C@@H]4[C@@H]2C(=O)N(C4=O)c5ccc(cc5)C(C)C)C)C(=O)N(C3=O)c6ccc(cc6)C(C)C)C
ZINC00702471	5.31	0.13	-23.1	2	7	0	521.614	6	CC1(CC2=C([C@@H](c3c(=O)[nH]c(nc3N2)SCc4ccccc4F)c5cccc(c5OC)OC)C(=O)C1)C
ZINC02062178	5.48	11.96	-26.37	1	5	0	371.525	11	CCCCCCCCCCC(=O)Nc1c(n(n(c1=O)c2ccccc2)C)C
ZINC08440334	5.55	5.99	-17.47	0	10	0	699.8	9	Cc1cc2c(cc1C)N(C(C3=C2C4(C(=C(S3)C(=O)OC)C(=O)OC)SC(=C(S4)C(=O)OC)C(=O)OC)(C)C)C(=O)c5cccc(c5)F
ZINC05917880	6.66	-1.17	-17.87	2	5	0	512.462	4	CC1(CC2=C([C@H](c3c(=O)[nH]c(nc3N2)SCc4ccccc4)c5ccc(c(c5)Cl)Cl)C(=O)C1)C
ZINC05917841	6.66	-1.16	-17.93	2	5	0	512.462	4	CC1(CC2=C([C@@H](c3c(=O)[nH]c(nc3N2)SCc4ccccc4)c5ccc(c(c5)Cl)Cl)C(=O)C1)C
ZINC00702469	3.59	8.51	-14.56	3	7	0	426.542	4	CC1(CC2=C([C@@H](c3c(nc(nc3N2)SC)N)c4ccc(c(c4)OC)OC)C(=O)C1)C
ZINC00659550	4.35	1.81	-17.87	0	7	0	445.537	9	COc1cc(ccc1OC(=O)c2cc(c(c(c2)OC)OC)OC)C(=S)N3CCCCC3
ZINC00702465	4.49	-0.63	-27.09	2	7	0	493.56	6	COc1ccc(cc1OC)[C@@H]2c3c(=O)[nH]c(nc3NC4=C2C(=O)CCC4)SCc5ccccc5F
ZINC08440332	4.17	2.67	-15.1	0	6	0	423.472	3	Cc1ccc2c(c1)/C(=C/c3nc4ccccc4c(=O)n3c5ccc(cc5)OC)/C(=O)N2C
ZINC08440331	4.13	2.49	-15.12	0	6	0	423.472	4	CCN1c2ccccc2/C(=C/c3nc4ccccc4c(=O)n3c5ccc(cc5)OC)/C1=O
ZINC08440330	5.74	2.8	-16.67	0	5	0	435.527	4	Cc1cccc(c1)n2c(nc3ccccc3c2=O)/C=C/c4c(n(c5c4cc(cc5)OC)C)C
ZINC08440329	6.62	3.18	-60.06	0	4	-1	430.789	4	Cc1ccc2c(c1)c(cc(n2)c3ccc(o3)c4ccc(c(c4)C(F)(F)F)Cl)C(=O)[O-]
ZINC08440328	6.44	3.19	-12.74	0	4	0	453.545	5	c1ccc(cc1)Cn2cc(c3c2cccc3)/C=C\c4nc5ccccc5c(=O)n4c6ccccc6
ZINC02840318	5.79	-3.44	-16.49	1	5	0	450.372	6	c1ccc2c(c1)nc(o2)SCC(=O)Nc3ncc(s3)Cc4ccc(cc4Cl)Cl
ZINC08974968	6.92	11.32	-15.63	2	6	0	445.544	8	Cc1ccc2c(c1)sc(n2)NC(=O)[C@H](Cc3ccccc3)NC(=O)OCc4ccccc4
ZINC08440326	5.84	-0.03	-12.01	1	2	0	562.112	5	CC1([C@@]2(CC[C@]1([C@@H]2Br)C(=O)Nc3ccc(cc3)SC(F)F)C(Br)Br)C
ZINC08440325	5.13	1.2	-10.63	0	7	0	496.398	11	CCCOc1cc(c(cc1OC)[C@@H]2C(C(=NC(=C2C(=O)OCC)C)C)C(=O)OCC)Br
ZINC08440325	5.13	1.17	-10.73	0	7	0	496.398	11	CCCOc1cc(c(cc1OC)[C@H]2C(C(=NC(=C2C(=O)OCC)C)C)C(=O)OCC)Br
ZINC08440325	5.31	-0.04	-8.94	0	7	0	496.398	11	CCCOc1cc(c(cc1OC)[C@H]2C(C(=NC(=C)C2C(=O)OCC)C)C(=O)OCC)Br
ZINC08974967	6.92	11.32	-15.64	2	6	0	445.544	8	Cc1ccc2c(c1)sc(n2)NC(=O)[C@@H](Cc3ccccc3)NC(=O)OCc4ccccc4
ZINC08440323	5.84	-0.22	-8.16	1	2	0	562.112	5	CC1([C@]2(CC[C@@]1([C@@H]2Br)C(=O)Nc3ccc(cc3)SC(F)F)C(Br)Br)C
ZINC08440321	6.19	16.14	-37.99	2	6	1	572.925	6	Cc1ccc(cc1)C2=NN([C@H](C2)c3ccc(cc3)Cl)C(=[NH2+])S[C@@H]4CC(=O)N(C4=O)c5ccc(c(c5)Cl)Cl
ZINC08440320	6.19	16.12	-40.08	2	6	1	572.925	6	Cc1ccc(cc1)C2=NN([C@@H](C2)c3ccc(cc3)Cl)C(=[NH2+])S[C@@H]4CC(=O)N(C4=O)c5ccc(c(c5)Cl)Cl
ZINC08440319	5.41	-1.37	-7.41	1	3	0	549.916	3	CC1([C@@]2(CC[C@]1([C@@H]2Br)C(=O)Nc3c(cc(cn3)Cl)Cl)C(Br)Br)C
ZINC08440318	5.69	15.79	-36.69	2	6	1	582.931	6	Cc1ccc(cc1)C2=NN([C@H](C2)c3ccc(cc3)Cl)C(=[NH2+])S[C@@H]4CC(=O)N(C4=O)c5cccc(c5)Br
ZINC08440317	5.69	15.77	-38.81	2	6	1	582.931	6	Cc1ccc(cc1)C2=NN([C@@H](C2)c3ccc(cc3)Cl)C(=[NH2+])S[C@@H]4CC(=O)N(C4=O)c5cccc(c5)Br
ZINC08440316	5.62	13.36	-18.84	2	7	0	574.475	6	CC1=C([C@H](C2=C(N1)C[C@H](CC2=O)c3ccccc3)c4ccc(cc4OC)OC)C(=O)Nc5ccc(cn5)Br
ZINC08440316	5.76	-0.67	-12.11	1	7	0	574.475	6	COc1ccc(c(c1)OC)[C@H]2[C@@H]3C(=NC(=C)C2C(=O)Nc4ccc(cn4)Br)C[C@H](CC3=O)c5ccccc5
ZINC08440315	5.41	-1.38	-6.05	1	3	0	549.916	3	CC1([C@]2(CC[C@@]1([C@@H]2Br)C(=O)Nc3c(cc(cn3)Cl)Cl)C(Br)Br)C
ZINC08440314	6.19	16.19	-37.12	2	6	1	572.925	6	Cc1ccc(cc1)C2=NN([C@H](C2)c3ccc(cc3)Cl)C(=[NH2+])S[C@@H]4CC(=O)N(C4=O)c5cc(cc(c5)Cl)Cl
ZINC08440313	6.19	16.17	-39.01	2	6	1	572.925	6	Cc1ccc(cc1)C2=NN([C@@H](C2)c3ccc(cc3)Cl)C(=[NH2+])S[C@@H]4CC(=O)N(C4=O)c5cc(cc(c5)Cl)Cl
ZINC08440312	5.14	-2.68	-8.29	1	3	0	541.147	3	CC1([C@@]2(CC[C@]1([C@@H]2Br)C(=O)Nc3nc4c(s3)CCCC4)C(Br)Br)C
ZINC08440311	5.71	15.8	-37.23	2	6	1	582.931	6	Cc1ccc(cc1)C2=NN([C@H](C2)c3ccc(cc3)Cl)C(=[NH2+])S[C@@H]4CC(=O)N(C4=O)c5ccc(cc5)Br
ZINC08440310	5.71	15.78	-39	2	6	1	582.931	6	Cc1ccc(cc1)C2=NN([C@@H](C2)c3ccc(cc3)Cl)C(=[NH2+])S[C@@H]4CC(=O)N(C4=O)c5ccc(cc5)Br
ZINC08440308	4.94	-1.62	-10.4	1	3	0	524.091	5	CC1([C@@]2(CC[C@]1([C@@H]2Br)C(=O)NCc3ccc(cc3)OC)C(Br)Br)C
ZINC08440307	5.07	15.22	-39.51	2	6	1	522.025	6	Cc1ccc(cc1)C2=NN([C@@H](C2)c3ccc(cc3)Cl)C(=[NH2+])S[C@@H]4CC(=O)N(C4=O)c5ccc(cc5)F
ZINC08440307	5.07	15.31	-14.51	1	6	0	521.017	6	[H]/N=C(/N1[C@@H](CC(=N1)c2ccc(cc2)C)c3ccc(cc3)Cl)\S[C@@H]4CC(=O)N(C4=O)c5ccc(cc5)F
ZINC08440305	5.62	13.35	-18.61	2	7	0	574.475	6	CC1=C([C@@H](C2=C(N1)C[C@H](CC2=O)c3ccccc3)c4ccc(cc4OC)OC)C(=O)Nc5ccc(cn5)Br
ZINC08440305	5.76	-0.63	-13.11	1	7	0	574.475	6	COc1ccc(c(c1)OC)[C@@H]2[C@H]3C(=NC(=C)C2C(=O)Nc4ccc(cn4)Br)C[C@H](CC3=O)c5ccccc5
ZINC08440305	5.76	-1.87	-13.15	1	7	0	574.475	6	COc1ccc(c(c1)OC)[C@@H]2C3C(=NC(=C)C2C(=O)Nc4ccc(cn4)Br)C[C@H](CC3=O)c5ccccc5
ZINC02666668	4.94	-1.82	-6.41	1	3	0	524.091	5	CC1([C@]2(CC[C@@]1([C@@H]2Br)C(=O)NCc3ccc(cc3)OC)C(Br)Br)C
ZINC08440303	5.56	15.69	-36.7	2	6	1	538.48	6	Cc1ccc(cc1)C2=NN([C@H](C2)c3ccc(cc3)Cl)C(=[NH2+])S[C@@H]4CC(=O)N(C4=O)c5cccc(c5)Cl
ZINC08440301	5.56	15.67	-38.85	2	6	1	538.48	6	Cc1ccc(cc1)C2=NN([C@@H](C2)c3ccc(cc3)Cl)C(=[NH2+])S[C@@H]4CC(=O)N(C4=O)c5cccc(c5)Cl
ZINC08440299	6.28	17.32	-34.83	2	7	1	555.724	12	CCCCCCOc1ccc(cc1)N2C(=O)C[C@@H](C2=O)SC(=[NH2+])N3[C@@H](CC(=N3)c4ccccc4)c5ccccc5
ZINC08440297	5.69	12.17	-7.85	0	2	0	540.178	2	C[C@@]12C[C@@H](CC(C1)(C)C)N(C2)C(=O)[C@@]34CC[C@@]([C@@H]3Br)(C4(C)C)C(Br)Br
ZINC08440296	5.62	13.35	-18.92	2	7	0	574.475	6	CC1=C([C@H](C2=C(N1)C[C@@H](CC2=O)c3ccccc3)c4ccc(cc4OC)OC)C(=O)Nc5ccc(cn5)Br
ZINC08440296	5.76	-0.67	-11.98	1	7	0	574.475	6	COc1ccc(c(c1)OC)[C@H]2[C@@H]3C(=NC(=C)C2C(=O)Nc4ccc(cn4)Br)C[C@@H](CC3=O)c5ccccc5

Supplementary Table S2:
Detailed table of XP Docking using Schrodinger's Glide module

ZINC_ID	xLogP	ApolarDesolvation	PolarDesolvation	H Donors	H Acceptors	Charge	MolecularWeight	RotatableBonds	SMILES
ZINC08440295	5.06	14.91	-35.09	2	6	1	524.453	6	c1ccc(cc1)[C@H]2CC(=NN2C(=[NH2+])S[C@@H]3CC(=O)N(C3=O)c4ccc(c(c4)Cl)Cl)c5ccccc5
ZINC08440294	5.06	14.91	-36.77	2	6	1	524.453	6	c1ccc(cc1)[C@@H]2CC(=NN2C(=[NH2+])S[C@@H]3CC(=O)N(C3=O)c4ccc(c(c4)Cl)Cl)c5ccccc5
ZINC08440293	5.69	11.98	-8.54	0	2	0	540.178	2	C[C@]12C[C@H](CC(C1)(C)C)N(C2)C(=O)[C@@]34CC[C@@]([C@@H]3Br)(C4(C)C)C(Br)Br
ZINC02666666	4.36	-1.91	-4.37	1	2	0	446.021	4	CCCNC(=O)[C@]12CC[C@]([C@@H]1Br)(C2(C)C)C(Br)Br
ZINC08440288	5.06	14.95	-34.21	2	6	1	524.453	6	c1ccc(cc1)[C@H]2CC(=NN2C(=[NH2+])S[C@@H]3CC(=O)N(C3=O)c4cc(cc(c4)Cl)Cl)c5ccccc5
ZINC08440287	5.06	14.95	-35.74	2	6	1	524.453	6	c1ccc(cc1)[C@@H]2CC(=NN2C(=[NH2+])S[C@@H]3CC(=O)N(C3=O)c4cc(cc(c4)Cl)Cl)c5ccccc5
ZINC08440286	5.58	-1.81	-11.91	1	7	0	574.475	6	CC1=NC2=C([C@H](C1C(=O)Nc3ccc(cn3)Br)c4ccc(cc4OC)OC)C(=O)C[C@H](C2)c5ccccc5
ZINC08440286	5.76	-0.33	-19.39	1	7	0	574.475	6	COc1ccc(c(c1)OC)[C@@H]2[C@H]3C(=NC(=C)C2C(=O)Nc4ccc(cn4)Br)C[C@@H](CC3=O)c5ccccc5
ZINC08440286	5.76	-1.58	-13.53	1	7	0	574.475	6	COc1ccc(c(c1)OC)[C@@H]2C3C(=NC(=C)C2C(=O)Nc4ccc(cn4)Br)C[C@@H](CC3=O)c5ccccc5
ZINC00659536	4.77	-1.09	-9.04	1	5	0	496.304	5	c1ccc(cc1)COc2ccccc2/C=C\3/C(=O)NN(C3=O)c4ccc(cc4)I
ZINC08440285	4.36	-1.7	-8.53	1	2	0	446.021	4	CCCNC(=O)[C@@]12CC[C@@]([C@@H]1Br)(C2(C)C)C(Br)Br
ZINC00659531	6.43	3.34	-18.09	0	5	0	550.433	4	CCOc1cc(cc(c1OC)I)C2C3=C(CC(CC3=O)(C)C)OC4=C2C(=O)CC(C4)(C)C
ZINC02062159	8.18	0.58	-8.58	1	3	0	528.479	4	CC1(CC2=C([C@H](Nc3c2c4ccccc4cc3)c5ccc(cc5)OCc6ccc(cc6Cl)Cl)C(=O)C1)C
ZINC02062160	8.18	0.57	-8.93	1	3	0	528.479	4	CC1(CC2=C([C@@H](Nc3c2c4ccccc4cc3)c5ccc(cc5)OCc6ccc(cc6Cl)Cl)C(=O)C1)C
ZINC08440284	5.24	0.85	-27.41	2	5	0	438.474	9	CCCCOc1ccc(cc1)C(NC(=O)c2ccccc2F)NC(=O)c3ccccc3F
ZINC08440282	5.3	-0.58	-10.89	1	5	0	517.15	4	CCOc1cc(cc(c1O)I)/C=C/2\C(=NN(C2=O)c3ccc(c(c3)Cl)Cl)C
ZINC05946144	6.1	-1.6	-12.79	3	4	0	478.795	12	CCCCCCC(=O)N[C@H](C(Cl)(Cl)Cl)NC(=S)Nc1cccc(c1)C(F)(F)F
ZINC05946142	6.1	10.61	-16.98	3	4	0	478.795	12	CCCCCCC(=O)N[C@@H](C(Cl)(Cl)Cl)NC(=S)Nc1cccc(c1)C(F)(F)F
ZINC00659496	4.58	-0.23	-17.88	1	6	0	518.742	3	Cc1c(c(n(n1)c2ccc(cc2)I)O)/C=C\3/C(=NN(C3=O)c4cccc(c4)Cl)C
ZINC08440274	4.56	9.24	-18.43	2	8	0	511.003	7	Cc1ccccc1NS(=O)(=O)c2ccc(c(c2)OC)N/C=C/3\C(=NN(C3=O)c4ccc(cc4)Cl)C
ZINC08440274	4.56	9.21	-22.37	2	8	0	511.003	7	Cc1ccccc1NS(=O)(=O)c2ccc(c(c2)OC)N/C=C\3/C(=NN(C3=O)c4ccc(cc4)Cl)C
ZINC08440274	4.56	9.27	-53.12	1	8	-1	509.995	7	Cc1ccccc1[N-]S(=O)(=O)c2ccc(c(c2)OC)N/C=C\3/C(=NN(C3=O)c4ccc(cc4)Cl)C
ZINC08440274	4.56	9.29	-49.42	1	8	-1	509.995	7	Cc1ccccc1[N-]S(=O)(=O)c2ccc(c(c2)OC)N/C=C/3\C(=NN(C3=O)c4ccc(cc4)Cl)C
ZINC08440273	4.61	9.17	-18.59	2	8	0	511.003	7	Cc1ccc(cc1)NS(=O)(=O)c2ccc(c(c2)OC)N/C=C/3\C(=NN(C3=O)c4ccc(cc4)Cl)C
ZINC08440273	4.61	9.14	-23.08	2	8	0	511.003	7	Cc1ccc(cc1)NS(=O)(=O)c2ccc(c(c2)OC)N/C=C\3/C(=NN(C3=O)c4ccc(cc4)Cl)C
ZINC08440273	4.61	9.19	-53.84	1	8	-1	509.995	7	Cc1ccc(cc1)[N-]S(=O)(=O)c2ccc(c(c2)OC)N/C=C\3/C(=NN(C3=O)c4ccc(cc4)Cl)C
ZINC08440273	4.61	9.21	-50.11	1	8	-1	509.995	7	Cc1ccc(cc1)[N-]S(=O)(=O)c2ccc(c(c2)OC)N/C=C/3\C(=NN(C3=O)c4ccc(cc4)Cl)C
ZINC08440272	5.92	-2.66	-15.39	3	4	0	596.627	8	c1cc(cc(c1)I)C(=O)N[C@H](C(Cl)(Cl)Cl)NC(=S)Nc2cccc(c2)C(F)(F)F
ZINC00659475	4.39	0.32	-18.2	1	6	0	518.742	3	Cc1c(c(n(n1)c2ccc(cc2)I)O)/C=C\3/C(=NN(C3=O)c4ccc(cc4)Cl)C
ZINC08440271	6.19	-1.01	-22.24	1	7	0	544.051	7	Cc1cccnc1NC(=O)C2[C@@H](C3=C(CCCC3=O)N=C2C)c4ccc(c(c4)COc5ccc(cc5)Cl)OC
ZINC08440271	6.19	-1.39	-22.17	1	7	0	544.051	7	Cc1cccnc1NC(=O)C2=C(N=C3CCCC(=O)C3[C@H]2c4ccc(c(c4)COc5ccc(cc5)Cl)OC)C
ZINC08440271	6.37	-2.24	-20.73	1	7	0	544.051	7	Cc1cccnc1NC(=O)C2[C@@H](C3C(=NC2=C)CCCC3=O)c4ccc(c(c4)COc5ccc(cc5)Cl)OC
ZINC08440270	5.92	-2.66	-11.6	3	4	0	596.627	8	c1cc(cc(c1)I)C(=O)N[C@@H](C(Cl)(Cl)Cl)NC(=S)Nc2cccc(c2)C(F)(F)F
ZINC08440264	4.85	-0.66	-18.92	2	6	0	473.356	8	CCOc1ccc(cc1OC)C(NC(=O)c2cccc(c2)Cl)NC(=O)c3cccc(c3)Cl
ZINC08440259	6.23	14.2	-31.49	2	7	0	544.051	7	Cc1cccnc1NC(=O)C2=C(NC3=C([C@@H]2c4ccc(c(c4)COc5ccc(cc5)Cl)OC)C(=O)CCC3)C
ZINC08440259	6.37	-1.84	-15.82	1	7	0	544.051	7	Cc1cccnc1NC(=O)C2[C@H](C3C(=NC2=C)CCCC3=O)c4ccc(c(c4)COc5ccc(cc5)Cl)OC
ZINC08440258	7.86	-4.46	-17.76	1	6	0	572.686	10	c1ccc(cc1)C(c2ccccc2)c3nc(c(o3)NCc4cccc(c4)Oc5ccccc5)S(=O)(=O)c6ccccc6
ZINC08440257	4.52	9.08	-25.18	2	8	0	490.585	7	Cc1ccc(cc1C)N2C(=O)/C(=C\Nc3ccc(cc3OC)S(=O)(=O)Nc4ccccc4)/C(=N2)C
ZINC08440257	4.52	9.21	-20.96	2	8	0	490.585	7	Cc1ccc(cc1C)N2C(=O)/C(=C/Nc3ccc(cc3OC)S(=O)(=O)Nc4ccccc4)/C(=N2)C
ZINC00702456	5.19	-3.05	-15.03	3	6	0	509.242	10	CCOC(=O)c1ccc(cc1)NC(=S)N[C@H](C(Cl)(Cl)Cl)NC(=O)c2cccc(c2)Cl
ZINC00702455	5.19	-3.05	-16.8	3	6	0	509.242	10	CCOC(=O)c1ccc(cc1)NC(=S)N[C@@H](C(Cl)(Cl)Cl)NC(=O)c2cccc(c2)Cl
ZINC08440254	3.79	-2.07	-19.94	3	9	0	550.848	12	COc1cc(cc(c1OC)OC)C(=O)N[C@H](C(Cl)(Cl)Cl)NC(=S)Nc2ccc(cc2)C(=O)OC
ZINC08440253	6.48	15.62	-21.39	1	9	0	585.653	12	CCOC(=O)[C@H]1[C@H](CC2=C(C1=O)[C@@H](C(=C(N2)C)C(=O)OCC)c3ccc(o3)COc4ccc(cc4)OC)c5ccccc5
ZINC08440253	6.44	2.16	-13.33	0	9	0	585.653	12	CCOC(=O)[C@H]1[C@H](CC2=C(C1=O)[C@@H]([C@@H](C(=N2)C)C(=O)OCC)c3ccc(o3)COc4ccc(cc4)OC)c5ccccc5
ZINC08440253	6.6	3.47	-18.45	0	9	0	585.653	12	CCOC(=O)[C@H]1[C@H](C=C2C(C1=O)[C@@H]([C@H](C(=N2)C)C(=O)OCC)c3ccc(o3)COc4ccc(cc4)OC)c5ccccc5
ZINC08440253	6.6	2.06	-13.05	0	9	0	585.653	12	CCOC(=O)[C@H]1[C@H](C=C2C(C1=O)[C@@H](C(C(=N2)C)C(=O)OCC)c3ccc(o3)COc4ccc(cc4)OC)c5ccccc5
ZINC08440248	3.79	-2.07	-19.84	3	9	0	550.848	12	COc1cc(cc(c1OC)OC)C(=O)N[C@@H](C(Cl)(Cl)Cl)NC(=S)Nc2ccc(cc2)C(=O)OC
ZINC08440246	4.33	9.39	-20.55	2	8	0	490.585	7	Cc1ccc(cc1)N2C(=O)/C(=C/Nc3ccc(cc3OC)S(=O)(=O)Nc4ccccc4C)/C(=N2)C
ZINC08440246	4.33	9.37	-25.05	2	8	0	490.585	7	Cc1ccc(cc1)N2C(=O)/C(=C\Nc3ccc(cc3OC)S(=O)(=O)Nc4ccccc4C)/C(=N2)C
ZINC08440246	4.33	9.42	-55.65	1	8	-1	489.577	7	Cc1ccc(cc1)N2C(=O)/C(=C\Nc3ccc(cc3OC)S(=O)(=O)[N-]c4ccccc4C)/C(=N2)C
ZINC08440246	4.33	9.45	-51.9	1	8	-1	489.577	7	Cc1ccc(cc1)N2C(=O)/C(=C/Nc3ccc(cc3OC)S(=O)(=O)[N-]c4ccccc4C)/C(=N2)C
ZINC00702452	5.05	-3.4	-17.88	3	5	0	494.831	8	CC(=O)c1ccc(cc1)NC(=S)N[C@H](C(Cl)(Cl)Cl)NC(=O)c2cccc3c2cccc3
ZINC08440243	3.99	10.08	-13.4	1	5	0	450.134	3	Cc1ccc(cc1)N2C(=O)/C(=C\Nc3c(cc(cn3)Br)Br)/C(=N2)C
ZINC08440243	3.99	10.11	-12.91	1	5	0	450.134	3	Cc1ccc(cc1)N2C(=O)/C(=C/Nc3c(cc(cn3)Br)Br)/C(=N2)C
ZINC00830700	3.55	0.78	-16.5	1	7	0	494.576	4	Cc1ccc(cc1)n2c(c(c(n2)C)C=C3C(=O)N(C(=S)N(C3=O)c4ccccc4)c5ccccc5)O
ZINC08440241	6.48	16.45	-21.34	1	9	0	585.653	12	CCOC(=O)[C@@H]1[C@H](CC2=C(C1=O)[C@@H](C(=C(N2)C)C(=O)OCC)c3ccc(o3)COc4ccc(cc4)OC)c5ccccc5
ZINC08440241	6.6	4.14	-27.6	0	9	0	585.653	12	CCOC(=O)[C@@H]1[C@H](C=C2C(C1=O)[C@@H]([C@@H](C(=N2)C)C(=O)OCC)c3ccc(o3)COc4ccc(cc4)OC)c5ccccc5
ZINC08440241	6.6	2.5	-12.7	0	9	0	585.653	12	CCOC(=O)[C@@H]1[C@H](C=C2C(C1=O)[C@@H](C(C(=N2)C)C(=O)OCC)c3ccc(o3)COc4ccc(cc4)OC)c5ccccc5
ZINC00702451	5.05	-3.44	-18.13	3	5	0	494.831	8	CC(=O)c1ccc(cc1)NC(=S)N[C@@H](C(Cl)(Cl)Cl)NC(=O)c2cccc3c2cccc3
ZINC08440238	4.63	-3.43	-15.96	3	5	0	438.808	11	CCCCCC(=O)N[C@H](C(Cl)(Cl)Cl)NC(=S)Nc1ccc(cc1)C(=O)C
ZINC08440235	4.63	-3.43	-15.61	3	5	0	438.808	11	CCCCCC(=O)N[C@@H](C(Cl)(Cl)Cl)NC(=S)Nc1ccc(cc1)C(=O)C

Supplementary Table S2:
Detailed table of XP Docking using Schrodinger's Glide module

ZINC_ID	xLogP	ApolarDesolvation	PolarDesolvation	H Donors	H Acceptors	Charge	MolecularWeight	RotatableBonds	SMILES
ZINC02062113	4.79	-3.03	-11.63	1	7	0	424.526	7	CCCCSc1nc2c(nn1)-c3ccccc3N[C@H](O2)c4ccc(c(c4)OC)OC
ZINC02062114	4.79	-3	-11.6	1	7	0	424.526	7	CCCCSc1nc2c(nn1)-c3ccccc3N[C@@H](O2)c4ccc(c(c4)OC)OC
ZINC00659419	6.01	1.85	-17.48	0	5	0	532.979	2	Cc1ccc(cc1)[C@@H]2C3([C@H](C4(O2)C(=O)c5ccccc5C4=O)c6cccc(c6)Cl)C(=O)c7ccccc7C3=O
ZINC00659421	6.01	1.84	-17.61	0	5	0	532.979	2	Cc1ccc(cc1)[C@H]2C3([C@@H](C4(O2)C(=O)c5ccccc5C4=O)c6cccc(c6)Cl)C(=O)c7ccccc7C3=O
ZINC08440232	6.57	-5.15	-11.72	3	4	0	608.998	7	Cc1cc(c(c(c1)Br)NC(=S)N[C@H](C(Cl)(Cl)Cl)NC(=O)c2cccc(c2)Cl)Br
ZINC00659423	6.01	1.92	-17.28	0	5	0	532.979	2	Cc1ccc(cc1)[C@@H]2C3([C@@H](C4(O2)C(=O)c5ccccc5C4=O)c6cccc(c6)Cl)C(=O)c7ccccc7C3=O
ZINC00210133	2.66	2.39	-9.87	1	4	0	310.202	4	c1cc(c(cc1Cl)S(=O)(=O)NC[C@H]2CCCO2)Cl
ZINC02904726	5.59	3.27	-14.33	0	5	0	428.495	4	c1ccc(cc1)Cc2nc3c(cnn3c4ccccc4)c(=O)n2c5cccc6c5cccc6
ZINC00970540	4.8	-3.26	-10.9	2	4	0	447.379	4	c1cc(sc1)C(=O)Nc2c(c3c(s2)CCC3)C(=O)Nc4ccc(cc4)Br
ZINC08440231	6.57	-5.15	-11.77	3	4	0	608.998	7	Cc1cc(c(c(c1)Br)NC(=S)N[C@@H](C(Cl)(Cl)Cl)NC(=O)c2cccc(c2)Cl)Br
ZINC00659408	5.15	-1.61	-13.92	2	5	0	440.952	6	CCOc1ccc(cc1)NC(=O)c2c3c(sc2NC(=O)c4ccccc4Cl)CCC3
ZINC00970533	5.07	-2.45	-12.07	2	5	0	426.563	5	COc1ccc(cc1)NC(=O)c2c3c(sc2NC(=O)c4cccs4)CCCCC3
ZINC02062105	6.17	-1.29	-11.9	2	4	0	438.98	4	Cc1cccc(c1)NC(=O)c2c3c(sc2NC(=O)c4ccccc4Cl)CCCCC3
ZINC08440230	6.1	-3.66	-14.83	3	4	0	499.678	7	Cc1ccc(cc1C)NC(=S)N[C@H](C(Cl)(Cl)Cl)NC(=O)c2ccc(cc2Cl)Cl
ZINC02062103	6.19	-1.29	-11.93	2	4	0	438.98	4	Cc1ccc(cc1)NC(=O)c2c3c(sc2NC(=O)c4ccccc4Cl)CCCCC3
ZINC02062101	6.55	-2.37	-11.41	2	4	0	503.849	4	c1ccc(c(c1)C(=O)Nc2c(c3c(s2)CCCCC3)C(=O)Nc4ccc(cc4)Br)Cl
ZINC02062100	5.92	-2.25	-10.2	2	4	0	469.404	4	c1ccc(cc1)C(=O)Nc2c(c3c(s2)CCCCC3)C(=O)Nc4ccc(cc4)Br
ZINC08440229	6.48	15.71	-20.75	1	9	0	585.653	12	CCOC(=O)[C@@H]1[C@@H](CC2=C(C1=O)[C@@H](C(=C(N2)C)C(=O)OCC)c3ccc(o3)COc4ccc(cc4)OC)c5ccccc5
ZINC08440229	6.6	2	-12.4	0	9	0	585.653	12	CCOC(=O)[C@@H]1[C@@H](C=C2C(C1=O)[C@@H](C(C(=N2)C)C(=O)OCC)c3ccc(o3)COc4ccc(cc4)OC)c5ccccc5
ZINC08440229	6.6	3.56	-17.17	0	9	0	585.653	12	CCOC(=O)[C@@H]1[C@@H](C=C2C(C1=O)[C@@H]([C@@H](C(=N2)C)C(=O)OCC)c3ccc(o3)COc4ccc(cc4)OC)c5ccccc5
ZINC08440229	6.44	2.2	-12.11	0	9	0	585.653	12	CCOC(=O)[C@@H]1[C@@H](CC2=C(C1=O)[C@@H]([C@H](C(=N2)C)C(=O)OCC)c3ccc(o3)COc4ccc(cc4)OC)c5ccccc5
ZINC08440228	6.1	-3.66	-14.79	3	4	0	499.678	7	Cc1ccc(cc1C)NC(=S)N[C@@H](C(Cl)(Cl)Cl)NC(=O)c2ccc(cc2Cl)Cl
ZINC02062099	6.42	-1.76	-11.44	2	4	0	459.398	4	c1ccc(c(c1)C(=O)Nc2c(c3c(s2)CCCCC3)C(=O)Nc4ccc(cc4)Cl)Cl
ZINC02062098	5.79	-1.64	-10.29	2	4	0	424.953	4	c1ccc(cc1)C(=O)Nc2c(c3c(s2)CCCCC3)C(=O)Nc4ccc(cc4)Cl
ZINC02062097	6.14	-1.07	-11.99	2	4	0	438.98	4	Cc1ccccc1NC(=O)c2c3c(sc2NC(=O)c4ccccc4Cl)CCCCC3
ZINC00659386	5.51	-1.15	-12.06	2	5	0	434.561	6	CCOc1ccc(cc1)NC(=O)c2c3c(sc2NC(=O)c4ccccc4)C[C@H](CC3)C
ZINC08440227	5.6	-4.14	-12.58	3	4	0	509.684	7	Cc1ccc(cc1C)NC(=S)N[C@H](C(Cl)(Cl)Cl)NC(=O)c2cccc(c2)Br
ZINC02062094	5.91	-1.82	-11.82	2	4	0	445.371	4	c1ccc(c(c1)C(=O)Nc2c(c3c(s2)CCCC3)C(=O)Nc4ccc(cc4)Cl)Cl
ZINC08440226	4.9	10.78	-16.15	1	6	0	409.902	4	Cc1c(cccc1Cl)N/N=C/2\C(=NN(C2=O)c3nc(cs3)c4ccccc4)C
ZINC08440223	5.6	-4.14	-12.59	3	4	0	509.684	7	Cc1ccc(cc1C)NC(=S)N[C@@H](C(Cl)(Cl)Cl)NC(=O)c2cccc(c2)Br
ZINC00198961	3.48	11.59	-14.78	0	5	0	322.393	3	Cn1cnnc1SCC(=O)n2c3ccccc3c4c2cccc4
ZINC08440222	5.5	5.39	-24.59	0	10	0	695.837	10	Cc1cc(c2c(c1)C3=C(C(N2C(=O)Cc4ccccc4)(C)C)SC(=C(C35SC(=C(S5)C(=O)OC)C(=O)OC)C(=O)OC)C(=O)OC)C
ZINC08440221	3.52	4.69	-23.58	0	11	0	649.765	11	CCC(=O)N1c2ccc(cc2C3=C(C1(C)C)SC(=C(C34SC(=C(S4)C(=O)OC)C(=O)OC)C(=O)OC)C(=O)OC)OCC
ZINC00702450	4.93	-2.72	-18.97	3	4	0	448.778	7	Cc1ccc(cc1C)NC(=S)N[C@H](C(Cl)(Cl)Cl)NC(=O)c2ccccc2F
ZINC08440220	4.64	4.95	-19.17	0	11	0	683.782	10	CC1(C2=C(c3cc(ccc3N1C(=O)c4ccccc4)OC)C5(C(=C(S2)C(=O)OC)C(=O)OC)SC(=C(S5)C(=O)OC)C(=O)OC)C
ZINC00970507	3.7	9.16	-7.14	1	3	0	296.395	2	C[C@H]1CCc2c(c(c(s2)NC(=O)c3ccccc3)C#N)C1
ZINC08440218	3.81	14.45	-73.69	1	6	0	449.938	7	Cc1ccc(c(c1)C(=O)C2=C(C(=O)N([C@H]2c3cccc(c3)Cl)CCCn4cc[nH+]c4)[O-])C
ZINC08440218	3.81	13.94	-60.48	0	6	-1	448.93	7	Cc1ccc(c(c1)C(=O)C2=C(C(=O)N([C@H]2c3cccc(c3)Cl)CCCn4ccnc4)[O-])C
ZINC00702446	3.81	14.06	-44.82	0	6	-1	448.93	7	Cc1ccc(c(c1)C(=O)C2=C(C(=O)N([C@@H]2c3cccc(c3)Cl)CCCn4ccnc4)[O-])C
ZINC00702446	3.23	1.32	-30.44	0	6	0	449.938	7	Cc1ccc(c(c1)C(=O)[C@H]2[C@H](N(C(=O)C2=O)CCCn3ccnc3)c4cccc(c4)Cl)C
ZINC00702446	4.25	1.61	-45.17	2	6	1	450.946	6	Cc1ccc(c(c1)/C(=C/2\[C@H](N(C(=O)C2=O)CCCn3cc[nH+]c3)c4cccc(c4)Cl)/O)C
ZINC00702446	4.25	1.22	-55.11	2	6	1	450.946	6	Cc1ccc(c(c1)/C(=C\2/[C@H](N(C(=O)C2=O)CCCn3cc[nH+]c3)c4cccc(c4)Cl)/O)C
ZINC00702446	3.23	1.6	-52.93	1	6	1	450.946	7	Cc1ccc(c(c1)C(=O)[C@H]2[C@H](N(C(=O)C2=O)CCCn3cc[nH+]c3)c4cccc(c4)Cl)C
ZINC00702446	3.81	14.54	-54.17	1	6	0	449.938	7	Cc1ccc(c(c1)C(=O)C2=C(C(=O)N([C@@H]2c3cccc(c3)Cl)CCCn4cc[nH+]c4)[O-])C
ZINC08440217	4.88	-2.07	-14.32	1	8	0	559.42	6	CC1=NC2=C([C@@H](C1C(=O)Nc3ccc(cn3)Br)c4ccc(o4)COc5ccc(cc5)C#N)C(=O)CCC2
ZINC08440217	5.06	-1.03	-14.7	1	8	0	559.42	6	C=C1C([C@H]([C@H]2C(=N1)CCCC2=O)c3ccc(o3)COc4ccc(cc4)C#N)C(=O)Nc5ccc(cn5)Br
ZINC08440217	5.06	-2.14	-15.55	1	8	0	559.42	6	C=C1C([C@H](C2C(=N1)CCCC2=O)c3ccc(o3)COc4ccc(cc4)C#N)C(=O)Nc5ccc(cn5)Br
ZINC08440217	4.88	-1.66	-17.14	1	8	0	559.42	6	CC1=C([C@H]([C@@H]2C(=N1)CCCC2=O)c3ccc(o3)COc4ccc(cc4)C#N)C(=O)Nc5ccc(cn5)Br
ZINC04676690	4.78	3.11	-10.42	1	6	0	397.899	13	CCCCC(CCC(=O)Nc1ccccc1Cl)(C(=O)OCC)C(=O)OCC
ZINC08440216	5.04	13.05	-43.77	0	6	-1	460.579	5	Cc1ccc(c(c1)C(=O)C2=C(C(=O)N([C@H]2c3ccc(cc3)C(C)(C)C)c4nnc(s4)C)[O-])C
ZINC08440216	5.48	1.74	-22.15	1	6	0	461.587	4	Cc1ccc(c(c1)/C(=C\2/[C@@H](N(C(=O)C2=O)c3nnc(s3)C)c4ccc(cc4)C(C)(C)C)/O)C
ZINC08440216	4.45	2	-16.33	0	6	0	461.587	5	Cc1ccc(c(c1)C(=O)[C@@H]2[C@@H](N(C(=O)C2=O)c3nnc(s3)C)c4ccc(cc4)C(C)(C)C)C
ZINC08440215	7.53	1.03	-15.95	1	6	0	588.154	9	CCOC(=O)c1c2c(sc1NC(=O)CSc3c(c(cc(n3)c4ccc(cc4)Cl)c5ccccc5)C#N)CCCC2
ZINC08440214	7.93	-2.07	-12.03	3	6	0	588.154	7	CCOC(=O)c1c2c(sc1NC(=O)c3c(c4c(cc(nc4s3)c5ccc(cc5)Cl)c6ccccc6)N)CCCC2
ZINC08440213	5.04	12.81	-43.97	0	6	-1	460.579	5	Cc1ccc(c(c1)C(=O)C2=C(C(=O)N([C@@H]2c3ccc(cc3)C(C)(C)C)c4nnc(s4)C)[O-])C
ZINC08440213	5.48	1.69	-22.02	1	6	0	461.587	4	Cc1ccc(c(c1)/C(=C\2/[C@H](N(C(=O)C2=O)c3nnc(s3)C)c4ccc(cc4)C(C)(C)C)/O)C
ZINC08440213	4.45	2.05	-16.07	0	6	0	461.587	5	Cc1ccc(c(c1)C(=O)[C@H]2[C@H](N(C(=O)C2=O)c3nnc(s3)C)c4ccc(cc4)C(C)(C)C)C
ZINC08440206	4.88	-2.33	-15.86	1	8	0	559.42	6	CC1=NC2=C([C@H](C1C(=O)Nc3ccc(cn3)Br)c4ccc(o4)COc5ccc(cc5)C#N)C(=O)CCC2
ZINC08440206	5.06	-1.01	-18.25	1	8	0	559.42	6	C=C1C([C@@H]([C@@H]2C(=N1)CCCC2=O)c3ccc(o3)COc4ccc(cc4)C#N)C(=O)Nc5ccc(cn5)Br
ZINC08440206	5.06	-2.27	-15.19	1	8	0	559.42	6	C=C1C([C@@H](C2C(=N1)CCCC2=O)c3ccc(o3)COc4ccc(cc4)C#N)C(=O)Nc5ccc(cn5)Br
ZINC08440206	4.88	-1.94	-16.84	1	8	0	559.42	6	CC1=C([C@@H]([C@H]2C(=N1)CCCC2=O)c3ccc(o3)COc4ccc(cc4)C#N)C(=O)Nc5ccc(cn5)Br

Supplementary Table S2:
Detailed table of XP Docking using Schrodinger's Glide module

ZINC_ID	xLogP	ApolarDesolvation	PolarDesolvation	H Donors	H Acceptors	Charge	MolecularWeight	RotatableBonds	SMILES
ZINC00702440	4.72	12.19	-60.43	1	6	-1	470.545	8	CCOc1cc(ccc1O)[C@@H]2C(=C(C(=O)N2CCc3ccccc3)[O-])C(=O)c4cc(ccc4C)C
ZINC00702440	5.17	-0.63	-17.67	2	6	0	471.553	7	CCOc1cc(ccc1O)[C@@H]2/C(=C(\c3cc(ccc3C)C)/O)/C(=O)C(=O)N2CCc4ccccc4
ZINC00702440	4.14	-0.48	-14.58	1	6	0	471.553	8	CCOc1cc(ccc1O)[C@@H]2[C@@H](C(=O)C(=O)N2CCc3ccccc3)C(=O)c4cc(ccc4C)C
ZINC00659345	4.25	3.14	-35.09	1	8	0	506.488	5	Cc1ccc(cc1)c2cc(n3c(n2)c(cn3)C(=O)Nc4c(n(n(c4=O)c5ccccc5)C)C)C(F)(F)F
ZINC00848684	4.75	1.85	-21.04	1	7	0	440.381	4	Cc1ccc(cc1)c2cc(n3c(n2)c(cn3)C(=O)Nc4ccc5c(c4)OCO5)C(F)(F)F
ZINC00702437	4.84	13.1	-53.98	0	6	-1	446.552	5	Cc1ccc(c(c1)C(=O)C2=C(C(=O)N([C@H]2c3ccc(cc3)C(C)C)c4nnc(s4)C)[O-])C
ZINC00702437	5.29	1.74	-16.13	1	6	0	447.56	4	Cc1ccc(c(c1)/C(=C/2\[C@@H](N(C(=O)C2=O)c3nnc(s3)C)c4ccc(cc4)C(C)C)/O)C
ZINC00702437	5.29	1.4	-22.17	1	6	0	447.56	4	Cc1ccc(c(c1)/C(=C\2/[C@@H](N(C(=O)C2=O)c3nnc(s3)C)c4ccc(cc4)C(C)C)/O)C
ZINC00702437	4.26	1.64	-16.23	0	6	0	447.56	5	Cc1ccc(c(c1)C(=O)[C@@H]2[C@@H](N(C(=O)C2=O)c3nnc(s3)C)c4ccc(cc4)C(C)C)C
ZINC00848672	6.03	13.15	-17.09	1	5	0	446.432	4	Cc1ccc(cc1)c2cc(n3c(n2)c(cn3)C(=O)Nc4cccc5c4cccc5)C(F)(F)F
ZINC08440205	4.84	12.94	-57.81	0	6	-1	446.552	5	Cc1ccc(c(c1)C(=O)C2=C(C(=O)N([C@@H]2c3ccc(cc3)C(C)C)c4nnc(s4)C)[O-])C
ZINC08440205	5.29	1.74	-16.09	1	6	0	447.56	4	Cc1ccc(c(c1)/C(=C/2\[C@H](N(C(=O)C2=O)c3nnc(s3)C)c4ccc(cc4)C(C)C)/O)C
ZINC08440205	5.29	1.36	-22.04	1	6	0	447.56	4	Cc1ccc(c(c1)/C(=C\2/[C@H](N(C(=O)C2=O)c3nnc(s3)C)c4ccc(cc4)C(C)C)/O)C
ZINC08440205	4.26	1.71	-16.11	0	6	0	447.56	5	Cc1ccc(c(c1)C(=O)[C@H]2[C@H](N(C(=O)C2=O)c3nnc(s3)C)c4ccc(cc4)C(C)C)C
ZINC08440204	6.43	16.29	-19.18	2	6	0	536.031	5	CC1=C([C@H](C2=C(N1)C[C@H](CC2=O)c3ccccc3)c4ccc(o4)c5cccc(c5)Cl)C(=O)Nc6ccccn6
ZINC08440204	6.57	-1.52	-12.69	1	6	0	536.031	5	C=C1C([C@H](C2C(=N1)C[C@H](CC2=O)c3ccccc3)c4ccc(o4)c5cccc(c5)Cl)C(=O)Nc6ccccn6
ZINC08440204	6.57	-0.23	-13.72	1	6	0	536.031	5	C=C1C([C@H]([C@H]2C(=N1)C[C@H](CC2=O)c3ccccc3)c4ccc(o4)c5cccc(c5)Cl)C(=O)Nc6ccccn6
ZINC00659343	4.87	1.44	-18.84	1	9	0	520.851	6	COc1ccc(cc1OC)c2cc(n3c(n2)c(c(n3)C(=O)Nc4ccc5c(c4)OCO5)Cl)C(F)(F)F
ZINC00702433	3.74	9.15	-48.18	1	6	-1	428.464	7	COc1cccc(c1)C(=O)C2=C(C(=O)N([C@H]2c3ccc(cc3)O)CCc4ccccc4)[O-]
ZINC00702433	4.18	-1.52	-29.3	2	6	0	429.472	6	COc1cccc(c1)C(=C2[C@@H](N(C(=O)C2=O)CCc3ccccc3)c4ccc(cc4)O)O
ZINC00702433	3.15	-1.23	-26.08	1	6	0	429.472	7	COc1cccc(c1)C(=O)[C@@H]2[C@@H](N(C(=O)C2=O)CCc3ccccc3)c4ccc(cc4)O
ZINC08440203	4.82	3.04	-51.4	1	8	1	560.984	7	COc1ccc(cc1OC)c2cc(n3c(n2)c(c(n3)C(=O)N4CC[NH+](CC4)Cc5ccccc5)Cl)C(F)(F)F
ZINC00659339	6.14	2.63	-16.12	1	7	0	526.902	6	COc1ccc(cc1OC)c2cc(n3c(n2)c(c(n3)C(=O)Nc4cccc5c4cccc5)Cl)C(F)(F)F
ZINC00848599	6.15	13.43	-11.56	0	6	0	520.342	4	c1ccc(cc1)N2CCN(CC2)C(=O)c3c(c4nc(cc(n4n3)C(F)(F)F)c5ccc(cc5)Cl)Cl
ZINC00702434	3.74	8.74	-46.24	1	6	-1	428.464	7	COc1cccc(c1)C(=O)C2=C(C(=O)N([C@@H]2c3ccc(cc3)O)CCc4ccccc4)[O-]
ZINC00702434	4.18	-1.82	-28.13	2	6	0	429.472	6	COc1cccc(c1)C(=C2[C@H](N(C(=O)C2=O)CCc3ccccc3)c4ccc(cc4)O)O
ZINC00702434	3.15	-1.58	-24.9	1	6	0	429.472	7	COc1cccc(c1)C(=O)[C@H]2[C@H](N(C(=O)C2=O)CCc3ccccc3)c4ccc(cc4)O
ZINC00659333	5.8	1.98	-12.81	0	6	0	521.877	4	c1cc(ccc1c2cc(n3c(n2)c(c(n3)C(=O)N4CCN(CC4)c5ccc(cc5)F)Cl)C(F)(F)F)F
ZINC00848555	5.6	9.57	-12.23	1	6	0	452.864	5	COc1ccc(cc1)c2cc(n3c(n2)c(c(n3)C(=O)NC4CCCCC4)Cl)C(F)(F)F
ZINC00702428	2.39	7.48	-44.42	1	7	-1	429.452	7	CCOc1cccc(c1)C(=O)C2=C(C(=O)N([C@H]2c3cccc(c3)O)Cc4ccncc4)[O-]
ZINC00702428	2.84	-2.93	-28.35	2	7	0	430.46	6	CCOc1cccc(c1)C(=C2[C@@H](N(C(=O)C2=O)Cc3ccncc3)c4cccc(c4)O)O
ZINC00702428	1.81	-2.68	-28.22	1	7	0	430.46	7	CCOc1cccc(c1)C(=O)[C@@H]2[C@@H](N(C(=O)C2=O)Cc3ccncc3)c4cccc(c4)O
ZINC08440202	6.39	-1.16	-17.4	1	6	0	536.031	5	CC1=NC2=C([C@H](C1C(=O)Nc3ccccn3)c4ccc(o4)c5cccc(c5)Cl)C(=O)C[C@@H](C2)c6ccccc6
ZINC08440202	6.57	-1.07	-16.14	1	6	0	536.031	5	C=C1C([C@@H](C2C(=N1)C[C@H](CC2=O)c3ccccc3)c4ccc(o4)c5cccc(c5)Cl)C(=O)Nc6ccccn6
ZINC08440202	6.57	-0.12	-16.65	1	6	0	536.031	5	C=C1C([C@@H]([C@H]2C(=N1)C[C@H](CC2=O)c3ccccc3)c4ccc(o4)c5cccc(c5)Cl)C(=O)Nc6ccccn6
ZINC00659331	5.57	2.32	-12.7	1	8	0	538.935	7	CCOC(=O)c1c2c(sc1NC(=O)c3c(c4nc(cc(n4n3)C(F)(F)F)c5ccco5)Cl)CCCC2
ZINC08440201	5.45	3.79	-9.53	1	7	0	490.747	6	c1cc(oc1)c2cc(n3c(n2)c(c(n3)C(=O)Nc4ccc(cc4)OC(F)(F)F)Cl)C(F)(F)F
ZINC08440200	2.39	6.29	-62.8	1	7	-1	429.452	7	CCOc1cccc(c1)C(=O)C2=C(C(=O)N([C@@H]2c3cccc(c3)O)Cc4ccncc4)[O-]
ZINC08440200	2.84	-2.53	-28.77	2	7	0	430.46	6	CCOc1cccc(c1)C(=C2[C@H](N(C(=O)C2=O)Cc3ccncc3)c4cccc(c4)O)O
ZINC08440200	1.81	-2.26	-25.17	1	7	0	430.46	7	CCOc1cccc(c1)C(=O)[C@H]2[C@H](N(C(=O)C2=O)Cc3ccncc3)c4cccc(c4)O
ZINC00659329	4.25	2.15	-15.78	1	6	0	449.882	7	CCOC(=O)c1c2c(sc1NC(=O)Cn3c(c(c(n3)C(F)(F)F)Cl)C)CCCC2
ZINC00702426	2.93	9.67	-45.57	0	7	-1	443.479	8	CCOc1cccc(c1)C(=O)C2=C(C(=O)N([C@H]2c3cccc(c3)OC)Cc4ccncc4)[O-]
ZINC00702426	3.38	-0.94	-26.49	1	7	0	444.487	7	CCOc1cccc(c1)C(=C2[C@@H](N(C(=O)C2=O)Cc3ccncc3)c4cccc(c4)OC)O
ZINC00702426	2.35	-0.69	-25.5	0	7	0	444.487	8	CCOc1cccc(c1)C(=O)[C@@H]2[C@@H](N(C(=O)C2=O)Cc3ccncc3)c4cccc(c4)OC
ZINC08440199	6.27	1.72	-9.01	1	5	0	479.289	5	C[C@H](c1ccccc1)NC(=O)c2cc3nc(cc(n3n2)C(F)(F)F)c4ccc(c(c4)Cl)Cl
ZINC08440198	6.27	1.74	-11.96	1	5	0	479.289	5	C[C@@H](c1ccccc1)NC(=O)c2cc3nc(cc(n3n2)C(F)(F)F)c4ccc(c(c4)Cl)Cl
ZINC00702427	2.93	9.69	-46.4	0	7	-1	443.479	8	CCOc1cccc(c1)C(=O)C2=C(C(=O)N([C@@H]2c3cccc(c3)OC)Cc4ccncc4)[O-]
ZINC00702427	3.38	-0.94	-27.06	1	7	0	444.487	7	CCOc1cccc(c1)C(=C2[C@H](N(C(=O)C2=O)Cc3ccncc3)c4cccc(c4)OC)O
ZINC00702427	2.35	-0.64	-24.92	0	7	0	444.487	8	CCOc1cccc(c1)C(=O)[C@H]2[C@H](N(C(=O)C2=O)Cc3ccncc3)c4cccc(c4)OC
ZINC17160093	2.26	9.95	-64.28	0	7	-1	422.486	8	COc1cccc(c1)C(=O)C2=C(C(=O)N([C@H]2c3cccs3)CCCn4ccnc4)[O-]
ZINC17160093	1.68	10.16	-18.42	0	7	0	423.494	8	COc1cccc(c1)C(=O)[C@@H]2[C@@H](N(C(=O)C2=O)CCCn3ccnc3)c4cccs4
ZINC17160093	2.71	10.8	-41.24	2	7	1	424.502	7	COc1cccc(c1)/C(=C/2\[C@@H](N(C(=O)C2=O)CCCn3cc[nH+]c3)c4cccs4)/O
ZINC17160093	1.68	10.67	-44.5	1	7	1	424.502	8	COc1cccc(c1)C(=O)[C@@H]2[C@@H](N(C(=O)C2=O)CCCn3cc[nH+]c3)c4cccs4
ZINC17160093	2.26	10.43	-77.85	1	7	0	423.494	8	COc1cccc(c1)C(=O)C2=C(C(=O)N([C@H]2c3cccs3)CCCn4cc[nH+]c4)[O-]
ZINC17160090	2.26	9.72	-65.96	0	7	-1	422.486	8	COc1cccc(c1)C(=O)C2=C(C(=O)N([C@@H]2c3cccs3)CCCn4ccnc4)[O-]
ZINC17160090	2.71	10.18	-43.3	2	7	1	424.502	7	COc1cccc(c1)/C(=C/2\[C@H](N(C(=O)C2=O)CCCn3cc[nH+]c3)c4cccs4)/O
ZINC17160090	2.26	10.2	-77.54	1	7	0	423.494	8	COc1cccc(c1)C(=O)C2=C(C(=O)N([C@@H]2c3cccs3)CCCn4cc[nH+]c4)[O-]
ZINC08440196	8.08	1.11	-14.35	1	5	0	568.913	8	CCOc1ccc(cc1)c2cc(c(c(n2)SCC(=O)Nc3c(cc(cc3Cl)Cl)Cl)C#N)c4ccccc4
ZINC08440195	6.43	16.29	-18.93	2	6	0	536.031	5	CC1=C([C@H](C2=C(N1)C[C@@H](CC2=O)c3ccccc3)c4ccc(o4)c5cccc(c5)Cl)C(=O)Nc6ccccn6
ZINC08440195	6.57	-1.67	-12.62	1	6	0	536.031	5	C=C1C([C@H](C2C(=N1)C[C@@H](CC2=O)c3ccccc3)c4ccc(o4)c5cccc(c5)Cl)C(=O)Nc6ccccn6
ZINC08440195	6.57	-0.3	-13.6	1	6	0	536.031	5	C=C1C([C@H]([C@H]2C(=N1)C[C@@H](CC2=O)c3ccccc3)c4ccc(o4)c5cccc(c5)Cl)C(=O)Nc6ccccn6

Supplementary Table S2:
Detailed table of XP Docking using Schrodinger's Glide module

ZINC_ID	xLogP	ApolarDesolvation	PolarDesolvation	H Donors	H Acceptors	Charge	MolecularWeight	RotatableBonds	SMILES
ZINC08440194	6.5	0.99	-15.57	1	5	0	485.996	7	COc1ccc(cc1)c2cc(nc(c2C#N)SCC(=O)Nc3ccc(cc3)Cl)c4ccccc4
ZINC05918873	3.46	8.67	-59.14	1	7	-1	457.506	9	CCCCOc1cccc(c1)C(=O)C2=C(C(=O)N([C@H]2c3cccc(c3)O)Cc4ccncc4)[O-]
ZINC05918873	3.9	-2.73	-28.08	2	7	0	458.514	8	CCCCOc1cccc(c1)C(=C2[C@@H](N(C(=O)C2=O)Cc3ccncc3)c4cccc(c4)O)O
ZINC05918873	2.87	-2.48	-28	1	7	0	458.514	9	CCCCOc1cccc(c1)C(=O)[C@@H]2[C@@H](N(C(=O)C2=O)Cc3ccncc3)c4cccc(c4)O
ZINC06442301	5.83	13.28	-17.58	1	6	0	495.604	8	Cc1cccc(c1)NC(=O)CSc2c(c(cc(n2)c3ccccc3)c4ccc(c(c4)OC)OC)C#N
ZINC05918870	3.46	7.87	-62.39	1	7	-1	457.506	9	CCCCOc1cccc(c1)C(=O)C2=C(C(=O)N([C@@H]2c3cccc(c3)O)Cc4ccncc4)[O-]
ZINC05918870	3.9	-2.34	-28.49	2	7	0	458.514	8	CCCCOc1cccc(c1)C(=C2[C@H](N(C(=O)C2=O)Cc3ccncc3)c4cccc(c4)O)O
ZINC05918870	2.87	-2.07	-24.96	1	7	0	458.514	9	CCCCOc1cccc(c1)C(=O)[C@H]2[C@H](N(C(=O)C2=O)Cc3ccncc3)c4cccc(c4)O
ZINC08440193	6.84	1.45	-12.8	1	4	0	469.997	6	Cc1ccc(cc1)c2cc(nc(c2C#N)SCC(=O)Nc3ccccc3Cl)c4ccccc4
ZINC08440192	7.02	0.67	-14.76	1	4	0	514.448	6	Cc1ccc(cc1)c2cc(nc(c2C#N)SCC(=O)Nc3ccc(cc3)Br)c4ccccc4
ZINC08440191	6.08	1.31	-17.53	1	6	0	516.022	8	COc1ccc(cc1OC)c2cc(nc(c2C#N)SCC(=O)Nc3ccc(cc3)Cl)c4ccccc4
ZINC00848231	5.31	0.66	-10.38	0	6	0	436.464	9	c1ccc(cc1)COc2ccc(cc2)OC(=O)CSc3nnc(o3)c4ccc(cc4)F
ZINC08738116	3.43	9.97	-16.25	1	8	0	478.621	7	CCOC(=O)c1c(nc(s1)NC(=O)CSc2nc3c(c4c(s3)CCCC4)c(=O)n2C)C
ZINC08440190	6.43	15.34	-21.09	2	6	0	536.031	5	CC1=C([C@@H](C2=C(N1)C[C@@H](CC2=O)c3ccccc3)c4ccc(o4)c5cccc(c5)Cl)C(=O)Nc6ccccn6
ZINC08440190	6.57	-0.88	-15.41	1	6	0	536.031	5	C=C1C([C@@H]([C@@H]2C(=N1)C[C@@H](CC2=O)c3ccccc3)c4ccc(o4)c5cccc(c5)Cl)C(=O)Nc6ccccn6
ZINC08440190	6.39	-1.8	-15.48	1	6	0	536.031	5	CC1=C([C@@H]([C@H]2C(=N1)C[C@@H](CC2=O)c3ccccc3)c4ccc(o4)c5cccc(c5)Cl)C(=O)Nc6ccccn6
ZINC08440190	6.57	-2.12	-13.55	1	6	0	536.031	5	C=C1C([C@@H](C2C(=N1)C[C@@H](CC2=O)c3ccccc3)c4ccc(o4)c5cccc(c5)Cl)C(=O)Nc6ccccn6
ZINC00970487	5.28	-2.27	-12.59	0	5	0	428.923	3	c1ccc(cc1)c2c3cc(ccc3nc(n2)N4CCN(CC4)C(=O)c5ccccc5)Cl
ZINC08440189	6.52	-0.87	-12.05	1	5	0	589.715	6	c1cc(ccc1[C@H]2CC(=NN2C(=O)CCC(=O)Nc3ccc(cc3)Cl)c4ccc(cc4)Br)Br
ZINC08440188	6.52	-0.88	-12.05	1	5	0	589.715	6	c1cc(ccc1[C@@H]2CC(=NN2C(=O)CCC(=O)Nc3ccc(cc3)Cl)c4ccc(cc4)Br)Br
ZINC13549728	3.5	10.25	-15.7	1	8	0	478.621	9	CCOC(=O)c1c(nc(s1)NC(=O)CSc2nc3c(c(c(s3)C)C)c(=O)n2CC=C)C
ZINC06446786	6.98	-1.28	-12.18	2	5	0	602.282	5	c1ccc(cc1)c2c3cc(ccc3[nH]c(=O)c2C(=O)Nc4ccc(cc4C(=O)c5ccccc5)Br)Br
ZINC01804641	6.62	0.6	-12.75	0	4	0	468.818	5	Cn1c(nnc1SCC(=O)c2ccc(cc2Cl)Cl)c3c(c4ccccc4s3)Cl
ZINC00702416	6.02	0.72	-12.15	0	4	0	434.373	5	Cn1c(nnc1SCC(=O)c2ccc(cc2)Cl)c3c(c4ccccc4s3)Cl
ZINC08440182	5.74	0.3	-17.59	0	6	0	533.391	3	c1cc(cc(c1)I)C(=O)O/N=C/2\CCCc3c2sc4c3c(=O)n5c(n4)CCCCC5
ZINC06445859	6.6	0.31	-55.8	1	6	-1	548.855	5	c1ccc(c(c1)[N+](=O)[O-])S[C@H]2C[C@H]3[C@@H]([C@@H]2Cl)c4cccc(c4N[C@@H]3c5ccc(cc5Cl)Cl)C(=O)[O-]
ZINC00702413	4.56	2.08	-14.78	0	7	0	468.362	8	Cn1c(nnc1SCC(=O)c2ccc(cc2Cl)Cl)c3cc(c(c(c3)OC)OC)OC
ZINC06445776	6.6	-0.11	-75.03	1	6	-1	548.855	5	c1ccc(c(c1)[N+](=O)[O-])S[C@@H]2C[C@H]3[C@@H]([C@@H]2Cl)c4cccc(c4N[C@@H]3c5ccc(cc5Cl)Cl)C(=O)[O-]
ZINC06445810	6.6	-0.26	-53.53	1	6	-1	548.855	5	c1ccc(c(c1)[N+](=O)[O-])S[C@H]2C[C@@H]3[C@@H]([C@@H]2Cl)c4cccc(c4N[C@@H]3c5ccc(cc5Cl)Cl)C(=O)[O-]
ZINC06445741	6.6	-0.12	-59.64	1	6	-1	548.855	5	c1ccc(c(c1)[N+](=O)[O-])S[C@@H]2C[C@@H]3[C@@H]([C@@H]2Cl)c4cccc(c4N[C@@H]3c5ccc(cc5Cl)Cl)C(=O)[O-]
ZINC00702412	5.07	2.88	-15.43	0	7	0	475.57	9	Cn1c(nnc1SCC(=O)c2ccc(cc2)c3ccccc3)c4cc(c(c(c4)OC)OC)OC
ZINC02062063	4.34	0.29	-24.7	2	10	0	509.54	9	COC(=O)c1c2c(sc1NC(=O)COc3cccc(c3)NC(=O)c4ccc(cc4)[N+](=O)[O-])CCCC2
ZINC08440179	6.84	-0.49	-13.04	3	7	0	523.614	7	Cc1ccc(cc1)c2cc(c3c(c(sc3n2)C(=O)Nc4ccc(cc4)C(=O)OC)N)c5ccc(cc5)OC
ZINC00702411	5.33	1.24	-15.24	0	6	0	429.501	6	Cn1c(nnc1SCC(=O)c2ccc(cc2)c3ccccc3)c4ccc5c(c4)OCO5
ZINC08440177	7.21	-0.53	-12.8	3	7	0	537.641	8	CCOC(=O)c1ccc(cc1)NC(=O)c2c(c3c(cc(nc3s2)c4ccc(cc4)C)c5ccc(cc5)OC)N
ZINC02617389	5.42	2.02	-12.91	0	5	0	448.551	7	c1ccc(cc1)c2ccc(cc2)C(=O)CSc3nnc(n3c4ccccc4)c5ccncc5
ZINC02690519	3.95	2.2	-14.03	0	7	0	433.917	8	Cn1c(nnc1SCC(=O)c2ccc(cc2)Cl)c3cc(c(c(c3)OC)OC)OC
ZINC08440172	2.69	-6.85	-20.11	4	11	0	547.593	10	C[C@H](C(=O)NNC(=O)CSc1nc2ccccc2o1)NC(=O)c3ccccc3NC(=O)c4ccc(cc4)OC
ZINC08440171	2.69	-6.85	-20.34	4	11	0	547.593	10	C[C@@H](C(=O)NNC(=O)CSc1nc2ccccc2o1)NC(=O)c3ccccc3NC(=O)c4ccc(cc4)OC
ZINC08440170	4.53	2.21	-11.28	0	4	0	424.234	6	COc1cc(cc(c1OC)OC)/C=C/C(=O)c2ccc(cc2)I
ZINC08440169	6.07	14.81	-20.53	2	6	0	541.449	3	CC1=C([C@H](C2=C(N1)CCCC2=O)c3ccc4c(c3)c5ccccc5n4C)C(=O)Nc6ccc(cn6)Br
ZINC01771078	6.49	15.29	-11.82	0	6	0	446.51	5	Cc1cccc(c1OCc2nc3c4c(c(oc4ncn3n2)c5ccccc5)c6ccccc6)C
ZINC08440168	5.91	-0.68	-43.56	2	5	1	433.388	1	CC1(Cc2c3c4nc(nn4cnc3sc2C([NH2+]1)(C)C)c5ccc(cc5Cl)Cl)C
ZINC08440167	4.54	2.44	-12.12	0	4	0	424.234	6	COc1ccc(c(c1OC)OC)/C=C/C(=O)c2ccc(cc2)I
ZINC08440166	4.86	-0.45	-11.01	0	6	0	456.369	4	C/C(=N\OCc1nc2c3c4c(sc3ncn2n1)CCCC4)/c5ccc(cc5)Br
ZINC02290294	5.64	1.36	-14.37	0	7	0	462.509	6	COc1ccc(cc1OC)Cc2nc3c4c(c(oc4ncn3n2)c5ccccc5)c6ccccc6
ZINC08440165	4.85	1.29	-10.57	1	7	0	423.494	6	CCOC(=O)C1=C(N=c2n(c(=O)/c(=C\c3ccc[nH]3)/s2)[C@H]1c4ccc(cc4)OC)C
ZINC08440164	5.68	0.36	-44.02	2	6	1	450.632	4	Cc1ccc(c(c1)OCc2nc3c4c5c(sc4ncn3n2)C([NH2+]C(C5)(C)C)(C)C)C(C)C
ZINC02277437	6.79	-1.73	-14.72	0	6	0	491.601	5	c1ccc(cc1)c2c3c4nc(nn4cnc3oc2c5ccccc5)CSc6nc7ccccc7s6
ZINC08440163	6.07	14.8	-20.42	2	6	0	541.449	3	CC1=C([C@@H](C2=C(N1)CCCC2=O)c3ccc4c(c3)c5ccccc5n4C)C(=O)Nc6ccc(cn6)Br
ZINC01902382	6.81	0.45	-11.75	0	5	0	467.326	3	c1ccc(cc1)c2c3c4nc(nn4cnc3oc2c5ccccc5)c6ccccc6Br
ZINC02062059	6.1	1.01	-11.46	0	6	0	418.456	4	COc1ccc(cc1)c2nc3c4c(c(oc4ncn3n2)c5ccccc5)c6ccccc6
ZINC06073489	5.24	0.51	-15.41	2	6	0	480.659	10	C1Cc2c(sc(c2C#N)NC(=O)CCCCCCCC(=O)Nc3c(c4c(s3)CCC4)C#N)C1
ZINC08440159	8.15	3.8	-52.33	0	4	-1	561.567	3	c1cc(ccc1C#Cc2ccc(cc2)F)c3c(nc4cc(ccc4n3)C(=O)[O-])c5ccc(cc5)C#Cc6ccc(cc6)F
ZINC08440158	5.59	-0.85	-15.97	0	7	0	472.469	5	C[C@@]12[C@H]([C@@H](c3ccccc3O1)n4c(=O)/c(=C\c5ccc(cc5)OC(F)F)/sc4=N2)C(=O)OC
ZINC08440157	5.71	13.96	-22.46	2	7	0	551.565	7	CC1=C([C@H](C2=C(N1)CC(CC2=O)(C)C)c3ccc(o3)COc4cccc(c4)C(F)(F)F)C(=O)Nc5ccccn5
ZINC08440157	5.85	2.5	-17.41	1	7	0	551.565	7	CC1(CC2=NC(=C)C([C@H]([C@H]2C(=O)C1)c3ccc(o3)COc4cccc(c4)C(F)(F)F)C(=O)Nc5ccccn5)C
ZINC08440157	5.85	1.49	-19.07	1	7	0	551.565	7	CC1(CC2=NC(=C)C([C@H](C2C(=O)C1)c3ccc(o3)COc4cccc(c4)C(F)(F)F)C(=O)Nc5ccccn5)C
ZINC08440156	5.59	-0.23	-16.01	0	7	0	472.469	5	C[C@@]12[C@@H]([C@@H](c3ccccc3O1)n4c(=O)/c(=C\c5ccc(cc5)OC(F)F)/sc4=N2)C(=O)OC
ZINC08440155	6.34	0.18	-5.62	2	2	0	446.553	2	C[C@]12CC[C@H](CC1=CC[C@@H]3[C@@H]2CC[C@@]4([C@@H]3C/C(=C\c5ccc(cc5)C(F)(F)F)/[C@@H]4O)C)O
ZINC08440154	5.99	1.65	-14.26	1	6	0	467.308	4	Cc1c(c(no1)c2c(cccc2Cl)Cl)C(=O)Nc3cc4c5ccccc5oc4cc3OC

Supplementary Table S2:
Detailed table of XP Docking using Schrodinger's Glide module

ZINC_ID	xLogP	ApolarDesolvation	PolarDesolvation	H Donors	H Acceptors	Charge	MolecularWeight	RotatableBonds	SMILES
ZINC08440153	6.34	0.37	-5.64	2	2	0	446.553	2	C[C@@]12CC[C@H](CC1=CC[C@@H]3[C@@H]2CC[C@@]4([C@@H]3C/C(=C\c5ccc(cc5)C(F)(F)F)/[C@@H]4O)C)O
ZINC08440152	5.36	1.88	-13.23	1	6	0	432.863	4	Cc1c(c(no1)c2ccccc2Cl)C(=O)Nc3cc4c5ccccc5oc4cc3OC
ZINC08440151	6.76	-0.91	-11.88	3	7	0	523.614	8	CCOC(=O)c1ccc(cc1)NC(=O)c2c(c3c(cc(nc3s2)c4ccccc4)c5ccc(cc5)OC)N
ZINC08440150	6.34	0.02	-5.99	2	2	0	446.553	2	C[C@]12CC[C@@H](CC1=CC[C@@H]3[C@@H]2CC[C@@]4([C@@H]3C/C(=C\c5ccc(cc5)C(F)(F)F)/[C@@H]4O)C)O
ZINC08440149	5.67	1.17	-15.65	1	7	0	551.565	7	CC1=NC2=C([C@H](C1C(=O)Nc3ccccn3)c4ccc(o4)COc5cccc(c5)C(F)(F)F)C(=O)CC(C2)(C)C
ZINC08440149	5.85	2.41	-22.13	1	7	0	551.565	7	CC1(CC2=NC(=C)C([C@@H]([C@@H]2C(=O)C1)c3ccc(o3)COc4cccc(c4)C(F)(F)F)C(=O)Nc5ccccn5)C
ZINC08440149	5.85	0.92	-17.13	1	7	0	551.565	7	CC1(CC2=NC(=C)C([C@@H](C2C(=O)C1)c3ccc(o3)COc4cccc(c4)C(F)(F)F)C(=O)Nc5ccccn5)C
ZINC08440148	7.5	-2.3	-8.62	3	5	0	520.441	5	COc1ccc(cc1)c2cc(nc3c2c(c(s3)C(=O)Nc4cc(cc(c4)Cl)Cl)N)c5ccccc5
ZINC00845371	6.26	1.46	-15.6	1	5	0	465.578	7	Cc1ccc(cc1)c2cc(c(c(n2)SCC(=O)Nc3ccccc3)C#N)c4ccc(cc4)OC
ZINC08440147	6.44	2.58	-18.17	1	7	0	523.614	9	Cc1ccc(cc1)c2cc(c(c(n2)SCC(=O)Nc3ccc(cc3)C(=O)OC)C#N)c4ccc(cc4)OC
ZINC08440146	6.34	0.04	-6.29	2	2	0	446.553	2	C[C@@]12CC[C@@H](CC1=CC[C@@H]3[C@@H]2CC[C@@]4([C@@H]3C/C(=C\c5ccc(cc5)C(F)(F)F)/[C@@H]4O)C)O
ZINC08440145	7.71	-3.28	-7.32	3	4	0	579.317	4	c1ccc(cc1)c2cc(nc3c2c(c(s3)C(=O)Nc4ccc(cc4Br)Br)N)c5ccccc5
ZINC02062041	8.22	1.45	-16.85	0	3	0	449.509	3	c1ccc(cc1)Oc2cccc(c2)c3c4c(c5c6ccccc6ccc5n3)-c7ccccc7C4=O
ZINC08440144	6.34	-0.33	-17.85	1	9	0	622.699	11	CCOc1ccc2c(c1)sc(n2)N3[C@H](C(=C(C3=O)O)C(=O)c4ccc(cc4)OC)c5ccc(c(c5)OC)OCc6ccccc6
ZINC08440144	5.75	-0.44	-22.26	0	9	0	622.699	11	CCOc1ccc2c(c1)sc(n2)N3[C@H]([C@H](C(=O)C3=O)C(=O)c4ccc(cc4)OC)c5ccc(c(c5)OC)OCc6ccccc6
ZINC08440143	3.86	-0.19	-62.28	0	6	-1	496.008	4	Cc1cc2c3c(c1)[C@H]4[C@H](C[C@@H]([C@@H]4Cl)Sc5ccccc5[N+](=O)[O-])[C@@H](N3C[C@@H]6[C@@H]2C=CC6)C(=O)[O-]
ZINC08440142	6.34	-0.41	-17.78	1	9	0	622.699	11	CCOc1ccc2c(c1)sc(n2)N3[C@@H](C(=C(C3=O)O)C(=O)c4ccc(cc4)OC)c5ccc(c(c5)OC)OCc6ccccc6
ZINC08440142	5.75	-0.49	-22.07	0	9	0	622.699	11	CCOc1ccc2c(c1)sc(n2)N3[C@@H]([C@@H](C(=O)C3=O)C(=O)c4ccc(cc4)OC)c5ccc(c(c5)OC)OCc6ccccc6
ZINC06194705	3.86	0.18	-57.67	0	6	-1	496.008	4	Cc1cc2c3c(c1)[C@@H]4[C@H](C[C@@H]([C@@H]4Cl)Sc5ccccc5[N+](=O)[O-])[C@@H](N3C[C@@H]6[C@@H]2C=CC6)C(=O)[O-]
ZINC05928972	6.42	14.37	-59.57	0	5	-1	467.57	5	CCc1ccc(cc1)[C@H]2C(=C(C(=O)N2c3nc4ccc(cc4s3)C)[O-])C(=O)c5ccc(cc5)C
ZINC05928972	6.87	13.29	-25.92	1	5	0	468.578	4	CCc1ccc(cc1)[C@H]2C(=C(c3ccc(cc3)C)O)C(=O)C(=O)N2c4nc5ccc(cc5s4)C
ZINC05928972	5.84	14.29	-15.32	0	5	0	468.578	5	CCc1ccc(cc1)[C@H]2[C@@H](C(=O)C(=O)N2c3nc4ccc(cc4s3)C)C(=O)c5ccc(cc5)C
ZINC05928795	6.42	14.37	-59.44	0	5	-1	467.57	5	CCc1ccc(cc1)[C@@H]2C(=C(C(=O)N2c3nc4ccc(cc4s3)C)[O-])C(=O)c5ccc(cc5)C
ZINC08440141	3.86	-0.01	-56.41	0	6	-1	496.008	4	Cc1cc2c3c(c1)[C@H]4[C@@H](C[C@@H]([C@@H]4Cl)Sc5ccccc5[N+](=O)[O-])[C@@H](N3C[C@@H]6[C@@H]2C=CC6)C(=O)[O-]
ZINC08440140	4.53	11.22	-16.26	2	5	0	472.408	3	CC1=C([C@H](C2=C(N1)CC(CC2=O)(C)C)c3ccc(s3)Br)C(=O)Nc4ccccn4
ZINC08440140	4.67	9.6	-13.52	1	5	0	472.408	3	CC1(CC2=NC(=C)C([C@H](C2C(=O)C1)c3ccc(s3)Br)C(=O)Nc4ccccn4)C
ZINC08440140	4.67	10.49	-16.35	1	5	0	472.408	3	CC1(CC2=NC(=C)C([C@H]([C@H]2C(=O)C1)c3ccc(s3)Br)C(=O)Nc4ccccn4)C
ZINC06194703	3.86	-0.03	-53.23	0	6	-1	496.008	4	Cc1cc2c3c(c1)[C@@H]4[C@@H](C[C@@H]([C@@H]4Cl)Sc5ccccc5[N+](=O)[O-])[C@@H](N3C[C@@H]6[C@@H]2C=CC6)C(=O)[O-]
ZINC08440139	3.33	8.98	-17.3	2	6	0	458.643	8	C[C@]12CC[C@](C1(C)C)(CC2=O)C(=O)NCCCCCNC(=O)[C@@]34CC[C@@](C3(C)C)(C(=O)C4)C
ZINC06194074	6.4	0.38	-18.79	1	7	0	574.884	5	Cc1cc(cc2c1N[C@H]([C@H]3[C@@H]2[C@@H]([C@@H](C3)Sc4ccccc4[N+](=O)[O-])Cl)c5ccc(cc5)Br)[N+](=O)[O-]
ZINC06194059	6.4	0.74	-15.11	1	7	0	574.884	5	Cc1cc(cc2c1N[C@@H]([C@H]3[C@@H]2[C@@H]([C@@H](C3)Sc4ccccc4[N+](=O)[O-])Cl)c5ccc(cc5)Br)[N+](=O)[O-]
ZINC06194066	6.4	0.38	-21.61	1	7	0	574.884	5	Cc1cc(cc2c1N[C@H]([C@@H]3[C@@H]2[C@@H]([C@@H](C3)Sc4ccccc4[N+](=O)[O-])Cl)c5ccc(cc5)Br)[N+](=O)[O-]
ZINC08440138	3.33	8.98	-17.37	2	6	0	458.643	8	C[C@]12CC[C@](C1(C)C)(CC2=O)C(=O)NCCCCCNC(=O)[C@]34CC[C@](C3(C)C)(C(=O)C4)C
ZINC06194053	6.4	0.51	-16.87	1	7	0	574.884	5	Cc1cc(cc2c1N[C@@H]([C@@H]3[C@@H]2[C@@H]([C@@H](C3)Sc4ccccc4[N+](=O)[O-])Cl)c5ccc(cc5)Br)[N+](=O)[O-]
ZINC08440137	4.48	10.7	-17.95	1	5	0	472.408	3	CC1=C([C@@H]([C@@H]2C(=N1)CC(CC2=O)(C)C)c3ccc(s3)Br)C(=O)Nc4ccccn4
ZINC08440136	5.15	12.38	-15.74	2	8	0	600.756	7	C[C@]12CC[C@](C1(C)C)(CC2=O)C(=O)Nc3ccc(cc3OC)c4ccc(c(c4)OC)NC(=O)[C@@]56CC[C@@](C5(C)C)(C(=O)C6)C
ZINC00842712	3.33	0.27	-29.71	3	8	0	459.502	6	Cc1ccc(cc1)NC(=O)COc2ccc(cc2OC)[C@H]3C(=C(OC4=C3C(=O)CCC4)N)C#N
ZINC00842713	3.33	0.27	-29.84	3	8	0	459.502	6	Cc1ccc(cc1)NC(=O)COc2ccc(cc2OC)[C@@H]3C(=C(OC4=C3C(=O)CCC4)N)C#N
ZINC08440135	5.15	12.39	-15.61	2	8	0	600.756	7	C[C@]12CC[C@](C1(C)C)(CC2=O)C(=O)Nc3ccc(cc3OC)c4ccc(c(c4)OC)NC(=O)[C@]56CC[C@](C5(C)C)(C(=O)C6)C
ZINC08440134	4.66	1.1	-11.83	0	6	0	438.318	8	CCOC(=O)C1[C@@H](C(=C(N=C1C)C)C(=O)OCC)c2cc(ccc2Br)OC
ZINC08440134	4.66	0.77	-10.72	0	6	0	438.318	8	CCOC(=O)C1[C@H](C(=C(N=C1C)C)C(=O)OCC)c2cc(ccc2Br)OC
ZINC08440134	4.84	-0.27	-9.3	0	6	0	438.318	8	CCOC(=O)C1[C@H](C(C(=C)N=C1C)C(=O)OCC)c2cc(ccc2Br)OC
ZINC00702388	4.59	11.1	-10.37	0	4	0	481.184	3	C[C@@]12CC[C@@](C1(C)C)(CC2=O)C(=O)Oc3c(cc(c4c3nccc4)Br)Br
ZINC02062020	1.73	-7.19	-21.23	6	8	0	450.586	9	Cc1c(sc(c1C(=O)N)NC(=O)CCCCC(=O)Nc2c(c(c(s2)C)C)C(=O)N)C
ZINC08440133	2.61	11.68	-18.18	2	10	0	604.7	7	C[C@]12CC[C@](C1(C)C)(OC2=O)C(=O)Nc3ccc(cc3OC)c4ccc(c(c4)OC)NC(=O)[C@@]56CC[C@@](C5(C)C)(C(=O)O6)C
ZINC00298314	3.31	8.36	-58.54	1	4	-1	313.16	3	c1cc(c(cc1Cl)NC(=O)[C@@H]2CC=CC[C@H]2C(=O)[O-])Cl
ZINC00854709	4.94	2.34	-13.54	1	6	0	419.264	6	CCOC(=O)c1ccc(cc1)NC(=O)c2c(onc2c3c(cccc3Cl)Cl)C
ZINC08440132	5.11	13.12	-19.69	2	6	0	465.553	5	CC1=C([C@H](C2=C(N1)C[C@H](CC2=O)c3ccccc3)c4cccc(c4)OC)C(=O)Nc5ccccn5
ZINC08440132	5.25	12.26	-17.44	1	6	0	465.553	5	COc1cccc(c1)[C@H]2[C@H]3C(=NC(=C)C2C(=O)Nc4ccccn4)C[C@H](CC3=O)c5ccccc5
ZINC08440132	5.25	11.34	-14.13	1	6	0	465.553	5	COc1cccc(c1)[C@H]2C3C(=NC(=C)C2C(=O)Nc4ccccn4)C[C@H](CC3=O)c5ccccc5
ZINC08440132	5.25	12.23	-12.56	1	6	0	465.553	5	COc1cccc(c1)[C@H]2[C@@H]3C(=NC(=C)C2C(=O)Nc4ccccn4)C[C@H](CC3=O)c5ccccc5
ZINC08440131	2.61	11.69	-17.85	2	10	0	604.7	7	C[C@]12CC[C@](C1(C)C)(OC2=O)C(=O)Nc3ccc(cc3OC)c4ccc(c(c4)OC)NC(=O)[C@]56CC[C@](C5(C)C)(C(=O)O6)C
ZINC00142373	3.14	6.62	-10.54	0	3	0	257.314	2	c1ccc(cc1)S(=O)(=O)n2ccc3c2cccc3
ZINC02062009	6.86	0.95	-10.14	0	4	0	424.496	6	c1ccc(cc1)c2ccc(cc2)O[C@@H]3[C@H](OCCO3)Oc4ccc(cc4)c5ccccc5
ZINC02062010	6.86	1.06	-9.99	0	4	0	424.496	6	c1ccc(cc1)c2ccc(cc2)O[C@H]3[C@@H](OCCO3)Oc4ccc(cc4)c5ccccc5
ZINC00828732	4.9	-2.66	-10.42	2	4	0	450.332	2	COc1cc(cc(c1O)Br)[C@@H]2C3=C(CCCC3=O)c4c5ccccc5ccc4N2
ZINC08440129	7.55	-5.21	-19.33	0	7	0	562.798	16	CCCCN(CCCC)S(=O)(=O)c1ccc-2c(c1)C(=O)c3c2ccc(c3)S(=O)(=O)N(CCCC)CCCC
ZINC08440128	5.11	13.12	-19.46	2	6	0	465.553	5	CC1=C([C@@H](C2=C(N1)C[C@H](CC2=O)c3ccccc3)c4cccc(c4)OC)C(=O)Nc5ccccn5
ZINC08440128	5.25	11.05	-19.12	1	6	0	465.553	5	COc1cccc(c1)[C@@H]2C3C(=NC(=C)C2C(=O)Nc4ccccn4)C[C@H](CC3=O)c5ccccc5
ZINC08440128	5.25	11.87	-33.47	1	6	0	465.553	5	COc1cccc(c1)[C@@H]2[C@H]3C(=NC(=C)C2C(=O)Nc4ccccn4)C[C@H](CC3=O)c5ccccc5
ZINC01306294	3.68	12.89	-45.77	0	5	-1	432.5	3	Cc1ccc(cc1)n2c(cc(c2C)/C=C/3\C(=NC(=S)N(C3=O)c4ccc(cc4)F)[O-])C

Supplementary Table S2:
Detailed table of XP Docking using Schrodinger's Glide module

ZINC_ID	xLogP	ApolarDesolvation	PolarDesolvation	H Donors	H Acceptors	Charge	MolecularWeight	RotatableBonds	SMILES
ZINC00849729	4.6	0.95	-11.73	1	5	0	461.301	3	c1cc(ccc1N2C(=O)/C(=C/c3ccc(o3)c4ccc(c(c4)Cl)Cl)/C(=O)NC2=S)F
ZINC02061992	6.84	2.36	-9.86	1	5	0	479.289	4	Cc1ccc(cc1C)NC(=O)c2cc3nc(cc(n3n2)C(F)(F)F)c4ccc(c(c4)Cl)Cl
ZINC08440126	6.03	14.72	-59.6	0	5	-1	468.573	10	CCCOc1ccc(cc1)C(=O)C2=C(C(=O)N([C@H]2c3ccc(cc3)CC)CCc4ccccc4)[O-]
ZINC08440126	6.48	0.95	-27.09	1	5	0	469.581	9	CCCOc1ccc(cc1)C(=C2[C@@H](N(C(=O)C2=O)CCc3ccccc3)c4ccc(cc4)CC)O
ZINC08440126	5.45	1.17	-19.22	0	5	0	469.581	10	CCCOc1ccc(cc1)C(=O)[C@H]2[C@@H](N(C(=O)C2=O)CCc3ccccc3)c4ccc(cc4)CC
ZINC08440125	5.35	-1.36	-11.78	1	7	0	528.399	6	CC1=NC2=C([C@@H](C1C(=O)OC)c3cc(c(c(c3)Br)O)OC)C(=O)C[C@@H](C2)c4ccc(cc4)OC
ZINC08440125	5.54	-1.12	-12.89	1	7	0	528.399	6	COc1ccc(cc1)[C@@H]2CC3=NC(=C)C([C@H](C3C(=O)C2)c4cc(c(c(c4)Br)O)OC)C(=O)OC
ZINC08440125	5.54	-0.14	-12.15	1	7	0	528.399	6	COc1ccc(cc1)[C@@H]2CC3=NC(=C)C([C@H]([C@H]3C(=O)C2)c4cc(c(c(c4)Br)O)OC)C(=O)OC
ZINC08440125	5.35	-1.24	-14.47	1	7	0	528.399	6	CC1=C([C@H]([C@H]2C(=N1)C[C@H](CC2=O)c3ccc(cc3)OC)c4cc(c(c(c4)Br)O)OC)C(=O)OC
ZINC08440124	5.35	-1.25	-13.75	1	7	0	528.399	6	CC1=NC2=C([C@H](C1C(=O)OC)c3cc(c(c(c3)Br)O)OC)C(=O)C[C@@H](C2)c4ccc(cc4)OC
ZINC08440124	5.54	-1.12	-13.54	1	7	0	528.399	6	COc1ccc(cc1)[C@@H]2CC3=NC(=C)C([C@@H](C3C(=O)C2)c4cc(c(c(c4)Br)O)OC)C(=O)OC
ZINC08440124	5.54	0.26	-25.83	1	7	0	528.399	6	COc1ccc(cc1)[C@@H]2CC3=NC(=C)C([C@@H]([C@@H]3C(=O)C2)c4cc(c(c(c4)Br)O)OC)C(=O)OC
ZINC08440124	5.35	-1.39	-15.02	1	7	0	528.399	6	CC1=C([C@@H]([C@H]2C(=N1)C[C@H](CC2=O)c3ccc(cc3)OC)c4cc(c(c(c4)Br)O)OC)C(=O)OC
ZINC08440123	5.11	13.11	-19.65	2	6	0	465.553	5	CC1=C([C@H](C2=C(N1)C[C@@H](CC2=O)c3ccccc3)c4cccc(c4)OC)C(=O)Nc5ccccn5
ZINC08440123	5.25	11.23	-20	1	6	0	465.553	5	COc1cccc(c1)[C@H]2C3C(=NC(=C)C2C(=O)Nc4ccccn4)C[C@@H](CC3=O)c5ccccc5
ZINC08440123	5.25	12.17	-22.54	1	6	0	465.553	5	COc1cccc(c1)[C@H]2[C@@H]3C(=NC(=C)C2C(=O)Nc4ccccn4)C[C@@H](CC3=O)c5ccccc5
ZINC08440122	5.35	-1.37	-10.98	1	7	0	528.399	6	CC1=NC2=C([C@@H](C1C(=O)OC)c3cc(c(c(c3)Br)O)OC)C(=O)C[C@H](C2)c4ccc(cc4)OC
ZINC08440122	5.54	0.1	-16.62	1	7	0	528.399	6	COc1ccc(cc1)[C@H]2CC3=NC(=C)C([C@H]([C@H]3C(=O)C2)c4cc(c(c(c4)Br)O)OC)C(=O)OC
ZINC08440122	5.35	-1.35	-15.06	1	7	0	528.399	6	CC1=C([C@H]([C@@H]2C(=N1)C[C@@H](CC2=O)c3ccc(cc3)OC)c4cc(c(c(c4)Br)O)OC)C(=O)OC
ZINC08440121	5.35	-1.25	-13.04	1	7	0	528.399	6	CC1=NC2=C([C@H](C1C(=O)OC)c3cc(c(c(c3)Br)O)OC)C(=O)C[C@H](C2)c4ccc(cc4)OC
ZINC08440121	5.54	-1.21	-13.46	1	7	0	528.399	6	COc1ccc(cc1)[C@H]2CC3=NC(=C)C([C@@H](C3C(=O)C2)c4cc(c(c(c4)Br)O)OC)C(=O)OC
ZINC08440121	5.54	0	-13.58	1	7	0	528.399	6	COc1ccc(cc1)[C@H]2CC3=NC(=C)C([C@@H]([C@@H]3C(=O)C2)c4cc(c(c(c4)Br)O)OC)C(=O)OC
ZINC08440121	5.35	-1.47	-15.08	1	7	0	528.399	6	CC1=C([C@@H]([C@H]2C(=N1)C[C@@H](CC2=O)c3ccc(cc3)OC)c4cc(c(c(c4)Br)O)OC)C(=O)OC
ZINC08440120	6.03	14.33	-59.81	0	5	-1	468.573	10	CCCOc1ccc(cc1)C(=O)C2=C(C(=O)N([C@@H]2c3ccc(cc3)CC)CCc4ccccc4)[O-]
ZINC08440120	6.48	0.7	-26.92	1	5	0	469.581	9	CCCOc1ccc(cc1)C(=C2[C@H](N(C(=O)C2=O)CCc3ccccc3)c4ccc(cc4)CC)O
ZINC08440120	5.45	0.99	-20.68	0	5	0	469.581	10	CCCOc1ccc(cc1)C(=O)[C@H]2[C@H](N(C(=O)C2=O)CCc3ccccc3)c4ccc(cc4)CC
ZINC08440119	6.19	11.9	-19.11	2	7	0	590.898	9	CCOc1cc(cc(c1O)Br)[C@H]2C3=C(C[C@H](CC3=O)c4ccc(cc4)Cl)NC(=C2C(=O)OCCOC)C
ZINC08440119	6.15	-2.17	-11.28	1	7	0	590.898	9	CCOc1cc(cc(c1O)Br)[C@@H]2C(C(=NC3=C2C(=O)C[C@@H](C3)c4ccc(cc4)Cl)C)C(=O)OCCOC
ZINC08440118	6.19	11.84	-19.33	2	7	0	590.898	9	CCOc1cc(cc(c1O)Br)[C@@H]2C3=C(C[C@H](CC3=O)c4ccc(cc4)Cl)NC(=C2C(=O)OCCOC)C
ZINC08440118	6.15	-2.03	-12.21	1	7	0	590.898	9	CCOc1cc(cc(c1O)Br)[C@H]2C(C(=NC3=C2C(=O)C[C@@H](C3)c4ccc(cc4)Cl)C)C(=O)OCCOC
ZINC08440117	6.13	14.41	-59.72	0	5	-1	468.573	9	CCOc1ccc(cc1)C(=O)C2=C(C(=O)N([C@H]2c3ccc(cc3)C(C)C)CCc4ccccc4)[O-]
ZINC08440117	6.57	1.19	-27.3	1	5	0	469.581	8	CCOc1ccc(cc1)C(=C2[C@@H](N(C(=O)C2=O)CCc3ccccc3)c4ccc(cc4)C(C)C)O
ZINC08440117	5.54	1.41	-19.46	0	5	0	469.581	9	CCOc1ccc(cc1)C(=O)[C@H]2[C@@H](N(C(=O)C2=O)CCc3ccccc3)c4ccc(cc4)C(C)C
ZINC08440116	6.19	11.89	-18.95	2	7	0	590.898	9	CCOc1cc(cc(c1O)Br)[C@H]2C3=C(C[C@@H](CC3=O)c4ccc(cc4)Cl)NC(=C2C(=O)OCCOC)C
ZINC08440116	6.31	-3.47	-9.9	1	7	0	590.898	9	CCOc1cc(cc(c1O)Br)[C@H]2C3C(=O)C[C@H](C=C3N=C(C2C(=O)OCCOC)C)c4ccc(cc4)Cl
ZINC08440115	5.11	13.11	-19.45	2	6	0	465.553	5	CC1=C([C@@H](C2=C(N1)C[C@@H](CC2=O)c3ccccc3)c4cccc(c4)OC)C(=O)Nc5ccccn5
ZINC08440115	5.25	11.39	-13.94	1	6	0	465.553	5	COc1cccc(c1)[C@@H]2C3C(=NC(=C)C2C(=O)Nc4ccccn4)C[C@@H](CC3=O)c5ccccc5
ZINC08440115	5.25	12.27	-14.53	1	6	0	465.553	5	COc1cccc(c1)[C@@H]2[C@H]3C(=NC(=C)C2C(=O)Nc4ccccn4)C[C@@H](CC3=O)c5ccccc5
ZINC08440114	6.19	11.84	-19.44	2	7	0	590.898	9	CCOc1cc(cc(c1O)Br)[C@@H]2C3=C(C[C@@H](CC3=O)c4ccc(cc4)Cl)NC(=C2C(=O)OCCOC)C
ZINC08440114	6.15	-2.06	-11.97	1	7	0	590.898	9	CCOc1cc(cc(c1O)Br)[C@H]2C(C(=NC3=C2C(=O)C[C@H](C3)c4ccc(cc4)Cl)C)C(=O)OCCOC
ZINC08440113	6.13	14.02	-60.24	0	5	-1	468.573	9	CCOc1ccc(cc1)C(=O)C2=C(C(=O)N([C@@H]2c3ccc(cc3)C(C)C)CCc4ccccc4)[O-]
ZINC08440113	6.57	0.94	-27.16	1	5	0	469.581	8	CCOc1ccc(cc1)C(=C2[C@H](N(C(=O)C2=O)CCc3ccccc3)c4ccc(cc4)C(C)C)O
ZINC08440113	5.54	1.24	-20.88	0	5	0	469.581	9	CCOc1ccc(cc1)C(=O)[C@H]2[C@H](N(C(=O)C2=O)CCc3ccccc3)c4ccc(cc4)C(C)C
ZINC02061975	5.99	0.65	-16.1	1	6	0	454.917	6	c1ccc(cc1)COc2cccc(c2)C(=O)Nc3ccc4c(c3)nn(n4)c5cccc(c5)Cl
ZINC08440108	5.86	1.02	-17.57	1	7	0	563.65	12	CCOc1ccc(cc1)C(=O)C2=C(C(=O)N([C@H]2c3ccc(c(c3)OC)OCc4ccccc4)CCc5ccccc5)O
ZINC08440108	5.27	0.87	-23.4	0	7	0	563.65	12	CCOc1ccc(cc1)C(=O)[C@H]2[C@@H](N(C(=O)C2=O)CCc3ccccc3)c4ccc(c(c4)OC)OCc5ccccc5
ZINC02061964	6.82	-1.32	-13.62	1	5	0	454.913	6	c1ccc(cc1)COc2cccc(c2)C(=O)Nc3ccc4c(c3)nc(o4)c5ccc(cc5)Cl
ZINC08440106	5.86	1.07	-17.49	1	7	0	563.65	12	CCOc1ccc(cc1)C(=O)C2=C(C(=O)N([C@@H]2c3ccc(c(c3)OC)OCc4ccccc4)CCc5ccccc5)O
ZINC08440106	5.27	0.96	-25.12	0	7	0	563.65	12	CCOc1ccc(cc1)C(=O)[C@H]2[C@H](N(C(=O)C2=O)CCc3ccccc3)c4ccc(c(c4)OC)OCc5ccccc5
ZINC08440105	6.97	-0.52	-17.74	1	5	0	448.522	6	Cc1cc(c2c(c1)nc(o2)c3ccc(cc3)NC(=O)c4cccc(c4)OCc5ccccc5)C
ZINC02061963	6.14	-1.16	-22.07	1	5	0	420.468	6	c1ccc(cc1)COc2cccc(c2)C(=O)Nc3cccc(c3)c4nc5ccccc5o4
ZINC02061962	7.53	-0.73	-18.28	1	5	0	428.532	10	CCCCCCCOc1ccc(cc1)C(=O)Nc2cccc(c2)c3nc4ccccc4o3
ZINC08440104	6.27	0.73	-15.18	1	6	0	533.624	11	CCOc1ccc(cc1)C(=O)C2=C(C(=O)N([C@H]2c3ccc(cc3)OCc4ccccc4)CCc5ccccc5)O
ZINC08440104	5.68	0.58	-21.25	0	6	0	533.624	11	CCOc1ccc(cc1)C(=O)[C@H]2[C@@H](N(C(=O)C2=O)CCc3ccccc3)c4ccc(cc4)OCc5ccccc5
ZINC00441990	1.01	0.02	-51.41	1	5	1	312.393	4	COc1ccc(cc1)C[NH+]2CCN(CC2)C(=O)c3cccnc3
ZINC08440103	5.76	12.75	-21.23	2	5	0	461.562	3	CC1=C([C@H](C2=C(N1)CC(CC2=O)(C)C)c3cccc(c3)F)C(=O)Nc4nc5ccccc5s4
ZINC08440103	5.9	-1.87	-12.96	1	5	0	461.562	3	CC1(CC2=NC(=C)C([C@H](C2C(=O)C1)c3cccc(c3)F)C(=O)Nc4nc5ccccc5s4)C
ZINC08440103	5.9	-0.46	-18.43	1	5	0	461.562	3	CC1(CC2=NC(=C)C([C@H]([C@H]2C(=O)C1)c3cccc(c3)F)C(=O)Nc4nc5ccccc5s4)C
ZINC08440102	7.16	-1.68	-11.09	1	6	0	523.77	5	COc1ccc(cc1Br)c2nc3cc(ccc3o2)NC(=O)c4ccc(o4)c5ccc(cc5)Cl
ZINC08440101	6.27	0.7	-15.13	1	6	0	533.624	11	CCOc1ccc(cc1)C(=O)C2=C(C(=O)N([C@@H]2c3ccc(cc3)OCc4ccccc4)CCc5ccccc5)O
ZINC08440101	5.68	0.62	-22.99	0	6	0	533.624	11	CCOc1ccc(cc1)C(=O)[C@H]2[C@H](N(C(=O)C2=O)CCc3ccccc3)c4ccc(cc4)OCc5ccccc5

Supplementary Table S2:
Detailed table of XP Docking using Schrodinger's Glide module

ZINC_ID	xLogP	ApolarDesolvation	PolarDesolvation	H Donors	H Acceptors	Charge	MolecularWeight	RotatableBonds	SMILES
ZINC02061961	7.75	1.04	-11.88	0	4	0	468.372	6	CCOc1ccc(cc1Br)C(=O)Oc2ccc(cc2)c3nc4ccc(cc4s3)C
ZINC02061959	7.64	0.84	-9.58	0	3	0	471.319	4	Cc1ccc2c(c1)sc(n2)c3ccc(cc3)OC(=O)c4cccc(c4)I
ZINC02061958	7	-2.96	-10.9	1	5	0	583.607	4	COc1ccc(cc1Br)c2nc3cc(ccc3o2)NC(=O)c4cc(ccc4Cl)I
ZINC08440100	6.11	14.36	-59.76	0	6	-1	498.599	12	CCCCOc1ccc(cc1)[C@H]2C(=C(C(=O)N2CCc3ccccc3)[O-])C(=O)c4ccc(cc4)OCC
ZINC08440100	6.55	0.64	-27.7	1	6	0	499.607	11	CCCCOc1ccc(cc1)[C@H]2C(=C(c3ccc(cc3)OCC)O)C(=O)C(=O)N2CCc4ccccc4
ZINC08440100	5.53	0.86	-20.48	0	6	0	499.607	12	CCCCOc1ccc(cc1)[C@H]2[C@@H](C(=O)C(=O)N2CCc3ccccc3)C(=O)c4ccc(cc4)OCC
ZINC00659045	1.75	-0.77	-43.38	1	5	1	391.289	4	COc1ccc(cc1C[NH+]2CCN(CC2)C(=O)c3cccnc3)Br
ZINC02061955	6.55	1.02	-13.5	0	4	0	454.345	6	Cc1ccc2c(c1)sc(n2)c3ccc(cc3)OC(=O)COc4ccc(cc4)Br
ZINC00659037	6.43	-0.98	-10.74	1	6	0	444.874	5	COc1ccc(cc1)c2nc3cc(ccc3o2)NC(=O)c4ccc(o4)c5ccc(cc5)Cl
ZINC08440099	5.76	12.75	-21.12	2	5	0	461.562	3	CC1=C([C@@H](C2=C(N1)CC(CC2=O)(C)C)c3cccc(c3)F)C(=O)Nc4nc5ccccc5s4
ZINC08440099	5.9	-1.83	-14.85	1	5	0	461.562	3	CC1(CC2=NC(=C)C([C@@H](C2C(=O)C1)c3cccc(c3)F)C(=O)Nc4nc5ccccc5s4)C
ZINC08440099	5.9	-0.94	-16.17	1	5	0	461.562	3	CC1(CC2=NC(=C)C([C@@H]([C@@H]2C(=O)C1)c3cccc(c3)F)C(=O)Nc4nc5ccccc5s4)C
ZINC08440098	6.11	13.93	-60.11	0	6	-1	498.599	12	CCCCOc1ccc(cc1)[C@@H]2C(=C(C(=O)N2CCc3ccccc3)[O-])C(=O)c4ccc(cc4)OCC
ZINC08440098	6.55	0.55	-28.14	1	6	0	499.607	11	CCCCOc1ccc(cc1)[C@@H]2C(=C(c3ccc(cc3)OCC)O)C(=O)C(=O)N2CCc4ccccc4
ZINC08440098	5.53	0.84	-22.26	0	6	0	499.607	12	CCCCOc1ccc(cc1)[C@@H]2[C@@H](C(=O)C(=O)N2CCc3ccccc3)C(=O)c4ccc(cc4)OCC
ZINC00659030	6.13	-1.4	-12.12	1	5	0	451.32	4	Cc1ccc(cc1C)C(=O)Nc2ccc3c(c2)nc(o3)c4ccc(c(c4)Br)OC
ZINC02061954	6.08	-1.69	-14.29	1	6	0	481.346	6	CC(C)Oc1cccc(c1)C(=O)Nc2ccc3c(c2)nc(o3)c4ccc(c(c4)Br)OC
ZINC02061953	7.77	-1.84	-10.32	1	6	0	558.215	5	COc1ccc(cc1Br)c2nc3cc(ccc3o2)NC(=O)c4ccc(o4)c5cc(ccc5Cl)Cl
ZINC08440097	6	-1.85	-14.11	1	6	0	479.33	6	COc1ccc(cc1)/C=C\C(=O)Nc2ccc3c(c2)nc(o3)c4ccc(c(c4)Br)OC
ZINC00702370	3.33	11.14	-38.77	0	6	-1	427.48	8	CCOc1ccc(cc1)C(=O)C2=C(C(=O)N([C@H]2c3ccncc3)CCc4ccccc4)[O-]
ZINC00702370	3.77	-0.7	-27.72	1	6	0	428.488	7	CCOc1ccc(cc1)C(=C2[C@@H](N(C(=O)C2=O)CCc3ccccc3)c4ccncc4)O
ZINC00702370	2.74	-0.47	-21.26	0	6	0	428.488	8	CCOc1ccc(cc1)C(=O)[C@H]2[C@@H](N(C(=O)C2=O)CCc3ccccc3)c4ccncc4
ZINC02061951	6.56	-2.25	-22.79	1	6	0	522.182	6	COc1ccc(cc1Br)c2nc3cc(ccc3o2)NC(=O)COc4ccc(cc4Cl)Cl
ZINC00659023	5.73	-1.68	-15.91	1	6	0	467.319	6	Cc1ccc(cc1)OCC(=O)Nc2ccc3c(c2)nc(o3)c4ccc(c(c4)Br)OC
ZINC08440096	3.33	10.73	-38.66	0	6	-1	427.48	8	CCOc1ccc(cc1)C(=O)C2=C(C(=O)N([C@@H]2c3ccncc3)CCc4ccccc4)[O-]
ZINC08440096	3.77	-0.49	-28.68	1	6	0	428.488	7	CCOc1ccc(cc1)C(=C2[C@H](N(C(=O)C2=O)CCc3ccccc3)c4ccncc4)O
ZINC08440096	2.74	-0.22	-23.64	0	6	0	428.488	8	CCOc1ccc(cc1)C(=O)[C@H]2[C@H](N(C(=O)C2=O)CCc3ccccc3)c4ccncc4
ZINC08440095	4.62	-1.04	-16.44	0	5	0	474.395	6	CCCCOc1cc(c(cc1OC)[C@@H]2C3C(=NC4=C2C(=O)CCC4)CCCC3=O)Br
ZINC08440095	4.78	-2.46	-13.04	0	5	0	474.395	6	CCCCOc1cc(c(cc1OC)[C@H]2C3C(=NC4=CCCC(=O)C24)CCCC3=O)Br
ZINC00659022	5.77	-2.77	-9.05	1	6	0	539.123	4	COc1ccc(cc1Br)c2nc3cc(ccc3o2)NC(=O)c4ccc(o4)I
ZINC02061950	6.72	-2.88	-11.21	1	5	0	536.607	4	COc1ccc(cc1Br)c2nc3cc(ccc3o2)NC(=O)c4cc(ccc4Cl)Br
ZINC00702368	3.95	10.21	-42.48	1	7	-1	472.517	9	CCOc1ccc(cc1)C(=O)C2=C(C(=O)N([C@H]2c3ccc(c(c3)OC)O)CCc4ccccc4)[O-]
ZINC00702368	4.4	-1.48	-29.91	2	7	0	473.525	8	CCOc1ccc(cc1)C(=C2[C@@H](N(C(=O)C2=O)CCc3ccccc3)c4ccc(c(c4)OC)O)O
ZINC00702368	3.37	-1.24	-22.16	1	7	0	473.525	9	CCOc1ccc(cc1)C(=O)[C@H]2[C@@H](N(C(=O)C2=O)CCc3ccccc3)c4ccc(c(c4)OC)O
ZINC02061949	6.48	-2.64	-14.61	1	6	0	546.215	6	CCOc1ccc(cc1Br)C(=O)Nc2ccc3c(c2)nc(o3)c4ccc(c(c4)Br)OC
ZINC08440094	4.93	-0.99	-6.23	1	3	0	479.055	3	COc1ccccc1NC(=O)c2cc(ccc2I)I
ZINC02061946	7.34	2.46	-12.24	0	6	0	477.582	10	CCOc1cc(cc(c1OCC)OCC)C(=O)Oc2ccc(cc2)c3nc4ccc(cc4s3)C
ZINC02061944	7.53	1.35	-10.25	0	3	0	414.313	4	Cc1ccc2c(c1)sc(n2)c3ccc(cc3)OC(=O)c4ccc(cc4Cl)Cl
ZINC00702369	3.95	10.21	-42.44	1	7	-1	472.517	9	CCOc1ccc(cc1)C(=O)C2=C(C(=O)N([C@@H]2c3ccc(c(c3)OC)O)CCc4ccccc4)[O-]
ZINC00702369	4.4	-1.42	-29.82	2	7	0	473.525	8	CCOc1ccc(cc1)C(=C2[C@H](N(C(=O)C2=O)CCc3ccccc3)c4ccc(c(c4)OC)O)O
ZINC00702369	3.37	-1.19	-23.19	1	7	0	473.525	9	CCOc1ccc(cc1)C(=O)[C@@H]2[C@H](N(C(=O)C2=O)CCc3ccccc3)c4ccc(c(c4)OC)O
ZINC02061941	7.28	0.86	-10.97	0	3	0	471.319	4	Cc1ccc2c(c1)sc(n2)c3ccc(cc3)OC(=O)c4ccccc4I
ZINC08440092	6.32	14.81	-59.85	0	5	-1	482.6	9	CCOc1ccc(cc1)C(=O)C2=C(C(=O)N([C@H]2c3ccc(cc3)C(C)(C)C)CCc4ccccc4)[O-]
ZINC08440092	6.77	1.31	-27.08	1	5	0	483.608	8	CCOc1ccc(cc1)C(=C2[C@@H](N(C(=O)C2=O)CCc3ccccc3)c4ccc(cc4)C(C)(C)C)O
ZINC08440092	5.74	1.55	-19.13	0	5	0	483.608	9	CCOc1ccc(cc1)C(=O)[C@H]2[C@@H](N(C(=O)C2=O)CCc3ccccc3)c4ccc(cc4)C(C)(C)C
ZINC00659009	3.88	0.03	-16.43	2	7	0	440.978	7	CCOC(=O)c1c2c(sc1NC(=S)NC(=O)c3c(cn(n3)C)Cl)C[C@H](CC2)C
ZINC02779333	3.88	0.03	-16.44	2	7	0	440.978	7	CCOC(=O)c1c2c(sc1NC(=S)NC(=O)c3c(cn(n3)C)Cl)C[C@@H](CC2)C
ZINC08440091	6.01	14.59	-16.72	0	5	0	464.594	5	Cc1ccc(cc1)c2csc(n2)N3C(=O)/C(=C/c4ccc(cc4)N(C)C)/C(=N3)c5ccccc5
ZINC08440089	6.32	14.41	-60.33	0	5	-1	482.6	9	CCOc1ccc(cc1)C(=O)C2=C(C(=O)N([C@@H]2c3ccc(cc3)C(C)(C)C)CCc4ccccc4)[O-]
ZINC08440089	6.77	1.47	-27.47	1	5	0	483.608	8	CCOc1ccc(cc1)C(=C2[C@H](N(C(=O)C2=O)CCc3ccccc3)c4ccc(cc4)C(C)(C)C)O
ZINC08440089	5.74	1.76	-21.29	0	5	0	483.608	9	CCOc1ccc(cc1)C(=O)[C@H]2[C@H](N(C(=O)C2=O)CCc3ccccc3)c4ccc(cc4)C(C)(C)C
ZINC00702366	5.06	13.59	-41.45	0	5	-1	440.519	8	CCOc1ccc(cc1)C(=O)C2=C(C(=O)N([C@H]2c3ccc(cc3)C)CCc4ccccc4)[O-]
ZINC00702366	5.51	0.73	-27.5	1	5	0	441.527	7	CCOc1ccc(cc1)C(=C2[C@@H](N(C(=O)C2=O)CCc3ccccc3)c4ccc(cc4)C)O
ZINC00702366	4.48	1.01	-21.41	0	5	0	441.527	8	CCOc1ccc(cc1)C(=O)[C@@H]2[C@@H](N(C(=O)C2=O)CCc3ccccc3)c4ccc(cc4)C
ZINC08440086	6.36	9.95	-18.39	0	10	0	658.744	16	Cc1cc(c(cc1C2(c3ccccc3C(=O)O2)c4cc(c(cc4C)OC(=O)CCC(=O)OC)C(C)C)C(C)C)OC(=O)CCC(=O)OC
ZINC08440085	3.61	-0.34	-14.54	1	7	0	467.573	4	Cc1ccc(nc1)NC(=O)C2[C@H](C3=C(C[C@@H](CC3=O)c4ccccc4)N=C2C)c5cnn(c5C)C
ZINC08440085	3.79	-1.58	-18	1	7	0	467.573	4	Cc1ccc(nc1)NC(=O)C2[C@H](C3C(=NC2=C)C[C@@H](CC3=O)c4ccccc4)c5cnn(c5C)C
ZINC08440084	4.74	11.59	-12.27	1	8	0	419.828	5	c1ccc(cc1)C\2=NN(C(=O)/C2=N/Nc3ccc(c(c3)[N+](=O)[O-])Cl)c4ccccc4
ZINC08440084	4.74	11.9	-13.08	1	8	0	419.828	5	c1ccc(cc1)C\2=NN(C(=O)/C2=N\Nc3ccc(c(c3)[N+](=O)[O-])Cl)c4ccccc4
ZINC00702367	5.06	13.19	-41	0	5	-1	440.519	8	CCOc1ccc(cc1)C(=O)C2=C(C(=O)N([C@@H]2c3ccc(cc3)C)CCc4ccccc4)[O-]
ZINC00702367	5.51	0.64	-27.78	1	5	0	441.527	7	CCOc1ccc(cc1)C(=C2[C@H](N(C(=O)C2=O)CCc3ccccc3)c4ccc(cc4)C)O

Supplementary Table S2:
Detailed table of XP Docking using Schrodinger's Glide module

ZINC_ID	xLogP	ApolarDesolvation	PolarDesolvation	H Donors	H Acceptors	Charge	MolecularWeight	RotatableBonds	SMILES
ZINC00702367	4.48	0.93	-21.48	0	5	0	441.527	8	CCOc1ccc(cc1)C(=O)[C@H]2[C@H](N(C(=O)C2=O)CCc3ccccc3)c4ccc(cc4)C
ZINC04458647	-2.54	-13.69	-68.23	3	10	0	582.751	12	[H]/N=C(/Cc1[n+](c2ccccc2s1)CCCS(=O)(=O)[O-])\C=C/3\N(c4ccccc4S3)CCCS(=O)(=O)ON
ZINC08440083	7.61	14.64	-11.67	1	6	0	660.189	5	c1ccc(cc1)c2csc(n2)N3C(=O)/C(=N\Nc4c(cc(cc4Br)Br)Br)/C(=N3)c5ccccc5
ZINC08440081	3.87	11.19	-42.88	0	8	-1	502.543	10	COc1ccc(cc1)C(=O)C2=C(C(=O)N([C@H]2c3cc(c(c(c3)OC)OC)OC)CCc4ccccc4)[O-]
ZINC08440081	4.32	1.25	-30.7	1	8	0	503.551	9	COc1ccc(cc1)C(=C2[C@@H](N(C(=O)C2=O)CCc3ccccc3)c4cc(c(c(c4)OC)OC)OC)O
ZINC08440081	3.29	1.46	-24.93	0	8	0	503.551	10	COc1ccc(cc1)C(=O)[C@H]2[C@@H](N(C(=O)C2=O)CCc3ccccc3)c4cc(c(c(c4)OC)OC)OC
ZINC08440080	4.94	3.09	-19.36	0	6	0	508.533	4	c1ccc2c(c1)-c3ccccc3C2(c4ccc(cc4)N5C(=O)C=CC5=O)c6ccc(cc6)N7C(=O)C=CC7=O
ZINC08440078	1.89	-2.94	-19.72	2	7	0	305.363	5	CCOc1ccc(cc1)/N=N/[C@@H]2C(=NN(C2=O)C(=S)N)C
ZINC08440077	3.87	10.75	-40.97	0	8	-1	502.543	10	COc1ccc(cc1)C(=O)C2=C(C(=O)N([C@@H]2c3cc(c(c(c3)OC)OC)OC)CCc4ccccc4)[O-]
ZINC08440077	4.32	1.36	-31.47	1	8	0	503.551	9	COc1ccc(cc1)C(=C2[C@H](N(C(=O)C2=O)CCc3ccccc3)c4cc(c(c(c4)OC)OC)OC)O
ZINC08440077	3.29	1.57	-23.99	0	8	0	503.551	10	COc1ccc(cc1)C(=O)[C@@H]2[C@H](N(C(=O)C2=O)CCc3ccccc3)c4cc(c(c(c4)OC)OC)OC
ZINC08440075	5.22	13.69	-61.22	0	6	-1	483.588	10	CCCOc1ccc(cc1)C(=O)C2=C(C(=O)N([C@H]2c3ccc(cc3)N(C)C)CCc4ccccc4)[O-]
ZINC02061504	7.54	2.4	-15.62	1	3	0	428.535	7	c1ccc(cc1)/C=C/C(=NNC(=O)c2ccc(cc2)c3ccccc3)/C=C/c4ccccc4
ZINC08440073	5.22	13.3	-61.61	0	6	-1	483.588	10	CCCOc1ccc(cc1)C(=O)C2=C(C(=O)N([C@@H]2c3ccc(cc3)N(C)C)CCc4ccccc4)[O-]
ZINC08440072	8.19	1.67	-10.96	1	2	0	398.679	15	CCCCCCCCCCCCCCCn1c2c(c(=N)c3c1CCC3)CCC2
ZINC08440071	5.96	15.46	-44.68	0	5	-1	474.536	7	Cc1ccc(cc1)C(=O)C2=C(C(=O)N([C@H]2c3cccc(c3)Oc4ccccc4)Cc5ccccc5)[O-]
ZINC08440071	6.4	0.76	-25.7	1	5	0	475.544	6	Cc1ccc(cc1)C(=C2[C@@H](N(C(=O)C2=O)Cc3ccccc3)c4cccc(c4)Oc5ccccc5)O
ZINC08440071	5.37	1.02	-20.21	0	5	0	475.544	7	Cc1ccc(cc1)C(=O)[C@@H]2[C@@H](N(C(=O)C2=O)Cc3ccccc3)c4cccc(c4)Oc5ccccc5
ZINC08440070	5.09	4.46	-26.86	0	2	1	457.641	2	CC\1(c2ccc3ccccc3c2N(/C1=C/C=C/C4=[N+](c5ccc6ccccc6c5C4(C)C)C)C)C
ZINC08440068	5.96	14.59	-43.96	0	5	-1	474.536	7	Cc1ccc(cc1)C(=O)C2=C(C(=O)N([C@@H]2c3cccc(c3)Oc4ccccc4)Cc5ccccc5)[O-]
ZINC08440068	6.4	0.77	-25.08	1	5	0	475.544	6	Cc1ccc(cc1)C(=C2[C@H](N(C(=O)C2=O)Cc3ccccc3)c4cccc(c4)Oc5ccccc5)O
ZINC08440068	5.37	1.02	-21.04	0	5	0	475.544	7	Cc1ccc(cc1)C(=O)[C@H]2[C@H](N(C(=O)C2=O)Cc3ccccc3)c4cccc(c4)Oc5ccccc5
ZINC03176061	6.51	1.14	-8.57	1	5	0	424.696	3	c1cc(c(cc1Cl)Cl)Nc2nnc(s2)c3cc4cc(ccc4oc3=O)Cl
ZINC02061497	6.68	1.75	-11.38	1	6	0	461.374	6	CCN(CC)c1ccc2cc(c(=O)oc2c1)c3nnc(s3)Nc4ccc(cc4Cl)Cl
ZINC00847183	5.96	-1.33	-26.84	1	6	0	429.863	4	COc1cc2ccccc2cc1C(=O)Nc3ccc(c(c3)c4nc5c(o4)cccn5)Cl
ZINC08440067	5.93	14.49	-62.1	0	4	-1	438.547	6	Cc1ccc(cc1)C(=O)C2=C(C(=O)N([C@H]2c3ccc(cc3)C(C)(C)C)Cc4ccccc4)[O-]
ZINC08440067	6.38	1.77	-26.7	1	4	0	439.555	5	Cc1ccc(cc1)C(=C2[C@@H](N(C(=O)C2=O)Cc3ccccc3)c4ccc(cc4)C(C)(C)C)O
ZINC08440067	5.35	2.06	-19.17	0	4	0	439.555	6	Cc1ccc(cc1)C(=O)[C@@H]2[C@@H](N(C(=O)C2=O)Cc3ccccc3)c4ccc(cc4)C(C)(C)C
ZINC01954273	6.64	0.53	-8.4	1	5	0	469.147	3	c1cc(c(cc1Cl)Cl)Nc2nnc(s2)c3cc4cc(ccc4oc3=O)Br
ZINC08440066	5.63	1.29	-10.42	0	7	0	452.894	5	CC1=NC2=C([C@@H](C1C(=O)OC)c3ccc(cc3)[N+](=O)[O-])C(=O)C[C@@H](C2)c4ccc(cc4)Cl
ZINC08440066	5.81	1.52	-11.47	0	7	0	452.894	5	COC(=O)C1[C@H](C2C(=NC1=C)C[C@H](CC2=O)c3ccc(cc3)Cl)c4ccc(cc4)[N+](=O)[O-]
ZINC08440066	5.81	2.74	-16.98	0	7	0	452.894	5	COC(=O)C1[C@H]([C@@H]2C(=NC1=C)C[C@H](CC2=O)c3ccc(cc3)Cl)c4ccc(cc4)[N+](=O)[O-]
ZINC08440066	5.63	1.4	-14.02	0	7	0	452.894	5	CC1=C([C@H]([C@H]2C(=N1)C[C@H](CC2=O)c3ccc(cc3)Cl)c4ccc(cc4)[N+](=O)[O-])C(=O)OC
ZINC08440065	5.93	13.62	-62.02	0	4	-1	438.547	6	Cc1ccc(cc1)C(=O)C2=C(C(=O)N([C@@H]2c3ccc(cc3)C(C)(C)C)Cc4ccccc4)[O-]
ZINC08440065	6.38	1.28	-26.64	1	4	0	439.555	5	Cc1ccc(cc1)C(=C2[C@H](N(C(=O)C2=O)Cc3ccccc3)c4ccc(cc4)C(C)(C)C)O
ZINC08440065	5.35	1.58	-21.16	0	4	0	439.555	6	Cc1ccc(cc1)C(=O)[C@H]2[C@H](N(C(=O)C2=O)Cc3ccccc3)c4ccc(cc4)C(C)(C)C
ZINC08440064	5.63	1.4	-12.79	0	7	0	452.894	5	CC1=NC2=C([C@H](C1C(=O)OC)c3ccc(cc3)[N+](=O)[O-])C(=O)C[C@@H](C2)c4ccc(cc4)Cl
ZINC08440064	5.81	1.51	-13.13	0	7	0	452.894	5	COC(=O)C1[C@@H](C2C(=NC1=C)C[C@H](CC2=O)c3ccc(cc3)Cl)c4ccc(cc4)[N+](=O)[O-]
ZINC08440064	5.81	2.65	-13.19	0	7	0	452.894	5	COC(=O)C1[C@@H]([C@H]2C(=NC1=C)C[C@H](CC2=O)c3ccc(cc3)Cl)c4ccc(cc4)[N+](=O)[O-]
ZINC08440064	5.63	1.26	-14.99	0	7	0	452.894	5	CC1=C([C@@H]([C@H]2C(=N1)C[C@H](CC2=O)c3ccc(cc3)Cl)c4ccc(cc4)[N+](=O)[O-])C(=O)OC
ZINC08440063	5.63	1.31	-10.34	0	7	0	452.894	5	CC1=NC2=C([C@@H](C1C(=O)OC)c3ccc(cc3)[N+](=O)[O-])C(=O)C[C@H](C2)c4ccc(cc4)Cl
ZINC08440063	5.81	1.34	-12.1	0	7	0	452.894	5	COC(=O)C1[C@H](C2C(=NC1=C)C[C@@H](CC2=O)c3ccc(cc3)Cl)c4ccc(cc4)[N+](=O)[O-]
ZINC08440063	5.81	2.62	-11.21	0	7	0	452.894	5	COC(=O)C1[C@H]([C@@H]2C(=NC1=C)C[C@@H](CC2=O)c3ccc(cc3)Cl)c4ccc(cc4)[N+](=O)[O-]
ZINC08440063	5.63	1.35	-13.96	0	7	0	452.894	5	CC1=C([C@H]([C@H]2C(=N1)C[C@@H](CC2=O)c3ccc(cc3)Cl)c4ccc(cc4)[N+](=O)[O-])C(=O)OC
ZINC08440062	5.63	1.28	-10.58	0	7	0	452.894	5	CC1=NC2=C([C@H](C1C(=O)OC)c3ccc(cc3)[N+](=O)[O-])C(=O)C[C@H](C2)c4ccc(cc4)Cl
ZINC08440062	5.81	1.51	-12.1	0	7	0	452.894	5	COC(=O)C1[C@@H](C2C(=NC1=C)C[C@@H](CC2=O)c3ccc(cc3)Cl)c4ccc(cc4)[N+](=O)[O-]
ZINC08440062	5.81	2.5	-10.88	0	7	0	452.894	5	COC(=O)C1[C@@H]([C@H]2C(=NC1=C)C[C@@H](CC2=O)c3ccc(cc3)Cl)c4ccc(cc4)[N+](=O)[O-]
ZINC08440062	5.63	1.4	-14.35	0	7	0	452.894	5	CC1=C([C@@H]([C@@H]2C(=N1)C[C@@H](CC2=O)c3ccc(cc3)Cl)c4ccc(cc4)[N+](=O)[O-])C(=O)OC
ZINC08440061	6.45	2.13	-11.03	0	6	0	499.607	7	CC1=NC2=C([C@@H](C1C(=O)OC3CCCCC3)c4ccc(cc4)OC(=O)C)C(=O)C[C@@H](C2)c5ccccc5
ZINC08440061	6.63	2.36	-12.28	0	6	0	499.607	7	CC(=O)Oc1ccc(cc1)[C@H]2C3C(=NC(=C)C2C(=O)OC4CCCCC4)C[C@H](CC3=O)c5ccccc5
ZINC08440061	6.63	3.58	-18.68	0	6	0	499.607	7	CC(=O)Oc1ccc(cc1)[C@H]2[C@@H]3C(=NC(=C)C2C(=O)OC4CCCCC4)C[C@H](CC3=O)c5ccccc5
ZINC08440061	6.45	2.24	-14.91	0	6	0	499.607	7	CC1=C([C@H]([C@H]2C(=N1)C[C@H](CC2=O)c3ccccc3)c4ccc(cc4)OC(=O)C)C(=O)OC5CCCCC5
ZINC08440060	6.45	2.02	-11.6	0	6	0	499.607	7	CC1=NC2=C([C@H](C1C(=O)OC3CCCCC3)c4ccc(cc4)OC(=O)C)C(=O)C[C@@H](C2)c5ccccc5
ZINC08440060	6.63	2.09	-13.25	0	6	0	499.607	7	CC(=O)Oc1ccc(cc1)[C@@H]2C3C(=NC(=C)C2C(=O)OC4CCCCC4)C[C@H](CC3=O)c5ccccc5
ZINC08440060	6.63	3.44	-19.2	0	6	0	499.607	7	CC(=O)Oc1ccc(cc1)[C@@H]2[C@H]3C(=NC(=C)C2C(=O)OC4CCCCC4)C[C@H](CC3=O)c5ccccc5
ZINC08440060	6.45	2.12	-15.41	0	6	0	499.607	7	CC1=C([C@@H]([C@H]2C(=N1)C[C@H](CC2=O)c3ccccc3)c4ccc(cc4)OC(=O)C)C(=O)OC5CCCCC5
ZINC08440059	5.99	1	-18.43	1	5	0	427.595	8	Cc1ccc(cc1)CSc2nnc(s2)NC(=O)COc3ccc(cc3)C(C)(C)C
ZINC08440058	6.45	2.13	-11.53	0	6	0	499.607	7	CC1=NC2=C([C@@H](C1C(=O)OC3CCCCC3)c4ccc(cc4)OC(=O)C)C(=O)C[C@H](C2)c5ccccc5
ZINC08440058	6.63	2.27	-12.42	0	6	0	499.607	7	CC(=O)Oc1ccc(cc1)[C@H]2C3C(=NC(=C)C2C(=O)OC4CCCCC4)C[C@@H](CC3=O)c5ccccc5
ZINC08440058	6.63	3.6	-17.77	0	6	0	499.607	7	CC(=O)Oc1ccc(cc1)[C@H]2[C@@H]3C(=NC(=C)C2C(=O)OC4CCCCC4)C[C@@H](CC3=O)c5ccccc5
ZINC08440058	6.45	2.17	-13.76	0	6	0	499.607	7	CC1=C([C@H]([C@H]2C(=N1)C[C@@H](CC2=O)c3ccccc3)c4ccc(cc4)OC(=O)C)C(=O)OC5CCCCC5

Supplementary Table S2:
Detailed table of XP Docking using Schrodinger's Glide module

ZINC_ID	xLogP	ApolarDesolvation	PolarDesolvation	H Donors	H Acceptors	Charge	MolecularWeight	RotatableBonds	SMILES
ZINC08440057	6.45	2.11	-11.23	0	6	0	499.607	7	CC1=NC2=C([C@H](C1C(=O)OC3CCCCC3)c4ccc(cc4)OC(=O)C)C(=O)C[C@H](C2)c5ccccc5
ZINC08440057	6.63	2.17	-13.33	0	6	0	499.607	7	CC(=O)Oc1ccc(cc1)[C@@H]2C3C(=NC(=C)C2C(=O)OC4CCCCC4)C[C@@H](CC3=O)c5ccccc5
ZINC08440057	6.63	3.59	-18.06	0	6	0	499.607	7	CC(=O)Oc1ccc(cc1)[C@@H]2[C@H]3C(=NC(=C)C2C(=O)OC4CCCCC4)C[C@@H](CC3=O)c5ccccc5
ZINC08440057	6.45	2.05	-15.92	0	6	0	499.607	7	CC1=C([C@@H]([C@H]2C(=N1)C[C@@H](CC2=O)c3ccccc3)c4ccc(cc4)OC(=O)C)C(=O)OC5CCCCC5
ZINC08440056	6.81	0.3	-14.73	1	5	0	441.622	11	CCCCCCOc1ccc(cc1)C(=O)Nc2nnc(s2)SCc3ccc(cc3)C
ZINC08440055	6.69	0.23	-10.95	0	6	0	507.586	7	CC1=NC2=C([C@@H](C1C(=O)OCCc3ccccc3)c4ccc5c(c4)OCO5)C(=O)C[C@@H](C2)c6ccccc6
ZINC08440055	6.87	0.18	-11.06	0	6	0	507.586	7	C=C1C([C@H](C2C(=N1)C[C@H](CC2=O)c3ccccc3)c4ccc5c(c4)OCO5)C(=O)OCCc6ccccc6
ZINC08440055	6.87	1.21	-11.14	0	6	0	507.586	7	C=C1C([C@H]([C@@H]2C(=N1)C[C@H](CC2=O)c3ccccc3)c4ccc5c(c4)OCO5)C(=O)OCCc6ccccc6
ZINC08440055	6.69	0.29	-13.82	0	6	0	507.586	7	CC1=C([C@H]([C@@H]2C(=N1)C[C@H](CC2=O)c3ccccc3)c4ccc5c(c4)OCO5)C(=O)OCCc6ccccc6
ZINC04391432	6.69	14.89	-13.77	0	6	0	507.586	7	CC1=NC2=C([C@H](C1C(=O)OCCc3ccccc3)c4ccc5c(c4)OCO5)C(=O)C[C@@H](C2)c6ccccc6
ZINC04391432	6.87	14.65	-12.96	0	6	0	507.586	7	C=C1C([C@@H](C2C(=N1)C[C@H](CC2=O)c3ccccc3)c4ccc5c(c4)OCO5)C(=O)OCCc6ccccc6
ZINC04391432	6.87	15.55	-14.39	0	6	0	507.586	7	C=C1C([C@@H]([C@H]2C(=N1)C[C@H](CC2=O)c3ccccc3)c4ccc5c(c4)OCO5)C(=O)OCCc6ccccc6
ZINC04391432	6.69	14.87	-14.93	0	6	0	507.586	7	CC1=C([C@@H]([C@H]2C(=N1)C[C@H](CC2=O)c3ccccc3)c4ccc5c(c4)OCO5)C(=O)OCCc6ccccc6
ZINC08440054	6.55	-1.18	-13.87	1	4	0	446.022	5	Cc1ccc(cc1)CSc2nnc(s2)NC(=O)c3c(c4ccc(cc4s3)C)Cl
ZINC08440053	6.69	0.26	-10.48	0	6	0	507.586	7	CC1=NC2=C([C@@H](C1C(=O)OCCc3ccccc3)c4ccc5c(c4)OCO5)C(=O)C[C@H](C2)c6ccccc6
ZINC08440053	6.87	0.39	-11.23	0	6	0	507.586	7	C=C1C([C@H](C2C(=N1)C[C@@H](CC2=O)c3ccccc3)c4ccc5c(c4)OCO5)C(=O)OCCc6ccccc6
ZINC08440053	6.87	1.67	-11.2	0	6	0	507.586	7	C=C1C([C@H]([C@@H]2C(=N1)C[C@@H](CC2=O)c3ccccc3)c4ccc5c(c4)OCO5)C(=O)OCCc6ccccc6
ZINC08440053	6.69	0.45	-13.19	0	6	0	507.586	7	CC1=C([C@H]([C@H]2C(=N1)C[C@@H](CC2=O)c3ccccc3)c4ccc5c(c4)OCO5)C(=O)OCCc6ccccc6
ZINC08440052	6.69	0.46	-14.36	0	6	0	507.586	7	CC1=NC2=C([C@H](C1C(=O)OCCc3ccccc3)c4ccc5c(c4)OCO5)C(=O)C[C@H](C2)c6ccccc6
ZINC08440052	6.87	0.49	-13.18	0	6	0	507.586	7	C=C1C([C@@H](C2C(=N1)C[C@@H](CC2=O)c3ccccc3)c4ccc5c(c4)OCO5)C(=O)OCCc6ccccc6
ZINC08440052	6.87	1.7	-14.94	0	6	0	507.586	7	C=C1C([C@@H]([C@H]2C(=N1)C[C@@H](CC2=O)c3ccccc3)c4ccc5c(c4)OCO5)C(=O)OCCc6ccccc6
ZINC08440052	6.69	0.23	-15.1	0	6	0	507.586	7	CC1=C([C@@H]([C@H]2C(=N1)C[C@@H](CC2=O)c3ccccc3)c4ccc5c(c4)OCO5)C(=O)OCCc6ccccc6
ZINC08440048	6.17	0.52	-19.47	1	5	0	448.013	8	CC(C)(C)c1ccc(cc1)OCC(=O)Nc2nnc(s2)SCc3ccccc3Cl
ZINC08440047	7	-0.18	-15.65	1	5	0	462.04	11	CCCCCCOc1ccc(cc1)C(=O)Nc2nnc(s2)SCc3ccccc3Cl
ZINC08440045	6.8	1.07	-10.91	0	5	0	514.021	7	CC1=NC2=C([C@@H](C1C(=O)OC)c3ccccc3OCc4ccccc4)C(=O)C[C@@H](C2)c5ccc(cc5)Cl
ZINC08440045	6.98	1.13	-11.84	0	5	0	514.021	7	COC(=O)C1[C@H](C2C(=NC1=C)C[C@H](CC2=O)c3ccc(cc3)Cl)c4ccccc4OCc5ccccc5
ZINC08440045	6.98	2.46	-12.92	0	5	0	514.021	7	COC(=O)C1[C@H]([C@@H]2C(=NC1=C)C[C@H](CC2=O)c3ccc(cc3)Cl)c4ccccc4OCc5ccccc5
ZINC08440045	6.8	1.01	-14.25	0	5	0	514.021	7	CC1=C([C@H]([C@@H]2C(=N1)C[C@H](CC2=O)c3ccc(cc3)Cl)c4ccccc4OCc5ccccc5)C(=O)OC
ZINC08440044	6.8	1.1	-11.02	0	5	0	514.021	7	CC1=NC2=C([C@H](C1C(=O)OC)c3ccccc3OCc4ccccc4)C(=O)C[C@@H](C2)c5ccc(cc5)Cl
ZINC08440044	6.98	0.85	-11.66	0	5	0	514.021	7	COC(=O)C1[C@@H](C2C(=NC1=C)C[C@H](CC2=O)c3ccc(cc3)Cl)c4ccccc4OCc5ccccc5
ZINC08440044	6.98	2.3	-18.52	0	5	0	514.021	7	COC(=O)C1[C@@H]([C@H]2C(=NC1=C)C[C@H](CC2=O)c3ccc(cc3)Cl)c4ccccc4OCc5ccccc5
ZINC08440044	6.8	1.2	-14.53	0	5	0	514.021	7	CC1=C([C@@H]([C@@H]2C(=N1)C[C@H](CC2=O)c3ccc(cc3)Cl)c4ccccc4OCc5ccccc5)C(=O)OC
ZINC08440043	6.8	1	-10.78	0	5	0	514.021	7	CC1=NC2=C([C@@H](C1C(=O)OC)c3ccccc3OCc4ccccc4)C(=O)C[C@H](C2)c5ccc(cc5)Cl
ZINC08440043	6.98	1.13	-11.97	0	5	0	514.021	7	COC(=O)C1[C@H](C2C(=NC1=C)C[C@@H](CC2=O)c3ccc(cc3)Cl)c4ccccc4OCc5ccccc5
ZINC08440043	6.98	2.25	-12.04	0	5	0	514.021	7	COC(=O)C1[C@H]([C@@H]2C(=NC1=C)C[C@@H](CC2=O)c3ccc(cc3)Cl)c4ccccc4OCc5ccccc5
ZINC08440043	6.8	1.14	-14.36	0	5	0	514.021	7	CC1=C([C@H]([C@H]2C(=N1)C[C@@H](CC2=O)c3ccc(cc3)Cl)c4ccccc4OCc5ccccc5)C(=O)OC
ZINC00702349	3.92	9.67	-18.84	1	6	0	419.915	5	c1ccc(c(c1)CSc2nnc(s2)NC(=O)[C@H]3COc4ccccc4O3)Cl
ZINC00702349	4.11	6.94	-49.65	0	6	-1	418.907	5	c1ccc(c(c1)CSc2nnc(s2)/N=C(/[C@H]3COc4ccccc4O3)\[O-])Cl
ZINC08440042	6.8	1.2	-15.15	0	5	0	514.021	7	CC1=NC2=C([C@H](C1C(=O)OC)c3ccccc3OCc4ccccc4)C(=O)C[C@H](C2)c5ccc(cc5)Cl
ZINC08440042	6.98	1.23	-14.93	0	5	0	514.021	7	COC(=O)C1[C@@H](C2C(=NC1=C)C[C@@H](CC2=O)c3ccc(cc3)Cl)c4ccccc4OCc5ccccc5
ZINC08440042	6.98	2.44	-15.96	0	5	0	514.021	7	COC(=O)C1[C@@H]([C@H]2C(=NC1=C)C[C@@H](CC2=O)c3ccc(cc3)Cl)c4ccccc4OCc5ccccc5
ZINC08440042	6.8	0.97	-16.52	0	5	0	514.021	7	CC1=C([C@@H]([C@H]2C(=N1)C[C@@H](CC2=O)c3ccc(cc3)Cl)c4ccccc4OCc5ccccc5)C(=O)OC
ZINC08440041	5.08	0.95	-11.17	0	6	0	433.504	6	CC1=NC2=C([C@@H](C1C(=O)OC)c3cccc(c3)OC)C(=O)C[C@@H](C2)c4ccc(cc4)OC
ZINC08440041	5.26	1.01	-12.05	0	6	0	433.504	6	COc1ccc(cc1)[C@@H]2CC3=NC(=C)C([C@H](C3C(=O)C2)c4cccc(c4)OC)C(=O)OC
ZINC08440041	5.26	2.34	-13.38	0	6	0	433.504	6	COc1ccc(cc1)[C@@H]2CC3=NC(=C)C([C@H]([C@H]3C(=O)C2)c4cccc(c4)OC)C(=O)OC
ZINC08440041	5.08	0.89	-14.63	0	6	0	433.504	6	CC1=C([C@H]([C@@H]2C(=N1)C[C@H](CC2=O)c3ccc(cc3)OC)c4cccc(c4)OC)C(=O)OC
ZINC08440040	5.08	1.06	-15.52	0	6	0	433.504	6	CC1=NC2=C([C@H](C1C(=O)OC)c3cccc(c3)OC)C(=O)C[C@@H](C2)c4ccc(cc4)OC
ZINC08440040	5.26	1.18	-14.92	0	6	0	433.504	6	COc1ccc(cc1)[C@@H]2CC3=NC(=C)C([C@@H](C3C(=O)C2)c4cccc(c4)OC)C(=O)OC
ZINC08440040	5.26	2.32	-16.25	0	6	0	433.504	6	COc1ccc(cc1)[C@@H]2CC3=NC(=C)C([C@@H]([C@@H]3C(=O)C2)c4cccc(c4)OC)C(=O)OC
ZINC08440040	5.08	0.93	-16.69	0	6	0	433.504	6	CC1=C([C@@H]([C@H]2C(=N1)C[C@H](CC2=O)c3ccc(cc3)OC)c4cccc(c4)OC)C(=O)OC
ZINC08440039	5.08	0.85	-11.33	0	6	0	433.504	6	CC1=NC2=C([C@@H](C1C(=O)OC)c3cccc(c3)OC)C(=O)C[C@H](C2)c4ccc(cc4)OC
ZINC08440039	5.26	0.92	-12.05	0	6	0	433.504	6	COc1ccc(cc1)[C@H]2CC3=NC(=C)C([C@H](C3C(=O)C2)c4cccc(c4)OC)C(=O)OC
ZINC08440039	5.26	2.18	-13.58	0	6	0	433.504	6	COc1ccc(cc1)[C@H]2CC3=NC(=C)C([C@H]([C@H]3C(=O)C2)c4cccc(c4)OC)C(=O)OC
ZINC08440039	5.08	0.96	-14.49	0	6	0	433.504	6	CC1=C([C@H]([C@@H]2C(=N1)C[C@@H](CC2=O)c3ccc(cc3)OC)c4cccc(c4)OC)C(=O)OC
ZINC08440038	5.08	1.07	-15.33	0	6	0	433.504	6	CC1=NC2=C([C@H](C1C(=O)OC)c3cccc(c3)OC)C(=O)C[C@H](C2)c4ccc(cc4)OC
ZINC08440038	5.26	1.28	-14.95	0	6	0	433.504	6	COc1ccc(cc1)[C@H]2CC3=NC(=C)C([C@@H](C3C(=O)C2)c4cccc(c4)OC)C(=O)OC
ZINC08440038	5.26	2.58	-28.93	0	6	0	433.504	6	COc1ccc(cc1)[C@H]2CC3=NC(=C)C([C@@H]([C@@H]3C(=O)C2)c4cccc(c4)OC)C(=O)OC
ZINC08440038	5.08	1.03	-16.48	0	6	0	433.504	6	CC1=C([C@@H]([C@@H]2C(=N1)C[C@@H](CC2=O)c3ccc(cc3)OC)c4cccc(c4)OC)C(=O)OC
ZINC00702350	3.92	9.67	-18.78	1	6	0	419.915	5	c1ccc(c(c1)CSc2nnc(s2)NC(=O)[C@@H]3COc4ccccc4O3)Cl
ZINC00702350	4.11	6.93	-49.68	0	6	-1	418.907	5	c1ccc(c(c1)CSc2nnc(s2)/N=C(/[C@@H]3COc4ccccc4O3)\[O-])Cl
ZINC08440037	6	0.16	-6.54	1	2	0	283.415	8	CCCCCCCCc1ccc(nc1)c2ccc(cc2)O

Supplementary Table S2:
Detailed table of XP Docking using Schrodinger's Glide module

ZINC_ID	xLogP	ApolarDesolvation	PolarDesolvation	H Donors	H Acceptors	Charge	MolecularWeight	RotatableBonds	SMILES
ZINC00702345	5.38	0.39	-13.96	1	4	0	432.373	6	CC1([C@H]([C@H]1C(=O)Nc2nnc(s2)SCc3ccccc3F)C=C(Cl)Cl)C
ZINC08440036	5.01	-0.83	-11.05	1	6	0	453.922	5	CC1=NC2=C([C@@H](C1C(=O)OC)c3ccc(c(c3)OC)O)C(=O)C[C@@H](C2)c4ccc(cc4)Cl
ZINC08440036	5.19	-0.77	-12.13	1	6	0	453.922	5	COc1cc(ccc1O)[C@H]2C3C(=NC(=C)C2C(=O)OC)C[C@H](CC3=O)c4ccc(cc4)Cl
ZINC08440036	5.19	0.55	-12.96	1	6	0	453.922	5	COc1cc(ccc1O)[C@H]2[C@@H]3C(=NC(=C)C2C(=O)OC)C[C@H](CC3=O)c4ccc(cc4)Cl
ZINC08440036	5.01	-0.9	-14.8	1	6	0	453.922	5	CC1=C([C@H]([C@@H]2C(=N1)C[C@H](CC2=O)c3ccc(cc3)Cl)c4ccc(c(c4)OC)O)C(=O)OC
ZINC08440035	5.01	-0.91	-13.32	1	6	0	453.922	5	CC1=NC2=C([C@H](C1C(=O)OC)c3ccc(c(c3)OC)O)C(=O)C[C@@H](C2)c4ccc(cc4)Cl
ZINC08440035	5.19	-0.78	-13.92	1	6	0	453.922	5	COc1cc(ccc1O)[C@@H]2C3C(=NC(=C)C2C(=O)OC)C[C@H](CC3=O)c4ccc(cc4)Cl
ZINC08440035	5.19	0.36	-13.87	1	6	0	453.922	5	COc1cc(ccc1O)[C@@H]2[C@H]3C(=NC(=C)C2C(=O)OC)C[C@H](CC3=O)c4ccc(cc4)Cl
ZINC08440035	5.01	-0.87	-16.18	1	6	0	453.922	5	CC1=C([C@@H]([C@H]2C(=N1)C[C@H](CC2=O)c3ccc(cc3)Cl)c4ccc(c(c4)OC)O)C(=O)OC
ZINC00702347	5.38	0.51	-15.11	1	4	0	432.373	6	CC1([C@@H]([C@H]1C(=O)Nc2nnc(s2)SCc3ccccc3F)C=C(Cl)Cl)C
ZINC08440034	5.01	-0.94	-11.41	1	6	0	453.922	5	CC1=NC2=C([C@@H](C1C(=O)OC)c3ccc(c(c3)OC)O)C(=O)C[C@H](C2)c4ccc(cc4)Cl
ZINC08440034	5.19	-0.86	-12.66	1	6	0	453.922	5	COc1cc(ccc1O)[C@H]2C3C(=NC(=C)C2C(=O)OC)C[C@@H](CC3=O)c4ccc(cc4)Cl
ZINC08440034	5.19	0.46	-18.45	1	6	0	453.922	5	COc1cc(ccc1O)[C@H]2[C@@H]3C(=NC(=C)C2C(=O)OC)C[C@@H](CC3=O)c4ccc(cc4)Cl
ZINC08440034	5.01	-0.82	-14.77	1	6	0	453.922	5	CC1=C([C@H]([C@@H]2C(=N1)C[C@@H](CC2=O)c3ccc(cc3)Cl)c4ccc(c(c4)OC)O)C(=O)OC
ZINC08440033	5.01	-0.72	-13.61	1	6	0	453.922	5	CC1=NC2=C([C@H](C1C(=O)OC)c3ccc(c(c3)OC)O)C(=O)C[C@H](C2)c4ccc(cc4)Cl
ZINC08440033	5.19	-0.51	-14.16	1	6	0	453.922	5	COc1cc(ccc1O)[C@@H]2C3C(=NC(=C)C2C(=O)OC)C[C@@H](CC3=O)c4ccc(cc4)Cl
ZINC08440033	5.19	0.79	-26.11	1	6	0	453.922	5	COc1cc(ccc1O)[C@@H]2[C@H]3C(=NC(=C)C2C(=O)OC)C[C@@H](CC3=O)c4ccc(cc4)Cl
ZINC08440033	5.01	-0.75	-15.91	1	6	0	453.922	5	CC1=C([C@@H]([C@@H]2C(=N1)C[C@@H](CC2=O)c3ccc(cc3)Cl)c4ccc(c(c4)OC)O)C(=O)OC
ZINC08440032	4.31	1.67	-15.81	0	15	0	576.522	8	COc1ccc(cc1)C2=NN([C@H](C2)c3cccc(c3)[N+](=O)[O-])C(=O)c4cc(c(c(c4)[N+](=O)[O-])N5CCOCC5)[N+](=O)[O-]
ZINC00702346	5.38	0.51	-15.11	1	4	0	432.373	6	CC1([C@H]([C@@H]1C(=O)Nc2nnc(s2)SCc3ccccc3F)C=C(Cl)Cl)C
ZINC08440031	4.31	1.63	-15.47	0	15	0	576.522	8	COc1ccc(cc1)C2=NN([C@@H](C2)c3cccc(c3)[N+](=O)[O-])C(=O)c4cc(c(c(c4)[N+](=O)[O-])N5CCOCC5)[N+](=O)[O-]
ZINC08440030	5.93	-0.43	-11.79	0	11	0	659.291	6	c1cc(ccc1[C@H]2CC(=NN2C(=O)c3cc(c(c(c3)[N+](=O)[O-])N4CCOCC4)[N+](=O)[O-])c5ccc(cc5)Br)Br
ZINC08440029	5.93	-0.41	-11.77	0	11	0	659.291	6	c1cc(ccc1[C@@H]2CC(=NN2C(=O)c3cc(c(c(c3)[N+](=O)[O-])N4CCOCC4)[N+](=O)[O-])c5ccc(cc5)Br)Br
ZINC00702348	5.38	0.39	-14.11	1	4	0	432.373	6	CC1([C@@H]([C@@H]1C(=O)Nc2nnc(s2)SCc3ccccc3F)C=C(Cl)Cl)C
ZINC08440028	10.75	2.82	-9.1	0	2	0	446.723	18	CCCCCCCCCCCCCCCCCn1c2ccccc2nc1c3ccccc3
ZINC08440026	9.93	-0.03	-10.28	1	3	0	428.705	20	CCCCCCCCCCCCCCCCCn1c2ccccc2nc1CCCO
ZINC00702344	5.33	-0.01	-19.05	1	5	0	419.959	7	Cc1ccc(cc1C)OCC(=O)Nc2nnc(s2)SCc3ccc(cc3)Cl
ZINC08440023	6.39	-0.88	-10.37	1	3	0	330.516	13	CCCCCCCCCCn1c2ccccc2nc1CCCO
ZINC08440022	5.38	-1.12	-10.48	1	3	0	302.462	11	CCCCCCCCn1c2ccccc2nc1CCCO
ZINC08440021	4.37	-1.36	-10.51	1	3	0	274.408	9	CCCCCCn1c2ccccc2nc1CCCO
ZINC00702343	5.7	0.29	-18.24	1	5	0	433.986	7	Cc1ccc(cc1C)O[C@H](C)C(=O)Nc2nnc(s2)SCc3ccc(cc3)Cl
ZINC08440020	5.89	-1	-10.44	1	3	0	316.489	12	CCCCCCCCCn1c2ccccc2nc1CCCO
ZINC08440019	7.78	0.43	-14.39	1	8	0	662.327	5	c1ccc(cc1)c2c3cc(ccc3[nH]c(=O)c2C4=NN([C@H](C4)c5cccc(c5)[N+](=O)[O-])C(=O)c6ccc(cc6Cl)Cl)Br
ZINC00702342	5.7	0.35	-18.46	1	5	0	433.986	7	Cc1ccc(cc1C)O[C@@H](C)C(=O)Nc2nnc(s2)SCc3ccc(cc3)Cl
ZINC08440018	7.78	0.44	-14.73	1	8	0	662.327	5	c1ccc(cc1)c2c3cc(ccc3[nH]c(=O)c2C4=NN([C@@H](C4)c5cccc(c5)[N+](=O)[O-])C(=O)c6ccc(cc6Cl)Cl)Br
ZINC08440017	3.35	-3.34	-17.17	1	4	0	425.354	3	c1ccc(c(c1)C(=O)NN2C(=O)/C(=C\c3cccs3)/SC2=S)Br
ZINC08440016	7.73	1.99	-14.21	0	5	0	508.984	5	COc1ccc(cc1)C2=NN([C@H](C2)c3ccc(cc3)F)c4nc5ccc(cc5c(n4)c6ccccc6)Cl
ZINC08440015	7.73	1.98	-14.17	0	5	0	508.984	5	COc1ccc(cc1)C2=NN([C@@H](C2)c3ccc(cc3)F)c4nc5ccc(cc5c(n4)c6ccccc6)Cl
ZINC00702340	4.57	-0.63	-20.22	1	6	0	421.931	8	COc1ccc(cc1)OCC(=O)Nc2nnc(s2)SCc3ccc(cc3)Cl
ZINC08440014	7.85	-0.06	-14.58	1	6	0	647.356	5	COc1ccccc1[C@H]2CC(=NN2C(=O)c3ccc(cc3Cl)Cl)c4c(c5cc(ccc5[nH]c4=O)Br)c6ccccc6
ZINC08440010	6.78	-1.65	-13.84	1	4	0	466.44	5	Cc1ccc2c(c1)sc(c2Cl)C(=O)Nc3nnc(s3)SCc4ccc(cc4)Cl
ZINC08440009	6.2	1.99	-12.01	0	3	0	457.73	3	c1ccc(c(c1)C(=O)N2[C@H](CC(=N2)c3ccc(cc3)Br)c4ccc(cc4)F)Cl
ZINC08440008	6.2	1.99	-11.99	0	3	0	457.73	3	c1ccc(c(c1)C(=O)N2[C@@H](CC(=N2)c3ccc(cc3)Br)c4ccc(cc4)F)Cl
ZINC08440007	6.26	-0.76	-14.37	1	4	0	449.985	5	Cc1ccc2c(c1)sc(c2Cl)C(=O)Nc3nnc(s3)SCc4ccc(cc4)F
ZINC13108926	5.18	11.25	-42.04	0	3	-1	410.114	2	c1cc(ccc1c2nnc(n2c3ccc(cc3)Br)[S-])Br
ZINC00276456	3.26	-4.95	-9.28	0	4	0	316.426	3	Cc1cccc(c1)N2CCN(CC2)S(=O)(=O)c3ccccc3
ZINC08440006	5.43	0.43	-12.76	1	4	0	432.373	6	CC1([C@H]([C@H]1C(=O)Nc2nnc(s2)SCc3ccc(cc3)F)C=C(Cl)Cl)C
ZINC08440005	4.3	1.45	-15.19	0	7	0	443.521	6	Cc1c(c2cc(ccc2o1)N(C(=O)C)S(=O)(=O)c3ccc(cc3)C(C)(C)C)C(=O)OC
ZINC08440004	5.63	1.27	-19.14	0	7	0	491.565	7	CCc1ccc(cc1)S(=O)(=O)N(c2ccc3c(c2)c(c(o3)C)C(=O)OC)C(=O)c4ccc(cc4)C
ZINC08440003	4.9	12.23	-18.92	0	8	0	479.51	7	Cc1ccc(cc1)S(=O)(=O)N(c2ccc3c(c2)c(c(o3)C)C(=O)OC)C(=O)Oc4ccccc4
ZINC08440002	5.35	1.44	-21.91	0	7	0	489.549	7	Cc1ccc(cc1)S(=O)(=O)N(c2ccc3c(c2)c(c(o3)C)C(=O)OC)C(=O)/C=C/c4ccccc4
ZINC00702335	5.43	0.55	-13.92	1	4	0	432.373	6	CC1([C@@H]([C@H]1C(=O)Nc2nnc(s2)SCc3ccc(cc3)F)C=C(Cl)Cl)C
ZINC02061447	6.25	1.72	-11.63	0	6	0	575.786	7	COc1cc(cc(c1OCc2ccccc2Cl)OC)/C=C\3/C(=O)OC(=N3)c4ccc(cc4)I
ZINC02061445	6.85	4.12	-10.35	0	5	0	524.734	6	COc1cc(cc(c1OCc2ccccc2Cl)Cl)/C=C\3/C(=O)OC(=N3)c4cc(c(cc4Cl)F)F
ZINC00702334	5.43	0.55	-13.93	1	4	0	432.373	6	CC1([C@H]([C@@H]1C(=O)Nc2nnc(s2)SCc3ccc(cc3)F)C=C(Cl)Cl)C
ZINC02061443	6.15	3.04	-10.11	0	5	0	472.299	6	COc1cc(cc(c1OCc2ccccc2Cl)Cl)/C=C\3/C(=O)OC(=N3)c4ccc(cc4)F
ZINC08440001	5.43	0.43	-12.73	1	4	0	432.373	6	CC1([C@@H]([C@@H]1C(=O)Nc2nnc(s2)SCc3ccc(cc3)F)C=C(Cl)Cl)C
ZINC08440000	4.88	0.81	-19.93	0	10	0	528.926	7	Cc1c(c2cc(ccc2o1)N(C(=O)c3cccc(c3)[N+](=O)[O-])S(=O)(=O)c4ccc(cc4)Cl)C(=O)OC
ZINC08439999	3.65	-0.49	-18.1	0	8	0	484.917	6	Cc1c(c2cc(ccc2o1)N(C(=O)c3ccncc3)S(=O)(=O)c4ccc(cc4)Cl)C(=O)OC
ZINC08439998	5.45	-2.67	-16.11	1	5	0	438.332	3	CC1(Cc2c(c3cc(ccc3o2)NS(=O)(=O)c4cc(ccc4Cl)Cl)C(=O)C1)C
ZINC08439997	6.48	0.68	-16.32	1	5	0	445.585	11	CCCCCCOc1ccc(cc1)C(=O)Nc2nnc(s2)SCc3ccccc3F

Supplementary Table S2:
Detailed table of XP Docking using Schrodinger's Glide module

ZINC_ID	xLogP	ApolarDesolvation	PolarDesolvation	H Donors	H Acceptors	Charge	MolecularWeight	RotatableBonds	SMILES
ZINC15906697	3.51	12.46	-35.1	1	8	0	480.524	5	Cc1c(c(=O)n(n1C)c2ccccc2)N/C=C/3\c4ccccc4C(=O)N(C3=O)c5ccccc5OC
ZINC15906697	3.51	12.35	-23.94	1	8	0	480.524	5	Cc1c(c(=O)n(n1C)c2ccccc2)N/C=C\3/c4ccccc4C(=O)N(C3=O)c5ccccc5OC
ZINC08439995	7.65	-0.32	-14.8	1	5	0	496.485	11	CCCCCCOc1ccc(cc1)C(=O)Nc2nnc(s2)SCc3ccc(cc3Cl)Cl
ZINC08439994	4.33	14.76	-31.6	1	7	0	478.552	4	Cc1ccc(c(c1)C)N2C(=O)c3ccccc3/C(=C\Nc4c(n(n(c4=O)c5ccccc5)C)C)/C2=O
ZINC08439994	4.33	14.64	-20.86	1	7	0	478.552	4	Cc1ccc(c(c1)C)N2C(=O)c3ccccc3/C(=C/Nc4c(n(n(c4=O)c5ccccc5)C)C)/C2=O
ZINC08439993	6.09	-0.45	-12.06	1	5	0	450.372	5	Cc1c2ccccc2oc1C(=O)Nc3nnc(s3)SCc4ccc(cc4Cl)Cl
ZINC00651676	5.58	0	-21.18	1	7	0	466.921	6	Cc1cc(c2c(c1)nc(o2)c3cc(ccc3Cl)NC(=O)c4cc(c(c(c4)OC)OC)OC)C
ZINC00702330	5.7	1.41	-18.82	1	5	0	431.558	8	CC(C)(C)c1ccc(cc1)OCC(=O)Nc2nnc(s2)SCc3ccc(cc3)F
ZINC00651674	6	0.42	-16.83	1	7	0	460.53	8	CC[C@H](C)c1ccc2c(c1)nc(o2)c3ccc(cc3)NC(=O)c4cc(c(c(c4)OC)OC)OC
ZINC00651672	6	0.42	-16.82	1	7	0	460.53	8	CC[C@@H](C)c1ccc2c(c1)nc(o2)c3ccc(cc3)NC(=O)c4cc(c(c(c4)OC)OC)OC
ZINC00702328	5.82	0.26	-20.29	1	5	0	435.574	7	Cc1cc(cc(c1)OCC(=O)Nc2nnc(s2)SCc3cccc4c3cccc4)C
ZINC02061423	5.31	3.29	-12.5	0	6	0	467.88	7	COc1cc(cc(c1OCc2ccccc2Cl)OC)/C=C\3/C(=O)OC(=N3)c4cccc(c4)F
ZINC02061421	7.65	2.43	-11.82	0	5	0	562.847	7	CCOc1cc(cc(c1OCc2ccccc2Cl)Br)/C=C\3/C(=O)OC(=N3)c4cccc5c4cccc5
ZINC08439992	6.27	-0.31	-19.02	1	4	0	441.581	6	c1ccc2c(c1)cccc2CC(=O)Nc3nnc(s3)SCc4cccc5c4cccc5
ZINC02061419	6.12	1.71	-10.71	0	5	0	498.76	6	COc1cc(cc(c1OCc2ccccc2Cl)Br)/C=C\3/C(=O)OC(=N3)c4ccccc4
ZINC02061417	5.9	0.72	-11.02	0	5	0	504.789	6	COc1cc(cc(c1OCc2ccccc2Cl)Br)/C=C\3/C(=O)OC(=N3)c4cccs4
ZINC08439991	8.11	-2.18	-8.56	3	5	0	554.886	5	COc1ccc(cc1)c2cc(nc3c2c(c(s3)C(=O)Nc4c(cc(cc4Cl)Cl)Cl)N)c5ccccc5
ZINC00702327	5.45	-0.36	-16.32	1	5	0	421.547	7	CCOc1ccc(cc1)C(=O)Nc2nnc(s2)SCc3cccc4c3cccc4
ZINC08439990	7.47	2.26	-14.78	1	4	0	477.633	7	CC(C)(C)c1ccc(cc1)c2cc(nc(c2C#N)SCC(=O)Nc3ccccc3)c4ccccc4
ZINC08439989	8.29	3.12	-14.76	1	4	0	505.687	7	Cc1ccc(c(c1)C)NC(=O)CSc2c(c(cc(n2)c3ccccc3)c4ccc(cc4)C(C)(C)C)C#N
ZINC08439988	7.47	-2.39	-9.29	3	5	0	544.474	5	Cc1ccc(cc1)c2cc(c3c(c(sc3n2)C(=O)Nc4ccc(cc4)Br)N)c5ccc(cc5)OC
ZINC08439987	6.84	1.45	-12.7	1	4	0	469.997	6	Cc1ccc(cc1)c2cc(c(c(n2)SCC(=O)Nc3ccccc3Cl)C#N)c4ccccc4
ZINC00702325	5.15	0.35	-20.77	1	5	0	425.51	7	c1ccc2c(c1)cccc2CSc3nnc(s3)NC(=O)COc4ccc(cc4)F
ZINC08439986	7.96	2.42	-13.72	1	4	0	511.65	7	Cc1ccc(cc1)c2cc(c(c(n2)SCC(=O)Nc3ccccc3c4ccccc4)C#N)c5ccccc5
ZINC08439985	6.82	0.37	-18.6	1	5	0	482.458	8	CC(C)(C)c1ccc(cc1)OCC(=O)Nc2nnc(s2)SCc3ccc(cc3Cl)Cl
ZINC08439984	5.83	2.2	-13.44	1	5	0	480.618	6	Cc1c(sc(c1C#N)NC(=O)CSc2c(c(cc(n2)c3ccccc3)c4ccccc4)C#N)C
ZINC08439983	6.57	0.35	-14.8	1	4	0	500.421	6	c1ccc(cc1)c2cc(nc(c2C#N)SCC(=O)Nc3ccc(cc3)Br)c4ccccc4
ZINC08439982	6.28	0.2	-18.28	1	5	0	468.431	7	Cc1cccc(c1C)O[C@H](C)C(=O)Nc2nnc(s2)SCc3ccc(cc3Cl)Cl
ZINC00844003	4.34	0.75	-15.45	2	8	0	472.925	10	CCOc1cc(cc(c1OCC)OCC)C(=O)Nc2ccc(c(c2)Cl)NC(=O)c3ccco3
ZINC02061403	7.02	-1.16	-14.89	1	5	0	449.293	4	c1cc(cc(c1)NC(=O)c2ccc(o2)c3ccc(cc3)Cl)c4nc5cc(ccc5o4)Cl
ZINC00651666	6.11	0.42	-21.78	2	8	0	496.306	6	c1cc(cc(c1)[N+](=O)[O-])c2ccc(o2)C(=O)Nc3ccc(cc3)NC(=O)c4ccc(cc4Cl)Cl
ZINC08439981	6.28	0.2	-18.31	1	5	0	468.431	7	Cc1cccc(c1C)O[C@@H](C)C(=O)Nc2nnc(s2)SCc3ccc(cc3Cl)Cl
ZINC02061400	6.61	1.18	-8.27	0	4	0	515.734	5	c1ccc(c(c1)COc2ccccc2/C=C\3/C(=O)OC(=N3)c4cccc(c4)I)Cl
ZINC02409214	-0.97	10.64	-30.57	3	8	1	432.504	7	Cc1ccc(cc1)C[n+]2c(c(cc3c2nc4ccccn4c3=O)C(=O)NCCCOC)N
ZINC08439980	7.83	1.14	-11.15	0	5	0	659.101	6	COc1cc(cc(c1OCc2cccc(c2)Br)Cl)/C=C\3/C(=O)OC(=N3)c4cc(ccc4Cl)I
ZINC00651665	4.87	1.5	-10.42	0	5	0	434.311	6	CC(=C)COc1c(cc(cc1Br)/C=C\2/C(=O)OC(=N2)c3cccs3)OC
ZINC02061394	6.14	1.66	-10.01	0	5	0	554.178	6	CC(=C)COc1c(cc(cc1Br)/C=C\2/C(=O)OC(=N2)c3cccc(c3)I)OC
ZINC08439979	-1.06	12.06	-32	3	8	1	451.51	5	Cc1ccc(cc1)C[n+]2c(c(cc3c2nc4ccccn4c3=O)C(=O)NCc5cccnc5)N
ZINC08439978	6.76	14.87	-12.46	0	3	0	460.377	5	c1ccc2c(c1)c(=O)n(c(n2)/C=C/c3cccc(c3)C(F)(F)F)c4cccc(c4)C(F)(F)F
ZINC02061390	6.63	2.45	-11.18	0	5	0	530.777	7	CCOc1cc(cc(c1OCc2ccccc2Br)Cl)/C=C\3/C(=O)OC(=N3)c4cccc(c4)F
ZINC02061388	6.44	1.77	-10.09	0	5	0	561.201	6	COc1cc(cc(c1OCc2cccc(c2)Br)Br)/C=C\3/C(=O)OC(=N3)c4ccc(cc4)F
ZINC02061386	6.27	2.63	-10.55	0	5	0	478.342	6	CC(=C)COc1c(cc(cc1Br)/C=C\2/C(=O)OC(=N2)c3ccc4ccccc4c3)OC
ZINC08439977	7.77	1.54	-7.72	0	4	0	621.212	5	CC(=C)COc1c(cc(cc1I)/C=C\2/C(=O)OC(=N2)c3ccc4ccccc4c3)I
ZINC08439976	6.28	-0.87	-11.41	3	6	0	510.644	5	Cc1c(sc(c1C#N)NC(=O)c2c(c3c(cc(nc3s2)c4ccccc4)c5ccc(cc5)OC)N)C
ZINC08439975	7.66	0.25	-9.26	3	4	0	523.967	5	c1ccc(cc1)c2cc(nc3c2c(c(s3)C(=O)Nc4cc(ccc4Cl)C(F)(F)F)N)c5ccccc5
ZINC00651655	5.27	9.23	-20.94	1	7	0	446.503	9	CCOc1cc(cc(c1OCC)OCC)C(=O)Nc2cccc(c2)c3nc4ccccc4o3
ZINC00651654	5.04	3.02	-12.1	0	8	0	473.279	7	CC(=C)COc1c(cc(cc1Br)/C=C\2/C(=O)OC(=N2)c3ccc(cc3)[N+](=O)[O-])OC
ZINC08439974	-0.2	-2.39	-86.5	4	10	2	475.553	6	Cc1cccn2c1nc3c(c2=O)cc(c(=[NH2+])n3Cc4ccncc4)C(=O)NCC[NH+]5CCOCC5
ZINC02061378	6.23	2.28	-7.99	0	4	0	479.701	5	CC(=C)COc1cccc(c1)/C=C\2/C(=O)OC(=N2)c3cc(ccc3Cl)I
ZINC00651647	3.93	2.5	-23.82	1	9	0	486.48	9	COc1cc(ccc1OCC(=O)Nc2ccc(cc2)C(=O)OC)/C=C\3/C(=O)OC(=N3)c4ccccc4
ZINC08439973	-2.4	10.33	-35.33	3	9	1	452.498	5	Cc1cccn2c1nc3c(c2=O)cc(c([n+]3Cc4ccncc4)N)C(=O)NCc5cccnc5
ZINC08439972	7.03	-2.77	-9.26	3	5	0	530.447	5	COc1ccc(cc1)c2cc(nc3c2c(c(s3)C(=O)Nc4ccc(cc4)Br)N)c5ccccc5
ZINC06177695	5.88	13.47	-14.21	1	6	0	510.644	7	Cc1c(sc(c1C#N)NC(=O)CSc2c(c(cc(n2)c3ccccc3)c4ccc(cc4)OC)C#N)C
ZINC08439971	5.38	2.06	-51.06	2	6	1	479.641	12	CC[NH+](CC)CCCN1[C@H](C(=C(C1=O)O)C(=O)c2ccc(cc2)OCC)c3ccc(cc3)C(C)C
ZINC08439971	4.79	1.9	-60.1	1	6	1	479.641	12	CC[NH+](CC)CCCN1[C@H]([C@@H](C(=O)C1=O)C(=O)c2ccc(cc2)OCC)c3ccc(cc3)C(C)C
ZINC00074217	4.43	10.08	-9	0	2	0	296.679	2	c1ccc(cc1)c2cn3cc(cc(c3n2)Cl)C(F)(F)F
ZINC04002657	4.48	9.38	-9.59	0	3	0	326.705	3	COc1ccc(cc1)c2cn3cc(cc(c3n2)Cl)C(F)(F)F
ZINC05728874	4.76	9.42	-10.11	1	4	0	407.701	4	c1cc(cc(c1)NC(=O)c2cn3cc(cc(c3n2)Cl)C(F)(F)F)C(F)(F)F
ZINC05728903	3.59	7.64	-8.25	2	5	0	339.157	3	c1cc(ccc1NNC(=O)c2cn3cc(cc(c3n2)Cl)Cl)F
ZINC00984064	1.42	4.22	-8.91	1	4	0	242.278	4	COc1ccc(cc1)CNC(=O)c2ccccn2
ZINC00448009	3.3	-2.6	-12.31	2	4	0	253.733	2	c1cc(ncc1Cl)NC(=O)NC2CCCCC2

Supplementary Table S2:
Detailed table of XP Docking using Schrodinger's Glide module

ZINC_ID	xLogP	ApolarDesolvation	PolarDesolvation	H Donors	H Acceptors	Charge	MolecularWeight	RotatableBonds	SMILES
ZINC00480133	3.89	-2.38	-7.33	2	4	0	282.13	2	c1ccc(cc1)NC(=O)Nc2ccc(nc2Cl)Cl
ZINC09089086	2.29	4.51	-41.47	1	5	-1	228.231	1	CCc1c([nH]c2nc3ccccc3n2c1=O)[O-]
ZINC09089086	2.29	-1.91	-32.04	3	5	1	230.247	1	CCc1c(=O)[nH]c2[nH+]c3ccccc3n2c1O
ZINC09089086	2.75	3.44	-115.83	0	5	-2	227.223	1	CCc1c(nc2nc3ccccc3n2c1[O-])[O-]
ZINC18085164	5.43	13.04	-19.31	2	7	0	445.519	4	Cc1ccccc1NC(=O)C2=C(NC3=C([C@H]2c4ccc(cc4)[N+](=O)[O-])C(=O)CC(C3)(C)C)C
ZINC18085164	5.55	14.02	-21.9	1	7	0	445.519	4	Cc1ccccc1NC(=O)[C@@H]2[C@@H]([C@@H]3C(=O)CC(C=C3N=C2C)(C)C)c4ccc(cc4)[N+](=O)[O-]
ZINC18085164	5.38	13.09	-57.04	1	7	0	445.519	4	Cc1ccccc1NC(=O)[C@@H]2[C@H](C3=C(CC(CC3=O)(C)C)N=C2C)c4ccc(cc4)[N+](=O)[O-]
ZINC00471779	2.3	4.66	-8.62	2	4	0	261.712	3	c1ccc(c(c1)NC(=O)NCc2ccccn2)Cl
ZINC06443371	7.69	18.24	-13.97	1	4	0	519.714	7	Cc1cccc(c1NC(=O)[C@H](C)Sc2c(c(cc(n2)c3ccccc3)c4ccc(cc4)C(C)(C)C)C#N)C
ZINC08439970	5.38	2.15	-44.42	2	6	1	479.641	12	CC[NH+](CC)CCCN1[C@@H](C(=C(C1=O)O)C(=O)c2ccc(cc2)OCC)c3ccc(cc3)C(C)C
ZINC08439970	4.79	2.06	-53.24	1	6	1	479.641	12	CC[NH+](CC)CCCN1[C@@H]([C@@H](C(=O)C1=O)C(=O)c2ccc(cc2)OCC)c3ccc(cc3)C(C)C
ZINC06443308	7.69	18.28	-14.04	1	4	0	519.714	7	Cc1cccc(c1NC(=O)[C@@H](C)Sc2c(c(cc(n2)c3ccccc3)c4ccc(cc4)C(C)(C)C)C#N)C
ZINC08439969	8.11	0.58	-9.11	3	4	0	537.994	5	Cc1ccc(cc1)c2cc(c3c(c(sc3n2)C(=O)Nc4cc(ccc4Cl)C(F)(F)F)N)c5ccccc5
ZINC08439968	6.86	1.27	-16.6	1	4	0	469.997	6	Cc1ccc(cc1)c2cc(c(c(n2)SCC(=O)Nc3cccc(c3)Cl)C#N)c4ccccc4
ZINC08439967	4.71	12.15	-40.76	0	5	-1	426.492	8	CCCOc1ccc(cc1)C(=O)C2=C(C(=O)N([C@H]2c3ccccc3)Cc4ccccc4)[O-]
ZINC08439967	5.16	-0.05	-26.86	1	5	0	427.5	7	CCCOc1ccc(cc1)C(=C2[C@@H](N(C(=O)C2=O)Cc3ccccc3)c4ccccc4)O
ZINC08439967	4.13	0.2	-22.44	0	5	0	427.5	8	CCCOc1ccc(cc1)C(=O)[C@@H]2[C@@H](N(C(=O)C2=O)Cc3ccccc3)c4ccccc4
ZINC00651640	4.36	1.37	-28.9	3	8	0	501.583	6	Cc1ccc(cc1C)NC(=O)COc2ccc(cc2OC)[C@H]3C(=C(OC4=C3C(=O)CC(C4)(C)C)N)C#N
ZINC00651643	4.36	1.37	-28.98	3	8	0	501.583	6	Cc1ccc(cc1C)NC(=O)COc2ccc(cc2OC)[C@@H]3C(=C(OC4=C3C(=O)CC(C4)(C)C)N)C#N
ZINC08439966	4.71	12.15	-40.37	0	5	-1	426.492	8	CCCOc1ccc(cc1)C(=O)C2=C(C(=O)N([C@@H]2c3ccccc3)Cc4ccccc4)[O-]
ZINC08439966	5.16	-0.03	-27.57	1	5	0	427.5	7	CCCOc1ccc(cc1)C(=C2[C@H](N(C(=O)C2=O)Cc3ccccc3)c4ccccc4)O
ZINC08439966	4.13	0.27	-21.86	0	5	0	427.5	8	CCCOc1ccc(cc1)C(=O)[C@H]2[C@H](N(C(=O)C2=O)Cc3ccccc3)c4ccccc4
ZINC08439965	8.01	2.45	-14.43	1	5	0	541.676	8	Cc1ccc(cc1)c2cc(c(c(n2)SCC(=O)Nc3ccccc3c4ccccc4)C#N)c5ccc(cc5)OC
ZINC08439964	6.39	1.13	-12.77	1	4	0	455.97	6	c1ccc(cc1)c2cc(nc(c2C#N)SCC(=O)Nc3ccccc3Cl)c4ccccc4
ZINC00702307	3.71	8.46	-42.02	1	6	-1	428.464	7	CCOc1ccc(cc1)C(=O)C2=C(C(=O)N([C@H]2c3cccc(c3)O)Cc4ccccc4)[O-]
ZINC00702307	4.15	-2.1	-27.39	2	6	0	429.472	6	CCOc1ccc(cc1)C(=C2[C@@H](N(C(=O)C2=O)Cc3ccccc3)c4cccc(c4)O)O
ZINC00702307	3.12	-1.85	-23.76	1	6	0	429.472	7	CCOc1ccc(cc1)C(=O)[C@@H]2[C@@H](N(C(=O)C2=O)Cc3ccccc3)c4cccc(c4)O
ZINC08439963	5.94	-0.28	-15.62	1	6	0	462.571	9	CCOc1cc(cc(c1OCC)OCC)C(=O)Nc2ccc(cc2)c3nc4ccccc4s3
ZINC00651633	4.37	8.45	-25.57	1	8	0	447.491	9	CCOc1cc(cc(c1OCC)OCC)C(=O)Nc2cccc(c2)c3nc4c(o3)cccn4
ZINC00702308	3.71	8.49	-41.66	1	6	-1	428.464	7	CCOc1ccc(cc1)C(=O)C2=C(C(=O)N([C@@H]2c3cccc(c3)O)Cc4ccccc4)[O-]
ZINC00702308	4.15	-2.1	-28.07	2	6	0	429.472	6	CCOc1ccc(cc1)C(=C2[C@H](N(C(=O)C2=O)Cc3ccccc3)c4cccc(c4)O)O
ZINC00702308	3.12	-1.86	-21.41	1	6	0	429.472	7	CCOc1ccc(cc1)C(=O)[C@@H]2[C@H](N(C(=O)C2=O)Cc3ccccc3)c4cccc(c4)O
ZINC02061364	7.19	-0.37	-13.3	1	5	0	442.902	4	Cc1cc(c2c(c1)nc(o2)c3ccc(cc3)NC(=O)c4ccc(o4)c5ccc(cc5)Cl)C
ZINC02061363	7.04	-0.71	-11.75	1	4	0	430.916	4	c1ccc2c(c1)nc(s2)c3ccc(cc3)NC(=O)c4ccc(o4)c5ccc(cc5)Cl
ZINC02061362	7.18	-1.78	-9.15	1	5	0	493.744	4	c1cc(ccc1c2ccc(o2)C(=O)Nc3ccc4c(c3)nc(o4)c5ccc(cc5)Br)Cl
ZINC02061361	6.36	1.25	-8.16	0	4	0	468.734	5	c1ccc(c(c1)COc2ccccc2/C=C\3/C(=O)OC(=N3)c4ccc(cc4)Br)Cl
ZINC02061359	6.13	2.84	-11.83	0	7	0	469.28	6	c1ccc(c(c1)COc2cccc(c2)/C=C\3/C(=O)OC(=N3)c4cc(ccc4Cl)[N+](=O)[O-])Cl
ZINC02061357	5.89	3.03	-15.48	0	8	0	492.915	8	CCOc1cc(ccc1OCc2ccccc2Cl)/C=C\3/C(=O)OC(=N3)c4ccc(c(c4)[N+](=O)[O-])C
ZINC02061355	6.32	2.24	-11.54	0	5	0	512.787	7	CCOc1cc(ccc1OCc2ccccc2Cl)/C=C\3/C(=O)OC(=N3)c4ccccc4Br
ZINC00651619	5.57	1.97	-8.55	0	4	0	445.256	5	CC(=C)COc1ccccc1/C=C\2/C(=O)OC(=N2)c3cccc(c3)I
ZINC00651610	5.6	1.97	-8.17	0	4	0	445.256	5	CC(=C)COc1ccccc1/C=C\2/C(=O)OC(=N2)c3ccc(cc3)I
ZINC00651607	5.23	2.26	-9.92	0	5	0	475.282	6	CC(=C)COc1ccc(cc1OC)/C=C\2/C(=O)OC(=N2)c3ccc(cc3)I
ZINC02061341	5.61	2.27	-9.71	0	5	0	489.309	7	CCOc1cc(ccc1OCC(=C)C)/C=C\2/C(=O)OC(=N2)c3ccc(cc3)I
ZINC08439961	7.3	1.5	-15.4	1	6	0	567.736	9	CCOC(=O)c1c2c(sc1NC(=O)CSc3c(c(cc(n3)c4ccc(cc4)C)c5ccccc5)C#N)CCCC2
ZINC08439960	7.09	2.06	-15.82	1	5	0	493.632	7	Cc1ccc(cc1)c2cc(c(c(n2)SCC(=O)Nc3ccc(c(c3)C)C)C#N)c4ccc(cc4)OC
ZINC08439959	6.33	-0.85	-12.51	3	6	0	479.561	6	COC(=O)c1ccc(cc1)NC(=O)c2c(c3c(cc(nc3s2)c4ccccc4)c5ccccc5)N
ZINC08439958	6.16	1.65	-14.99	1	4	0	435.552	6	Cc1ccccc1NC(=O)CSc2c(c(cc(n2)c3ccccc3)c4ccccc4)C#N
ZINC02061330	6.08	-1.34	-12.55	1	7	0	525.399	9	CCOc1cc(cc(c1OCC)OCC)C(=O)Nc2ccc3c(c2)nc(o3)c4ccc(cc4)Br
ZINC02061329	5.87	2.12	-8.27	0	4	0	452.279	5	c1ccc(c(c1)COc2cccc(c2)/C=C\3/C(=O)OC(=N3)c4ccc(cc4)F)Br
ZINC08439957	6.71	1.71	-9.77	0	5	0	608.201	6	COc1cc(cc(c1OCc2cccc(c2)Br)I)/C=C\3/C(=O)OC(=N3)c4ccc(cc4)F
ZINC00651597	5.29	3.03	-11.38	0	5	0	442.309	7	CCOc1cc(ccc1OCC(=C)C)/C=C\2/C(=O)OC(=N2)c3ccccc3Br
ZINC08439956	7.72	0.64	-15.93	0	5	0	535.445	5	COc1ccc(cc1)C2=NN([C@H](C2)c3ccc(cc3)Br)c4nc5ccccc5c(n4)c6ccccc6
ZINC08439955	7.72	0.63	-15.9	0	5	0	535.445	5	COc1ccc(cc1)C2=NN([C@@H](C2)c3ccc(cc3)Br)c4nc5ccccc5c(n4)c6ccccc6
ZINC08439954	9.12	-0.29	-11.86	0	4	0	618.76	4	c1ccc(cc1)c2c3cc(ccc3nc(n2)N4[C@H](CC(=N4)c5ccc(cc5)Br)c6ccc(cc6)Br)Cl
ZINC08439953	9.12	-0.3	-11.72	0	4	0	618.76	4	c1ccc(cc1)c2c3cc(ccc3nc(n2)N4[C@@H](CC(=N4)c5ccc(cc5)Br)c6ccc(cc6)Br)Cl
ZINC02061324	7.8	-3.79	-8.53	0	4	0	514.254	3	c1ccc(c(c1)c2c3cc(ccc3nc(n2)N4CCN(CC4)c5ccccc5Cl)Br)Cl
ZINC02282159	7.25	-1.19	-14.75	2	6	0	525.406	6	COc1ccc(cc1)NC(=O)c2ccc(cc2)Nc3nc4ccc(cc4c(n3)c5ccccc5)Br
ZINC02061320	7.72	-0.85	-13.31	2	5	0	485.374	5	c1ccc(cc1)c2c3cc(ccc3nc(n2)Nc4ccc(cc4)C(=O)Nc5cccc(c5)Cl)Cl
ZINC02811564	7.12	-0.58	-14.81	2	6	0	480.955	6	COc1ccc(cc1)NC(=O)c2ccc(cc2)Nc3nc4ccc(cc4c(n3)c5ccccc5)Cl
ZINC06443234	7.29	-3.63	-8.76	2	3	0	596.25	5	Cc1cccc(c1C)NC[C@H](Cn2c3ccc(cc3c4c2ccc(c4)I)I)O
ZINC06443231	7.29	-3.57	-8.68	2	3	0	596.25	5	Cc1cccc(c1C)NC[C@@H](Cn2c3ccc(cc3c4c2ccc(c4)I)I)O

Supplementary Table S2:
Detailed table of XP Docking using Schrodinger's Glide module

ZINC_ID	xLogP	ApolarDesolvation	PolarDesolvation	H Donors	H Acceptors	Charge	MolecularWeight	RotatableBonds	SMILES
ZINC02061318	6.05	1.96	-14.29	1	3	0	441.275	4	c1cc(ccc1[C@H]2C=C(C(=O)N2c3ccc(cc3)F)Nc4ccc(cc4)F)Br
ZINC02061319	6.05	1.97	-13.74	1	3	0	441.275	4	c1cc(ccc1[C@@H]2C=C(C(=O)N2c3ccc(cc3)F)Nc4ccc(cc4)F)Br
ZINC02061316	5.97	13.11	-8.16	0	6	0	440.286	4	c1ccc(c(c1)[C@H]2C/C(=N/c3ccc(cc3)Cl)/C(=O)N2c4ccc(cc4)Cl)[N+](=O)[O-]
ZINC02061317	5.97	13.06	-7.97	0	6	0	440.286	4	c1ccc(c(c1)[C@@H]2C/C(=N/c3ccc(cc3)Cl)/C(=O)N2c4ccc(cc4)Cl)[N+](=O)[O-]
ZINC02061314	6	13.43	-12.3	0	6	0	440.286	4	c1cc(cc(c1)[N+](=O)[O-])[C@H]2C/C(=N/c3ccc(cc3)Cl)/C(=O)N2c4ccc(cc4)Cl
ZINC02061315	6	13.43	-12.18	0	6	0	440.286	4	c1cc(cc(c1)[N+](=O)[O-])[C@@H]2C/C(=N/c3ccc(cc3)Cl)/C(=O)N2c4ccc(cc4)Cl
ZINC02061312	6.02	13.43	-16.84	0	6	0	440.286	4	c1cc(ccc1[C@H]2C/C(=N/c3ccc(cc3)Cl)/C(=O)N2c4ccc(cc4)Cl)[N+](=O)[O-]
ZINC02061313	6.02	13.43	-16.93	0	6	0	440.286	4	c1cc(ccc1[C@@H]2C/C(=N/c3ccc(cc3)Cl)/C(=O)N2c4ccc(cc4)Cl)[N+](=O)[O-]
ZINC02061308	6.95	0.8	-13.06	1	3	0	429.734	4	c1cc(ccc1[C@H]2C=C(C(=O)N2c3ccc(cc3)Cl)Nc4ccc(cc4)Cl)Cl
ZINC02061309	6.95	0.82	-12.56	1	3	0	429.734	4	c1cc(ccc1[C@@H]2C=C(C(=O)N2c3ccc(cc3)Cl)Nc4ccc(cc4)Cl)Cl
ZINC02061306	7.08	0.18	-12.98	1	3	0	474.185	4	c1cc(ccc1[C@H]2C=C(C(=O)N2c3ccc(cc3)Cl)Nc4ccc(cc4)Cl)Br
ZINC02061307	7.08	0.2	-12.52	1	3	0	474.185	4	c1cc(ccc1[C@@H]2C=C(C(=O)N2c3ccc(cc3)Cl)Nc4ccc(cc4)Cl)Br
ZINC00843897	5.22	11.93	-14.9	1	6	0	479.458	6	COc1ccc(cc1OC)[C@H]2c3c(n[nH]c3C(=O)N2c4cccc(c4)C(F)(F)F)c5ccccc5
ZINC00843898	5.22	11.45	-13	1	6	0	479.458	6	COc1ccc(cc1OC)[C@@H]2c3c(n[nH]c3C(=O)N2c4cccc(c4)C(F)(F)F)c5ccccc5
ZINC00651584	5.69	12.77	-17.17	1	4	0	437.396	4	c1ccc(cc1)c2c3c([nH]n2)C(=O)N([C@@H]3c4ccccc4F)c5cccc(c5)C(F)(F)F
ZINC00843893	6.26	13.25	-13.01	1	4	0	453.851	4	c1ccc(cc1)c2c3c([nH]n2)C(=O)N([C@H]3c4ccc(cc4)Cl)c5cccc(c5)C(F)(F)F
ZINC00843894	6.26	12.88	-11.96	1	4	0	453.851	4	c1ccc(cc1)c2c3c([nH]n2)C(=O)N([C@@H]3c4ccc(cc4)Cl)c5cccc(c5)C(F)(F)F
ZINC06446775	6.39	13.35	-12.84	1	4	0	498.302	4	c1ccc(cc1)c2c3c([nH]n2)C(=O)N([C@H]3c4ccc(cc4)Br)c5cccc(c5)C(F)(F)F
ZINC06446774	6.39	12.98	-11.88	1	4	0	498.302	4	c1ccc(cc1)c2c3c([nH]n2)C(=O)N([C@@H]3c4ccc(cc4)Br)c5cccc(c5)C(F)(F)F
ZINC00843889	5.58	12.73	-12.71	1	4	0	419.406	4	c1ccc(cc1)c2c3c([nH]n2)C(=O)N([C@H]3c4ccccc4)c5cccc(c5)C(F)(F)F
ZINC00843890	5.58	12.36	-12.1	1	4	0	419.406	4	c1ccc(cc1)c2c3c([nH]n2)C(=O)N([C@@H]3c4ccccc4)c5cccc(c5)C(F)(F)F
ZINC08439949	5.76	10.93	-11.66	1	5	0	532.312	6	COc1ccccc1[C@H]2C(=C(C(=O)N2c3cccc(c3)C(F)(F)F)O)C(=O)c4ccc(cc4)Br
ZINC08439949	5.18	11.67	-16.25	0	5	0	532.312	6	COc1ccccc1[C@H]2[C@@H](C(=O)C(=O)N2c3cccc(c3)C(F)(F)F)C(=O)c4ccc(cc4)Br
ZINC08439948	5.76	10.95	-11.71	1	5	0	532.312	6	COc1ccccc1[C@@H]2C(=C(C(=O)N2c3cccc(c3)C(F)(F)F)O)C(=O)c4ccc(cc4)Br
ZINC08439948	5.18	11.69	-16.22	0	5	0	532.312	6	COc1ccccc1[C@@H]2[C@H](C(=O)C(=O)N2c3cccc(c3)C(F)(F)F)C(=O)c4ccc(cc4)Br
ZINC08439947	6.39	11.66	-10.57	1	4	0	536.731	5	c1ccc(c(c1)[C@H]2C(=C(C(=O)N2c3cccc(c3)C(F)(F)F)O)C(=O)c4ccc(cc4)Br)Cl
ZINC08439947	5.8	12.35	-16.18	0	4	0	536.731	5	c1ccc(c(c1)[C@H]2[C@H](C(=O)C(=O)N2c3cccc(c3)C(F)(F)F)C(=O)c4ccc(cc4)Br)Cl
ZINC08439946	6.39	12.02	-9.45	1	4	0	536.731	5	c1ccc(c(c1)[C@@H]2C(=C(C(=O)N2c3cccc(c3)C(F)(F)F)O)C(=O)c4ccc(cc4)Br)Cl
ZINC08439946	5.8	12.76	-12.27	0	4	0	536.731	5	c1ccc(c(c1)[C@@H]2[C@H](C(=O)C(=O)N2c3cccc(c3)C(F)(F)F)C(=O)c4ccc(cc4)Br)Cl
ZINC00843877	5.47	13.04	-14.31	1	7	0	475.302	4	c1ccc(cc1)N2[C@H](c3c(n[nH]c3C2=O)c4ccc(cc4)Br)c5ccc(cc5)[N+](=O)[O-]
ZINC00843878	5.47	12.68	-12.46	1	7	0	475.302	4	c1ccc(cc1)N2[C@@H](c3c(n[nH]c3C2=O)c4ccc(cc4)Br)c5ccc(cc5)[N+](=O)[O-]
ZINC06446668	6.33	12.94	-9.97	1	4	0	509.201	3	c1ccc(cc1)N2[C@H](c3c(n[nH]c3C2=O)c4ccc(cc4)Br)c5ccc(cc5)Br
ZINC06446667	6.33	12.59	-8.74	1	4	0	509.201	3	c1ccc(cc1)N2[C@@H](c3c(n[nH]c3C2=O)c4ccc(cc4)Br)c5ccc(cc5)Br
ZINC06406341	5.45	11.54	-61.42	0	5	-1	466.435	6	Cc1ccc(cc1)C(=O)C2=C(C(=O)N([C@H]2c3ccc(cc3)OC)c4cccc(c4)C(F)(F)F)[O-]
ZINC06406755	5.45	11.62	-61.01	0	5	-1	466.435	6	Cc1ccc(cc1)C(=O)C2=C(C(=O)N([C@@H]2c3ccc(cc3)OC)c4cccc(c4)C(F)(F)F)[O-]
ZINC08439934	4.06	4.86	-70.18	1	13	1	428.337	4	C[NH+]1CCCC1=C2c3cc(cc(c3-c4c2cc(cc4[N+](=O)[O-])[N+](=O)[O-])[N+](=O)[O-])[N+](=O)[O-]
ZINC08439933	6.76	-4.3	-57.51	2	4	1	578.756	5	c1cc(ccc1N2CC[NH+](CC2)C[C@H](Cn3c4ccc(cc4c5c3ccc(c5)Br)Br)O)Cl
ZINC08439932	6.76	-4.34	-57.65	2	4	1	578.756	5	c1cc(ccc1N2CC[NH+](CC2)C[C@@H](Cn3c4ccc(cc4c5c3ccc(c5)Br)Br)O)Cl
ZINC00843796	3.88	1.35	-16.27	1	4	0	427.22	4	c1cc(cc(c1)NC(=O)[C@H]2CC(=O)N(C2)c3ccc(cc3)Br)C(F)(F)F
ZINC00843797	3.88	1.35	-20.49	1	4	0	427.22	4	c1cc(cc(c1)NC(=O)[C@@H]2CC(=O)N(C2)c3ccc(cc3)Br)C(F)(F)F
ZINC08439931	5.27	0.4	-17.32	0	7	0	497.956	7	CCOC(=O)c1c(oc2c1cc(cc2)N(C(=O)c3ccccc3Cl)S(=O)(=O)c4ccccc4)C
ZINC08439930	5.32	0.31	-18.38	0	7	0	497.956	7	CCOC(=O)c1c(oc2c1cc(cc2)N(C(=O)c3ccc(cc3)Cl)S(=O)(=O)c4ccccc4)C
ZINC02484263	4.37	0.71	-14.81	0	6	0	427.522	5	Cc1c(c2cc(ccc2o1)N(C(=O)C)S(=O)(=O)c3ccc(cc3)C(C)(C)C)C(=O)C
ZINC08439929	5.88	1.01	-19.9	0	6	0	487.577	7	CCc1ccc(cc1)S(=O)(=O)N(c2ccc3c(c2)c(c(o3)C)C(=O)C)C(=O)/C=C/c4ccccc4
ZINC08439928	5.43	1.06	-15.95	0	7	0	477.538	7	CCc1ccc(cc1)S(=O)(=O)N(c2ccc3c(c2)c(c(o3)C)C(=O)C)C(=O)Oc4ccccc4
ZINC08439927	5.92	0.26	-16.83	0	6	0	495.984	6	CCc1ccc(cc1)S(=O)(=O)N(c2ccc3c(c2)c(c(o3)C)C(=O)C)C(=O)c4ccc(cc4)Cl
ZINC08439926	5.65	0.38	-18.02	0	6	0	493.968	6	Cc1c(c2cc(ccc2o1)N(C(=O)/C=C/c3ccccc3)S(=O)(=O)c4ccc(cc4)Cl)C(=O)C
ZINC02061254	4.78	0.18	-17.25	0	6	0	447.512	5	Cc1ccc(cc1)S(=O)(=O)N(c2ccc3c(c2)c(c(o3)C)C(=O)C)C(=O)c4ccccc4
ZINC08439925	4.96	-0.15	-20.47	0	6	0	467.93	5	Cc1c(c2cc(ccc2o1)N(C(=O)c3ccccc3Cl)S(=O)(=O)c4ccccc4)C(=O)C
ZINC08439924	5.01	-0.22	-17.69	0	6	0	467.93	5	Cc1c(c2cc(ccc2o1)N(C(=O)c3ccc(cc3)Cl)S(=O)(=O)c4ccccc4)C(=O)C
ZINC08439923	4.33	10.23	-19.4	0	6	0	433.485	5	Cc1c(c2cc(ccc2o1)N(C(=O)c3ccccc3)S(=O)(=O)c4ccccc4)C(=O)C
ZINC08439922	7.83	1.35	-14.64	0	4	0	523.409	4	c1ccc(cc1)c2c3ccccc3nc(n2)N4[C@H](CC(=N4)c5ccc(cc5)Br)c6ccc(cc6)F
ZINC08439921	7.83	1.35	-14.59	0	4	0	523.409	4	c1ccc(cc1)c2c3ccccc3nc(n2)N4[C@@H](CC(=N4)c5ccc(cc5)Br)c6ccc(cc6)F
ZINC08439920	4.03	-0.43	-17.47	0	8	0	498.944	7	CCOC(=O)c1c(oc2c1cc(cc2)N(C(=O)c3ccncc3)S(=O)(=O)c4ccc(cc4)Cl)C
ZINC08439919	5.7	0.71	-19.12	0	7	0	499.544	6	Cc1c(c2cc(ccc2o1)N(C(=O)Oc3ccccc3)S(=O)(=O)c4ccc5ccccc5c4)C(=O)C
ZINC08439918	5.51	-0.01	-18	0	6	0	483.545	5	Cc1c(c2cc(ccc2o1)N(C(=O)c3ccccc3)S(=O)(=O)c4ccc5ccccc5c4)C(=O)C
ZINC02061239	6.29	-1.85	-5.01	0	3	0	355.301	8	CCCCCCCSc1nnc(o1)c2ccc(cc2)Br
ZINC02061238	6.22	-2.54	-6.01	0	3	0	416.127	4	c1cc(ccc1c2nnc(o2)SCc3ccc(cc3Cl)Cl)Br
ZINC08439917	5.62	-1.11	-8.16	0	6	0	456.321	3	c1cc(cc(c1)[N+](=O)[O-])[C@H]2N3[C@@H](CC(=N3)c4cccs4)c5cc(ccc5O2)Br
ZINC08439916	5.62	-0.65	-10.13	0	6	0	456.321	3	c1cc(cc(c1)[N+](=O)[O-])[C@@H]2N3[C@@H](CC(=N3)c4cccs4)c5cc(ccc5O2)Br
ZINC08439915	6.97	-1.93	-5.01	0	3	0	480.214	2	c1cc(sc1)C2=NN3[C@H](C2)c4cc(ccc4O[C@@H]3c5ccc(cc5Cl)Cl)Br

Supplementary Table S2:
Detailed table of XP Docking using Schrodinger's Glide module

ZINC_ID	xLogP	ApolarDesolvation	PolarDesolvation	H Donors	H Acceptors	Charge	MolecularWeight	RotatableBonds	SMILES
ZINC08439914	6.97	-0.66	-4.86	0	3	0	480.214	2	c1cc(sc1)C2=NN3[C@@H](C2)c4cc(ccc4O[C@@H]3c5ccc(cc5Cl)Cl)Br
ZINC08439913	6.49	-2.38	-6.17	0	3	0	490.22	2	c1cc(sc1)C2=NN3[C@H](C2)c4cc(ccc4O[C@@H]3c5ccc(cc5)Br)Br
ZINC08439912	6.49	-1.93	-4.83	0	3	0	490.22	2	c1cc(sc1)C2=NN3[C@@H](C2)c4cc(ccc4O[C@@H]3c5ccc(cc5)Br)Br
ZINC08439911	6.36	-1.77	-6.23	0	3	0	445.769	2	c1cc(sc1)C2=NN3[C@H](C2)c4cc(ccc4O[C@@H]3c5ccc(cc5)Cl)Br
ZINC08439910	6.36	-1.32	-4.9	0	3	0	445.769	2	c1cc(sc1)C2=NN3[C@@H](C2)c4cc(ccc4O[C@@H]3c5ccc(cc5)Cl)Br
ZINC08439909	6.05	-2.2	-9.26	0	4	0	518.23	3	c1cc(sc1)C2=NN3[C@H](C2)c4cc(ccc4O[C@@H]3C(=O)c5ccc(cc5)Br)Br
ZINC08439908	6.05	-1.91	-7.94	0	4	0	518.23	3	c1cc(sc1)C2=NN3[C@@H](C2)c4cc(ccc4O[C@@H]3C(=O)c5ccc(cc5)Br)Br
ZINC08439907	6.28	-3.66	-8.51	1	4	0	461.768	2	c1cc(sc1)C2=NN3[C@H](C2)c4cc(ccc4O[C@@H]3c5cc(ccc5O)Br)Cl
ZINC08439906	6.28	-2.32	-6.04	1	4	0	461.768	2	c1cc(sc1)C2=NN3[C@@H](C2)c4cc(ccc4O[C@@H]3c5cc(ccc5O)Br)Cl
ZINC08439904	5.84	0.65	-11.75	0	5	0	434.561	10	Cc1ccc(cc1)OCCSc2nc3ccccc3n2CCOc4ccccc4OC
ZINC08310925	6.6	14.31	-10.08	0	4	0	418.562	9	Cc1ccc(cc1C)OCCSc2nc3ccccc3n2CCOc4ccccc4
ZINC08439901	6.18	2.58	-16.82	2	13	0	503.427	7	Cc1ccc(cc1/N=C/2\c3cccc(c3C(=C2C(=O)C)O)[N+](=O)[O-])Nc4ccc(cc4[N+](=O)[O-])[N+](=O)[O-]
ZINC08439898	5.01	10.76	-89.44	0	6	-2	442.6	13	CCC/C(=N\CCCCCC/N=C(/C1=C(CCCC1=O)[O-])\CCC)/C2=C(CCCC2=O)[O-]
ZINC08439898	5.01	10.38	-89.95	0	6	-2	442.6	13	CCC/C(=N/CCCCCC/N=C(\C1=C(CCCC1=O)[O-])/CCC)/C2=C(CCCC2=O)[O-]
ZINC08439898	4.47	11.08	-46.62	1	6	-1	443.608	13	CCC/C(=N\CCCCCCNC(=C1C(=O)CCCC1=O)CCC)/C2=C(CCCC2=O)[O-]
ZINC08439898	4.47	11.07	-43.87	1	6	-1	443.608	13	CCC/C(=N/CCCCCCNC(=C1C(=O)CCCC1=O)CCC)/C2=C(CCCC2=O)[O-]
ZINC13286748	2.57	7.04	-15.85	1	4	0	288.13	6	COC(=O)CN/C=C\C(=O)c1ccc(c(c1)Cl)Cl
ZINC13286748	2.57	6.62	-10.31	1	4	0	288.13	6	COC(=O)CN/C=C/C(=O)c1ccc(c(c1)Cl)Cl
ZINC13286748	2.57	7.24	-51.69	2	4	1	289.138	6	COC(=O)C[NH2+]/C=C/C(=O)c1ccc(c(c1)Cl)Cl
ZINC00721236	4.95	1.87	-13.81	1	5	0	425.484	6	Cc1ccc(cc1)N2[C@H](C(=C(C2=O)O)C(=O)/C=C/c3ccccc3)c4ccc(cc4)OC
ZINC00721236	4.95	1.84	-22.04	1	5	0	425.484	5	Cc1ccc(cc1)N2[C@H](/C(=C(\C=C\c3ccccc3)/O)/C(=O)C2=O)c4ccc(cc4)OC
ZINC00721236	4.37	1.66	-22.26	0	5	0	425.484	6	Cc1ccc(cc1)N2[C@H]([C@H](C(=O)C2=O)C(=O)/C=C/c3ccccc3)c4ccc(cc4)OC
ZINC00721238	4.95	1.85	-13.87	1	5	0	425.484	6	Cc1ccc(cc1)N2[C@@H](C(=C(C2=O)O)C(=O)/C=C/c3ccccc3)c4ccc(cc4)OC
ZINC00721238	4.95	1.82	-22.05	1	5	0	425.484	5	Cc1ccc(cc1)N2[C@@H](/C(=C(\C=C\c3ccccc3)/O)/C(=O)C2=O)c4ccc(cc4)OC
ZINC00721238	4.37	1.63	-22.26	0	5	0	425.484	6	Cc1ccc(cc1)N2[C@@H]([C@@H](C(=O)C2=O)C(=O)/C=C/c3ccccc3)c4ccc(cc4)OC
ZINC09109830	4.09	10.08	-59.68	1	6	-1	463.872	5	CC(=O)Nc1ccc(cc1)N2[C@H](C(=C(C2=O)[O-])C(=O)c3ccc(cc3)Cl)c4ccccc4F
ZINC09109830	3.5	0.45	-15.78	1	6	0	464.88	5	CC(=O)Nc1ccc(cc1)N2[C@H]([C@@H](C(=O)C2=O)C(=O)c3ccc(cc3)Cl)c4ccccc4F
ZINC09109829	4.09	10.08	-59.34	1	6	-1	463.872	5	CC(=O)Nc1ccc(cc1)N2[C@@H](C(=C(C2=O)[O-])C(=O)c3ccc(cc3)Cl)c4ccccc4F
ZINC09109829	3.5	0.19	-16.24	1	6	0	464.88	5	CC(=O)Nc1ccc(cc1)N2[C@@H]([C@@H](C(=O)C2=O)C(=O)c3ccc(cc3)Cl)c4ccccc4F
ZINC09041905	3.4	1.01	-59.66	1	6	-1	416.812	6	c1ccc(c(c1)[C@H]2/C(=C(/c3ccc(cc3)Cl)\O)/C(=O)C(=O)N2CCCC(=O)[O-])F
ZINC09041905	2.38	0.71	-53.23	0	6	-1	416.812	7	c1ccc(c(c1)[C@H]2[C@H](C(=O)C(=O)N2CCCC(=O)[O-])C(=O)c3ccc(cc3)Cl)F
ZINC09122892	3.41	8.27	-60.91	1	6	-1	416.812	6	c1ccc(c(c1)[C@@H]2/C(=C(/c3ccc(cc3)Cl)\O)/C(=O)C(=O)N2CCCC(=O)[O-])F
ZINC09008533	3.45	0.81	-59.73	1	6	-1	416.812	6	c1cc(ccc1[C@H]2/C(=C(/c3ccc(cc3)Cl)\O)/C(=O)C(=O)N2CCCC(=O)[O-])F
ZINC09072303	3.45	9.29	-59.87	1	6	-1	416.812	6	c1cc(ccc1[C@@H]2/C(=C(/c3ccc(cc3)Cl)\O)/C(=O)C(=O)N2CCCC(=O)[O-])F
ZINC00721191	5.94	0.06	-9.37	0	3	0	437.362	6	c1ccc(cc1)Cn2c3ccc(cc3nc2SCC(=O)c4ccccc4)Br
ZINC08439892	4.42	-4	-8.27	1	8	0	443.475	6	CCN1c2cc(c(cc2N[C@H]3[C@@H]1OC(=C3C(=O)OCc4ccco4)C)F)N5CCOCC5
ZINC08439891	4.42	-4.59	-8.17	1	8	0	443.475	6	CCN1c2cc(c(cc2N[C@@H]3[C@@H]1OC(=C3C(=O)OCc4ccco4)C)F)N5CCOCC5
ZINC08439890	6.58	-3.51	-7.41	1	7	0	501.643	6	CCN1c2cc(c(cc2N[C@H]3[C@@H]1OC(=C3C(=O)OC4CCC(CC4)C(C)(C)C)C)F)N5CCOCC5
ZINC08439889	6.58	-3.85	-5.26	1	7	0	501.643	6	CCN1c2cc(c(cc2N[C@@H]3[C@@H]1OC(=C3C(=O)OC4CCC(CC4)C(C)(C)C)C)F)N5CCOCC5
ZINC08439888	7.49	13.99	-49.31	0	4	-1	489.035	4	CC1(CC2=C([C@H](C3=C(CC(CC3=N2)(C)C)[O-])c4ccc(cc4)OCc5ccc(cc5)Cl)C(=O)C1)C
ZINC08439888	7.07	0.82	-13.04	0	4	0	490.043	4	CC1(CC2=NC3=CC(CC(=O)C3[C@@H](C2C(=O)C1)c4ccc(cc4)OCc5ccc(cc5)Cl)(C)C)C
ZINC08439888	7.07	2.04	-20.69	0	4	0	490.043	4	CC1(CC2=NC3=CC(CC(=O)C3[C@@H]([C@@H]2C(=O)C1)c4ccc(cc4)OCc5ccc(cc5)Cl)(C)C)C
ZINC08439888	6.91	1.07	-15.78	0	4	0	490.043	4	CC1(CC2=NC3=C([C@@H]([C@H]2C(=O)C1)c4ccc(cc4)OCc5ccc(cc5)Cl)C(=O)CC(C3)(C)C)C
ZINC00720885	4.58	-1.79	-12.85	2	8	0	470.529	6	CC1=C([C@@H](Nc2n1c3ccccc3n2)c4ccc(cc4)OC)C(=O)Nc5ccc(cc5OC)OC
ZINC00717892	4.1	11.33	-38.22	0	6	-1	477.718	7	CCOC(=O)CN1[C@H](C(=C(C1=O)[O-])C(=O)c2ccc(cc2)Cl)c3ccc(cc3)Br
ZINC00717892	4.55	-0.18	-30.79	1	6	0	478.726	6	CCOC(=O)CN1[C@H](C(=C(c2ccc(cc2)Cl)O)C(=O)C1=O)c3ccc(cc3)Br
ZINC00717892	3.52	0.08	-25.08	0	6	0	478.726	7	CCOC(=O)CN1[C@H]([C@@H](C(=O)C1=O)C(=O)c2ccc(cc2)Cl)c3ccc(cc3)Br
ZINC00717893	4.1	11.37	-37.54	0	6	-1	477.718	7	CCOC(=O)CN1[C@@H](C(=C(C1=O)[O-])C(=O)c2ccc(cc2)Cl)c3ccc(cc3)Br
ZINC00717893	4.55	-0.16	-31.18	1	6	0	478.726	6	CCOC(=O)CN1[C@@H](C(=C(c2ccc(cc2)Cl)O)C(=O)C1=O)c3ccc(cc3)Br
ZINC00717893	3.52	0.08	-25.35	0	6	0	478.726	7	CCOC(=O)CN1[C@@H]([C@H](C(=O)C1=O)C(=O)c2ccc(cc2)Cl)c3ccc(cc3)Br
ZINC08439886	3.4	2.62	-17.49	1	12	0	503.519	8	Cn1c2c(nc1N(CC(=O)Nc3ccccc3C(=O)OC)/N=C/c4ccccc4)n(c(=O)n(c2=O)C)C
ZINC08439886	3.4	2.87	-37.22	2	12	1	504.527	8	Cn1c2c([nH+]c1N(CC(=O)Nc3ccccc3C(=O)OC)/N=C/c4ccccc4)n(c(=O)n(c2=O)C)C
ZINC00717758	4.71	-0.02	-11.17	1	4	0	423.921	6	c1ccc(cc1)CCN2[C@H](C(=C(C2=O)O)C(=O)c3cccs3)c4ccccc4Cl
ZINC00717758	4.13	-0.2	-14.91	0	4	0	423.921	6	c1ccc(cc1)CCN2[C@H]([C@@H](C(=O)C2=O)C(=O)c3cccs3)c4ccccc4Cl
ZINC00717759	4.71	-0.12	-10.92	1	4	0	423.921	6	c1ccc(cc1)CCN2[C@@H](C(=C(C2=O)O)C(=O)c3cccs3)c4ccccc4Cl
ZINC00717759	4.13	-0.09	-17.14	0	4	0	423.921	6	c1ccc(cc1)CCN2[C@@H]([C@H](C(=O)C2=O)C(=O)c3cccs3)c4ccccc4Cl
ZINC08439885	5.06	1.66	-13.02	0	8	0	567.704	12	CCSCCOC(=O)C1[C@@H](C2=C(C[C@@H](CC2=O)c3ccccc3OC)N=C1C)c4cc(c(c(c4)OC)OC)OC
ZINC08439885	5.06	1.31	-16.62	0	8	0	567.704	12	CCSCCOC(=O)C1=C(N=C2C[C@@H](CC(=O)C2[C@H]1c3cc(c(c(c3)OC)OC)OC)c4ccccc4OC)C
ZINC08439885	5.24	0.39	-14.4	0	8	0	567.704	12	CCSCCOC(=O)C1[C@@H](C2C(=NC1=C)C[C@@H](CC2=O)c3ccccc3OC)c4cc(c(c(c4)OC)OC)OC
ZINC08439884	5.06	1.69	-12.98	0	8	0	567.704	12	CCSCCOC(=O)C1[C@H](C2=C(C[C@@H](CC2=O)c3ccccc3OC)N=C1C)c4cc(c(c(c4)OC)OC)OC
ZINC08439884	5.06	1.43	-15.21	0	8	0	567.704	12	CCSCCOC(=O)C1=C(N=C2C[C@@H](CC(=O)C2[C@@H]1c3cc(c(c(c3)OC)OC)OC)c4ccccc4OC)C

Supplementary Table S2:
Detailed table of XP Docking using Schrodinger's Glide module

ZINC_ID	xLogP	ApolarDesolvation	PolarDesolvation	H Donors	H Acceptors	Charge	MolecularWeight	RotatableBonds	SMILES
ZINC08439884	5.24	0.56	-13.68	0	8	0	567.704	12	CCSCCOC(=O)C1[C@H](C2C(=NC1=C)C[C@@H](CC2=O)c3ccccc3OC)c4cc(c(c(c4)OC)OC)OC
ZINC08439883	5.06	1.65	-15.5	0	8	0	567.704	12	CCSCCOC(=O)C1[C@@H](C2=C(C[C@H](CC2=O)c3ccccc3OC)N=C1C)c4cc(c(c(c4)OC)OC)OC
ZINC08439883	5.06	1.36	-16.45	0	8	0	567.704	12	CCSCCOC(=O)C1=C(N=C2C[C@H](CC(=O)C2[C@H]1c3cc(c(c(c3)OC)OC)OC)c4ccccc4OC)C
ZINC08439883	5.24	0.35	-14.24	0	8	0	567.704	12	CCSCCOC(=O)C1[C@@H](C2C(=NC1=C)C[C@H](CC2=O)c3ccccc3OC)c4cc(c(c(c4)OC)OC)OC
ZINC08439882	5.06	1.68	-13.68	0	8	0	567.704	12	CCSCCOC(=O)C1[C@H](C2=C(C[C@H](CC2=O)c3ccccc3OC)N=C1C)c4cc(c(c(c4)OC)OC)OC
ZINC08439882	5.06	1.33	-16.73	0	8	0	567.704	12	CCSCCOC(=O)C1=C(N=C2C[C@H](CC(=O)C2[C@@H]1c3cc(c(c(c3)OC)OC)OC)c4ccccc4OC)C
ZINC08439882	5.24	0.45	-14.86	0	8	0	567.704	12	CCSCCOC(=O)C1[C@H](C2C(=NC1=C)C[C@H](CC2=O)c3ccccc3OC)c4cc(c(c(c4)OC)OC)OC
ZINC08439881	4.82	2.87	-14.46	0	7	0	489.568	8	CC1=NC2=C([C@@H](C1C(=O)OC(C)C)c3ccc(cc3)OC(=O)C)C(=O)C[C@@H](C2)c4ccccc4OC
ZINC08439881	5	2.93	-15.29	0	7	0	489.568	8	CC(C)OC(=O)C1[C@H](C2C(=NC1=C)C[C@H](CC2=O)c3ccccc3OC)c4ccc(cc4)OC(=O)C
ZINC08439881	5	4.33	-21.04	0	7	0	489.568	8	CC(C)OC(=O)C1[C@H]([C@@H]2C(=NC1=C)C[C@H](CC2=O)c3ccccc3OC)c4ccc(cc4)OC(=O)C
ZINC08439881	4.82	2.8	-16.76	0	7	0	489.568	8	CC1=C([C@H]([C@@H]2C(=N1)C[C@H](CC2=O)c3ccccc3OC)c4ccc(cc4)OC(=O)C)C(=O)OC(C)C
ZINC08439880	4.82	2.85	-12.74	0	7	0	489.568	8	CC1=NC2=C([C@H](C1C(=O)OC(C)C)c3ccc(cc3)OC(=O)C)C(=O)C[C@@H](C2)c4ccccc4OC
ZINC08439880	5	2.98	-13.82	0	7	0	489.568	8	CC(C)OC(=O)C1[C@@H](C2C(=NC1=C)C[C@H](CC2=O)c3ccccc3OC)c4ccc(cc4)OC(=O)C
ZINC08439880	5	4.25	-15.15	0	7	0	489.568	8	CC(C)OC(=O)C1[C@@H]([C@H]2C(=NC1=C)C[C@H](CC2=O)c3ccccc3OC)c4ccc(cc4)OC(=O)C
ZINC08439880	4.82	2.87	-16.24	0	7	0	489.568	8	CC1=C([C@@H]([C@H]2C(=N1)C[C@H](CC2=O)c3ccccc3OC)c4ccc(cc4)OC(=O)C)C(=O)OC(C)C
ZINC08439879	4.82	2.79	-13.76	0	7	0	489.568	8	CC1=NC2=C([C@@H](C1C(=O)OC(C)C)c3ccc(cc3)OC(=O)C)C(=O)C[C@H](C2)c4ccccc4OC
ZINC08439879	5	2.93	-13.59	0	7	0	489.568	8	CC(C)OC(=O)C1[C@H](C2C(=NC1=C)C[C@@H](CC2=O)c3ccccc3OC)c4ccc(cc4)OC(=O)C
ZINC08439879	5	4.2	-14.84	0	7	0	489.568	8	CC(C)OC(=O)C1[C@H]([C@@H]2C(=NC1=C)C[C@@H](CC2=O)c3ccccc3OC)c4ccc(cc4)OC(=O)C
ZINC08439879	4.82	3	-15.01	0	7	0	489.568	8	CC1=C([C@H]([C@H]2C(=N1)C[C@@H](CC2=O)c3ccccc3OC)c4ccc(cc4)OC(=O)C)C(=O)OC(C)C
ZINC08439878	4.82	2.98	-15.35	0	7	0	489.568	8	CC1=NC2=C([C@H](C1C(=O)OC(C)C)c3ccc(cc3)OC(=O)C)C(=O)C[C@H](C2)c4ccccc4OC
ZINC08439878	5	3.02	-14.7	0	7	0	489.568	8	CC(C)OC(=O)C1[C@@H](C2C(=NC1=C)C[C@@H](CC2=O)c3ccccc3OC)c4ccc(cc4)OC(=O)C
ZINC08439878	4.82	3.01	-16.44	0	7	0	489.568	8	CC1=C([C@@H]([C@@H]2C(=N1)C[C@@H](CC2=O)c3ccccc3OC)c4ccc(cc4)OC(=O)C)C(=O)OC(C)C
ZINC08439877	5.32	0.43	-11.09	0	6	0	512.4	6	CC1=NC2=C([C@@H](C1C(=O)OC)c3ccc(c(c3)Br)OC)C(=O)C[C@@H](C2)c4ccccc4OC
ZINC08439877	5.5	0.69	-11.96	0	6	0	512.4	6	COc1ccccc1[C@@H]2CC3=NC(=C)C([C@H](C3C(=O)C2)c4ccc(c(c4)Br)OC)C(=O)OC
ZINC08439877	5.5	1.73	-12.13	0	6	0	512.4	6	COc1ccccc1[C@@H]2CC3=NC(=C)C([C@H]([C@H]3C(=O)C2)c4ccc(c(c4)Br)OC)C(=O)OC
ZINC08439877	5.32	0.53	-12.81	0	6	0	512.4	6	CC1=C([C@H]([C@H]2C(=N1)C[C@H](CC2=O)c3ccccc3OC)c4ccc(c(c4)Br)OC)C(=O)OC
ZINC08439876	5.32	0.39	-10.23	0	6	0	512.4	6	CC1=NC2=C([C@H](C1C(=O)OC)c3ccc(c(c3)Br)OC)C(=O)C[C@@H](C2)c4ccccc4OC
ZINC08439876	5.5	0.52	-11.19	0	6	0	512.4	6	COc1ccccc1[C@@H]2CC3=NC(=C)C([C@@H](C3C(=O)C2)c4ccc(c(c4)Br)OC)C(=O)OC
ZINC08439876	5.5	1.71	-17.39	0	6	0	512.4	6	COc1ccccc1[C@@H]2CC3=NC(=C)C([C@@H]([C@@H]3C(=O)C2)c4ccc(c(c4)Br)OC)C(=O)OC
ZINC08439876	5.32	0.4	-12.43	0	6	0	512.4	6	CC1=C([C@@H]([C@H]2C(=N1)C[C@H](CC2=O)c3ccccc3OC)c4ccc(c(c4)Br)OC)C(=O)OC
ZINC08439875	5.32	0.32	-10.77	0	6	0	512.4	6	CC1=NC2=C([C@@H](C1C(=O)OC)c3ccc(c(c3)Br)OC)C(=O)C[C@H](C2)c4ccccc4OC
ZINC08439875	5.5	0.45	-11.22	0	6	0	512.4	6	COc1ccccc1[C@H]2CC3=NC(=C)C([C@H](C3C(=O)C2)c4ccc(c(c4)Br)OC)C(=O)OC
ZINC08439875	5.5	1.56	-12.08	0	6	0	512.4	6	COc1ccccc1[C@H]2CC3=NC(=C)C([C@H]([C@H]3C(=O)C2)c4ccc(c(c4)Br)OC)C(=O)OC
ZINC08439875	5.32	0.45	-13.24	0	6	0	512.4	6	CC1=C([C@H]([C@H]2C(=N1)C[C@@H](CC2=O)c3ccccc3OC)c4ccc(c(c4)Br)OC)C(=O)OC
ZINC08439874	5.32	0.39	-11.21	0	6	0	512.4	6	CC1=NC2=C([C@H](C1C(=O)OC)c3ccc(c(c3)Br)OC)C(=O)C[C@H](C2)c4ccccc4OC
ZINC08439874	5.5	0.62	-11.96	0	6	0	512.4	6	COc1ccccc1[C@H]2CC3=NC(=C)C([C@@H](C3C(=O)C2)c4ccc(c(c4)Br)OC)C(=O)OC
ZINC08439874	5.5	1.61	-11.46	0	6	0	512.4	6	COc1ccccc1[C@H]2CC3=NC(=C)C([C@@H]([C@@H]3C(=O)C2)c4ccc(c(c4)Br)OC)C(=O)OC
ZINC08439874	5.32	0.51	-12.88	0	6	0	512.4	6	CC1=C([C@@H]([C@@H]2C(=N1)C[C@@H](CC2=O)c3ccccc3OC)c4ccc(c(c4)Br)OC)C(=O)OC
ZINC00717718	5.55	2.13	-12.68	0	7	0	500.356	2	Cn1c2c(c(=O)n(c1=O)C)n3cc(n(c3n2)c4cccc5c4cccc5)c6ccc(cc6)Br
ZINC08439872	2.03	-2.91	-10.48	1	9	0	433.266	3	Cn1c2c(c(=O)n(c1=O)C)n3c(n2)N(N=C(C3)c4ccc(cc4)Br)CCO
ZINC08439872	2.03	-2.62	-38.67	2	9	1	434.274	3	Cn1c2c(c(=O)n(c1=O)C)n3c([nH+]2)N(N=C(C3)c4ccc(cc4)Br)CCO
ZINC08439871	4.36	1.12	-9.85	0	8	0	465.311	2	Cn1c2c(c(=O)n(c1=O)C)n3c(n2)N(N=C(C3)c4ccc(cc4)Br)c5ccccc5
ZINC08439871	4.36	1.25	-32.13	1	8	1	466.319	2	Cn1c2c(c(=O)n(c1=O)C)n3c([nH+]2)N(N=C(C3)c4ccc(cc4)Br)c5ccccc5
ZINC08439870	5.17	1.16	-10.6	0	7	0	511.64	9	CC1=NC2=C([C@@H](C1C(=O)OCC(C)C)c3cc(c(c(c3)OC)OC)OC)C(=O)C[C@@H](C2)c4cccs4
ZINC08439870	5.35	1.39	-11.05	0	7	0	511.64	9	CC(C)COC(=O)C1[C@H](C2C(=NC1=C)C[C@H](CC2=O)c3cccs3)c4cc(c(c(c4)OC)OC)OC
ZINC08439870	5.35	2.38	-11.03	0	7	0	511.64	9	CC(C)COC(=O)C1[C@H]([C@@H]2C(=NC1=C)C[C@H](CC2=O)c3cccs3)c4cc(c(c(c4)OC)OC)OC
ZINC08439870	5.17	1.27	-12.93	0	7	0	511.64	9	CC1=C([C@H]([C@H]2C(=N1)C[C@H](CC2=O)c3cccs3)c4cc(c(c(c4)OC)OC)OC)C(=O)OCC(C)C
ZINC08439869	5.17	1.14	-10.61	0	7	0	511.64	9	CC1=NC2=C([C@H](C1C(=O)OCC(C)C)c3cc(c(c(c3)OC)OC)OC)C(=O)C[C@@H](C2)c4cccs4
ZINC08439869	5.35	1.14	-11.77	0	7	0	511.64	9	CC(C)COC(=O)C1[C@@H](C2C(=NC1=C)C[C@H](CC2=O)c3cccs3)c4cc(c(c(c4)OC)OC)OC
ZINC08439869	5.35	2.36	-10.88	0	7	0	511.64	9	CC(C)COC(=O)C1[C@@H]([C@H]2C(=NC1=C)C[C@H](CC2=O)c3cccs3)c4cc(c(c(c4)OC)OC)OC
ZINC08439869	5.17	1.19	-13.37	0	7	0	511.64	9	CC1=C([C@@H]([C@@H]2C(=N1)C[C@H](CC2=O)c3cccs3)c4cc(c(c(c4)OC)OC)OC)C(=O)OCC(C)C
ZINC08439868	5.17	1.36	-13.33	0	7	0	511.64	9	CC1=NC2=C([C@@H](C1C(=O)OCC(C)C)c3cc(c(c(c3)OC)OC)OC)C(=O)C[C@H](C2)c4cccs4
ZINC08439868	5.35	1.5	-12.39	0	7	0	511.64	9	CC(C)COC(=O)C1[C@H](C2C(=NC1=C)C[C@@H](CC2=O)c3cccs3)c4cc(c(c(c4)OC)OC)OC
ZINC08439868	5.35	2.65	-16.27	0	7	0	511.64	9	CC(C)COC(=O)C1[C@H]([C@@H]2C(=NC1=C)C[C@@H](CC2=O)c3cccs3)c4cc(c(c(c4)OC)OC)OC
ZINC08439868	5.17	1.24	-13.83	0	7	0	511.64	9	CC1=C([C@H]([C@H]2C(=N1)C[C@@H](CC2=O)c3cccs3)c4cc(c(c(c4)OC)OC)OC)C(=O)OCC(C)C
ZINC08439867	5.17	1.14	-10.43	0	7	0	511.64	9	CC1=NC2=C([C@H](C1C(=O)OCC(C)C)c3cc(c(c(c3)OC)OC)OC)C(=O)C[C@H](C2)c4cccs4
ZINC08439867	5.35	1.2	-11.46	0	7	0	511.64	9	CC(C)COC(=O)C1[C@@H](C2C(=NC1=C)C[C@@H](CC2=O)c3cccs3)c4cc(c(c(c4)OC)OC)OC
ZINC08439867	5.35	2.62	-16.9	0	7	0	511.64	9	CC(C)COC(=O)C1[C@@H]([C@H]2C(=NC1=C)C[C@@H](CC2=O)c3cccs3)c4cc(c(c(c4)OC)OC)OC
ZINC08439867	5.17	1.08	-13.74	0	7	0	511.64	9	CC1=C([C@@H]([C@H]2C(=N1)C[C@@H](CC2=O)c3cccs3)c4cc(c(c(c4)OC)OC)OC)C(=O)OCC(C)C
ZINC08439866	4.98	0.24	-11.21	0	7	0	523.651	8	CC1=NC2=C([C@@H](C1C(=O)OC3CCCC3)c4cc(c(cc4OC)OC)OC)C(=O)C[C@@H](C2)c5cccs5
ZINC08439866	5.16	0.5	-12.15	0	7	0	523.651	8	COc1cc(c(cc1OC)OC)[C@H]2C3C(=NC(=C)C2C(=O)OC4CCCC4)C[C@H](CC3=O)c5cccs5

Supplementary Table S2:

Detailed table of XP Docking using Schrodinger's Glide module

ZINC_ID	xLogP	ApolarDesolvation	PolarDesolvation	H Donors	H Acceptors	Charge	MolecularWeight	RotatableBonds	SMILES
ZINC08439866	5.16	1.54	-12.65	0	7	0	523.651	8	COc1cc(c(cc1OC)OC)[C@H]2[C@@H]3C(=NC(=C)C2C(=O)OC4CCCC4)C[C@H](CC3=O)c5cccs5
ZINC08439866	4.98	0.34	-13.83	0	7	0	523.651	8	CC1=C([C@H]([C@H]2C(=N1)C[C@H](CC2=O)c3cccs3)c4cc(c(cc4OC)OC)OC)C(=O)OC5CCCC5
ZINC08439865	4.98	0.32	-14.77	0	7	0	523.651	8	CC1=NC2=C([C@H](C1C(=O)OC3CCCC3)c4cc(c(cc4OC)OC)OC)C(=O)C[C@@H](C2)c5cccs5
ZINC08439865	5.16	0.44	-14.13	0	7	0	523.651	8	COc1cc(c(cc1OC)OC)[C@@H]2C3C(=NC(=C)C2C(=O)OC4CCCC4)C[C@H](CC3=O)c5cccs5
ZINC08439865	5.16	1.58	-15.56	0	7	0	523.651	8	COc1cc(c(cc1OC)OC)[C@@H]2[C@H]3C(=NC(=C)C2C(=O)OC4CCCC4)C[C@H](CC3=O)c5cccs5
ZINC08439865	4.98	0.18	-15.74	0	7	0	523.651	8	CC1=C([C@@H]([C@H]2C(=N1)C[C@H](CC2=O)c3cccs3)c4cc(c(cc4OC)OC)OC)C(=O)OC5CCCC5
ZINC08439864	4.98	0.13	-11.52	0	7	0	523.651	8	CC1=NC2=C([C@@H](C1C(=O)OC3CCCC3)c4cc(c(cc4OC)OC)OC)C(=O)C[C@H](C2)c5cccs5
ZINC08439864	5.16	0.26	-11.93	0	7	0	523.651	8	COc1cc(c(cc1OC)OC)[C@H]2C3C(=NC(=C)C2C(=O)OC4CCCC4)C[C@@H](CC3=O)c5cccs5
ZINC08439864	5.16	1.38	-12.86	0	7	0	523.651	8	COc1cc(c(cc1OC)OC)[C@H]2[C@@H]3C(=NC(=C)C2C(=O)OC4CCCC4)C[C@@H](CC3=O)c5cccs5
ZINC08439864	4.98	0.26	-13.87	0	7	0	523.651	8	CC1=C([C@H]([C@H]2C(=N1)C[C@@H](CC2=O)c3cccs3)c4cc(c(cc4OC)OC)OC)C(=O)OC5CCCC5
ZINC08439863	4.98	0.21	-11.29	0	7	0	523.651	8	CC1=NC2=C([C@H](C1C(=O)OC3CCCC3)c4cc(c(cc4OC)OC)OC)C(=O)C[C@H](C2)c5cccs5
ZINC08439863	5.16	0.27	-12.07	0	7	0	523.651	8	COc1cc(c(cc1OC)OC)[C@@H]2C3C(=NC(=C)C2C(=O)OC4CCCC4)C[C@@H](CC3=O)c5cccs5
ZINC08439863	5.16	1.42	-13.5	0	7	0	523.651	8	COc1cc(c(cc1OC)OC)[C@@H]2[C@H]3C(=NC(=C)C2C(=O)OC4CCCC4)C[C@@H](CC3=O)c5cccs5
ZINC08439863	4.98	0.16	-14.49	0	7	0	523.651	8	CC1=C([C@@H]([C@H]2C(=N1)C[C@@H](CC2=O)c3cccs3)c4cc(c(cc4OC)OC)OC)C(=O)OC5CCCC5
ZINC08439862	5.75	-0.22	-12.14	0	4	0	550.515	8	CCSCCOC(=O)C1[C@@H](C2=C(C[C@@H](CC2=O)c3cccs3)N=C1C)c4cc(ccc4F)Br
ZINC08439862	5.75	-0.4	-12.86	0	4	0	550.515	8	CCSCCOC(=O)C1=C(N=C2C[C@@H](CC(=O)C2[C@H]1c3cc(ccc3F)Br)c4cccs4)C
ZINC08439862	5.93	-1.27	-11.52	0	4	0	550.515	8	CCSCCOC(=O)C1[C@@H](C2C(=NC1=C)C[C@@H](CC2=O)c3cccs3)c4cc(ccc4F)Br
ZINC08439861	5.75	-0.34	-7.78	0	4	0	550.515	8	CCSCCOC(=O)C1[C@H](C2=C(C[C@@H](CC2=O)c3cccs3)N=C1C)c4cc(ccc4F)Br
ZINC08439861	5.75	-0.42	-10.21	0	4	0	550.515	8	CCSCCOC(=O)C1=C(N=C2C[C@@H](CC(=O)C2[C@@H]1c3cc(ccc3F)Br)c4cccs4)C
ZINC08439861	5.93	-1.28	-8.8	0	4	0	550.515	8	CCSCCOC(=O)C1[C@H](C2C(=NC1=C)C[C@@H](CC2=O)c3cccs3)c4cc(ccc4F)Br
ZINC08439860	5.75	-0.18	-7.97	0	4	0	550.515	8	CCSCCOC(=O)C1[C@@H](C2=C(C[C@H](CC2=O)c3cccs3)N=C1C)c4cc(ccc4F)Br
ZINC08439860	5.75	-0.44	-10.46	0	4	0	550.515	8	CCSCCOC(=O)C1=C(N=C2C[C@H](CC(=O)C2[C@H]1c3cc(ccc3F)Br)c4cccs4)C
ZINC08439860	5.93	-1.57	-8.58	0	4	0	550.515	8	CCSCCOC(=O)C1[C@@H](C2C(=NC1=C)C[C@H](CC2=O)c3cccs3)c4cc(ccc4F)Br
ZINC08439859	5.75	-0.17	-7.98	0	4	0	550.515	8	CCSCCOC(=O)C1[C@H](C2=C(C[C@H](CC2=O)c3cccs3)N=C1C)c4cc(ccc4F)Br
ZINC08439859	5.75	-0.35	-10.58	0	4	0	550.515	8	CCSCCOC(=O)C1=C(N=C2C[C@H](CC(=O)C2[C@@H]1c3cc(ccc3F)Br)c4cccs4)C
ZINC08439859	5.93	-1.27	-8.99	0	4	0	550.515	8	CCSCCOC(=O)C1[C@H](C2C(=NC1=C)C[C@H](CC2=O)c3cccs3)c4cc(ccc4F)Br
ZINC08439858	6.21	2	-10.06	0	4	0	521.626	9	CCSCCOC(=O)C1[C@@H](C2=C(C[C@@H](CC2=O)c3cccs3)N=C1C)c4ccc(cc4)C(F)(F)F
ZINC08439858	6.21	1.65	-10.68	0	4	0	521.626	9	CCSCCOC(=O)C1=C(N=C2C[C@@H](CC(=O)C2[C@H]1c3ccc(cc3)C(F)(F)F)c4cccs4)C
ZINC08439858	6.4	0.77	-9.38	0	4	0	521.626	9	CCSCCOC(=O)C1[C@@H](C2C(=NC1=C)C[C@@H](CC2=O)c3cccs3)c4ccc(cc4)C(F)(F)F
ZINC08439857	6.21	1.88	-7.64	0	4	0	521.626	9	CCSCCOC(=O)C1[C@H](C2=C(C[C@@H](CC2=O)c3cccs3)N=C1C)c4ccc(cc4)C(F)(F)F
ZINC08439857	6.21	1.8	-9.88	0	4	0	521.626	9	CCSCCOC(=O)C1=C(N=C2C[C@@H](CC(=O)C2[C@@H]1c3ccc(cc3)C(F)(F)F)c4cccs4)C
ZINC08439857	6.4	0.75	-8.67	0	4	0	521.626	9	CCSCCOC(=O)C1[C@H](C2C(=NC1=C)C[C@@H](CC2=O)c3cccs3)c4ccc(cc4)C(F)(F)F
ZINC08439856	6.21	2.03	-7.33	0	4	0	521.626	9	CCSCCOC(=O)C1[C@@H](C2=C(C[C@H](CC2=O)c3cccs3)N=C1C)c4ccc(cc4)C(F)(F)F
ZINC08439856	6.21	1.78	-9.98	0	4	0	521.626	9	CCSCCOC(=O)C1=C(N=C2C[C@H](CC(=O)C2[C@H]1c3ccc(cc3)C(F)(F)F)c4cccs4)C
ZINC08439856	6.4	0.85	-8.66	0	4	0	521.626	9	CCSCCOC(=O)C1[C@@H](C2C(=NC1=C)C[C@H](CC2=O)c3cccs3)c4ccc(cc4)C(F)(F)F
ZINC08439855	6.21	2.04	-7.26	0	4	0	521.626	9	CCSCCOC(=O)C1[C@H](C2=C(C[C@H](CC2=O)c3cccs3)N=C1C)c4ccc(cc4)C(F)(F)F
ZINC08439855	6.21	1.87	-10.01	0	4	0	521.626	9	CCSCCOC(=O)C1=C(N=C2C[C@H](CC(=O)C2[C@@H]1c3ccc(cc3)C(F)(F)F)c4cccs4)C
ZINC08439855	6.4	0.95	-8.73	0	4	0	521.626	9	CCSCCOC(=O)C1[C@H](C2C(=NC1=C)C[C@H](CC2=O)c3cccs3)c4ccc(cc4)C(F)(F)F
ZINC08439854	5.56	1.54	-13.09	0	8	0	547.648	9	CC1=NC2=C([C@@H](C1C(=O)OC3CCCC3)c4cc(c(c(c4)OC)OC)OC)C(=O)C[C@@H](C2)c5ccccc5OC
ZINC08439854	5.74	1.82	-13.13	0	8	0	547.648	9	COc1ccccc1[C@@H]2CC3=NC(=C)C([C@H](C3C(=O)C2)c4cc(c(c(c4)OC)OC)OC)C(=O)OC5CCCC5
ZINC08439854	5.74	2.85	-14.36	0	8	0	547.648	9	COc1ccccc1[C@@H]2CC3=NC(=C)C([C@H]([C@H]3C(=O)C2)c4cc(c(c(c4)OC)OC)OC)C(=O)OC5CCCC5
ZINC08439854	5.56	1.65	-14.79	0	8	0	547.648	9	CC1=C([C@H]([C@H]2C(=N1)C[C@H](CC2=O)c3ccccc3OC)c4cc(c(c(c4)OC)OC)OC)C(=O)OC5CCCC5
ZINC08439853	5.56	1.55	-12.38	0	8	0	547.648	9	CC1=NC2=C([C@H](C1C(=O)OC3CCCC3)c4cc(c(c(c4)OC)OC)OC)C(=O)C[C@@H](C2)c5ccccc5OC
ZINC08439853	5.74	1.3	-12.9	0	8	0	547.648	9	COc1ccccc1[C@@H]2CC3=NC(=C)C([C@@H](C3C(=O)C2)c4cc(c(c(c4)OC)OC)OC)C(=O)OC5CCCC5
ZINC08439853	5.74	2.75	-20.02	0	8	0	547.648	9	COc1ccccc1[C@@H]2CC3=NC(=C)C([C@@H]([C@@H]3C(=O)C2)c4cc(c(c(c4)OC)OC)OC)C(=O)OC5CCCC5
ZINC08439853	5.56	1.65	-15.29	0	8	0	547.648	9	CC1=C([C@@H]([C@@H]2C(=N1)C[C@H](CC2=O)c3ccccc3OC)c4cc(c(c(c4)OC)OC)OC)C(=O)OC5CCCC5
ZINC08439852	5.56	1.52	-12.49	0	8	0	547.648	9	CC1=NC2=C([C@@H](C1C(=O)OC3CCCC3)c4cc(c(c(c4)OC)OC)OC)C(=O)C[C@H](C2)c5ccccc5OC
ZINC08439852	5.74	1.66	-12.88	0	8	0	547.648	9	COc1ccccc1[C@H]2CC3=NC(=C)C([C@H](C3C(=O)C2)c4cc(c(c(c4)OC)OC)OC)C(=O)OC5CCCC5
ZINC08439852	5.74	2.91	-14.6	0	8	0	547.648	9	COc1ccccc1[C@H]2CC3=NC(=C)C([C@H]([C@H]3C(=O)C2)c4cc(c(c(c4)OC)OC)OC)C(=O)OC5CCCC5
ZINC08439852	5.56	1.54	-14.99	0	8	0	547.648	9	CC1=C([C@H]([C@@H]2C(=N1)C[C@@H](CC2=O)c3ccccc3OC)c4cc(c(c(c4)OC)OC)OC)C(=O)OC5CCCC5
ZINC08439851	5.56	1.53	-12.81	0	8	0	547.648	9	CC1=NC2=C([C@H](C1C(=O)OC3CCCC3)c4cc(c(c(c4)OC)OC)OC)C(=O)C[C@H](C2)c5ccccc5OC
ZINC08439851	5.74	1.75	-13.35	0	8	0	547.648	9	COc1ccccc1[C@H]2CC3=NC(=C)C([C@@H](C3C(=O)C2)c4cc(c(c(c4)OC)OC)OC)C(=O)OC5CCCC5
ZINC08439851	5.74	2.74	-13.12	0	8	0	547.648	9	COc1ccccc1[C@H]2CC3=NC(=C)C([C@@H]([C@@H]3C(=O)C2)c4cc(c(c(c4)OC)OC)OC)C(=O)OC5CCCC5
ZINC08439851	5.56	1.63	-15.34	0	8	0	547.648	9	CC1=C([C@@H]([C@@H]2C(=N1)C[C@@H](CC2=O)c3ccccc3OC)c4cc(c(c(c4)OC)OC)OC)C(=O)OC5CCCC5
ZINC08439850	6.25	-0.42	-7.69	0	4	0	530.459	5	CC1=NC2=C([C@H](C1C(=O)OC3CCCC3)c4cc(ccc4F)Br)C(=O)C[C@@H](C2)c5cccs5
ZINC08439850	6.43	-0.32	-7.93	0	4	0	530.459	5	C=C1C([C@@H](C2C(=N1)C[C@H](CC2=O)c3cccs3)c4cc(ccc4F)Br)C(=O)OC5CCCC5
ZINC08439850	6.43	0.99	-15.99	0	4	0	530.459	5	C=C1C([C@@H]([C@H]2C(=N1)C[C@H](CC2=O)c3cccs3)c4cc(ccc4F)Br)C(=O)OC5CCCC5
ZINC08439850	6.25	-0.28	-10.09	0	4	0	530.459	5	CC1=C([C@@H]([C@@H]2C(=N1)C[C@H](CC2=O)c3cccs3)c4cc(ccc4F)Br)C(=O)OC5CCCC5
ZINC08439849	6.25	-0.42	-7.54	0	4	0	530.459	5	CC1=NC2=C([C@@H](C1C(=O)OC3CCCC3)c4cc(ccc4F)Br)C(=O)C[C@H](C2)c5cccs5
ZINC08439849	6.43	-0.33	-8.04	0	4	0	530.459	5	C=C1C([C@H](C2C(=N1)C[C@@H](CC2=O)c3cccs3)c4cc(ccc4F)Br)C(=O)OC5CCCC5
ZINC08439849	6.43	0.74	-8	0	4	0	530.459	5	C=C1C([C@H]([C@@H]2C(=N1)C[C@@H](CC2=O)c3cccs3)c4cc(ccc4F)Br)C(=O)OC5CCCC5

Supplementary Table S2:
Detailed table of XP Docking using Schrodinger's Glide module

ZINC_ID	xLogP	ApolarDesolvation	PolarDesolvation	H Donors	H Acceptors	Charge	MolecularWeight	RotatableBonds	SMILES
ZINC08439849	6.25	-0.28	-9.92	0	4	0	530.459	5	CC1=C([C@H]([C@H]2C(=N1)C[C@@H](CC2=O)c3cccs3)c4cc(ccc4F)Br)C(=O)OC5CCCC5
ZINC08439848	6.25	-0.33	-7.39	0	4	0	530.459	5	CC1=NC2=C([C@H](C1C(=O)OC3CCCC3)c4cc(ccc4F)Br)C(=O)C[C@H](C2)c5cccs5
ZINC08439848	6.43	-0.26	-8.14	0	4	0	530.459	5	C=C1C([C@@H](C2C(=N1)C[C@@H](CC2=O)c3cccs3)c4cc(ccc4F)Br)C(=O)OC5CCCC5
ZINC08439848	6.43	0.89	-7.98	0	4	0	530.459	5	C=C1C([C@@H]([C@H]2C(=N1)C[C@@H](CC2=O)c3cccs3)c4cc(ccc4F)Br)C(=O)OC5CCCC5
ZINC08439848	6.25	-0.21	-9.97	0	4	0	530.459	5	CC1=C([C@@H]([C@@H]2C(=N1)C[C@@H](CC2=O)c3cccs3)c4cc(ccc4F)Br)C(=O)OC5CCCC5
ZINC08439847	6.71	1.9	-6.37	0	4	0	501.57	6	CC1=NC2=C([C@@H](C1C(=O)OC3CCCC3)c4ccc(cc4)C(F)(F)F)C(=O)C[C@@H](C2)c5cccs5
ZINC08439847	6.89	2.13	-7.3	0	4	0	501.57	6	C=C1C([C@H](C2C(=N1)C[C@H](CC2=O)c3cccs3)c4ccc(cc4)C(F)(F)F)C(=O)OC5CCCC5
ZINC08439847	6.89	3.35	-13.78	0	4	0	501.57	6	C=C1C([C@H]([C@@H]2C(=N1)C[C@H](CC2=O)c3cccs3)c4ccc(cc4)C(F)(F)F)C(=O)OC5CCCC5
ZINC08439847	6.71	2.02	-8.96	0	4	0	501.57	6	CC1=C([C@H]([C@H]2C(=N1)C[C@H](CC2=O)c3cccs3)c4ccc(cc4)C(F)(F)F)C(=O)OC5CCCC5
ZINC08439846	6.71	1.9	-6.51	0	4	0	501.57	6	CC1=NC2=C([C@H](C1C(=O)OC3CCCC3)c4ccc(cc4)C(F)(F)F)C(=O)C[C@@H](C2)c5cccs5
ZINC08439846	6.89	2.05	-7.48	0	4	0	501.57	6	C=C1C([C@@H](C2C(=N1)C[C@H](CC2=O)c3cccs3)c4ccc(cc4)C(F)(F)F)C(=O)OC5CCCC5
ZINC08439846	6.89	3.12	-6.82	0	4	0	501.57	6	C=C1C([C@@H]([C@H]2C(=N1)C[C@H](CC2=O)c3cccs3)c4ccc(cc4)C(F)(F)F)C(=O)OC5CCCC5
ZINC08439846	6.71	1.95	-9.08	0	4	0	501.57	6	CC1=C([C@@H]([C@@H]2C(=N1)C[C@H](CC2=O)c3cccs3)c4ccc(cc4)C(F)(F)F)C(=O)OC5CCCC5
ZINC08439845	6.71	1.9	-6.5	0	4	0	501.57	6	CC1=NC2=C([C@@H](C1C(=O)OC3CCCC3)c4ccc(cc4)C(F)(F)F)C(=O)C[C@H](C2)c5cccs5
ZINC08439845	6.89	2.04	-7.45	0	4	0	501.57	6	C=C1C([C@H](C2C(=N1)C[C@@H](CC2=O)c3cccs3)c4ccc(cc4)C(F)(F)F)C(=O)OC5CCCC5
ZINC08439845	6.89	3.29	-8.5	0	4	0	501.57	6	C=C1C([C@H]([C@@H]2C(=N1)C[C@@H](CC2=O)c3cccs3)c4ccc(cc4)C(F)(F)F)C(=O)OC5CCCC5
ZINC08439845	6.71	1.92	-9.4	0	4	0	501.57	6	CC1=C([C@H]([C@@H]2C(=N1)C[C@@H](CC2=O)c3cccs3)c4ccc(cc4)C(F)(F)F)C(=O)OC5CCCC5
ZINC08439844	6.71	2.02	-9.1	0	4	0	501.57	6	CC1=NC2=C([C@H](C1C(=O)OC3CCCC3)c4ccc(cc4)C(F)(F)F)C(=O)C[C@H](C2)c5cccs5
ZINC08439844	6.89	3.27	-9.71	0	4	0	501.57	6	C=C1C([C@@H]([C@H]2C(=N1)C[C@@H](CC2=O)c3cccs3)c4ccc(cc4)C(F)(F)F)C(=O)OC5CCCC5
ZINC08439844	6.71	1.8	-10.06	0	4	0	501.57	6	CC1=C([C@@H]([C@H]2C(=N1)C[C@@H](CC2=O)c3cccs3)c4ccc(cc4)C(F)(F)F)C(=O)OC5CCCC5
ZINC08439843	5.97	-0.28	-7.36	0	4	0	488.074	8	CCSCCOC(=O)C1[C@@H](C2=C(C[C@@H](CC2=O)c3cccs3)N=C1C)c4cccc(c4)Cl
ZINC08439843	5.97	-0.45	-9.95	0	4	0	488.074	8	CCSCCOC(=O)C1=C(N=C2C[C@@H](CC(=O)C2[C@H]1c3cccc(c3)Cl)c4cccs4)C
ZINC08439843	6.16	-1.38	-8.42	0	4	0	488.074	8	CCSCCOC(=O)C1[C@@H](C2C(=NC1=C)C[C@@H](CC2=O)c3cccs3)c4cccc(c4)Cl
ZINC08439842	5.97	-0.44	-7.67	0	4	0	488.074	8	CCSCCOC(=O)C1[C@H](C2=C(C[C@@H](CC2=O)c3cccs3)N=C1C)c4cccc(c4)Cl
ZINC08439842	5.97	-0.51	-9.79	0	4	0	488.074	8	CCSCCOC(=O)C1=C(N=C2C[C@@H](CC(=O)C2[C@@H]1c3cccc(c3)Cl)c4cccs4)C
ZINC08439842	6.16	-1.38	-9.09	0	4	0	488.074	8	CCSCCOC(=O)C1[C@H](C2C(=NC1=C)C[C@@H](CC2=O)c3cccs3)c4cccc(c4)Cl
ZINC08439841	5.97	-0.45	-7.53	0	4	0	488.074	8	CCSCCOC(=O)C1[C@@H](C2=C(C[C@H](CC2=O)c3cccs3)N=C1C)c4cccc(c4)Cl
ZINC08439841	5.97	-0.55	-10.38	0	4	0	488.074	8	CCSCCOC(=O)C1=C(N=C2C[C@H](CC(=O)C2[C@H]1c3cccc(c3)Cl)c4cccs4)C
ZINC08439841	6.16	-1.63	-8.19	0	4	0	488.074	8	CCSCCOC(=O)C1[C@@H](C2C(=NC1=C)C[C@H](CC2=O)c3cccs3)c4cccc(c4)Cl
ZINC08439840	5.97	-0.27	-7.3	0	4	0	488.074	8	CCSCCOC(=O)C1[C@H](C2=C(C[C@H](CC2=O)c3cccs3)N=C1C)c4cccc(c4)Cl
ZINC08439840	5.97	-0.63	-10.24	0	4	0	488.074	8	CCSCCOC(=O)C1=C(N=C2C[C@H](CC(=O)C2[C@@H]1c3cccc(c3)Cl)c4cccs4)C
ZINC08439840	6.16	-1.55	-8.48	0	4	0	488.074	8	CCSCCOC(=O)C1[C@H](C2C(=NC1=C)C[C@H](CC2=O)c3cccs3)c4cccc(c4)Cl
ZINC08439839	3.57	0.49	-18.29	0	8	0	513.612	10	CC1=NC2=C([C@@H](C1C(=O)OCCOC)c3ccc(c(c3OC)OC)OC)C(=O)C[C@@H](C2)c4cccs4
ZINC08439839	3.75	0.68	-16.34	0	8	0	513.612	10	COCCOC(=O)C1[C@H](C2C(=NC1=C)C[C@H](CC2=O)c3cccs3)c4ccc(c(c4OC)OC)OC
ZINC08439839	3.75	1.76	-21.54	0	8	0	513.612	10	COCCOC(=O)C1[C@H]([C@@H]2C(=NC1=C)C[C@H](CC2=O)c3cccs3)c4ccc(c(c4OC)OC)OC
ZINC08439839	3.57	0.41	-17.68	0	8	0	513.612	10	CC1=C([C@H]([C@H]2C(=N1)C[C@H](CC2=O)c3cccs3)c4ccc(c(c4OC)OC)OC)C(=O)OCCOC
ZINC08439838	3.57	0.57	-10.91	0	8	0	513.612	10	CC1=NC2=C([C@H](C1C(=O)OCCOC)c3ccc(c(c3OC)OC)OC)C(=O)C[C@@H](C2)c4cccs4
ZINC08439838	3.75	0.71	-12.23	0	8	0	513.612	10	COCCOC(=O)C1[C@@H](C2C(=NC1=C)C[C@H](CC2=O)c3cccs3)c4ccc(c(c4OC)OC)OC
ZINC08439838	3.75	1.84	-13.98	0	8	0	513.612	10	COCCOC(=O)C1[C@@H]([C@H]2C(=NC1=C)C[C@H](CC2=O)c3cccs3)c4ccc(c(c4OC)OC)OC
ZINC08439838	3.57	0.62	-15.27	0	8	0	513.612	10	CC1=C([C@@H]([C@H]2C(=N1)C[C@H](CC2=O)c3cccs3)c4ccc(c(c4OC)OC)OC)C(=O)OCCOC
ZINC08439837	3.57	0.5	-18.34	0	8	0	513.612	10	CC1=NC2=C([C@@H](C1C(=O)OCCOC)c3ccc(c(c3OC)OC)OC)C(=O)C[C@H](C2)c4cccs4
ZINC08439837	3.75	0.66	-16.41	0	8	0	513.612	10	COCCOC(=O)C1[C@H](C2C(=NC1=C)C[C@@H](CC2=O)c3cccs3)c4ccc(c(c4OC)OC)OC
ZINC08439837	3.75	1.8	-22.33	0	8	0	513.612	10	COCCOC(=O)C1[C@H]([C@@H]2C(=NC1=C)C[C@@H](CC2=O)c3cccs3)c4ccc(c(c4OC)OC)OC
ZINC08439837	3.57	0.41	-17.86	0	8	0	513.612	10	CC1=C([C@H]([C@H]2C(=N1)C[C@@H](CC2=O)c3cccs3)c4ccc(c(c4OC)OC)OC)C(=O)OCCOC
ZINC08439836	3.57	0.88	-11.44	0	8	0	513.612	10	CC1=NC2=C([C@H](C1C(=O)OCCOC)c3ccc(c(c3OC)OC)OC)C(=O)C[C@H](C2)c4cccs4
ZINC08439836	3.75	0.89	-11.44	0	8	0	513.612	10	COCCOC(=O)C1[C@@H](C2C(=NC1=C)C[C@@H](CC2=O)c3cccs3)c4ccc(c(c4OC)OC)OC
ZINC08439836	3.75	1.9	-12.25	0	8	0	513.612	10	COCCOC(=O)C1[C@@H]([C@H]2C(=NC1=C)C[C@@H](CC2=O)c3cccs3)c4ccc(c(c4OC)OC)OC
ZINC08439836	3.57	0.91	-13.07	0	8	0	513.612	10	CC1=C([C@@H]([C@H]2C(=N1)C[C@@H](CC2=O)c3cccs3)c4ccc(c(c4OC)OC)OC)C(=O)OCCOC
ZINC08439835	5.38	-0.6	-12.48	0	5	0	506.451	8	CCOCCOC(=O)C1[C@@H](C2=C(C[C@@H](CC2=O)c3cccs3)N=C1C)c4cccc(c4Cl)Cl
ZINC08439835	5.38	-0.78	-14.15	0	5	0	506.451	8	CCOCCOC(=O)C1=C(N=C2C[C@@H](CC(=O)C2[C@H]1c3cccc(c3Cl)Cl)c4cccs4)C
ZINC08439835	5.56	-1.65	-13.07	0	5	0	506.451	8	CCOCCOC(=O)C1[C@@H](C2C(=NC1=C)C[C@@H](CC2=O)c3cccs3)c4cccc(c4Cl)Cl
ZINC08439834	5.38	-0.71	-8.94	0	5	0	506.451	8	CCOCCOC(=O)C1[C@H](C2=C(C[C@@H](CC2=O)c3cccs3)N=C1C)c4cccc(c4Cl)Cl
ZINC08439834	5.38	-0.79	-11.3	0	5	0	506.451	8	CCOCCOC(=O)C1=C(N=C2C[C@@H](CC(=O)C2[C@@H]1c3cccc(c3Cl)Cl)c4cccs4)C
ZINC08439834	5.56	-1.65	-9.89	0	5	0	506.451	8	CCOCCOC(=O)C1[C@H](C2C(=NC1=C)C[C@@H](CC2=O)c3cccs3)c4cccc(c4Cl)Cl
ZINC08439833	5.38	-0.53	-8.91	0	5	0	506.451	8	CCOCCOC(=O)C1[C@@H](C2=C(C[C@H](CC2=O)c3cccs3)N=C1C)c4cccc(c4Cl)Cl
ZINC08439833	5.38	-0.79	-11.2	0	5	0	506.451	8	CCOCCOC(=O)C1=C(N=C2C[C@H](CC(=O)C2[C@H]1c3cccc(c3Cl)Cl)c4cccs4)C
ZINC08439833	5.56	-1.92	-9.17	0	5	0	506.451	8	CCOCCOC(=O)C1[C@@H](C2C(=NC1=C)C[C@H](CC2=O)c3cccs3)c4cccc(c4Cl)Cl
ZINC08439832	5.55	-3.22	-36.22	2	10	0	635.142	11	Cc1ccc(cc1)S(=O)(=O)N(CC(=O)N/N=C(\C)/c2cccc(c2)NC(=O)c3cccc(c3)Cl)c4ccc(c(c4)OC)OC
ZINC08439831	5.38	-0.52	-9.74	0	5	0	506.451	8	CCOCCOC(=O)C1[C@H](C2=C(C[C@H](CC2=O)c3cccs3)N=C1C)c4cccc(c4Cl)Cl
ZINC08439831	5.38	-0.71	-11.9	0	5	0	506.451	8	CCOCCOC(=O)C1=C(N=C2C[C@H](CC(=O)C2[C@@H]1c3cccc(c3Cl)Cl)c4cccs4)C
ZINC08439831	5.56	-1.62	-10.27	0	5	0	506.451	8	CCOCCOC(=O)C1[C@H](C2C(=NC1=C)C[C@H](CC2=O)c3cccs3)c4cccc(c4Cl)Cl

Supplementary Table S2:
Detailed table of XP Docking using Schrodinger's Glide module

ZINC_ID	xLogP	ApolarDesolvation	PolarDesolvation	H Donors	H Acceptors	Charge	MolecularWeight	RotatableBonds	SMILES
ZINC08439830	4.95	0.65	-11.26	0	7	0	543.707	11	CCSCCOC(=O)C1[C@@H](C2=C(C[C@@H](CC2=O)c3cccs3)N=C1C)c4cc(c(c(c4)OC)OC)OC
ZINC08439830	4.95	0.3	-14.93	0	7	0	543.707	11	CCSCCOC(=O)C1=C(N=C2C[C@@H](CC(=O)C2[C@H]1c3cc(c(c(c3)OC)OC)OC)c4cccs4)C
ZINC08439830	5.13	-0.63	-12.73	0	7	0	543.707	11	CCSCCOC(=O)C1[C@@H](C2C(=NC1=C)C[C@@H](CC2=O)c3cccs3)c4cc(c(c(c4)OC)OC)OC
ZINC08439829	4.52	-2.64	-28.09	1	10	0	555.653	13	CCOc1ccc(cc1OCC)/C=N/NC(=O)CN(c2ccc(c(c2)OC)OC)S(=O)(=O)c3ccc(cc3)C
ZINC08439828	4.95	0.67	-11.54	0	7	0	543.707	11	CCSCCOC(=O)C1[C@H](C2=C(C[C@@H](CC2=O)c3cccs3)N=C1C)c4cc(c(c(c4)OC)OC)OC
ZINC08439828	4.95	0.41	-13.93	0	7	0	543.707	11	CCSCCOC(=O)C1=C(N=C2C[C@@H](CC(=O)C2[C@@H]1c3cc(c(c(c3)OC)OC)OC)c4cccs4)C
ZINC08439828	5.13	-0.47	-12.2	0	7	0	543.707	11	CCSCCOC(=O)C1[C@H](C2C(=NC1=C)C[C@@H](CC2=O)c3cccs3)c4cc(c(c(c4)OC)OC)OC
ZINC08439827	4.79	-2.53	-25.45	1	9	0	523.611	12	Cc1ccc(cc1)S(=O)(=O)N(CC(=O)N/N=C/c2cccc(c2)OCC=C)c3ccc(c(c3)OC)OC
ZINC08439826	4.95	0.65	-11.43	0	7	0	543.707	11	CCSCCOC(=O)C1[C@@H](C2=C(C[C@H](CC2=O)c3cccs3)N=C1C)c4cc(c(c(c4)OC)OC)OC
ZINC08439826	4.95	0.4	-14.46	0	7	0	543.707	11	CCSCCOC(=O)C1=C(N=C2C[C@H](CC(=O)C2[C@H]1c3cc(c(c(c3)OC)OC)OC)c4cccs4)C
ZINC08439826	5.13	-0.53	-12.5	0	7	0	543.707	11	CCSCCOC(=O)C1[C@@H](C2C(=NC1=C)C[C@H](CC2=O)c3cccs3)c4cc(c(c(c4)OC)OC)OC
ZINC08439825	3.54	-3.29	-30.92	1	10	0	539.61	9	Cc1ccc(cc1)S(=O)(=O)N(CC(=O)N/N=C(\C)/c2ccc3c(c2)OCCO3)c4ccc(c(c4)OC)OC
ZINC08439824	5.37	-2.76	-35.86	2	8	0	558.635	9	Cc1ccc(cc1)S(=O)(=O)N(CC(=O)N/N=C(\C)/c2cccc(c2)NC(=O)c3ccccc3)c4ccccc4F
ZINC08439823	4.95	0.67	-11.36	0	7	0	543.707	11	CCSCCOC(=O)C1[C@H](C2=C(C[C@H](CC2=O)c3cccs3)N=C1C)c4cc(c(c(c4)OC)OC)OC
ZINC08439823	4.95	0.48	-14.06	0	7	0	543.707	11	CCSCCOC(=O)C1=C(N=C2C[C@H](CC(=O)C2[C@@H]1c3cc(c(c(c3)OC)OC)OC)c4cccs4)C
ZINC08439823	5.13	-0.43	-12.22	0	7	0	543.707	11	CCSCCOC(=O)C1[C@H](C2C(=NC1=C)C[C@H](CC2=O)c3cccs3)c4cc(c(c(c4)OC)OC)OC
ZINC08439822	4.43	-1.5	-32.8	1	8	0	480.521	9	Cc1ccc(cc1)S(=O)(=O)N(CC(=O)N/N=C/c2ccc(cc2)OCC#N)c3ccccc3F
ZINC08439821	4.03	-4.48	-36.17	2	8	0	543.443	8	C/C(=N\NC(=O)CN(c1ccccc1Br)S(=O)(=O)C)/c2cccc(c2)NC(=O)c3ccccc3
ZINC08439820	5.18	1.1	-8.96	0	4	0	439.552	6	CC1=NC2=C([C@@H](C1C(=O)OCC(C)C)c3ccccc3F)C(=O)C[C@@H](C2)c4cccs4
ZINC08439820	5.36	1.33	-9.62	0	4	0	439.552	6	CC(C)COC(=O)C1[C@H](C2C(=NC1=C)C[C@H](CC2=O)c3cccs3)c4ccccc4F
ZINC08439820	5.36	2.32	-9.62	0	4	0	439.552	6	CC(C)COC(=O)C1[C@H]([C@@H]2C(=NC1=C)C[C@H](CC2=O)c3cccs3)c4ccccc4F
ZINC08439820	5.18	1.22	-11.53	0	4	0	439.552	6	CC1=C([C@H]([C@H]2C(=N1)C[C@H](CC2=O)c3cccs3)c4ccccc4F)C(=O)OCC(C)C
ZINC08439819	2.38	5.49	-33.07	2	8	0	481.372	7	C/C(=N\NC(=O)CN(c1ccccc1Br)S(=O)(=O)C)/c2ccc(cc2)NC(=O)C
ZINC08439818	3.06	5.95	-14.07	1	6	0	430.349	6	C/C(=N\NC(=O)CN(c1ccccc1Br)S(=O)(=O)C)/c2cccs2
ZINC08439817	2.67	5.99	-18.47	1	8	0	482.356	6	C/C(=N\NC(=O)CN(c1ccccc1Br)S(=O)(=O)C)/c2ccc3c(c2)OCCO3
ZINC08439816	5.18	0.96	-8.72	0	4	0	439.552	6	CC1=NC2=C([C@H](C1C(=O)OCC(C)C)c3ccccc3F)C(=O)C[C@@H](C2)c4cccs4
ZINC08439816	5.36	1.05	-9.17	0	4	0	439.552	6	CC(C)COC(=O)C1[C@@H](C2C(=NC1=C)C[C@H](CC2=O)c3cccs3)c4ccccc4F
ZINC08439816	5.36	2.12	-9.08	0	4	0	439.552	6	CC(C)COC(=O)C1[C@@H]([C@H]2C(=NC1=C)C[C@H](CC2=O)c3cccs3)c4ccccc4F
ZINC08439816	5.18	1.1	-11.2	0	4	0	439.552	6	CC1=C([C@@H]([C@@H]2C(=N1)C[C@H](CC2=O)c3cccs3)c4ccccc4F)C(=O)OCC(C)C
ZINC08439815	2.37	6.33	-36.18	1	10	0	508.984	9	CS(=O)(=O)N(CC(=O)N/N=C/c1ccc(cc1)OCC(=O)N2CCOCC2)c3ccccc3Cl
ZINC08439813	5.11	-2.9	-24.67	1	8	0	493.585	12	CCOc1ccccc1N(CC(=O)N/N=C/c2ccc(cc2)OCC=C)S(=O)(=O)c3ccccc3
ZINC08439812	5.18	1.03	-8.66	0	4	0	439.552	6	CC1=NC2=C([C@@H](C1C(=O)OCC(C)C)c3ccccc3F)C(=O)C[C@H](C2)c4cccs4
ZINC08439812	5.36	1.16	-8.91	0	4	0	439.552	6	CC(C)COC(=O)C1[C@H](C2C(=NC1=C)C[C@@H](CC2=O)c3cccs3)c4ccccc4F
ZINC08439812	5.36	2.44	-10.35	0	4	0	439.552	6	CC(C)COC(=O)C1[C@H]([C@@H]2C(=NC1=C)C[C@@H](CC2=O)c3cccs3)c4ccccc4F
ZINC08439812	5.18	1.22	-10.89	0	4	0	439.552	6	CC1=C([C@H]([C@H]2C(=N1)C[C@@H](CC2=O)c3cccs3)c4ccccc4F)C(=O)OCC(C)C
ZINC08439811	4.72	-3.96	-30.67	1	10	0	578.691	12	CCOc1ccccc1N(CC(=O)N/N=C/c2ccc(cc2)OCC(=O)N3CCCCC3)S(=O)(=O)c4ccccc4
ZINC08439810	5.69	-3.42	-27.4	1	7	0	513.619	9	CCOc1ccccc1N(CC(=O)N/N=C/c2ccc3c4c2cccc4CC3)S(=O)(=O)c5ccccc5
ZINC08439809	5.18	1.24	-13.78	0	4	0	439.552	6	CC1=NC2=C([C@H](C1C(=O)OCC(C)C)c3ccccc3F)C(=O)C[C@H](C2)c4cccs4
ZINC08439809	5.36	1.45	-13.28	0	4	0	439.552	6	CC(C)COC(=O)C1[C@@H](C2C(=NC1=C)C[C@@H](CC2=O)c3cccs3)c4ccccc4F
ZINC08439809	5.36	2.75	-28.13	0	4	0	439.552	6	CC(C)COC(=O)C1[C@@H]([C@H]2C(=NC1=C)C[C@@H](CC2=O)c3cccs3)c4ccccc4F
ZINC08439809	5.18	1.2	-14.58	0	4	0	439.552	6	CC1=C([C@@H]([C@@H]2C(=N1)C[C@@H](CC2=O)c3cccs3)c4ccccc4F)C(=O)OCC(C)C
ZINC08439808	7.25	-0.04	-34.5	1	9	0	634.032	12	CCOC(=O)c1ccc(cc1)c2ccc(o2)/C=N/NC(=O)CN(c3ccc(c(c3)C(F)(F)F)Cl)S(=O)(=O)c4ccccc4
ZINC08439807	4.63	-2.06	-35.09	2	8	0	566.989	9	C/C(=N\NC(=O)CN(c1ccc(c(c1)C(F)(F)F)Cl)S(=O)(=O)c2ccccc2)/c3cccc(c3)NC(=O)C
ZINC08439806	4.66	-2.15	-27.51	2	8	0	566.989	9	C/C(=N\NC(=O)CN(c1ccc(c(c1)C(F)(F)F)Cl)S(=O)(=O)c2ccccc2)/c3ccc(cc3)NC(=O)C
ZINC08439805	5.85	-0.23	-8.74	0	5	0	463.599	6	CC1=NC2=C([C@@H](C1C(=O)OC3CCCC3)c4cccc(c4)OC)C(=O)C[C@@H](C2)c5cccs5
ZINC08439805	6.03	-0.17	-9.41	0	5	0	463.599	6	COc1cccc(c1)[C@H]2C3C(=NC(=C)C2C(=O)OC4CCCC4)C[C@H](CC3=O)c5cccs5
ZINC08439805	6.03	1.23	-15.32	0	5	0	463.599	6	COc1cccc(c1)[C@H]2[C@@H]3C(=NC(=C)C2C(=O)OC4CCCC4)C[C@H](CC3=O)c5cccs5
ZINC08439805	5.85	-0.29	-11.51	0	5	0	463.599	6	CC1=C([C@H]([C@@H]2C(=N1)C[C@H](CC2=O)c3cccs3)c4cccc(c4)OC)C(=O)OC5CCCC5
ZINC08439804	5.82	-0.83	-19.22	1	8	0	582	12	COc1cc(ccc1OCC=C)/C=N/NC(=O)CN(c2ccc(c(c2)C(F)(F)F)Cl)S(=O)(=O)c3ccccc3
ZINC08439803	5.95	-2.84	-24.56	1	7	0	584.041	10	c1ccc(cc1)S(=O)(=O)N(CC(=O)N/N=C/c2ccc(cc2)OC3CSC3)c4ccc(c(c4)C(F)(F)F)Cl
ZINC08439802	5.85	-0.34	-9.19	0	5	0	463.599	6	CC1=NC2=C([C@H](C1C(=O)OC3CCCC3)c4cccc(c4)OC)C(=O)C[C@@H](C2)c5cccs5
ZINC08439802	6.03	-0.27	-9.74	0	5	0	463.599	6	COc1cccc(c1)[C@@H]2C3C(=NC(=C)C2C(=O)OC4CCCC4)C[C@H](CC3=O)c5cccs5
ZINC08439802	6.03	0.97	-11.21	0	5	0	463.599	6	COc1cccc(c1)[C@@H]2[C@H]3C(=NC(=C)C2C(=O)OC4CCCC4)C[C@H](CC3=O)c5cccs5
ZINC08439802	5.85	-0.23	-11.83	0	5	0	463.599	6	CC1=C([C@@H]([C@H]2C(=N1)C[C@H](CC2=O)c3cccs3)c4cccc(c4)OC)C(=O)OC5CCCC5
ZINC08439801	4.78	-3.4	-32.23	1	10	0	639.052	11	c1ccc(cc1)S(=O)(=O)N(CC(=O)N/N=C/c2ccc(cc2)OCC(=O)N3CCOCC3)c4ccc(c(c4)C(F)(F)F)Cl
ZINC08439800	5.85	-0.24	-8.73	0	5	0	463.599	6	CC1=NC2=C([C@@H](C1C(=O)OC3CCCC3)c4cccc(c4)OC)C(=O)C[C@H](C2)c5cccs5
ZINC08439800	6.03	-0.11	-9.4	0	5	0	463.599	6	COc1cccc(c1)[C@H]2C3C(=NC(=C)C2C(=O)OC4CCCC4)C[C@@H](CC3=O)c5cccs5
ZINC08439800	6.03	1.15	-11.04	0	5	0	463.599	6	COc1cccc(c1)[C@H]2[C@@H]3C(=NC(=C)C2C(=O)OC4CCCC4)C[C@@H](CC3=O)c5cccs5
ZINC08439800	5.85	-0.23	-11.44	0	5	0	463.599	6	CC1=C([C@H]([C@@H]2C(=N1)C[C@@H](CC2=O)c3cccs3)c4cccc(c4)OC)C(=O)OC5CCCC5
ZINC08439799	5.84	-2.3	-31.58	1	9	0	637.08	11	c1ccc(cc1)S(=O)(=O)N(CC(=O)N/N=C/c2ccc(cc2)OCC(=O)N3CCCCC3)c4ccc(c(c4)C(F)(F)F)Cl
ZINC08439798	6.63	-0.14	-28.61	1	9	0	626.053	13	CC(C)(C)OC(=O)COc1ccc(cc1)/C=N/NC(=O)CN(c2ccc(c(c2)C(F)(F)F)Cl)S(=O)(=O)c3ccccc3

Supplementary Table S2:
Detailed table of XP Docking using Schrodinger's Glide module

ZINC_ID	xLogP	ApolarDesolvation	PolarDesolvation	H Donors	H Acceptors	Charge	MolecularWeight	RotatableBonds	SMILES
ZINC08439797	5.85	-0.24	-8.91	0	5	0	463.599	6	CC1=NC2=C([C@H](C1C(=O)OC3CCCC3)c4cccc(c4)OC)C(=O)C[C@H](C2)c5cccs5
ZINC08439797	6.03	-0.18	-9.5	0	5	0	463.599	6	COc1cccc(c1)[C@@H]2C3C(=NC(=C)C2C(=O)OC4CCCC4)C[C@@H](CC3=O)c5cccs5
ZINC08439797	6.03	1.13	-10.79	0	5	0	463.599	6	COc1cccc(c1)[C@@H]2[C@H]3C(=NC(=C)C2C(=O)OC4CCCC4)C[C@@H](CC3=O)c5cccs5
ZINC08439797	5.85	-0.3	-11.85	0	5	0	463.599	6	CC1=C([C@@H]([C@H]2C(=N1)C[C@@H](CC2=O)c3cccs3)c4cccc(c4)OC)C(=O)OC5CCCC5
ZINC08439796	5.44	-0.53	-29.48	1	9	0	583.972	12	COC(=O)COc1ccc(cc1)/C=N/NC(=O)CN(c2ccc(c(c2)C(F)(F)F)Cl)S(=O)(=O)c3ccccc3
ZINC08439795	4.95	-2.01	-30.37	1	8	0	567.973	8	C/C(=N\NC(=O)CN(c1ccc(c(c1)C(F)(F)F)Cl)S(=O)(=O)c2ccccc2)/c3ccc4c(c3)OCCO4
ZINC08439794	4.13	0.91	-15.37	0	6	0	461.539	5	CCOC(=O)C1[C@@H](C2=C(C[C@@H](CC2=O)c3cccs3)N=C1C)c4coc5ccccc5c4=O
ZINC08439794	4.13	0.57	-17.32	0	6	0	461.539	5	CCOC(=O)C1=C(N=C2C[C@@H](CC(=O)C2[C@H]1c3coc4ccccc4c3=O)c5cccs5)C
ZINC08439794	4.31	-0.36	-14.76	0	6	0	461.539	5	CCOC(=O)C1[C@@H](C2C(=NC1=C)C[C@@H](CC2=O)c3cccs3)c4coc5ccccc5c4=O
ZINC08439793	6.36	-3.39	-23.89	1	7	0	617.875	9	CN(C)c1ccc(cc1Br)/C=N/NC(=O)CN(c2ccc(c(c2)C(F)(F)F)Cl)S(=O)(=O)c3ccccc3
ZINC08439792	4.13	0.75	-14.66	0	6	0	461.539	5	CCOC(=O)C1[C@H](C2=C(C[C@@H](CC2=O)c3cccs3)N=C1C)c4coc5ccccc5c4=O
ZINC08439792	4.13	0.65	-17.28	0	6	0	461.539	5	CCOC(=O)C1=C(N=C2C[C@@H](CC(=O)C2[C@@H]1c3coc4ccccc4c3=O)c5cccs5)C
ZINC08439792	4.31	-0.44	-14.26	0	6	0	461.539	5	CCOC(=O)C1[C@H](C2C(=NC1=C)C[C@@H](CC2=O)c3cccs3)c4coc5ccccc5c4=O
ZINC08439791	6.15	-3.09	-25.94	1	8	0	618.815	8	c1ccc(cc1)S(=O)(=O)N(CC(=O)N/N=C/c2cc3c(cc2Br)OCO3)c4ccc(c(c4)C(F)(F)F)Cl
ZINC08439790	4.13	0.89	-21.67	0	6	0	461.539	5	CCOC(=O)C1[C@@H](C2=C(C[C@H](CC2=O)c3cccs3)N=C1C)c4coc5ccccc5c4=O
ZINC08439790	4.13	0.63	-21.61	0	6	0	461.539	5	CCOC(=O)C1=C(N=C2C[C@H](CC(=O)C2[C@H]1c3coc4ccccc4c3=O)c5cccs5)C
ZINC08439790	4.31	-0.37	-18.32	0	6	0	461.539	5	CCOC(=O)C1[C@@H](C2C(=NC1=C)C[C@H](CC2=O)c3cccs3)c4coc5ccccc5c4=O
ZINC08439789	4.78	-7	-26.26	1	9	0	618.055	9	c1ccc(cc1)S(=O)(=O)N(CC(=O)Nc2ccc(cc2)S(=O)(=O)N3CCOCC3)c4ccc(c(c4)C(F)(F)F)Cl
ZINC00189848	2.8	5.34	-11.22	0	5	0	338.454	4	COc1ccccc1N2CCN(CC2)S(=O)(=O)c3cccs3
ZINC08439788	4.13	0.92	-15.38	0	6	0	461.539	5	CCOC(=O)C1[C@H](C2=C(C[C@H](CC2=O)c3cccs3)N=C1C)c4coc5ccccc5c4=O
ZINC08439788	4.13	0.73	-17.66	0	6	0	461.539	5	CCOC(=O)C1=C(N=C2C[C@H](CC(=O)C2[C@@H]1c3coc4ccccc4c3=O)c5cccs5)C
ZINC08439788	4.31	-0.19	-15.22	0	6	0	461.539	5	CCOC(=O)C1[C@H](C2C(=NC1=C)C[C@H](CC2=O)c3cccs3)c4coc5ccccc5c4=O
ZINC08439787	5.22	0.27	-7.87	0	4	0	490.394	5	CCOC(=O)C1[C@@H](C2=C(C[C@@H](CC2=O)c3cccs3)N=C1C)c4cc(ccc4F)Br
ZINC08439787	5.22	-0.08	-10.33	0	4	0	490.394	5	CCOC(=O)C1=C(N=C2C[C@@H](CC(=O)C2[C@H]1c3cc(ccc3F)Br)c4cccs4)C
ZINC08439787	5.4	-1.14	-7.97	0	4	0	490.394	5	CCOC(=O)C1[C@@H](C2C(=NC1=C)C[C@@H](CC2=O)c3cccs3)c4cc(ccc4F)Br
ZINC00645912	4.36	0.53	-14.54	2	6	0	441.531	6	CCc1ccc(cc1)N2C[C@H](CC2=O)C(=O)Nc3ccccc3C(=O)Nc4ccccc4C
ZINC00645913	4.36	0.57	-14.36	2	6	0	441.531	6	CCc1ccc(cc1)N2C[C@@H](CC2=O)C(=O)Nc3ccccc3C(=O)Nc4ccccc4C
ZINC08439786	5.22	0.28	-7.84	0	4	0	490.394	5	CCOC(=O)C1[C@H](C2=C(C[C@@H](CC2=O)c3cccs3)N=C1C)c4cc(ccc4F)Br
ZINC08439786	5.22	0	-10.12	0	4	0	490.394	5	CCOC(=O)C1=C(N=C2C[C@@H](CC(=O)C2[C@@H]1c3cc(ccc3F)Br)c4cccs4)C
ZINC08439786	5.4	-0.88	-8.54	0	4	0	490.394	5	CCOC(=O)C1[C@H](C2C(=NC1=C)C[C@@H](CC2=O)c3cccs3)c4cc(ccc4F)Br
ZINC00645910	3.83	0.91	-13.92	2	6	0	455.558	6	CCc1ccc(cc1)N2C[C@H](CC2=O)C(=O)Nc3ccccc3C(=O)Nc4c(cccc4C)C
ZINC08439785	5.22	0.04	-10.45	0	4	0	490.394	5	CCOC(=O)C1[C@@H](C2=C(C[C@H](CC2=O)c3cccs3)N=C1C)c4cc(ccc4F)Br
ZINC08439785	5.22	-0.07	-11.83	0	4	0	490.394	5	CCOC(=O)C1=C(N=C2C[C@H](CC(=O)C2[C@H]1c3cc(ccc3F)Br)c4cccs4)C
ZINC08439785	5.4	-1.1	-9.71	0	4	0	490.394	5	CCOC(=O)C1[C@@H](C2C(=NC1=C)C[C@H](CC2=O)c3cccs3)c4cc(ccc4F)Br
ZINC00645911	3.83	1	-16.83	2	6	0	455.558	6	CCc1ccc(cc1)N2C[C@@H](CC2=O)C(=O)Nc3ccccc3C(=O)Nc4c(cccc4C)C
ZINC00645908	4.17	-0.95	-16.74	2	6	0	433.552	6	CCc1ccc(cc1)N2C[C@H](CC2=O)C(=O)Nc3ccccc3C(=O)NC4CCCCC4
ZINC08439784	5.22	0.28	-7.88	0	4	0	490.394	5	CCOC(=O)C1[C@H](C2=C(C[C@H](CC2=O)c3cccs3)N=C1C)c4cc(ccc4F)Br
ZINC08439784	5.22	0.1	-10.12	0	4	0	490.394	5	CCOC(=O)C1=C(N=C2C[C@H](CC(=O)C2[C@@H]1c3cc(ccc3F)Br)c4cccs4)C
ZINC08439784	5.4	-0.77	-8.71	0	4	0	490.394	5	CCOC(=O)C1[C@H](C2C(=NC1=C)C[C@H](CC2=O)c3cccs3)c4cc(ccc4F)Br
ZINC00645909	4.17	-0.91	-16.89	2	6	0	433.552	6	CCc1ccc(cc1)N2C[C@@H](CC2=O)C(=O)Nc3ccccc3C(=O)NC4CCCCC4
ZINC08439782	3.71	-1.05	-10.34	0	5	0	443.59	8	CCOCCOC(=O)C1[C@@H](C2=C(C[C@@H](CC2=O)c3cccs3)N=C1C)c4ccsc4
ZINC08439782	3.71	-1.35	-11.57	0	5	0	443.59	8	CCOCCOC(=O)C1=C(N=C2C[C@@H](CC(=O)C2[C@H]1c3ccsc3)c4cccs4)C
ZINC08439782	3.89	-2.3	-10.56	0	5	0	443.59	8	CCOCCOC(=O)C1[C@@H](C2C(=NC1=C)C[C@@H](CC2=O)c3cccs3)c4ccsc4
ZINC00645898	3.93	10.29	-15.68	2	6	0	439.943	5	c1ccc(c(c1)C(=O)NC2CCCCC2)NC(=O)[C@H]3CC(=O)N(C3)c4ccc(cc4)Cl
ZINC08439781	3.71	-1.11	-8.39	0	5	0	443.59	8	CCOCCOC(=O)C1[C@H](C2=C(C[C@@H](CC2=O)c3cccs3)N=C1C)c4ccsc4
ZINC08439781	3.71	-1.21	-10.62	0	5	0	443.59	8	CCOCCOC(=O)C1=C(N=C2C[C@@H](CC(=O)C2[C@@H]1c3ccsc3)c4cccs4)C
ZINC08439781	3.89	-2.16	-9.44	0	5	0	443.59	8	CCOCCOC(=O)C1[C@H](C2C(=NC1=C)C[C@@H](CC2=O)c3cccs3)c4ccsc4
ZINC00645899	3.93	10.25	-12.62	2	6	0	439.943	5	c1ccc(c(c1)C(=O)NC2CCCCC2)NC(=O)[C@@H]3CC(=O)N(C3)c4ccc(cc4)Cl
ZINC08439780	3.71	-1.02	-8.31	0	5	0	443.59	8	CCOCCOC(=O)C1[C@@H](C2=C(C[C@H](CC2=O)c3cccs3)N=C1C)c4ccsc4
ZINC08439780	3.71	-1.23	-11.21	0	5	0	443.59	8	CCOCCOC(=O)C1=C(N=C2C[C@H](CC(=O)C2[C@H]1c3ccsc3)c4cccs4)C
ZINC08439780	3.89	-2.23	-8.8	0	5	0	443.59	8	CCOCCOC(=O)C1[C@@H](C2C(=NC1=C)C[C@H](CC2=O)c3cccs3)c4ccsc4
ZINC02171978	5.78	1.11	-18.66	2	5	0	457.357	7	CCc1cccc(c1NC(=O)COc2ccccc2C(=O)Nc3cccc(c3Cl)Cl)C
ZINC02171975	5.01	0.05	-24.26	2	7	0	448.519	10	Cc1cccc(c1OCCNC(=O)c2ccccc2OCC(=O)Nc3cccc(c3)OC)C
ZINC08439779	3.71	-1.01	-8.75	0	5	0	443.59	8	CCOCCOC(=O)C1[C@H](C2=C(C[C@H](CC2=O)c3cccs3)N=C1C)c4ccsc4
ZINC08439779	3.71	-1.15	-10.9	0	5	0	443.59	8	CCOCCOC(=O)C1=C(N=C2C[C@H](CC(=O)C2[C@@H]1c3ccsc3)c4cccs4)C
ZINC08439779	3.89	-2.15	-9.52	0	5	0	443.59	8	CCOCCOC(=O)C1[C@H](C2C(=NC1=C)C[C@H](CC2=O)c3cccs3)c4ccsc4
ZINC00645888	4.68	1.59	-13.74	2	7	0	440.883	7	COc1ccc(c(c1OC)OC)C(=O)Nc2ccccc2C(=O)Nc3ccccc3Cl
ZINC00645878	4.78	-3.88	-14.27	1	5	0	463.987	6	c1cc(ccc1c2csc(n2)SCC(=O)Nc3ccc(c(c3)Cl)N4CCOCC4)F
ZINC08439778	4.15	-0.25	-17.67	0	8	0	539.65	9	CC1=NC2=C([C@@H](C1C(=O)OC[C@@H]3CCCO3)c4ccc(c(c4OC)OC)OC)C(=O)C[C@@H](C2)c5cccs5
ZINC08439778	4.33	-0.22	-16.11	0	8	0	539.65	9	COc1ccc(c(c1OC)OC)[C@H]2C3C(=NC(=C)C2C(=O)OC[C@@H]4CCCO4)C[C@H](CC3=O)c5cccs5
ZINC08439778	4.33	1.06	-20.92	0	8	0	539.65	9	COc1ccc(c(c1OC)OC)[C@H]2[C@@H]3C(=NC(=C)C2C(=O)OC[C@@H]4CCCO4)C[C@H](CC3=O)c5cccs5

Supplementary Table S2:
Detailed table of XP Docking using Schrodinger's Glide module

ZINC_ID	xLogP	ApolarDesolvation	PolarDesolvation	H Donors	H Acceptors	Charge	MolecularWeight	RotatableBonds	SMILES
ZINC08439778	4.15	-0.5	-17.88	0	8	0	539.65	9	CC1=C([C@H]([C@@H]2C(=N1)C[C@H](CC2=O)c3cccs3)c4ccc(c(c4OC)OC)OC)C(=O)OC[C@@H]5CCCO5
ZINC08439777	3.45	7.91	-34.77	3	9	0	477.517	11	CC(=O)Nc1ccc(cc1)NC(=O)COc2ccccc2C(=O)NCCOc3ccc(cc3)OC
ZINC08439776	3.27	-5.22	-22.38	2	8	0	493.585	9	c1ccc(cc1)OCCNC(=O)c2ccccc2NC(=O)[C@H]3CCCN3S(=O)(=O)c4ccccc4
ZINC08439775	4.15	0.11	-11.26	0	8	0	539.65	9	CC1=NC2=C([C@H](C1C(=O)OC[C@@H]3CCCO3)c4ccc(c(c4OC)OC)OC)C(=O)C[C@@H](C2)c5cccs5
ZINC08439775	4.33	0.27	-12.37	0	8	0	539.65	9	COc1ccc(c(c1OC)OC)[C@@H]2C3C(=NC(=C)C2C(=O)OC[C@@H]4CCCO4)C[C@H](CC3=O)c5cccs5
ZINC08439775	4.33	1.32	-14.35	0	8	0	539.65	9	COc1ccc(c(c1OC)OC)[C@@H]2[C@H]3C(=NC(=C)C2C(=O)OC[C@@H]4CCCO4)C[C@H](CC3=O)c5cccs5
ZINC08439775	4.15	0.19	-14.78	0	8	0	539.65	9	CC1=C([C@@H]([C@H]2C(=N1)C[C@H](CC2=O)c3cccs3)c4ccc(c(c4OC)OC)OC)C(=O)OC[C@@H]5CCCO5
ZINC08439774	3.27	-5.88	-23.85	2	8	0	493.585	9	c1ccc(cc1)OCCNC(=O)c2ccccc2NC(=O)[C@@H]3CCCN3S(=O)(=O)c4ccccc4
ZINC08439773	4.15	-0.19	-12.08	0	8	0	539.65	9	CC1=NC2=C([C@@H](C1C(=O)OC[C@@H]3CCCO3)c4ccc(c(c4OC)OC)OC)C(=O)C[C@H](C2)c5cccs5
ZINC08439773	4.33	-0.04	-12.66	0	8	0	539.65	9	COc1ccc(c(c1OC)OC)[C@H]2C3C(=NC(=C)C2C(=O)OC[C@@H]4CCCO4)C[C@@H](CC3=O)c5cccs5
ZINC08439773	4.33	1.12	-14.05	0	8	0	539.65	9	COc1ccc(c(c1OC)OC)[C@H]2[C@@H]3C(=NC(=C)C2C(=O)OC[C@@H]4CCCO4)C[C@@H](CC3=O)c5cccs5
ZINC08439773	4.15	-0.15	-14.7	0	8	0	539.65	9	CC1=C([C@H]([C@@H]2C(=N1)C[C@@H](CC2=O)c3cccs3)c4ccc(c(c4OC)OC)OC)C(=O)OC[C@@H]5CCCO5
ZINC08439772	4.34	-1.48	-26.07	2	7	0	448.519	12	COc1ccc(cc1)OCCNC(=O)c2ccccc2OCC(=O)NCCc3ccccc3
ZINC00645872	5.97	1.03	-21.07	2	6	0	452.938	8	Cc1c(cccc1Cl)NC(=O)c2ccccc2OCC(=O)Nc3ccccc3OC(C)C
ZINC08439771	4.15	-0.11	-11.74	0	8	0	539.65	9	CC1=NC2=C([C@H](C1C(=O)OC[C@@H]3CCCO3)c4ccc(c(c4OC)OC)OC)C(=O)C[C@H](C2)c5cccs5
ZINC08439771	4.33	0.06	-11.37	0	8	0	539.65	9	COc1ccc(c(c1OC)OC)[C@@H]2C3C(=NC(=C)C2C(=O)OC[C@@H]4CCCO4)C[C@@H](CC3=O)c5cccs5
ZINC08439771	4.33	1.05	-18.83	0	8	0	539.65	9	COc1ccc(c(c1OC)OC)[C@@H]2[C@H]3C(=NC(=C)C2C(=O)OC[C@@H]4CCCO4)C[C@@H](CC3=O)c5cccs5
ZINC08439771	4.15	0.16	-13.27	0	8	0	539.65	9	CC1=C([C@@H]([C@@H]2C(=N1)C[C@@H](CC2=O)c3cccs3)c4ccc(c(c4OC)OC)OC)C(=O)OC[C@@H]5CCCO5
ZINC00645865	3.88	-1.21	-12	2	6	0	428.945	8	Cc1c(cccc1Cl)NC(=O)CSc2nnc(n2CC=C)[C@H](c3ccccc3)O
ZINC00645860	5.28	1.53	-24.18	1	6	0	443.547	8	CCN(CC)c1ccc(cc1)NC(=O)c2ccccc2OCC(=O)N3CCc4c3cccc4
ZINC00630257	4.34	-0.99	-24.55	1	6	0	475.595	6	CC(=O)c1cccc(c1)NC(=O)CSc2nc3c(c4c(s3)CCC4)c(=O)n2c5ccccc5
ZINC08439770	4.92	-0.39	-23.44	2	7	0	448.519	11	CC(C)Oc1ccccc1NC(=O)c2ccccc2OCC(=O)NCCOc3ccccc3
ZINC08439769	5.96	0.49	-7.56	0	4	0	451.563	5	CC1=NC2=C([C@@H](C1C(=O)OC3CCCC3)c4cccc(c4)F)C(=O)C[C@@H](C2)c5cccs5
ZINC08439769	6.14	0.55	-8.16	0	4	0	451.563	5	C=C1C([C@H](C2C(=N1)C[C@H](CC2=O)c3cccs3)c4cccc(c4)F)C(=O)OC5CCCC5
ZINC08439769	6.14	1.95	-13.87	0	4	0	451.563	5	C=C1C([C@H]([C@@H]2C(=N1)C[C@H](CC2=O)c3cccs3)c4cccc(c4)F)C(=O)OC5CCCC5
ZINC08439769	5.96	0.43	-10.26	0	4	0	451.563	5	CC1=C([C@H]([C@@H]2C(=N1)C[C@H](CC2=O)c3cccs3)c4cccc(c4)F)C(=O)OC5CCCC5
ZINC02171959	5.21	0.87	-23.16	1	6	0	444.531	8	Cc1cccc(c1OCCNC(=O)c2ccccc2OCC(=O)N3CCc4c3cccc4)C
ZINC02171957	3.29	-0.15	-14.17	2	8	0	450.491	10	COc1cc(cc(c1OC)OC)C(=O)Nc2ccccc2C(=O)NCCOc3ccccc3
ZINC00630249	5.33	-0.54	-23.11	1	6	0	503.649	6	C[C@H]1CCc2c(sc3c2c(=O)n(c(n3)SCC(=O)Nc4cccc(c4)C(=O)C)c5ccccc5)C1
ZINC08439768	7.22	-1.67	-10.76	2	6	0	460.574	13	CCCc1ccc(cc1)OCCNC(=O)[C@H](Cc2ccccc2)NC(=O)OCc3ccccc3
ZINC00630251	5.33	-0.54	-23.17	1	6	0	503.649	6	C[C@@H]1CCc2c(sc3c2c(=O)n(c(n3)SCC(=O)Nc4cccc(c4)C(=O)C)c5ccccc5)C1
ZINC08439767	5.96	0.48	-7.23	0	4	0	451.563	5	CC1=NC2=C([C@H](C1C(=O)OC3CCCC3)c4cccc(c4)F)C(=O)C[C@@H](C2)c5cccs5
ZINC08439767	6.14	0.61	-8.83	0	4	0	451.563	5	C=C1C([C@@H](C2C(=N1)C[C@H](CC2=O)c3cccs3)c4cccc(c4)F)C(=O)OC5CCCC5
ZINC08439767	6.14	1.95	-13.69	0	4	0	451.563	5	C=C1C([C@@H]([C@H]2C(=N1)C[C@H](CC2=O)c3cccs3)c4cccc(c4)F)C(=O)OC5CCCC5
ZINC08439767	5.96	0.49	-10.24	0	4	0	451.563	5	CC1=C([C@@H]([C@H]2C(=N1)C[C@H](CC2=O)c3cccs3)c4cccc(c4)F)C(=O)OC5CCCC5
ZINC08439766	7.22	-1.67	-11.31	2	6	0	460.574	13	CCCc1ccc(cc1)OCCNC(=O)[C@@H](Cc2ccccc2)NC(=O)OCc3ccccc3
ZINC08439765	4.73	-0.8	-20.88	1	6	0	467.616	7	CC(=O)c1cccc(c1)NC(=O)CSc2nc3c(c4c(s3)CCCCC4)c(=O)n2CC=C
ZINC02171943	4.51	-1.03	-11.47	2	5	0	416.521	9	Cc1cccc(c1OCCNC(=O)[C@@H](Cc2ccccc2)NC(=O)c3ccccc3)C
ZINC02171941	6.16	0.46	-14.15	1	3	0	429.566	7	c1ccc(cc1)CSc2ccccc2C(=O)Nc3ccc(cc3)C(=O)c4cccs4
ZINC08439764	5.96	0.38	-7.62	0	4	0	451.563	5	CC1=NC2=C([C@@H](C1C(=O)OC3CCCC3)c4cccc(c4)F)C(=O)C[C@H](C2)c5cccs5
ZINC08439764	6.14	0.63	-8.24	0	4	0	451.563	5	C=C1C([C@H](C2C(=N1)C[C@@H](CC2=O)c3cccs3)c4cccc(c4)F)C(=O)OC5CCCC5
ZINC08439764	6.14	1.83	-15.8	0	4	0	451.563	5	C=C1C([C@H]([C@@H]2C(=N1)C[C@@H](CC2=O)c3cccs3)c4cccc(c4)F)C(=O)OC5CCCC5
ZINC08439764	5.96	0.52	-10.32	0	4	0	451.563	5	CC1=C([C@H]([C@@H]2C(=N1)C[C@@H](CC2=O)c3cccs3)c4cccc(c4)F)C(=O)OC5CCCC5
ZINC00630239	4.17	-3.67	-14.6	1	6	0	469.632	6	c1ccc(cc1)n2c(=O)c3c4c(sc3nc2SCC(=O)NC[C@H]5CCCO5)CCCCC4
ZINC00645843	3.15	-2.16	-17.03	2	7	0	435.619	9	CC(C)(C)NC(=O)CSc1nc(n(n1)c2ccccc2)SCC(=O)NC(C)(C)C
ZINC00630241	4.17	-3.73	-14.88	1	6	0	469.632	6	c1ccc(cc1)n2c(=O)c3c4c(sc3nc2SCC(=O)NC[C@@H]5CCCO5)CCCCC4
ZINC02171936	5.94	-1.44	-10.07	1	4	0	420.299	2	c1ccc2c(c1)cc(c(n2)Cl)[C@H]3Nc4ccccc4C(=O)N3c5ccc(cc5)Cl
ZINC08439763	5.96	0.47	-7.01	0	4	0	451.563	5	CC1=NC2=C([C@H](C1C(=O)OC3CCCC3)c4cccc(c4)F)C(=O)C[C@H](C2)c5cccs5
ZINC08439763	6.14	0.55	-8.27	0	4	0	451.563	5	C=C1C([C@@H](C2C(=N1)C[C@@H](CC2=O)c3cccs3)c4cccc(c4)F)C(=O)OC5CCCC5
ZINC08439763	6.14	1.7	-7.5	0	4	0	451.563	5	C=C1C([C@@H]([C@H]2C(=N1)C[C@@H](CC2=O)c3cccs3)c4cccc(c4)F)C(=O)OC5CCCC5
ZINC08439763	5.96	0.6	-9.67	0	4	0	451.563	5	CC1=C([C@@H]([C@@H]2C(=N1)C[C@@H](CC2=O)c3cccs3)c4cccc(c4)F)C(=O)OC5CCCC5
ZINC02171937	5.94	-1.44	-10.05	1	4	0	420.299	2	c1ccc2c(c1)cc(c(n2)Cl)[C@@H]3Nc4ccccc4C(=O)N3c5ccc(cc5)Cl
ZINC00630237	5.06	-1.31	-18.76	1	6	0	469.632	9	CCOc1ccc(cc1)NC(=O)CCSc2nc3c(c4c(s3)CCCC4)c(=O)n2CC=C
ZINC00645842	4.57	0.76	-15.1	1	6	0	424.884	5	COc1cc(cc(c1OC)OC)[C@@H]2Nc3ccccc3C(=O)N2c4ccccc4Cl
ZINC00630228	4.7	-0.47	-20.49	1	6	0	467.616	7	C[C@H]1CCc2c(sc3c2c(=O)n(c(n3)SCC(=O)Nc4cccc(c4)C(=O)C)CC=C)C1
ZINC08439762	4.77	-0.5	-51.05	0	9	-1	543.643	10	CCSCCOC(=O)C1[C@@H](C2=C(C[C@@H](CC2=O)c3cccs3)N=C1C)c4cc(c(c(c4)OC)[O-])[N+](=O)[O-]
ZINC08439762	4.77	-0.86	-54.25	0	9	-1	543.643	10	CCSCCOC(=O)C1=C(N=C2C[C@@H](CC(=O)C2[C@H]1c3cc(c(c(c3)OC)[O-])[N+](=O)[O-])c4cccs4)C
ZINC08439762	4.96	-1.73	-50.91	0	9	-1	543.643	10	CCSCCOC(=O)C1[C@@H](C2C(=NC1=C)C[C@@H](CC2=O)c3cccs3)c4cc(c(c(c4)OC)[O-])[N+](=O)[O-]
ZINC08439761	5.44	-5.42	-13.18	1	5	0	426.544	4	CSc1nc2ccc(cc2s1)S(=O)(=O)Nc3ccc4c5ccccc5oc4c3
ZINC00630229	4.7	-0.48	-20.49	1	6	0	467.616	7	C[C@@H]1CCc2c(sc3c2c(=O)n(c(n3)SCC(=O)Nc4cccc(c4)C(=O)C)CC=C)C1
ZINC08439760	5.25	-0.79	-10.99	0	4	0	446.022	6	Cc1nc(cs1)CSc2nc3c(c(cs3)c4ccc(cc4)Cl)c(=O)n2CC=C

Supplementary Table S2:
Detailed table of XP Docking using Schrodinger's Glide module

ZINC_ID	xLogP	ApolarDesolvation	PolarDesolvation	H Donors	H Acceptors	Charge	MolecularWeight	RotatableBonds	SMILES
ZINC08439759	4.77	-0.63	-54.56	0	9	-1	543.643	10	CCSCCOC(=O)C1[C@H](C2=C(C[C@@H](CC2=O)c3cccs3)N=C1C)c4cc(c(c(c4)OC)[O-])[N+](=O)[O-]
ZINC08439759	4.77	-0.71	-53.94	0	9	-1	543.643	10	CCSCCOC(=O)C1=C(N=C2C[C@@H](CC(=O)C2[C@@H]1c3cc(c(c(c3)OC)[O-])[N+](=O)[O-])c4cccs4)C
ZINC08439759	4.96	-1.77	-53.66	0	9	-1	543.643	10	CCSCCOC(=O)C1[C@H](C2C(=NC1=C)C[C@@H](CC2=O)c3cccs3)c4cc(c(c(c4)OC)[O-])[N+](=O)[O-]
ZINC00630220	3.92	-1.52	-14.04	0	6	0	461.996	6	C=CCn1c(=O)c2c(csc2nc1SCC(=O)N3CCOCC3)c4ccccc4Cl
ZINC00645825	4.34	1.23	-17.82	1	7	0	458.514	8	COc1cc2ccnc(c2cc1OC)Cc3cc(c(cc3NC(=O)c4ccccc4)OC)OC
ZINC08439758	5.89	-0.14	-42.43	1	4	1	442.608	6	COc1ccc(cc1)c2csc(n2)N3CC[NH+](CC3)C(c4ccccc4)c5ccccc5
ZINC08439757	4.77	-0.65	-55	0	9	-1	543.643	10	CCSCCOC(=O)C1[C@@H](C2=C(C[C@H](CC2=O)c3cccs3)N=C1C)c4cc(c(c(c4)OC)[O-])[N+](=O)[O-]
ZINC08439757	4.77	-0.75	-57.32	0	9	-1	543.643	10	CCSCCOC(=O)C1=C(N=C2C[C@H](CC(=O)C2[C@H]1c3cc(c(c(c3)OC)[O-])[N+](=O)[O-])c4cccs4)C
ZINC08439757	4.96	-1.82	-56.61	0	9	-1	543.643	10	CCSCCOC(=O)C1[C@@H](C2C(=NC1=C)C[C@H](CC2=O)c3cccs3)c4cc(c(c(c4)OC)[O-])[N+](=O)[O-]
ZINC00630219	4.21	-3.01	-13.92	1	6	0	476.023	8	C=CCn1c(=O)c2c(csc2nc1SCC(=O)NC[C@@H]3CCCO3)c4ccccc4Cl
ZINC08439756	4.55	-2.59	-14.95	3	6	0	447.579	9	CC(=O)N[C@H](Cc1c[nH]c2c1cccc2)C(=O)NCCOc3ccc(cc3)C4CCCCC4
ZINC00630211	5.11	-1.23	-27.76	1	6	0	431.546	6	c1ccc2c(c1)nc(s2)c3ccc(cc3)NC(=O)CCSc4nnc5n4cccc5
ZINC08439755	4.77	-0.46	-51.02	0	9	-1	543.643	10	CCSCCOC(=O)C1[C@H](C2=C(C[C@H](CC2=O)c3cccs3)N=C1C)c4cc(c(c(c4)OC)[O-])[N+](=O)[O-]
ZINC08439755	4.77	-0.82	-56.6	0	9	-1	543.643	10	CCSCCOC(=O)C1=C(N=C2C[C@H](CC(=O)C2[C@@H]1c3cc(c(c(c3)OC)[O-])[N+](=O)[O-])c4cccs4)C
ZINC08439755	4.96	-1.74	-52.7	0	9	-1	543.643	10	CCSCCOC(=O)C1[C@H](C2C(=NC1=C)C[C@H](CC2=O)c3cccs3)c4cc(c(c(c4)OC)[O-])[N+](=O)[O-]
ZINC08439754	4.55	-2.81	-18.25	3	6	0	447.579	9	CC(=O)N[C@@H](Cc1c[nH]c2c1cccc2)C(=O)NCCOc3ccc(cc3)C4CCCCC4
ZINC08439753	6.99	-2.38	-20.16	1	4	0	455.608	6	Cc1cc(nc2c1cccc2)SCCC(=O)Nc3ccc(cc3)c4nc5ccccc5s4
ZINC00630201	5.87	1.12	-20.56	1	5	0	436.606	7	CC(C)(C)c1ccc(cc1)NC(=O)CCSc2nnc3n2c(cs3)c4ccccc4
ZINC02502423	2.59	5.53	-23.63	3	8	0	427.511	5	Cc1cc2ccccc2n3c1nnc3SCC(=O)Nc4ccc(cc4)S(=O)(=O)N
ZINC02171881	1.15	-0.19	-18.51	2	10	0	490.574	9	c1ccc(cc1)n2c(nnc2SCC#N)[C@@H]([C@H](c3nnc(n3c4ccccc4)SCC#N)O)O
ZINC08439752	5	0.09	-50.9	0	9	-1	511.576	8	CC1=NC2=C([C@@H](C1C(=O)OCC(C)C)c3cc(c(c(c3)OC)[O-])[N+](=O)[O-])C(=O)C[C@@H](C2)c4cccs4
ZINC08439752	5.18	0.32	-50.74	0	9	-1	511.576	8	CC(C)COC(=O)C1[C@H](C2C(=NC1=C)C[C@H](CC2=O)c3cccs3)c4cc(c(c(c4)OC)[O-])[N+](=O)[O-]
ZINC08439752	5.18	1.31	-50.89	0	9	-1	511.576	8	CC(C)COC(=O)C1[C@H]([C@@H]2C(=NC1=C)C[C@H](CC2=O)c3cccs3)c4cc(c(c(c4)OC)[O-])[N+](=O)[O-]
ZINC08439752	5	0.2	-52.98	0	9	-1	511.576	8	CC1=C([C@H]([C@H]2C(=N1)C[C@H](CC2=O)c3cccs3)c4cc(c(c(c4)OC)[O-])[N+](=O)[O-])C(=O)OCC(C)C
ZINC02171879	1.15	-0.19	-18.53	2	10	0	490.574	9	c1ccc(cc1)n2c(nnc2SCC#N)[C@H]([C@@H](c3nnc(n3c4ccccc4)SCC#N)O)O
ZINC00630196	4.9	-0.17	-21.95	1	6	0	444.969	7	COc1ccc(cc1)NC(=O)CCSc2nnc3n2c(cs3)c4ccc(cc4)Cl
ZINC02171875	5.13	-0.43	-12.65	2	5	0	450.538	9	c1ccc(cc1)C(c2ccccc2)C(=O)Nc3ccccc3C(=O)NCCOc4ccccc4
ZINC00630191	4.74	11.36	-14.53	1	5	0	418.906	5	c1cc(ccc1c2csc3n2c(nn3)SCC(=O)Nc4ccc(cc4)F)Cl
ZINC08439751	5	-0.02	-54.12	0	9	-1	511.576	8	CC1=NC2=C([C@H](C1C(=O)OCC(C)C)c3cc(c(c(c3)OC)[O-])[N+](=O)[O-])C(=O)C[C@@H](C2)c4cccs4
ZINC08439751	5.18	1.29	-57.68	0	9	-1	511.576	8	CC(C)COC(=O)C1[C@@H]([C@H]2C(=NC1=C)C[C@H](CC2=O)c3cccs3)c4cc(c(c(c4)OC)[O-])[N+](=O)[O-]
ZINC08439751	5.18	0.05	-55.43	0	9	-1	511.576	8	CC(C)COC(=O)C1[C@@H](C2C(=NC1=C)C[C@H](CC2=O)c3cccs3)c4cc(c(c(c4)OC)[O-])[N+](=O)[O-]
ZINC08439751	5	0.09	-56.62	0	9	-1	511.576	8	CC1=C([C@@H]([C@H]2C(=N1)C[C@H](CC2=O)c3cccs3)c4cc(c(c(c4)OC)[O-])[N+](=O)[O-])C(=O)OCC(C)C
ZINC00381968	3.73	7.03	-9.26	0	3	0	338.226	2	c1ccc(cc1)S(=O)(=O)N2CCc3c2ccc(c3)Br
ZINC00630190	5.38	-2.31	-10.05	1	4	0	367.47	6	CCOc1ccc(cc1)NS(=O)(=O)c2ccc(cc2)c3ccc(cc3)C
ZINC02171123	2.79	7.55	-9.94	1	4	0	297.354	8	c1ccc(cc1)C(=O)CCC(=O)NCCOc2ccccc2
ZINC00147704	3.06	-4.43	-13.34	1	5	0	343.763	4	c1cc2c(cc1CNS(=O)(=O)c3ccc(c(c3)Cl)F)OCO2
ZINC08439750	5	0.07	-54.49	0	9	-1	511.576	8	CC1=NC2=C([C@@H](C1C(=O)OCC(C)C)c3cc(c(c(c3)OC)[O-])[N+](=O)[O-])C(=O)C[C@H](C2)c4cccs4
ZINC08439750	5.18	0.21	-52.51	0	9	-1	511.576	8	CC(C)COC(=O)C1[C@H](C2C(=NC1=C)C[C@@H](CC2=O)c3cccs3)c4cc(c(c(c4)OC)[O-])[N+](=O)[O-]
ZINC08439750	5.18	1.36	-58.13	0	9	-1	511.576	8	CC(C)COC(=O)C1[C@H]([C@@H]2C(=NC1=C)C[C@@H](CC2=O)c3cccs3)c4cc(c(c(c4)OC)[O-])[N+](=O)[O-]
ZINC08439750	5	0.13	-53.12	0	9	-1	511.576	8	CC1=C([C@H]([C@H]2C(=N1)C[C@@H](CC2=O)c3cccs3)c4cc(c(c(c4)OC)[O-])[N+](=O)[O-])C(=O)OCC(C)C
ZINC04112603	3.3	-1.66	-57.18	1	6	1	429.44	5	c1cc(cc(c1)S(=O)(=O)N2CC[NH+](CC2)Cc3ccc4c(c3)OCO4)C(F)(F)F
ZINC00645788	4.4	-0.55	-11.15	2	6	0	436.533	8	CCOC(=O)c1c(c(sc1NC(=O)c2ccc(cc2)C)C(=O)NCc3ccccc3)C
ZINC13545842	4.64	10.58	-18.95	2	5	0	436.986	3	CC(=O)Oc1cc2c(cc1Cl)[C@H](C[C@]3(O2)CC(NC(=S)N3)(C)C)c4cccs4
ZINC13545838	4.64	10.06	-18.69	2	5	0	436.986	3	CC(=O)Oc1cc2c(cc1Cl)[C@@H](C[C@]3(O2)CC(NC(=S)N3)(C)C)c4cccs4
ZINC01013300	4.99	-0.81	-19.48	1	7	0	457.559	5	Cc1cc(c(c(c1)C)NC(=O)CSc2nc3cc(cc(c3o2)n4nc5ccccc5n4)C)C
ZINC08439749	5	0.2	-52.19	0	9	-1	511.576	8	CC1=NC2=C([C@H](C1C(=O)OCC(C)C)c3cc(c(c(c3)OC)[O-])[N+](=O)[O-])C(=O)C[C@H](C2)c4cccs4
ZINC08439749	5.18	0.23	-51.92	0	9	-1	511.576	8	CC(C)COC(=O)C1[C@@H](C2C(=NC1=C)C[C@@H](CC2=O)c3cccs3)c4cc(c(c(c4)OC)[O-])[N+](=O)[O-]
ZINC08439749	5.18	1.44	-52.89	0	9	-1	511.576	8	CC(C)COC(=O)C1[C@@H]([C@H]2C(=NC1=C)C[C@@H](CC2=O)c3cccs3)c4cc(c(c(c4)OC)[O-])[N+](=O)[O-]
ZINC08439749	5	-0.03	-54.81	0	9	-1	511.576	8	CC1=C([C@@H]([C@H]2C(=N1)C[C@@H](CC2=O)c3cccs3)c4cc(c(c(c4)OC)[O-])[N+](=O)[O-])C(=O)OCC(C)C
ZINC13545844	4.64	10.16	-16.33	2	5	0	436.986	3	CC(=O)Oc1cc2c(cc1Cl)[C@H](C[C@@]3(O2)CC(NC(=S)N3)(C)C)c4cccs4
ZINC01013299	6.25	-0.37	-18.92	0	6	0	450.523	5	Cc1cc2c(c(c1)n3nc4ccccc4n3)oc(n2)SCC(=O)c5ccc6ccccc6c5
ZINC13545840	4.64	10.57	-17.95	2	5	0	436.986	3	CC(=O)Oc1cc2c(cc1Cl)[C@@H](C[C@@]3(O2)CC(NC(=S)N3)(C)C)c4cccs4
ZINC02171098	6.36	1.6	-12.53	0	3	0	418.521	3	c1ccc(cc1)n2c(=O)c3ccccc3nc2SC4c5ccccc5-c6c4cccc6
ZINC00630103	4.75	6.28	-18.78	0	7	0	478.541	7	CC(=O)Oc1ccc2c(c1)O[C@@]3(CCC[C@H]3C24CCCC4)c5ccc(cc5OC(=O)C)OC(=O)C
ZINC08439748	5.47	0.94	-10.83	0	7	0	466.559	7	CC1=NC2=C([C@@H](C1C(=O)OCC(C)C)c3cccc(c3)[N+](=O)[O-])C(=O)C[C@@H](C2)c4cccs4
ZINC08439748	5.66	1.17	-11.29	0	7	0	466.559	7	CC(C)COC(=O)C1[C@H](C2C(=NC1=C)C[C@H](CC2=O)c3cccs3)c4cccc(c4)[N+](=O)[O-]
ZINC08439748	5.66	2.39	-19.72	0	7	0	466.559	7	CC(C)COC(=O)C1[C@H]([C@@H]2C(=NC1=C)C[C@H](CC2=O)c3cccs3)c4cccc(c4)[N+](=O)[O-]
ZINC08439748	5.47	1.05	-13.19	0	7	0	466.559	7	CC1=C([C@H]([C@H]2C(=N1)C[C@H](CC2=O)c3cccs3)c4cccc(c4)[N+](=O)[O-])C(=O)OCC(C)C
ZINC08439747	3.23	-3.63	-16.2	1	6	0	430.86	4	Cc1ccc(cc1S(=O)(=O)N2CCOCC2)NC(=O)c3cc(cc(cc3Cl)F)F
ZINC00630102	4.75	5.72	-20.06	0	7	0	478.541	7	CC(=O)Oc1ccc2c(c1)O[C@]3(CCC[C@@H]3C24CCCC4)c5ccc(cc5OC(=O)C)OC(=O)C
ZINC08439746	6.3	-2.66	-12.68	2	5	0	434.561	11	c1ccc(cc1)C[C@H](C(=O)NCCSc2ccccc2)NC(=O)OCc3ccccc3

Supplementary Table S2:
Detailed table of XP Docking using Schrodinger's Glide module

ZINC_ID	xLogP	ApolarDesolvation	PolarDesolvation	H Donors	H Acceptors	Charge	MolecularWeight	RotatableBonds	SMILES
ZINC00630105	4.75	6.66	-18.01	0	7	0	478.541	7	CC(=O)Oc1ccc2c(c1)O[C@@]3(CCC[C@@H]3C24CCCC4)c5ccc(cc5OC(=O)C)OC(=O)C
ZINC08439745	6.3	-2.66	-12.68	2	5	0	434.561	11	c1ccc(cc1)C[C@@H](C(=O)NCCSc2ccccc2)NC(=O)OCc3ccccc3
ZINC08439744	8.63	-1.1	-10.23	0	6	0	562.728	6	Cc1ccc(cc1)S(=O)(=O)Oc2cc(ccc2[C@]34CCCC[C@H]3C5(CCCCC5)c6ccc(cc6O4)OC)OC
ZINC02171084	2.8	6.71	-17.02	1	6	0	388.489	7	Cc1cc(cc(c1S(=O)(=O)NCCOc2ccccc2)C)N3CCCC3=O
ZINC08439743	5.47	0.82	-10.67	0	7	0	466.559	7	CC1=NC2=C([C@H](C1C(=O)OCC(C)C)c3cccc(c3)[N+](=O)[O-])C(=O)C[C@@H](C2)c4cccs4
ZINC08439743	5.66	0.89	-11.11	0	7	0	466.559	7	CC(C)COC(=O)C1[C@@H](C2C(=NC1=C)C[C@H](CC2=O)c3cccs3)c4cccc(c4)[N+](=O)[O-]
ZINC08439743	5.66	2.24	-18.59	0	7	0	466.559	7	CC(C)COC(=O)C1[C@@H]([C@H]2C(=NC1=C)C[C@H](CC2=O)c3cccs3)c4cccc(c4)[N+](=O)[O-]
ZINC08439743	5.47	0.93	-12.87	0	7	0	466.559	7	CC1=C([C@@H]([C@H]2C(=N1)C[C@H](CC2=O)c3cccs3)c4cccc(c4)[N+](=O)[O-])C(=O)OCC(C)C
ZINC08439742	8.63	-1.63	-18.2	0	6	0	562.728	6	Cc1ccc(cc1)S(=O)(=O)Oc2cc(ccc2[C@@]34CCCC[C@H]3C5(CCCCC5)c6ccc(cc6O4)OC)OC
ZINC02171078	6.47	0.65	-11.45	1	4	0	460.502	7	c1ccc(cc1)Oc2ccccc2NC(=O)CSc3nc4cc(ccc4s3)C(F)(F)F
ZINC08439741	8.63	-0.9	-19.26	0	6	0	562.728	6	Cc1ccc(cc1)S(=O)(=O)Oc2cc(ccc2[C@]34CCCC[C@@H]3C5(CCCCC5)c6ccc(cc6O4)OC)OC
ZINC00643564	3.22	-3.7	-13.52	0	7	0	468.325	7	c1cc(ccc1C(=O)OCC(=O)c2ccc(cc2)Br)S(=O)(=O)N3CCOCC3
ZINC08439740	8.63	-1.02	-11.34	0	6	0	562.728	6	Cc1ccc(cc1)S(=O)(=O)Oc2cc(ccc2[C@@]34CCCC[C@@H]3C5(CCCCC5)c6ccc(cc6O4)OC)OC
ZINC08439739	5.47	0.94	-10.58	0	7	0	466.559	7	CC1=NC2=C([C@@H](C1C(=O)OCC(C)C)c3cccc(c3)[N+](=O)[O-])C(=O)C[C@H](C2)c4cccs4
ZINC08439739	5.66	0.93	-10.68	0	7	0	466.559	7	CC(C)COC(=O)C1[C@H](C2C(=NC1=C)C[C@@H](CC2=O)c3cccs3)c4cccc(c4)[N+](=O)[O-]
ZINC08439739	5.66	2.39	-19.24	0	7	0	466.559	7	CC(C)COC(=O)C1[C@H]([C@@H]2C(=NC1=C)C[C@@H](CC2=O)c3cccs3)c4cccc(c4)[N+](=O)[O-]
ZINC08439739	5.47	0.98	-12.62	0	7	0	466.559	7	CC1=C([C@H]([C@H]2C(=N1)C[C@@H](CC2=O)c3cccs3)c4cccc(c4)[N+](=O)[O-])C(=O)OCC(C)C
ZINC00643559	4.85	-1.04	-14.97	1	5	0	448.32	1	Cc1ccc(cc1)N2C(=O)c3ccccc3N[C@@]24c5cc(ccc5N(C4=O)C)Br
ZINC00643556	4.59	4.47	-13.27	0	6	0	427.884	9	COC(=O)C1=CN(C=C(C1c2ccc(cc2)Cl)C(=O)OC)CCOc3ccccc3
ZINC02171063	4.8	-1.9	-9.61	1	4	0	338.451	7	CCCCOc1ccc(cc1)C(=O)Nc2cccc(c2)N3CCCC3
ZINC00643543	5.75	-0.94	-12.24	1	5	0	429.907	3	Cc1ccc(cc1)N2[C@H](Nc3ccccc3C2=O)c4cc5cc(ccc5nc4Cl)OC
ZINC08439735	5.47	0.94	-10.82	0	7	0	466.559	7	CC1=NC2=C([C@H](C1C(=O)OCC(C)C)c3cccc(c3)[N+](=O)[O-])C(=O)C[C@H](C2)c4cccs4
ZINC08439735	5.66	1.17	-11.15	0	7	0	466.559	7	CC(C)COC(=O)C1[C@@H](C2C(=NC1=C)C[C@@H](CC2=O)c3cccs3)c4cccc(c4)[N+](=O)[O-]
ZINC08439735	5.66	2.16	-11.27	0	7	0	466.559	7	CC(C)COC(=O)C1[C@@H]([C@H]2C(=NC1=C)C[C@@H](CC2=O)c3cccs3)c4cccc(c4)[N+](=O)[O-]
ZINC08439735	5.47	1.06	-13.17	0	7	0	466.559	7	CC1=C([C@@H]([C@@H]2C(=N1)C[C@@H](CC2=O)c3cccs3)c4cccc(c4)[N+](=O)[O-])C(=O)OCC(C)C
ZINC00643544	5.75	-0.97	-12.48	1	5	0	429.907	3	Cc1ccc(cc1)N2[C@@H](Nc3ccccc3C2=O)c4cc5cc(ccc5nc4Cl)OC
ZINC08439733	5.88	2.6	-13.53	0	4	0	492.442	8	Cc1ccc(cc1)S[C@H](c2ccccc2)[C@H](Cn3cncn3)C(=O)c4ccc(cc4)Br
ZINC02171057	5.51	-1.38	-11.65	2	5	0	421.353	5	Cc1cc(c(c(c1)C)C(=O)CSc2nnc(n2N)c3ccc(cc3Cl)Cl)C
ZINC08439732	5.88	2.46	-11.58	0	4	0	492.442	8	Cc1ccc(cc1)S[C@@H](c2ccccc2)[C@H](Cn3cncn3)C(=O)c4ccc(cc4)Br
ZINC08439731	5.06	-2.27	-9.85	2	5	0	454.636	11	CSCC[C@H](C(=O)NCCOc1ccc(cc1)C2CCCCC2)NC(=O)c3ccccc3
ZINC08439730	5.88	2.57	-14.12	0	4	0	492.442	8	Cc1ccc(cc1)S[C@H](c2ccccc2)[C@@H](Cn3cncn3)C(=O)c4ccc(cc4)Br
ZINC08439729	5.06	-2.27	-9.64	2	5	0	454.636	11	CSCC[C@@H](C(=O)NCCOc1ccc(cc1)C2CCCCC2)NC(=O)c3ccccc3
ZINC08439728	5.88	2.28	-9.72	0	4	0	492.442	8	Cc1ccc(cc1)S[C@@H](c2ccccc2)[C@@H](Cn3cncn3)C(=O)c4ccc(cc4)Br
ZINC02171029	7.98	2.1	-12.87	1	4	0	462.593	7	CCCCOc1ccc(cc1[C@H]2Nc3ccccc3C(=O)N2c4ccccc4)c5ccc(cc5)C
ZINC00630097	5.43	2.27	-13.45	0	4	0	478.415	8	c1ccc(cc1)[C@H]([C@H](Cn2cncn2)C(=O)c3ccc(cc3)Br)Sc4ccccc4
ZINC02171032	7.98	2.09	-12.87	1	4	0	462.593	7	CCCCOc1ccc(cc1[C@@H]2Nc3ccccc3C(=O)N2c4ccccc4)c5ccc(cc5)C
ZINC00630094	5.43	2.14	-11.61	0	4	0	478.415	8	c1ccc(cc1)[C@@H]([C@H](Cn2cncn2)C(=O)c3ccc(cc3)Br)Sc4ccccc4
ZINC00630092	5.43	2.23	-14.32	0	4	0	478.415	8	c1ccc(cc1)[C@H]([C@@H](Cn2cncn2)C(=O)c3ccc(cc3)Br)Sc4ccccc4
ZINC02171026	4.52	0.4	-10	2	5	0	430.838	7	c1ccc(cc1)OCCNC(=O)c2ccccc2NC(=O)c3cc(c(cc3Cl)F)F
ZINC00381912	4.69	-2.42	-8.96	2	3	0	334.463	5	Cc1ccc(cc1)NC[C@H](Cn2c3ccccc3c4c2CCCC4)O
ZINC00381911	4.69	9.7	-6.45	2	3	0	334.463	5	Cc1ccc(cc1)NC[C@@H](Cn2c3ccccc3c4c2CCCC4)O
ZINC00630089	5.43	2.22	-8.9	0	4	0	478.415	8	c1ccc(cc1)[C@@H]([C@@H](Cn2cncn2)C(=O)c3ccc(cc3)Br)Sc4ccccc4
ZINC00643502	3.41	0.28	-14.61	2	7	0	434.492	9	COc1cc(cc(c1OC)OC)C(=O)Nc2ccccc2C(=O)NCCc3ccccc3
ZINC00630075	4.67	3.02	-10.29	0	5	0	429.545	9	COc1ccc(cc1)C(=O)[C@H](Cn2cncn2)[C@H](c3ccccc3)Sc4ccccc4
ZINC00630073	4.67	2.94	-13.12	0	5	0	429.545	9	COc1ccc(cc1)C(=O)[C@@H](Cn2cncn2)[C@H](c3ccccc3)Sc4ccccc4
ZINC08439724	4.76	-2.72	-20.86	3	8	0	473.558	7	Cc1ccc(cc1C)OCc2nnc(n2N)SCC(=O)Nc3ccc4c5ccccc5oc4c3
ZINC00630070	4.67	3.02	-16.26	0	5	0	429.545	9	COc1ccc(cc1)C(=O)[C@H](Cn2cncn2)[C@@H](c3ccccc3)Sc4ccccc4
ZINC08439723	0.97	-2.9	-62.01	3	10	-1	456.438	5	CCOc1ccc(cc1)c2c3c([nH]n2)OC(=C([C@]34c5ccccc5N(C4=O)CC(=O)[O-])C#N)N
ZINC08439722	0.97	-2.45	-68.13	3	10	-1	456.438	5	CCOc1ccc(cc1)c2c3c([nH]n2)OC(=C([C@@]34c5ccccc5N(C4=O)CC(=O)[O-])C#N)N
ZINC00643498	3.17	-1.34	-13.12	3	7	0	423.42	8	c1ccc(cc1)OCc2nnc(n2N)SCC(=O)Nc3ccccc3C(F)(F)F
ZINC00630068	4.67	3.07	-15.42	0	5	0	429.545	9	COc1ccc(cc1)C(=O)[C@@H](Cn2cncn2)[C@@H](c3ccccc3)Sc4ccccc4
ZINC00717443	-0.26	-5.46	-15.4	2	12	0	373.377	3	Cn1c2c(c(=O)n(c1=O)C)n(cn2)Cc3nc(nc(n3)N4CCOCC4)N
ZINC00717443	-0.26	-5.2	-50.52	3	12	1	374.385	3	Cn1c2c(c(=O)n(c1=O)C)n(c[nH+]2)Cc3nc(nc(n3)N4CCOCC4)N
ZINC00630064	5.3	17.13	-14.16	0	4	0	433.964	8	c1ccc(cc1)[C@H]([C@H](Cn2cncn2)C(=O)c3ccc(cc3)Cl)Sc4ccccc4
ZINC08439721	4.42	0.27	-17.87	2	8	0	499.596	11	C=CCn1c(nnc1SCC(=O)Nc2ccccc2C(=O)Nc3ccccc3)COc4ccccc4
ZINC08439720	2.73	-2.67	-55.93	3	10	-1	418.389	5	CCOc1cc(cc(c1[O-])[N+](=O)[O-])[C@H]2c3c(n[nH]c3OC(=C2C#N)N)c4ccccc4
ZINC00630061	5.3	16.76	-11.92	0	4	0	433.964	8	c1ccc(cc1)[C@@H]([C@H](Cn2cncn2)C(=O)c3ccc(cc3)Cl)Sc4ccccc4
ZINC00643494	3.85	-1.85	-11.11	2	6	0	428.535	8	CCOC(=O)c1c(c(sc1NC(=O)c2cccs2)C(=O)NCc3ccccc3)C
ZINC08439719	2.73	-2.67	-56.11	3	10	-1	418.389	5	CCOc1cc(cc(c1[O-])[N+](=O)[O-])[C@@H]2c3c(n[nH]c3OC(=C2C#N)N)c4ccccc4
ZINC00630058	5.3	17.03	-14.72	0	4	0	433.964	8	c1ccc(cc1)[C@H]([C@@H](Cn2cncn2)C(=O)c3ccc(cc3)Cl)Sc4ccccc4
ZINC00630054	5.3	16.46	-8.93	0	4	0	433.964	8	c1ccc(cc1)[C@@H]([C@@H](Cn2cncn2)C(=O)c3ccc(cc3)Cl)Sc4ccccc4

Supplementary Table S2:
Detailed table of XP Docking using Schrodinger's Glide module

ZINC_ID	xLogP	ApolarDesolvation	PolarDesolvation	H Donors	H Acceptors	Charge	MolecularWeight	RotatableBonds	SMILES
ZINC08439716	3.56	7.19	-18.8	3	10	0	499.527	8	CCCCOc1ccc(cc1)c2c3c([nH]n2)OC(=C([C@]34c5ccccc5N(C4=O)CC(=O)OC)C#N)N
ZINC00643493	1.8	5.77	-19.97	1	7	0	420.263	5	c1cc(ccc1NC(=O)c2cc(cnc2)Br)OCC(=O)N3CCOCC3
ZINC00381903	2.12	7.97	-15.82	0	2	0	213.28	0	Cn1c2ccccc2c(=O)c3c1CCCC3
ZINC00630050	5.3	2.88	-14.22	0	4	0	433.964	8	c1ccc(cc1)[C@H]([C@H](Cn2cncn2)C(=O)c3ccccc3)Sc4ccc(cc4)Cl
ZINC08439715	3.56	6.7	-16.41	3	10	0	499.527	8	CCCCOc1ccc(cc1)c2c3c([nH]n2)OC(=C([C@@]34c5ccccc5N(C4=O)CC(=O)OC)C#N)N
ZINC00630047	5.3	2.75	-11.04	0	4	0	433.964	8	c1ccc(cc1)[C@@H]([C@H](Cn2cncn2)C(=O)c3ccccc3)Sc4ccc(cc4)Cl
ZINC02171008	6.56	-0.89	-14.39	1	6	0	486.622	8	CCOc1ccc(cc1)Nc2nc(cs2)CSc3nc4ccccc4c(=O)n3c5ccccc5
ZINC00630043	5.3	2.56	-7.48	0	4	0	433.964	8	c1ccc(cc1)[C@H]([C@@H](Cn2cncn2)C(=O)c3ccccc3)Sc4ccc(cc4)Cl
ZINC00643489	3.06	-0.86	-14.69	2	7	0	448.519	10	COc1cc(cc(c1OC)OC)CNC(=O)[C@H](Cc2ccccc2)NC(=O)c3ccccc3
ZINC00630040	5.3	2.59	-10.65	0	4	0	433.964	8	c1ccc(cc1)[C@@H]([C@@H](Cn2cncn2)C(=O)c3ccccc3)Sc4ccc(cc4)Cl
ZINC00643487	3.06	-0.86	-14.94	2	7	0	448.519	10	COc1cc(cc(c1OC)OC)CNC(=O)[C@@H](Cc2ccccc2)NC(=O)c3ccccc3
ZINC08439712	6.61	16.17	-20.14	0	6	0	488.666	13	CCCOP(=S)(OCCC)SCCCSc1nn2c(nnc2s1)c3ccccc3
ZINC02170998	6.2	-2.43	-11.53	1	4	0	445.975	2	c1ccc(cc1)N2[C@H](Nc3c(c4c(s3)CCCC4)C2=O)c5cc6ccccc6nc5Cl
ZINC08439711	5.42	4.88	-19.62	3	7	0	442.89	9	CCO[P@](=S)(NC(=S)NNc1nc2ccccc2o1)Oc3ccccc3Cl
ZINC00717417	3.23	2.72	-13.33	0	9	0	431.452	4	Cn1c2c(c(=O)n(c1=O)C)n3cc(n(c3n2)c4ccc(c(c4)OC)OC)c5ccccc5
ZINC02171001	6.2	-2.43	-11.54	1	4	0	445.975	2	c1ccc(cc1)N2[C@@H](Nc3c(c4c(s3)CCCC4)C2=O)c5cc6ccccc6nc5Cl
ZINC08439709	5.42	4.13	-18.11	3	7	0	442.89	9	CCO[P@@](=S)(NC(=S)NNc1nc2ccccc2o1)Oc3ccccc3Cl
ZINC00643478	3.62	-1.29	-15.88	2	7	0	444.516	6	Cn1c(=O)c2ccccc2nc1SCC(=O)Nc3ccccc3C(=O)Nc4ccccc4
ZINC08439707	4.14	0.54	-6.82	0	4	0	422.735	6	c1cc(ccc1C(=O)[C@H](CSc2ccc(cc2)Cl)n3cncn3)Br
ZINC02170984	3.89	0.76	-18.15	1	7	0	472.52	5	CCN(CC)C(=O)CN1c2ccccc2[C@]3(C1=O)Nc4ccccc4C(=O)N3c5ccc(cc5)F
ZINC08439706	4.14	0.53	-6.71	0	4	0	422.735	6	c1cc(ccc1C(=O)[C@@H](CSc2ccc(cc2)Cl)n3cncn3)Br
ZINC08439705	4.55	10.8	-17.67	2	6	0	453.56	7	CCOc1cc(ccc1O)[C@H]2C3=C(C[C@H](CC3=O)c4cccs4)NC(=C2C(=O)OCC)C
ZINC08439705	4.67	-2.79	-8.63	1	6	0	453.56	7	CCOc1cc(ccc1O)[C@H]2C3C(=O)C[C@@H](C=C3N=C(C2C(=O)OCC)C)c4cccs4
ZINC02170981	3.89	0.77	-18.07	1	7	0	472.52	5	CCN(CC)C(=O)CN1c2ccccc2[C@@]3(C1=O)Nc4ccccc4C(=O)N3c5ccc(cc5)F
ZINC00630007	5.52	17.97	-14.83	0	4	0	427.573	8	Cc1ccc(cc1)[C@H]([C@H](Cn2cncn2)C(=O)c3ccccc3)Sc4ccc(cc4)C
ZINC00630004	5.52	17.59	-12.38	0	4	0	427.573	8	Cc1ccc(cc1)[C@@H]([C@H](Cn2cncn2)C(=O)c3ccccc3)Sc4ccc(cc4)C
ZINC00643455	6.04	1.98	-19.69	1	7	0	468.509	7	CCOC(=O)c1ccc(cc1)c2ccc(o2)[C@@H]3Nc4ccccc4C(=O)N3c5ccccc5OC
ZINC00630000	5.52	17.87	-15.28	0	4	0	427.573	8	Cc1ccc(cc1)[C@H]([C@@H](Cn2cncn2)C(=O)c3ccccc3)Sc4ccc(cc4)C
ZINC08439704	4.55	10.79	-17.46	2	6	0	453.56	7	CCOc1cc(ccc1O)[C@@H]2C3=C(C[C@H](CC3=O)c4cccs4)NC(=C2C(=O)OCC)C
ZINC08439704	4.67	-2.78	-10.73	1	6	0	453.56	7	CCOc1cc(ccc1O)[C@@H]2C3C(=O)C[C@@H](C=C3N=C(C2C(=O)OCC)C)c4cccs4
ZINC02170961	4.7	-1.43	-13.27	2	5	0	440.952	8	CSc1ccc(c(c1)C(=O)Nc2ccccc2C(=O)NCCOc3ccccc3)Cl
ZINC00629997	5.52	17.29	-9.46	0	4	0	427.573	8	Cc1ccc(cc1)[C@@H]([C@@H](Cn2cncn2)C(=O)c3ccccc3)Sc4ccc(cc4)C
ZINC08439703	4.55	10.79	-17.55	2	6	0	453.56	7	CCOc1cc(ccc1O)[C@H]2C3=C(C[C@@H](CC3=O)c4cccs4)NC(=C2C(=O)OCC)C
ZINC08439703	4.67	-2.91	-8.95	1	6	0	453.56	7	CCOc1cc(ccc1O)[C@H]2C3C(=O)C[C@H](C=C3N=C(C2C(=O)OCC)C)c4cccs4
ZINC00643446	3.56	1.97	-15.82	0	7	0	419.462	6	c1ccc(cc1)n2c(nnc2SCC(=O)c3ccc4c(c3)OCCO4)c5ccco5
ZINC08439699	6.72	-1.37	-10.9	2	6	0	446.547	11	Cc1cccc(c1OCCNC(=O)[C@H](Cc2ccccc2)NC(=O)OCc3ccccc3)C
ZINC08439698	6.72	-1.37	-11.46	2	6	0	446.547	11	Cc1cccc(c1OCCNC(=O)[C@@H](Cc2ccccc2)NC(=O)OCc3ccccc3)C
ZINC08439697	7.11	1.95	-38.69	2	6	1	598.767	8	CCOC(=O)[C@@H]1C[NH+](CC[C@@]12c3c(c4ccccc4[nH]3)CCN2C(=O)C(c5ccccc5)c6ccccc6)Cc7ccccc7
ZINC02170947	5.82	3.1	-13.45	0	5	0	422.513	6	c1ccc(cc1)c2ccc(cc2)C(=O)CSc3nnnn3c4cccc5c4cccc5
ZINC01001468	4.94	-0.26	-15.97	0	8	0	511.405	4	Cn1c2c(nc1Sc3nc4ccccc4n3Cc5cccc(c5)Br)n(c(=O)n(c2=O)C)C
ZINC08439694	4.55	-2.94	-12.48	0	8	0	507.583	6	CC1(OC[C@H](O1)[C@H]2[C@@H]([C@@H]3[C@@H](O2)OC(O3)(C)C)N4[C@@H]([C@@H](C4=O)Oc5ccccc5)/C=C/c6ccccc6)C
ZINC00968794	5.29	0.26	-13.1	1	5	0	465.578	8	c1ccc(cc1)c2c(n(c(n2)SCC(=O)NCc3ccco3)c4ccccc4)c5ccccc5
ZINC08439693	6.6	-2.42	-11.95	2	6	0	452.938	11	c1ccc(cc1)C[C@H](C(=O)NCCOc2ccc(cc2)Cl)NC(=O)OCc3ccccc3
ZINC08439692	4.55	-2.68	-12.44	0	8	0	507.583	6	CC1(OC[C@@H](O1)[C@H]2[C@@H]([C@@H]3[C@@H](O2)OC(O3)(C)C)N4[C@@H]([C@@H](C4=O)Oc5ccccc5)/C=C/c6ccccc6)C
ZINC08439691	5.91	-3.31	-58.53	2	3	1	431.17	3	CC1=N[C@@H]([NH2+][C@@H](C1C(=O)C)c2ccc(c(c2)Cl)Cl)c3ccc(c(c3)Cl)Cl
ZINC08439691	5.91	-1.43	-144.08	3	3	2	432.178	3	CC1=[NH+][C@@H]([NH2+][C@@H](C1C(=O)C)c2ccc(c(c2)Cl)Cl)c3ccc(c(c3)Cl)Cl
ZINC08439690	6.6	-2.42	-11.54	2	6	0	452.938	11	c1ccc(cc1)C[C@@H](C(=O)NCCOc2ccc(cc2)Cl)NC(=O)OCc3ccccc3
ZINC08439689	4.55	-1.3	-10.62	0	8	0	507.583	6	CC1(OC[C@H](O1)[C@@H]2[C@@H]([C@@H]3[C@@H](O2)OC(O3)(C)C)N4[C@@H]([C@@H](C4=O)Oc5ccccc5)/C=C/c6ccccc6)C
ZINC08439688	5.91	-3.54	-53.01	2	3	1	431.17	3	CC1=N[C@@H]([NH2+][C@H](C1C(=O)C)c2ccc(c(c2)Cl)Cl)c3ccc(c(c3)Cl)Cl
ZINC08439688	5.91	-1.59	-137.87	3	3	2	432.178	3	CC1=[NH+][C@@H]([NH2+][C@H](C1C(=O)C)c2ccc(c(c2)Cl)Cl)c3ccc(c(c3)Cl)Cl
ZINC08439687	8.87	2.31	-12.68	1	6	0	520.743	17	CCCCCCCCCCOc1ccc(cc1)c2nnc(n2CC=C)SCC(=O)Nc3ccccc3C
ZINC08439686	4.55	-1.42	-11.82	0	8	0	507.583	6	CC1(OC[C@@H](O1)[C@@H]2[C@@H]([C@@H]3[C@@H](O2)OC(O3)(C)C)N4[C@@H]([C@@H](C4=O)Oc5ccccc5)/C=C/c6ccccc6)C
ZINC08439685	5.91	-3.58	-49.62	2	3	1	431.17	3	CC1=N[C@H]([NH2+][C@@H](C1C(=O)C)c2ccc(c(c2)Cl)Cl)c3ccc(c(c3)Cl)Cl
ZINC08439685	5.91	-1.62	-138.26	3	3	2	432.178	3	CC1=[NH+][C@H]([NH2+][C@@H](C1C(=O)C)c2ccc(c(c2)Cl)Cl)c3ccc(c(c3)Cl)Cl
ZINC02170920	6.99	3.17	-13.29	0	6	0	500.62	10	CCc1c(nc(s1)N(c2ccc(cc2)C(=O)OC)C(=O)Cc3ccccc3)c4ccc(cc4)OCC
ZINC08439684	5.91	-3.34	-57.44	2	3	1	431.17	3	CC1=N[C@H]([NH2+][C@H](C1C(=O)C)c2ccc(c(c2)Cl)Cl)c3ccc(c(c3)Cl)Cl
ZINC08439684	5.91	-1.45	-142.15	3	3	2	432.178	3	CC1=[NH+][C@H]([NH2+][C@H](C1C(=O)C)c2ccc(c(c2)Cl)Cl)c3ccc(c(c3)Cl)Cl
ZINC08439683	6.45	3.5	-20.72	0	6	0	492.575	8	Cc1cccc(c1)N2[C@H]([C@H](C2=O)Oc3ccccc3)N(c4cccc(c4)C)C(=O)COc5ccccc5
ZINC02170915	7.78	3.09	-10.3	0	4	0	470.638	8	CCc1c(nc(s1)N(c2ccc(cc2C)C)C(=O)CCc3ccccc3)c4ccc(cc4)OC
ZINC08439682	6.88	0.84	-7.97	0	7	0	513.425	5	Cn1c2c(c(=O)n(c1=O)C)n(c(n2)Oc3ccc(cc3C4CCCCC4)Cl)Cc5cccc(c5)Cl
ZINC08439682	6.88	1.21	-36.76	1	7	1	514.433	5	Cn1c2c(c(=O)n(c1=O)C)n(c([nH+]2)Oc3ccc(cc3C4CCCCC4)Cl)Cc5cccc(c5)Cl

Supplementary Table S2:
Detailed table of XP Docking using Schrodinger's Glide module

ZINC_ID	xLogP	ApolarDesolvation	PolarDesolvation	H Donors	H Acceptors	Charge	MolecularWeight	RotatableBonds	SMILES
ZINC08439681	6.45	2.9	-18.57	0	6	0	492.575	8	Cc1cccc(c1)N2[C@@H]([C@H](C2=O)Oc3ccccc3)N(c4cccc(c4)C)C(=O)COc5ccccc5
ZINC02170910	2.17	6.71	-23.68	1	6	0	358.419	7	Cc1ccccc1C(=O)OCCCNC2=NS(=O)(=O)c3c2cccc3
ZINC08439680	5.86	-3.22	-14.53	2	7	0	609.571	8	Cc1ccc(s1)[C@H]2C(C(=NC(=C2C(=O)Nc3ccccc3OC)C)SCC(=O)Nc4ccc(cc4)Br)C#N
ZINC08439680	5.48	-3.26	-16.97	2	7	0	609.571	8	Cc1ccc(s1)[C@@H]2C(C(=NC(=C2C#N)SCC(=O)Nc3ccc(cc3)Br)C)C(=O)Nc4ccccc4OC
ZINC00643427	5.16	0.81	-12.1	2	5	0	436.511	7	COc1ccccc1NC(=O)c2ccccc2NC(=O)C(c3ccccc3)c4ccccc4
ZINC08439679	6.45	2.94	-18.96	0	6	0	492.575	8	Cc1cccc(c1)N2[C@H]([C@@H](C2=O)Oc3ccccc3)N(c4cccc(c4)C)C(=O)COc5ccccc5
ZINC08439678	5.86	-3.28	-15.07	2	7	0	609.571	8	Cc1ccc(s1)[C@@H]2C(C(=NC(=C2C(=O)Nc3ccccc3OC)C)SCC(=O)Nc4ccc(cc4)Br)C#N
ZINC08439678	5.48	-3.16	-16.51	2	7	0	609.571	8	Cc1ccc(s1)[C@H]2C(C(=NC(=C2C#N)SCC(=O)Nc3ccc(cc3)Br)C)C(=O)Nc4ccccc4OC
ZINC08439677	6.45	3.12	-21.12	0	6	0	492.575	8	Cc1cccc(c1)N2[C@@H]([C@@H](C2=O)Oc3ccccc3)N(c4cccc(c4)C)C(=O)COc5ccccc5
ZINC00643426	5.63	0.7	-19.85	1	8	0	542.595	4	Cc1cccc(c1N2C(=O)c3ccccc3N[C@@]24c5ccccc5N(C4=O)CCN6C(=O)c7ccccc7C6=O)C
ZINC08439676	5.05	-2.52	-15.11	2	7	0	530.675	8	Cc1ccc(s1)[C@H]2C(C(=NC(=C2C(=O)Nc3ccccc3OC)C)SCC(=O)Nc4ccccc4)C#N
ZINC08439676	4.67	-2.41	-16.87	2	7	0	530.675	8	Cc1ccc(s1)[C@@H]2C(C(=NC(=C2C#N)SCC(=O)Nc3ccccc3)C)C(=O)Nc4ccccc4OC
ZINC08439675	5.71	2.85	-22.86	0	8	0	524.573	10	COc1ccc(cc1)N2[C@H]([C@H](C2=O)Oc3ccccc3)N(c4ccc(cc4)OC)C(=O)COc5ccccc5
ZINC02170897	8.76	2.12	-9.33	0	3	0	497.063	7	CCc1c(nc(s1)N(c2ccc3ccccc3c2)C(=O)CCc4ccccc4)c5ccc(cc5)Cl
ZINC08439674	5.71	2.49	-20.23	0	8	0	524.573	10	COc1ccc(cc1)N2[C@@H]([C@H](C2=O)Oc3ccccc3)N(c4ccc(cc4)OC)C(=O)COc5ccccc5
ZINC08439673	5.05	-2.51	-15.02	2	7	0	530.675	8	Cc1ccc(s1)[C@@H]2C(C(=NC(=C2C(=O)Nc3ccccc3OC)C)SCC(=O)Nc4ccccc4)C#N
ZINC08439673	4.67	-2.48	-17.01	2	7	0	530.675	8	Cc1ccc(s1)[C@H]2C(C(=NC(=C2C#N)SCC(=O)Nc3ccccc3)C)C(=O)Nc4ccccc4OC
ZINC02170894	7.34	2.84	-11.45	0	4	0	456.611	8	CCCc1c(nc(s1)N(c2ccccc2C)C(=O)Cc3ccccc3)c4ccc(cc4)OC
ZINC08439672	5.71	2.12	-20.9	0	8	0	524.573	10	COc1ccc(cc1)N2[C@H]([C@@H](C2=O)Oc3ccccc3)N(c4ccc(cc4)OC)C(=O)COc5ccccc5
ZINC08439671	9.37	-2.84	-9.59	1	3	0	441.681	15	CCCCCCCCCCCCCCNS(=O)(=O)c1ccc-2c(c1)Cc3c2cccc3
ZINC08439670	5.71	2.84	-22.84	0	8	0	524.573	10	COc1ccc(cc1)N2[C@@H]([C@@H](C2=O)Oc3ccccc3)N(c4ccc(cc4)OC)C(=O)COc5ccccc5
ZINC08439669	4.93	1.89	-12.52	0	7	0	455.551	10	CCCOCCOC(=O)C1[C@@H](C2=C(CC(CC2=O)(C)C)N=C1C)c3ccc(cc3)C(=O)OC
ZINC08439669	4.93	1.54	-14.56	0	7	0	455.551	10	CCCOCCOC(=O)C1=C(N=C2CC(CC(=O)C2[C@H]1c3ccc(cc3)C(=O)OC)(C)C)C
ZINC08439669	5.11	0.66	-12.89	0	7	0	455.551	10	CCCOCCOC(=O)C1[C@@H](C2C(=NC1=C)CC(CC2=O)(C)C)c3ccc(cc3)C(=O)OC
ZINC02170889	4.75	0.91	-10.6	0	6	0	453.589	6	Cc1ccc(cc1)N2[C@]3(N(C(=C(S3)C(=O)OC)C)Cc4ccccc4)SC(=N2)C(=O)C
ZINC08439668	5.95	-2.12	-13.91	0	6	0	484.577	7	Cc1ccc(cc1)S(=O)(=O)N(c2ccccc2)[C@H]3[C@@H](C(=O)N3c4ccccc4)Oc5ccccc5
ZINC08439667	4.93	1.93	-10.84	0	7	0	455.551	10	CCCOCCOC(=O)C1[C@H](C2=C(CC(CC2=O)(C)C)N=C1C)c3ccc(cc3)C(=O)OC
ZINC08439667	4.93	2.01	-13.74	0	7	0	455.551	10	CCCOCCOC(=O)C1=C(N=C2CC(CC(=O)C2[C@@H]1c3ccc(cc3)C(=O)OC)(C)C)C
ZINC08439667	5.11	1.1	-11.47	0	7	0	455.551	10	CCCOCCOC(=O)C1[C@H](C2C(=NC1=C)CC(CC2=O)(C)C)c3ccc(cc3)C(=O)OC
ZINC02170892	4.75	1.2	-11.42	0	6	0	453.589	6	Cc1ccc(cc1)N2[C@@]3(N(C(=C(S3)C(=O)OC)C)Cc4ccccc4)SC(=N2)C(=O)C
ZINC08439666	5.95	-1.52	-14.74	0	6	0	484.577	7	Cc1ccc(cc1)S(=O)(=O)N(c2ccccc2)[C@@H]3[C@@H](C(=O)N3c4ccccc4)Oc5ccccc5
ZINC02170886	8.18	1.65	-13.96	0	3	0	459.442	5	CCc1c(nc(s1)N(c2ccccc2Cl)C(=O)C3CCCCC3)c4ccc(cc4)Cl
ZINC08439665	6.8	-1.53	-14.34	0	6	0	512.631	7	Cc1ccc(cc1)S(=O)(=O)N(c2cccc(c2)C)[C@H]3[C@@H](C(=O)N3c4cccc(c4)C)Oc5ccccc5
ZINC08439664	6.8	0.38	-21.15	0	6	0	512.631	7	Cc1ccc(cc1)S(=O)(=O)N(c2cccc(c2)C)[C@@H]3[C@@H](C(=O)N3c4cccc(c4)C)Oc5ccccc5
ZINC08439663	6.69	-0.14	-12.8	2	4	0	478.636	7	c1ccc(cc1)C(CC(=O)Nc2ccc(cc2)NC(=O)C34CC5CC(C3)CC(C5)C4)c6ccccc6
ZINC08439662	3.56	1.84	-13.3	0	7	0	467.543	7	CC1=NC2=C([C@@H](C1C(=O)OC)c3ccc(c(c3)OC)OC(=O)C)C(=O)C[C@@H](C2)c4cccs4
ZINC08439662	3.74	1.9	-14.35	0	7	0	467.543	7	CC(=O)Oc1ccc(cc1OC)[C@H]2C3C(=NC(=C)C2C(=O)OC)C[C@H](CC3=O)c4cccs4
ZINC08439662	3.74	3.23	-15.33	0	7	0	467.543	7	CC(=O)Oc1ccc(cc1OC)[C@H]2[C@@H]3C(=NC(=C)C2C(=O)OC)C[C@H](CC3=O)c4cccs4
ZINC08439662	3.56	1.78	-16.9	0	7	0	467.543	7	CC1=C([C@H]([C@@H]2C(=N1)C[C@H](CC2=O)c3cccs3)c4ccc(c(c4)OC)OC(=O)C)C(=O)OC
ZINC02170874	7.44	2.28	-13.17	0	5	0	497.664	9	Cn1c(nnc1SCC(=O)c2ccc(cc2)c3ccccc3)COc4ccc(cc4)C5CCCCC5
ZINC08439659	3.56	1.85	-14.63	0	7	0	467.543	7	CC1=NC2=C([C@H](C1C(=O)OC)c3ccc(c(c3)OC)OC(=O)C)C(=O)C[C@@H](C2)c4cccs4
ZINC08439659	3.74	2.01	-14.37	0	7	0	467.543	7	CC(=O)Oc1ccc(cc1OC)[C@@H]2C3C(=NC(=C)C2C(=O)OC)C[C@H](CC3=O)c4cccs4
ZINC08439659	3.74	3.32	-23.63	0	7	0	467.543	7	CC(=O)Oc1ccc(cc1OC)[C@@H]2[C@H]3C(=NC(=C)C2C(=O)OC)C[C@H](CC3=O)c4cccs4
ZINC08439659	3.56	1.9	-15.96	0	7	0	467.543	7	CC1=C([C@@H]([C@@H]2C(=N1)C[C@H](CC2=O)c3cccs3)c4ccc(c(c4)OC)OC(=O)C)C(=O)OC
ZINC08439657	7.11	0.13	-15.36	2	7	0	571.464	6	Cc1c(c(n(n1)c2ccccc2)O)C(c3ccc(o3)c4ccc(cc4Cl)Cl)c5c(nn(c5O)c6ccccc6)C
ZINC02170872	5.44	1.87	-10.77	0	7	0	483.615	8	CCOC(=O)C1=C(N([C@]2(S1)N(N=C(S2)C(=O)OC)c3ccc(cc3)C)Cc4ccccc4)C
ZINC02170870	7.12	3.28	-11.59	0	6	0	466.603	11	CCCc1c(nc(s1)N(c2ccc(cc2)C(=O)OC)C(=O)CCC)c3ccc(cc3)OCC
ZINC08439655	4.64	-1.5	-20.82	0	5	0	476.642	5	CC1([C@@H]2CC[C@]13CS(=O)(=O)N([C@@H]3C2)[C@@H]4[C@@H](N(C4=O)Cc5ccccc5)/C=C/c6ccccc6)C
ZINC08439654	3.56	1.77	-13.86	0	7	0	467.543	7	CC1=NC2=C([C@@H](C1C(=O)OC)c3ccc(c(c3)OC)OC(=O)C)C(=O)C[C@H](C2)c4cccs4
ZINC08439654	3.74	1.89	-14.47	0	7	0	467.543	7	CC(=O)Oc1ccc(cc1OC)[C@H]2C3C(=NC(=C)C2C(=O)OC)C[C@@H](CC3=O)c4cccs4
ZINC08439654	3.74	3.01	-15.24	0	7	0	467.543	7	CC(=O)Oc1ccc(cc1OC)[C@H]2[C@@H]3C(=NC(=C)C2C(=O)OC)C[C@@H](CC3=O)c4cccs4
ZINC08439654	3.56	1.9	-16.19	0	7	0	467.543	7	CC1=C([C@H]([C@H]2C(=N1)C[C@@H](CC2=O)c3cccs3)c4ccc(c(c4)OC)OC(=O)C)C(=O)OC
ZINC08439653	4.58	-1.72	-46.84	3	8	1	574.455	4	Cc1ccc(cc1)NC(=O)CN2c3ccccc3[C@]4(C2=O)[C@H]5[C@@H]([C@@H]([NH2+]4)C)C(=O)N(C5=O)c6ccc(cc6)Br
ZINC08439652	5.2	-0.7	-21.01	0	5	0	490.669	5	C[C@@H](c1ccccc1)N2[C@H]([C@H](C2=O)N3[C@@H]4C[C@H]5CC[C@@]4(C5(C)C)CS3(=O)=O)/C=C/c6ccccc6
ZINC08439651	4.58	-1.05	-58.16	3	8	1	574.455	4	Cc1ccc(cc1)NC(=O)CN2c3ccccc3[C@@]4(C2=O)[C@H]5[C@@H]([C@@H]([NH2+]4)C)C(=O)N(C5=O)c6ccc(cc6)Br
ZINC00629977	3.9	0.3	-13.6	1	6	0	426.538	9	CCOc1cc(cc(c1OCC)OCC)C(=O)Nc2ccc(cc2)c3csc(n3)C
ZINC00387112	3.72	8.25	-10.27	1	3	0	300.427	3	Cc1nc(cs1)c2ccc(cc2)NC(=O)C3CCCCC3
ZINC08439649	3.56	1.86	-14.1	0	7	0	467.543	7	CC1=NC2=C([C@H](C1C(=O)OC)c3ccc(c(c3)OC)OC(=O)C)C(=O)C[C@H](C2)c4cccs4
ZINC08439649	3.74	1.92	-14.29	0	7	0	467.543	7	CC(=O)Oc1ccc(cc1OC)[C@@H]2C3C(=NC(=C)C2C(=O)OC)C[C@@H](CC3=O)c4cccs4
ZINC08439649	3.74	3.09	-17.15	0	7	0	467.543	7	CC(=O)Oc1ccc(cc1OC)[C@@H]2[C@H]3C(=NC(=C)C2C(=O)OC)C[C@@H](CC3=O)c4cccs4
ZINC08439649	3.56	1.81	-15.79	0	7	0	467.543	7	CC1=C([C@@H]([C@H]2C(=N1)C[C@@H](CC2=O)c3cccs3)c4ccc(c(c4)OC)OC(=O)C)C(=O)OC

Supplementary Table S2:
Detailed table of XP Docking using Schrodinger's Glide module

ZINC_ID	xLogP	ApolarDesolvation	PolarDesolvation	H Donors	H Acceptors	Charge	MolecularWeight	RotatableBonds	SMILES
ZINC00629925	5.07	0.1	-21.84	1	5	0	430.529	7	COc1ccc(cc1OC)CC(=O)Nc2cccc(c2)c3csc(n3)c4ccccc4
ZINC08439647	4.59	12.44	-23.42	1	6	0	426.501	5	c1ccc(cc1)c2ccc(cc2)S(=O)(=O)Nc3cccc(c3)c4cn5cccnc5n4
ZINC08439647	4.59	12.57	-67.66	0	6	-1	425.493	5	c1ccc(cc1)c2ccc(cc2)S(=O)(=O)[N-]c3cccc(c3)c4cn5cccnc5n4
ZINC08439646	5.08	0.38	-42.68	3	8	1	537.64	6	Cc1ccc(cc1)NC(=O)CN2c3ccccc3[C@]4(C2=O)[C@H]5[C@@H]([C@@H]([NH2+]4)CC(C)C)C(=O)N(C5=O)c6ccccc6
ZINC08439644	5.08	0.59	-50.33	3	8	1	537.64	6	Cc1ccc(cc1)NC(=O)CN2c3ccccc3[C@@]4(C2=O)[C@H]5[C@@H]([C@@H]([NH2+]4)CC(C)C)C(=O)N(C5=O)c6ccccc6
ZINC00629528	5.3	0.27	-19.82	1	4	0	453.283	3	Cc1ccn2cc(nc2c1)c3cccc(c3)NC(=O)c4cccc(c4)I
ZINC00629528	5.3	0.5	-28.5	2	4	1	454.291	3	Cc1ccn2cc([nH+]c2c1)c3cccc(c3)NC(=O)c4cccc(c4)I
ZINC08439640	6.54	-2.5	-11.77	0	4	0	467.656	7	CC(C)(C)c1ccc(cc1)S(=O)(=O)N2CCS[C@H]2c3cccc(c3)OCc4ccccc4
ZINC02170859	7.7	2.27	-16.98	0	4	0	448.632	7	CCc1c(nc(s1)N(c2ccccc2OCC)C(=O)C3CCCCC3)c4ccc(cc4)C
ZINC08439639	6.54	-2.13	-12.09	0	4	0	467.656	7	CC(C)(C)c1ccc(cc1)S(=O)(=O)N2CCS[C@@H]2c3cccc(c3)OCc4ccccc4
ZINC08439637	4.14	-0.05	-44.24	2	6	1	481.37	4	CC[C@H]1[C@H]2[C@@H](C(=O)N(C2=O)c3ccc(cc3)Br)[C@]4([NH2+]1)c5ccccc5N(C4=O)CC=C
ZINC08439636	7.8	3.84	-23.31	1	5	0	483.656	9	CCC(C)(C)c1ccc(c(c1)C(C)(C)CC)OCC(=O)Nc2cccc(c2)c3cn4ccccc4n3
ZINC08439636	7.8	4.07	-31.05	2	5	1	484.664	9	CCC(C)(C)c1ccc(c(c1)C(C)(C)CC)OCC(=O)Nc2cccc(c2)c3cn4ccccc4[nH+]3
ZINC08439635	1.08	-1.89	-12.6	2	11	0	402.411	7	Cn1c2c(c(=O)n(c1=O)C)n(c(n2)NN=Cc3ccc(cc3OC)OC)CCO
ZINC08439635	1.08	-1.63	-34.17	3	11	1	403.419	7	Cn1c2c(c(=O)n(c1=O)C)n(c([nH+]2)NN=Cc3ccc(cc3OC)OC)CCO
ZINC08439634	4.14	-0.11	-43.3	2	6	1	481.37	4	CC[C@@H]1[C@H]2[C@@H](C(=O)N(C2=O)c3ccc(cc3)Br)[C@@]4([NH2+]1)c5ccccc5N(C4=O)CC=C
ZINC08439633	7.08	2.59	-16.97	1	4	0	464.675	9	CCC(C)(C)c1ccc(c(c1)C(C)(C)CC)OCC(=O)Nc2cccc(c2)c3csc(n3)C
ZINC08439631	8.61	-4.01	-21.99	0	7	0	641.774	8	c1ccc(cc1)c2ccc(cc2)S(=O)(=O)N(c3c(nc4n3cccc4)c5ccccc5)S(=O)(=O)c6ccc(cc6)c7ccccc7
ZINC08439631	8.61	-3.83	-41.55	1	7	1	642.782	8	c1ccc(cc1)c2ccc(cc2)S(=O)(=O)N(c3c([nH+]c4n3cccc4)c5ccccc5)S(=O)(=O)c6ccc(cc6)c7ccccc7
ZINC08439630	4.1	1.54	-11.04	0	7	0	457.567	10	CCCOCCOC(=O)C1[C@@H](C2=C(CC(CC2=O)(C)C)N=C1C)c3cccc(c3OC)OC
ZINC08439630	4.1	1.05	-12.42	0	7	0	457.567	10	CCCOCCOC(=O)C1=C(N=C2CC(CC(=O)C2[C@H]1c3cccc(c3OC)OC)(C)C)C
ZINC08439630	4.29	0.46	-11.28	0	7	0	457.567	10	CCCOCCOC(=O)C1[C@@H](C2C(=NC1=C)CC(CC2=O)(C)C)c3cccc(c3OC)OC
ZINC01019636	6.85	-4.13	-22.37	0	7	0	545.686	8	CCc1ccc(cc1)S(=O)(=O)N(c2c(nc3n2cccc3)c4ccccc4)S(=O)(=O)c5ccc(cc5)CC
ZINC01019636	6.85	-3.95	-39.37	1	7	1	546.694	8	CCc1ccc(cc1)S(=O)(=O)N(c2c([nH+]c3n2cccc3)c4ccccc4)S(=O)(=O)c5ccc(cc5)CC
ZINC08439628	6.63	-6.67	-16.29	0	7	0	647.37	6	c1ccc(cc1)c2c(n3ccccc3n2)N(S(=O)(=O)c4ccc(cc4)Br)S(=O)(=O)c5ccc(cc5)Br
ZINC08439628	6.63	-6.49	-37.98	1	7	1	648.378	6	c1ccc(cc1)c2c(n3ccccc3[nH+]2)N(S(=O)(=O)c4ccc(cc4)Br)S(=O)(=O)c5ccc(cc5)Br
ZINC02170853	7.07	2.54	-12.53	0	6	0	526.706	7	Cc1ccc(cc1C)OCc2nnc(n2c3ccccc3)SCC(=O)n4c5c(c6c4CCCC6)CCCC5
ZINC08439627	4.1	1.57	-11.83	0	7	0	457.567	10	CCCOCCOC(=O)C1[C@H](C2=C(CC(CC2=O)(C)C)N=C1C)c3cccc(c3OC)OC
ZINC08439627	4.1	1.02	-13.07	0	7	0	457.567	10	CCCOCCOC(=O)C1=C(N=C2CC(CC(=O)C2[C@@H]1c3cccc(c3OC)OC)(C)C)C
ZINC08439627	4.29	0.4	-11.78	0	7	0	457.567	10	CCCOCCOC(=O)C1[C@H](C2C(=NC1=C)CC(CC2=O)(C)C)c3cccc(c3OC)OC
ZINC00629392	4.66	-0.65	-13.92	1	4	0	445.285	3	c1cc(cc(c1)I)C(=O)Nc2ccc(cc2)c3cn4ccsc4n3
ZINC00629389	4.68	10.99	-14.58	1	4	0	445.285	3	c1cc(ccc1c2cn3ccsc3n2)NC(=O)c4ccc(cc4)I
ZINC00643406	5.21	0.5	-13.23	1	6	0	458.587	9	C[C@H](c1ccccc1)NC(=O)CSc2nnc(n2Cc3ccccc3)c4ccc(cc4)OC
ZINC08439626	4.95	-3.5	-16.99	1	6	0	427.551	8	CCCCOc1ccc(cc1)S(=O)(=O)Nc2cccc(c2)c3cn4ccsc4n3
ZINC00643405	5.21	13.38	-13.26	1	6	0	458.587	9	C[C@@H](c1ccccc1)NC(=O)CSc2nnc(n2Cc3ccccc3)c4ccc(cc4)OC
ZINC08439625	3.14	-0.62	-11.09	0	7	0	480.355	8	CC1=NC2=C([C@@H](C1C(=O)OCCOC)c3cc(c(cc3Br)OC)OC)C(=O)CCC2
ZINC08439625	3.32	-0.5	-11.88	0	7	0	480.355	8	COCCOC(=O)C1[C@H](C2C(=NC1=C)CCCC2=O)c3cc(c(cc3Br)OC)OC
ZINC08439625	3.32	0.76	-12.88	0	7	0	480.355	8	COCCOC(=O)C1[C@H]([C@@H]2C(=NC1=C)CCCC2=O)c3cc(c(cc3Br)OC)OC
ZINC08439625	3.14	-0.62	-14.69	0	7	0	480.355	8	CC1=C([C@H]([C@@H]2C(=N1)CCCC2=O)c3cc(c(cc3Br)OC)OC)C(=O)OCCOC
ZINC02170850	7.71	2.73	-10.77	0	3	0	440.612	7	CCCc1c(nc(s1)N(c2ccc(c(c2)C)C)C(=O)Cc3ccccc3)c4ccccc4
ZINC02170847	5.67	1.4	-9.7	0	7	0	504.033	8	CCOC(=O)C1=C(N([C@]2(S1)N(N=C(S2)C(=O)OC)c3ccc(cc3)Cl)Cc4ccccc4)C
ZINC08439624	5.25	-2.92	-17.83	1	5	0	431.542	5	c1ccc(cc1)c2ccc(cc2)S(=O)(=O)Nc3cccc(c3)c4cn5ccsc5n4
ZINC02170845	5.67	1.12	-8.91	0	7	0	504.033	8	CCOC(=O)C1=C(N([C@@]2(S1)N(N=C(S2)C(=O)OC)c3ccc(cc3)Cl)Cc4ccccc4)C
ZINC02170839	7.15	15.98	-12.76	1	5	0	510.585	8	CC(C)(C)c1ccc(cc1)c2nnc(n2c3ccccc3)SCC(=O)Nc4cccc(c4)C(F)(F)F
ZINC08439623	3.14	-0.59	-11.05	0	7	0	480.355	8	CC1=NC2=C([C@H](C1C(=O)OCCOC)c3cc(c(cc3Br)OC)OC)C(=O)CCC2
ZINC08439623	3.32	-0.54	-12.09	0	7	0	480.355	8	COCCOC(=O)C1[C@@H](C2C(=NC1=C)CCCC2=O)c3cc(c(cc3Br)OC)OC
ZINC08439623	3.32	0.78	-12.66	0	7	0	480.355	8	COCCOC(=O)C1[C@@H]([C@H]2C(=NC1=C)CCCC2=O)c3cc(c(cc3Br)OC)OC
ZINC08439623	3.14	-0.66	-15.39	0	7	0	480.355	8	CC1=C([C@@H]([C@H]2C(=N1)CCCC2=O)c3cc(c(cc3Br)OC)OC)C(=O)OCCOC
ZINC00643400	5.46	11.79	-11.28	1	5	0	367.445	7	COC(=O)c1ccccc1NC(=O)COc2ccc(cc2)C3CCCCC3
ZINC00643398	6.16	2.21	-10.35	0	5	0	430.957	7	CCCC(=O)N(c1ccc(cc1OC)OC)c2nc(c(s2)C)c3ccc(cc3)Cl
ZINC08439620	4.91	0.51	-8.2	0	5	0	431.96	8	CCCOCCOC(=O)C1[C@@H](C2=C(CC(CC2=O)(C)C)N=C1C)c3ccccc3Cl
ZINC08439620	4.91	0.43	-11.6	0	5	0	431.96	8	CCCOCCOC(=O)C1=C(N=C2CC(CC(=O)C2[C@H]1c3ccccc3Cl)(C)C)C
ZINC08439620	5.1	-0.67	-9.23	0	5	0	431.96	8	CCCOCCOC(=O)C1[C@@H](C2C(=NC1=C)CC(CC2=O)(C)C)c3ccccc3Cl
ZINC02170835	8.6	1.87	-9.55	0	4	0	525.501	9	CCCc1c(nc(s1)N(c2ccc(c(c2)Cl)Cl)C(=O)Cc3ccccc3)c4ccc(cc4)OCC
ZINC08439619	8.2	4.08	-23.25	1	5	0	497.683	9	CCC(C)(C)c1ccc(c(c1)C(C)(C)CC)OCC(=O)Nc2cccc(c2)c3cn4cccc(c4n3)C
ZINC08439619	8.2	4.35	-30.17	2	5	1	498.691	9	CCC(C)(C)c1ccc(c(c1)C(C)(C)CC)OCC(=O)Nc2cccc(c2)c3cn4cccc(c4[nH+]3)C
ZINC05918543	7.78	18.11	-17.65	1	5	0	497.683	9	CCC(C)(C)c1ccc(c(c1)C(C)(C)CC)OCC(=O)Nc2c(nc3n2c(ccc3)C)c4ccccc4
ZINC05918543	7.78	18.56	-31.83	2	5	1	498.691	9	CCC(C)(C)c1ccc(c(c1)C(C)(C)CC)OCC(=O)Nc2c([nH+]c3n2c(ccc3)C)c4ccccc4
ZINC02170831	8.12	2.36	-10.24	0	3	0	481.448	6	CCc1c(nc(s1)N(c2ccc(cc2Cl)Cl)C(=O)Cc3ccccc3)c4ccc(cc4)C
ZINC00629164	3.62	-0.63	-14.14	0	4	0	428.105	3	c1cc(sc1)C(=O)Cn2cnc3c(c2=O)cc(cc3Br)Br
ZINC08439618	4.91	0.72	-9.35	0	5	0	431.96	8	CCCOCCOC(=O)C1[C@H](C2=C(CC(CC2=O)(C)C)N=C1C)c3ccccc3Cl

Supplementary Table S2:
Detailed table of XP Docking using Schrodinger's Glide module

ZINC_ID	xLogP	ApolarDesolvation	PolarDesolvation	H Donors	H Acceptors	Charge	MolecularWeight	RotatableBonds	SMILES
ZINC08439618	4.91	0.79	-12.2	0	5	0	431.96	8	CCCOCCOC(=O)C1=C(N=C2CC(CC(=O)C2[C@@H]1c3ccccc3Cl)(C)C)C
ZINC08439618	5.1	-0.08	-10.77	0	5	0	431.96	8	CCCOCCOC(=O)C1[C@H](C2C(=NC1=C)CC(CC2=O)(C)C)c3ccccc3Cl
ZINC02170829	6.71	1.95	-14.57	1	6	0	486.641	9	CCOc1ccc(cc1)NC(=O)CSc2nnc(n2c3ccccc3)c4ccc(cc4)C(C)(C)C
ZINC00624874	2.62	0.69	-17.59	1	6	0	430.205	3	c1ccc(c(c1)C#N)NC(=O)Cn2cnc3ccc(cc3c2=O)I
ZINC08439617	4.91	-0.65	-8.93	0	7	0	520.42	8	CCCOCCOC(=O)C1[C@@H](C2=C(CC(CC2=O)(C)C)N=C1C)c3cc4c(cc3Br)OCO4
ZINC08439617	4.91	-0.66	-12.11	0	7	0	520.42	8	CCCOCCOC(=O)C1=C(N=C2CC(CC(=O)C2[C@H]1c3cc4c(cc3Br)OCO4)(C)C)C
ZINC08439617	5.09	-1.59	-10.01	0	7	0	520.42	8	CCCOCCOC(=O)C1[C@@H](C2C(=NC1=C)CC(CC2=O)(C)C)c3cc4c(cc3Br)OCO4
ZINC02170827	7.79	2.36	-13.73	0	3	0	434.992	6	CCCCC(=O)N(c1cccc2c1cccc2)c3nc(c(s3)C)c4ccc(cc4)Cl
ZINC00643395	5.84	0.02	-18.04	1	6	0	494.016	5	Cc1c(c2cc(ccc2n1C(=O)c3ccc(cc3)Cl)OC)CC(=O)Nc4nc5c(s4)CCCC5
ZINC08439616	4.75	-0.63	-9.28	0	6	0	450.329	7	CC1=NC(=C([C@@H](C1C(=O)OC)c2cccc(c2)Br)C(=O)OC[C@H]3CCCO3)C
ZINC08439616	4.93	-0.63	-7.58	0	6	0	450.329	7	CC1=NC(=C)C([C@H](C1C(=O)OC[C@H]2CCCO2)c3cccc(c3)Br)C(=O)OC
ZINC08439616	4.93	0.72	-12.33	0	6	0	450.329	7	CC1=NC(=C)C([C@H]([C@H]1C(=O)OC[C@H]2CCCO2)c3cccc(c3)Br)C(=O)OC
ZINC08439616	4.75	-0.72	-8.32	0	6	0	450.329	7	CC1=NC(=C([C@H]([C@H]1C(=O)OC[C@H]2CCCO2)c3cccc(c3)Br)C(=O)OC)C
ZINC08439615	4.91	-0.65	-9.4	0	7	0	520.42	8	CCCOCCOC(=O)C1[C@H](C2=C(CC(CC2=O)(C)C)N=C1C)c3cc4c(cc3Br)OCO4
ZINC08439615	4.91	-1.02	-12.24	0	7	0	520.42	8	CCCOCCOC(=O)C1=C(N=C2CC(CC(=O)C2[C@@H]1c3cc4c(cc3Br)OCO4)(C)C)C
ZINC08439615	5.09	-2.08	-9.83	0	7	0	520.42	8	CCCOCCOC(=O)C1[C@H](C2C(=NC1=C)CC(CC2=O)(C)C)c3cc4c(cc3Br)OCO4
ZINC08439614	4.75	-0.57	-9.83	0	6	0	450.329	7	CC1=NC(=C([C@H](C1C(=O)OC)c2cccc(c2)Br)C(=O)OC[C@H]3CCCO3)C
ZINC08439614	4.93	-0.49	-9.82	0	6	0	450.329	7	CC1=NC(=C)C([C@@H](C1C(=O)OC[C@H]2CCCO2)c3cccc(c3)Br)C(=O)OC
ZINC08439614	4.93	0.94	-15.72	0	6	0	450.329	7	CC1=NC(=C)C([C@@H]([C@@H]1C(=O)OC[C@H]2CCCO2)c3cccc(c3)Br)C(=O)OC
ZINC08439614	4.75	-0.61	-9.59	0	6	0	450.329	7	CC1=NC(=C([C@@H]([C@@H]1C(=O)OC[C@H]2CCCO2)c3cccc(c3)Br)C(=O)OC)C
ZINC02170825	7.06	2.36	-14.41	0	4	0	438.568	6	CCc1c(nc(s1)N(c2cccc(c2)F)C(=O)C3CCCCC3)c4ccc(cc4)OC
ZINC08439613	4.75	-0.68	-9.36	0	6	0	450.329	7	CC1=NC(=C([C@@H](C1C(=O)OC)c2cccc(c2)Br)C(=O)OC[C@@H]3CCCO3)C
ZINC08439613	4.93	-0.58	-7.83	0	6	0	450.329	7	CC1=NC(=C)C([C@H](C1C(=O)OC[C@@H]2CCCO2)c3cccc(c3)Br)C(=O)OC
ZINC08439613	4.93	0.76	-10.91	0	6	0	450.329	7	CC1=NC(=C)C([C@H]([C@H]1C(=O)OC[C@@H]2CCCO2)c3cccc(c3)Br)C(=O)OC
ZINC08439613	4.75	-0.62	-8.09	0	6	0	450.329	7	CC1=NC(=C([C@H]([C@@H]1C(=O)OC[C@@H]2CCCO2)c3cccc(c3)Br)C(=O)OC)C
ZINC02170823	6.1	0.97	-13.6	0	4	0	445.382	6	CCC(=O)N(c1cccc(c1)Br)c2nc(c(s2)C)c3ccc(cc3)OCC
ZINC08439612	5.23	0.1	-13.75	0	6	0	475.585	11	CCCOCCOC(=O)C1[C@@H](C2=C(CCCC2=O)N=C1C)c3ccccc3OCc4ccccc4
ZINC08439612	5.23	-0.02	-17.16	0	6	0	475.585	11	CCCOCCOC(=O)C1=C(N=C2CCCC(=O)C2[C@H]1c3ccccc3OCc4ccccc4)C
ZINC08439612	5.42	-1.05	-14.66	0	6	0	475.585	11	CCCOCCOC(=O)C1[C@@H](C2C(=NC1=C)CCCC2=O)c3ccccc3OCc4ccccc4
ZINC08439611	4.75	-0.69	-7.03	0	6	0	450.329	7	CC1=NC(=C([C@H](C1C(=O)OC)c2cccc(c2)Br)C(=O)OC[C@@H]3CCCO3)C
ZINC08439611	4.93	-0.69	-6.7	0	6	0	450.329	7	CC1=NC(=C)C([C@@H](C1C(=O)OC[C@@H]2CCCO2)c3cccc(c3)Br)C(=O)OC
ZINC08439611	4.93	0.81	-13.48	0	6	0	450.329	7	CC1=NC(=C)C([C@@H]([C@@H]1C(=O)OC[C@@H]2CCCO2)c3cccc(c3)Br)C(=O)OC
ZINC08439611	4.75	-0.82	-6.83	0	6	0	450.329	7	CC1=NC(=C([C@@H]([C@@H]1C(=O)OC[C@@H]2CCCO2)c3cccc(c3)Br)C(=O)OC)C
ZINC02170814	5.2	0.14	-14.26	1	5	0	471.56	5	c1ccc(cc1)CN2C(=O)c3ccccc3N[C@]24c5ccccc5N(C4=O)C/C=C/c6ccccc6
ZINC08439610	5.19	4.59	-71.34	0	6	2	718.864	6	c1ccc2c(c1)n(c3[n+]2nc-4c(c3)-c5cccc6c5c4ccc6)CCCC#CC#CCCCn7c8ccccc8[n+]9c7cc-1c(n9)-c2cccc3c2c1ccc3
ZINC02170819	5.2	0.15	-14.58	1	5	0	471.56	5	c1ccc(cc1)CN2C(=O)c3ccccc3N[C@@]24c5ccccc5N(C4=O)C/C=C/c6ccccc6
ZINC08439609	5.23	0.54	-15.48	0	6	0	475.585	11	CCCOCCOC(=O)C1[C@H](C2=C(CCCC2=O)N=C1C)c3ccccc3OCc4ccccc4
ZINC08439609	5.23	0.4	-18.63	0	6	0	475.585	11	CCCOCCOC(=O)C1=C(N=C2CCCC(=O)C2[C@@H]1c3ccccc3OCc4ccccc4)C
ZINC08439609	5.42	-0.58	-15.38	0	6	0	475.585	11	CCCOCCOC(=O)C1[C@H](C2C(=NC1=C)CCCC2=O)c3ccccc3OCc4ccccc4
ZINC08439608	2.48	6.26	-67.1	0	6	2	582.796	10	CCc1cc2n(c3ccccc3[n+]2nc1CC)CCCC#CC#CCCCn4c5ccccc5[n+]6c4cc(c(n6)CC)CC
ZINC02170809	8.11	1.78	-13.71	0	4	0	470.638	6	CCc1c(nc(s1)N(c2ccc3ccccc3c2)C(=O)C4CCCCC4)c5ccc(cc5)OC
ZINC02170805	6.7	-0.25	-14.67	1	5	0	514.412	3	Cc1cccc(c1)N2C(=O)c3ccccc3N[C@]24c5ccccc5N(C4=O)Cc6c(cccc6Cl)Cl
ZINC08439607	6.04	0.47	-8.72	0	5	0	466.405	8	CCCOCCOC(=O)C1[C@@H](C2=C(CC(CC2=O)(C)C)N=C1C)c3ccc(c(c3)Cl)Cl
ZINC08439607	6.04	0.47	-11.02	0	5	0	466.405	8	CCCOCCOC(=O)C1=C(N=C2CC(CC(=O)C2[C@H]1c3ccc(c(c3)Cl)Cl)(C)C)C
ZINC08439607	6.22	-0.53	-9.35	0	5	0	466.405	8	CCCOCCOC(=O)C1[C@@H](C2C(=NC1=C)CC(CC2=O)(C)C)c3ccc(c(c3)Cl)Cl
ZINC08439606	9.17	3.95	-15	0	6	0	663.612	8	c1ccc(cc1)C(c2ccccc2)(c3ccccc3)n4nc(nn4)c5ccccc5c6ccc(cc6)Cn7cnc8c7cc(c(c8)Cl)Cl
ZINC02170807	6.7	-0.29	-15.01	1	5	0	514.412	3	Cc1cccc(c1)N2C(=O)c3ccccc3N[C@@]24c5ccccc5N(C4=O)Cc6c(cccc6Cl)Cl
ZINC00643391	6.55	2.78	-13.43	0	4	0	446.547	7	Cc1c(nc(s1)N(c2ccc(cc2)F)C(=O)CCc3ccccc3)c4ccc(cc4)OC
ZINC08439605	9.89	4.19	-16.04	0	6	0	650.83	11	CCCCc1nc2ccccc2n1Cc3ccc(cc3)c4ccccc4c5nnn(n5)C(c6ccccc6)(c7ccccc7)c8ccccc8
ZINC08439604	6.04	0.55	-7.54	0	5	0	466.405	8	CCCOCCOC(=O)C1[C@H](C2=C(CC(CC2=O)(C)C)N=C1C)c3ccc(c(c3)Cl)Cl
ZINC08439604	6.04	0.62	-10.39	0	5	0	466.405	8	CCCOCCOC(=O)C1=C(N=C2CC(CC(=O)C2[C@@H]1c3ccc(c(c3)Cl)Cl)(C)C)C
ZINC08439604	6.22	-0.24	-9.1	0	5	0	466.405	8	CCCOCCOC(=O)C1[C@H](C2C(=NC1=C)CC(CC2=O)(C)C)c3ccc(c(c3)Cl)Cl
ZINC02170802	8.36	1.18	-8.08	0	3	0	525.899	7	CCc1c(nc(s1)N(c2cccc(c2)Br)C(=O)CCc3ccccc3)c4ccc(cc4)Cl
ZINC00629054	4.56	-2.84	-14.74	2	5	0	440.396	9	COc1cc(ccc1OC2CCCC2)CCNC(=S)Nc3c(cncc3Cl)Cl
ZINC02170798	8.37	2.35	-8.14	0	3	0	453.051	5	CCc1c(nc(s1)N(c2ccc(cc2C)C)C(=O)C3CCCCC3)c4ccc(cc4)Cl
ZINC00643388	4.79	14.33	-18.01	1	7	0	487.61	8	C[C@H](C(=O)Nc1nccs1)Sc2nnc(n2c3ccccc3)COc4cccc5c4cccc5
ZINC08439603	5.57	-0.01	-9.36	0	5	0	476.411	8	CCCOCCOC(=O)C1[C@@H](C2=C(CC(CC2=O)(C)C)N=C1C)c3ccc(cc3)Br
ZINC08439603	5.57	-0.36	-11.27	0	5	0	476.411	8	CCCOCCOC(=O)C1=C(N=C2CC(CC(=O)C2[C@H]1c3ccc(cc3)Br)(C)C)C
ZINC08439603	5.75	-1.24	-9.85	0	5	0	476.411	8	CCCOCCOC(=O)C1[C@@H](C2C(=NC1=C)CC(CC2=O)(C)C)c3ccc(cc3)Br
ZINC08439602	3.15	16.24	-37.83	0	7	1	428.449	4	c1ccc2c(c1)[n+]3cc(c(cc3s2)c4cccc(c4)[N+](=O)[O-])c5cccc(c5)[N+](=O)[O-]
ZINC02170795	8.27	1.51	-9.38	0	4	0	535.507	9	CCCc1c(nc(s1)N(c2ccc(cc2)Br)C(=O)CCc3ccccc3)c4ccc(cc4)OC

Supplementary Table S2:
Detailed table of XP Docking using Schrodinger's Glide module

ZINC_ID	xLogP	ApolarDesolvation	PolarDesolvation	H Donors	H Acceptors	Charge	MolecularWeight	RotatableBonds	SMILES
ZINC08439601	3.57	3.63	-39.06	0	7	1	454.487	5	c1ccc(cc1)c2csc3[n+]2cc(c(c3)c4cccc(c4)[N+](=O)[O-])c5cccc(c5)[N+](=O)[O-]
ZINC02170789	6.57	1.59	-16.52	0	3	0	463.4	5	Cc1c(nc(s1)N(c2ccccc2Br)C(=O)Cc3ccccc3)c4ccccc4
ZINC08439600	1.57	-2.26	-12.46	3	11	0	427.417	7	CC1=C([C@H](n2cnc(c2N1)C(=O)N)c3ccccc3[N+](=O)[O-])C(=O)OC[C@H]4CCCO4
ZINC08439600	1.57	-1.95	-39.09	4	11	1	428.425	7	CC1=C([C@H](n2c[nH+]c(c2N1)C(=O)N)c3ccccc3[N+](=O)[O-])C(=O)OC[C@H]4CCCO4
ZINC08439599	5.57	0.03	-7.78	0	5	0	476.411	8	CCCOCCOC(=O)C1[C@H](C2=C(CC(CC2=O)(C)C)N=C1C)c3ccc(cc3)Br
ZINC08439599	5.57	0.11	-10.63	0	5	0	476.411	8	CCCOCCOC(=O)C1=C(N=C2CC(CC(=O)C2[C@@H]1c3ccc(cc3)Br)(C)C)C
ZINC08439599	5.75	-0.81	-8.61	0	5	0	476.411	8	CCCOCCOC(=O)C1[C@H](C2C(=NC1=C)CC(CC2=O)(C)C)c3ccc(cc3)Br
ZINC02170779	4.16	0.16	-12.43	0	6	0	432.354	4	CC1=C(S[C@]2(N1C)N(N=C(S2)C(=O)C)c3c(cccc3Cl)Cl)C(=O)OC
ZINC08439598	1.57	-2.09	-13.86	3	11	0	427.417	7	CC1=C([C@@H](n2cnc(c2N1)C(=O)N)c3ccccc3[N+](=O)[O-])C(=O)OC[C@H]4CCCO4
ZINC08439598	1.57	-1.78	-41.17	4	11	1	428.425	7	CC1=C([C@@H](n2c[nH+]c(c2N1)C(=O)N)c3ccccc3[N+](=O)[O-])C(=O)OC[C@H]4CCCO4
ZINC02170776	4.16	0.36	-12.61	0	6	0	432.354	4	CC1=C(S[C@@]2(N1C)N(N=C(S2)C(=O)C)c3c(cccc3Cl)Cl)C(=O)OC
ZINC08439597	1.57	-2.07	-13.85	3	11	0	427.417	7	CC1=C([C@H](n2cnc(c2N1)C(=O)N)c3ccccc3[N+](=O)[O-])C(=O)OC[C@@H]4CCCO4
ZINC08439596	5.01	2.23	-9.46	0	5	0	433.495	8	CCCOCCOC(=O)C1[C@@H](C2=C(CC(CC2=O)(C)C)N=C1C)c3ccc(c(c3)F)F
ZINC08439596	5.01	2.22	-11.91	0	5	0	433.495	8	CCCOCCOC(=O)C1=C(N=C2CC(CC(=O)C2[C@H]1c3ccc(c(c3)F)F)(C)C)C
ZINC08439596	5.19	1.22	-10.43	0	5	0	433.495	8	CCCOCCOC(=O)C1[C@@H](C2C(=NC1=C)CC(CC2=O)(C)C)c3ccc(c(c3)F)F
ZINC02170771	8.24	1.87	-8.69	0	4	0	477.457	9	CCCc1c(nc(s1)N(c2ccc(c(c2)Cl)Cl)C(=O)CCC)c3ccc(cc3)OCC
ZINC08439595	1.57	-2.47	-12.05	3	11	0	427.417	7	CC1=C([C@@H](n2cnc(c2N1)C(=O)N)c3ccccc3[N+](=O)[O-])C(=O)OC[C@@H]4CCCO4
ZINC08439595	1.57	-2.16	-39.57	4	11	1	428.425	7	CC1=C([C@@H](n2c[nH+]c(c2N1)C(=O)N)c3ccccc3[N+](=O)[O-])C(=O)OC[C@@H]4CCCO4
ZINC02170769	7.1	5.1	-15.55	2	4	0	549.298	8	c1ccc(cc1)CC(C(=O)Nc2cc(ccc2Cl)C(F)(F)F)C(=O)Nc3cc(ccc3Cl)C(F)(F)F
ZINC02170766	8.28	14.71	-11.33	1	4	0	469.503	7	COc1ccccc1C(=O)Nc2cc(ccc2Oc3ccc(cc3)C4CCCCC4)C(F)(F)F
ZINC08439594	5.01	2.3	-8.35	0	5	0	433.495	8	CCCOCCOC(=O)C1[C@H](C2=C(CC(CC2=O)(C)C)N=C1C)c3ccc(c(c3)F)F
ZINC08439594	5.01	2.38	-11.27	0	5	0	433.495	8	CCCOCCOC(=O)C1=C(N=C2CC(CC(=O)C2[C@@H]1c3ccc(c(c3)F)F)(C)C)C
ZINC08439594	5.19	1.51	-10.01	0	5	0	433.495	8	CCCOCCOC(=O)C1[C@H](C2C(=NC1=C)CC(CC2=O)(C)C)c3ccc(c(c3)F)F
ZINC00717214	3.77	1.13	-16.57	0	7	0	469.559	7	CC1=NC2=C([C@@H](C1C(=O)OC)c3ccc(c(c3OC)OC)OC)C(=O)C[C@@H](C2)c4cccs4
ZINC00717214	3.96	1.17	-14.58	0	7	0	469.559	7	COc1ccc(c(c1OC)OC)[C@H]2C3C(=NC(=C)C2C(=O)OC)C[C@H](CC3=O)c4cccs4
ZINC00717214	3.96	2.44	-19.34	0	7	0	469.559	7	COc1ccc(c(c1OC)OC)[C@H]2[C@@H]3C(=NC(=C)C2C(=O)OC)C[C@H](CC3=O)c4cccs4
ZINC00717214	3.77	0.89	-15.75	0	7	0	469.559	7	CC1=C([C@H]([C@@H]2C(=N1)C[C@H](CC2=O)c3cccs3)c4ccc(c(c4OC)OC)OC)C(=O)OC
ZINC00717215	3.77	1.76	-11.42	0	7	0	469.559	7	CC1=NC2=C([C@H](C1C(=O)OC)c3ccc(c(c3OC)OC)OC)C(=O)C[C@@H](C2)c4cccs4
ZINC00717215	3.96	1.81	-11.62	0	7	0	469.559	7	COc1ccc(c(c1OC)OC)[C@@H]2C3C(=NC(=C)C2C(=O)OC)C[C@H](CC3=O)c4cccs4
ZINC00717215	3.96	2.89	-14.76	0	7	0	469.559	7	COc1ccc(c(c1OC)OC)[C@@H]2[C@H]3C(=NC(=C)C2C(=O)OC)C[C@H](CC3=O)c4cccs4
ZINC00717215	3.77	1.67	-13.04	0	7	0	469.559	7	CC1=C([C@@H]([C@H]2C(=N1)C[C@H](CC2=O)c3cccs3)c4ccc(c(c4OC)OC)OC)C(=O)OC
ZINC00717216	3.77	1.12	-16.32	0	7	0	469.559	7	CC1=NC2=C([C@@H](C1C(=O)OC)c3ccc(c(c3OC)OC)OC)C(=O)C[C@H](C2)c4cccs4
ZINC00717216	3.96	1.24	-14.45	0	7	0	469.559	7	COc1ccc(c(c1OC)OC)[C@H]2C3C(=NC(=C)C2C(=O)OC)C[C@@H](CC3=O)c4cccs4
ZINC00717216	3.96	2.41	-19.98	0	7	0	469.559	7	COc1ccc(c(c1OC)OC)[C@H]2[C@@H]3C(=NC(=C)C2C(=O)OC)C[C@@H](CC3=O)c4cccs4
ZINC00717216	3.77	0.97	-15.41	0	7	0	469.559	7	CC1=C([C@H]([C@H]2C(=N1)C[C@@H](CC2=O)c3cccs3)c4ccc(c(c4OC)OC)OC)C(=O)OC
ZINC08439593	4.1	1.76	-41.38	2	6	1	430.528	5	CC(C)C[C@H]1[C@H]2[C@@H](C(=O)N(C2=O)c3ccccc3)[C@@]4([NH2+]1)c5ccccc5N(C4=O)CC=C
ZINC00717217	3.77	1.26	-10.07	0	7	0	469.559	7	CC1=NC2=C([C@H](C1C(=O)OC)c3ccc(c(c3OC)OC)OC)C(=O)C[C@H](C2)c4cccs4
ZINC00717217	3.96	1.33	-11.12	0	7	0	469.559	7	COc1ccc(c(c1OC)OC)[C@@H]2C3C(=NC(=C)C2C(=O)OC)C[C@@H](CC3=O)c4cccs4
ZINC00717217	3.96	2.76	-16.48	0	7	0	469.559	7	COc1ccc(c(c1OC)OC)[C@@H]2[C@H]3C(=NC(=C)C2C(=O)OC)C[C@@H](CC3=O)c4cccs4
ZINC00717217	3.77	1.22	-13.46	0	7	0	469.559	7	CC1=C([C@@H]([C@H]2C(=N1)C[C@@H](CC2=O)c3cccs3)c4ccc(c(c4OC)OC)OC)C(=O)OC
ZINC08439592	2.52	-1.98	-73.38	4	8	2	718.946	13	Cc1cc2c(cc1C)[nH]c3c2c[n+](c4c3cccc4)CC(=O)NCCCCCCCCNC(=O)C[n+]5cc6c7cc(c(cc7[nH]c6c8c5cccc8)C)C
ZINC08439591	4.1	1.54	-44.85	2	6	1	430.528	5	CC(C)C[C@@H]1[C@H]2[C@@H](C(=O)N(C2=O)c3ccccc3)[C@@]4([NH2+]1)c5ccccc5N(C4=O)CC=C
ZINC08439590	4.34	2.23	-7.87	0	6	0	439.43	8	CC1=NC(=C([C@@H](C1C(=O)OC)c2ccccc2C(F)(F)F)C(=O)OC[C@H]3CCCO3)C
ZINC08439590	4.52	2.16	-7.41	0	6	0	439.43	8	CC1=NC(=C)C([C@H](C1C(=O)OC[C@H]2CCCO2)c3ccccc3C(F)(F)F)C(=O)OC
ZINC08439590	4.52	3.69	-15.55	0	6	0	439.43	8	CC1=NC(=C)C([C@H]([C@H]1C(=O)OC[C@H]2CCCO2)c3ccccc3C(F)(F)F)C(=O)OC
ZINC08439590	4.34	2.21	-7.94	0	6	0	439.43	8	CC1=NC(=C([C@H]([C@H]1C(=O)OC[C@H]2CCCO2)c3ccccc3C(F)(F)F)C(=O)OC)C
ZINC08439588	4.89	-0.77	-8.32	0	5	0	448.357	8	CCCOCCOC(=O)C1[C@@H](C2=C(CCCC2=O)N=C1C)c3cccc(c3)Br
ZINC08439588	4.89	-0.95	-12.08	0	5	0	448.357	8	CCCOCCOC(=O)C1=C(N=C2CCCC(=O)C2[C@H]1c3cccc(c3)Br)C
ZINC08439588	5.07	-1.87	-9.72	0	5	0	448.357	8	CCCOCCOC(=O)C1[C@@H](C2C(=NC1=C)CCCC2=O)c3cccc(c3)Br
ZINC08439587	4.34	2.27	-14.97	0	6	0	439.43	8	CC1=NC(=C([C@H](C1C(=O)OC)c2ccccc2C(F)(F)F)C(=O)OC[C@H]3CCCO3)C
ZINC08439587	4.52	2.33	-12.29	0	6	0	439.43	8	CC1=NC(=C)C([C@@H](C1C(=O)OC[C@H]2CCCO2)c3ccccc3C(F)(F)F)C(=O)OC
ZINC08439587	4.52	3.57	-15.7	0	6	0	439.43	8	CC1=NC(=C)C([C@@H]([C@@H]1C(=O)OC[C@H]2CCCO2)c3ccccc3C(F)(F)F)C(=O)OC
ZINC08439587	4.34	2.07	-12.36	0	6	0	439.43	8	CC1=NC(=C([C@@H]([C@@H]1C(=O)OC[C@H]2CCCO2)c3ccccc3C(F)(F)F)C(=O)OC)C
ZINC08439585	4.34	1.92	-7.59	0	6	0	439.43	8	CC1=NC(=C([C@@H](C1C(=O)OC)c2ccccc2C(F)(F)F)C(=O)OC[C@@H]3CCCO3)C
ZINC08439585	4.52	2.02	-7.34	0	6	0	439.43	8	CC1=NC(=C)C([C@H](C1C(=O)OC[C@@H]2CCCO2)c3ccccc3C(F)(F)F)C(=O)OC
ZINC08439585	4.52	3.38	-15.51	0	6	0	439.43	8	CC1=NC(=C)C([C@H]([C@H]1C(=O)OC[C@@H]2CCCO2)c3ccccc3C(F)(F)F)C(=O)OC
ZINC08439585	4.34	1.91	-7.48	0	6	0	439.43	8	CC1=NC(=C([C@H]([C@H]1C(=O)OC[C@@H]2CCCO2)c3ccccc3C(F)(F)F)C(=O)OC)C
ZINC02170763	7.62	1.59	-11.15	0	3	0	491.454	6	CCc1c(nc(s1)N(c2cccc(c2)Br)C(=O)Cc3ccccc3)c4ccc(cc4)C
ZINC08439584	4.34	2.1	-12.56	0	6	0	439.43	8	CC1=NC(=C([C@H](C1C(=O)OC)c2ccccc2C(F)(F)F)C(=O)OC[C@@H]3CCCO3)C
ZINC08439584	4.52	2.25	-11.49	0	6	0	439.43	8	CC1=NC(=C)C([C@@H](C1C(=O)OC[C@@H]2CCCO2)c3ccccc3C(F)(F)F)C(=O)OC
ZINC08439584	4.52	3.64	-25.54	0	6	0	439.43	8	CC1=NC(=C)C([C@@H]([C@@H]1C(=O)OC[C@@H]2CCCO2)c3ccccc3C(F)(F)F)C(=O)OC

Supplementary Table S2:
Detailed table of XP Docking using Schrodinger's Glide module

ZINC_ID	xLogP	ApolarDesolvation	PolarDesolvation	H Donors	H Acceptors	Charge	MolecularWeight	RotatableBonds	SMILES
ZINC08439584	4.34	2	-11.72	0	6	0	439.43	8	CC1=NC(=C([C@@H]([C@H]1C(=O)OC[C@@H]2CCCO2)c3ccccc3C(F)(F)F)C(=O)OC)C
ZINC08439583	4.89	-0.74	-9.5	0	5	0	448.357	8	CCCOCCOC(=O)C1[C@H](C2=C(CCCC2=O)N=C1C)c3cccc(c3)Br
ZINC08439583	4.89	-0.94	-12.74	0	5	0	448.357	8	CCCOCCOC(=O)C1=C(N=C2CCCC(=O)C2[C@@H]1c3cccc(c3)Br)C
ZINC08439583	5.07	-1.85	-10.54	0	5	0	448.357	8	CCCOCCOC(=O)C1[C@H](C2C(=NC1=C)CCCC2=O)c3cccc(c3)Br
ZINC02170761	7.71	0.86	-13.83	0	4	0	499.474	6	CCc1c(nc(s1)N(c2cccc(c2)Br)C(=O)C3CCCCC3)c4ccc(cc4)OC
ZINC08439582	4.53	0.39	-11.25	0	9	0	450.875	8	CC1=NC(=C([C@@H](C1C(=O)OC)c2ccc(c(c2)[N+](=O)[O-])Cl)C(=O)OC[C@H]3CCCO3)C
ZINC08439582	4.71	0.57	-11.09	0	9	0	450.875	8	CC1=NC(=C)C([C@H](C1C(=O)OC[C@H]2CCCO2)c3ccc(c(c3)[N+](=O)[O-])Cl)C(=O)OC
ZINC08439582	4.71	1.81	-18.84	0	9	0	450.875	8	CC1=NC(=C)C([C@H]([C@H]1C(=O)OC[C@H]2CCCO2)c3ccc(c(c3)[N+](=O)[O-])Cl)C(=O)OC
ZINC08439582	4.53	0.61	-12.23	0	9	0	450.875	8	CC1=NC(=C([C@H]([C@@H]1C(=O)OC[C@H]2CCCO2)c3ccc(c(c3)[N+](=O)[O-])Cl)C(=O)OC)C
ZINC02170759	8.31	1.88	-8.32	0	3	0	447.431	8	CCCCC(=O)N(c1ccc(c(c1)Cl)Cl)c2nc(c(s2)CCC)c3ccccc3
ZINC08439581	4.53	0.4	-12.17	0	9	0	450.875	8	CC1=NC(=C([C@H](C1C(=O)OC)c2ccc(c(c2)[N+](=O)[O-])Cl)C(=O)OC[C@H]3CCCO3)C
ZINC08439581	4.71	0.4	-10.39	0	9	0	450.875	8	CC1=NC(=C)C([C@@H](C1C(=O)OC[C@H]2CCCO2)c3ccc(c(c3)[N+](=O)[O-])Cl)C(=O)OC
ZINC08439581	4.71	1.7	-12.65	0	9	0	450.875	8	CC1=NC(=C)C([C@@H]([C@@H]1C(=O)OC[C@H]2CCCO2)c3ccc(c(c3)[N+](=O)[O-])Cl)C(=O)OC
ZINC08439581	4.53	0.31	-11.14	0	9	0	450.875	8	CC1=NC(=C([C@@H]([C@@H]1C(=O)OC[C@H]2CCCO2)c3ccc(c(c3)[N+](=O)[O-])Cl)C(=O)OC)C
ZINC02170755	7.5	1.13	-7.92	0	3	0	463.828	6	CCCC(=O)N(c1ccc(cc1)Br)c2nc(c(s2)CC)c3ccc(cc3)Cl
ZINC08439580	4.53	0.43	-10.04	0	9	0	450.875	8	CC1=NC(=C([C@@H](C1C(=O)OC)c2ccc(c(c2)[N+](=O)[O-])Cl)C(=O)OC[C@@H]3CCCO3)C
ZINC08439580	4.71	0.42	-9.65	0	9	0	450.875	8	CC1=NC(=C)C([C@H](C1C(=O)OC[C@@H]2CCCO2)c3ccc(c(c3)[N+](=O)[O-])Cl)C(=O)OC
ZINC08439580	4.71	1.91	-15.85	0	9	0	450.875	8	CC1=NC(=C)C([C@H]([C@H]1C(=O)OC[C@@H]2CCCO2)c3ccc(c(c3)[N+](=O)[O-])Cl)C(=O)OC
ZINC08439580	4.53	0.3	-10.25	0	9	0	450.875	8	CC1=NC(=C([C@H]([C@H]1C(=O)OC[C@@H]2CCCO2)c3ccc(c(c3)[N+](=O)[O-])Cl)C(=O)OC)C
ZINC08439579	5.44	2.92	-12.51	0	6	0	473.569	8	CC1=NC2=C([C@@H](C1C(=O)OCCc3ccccc3)c4ccc(cc4)OC(=O)C)C(=O)CC(C2)(C)C
ZINC08439579	5.62	3.35	-13.1	0	6	0	473.569	8	CC(=O)Oc1ccc(cc1)[C@H]2C3C(=NC(=C)C2C(=O)OCCc4ccccc4)CC(CC3=O)(C)C
ZINC08439579	5.62	4.33	-15.75	0	6	0	473.569	8	CC(=O)Oc1ccc(cc1)[C@H]2[C@@H]3C(=NC(=C)C2C(=O)OCCc4ccccc4)CC(CC3=O)(C)C
ZINC08439579	5.44	3.19	-15.07	0	6	0	473.569	8	CC1=C([C@H]([C@@H]2C(=N1)CC(CC2=O)(C)C)c3ccc(cc3)OC(=O)C)C(=O)OCCc4ccccc4
ZINC02170752	7.25	1.26	-10.33	0	4	0	507.453	7	CCc1c(nc(s1)N(c2ccc(cc2)Br)C(=O)Cc3ccccc3)c4ccc(cc4)OC
ZINC08439578	4.53	0.32	-9.37	0	9	0	450.875	8	CC1=NC(=C([C@H](C1C(=O)OC)c2ccc(c(c2)[N+](=O)[O-])Cl)C(=O)OC[C@@H]3CCCO3)C
ZINC08439578	4.71	0.36	-8.95	0	9	0	450.875	8	CC1=NC(=C)C([C@@H](C1C(=O)OC[C@@H]2CCCO2)c3ccc(c(c3)[N+](=O)[O-])Cl)C(=O)OC
ZINC08439578	4.71	1.67	-9.59	0	9	0	450.875	8	CC1=NC(=C)C([C@@H]([C@@H]1C(=O)OC[C@@H]2CCCO2)c3ccc(c(c3)[N+](=O)[O-])Cl)C(=O)OC
ZINC08439578	4.53	0.41	-9.46	0	9	0	450.875	8	CC1=NC(=C([C@@H]([C@H]1C(=O)OC[C@@H]2CCCO2)c3ccc(c(c3)[N+](=O)[O-])Cl)C(=O)OC)C
ZINC02170749	8.33	0.69	-12.22	0	3	0	503.893	5	CCc1c(nc(s1)N(c2cccc(c2)Br)C(=O)C3CCCCC3)c4ccc(cc4)Cl
ZINC08439577	5.44	3	-14.28	0	6	0	473.569	8	CC1=NC2=C([C@H](C1C(=O)OCCc3ccccc3)c4ccc(cc4)OC(=O)C)C(=O)CC(C2)(C)C
ZINC08439577	5.62	3.06	-14.51	0	6	0	473.569	8	CC(=O)Oc1ccc(cc1)[C@@H]2C3C(=NC(=C)C2C(=O)OCCc4ccccc4)CC(CC3=O)(C)C
ZINC08439577	5.62	4.28	-15.08	0	6	0	473.569	8	CC(=O)Oc1ccc(cc1)[C@@H]2[C@H]3C(=NC(=C)C2C(=O)OCCc4ccccc4)CC(CC3=O)(C)C
ZINC08439577	5.44	2.79	-16.59	0	6	0	473.569	8	CC1=C([C@@H]([C@H]2C(=N1)CC(CC2=O)(C)C)c3ccc(cc3)OC(=O)C)C(=O)OCCc4ccccc4
ZINC08439576	4.54	-2.56	-45.34	1	12	1	620.104	11	CC1=NC(=C([C@@H](C1C(=O)OC)c2ccccc2[N+](=O)[O-])C(=O)OCC[NH+]3CCN(CC3)S(=O)(=O)c4ccc(cc4)Cl)C
ZINC08439576	4.72	-2.54	-48.69	1	12	1	620.104	11	CC1=NC(=C)C([C@H](C1C(=O)OCC[NH+]2CCN(CC2)S(=O)(=O)c3ccc(cc3)Cl)c4ccccc4[N+](=O)[O-])C(=O)OC
ZINC08439576	4.72	-1.18	-45.66	1	12	1	620.104	11	CC1=NC(=C)C([C@H]([C@H]1C(=O)OCC[NH+]2CCN(CC2)S(=O)(=O)c3ccc(cc3)Cl)c4ccccc4[N+](=O)[O-])C(=O)OC
ZINC08439576	4.54	-2.5	-50.19	1	12	1	620.104	11	CC1=NC(=C([C@H]([C@@H]1C(=O)OCC[NH+]2CCN(CC2)S(=O)(=O)c3ccc(cc3)Cl)c4ccccc4[N+](=O)[O-])C(=O)OC)C
ZINC02170744	7.72	2.06	-10.67	0	4	0	497.447	7	CCc1c(nc(s1)N(c2ccc(cc2Cl)Cl)C(=O)Cc3ccccc3)c4ccc(cc4)OC
ZINC08439575	4.54	-2.65	-57.72	1	12	1	620.104	11	CC1=NC(=C([C@H](C1C(=O)OC)c2ccccc2[N+](=O)[O-])C(=O)OCC[NH+]3CCN(CC3)S(=O)(=O)c4ccc(cc4)Cl)C
ZINC08439575	4.72	-2.41	-61.63	1	12	1	620.104	11	CC1=NC(=C)C([C@@H](C1C(=O)OCC[NH+]2CCN(CC2)S(=O)(=O)c3ccc(cc3)Cl)c4ccccc4[N+](=O)[O-])C(=O)OC
ZINC08439575	4.72	-1.27	-58.61	1	12	1	620.104	11	CC1=NC(=C)C([C@@H]([C@@H]1C(=O)OCC[NH+]2CCN(CC2)S(=O)(=O)c3ccc(cc3)Cl)c4ccccc4[N+](=O)[O-])C(=O)OC
ZINC08439575	4.54	-2.57	-65.41	1	12	1	620.104	11	CC1=NC(=C([C@@H]([C@@H]1C(=O)OCC[NH+]2CCN(CC2)S(=O)(=O)c3ccc(cc3)Cl)c4ccccc4[N+](=O)[O-])C(=O)OC)C
ZINC08439574	5.61	1.26	-11.25	0	6	0	468.638	11	CCCOCCOC(=O)C1[C@@H](C2=C(CC(CC2=O)(C)C)N=C1C)c3ccc(cc3)N(CC)CC
ZINC00643372	6.42	2.06	-14.93	0	5	0	458.583	8	Cc1c(nc(s1)N(c2cccc(c2)OC)C(=O)CCc3ccccc3)c4ccc(cc4)OC
ZINC08439573	4.99	-2.45	-56.13	1	12	1	620.104	11	CC1=NC(=C([C@@H](C1C(=O)OC)c2cccc(c2)[N+](=O)[O-])C(=O)OCC[NH+]3CCN(CC3)S(=O)(=O)c4ccccc4Cl)C
ZINC08439573	5.17	-2.32	-59.6	1	12	1	620.104	11	CC1=NC(=C)C([C@H](C1C(=O)OCC[NH+]2CCN(CC2)S(=O)(=O)c3ccccc3Cl)c4cccc(c4)[N+](=O)[O-])C(=O)OC
ZINC08439573	5.17	-1.1	-55.06	1	12	1	620.104	11	CC1=NC(=C)C([C@H]([C@H]1C(=O)OCC[NH+]2CCN(CC2)S(=O)(=O)c3ccccc3Cl)c4cccc(c4)[N+](=O)[O-])C(=O)OC
ZINC08439573	4.99	-2.23	-62.7	1	12	1	620.104	11	CC1=NC(=C([C@H]([C@@H]1C(=O)OCC[NH+]2CCN(CC2)S(=O)(=O)c3ccccc3Cl)c4cccc(c4)[N+](=O)[O-])C(=O)OC)C
ZINC02170739	7.69	2.19	-12.88	0	3	0	442.987	5	CCc1c(nc(s1)N(c2cccc(c2)F)C(=O)C3CCCCC3)c4ccc(cc4)Cl
ZINC08439572	5.61	1.13	-9.04	0	6	0	468.638	11	CCCOCCOC(=O)C1[C@H](C2=C(CC(CC2=O)(C)C)N=C1C)c3ccc(cc3)N(CC)CC
ZINC08439571	4.99	-2.44	-51.59	1	12	1	620.104	11	CC1=NC(=C([C@H](C1C(=O)OC)c2cccc(c2)[N+](=O)[O-])C(=O)OCC[NH+]3CCN(CC3)S(=O)(=O)c4ccccc4Cl)C
ZINC08439571	5.17	-2.27	-56.36	1	12	1	620.104	11	CC1=NC(=C)C([C@@H](C1C(=O)OCC[NH+]2CCN(CC2)S(=O)(=O)c3ccccc3Cl)c4cccc(c4)[N+](=O)[O-])C(=O)OC
ZINC08439571	5.17	-1.15	-52.11	1	12	1	620.104	11	CC1=NC(=C)C([C@@H]([C@@H]1C(=O)OCC[NH+]2CCN(CC2)S(=O)(=O)c3ccccc3Cl)c4cccc(c4)[N+](=O)[O-])C(=O)OC
ZINC08439571	4.99	-2.5	-56.56	1	12	1	620.104	11	CC1=NC(=C([C@@H]([C@@H]1C(=O)OCC[NH+]2CCN(CC2)S(=O)(=O)c3ccccc3Cl)c4cccc(c4)[N+](=O)[O-])C(=O)OC)C
ZINC00643366	6.34	2.23	-16.68	0	4	0	428.557	7	Cc1c(nc(s1)N(c2ccccc2OC)C(=O)CCc3ccccc3)c4ccccc4
ZINC08439570	2.61	9.12	-13.37	1	11	0	480.477	10	CC1=NC(=C([C@@H](C1C(=O)OC)c2ccccc2[N+](=O)[O-])C(=O)OCCNC(=O)c3cccnc3)C
ZINC08439570	2.79	8.92	-13.36	1	11	0	480.477	10	CC1=NC(=C)C([C@H](C1C(=O)OCCNC(=O)c2cccnc2)c3ccccc3[N+](=O)[O-])C(=O)OC
ZINC08439570	2.79	9.85	-13.99	1	11	0	480.477	10	CC1=NC(=C)C([C@H]([C@H]1C(=O)OCCNC(=O)c2cccnc2)c3ccccc3[N+](=O)[O-])C(=O)OC
ZINC08439570	2.61	9.11	-14.85	1	11	0	480.477	10	CC1=NC(=C([C@H]([C@@H]1C(=O)OCCNC(=O)c2cccnc2)c3ccccc3[N+](=O)[O-])C(=O)OC)C
ZINC02170736	7.14	2.26	-10.54	0	5	0	438.593	10	CCCCC(=O)N(c1ccc(cc1)OC)c2nc(c(s2)CCC)c3ccc(cc3)OC
ZINC08439569	2.61	-0.56	-16.84	1	11	0	480.477	10	CC1=NC(=C([C@H](C1C(=O)OC)c2ccccc2[N+](=O)[O-])C(=O)OCCNC(=O)c3cccnc3)C

Supplementary Table S2:
Detailed table of XP Docking using Schrodinger's Glide module

ZINC_ID	xLogP	ApolarDesolvation	PolarDesolvation	H Donors	H Acceptors	Charge	MolecularWeight	RotatableBonds	SMILES
ZINC08439569	2.79	-0.28	-17.29	1	11	0	480.477	10	CC1=NC(=C)C([C@@H](C1C(=O)OCCNC(=O)c2cccnc2)c3ccccc3[N+](=O)[O-])C(=O)OC
ZINC08439569	2.79	0.8	-18.5	1	11	0	480.477	10	CC1=NC(=C)C([C@@H]([C@@H]1C(=O)OCCNC(=O)c2cccnc2)c3ccccc3[N+](=O)[O-])C(=O)OC
ZINC08439569	2.61	-0.4	-18.96	1	11	0	480.477	10	CC1=NC(=C([C@@H]([C@@H]1C(=O)OCCNC(=O)c2cccnc2)c3ccccc3[N+](=O)[O-])C(=O)OC)C
ZINC00717199	4.89	-1.17	-12.42	1	4	0	468.372	6	c1ccc(cc1)CCN2[C@@H](C(=C(C2=O)O)C(=O)c3cccs3)c4ccc(cc4)Br
ZINC00717199	4.31	-1.3	-19.64	0	4	0	468.372	6	c1ccc(cc1)CCN2[C@@H]([C@@H](C(=O)C2=O)C(=O)c3cccs3)c4ccc(cc4)Br
ZINC02170733	6.04	0.95	-13.09	1	6	0	479.005	8	CCOc1ccc(cc1)NC(=O)[C@H](C)Sc2nnc(n2c3ccccc3)c4ccc(cc4)Cl
ZINC08439568	4.23	1.14	-21.65	1	13	0	607.616	14	CC1=NC(=C([C@@H](C1C(=O)OCCOC(=O)c2ccc(cc2)NC(=O)C)c3ccccc3[N+](=O)[O-])C(=O)OC[C@H]4CCCO4)C
ZINC08439568	4.41	1.12	-20.35	1	13	0	607.616	14	CC1=NC(=C)C([C@H](C1C(=O)OC[C@H]2CCCO2)c3ccccc3[N+](=O)[O-])C(=O)OCCOC(=O)c4ccc(cc4)NC(=O)C
ZINC08439568	4.41	2.53	-23.99	1	13	0	607.616	14	CC1=NC(=C)C([C@H]([C@H]1C(=O)OC[C@H]2CCCO2)c3ccccc3[N+](=O)[O-])C(=O)OCCOC(=O)c4ccc(cc4)NC(=O)C
ZINC08439568	4.23	0.98	-19.89	1	13	0	607.616	14	CC1=NC(=C([C@H]([C@H]1C(=O)OC[C@H]2CCCO2)c3ccccc3[N+](=O)[O-])C(=O)OCCOC(=O)c4ccc(cc4)NC(=O)C)C
ZINC02170731	6.04	0.94	-12.87	1	6	0	479.005	8	CCOc1ccc(cc1)NC(=O)[C@@H](C)Sc2nnc(n2c3ccccc3)c4ccc(cc4)Cl
ZINC00717196	4.76	-0.25	-12.69	1	4	0	423.921	6	c1ccc(cc1)CCN2[C@H](C(=C(C2=O)O)C(=O)c3cccs3)c4ccc(cc4)Cl
ZINC00717196	4.17	-0.42	-18.37	0	4	0	423.921	6	c1ccc(cc1)CCN2[C@H]([C@@H](C(=O)C2=O)C(=O)c3cccs3)c4ccc(cc4)Cl
ZINC00643364	6.34	1.74	-12.27	0	5	0	436.577	6	Cc1c(nc(s1)N(c2ccc(cc2OC)OC)C(=O)C3CCCCC3)c4ccccc4
ZINC08439566	4.23	0.65	-24.67	1	13	0	607.616	14	CC1=NC(=C([C@@H](C1C(=O)OCCOC(=O)c2ccc(cc2)NC(=O)C)c3ccccc3[N+](=O)[O-])C(=O)OC[C@@H]4CCCO4)C
ZINC08439566	4.41	0.58	-22.4	1	13	0	607.616	14	CC1=NC(=C)C([C@H](C1C(=O)OC[C@@H]2CCCO2)c3ccccc3[N+](=O)[O-])C(=O)OCCOC(=O)c4ccc(cc4)NC(=O)C
ZINC08439566	4.41	1.93	-28.84	1	13	0	607.616	14	CC1=NC(=C)C([C@H]([C@H]1C(=O)OC[C@@H]2CCCO2)c3ccccc3[N+](=O)[O-])C(=O)OCCOC(=O)c4ccc(cc4)NC(=O)C
ZINC08439566	4.23	0.47	-23.37	1	13	0	607.616	14	CC1=NC(=C([C@H]([C@H]1C(=O)OC[C@@H]2CCCO2)c3ccccc3[N+](=O)[O-])C(=O)OCCOC(=O)c4ccc(cc4)NC(=O)C)C
ZINC02170729	7.06	3	-13.59	0	4	0	460.574	8	CCCc1c(nc(s1)N(c2ccccc2F)C(=O)Cc3ccccc3)c4ccc(cc4)OC
ZINC00717197	4.76	-0.56	-12.51	1	4	0	423.921	6	c1ccc(cc1)CCN2[C@@H](C(=C(C2=O)O)C(=O)c3cccs3)c4ccc(cc4)Cl
ZINC00717197	4.17	-0.69	-19.73	0	4	0	423.921	6	c1ccc(cc1)CCN2[C@@H]([C@@H](C(=O)C2=O)C(=O)c3cccs3)c4ccc(cc4)Cl
ZINC08439565	4.23	0.46	-25.99	1	13	0	607.616	14	CC1=NC(=C([C@H](C1C(=O)OCCOC(=O)c2ccc(cc2)NC(=O)C)c3ccccc3[N+](=O)[O-])C(=O)OC[C@@H]4CCCO4)C
ZINC08439565	4.41	0.42	-24.42	1	13	0	607.616	14	CC1=NC(=C)C([C@@H](C1C(=O)OC[C@@H]2CCCO2)c3ccccc3[N+](=O)[O-])C(=O)OCCOC(=O)c4ccc(cc4)NC(=O)C
ZINC08439565	4.41	1.7	-27.47	1	13	0	607.616	14	CC1=NC(=C)C([C@@H]([C@@H]1C(=O)OC[C@@H]2CCCO2)c3ccccc3[N+](=O)[O-])C(=O)OCCOC(=O)c4ccc(cc4)NC(=O)C
ZINC08439565	4.23	0.29	-23.71	1	13	0	607.616	14	CC1=NC(=C([C@@H]([C@@H]1C(=O)OC[C@@H]2CCCO2)c3ccccc3[N+](=O)[O-])C(=O)OCCOC(=O)c4ccc(cc4)NC(=O)C)C
ZINC02170722	6.97	16.32	-12.11	0	3	0	430.548	6	CCc1c(nc(s1)N(c2cccc(c2)F)C(=O)Cc3ccccc3)c4ccc(cc4)C
ZINC08439564	7.18	-0.5	-23.59	2	11	0	597.624	13	CC1=NC(=C([C@@H](C1C(=O)OCCNC(=O)c2ccccc2O)c3cccc(c3)[N+](=O)[O-])C(=O)OC/C=C/c4ccccc4)C
ZINC08439564	7.37	-0.31	-20.9	2	11	0	597.624	13	CC1=NC(=C)C([C@H](C1C(=O)OC/C=C/c2ccccc2)c3cccc(c3)[N+](=O)[O-])C(=O)OCCNC(=O)c4ccccc4O
ZINC08439564	7.37	0.85	-24.78	2	11	0	597.624	13	CC1=NC(=C)C([C@H]([C@H]1C(=O)OC/C=C/c2ccccc2)c3cccc(c3)[N+](=O)[O-])C(=O)OCCNC(=O)c4ccccc4O
ZINC08439564	7.18	-0.39	-21.77	2	11	0	597.624	13	CC1=NC(=C([C@H]([C@@H]1C(=O)OC/C=C/c2ccccc2)c3cccc(c3)[N+](=O)[O-])C(=O)OCCNC(=O)c4ccccc4O)C
ZINC00643360	3.82	1.06	-18.19	1	8	0	462.579	8	CCn1c(c(c(n1)C)NC(=O)CSc2nnc(n2c3ccccc3)c4ccc(cc4)OC)C
ZINC08439562	5.31	2.23	-7.03	0	4	0	447.478	5	CC1=NC2=C([C@@H](C1C(=O)OC)c3ccc(cc3)C(F)(F)F)C(=O)C[C@@H](C2)c4cccs4
ZINC08439562	5.5	2.34	-8.03	0	4	0	447.478	5	COC(=O)C1[C@H](C2C(=NC1=C)C[C@H](CC2=O)c3cccs3)c4ccc(cc4)C(F)(F)F
ZINC08439562	5.5	3.65	-9.97	0	4	0	447.478	5	COC(=O)C1[C@H]([C@@H]2C(=NC1=C)C[C@H](CC2=O)c3cccs3)c4ccc(cc4)C(F)(F)F
ZINC08439562	5.31	2.17	-9.49	0	4	0	447.478	5	CC1=C([C@H]([C@@H]2C(=N1)C[C@H](CC2=O)c3cccs3)c4ccc(cc4)C(F)(F)F)C(=O)OC
ZINC08439561	4.37	-0.49	-56.57	3	12	1	553.592	13	CC1=NC(=C([C@@H](C1C(=O)OCCNC(=O)c2ccccc2O)c3ccccc3[N+](=O)[O-])C(=O)OCC[NH+](C)C)C
ZINC08439561	4.55	-0.25	-59.67	3	12	1	553.592	13	CC1=NC(=C)C([C@H](C1C(=O)OCC[NH+](C)C)c2ccccc2[N+](=O)[O-])C(=O)OCCNC(=O)c3ccccc3O
ZINC08439561	4.55	0.87	-58.47	3	12	1	553.592	13	CC1=NC(=C)C([C@H]([C@H]1C(=O)OCC[NH+](C)C)c2ccccc2[N+](=O)[O-])C(=O)OCCNC(=O)c3ccccc3O
ZINC08439561	4.37	-0.33	-62.19	3	12	1	553.592	13	CC1=NC(=C([C@H]([C@@H]1C(=O)OCC[NH+](C)C)c2ccccc2[N+](=O)[O-])C(=O)OCCNC(=O)c3ccccc3O)C
ZINC02170719	5.7	1.13	-16.1	1	6	0	463.632	7	C[C@H](C(=O)Nc1nccs1)Sc2nnc(n2c3ccccc3)c4ccc(cc4)C(C)(C)C
ZINC08439560	4.37	-0.16	-40.91	3	12	1	553.592	13	CC1=NC(=C([C@H](C1C(=O)OCCNC(=O)c2ccccc2O)c3ccccc3[N+](=O)[O-])C(=O)OCC[NH+](C)C)C
ZINC08439560	4.55	-0.13	-43.24	3	12	1	553.592	13	CC1=NC(=C)C([C@@H](C1C(=O)OCC[NH+](C)C)c2ccccc2[N+](=O)[O-])C(=O)OCCNC(=O)c3ccccc3O
ZINC08439560	4.55	1.26	-45.26	3	12	1	553.592	13	CC1=NC(=C)C([C@@H]([C@@H]1C(=O)OCC[NH+](C)C)c2ccccc2[N+](=O)[O-])C(=O)OCCNC(=O)c3ccccc3O
ZINC08439559	3.92	-3.69	-24.41	3	14	0	629.626	14	CC1=NC(=C([C@@H](C1C(=O)OCCNC(=O)c2cccnc2)c3ccccc3[N+](=O)[O-])C(=O)OCCNC(=O)c4ccccc4O)C
ZINC08439559	4.1	-3.7	-23.86	3	14	0	629.626	14	CC1=NC(=C)C([C@H](C1C(=O)OCCNC(=O)c2ccccc2O)c3ccccc3[N+](=O)[O-])C(=O)OCCNC(=O)c4cccnc4
ZINC08439559	4.1	-2.17	-31.71	3	14	0	629.626	14	CC1=NC(=C)C([C@H]([C@H]1C(=O)OCCNC(=O)c2ccccc2O)c3ccccc3[N+](=O)[O-])C(=O)OCCNC(=O)c4cccnc4
ZINC08439559	3.92	-3.7	-24.93	3	14	0	629.626	14	CC1=NC(=C([C@H]([C@H]1C(=O)OCCNC(=O)c2ccccc2O)c3ccccc3[N+](=O)[O-])C(=O)OCCNC(=O)c4cccnc4)C
ZINC02170717	5.7	1.14	-16.04	1	6	0	463.632	7	C[C@@H](C(=O)Nc1nccs1)Sc2nnc(n2c3ccccc3)c4ccc(cc4)C(C)(C)C
ZINC08439558	5.31	2.11	-7.56	0	4	0	447.478	5	CC1=NC2=C([C@H](C1C(=O)OC)c3ccc(cc3)C(F)(F)F)C(=O)C[C@@H](C2)c4cccs4
ZINC08439558	5.5	2.2	-8.03	0	4	0	447.478	5	COC(=O)C1[C@@H](C2C(=NC1=C)C[C@H](CC2=O)c3cccs3)c4ccc(cc4)C(F)(F)F
ZINC08439558	5.5	3.26	-7.98	0	4	0	447.478	5	COC(=O)C1[C@@H]([C@H]2C(=NC1=C)C[C@H](CC2=O)c3cccs3)c4ccc(cc4)C(F)(F)F
ZINC08439558	5.31	2.25	-9.4	0	4	0	447.478	5	CC1=C([C@@H]([C@@H]2C(=N1)C[C@H](CC2=O)c3cccs3)c4ccc(cc4)C(F)(F)F)C(=O)OC
ZINC02170714	7.98	1.52	-12.23	0	4	0	469.05	7	CCc1c(nc(s1)N(c2ccc(cc2)Cl)C(=O)C3CCCCC3)c4ccc(cc4)OCC
ZINC00643358	5.07	10.6	-18.75	2	8	0	514.607	11	COc1ccc(cc1)c2nnc(n2CC=C)SCC(=O)NCc3cc(ccc3O)C(=O)c4ccccc4
ZINC08439557	3.92	-3.56	-26.15	3	14	0	629.626	14	CC1=NC(=C([C@H](C1C(=O)OCCNC(=O)c2cccnc2)c3ccccc3[N+](=O)[O-])C(=O)OCCNC(=O)c4ccccc4O)C
ZINC08439557	4.1	-3.43	-25.31	3	14	0	629.626	14	CC1=NC(=C)C([C@@H](C1C(=O)OCCNC(=O)c2ccccc2O)c3ccccc3[N+](=O)[O-])C(=O)OCCNC(=O)c4cccnc4
ZINC08439557	4.1	-2.33	-26.7	3	14	0	629.626	14	CC1=NC(=C)C([C@@H]([C@@H]1C(=O)OCCNC(=O)c2ccccc2O)c3ccccc3[N+](=O)[O-])C(=O)OCCNC(=O)c4cccnc4
ZINC08439557	3.92	-3.57	-26.38	3	14	0	629.626	14	CC1=NC(=C([C@@H]([C@H]1C(=O)OCCNC(=O)c2ccccc2O)c3ccccc3[N+](=O)[O-])C(=O)OCCNC(=O)c4cccnc4)C
ZINC08439556	4.01	0.56	-44.18	2	6	1	436.919	4	CC[C@H]1[C@H]2[C@@H](C(=O)N(C2=O)c3ccc(cc3)Cl)[C@@]4([NH2+]1)c5ccccc5N(C4=O)CC=C
ZINC08439555	4.71	-1.9	-16.82	2	12	0	565.579	12	CC1=NC(=C([C@@H](C1C(=O)OCCNC(=O)c2ccccc2O)c3ccccc3[N+](=O)[O-])C(=O)OC[C@H]4CCCO4)C
ZINC08439555	4.89	-1.89	-16.05	2	12	0	565.579	12	CC1=NC(=C)C([C@H](C1C(=O)OC[C@H]2CCCO2)c3ccccc3[N+](=O)[O-])C(=O)OCCNC(=O)c4ccccc4O

Supplementary Table S2:
Detailed table of XP Docking using Schrodinger's Glide module

ZINC_ID	xLogP	ApolarDesolvation	PolarDesolvation	H Donors	H Acceptors	Charge	MolecularWeight	RotatableBonds	SMILES
ZINC08439555	4.89	-0.48	-18.95	2	12	0	565.579	12	CC1=NC(=C)C([C@H]([C@H]1C(=O)OC[C@H]2CCCO2)c3ccccc3[N+](=O)[O-])C(=O)OCCNC(=O)c4ccccc4O
ZINC08439555	4.71	-2	-16.35	2	12	0	565.579	12	CC1=NC(=C([C@H]([C@H]1C(=O)OC[C@H]2CCCO2)c3ccccc3[N+](=O)[O-])C(=O)OCCNC(=O)c4ccccc4O)C
ZINC08439554	4.01	0.5	-43.24	2	6	1	436.919	4	CC[C@@H]1[C@H]2[C@@H](C(=O)N(C2=O)c3ccc(cc3)Cl)[C@@]4([NH2+]1)c5ccccc5N(C4=O)CC=C
ZINC08439553	5.31	2.21	-7.21	0	4	0	447.478	5	CC1=NC2=C([C@@H](C1C(=O)OC)c3ccc(cc3)C(F)(F)F)C(=O)C[C@H](C2)c4cccs4
ZINC08439553	5.5	2.35	-7.95	0	4	0	447.478	5	COC(=O)C1[C@H](C2C(=NC1=C)C[C@@H](CC2=O)c3cccs3)c4ccc(cc4)C(F)(F)F
ZINC08439553	5.5	3.6	-9.1	0	4	0	447.478	5	COC(=O)C1[C@H]([C@@H]2C(=NC1=C)C[C@@H](CC2=O)c3cccs3)c4ccc(cc4)C(F)(F)F
ZINC08439553	5.31	2.22	-9.81	0	4	0	447.478	5	CC1=C([C@H]([C@@H]2C(=N1)C[C@@H](CC2=O)c3cccs3)c4ccc(cc4)C(F)(F)F)C(=O)OC
ZINC08439552	4.71	-2.57	-22.93	2	12	0	565.579	12	CC1=NC(=C([C@H](C1C(=O)OCCNC(=O)c2ccccc2O)c3ccccc3[N+](=O)[O-])C(=O)OC[C@H]4CCCO4)C
ZINC08439552	4.89	-2.45	-19.8	2	12	0	565.579	12	CC1=NC(=C)C([C@@H](C1C(=O)OC[C@H]2CCCO2)c3ccccc3[N+](=O)[O-])C(=O)OCCNC(=O)c4ccccc4O
ZINC08439552	4.89	-1.04	-38.28	2	12	0	565.579	12	CC1=NC(=C)C([C@@H]([C@@H]1C(=O)OC[C@H]2CCCO2)c3ccccc3[N+](=O)[O-])C(=O)OCCNC(=O)c4ccccc4O
ZINC08439552	4.71	-2.54	-20.9	2	12	0	565.579	12	CC1=NC(=C([C@@H]([C@H]1C(=O)OC[C@H]2CCCO2)c3ccccc3[N+](=O)[O-])C(=O)OCCNC(=O)c4ccccc4O)C
ZINC08439550	4.71	-2.27	-22.79	2	12	0	565.579	12	CC1=NC(=C([C@@H](C1C(=O)OCCNC(=O)c2ccccc2O)c3ccccc3[N+](=O)[O-])C(=O)OC[C@@H]4CCCO4)C
ZINC08439550	4.89	-2.33	-19.79	2	12	0	565.579	12	CC1=NC(=C)C([C@H](C1C(=O)OC[C@@H]2CCCO2)c3ccccc3[N+](=O)[O-])C(=O)OCCNC(=O)c4ccccc4O
ZINC08439550	4.89	-0.98	-27.43	2	12	0	565.579	12	CC1=NC(=C)C([C@H]([C@H]1C(=O)OC[C@@H]2CCCO2)c3ccccc3[N+](=O)[O-])C(=O)OCCNC(=O)c4ccccc4O
ZINC08439550	4.71	-2.44	-20.69	2	12	0	565.579	12	CC1=NC(=C([C@H]([C@H]1C(=O)OC[C@@H]2CCCO2)c3ccccc3[N+](=O)[O-])C(=O)OCCNC(=O)c4ccccc4O)C
ZINC08439548	4.71	-2.33	-23.61	2	12	0	565.579	12	CC1=NC(=C([C@H](C1C(=O)OCCNC(=O)c2ccccc2O)c3ccccc3[N+](=O)[O-])C(=O)OC[C@@H]4CCCO4)C
ZINC08439548	4.89	-2.2	-21.15	2	12	0	565.579	12	CC1=NC(=C)C([C@@H](C1C(=O)OC[C@@H]2CCCO2)c3ccccc3[N+](=O)[O-])C(=O)OCCNC(=O)c4ccccc4O
ZINC08439548	4.89	-0.82	-38.44	2	12	0	565.579	12	CC1=NC(=C)C([C@@H]([C@@H]1C(=O)OC[C@@H]2CCCO2)c3ccccc3[N+](=O)[O-])C(=O)OCCNC(=O)c4ccccc4O
ZINC08439548	4.71	-2.29	-22.63	2	12	0	565.579	12	CC1=NC(=C([C@@H]([C@H]1C(=O)OC[C@@H]2CCCO2)c3ccccc3[N+](=O)[O-])C(=O)OCCNC(=O)c4ccccc4O)C
ZINC08439546	5.31	2.21	-7.06	0	4	0	447.478	5	CC1=NC2=C([C@H](C1C(=O)OC)c3ccc(cc3)C(F)(F)F)C(=O)C[C@H](C2)c4cccs4
ZINC08439546	5.5	2.28	-7.79	0	4	0	447.478	5	COC(=O)C1[C@@H](C2C(=NC1=C)C[C@@H](CC2=O)c3cccs3)c4ccc(cc4)C(F)(F)F
ZINC08439546	5.5	3.59	-8.6	0	4	0	447.478	5	COC(=O)C1[C@@H]([C@H]2C(=NC1=C)C[C@@H](CC2=O)c3cccs3)c4ccc(cc4)C(F)(F)F
ZINC08439546	5.31	2.15	-9.78	0	4	0	447.478	5	CC1=C([C@@H]([C@H]2C(=N1)C[C@@H](CC2=O)c3cccs3)c4ccc(cc4)C(F)(F)F)C(=O)OC
ZINC08439544	6.68	1.26	-20.03	1	11	0	611.651	14	CC1=NC(=C([C@@H](C1C(=O)OCCOc2ccc(cc2)NC(=O)C)c3cccc(c3)[N+](=O)[O-])C(=O)OC/C=C/c4ccccc4)C
ZINC08439544	6.86	1.44	-19.63	1	11	0	611.651	14	CC1=NC(=C)C([C@H](C1C(=O)OC/C=C/c2ccccc2)c3cccc(c3)[N+](=O)[O-])C(=O)OCCOc4ccc(cc4)NC(=O)C
ZINC08439544	6.86	2.61	-21.64	1	11	0	611.651	14	CC1=NC(=C)C([C@H]([C@H]1C(=O)OC/C=C/c2ccccc2)c3cccc(c3)[N+](=O)[O-])C(=O)OCCOc4ccc(cc4)NC(=O)C
ZINC08439544	6.68	1.32	-20.18	1	11	0	611.651	14	CC1=NC(=C([C@H]([C@H]1C(=O)OC/C=C/c2ccccc2)c3cccc(c3)[N+](=O)[O-])C(=O)OCCOc4ccc(cc4)NC(=O)C)C
ZINC00350684	3.07	10.89	-43.85	2	4	1	342.438	6	CC[NH+](CC)CCN1[C@@H](Nc2ccccc2C1=O)c3cccc(c3)F
ZINC08439543	6.68	1.25	-20.94	1	11	0	611.651	14	CC1=NC(=C([C@H](C1C(=O)OCCOc2ccc(cc2)NC(=O)C)c3cccc(c3)[N+](=O)[O-])C(=O)OC/C=C/c4ccccc4)C
ZINC08439543	6.86	1.29	-20.14	1	11	0	611.651	14	CC1=NC(=C)C([C@@H](C1C(=O)OC/C=C/c2ccccc2)c3cccc(c3)[N+](=O)[O-])C(=O)OCCOc4ccc(cc4)NC(=O)C
ZINC08439543	6.86	2.7	-30.1	1	11	0	611.651	14	CC1=NC(=C)C([C@@H]([C@@H]1C(=O)OC/C=C/c2ccccc2)c3cccc(c3)[N+](=O)[O-])C(=O)OCCOc4ccc(cc4)NC(=O)C
ZINC08439543	6.68	1.33	-20.24	1	11	0	611.651	14	CC1=NC(=C([C@@H]([C@H]1C(=O)OC/C=C/c2ccccc2)c3cccc(c3)[N+](=O)[O-])C(=O)OCCOc4ccc(cc4)NC(=O)C)C
ZINC08439542	3.14	-0.1	-51.32	2	12	1	538.581	13	CC1=NC(=C([C@@H](C1C(=O)OCCNC(=O)c2cccnc2)c3cccc(c3)[N+](=O)[O-])C(=O)OCC[NH+](C)C)C
ZINC08439542	3.32	-0.09	-53.37	2	12	1	538.581	13	CC1=NC(=C)C([C@H](C1C(=O)OCC[NH+](C)C)c2cccc(c2)[N+](=O)[O-])C(=O)OCCNC(=O)c3cccnc3
ZINC08439542	3.32	1.25	-52.27	2	12	1	538.581	13	CC1=NC(=C)C([C@H]([C@H]1C(=O)OCC[NH+](C)C)c2cccc(c2)[N+](=O)[O-])C(=O)OCCNC(=O)c3cccnc3
ZINC08439542	3.14	-0.05	-57.87	2	12	1	538.581	13	CC1=NC(=C([C@H]([C@H]1C(=O)OCC[NH+](C)C)c2cccc(c2)[N+](=O)[O-])C(=O)OCCNC(=O)c3cccnc3)C
ZINC02170707	5.8	1.25	-16.92	0	5	0	456.567	6	COc1ccccc1n2c(=O)c3ccccc3nc2SCC(=O)c4ccc5c(c4)CCCC5
ZINC08439541	3.14	0.14	-46.13	2	12	1	538.581	13	CC1=NC(=C([C@H](C1C(=O)OCCNC(=O)c2cccnc2)c3cccc(c3)[N+](=O)[O-])C(=O)OCC[NH+](C)C)C
ZINC08439541	3.32	0.39	-50.06	2	12	1	538.581	13	CC1=NC(=C)C([C@@H](C1C(=O)OCC[NH+](C)C)c2cccc(c2)[N+](=O)[O-])C(=O)OCCNC(=O)c3cccnc3
ZINC08439541	3.32	1.39	-46.53	2	12	1	538.581	13	CC1=NC(=C)C([C@@H]([C@@H]1C(=O)OCC[NH+](C)C)c2cccc(c2)[N+](=O)[O-])C(=O)OCCNC(=O)c3cccnc3
ZINC08439541	3.14	0.27	-51.85	2	12	1	538.581	13	CC1=NC(=C([C@@H]([C@H]1C(=O)OCC[NH+](C)C)c2cccc(c2)[N+](=O)[O-])C(=O)OCCNC(=O)c3cccnc3)C
ZINC02170705	6.22	0.7	-11.54	1	6	0	513.45	9	Cc1ccccc1OCc2nnc(n2Cc3ccccc3)SCC(=O)Nc4ccc(cc4Cl)Cl
ZINC02170703	7.92	1.54	-7.64	0	3	0	453.822	6	CCCc1c(nc(s1)N(c2ccc(c(c2)Cl)Cl)C(=O)CC)c3ccc(cc3)Cl
ZINC08439540	5.38	-2.33	-62.51	1	12	1	634.131	12	CCOC(=O)C1[C@@H](C(=C(N=C1C)C)C(=O)OCC[NH+]2CCN(CC2)S(=O)(=O)c3cccc(c3)Cl)c4cccc(c4)[N+](=O)[O-]
ZINC08439540	5.38	-2.49	-71.06	1	12	1	634.131	12	CCOC(=O)C1=C(N=C(C([C@H]1c2cccc(c2)[N+](=O)[O-])C(=O)OCC[NH+]3CCN(CC3)S(=O)(=O)c4cccc(c4)Cl)C)C
ZINC08439540	5.57	-3.39	-69.88	1	12	1	634.131	12	CCOC(=O)C1[C@@H](C(C(=NC1=C)C)C(=O)OCC[NH+]2CCN(CC2)S(=O)(=O)c3cccc(c3)Cl)c4cccc(c4)[N+](=O)[O-]
ZINC02170700	7.76	1.99	-9.46	0	4	0	443.012	9	CCCCC(=O)N(c1ccc(cc1)Cl)c2nc(c(s2)CCC)c3ccc(cc3)OC
ZINC08439539	2.95	-0.32	-51.6	2	6	1	462.595	7	CSCC[C@H]1[C@H]2[C@@H](C(=O)N(C2=O)Cc3ccccc3)[C@@]4([NH2+]1)c5ccccc5N(C4=O)CC=C
ZINC08439538	5.38	-2.44	-56.44	1	12	1	634.131	12	CCOC(=O)C1[C@H](C(=C(N=C1C)C)C(=O)OCC[NH+]2CCN(CC2)S(=O)(=O)c3cccc(c3)Cl)c4cccc(c4)[N+](=O)[O-]
ZINC08439538	5.38	-2.41	-66.97	1	12	1	634.131	12	CCOC(=O)C1=C(N=C(C([C@@H]1c2cccc(c2)[N+](=O)[O-])C(=O)OCC[NH+]3CCN(CC3)S(=O)(=O)c4cccc(c4)Cl)C)C
ZINC08439538	5.57	-3.54	-62.7	1	12	1	634.131	12	CCOC(=O)C1[C@H](C(C(=NC1=C)C)C(=O)OCC[NH+]2CCN(CC2)S(=O)(=O)c3cccc(c3)Cl)c4cccc(c4)[N+](=O)[O-]
ZINC08439537	3.82	2.37	-120.2	2	10	2	474.558	10	CCOC(=O)C1[C@@H](C(=C(N=C1C)C)C(=O)OCC[NH+]2CC[NH+](CC2)C)c3cccc(c3)[N+](=O)[O-]
ZINC08439537	3.82	2.13	-125	2	10	2	474.558	10	CCOC(=O)C1=C(N=C(C([C@H]1c2cccc(c2)[N+](=O)[O-])C(=O)OCC[NH+]3CC[NH+](CC3)C)C)C
ZINC08439537	4	1.07	-128.2	2	10	2	474.558	10	CCOC(=O)C1[C@@H](C(C(=NC1=C)C)C(=O)OCC[NH+]2CC[NH+](CC2)C)c3cccc(c3)[N+](=O)[O-]
ZINC08439536	2.95	0.06	-44.5	2	6	1	462.595	7	CSCC[C@@H]1[C@H]2[C@@H](C(=O)N(C2=O)Cc3ccccc3)[C@@]4([NH2+]1)c5ccccc5N(C4=O)CC=C
ZINC08439535	5.37	0.54	-14.01	1	6	0	483.593	7	Cc1ccc(cc1)C(=O)CSC2=NC(=C([C@@H](C2C#N)c3ccco3)C(=O)Nc4ccccc4C)C
ZINC08439535	4.99	0.69	-18.2	1	6	0	483.593	7	Cc1ccc(cc1)C(=O)CSC2=C([C@H](C(C(=N2)C)C(=O)Nc3ccccc3C)c4ccco4)C#N
ZINC08439535	4.99	2.46	-14.86	1	6	0	483.593	7	Cc1ccc(cc1)C(=O)CSC2=C([C@H]([C@H](C(=N2)C)C(=O)Nc3ccccc3C)c4ccco4)C#N
ZINC08439534	3.82	2.4	-129.04	2	10	2	474.558	10	CCOC(=O)C1[C@H](C(=C(N=C1C)C)C(=O)OCC[NH+]2CC[NH+](CC2)C)c3cccc(c3)[N+](=O)[O-]
ZINC08439534	3.82	2.17	-133.86	2	10	2	474.558	10	CCOC(=O)C1=C(N=C(C([C@@H]1c2cccc(c2)[N+](=O)[O-])C(=O)OCC[NH+]3CC[NH+](CC3)C)C)C
ZINC08439534	4	1.04	-136.02	2	10	2	474.558	10	CCOC(=O)C1[C@H](C(C(=NC1=C)C)C(=O)OCC[NH+]2CC[NH+](CC2)C)c3cccc(c3)[N+](=O)[O-]

Supplementary Table S2:
Detailed table of XP Docking using Schrodinger's Glide module

ZINC_ID	xLogP	ApolarDesolvation	PolarDesolvation	H Donors	H Acceptors	Charge	MolecularWeight	RotatableBonds	SMILES
ZINC08439532	3.45	2.44	-118.21	2	10	2	460.531	9	CC1=NC(=C([C@@H](C1C(=O)OC)c2cccc(c2)[N+](=O)[O-])C(=O)OCC[NH+]3CC[NH+](CC3)C)C
ZINC08439532	3.63	2.52	-125.27	2	10	2	460.531	9	CC1=NC(=C)C([C@H](C1C(=O)OCC[NH+]2CC[NH+](CC2)C)c3cccc(c3)[N+](=O)[O-])C(=O)OC
ZINC08439532	3.63	3.71	-119.25	2	10	2	460.531	9	CC1=NC(=C)C([C@H]([C@H]1C(=O)OCC[NH+]2CC[NH+](CC2)C)c3cccc(c3)[N+](=O)[O-])C(=O)OC
ZINC08439532	3.45	2.47	-127.3	2	10	2	460.531	9	CC1=NC(=C([C@H]([C@@H]1C(=O)OCC[NH+]2CC[NH+](CC2)C)c3cccc(c3)[N+](=O)[O-])C(=O)OC)C
ZINC08439530	5.37	0.66	-12.74	1	6	0	483.593	7	Cc1ccc(cc1)C(=O)CSC2=NC(=C([C@H](C2C#N)c3ccco3)C(=O)Nc4ccccc4C)C
ZINC08439530	4.99	0.77	-14.88	1	6	0	483.593	7	Cc1ccc(cc1)C(=O)CSC2=C([C@@H](C(C(=N2)C)C(=O)Nc3ccccc3C)c4ccco4)C#N
ZINC08439530	4.99	2.61	-14.76	1	6	0	483.593	7	Cc1ccc(cc1)C(=O)CSC2=C([C@@H]([C@H](C(=N2)C)C(=O)Nc3ccccc3C)c4ccco4)C#N
ZINC08439529	3.9	0.59	-44.69	2	6	1	484.551	5	CC[C@H]1[C@H]2[C@@H](C(=O)N(C2=O)Cc3ccccc3)[C@@]4([NH2+]1)c5ccccc5N(C4=O)Cc6ccccc6F
ZINC08439528	3.45	2.32	-125.13	2	10	2	460.531	9	CC1=NC(=C([C@H](C1C(=O)OC)c2cccc(c2)[N+](=O)[O-])C(=O)OCC[NH+]3CC[NH+](CC3)C)C
ZINC08439528	3.63	2.32	-131.17	2	10	2	460.531	9	CC1=NC(=C)C([C@@H](C1C(=O)OCC[NH+]2CC[NH+](CC2)C)c3cccc(c3)[N+](=O)[O-])C(=O)OC
ZINC08439528	3.63	3.56	-128.98	2	10	2	460.531	9	CC1=NC(=C)C([C@@H]([C@@H]1C(=O)OCC[NH+]2CC[NH+](CC2)C)c3cccc(c3)[N+](=O)[O-])C(=O)OC
ZINC08439528	3.45	2.23	-132.62	2	10	2	460.531	9	CC1=NC(=C([C@@H]([C@@H]1C(=O)OCC[NH+]2CC[NH+](CC2)C)c3cccc(c3)[N+](=O)[O-])C(=O)OC)C
ZINC08439527	3.9	0.53	-41.19	2	6	1	484.551	5	CC[C@@H]1[C@H]2[C@@H](C(=O)N(C2=O)Cc3ccccc3)[C@@]4([NH2+]1)c5ccccc5N(C4=O)Cc6ccccc6F
ZINC08439526	4.79	-2.2	-55.3	1	13	1	629.712	13	CCOC(=O)C1[C@@H](C(=C(N=C1C)C)C(=O)OCC[NH+]2CCN(CC2)S(=O)(=O)c3ccc(cc3)OC)c4cccc(c4)[N+](=O)[O-]
ZINC08439526	4.79	-2.44	-60.71	1	13	1	629.712	13	CCOC(=O)C1=C(N=C(C([C@H]1c2cccc(c2)[N+](=O)[O-])C(=O)OCC[NH+]3CCN(CC3)S(=O)(=O)c4ccc(cc4)OC)C)C
ZINC08439526	4.97	-3.51	-61.65	1	13	1	629.712	13	CCOC(=O)C1[C@@H](C(C(=NC1=C)C)C(=O)OCC[NH+]2CCN(CC2)S(=O)(=O)c3ccc(cc3)OC)c4cccc(c4)[N+](=O)[O-]
ZINC08439523	4.79	-2.24	-58.4	1	13	1	629.712	13	CCOC(=O)C1[C@H](C(=C(N=C1C)C)C(=O)OCC[NH+]2CCN(CC2)S(=O)(=O)c3ccc(cc3)OC)c4cccc(c4)[N+](=O)[O-]
ZINC08439523	4.79	-2.48	-63.68	1	13	1	629.712	13	CCOC(=O)C1=C(N=C(C([C@@H]1c2cccc(c2)[N+](=O)[O-])C(=O)OCC[NH+]3CCN(CC3)S(=O)(=O)c4ccc(cc4)OC)C)C
ZINC08439523	4.97	-3.6	-64.16	1	13	1	629.712	13	CCOC(=O)C1[C@H](C(C(=NC1=C)C)C(=O)OCC[NH+]2CCN(CC2)S(=O)(=O)c3ccc(cc3)OC)c4cccc(c4)[N+](=O)[O-]
ZINC08439522	4.92	12.31	-27.82	3	7	0	519.026	7	Cc1ccccc1NC(=O)C2=C(NC(=C([C@H]2c3ccco3)C#N)SCC(=O)Nc4ccc(cc4)Cl)C
ZINC08439522	5.26	-0.95	-15.34	2	7	0	519.026	7	Cc1ccccc1NC(=O)C2=C(N=C(C([C@H]2c3ccco3)C#N)SCC(=O)Nc4ccc(cc4)Cl)C
ZINC08439522	5.44	-1.89	-15.71	2	7	0	519.026	7	Cc1ccccc1NC(=O)C2[C@@H](C(C(=NC2=C)SCC(=O)Nc3ccc(cc3)Cl)C#N)c4ccco4
ZINC08439521	7.48	1.74	-14.58	0	5	0	464.631	8	CCCc1c(nc(s1)N(c2ccc(cc2)OC)C(=O)C3CCCCC3)c4ccc(cc4)OC
ZINC08439520	5.16	0.59	-34.12	2	15	0	700.701	17	CC1=NC(=C([C@@H](C1C(=O)OCCOc2ccc(cc2)NC(=O)C)c3cccc(c3)[N+](=O)[O-])C(=O)OCCOC(=O)c4ccc(cc4)NC(=O)C)C
ZINC08439520	5.34	0.59	-32.42	2	15	0	700.701	17	CC1=NC(=C)C([C@H](C1C(=O)OCCOC(=O)c2ccc(cc2)NC(=O)C)c3cccc(c3)[N+](=O)[O-])C(=O)OCCOc4ccc(cc4)NC(=O)C
ZINC08439520	5.34	1.94	-37.62	2	15	0	700.701	17	CC1=NC(=C)C([C@H]([C@H]1C(=O)OCCOC(=O)c2ccc(cc2)NC(=O)C)c3cccc(c3)[N+](=O)[O-])C(=O)OCCOc4ccc(cc4)NC(=O)C
ZINC08439520	5.16	0.5	-33.64	2	15	0	700.701	17	CC1=NC(=C([C@H]([C@H]1C(=O)OCCOC(=O)c2ccc(cc2)NC(=O)C)c3cccc(c3)[N+](=O)[O-])C(=O)OCCOc4ccc(cc4)NC(=O)C)C
ZINC08439518	3.92	-1.26	-42.35	3	8	1	569.707	8	Cc1ccc(cc1)NC(=O)CN2c3ccccc3[C@]4(C2=O)[C@H]5[C@@H]([C@@H]([NH2+]4)CCSC)C(=O)N(C5=O)Cc6ccccc6
ZINC08439517	4.8	-1.77	-66.11	1	12	1	599.686	11	Cc1ccc(cc1)S(=O)(=O)N2CC[NH+](CC2)CCOC(=O)C3[C@@H](C(=C(N=C3C)C)C(=O)OC)c4cccc(c4)[N+](=O)[O-]
ZINC08439517	4.8	-2.22	-56.08	1	12	1	599.686	11	Cc1ccc(cc1)S(=O)(=O)N2CC[NH+](CC2)CCOC(=O)C3=C(N=C(C([C@H]3c4cccc(c4)[N+](=O)[O-])C(=O)OC)C)C
ZINC08439517	4.99	-3.09	-58.01	1	12	1	599.686	11	Cc1ccc(cc1)S(=O)(=O)N2CC[NH+](CC2)CCOC(=O)C3[C@@H](C(C(=NC3=C)C)C(=O)OC)c4cccc(c4)[N+](=O)[O-]
ZINC08439516	3.92	-0.49	-55.96	3	8	1	569.707	8	Cc1ccc(cc1)NC(=O)CN2c3ccccc3[C@@]4(C2=O)[C@H]5[C@@H]([C@@H]([NH2+]4)CCSC)C(=O)N(C5=O)Cc6ccccc6
ZINC08439515	4.92	11.92	-33.23	3	7	0	519.026	7	Cc1ccccc1NC(=O)C2=C(NC(=C([C@@H]2c3ccco3)C#N)SCC(=O)Nc4ccc(cc4)Cl)C
ZINC08439515	5.26	-1.29	-16.7	2	7	0	519.026	7	Cc1ccccc1NC(=O)C2=C(N=C(C([C@@H]2c3ccco3)C#N)SCC(=O)Nc4ccc(cc4)Cl)C
ZINC08439515	5.44	-2.16	-16.44	2	7	0	519.026	7	Cc1ccccc1NC(=O)C2[C@H](C(C(=NC2=C)SCC(=O)Nc3ccc(cc3)Cl)C#N)c4ccco4
ZINC08439513	4.8	-1.77	-60.04	1	12	1	599.686	11	Cc1ccc(cc1)S(=O)(=O)N2CC[NH+](CC2)CCOC(=O)C3[C@H](C(=C(N=C3C)C)C(=O)OC)c4cccc(c4)[N+](=O)[O-]
ZINC08439513	4.8	-2.09	-55.82	1	12	1	599.686	11	Cc1ccc(cc1)S(=O)(=O)N2CC[NH+](CC2)CCOC(=O)C3=C(N=C(C([C@@H]3c4cccc(c4)[N+](=O)[O-])C(=O)OC)C)C
ZINC08439513	4.99	-3.03	-59.08	1	12	1	599.686	11	Cc1ccc(cc1)S(=O)(=O)N2CC[NH+](CC2)CCOC(=O)C3[C@H](C(C(=NC3=C)C)C(=O)OC)c4cccc(c4)[N+](=O)[O-]
ZINC08439511	5.66	-1.38	-30.09	3	15	0	686.674	16	CC1=NC(=C([C@@H](C1C(=O)OCCNC(=O)c2ccccc2O)c3cccc(c3)[N+](=O)[O-])C(=O)OCCOC(=O)c4ccc(cc4)NC(=O)C)C
ZINC08439511	5.84	-0.02	-32.27	3	15	0	686.674	16	CC1=NC(=C)C([C@H]([C@H]1C(=O)OCCOC(=O)c2ccc(cc2)NC(=O)C)c3cccc(c3)[N+](=O)[O-])C(=O)OCCNC(=O)c4ccccc4O
ZINC08439511	5.66	-1.31	-30.94	3	15	0	686.674	16	CC1=NC(=C([C@H]([C@H]1C(=O)OCCOC(=O)c2ccc(cc2)NC(=O)C)c3cccc(c3)[N+](=O)[O-])C(=O)OCCNC(=O)c4ccccc4O)C
ZINC00643347	5.99	13.19	-13.67	1	5	0	446.934	5	Cc1ccc(cc1)NC(=O)Cc2c(n(c3c2cc(cc3)OC)C(=O)c4ccc(cc4)Cl)C
ZINC08439509	5.66	-1.31	-33.48	3	15	0	686.674	16	CC1=NC(=C([C@H](C1C(=O)OCCNC(=O)c2ccccc2O)c3cccc(c3)[N+](=O)[O-])C(=O)OCCOC(=O)c4ccc(cc4)NC(=O)C)C
ZINC08439509	5.84	-1.31	-30.67	3	15	0	686.674	16	CC1=NC(=C)C([C@@H](C1C(=O)OCCOC(=O)c2ccc(cc2)NC(=O)C)c3cccc(c3)[N+](=O)[O-])C(=O)OCCNC(=O)c4ccccc4O
ZINC08439509	5.84	0	-34.76	3	15	0	686.674	16	CC1=NC(=C)C([C@@H]([C@@H]1C(=O)OCCOC(=O)c2ccc(cc2)NC(=O)C)c3cccc(c3)[N+](=O)[O-])C(=O)OCCNC(=O)c4ccccc4O
ZINC08439509	5.66	-1.4	-30.92	3	15	0	686.674	16	CC1=NC(=C([C@@H]([C@@H]1C(=O)OCCOC(=O)c2ccc(cc2)NC(=O)C)c3cccc(c3)[N+](=O)[O-])C(=O)OCCNC(=O)c4ccccc4O)C
ZINC02170682	7.89	2.88	-9.47	0	3	0	461.03	6	CCc1c(nc(s1)N(c2ccc(cc2C)C)C(=O)Cc3ccccc3)c4ccc(cc4)Cl
ZINC08439508	5.17	-4.93	-29.52	4	14	0	644.637	14	CC1=NC(=C([C@@H](C1C(=O)OCCNC(=O)c2ccc(cc2)O)c3cccc(c3)[N+](=O)[O-])C(=O)OCCNC(=O)c4ccccc4O)C
ZINC08439508	5.35	-4.94	-28.1	4	14	0	644.637	14	CC1=NC(=C)C([C@H](C1C(=O)OCCNC(=O)c2ccccc2O)c3cccc(c3)[N+](=O)[O-])C(=O)OCCNC(=O)c4ccc(cc4)O
ZINC08439508	5.35	-3.65	-33.53	4	14	0	644.637	14	CC1=NC(=C)C([C@H]([C@H]1C(=O)OCCNC(=O)c2ccccc2O)c3cccc(c3)[N+](=O)[O-])C(=O)OCCNC(=O)c4ccc(cc4)O
ZINC08439508	5.17	-5.04	-29.15	4	14	0	644.637	14	CC1=NC(=C([C@H]([C@H]1C(=O)OCCNC(=O)c2ccccc2O)c3cccc(c3)[N+](=O)[O-])C(=O)OCCNC(=O)c4ccc(cc4)O)C
ZINC02170679	7.02	2.23	-15.42	0	4	0	463.002	7	Cc1c(nc(s1)N(c2ccccc2Cl)C(=O)CCc3ccccc3)c4ccc(cc4)OC
ZINC02170675	8.86	1.98	-8.3	0	3	0	515.893	7	CCc1c(nc(s1)N(c2ccc(cc2Cl)Cl)C(=O)CCc3ccccc3)c4ccc(cc4)Cl
ZINC08439505	4.35	1.97	-24.19	1	12	0	537.525	12	CC1=NC(=C([C@@H](C1C(=O)OC)c2cccc(c2)[N+](=O)[O-])C(=O)OCCOC(=O)c3ccc(cc3)NC(=O)C)C
ZINC08439505	4.53	1.89	-22.53	1	12	0	537.525	12	CC1=NC(=C)C([C@H](C1C(=O)OCCOC(=O)c2ccc(cc2)NC(=O)C)c3cccc(c3)[N+](=O)[O-])C(=O)OC
ZINC08439505	4.53	3.28	-26.74	1	12	0	537.525	12	CC1=NC(=C)C([C@H]([C@H]1C(=O)OCCOC(=O)c2ccc(cc2)NC(=O)C)c3cccc(c3)[N+](=O)[O-])C(=O)OC
ZINC00643346	4.59	1.05	-15.65	2	5	0	416.811	5	COc1cccc(c1)C(=O)Nc2cccc(c2)NC(=O)c3cc(c(cc3Cl)F)F
ZINC08439504	4.35	1.89	-20.34	1	12	0	537.525	12	CC1=NC(=C([C@H](C1C(=O)OC)c2cccc(c2)[N+](=O)[O-])C(=O)OCCOC(=O)c3ccc(cc3)NC(=O)C)C
ZINC08439504	4.53	1.86	-20.58	1	12	0	537.525	12	CC1=NC(=C)C([C@@H](C1C(=O)OCCOC(=O)c2ccc(cc2)NC(=O)C)c3cccc(c3)[N+](=O)[O-])C(=O)OC
ZINC08439504	4.53	3.14	-23.59	1	12	0	537.525	12	CC1=NC(=C)C([C@@H]([C@@H]1C(=O)OCCOC(=O)c2ccc(cc2)NC(=O)C)c3cccc(c3)[N+](=O)[O-])C(=O)OC
ZINC08439504	4.35	1.8	-21.46	1	12	0	537.525	12	CC1=NC(=C([C@@H]([C@@H]1C(=O)OCCOC(=O)c2ccc(cc2)NC(=O)C)c3cccc(c3)[N+](=O)[O-])C(=O)OC)C

Supplementary Table S2:
Detailed table of XP Docking using Schrodinger's Glide module

ZINC_ID	xLogP	ApolarDesolvation	PolarDesolvation	H Donors	H Acceptors	Charge	MolecularWeight	RotatableBonds	SMILES
ZINC08439503	6.13	13.77	-21.73	1	5	0	445.559	7	CCOc1cccc(c1)[C@H]2C3=C(CC(CC3=O)(C)C)NC(=C2C(=O)OCc4ccccc4)C
ZINC08439503	6.25	0.04	-9.3	0	5	0	445.559	7	CCOc1cccc(c1)[C@H]2C3C(=O)CC(C=C3N=C(C2C(=O)OCc4ccccc4)C)(C)C
ZINC02170668	6.61	6.79	-4.82	0	2	0	429.404	3	Cc1ccc2c(c1)[C@H](CC(N2C(=O)c3cc(cc(c3)C(F)(F)F)C(F)(F)F)(C)C)C
ZINC08439502	4.72	0.47	-24.2	1	13	0	607.616	14	CC1=NC(=C([C@@H](C1C(=O)OCCOC(=O)c2ccc(cc2)NC(=O)C)c3cccc(c3)[N+](=O)[O-])C(=O)OC[C@H]4CCCO4)C
ZINC08439502	4.91	0.48	-21.53	1	13	0	607.616	14	CC1=NC(=C)C([C@H](C1C(=O)OC[C@H]2CCCO2)c3cccc(c3)[N+](=O)[O-])C(=O)OCCOC(=O)c4ccc(cc4)NC(=O)C
ZINC08439502	4.91	1.86	-26.98	1	13	0	607.616	14	CC1=NC(=C)C([C@H]([C@H]1C(=O)OC[C@H]2CCCO2)c3cccc(c3)[N+](=O)[O-])C(=O)OCCOC(=O)c4ccc(cc4)NC(=O)C
ZINC08439502	4.72	0.44	-22.01	1	13	0	607.616	14	CC1=NC(=C([C@H]([C@@H]1C(=O)OC[C@H]2CCCO2)c3cccc(c3)[N+](=O)[O-])C(=O)OCCOC(=O)c4ccc(cc4)NC(=O)C)C
ZINC02170670	6.61	6.82	-4.84	0	2	0	429.404	3	Cc1ccc2c(c1)[C@@H](CC(N2C(=O)c3cc(cc(c3)C(F)(F)F)C(F)(F)F)(C)C)C
ZINC08439501	4.72	0.32	-22.83	1	13	0	607.616	14	CC1=NC(=C([C@H](C1C(=O)OCCOC(=O)c2ccc(cc2)NC(=O)C)c3cccc(c3)[N+](=O)[O-])C(=O)OC[C@H]4CCCO4)C
ZINC08439501	4.91	0.35	-21.78	1	13	0	607.616	14	CC1=NC(=C)C([C@@H](C1C(=O)OC[C@H]2CCCO2)c3cccc(c3)[N+](=O)[O-])C(=O)OCCOC(=O)c4ccc(cc4)NC(=O)C
ZINC08439501	4.91	1.73	-31.09	1	13	0	607.616	14	CC1=NC(=C)C([C@@H]([C@@H]1C(=O)OC[C@H]2CCCO2)c3cccc(c3)[N+](=O)[O-])C(=O)OCCOC(=O)c4ccc(cc4)NC(=O)C
ZINC08439501	4.72	0.4	-21.79	1	13	0	607.616	14	CC1=NC(=C([C@@H]([C@H]1C(=O)OC[C@H]2CCCO2)c3cccc(c3)[N+](=O)[O-])C(=O)OCCOC(=O)c4ccc(cc4)NC(=O)C)C
ZINC08439500	6.13	13.76	-21.68	1	5	0	445.559	7	CCOc1cccc(c1)[C@@H]2C3=C(CC(CC3=O)(C)C)NC(=C2C(=O)OCc4ccccc4)C
ZINC08439500	6.25	-0.1	-8.59	0	5	0	445.559	7	CCOc1cccc(c1)[C@@H]2C3C(=O)CC(C=C3N=C(C2C(=O)OCc4ccccc4)C)(C)C
ZINC00969336	5.7	-0.23	-6.88	0	4	0	448.371	4	CC1=NC2=C([C@@H](C1C(=O)OC)c3ccc(c(c3)Cl)Cl)C(=O)C[C@@H](C2)c4cccs4
ZINC00969336	5.88	0	-7.63	0	4	0	448.371	4	COC(=O)C1[C@H](C2C(=NC1=C)C[C@H](CC2=O)c3cccs3)c4ccc(c(c4)Cl)Cl
ZINC00969336	5.88	1.22	-13.48	0	4	0	448.371	4	COC(=O)C1[C@H]([C@@H]2C(=NC1=C)C[C@H](CC2=O)c3cccs3)c4ccc(c(c4)Cl)Cl
ZINC00969336	5.7	-0.12	-9.05	0	4	0	448.371	4	CC1=C([C@H]([C@H]2C(=N1)C[C@H](CC2=O)c3cccs3)c4ccc(c(c4)Cl)Cl)C(=O)OC
ZINC00969337	5.7	-0.23	-7.01	0	4	0	448.371	4	CC1=NC2=C([C@H](C1C(=O)OC)c3ccc(c(c3)Cl)Cl)C(=O)C[C@@H](C2)c4cccs4
ZINC00969337	5.88	-0.24	-7.99	0	4	0	448.371	4	COC(=O)C1[C@@H](C2C(=NC1=C)C[C@H](CC2=O)c3cccs3)c4ccc(c(c4)Cl)Cl
ZINC00969337	5.88	0.99	-7.38	0	4	0	448.371	4	COC(=O)C1[C@@H]([C@H]2C(=NC1=C)C[C@H](CC2=O)c3cccs3)c4ccc(c(c4)Cl)Cl
ZINC00969337	5.7	-0.18	-9.19	0	4	0	448.371	4	CC1=C([C@@H]([C@@H]2C(=N1)C[C@H](CC2=O)c3cccs3)c4ccc(c(c4)Cl)Cl)C(=O)OC
ZINC00969338	5.7	-0.32	-7.37	0	4	0	448.371	4	CC1=NC2=C([C@@H](C1C(=O)OC)c3ccc(c(c3)Cl)Cl)C(=O)C[C@H](C2)c4cccs4
ZINC00969338	5.88	-0.23	-7.64	0	4	0	448.371	4	COC(=O)C1[C@H](C2C(=NC1=C)C[C@@H](CC2=O)c3cccs3)c4ccc(c(c4)Cl)Cl
ZINC00969338	5.88	0.98	-7.31	0	4	0	448.371	4	COC(=O)C1[C@H]([C@@H]2C(=NC1=C)C[C@@H](CC2=O)c3cccs3)c4ccc(c(c4)Cl)Cl
ZINC00969338	5.7	-0.11	-9.02	0	4	0	448.371	4	CC1=C([C@H]([C@H]2C(=N1)C[C@@H](CC2=O)c3cccs3)c4ccc(c(c4)Cl)Cl)C(=O)OC
ZINC00969339	5.7	-0.23	-6.86	0	4	0	448.371	4	CC1=NC2=C([C@H](C1C(=O)OC)c3ccc(c(c3)Cl)Cl)C(=O)C[C@H](C2)c4cccs4
ZINC00969339	5.88	-0.17	-8.15	0	4	0	448.371	4	COC(=O)C1[C@@H](C2C(=NC1=C)C[C@@H](CC2=O)c3cccs3)c4ccc(c(c4)Cl)Cl
ZINC00969339	5.88	1.25	-12.89	0	4	0	448.371	4	COC(=O)C1[C@@H]([C@H]2C(=NC1=C)C[C@@H](CC2=O)c3cccs3)c4ccc(c(c4)Cl)Cl
ZINC00969339	5.7	-0.29	-9.62	0	4	0	448.371	4	CC1=C([C@@H]([C@H]2C(=N1)C[C@@H](CC2=O)c3cccs3)c4ccc(c(c4)Cl)Cl)C(=O)OC
ZINC04113089	3.87	-0.3	-51.72	0	9	-1	469.495	6	CC1=NC2=C([C@@H](C1C(=O)OC)c3cc(c(c(c3)OC)[O-])[N+](=O)[O-])C(=O)C[C@@H](C2)c4cccs4
ZINC04113089	4.05	-0.24	-53.07	0	9	-1	469.495	6	COc1cc(cc(c1[O-])[N+](=O)[O-])[C@H]2C3C(=NC(=C)C2C(=O)OC)C[C@H](CC3=O)c4cccs4
ZINC04113089	4.05	1.09	-54.64	0	9	-1	469.495	6	COc1cc(cc(c1[O-])[N+](=O)[O-])[C@H]2[C@@H]3C(=NC(=C)C2C(=O)OC)C[C@H](CC3=O)c4cccs4
ZINC04113089	3.87	-0.36	-56.02	0	9	-1	469.495	6	CC1=C([C@H]([C@@H]2C(=N1)C[C@H](CC2=O)c3cccs3)c4cc(c(c(c4)OC)[O-])[N+](=O)[O-])C(=O)OC
ZINC08439499	4.86	-0.09	-50.94	2	6	1	562.715	7	CSCC[C@H]1[C@H]2[C@@H](C(=O)N(C2=O)Cc3ccccc3)[C@@]4([NH2+]1)c5ccccc5N(C4=O)Cc6cccc7c6cccc7
ZINC04113088	3.87	-0.4	-54.64	0	9	-1	469.495	6	CC1=NC2=C([C@H](C1C(=O)OC)c3cc(c(c(c3)OC)[O-])[N+](=O)[O-])C(=O)C[C@@H](C2)c4cccs4
ZINC04113088	4.05	-0.3	-53.46	0	9	-1	469.495	6	COc1cc(cc(c1[O-])[N+](=O)[O-])[C@@H]2C3C(=NC(=C)C2C(=O)OC)C[C@H](CC3=O)c4cccs4
ZINC04113088	4.05	1.01	-62.41	0	9	-1	469.495	6	COc1cc(cc(c1[O-])[N+](=O)[O-])[C@@H]2[C@H]3C(=NC(=C)C2C(=O)OC)C[C@H](CC3=O)c4cccs4
ZINC04113088	3.87	-0.26	-54.15	0	9	-1	469.495	6	CC1=C([C@@H]([C@@H]2C(=N1)C[C@H](CC2=O)c3cccs3)c4cc(c(c(c4)OC)[O-])[N+](=O)[O-])C(=O)OC
ZINC04113087	3.87	-0.29	-52.25	0	9	-1	469.495	6	CC1=NC2=C([C@@H](C1C(=O)OC)c3cc(c(c(c3)OC)[O-])[N+](=O)[O-])C(=O)C[C@H](C2)c4cccs4
ZINC04113087	4.05	-0.14	-51.61	0	9	-1	469.495	6	COc1cc(cc(c1[O-])[N+](=O)[O-])[C@H]2C3C(=NC(=C)C2C(=O)OC)C[C@@H](CC3=O)c4cccs4
ZINC04113087	4.05	1.16	-58.71	0	9	-1	469.495	6	COc1cc(cc(c1[O-])[N+](=O)[O-])[C@H]2[C@@H]3C(=NC(=C)C2C(=O)OC)C[C@@H](CC3=O)c4cccs4
ZINC04113087	3.87	-0.26	-53.87	0	9	-1	469.495	6	CC1=C([C@H]([C@H]2C(=N1)C[C@@H](CC2=O)c3cccs3)c4cc(c(c(c4)OC)[O-])[N+](=O)[O-])C(=O)OC
ZINC04113086	3.87	-0.3	-51.44	0	9	-1	469.495	6	CC1=NC2=C([C@H](C1C(=O)OC)c3cc(c(c(c3)OC)[O-])[N+](=O)[O-])C(=O)C[C@H](C2)c4cccs4
ZINC04113086	4.05	-0.24	-53.14	0	9	-1	469.495	6	COc1cc(cc(c1[O-])[N+](=O)[O-])[C@@H]2C3C(=NC(=C)C2C(=O)OC)C[C@@H](CC3=O)c4cccs4
ZINC04113086	4.05	1.18	-59.82	0	9	-1	469.495	6	COc1cc(cc(c1[O-])[N+](=O)[O-])[C@@H]2[C@H]3C(=NC(=C)C2C(=O)OC)C[C@@H](CC3=O)c4cccs4
ZINC04113086	3.87	-0.36	-55.58	0	9	-1	469.495	6	CC1=C([C@@H]([C@H]2C(=N1)C[C@@H](CC2=O)c3cccs3)c4cc(c(c(c4)OC)[O-])[N+](=O)[O-])C(=O)OC
ZINC08439498	4.72	0.35	-23.42	1	13	0	607.616	14	CC1=NC(=C([C@@H](C1C(=O)OCCOC(=O)c2ccc(cc2)NC(=O)C)c3cccc(c3)[N+](=O)[O-])C(=O)OC[C@@H]4CCCO4)C
ZINC08439498	4.91	0.28	-22.95	1	13	0	607.616	14	CC1=NC(=C)C([C@H](C1C(=O)OC[C@@H]2CCCO2)c3cccc(c3)[N+](=O)[O-])C(=O)OCCOC(=O)c4ccc(cc4)NC(=O)C
ZINC08439498	4.91	1.69	-26.02	1	13	0	607.616	14	CC1=NC(=C)C([C@H]([C@H]1C(=O)OC[C@@H]2CCCO2)c3cccc(c3)[N+](=O)[O-])C(=O)OCCOC(=O)c4ccc(cc4)NC(=O)C
ZINC08439498	4.72	0.32	-22.96	1	13	0	607.616	14	CC1=NC(=C([C@H]([C@H]1C(=O)OC[C@@H]2CCCO2)c3cccc(c3)[N+](=O)[O-])C(=O)OCCOC(=O)c4ccc(cc4)NC(=O)C)C
ZINC08439497	4.86	0.29	-50.16	2	6	1	562.715	7	CSCC[C@@H]1[C@H]2[C@@H](C(=O)N(C2=O)Cc3ccccc3)[C@@]4([NH2+]1)c5ccccc5N(C4=O)Cc6cccc7c6cccc7
ZINC08439495	4.72	0.24	-21.67	1	13	0	607.616	14	CC1=NC(=C([C@H](C1C(=O)OCCOC(=O)c2ccc(cc2)NC(=O)C)c3cccc(c3)[N+](=O)[O-])C(=O)OC[C@@H]4CCCO4)C
ZINC08439495	4.91	0.27	-20.67	1	13	0	607.616	14	CC1=NC(=C)C([C@@H](C1C(=O)OC[C@@H]2CCCO2)c3cccc(c3)[N+](=O)[O-])C(=O)OCCOC(=O)c4ccc(cc4)NC(=O)C
ZINC08439495	4.91	1.42	-21.92	1	13	0	607.616	14	CC1=NC(=C)C([C@@H]([C@@H]1C(=O)OC[C@@H]2CCCO2)c3cccc(c3)[N+](=O)[O-])C(=O)OCCOC(=O)c4ccc(cc4)NC(=O)C
ZINC08439495	4.72	0.32	-21.07	1	13	0	607.616	14	CC1=NC(=C([C@@H]([C@H]1C(=O)OC[C@@H]2CCCO2)c3cccc(c3)[N+](=O)[O-])C(=O)OCCOC(=O)c4ccc(cc4)NC(=O)C)C
ZINC08439494	5.31	-0.21	-8.64	0	4	0	452.348	5	CC1=NC2=C([C@@H](C1C(=O)OCc3ccccc3)c4ccccc4Br)C(=O)CCC2
ZINC08439494	5.49	-0.15	-9.56	0	4	0	452.348	5	C=C1C([C@H](C2C(=N1)CCCC2=O)c3ccccc3Br)C(=O)OCc4ccccc4
ZINC08439494	5.49	1.18	-10.69	0	4	0	452.348	5	C=C1C([C@H]([C@@H]2C(=N1)CCCC2=O)c3ccccc3Br)C(=O)OCc4ccccc4
ZINC08439494	5.31	-0.27	-12.23	0	4	0	452.348	5	CC1=C([C@H]([C@@H]2C(=N1)CCCC2=O)c3ccccc3Br)C(=O)OCc4ccccc4
ZINC08439493	5.78	2.93	-63.83	2	13	1	671.727	17	CC1=NC(=C([C@@H](C1C(=O)OCC[NH+](C)Cc2ccccc2)c3cccc(c3)[N+](=O)[O-])C(=O)OCCOC(=O)c4ccc(cc4)NC(=O)C)C

Supplementary Table S2:
Detailed table of XP Docking using Schrodinger's Glide module

ZINC_ID	xLogP	ApolarDesolvation	PolarDesolvation	H Donors	H Acceptors	Charge	MolecularWeight	RotatableBonds	SMILES
ZINC08439493	5.96	3.05	-57.23	2	13	1	671.727	17	CC1=NC(=C)C([C@H](C1C(=O)OCCOC(=O)c2ccc(cc2)NC(=O)C)c3cccc(c3)[N+](=O)[O-])C(=O)OCC[NH+](C)Cc4ccccc4
ZINC08439493	5.96	4.28	-65	2	13	1	671.727	17	CC1=NC(=C)C([C@H]([C@H]1C(=O)OCCOC(=O)c2ccc(cc2)NC(=O)C)c3cccc(c3)[N+](=O)[O-])C(=O)OCC[NH+](C)Cc4ccccc4
ZINC08439493	5.78	2.79	-57.11	2	13	1	671.727	17	CC1=NC(=C([C@H]([C@@H]1C(=O)OCCOC(=O)c2ccc(cc2)NC(=O)C)c3cccc(c3)[N+](=O)[O-])C(=O)OCC[NH+](C)Cc4ccccc4)C
ZINC08439492	5.78	2.87	-55.72	2	13	1	671.727	17	CC1=NC(=C([C@H](C1C(=O)OCC[NH+](C)Cc2ccccc2)c3cccc(c3)[N+](=O)[O-])C(=O)OCCOC(=O)c4ccc(cc4)NC(=O)C)C
ZINC08439492	5.96	2.95	-53.96	2	13	1	671.727	17	CC1=NC(=C)C([C@@H](C1C(=O)OCCOC(=O)c2ccc(cc2)NC(=O)C)c3cccc(c3)[N+](=O)[O-])C(=O)OCC[NH+](C)Cc4ccccc4
ZINC08439492	5.96	4.08	-55.49	2	13	1	671.727	17	CC1=NC(=C)C([C@@H]([C@@H]1C(=O)OCCOC(=O)c2ccc(cc2)NC(=O)C)c3cccc(c3)[N+](=O)[O-])C(=O)OCC[NH+](C)Cc4ccccc4
ZINC08439492	5.78	2.8	-51.87	2	13	1	671.727	17	CC1=NC(=C([C@@H]([C@H]1C(=O)OCCOC(=O)c2ccc(cc2)NC(=O)C)c3cccc(c3)[N+](=O)[O-])C(=O)OCC[NH+](C)Cc4ccccc4)C
ZINC00643341	4.5	-1.96	-18.64	1	5	0	448.997	9	COc1ccc(cc1OC)CCNC(=O)CSc2nc(cs2)c3ccc(cc3)Cl
ZINC08439491	4.41	-3.74	-24.21	3	14	0	629.626	14	CC1=NC(=C([C@@H](C1C(=O)OCCNC(=O)c2cccnc2)c3cccc(c3)[N+](=O)[O-])C(=O)OCCNC(=O)c4ccccc4O)C
ZINC08439491	4.59	-3.68	-22.21	3	14	0	629.626	14	CC1=NC(=C)C([C@H](C1C(=O)OCCNC(=O)c2ccccc2O)c3cccc(c3)[N+](=O)[O-])C(=O)OCCNC(=O)c4cccnc4
ZINC08439491	4.59	-2.5	-27.94	3	14	0	629.626	14	CC1=NC(=C)C([C@H]([C@H]1C(=O)OCCNC(=O)c2ccccc2O)c3cccc(c3)[N+](=O)[O-])C(=O)OCCNC(=O)c4cccnc4
ZINC08439491	4.41	-3.94	-22.95	3	14	0	629.626	14	CC1=NC(=C([C@H]([C@H]1C(=O)OCCNC(=O)c2ccccc2O)c3cccc(c3)[N+](=O)[O-])C(=O)OCCNC(=O)c4cccnc4)C
ZINC00643337	4.57	-1.5	-12.65	1	6	0	443.594	6	CCOc1ccccc1NC(=O)CSc2nc3c(c4c(s3)C[C@H](CC4)C)c(=O)n2C
ZINC08439490	5.31	-0.2	-8.68	0	4	0	452.348	5	CC1=NC2=C([C@H](C1C(=O)OCc3ccccc3)c4ccccc4Br)C(=O)CCC2
ZINC08439490	5.49	-0.38	-9.18	0	4	0	452.348	5	C=C1C([C@@H](C2C(=N1)CCCC2=O)c3ccccc3Br)C(=O)OCc4ccccc4
ZINC08439490	5.49	1.01	-15.95	0	4	0	452.348	5	C=C1C([C@@H]([C@H]2C(=N1)CCCC2=O)c3ccccc3Br)C(=O)OCc4ccccc4
ZINC08439490	5.31	-0.03	-12.21	0	4	0	452.348	5	CC1=C([C@@H]([C@@H]2C(=N1)CCCC2=O)c3ccccc3Br)C(=O)OCc4ccccc4
ZINC08439489	4.41	-3.67	-28.05	3	14	0	629.626	14	CC1=NC(=C([C@H](C1C(=O)OCCNC(=O)c2cccnc2)c3cccc(c3)[N+](=O)[O-])C(=O)OCCNC(=O)c4ccccc4O)C
ZINC08439489	4.59	-3.51	-26.66	3	14	0	629.626	14	CC1=NC(=C)C([C@@H](C1C(=O)OCCNC(=O)c2ccccc2O)c3cccc(c3)[N+](=O)[O-])C(=O)OCCNC(=O)c4cccnc4
ZINC08439489	4.59	-2.16	-39.94	3	14	0	629.626	14	CC1=NC(=C)C([C@@H]([C@@H]1C(=O)OCCNC(=O)c2ccccc2O)c3cccc(c3)[N+](=O)[O-])C(=O)OCCNC(=O)c4cccnc4
ZINC08439489	4.41	-3.76	-27.95	3	14	0	629.626	14	CC1=NC(=C([C@@H]([C@H]1C(=O)OCCNC(=O)c2ccccc2O)c3cccc(c3)[N+](=O)[O-])C(=O)OCCNC(=O)c4cccnc4)C
ZINC00643339	4.57	-1.5	-12.68	1	6	0	443.594	6	CCOc1ccccc1NC(=O)CSc2nc3c(c4c(s3)C[C@@H](CC4)C)c(=O)n2C
ZINC08439487	6.14	-4.09	-26.41	4	14	0	644.637	14	CC1=NC(=C([C@H](C1C(=O)OCCNC(=O)c2ccccc2O)c3cccc(c3)[N+](=O)[O-])C(=O)OCCNC(=O)c4ccccc4O)C
ZINC08439487	6.32	-4.12	-25.49	4	14	0	644.637	14	CC1=NC(=C)C([C@@H](C1C(=O)OCCNC(=O)c2ccccc2O)c3cccc(c3)[N+](=O)[O-])C(=O)OCCNC(=O)c4ccccc4O
ZINC08439487	6.32	-2.62	-32.15	4	14	0	644.637	14	CC1=NC(=C)C([C@@H]([C@@H]1C(=O)OCCNC(=O)c2ccccc2O)c3cccc(c3)[N+](=O)[O-])C(=O)OCCNC(=O)c4ccccc4O
ZINC08439487	6.14	-4.25	-25.52	4	14	0	644.637	14	CC1=NC(=C([C@@H]([C@@H]1C(=O)OCCNC(=O)c2ccccc2O)c3cccc(c3)[N+](=O)[O-])C(=O)OCCNC(=O)c4ccccc4O)C
ZINC08439486	5.18	1.72	-33.04	2	16	0	728.711	18	CC1=NC(=C([C@H](C1C(=O)OCCOC(=O)c2ccc(cc2)NC(=O)C)c3cccc(c3)[N+](=O)[O-])C(=O)OCCOC(=O)c4ccc(cc4)NC(=O)C)C
ZINC08439486	5.36	1.71	-31.89	2	16	0	728.711	18	CC1=NC(=C)C([C@@H](C1C(=O)OCCOC(=O)c2ccc(cc2)NC(=O)C)c3cccc(c3)[N+](=O)[O-])C(=O)OCCOC(=O)c4ccc(cc4)NC(=O)C
ZINC08439486	5.36	3.22	-39.65	2	16	0	728.711	18	CC1=NC(=C)C([C@@H]([C@@H]1C(=O)OCCOC(=O)c2ccc(cc2)NC(=O)C)c3cccc(c3)[N+](=O)[O-])C(=O)OCCOC(=O)c4ccc(cc4)NC(=O)C
ZINC08439486	5.18	1.59	-32.49	2	16	0	728.711	18	CC1=NC(=C([C@@H]([C@@H]1C(=O)OCCOC(=O)c2ccc(cc2)NC(=O)C)c3cccc(c3)[N+](=O)[O-])C(=O)OCCOC(=O)c4ccc(cc4)NC(=O)C)C
ZINC08439484	3.86	-1.66	-20.21	2	11	0	495.488	10	CC1=NC(=C([C@@H](C1C(=O)OC)c2cccc(c2)[N+](=O)[O-])C(=O)OCCNC(=O)c3ccc(cc3)O)C
ZINC08439484	4.05	-1.55	-20.28	2	11	0	495.488	10	CC1=NC(=C)C([C@H](C1C(=O)OCCNC(=O)c2ccc(cc2)O)c3cccc(c3)[N+](=O)[O-])C(=O)OC
ZINC08439484	4.05	-0.38	-21.45	2	11	0	495.488	10	CC1=NC(=C)C([C@H]([C@H]1C(=O)OCCNC(=O)c2ccc(cc2)O)c3cccc(c3)[N+](=O)[O-])C(=O)OC
ZINC08439484	3.86	-1.61	-22.2	2	11	0	495.488	10	CC1=NC(=C([C@H]([C@@H]1C(=O)OCCNC(=O)c2ccc(cc2)O)c3cccc(c3)[N+](=O)[O-])C(=O)OC)C
ZINC08439483	6.04	0.23	-10.18	0	5	0	485.605	7	CC1=NC2=C([C@@H](C1C(=O)OC)c3cccc(c3)OCc4ccccc4)C(=O)C[C@@H](C2)c5cccs5
ZINC08439483	6.23	0.29	-10.64	0	5	0	485.605	7	COC(=O)C1[C@H](C2C(=NC1=C)C[C@H](CC2=O)c3cccs3)c4cccc(c4)OCc5ccccc5
ZINC08439483	6.23	1.62	-12.42	0	5	0	485.605	7	COC(=O)C1[C@H]([C@@H]2C(=NC1=C)C[C@H](CC2=O)c3cccs3)c4cccc(c4)OCc5ccccc5
ZINC08439483	6.04	0.17	-12.67	0	5	0	485.605	7	CC1=C([C@H]([C@@H]2C(=N1)C[C@H](CC2=O)c3cccs3)c4cccc(c4)OCc5ccccc5)C(=O)OC
ZINC02170649	6.8	1.9	-9.46	0	4	0	435.376	6	CCc1c(nc(s1)N(c2ccc(cc2Cl)Cl)C(=O)CC)c3ccc(cc3)OC
ZINC08439482	3.86	-1.77	-16.9	2	11	0	495.488	10	CC1=NC(=C([C@H](C1C(=O)OC)c2cccc(c2)[N+](=O)[O-])C(=O)OCCNC(=O)c3ccc(cc3)O)C
ZINC08439482	4.05	-1.83	-17.62	2	11	0	495.488	10	CC1=NC(=C)C([C@@H](C1C(=O)OCCNC(=O)c2ccc(cc2)O)c3cccc(c3)[N+](=O)[O-])C(=O)OC
ZINC08439482	4.05	-0.28	-24.05	2	11	0	495.488	10	CC1=NC(=C)C([C@@H]([C@@H]1C(=O)OCCNC(=O)c2ccc(cc2)O)c3cccc(c3)[N+](=O)[O-])C(=O)OC
ZINC08439482	3.86	-1.78	-18.11	2	11	0	495.488	10	CC1=NC(=C([C@@H]([C@H]1C(=O)OCCNC(=O)c2ccc(cc2)O)c3cccc(c3)[N+](=O)[O-])C(=O)OC)C
ZINC02170646	5.82	-3.13	-11.37	1	5	0	459.681	5	C1CCC(CC1)NC(=O)CSc2nc3c(c4c(s3)CCCC4)c(=O)n2C5CCCCC5
ZINC02170635	7.97	16.98	-12.53	1	5	0	545.774	8	CCc1ccccc1NC(=O)CSc2nc3c(c4c(s3)C[C@H](CC4)C(C)(C)C)c(=O)n2Cc5ccccc5
ZINC08439481	4.71	-0.61	-24.19	1	12	0	579.606	13	CC1=NC(=C([C@@H](C1C(=O)OCCOc2ccc(cc2)NC(=O)C)c3cccc(c3)[N+](=O)[O-])C(=O)OC[C@H]4CCCO4)C
ZINC08439481	4.89	-0.52	-21.58	1	12	0	579.606	13	CC1=NC(=C)C([C@H](C1C(=O)OC[C@H]2CCCO2)c3cccc(c3)[N+](=O)[O-])C(=O)OCCOc4ccc(cc4)NC(=O)C
ZINC08439481	4.89	0.87	-27.51	1	12	0	579.606	13	CC1=NC(=C)C([C@H]([C@H]1C(=O)OC[C@H]2CCCO2)c3cccc(c3)[N+](=O)[O-])C(=O)OCCOc4ccc(cc4)NC(=O)C
ZINC08439481	4.71	-0.57	-22.68	1	12	0	579.606	13	CC1=NC(=C([C@H]([C@@H]1C(=O)OC[C@H]2CCCO2)c3cccc(c3)[N+](=O)[O-])C(=O)OCCOc4ccc(cc4)NC(=O)C)C
ZINC08439480	4.71	-0.72	-22.71	1	12	0	579.606	13	CC1=NC(=C([C@H](C1C(=O)OCCOc2ccc(cc2)NC(=O)C)c3cccc(c3)[N+](=O)[O-])C(=O)OC[C@H]4CCCO4)C
ZINC08439480	4.71	-0.74	-22.16	1	12	0	579.606	13	CC1=NC(=C([C@@H]([C@@H]1C(=O)OC[C@H]2CCCO2)c3cccc(c3)[N+](=O)[O-])C(=O)OCCOc4ccc(cc4)NC(=O)C)C
ZINC08439479	6.04	0.23	-10.3	0	5	0	485.605	7	CC1=NC2=C([C@H](C1C(=O)OC)c3cccc(c3)OCc4ccccc4)C(=O)C[C@@H](C2)c5cccs5
ZINC08439479	6.23	0.4	-10.67	0	5	0	485.605	7	COC(=O)C1[C@@H](C2C(=NC1=C)C[C@H](CC2=O)c3cccs3)c4cccc(c4)OCc5ccccc5
ZINC08439479	6.23	1.71	-19.68	0	5	0	485.605	7	COC(=O)C1[C@@H]([C@H]2C(=NC1=C)C[C@H](CC2=O)c3cccs3)c4cccc(c4)OCc5ccccc5
ZINC08439479	6.04	0.28	-12.58	0	5	0	485.605	7	CC1=C([C@@H]([C@@H]2C(=N1)C[C@H](CC2=O)c3cccs3)c4cccc(c4)OCc5ccccc5)C(=O)OC
ZINC02170638	7.97	0.56	-11.86	1	5	0	545.774	8	CCc1ccccc1NC(=O)CSc2nc3c(c4c(s3)C[C@@H](CC4)C(C)(C)C)c(=O)n2Cc5ccccc5
ZINC08439478	4.71	-0.52	-24.16	1	12	0	579.606	13	CC1=NC(=C([C@@H](C1C(=O)OCCOc2ccc(cc2)NC(=O)C)c3cccc(c3)[N+](=O)[O-])C(=O)OC[C@@H]4CCCO4)C
ZINC08439478	4.89	-0.42	-21.4	1	12	0	579.606	13	CC1=NC(=C)C([C@H](C1C(=O)OC[C@@H]2CCCO2)c3cccc(c3)[N+](=O)[O-])C(=O)OCCOc4ccc(cc4)NC(=O)C
ZINC08439478	4.71	-0.46	-22.16	1	12	0	579.606	13	CC1=NC(=C([C@H]([C@@H]1C(=O)OC[C@@H]2CCCO2)c3cccc(c3)[N+](=O)[O-])C(=O)OCCOc4ccc(cc4)NC(=O)C)C
ZINC02170633	7.28	1.6	-8.54	0	3	0	443.41	6	CCCC(=O)N(c1ccc(cc1)Br)c2nc(c(s2)CC)c3ccc(cc3)C
ZINC08439477	5.9	0.93	-47.8	2	6	1	537.039	4	CC[C@H]1[C@H]2[C@@H](C(=O)N(C2=O)c3cccc(c3)Cl)[C@@]4([NH2+]1)c5ccccc5N(C4=O)Cc6cccc7c6cccc7

Supplementary Table S2:
Detailed table of XP Docking using Schrodinger's Glide module

ZINC_ID	xLogP	ApolarDesolvation	PolarDesolvation	H Donors	H Acceptors	Charge	MolecularWeight	RotatableBonds	SMILES
ZINC08439476	4.71	-0.71	-21.15	1	12	0	579.606	13	CC1=NC(=C([C@H](C1C(=O)OCCOc2ccc(cc2)NC(=O)C)c3cccc(c3)[N+](=O)[O-])C(=O)OC[C@@H]4CCCO4)C
ZINC08439476	4.89	-0.68	-20.04	1	12	0	579.606	13	CC1=NC(=C)C([C@@H](C1C(=O)OC[C@@H]2CCCO2)c3cccc(c3)[N+](=O)[O-])C(=O)OCCOc4ccc(cc4)NC(=O)C
ZINC08439476	4.89	0.47	-21.48	1	12	0	579.606	13	CC1=NC(=C)C([C@@H]([C@@H]1C(=O)OC[C@@H]2CCCO2)c3cccc(c3)[N+](=O)[O-])C(=O)OCCOc4ccc(cc4)NC(=O)C
ZINC08439476	4.71	-0.63	-20.34	1	12	0	579.606	13	CC1=NC(=C([C@@H]([C@H]1C(=O)OC[C@@H]2CCCO2)c3cccc(c3)[N+](=O)[O-])C(=O)OCCOc4ccc(cc4)NC(=O)C)C
ZINC08439475	6.04	0.15	-10.43	0	5	0	485.605	7	CC1=NC2=C([C@@H](C1C(=O)OC)c3cccc(c3)OCc4ccccc4)C(=O)C[C@H](C2)c5cccs5
ZINC08439475	6.23	0.3	-10.54	0	5	0	485.605	7	COC(=O)C1[C@H](C2C(=NC1=C)C[C@@H](CC2=O)c3cccs3)c4cccc(c4)OCc5ccccc5
ZINC08439475	6.23	1.56	-11.4	0	5	0	485.605	7	COC(=O)C1[C@H]([C@@H]2C(=NC1=C)C[C@@H](CC2=O)c3cccs3)c4cccc(c4)OCc5ccccc5
ZINC08439475	6.04	0.43	-12.21	0	5	0	485.605	7	CC1=C([C@H]([C@H]2C(=N1)C[C@@H](CC2=O)c3cccs3)c4cccc(c4)OCc5ccccc5)C(=O)OC
ZINC08439474	5.9	0.52	-51.13	2	6	1	537.039	4	CC[C@@H]1[C@H]2[C@@H](C(=O)N(C2=O)c3cccc(c3)Cl)[C@@]4([NH2+]1)c5ccccc5N(C4=O)Cc6cccc7c6cccc7
ZINC08439473	4.78	-1.8	-16.74	2	11	0	515.544	11	CCOC(=O)C1[C@@H](C(=C(N=C1C)C)C(=O)OCCNC(=O)c2ccccc2O)c3ccc(s3)[N+](=O)[O-]
ZINC08439473	4.78	-2.2	-19.71	2	11	0	515.544	11	CCOC(=O)C1=C(N=C(C([C@H]1c2ccc(s2)[N+](=O)[O-])C(=O)OCCNC(=O)c3ccccc3O)C)C
ZINC08439473	4.96	-3.16	-19.01	2	11	0	515.544	11	CCOC(=O)C1[C@@H](C(C(=NC1=C)C)C(=O)OCCNC(=O)c2ccccc2O)c3ccc(s3)[N+](=O)[O-]
ZINC08439471	4.78	-1.97	-14.84	2	11	0	515.544	11	CCOC(=O)C1[C@H](C(=C(N=C1C)C)C(=O)OCCNC(=O)c2ccccc2O)c3ccc(s3)[N+](=O)[O-]
ZINC08439471	4.78	-2.21	-16.43	2	11	0	515.544	11	CCOC(=O)C1=C(N=C(C([C@@H]1c2ccc(s2)[N+](=O)[O-])C(=O)OCCNC(=O)c3ccccc3O)C)C
ZINC08439471	4.96	-3.32	-16.45	2	11	0	515.544	11	CCOC(=O)C1[C@H](C(C(=NC1=C)C)C(=O)OCCNC(=O)c2ccccc2O)c3ccc(s3)[N+](=O)[O-]
ZINC08439469	5.57	-0.93	-19.18	2	11	0	523.542	11	CC1=NC(=C([C@@H](C1C(=O)OCCNC(=O)c2ccccc2O)c3cccc(c3)[N+](=O)[O-])C(=O)OC(C)C)C
ZINC08439469	5.75	0.42	-21.28	2	11	0	523.542	11	CC1=NC(=C)C([C@H]([C@H]1C(=O)OC(C)C)c2cccc(c2)[N+](=O)[O-])C(=O)OCCNC(=O)c3ccccc3O
ZINC08439469	5.57	-0.88	-18.92	2	11	0	523.542	11	CC1=NC(=C([C@H]([C@H]1C(=O)OC(C)C)c2cccc(c2)[N+](=O)[O-])C(=O)OCCNC(=O)c3ccccc3O)C
ZINC08439468	6.04	0.25	-10.17	0	5	0	485.605	7	CC1=NC2=C([C@H](C1C(=O)OC)c3cccc(c3)OCc4ccccc4)C(=O)C[C@H](C2)c5cccs5
ZINC08439468	6.23	0.31	-10.53	0	5	0	485.605	7	COC(=O)C1[C@@H](C2C(=NC1=C)C[C@@H](CC2=O)c3cccs3)c4cccc(c4)OCc5ccccc5
ZINC08439468	6.23	1.47	-12.78	0	5	0	485.605	7	COC(=O)C1[C@@H]([C@H]2C(=NC1=C)C[C@@H](CC2=O)c3cccs3)c4cccc(c4)OCc5ccccc5
ZINC08439468	6.04	0.2	-12.46	0	5	0	485.605	7	CC1=C([C@@H]([C@H]2C(=N1)C[C@@H](CC2=O)c3cccs3)c4cccc(c4)OCc5ccccc5)C(=O)OC
ZINC02170629	7.2	1.24	-8.86	0	4	0	473.436	8	CCCc1c(nc(s1)N(c2ccc(cc2)Br)C(=O)CC)c3ccc(cc3)OCC
ZINC08439467	5.57	-0.86	-20.04	2	11	0	523.542	11	CC1=NC(=C([C@H](C1C(=O)OCCNC(=O)c2ccccc2O)c3cccc(c3)[N+](=O)[O-])C(=O)OC(C)C)C
ZINC08439467	5.75	-0.87	-16.89	2	11	0	523.542	11	CC1=NC(=C)C([C@@H](C1C(=O)OC(C)C)c2cccc(c2)[N+](=O)[O-])C(=O)OCCNC(=O)c3ccccc3O
ZINC08439467	5.75	0.41	-20.71	2	11	0	523.542	11	CC1=NC(=C)C([C@@H]([C@@H]1C(=O)OC(C)C)c2cccc(c2)[N+](=O)[O-])C(=O)OCCNC(=O)c3ccccc3O
ZINC08439467	5.57	-0.96	-16.9	2	11	0	523.542	11	CC1=NC(=C([C@@H]([C@@H]1C(=O)OC(C)C)c2cccc(c2)[N+](=O)[O-])C(=O)OCCNC(=O)c3ccccc3O)C
ZINC02170623	7.54	2.61	-15.4	0	4	0	478.617	7	Cc1c(nc(s1)N(c2cccc3c2cccc3)C(=O)CCc4ccccc4)c5ccc(cc5)OC
ZINC06299035	6.29	18.46	-14.63	1	5	0	518.686	9	C[C@H](C(=O)NC(c1ccccc1)c2ccccc2)Sc3nnc(n3C)C(c4ccccc4)c5ccccc5
ZINC06298704	6.29	18.46	-14.77	1	5	0	518.686	9	C[C@@H](C(=O)NC(c1ccccc1)c2ccccc2)Sc3nnc(n3C)C(c4ccccc4)c5ccccc5
ZINC08439466	4.73	-2.22	-54.27	1	12	1	599.686	12	CCOC(=O)C1[C@@H](C(=C(N=C1C)C)C(=O)OCC[NH+]2CCN(CC2)S(=O)(=O)c3ccccc3)c4cccc(c4)[N+](=O)[O-]
ZINC08439466	4.73	-2.46	-59.5	1	12	1	599.686	12	CCOC(=O)C1=C(N=C(C([C@H]1c2cccc(c2)[N+](=O)[O-])C(=O)OCC[NH+]3CCN(CC3)S(=O)(=O)c4ccccc4)C)C
ZINC08439466	4.91	-3.53	-60.52	1	12	1	599.686	12	CCOC(=O)C1[C@@H](C(C(=NC1=C)C)C(=O)OCC[NH+]2CCN(CC2)S(=O)(=O)c3ccccc3)c4cccc(c4)[N+](=O)[O-]
ZINC08439465	4.29	-0.53	-13.4	0	6	0	478.383	8	CC1=NC2=C([C@@H](C1C(=O)OCCOC(C)C)c3cc(ccc3OC)Br)C(=O)CCC2
ZINC08439465	4.47	-0.42	-13.66	0	6	0	478.383	8	CC(C)OCCOC(=O)C1[C@H](C2C(=NC1=C)CCCC2=O)c3cc(ccc3OC)Br
ZINC08439465	4.47	0.79	-16.62	0	6	0	478.383	8	CC(C)OCCOC(=O)C1[C@H]([C@@H]2C(=NC1=C)CCCC2=O)c3cc(ccc3OC)Br
ZINC08439465	4.29	-0.68	-15.22	0	6	0	478.383	8	CC1=C([C@H]([C@@H]2C(=N1)CCCC2=O)c3cc(ccc3OC)Br)C(=O)OCCOC(C)C
ZINC08439464	4.73	-2.27	-56.95	1	12	1	599.686	12	CCOC(=O)C1[C@H](C(=C(N=C1C)C)C(=O)OCC[NH+]2CCN(CC2)S(=O)(=O)c3ccccc3)c4cccc(c4)[N+](=O)[O-]
ZINC08439464	4.73	-2.5	-62.21	1	12	1	599.686	12	CCOC(=O)C1=C(N=C(C([C@@H]1c2cccc(c2)[N+](=O)[O-])C(=O)OCC[NH+]3CCN(CC3)S(=O)(=O)c4ccccc4)C)C
ZINC08439464	4.91	-3.62	-62.66	1	12	1	599.686	12	CCOC(=O)C1[C@H](C(C(=NC1=C)C)C(=O)OCC[NH+]2CCN(CC2)S(=O)(=O)c3ccccc3)c4cccc(c4)[N+](=O)[O-]
ZINC08439463	6.64	12.64	-14.07	2	7	0	581.47	6	Cc1c(c(n(n1)c2ccccc2)O)C(c3ccc(o3)c4ccc(cc4)Br)c5c(nn(c5O)c6ccccc6)C
ZINC08439462	3.9	-0.31	-20.02	1	11	0	515.544	11	CC1=NC(=C([C@@H](C1C(=O)OC)c2ccc(s2)[N+](=O)[O-])C(=O)OCCOc3ccc(cc3)NC(=O)C)C
ZINC08439462	4.09	-0.31	-20.16	1	11	0	515.544	11	CC1=NC(=C)C([C@H](C1C(=O)OCCOc2ccc(cc2)NC(=O)C)c3ccc(s3)[N+](=O)[O-])C(=O)OC
ZINC08439462	4.09	1.12	-25.88	1	11	0	515.544	11	CC1=NC(=C)C([C@H]([C@H]1C(=O)OCCOc2ccc(cc2)NC(=O)C)c3ccc(s3)[N+](=O)[O-])C(=O)OC
ZINC08439462	3.9	-0.27	-20.02	1	11	0	515.544	11	CC1=NC(=C([C@H]([C@H]1C(=O)OCCOc2ccc(cc2)NC(=O)C)c3ccc(s3)[N+](=O)[O-])C(=O)OC)C
ZINC00643314	6.06	2.9	-11.85	0	6	0	438.549	9	CCc1c(nc(s1)N(c2ccc(cc2)C(=O)OC)C(=O)CC)c3ccc(cc3)OCC
ZINC08439461	3.9	-0.25	-19.3	1	11	0	515.544	11	CC1=NC(=C([C@H](C1C(=O)OC)c2ccc(s2)[N+](=O)[O-])C(=O)OCCOc3ccc(cc3)NC(=O)C)C
ZINC08439461	4.09	-0.09	-19.27	1	11	0	515.544	11	CC1=NC(=C)C([C@@H](C1C(=O)OCCOc2ccc(cc2)NC(=O)C)c3ccc(s3)[N+](=O)[O-])C(=O)OC
ZINC08439461	4.09	1.02	-19.83	1	11	0	515.544	11	CC1=NC(=C)C([C@@H]([C@@H]1C(=O)OCCOc2ccc(cc2)NC(=O)C)c3ccc(s3)[N+](=O)[O-])C(=O)OC
ZINC08439461	3.9	-0.34	-19.5	1	11	0	515.544	11	CC1=NC(=C([C@@H]([C@@H]1C(=O)OCCOc2ccc(cc2)NC(=O)C)c3ccc(s3)[N+](=O)[O-])C(=O)OC)C
ZINC08439460	5.64	-2.26	-31.11	3	14	0	658.664	15	CC1=NC(=C([C@@H](C1C(=O)OCCNC(=O)c2ccccc2O)c3cccc(c3)[N+](=O)[O-])C(=O)OCCOc4ccc(cc4)NC(=O)C)C
ZINC08439460	5.82	-2.12	-27.88	3	14	0	658.664	15	CC1=NC(=C)C([C@H](C1C(=O)OCCOc2ccc(cc2)NC(=O)C)c3cccc(c3)[N+](=O)[O-])C(=O)OCCNC(=O)c4ccccc4O
ZINC08439460	5.82	-0.9	-33.8	3	14	0	658.664	15	CC1=NC(=C)C([C@H]([C@H]1C(=O)OCCOc2ccc(cc2)NC(=O)C)c3cccc(c3)[N+](=O)[O-])C(=O)OCCNC(=O)c4ccccc4O
ZINC08439460	5.64	-2.37	-28.66	3	14	0	658.664	15	CC1=NC(=C([C@H]([C@@H]1C(=O)OCCOc2ccc(cc2)NC(=O)C)c3cccc(c3)[N+](=O)[O-])C(=O)OCCNC(=O)c4ccccc4O)C
ZINC08439459	4.29	-0.6	-12.49	0	6	0	478.383	8	CC1=NC2=C([C@H](C1C(=O)OCCOC(C)C)c3cc(ccc3OC)Br)C(=O)CCC2
ZINC08439459	4.47	-0.47	-13.64	0	6	0	478.383	8	CC(C)OCCOC(=O)C1[C@@H](C2C(=NC1=C)CCCC2=O)c3cc(ccc3OC)Br
ZINC08439459	4.47	0.48	-12.37	0	6	0	478.383	8	CC(C)OCCOC(=O)C1[C@@H]([C@H]2C(=NC1=C)CCCC2=O)c3cc(ccc3OC)Br
ZINC08439459	4.29	-0.74	-15.04	0	6	0	478.383	8	CC1=C([C@@H]([C@H]2C(=N1)CCCC2=O)c3cc(ccc3OC)Br)C(=O)OCCOC(C)C
ZINC02170620	4.93	0.59	-24.2	0	6	0	474.007	6	CC1=C(S[C@]2(N1Cc3ccccc3)N(N=C(S2)C(=O)OC)c4ccccc4Cl)C(=O)C
ZINC08439458	5.64	-2.12	-29.12	3	14	0	658.664	15	CC1=NC(=C([C@H](C1C(=O)OCCNC(=O)c2ccccc2O)c3cccc(c3)[N+](=O)[O-])C(=O)OCCOc4ccc(cc4)NC(=O)C)C
ZINC08439458	5.82	-2.14	-25.91	3	14	0	658.664	15	CC1=NC(=C)C([C@@H](C1C(=O)OCCOc2ccc(cc2)NC(=O)C)c3cccc(c3)[N+](=O)[O-])C(=O)OCCNC(=O)c4ccccc4O

Supplementary Table S2:
Detailed table of XP Docking using Schrodinger's Glide module

ZINC_ID	xLogP	ApolarDesolvation	PolarDesolvation	H Donors	H Acceptors	Charge	MolecularWeight	RotatableBonds	SMILES
ZINC08439458	5.82	-0.87	-29.6	3	14	0	658.664	15	CC1=NC(=C)C([C@@H]([C@@H]1C(=O)OCCOc2ccc(cc2)NC(=O)C)c3cccc(c3)[N+](=O)[O-])C(=O)OCCNC(=O)c4ccccc4O
ZINC08439458	5.64	-2.42	-26.21	3	14	0	658.664	15	CC1=NC(=C([C@@H]([C@@H]1C(=O)OCCOc2ccc(cc2)NC(=O)C)c3cccc(c3)[N+](=O)[O-])C(=O)OCCNC(=O)c4ccccc4O)C
ZINC02170616	4.93	0.84	-10.38	0	6	0	474.007	6	CC1=C(S[C@@]2(N1Cc3ccccc3)N(N=C(S2)C(=O)OC)c4ccccc4Cl)C(=O)C
ZINC08439457	5.21	-0.84	-21.01	2	11	0	509.515	11	CCOC(=O)C1[C@@H](C(=C(N=C1C)C)C(=O)OCCNC(=O)c2ccccc2O)c3cccc(c3)[N+](=O)[O-]
ZINC08439457	5.21	-1.08	-22.5	2	11	0	509.515	11	CCOC(=O)C1=C(N=C(C([C@H]1c2cccc(c2)[N+](=O)[O-])C(=O)OCCNC(=O)c3ccccc3O)C)C
ZINC08439457	5.39	-1.97	-20.99	2	11	0	509.515	11	CCOC(=O)C1[C@@H](C(C(=NC1=C)C)C(=O)OCCNC(=O)c2ccccc2O)c3cccc(c3)[N+](=O)[O-]
ZINC08439456	4.17	0.55	-56.19	2	7	1	546.644	7	COc1ccc(cc1)N2C(=O)[C@H]3[C@H]([NH2+][C@@]4([C@@H]3C2=O)c5ccccc5N(C4=O)Cc6ccccc6F)CCSC
ZINC08439455	6.14	13.92	-20.87	1	5	0	445.559	7	Cc1cccc(c1)[C@H]2C3=C(CC(CC3=O)(C)C)NC(=C2C(=O)OCCOc4ccccc4)C
ZINC08439455	6.26	0.27	-9.7	0	5	0	445.559	7	Cc1cccc(c1)[C@H]2C3C(=O)CC(C=C3N=C(C2C(=O)OCCOc4ccccc4)C)(C)C
ZINC08439454	5.21	-0.94	-15.52	2	11	0	509.515	11	CCOC(=O)C1[C@H](C(=C(N=C1C)C)C(=O)OCCNC(=O)c2ccccc2O)c3cccc(c3)[N+](=O)[O-]
ZINC08439454	5.21	-1.18	-17.76	2	11	0	509.515	11	CCOC(=O)C1=C(N=C(C([C@@H]1c2cccc(c2)[N+](=O)[O-])C(=O)OCCNC(=O)c3ccccc3O)C)C
ZINC08439454	5.39	-2.3	-16.17	2	11	0	509.515	11	CCOC(=O)C1[C@H](C(C(=NC1=C)C)C(=O)OCCNC(=O)c2ccccc2O)c3cccc(c3)[N+](=O)[O-]
ZINC08439452	4.21	1.2	-18.96	1	11	0	523.542	12	CCOC(=O)C1[C@@H](C(=C(N=C1C)C)C(=O)OCCOc2ccc(cc2)NC(=O)C)c3ccccc3[N+](=O)[O-]
ZINC08439452	4.21	0.81	-19.11	1	11	0	523.542	12	CCOC(=O)C1=C(N=C(C([C@H]1c2ccccc2[N+](=O)[O-])C(=O)OCCOc3ccc(cc3)NC(=O)C)C)C
ZINC08439452	4.39	-0.09	-18.9	1	11	0	523.542	12	CCOC(=O)C1[C@@H](C(C(=NC1=C)C)C(=O)OCCOc2ccc(cc2)NC(=O)C)c3ccccc3[N+](=O)[O-]
ZINC08439451	4.17	0.44	-48.84	2	7	1	546.644	7	COc1ccc(cc1)N2C(=O)[C@H]3[C@@H]([NH2+][C@@]4([C@@H]3C2=O)c5ccccc5N(C4=O)Cc6ccccc6F)CCSC
ZINC08439450	6.14	13.69	-20.28	1	5	0	445.559	7	Cc1cccc(c1)[C@@H]2C3=C(CC(CC3=O)(C)C)NC(=C2C(=O)OCCOc4ccccc4)C
ZINC08439450	6.26	-0.07	-8.68	0	5	0	445.559	7	Cc1cccc(c1)[C@@H]2C3C(=O)CC(C=C3N=C(C2C(=O)OCCOc4ccccc4)C)(C)C
ZINC08439449	4.21	1.42	-15.72	1	11	0	523.542	12	CCOC(=O)C1[C@H](C(=C(N=C1C)C)C(=O)OCCOc2ccc(cc2)NC(=O)C)c3ccccc3[N+](=O)[O-]
ZINC08439449	4.21	1.28	-16.43	1	11	0	523.542	12	CCOC(=O)C1=C(N=C(C([C@@H]1c2ccccc2[N+](=O)[O-])C(=O)OCCOc3ccc(cc3)NC(=O)C)C)C
ZINC08439449	4.39	0.33	-16.07	1	11	0	523.542	12	CCOC(=O)C1[C@H](C(C(=NC1=C)C)C(=O)OCCOc2ccc(cc2)NC(=O)C)c3ccccc3[N+](=O)[O-]
ZINC08439447	4.57	1.05	-26.51	1	11	0	537.569	12	CC1=NC(=C([C@@H](C1C(=O)OCCOc2ccc(cc2)NC(=O)C)c3ccccc3[N+](=O)[O-])C(=O)OC(C)C)C
ZINC08439447	4.75	1.28	-24.86	1	11	0	537.569	12	CC1=NC(=C)C([C@H](C1C(=O)OC(C)C)c2ccccc2[N+](=O)[O-])C(=O)OCCOc3ccc(cc3)NC(=O)C
ZINC08439447	4.75	2.44	-31.34	1	11	0	537.569	12	CC1=NC(=C)C([C@H]([C@H]1C(=O)OC(C)C)c2ccccc2[N+](=O)[O-])C(=O)OCCOc3ccc(cc3)NC(=O)C
ZINC08439447	4.57	1.05	-26.04	1	11	0	537.569	12	CC1=NC(=C([C@H]([C@H]1C(=O)OC(C)C)c2ccccc2[N+](=O)[O-])C(=O)OCCOc3ccc(cc3)NC(=O)C)C
ZINC02170611	6.03	5.29	-8.25	1	2	0	423.356	6	c1ccc(cc1)C(c2ccccc2)NC(=O)c3cc(cc(c3)C(F)(F)F)C(F)(F)F
ZINC08439446	6.12	13.49	-24.22	1	6	0	475.585	9	CCOc1cccc(c1)[C@H]2C3=C(CC(CC3=O)(C)C)NC(=C2C(=O)OCCOc4ccccc4)C
ZINC08439446	6.24	-0.01	-11.24	0	6	0	475.585	9	CCOc1cccc(c1)[C@H]2C3C(=O)CC(C=C3N=C(C2C(=O)OCCOc4ccccc4)C)(C)C
ZINC08439445	4.57	1.11	-20.9	1	11	0	537.569	12	CC1=NC(=C([C@H](C1C(=O)OCCOc2ccc(cc2)NC(=O)C)c3ccccc3[N+](=O)[O-])C(=O)OC(C)C)C
ZINC08439445	4.75	1.14	-20.51	1	11	0	537.569	12	CC1=NC(=C)C([C@@H](C1C(=O)OC(C)C)c2ccccc2[N+](=O)[O-])C(=O)OCCOc3ccc(cc3)NC(=O)C
ZINC08439445	4.75	2.26	-20	1	11	0	537.569	12	CC1=NC(=C)C([C@@H]([C@@H]1C(=O)OC(C)C)c2ccccc2[N+](=O)[O-])C(=O)OCCOc3ccc(cc3)NC(=O)C
ZINC08439445	4.57	0.92	-21.04	1	11	0	537.569	12	CC1=NC(=C([C@@H]([C@@H]1C(=O)OC(C)C)c2ccccc2[N+](=O)[O-])C(=O)OCCOc3ccc(cc3)NC(=O)C)C
ZINC08439444	5.98	-1.34	-12.33	2	4	0	455.023	5	CSc1ccc(c(c1)C(=O)Nc2ccc(cc2)NC(=O)C34CC5CC(C3)CC(C5)C4)Cl
ZINC08439443	5.14	-0.49	-28.89	2	14	0	672.691	16	CC1=NC(=C([C@H](C1C(=O)OCCOc2ccc(cc2)NC(=O)C)c3cccc(c3)[N+](=O)[O-])C(=O)OCCOc4ccc(cc4)NC(=O)C)C
ZINC08439443	5.32	-0.49	-28.31	2	14	0	672.691	16	CC1=NC(=C)C([C@@H](C1C(=O)OCCOc2ccc(cc2)NC(=O)C)c3cccc(c3)[N+](=O)[O-])C(=O)OCCOc4ccc(cc4)NC(=O)C
ZINC08439443	5.32	1.02	-35.44	2	14	0	672.691	16	CC1=NC(=C)C([C@@H]([C@@H]1C(=O)OCCOc2ccc(cc2)NC(=O)C)c3cccc(c3)[N+](=O)[O-])C(=O)OCCOc4ccc(cc4)NC(=O)C
ZINC08439443	5.14	-0.61	-28.86	2	14	0	672.691	16	CC1=NC(=C([C@@H]([C@@H]1C(=O)OCCOc2ccc(cc2)NC(=O)C)c3cccc(c3)[N+](=O)[O-])C(=O)OCCOc4ccc(cc4)NC(=O)C)C
ZINC08439442	6.12	13.31	-23.47	1	6	0	475.585	9	CCOc1cccc(c1)[C@@H]2C3=C(CC(CC3=O)(C)C)NC(=C2C(=O)OCCOc4ccccc4)C
ZINC08439442	6.24	-0.03	-10.89	0	6	0	475.585	9	CCOc1cccc(c1)[C@@H]2C3C(=O)CC(C=C3N=C(C2C(=O)OCCOc4ccccc4)C)(C)C
ZINC02170573	5.53	0.51	-13.7	1	6	0	476.96	8	Cc1c(c2cc(ccc2n1C(=O)c3ccc(cc3)Cl)OC)CC(=O)NCCOc4ccccc4
ZINC08439441	3.74	1.81	-54.5	1	12	1	539.565	10	CC1=NC(=C([C@H](C1C(=O)OC)c2cccc(c2)[N+](=O)[O-])C(=O)OCC[NH+]3CCN(CC3)C(=O)c4ccco4)C
ZINC08439441	3.92	1.91	-59.17	1	12	1	539.565	10	CC1=NC(=C)C([C@@H](C1C(=O)OCC[NH+]2CCN(CC2)C(=O)c3ccco3)c4cccc(c4)[N+](=O)[O-])C(=O)OC
ZINC08439441	3.92	3.06	-54.41	1	12	1	539.565	10	CC1=NC(=C)C([C@@H]([C@@H]1C(=O)OCC[NH+]2CCN(CC2)C(=O)c3ccco3)c4cccc(c4)[N+](=O)[O-])C(=O)OC
ZINC08439441	3.74	1.8	-59.83	1	12	1	539.565	10	CC1=NC(=C([C@@H]([C@H]1C(=O)OCC[NH+]2CCN(CC2)C(=O)c3ccco3)c4cccc(c4)[N+](=O)[O-])C(=O)OC)C
ZINC02170558	6.02	2.36	-10.11	0	3	0	428.891	5	c1ccc(cc1)Cn2c(=O)c3ccccc3nc2SCc4cc(c(cc4Cl)F)F
ZINC08439440	4.13	0.13	-23.08	1	12	0	553.568	14	CC1=NC(=C([C@@H](C1C(=O)OCCOC)c2cccc(c2)[N+](=O)[O-])C(=O)OCCOc3ccc(cc3)NC(=O)C)C
ZINC08439440	4.31	0.21	-21.5	1	12	0	553.568	14	CC1=NC(=C)C([C@H](C1C(=O)OCCOc2ccc(cc2)NC(=O)C)c3cccc(c3)[N+](=O)[O-])C(=O)OCCOC
ZINC08439440	4.31	1.42	-25.79	1	12	0	553.568	14	CC1=NC(=C)C([C@H]([C@H]1C(=O)OCCOc2ccc(cc2)NC(=O)C)c3cccc(c3)[N+](=O)[O-])C(=O)OCCOC
ZINC08439440	4.13	0.13	-22.45	1	12	0	553.568	14	CC1=NC(=C([C@H]([C@@H]1C(=O)OCCOc2ccc(cc2)NC(=O)C)c3cccc(c3)[N+](=O)[O-])C(=O)OCCOC)C
ZINC08439439	5.8	-0.58	-11.11	0	5	0	482.374	7	CC1=NC2=C([C@@H](C1C(=O)OCCOc3ccccc3)c4cccc(c4)Br)C(=O)CCC2
ZINC08439439	5.98	-0.37	-11.72	0	5	0	482.374	7	C=C1C([C@H](C2C(=N1)CCCC2=O)c3cccc(c3)Br)C(=O)OCCOc4ccccc4
ZINC08439439	5.98	0.63	-11.69	0	5	0	482.374	7	C=C1C([C@H]([C@@H]2C(=N1)CCCC2=O)c3cccc(c3)Br)C(=O)OCCOc4ccccc4
ZINC08439439	5.8	-0.49	-14.08	0	5	0	482.374	7	CC1=C([C@H]([C@H]2C(=N1)CCCC2=O)c3cccc(c3)Br)C(=O)OCCOc4ccccc4
ZINC08439438	4.13	0.33	-20.44	1	12	0	553.568	14	CC1=NC(=C([C@H](C1C(=O)OCCOC)c2cccc(c2)[N+](=O)[O-])C(=O)OCCOc3ccc(cc3)NC(=O)C)C
ZINC08439438	4.31	0.4	-20.28	1	12	0	553.568	14	CC1=NC(=C)C([C@@H](C1C(=O)OCCOc2ccc(cc2)NC(=O)C)c3cccc(c3)[N+](=O)[O-])C(=O)OCCOC
ZINC08439438	4.31	1.85	-32.79	1	12	0	553.568	14	CC1=NC(=C)C([C@@H]([C@@H]1C(=O)OCCOc2ccc(cc2)NC(=O)C)c3cccc(c3)[N+](=O)[O-])C(=O)OCCOC
ZINC08439438	4.13	0.16	-20.74	1	12	0	553.568	14	CC1=NC(=C([C@@H]([C@H]1C(=O)OCCOc2ccc(cc2)NC(=O)C)c3cccc(c3)[N+](=O)[O-])C(=O)OCCOC)C
ZINC08439437	4.53	-1.06	-21.73	2	6	0	496.654	12	COc1ccccc1NC(=O)CSCc2cccc(c2)CSCC(=O)Nc3ccccc3OC
ZINC08439436	3.63	0.02	-22.83	1	12	0	553.568	14	CC1=NC(=C([C@@H](C1C(=O)OCCOC)c2ccccc2[N+](=O)[O-])C(=O)OCCOc3ccc(cc3)NC(=O)C)C
ZINC08439436	3.81	0.2	-23.06	1	12	0	553.568	14	CC1=NC(=C)C([C@H](C1C(=O)OCCOc2ccc(cc2)NC(=O)C)c3ccccc3[N+](=O)[O-])C(=O)OCCOC
ZINC08439436	3.81	1.46	-31.45	1	12	0	553.568	14	CC1=NC(=C)C([C@H]([C@H]1C(=O)OCCOc2ccc(cc2)NC(=O)C)c3ccccc3[N+](=O)[O-])C(=O)OCCOC

Supplementary Table S2:
Detailed table of XP Docking using Schrodinger's Glide module

ZINC_ID	xLogP	ApolarDesolvation	PolarDesolvation	H Donors	H Acceptors	Charge	MolecularWeight	RotatableBonds	SMILES
ZINC08439436	3.63	0.23	-23.7	1	12	0	553.568	14	CC1=NC(=C([C@H]([C@@H]1C(=O)OCCOc2ccc(cc2)NC(=O)C)c3ccccc3[N+](=O)[O-])C(=O)OCCOC)C
ZINC00643290	5.12	0.1	-12.86	1	6	0	459.571	8	CC(C)Oc1ccccc1NC(=O)CSc2nc3ccccc3c(=O)n2Cc4ccccc4
ZINC08439435	3.63	0.41	-19.25	1	12	0	553.568	14	CC1=NC(=C([C@H](C1C(=O)OCCOC)c2ccccc2[N+](=O)[O-])C(=O)OCCOc3ccc(cc3)NC(=O)C)C
ZINC08439435	3.81	0.47	-19.39	1	12	0	553.568	14	CC1=NC(=C)C([C@@H](C1C(=O)OCCOc2ccc(cc2)NC(=O)C)c3ccccc3[N+](=O)[O-])C(=O)OCCOC
ZINC08439435	3.81	1.64	-20	1	12	0	553.568	14	CC1=NC(=C)C([C@@H]([C@@H]1C(=O)OCCOc2ccc(cc2)NC(=O)C)c3ccccc3[N+](=O)[O-])C(=O)OCCOC
ZINC08439435	3.63	0.34	-19.55	1	12	0	553.568	14	CC1=NC(=C([C@@H]([C@H]1C(=O)OCCOc2ccc(cc2)NC(=O)C)c3ccccc3[N+](=O)[O-])C(=O)OCCOC)C
ZINC00643280	4.08	-2.87	-11.83	3	6	0	412.277	5	c1ccc(c(c1)NC(=O)CSc2nnc(n2N)c3ccc(cc3Cl)Cl)F
ZINC08439434	3.48	-0.44	-18.42	1	11	0	494.504	11	CCOC(=O)C1[C@@H](C(=C(N=C1C)C)C(=O)OCCNC(=O)c2cccnc2)c3cccc(c3)[N+](=O)[O-]
ZINC08439434	3.48	-0.68	-19.61	1	11	0	494.504	11	CCOC(=O)C1=C(N=C(C([C@H]1c2cccc(c2)[N+](=O)[O-])C(=O)OCCNC(=O)c3cccnc3)C)C
ZINC08439434	3.66	-1.57	-18.39	1	11	0	494.504	11	CCOC(=O)C1[C@@H](C(C(=NC1=C)C)C(=O)OCCNC(=O)c2cccnc2)c3cccc(c3)[N+](=O)[O-]
ZINC08439433	5.8	-0.59	-9.64	0	5	0	482.374	7	CC1=NC2=C([C@H](C1C(=O)OCCOc3ccccc3)c4cccc(c4)Br)C(=O)CCC2
ZINC08439433	5.98	-0.46	-10.69	0	5	0	482.374	7	C=C1C([C@@H](C2C(=N1)CCCC2=O)c3cccc(c3)Br)C(=O)OCCOc4ccccc4
ZINC08439433	5.98	0.78	-11.46	0	5	0	482.374	7	C=C1C([C@@H]([C@H]2C(=N1)CCCC2=O)c3cccc(c3)Br)C(=O)OCCOc4ccccc4
ZINC08439433	5.8	-0.59	-13.35	0	5	0	482.374	7	CC1=C([C@@H]([C@H]2C(=N1)CCCC2=O)c3cccc(c3)Br)C(=O)OCCOc4ccccc4
ZINC08439432	3.48	-0.4	-11.86	1	11	0	494.504	11	CCOC(=O)C1[C@H](C(=C(N=C1C)C)C(=O)OCCNC(=O)c2cccnc2)c3cccc(c3)[N+](=O)[O-]
ZINC08439432	3.48	-0.64	-13.63	1	11	0	494.504	11	CCOC(=O)C1=C(N=C(C([C@@H]1c2cccc(c2)[N+](=O)[O-])C(=O)OCCNC(=O)c3cccnc3)C)C
ZINC08439431	4.79	1.8	-8.7	0	8	0	434.514	10	CC1=NC(=C([C@@H](C1C(=O)OCCSC)c2cccc(c2)[N+](=O)[O-])C(=O)OC(C)C)C
ZINC08439431	4.97	1.85	-8.37	0	8	0	434.514	10	CC1=NC(=C)C([C@H](C1C(=O)OC(C)C)c2cccc(c2)[N+](=O)[O-])C(=O)OCCSC
ZINC08439431	4.97	3.41	-17.34	0	8	0	434.514	10	CC1=NC(=C)C([C@H]([C@H]1C(=O)OC(C)C)c2cccc(c2)[N+](=O)[O-])C(=O)OCCSC
ZINC08439431	4.79	1.93	-8.53	0	8	0	434.514	10	CC1=NC(=C([C@H]([C@@H]1C(=O)OC(C)C)c2cccc(c2)[N+](=O)[O-])C(=O)OCCSC)C
ZINC02170530	5.46	-3.49	-14.48	3	7	0	466.394	9	CCCCOc1ccc(cc1)NC(=O)CSc2nnc(n2N)c3ccc(cc3Cl)Cl
ZINC08439430	4.79	2.01	-14.66	0	8	0	434.514	10	CC1=NC(=C([C@H](C1C(=O)OCCSC)c2cccc(c2)[N+](=O)[O-])C(=O)OC(C)C)C
ZINC08439430	4.97	3.14	-13.74	0	8	0	434.514	10	CC1=NC(=C)C([C@@H]([C@@H]1C(=O)OC(C)C)c2cccc(c2)[N+](=O)[O-])C(=O)OCCSC
ZINC08439429	6.03	1.99	-8.6	0	4	0	433.523	6	CC1=NC2=C([C@@H](C1C(=O)OCCc3ccccc3)c4cccc(c4)F)C(=O)CC(C2)(C)C
ZINC08439429	6.21	2.47	-9.89	0	4	0	433.523	6	CC1(CC2=NC(=C)C([C@H](C2C(=O)C1)c3cccc(c3)F)C(=O)OCCc4ccccc4)C
ZINC08439429	6.21	3.41	-9.25	0	4	0	433.523	6	CC1(CC2=NC(=C)C([C@H]([C@H]2C(=O)C1)c3cccc(c3)F)C(=O)OCCc4ccccc4)C
ZINC08439429	6.03	2.52	-11.45	0	4	0	433.523	6	CC1=C([C@H]([C@H]2C(=N1)CC(CC2=O)(C)C)c3cccc(c3)F)C(=O)OCCc4ccccc4
ZINC08439428	4.54	1.08	-12.06	0	9	0	442.468	10	CC1=NC(=C([C@@H](C1C(=O)OCC=C)c2cccc(c2)[N+](=O)[O-])C(=O)OC[C@H]3CCCO3)C
ZINC08439428	4.72	1.18	-10.56	0	9	0	442.468	10	CC1=NC(=C)C([C@H](C1C(=O)OC[C@H]2CCCO2)c3cccc(c3)[N+](=O)[O-])C(=O)OCC=C
ZINC08439428	4.54	1.13	-11.85	0	9	0	442.468	10	CC1=NC(=C([C@H]([C@@H]1C(=O)OC[C@H]2CCCO2)c3cccc(c3)[N+](=O)[O-])C(=O)OCC=C)C
ZINC08439426	4.54	1.1	-10.55	0	9	0	442.468	10	CC1=NC(=C([C@H](C1C(=O)OCC=C)c2cccc(c2)[N+](=O)[O-])C(=O)OC[C@H]3CCCO3)C
ZINC08439426	4.72	1.18	-9.76	0	9	0	442.468	10	CC1=NC(=C)C([C@@H](C1C(=O)OC[C@H]2CCCO2)c3cccc(c3)[N+](=O)[O-])C(=O)OCC=C
ZINC08439426	4.72	2.61	-16.59	0	9	0	442.468	10	CC1=NC(=C)C([C@@H]([C@@H]1C(=O)OC[C@H]2CCCO2)c3cccc(c3)[N+](=O)[O-])C(=O)OCC=C
ZINC08439426	4.54	1.06	-10.18	0	9	0	442.468	10	CC1=NC(=C([C@@H]([C@@H]1C(=O)OC[C@H]2CCCO2)c3cccc(c3)[N+](=O)[O-])C(=O)OCC=C)C
ZINC08439424	6.03	2.19	-12.99	0	4	0	433.523	6	CC1=NC2=C([C@H](C1C(=O)OCCc3ccccc3)c4cccc(c4)F)C(=O)CC(C2)(C)C
ZINC08439424	6.21	2.22	-11.73	0	4	0	433.523	6	CC1(CC2=NC(=C)C([C@@H](C2C(=O)C1)c3cccc(c3)F)C(=O)OCCc4ccccc4)C
ZINC08439424	6.21	3.44	-13.72	0	4	0	433.523	6	CC1(CC2=NC(=C)C([C@@H]([C@@H]2C(=O)C1)c3cccc(c3)F)C(=O)OCCc4ccccc4)C
ZINC08439424	6.03	1.97	-13.58	0	4	0	433.523	6	CC1=C([C@@H]([C@H]2C(=N1)CC(CC2=O)(C)C)c3cccc(c3)F)C(=O)OCCc4ccccc4
ZINC00717017	6.15	0.71	-10	0	5	0	471.578	6	CC1=NC2=C([C@@H](C1C(=O)OC)c3cccc(c3)Oc4ccccc4)C(=O)C[C@@H](C2)c5cccs5
ZINC00717017	6.33	0.77	-10.43	0	5	0	471.578	6	COC(=O)C1[C@H](C2C(=NC1=C)C[C@H](CC2=O)c3cccs3)c4cccc(c4)Oc5ccccc5
ZINC00717017	6.33	2.1	-12.01	0	5	0	471.578	6	COC(=O)C1[C@H]([C@@H]2C(=NC1=C)C[C@H](CC2=O)c3cccs3)c4cccc(c4)Oc5ccccc5
ZINC00717017	6.15	0.65	-12.49	0	5	0	471.578	6	CC1=C([C@H]([C@@H]2C(=N1)C[C@H](CC2=O)c3cccs3)c4cccc(c4)Oc5ccccc5)C(=O)OC
ZINC08439423	4.54	1.11	-10.03	0	9	0	442.468	10	CC1=NC(=C([C@@H](C1C(=O)OCC=C)c2cccc(c2)[N+](=O)[O-])C(=O)OC[C@@H]3CCCO3)C
ZINC08439423	4.72	1.22	-9.56	0	9	0	442.468	10	CC1=NC(=C)C([C@H](C1C(=O)OC[C@@H]2CCCO2)c3cccc(c3)[N+](=O)[O-])C(=O)OCC=C
ZINC08439423	4.72	2.61	-19.14	0	9	0	442.468	10	CC1=NC(=C)C([C@H]([C@H]1C(=O)OC[C@@H]2CCCO2)c3cccc(c3)[N+](=O)[O-])C(=O)OCC=C
ZINC08439423	4.54	1.1	-9.93	0	9	0	442.468	10	CC1=NC(=C([C@H]([C@@H]1C(=O)OC[C@@H]2CCCO2)c3cccc(c3)[N+](=O)[O-])C(=O)OCC=C)C
ZINC00717018	6.15	0.6	-10.29	0	5	0	471.578	6	CC1=NC2=C([C@H](C1C(=O)OC)c3cccc(c3)Oc4ccccc4)C(=O)C[C@@H](C2)c5cccs5
ZINC00717018	6.33	0.7	-10.07	0	5	0	471.578	6	COC(=O)C1[C@@H](C2C(=NC1=C)C[C@H](CC2=O)c3cccs3)c4cccc(c4)Oc5ccccc5
ZINC00717018	6.33	2.01	-19.03	0	5	0	471.578	6	COC(=O)C1[C@@H]([C@H]2C(=NC1=C)C[C@H](CC2=O)c3cccs3)c4cccc(c4)Oc5ccccc5
ZINC00717018	6.15	0.74	-12.06	0	5	0	471.578	6	CC1=C([C@@H]([C@@H]2C(=N1)C[C@H](CC2=O)c3cccs3)c4cccc(c4)Oc5ccccc5)C(=O)OC
ZINC00717019	6.15	0.72	-9.58	0	5	0	471.578	6	CC1=NC2=C([C@@H](C1C(=O)OC)c3cccc(c3)Oc4ccccc4)C(=O)C[C@H](C2)c5cccs5
ZINC00717019	6.33	0.85	-10.11	0	5	0	471.578	6	COC(=O)C1[C@H](C2C(=NC1=C)C[C@@H](CC2=O)c3cccs3)c4cccc(c4)Oc5ccccc5
ZINC00717019	6.33	2.11	-11.73	0	5	0	471.578	6	COC(=O)C1[C@H]([C@@H]2C(=NC1=C)C[C@@H](CC2=O)c3cccs3)c4cccc(c4)Oc5ccccc5
ZINC00717019	6.15	0.73	-12.31	0	5	0	471.578	6	CC1=C([C@H]([C@@H]2C(=N1)C[C@@H](CC2=O)c3cccs3)c4cccc(c4)Oc5ccccc5)C(=O)OC
ZINC00717020	6.15	0.7	-9.76	0	5	0	471.578	6	CC1=NC2=C([C@H](C1C(=O)OC)c3cccc(c3)Oc4ccccc4)C(=O)C[C@H](C2)c5cccs5
ZINC00717020	6.33	0.76	-10.03	0	5	0	471.578	6	COC(=O)C1[C@@H](C2C(=NC1=C)C[C@@H](CC2=O)c3cccs3)c4cccc(c4)Oc5ccccc5
ZINC00717020	6.33	1.93	-12.42	0	5	0	471.578	6	COC(=O)C1[C@@H]([C@H]2C(=NC1=C)C[C@@H](CC2=O)c3cccs3)c4cccc(c4)Oc5ccccc5
ZINC00717020	6.15	0.65	-11.82	0	5	0	471.578	6	CC1=C([C@@H]([C@H]2C(=N1)C[C@@H](CC2=O)c3cccs3)c4cccc(c4)Oc5ccccc5)C(=O)OC
ZINC08439421	4.54	1.02	-9.63	0	9	0	442.468	10	CC1=NC(=C([C@H](C1C(=O)OCC=C)c2cccc(c2)[N+](=O)[O-])C(=O)OC[C@@H]3CCCO3)C
ZINC08439421	4.72	1.05	-9.15	0	9	0	442.468	10	CC1=NC(=C)C([C@@H](C1C(=O)OC[C@@H]2CCCO2)c3cccc(c3)[N+](=O)[O-])C(=O)OCC=C
ZINC08439421	4.72	2.21	-9.96	0	9	0	442.468	10	CC1=NC(=C)C([C@@H]([C@@H]1C(=O)OC[C@@H]2CCCO2)c3cccc(c3)[N+](=O)[O-])C(=O)OCC=C

Supplementary Table S2:
Detailed table of XP Docking using Schrodinger's Glide module

ZINC_ID	xLogP	ApolarDesolvation	PolarDesolvation	H Donors	H Acceptors	Charge	MolecularWeight	RotatableBonds	SMILES
ZINC08439421	4.54	1.1	-9.73	0	9	0	442.468	10	CC1=NC(=C([C@@H]([C@H]1C(=O)OC[C@@H]2CCCO2)c3cccc(c3)[N+](=O)[O-])C(=O)OCC=C)C
ZINC08439419	4.33	1	-21.17	1	11	0	509.515	11	CC1=NC(=C([C@@H](C1C(=O)OC)c2cccc(c2)[N+](=O)[O-])C(=O)OCCOc3ccc(cc3)NC(=O)C)C
ZINC08439419	4.51	0.92	-19.52	1	11	0	509.515	11	CC1=NC(=C)C([C@H](C1C(=O)OCCOc2ccc(cc2)NC(=O)C)c3cccc(c3)[N+](=O)[O-])C(=O)OC
ZINC08439419	4.51	2.32	-23.43	1	11	0	509.515	11	CC1=NC(=C)C([C@H]([C@H]1C(=O)OCCOc2ccc(cc2)NC(=O)C)c3cccc(c3)[N+](=O)[O-])C(=O)OC
ZINC08439419	4.33	0.87	-20.21	1	11	0	509.515	11	CC1=NC(=C([C@H]([C@@H]1C(=O)OCCOc2ccc(cc2)NC(=O)C)c3cccc(c3)[N+](=O)[O-])C(=O)OC)C
ZINC08439418	4.63	11.98	-29.96	1	6	0	435.52	4	Cc1ccc2c(c1)c(=O)c(co2)[C@H]3C4=C(CC(CC4=O)(C)C)NC(=C3C(=O)OC(C)C)C
ZINC08439418	4.74	1.06	-13.6	0	6	0	435.52	4	Cc1ccc2c(c1)c(=O)c(co2)[C@H]3C4C(=O)CC(C=C4N=C(C3C(=O)OC(C)C)C)(C)C
ZINC08439417	4.33	0.84	-20.73	1	11	0	509.515	11	CC1=NC(=C([C@H](C1C(=O)OC)c2cccc(c2)[N+](=O)[O-])C(=O)OCCOc3ccc(cc3)NC(=O)C)C
ZINC08439417	4.51	0.94	-20.52	1	11	0	509.515	11	CC1=NC(=C)C([C@@H](C1C(=O)OCCOc2ccc(cc2)NC(=O)C)c3cccc(c3)[N+](=O)[O-])C(=O)OC
ZINC08439417	4.51	2.08	-22.27	1	11	0	509.515	11	CC1=NC(=C)C([C@@H]([C@@H]1C(=O)OCCOc2ccc(cc2)NC(=O)C)c3cccc(c3)[N+](=O)[O-])C(=O)OC
ZINC08439416	5.07	2.35	-9.82	0	8	0	446.525	12	CCSCCOC(=O)C1[C@@H](C(=C(N=C1C)C)C(=O)OCC=C)c2cccc(c2)[N+](=O)[O-]
ZINC08439416	5.07	2.19	-10.84	0	8	0	446.525	12	CCSCCOC(=O)C1=C(N=C(C([C@H]1c2cccc(c2)[N+](=O)[O-])C(=O)OCC=C)C)C
ZINC08439416	5.25	1.24	-10.05	0	8	0	446.525	12	CCSCCOC(=O)C1[C@@H](C(C(=NC1=C)C)C(=O)OCC=C)c2cccc(c2)[N+](=O)[O-]
ZINC01013249	5.95	0.36	-12.68	2	6	0	494.418	7	Cc1ccc(c(c1)Br)NC(=O)CSc2nnc(n2c3ccccc3)Nc4ccccc4
ZINC08439415	4.63	11.98	-29.8	1	6	0	435.52	4	Cc1ccc2c(c1)c(=O)c(co2)[C@@H]3C4=C(CC(CC4=O)(C)C)NC(=C3C(=O)OC(C)C)C
ZINC08439415	4.74	1.04	-13.51	0	6	0	435.52	4	Cc1ccc2c(c1)c(=O)c(co2)[C@@H]3C4C(=O)CC(C=C4N=C(C3C(=O)OC(C)C)C)(C)C
ZINC08439414	5.07	2.17	-8.45	0	8	0	446.525	12	CCSCCOC(=O)C1[C@H](C(=C(N=C1C)C)C(=O)OCC=C)c2cccc(c2)[N+](=O)[O-]
ZINC08439414	5.07	2.02	-8.66	0	8	0	446.525	12	CCSCCOC(=O)C1=C(N=C(C([C@@H]1c2cccc(c2)[N+](=O)[O-])C(=O)OCC=C)C)C
ZINC08439414	5.25	0.9	-8.2	0	8	0	446.525	12	CCSCCOC(=O)C1[C@H](C(C(=NC1=C)C)C(=O)OCC=C)c2cccc(c2)[N+](=O)[O-]
ZINC02170517	5.42	0.14	-14.14	1	6	0	490.987	9	Cc1c(c2cc(ccc2n1C(=O)c3ccc(cc3)Cl)OC)CC(=O)NCCOCc4ccccc4
ZINC08439413	4.58	1.15	-11.95	0	8	0	466.581	12	CC1=NC(=C([C@H](C1C(=O)OCCSC)c2cccc(c2)[N+](=O)[O-])C(=O)OCCSC)C
ZINC08439413	4.76	1.32	-10.38	0	8	0	466.581	12	CC1=NC(=C)C([C@@H](C1C(=O)OCCSC)c2cccc(c2)[N+](=O)[O-])C(=O)OCCSC
ZINC08439413	4.76	2.45	-12.34	0	8	0	466.581	12	CC1=NC(=C)C([C@@H]([C@@H]1C(=O)OCCSC)c2cccc(c2)[N+](=O)[O-])C(=O)OCCSC
ZINC08439413	4.58	1.06	-10.74	0	8	0	466.581	12	CC1=NC(=C([C@@H]([C@@H]1C(=O)OCCSC)c2cccc(c2)[N+](=O)[O-])C(=O)OCCSC)C
ZINC08439412	5.63	13.39	-21.44	1	6	0	475.585	9	CCOc1ccccc1[C@H]2C3=C(CC(CC3=O)(C)C)NC(=C2C(=O)OCCOc4ccccc4)C
ZINC08439412	5.74	-0.48	-9.94	0	6	0	475.585	9	CCOc1ccccc1[C@H]2C3C(=O)CC(C=C3N=C(C2C(=O)OCCOc4ccccc4)C)(C)C
ZINC08439411	4.08	1.03	-13.35	0	8	0	466.581	12	CC1=NC(=C([C@@H](C1C(=O)OCCSC)c2ccccc2[N+](=O)[O-])C(=O)OCCSC)C
ZINC08439411	4.26	1.04	-12.73	0	8	0	466.581	12	CC1=NC(=C)C([C@H](C1C(=O)OCCSC)c2ccccc2[N+](=O)[O-])C(=O)OCCSC
ZINC08439411	4.26	2.38	-14.96	0	8	0	466.581	12	CC1=NC(=C)C([C@H]([C@H]1C(=O)OCCSC)c2ccccc2[N+](=O)[O-])C(=O)OCCSC
ZINC08439411	4.08	1.08	-13.86	0	8	0	466.581	12	CC1=NC(=C([C@H]([C@H]1C(=O)OCCSC)c2ccccc2[N+](=O)[O-])C(=O)OCCSC)C
ZINC02170506	6.35	0.4	-22.45	1	6	0	439.515	8	CCCCCOc1ccc(cc1)NC(=O)Cn2c3cccc4c3c(n2)-c5ccccc5C4=O
ZINC00249053	2.95	2.91	-11.96	1	5	0	299.392	5	COc1ccc(c(c1)S(=O)(=O)NC2CCCCC2)OC
ZINC08439410	4.7	1.98	-8.73	0	8	0	432.498	11	CC1=NC(=C([C@@H](C1C(=O)OCCSC)c2cccc(c2)[N+](=O)[O-])C(=O)OCC=C)C
ZINC08439410	4.88	2.01	-8.48	0	8	0	432.498	11	CC1=NC(=C)C([C@H](C1C(=O)OCC=C)c2cccc(c2)[N+](=O)[O-])C(=O)OCCSC
ZINC08439410	4.88	3.41	-16.48	0	8	0	432.498	11	CC1=NC(=C)C([C@H]([C@H]1C(=O)OCC=C)c2cccc(c2)[N+](=O)[O-])C(=O)OCCSC
ZINC08439410	4.7	2.05	-8.73	0	8	0	432.498	11	CC1=NC(=C([C@H]([C@@H]1C(=O)OCC=C)c2cccc(c2)[N+](=O)[O-])C(=O)OCCSC)C
ZINC02170492	6.03	0.99	-15.85	0	4	0	478.614	7	c1ccc(cc1)n2c(=O)c3c4c(sc3nc2SCCCC(=O)c5ccc(cc5)F)CCCC4
ZINC08439409	4.7	2.07	-8.61	0	8	0	432.498	11	CC1=NC(=C([C@H](C1C(=O)OCCSC)c2cccc(c2)[N+](=O)[O-])C(=O)OCC=C)C
ZINC08439409	4.88	2.08	-8.41	0	8	0	432.498	11	CC1=NC(=C)C([C@@H](C1C(=O)OCC=C)c2cccc(c2)[N+](=O)[O-])C(=O)OCCSC
ZINC08439409	4.7	1.96	-9.05	0	8	0	432.498	11	CC1=NC(=C([C@@H]([C@@H]1C(=O)OCC=C)c2cccc(c2)[N+](=O)[O-])C(=O)OCCSC)C
ZINC08439408	5.63	14.13	-26.4	1	6	0	475.585	9	CCOc1ccccc1[C@@H]2C3=C(CC(CC3=O)(C)C)NC(=C2C(=O)OCCOc4ccccc4)C
ZINC08439408	5.74	0.23	-13.12	0	6	0	475.585	9	CCOc1ccccc1[C@@H]2C3C(=O)CC(C=C3N=C(C2C(=O)OCCOc4ccccc4)C)(C)C
ZINC00643217	4.12	-0.7	-16.91	1	6	0	437.323	5	Cc1cc(cc(c1OCC(=O)N2CCOCC2)C)NC(=O)c3ccc(c(c3)Cl)Cl
ZINC08439407	3.35	1.33	-10.43	0	9	0	450.513	12	CC1=NC(=C([C@@H](C1C(=O)OCCOC)c2ccccc2[N+](=O)[O-])C(=O)OCCSC)C
ZINC08439407	3.53	1.33	-9.87	0	9	0	450.513	12	CC1=NC(=C)C([C@H](C1C(=O)OCCSC)c2ccccc2[N+](=O)[O-])C(=O)OCCOC
ZINC08439407	3.35	1.24	-10.07	0	9	0	450.513	12	CC1=NC(=C([C@H]([C@H]1C(=O)OCCSC)c2ccccc2[N+](=O)[O-])C(=O)OCCOC)C
ZINC08439407	3.53	2.84	-16.71	0	9	0	450.513	12	CC1=NC(=C)C([C@H]([C@H]1C(=O)OCCSC)c2ccccc2[N+](=O)[O-])C(=O)OCCOC
ZINC08439405	3.35	0.89	-12.95	0	9	0	450.513	12	CC1=NC(=C([C@H](C1C(=O)OCCOC)c2ccccc2[N+](=O)[O-])C(=O)OCCSC)C
ZINC08439405	3.53	1.05	-12.78	0	9	0	450.513	12	CC1=NC(=C)C([C@@H](C1C(=O)OCCSC)c2ccccc2[N+](=O)[O-])C(=O)OCCOC
ZINC08439405	3.53	2.17	-13.28	0	9	0	450.513	12	CC1=NC(=C)C([C@@H]([C@@H]1C(=O)OCCSC)c2ccccc2[N+](=O)[O-])C(=O)OCCOC
ZINC08439405	3.35	0.83	-13.12	0	9	0	450.513	12	CC1=NC(=C([C@@H]([C@@H]1C(=O)OCCSC)c2ccccc2[N+](=O)[O-])C(=O)OCCOC)C
ZINC06321516	5.2	11.83	-16.08	1	5	0	477.655	5	Cc1ccc(c(c1)C)NC(=O)CSC2=NC3=C([C@@H](C2C#N)c4cccs4)C(=O)CC(C3)(C)C
ZINC06321516	4.82	12.87	-16.08	1	5	0	477.655	5	Cc1ccc(c(c1)C)NC(=O)CSC2=C([C@H]([C@@H]3C(=N2)CC(CC3=O)(C)C)c4cccs4)C#N
ZINC02170483	7.35	5.14	-17.24	0	6	0	480.516	12	c1ccc(cc1)C(=O)OCC(COC(=O)c2ccccc2)(c3ccccc3)OC(=O)c4ccccc4
ZINC08439404	4.09	1	-15.51	0	9	0	478.567	13	CC1=NC(=C([C@@H](C1C(=O)OCCOC)c2ccccc2[N+](=O)[O-])C(=O)OCCSC(C)C)C
ZINC08439404	4.27	2.43	-24.55	0	9	0	478.567	13	CC1=NC(=C)C([C@H]([C@H]1C(=O)OCCSC(C)C)c2ccccc2[N+](=O)[O-])C(=O)OCCOC
ZINC08439404	4.27	1.18	-15.8	0	9	0	478.567	13	CC1=NC(=C)C([C@H](C1C(=O)OCCSC(C)C)c2ccccc2[N+](=O)[O-])C(=O)OCCOC
ZINC08439403	4.09	11.85	-9.89	0	9	0	478.567	13	CC1=NC(=C([C@H](C1C(=O)OCCOC)c2ccccc2[N+](=O)[O-])C(=O)OCCSC(C)C)C
ZINC08439403	4.27	11.65	-9.2	0	9	0	478.567	13	CC1=NC(=C)C([C@@H](C1C(=O)OCCSC(C)C)c2ccccc2[N+](=O)[O-])C(=O)OCCOC
ZINC08439403	4.27	12.49	-19.23	0	9	0	478.567	13	CC1=NC(=C)C([C@@H]([C@@H]1C(=O)OCCSC(C)C)c2ccccc2[N+](=O)[O-])C(=O)OCCOC
ZINC08439403	4.09	11.78	-9.42	0	9	0	478.567	13	CC1=NC(=C([C@@H]([C@H]1C(=O)OCCSC(C)C)c2ccccc2[N+](=O)[O-])C(=O)OCCOC)C

Supplementary Table S2:
Detailed table of XP Docking using Schrodinger's Glide module

ZINC_ID	xLogP	ApolarDesolvation	PolarDesolvation	H Donors	H Acceptors	Charge	MolecularWeight	RotatableBonds	SMILES
ZINC06321511	5.2	12.22	-16	1	5	0	477.655	5	Cc1ccc(c(c1)C)NC(=O)CSC2=NC3=C([C@H](C2C#N)c4cccs4)C(=O)CC(C3)(C)C
ZINC06321511	4.82	13.19	-16.81	1	5	0	477.655	5	Cc1ccc(c(c1)C)NC(=O)CSC2=C([C@@H]([C@H]3C(=N2)CC(CC3=O)(C)C)c4cccs4)C#N
ZINC08439401	1.58	-1.91	-40.41	2	9	1	558.618	8	COc1cc(cc(c1)OC)C(=O)NCCNC(=O)Cn2c3ccccc3[n+]4c2cc-5c(n4)-c6cccc7c6c5ccc7
ZINC02170479	7.48	2.8	-15.18	2	4	0	442.603	6	Cc1c(cccc1NC(=O)c2ccc(cc2)C(C)(C)C)NC(=O)c3ccc(cc3)C(C)(C)C
ZINC08439400	2.17	-1.65	-82.24	2	10	2	770.897	10	c1ccc2c(c1)n(c3[n+]2nc-4c(c3)-c5cccc6c5c4ccc6)CCNC(=O)CCCC(=O)NCCn7c8ccccc8[n+]9c7cc-1c(n9)-c2cccc3c2c1ccc3
ZINC02170469	6.96	3.66	-14.33	0	6	0	540.394	10	CC(C)Oc1ccc(cc1)C(=O)Oc2c(c(cc(c2I)OC(C)C)OC(C)C)C(=O)C
ZINC08439399	5.71	13.41	-17.03	1	5	0	445.559	6	Cc1ccccc1[C@H]2C3=C(CC(CC3=O)(C)C)NC(=C2C(=O)OCc4ccc(cc4)OC)C
ZINC08439399	5.82	0.6	-7.78	0	5	0	445.559	6	Cc1ccccc1[C@H]2C3C(=O)CC(C=C3N=C(C2C(=O)OCc4ccc(cc4)OC)C)(C)C
ZINC08439398	3.12	8.17	-16.29	1	5	0	301.353	0	CC1(c2ccccc2N(C3=C(C(=C(C#N)C#N)N[C@H]31)C#N)C)C
ZINC08439396	3.12	1.7	-65.19	0	5	0	427.463	3	c1ccc2c(c1)n(c3[n+]2nc-4c(c3)-c5cccc6c5c4ccc6)Cc7ccc(cc7)C(=O)[O-]
ZINC08439395	3.12	8.17	-16.28	1	5	0	301.353	0	CC1(c2ccccc2N(C3=C(C(=C(C#N)C#N)N[C@@H]31)C#N)C)C
ZINC08439394	5.71	13.37	-16.96	1	5	0	445.559	6	Cc1ccccc1[C@@H]2C3=C(CC(CC3=O)(C)C)NC(=C2C(=O)OCc4ccc(cc4)OC)C
ZINC08439394	5.82	0.37	-8.11	0	5	0	445.559	6	Cc1ccccc1[C@@H]2C3C(=O)CC(C=C3N=C(C2C(=O)OCc4ccc(cc4)OC)C)(C)C
ZINC00643196	6.22	3.37	-14.67	0	6	0	512.34	9	CC(C)Oc1cc(c(c(c1C(=O)C)OC(=O)c2ccc(cc2)OC)I)OC(C)C
ZINC08439393	1.93	7.54	-43.13	4	11	0	427.417	6	CC1=C([C@H](c2c([nH]c(nc2=O)N)N1)c3cccc(c3)[N+](=O)[O-])C(=O)OC[C@H]4CCCO4
ZINC08439392	1.93	7.57	-44.27	4	11	0	427.417	6	CC1=C([C@@H](c2c([nH]c(nc2=O)N)N1)c3cccc(c3)[N+](=O)[O-])C(=O)OC[C@H]4CCCO4
ZINC00643194	4.74	2.78	-15.78	0	6	0	456.232	7	CC(=O)c1c(cc(c(c1OC(=O)c2ccc(cc2)OC)I)OC)OC
ZINC08439391	5.69	13.63	-21.27	1	5	0	431.532	8	CCOc1cccc(c1)[C@H]2C3=C(CCCC3=O)NC(=C2C(=O)OCCc4ccccc4)C
ZINC08439391	5.81	-0.97	-9.58	0	5	0	431.532	8	CCOc1cccc(c1)[C@H]2C3C(=O)CCC=C3N=C(C2C(=O)OCCc4ccccc4)C
ZINC00643192	4.74	2.75	-15.87	0	6	0	456.232	7	CC(=O)c1c(cc(cc1OC(=O)c2ccc(c(c2)I)OC)OC)OC
ZINC08439390	1.93	7.62	-44.37	4	11	0	427.417	6	CC1=C([C@H](c2c([nH]c(nc2=O)N)N1)c3cccc(c3)[N+](=O)[O-])C(=O)OC[C@@H]4CCCO4
ZINC08439389	1.93	7.64	-44	4	11	0	427.417	6	CC1=C([C@@H](c2c([nH]c(nc2=O)N)N1)c3cccc(c3)[N+](=O)[O-])C(=O)OC[C@@H]4CCCO4
ZINC00643190	5.56	2.4	-12.43	0	5	0	466.271	5	CC(C)Oc1ccc(cc1I)c2cc(=O)c3c(o2)cc(cc3OC)OC
ZINC08439388	5.69	14.16	-29.58	1	5	0	431.532	8	CCOc1cccc(c1)[C@@H]2C3=C(CCCC3=O)NC(=C2C(=O)OCCc4ccccc4)C
ZINC08439388	5.81	-0.85	-12.23	0	5	0	431.532	8	CCOc1cccc(c1)[C@@H]2C3C(=O)CCC=C3N=C(C2C(=O)OCCc4ccccc4)C
ZINC00628953	5.51	1.18	-10.37	1	5	0	426.314	3	Cc1cc2c(cc1C)n3c(n2)NC(=C([C@@H]3c4cccc(c4)Br)C(=O)OC)C
ZINC08439387	7.34	5.23	-24.52	0	10	0	622.626	9	COc1ccc(cc1)c2cc(=O)c3c(cc(c(c3o2)c4cc(ccc4OC)c5cc(=O)c6c(o5)cc(cc6OC)OC)OC)OC
ZINC02170457	7.04	3	-11.07	0	5	0	522.379	7	CC(C)Oc1ccc(cc1)c2cc(=O)c3c(cc(c(c3o2)I)OC(C)C)OC(C)C
ZINC08439386	2.25	-1.18	-25.44	2	10	0	444.469	6	CC1=C([C@H](n2c(cc(=O)[nH]c2=S)N1)c3ccccc3[N+](=O)[O-])C(=O)OC[C@H]4CCCO4
ZINC08439385	-1.01	5.74	-13.5	0	9	0	311.305	3	Cn1c(c(c(=O)n(c1=O)C)C(=C(C#N)C#N)C#N)/N=C/N(C)C
ZINC08439384	2.25	-1.71	-22.45	2	10	0	444.469	6	CC1=C([C@@H](n2c(cc(=O)[nH]c2=S)N1)c3ccccc3[N+](=O)[O-])C(=O)OC[C@H]4CCCO4
ZINC08439383	4.91	0.18	-12.98	0	5	0	476.411	7	CC1=NC2=C([C@@H](C1C(=O)OCCOC(C)C)c3ccccc3Br)C(=O)CC(C2)(C)C
ZINC08439383	5.09	0.66	-13.39	0	5	0	476.411	7	CC(C)OCCOC(=O)C1[C@H](C2C(=NC1=C)CC(CC2=O)(C)C)c3ccccc3Br
ZINC08439383	5.09	1.47	-16.05	0	5	0	476.411	7	CC(C)OCCOC(=O)C1[C@H]([C@@H]2C(=NC1=C)CC(CC2=O)(C)C)c3ccccc3Br
ZINC08439383	4.91	0.4	-14.85	0	5	0	476.411	7	CC1=C([C@H]([C@H]2C(=N1)CC(CC2=O)(C)C)c3ccccc3Br)C(=O)OCCOC(C)C
ZINC08439382	2.25	-2.06	-28.12	2	10	0	444.469	6	CC1=C([C@H](n2c(cc(=O)[nH]c2=S)N1)c3ccccc3[N+](=O)[O-])C(=O)OC[C@@H]4CCCO4
ZINC08439381	1.32	5.95	-9.11	0	5	0	220.18	3	COc1ccccc1OC2=CC(=O)OC2=O
ZINC08439380	2.25	-0.82	-21.35	2	10	0	444.469	6	CC1=C([C@@H](n2c(cc(=O)[nH]c2=S)N1)c3ccccc3[N+](=O)[O-])C(=O)OC[C@@H]4CCCO4
ZINC08439379	3.17	-0.66	-20.88	1	13	0	608.648	8	CC1(O[C@H]2[C@H]3[C@@H]4[C@@H](C(=O)N4N([C@@H]([C@@H]2O1)ON3C(=O)OCc5ccccc5)C(=O)OC(C)(C)C)NC(=O)Cc6ccccc6)C
ZINC08439378	4.91	-0.04	-8.12	0	5	0	476.411	7	CC1=NC2=C([C@H](C1C(=O)OCCOC(C)C)c3ccccc3Br)C(=O)CC(C2)(C)C
ZINC08439378	5.09	0.29	-9.14	0	5	0	476.411	7	CC(C)OCCOC(=O)C1[C@@H](C2C(=NC1=C)CC(CC2=O)(C)C)c3ccccc3Br
ZINC08439378	5.09	1.38	-15.43	0	5	0	476.411	7	CC(C)OCCOC(=O)C1[C@@H]([C@H]2C(=NC1=C)CC(CC2=O)(C)C)c3ccccc3Br
ZINC08439378	4.91	0.33	-11.42	0	5	0	476.411	7	CC1=C([C@@H]([C@H]2C(=N1)CC(CC2=O)(C)C)c3ccccc3Br)C(=O)OCCOC(C)C
ZINC00628601	4.21	0.77	-14.06	1	9	0	434.452	6	CC1=C([C@H](n2c3ccccc3nc2N1)c4ccccc4[N+](=O)[O-])C(=O)OC[C@H]5CCCO5
ZINC00628603	4.21	0.09	-17	1	9	0	434.452	6	CC1=C([C@@H](n2c3ccccc3nc2N1)c4ccccc4[N+](=O)[O-])C(=O)OC[C@H]5CCCO5
ZINC08439377	3.17	-1.73	-16.24	1	13	0	608.648	8	CC1(O[C@@H]2[C@H]3[C@@H]4[C@@H](C(=O)N4N([C@@H]([C@@H]2O1)ON3C(=O)OCc5ccccc5)C(=O)OC(C)(C)C)NC(=O)Cc6ccccc6)C
ZINC00628605	4.21	0.49	-19.16	1	9	0	434.452	6	CC1=C([C@H](n2c3ccccc3nc2N1)c4ccccc4[N+](=O)[O-])C(=O)OC[C@@H]5CCCO5
ZINC00628608	4.21	0.42	-17.99	1	9	0	434.452	6	CC1=C([C@@H](n2c3ccccc3nc2N1)c4ccccc4[N+](=O)[O-])C(=O)OC[C@@H]5CCCO5
ZINC08439375	6.06	2.49	-10.31	0	6	0	473.569	8	CC1=NC2=C([C@@H](C1C(=O)OCCc3ccccc3)c4ccc(cc4)C(=O)OC)C(=O)CC(C2)(C)C
ZINC08439375	6.24	2.92	-11.23	0	6	0	473.569	8	CC1(CC2=NC(=C)C([C@H](C2C(=O)C1)c3ccc(cc3)C(=O)OC)C(=O)OCCc4ccccc4)C
ZINC08439375	6.24	3.9	-13.25	0	6	0	473.569	8	CC1(CC2=NC(=C)C([C@H]([C@H]2C(=O)C1)c3ccc(cc3)C(=O)OC)C(=O)OCCc4ccccc4)C
ZINC08439375	6.06	2.76	-13.49	0	6	0	473.569	8	CC1=C([C@H]([C@@H]2C(=N1)CC(CC2=O)(C)C)c3ccc(cc3)C(=O)OC)C(=O)OCCc4ccccc4
ZINC08439372	3.63	-0.12	-26.89	1	10	0	552.587	7	CC1=C[C@@H]2N(O[C@H]1[C@@H]3[C@@H](C(=O)N3N2C(=O)OCc4ccccc4)NC(=O)Cc5ccccc5)C(=O)c6ccccc6
ZINC08439370	3.63	-0.14	-24.48	1	10	0	552.587	7	CC1=C[C@H]2N(O[C@@H]1[C@@H]3[C@@H](C(=O)N3N2C(=O)OCc4ccccc4)NC(=O)Cc5ccccc5)C(=O)c6ccccc6
ZINC00381730	2	5.67	-16.53	0	4	0	218.208	1	COc1c2c(ccc(=O)o2)cc3c1OCC3
ZINC08439369	6.06	2.47	-10.38	0	6	0	473.569	8	CC1=NC2=C([C@H](C1C(=O)OCCc3ccccc3)c4ccc(cc4)C(=O)OC)C(=O)CC(C2)(C)C
ZINC08439369	6.24	2.97	-11.28	0	6	0	473.569	8	CC1(CC2=NC(=C)C([C@@H](C2C(=O)C1)c3ccc(cc3)C(=O)OC)C(=O)OCCc4ccccc4)C
ZINC08439369	6.24	3.94	-19	0	6	0	473.569	8	CC1(CC2=NC(=C)C([C@@H]([C@@H]2C(=O)C1)c3ccc(cc3)C(=O)OC)C(=O)OCCc4ccccc4)C
ZINC08439369	6.06	2.85	-13.78	0	6	0	473.569	8	CC1=C([C@@H]([C@@H]2C(=N1)CC(CC2=O)(C)C)c3ccc(cc3)C(=O)OC)C(=O)OCCc4ccccc4
ZINC08439368	4.77	-3.41	-34.2	1	9	0	605.709	6	CC1(CC2=C(C(C3=C(O2)CC(CC3=O)(C)C)c4ccc(cc4)OC[C@H](CN5C(=O)c6ccccc6S5(=O)=O)O)C(=O)C1)C
ZINC08439367	5.56	2.46	-19.51	0	6	0	438.52	9	CC1=CC(=O)O[C@H]2[C@H]1C=CC(=C2)OCCCCCCOC3=C[C@@H]4[C@@H](C=C3)C(=CC(=O)O4)C

Supplementary Table S2:
Detailed table of XP Docking using Schrodinger's Glide module

ZINC_ID	xLogP	ApolarDesolvation	PolarDesolvation	H Donors	H Acceptors	Charge	MolecularWeight	RotatableBonds	SMILES
ZINC08439366	4.77	-3.91	-33.84	1	9	0	605.709	6	CC1(CC2=C(C(C3=C(O2)CC(CC3=O)(C)C)c4ccc(cc4)OC[C@@H](CN5C(=O)c6ccccc6S5(=O)=O)O)C(=O)C1)C
ZINC06147567	3.74	5.66	-9.59	1	3	0	267.394	3	Cc1ccc(c(c1)C)S(=O)(=O)NC2CCCCC2
ZINC08439365	5.56	13.48	-19.98	0	6	0	438.52	9	CC1=CC(=O)O[C@H]2[C@H]1C=CC(=C2)OCCCCCCOC3=C[C@H]4[C@@H](C=C3)C(=CC(=O)O4)C
ZINC08439364	4.97	1.2	-24.23	1	7	0	462.509	3	Cc1c(c(=O)n(n1C)c2ccccc2)Nc3ccc4c5c3C(=O)c6ccccc6-c5cc(=O)n4C
ZINC08439362	4.23	-7.16	-14.18	1	6	0	427.913	3	c1ccc(c(c1)N2CCN(CC2)S(=O)(=O)c3ccc4c5c3cccc5C(=O)N4)Cl
ZINC08439361	5.56	13.42	-19.93	0	6	0	438.52	9	CC1=CC(=O)O[C@H]2[C@H]1C=CC(=C2)OCCCCCCOC3=C[C@@H]4[C@H](C=C3)C(=CC(=O)O4)C
ZINC08439360	4.47	-5.12	-14.87	1	6	0	461.465	4	c1cc(cc(c1)N2CCN(CC2)S(=O)(=O)c3ccc4c5c3cccc5C(=O)N4)C(F)(F)F
ZINC08439359	5.56	13.18	-18.91	0	6	0	438.52	9	CC1=CC(=O)O[C@H]2[C@H]1C=CC(=C2)OCCCCCCOC3=C[C@H]4[C@H](C=C3)C(=CC(=O)O4)C
ZINC08439357	6.57	2.7	-19.43	0	6	0	466.574	11	CC1=CC(=O)O[C@H]2[C@H]1C=CC(=C2)OCCCCCCCCOC3=C[C@@H]4[C@@H](C=C3)C(=CC(=O)O4)C
ZINC08439356	3.72	-7.48	-16.35	2	7	0	468.641	9	Cc1cc(c(c(c1)C)S(=O)(=O)NCCN(CCO)S(=O)(=O)c2c(cc(cc2C)C)C)C
ZINC08439355	2.25	-5.33	-55.51	2	8	-1	462.507	7	CCN1c2ccc(c3c2c(ccc3)C1=O)S(=O)(=O)N[C@H](Cc4cc5ccccc5[nH]4)C(=O)[O-]
ZINC08439354	6.57	15.04	-20.07	0	6	0	466.574	11	CC1=CC(=O)O[C@H]2[C@H]1C=CC(=C2)OCCCCCCCCOC3=C[C@H]4[C@@H](C=C3)C(=CC(=O)O4)C
ZINC08439353	2.25	-5.31	-50.95	2	8	-1	462.507	7	CCN1c2ccc(c3c2c(ccc3)C1=O)S(=O)(=O)N[C@@H](Cc4cc5ccccc5[nH]4)C(=O)[O-]
ZINC08439351	6.57	14.98	-19.99	0	6	0	466.574	11	CC1=CC(=O)O[C@H]2[C@H]1C=CC(=C2)OCCCCCCCCOC3=C[C@@H]4[C@H](C=C3)C(=CC(=O)O4)C
ZINC08439350	6.51	-3.23	-12.29	2	5	0	467.612	7	CC(C)(C)c1ccc(cc1)S(=O)(=O)Nc2cc(c(c3c2cccc3)O)SCc4ccco4
ZINC08439349	6.57	14.75	-18.96	0	6	0	466.574	11	CC1=CC(=O)O[C@H]2[C@H]1C=CC(=C2)OCCCCCCCCOC3=C[C@H]4[C@H](C=C3)C(=CC(=O)O4)C
ZINC08439348	4.63	-3.91	-19.23	2	8	0	475.555	6	c1ccc(cc1)n2c(nnn2)Sc3cc(c4ccccc4c3O)NS(=O)(=O)c5ccccc5
ZINC08439347	8.08	0.95	-5.3	0	2	0	440.722	5	CC(C)Sc1c2c(cc(n1)c3cc4c(c(n3)SC(C)C)CCCC4)CCCC2
ZINC08439345	9	16.07	-7.73	1	3	0	436.599	9	CCCCc1ccc(cc1)NC2=C/C(=N/c3ccc(cc3)CCCC)/c4ccccc4C2=O
ZINC08439345	9	16.59	-7.72	1	3	0	436.599	9	CCCCc1ccc(cc1)NC2=C/C(=N\c3ccc(cc3)CCCC)/c4ccccc4C2=O
ZINC08439344	3.41	-0.99	-12.17	0	6	0	491.157	5	CC(=O)N(c1ccc(cc1)OC(=O)C)S(=O)(=O)c2cc(ccc2Br)Br
ZINC08439343	4.63	0.01	-8.88	0	5	0	434.33	6	CCCOC(=O)C1[C@@H](C2=C(CCCC2=O)N=C1C)c3cc(ccc3OC)Br
ZINC08439343	4.63	-0.35	-12.83	0	5	0	434.33	6	CCCOC(=O)C1=C(N=C2CCCC(=O)C2[C@H]1c3cc(ccc3OC)Br)C
ZINC08439342	3.14	-1.54	-61.29	1	7	-1	448.473	8	CC(=O)Oc1c2ccccc2c(cc1SCC(=O)[O-])NS(=O)(=O)c3ccc(cc3)F
ZINC08439341	4.63	0.01	-8.73	0	5	0	434.33	6	CCCOC(=O)C1[C@H](C2=C(CCCC2=O)N=C1C)c3cc(ccc3OC)Br
ZINC08439341	4.63	-0.27	-12.28	0	5	0	434.33	6	CCCOC(=O)C1=C(N=C2CCCC(=O)C2[C@@H]1c3cc(ccc3OC)Br)C
ZINC08439340	3.85	-7.23	-52.11	2	6	-1	450.084	4	c1cc(cc(c1)S(=O)(=O)Nc2cc(c(c(c2)Br)O)Br)C(=O)[O-]
ZINC08439339	5.46	-1.4	-56.85	1	8	-1	480.518	9	CCCc1c(c2cc(c3ccccc3c2o1)NS(=O)(=O)c4ccc(cc4)C(=O)[O-])C(=O)OCC
ZINC08439338	3.8	-2.25	-60.09	1	9	-1	482.49	9	Cc1c(c2cc(c3ccccc3c2o1)NS(=O)(=O)c4ccc(cc4)C(=O)[O-])C(=O)OCCOC
ZINC08439336	5.98	-2.24	-52.23	1	6	-1	440.541	6	CCC(C)(C)[C@H]1CCc2c(c3cc(ccc3o2)NS(=O)(=O)c4ccc(cc4)C(=O)[O-])C1
ZINC00622385	5.17	-1.66	-10.61	2	5	0	489.309	7	CCOc1cc(cc(c1OCc2ccccc2)I)C(=O)Nc3ccc(cc3)O
ZINC08439335	5.98	-2.26	-52.56	1	6	-1	440.541	6	CCC(C)(C)[C@@H]1CCc2c(c3cc(ccc3o2)NS(=O)(=O)c4ccc(cc4)C(=O)[O-])C1
ZINC00622382	4.78	-1.67	-12.08	2	5	0	475.282	6	COc1cc(cc(c1OCc2ccccc2)I)C(=O)Nc3cccc(c3)O
ZINC08439334	4.38	10.08	-55.64	1	8	-1	452.464	7	CCOC(=O)c1c(oc2c1cc(c3c2cccc3)NS(=O)(=O)c4ccc(cc4)C(=O)[O-])C
ZINC08439334	4.38	10.16	-110.37	0	8	-2	451.456	7	CCOC(=O)c1c(oc2c1cc(c3c2cccc3)[N-]S(=O)(=O)c4ccc(cc4)C(=O)[O-])C
ZINC08439332	4.49	-1.79	-56.07	1	8	-1	464.475	8	Cc1c(c2cc(ccc2o1)NS(=O)(=O)c3ccc(cc3)C(=O)[O-])C(=O)OCc4ccccc4
ZINC00622380	5.29	0.45	-9.12	1	5	0	489.309	7	COc1ccccc1NC(=O)c2cc(c(c(c2)I)OCc3ccccc3)OC
ZINC05396721	5.71	12.08	-10.45	0	5	0	431.532	6	CC1=NC2=C([C@@H](C1C(=O)OC)c3cccc(c3)OCc4ccccc4)C(=O)CC(C2)(C)C
ZINC05396721	5.89	11.87	-11.23	0	5	0	431.532	6	CC1(CC2=NC(=C)C([C@H](C2C(=O)C1)c3cccc(c3)OCc4ccccc4)C(=O)OC)C
ZINC05396721	5.89	12.71	-18.76	0	5	0	431.532	6	CC1(CC2=NC(=C)C([C@H]([C@H]2C(=O)C1)c3cccc(c3)OCc4ccccc4)C(=O)OC)C
ZINC05396721	5.71	12.07	-13.5	0	5	0	431.532	6	CC1=C([C@H]([C@H]2C(=N1)CC(CC2=O)(C)C)c3cccc(c3)OCc4ccccc4)C(=O)OC
ZINC08439331	4.01	9.16	-58.01	1	8	-1	438.437	6	Cc1c(c2cc(c3ccccc3c2o1)NS(=O)(=O)c4ccc(cc4)C(=O)[O-])C(=O)OC
ZINC08439331	4.01	9.24	-109.57	0	8	-2	437.429	6	Cc1c(c2cc(c3ccccc3c2o1)[N-]S(=O)(=O)c4ccc(cc4)C(=O)[O-])C(=O)OC
ZINC08439330	4.63	-1.01	-21.84	0	6	0	452.919	5	COc1ccc(cc1)N(C(=O)c2ccccc2Cl)S(=O)(=O)c3cccc4c3nccc4
ZINC00622370	5.41	0.91	-9.41	1	4	0	426.31	6	Cc1ccccc1NC(=O)c2cc(c(c(c2)Br)OCc3ccccc3)OC
ZINC05396702	5.71	12.08	-10.44	0	5	0	431.532	6	CC1=NC2=C([C@H](C1C(=O)OC)c3cccc(c3)OCc4ccccc4)C(=O)CC(C2)(C)C
ZINC05396702	5.89	11.86	-11.45	0	5	0	431.532	6	CC1(CC2=NC(=C)C([C@@H](C2C(=O)C1)c3cccc(c3)OCc4ccccc4)C(=O)OC)C
ZINC05396702	5.89	12.79	-10.9	0	5	0	431.532	6	CC1(CC2=NC(=C)C([C@@H]([C@@H]2C(=O)C1)c3cccc(c3)OCc4ccccc4)C(=O)OC)C
ZINC05396702	5.71	12.05	-13.53	0	5	0	431.532	6	CC1=C([C@@H]([C@@H]2C(=N1)CC(CC2=O)(C)C)c3cccc(c3)OCc4ccccc4)C(=O)OC
ZINC08439329	4.67	-1.01	-17.69	0	6	0	452.919	5	COc1ccc(cc1)N(C(=O)c2ccc(cc2)Cl)S(=O)(=O)c3cccc4c3nccc4
ZINC02170396	5.13	2.74	-6.87	1	4	0	432.236	7	CCCOc1c(cc(cc1Br)C(=O)Nc2ccccc2C(F)(F)F)OC
ZINC08439328	6.64	0.15	-13.45	1	6	0	477.582	7	CCOC(=O)c1c2cc(ccc2oc1c3ccccc3)NS(=O)(=O)c4c(c(cc(c4C)C)C)C
ZINC08439327	4.84	3.55	-17.1	0	7	0	469.578	9	CC[C@H](C)OC(=O)C1[C@@H](C2=C(CC(CC2=O)(C)C)N=C1C)c3ccc(c(c3)OCC)OC(=O)C
ZINC08439327	4.84	3.53	-17.85	0	7	0	469.578	9	CC[C@H](C)OC(=O)C1=C(N=C2CC(CC(=O)C2[C@H]1c3ccc(c(c3)OCC)OC(=O)C)(C)C)C
ZINC08439327	5.02	2.66	-15.88	0	7	0	469.578	9	CC[C@H](C)OC(=O)C1[C@@H](C2C(=NC1=C)CC(CC2=O)(C)C)c3ccc(c(c3)OCC)OC(=O)C
ZINC08439326	7.56	-5.28	-12.07	2	5	0	498.695	6	Cc1cc(cc(c1C)S(=O)(=O)Nc2ccc(c(c2)Sc3nc4ccccc4s3)O)C(C)(C)C
ZINC08439325	4.84	3.59	-13.55	0	7	0	469.578	9	CC[C@@H](C)OC(=O)C1[C@@H](C2=C(CC(CC2=O)(C)C)N=C1C)c3ccc(c(c3)OCC)OC(=O)C
ZINC08439325	4.84	3.57	-16.29	0	7	0	469.578	9	CC[C@@H](C)OC(=O)C1=C(N=C2CC(CC(=O)C2[C@H]1c3ccc(c(c3)OCC)OC(=O)C)(C)C)C
ZINC08439325	5.02	2.64	-13.79	0	7	0	469.578	9	CC[C@@H](C)OC(=O)C1[C@@H](C2C(=NC1=C)CC(CC2=O)(C)C)c3ccc(c(c3)OCC)OC(=O)C
ZINC08439324	6.17	-5.14	-11.34	2	4	0	441.961	5	c1ccc(cc1)S(=O)(=O)Nc2cc(c(c3c2cccc3)O)Sc4ccc(cc4)Cl
ZINC08439323	6.76	-3.93	-13.14	2	4	0	449.597	5	Cc1ccc(cc1)S(=O)(=O)Nc2cc(c(c3c2cccc3)O)Sc4ccc(cc4C)C

Supplementary Table S2:
Detailed table of XP Docking using Schrodinger's Glide module

ZINC_ID	xLogP	ApolarDesolvation	PolarDesolvation	H Donors	H Acceptors	Charge	MolecularWeight	RotatableBonds	SMILES
ZINC03765149	5	-2.64	-58.01	1	8	-1	467.523	9	CCOc1ccccc1N(CC(=O)Nc2ccccc2C(=O)[O-])S(=O)(=O)c3ccc(cc3)C
ZINC02987093	3.27	-3.62	-22.35	0	1	0	406.504	7	CCOc1ccccc1N(CC(=O)NCCc2ccc(cc2)OC)S(=O)(=O)C
ZINC08439322	4.84	3.58	-12.85	0	7	0	469.578	9	CC[C@H](C)OC(=O)C1[C@H](C2=C(CC(CC2=O)(C)C)N=C1C)c3ccc(c(c3)OCC)OC(=O)C
ZINC08439322	4.84	3.66	-17.23	0	7	0	469.578	9	CC[C@H](C)OC(=O)C1=C(N=C2CC(CC(=O)C2[C@@H]1c3ccc(c(c3)OCC)OC(=O)C)(C)C)C
ZINC08439322	5.02	2.74	-14.64	0	7	0	469.578	9	CC[C@H](C)OC(=O)C1[C@H](C2C(=NC1=C)CC(CC2=O)(C)C)c3ccc(c(c3)OCC)OC(=O)C
ZINC08439321	6.32	-4.25	-13.3	2	4	0	435.57	5	Cc1ccc(c(c1)C)Sc2cc(c3ccccc3c2O)NS(=O)(=O)c4ccccc4
ZINC04174231	5.64	10.69	-52.41	1	7	-1	471.942	7	Cc1ccc(cc1)S(=O)(=O)N(CC(=O)Nc2ccccc2C(=O)[O-])c3cccc(c3C)Cl
ZINC08439320	3.74	-4.42	-58.06	2	6	-1	420.463	6	Cc1cc(ccc1S(=O)(=O)Nc2cc(c(c3c2cccc3)O)SCC(=O)[O-])F
ZINC08439319	4.84	3.58	-12.7	0	7	0	469.578	9	CC[C@@H](C)OC(=O)C1[C@H](C2=C(CC(CC2=O)(C)C)N=C1C)c3ccc(c(c3)OCC)OC(=O)C
ZINC08439319	4.84	3.65	-17.03	0	7	0	469.578	9	CC[C@@H](C)OC(=O)C1=C(N=C2CC(CC(=O)C2[C@@H]1c3ccc(c(c3)OCC)OC(=O)C)(C)C)C
ZINC08439319	5.02	2.73	-14.52	0	7	0	469.578	9	CC[C@@H](C)OC(=O)C1[C@H](C2C(=NC1=C)CC(CC2=O)(C)C)c3ccc(c(c3)OCC)OC(=O)C
ZINC08439318	4.64	-0.27	-15.66	1	6	0	427.453	5	Cc1cc(ccc1S(=O)(=O)Nc2cc3c(c(oc3c4c2cccc4)C)C(=O)OC)F
ZINC08439317	5.66	-2.87	-20.83	1	7	0	488.993	9	CCOc1ccc(cc1)NC(=O)CN(c2ccc(c(c2)Cl)OC)S(=O)(=O)c3ccc(cc3)C
ZINC06270540	2.27	1.91	-47.82	2	7	-1	353.43	5	c1cc(sc1)S(=O)(=O)[N-]c2ccc(c(c2)Sc3[nH]ncn3)O
ZINC08439316	5.58	14.81	-16.72	1	4	0	435.589	6	Cc1ccc(s1)[C@H]2C3=C(CC(CC3=O)(C)C)NC(=C2C(=O)OCCc4ccccc4)C
ZINC08439316	5.7	-0.58	-8.02	0	4	0	435.589	6	Cc1ccc(s1)[C@H]2C3C(=O)CC(C=C3N=C(C2C(=O)OCCc4ccccc4)C)(C)C
ZINC08439315	2.91	-3.06	-25.6	1	8	0	436.53	10	Cc1ccc(c(c1)N(CC(=O)NCCc2ccc(c(c2)OC)OC)S(=O)(=O)C)OC
ZINC08439312	5.58	14.81	-16.83	1	4	0	435.589	6	Cc1ccc(s1)[C@@H]2C3=C(CC(CC3=O)(C)C)NC(=C2C(=O)OCCc4ccccc4)C
ZINC08439312	5.7	-0.48	-10.39	0	4	0	435.589	6	Cc1ccc(s1)[C@@H]2C3C(=O)CC(C=C3N=C(C2C(=O)OCCc4ccccc4)C)(C)C
ZINC08439311	4.43	-0.93	-17.02	1	7	0	471.506	8	Cc1cc(ccc1S(=O)(=O)Nc2cc3c(c(oc3c4c2cccc4)C)C(=O)OCCOC)F
ZINC02986980	4.39	-2.96	-25.93	1	6	0	404.532	9	CC(C)c1ccc(cc1)N(CC(=O)NCCc2ccc(cc2)OC)S(=O)(=O)C
ZINC00969280	4.46	-3.55	-14.2	2	5	0	436.278	5	c1ccc(c(c1)/C=N/NC(=O)CSc2[nH]c3ccccc3n2)I
ZINC08439310	4.55	-4.9	-16.13	2	6	0	452.504	6	Cc1cc(ccc1S(=O)(=O)Nc2ccc(cc2)NS(=O)(=O)c3ccc(cc3C)F)F
ZINC04174238	5.55	-3.12	-21.22	1	6	0	468.619	7	COc1ccc(cc1)NC(=O)CN(c2ccc(cc2)C34CC5CC(C3)CC(C5)C4)S(=O)(=O)C
ZINC08439309	3.85	-2.44	-23.81	1	8	0	490.537	8	Cc1c(c2cc(c3ccccc3c2o1)NS(=O)(=O)c4cccc5c4nccc5)C(=O)OCCOC
ZINC08439308	5.71	1.03	-13.39	0	5	0	454.942	3	c1ccc(cc1)n2cc(c(n2)c3ccc(cc3)Cl)/C=c\4/c(=O)n5c6ccccc6nc5s4
ZINC08439307	3.51	1.46	-13.91	0	7	0	433.457	6	CCOC(=O)c1c(oc2c1cc(cc2)N(C(=O)C)S(=O)(=O)c3ccc(cc3C)F)C
ZINC01013182	4	-0.21	-11.39	0	6	0	480.723	4	c1cc(ccc1C(=O)CN2C(=O)/C(=C/c3cc4c(cc3Br)OCO4)/SC2=O)Cl
ZINC08439306	4.91	1.75	-12.66	0	7	0	461.511	8	CCCC(=O)N(c1ccc2c(c1)c(c(o2)C)C(=O)OCC)S(=O)(=O)c3ccc(cc3C)F
ZINC01013180	3.87	0.37	-11.55	0	6	0	436.272	4	c1cc(ccc1C(=O)CN2C(=O)/C(=C/c3cc4c(cc3Cl)OCO4)/SC2=O)Cl
ZINC09042127	3.65	2.5	-43.63	2	7	1	439.532	9	CC[NH+](CC)CCN1[C@H](/C(=C(/c2ccccc2)\O)/C(=O)C1=O)c3cc(ccc3OC)OC
ZINC08439304	4.6	1.2	-14.09	0	6	0	431.485	6	CCCC(=O)N(c1ccc2c(c1)c(c(o2)C)C(=O)C)S(=O)(=O)c3ccc(cc3C)F
ZINC02853302	4.21	10.21	-17.4	0	7	0	455.513	6	Cc1c(c2cc(ccc2o1)N(C(=O)c3ccccc3)S(=O)(=O)c4cccs4)C(=O)OC
ZINC02170296	5.66	-3.58	-29.83	2	5	0	480.387	5	Cc1cccc(c1C)c2nc3cc(ccc3o2)NC(=S)NC(=O)c4cccc(c4)Br
ZINC08439303	5.71	0.14	-15.11	0	5	0	433.529	4	Cc1cc(c(cc1C)S(=O)(=O)N(c2ccc3c(c2)cc(o3)C)C(=O)c4ccccc4)C
ZINC06270551	4.18	7.51	-69.18	1	6	-1	436.9	5	c1ccc(c(c1)C(=O)Nc2ccc(cc2)Cl)[N-]S(=O)(=O)c3cccc4c3ncccc4
ZINC06270551	4.18	8.08	-46.26	2	6	0	437.908	5	c1ccc(c(c1)C(=O)Nc2ccc(cc2)Cl)[N-]S(=O)(=O)c3cccc4c3[nH+]ccc4
ZINC00622060	6.51	9.08	-16.13	1	6	0	489.325	5	COc1ccc(cc1NC(=O)c2ccc(o2)c3ccc(cc3)Br)c4nc5ccccc5o4
ZINC09042124	3.65	1.19	-40.95	2	7	1	439.532	9	CC[NH+](CC)CCN1[C@@H](/C(=C(/c2ccccc2)\O)/C(=O)C1=O)c3cc(ccc3OC)OC
ZINC02170293	5.91	0.76	-6.86	1	3	0	439.136	3	Cc1cc(cc(c1NC(=O)c2ccc(o2)c3ccc(c(c3)Cl)Cl)C)Br
ZINC08439301	5.44	-0.24	-16.82	0	7	0	492.94	5	COc1cc(ccc1OCc2cc3c(cc2Cl)OCO3)/C=c\4/c(=O)n5c6ccccc6nc5s4
ZINC08439300	5.99	0.32	-14.8	1	6	0	455.507	6	CC(C)(C)c1c(c2cc(c3ccccc3c2o1)NS(=O)(=O)c4ccc(cc4)F)C(=O)OC
ZINC08439299	7.44	0.77	-12.83	0	5	0	531.468	4	Cc1ccc2c(c1C)nc3n2c(=O)/c(=C\c4cn(nc4c5ccccc5)Cc6ccc(cc6Cl)Cl)/s3
ZINC08439298	6.25	-0.38	-15.81	1	7	0	481.57	8	CCOc1ccc(cc1)S(=O)(=O)Nc2cc3c(c(oc3c4c2cccc4)C(C)(C)C)C(=O)OC
ZINC00622047	2.19	-3.67	-22.45	2	7	0	434.521	6	c1ccc(cc1)C(=O)N2CCN(CC2)c3ccc(cc3)NC(=S)NC(=O)c4ccco4
ZINC08439297	1.38	-10.73	-15	0	9	0	484.577	4	c1cc2c(cc(c3c2c(c1)C(=O)S3)S(=O)(=O)N4CCOCC4)S(=O)(=O)N5CCOCC5
ZINC02170288	5.36	9.61	-21.8	3	5	0	433.558	6	CCc1ccc(cc1)C(=O)NC(=S)Nc2ccc(c(c2)c3nc4ccccc4s3)O
ZINC08439296	5.86	12.71	-58.13	0	6	-1	488.516	7	Cc1c(c2cc(c3ccccc3c2o1)[N-]S(=O)(=O)c4ccc(cc4)F)C(=O)OCc5ccccc5
ZINC08439296	5.86	12.64	-15.57	1	6	0	489.524	7	Cc1c(c2cc(c3ccccc3c2o1)NS(=O)(=O)c4ccc(cc4)F)C(=O)OCc5ccccc5
ZINC01013175	4.87	0.7	-21.57	1	9	0	581.046	9	CCOc1cc(ccc1OCC(=O)Nc2ccccc2C)/C=C\3/C(=O)N(C(=O)S3)Cc4cc5c(cc4Cl)OCO5
ZINC08439294	4.64	-1.36	-14.11	1	7	0	451.5	8	Cc1c(c2cc(ccc2o1)NS(=O)(=O)c3ccc(cc3)OC)C(=O)OCc4ccccc4
ZINC08439293	4.47	-0.44	-11.97	0	5	0	431.311	4	CCOc1cc(c(cc1OC)/C=c/2\c(=O)n3c4ccccc4nc3s2)Br
ZINC02170277	5.56	0.26	-11.58	0	7	0	544.412	7	COc1cc(ccc1OCc2ccc(cc2)Cl)/C=C/3\C(=O)N(C(=O)S3)Cc4cc5c(cc4Cl)OCO5
ZINC08439292	8.05	-0.27	-13.13	1	4	0	475.654	4	Cc1cc(c(cc1C)S(=O)(=O)Nc2cc3c4c(oc3c5c2cccc5)CC[C@H](C4)C(C)(C)C)C
ZINC08439291	8.05	-0.27	-13.05	1	4	0	475.654	4	Cc1cc(c(cc1C)S(=O)(=O)Nc2cc3c4c(oc3c5c2cccc5)CC[C@@H](C4)C(C)(C)C)C
ZINC08439290	6.5	0.21	-13.56	0	7	0	623.308	7	COc1cc(cc(c1OCc2ccc(cc2)Cl)Br)/C=C/3\C(=O)N(C(=O)S3)Cc4cc5c(cc4Cl)OCO5
ZINC08439289	6.48	-0.74	-22.03	1	5	0	461.583	3	Cc1cc(c(cc1C)S(=O)(=O)Nc2cc3c4c(oc3c5c2cccc5)CC(CC4=O)(C)C)C
ZINC08439287	5.59	-1.53	-22.98	1	5	0	433.529	3	Cc1cc(c(cc1C)S(=O)(=O)Nc2cc3c4c(oc3c5c2cccc5)CCCC4=O)C
ZINC02170255	6.17	0.18	-10.17	0	7	0	578.857	7	COc1cc(ccc1OCc2ccc(cc2Cl)Cl)/C=C/3\C(=O)N(C(=O)S3)Cc4cc5c(cc4Cl)OCO5
ZINC02497152	4.99	-1.71	-13.53	1	5	0	450.354	4	Cc1cc(c(cc1C)S(=O)(=O)Nc2cc3c(c(oc3c(c2)Br)C)C(=O)C)C
ZINC02170251	5.25	0.14	-8.37	0	6	0	479.941	6	c1ccc(cc1)COc2ccccc2/C=C/3\C(=O)N(C(=O)S3)Cc4cc5c(cc4Cl)OCO5

Supplementary Table S2:
Detailed table of XP Docking using Schrodinger's Glide module

ZINC_ID	xLogP	ApolarDesolvation	PolarDesolvation	H Donors	H Acceptors	Charge	MolecularWeight	RotatableBonds	SMILES
ZINC08439286	6.12	-2.19	-19.02	1	6	0	477.563	7	CCOC(=O)c1c2cc(c3ccccc3c2oc1c4ccccc4)NS(=O)(=O)c5cccs5
ZINC08439285	4.98	13.62	-24.71	1	4	0	457.382	6	CC1=C([C@H](C(=C(N1)SCC(=O)c2ccc(cc2)Cl)C#N)c3ccccc3Cl)C(=O)C
ZINC08439285	5.5	0.78	-11.69	0	4	0	457.382	6	CC(=O)C1[C@H](C(C(=NC1=C)SCC(=O)c2ccc(cc2)Cl)C#N)c3ccccc3Cl
ZINC08439285	5.32	0.55	-11.65	0	4	0	457.382	6	CC1=C([C@H]([C@H](C(=N1)SCC(=O)c2ccc(cc2)Cl)C#N)c3ccccc3Cl)C(=O)C
ZINC01013166	4.4	0.33	-12.33	0	7	0	475.95	7	CCOc1cc(ccc1OC(C)C)/C=C/2\C(=O)N(C(=O)S2)Cc3cc4c(cc3Cl)OCO4
ZINC08439284	4.8	-2.7	-15.99	1	6	0	430.485	3	Cc1ccc(cc1)S(=O)(=O)Nc2ccc3c4c2C(=O)c5ccccc5-c4cc(=O)n3C
ZINC01013164	4.19	-1.52	-11.29	1	7	0	468.314	5	CCOc1cc(cc(c1O)Cl)/C=C\2/C(=O)N(C(=O)S2)Cc3cc4c(cc3Cl)OCO4
ZINC08439283	5.54	9.06	-17.22	1	7	0	464.474	6	COC(=O)c1ccc(cc1)NC2=CC(=NS(=O)(=O)c3ccc(cc3)F)c4ccccc4C2=O
ZINC08439282	7.23	-1.19	-11.5	1	4	0	447.6	4	C[C@H]1CCc2c(c3cc(c4ccccc4c3o2)NS(=O)(=O)c5ccc(cc5)C(C)(C)C)C1
ZINC02170239	4.38	1	-12.91	0	7	0	536.787	6	COc1cc(cc(c1OCC#C)Br)/C=C\2/C(=O)N(C(=O)S2)Cc3cc4c(cc3Cl)OCO4
ZINC08439281	4.98	13.01	-20.76	1	4	0	457.382	6	CC1=C([C@@H](C(=C(N1)SCC(=O)c2ccc(cc2)Cl)C#N)c3ccccc3Cl)C(=O)C
ZINC08439281	5.5	0.56	-11.5	0	4	0	457.382	6	CC(=O)C1[C@@H](C(C(=NC1=C)SCC(=O)c2ccc(cc2)Cl)C#N)c3ccccc3Cl
ZINC08439281	5.32	0.43	-9.95	0	4	0	457.382	6	CC1=C([C@@H]([C@@H](C(=N1)SCC(=O)c2ccc(cc2)Cl)C#N)c3ccccc3Cl)C(=O)C
ZINC08439280	7.23	-1.19	-11.53	1	4	0	447.6	4	C[C@@H]1CCc2c(c3cc(c4ccccc4c3o2)NS(=O)(=O)c5ccc(cc5)C(C)(C)C)C1
ZINC02170234	5.31	1.52	-13.06	0	7	0	523.994	7	Cc1cccc(c1)COc2ccc(cc2OC)/C=C\3/C(=O)N(C(=O)S3)Cc4cc5c(cc4Cl)OCO5
ZINC08439279	5.12	9.34	-14.59	1	7	0	451.5	8	CCOC(=O)c1c2cc(ccc2oc1c3ccccc3)NS(=O)(=O)c4ccc(cc4)OC
ZINC08439279	5.12	9.37	-50.55	0	7	-1	450.492	8	CCOC(=O)c1c2cc(ccc2oc1c3ccccc3)[N-]S(=O)(=O)c4ccc(cc4)OC
ZINC02170228	4.66	1.28	-13.03	0	7	0	583.787	6	COc1cc(cc(c1OCC#C)I)/C=C/2\C(=O)N(C(=O)S2)Cc3cc4c(cc3Cl)OCO4
ZINC02502128	4.53	-2.29	-14.47	1	6	0	452.326	6	Cc1c(c2cc(ccc2o1)NS(=O)(=O)c3ccc(cc3)Br)C(=O)OC(C)C
ZINC08439278	5.98	1.09	-13.98	0	7	0	606.853	7	COc1cc(cc(c1OCc2ccc(cc2)F)Br)/C=C/3\C(=O)N(C(=O)S3)Cc4cc5c(cc4Cl)OCO5
ZINC08439277	5.87	15.92	-18.51	2	6	0	530.049	9	CC1=C([C@H](C(=C(N1)SCC(=O)OCc2ccccc2)C#N)c3ccccc3Cl)C(=O)Nc4ccccc4
ZINC08439277	6.39	0.26	-12.43	1	6	0	530.049	9	C=C1C([C@H](C(C(=N1)SCC(=O)OCc2ccccc2)C#N)c3ccccc3Cl)C(=O)Nc4ccccc4
ZINC08439277	6.39	2.04	-22.4	1	6	0	530.049	9	C=C1C([C@H]([C@H](C(=N1)SCC(=O)OCc2ccccc2)C#N)c3ccccc3Cl)C(=O)Nc4ccccc4
ZINC02484247	5.43	-0.37	-15.22	1	6	0	429.538	7	Cc1c(c2cc(ccc2o1)NS(=O)(=O)c3ccc(cc3)C(C)(C)C)C(=O)OC(C)C
ZINC01013162	4.66	-0.55	-9.14	1	6	0	440.908	4	CCc1cccc2c1[nH]cc2/C=C\3/C(=O)N(C(=O)S3)Cc4cc5c(cc4Cl)OCO5
ZINC08439276	4.07	-2.63	-15.64	1	7	0	458.319	8	Cc1c(c2cc(ccc2o1)NS(=O)(=O)c3cc(ccc3Cl)Cl)C(=O)OCCOC
ZINC01013161	4.58	-1.77	-9.7	1	6	0	491.75	3	c1cc2c(cc1Br)c(c[nH]2)/C=C\3/C(=O)N(C(=O)S3)Cc4cc5c(cc4Cl)OCO5
ZINC02484255	3.98	-0.95	-15.38	1	7	0	431.51	8	Cc1cc(c(c(c1)C)S(=O)(=O)Nc2ccc3c(c2)c(c(o3)C)C(=O)OCCOC)C
ZINC01013156	3.68	-0.08	-10.23	0	7	0	433.869	5	COc1cc(cc(c1)OC)/C=C/2\C(=O)N(C(=O)S2)Cc3cc4c(cc3Cl)OCO4
ZINC04149911	5.19	8.66	-49.01	1	8	-1	477.518	8	CCCCOC(=O)c1c(oc2c1cc(cc2)[N-]S(=O)(=O)c3ccc4c5c3cccc5C(=O)N4)C
ZINC04149911	5.19	8.63	-16.98	2	8	0	478.526	8	CCCCOC(=O)c1c(oc2c1cc(cc2)NS(=O)(=O)c3ccc4c5c3cccc5C(=O)N4)C
ZINC08439275	5.87	15.93	-18.48	2	6	0	530.049	9	CC1=C([C@@H](C(=C(N1)SCC(=O)OCc2ccccc2)C#N)c3ccccc3Cl)C(=O)Nc4ccccc4
ZINC08439275	6.39	0.25	-11.97	1	6	0	530.049	9	C=C1C([C@@H](C(C(=N1)SCC(=O)OCc2ccccc2)C#N)c3ccccc3Cl)C(=O)Nc4ccccc4
ZINC08439275	6.39	1.78	-13.07	1	6	0	530.049	9	C=C1C([C@@H]([C@@H](C(=N1)SCC(=O)OCc2ccccc2)C#N)c3ccccc3Cl)C(=O)Nc4ccccc4
ZINC08439275	6.21	0.56	-11.17	1	6	0	530.049	9	CC1=C([C@@H]([C@H](C(=N1)SCC(=O)OCc2ccccc2)C#N)c3ccccc3Cl)C(=O)Nc4ccccc4
ZINC08439274	4.76	1.16	-19.57	1	7	0	541	8	Cc1cccc(c1)NC(=O)COc2ccc(cc2OC)/C=C/3\C(=O)N(C(=O)S3)Cc4c(cccc4Cl)F
ZINC02497138	5.61	-1.17	-13.34	1	6	0	437.517	8	CCCCOC(=O)c1c(oc2c1cc(cc2)NS(=O)(=O)c3ccc4ccccc4c3)C
ZINC02170185	5.54	1.58	-9.92	0	4	0	453.922	6	c1ccc(cc1)COc2ccc(cc2)/C=C/3\C(=O)N(C(=O)S3)Cc4c(cccc4Cl)F
ZINC02480679	4.86	-1.55	-15.25	1	7	0	431.51	10	CCCCOC(=O)c1c(oc2c1cc(cc2)NS(=O)(=O)c3ccc(cc3)OCC)C
ZINC02170179	6.8	1.34	-9.91	0	4	0	522.812	6	c1cc(cc(c1)Cl)COc2ccc(cc2/C=C/3\C(=O)N(C(=O)S3)Cc4c(cccc4Cl)F)Cl
ZINC02502130	5.23	-2.35	-13.93	1	6	0	466.353	8	CCCCOC(=O)c1c(oc2c1cc(cc2)NS(=O)(=O)c3ccc(cc3)Br)C
ZINC00716242	5.21	13.5	-18.99	1	6	0	439.924	6	CC1=C([C@H](C(=C(N1)SCc2cccc(c2)[N+](=O)[O-])C#N)c3ccc(cc3)Cl)C(=O)C
ZINC00716242	5.72	12.19	-14.08	0	6	0	439.924	6	CC(=O)C1[C@H](C(C(=NC1=C)SCc2cccc(c2)[N+](=O)[O-])C#N)c3ccc(cc3)Cl
ZINC00716242	5.72	13.26	-21.61	0	6	0	439.924	6	CC(=O)C1[C@H]([C@H](C(=NC1=C)SCc2cccc(c2)[N+](=O)[O-])C#N)c3ccc(cc3)Cl
ZINC00716242	5.54	12.38	-13.26	0	6	0	439.924	6	CC1=C([C@H]([C@@H](C(=N1)SCc2cccc(c2)[N+](=O)[O-])C#N)c3ccc(cc3)Cl)C(=O)C
ZINC08439272	6.73	-0.85	-16.95	1	6	0	487.577	8	CCCc1c(c2cc(c3ccccc3c2o1)NS(=O)(=O)c4ccc5ccccc5c4)C(=O)OCC
ZINC02170170	5.12	1.16	-10.69	0	5	0	547.773	6	CCOc1cc(cc(c1OC)I)/C=C/2\C(=O)N(C(=O)S2)Cc3c(cccc3Cl)F
ZINC08439271	5.62	-1.13	-13.2	1	6	0	437.517	8	CCCc1c(c2cc(ccc2o1)NS(=O)(=O)c3ccc4ccccc4c3)C(=O)OCC
ZINC08439270	7.6	1.48	-13.4	0	5	0	626.931	8	CCOc1cc(cc(c1OCc2cccc3c2cccc3)Br)/C=C/4\C(=O)N(C(=O)S4)Cc5c(cccc5Cl)F
ZINC08439269	5.72	-1.77	-13.24	1	6	0	456.347	8	CCCc1c(c2cc(ccc2o1)NS(=O)(=O)c3cc(ccc3Cl)Cl)C(=O)OCC
ZINC08439268	5.21	14.03	-21.93	1	6	0	439.924	6	CC1=C([C@@H](C(=C(N1)SCc2cccc(c2)[N+](=O)[O-])C#N)c3ccc(cc3)Cl)C(=O)C
ZINC08439268	5.72	1.23	-15.89	0	6	0	439.924	6	CC(=O)C1[C@@H](C(C(=NC1=C)SCc2cccc(c2)[N+](=O)[O-])C#N)c3ccc(cc3)Cl
ZINC08439268	5.54	1.09	-14.57	0	6	0	439.924	6	CC1=C([C@@H]([C@@H](C(=N1)SCc2cccc(c2)[N+](=O)[O-])C#N)c3ccc(cc3)Cl)C(=O)C
ZINC08439267	7.02	1.11	-11.23	0	5	0	644.289	7	COc1cc(cc(c1OCc2ccc(cc2)Cl)I)/C=C/3\C(=O)N(C(=O)S3)Cc4c(cccc4Cl)F
ZINC04149912	5.54	-1.02	-16.21	1	6	0	437.517	8	CCCCOC(=O)c1c(oc2c1cc(c3c2cccc3)NS(=O)(=O)c4ccccc4)C
ZINC08439266	5.58	0.38	-15.73	0	5	0	448.931	5	COc1cc(ccc1OCc2ccccc2Cl)/C=c/3\c(=O)n4c5ccccc5nc4s3
ZINC02170161	5.25	2.69	-11.97	0	5	0	501.938	7	COc1cc(ccc1OCc2ccccc2F)/C=C/3\C(=O)N(C(=O)S3)Cc4c(cccc4Cl)F
ZINC08439265	6.1	-0.88	-16.19	1	7	0	481.57	10	CCCCCOC(=O)c1c(oc2c1cc(c3c2cccc3)NS(=O)(=O)c4ccc(cc4)OC)C
ZINC08439264	6.51	-0.22	-16.96	0	5	0	527.827	5	COc1cc(cc(c1OCc2ccccc2Cl)Br)/C=c/3\c(=O)n4c5ccccc5nc4s3
ZINC02170156	6.33	1.82	-10.14	0	4	0	511.405	5	c1ccc(c(c1)Cn2cc(c3c2cccc3)/C=C/4\C(=O)N(C(=O)S4)Cc5c(cccc5Cl)F)Cl
ZINC08439263	5.61	-1.61	-14	1	6	0	435.929	9	CCCCCOC(=O)c1c(oc2c1cc(cc2)NS(=O)(=O)c3ccc(cc3)Cl)C

Supplementary Table S2:
Detailed table of XP Docking using Schrodinger's Glide module

ZINC_ID	xLogP	ApolarDesolvation	PolarDesolvation	H Donors	H Acceptors	Charge	MolecularWeight	RotatableBonds	SMILES
ZINC03219042	5.87	-2.28	-12.98	1	6	0	500.37	7	CCOC(=O)c1c2cc(ccc2oc1c3ccccc3)NS(=O)(=O)c4ccc(cc4)Br
ZINC08439262	7.07	1.24	-12.15	0	5	0	611.316	8	CCOc1cc(cc(c1OCc2ccccc2Cl)Br)/C=C/3\C(=O)N(C(=O)S3)Cc4c(cccc4Cl)F
ZINC02694664	4.96	9.99	-15.03	0	7	0	469.559	6	Cc1c(c2cc(ccc2o1)N(C(=O)C3CCCCC3)S(=O)(=O)c4ccc(cc4)OC)C(=O)C
ZINC08439261	6.49	0.3	-14.49	0	4	0	434.52	4	c1ccc2c(c1)cccc2COc3cccc(c3)/C=c/4\c(=O)n5c6ccccc6nc5s4
ZINC03635711	6.86	13.71	-12.92	0	5	0	465.615	4	Cc1ccc(c(c1)C)S(=O)(=O)N(c2ccc3c(c2)c4c(o3)CCCC4)C(=O)C5CCCCC5
ZINC08439260	4.69	-1.29	-16.53	1	8	0	475.478	5	Cc1c(c2cc(ccc2o1)NS(=O)(=O)c3ccc4c(c3)C(=O)c5ccccc5C4=O)C(=O)OC
ZINC03635679	5.67	12.02	-13.47	0	7	0	483.586	6	Cc1ccc(c(c1)C)S(=O)(=O)N(c2ccc3c(c2)c(c(o3)C)C(=O)OC)C(=O)C4CCCCC4
ZINC08439259	6.79	-0.28	-16.26	0	5	0	574.827	5	COc1cc(cc(c1OCc2ccccc2Cl)I)/C=c/3\c(=O)n4c5ccccc5nc4s3
ZINC02503435	4.88	-0.89	-14.62	1	6	0	423.49	6	Cc1c(c2cc(ccc2o1)NS(=O)(=O)c3cccc4c3cccc4)C(=O)OC(C)C
ZINC06142777	5.87	-1.98	-17.96	1	7	0	499.544	3	CC1(Cc2c(c3cc(ccc3o2)NS(=O)(=O)c4ccc5c(c4)C(=O)c6ccccc6C5=O)C(=O)C1)C
ZINC08439257	5.27	11.42	-18.61	0	8	0	499.585	8	CCOC(=O)c1c(oc2c1cc(cc2)N(C(=O)C3CCCCC3)S(=O)(=O)c4ccc(cc4)OC)C
ZINC02170138	4.57	-0.87	-11.13	1	5	0	486.746	5	CCOc1cc(cc(c1O)Br)/C=C/2\C(=O)N(C(=O)S2)Cc3c(cccc3Cl)F
ZINC02503421	4.71	-4.7	-11.54	1	4	0	420.309	3	c1cc2c(ccc3c2c(c1)C(=O)S3)S(=O)(=O)Nc4ccc(cc4)Br
ZINC00715097	3.25	9.95	-53.53	0	8	-1	424.389	5	c1cc(ccc1[C@H]2C(=C(C(=O)N2c3nccs3)[O-])C(=O)c4ccc(cc4)[N+](=O)[O-])F
ZINC00715097	3.7	0.31	-36.78	1	8	0	425.397	4	c1cc(ccc1[C@H]2C(=C(c3ccc(cc3)[N+](=O)[O-])O)C(=O)C(=O)N2c4nccs4)F
ZINC00715097	2.67	0.54	-24.55	0	8	0	425.397	5	c1cc(ccc1[C@H]2[C@@H](C(=O)C(=O)N2c3nccs3)C(=O)c4ccc(cc4)[N+](=O)[O-])F
ZINC08439256	5.87	-2.22	-15.42	1	6	0	458.539	4	CC(C)c1ccc(cc1)S(=O)(=O)Nc2ccc3c4c2C(=O)c5ccccc5-c4cc(=O)n3C
ZINC08439255	6.08	3.21	-11.29	0	5	0	524.013	9	COc1cc(cc(c1OCc2ccccc2)CC=C)/C=C\3/C(=O)N(C(=O)S3)Cc4c(cccc4Cl)F
ZINC02503430	5.18	-2.12	-14.85	1	6	0	444.512	3	Cc1ccc(c(c1)C)S(=O)(=O)Nc2ccc3c4c2C(=O)c5ccccc5-c4cc(=O)n3C
ZINC03635676	5.29	11.72	-14.51	0	7	0	469.559	6	Cc1ccc(cc1)S(=O)(=O)N(c2ccc3c(c2)c(c(o3)C)C(=O)OC)C(=O)C4CCCCC4
ZINC03635660	5.67	12.35	-15.25	0	7	0	483.586	7	CCOC(=O)c1c(oc2c1cc(cc2)N(C(=O)C3CCCCC3)S(=O)(=O)c4ccc(cc4)C)C
ZINC08439254	4.44	1.54	-22.42	1	7	0	502.592	8	Cc1ccc(cc1)CN2C(=O)/C(=C/c3ccc(c(c3)OC)OCC(=O)Nc4ccccc4C)/SC2=O
ZINC00715098	3.25	9.95	-53.52	0	8	-1	424.389	5	c1cc(ccc1[C@@H]2C(=C(C(=O)N2c3nccs3)[O-])C(=O)c4ccc(cc4)[N+](=O)[O-])F
ZINC00715098	3.7	0.31	-36.34	1	8	0	425.397	4	c1cc(ccc1[C@@H]2C(=C(c3ccc(cc3)[N+](=O)[O-])O)C(=O)C(=O)N2c4nccs4)F
ZINC00715098	2.67	0.54	-24.5	0	8	0	425.397	5	c1cc(ccc1[C@@H]2[C@H](C(=O)C(=O)N2c3nccs3)C(=O)c4ccc(cc4)[N+](=O)[O-])F
ZINC03635694	5.52	11.41	-13.04	0	7	0	489.977	6	Cc1c(c2cc(ccc2o1)N(C(=O)C3CCCCC3)S(=O)(=O)c4ccc(cc4)Cl)C(=O)OC
ZINC03635668	4.84	11.03	-14.9	0	7	0	455.532	6	Cc1c(c2cc(ccc2o1)N(C(=O)C3CCCCC3)S(=O)(=O)c4ccccc4)C(=O)OC
ZINC03635652	5.36	11.37	-15.11	0	6	0	453.56	5	Cc1ccc(cc1)S(=O)(=O)N(c2ccc3c(c2)c(c(o3)C)C(=O)C)C(=O)C4CCCCC4
ZINC00950300	5.38	11.88	-12.96	0	7	0	487.549	7	CCOC(=O)c1c(oc2c1cc(cc2)N(C(=O)C3CCCCC3)S(=O)(=O)c4ccc(cc4)F)C
ZINC02503431	5.03	-3.17	-14.96	1	6	0	450.903	3	Cn1c2ccc(c3c2c(cc1=O)-c4ccccc4C3=O)NS(=O)(=O)c5ccc(cc5)Cl
ZINC03635671	5.76	12.48	-14.17	0	7	0	483.586	7	CCc1ccc(cc1)S(=O)(=O)N(c2ccc3c(c2)c(c(o3)C)C(=O)OC)C(=O)C4CCCCC4
ZINC01013140	4.85	1.25	-16.62	1	6	0	472.566	7	Cc1ccc(cc1)CN2C(=O)/C(=C/c3ccc(cc3)OCC(=O)Nc4ccccc4C)/SC2=O
ZINC08439251	5.07	-1.32	-16.36	1	8	0	489.505	6	CCOC(=O)c1c(oc2c1cc(cc2)NS(=O)(=O)c3ccc4c(c3)C(=O)c5ccccc5C4=O)C
ZINC02170096	4.95	1.43	-55.68	0	6	-1	452.895	5	Cc1ccc(cc1)CN2C(=O)/C(=C/c3ccc(o3)c4ccc(c(c4)Cl)C(=O)[O-])/SC2=O
ZINC08439250	7.44	-0.43	-15.03	0	5	0	609.272	5	COc1cc(cc(c1OCc2ccc(cc2Cl)Cl)I)/C=c/3\c(=O)n4c5ccccc5nc4s3
ZINC02503433	4.81	-3.73	-16.66	1	5	0	428.513	6	c1ccc(cc1)CN(Cc2ccccc2)S(=O)(=O)c3ccc4c5c3cccc5C(=O)N4
ZINC08439249	6.36	2.26	-8.76	0	4	0	465.574	6	Cc1ccc(cc1)CN2C(=O)/C(=C\c3c4ccccc4ccc3OCc5ccccc5)/SC2=O
ZINC08439248	5.59	11.19	-13.77	0	6	0	473.978	5	Cc1c(c2cc(ccc2o1)N(C(=O)C3CCCCC3)S(=O)(=O)c4ccc(cc4)Cl)C(=O)C
ZINC02694666	5.34	11.19	-13.59	0	7	0	483.586	7	CCOc1ccc(cc1)S(=O)(=O)N(c2ccc3c(c2)c(c(o3)C)C(=O)C)C(=O)C4CCCCC4
ZINC08439247	6.11	0.61	-17.14	0	5	0	464.546	5	COc1cc(ccc1OCc2cccc3c2cccc3)/C=c/4\c(=O)n5c6ccccc6nc5s4
ZINC08439246	5.83	1.24	-8.59	0	4	0	615.23	5	Cc1ccc(cc1)CN2C(=O)/C(=C/c3cc(c(c(c3)I)OCC#C)I)/SC2=O
ZINC08439245	5.24	-2.51	-14.68	0	4	0	445.565	6	c1ccc(cc1)CN(Cc2ccccc2)S(=O)(=O)c3ccc4c5c3cccc5C(=O)S4
ZINC08439244	5.45	1.13	-26.86	0	7	0	423.837	3	c1ccc2c(c1)nc3n2c(=O)/c(=C\c4ccc(o4)c5ccc(cc5[N+](=O)[O-])Cl)/s3
ZINC03635715	7.24	14.55	-13.57	0	5	0	479.642	4	Cc1cc(c(cc1C)S(=O)(=O)N(c2ccc3c(c2)c4c(o3)CCCC4)C(=O)C5CCCCC5)C
ZINC02170054	4.97	1.74	-14.66	3	7	0	549.469	7	CC1(CC2=C([C@H](C(=C(N2Nc3ccccc3)N)C#N)c4cc(c(c(c4)Br)OCC=C)OC)C(=O)C1)C
ZINC06142780	6.12	-2.73	-15.92	1	6	0	471.534	3	C[C@H]1CCc2c(c3cc(ccc3o2)NS(=O)(=O)c4ccc5c(c4)C(=O)c6ccccc6C5=O)C1
ZINC02170057	4.97	1.76	-14.66	3	7	0	549.469	7	CC1(CC2=C([C@@H](C(=C(N2Nc3ccccc3)N)C#N)c4cc(c(c(c4)Br)OCC=C)OC)C(=O)C1)C
ZINC06142778	6.12	-2.73	-15.89	1	6	0	471.534	3	C[C@@H]1CCc2c(c3cc(ccc3o2)NS(=O)(=O)c4ccc5c(c4)C(=O)c6ccccc6C5=O)C1
ZINC08439242	5.11	14.72	-38.71	2	9	1	486.552	6	CC1CCN(CC1)c2ccc(cc2[N+](=O)[O-])/C=N/c3c([nH+]c4n3cccc4)c5ccc(c(c5)OC)O
ZINC08439242	5.11	13.58	-40.46	2	9	1	486.552	6	CC1CCN(CC1)c2ccc(cc2[N+](=O)[O-])/C=N\c3c([nH+]c4n3cccc4)c5ccc(c(c5)OC)O
ZINC08439241	5.23	8.69	-13.14	0	4	0	418.312	2	Cc1cc(c(cc1C)S(=O)(=O)/N=C/2\C=C(C(=O)c3c2cccc3)Br)C
ZINC02170050	4.99	15.58	-18.52	3	7	0	520.633	7	CC1(CC2=C([C@@H](C(=C(N2Nc3ccccc3)N)C#N)c4ccc(c(c4)OC)OCc5ccccc5)C(=O)C1)C
ZINC08439240	6.47	15.83	-15.26	0	6	0	492.362	7	COc1cccc(c1OCc2ccc(cc2Cl)Cl)/C=N/c3c(nc4n3cccc4)c5ccco5
ZINC08439240	6.47	14.21	-13.14	0	6	0	492.362	7	COc1cccc(c1OCc2ccc(cc2Cl)Cl)/C=N\c3c(nc4n3cccc4)c5ccco5
ZINC08439240	6.47	14.74	-31.91	1	6	1	493.37	7	COc1cccc(c1OCc2ccc(cc2Cl)Cl)/C=N\c3c([nH+]c4n3cccc4)c5ccco5
ZINC08439240	6.47	16.24	-30.01	1	6	1	493.37	7	COc1cccc(c1OCc2ccc(cc2Cl)Cl)/C=N/c3c([nH+]c4n3cccc4)c5ccco5
ZINC08439239	7.24	0.13	-15.54	1	6	0	493.625	9	CCCCOC(=O)c1c(oc2c1cc(c3c2cccc3)NS(=O)(=O)c4ccc(cc4)C(C)(C)C)C
ZINC08439238	6.31	1.46	-10.21	1	5	0	456.977	8	CCN(CC)c1ccc(c(c1)OCc2ccc(cc2)Cl)/C=C(\C#N)/c3[nH]c4ccccc4n3
ZINC02170042	4.04	13	-17.99	3	7	0	470.573	7	CC1(CC2=C([C@H](C(=C(N2Nc3ccccc3)N)C#N)c4ccc(c(c4)OC)OCC=C)C(=O)C1)C
ZINC02502136	5.59	-1	-16.14	1	7	0	467.543	9	CCCCOC(=O)c1c(oc2c1cc(c3c2cccc3)NS(=O)(=O)c4ccc(cc4)OC)C
ZINC08439237	7.39	0.76	-12.85	0	4	0	481.404	4	Cc1ccc2c(c1C)nc3n2c(=O)/c(=C/c4ccccc4OCc5ccc(cc5Cl)Cl)/s3

Supplementary Table S2:
Detailed table of XP Docking using Schrodinger's Glide module

ZINC_ID	xLogP	ApolarDesolvation	PolarDesolvation	H Donors	H Acceptors	Charge	MolecularWeight	RotatableBonds	SMILES
ZINC02170045	4.04	13.11	-17.58	3	7	0	470.573	7	CC1(CC2=C([C@@H](C(=C(N2Nc3ccccc3)N)C#N)c4ccc(c(c4)OC)OCC=C)C(=O)C1)C
ZINC08439236	6.74	0.04	-12.4	1	6	0	479.598	8	CCCCOC(=O)c1c(oc2c1cc(c3c2cccc3)NS(=O)(=O)c4c(cc(cc4C)C)C)C
ZINC00714683	5.64	0.46	-17.96	1	7	0	510.619	4	CC1(CC2=C([C@@H](Nc3c2c4ccccc4cc3)c5ccc(c(c5)[N+](=O)[O-])Sc6nccn6C)C(=O)C1)C
ZINC00621935	4.18	12.43	-15.47	3	6	0	428.536	4	Cc1cc(ccc1OC)[C@@H]2C(=C(N(C3=C2C(=O)CC(C3)(C)C)Nc4ccccc4)N)C#N
ZINC08439235	5.7	-0.28	-14.78	1	6	0	455.507	8	CCCCOC(=O)c1c(oc2c1cc(c3c2cccc3)NS(=O)(=O)c4ccc(cc4)F)C
ZINC08439234	4.38	-1.18	-42.66	0	7	-1	464.22	2	c1ccc2c(c1)nc3n2c(=O)/c(=C/c4cc(c(c(c4)I)[O-])[N+](=O)[O-])/s3
ZINC02170039	5.13	2.79	-19.33	3	7	0	538.623	7	CC1(CC2=C([C@@H](C(=C(N2Nc3ccccc3)N)C#N)c4ccc(c(c4)OC)OCc5cccc(c5)F)C(=O)C1)C
ZINC08439233	6.2	-0.16	-14.76	1	6	0	469.534	9	CCCCCOC(=O)c1c(oc2c1cc(c3c2cccc3)NS(=O)(=O)c4ccc(cc4)F)C
ZINC08439232	6.43	-0.95	-12.17	0	4	0	586.192	4	C=CCOc1c(cc(cc1I)/C=c/2\c(=O)n3c4ccccc4nc3s2)I
ZINC00621932	5.07	0.82	-12.91	2	6	0	512.4	6	CC1(CC2=C([C@@H](C(=C(O2)N)C(=O)OC)c3cc(ccc3OCc4ccccc4)Br)C(=O)C1)C
ZINC08439231	5.36	-1.43	-15.22	1	7	0	445.537	11	CCCCCOC(=O)c1c(oc2c1cc(cc2)NS(=O)(=O)c3ccc(cc3)OCC)C
ZINC08439230	7.38	-1.25	-13.56	0	4	0	636.252	4	c1ccc(cc1)COc2c(cc(cc2I)/C=c/3\c(=O)n4c5ccccc5nc4s3)I
ZINC02170034	4.22	-4.15	-26.14	1	5	0	426.991	9	Cc1ccc(cc1)N(CC(=O)NCCSCc2ccccc2Cl)S(=O)(=O)C
ZINC02480684	4.99	-1.45	-15.55	1	7	0	431.51	10	CCCCCOC(=O)c1c(oc2c1cc(cc2)NS(=O)(=O)c3ccc(cc3)OC)C
ZINC02170026	5.88	1.62	-8.92	2	6	0	517.384	7	CN(C)c1ccc2c(c1)OC(=C([C@H]2c3cc(c(c(c3)Cl)OCc4ccccc4F)Cl)C(=O)OC)N
ZINC08439229	5.84	-0.99	-15.08	1	6	0	429.538	10	CCCCCOC(=O)c1c(oc2c1cc(cc2)NS(=O)(=O)c3ccc(cc3)CC)C
ZINC02170029	5.88	1.43	-8.88	2	6	0	517.384	7	CN(C)c1ccc2c(c1)OC(=C([C@@H]2c3cc(c(c(c3)Cl)OCc4ccccc4F)Cl)C(=O)OC)N
ZINC08439228	5.75	-0.37	-11.91	1	6	0	429.538	9	CCCCCOC(=O)c1c(oc2c1cc(cc2)NS(=O)(=O)c3cc(ccc3C)C)C
ZINC02170023	5.29	0.15	-9.05	2	6	0	509.4	7	CN(C)c1ccc2c(c1)OC(=C([C@@H]2c3cc(ccc3OCc4ccccc4)Br)C(=O)OC)N
ZINC08439227	3.43	10.82	-26.15	2	7	0	540.468	6	CC1=C([C@H](C(=C(N1)SCC(=O)Nc2nc3ccc(cc3s2)Br)C#N)c4cccnc4)C(=O)C
ZINC08439227	3.95	-3.61	-21.08	1	7	0	540.468	6	CC(=O)C1[C@H](C(C(=NC1=C)SCC(=O)Nc2nc3ccc(cc3s2)Br)C#N)c4cccnc4
ZINC08439227	3.77	-3.67	-19.36	1	7	0	540.468	6	CC1=C([C@H]([C@H](C(=N1)SCC(=O)Nc2nc3ccc(cc3s2)Br)C#N)c4cccnc4)C(=O)C
ZINC08439226	6.21	-1.69	-13.25	1	6	0	470.374	9	CCCCCOC(=O)c1c(oc2c1cc(cc2)NS(=O)(=O)c3cc(ccc3Cl)Cl)C
ZINC08439225	5.74	-2.23	-13.94	1	6	0	480.38	9	CCCCCOC(=O)c1c(oc2c1cc(cc2)NS(=O)(=O)c3ccc(cc3)Br)C
ZINC01013124	3.77	-1.77	-15.83	2	6	0	437.304	4	Cc1cccc(c1)NC(=O)CN2C(=O)/C(=C/c3cc(c(c(c3)Cl)O)Cl)/SC2=O
ZINC01013116	3.37	1.85	-16.53	1	7	0	452.532	8	Cc1cccc(c1)NC(=O)CN2C(=O)/C(=C/c3cc(c(c(c3)OC)OC)CC=C)/SC2=O
ZINC08439224	5.71	-1.81	-13.37	1	6	0	456.347	8	CCCCOC(=O)c1c(oc2c1cc(cc2)NS(=O)(=O)c3cc(ccc3Cl)Cl)C
ZINC08439222	7.25	0.17	-14.98	1	6	0	493.625	9	CCCc1c(c2cc(c3ccccc3c2o1)NS(=O)(=O)c4ccc(cc4)C(C)(C)C)C(=O)OCC
ZINC01013096	3.48	1.23	-13.9	1	6	0	437.496	5	CC(C)n1cc(c2c1cccc2)/C=C/3\C(=O)N(C(=O)S3)CC(=O)Nc4ccc(cc4)F
ZINC08439221	3.43	10.83	-26.23	2	7	0	540.468	6	CC1=C([C@@H](C(=C(N1)SCC(=O)Nc2nc3ccc(cc3s2)Br)C#N)c4cccnc4)C(=O)C
ZINC08439221	3.95	-3.77	-18.31	1	7	0	540.468	6	CC(=O)C1[C@@H](C(C(=NC1=C)SCC(=O)Nc2nc3ccc(cc3s2)Br)C#N)c4cccnc4
ZINC08439221	3.77	-3.87	-17.01	1	7	0	540.468	6	CC1=C([C@@H]([C@@H](C(=N1)SCC(=O)Nc2nc3ccc(cc3s2)Br)C#N)c4cccnc4)C(=O)C
ZINC08439220	6.46	-0.51	-15.68	1	6	0	465.571	9	CCCc1c(c2cc(c3ccccc3c2o1)NS(=O)(=O)c4ccc(cc4)CC)C(=O)OCC
ZINC08439219	3.66	1.61	-17.29	1	7	0	472.538	9	CCOc1cc(ccc1OCC(C)C)/C=C\2/C(=O)N(C(=O)S2)CC(=O)Nc3ccc(cc3)F
ZINC02993022	4.51	10.82	-15.78	1	4	0	339.464	6	CCc1ccc(cc1)NC(=O)Cn2c3ccccc3nc2SCC
ZINC08439218	4.87	8.99	-12.33	1	7	0	431.51	10	CCCc1c(c2cc(ccc2o1)NS(=O)(=O)c3ccc(cc3)OCC)C(=O)OCC
ZINC08439218	4.87	9.02	-47.5	0	7	-1	430.502	10	CCCc1c(c2cc(ccc2o1)[N-]S(=O)(=O)c3ccc(cc3)OCC)C(=O)OCC
ZINC01013094	3.28	1.22	-17.28	1	7	0	458.511	8	CCOc1cc(ccc1OC(C)C)/C=C/2\C(=O)N(C(=O)S2)CC(=O)Nc3ccc(cc3)F
ZINC15941858	4.89	7.43	-55.54	0	7	-1	479.559	6	CCc1ccc(cc1)S(=O)(=O)N=C2C=C(C(=O)c3c2cccc3)[N-]S(=O)(=O)c4ccccc4
ZINC15941858	4.89	7.11	-17.01	1	7	0	480.567	6	CCc1ccc(cc1)S(=O)(=O)N=C2C=C(C(=O)c3c2cccc3)NS(=O)(=O)c4ccccc4
ZINC01013092	3.69	1.34	-17.91	1	6	0	422.437	5	c1ccc(cc1)c2ccc(o2)/C=C\3/C(=O)N(C(=O)S3)CC(=O)Nc4ccc(cc4)F
ZINC08439215	6.88	0.12	-8.5	1	4	0	499.195	5	c1ccc2c(c1)[nH]c(n2)/C(=C/c3cc(ccc3OCc4ccc(cc4Cl)Cl)Br)/C#N
ZINC15941856	5.18	9.67	-28.78	1	7	0	494.594	5	Cc1cc(c(c(c1)C)S(=O)(=O)NC2=C/C(=N/S(=O)(=O)c3ccccc3)/C(=O)c4c2cccc4)C
ZINC08439213	3.85	1.27	-17.62	1	7	0	466.559	9	CCOc1cc(ccc1OCC=C)/C=C/2\C(=O)N(C(=O)S2)CC(=O)Nc3ccc(c(c3)C)C
ZINC08439212	7	-0.29	-16.99	1	6	0	487.577	6	CC(C)(C)c1c(c2cc(c3ccccc3c2o1)NS(=O)(=O)c4ccc5ccccc5c4)C(=O)OC
ZINC08439211	3.64	0.4	-20.47	3	11	0	571.335	8	COc1cc(cc(c1OCC#C)I)C=NNC(=O)Cn2c3ccccc3nc2c4c(non4)N
ZINC08439210	3.47	1.25	-18.3	1	7	0	452.532	8	Cc1ccc(cc1C)NC(=O)CN2C(=O)/C(=C\c3ccc(c(c3)OC)OCC=C)/SC2=O
ZINC08439209	7.02	0.64	-12.34	1	6	0	479.598	6	Cc1cc(c(c(c1)C)S(=O)(=O)Nc2cc3c(c(oc3c4c2cccc4)C(C)(C)C)C(=O)OC)C
ZINC02169926	4.87	0.65	-65.31	1	8	-1	509.947	6	Cc1ccc(cc1C)NC(=O)CN2C(=O)/C(=C/c3ccc(o3)c4cc(ccc4Cl)C(=O)[O-])/SC2=O
ZINC08439208	5.06	7.57	-8.64	2	4	0	433.103	2	Cc1ccc2c(c1)nc([nH]2)/C(=C/c3cc(c(c(c3)Br)O)Br)/C#N
ZINC08439208	5.06	8.33	-38.58	1	4	-1	432.095	2	Cc1ccc2c(c1)[nH]c(n2)/C(=C/c3cc(c(c(c3)Br)[O-])Br)/C#N
ZINC08439208	5.06	8.34	-38.61	1	4	-1	432.095	2	Cc1ccc2c(c1)nc([nH]2)/C(=C/c3cc(c(c(c3)Br)[O-])Br)/C#N
ZINC02502145	5.61	-0.38	-16.03	1	6	0	437.517	6	Cc1c(c2cc(c3ccccc3c2o1)NS(=O)(=O)c4ccc(cc4)C(C)C)C(=O)OC
ZINC02169920	4.28	12.5	-69.6	1	8	-1	489.529	6	Cc1ccc(cc1C)NC(=O)CN2C(=O)/C(=C/c3ccc(o3)c4cccc(c4C)C(=O)[O-])/SC2=O
ZINC08439206	4.27	-1.97	-14	1	6	0	414.266	5	Cc1c(c2cc(ccc2o1)NS(=O)(=O)c3cc(ccc3Cl)Cl)C(=O)OC
ZINC08439205	2.42	5.29	-11.03	3	11	0	384.352	3	Cc1c(c(c(c(c1[N+](=O)[O-])C)[N+](=O)[O-])C)[C@H]2c3c(n[nH]c3OC(=C2C#N)N)C
ZINC08439204	3.78	1.38	-16.19	1	7	0	472.538	9	CC[C@H](C)Oc1ccc(cc1OCC)/C=C/2\C(=O)N(C(=O)S2)CC(=O)Nc3ccc(cc3)F
ZINC08439203	5.88	-0.71	-13.86	1	6	0	449.528	7	CCOC(=O)c1c2cc(ccc2oc1c3ccccc3)NS(=O)(=O)c4cc(ccc4C)C
ZINC08439202	2.42	5.07	-11.44	3	11	0	384.352	3	Cc1c(c(c(c(c1[N+](=O)[O-])C)[N+](=O)[O-])C)[C@@H]2c3c(n[nH]c3OC(=C2C#N)N)C
ZINC08439201	3.78	1.39	-16.23	1	7	0	472.538	9	CC[C@@H](C)Oc1ccc(cc1OCC)/C=C/2\C(=O)N(C(=O)S2)CC(=O)Nc3ccc(cc3)F
ZINC08439200	5.17	-0.51	-12.31	1	6	0	435.929	6	CCOC(=O)c1c(oc2c1cc(cc2Cl)NS(=O)(=O)c3cc(c(cc3C)C)C)C

Supplementary Table S2:
Detailed table of XP Docking using Schrodinger's Glide module

ZINC_ID	xLogP	ApolarDesolvation	PolarDesolvation	H Donors	H Acceptors	Charge	MolecularWeight	RotatableBonds	SMILES
ZINC01013082	4.07	1.43	-16.61	1	7	0	468.575	8	CC[C@H](C)Oc1ccc(cc1OC)/C=C\2/C(=O)N(C(=O)S2)CC(=O)Nc3ccc(c(c3)C)C
ZINC08439199	5.24	-0.21	-19.36	1	8	0	403.508	11	CCCCCCCC(=O)N/N=C/c1ccc(c(c1)[N+](=O)[O-])Sc2nccn2C
ZINC08439199	5.24	0.05	-37.78	2	8	1	404.516	11	CCCCCCCC(=O)N/N=C/c1ccc(c(c1)[N+](=O)[O-])Sc2[nH+]ccn2C
ZINC08439198	5.87	-5.16	-18.12	2	6	0	472.632	6	Cc1cc(c(cc1C)S(=O)(=O)Nc2ccc(cc2)NS(=O)(=O)c3cc(c(cc3C)C)C)C
ZINC08439197	3.23	-0.76	-19.86	3	13	0	483.876	7	CN(C)c1cc(c(cc1[N+](=O)[O-])/C=N/NC(=O)Cn2c3ccccc3nc2c4c(non4)N)Cl
ZINC08439196	6.74	0.12	-17.63	1	6	0	479.598	8	CCCCOC(=O)c1c(oc2c1cc(c3c2cccc3)NS(=O)(=O)c4cc(c(cc4C)C)C)C
ZINC06446903	7.61	14.23	-6.46	1	4	0	578.091	5	c1ccc2c(c1)[nH]c(n2)/C(=C/c3cc(cc(c3OCc4ccc(cc4Cl)Cl)Br)Br)/C#N
ZINC08439195	7.24	0.24	-17.58	1	6	0	493.625	9	CCCCCOC(=O)c1c(oc2c1cc(c3c2cccc3)NS(=O)(=O)c4cc(c(cc4C)C)C)C
ZINC01013078	3.94	1.1	-17.37	1	7	0	468.575	8	CCOc1cc(ccc1OC(C)C)/C=C/2\C(=O)N(C(=O)S2)CC(=O)Nc3ccc(c(c3)C)C
ZINC02483499	5.09	-0.74	-20.36	1	7	0	481.57	8	Cc1cc(c(cc1C)S(=O)(=O)Nc2cc3c(c(oc3c4c2cccc4)C)C(=O)OCCOC)C
ZINC08439193	4.44	1.28	-16.34	1	7	0	482.602	9	CC[C@H](C)Oc1ccc(cc1OCC)/C=C/2\C(=O)N(C(=O)S2)CC(=O)Nc3ccc(c(c3)C)C
ZINC08439192	5.41	-0.53	-13.21	1	6	0	449.528	7	Cc1ccc(c(c1)C)S(=O)(=O)Nc2ccc3c(c2)c(c(o3)C)C(=O)OCc4ccccc4
ZINC02184849	4.37	-2.26	-12.23	1	6	0	393.486	8	CCCCOC(=O)c1c(oc2c1cc(cc2)NS(=O)(=O)c3cccs3)C
ZINC08439191	4.44	1.27	-16.27	1	7	0	482.602	9	CC[C@@H](C)Oc1ccc(cc1OCC)/C=C/2\C(=O)N(C(=O)S2)CC(=O)Nc3ccc(c(c3)C)C
ZINC08439190	7.03	1.09	-14.16	0	5	0	511.43	5	Cc1cc(c2c(c1)n3c(=O)/c(=C/c4ccc(c(c4)OC)OCc5ccc(cc5Cl)Cl)/sc3n2)C
ZINC08439189	5.5	-1.93	-16.51	1	6	0	443.546	8	CCCc1c(c2cc(c3ccccc3c2o1)NS(=O)(=O)c4cccs4)C(=O)OCC
ZINC02169882	4.5	1.11	-55.58	0	6	-1	438.868	5	c1ccc(cc1)CN2C(=O)/C(=C/c3ccc(o3)c4ccc(c(c4)Cl)C(=O)[O-])/SC2=O
ZINC02497182	3.84	-2.79	-19.97	1	7	0	445.518	8	Cc1c(c2cc(c3ccccc3c2o1)NS(=O)(=O)c4cccs4)C(=O)OCCOC
ZINC02184848	2.73	5.44	-50.95	0	7	-1	394.45	8	Cc1c(c2cc(ccc2o1)[N-]S(=O)(=O)c3cccs3)C(=O)OCCOC
ZINC02169869	3.89	1.74	-7.38	0	5	0	458.333	6	COc1cc(cc(c1OCC#C)/C=C/2\C(=O)N(C(=O)S2)Cc3ccccc3)Br
ZINC02497178	5.3	-0.04	-18.04	1	6	0	437.517	5	Cc1cc(c(cc1C)S(=O)(=O)Nc2cc3c(c(oc3c4c2cccc4)C)C(=O)OC)C
ZINC08439187	3.1	10.93	-11.98	0	6	0	432.501	7	COc1cc(ccc1OCc2ccncc2)/C=C/3\C(=O)N(C(=O)S3)Cc4ccccc4
ZINC08439186	6.09	9.41	-14.54	1	6	0	446.528	6	CCOc1ccc(cc1)S(=O)(=O)/N=C/2\C=C(C(=O)c3c2cccc3)Nc4ccc(cc4)C
ZINC08439185	6.57	10.62	-14.48	1	5	0	430.529	5	CCc1ccc(cc1)S(=O)(=O)/N=C/2\C=C(C(=O)c3c2cccc3)Nc4ccc(cc4)C
ZINC02169823	4.16	1.72	-10.08	0	5	0	505.333	6	COc1cc(cc(c1OCC#C)I)/C=C/2\C(=O)N(C(=O)S2)Cc3ccccc3
ZINC08439183	5.75	10.09	-17.46	1	7	0	460.511	7	CCOC(=O)c1ccc(cc1)NC2=C/C(=N\S(=O)(=O)c3ccccc3)/c4ccccc4C2=O
ZINC08439182	5.59	1.66	-24.57	0	7	0	417.446	3	Cc1cc(c(cc1C)[N+](=O)[O-])c2ccc(o2)/C=c\3/c(=O)n4c5ccccc5nc4s3
ZINC08439181	5.21	7.96	-16.71	1	6	0	418.474	5	COc1ccccc1NC2=C/C(=N\S(=O)(=O)c3ccccc3)/c4ccccc4C2=O
ZINC08439180	3.73	11.23	-12.31	0	6	0	466.946	7	COc1cc(ccc1OCc2ccncc2)/C=C/3\C(=O)N(C(=O)S3)Cc4ccccc4Cl
ZINC02497202	4.82	-2.64	-20.52	1	6	0	435.501	5	CCOc1ccc(cc1)S(=O)(=O)Nc2cc3c4c(oc3c5c2cccc5)CCCC4=O
ZINC01013036	3.68	1.12	-15.63	0	6	0	448.331	3	Cn1c2ccc(cc2n(c1=O)C)/C=C/3\C(=O)N(C(=O)S3)Cc4ccc(cc4Cl)Cl
ZINC08439179	4.89	-4.43	-15.23	0	5	0	444.556	4	Cc1cc(c(c(c1)C)S(=O)(=O)N=C2C/C(=N\Cc3ccccc3)/C(=O)c4c2cccc4)C
ZINC02169772	4.66	1.85	-55.13	0	6	-1	456.858	5	c1cc(ccc1CN2C(=O)/C(=C/c3ccc(o3)c4ccc(c(c4)Cl)C(=O)[O-])/SC2=O)F
ZINC08439178	6.19	11.8	-29.91	2	3	0	470.164	2	c1ccc(cc1)C2=C(NC(=C3C=C(C(=O)C(=C3)Br)Br)N2)c4ccccc4
ZINC08439178	6.64	-2.66	-31.74	3	3	1	471.172	3	c1ccc(cc1)c2c([nH+]c([nH]2)c3cc(c(c(c3)Br)O)Br)c4ccccc4
ZINC08439178	6.64	11.81	-29.97	2	3	0	470.164	3	c1ccc(cc1)c2c([nH+]c([nH]2)c3cc(c(c(c3)Br)[O-])Br)c4ccccc4
ZINC08439178	6.64	10.29	-8	2	3	0	470.164	3	c1ccc(cc1)c2c(nc([nH]2)c3cc(c(c(c3)Br)O)Br)c4ccccc4
ZINC08439177	5.94	9.35	-20.04	2	7	0	487.581	6	CC(C)c1ccc(cc1)S(=O)(=O)/N=C/2\C=C(C(=O)c3c2cccc3)Nc4ccc(cc4)NC(=O)C
ZINC01013027	4.77	2.25	-53.71	0	6	-1	436.44	5	Cc1ccc(cc1c2ccc(o2)/C=C\3/C(=O)N(C(=O)S3)Cc4ccc(cc4)F)C(=O)[O-]
ZINC08439176	6.04	9.51	-14.53	1	6	0	446.528	6	CCOc1ccc(cc1)S(=O)(=O)/N=C/2\C=C(C(=O)c3c2cccc3)Nc4ccccc4C
ZINC13108366	5.66	14.18	-20.38	0	8	0	426.432	5	Cc1cc(c(cc1c2ccc(o2)/C=N/c3c(nc4n3cccc4)c5ccco5)[N+](=O)[O-])C
ZINC13108366	5.66	13.15	-22.33	0	8	0	426.432	5	Cc1cc(c(cc1c2ccc(o2)/C=N\c3c(nc4n3cccc4)c5ccco5)[N+](=O)[O-])C
ZINC02169761	4.41	2.13	-59.21	0	6	-1	436.44	5	Cc1c(cccc1C(=O)[O-])c2ccc(o2)/C=C\3/C(=O)N(C(=O)S3)Cc4ccc(cc4)F
ZINC08439174	5.66	8.62	-14.85	1	6	0	432.501	5	Cc1ccccc1NC2=C/C(=N\S(=O)(=O)c3ccc(cc3)OC)/c4ccccc4C2=O
ZINC08439173	3.26	1.37	-13.07	0	6	0	450.491	7	COc1cc(ccc1OCc2ccncc2)/C=C/3\C(=O)N(C(=O)S3)Cc4ccc(cc4)F
ZINC08439172	6.46	9.93	-14.75	1	6	0	460.555	6	CCOc1ccc(cc1)S(=O)(=O)/N=C/2\C=C(C(=O)c3c2cccc3)Nc4ccc(c(c4)C)C
ZINC08439171	6.09	9.04	-15.1	1	6	0	446.528	5	Cc1ccc(cc1C)NC2=C/C(=N\S(=O)(=O)c3ccc(cc3)OC)/c4ccccc4C2=O
ZINC02169720	4.38	3.25	-12.68	0	5	0	421.449	5	CC(=O)c1ccc(cc1)c2ccc(o2)/C=C/3\C(=O)N(C(=O)S3)Cc4ccc(cc4)F
ZINC08439170	6.69	10.03	-14.32	1	6	0	480.973	6	CCOc1ccc(cc1)S(=O)(=O)/N=C/2\C=C(C(=O)c3c2cccc3)Nc4cc(ccc4C)Cl
ZINC02169708	4.62	1.64	-56.46	0	6	-1	456.858	5	c1ccc(c(c1)CN2C(=O)/C(=C/c3ccc(o3)c4ccc(c(c4)Cl)C(=O)[O-])/SC2=O)F
ZINC08439169	5.98	8.98	-16.06	1	5	0	422.531	4	Cc1ccc(c(c1)NC2=C/C(=N\S(=O)(=O)c3cccs3)/c4ccccc4C2=O)C
ZINC02169664	4.34	2.98	-13.57	0	5	0	421.449	5	CC(=O)c1ccc(cc1)c2ccc(o2)/C=C/3\C(=O)N(C(=O)S3)Cc4ccccc4F
ZINC08439168	6.46	10.19	-14.78	1	6	0	460.555	6	CCOc1ccc(cc1)S(=O)(=O)/N=C/2\C=C(C(=O)c3c2cccc3)Nc4cc(ccc4C)C
ZINC02169629	4.28	2.28	-12.75	0	5	0	523.323	6	COc1cc(cc(c1OCC#C)I)/C=C/2\C(=O)N(C(=O)S2)Cc3ccccc3F
ZINC08439167	6.2	10.07	-13.2	1	5	0	434.492	4	Cc1ccc(c(c1)NC2=C/C(=N\S(=O)(=O)c3ccc(cc3)F)/c4ccccc4C2=O)C
ZINC08439166	5.43	8.44	-17.16	1	8	0	476.51	7	COc1ccc(cc1)S(=O)(=O)/N=C/2\C=C(C(=O)c3c2cccc3)Nc4ccc(cc4)C(=O)OC
ZINC08439165	3.94	-2.82	-5.56	1	4	0	501.083	3	CCOc1c(cc(cc1I)I)/C=C/2\C(=O)NC(=O)S2
ZINC08439164	6.29	10.58	-16.78	1	7	0	474.538	7	CCc1ccc(cc1)S(=O)(=O)/N=C/2\C=C(C(=O)c3c2cccc3)Nc4ccc(cc4)C(=O)OC
ZINC02169522	1.63	1.32	-24.45	0	8	0	423.45	4	c1ccc(c(c1)C#N)c2ccc(o2)/C=C/3\C(=O)N(C(=O)S3)CC(=O)N4CCOCC4
ZINC08439163	6.36	9.84	-16.65	1	5	0	436.558	4	Cc1cc(c(c(c1)C)NC2=C/C(=N\S(=O)(=O)c3cccs3)/c4ccccc4C2=O)C
ZINC15941298	4.35	4.08	-18.73	2	7	0	434.473	5	COc1ccc(cc1)S(=O)(=O)NC2=C/C(=N\c3ccc(cc3)O)/C(=O)c4c2cccc4

Supplementary Table S2:
Detailed table of XP Docking using Schrodinger's Glide module

ZINC_ID	xLogP	ApolarDesolvation	PolarDesolvation	H Donors	H Acceptors	Charge	MolecularWeight	RotatableBonds	SMILES
ZINC08439160	1.04	0.27	-19.66	0	9	0	434.47	8	CCOC(=O)COc1ccc(cc1)/C=C\2/C(=O)N(C(=O)S2)CC(=O)N3CCOCC3
ZINC08439158	2.02	-1.04	-26.74	1	10	0	539.61	8	Cc1ccc(cc1C)NC(=O)COc2ccc(cc2OC)/C=C/3\C(=O)N(C(=O)S3)CC(=O)N4CCOCC4
ZINC08439157	5.7	9.06	-18.03	1	7	0	466.54	7	CCOC(=O)c1ccc(cc1)NC2=C/C(=N\S(=O)(=O)c3cccs3)/c4ccccc4C2=O
ZINC08439156	6.67	11.51	-16.49	1	7	0	488.565	8	CCc1ccc(cc1)S(=O)(=O)/N=C/2\C=C(C(=O)c3c2cccc3)Nc4ccc(cc4)C(=O)OCC
ZINC01012880	1.82	0.49	-23.72	0	8	0	440.477	5	CC(=O)c1ccc(cc1)c2ccc(o2)/C=C/3\C(=O)N(C(=O)S3)CC(=O)N4CCOCC4
ZINC08439155	6.58	11.52	-17.64	1	7	0	488.565	7	CCOC(=O)c1ccc(cc1)NC2=C/C(=N\S(=O)(=O)c3ccc(cc3C)C)/c4ccccc4C2=O
ZINC02169356	1.51	-0.49	-16.66	0	8	0	434.514	7	CCOc1cc(ccc1OC(C)C)/C=C/2\C(=O)N(C(=O)S2)CC(=O)N3CCOCC3
ZINC08439154	5.92	10.16	-15.5	1	7	0	478.501	7	CCOC(=O)c1ccc(cc1)NC2=C/C(=N\S(=O)(=O)c3ccc(cc3)F)/c4ccccc4C2=O
ZINC08439153	3.71	0.39	-71.76	1	8	-1	481.893	6	c1ccc(cc1)NC(=O)CN2C(=O)/C(=C\c3ccc(o3)c4ccc(c(c4)C(=O)[O-])Cl)/SC2=O
ZINC13143552	5.98	8.71	-15.98	1	5	0	422.531	4	Cc1ccc(cc1C)NC2=C/C(=N\S(=O)(=O)c3cccs3)/c4ccccc4C2=O
ZINC02169330	4.05	0.02	-63.27	1	8	-1	481.893	6	c1ccc(cc1)NC(=O)CN2C(=O)/C(=C/c3ccc(o3)c4cc(ccc4Cl)C(=O)[O-])/SC2=O
ZINC08439151	7.23	11.59	-12.55	1	5	0	462.546	7	CCCCc1ccc(cc1)NC2=C/C(=N\S(=O)(=O)c3ccc(cc3)F)/c4ccccc4C2=O
ZINC01012866	3.82	11.29	-61.73	1	8	-1	461.475	6	Cc1ccc(cc1c2ccc(o2)/C=C\3/C(=O)N(C(=O)S3)CC(=O)Nc4ccccc4)C(=O)[O-]
ZINC08439150	7.52	12.2	-14.64	1	5	0	458.583	7	CCCCc1ccc(cc1)NC2=C/C(=N\S(=O)(=O)c3ccc(cc3)C)/c4ccccc4C2=O
ZINC08439149	1.52	-1.47	-19.53	0	12	0	500.489	7	COc1cc(ccc1Oc2ccc(cn2)[N+](=O)[O-])/C=C/3\C(=O)N(C(=O)S3)CC(=O)N4CCOCC4
ZINC08439148	6.42	9.94	-17.72	1	6	0	460.555	5	Cc1cc(c(cc1C)S(=O)(=O)/N=C/2\C=C(C(=O)c3c2cccc3)Nc4ccccc4OC)C
ZINC08439147	6.73	9.84	-15.9	1	6	0	460.555	6	CC(C)c1ccc(cc1)S(=O)(=O)/N=C/2\C=C(C(=O)c3c2cccc3)Nc4ccccc4OC
ZINC08439146	3.76	0.79	-15.75	1	6	0	433.533	5	Cc1ccc(cc1)NC(=O)CN2C(=O)/C(=C\c3cn(c4c3cccc4)C(C)C)/SC2=O
ZINC08439145	6.4	10.24	-17.26	1	6	0	468.534	5	COc1ccccc1NC2=C/C(=N\S(=O)(=O)c3ccc4ccccc4c3)/c5ccccc5C2=O
ZINC08439144	4.11	0.93	-72.87	1	8	-1	495.92	6	Cc1ccccc1NC(=O)CN2C(=O)/C(=C\c3ccc(o3)c4ccc(c(c4)C(=O)[O-])Cl)/SC2=O
ZINC08439143	6.92	10.25	-15.87	1	6	0	474.582	6	CC(C)(C)c1ccc(cc1)S(=O)(=O)/N=C/2\C=C(C(=O)c3c2cccc3)Nc4ccccc4OC
ZINC08439142	2	0.36	-21.8	1	8	0	436.493	4	Cc1ccccc1NC(=O)CN2C(=O)/C(=C\c3ccc4c(c3)n(c(=O)n4C)C)/SC2=O
ZINC08439141	5.65	8.14	-16.11	1	7	0	462.527	7	CCOc1ccc(cc1)S(=O)(=O)/N=C/2\C=C(C(=O)c3c2cccc3)Nc4ccccc4OC
ZINC02169139	4.45	0.56	-62.84	1	8	-1	495.92	6	Cc1ccccc1NC(=O)CN2C(=O)/C(=C/c3ccc(o3)c4cc(ccc4Cl)C(=O)[O-])/SC2=O
ZINC08439140	6.13	9.36	-16.2	1	6	0	446.528	6	CCc1ccc(cc1)S(=O)(=O)/N=C/2\C=C(C(=O)c3c2cccc3)Nc4ccccc4OC
ZINC02169133	4.22	12.19	-59.97	1	8	-1	475.502	6	Cc1ccccc1NC(=O)CN2C(=O)/C(=C/c3ccc(o3)c4cc(ccc4C)C(=O)[O-])/SC2=O
ZINC08439139	6.42	10.21	-14.14	1	6	0	460.555	5	Cc1cc(c(c(c1)C)S(=O)(=O)N=C2C=C(C(=O)c3c2cccc3)Nc4ccccc4OC)C
ZINC08439139	6.42	10.29	-15.98	1	6	0	460.555	5	Cc1cc(c(c(c1)C)S(=O)(=O)/N=C/2\C=C(C(=O)c3c2cccc3)Nc4ccccc4OC)C
ZINC02169126	3.86	12.18	-69.82	1	8	-1	475.502	6	Cc1ccccc1NC(=O)CN2C(=O)/C(=C/c3ccc(o3)c4cccc(c4C)C(=O)[O-])/SC2=O
ZINC08439138	6.02	8.58	-14.06	1	6	0	497.37	5	COc1ccccc1NC2=C/C(=N\S(=O)(=O)c3ccc(cc3)Br)/c4ccccc4C2=O
ZINC01012792	3.75	-1.55	-15.37	2	6	0	437.304	4	Cc1ccccc1NC(=O)CN2C(=O)/C(=C/c3cc(c(c(c3)Cl)O)Cl)/SC2=O
ZINC08439137	5.89	8.48	-14.21	1	6	0	452.919	5	COc1ccccc1NC2=C/C(=N\S(=O)(=O)c3ccc(cc3)Cl)/c4ccccc4C2=O
ZINC08439136	3.71	1.04	-13.61	1	6	0	433.533	5	Cc1ccccc1NC(=O)CN2C(=O)/C(=C\c3cn(c4c3cccc4)C(C)C)/SC2=O
ZINC08439135	5.66	8.59	-16.67	1	6	0	432.501	5	Cc1ccc(cc1)S(=O)(=O)/N=C/2\C=C(C(=O)c3c2cccc3)Nc4ccccc4OC
ZINC01012776	3.35	2.09	-16.15	1	7	0	452.532	8	Cc1ccccc1NC(=O)CN2C(=O)/C(=C/c3cc(c(c(c3)OC)OC)CC=C)/SC2=O
ZINC08439133	6.83	-1.06	-14.95	0	4	0	461.608	4	Cc1ccc(cc1)SC2=C/C(=N\S(=O)(=O)c3cc(c(cc3C)C)C)/c4ccccc4C2=O
ZINC01012772	3.71	-1.96	-16.76	2	7	0	538.363	6	CCOc1cc(cc(c1O)I)/C=C/2\C(=O)N(C(=O)S2)CC(=O)Nc3ccccc3C
ZINC08439132	7.14	-1.51	-13.55	0	4	0	461.608	5	Cc1ccc(cc1)SC2=C/C(=N\S(=O)(=O)c3ccc(cc3)C(C)C)/c4ccccc4C2=O
ZINC08439131	4.18	-0.65	-18.9	1	9	0	542.976	8	CCOc1cc(ccc1Oc2c(cnc(n2)Cl)F)/C=C/3\C(=O)N(C(=O)S3)CC(=O)Nc4ccccc4C
ZINC08439130	6.46	-1.48	-15.02	0	4	0	447.581	4	Cc1ccc(cc1)SC2=C/C(=N\S(=O)(=O)c3cc(ccc3C)C)/c4ccccc4C2=O
ZINC08439129	6.06	11.08	-14.45	0	5	0	463.58	6	CCOc1ccc(cc1)S(=O)(=O)/N=C/2\C=C(C(=O)c3c2cccc3)Sc4ccc(cc4)C
ZINC01012762	4.51	-0.75	-19.06	1	9	0	552.992	8	COc1ccccc1NC(=O)COc2ccc(cc2)/C=C/3\C(=O)N(C(=O)S3)Cc4cc5c(cc4Cl)OCO5
ZINC01012760	4.07	-0.08	-9.01	0	6	0	417.87	5	CCOc1ccc(cc1)/C=C/2\C(=O)N(C(=O)S2)Cc3cc4c(cc3Cl)OCO4
ZINC08439128	5.69	10.18	-14.55	0	5	0	449.553	5	Cc1ccc(cc1)SC2=C/C(=N\S(=O)(=O)c3ccc(cc3)OC)/c4ccccc4C2=O
ZINC00683837	4.82	11.74	-16.67	0	7	0	490.821	7	COc1cc(ccc1OC(=O)c2ccco2)C=C3C(=NN(C3=O)c4ccc(cc4)Cl)C(F)(F)F
ZINC08439127	6.54	-1.88	-15.02	0	4	0	447.581	5	CCc1ccc(cc1)S(=O)(=O)/N=C/2\C=C(C(=O)c3c2cccc3)Sc4ccc(cc4)C
ZINC08439126	2.59	-5.28	-19.6	3	8	0	423.45	4	Cc1cccc(c1)N2C(=O)/C(=C\c3ccc(o3)c4ccc(cc4)S(=O)(=O)N)/C(=O)N2
ZINC08439125	6.83	13.22	-13.62	0	4	0	461.608	4	Cc1ccc(cc1)SC2=C/C(=N\S(=O)(=O)c3c(cc(cc3C)C)C)/c4ccccc4C2=O
ZINC08439124	4.07	11.55	-68.61	1	7	-1	421.816	4	Cc1cccc(c1)N2C(=O)/C(=C\c3ccc(o3)c4ccc(c(c4)C(=O)[O-])Cl)/C(=O)N2
ZINC08439123	5.79	10.94	-12.55	0	4	0	437.517	4	Cc1ccc(cc1)SC2=C/C(=N\S(=O)(=O)c3ccc(cc3)F)/c4ccccc4C2=O
ZINC08439121	6.44	-3.1	-12.85	0	4	0	498.423	4	Cc1ccc(cc1)SC2=C/C(=N\S(=O)(=O)c3ccc(cc3)Br)/c4ccccc4C2=O
ZINC08439120	6.31	11.4	-12.48	0	4	0	453.972	4	Cc1ccc(cc1)SC2=C/C(=N\S(=O)(=O)c3ccc(cc3)Cl)/c4ccccc4C2=O
ZINC08439119	5.63	10.88	-15.03	0	4	0	419.527	4	Cc1ccc(cc1)SC2=C/C(=N\S(=O)(=O)c3ccccc3)/c4ccccc4C2=O
ZINC08439118	5.91	12.63	-14.7	0	5	0	445.54	4	Cc1ccc(cc1)S(=O)(=O)N(c2ccc3c(c2)c4c(o3)CCCC4)C(=O)c5ccccc5
ZINC02168976	6.24	-4.12	-10.82	2	4	0	548.098	4	Cc1cc2c(cc1C)nc([nH]2)SCC(=O)Nc3cc(c(c3)Br)Br)Br
ZINC02168967	2.62	-3.43	-21.61	4	6	0	371.462	9	CCCCOc1cccc(c1)C(=O)NC(=S)Nc2ccccc2C(=O)N
ZINC08439117	6.14	-0.99	-12.25	0	5	0	465.958	4	c1ccc(cc1)S(=O)(=O)N(c2ccc3c(c2)c4c(o3)CCCC4)C(=O)c5ccc(cc5)Cl
ZINC08439116	5.85	10.57	-13.8	2	4	0	425.326	6	CCCCC(=O)Nc1cccc(c1)NC(=O)c2cccc3c2cccc3Br
ZINC08439115	5.91	-0.64	-13.72	0	5	0	445.54	4	Cc1ccc(cc1)C(=O)N(c2ccc3c(c2)c4c(o3)CCCC4)S(=O)(=O)c5ccccc5
ZINC02168965	4.76	-2.38	-26.3	3	5	0	424.353	8	CCCCC(=O)Nc1cccc(c1)NC(=S)NC(=O)c2ccc(cc2Cl)Cl
ZINC08439114	5.47	2.11	-14.9	0	7	0	491.565	6	Cc1cc(c(c(c1)C)S(=O)(=O)N(c2ccc3c(c2)c(c(o3)C)C(=O)OC)C(=O)c4ccccc4)C

Supplementary Table S2:
Detailed table of XP Docking using Schrodinger's Glide module

ZINC_ID	xLogP	ApolarDesolvation	PolarDesolvation	H Donors	H Acceptors	Charge	MolecularWeight	RotatableBonds	SMILES
ZINC02168963	4.82	-2.21	-24.07	3	6	0	464.385	9	CCCCC(=O)Nc1cccc(c1)NC(=S)NC(=O)c2cc(ccc2OC)Br
ZINC08439113	5.53	1.53	-16.46	0	6	0	475.566	5	Cc1cc(c(c(c1)C)S(=O)(=O)N(c2ccc3c(c2)c(c(o3)C)C(=O)C)C(=O)c4ccccc4)C
ZINC02168962	5.28	-1.58	-26.5	3	5	0	431.561	9	CCCCC(=O)Nc1cccc(c1)NC(=S)NC(=O)c2ccc(cc2)c3ccccc3
ZINC08439112	5.85	-1.52	-15.75	0	5	0	451.569	4	Cc1ccc(cc1)C(=O)N(c2ccc3c(c2)c4c(o3)CCCC4)S(=O)(=O)c5cccs5
ZINC08439111	7.11	0.56	-13.2	0	5	0	487.621	4	Cc1ccc(cc1)C(=O)N(c2ccc3c(c2)c4c(o3)CCCC4)S(=O)(=O)c5cc(c(cc5C)C)C
ZINC00683820	4.74	-3.03	-20.1	3	5	0	488.794	6	c1cc(cc(c1)Cl)C(=O)Nc2ccc(cc2)NC(=S)NC(=O)c3ccc(cc3)Br
ZINC08439110	6.5	-1.79	-11	1	5	0	441.593	7	CCC(C)(C)[C@H]1CCc2c(c3cc(ccc3o2)NS(=O)(=O)c4ccc(cc4)OCC)C1
ZINC00683819	4.04	-1.58	-26.5	3	5	0	427.888	6	c1ccc(c(c1)C(=O)NC(=S)Nc2ccc(cc2)NC(=O)c3cccc(c3)Cl)F
ZINC08439109	6.5	-1.81	-10.09	1	5	0	441.593	7	CCC(C)(C)[C@@H]1CCc2c(c3cc(ccc3o2)NS(=O)(=O)c4ccc(cc4)OCC)C1
ZINC00683818	4.7	-1.96	-24.95	3	6	0	467.978	8	CC(C)Oc1cccc(c1)C(=O)NC(=S)Nc2ccc(cc2)NC(=O)c3cccc(c3)Cl
ZINC08439108	5.62	-0.91	-12.08	0	6	0	441.549	7	CCCC(=O)N(c1ccc2c(c1)c3c(o2)CCCC3)S(=O)(=O)c4ccc(cc4)OCC
ZINC00683816	2.09	-3.94	-18.41	2	6	0	420.288	5	COc1cc(cc(c1OCc2ccccn2)Br)/C=C/3\C(=O)NC(=S)N3
ZINC02483513	4.42	0.57	-16.68	0	8	0	459.52	9	CCCC(=O)N(c1ccc2c(c1)c(c(o2)C)C(=O)OC)S(=O)(=O)c3ccc(cc3)OCC
ZINC08439107	2.74	0.23	-12.32	1	6	0	496.326	7	CCOc1cc(cc(c1OCC#C)I)/C=C/2\C(=O)NC(=S)N(C2=O)CC=C
ZINC08439106	4.8	0.54	-16.66	0	8	0	473.547	10	CCCC(=O)N(c1ccc2c(c1)c(c(o2)C)C(=O)OCC)S(=O)(=O)c3ccc(cc3)OCC
ZINC00683809	4.05	2.97	-21.7	0	7	0	487.35	9	CCOc1cc(cc(c1OCC(=O)OCC)Br)/C=C\2/C(=NN(C2=O)c3ccccc3)C
ZINC08439105	5.21	0.19	-20.18	0	7	0	491.565	7	CCOc1ccc(cc1)S(=O)(=O)N(c2ccc3c(c2)c(c(o3)C)C(=O)C)C(=O)c4ccc(cc4)C
ZINC08439104	4.7	11.01	-16.13	0	8	0	493.537	8	CCOc1ccc(cc1)S(=O)(=O)N(c2ccc3c(c2)c(c(o3)C)C(=O)OC)C(=O)c4ccccc4
ZINC02497224	3.47	-0.95	-18.68	0	8	0	478.526	7	CCOc1ccc(cc1)S(=O)(=O)N(c2ccc3c(c2)c(c(o3)C)C(=O)C)C(=O)c4ccncc4
ZINC08439102	7.27	0.02	-9.37	1	4	0	439.621	5	CCC(C)(C)[C@H]1CCc2c(c3cc(ccc3o2)NS(=O)(=O)c4c(cc(cc4C)C)C)C1
ZINC08439101	7.27	0.1	-8.99	1	4	0	439.621	5	CCC(C)(C)[C@@H]1CCc2c(c3cc(ccc3o2)NS(=O)(=O)c4c(cc(cc4C)C)C)C1
ZINC00683801	3.48	2.87	-19.34	0	7	0	473.323	8	CCOc1cc(c(cc1OCC(=O)OC)Br)/C=C\2/C(=NN(C2=O)c3ccccc3)C
ZINC08439100	7.05	1.82	-14.47	0	6	0	499.588	7	Cc1cc(c(c(c1)C)S(=O)(=O)N(c2ccc(cc2)OC(=O)c3ccccc3)C(=O)c4ccccc4)C
ZINC08439099	5.37	-0.52	-11.66	0	6	0	474.582	4	Cc1cc(c(c(c1)C)S(=O)(=O)N(c2ccc3c(c2)c4c(o3)CCCC4)C(=O)c5ccncc5)C
ZINC08439098	4.61	3.41	-18.86	0	5	0	430.479	7	CCOc1cc(ccc1OCc2cccc(c2)F)/C=C\3/C(=NN(C3=O)c4ccccc4)C
ZINC08439097	6.38	0.5	-11.06	0	5	0	439.577	5	CCCC(=O)N(c1ccc2c(c1)c3c(o2)CCCC3)S(=O)(=O)c4c(cc(cc4C)C)C
ZINC00683795	3.48	2.82	-18.95	0	7	0	473.323	8	CCOC(=O)COc1cc(c(cc1OC)/C=C\2/C(=NN(C2=O)c3ccccc3)C)Br
ZINC02497221	4.17	10.4	-14.18	0	7	0	443.521	6	CCOC(=O)c1c(oc2c1cc(cc2)N(C(=O)C)S(=O)(=O)c3c(cc(cc3C)C)C)C
ZINC04198939	3.21	-0.26	-16.9	0	7	0	452.463	5	Cc1c(c2cc(ccc2o1)N(C(=O)c3ccncc3)S(=O)(=O)c4ccc(cc4)F)C(=O)C
ZINC08439096	5.25	1.51	-17.47	0	7	0	495.528	7	CCOC(=O)c1c(oc2c1cc(cc2)N(C(=O)c3ccc(cc3)C)S(=O)(=O)c4ccc(cc4)F)C
ZINC08439095	5.26	2.11	-14.08	0	7	0	463.559	7	CCOC(=O)C1=C(N=c2n(c(=O)c(=Cc3ccccn3)s2)[C@H]1c4ccc(cc4)OC(C)C)C
ZINC08439094	4.88	1.52	-18.15	0	7	0	481.501	6	Cc1ccc(cc1)C(=O)N(c2ccc3c(c2)c(c(o3)C)C(=O)OC)S(=O)(=O)c4ccc(cc4)F
ZINC08439093	4.43	10.82	-14.84	0	7	0	467.474	6	Cc1c(c2cc(ccc2o1)N(C(=O)c3ccccc3)S(=O)(=O)c4ccc(cc4)F)C(=O)OC
ZINC08439092	5.26	2.29	-14.17	0	7	0	463.559	7	CCOC(=O)C1=C(N=c2n(c(=O)/c(=C\c3ccccn3)/s2)[C@@H]1c4ccc(cc4)OC(C)C)C
ZINC08439091	4.42	0.52	-16.91	0	8	0	459.52	9	CCCC(=O)N(c1ccc2c(c1)c(c(o2)C)C(=O)OCC)S(=O)(=O)c3ccc(cc3)OC
ZINC08439090	4.7	0.55	-16.13	0	8	0	493.537	8	CCOC(=O)c1c(oc2c1cc(cc2)N(C(=O)c3ccccc3)S(=O)(=O)c4ccc(cc4)OC)C
ZINC08439089	6.26	-0.02	-12.22	1	5	0	475.57	4	CC1=C([C@H](n2c(=O)/c(=C\c3cccs3)/sc2=N1)c4ccc(cc4)F)C(=O)Nc5ccccc5
ZINC08439088	4.77	0.8	-20.37	0	8	0	493.537	7	Cc1ccc(cc1)C(=O)N(c2ccc3c(c2)c(c(o3)C)C(=O)OC)S(=O)(=O)c4ccc(cc4)OC
ZINC08439087	4.32	10.06	-16.47	0	8	0	479.51	7	Cc1c(c2cc(ccc2o1)N(C(=O)c3ccccc3)S(=O)(=O)c4ccc(cc4)OC)C(=O)OC
ZINC08439086	6.26	-0.32	-12.59	1	5	0	475.57	4	CC1=C([C@@H](n2c(=O)/c(=C\c3cccs3)/sc2=N1)c4ccc(cc4)F)C(=O)Nc5ccccc5
ZINC08439085	3.33	8.5	-14.76	0	9	0	489.509	8	COc1ccc(cc1)S(=O)(=O)N(c2ccc(cc2)OC(=O)c3ccncc3)C(=O)c4ccncc4
ZINC08439084	8.85	0.21	-28.57	0	7	0	779.737	8	CCOc1cc(cc(c1OS(=O)(=O)c2ccc(cc2)C)I)C3C4=C(CC(CC4=O)(C)C)N(C5=C3C(=O)CC(C5)(C)C)Cc6ccccc6
ZINC08439083	6.27	-6.88	-14.78	2	6	0	494.619	6	COc1ccc(cc1)S(=O)(=O)Nc2cc(c(c3c2cccc3)O)Sc4nc5ccccc5s4
ZINC00683779	6.4	2.31	-19.57	0	4	0	497.36	4	c1ccc(c(c1)COc2ccc(cc2Br)C3C4=C(CCCC4=O)OC5=C3C(=O)CCC5)F
ZINC02497232	4.53	-1.2	-16.53	1	7	0	439.489	7	CCOC(=O)c1c(oc2c1cc(c3c2cccc3)NS(=O)(=O)c4ccc(cc4)OC)C
ZINC02497229	4.44	8.19	-21.53	1	6	0	421.474	4	COc1ccc(cc1)S(=O)(=O)Nc2cc3c4c(oc3c5c2cccc5)CCCC4=O
ZINC02497229	4.44	8.26	-71.95	0	6	-1	420.466	4	COc1ccc(cc1)S(=O)(=O)[N-]c2cc3c4c(oc3c5c2cccc5)CCCC4=O
ZINC02168813	5.34	4.02	-27.29	0	4	0	496.445	3	CC1(CC2=C(C(C3=C(N2C)CC(CC3=O)(C)C)c4cc(ccc4OCC#C)Br)C(=O)C1)C
ZINC08439082	5.57	1.64	-13.39	0	7	0	471.575	8	CCCC(=O)N(c1ccc2c(c1)c(c(o2)C)C(=O)OCC)S(=O)(=O)c3cc(c(cc3C)C)C
ZINC08439081	5.19	1.67	-13.46	0	7	0	457.548	7	CCCC(=O)N(c1ccc2c(c1)c(c(o2)C)C(=O)OC)S(=O)(=O)c3cc(c(cc3C)C)C
ZINC08439080	5.98	1.49	-14.96	0	6	0	489.593	5	Cc1ccc(cc1)C(=O)N(c2ccc3c(c2)c(c(o3)C)C(=O)C)S(=O)(=O)c4cc(c(cc4C)C)C
ZINC08439079	5.53	12.45	-17.08	0	6	0	475.566	5	Cc1cc(c(cc1C)S(=O)(=O)N(c2ccc3c(c2)c(c(o3)C)C(=O)C)C(=O)c4ccccc4)C
ZINC08439078	5.47	12.58	-18.49	0	7	0	491.565	6	Cc1cc(c(cc1C)S(=O)(=O)N(c2ccc3c(c2)c(c(o3)C)C(=O)OC)C(=O)c4ccccc4)C
ZINC08439077	6.38	0.1	-11.7	0	5	0	439.577	5	CCCC(=O)N(c1ccc2c(c1)c3c(o2)CCCC3)S(=O)(=O)c4cc(c(cc4C)C)C
ZINC02168795	4.5	6.61	-15.46	0	7	0	486.446	10	CCOc1cc(ccc1OCC#C)/C=C\2/C(=NN(C2=O)c3ccc(cc3)C(=O)OCC)C(F)(F)F
ZINC08439076	5.37	-0.56	-15.1	0	6	0	474.582	4	Cc1cc(c(cc1C)S(=O)(=O)N(c2ccc3c(c2)c4c(o3)CCCC4)C(=O)c5ccncc5)C
ZINC08439075	7.7	0.73	-10.46	0	5	0	531.859	5	CCN\1C(=O)/C(=C/c2cc(ccc3c2cc(cc3)OC)OC)/S/C1=N\c4ccc(c(c4)Cl)Br
ZINC08439074	5.99	-1.7	-10.92	0	5	0	476.392	5	CCCC(=O)N(c1ccc2c(c1)c3c(o2)CCCC3)S(=O)(=O)c4ccc(cc4)Br
ZINC02168759	5.76	3.6	-16.75	1	5	0	484.547	6	Cc1cc(c(n1c2c(c3c(s2)CCCC3)C#N)C)/C=N/NC(=O)Cc4cccc(c4)C(F)(F)F
ZINC08439073	3.44	9.21	-13.86	0	8	0	477.473	7	c1cc(ccc1N(C(=O)c2ccncc2)S(=O)(=O)c3ccc(cc3)F)OC(=O)c4ccncc4
ZINC08439072	3.72	-5.56	-16.15	3	7	0	569.153	4	c1cc(ccc1N2C(=O)/C(=C\c3cc(c(c(c3)Br)O)Br)/C(=N2)C(F)(F)F)S(=O)(=O)N

Supplementary Table S2:
Detailed table of XP Docking using Schrodinger's Glide module

ZINC_ID	xLogP	ApolarDesolvation	PolarDesolvation	H Donors	H Acceptors	Charge	MolecularWeight	RotatableBonds	SMILES
ZINC02497236	4.64	-0.47	-15.17	1	6	0	427.453	6	CCOC(=O)c1c(oc2c1cc(c3c2cccc3)NS(=O)(=O)c4ccc(cc4)F)C
ZINC08439071	4.92	-1.37	-17.77	2	7	0	530.311	5	c1cc(ccc1N2C(=O)/C(=C\c3ccc(o3)c4ccc(c(c4)Cl)Cl)/C(=N2)C(F)(F)F)S(=O)(=O)N
ZINC08439070	3.87	9.74	-17	0	7	0	462.527	5	Cc1ccc(c(c1)C)S(=O)(=O)N(c2ccc3c(c2)c(c(o3)C)C(=O)C)C(=O)c4ccncc4
ZINC00683625	4.86	3.44	-11.12	1	7	0	482.842	8	CCOc1cc(cc(c1O)Cl)/C=C\2/C(=NN(C2=O)c3ccc(cc3)C(=O)OCC)C(F)(F)F
ZINC08439069	5.19	1.33	-13.31	0	7	0	457.548	8	CCCC(=O)N(c1ccc2c(c1)c(c(o2)C)C(=O)OCC)S(=O)(=O)c3ccc(cc3C)C
ZINC08439068	5.49	0.95	-16.62	0	7	0	491.565	7	CCOC(=O)c1c2cc(ccc2oc1c3ccccc3)N(C(=O)C)S(=O)(=O)c4ccc(cc4C)C
ZINC02168705	4.84	5.56	-13.14	0	7	0	476.451	10	CCCOc1ccc(cc1OC)/C=C\2/C(=NN(C2=O)c3ccc(cc3)C(=O)OCC)C(F)(F)F
ZINC02483517	5.3	-0.32	-17.79	1	6	0	437.517	6	CCOC(=O)c1c(oc2c1cc(c3c2cccc3)NS(=O)(=O)c4cc(ccc4C)C)C
ZINC08439067	4.62	8.33	-13.49	1	7	0	513.266	7	CCOC(=O)c1ccc(cc1)N2C(=O)C(=Cc3cc(c(c(c3)Br)O)OC)C(=N2)C(F)(F)F
ZINC08439066	4.99	-0.85	-14.91	0	6	0	460.555	4	Cc1ccc(c(c1)S(=O)(=O)N(c2ccc3c(c2)c4c(o3)CCCC4)C(=O)c5ccncc5)C
ZINC08439065	4.7	4.73	-9.46	0	7	0	527.293	8	CCOC(=O)c1ccc(cc1)N2C(=O)/C(=C\c3cc(c(cc3Br)OC)OC)/C(=N2)C(F)(F)F
ZINC08439064	4.82	1.34	-13.41	0	7	0	443.521	7	CCCC(=O)N(c1ccc2c(c1)c(c(o2)C)C(=O)OC)S(=O)(=O)c3cc(ccc3C)C
ZINC15277133	3.96	9.82	-12.18	0	7	0	448.397	8	CCOC(=O)c1ccc(cc1)N2C(=O)C(=Cc3ccc(c(c3)OC)OC)C(=N2)C(F)(F)F
ZINC08439062	5.19	1.31	-13.26	0	7	0	457.548	8	CCCC(=O)N(c1ccc2c(c1)c(c(o2)C)C(=O)OCC)S(=O)(=O)c3cc(ccc3C)C
ZINC08439061	5.55	-5.88	-32.34	3	8	0	616.79	13	c1ccc(cc1)OCCNC(=O)CSc2nc3ccc(cc3s2)NC(=O)CSCC(=O)Nc4ccc5ccccc5c4
ZINC08439060	5.54	1.51	-14.35	0	7	0	491.565	6	Cc1ccc(cc1)C(=O)N(c2ccc3c(c2)c(c(o3)C)C(=O)OC)S(=O)(=O)c4cc(ccc4C)C
ZINC08439059	5.47	11.43	-16.85	0	7	0	491.565	7	CCOC(=O)c1c(oc2c1cc(cc2)N(C(=O)c3ccccc3)S(=O)(=O)c4cc(ccc4C)C)C
ZINC08439058	5.09	0.88	-17.06	0	7	0	477.538	6	Cc1ccc(c(c1)S(=O)(=O)N(c2ccc3c(c2)c(c(o3)C)C(=O)OC)C(=O)c4ccccc4)C
ZINC08439056	5.6	0.93	-16.01	0	6	0	475.566	5	Cc1ccc(cc1)C(=O)N(c2ccc3c(c2)c(c(o3)C)C(=O)C)S(=O)(=O)c4cc(ccc4C)C
ZINC08439055	7.58	-1.03	-10.55	1	4	0	439.621	6	CCC(C)(C)[C@H]1CCc2c(c3cc(ccc3o2)NS(=O)(=O)c4ccc(cc4)C(C)C)C1
ZINC08439054	4.48	-4.27	-26.39	4	11	0	619.754	11	Cn1c(nnc1SCC(=O)Nc2ccc3c(c2)sc(n3)SCC(=O)Nc4ccc(cc4)C(=O)OC)c5ccc(cc5)N
ZINC08439053	7.58	-1.05	-9.57	1	4	0	439.621	6	CCC(C)(C)[C@@H]1CCc2c(c3cc(ccc3o2)NS(=O)(=O)c4ccc(cc4)C(C)C)C1
ZINC02168677	6.87	0.05	-12.03	0	7	0	551.045	6	CCOC(=O)C1=C(N=c2n(c(=O)/c(=C/c3cc4c(cc3Cl)OCO4)/s2)[C@H]1c5cccs5)c6ccccc6
ZINC08439052	6.7	-0.15	-11.51	0	5	0	439.577	6	CCCC(=O)N(c1ccc2c(c1)c3c(o2)CCCC3)S(=O)(=O)c4ccc(cc4)C(C)C
ZINC02168681	6.87	0.1	-12.07	0	7	0	551.045	6	CCOC(=O)C1=C(N=c2n(c(=O)/c(=C/c3cc4c(cc3Cl)OCO4)/s2)[C@@H]1c5cccs5)c6ccccc6
ZINC08439051	4.99	-1.01	-13.01	0	6	0	460.555	4	Cc1ccc(c(c1)C)S(=O)(=O)N(c2ccc3c(c2)c4c(o3)CCCC4)C(=O)c5ccncc5
ZINC08439050	5.44	-1.91	-26.67	2	11	0	648.792	13	Cc1ccccc1OCc2nnc(n2C)SCC(=O)Nc3ccc4c(c3)sc(n4)SCC(=O)Nc5ccc(cc5)C(=O)OC
ZINC08439049	5.46	1.21	-17.15	0	7	0	491.565	7	CCOC(=O)c1c(oc2c1cc(cc2)N(C(=O)c3ccccc3)S(=O)(=O)c4ccc(cc4C)C)C
ZINC08439048	4.74	-1.76	-23.78	2	11	0	605.727	12	COC(=O)c1ccc(cc1)NC(=O)CSc2nc3ccc(cc3s2)NC(=O)CSc4nnnn4Cc5ccccc5
ZINC08439047	5.54	1.49	-20.21	0	7	0	491.565	6	Cc1ccc(cc1)C(=O)N(c2ccc3c(c2)c(c(o3)C)C(=O)OC)S(=O)(=O)c4ccc(cc4C)C
ZINC08439045	3.95	9.65	-13.55	0	8	0	493.928	7	c1cc(ccc1N(C(=O)c2ccncc2)S(=O)(=O)c3ccc(cc3)Cl)OC(=O)c4ccncc4
ZINC08439043	5.9	9.84	-18.23	0	5	0	462.577	4	c1ccc(cc1)S(=O)(=O)/N=C/2\C=C(C(=O)c3c2cccc3)Sc4nc5ccccc5s4
ZINC02168673	2.08	-1.08	-15.06	0	8	0	499.383	7	CCOc1cc(cc(c1OCC)Br)/C=C\2/C(=O)N(C(=O)S2)CC(=O)N3CCOCC3
ZINC08439042	6.73	11.31	-18.94	0	5	0	490.631	4	Cc1ccc(c(c1)S(=O)(=O)/N=C/2\C=C(C(=O)c3c2cccc3)Sc4nc5ccccc5s4)C
ZINC02168668	1.54	-1.55	-14.9	0	7	0	441.303	4	COc1ccc(cc1Br)/C=C\2/C(=O)N(C(=O)S2)CC(=O)N3CCOCC3
ZINC08439041	6.73	11.28	-18.47	0	5	0	490.631	4	Cc1ccc(c(c1)C)S(=O)(=O)/N=C/2\C=C(C(=O)c3c2cccc3)Sc4nc5ccccc5s4
ZINC02168664	3.21	-1.46	-16.21	0	7	0	517.401	6	c1cc(ccc1COc2ccc(cc2)/C=C\3/C(=O)N(C(=O)S3)CC(=O)N4CCOCC4)Br
ZINC08439038	5.94	9.53	-20.12	1	8	0	490.537	8	CCOc1ccc(cc1)S(=O)(=O)/N=C/2\C=C(C(=O)c3c2cccc3)Nc4ccccc4C(=O)OC
ZINC08439037	6.42	10.75	-20.48	1	7	0	474.538	7	CCc1ccc(cc1)S(=O)(=O)/N=C/2\C=C(C(=O)c3c2cccc3)Nc4ccccc4C(=O)OC
ZINC08439036	6.34	10.76	-21.43	1	7	0	474.538	6	Cc1ccc(c(c1)C)S(=O)(=O)/N=C/2\C=C(C(=O)c3c2cccc3)Nc4ccccc4C(=O)OC
ZINC02168632	6.42	2.7	-11.99	1	5	0	486.033	6	CC(C)(C)c1cc(cc(c1O)C(C)(C)C)/C=C\2/C(=O)N(C(=O)S2)CC(=O)c3ccc(cc3)Cl
ZINC08439035	7.34	11.06	-15	1	6	0	488.609	7	CCOc1ccc(cc1)NC2=C/C(=N\S(=O)(=O)c3ccc(cc3)C(C)(C)C)/c4ccccc4C2=O
ZINC08439034	5.54	-0.77	-9.38	1	4	0	590.224	3	Cc1ccc(cc1C)N2C(=O)/C(=C/c3cc(cc(c3OC)I)I)/NC2=S
ZINC08439033	7.15	10.67	-15.1	1	6	0	474.582	7	CCOc1ccc(cc1)NC2=C/C(=N\S(=O)(=O)c3ccc(cc3)C(C)C)/c4ccccc4C2=O
ZINC08439032	4.64	-1.14	-11.48	1	5	0	501.729	4	CCOc1cc(cc(c1O)I)/C=C\2/C(=O)N(C(=O)S2)c3cccc(c3)Cl
ZINC08439031	6.55	10.16	-15.14	1	6	0	460.555	7	CCc1ccc(cc1)S(=O)(=O)/N=C/2\C=C(C(=O)c3c2cccc3)Nc4ccc(cc4)OCC
ZINC08439030	2.48	0.45	-16.99	0	8	0	500.548	8	CCOc1cc(ccc1OCc2ccccc2F)/C=C\3/C(=O)N(C(=O)S3)CC(=O)N4CCOCC4
ZINC08439029	6.46	10.17	-16.13	1	6	0	460.555	6	CCOc1ccc(cc1)NC2=C/C(=N\S(=O)(=O)c3ccc(cc3C)C)/c4ccccc4C2=O
ZINC02168617	2.85	-0.38	-16	0	7	0	452.532	6	Cc1ccc(cc1)COc2ccc(cc2)/C=C\3/C(=O)N(C(=O)S3)CC(=O)N4CCOCC4
ZINC02168561	6.38	1.4	-34.57	0	10	-1	556.576	8	CCOC(=O)C1=C(N=c2n(c(=O)/c(=C/c3cc(ccc3[O-])[N+](=O)[O-])/s2)[C@H]1c4cccc(c4)OC)c5ccccc5
ZINC08439028	6.09	9.41	-15.75	1	6	0	446.528	6	CCOc1ccc(cc1)NC2=C/C(=N\S(=O)(=O)c3ccc(cc3)C)/c4ccccc4C2=O
ZINC02168569	6.38	1.39	-34.6	0	10	-1	556.576	8	CCOC(=O)C1=C(N=c2n(c(=O)/c(=C/c3cc(ccc3[O-])[N+](=O)[O-])/s2)[C@@H]1c4cccc(c4)OC)c5ccccc5
ZINC08439027	6.09	9.29	-14.96	1	6	0	446.528	5	Cc1ccc(c(c1)C)NC2=C/C(=N\S(=O)(=O)c3ccc(cc3)OC)/c4ccccc4C2=O
ZINC02168549	6.55	2.57	-14.77	0	8	0	556.64	9	CCOC(=O)C1=C(N=c2n(c(=O)/c(=C/c3cc(ccc3OC)OC)/s2)[C@H]1c4cccc(c4)OC)c5ccccc5
ZINC02168555	6.55	2.65	-14.93	0	8	0	556.64	9	CCOC(=O)C1=C(N=c2n(c(=O)/c(=C/c3cc(ccc3OC)OC)/s2)[C@@H]1c4cccc(c4)OC)c5ccccc5
ZINC08439026	6.46	9.82	-16.68	1	6	0	460.555	5	Cc1cc(c(cc1C)S(=O)(=O)/N=C/2\C=C(C(=O)c3c2cccc3)Nc4ccc(cc4)OC)C
ZINC08439025	6.09	9.27	-16.57	1	6	0	446.528	5	Cc1ccc(c(c1)S(=O)(=O)/N=C/2\C=C(C(=O)c3c2cccc3)Nc4ccc(cc4)OC)C
ZINC02168536	6.55	2.43	-14.22	0	8	0	556.64	9	CCOC(=O)C1=C(N=c2n(c(=O)/c(=C/c3ccc(cc3OC)OC)/s2)[C@H]1c4cccc(c4)OC)c5ccccc5
ZINC02168541	6.55	2.65	-14.09	0	8	0	556.64	9	CCOC(=O)C1=C(N=c2n(c(=O)/c(=C/c3ccc(cc3OC)OC)/s2)[C@@H]1c4cccc(c4)OC)c5ccccc5
ZINC08439024	6.97	10.12	-14.99	1	6	0	474.582	6	CC(C)(C)c1ccc(cc1)S(=O)(=O)/N=C/2\C=C(C(=O)c3c2cccc3)Nc4ccc(cc4)OC
ZINC01836700	6.66	1.36	-15.28	1	7	0	535.625	7	CCOC(=O)C1=C(N=c2n(c(=O)/c(=C/c3c[nH]c4c3cccc4)/s2)[C@H]1c5cccc(c5)OC)c6ccccc6

Supplementary Table S2:
Detailed table of XP Docking using Schrodinger's Glide module

ZINC_ID	xLogP	ApolarDesolvation	PolarDesolvation	H Donors	H Acceptors	Charge	MolecularWeight	RotatableBonds	SMILES
ZINC08439023	5.32	6.96	-16.26	1	7	0	448.5	6	COc1ccc(cc1)NC2=CC(=NS(=O)(=O)c3ccc(cc3)OC)c4ccccc4C2=O
ZINC01836702	6.66	1.44	-15.06	1	7	0	535.625	7	CCOC(=O)C1=C(N=c2n(c(=O)/c(=C/c3c[nH]c4c3cccc4)/s2)[C@@H]1c5cccc(c5)OC)c6ccccc6
ZINC08439022	6.18	9.24	-15.31	1	6	0	446.528	6	CCc1ccc(cc1)S(=O)(=O)/N=C/2\C=C(C(=O)c3c2cccc3)Nc4ccc(cc4)OC
ZINC08439021	6.45	0.35	-14.74	1	7	0	512.587	7	CCOC(=O)C1=C(N=c2n(c(=O)/c(=C\c3ccccc3O)/s2)[C@H]1c4cccc(c4)OC)c5ccccc5
ZINC08439020	5.43	7.88	-13.93	1	6	0	436.464	5	COc1ccc(cc1)NC2=C/C(=N\S(=O)(=O)c3ccc(cc3)F)/c4ccccc4C2=O
ZINC08439019	6.45	0.43	-12.65	1	7	0	512.587	7	CCOC(=O)C1=C(N=c2n(c(=O)/c(=C\c3ccccc3O)/s2)[C@@H]1c4cccc(c4)OC)c5ccccc5
ZINC08439018	6.07	8.44	-13.72	1	6	0	497.37	5	COc1ccc(cc1)NC2=C/C(=N\S(=O)(=O)c3ccc(cc3)Br)/c4ccccc4C2=O
ZINC02168507	5.85	0.71	-17.05	1	8	0	542.613	8	CCOC(=O)C1=C(N=c2n(c(=O)/c(=C/c3ccc(c(c3)O)OC)/s2)[C@H]1c4cccc(c4)OC)c5ccccc5
ZINC08439017	7.42	0.16	-12.94	0	5	0	487.621	5	Cc1ccc(cc1)C(=O)N(c2ccc3c(c2)c4c(o3)CCCC4)S(=O)(=O)c5ccc(cc5)C(C)C
ZINC08439016	6.97	13.91	-14.13	0	5	0	473.594	5	CC(C)c1ccc(cc1)S(=O)(=O)N(c2ccc3c(c2)c4c(o3)CCCC4)C(=O)c5ccccc5
ZINC08439015	6.28	11.62	-15.19	0	5	0	459.567	4	Cc1ccc(c(c1)S(=O)(=O)N(c2ccc3c(c2)c4c(o3)CCCC4)C(=O)c5ccccc5)C
ZINC02168515	5.85	0.89	-17.13	1	8	0	542.613	8	CCOC(=O)C1=C(N=c2n(c(=O)/c(=C/c3ccc(c(c3)O)OC)/s2)[C@@H]1c4cccc(c4)OC)c5ccccc5
ZINC08439014	6.96	-0.24	-11.57	0	5	0	494.012	4	Cc1ccc(c(c1)S(=O)(=O)N(c2ccc3c(c2)c4c(o3)CCCC4)C(=O)c5ccc(cc5)Cl)C
ZINC08439013	6.28	2.79	-16.96	0	8	0	590.705	8	CCOC(=O)C1=C(N=c2n(c(=O)/c(=C\c3cc(n(c3C)c4cccnc4)C)/s2)[C@H]1c5cccc(c5)OC)c6ccccc6
ZINC08439012	6.73	0.27	-13.03	0	5	0	473.594	4	Cc1ccc(cc1)C(=O)N(c2ccc3c(c2)c4c(o3)CCCC4)S(=O)(=O)c5cc(ccc5C)C
ZINC08439011	7.16	14.29	-14.01	0	5	0	487.621	5	CC(C)(C)c1ccc(cc1)S(=O)(=O)N(c2ccc3c(c2)c4c(o3)CCCC4)C(=O)c5ccccc5
ZINC08439010	5.89	12.2	-14.73	0	6	0	475.566	6	CCOc1ccc(cc1)S(=O)(=O)N(c2ccc3c(c2)c4c(o3)CCCC4)C(=O)c5ccccc5
ZINC08439009	6.28	2.91	-15.94	0	8	0	590.705	8	CCOC(=O)C1=C(N=c2n(c(=O)/c(=C\c3cc(n(c3C)c4cccnc4)C)/s2)[C@@H]1c5cccc(c5)OC)c6ccccc6
ZINC08439008	6.19	-1.26	-13.06	0	6	0	495.984	5	COc1ccc(cc1)S(=O)(=O)N(c2ccc3c(c2)c4c(o3)CCCC4)C(=O)c5ccc(cc5)Cl
ZINC08439007	5.84	-1.2	-16.71	0	9	0	571.655	8	CCOC(=O)C1=C(N=c2n(c(=O)/c(=C/c3ccc(o3)N4CCOCC4)/s2)[C@H]1c5cccc(c5)OC)c6ccccc6
ZINC08439006	5.96	-0.62	-13.76	0	6	0	475.566	5	Cc1ccc(cc1)C(=O)N(c2ccc3c(c2)c4c(o3)CCCC4)S(=O)(=O)c5ccc(cc5)OC
ZINC08439005	5.51	11.28	-15	0	6	0	461.539	5	COc1ccc(cc1)S(=O)(=O)N(c2ccc3c(c2)c4c(o3)CCCC4)C(=O)c5ccccc5
ZINC08439004	6.37	12.95	-14.09	0	5	0	459.567	5	CCc1ccc(cc1)S(=O)(=O)N(c2ccc3c(c2)c4c(o3)CCCC4)C(=O)c5ccccc5
ZINC08439003	6.66	13.25	-13.09	0	5	0	473.594	4	Cc1cc(c(c(c1)C)S(=O)(=O)N(c2ccc3c(c2)c4c(o3)CCCC4)C(=O)c5ccccc5)C
ZINC08439002	5.84	-1.12	-16.55	0	9	0	571.655	8	CCOC(=O)C1=C(N=c2n(c(=O)/c(=C/c3ccc(o3)N4CCOCC4)/s2)[C@@H]1c5cccc(c5)OC)c6ccccc6
ZINC08439001	6.3	-0.37	-11.48	0	5	0	483.948	4	c1cc(ccc1C(=O)N(c2ccc3c(c2)c4c(o3)CCCC4)S(=O)(=O)c5ccc(cc5)F)Cl
ZINC02168471	6.74	2.97	-16.59	0	8	0	557.672	10	CCN(CC)c1ccc(o1)/C=c\2/c(=O)n3c(=NC(=C([C@H]3c4cccc(c4)OC)C(=O)OCC)c5ccccc5)s2
ZINC08439000	6.07	0.13	-12.68	0	5	0	463.53	4	Cc1ccc(cc1)C(=O)N(c2ccc3c(c2)c4c(o3)CCCC4)S(=O)(=O)c5ccc(cc5)F
ZINC02168478	6.74	2.72	-16.84	0	8	0	557.672	10	CCN(CC)c1ccc(o1)/C=c\2/c(=O)n3c(=NC(=C([C@@H]3c4cccc(c4)OC)C(=O)OCC)c5ccccc5)s2
ZINC02497214	4.59	-2.02	-11.73	0	5	0	448.338	3	CC(=O)N(c1ccc2c(c1)c3c(o2)CCCC3)S(=O)(=O)c4ccc(cc4)Br
ZINC08438999	5.27	1.73	-14.65	0	7	0	497.576	7	CCOC(=O)C1=C(N=c2n(c(=O)/c(=C/c3cccnc3)/s2)[C@H]1c4cccc(c4)OC)c5ccccc5
ZINC08438998	6.33	-3.1	-10.84	1	4	0	470.388	3	C[C@H]1CCc2c(c3cc(c4ccccc4c3o2)NS(=O)(=O)c5ccc(cc5)Br)C1
ZINC08438997	5.27	1.56	-14.54	0	7	0	497.576	7	CCOC(=O)C1=C(N=c2n(c(=O)/c(=C/c3cccnc3)/s2)[C@@H]1c4cccc(c4)OC)c5ccccc5
ZINC08438996	6.33	-3.1	-10.85	1	4	0	470.388	3	C[C@@H]1CCc2c(c3cc(c4ccccc4c3o2)NS(=O)(=O)c5ccc(cc5)Br)C1
ZINC08438995	5.22	1.56	-14.41	0	7	0	497.576	7	CCOC(=O)C1=C(N=c2n(c(=O)/c(=C/c3ccncc3)/s2)[C@H]1c4cccc(c4)OC)c5ccccc5
ZINC08438994	6.36	-0.32	-13.5	0	5	0	459.567	4	Cc1ccc(cc1)C(=O)N(c2ccc3c(c2)c4c(o3)CCCC4)S(=O)(=O)c5ccc(cc5)C
ZINC08438993	5.22	1.58	-14.46	0	7	0	497.576	7	CCOC(=O)C1=C(N=c2n(c(=O)/c(=C/c3ccncc3)/s2)[C@@H]1c4cccc(c4)OC)c5ccccc5
ZINC08438992	5.95	-2.3	-12.18	1	5	0	435.545	5	CCOc1ccc(cc1)S(=O)(=O)Nc2cc3c4c(oc3c5c2cccc5)CC[C@H](C4)C
ZINC02168413	5.85	2.29	-22.07	0	10	0	531.546	8	CCOC(=O)C1=C(N=c2n(c(=O)/c(=C/c3ccc(o3)[N+](=O)[O-])/s2)[C@H]1c4cccc(c4)OC)c5ccccc5
ZINC08438991	5.95	-2.3	-12.18	1	5	0	435.545	5	CCOc1ccc(cc1)S(=O)(=O)Nc2cc3c4c(oc3c5c2cccc5)CC[C@@H](C4)C
ZINC02168417	5.85	2.36	-21.94	0	10	0	531.546	8	CCOC(=O)C1=C(N=c2n(c(=O)/c(=C/c3ccc(o3)[N+](=O)[O-])/s2)[C@@H]1c4cccc(c4)OC)c5ccccc5
ZINC02502146	5.71	-1.85	-19.42	1	6	0	463.555	5	CCOc1ccc(cc1)S(=O)(=O)Nc2cc3c4c(oc3c5c2cccc5)CC(CC4=O)(C)C
ZINC00683577	5.76	2.7	-14.88	0	7	0	486.549	7	CCOC(=O)C1=C(N=c2n(c(=O)/c(=C/c3ccco3)/s2)[C@H]1c4cccc(c4)OC)c5ccccc5
ZINC08438990	4.53	-1.15	-16.42	1	7	0	439.489	7	CCOc1ccc(cc1)S(=O)(=O)Nc2cc3c(c(oc3c4c2cccc4)C)C(=O)OC
ZINC00683579	5.76	2.95	-14.55	0	7	0	486.549	7	CCOC(=O)C1=C(N=c2n(c(=O)/c(=C/c3ccco3)/s2)[C@@H]1c4cccc(c4)OC)c5ccccc5
ZINC08438989	7	-0.35	-19.41	1	6	0	499.588	7	CCOC(=O)c1c2cc(c3ccccc3c2oc1c4ccccc4)NS(=O)(=O)c5cc(ccc5C)C
ZINC08438988	6.04	3.32	-15.49	0	11	0	601.637	10	CCOC(=O)C1=C(N=c2n(c(=O)/c(=C/c3cc(c(cc3[N+](=O)[O-])OC)OC)/s2)[C@H]1c4cccc(c4)OC)c5ccccc5
ZINC08438987	1.4	-9.27	-19.57	0	9	0	456.542	5	COc1ccc2cc(ccc2c1S(=O)(=O)N3CCOCC3)S(=O)(=O)N4CCOCC4
ZINC08438986	6.04	3.39	-14.95	0	11	0	601.637	10	CCOC(=O)C1=C(N=c2n(c(=O)/c(=C/c3cc(c(cc3[N+](=O)[O-])OC)OC)/s2)[C@@H]1c4cccc(c4)OC)c5ccccc5
ZINC08438985	6.56	-2.35	-15.61	1	5	0	488.392	3	CC1(Cc2c(c3cc(c4ccccc4c3o2)NS(=O)(=O)c5cc(ccc5Cl)Cl)C(=O)C1)C
ZINC08438983	4.06	-1.1	-17.16	1	7	0	457.479	8	Cc1c(c2cc(c3ccccc3c2o1)NS(=O)(=O)c4ccc(cc4)F)C(=O)OCCOC
ZINC08438981	4.34	-1.51	-18.87	1	7	0	453.516	8	Cc1ccc(cc1)S(=O)(=O)Nc2cc3c(c(oc3c4c2cccc4)C)C(=O)OCCOC
ZINC08438980	1.7	1.01	-18.8	0	9	0	493.541	7	COc1cc(ccc1OCc2ccccc2C#N)/C=C\3/C(=O)N(C(=O)S3)CC(=O)N4CCOCC4
ZINC08438979	4.81	-1.36	-18.6	1	7	0	467.543	9	CCc1ccc(cc1)S(=O)(=O)Nc2cc3c(c(oc3c4c2cccc4)C)C(=O)OCCOC
ZINC08438978	5.09	-0.78	-14.66	1	7	0	481.57	8	Cc1cc(c(c(c1)C)S(=O)(=O)Nc2cc3c(c(oc3c4c2cccc4)C)C(=O)OCCOC)C
ZINC02168339	4.56	2.91	-12.07	0	6	0	456.951	8	CCCN1C(=O)/C(=C/c2cc(c(c(c2)Cl)OCc3ccccc3C#N)OCC)/SC1=O
ZINC08438977	5.6	-0.68	-18.28	1	7	0	495.597	9	Cc1c(c2cc(c3ccccc3c2o1)NS(=O)(=O)c4ccc(cc4)C(C)(C)C)C(=O)OCCOC
ZINC02168334	2.54	0.25	-13.74	1	5	0	442.278	5	CCOc1cc(cc(c1OCC#C)I)/C=C\2/C(=O)N(C(=S)N2)C
ZINC02476467	3.97	-1.99	-15.59	1	7	0	439.489	8	Cc1c(c2cc(ccc2o1)NS(=O)(=O)c3ccc4ccccc4c3)C(=O)OCCOC
ZINC08438976	3.88	-1.22	-12.63	1	4	0	450.301	4	CN1C(=O)/C(=C/c2ccc(cc2)OCc3ccc(cc3)I)/NC1=S
ZINC08438975	6.38	6.43	-46.06	1	5	-1	482.415	5	c1ccc2c(c1)nc(s2)Sc3cc(ccc3O)[N-]S(=O)(=O)c4cc(ccc4Cl)Cl

Supplementary Table S2:
Detailed table of XP Docking using Schrodinger's Glide module

ZINC_ID	xLogP	ApolarDesolvation	PolarDesolvation	H Donors	H Acceptors	Charge	MolecularWeight	RotatableBonds	SMILES
ZINC08438975	6.38	6.41	-14.04	2	5	0	483.423	5	c1ccc2c(c1)nc(s2)Sc3cc(ccc3O)NS(=O)(=O)c4cc(ccc4Cl)Cl
ZINC08438974	4.82	-1.23	-12.71	1	4	0	472.191	4	CN1C(=O)/C(=C/c2ccc(c(c2)Br)OCc3ccc(cc3Cl)Cl)/NC1=S
ZINC08438973	6.01	-6.8	-14.48	2	5	0	442.587	6	CCc1ccc(cc1)S(=O)(=O)Nc2ccc(c(c2)Sc3nc4ccccc4s3)O
ZINC02168314	5.63	1.32	-9.93	0	5	0	474.365	5	COc1ccc2ccc(c(c2c1)/C=C\3/C(=O)N(C(=O)S3)Cc4ccc(c(c4)Cl)Cl)OC
ZINC03768202	5.53	-7.24	-15.09	2	6	0	458.586	7	CCOc1ccc(cc1)S(=O)(=O)Nc2ccc(c(c2)Sc3nc4ccccc4s3)O
ZINC02168309	4.5	-0.89	-7.16	1	5	0	489.174	4	COc1cc(c(cc1O)Br)/C=C\2/C(=O)N(C(=O)S2)Cc3ccc(cc3Cl)Cl
ZINC08438972	3.8	-0.9	-10.77	1	5	0	467.772	5	CN1C(=O)/C(=C/c2cc(c(cc2Br)OCc3ccc(cc3)Cl)OC)/NC1=S
ZINC08438971	7.04	-6.34	-19.11	2	5	0	492.647	5	Cc1ccc(c(c1)C)S(=O)(=O)Nc2cc(c(c3c2cccc3)O)Sc4nc5ccccc5s4
ZINC02168294	3.47	0.91	-55.89	0	7	-1	467.306	7	COc1cc(cc(c1OCC(=O)[O-])Cl)/C=C\2/C(=O)N(C(=O)S2)Cc3ccccc3Cl
ZINC08438970	6.89	-7.05	-13.88	2	5	0	499.038	5	c1ccc2c(c1)c(cc(c2O)Sc3nc4ccccc4s3)NS(=O)(=O)c5ccc(cc5)Cl
ZINC08438969	4.7	4.01	-11.03	0	6	0	462.571	10	CCCN1C(=O)/C(=C/c2cc(c(c(c2)OCC)OCc3ccccc3C#N)CC=C)/SC1=O
ZINC08438968	6.21	-6.91	-16.95	2	5	0	464.593	5	c1ccc(cc1)S(=O)(=O)Nc2cc(c(c3c2cccc3)O)Sc4nc5ccccc5s4
ZINC08438967	4.91	-1.1	-13.84	1	6	0	423.49	6	Cc1c(c2cc(ccc2o1)NS(=O)(=O)c3ccc4ccccc4c3)C(=O)OC(C)C
ZINC08438966	5.66	-0.06	-18.07	1	6	0	451.544	6	Cc1ccc(c(c1)S(=O)(=O)Nc2cc3c(c(oc3c4c2cccc4)C)C(=O)OC(C)C)C
ZINC02168266	6.63	11.87	-9.14	0	5	0	442.968	6	Cc1ccc(cc1C)/N=C/2\N(C(=O)/C(=C/c3cc(c(c(c3)Cl)OCC=C)OC)/S2)C
ZINC02168266	6.63	12.3	-9.52	0	5	0	442.968	6	Cc1ccc(cc1C)/N=C\2/N(C(=O)/C(=C/c3cc(c(c(c3)Cl)OCC=C)OC)/S2)C
ZINC08438965	3.6	0.94	-15.97	1	6	0	487.375	7	CCOc1ccc(cc1)N2C(=O)/C(=C/c3cc(c(c(c3)Br)OCC#C)OC)/NC2=S
ZINC02484245	5	-0.22	-15.28	1	6	0	441.48	6	Cc1c(c2cc(c3ccccc3c2o1)NS(=O)(=O)c4ccc(cc4)F)C(=O)OC(C)C
ZINC02168231	6.47	13.61	-10.42	0	5	0	439.54	5	Cc1ccc(cc1)/N=C/2\N(C(=O)/C(=C/c3ccccc3OCc4ccccc4C#N)/S2)C
ZINC02168231	6.47	14.04	-10.46	0	5	0	439.54	5	Cc1ccc(cc1)/N=C\2/N(C(=O)/C(=C/c3ccccc3OCc4ccccc4C#N)/S2)C
ZINC08438964	6.12	-1.1	-15.17	1	6	0	492.38	6	Cc1c(c2cc(c3ccccc3c2o1)NS(=O)(=O)c4cc(ccc4Cl)Cl)C(=O)OC(C)C
ZINC08438963	3.98	-0.22	-61.2	1	6	-1	429.477	6	c1ccc(cc1)N2C(=O)/C(=C/c3ccc(cc3)OCc4ccc(cc4)C(=O)[O-])/NC2=S
ZINC08438962	6.02	-0.82	-17.55	1	6	0	473.55	6	Cc1c(c2cc(c3ccccc3c2o1)NS(=O)(=O)c4ccc5ccccc5c4)C(=O)OC(C)C
ZINC00683546	4.32	3.01	-16.4	1	6	0	481.577	8	COc1cc(cc(c1OCc2ccccc2C#N)CC=C)/C=C\3/C(=O)N(C(=S)N3)c4ccccc4
ZINC02484241	5.51	-1.1	-15.06	1	6	0	457.935	6	Cc1c(c2cc(c3ccccc3c2o1)NS(=O)(=O)c4ccc(cc4)Cl)C(=O)OC(C)C
ZINC08438961	5.6	2.96	-10.7	0	5	0	436.455	6	CCOc1ccc(cc1OC)/C=C\2/C(=O)N(/C(=N\c3cccc(c3)C(F)(F)F)/S2)C
ZINC08438960	5.75	-0.48	-16.37	1	6	0	451.544	7	CCc1ccc(cc1)S(=O)(=O)Nc2cc3c(c(oc3c4c2cccc4)C)C(=O)OC(C)C
ZINC00683536	4.09	-0.23	-10.52	1	4	0	430.371	5	CCN(CC)c1ccc(cc1)/C=C\2/C(=O)N(C(=S)N2)c3ccc(cc3)Br
ZINC02484239	5.27	-0.92	-16.47	1	7	0	467.543	8	CCOc1ccc(cc1)S(=O)(=O)Nc2cc3c(c(oc3c4c2cccc4)C)C(=O)OC(C)C
ZINC08438959	6.48	2.54	-12.38	0	6	0	483.593	7	CCOc1cc(ccc1OCc2ccccc2C#N)/C=C\3/C(=O)N(/C(=N\c4ccc(cc4)C)/S3)C
ZINC08438958	5.66	0.01	-18.02	1	6	0	451.544	6	Cc1ccc(c(c1)C)S(=O)(=O)Nc2cc3c(c(oc3c4c2cccc4)C)C(=O)OC(C)C
ZINC02168153	4.37	-0.11	-61.67	1	7	-1	473.53	8	CCOc1ccc(cc1)N2C(=O)/C(=C/c3ccccc3OCc4ccc(cc4)C(=O)[O-])/NC2=S
ZINC08438957	8.01	-0.4	-12.68	1	4	0	475.654	5	CCC(C)(C)[C@H]1CCc2c(c3cc(c4ccccc4c3o2)NS(=O)(=O)c5ccc(cc5C)C)C1
ZINC02168095	3.22	-1.17	-7.28	1	5	0	420.284	4	COc1cc(c(cc1O)Br)/C=C\2/C(=O)N(C(=O)S2)Cc3ccccc3
ZINC08438956	8.01	-0.4	-12.77	1	4	0	475.654	5	CCC(C)(C)[C@@H]1CCc2c(c3cc(c4ccccc4c3o2)NS(=O)(=O)c5ccc(cc5C)C)C1
ZINC08438955	4.6	1.05	-14.43	1	5	0	448.519	7	CCOc1ccc(cc1)N2C(=O)/C(=C/c3ccccc3OCc4ccc(cc4)F)/NC2=S
ZINC08438954	2.85	1.66	-8.78	0	3	0	277.073	0	c1cc2c(ccc3c2c(c1)C(=O)OC3=O)Br
ZINC08438953	4.67	1.28	-60.1	0	7	-1	488.541	9	CCOc1cc(ccc1OCc2ccc(cc2)C(=O)[O-])/C=C\3/C(=O)N(C(=O)S3)Cc4ccccc4
ZINC02168082	2.77	0.37	-48.76	0	7	-1	477.312	7	COc1cc(c(cc1OCC(=O)[O-])Br)/C=C\2/C(=O)N(C(=O)S2)Cc3ccccc3
ZINC01012622	3.01	-1.28	-9.32	1	5	0	419.24	4	CCCN1C(=O)/C(=C/c2cc(c(c(c2)I)O)OC)/SC1=O
ZINC08438951	5.22	-4.06	-11.24	1	5	0	442.29	3	c1ccc2c(c1)C(=O)c3ccc(cc3C2=O)S(=O)(=O)Nc4ccc(cc4)Br
ZINC01012618	5.46	7.74	-9.11	1	5	0	433.327	3	Cc1ccc(cc1)/N=C/2\N(C(=O)/C(=C/c3cc(c(c(c3)Br)O)OC)/S2)C
ZINC01012618	5.46	8.18	-9.56	1	5	0	433.327	3	Cc1ccc(cc1)/N=C\2/N(C(=O)/C(=C/c3cc(c(c(c3)Br)O)OC)/S2)C
ZINC08438950	6.34	-2.03	-14.66	1	6	0	490.364	7	CCOC(=O)c1c2cc(ccc2oc1c3ccccc3)NS(=O)(=O)c4cc(ccc4Cl)Cl
ZINC08438949	7.29	-1.09	-11.96	1	4	0	447.6	4	Cc1ccc(cc1)S(=O)(=O)Nc2cc3c4c(oc3c5c2cccc5)CC[C@H](C4)C(C)(C)C
ZINC02167991	5.56	-0.29	-7.84	0	5	0	433.327	4	CN\1C(=O)/C(=C/c2ccc(cc2OC)OC)/S/C1=N/c3ccc(cc3)Br
ZINC08438948	7.29	-1.1	-11.84	1	4	0	447.6	4	Cc1ccc(cc1)S(=O)(=O)Nc2cc3c4c(oc3c5c2cccc5)CC[C@@H](C4)C(C)(C)C
ZINC02167981	4.43	0.99	-54.73	0	7	-1	425.486	6	Cc1ccc(cc1C)/N=C/2\N(C(=O)/C(=C/c3ccc(c(c3)OC)OCC(=O)[O-])/S2)C
ZINC08438947	5.22	-3.67	-9.89	1	4	0	420.328	3	C[C@H]1CCc2c(c3cc(ccc3o2)NS(=O)(=O)c4ccc(cc4)Br)C1
ZINC08438946	5.22	-3.24	-8.95	1	4	0	420.328	3	C[C@@H]1CCc2c(c3cc(ccc3o2)NS(=O)(=O)c4ccc(cc4)Br)C1
ZINC02167933	3.77	-0.46	-6.79	0	5	0	418.268	3	c1ccc(cc1)CN2C(=O)/C(=C/c3cc4c(cc3Br)OCO4)/SC2=O
ZINC02502143	3.4	-0.68	-15.82	0	7	0	466.309	5	Cc1c(c2cc(ccc2o1)N(C(=O)C)S(=O)(=O)c3ccc(cc3)Br)C(=O)OC
ZINC08438944	5.77	2.07	-15.84	0	8	0	489.505	9	c1ccc(cc1)OC(=O)N(c2ccc(cc2)OC(=O)Oc3ccccc3)S(=O)(=O)c4ccccc4
ZINC08438942	5.21	8.96	-12.87	0	4	0	426.291	2	c1ccc2cc(ccc2c1)S(=O)(=O)/N=C/3\C=C(C(=O)c4c3cccc4)Br
ZINC00577331	4.13	8.03	-10.7	0	3	0	297.379	3	CN(c1ccccc1)S(=O)(=O)c2ccc3ccccc3c2
ZINC08438941	4.79	-4.13	-22.16	1	6	0	472.994	10	COc1ccc(cc1)CCNC(=O)CN(Cc2ccc(cc2)Cl)S(=O)(=O)c3ccccc3
ZINC00683416	4.93	-2.97	-19.24	1	6	0	458.967	8	Cc1ccc(cc1)CN(CC(=O)Nc2cc(ccc2OC)Cl)S(=O)(=O)c3ccccc3
ZINC08438940	4.62	8.12	-22.05	1	6	0	430.57	9	COc1ccc(cc1)CCNC(=O)CN(C2CCCCC2)S(=O)(=O)c3ccccc3
ZINC08438939	4.8	-2.5	-22.37	0	7	0	478.57	6	COc1cc2c(cc1N(CC(=O)N3CCCCC3)S(=O)(=O)c4ccccc4)c5ccccc5o2
ZINC08438938	3.45	9.75	-15.32	0	7	0	438.505	6	CN(C)C(=O)CN(c1cc2c3ccccc3oc2cc1OC)S(=O)(=O)c4ccccc4
ZINC02167890	5.06	-1.95	-56.87	1	7	-1	452.295	4	CN\1C(=O)/C(=C/c2cc(c(c(c2)Cl)O)OC)/S/C1=N/c3ccc(c(c3)C(=O)[O-])Cl

Supplementary Table S2:

Detailed table of XP Docking using Schrodinger's Glide module

ZINC_ID	xLogP	ApolarDesolvation	PolarDesolvation	H Donors	H Acceptors	Charge	MolecularWeight	RotatableBonds	SMILES
ZINC08438937	4	-2.69	-67.73	1	7	-1	430.484	6	COc1ccccc1N2CCN(CC2)c3ccccc3NC(=O)c4ccccc4C(=O)[O-]
ZINC02167870	6.16	-0.2	-52.59	0	6	-1	470.741	4	CN\1C(=O)/C(=C/c2cc(cc(c2OC)Cl)Cl)/S/C1=N/c3ccc(c(c3)C(=O)[O-])Cl
ZINC01019586	5.05	-3.62	-9.26	1	5	0	432.955	6	c1ccc(cc1)Cn2c3ccc(cc3nc2CNc4ccc(cc4)N5CCOCC5)Cl
ZINC08438933	5.23	-5.25	-9.92	1	5	0	431.971	5	Cn1c2ccccc2nc1CNc3ccc(cc3)N4CCN(CC4)c5ccc(cc5)Cl
ZINC02167834	4.52	0.65	-57.11	0	8	-1	461.903	6	CN\1C(=O)/C(=C/c2cc(c(c(c2)OC)OC)OC)/S/C1=N/c3ccc(c(c3)C(=O)[O-])Cl
ZINC02167815	5.68	-0.82	-58.86	0	6	-1	480.747	4	CN\1C(=O)/C(=C/c2ccc(c(c2)Br)OC)/S/C1=N/c3ccc(c(c3)C(=O)[O-])Cl
ZINC02167795	4.96	-2.54	-54.49	1	7	-1	496.746	4	CN\1C(=O)/C(=C/c2cc(c(cc2Br)O)OC)/S/C1=N/c3ccc(c(c3)C(=O)[O-])Cl
ZINC08438932	6.59	-0.09	-12.8	1	6	0	489.391	8	CCc1ccc(cc1)/N=C\2/NC(=O)/C(=C\c3ccc(c(c3)Br)OCC(=O)OCC)/S2
ZINC02184672	7.08	-0.08	-9.85	1	4	0	420.921	6	COc1ccc(cc1)C(=O)c2c(nc(s2)Nc3ccc(cc3)Cl)c4ccccc4
ZINC08438931	5.79	0.87	-13.27	1	6	0	427.913	6	CCc1ccc(cc1)/N=C\2/NC(=O)/C(=C\c3cc(c(c(c3)Cl)OCC#N)OC)/S2
ZINC01012452	4.54	3.96	-18.61	0	6	0	459.292	8	CCOC(=O)COc1ccc(cc1OC)/C=C/2\C=C(OC2=O)c3ccc(cc3)Br
ZINC00627695	4.48	-0.56	-70.88	2	6	-1	428.251	5	c1ccc(c(c1)C(=O)Nc2ccccc2C(=O)Nc3ccc(c(c3)Cl)Cl)C(=O)[O-]
ZINC02167711	6.57	14.51	-12.07	0	7	0	497.576	8	CCOC(=O)c1ccc(cc1)/N=C/2\N(C(=O)/C(=C/c3ccccc3OCc4ccccc4C#N)/S2)C
ZINC02167711	6.57	14.92	-11.86	0	7	0	497.576	8	CCOC(=O)c1ccc(cc1)/N=C\2/N(C(=O)/C(=C/c3ccccc3OCc4ccccc4C#N)/S2)C
ZINC00627694	3.31	12.57	-72.32	1	7	-1	442.495	5	Cc1ccc(cc1)N2CCN(CC2)C(=O)c3ccccc3NC(=O)c4ccccc4C(=O)[O-]
ZINC08438929	6.47	2.05	-10.55	0	7	0	481.618	10	CCN(CC)c1ccc(c(c1)OCC)/C=C\2/C(=O)N(/C(=N\c3ccc(cc3)C(=O)OCC)/S2)C
ZINC00627687	3.26	13.37	-70.72	1	7	-1	442.495	5	Cc1ccccc1N2CCN(CC2)C(=O)c3ccccc3NC(=O)c4ccccc4C(=O)[O-]
ZINC08438928	6.43	0.76	-9.67	0	6	0	489.391	7	CCOc1ccc(cc1/C=C\2/C(=O)N(/C(=N\c3ccc(cc3)C(=O)OCC)/S2)C)Br
ZINC02167675	6.08	12.51	-11.86	0	6	0	454.935	7	CCOC(=O)c1ccc(cc1)/N=C\2/N(C(=O)/C(=C/c3ccc(c(c3)Cl)OCC#C)/S2)C
ZINC02167675	6.08	12.04	-12.43	0	6	0	454.935	7	CCOC(=O)c1ccc(cc1)/N=C/2\N(C(=O)/C(=C/c3ccc(c(c3)Cl)OCC#C)/S2)C
ZINC08438927	5.18	-0.11	-11.36	1	7	0	426.494	7	CCOc1cccc(c1O)/C=C\2/C(=O)N(/C(=N\c3ccc(cc3)C(=O)OCC)/S2)C
ZINC08438926	5.14	2.84	-17.78	0	9	0	498.557	11	CCOC(=O)COc1ccc(cc1OC)/C=C\2/C(=O)N(/C(=N\c3ccc(cc3)C(=O)OCC)/S2)C
ZINC08438925	6.9	1.38	-9.95	0	6	0	479.385	7	CCOc1c(cc(cc1Cl)/C=C\2/C(=O)N(/C(=N\c3ccc(cc3)C(=O)OCC)/S2)C)Cl
ZINC01012434	5.98	8.58	-10.7	1	6	0	461.337	5	CCOC(=O)c1ccc(cc1)/N=C/2\N(C(=O)/C(=C/c3cc(ccc3O)Br)/S2)C
ZINC01012434	5.98	8.99	-10.84	1	6	0	461.337	5	CCOC(=O)c1ccc(cc1)/N=C\2/N(C(=O)/C(=C/c3cc(ccc3O)Br)/S2)C
ZINC01012430	5.43	8.5	-11.37	1	7	0	446.912	6	CCOC(=O)c1ccc(cc1)/N=C/2\N(C(=O)/C(=C/c3cc(c(c(c3)Cl)O)OC)/S2)C
ZINC01012430	5.43	8.95	-11.1	1	7	0	446.912	6	CCOC(=O)c1ccc(cc1)/N=C\2/N(C(=O)/C(=C/c3cc(c(c(c3)Cl)O)OC)/S2)C
ZINC00627551	4.64	-0.78	-67.37	2	6	-1	434.299	5	c1ccc(c(c1)C(=O)Nc2ccc(c(c2)Cl)Cl)NC(=O)[C@H]3CCCC[C@H]3C(=O)[O-]
ZINC00627553	4.64	-0.66	-69.29	2	6	-1	434.299	5	c1ccc(c(c1)C(=O)Nc2ccc(c(c2)Cl)Cl)NC(=O)[C@@H]3CCCC[C@H]3C(=O)[O-]
ZINC08438924	5.81	-0.24	-12.16	1	7	0	460.939	7	CCOc1cc(cc(c1O)Cl)/C=C\2/C(=O)N(/C(=N\c3ccc(cc3)C(=O)OCC)/S2)C
ZINC00627555	4.64	-0.6	-68.47	2	6	-1	434.299	5	c1ccc(c(c1)C(=O)Nc2ccc(c(c2)Cl)Cl)NC(=O)[C@H]3CCCC[C@@H]3C(=O)[O-]
ZINC01012422	6.77	13.61	-11.39	0	6	0	464.568	7	CCOC(=O)c1ccc(cc1)/N=C/2\N(C(=O)/C(=C/c3ccc(o3)Sc4ccccc4)/S2)C
ZINC01012422	6.77	13.98	-12.32	0	6	0	464.568	7	CCOC(=O)c1ccc(cc1)/N=C\2/N(C(=O)/C(=C/c3ccc(o3)Sc4ccccc4)/S2)C
ZINC00627559	4.64	-0.79	-67.11	2	6	-1	434.299	5	c1ccc(c(c1)C(=O)Nc2ccc(c(c2)Cl)Cl)NC(=O)[C@@H]3CCCC[C@@H]3C(=O)[O-]
ZINC02167597	4.15	1.37	-55.67	0	9	-1	469.495	9	CCOC(=O)c1ccc(cc1)/N=C/2\N(C(=O)/C(=C/c3ccc(c(c3)OC)OCC(=O)[O-])/S2)C
ZINC00627481	4.04	13.53	-52.6	1	7	-1	474.581	6	CC(C)c1ccc(cc1)N2CCN(CC2)C(=O)c3ccccc3NC(=O)[C@H]4CC=CC[C@H]4C(=O)[O-]
ZINC08438923	4.69	11.08	-17.21	0	8	0	451.504	8	CCOC(=O)c1ccc(cc1)/N=C\2/N(C(=O)/C(=C/c3ccc(c(c3)OC)OCC#N)/S2)C
ZINC08438923	4.69	10.62	-18.28	0	8	0	451.504	8	CCOC(=O)c1ccc(cc1)/N=C/2\N(C(=O)/C(=C/c3ccc(c(c3)OC)OCC#N)/S2)C
ZINC00627483	4.04	13.37	-68.54	1	7	-1	474.581	6	CC(C)c1ccc(cc1)N2CCN(CC2)C(=O)c3ccccc3NC(=O)[C@@H]4CC=CC[C@H]4C(=O)[O-]
ZINC00627488	4.04	13.78	-58.3	1	7	-1	474.581	6	CC(C)c1ccc(cc1)N2CCN(CC2)C(=O)c3ccccc3NC(=O)[C@H]4CC=CC[C@@H]4C(=O)[O-]
ZINC02167569	4.92	0.83	-42.18	0	9	-1	426.43	6	CCOC(=O)c1ccc(cc1)/N=C/2\N(C(=O)/C(=C/c3ccc(c(c3)[N+](=O)[O-])[O-])/S2)C
ZINC00627492	4.04	13.5	-60.23	1	7	-1	474.581	6	CC(C)c1ccc(cc1)N2CCN(CC2)C(=O)c3ccccc3NC(=O)[C@@H]4CC=CC[C@@H]4C(=O)[O-]
ZINC02167561	6.38	9.13	-9.75	1	6	0	495.782	5	CCOC(=O)c1ccc(cc1)/N=C/2\N(C(=O)/C(=C/c3cc(cc(c3O)Cl)Br)/S2)C
ZINC02167561	6.38	9.54	-9.6	1	6	0	495.782	5	CCOC(=O)c1ccc(cc1)/N=C\2/N(C(=O)/C(=C/c3cc(cc(c3O)Cl)Br)/S2)C
ZINC02167561	6.38	10.32	-35.63	0	6	-1	494.774	5	CCOC(=O)c1ccc(cc1)/N=C\2/N(C(=O)/C(=C/c3cc(cc(c3[O-])Cl)Br)/S2)C
ZINC02167561	6.38	9.9	-34.21	0	6	-1	494.774	5	CCOC(=O)c1ccc(cc1)/N=C/2\N(C(=O)/C(=C/c3cc(cc(c3[O-])Cl)Br)/S2)C
ZINC08438922	5.06	2.89	-18.17	0	8	0	465.531	9	CCOc1cc(ccc1OCC#N)/C=C\2/C(=O)N(/C(=N\c3ccc(cc3)C(=O)OCC)/S2)C
ZINC08438921	5.28	1.78	-12	0	7	0	440.521	8	CCOc1ccc(cc1OC)/C=C\2/C(=O)N(/C(=N\c3ccc(cc3)C(=O)OCC)/S2)C
ZINC08438919	6.54	3.77	-10.66	0	6	0	473.598	5	Cc1ccc(c(c1)C)n2c(cc(c2C)/C=C\3/C(=O)N(/C(=N\c4ccc(cc4)C(=O)OC)/S3)C)C
ZINC08438918	6.42	3.3	-10.74	0	6	0	473.598	6	CCc1ccc(cc1)n2c(cc(c2C)/C=C\3/C(=O)N(/C(=N\c4ccc(cc4)C(=O)OC)/S3)C)C
ZINC08438917	5.67	3.56	-11.39	0	6	0	463.534	5	Cc1cc(c(n1c2ccc(cc2)F)C)/C=C\3/C(=O)N(/C(=N\c4ccc(cc4)C(=O)OC)/S3)C
ZINC08438916	6.12	3.45	-10.95	0	6	0	459.571	5	Cc1ccccc1n2c(cc(c2C)/C=C\3/C(=O)N(/C(=N\c4ccc(cc4)C(=O)OC)/S3)C)C
ZINC08438914	5.61	2.79	-11.64	0	7	0	488.613	6	Cc1cc(c(n1c2ccc(cc2)N(C)C)C)/C=C\3/C(=O)N(/C(=N\c4ccc(cc4)C(=O)OC)/S3)C
ZINC08438913	5.57	2.83	-11.97	0	7	0	475.57	6	Cc1cc(c(n1c2ccc(cc2)OC)C)/C=C\3/C(=O)N(/C(=N\c4ccc(cc4)C(=O)OC)/S3)C
ZINC08438912	6.14	3.14	-11.11	0	6	0	459.571	5	Cc1cccc(c1)n2c(cc(c2C)/C=C\3/C(=O)N(/C(=N\c4ccc(cc4)C(=O)OC)/S3)C)C
ZINC00627464	2.98	12.28	-52.89	1	7	-1	446.527	5	Cc1ccc(cc1)N2CCN(CC2)C(=O)c3ccccc3NC(=O)[C@H]4CC=CC[C@H]4C(=O)[O-]
ZINC08438911	5.84	3.66	-13.1	0	6	0	463.534	5	Cc1cc(c(n1c2ccccc2F)C)/C=C\3/C(=O)N(/C(=N\c4ccc(cc4)C(=O)OC)/S3)C
ZINC00627467	2.98	12.12	-68.81	1	7	-1	446.527	5	Cc1ccc(cc1)N2CCN(CC2)C(=O)c3ccccc3NC(=O)[C@@H]4CC=CC[C@H]4C(=O)[O-]
ZINC08438910	5.66	2.84	-19.73	0	9	0	490.541	6	Cc1cc(c(n1c2cccc(c2)[N+](=O)[O-])C)/C=C\3/C(=O)N(/C(=N\c4ccc(cc4)C(=O)OC)/S3)C
ZINC08438909	4.76	2.93	-18.38	0	9	0	484.53	10	CCOc1cc(ccc1OCC(=O)OC)/C=C\2/C(=O)N(/C(=N\c3ccc(cc3)C(=O)OC)/S2)C
ZINC00627468	2.98	12.53	-58.55	1	7	-1	446.527	5	Cc1ccc(cc1)N2CCN(CC2)C(=O)c3ccccc3NC(=O)[C@H]4CC=CC[C@@H]4C(=O)[O-]

Supplementary Table S2:
Detailed table of XP Docking using Schrodinger's Glide module

ZINC_ID	xLogP	ApolarDesolvation	PolarDesolvation	H Donors	H Acceptors	Charge	MolecularWeight	RotatableBonds	SMILES
ZINC00627471	2.98	12.25	-60.56	1	7	-1	446.527	5	Cc1ccc(cc1)N2CCN(CC2)C(=O)c3ccccc3NC(=O)[C@@H]4CC=CC[C@@H]4C(=O)[O-]
ZINC01012358	6.19	3.09	-12.39	0	7	0	483.549	7	CN\1C(=O)/C(=C/c2ccccc2OCc3ccccc3C#N)/S/C1=N/c4ccc(cc4)C(=O)OC
ZINC08438908	6.09	2.08	-10.65	0	7	0	467.591	9	CCN(CC)c1ccc(c(c1)OCC)/C=C\2/C(=O)N(/C(=N\c3ccc(cc3)C(=O)OC)/S2)C
ZINC00627452	2.96	12.27	-52.99	1	7	-1	446.527	5	Cc1cccc(c1)N2CCN(CC2)C(=O)c3ccccc3NC(=O)[C@H]4CC=CC[C@H]4C(=O)[O-]
ZINC00627455	2.96	12.11	-68.88	1	7	-1	446.527	5	Cc1cccc(c1)N2CCN(CC2)C(=O)c3ccccc3NC(=O)[C@@H]4CC=CC[C@H]4C(=O)[O-]
ZINC08438907	6.52	3.68	-10.82	0	6	0	473.598	5	Cc1cccc(c1C)n2c(cc(c2C)/C=C\3/C(=O)N(/C(=N\c4ccc(cc4)C(=O)OC)/S3)C)C
ZINC00627458	2.96	12.52	-58.57	1	7	-1	446.527	5	Cc1cccc(c1)N2CCN(CC2)C(=O)c3ccccc3NC(=O)[C@H]4CC=CC[C@@H]4C(=O)[O-]
ZINC08438906	6.05	0.79	-9.94	0	6	0	475.364	6	CCOc1ccc(cc1/C=C\2/C(=O)N(/C(=N\c3ccc(cc3)C(=O)OC)/S2)C)Br
ZINC00627460	2.96	12.24	-60.63	1	7	-1	446.527	5	Cc1cccc(c1)N2CCN(CC2)C(=O)c3ccccc3NC(=O)[C@@H]4CC=CC[C@@H]4C(=O)[O-]
ZINC01012347	5.52	2.42	-16.77	0	9	0	462.487	6	CN\1C(=O)/C(=C/c2cccn2c3cccc(c3)[N+](=O)[O-])/S/C1=N/c4ccc(cc4)C(=O)OC
ZINC00627441	2.93	13.17	-52.98	1	7	-1	446.527	5	Cc1ccccc1N2CCN(CC2)C(=O)c3ccccc3NC(=O)[C@H]4CC=CC[C@H]4C(=O)[O-]
ZINC02167345	5.97	2.28	-12.42	0	7	0	472.95	8	CN\1C(=O)/C(=C/c2cc(c(c(c2)Cl)OCC=C)OC)/S/C1=N/c3ccc(cc3)C(=O)OC
ZINC00627444	2.93	12.93	-67.16	1	7	-1	446.527	5	Cc1ccccc1N2CCN(CC2)C(=O)c3ccccc3NC(=O)[C@@H]4CC=CC[C@H]4C(=O)[O-]
ZINC01012339	5.61	-1.13	-10.8	1	6	0	447.31	4	CN\1C(=O)/C(=C/c2cc(ccc2O)Br)/S/C1=N/c3ccc(cc3)C(=O)OC
ZINC00627446	2.93	13.32	-57.34	1	7	-1	446.527	5	Cc1ccccc1N2CCN(CC2)C(=O)c3ccccc3NC(=O)[C@H]4CC=CC[C@@H]4C(=O)[O-]
ZINC01012331	6.39	1.2	-12.27	0	6	0	450.541	6	CN\1C(=O)/C(=C/c2ccc(o2)Sc3ccccc3)/S/C1=N/c4ccc(cc4)C(=O)OC
ZINC00627449	2.93	12.74	-61.08	1	7	-1	446.527	5	Cc1ccccc1N2CCN(CC2)C(=O)c3ccccc3NC(=O)[C@@H]4CC=CC[C@@H]4C(=O)[O-]
ZINC02167297	3.77	1.4	-55.99	0	9	-1	455.468	8	CN\1C(=O)/C(=C/c2ccc(c(c2)OC)OCC(=O)[O-])/S/C1=N/c3ccc(cc3)C(=O)OC
ZINC08438905	5.49	2.94	-14.1	0	7	0	470.934	7	CN\1C(=O)/C(=C/c2cc(c(c(c2)Cl)OCC#C)OC)/S/C1=N/c3ccc(cc3)C(=O)OC
ZINC02167279	6.31	2.52	-11.97	0	6	0	475.353	6	CN\1C(=O)/C(=C/c2cc(c(c(c2)Cl)OCC#C)Cl)/S/C1=N/c3ccc(cc3)C(=O)OC
ZINC01019550	4.13	10.67	-53.94	2	6	-1	432.283	5	c1ccc(c(c1)C(=O)Nc2cccc(c2Cl)Cl)NC(=O)[C@H]3CC=CC[C@H]3C(=O)[O-]
ZINC01019551	4.13	10.8	-63.84	2	6	-1	432.283	5	c1ccc(c(c1)C(=O)Nc2cccc(c2Cl)Cl)NC(=O)[C@@H]3CC=CC[C@H]3C(=O)[O-]
ZINC08438903	4.13	11.17	-57.77	2	6	-1	432.283	5	c1ccc(c(c1)C(=O)Nc2cccc(c2Cl)Cl)NC(=O)[C@H]3CC=CC[C@@H]3C(=O)[O-]
ZINC02167211	5.68	-0.92	-55.82	0	6	-1	480.747	4	CN\1C(=O)/C(=C/c2cc(ccc2OC)Br)/S/C1=N/c3ccc(c(c3)C(=O)[O-])Cl
ZINC01019553	4.13	11.12	-53.06	2	6	-1	432.283	5	c1ccc(c(c1)C(=O)Nc2cccc(c2Cl)Cl)NC(=O)[C@@H]3CC=CC[C@@H]3C(=O)[O-]
ZINC08438901	3.19	12.02	-64.1	1	7	-1	452.506	5	c1ccc(c(c1)C(=O)N2CCN(CC2)c3ccc(cc3)F)NC(=O)[C@H]4CCCC[C@H]4C(=O)[O-]
ZINC08438900	3.19	11.61	-65.38	1	7	-1	452.506	5	c1ccc(c(c1)C(=O)N2CCN(CC2)c3ccc(cc3)F)NC(=O)[C@@H]4CCCC[C@H]4C(=O)[O-]
ZINC00627368	3.19	12.26	-65.01	1	7	-1	452.506	5	c1ccc(c(c1)C(=O)N2CCN(CC2)c3ccc(cc3)F)NC(=O)[C@H]4CCCC[C@@H]4C(=O)[O-]
ZINC08438899	3.19	11.41	-62.77	1	7	-1	452.506	5	c1ccc(c(c1)C(=O)N2CCN(CC2)c3ccc(cc3)F)NC(=O)[C@@H]4CCCC[C@@H]4C(=O)[O-]
ZINC00627359	3.03	11.72	-61.8	1	8	-1	464.542	6	COc1ccccc1N2CCN(CC2)C(=O)c3ccccc3NC(=O)[C@H]4CCCC[C@H]4C(=O)[O-]
ZINC08438898	4.76	11.84	-17.36	0	9	0	484.53	10	CCOC(=O)c1ccc(cc1)/N=C\2/N(C(=O)/C(=C/c3ccc(c(c3)OC)OCC(=O)OC)/S2)C
ZINC08438898	4.76	11.38	-18.17	0	9	0	484.53	10	CCOC(=O)c1ccc(cc1)/N=C/2\N(C(=O)/C(=C/c3ccc(c(c3)OC)OCC(=O)OC)/S2)C
ZINC00627361	3.03	11.31	-69.72	1	8	-1	464.542	6	COc1ccccc1N2CCN(CC2)C(=O)c3ccccc3NC(=O)[C@@H]4CCCC[C@H]4C(=O)[O-]
ZINC00627363	3.03	11.96	-63.14	1	8	-1	464.542	6	COc1ccccc1N2CCN(CC2)C(=O)c3ccccc3NC(=O)[C@H]4CCCC[C@@H]4C(=O)[O-]
ZINC08438897	5.32	0.5	-50.04	0	7	-1	439.513	9	CCCN1C(=O)/C(=C/c2ccc(cc2)OCC(=O)[O-])/SC1=Nc3ccc(cc3)OCC
ZINC00627365	3.03	11.11	-67.68	1	8	-1	464.542	6	COc1ccccc1N2CCN(CC2)C(=O)c3ccccc3NC(=O)[C@@H]4CCCC[C@@H]4C(=O)[O-]
ZINC00627348	3.47	12.62	-60.07	1	7	-1	448.543	5	Cc1ccc(cc1)N2CCN(CC2)C(=O)c3ccccc3NC(=O)[C@H]4CCCC[C@H]4C(=O)[O-]
ZINC00627350	3.47	12.18	-68.98	1	7	-1	448.543	5	Cc1ccc(cc1)N2CCN(CC2)C(=O)c3ccccc3NC(=O)[C@@H]4CCCC[C@H]4C(=O)[O-]
ZINC08438894	5.69	-0.16	-41.99	0	8	-1	426.474	7	CCCN1C(=O)/C(=C/c2ccc(c(c2)[N+](=O)[O-])[O-])/SC1=Nc3ccc(cc3)OCC
ZINC00627353	3.47	12.84	-61.66	1	7	-1	448.543	5	Cc1ccc(cc1)N2CCN(CC2)C(=O)c3ccccc3NC(=O)[C@H]4CCCC[C@@H]4C(=O)[O-]
ZINC00627356	3.47	11.98	-67.05	1	7	-1	448.543	5	Cc1ccc(cc1)N2CCN(CC2)C(=O)c3ccccc3NC(=O)[C@@H]4CCCC[C@@H]4C(=O)[O-]
ZINC00627339	3.45	12.61	-60.08	1	7	-1	448.543	5	Cc1cccc(c1)N2CCN(CC2)C(=O)c3ccccc3NC(=O)[C@H]4CCCC[C@H]4C(=O)[O-]
ZINC00627341	3.45	12.17	-69.08	1	7	-1	448.543	5	Cc1cccc(c1)N2CCN(CC2)C(=O)c3ccccc3NC(=O)[C@@H]4CCCC[C@H]4C(=O)[O-]
ZINC00627343	3.45	12.84	-61.66	1	7	-1	448.543	5	Cc1cccc(c1)N2CCN(CC2)C(=O)c3ccccc3NC(=O)[C@H]4CCCC[C@@H]4C(=O)[O-]
ZINC00627346	3.45	11.97	-67.11	1	7	-1	448.543	5	Cc1cccc(c1)N2CCN(CC2)C(=O)c3ccccc3NC(=O)[C@@H]4CCCC[C@@H]4C(=O)[O-]
ZINC08438888	3.86	10.93	-61.71	1	6	-1	419.288	5	c1cc(ccc1NC(=O)/C=C\C(=O)[O-])N2CCN(CC2)c3ccc(c(c3)Cl)Cl
ZINC08438886	3.83	-4.03	-66.68	1	6	-1	419.288	5	c1cc(c(c(c1)Cl)Cl)N2CCN(CC2)c3ccc(cc3)NC(=O)C=CC(=O)[O-]
ZINC08438884	3.57	3.79	-14.58	0	5	0	253.261	3	Cc1ccc(cc1)C(=O)On2c3ccccc3nn2
ZINC00627177	5.47	-3.87	-66.41	1	6	-1	475.396	5	c1cc(c(c(c1)Cl)Cl)N2CCN(CC2)c3ccc(cc3)NC(=O)[C@H]4CCCC[C@H]4C(=O)[O-]
ZINC00627180	5.47	-3.7	-65.44	1	6	-1	475.396	5	c1cc(c(c(c1)Cl)Cl)N2CCN(CC2)c3ccc(cc3)NC(=O)[C@@H]4CCCC[C@H]4C(=O)[O-]
ZINC00627181	5.47	-3.68	-64.41	1	6	-1	475.396	5	c1cc(c(c(c1)Cl)Cl)N2CCN(CC2)c3ccc(cc3)NC(=O)[C@H]4CCCC[C@@H]4C(=O)[O-]
ZINC00627184	5.47	-3.88	-64.91	1	6	-1	475.396	5	c1cc(c(c(c1)Cl)Cl)N2CCN(CC2)c3ccc(cc3)NC(=O)[C@@H]4CCCC[C@@H]4C(=O)[O-]
ZINC08438879	4.88	-4.28	-64.03	1	6	-1	440.951	5	c1cc(ccc1NC(=O)[C@H]2CCCC[C@H]2C(=O)[O-])N3CCN(CC3)c4ccc(cc4)Cl
ZINC02166906	6.83	2.32	-10.84	0	6	0	483.593	8	CCN\1C(=O)/C(=C/c2ccccc2OCc3ccccc3C#N)/S/C1=N/c4ccc(cc4)OCC
ZINC08438878	4.88	-4.1	-62.67	1	6	-1	440.951	5	c1cc(ccc1NC(=O)[C@@H]2CCCC[C@H]2C(=O)[O-])N3CCN(CC3)c4ccc(cc4)Cl
ZINC08438876	4.88	-4.09	-62.64	1	6	-1	440.951	5	c1cc(ccc1NC(=O)[C@H]2CCCC[C@@H]2C(=O)[O-])N3CCN(CC3)c4ccc(cc4)Cl
ZINC01012227	6.31	0.01	-8.93	0	5	0	461.381	6	CCN\1C(=O)/C(=C/c2cc(ccc2OC)Br)/S/C1=N/c3ccc(cc3)OCC
ZINC08438875	4.88	-4.28	-64.25	1	6	-1	440.951	5	c1cc(ccc1NC(=O)[C@@H]2CCCC[C@@H]2C(=O)[O-])N3CCN(CC3)c4ccc(cc4)Cl
ZINC02166882	7.37	1.16	-10.3	0	5	0	452.963	6	CCN\1C(=O)/C(=C/c2ccc(o2)c3ccc(cc3)Cl)/S/C1=N/c4ccc(cc4)OCC
ZINC01012211	6.16	1.56	-14.87	0	8	0	462.531	7	CCN\1C(=O)/C(=C/c2cccn2c3cccc(c3)[N+](=O)[O-])/S/C1=N/c4ccc(cc4)OCC
ZINC00627106	4.97	-3.66	-49.87	1	6	-1	473.38	5	c1cc(c(c(c1)Cl)Cl)N2CCN(CC2)c3ccc(cc3)NC(=O)[C@H]4CC=CC[C@H]4C(=O)[O-]

Supplementary Table S2:
Detailed table of XP Docking using Schrodinger's Glide module

ZINC_ID	xLogP	ApolarDesolvation	PolarDesolvation	H Donors	H Acceptors	Charge	MolecularWeight	RotatableBonds	SMILES
ZINC00627108	4.97	-3.53	-63.15	1	6	-1	473.38	5	c1cc(c(c(c1)Cl)Cl)N2CCN(CC2)c3ccc(cc3)NC(=O)[C@@H]4CC=CC[C@H]4C(=O)[O-]
ZINC00627111	4.97	-3.55	-63.97	1	6	-1	473.38	5	c1cc(c(c(c1)Cl)Cl)N2CCN(CC2)c3ccc(cc3)NC(=O)[C@H]4CC=CC[C@@H]4C(=O)[O-]
ZINC00627114	4.97	-3.61	-49.39	1	6	-1	473.38	5	c1cc(c(c(c1)Cl)Cl)N2CCN(CC2)c3ccc(cc3)NC(=O)[C@@H]4CC=CC[C@@H]4C(=O)[O-]
ZINC08438868	5.21	0.68	-56.62	0	8	-1	489.957	9	CCN1C(=O)/C(=C/c2cc(c(c(c2)Cl)OCC(=O)[O-])OC)/SC1=Nc3ccc(cc3)OCC
ZINC08438867	4.39	-4.06	-48.46	1	6	-1	438.935	5	c1cc(ccc1NC(=O)[C@H]2CC=CC[C@H]2C(=O)[O-])N3CCN(CC3)c4ccc(cc4)Cl
ZINC08438866	6.99	1.5	-10.31	0	6	0	487.021	10	CCN\1C(=O)/C(=C/c2cc(c(c(c2)Cl)OCC=C)OCC)/S/C1=N/c3ccc(cc3)OCC
ZINC08438865	4.39	-3.93	-62.53	1	6	-1	438.935	5	c1cc(ccc1NC(=O)[C@@H]2CC=CC[C@H]2C(=O)[O-])N3CCN(CC3)c4ccc(cc4)Cl
ZINC01011974	6.24	-1.97	-9.28	1	5	0	447.354	5	CCN\1C(=O)/C(=C/c2cc(ccc2O)Br)/S/C1=N/c3ccc(cc3)OCC
ZINC08438864	4.39	-3.95	-62.29	1	6	-1	438.935	5	c1cc(ccc1NC(=O)[C@H]2CC=CC[C@@H]2C(=O)[O-])N3CCN(CC3)c4ccc(cc4)Cl
ZINC01011970	6.07	-0.99	-10.53	1	6	0	446.956	7	CCN\1C(=O)/C(=C/c2cc(c(c(c2)Cl)O)OCC)/S/C1=N/c3ccc(cc3)OCC
ZINC08438863	4.39	-4	-48.31	1	6	-1	438.935	5	c1cc(ccc1NC(=O)[C@@H]2CC=CC[C@@H]2C(=O)[O-])N3CCN(CC3)c4ccc(cc4)Cl
ZINC00627094	4.37	-4.06	-48.49	1	6	-1	438.935	5	c1cc(cc(c1)Cl)N2CCN(CC2)c3ccc(cc3)NC(=O)[C@H]4CC=CC[C@H]4C(=O)[O-]
ZINC02166766	7.03	0.52	-9.84	0	5	0	450.585	7	CCN\1C(=O)/C(=C/c2ccc(o2)Sc3ccccc3)/S/C1=N/c4ccc(cc4)OCC
ZINC00627096	4.37	-3.95	-63.19	1	6	-1	438.935	5	c1cc(cc(c1)Cl)N2CCN(CC2)c3ccc(cc3)NC(=O)[C@@H]4CC=CC[C@H]4C(=O)[O-]
ZINC00627097	4.37	-3.95	-63.34	1	6	-1	438.935	5	c1cc(cc(c1)Cl)N2CCN(CC2)c3ccc(cc3)NC(=O)[C@H]4CC=CC[C@@H]4C(=O)[O-]
ZINC00627098	4.37	-4	-48.66	1	6	-1	438.935	5	c1cc(cc(c1)Cl)N2CCN(CC2)c3ccc(cc3)NC(=O)[C@@H]4CC=CC[C@@H]4C(=O)[O-]
ZINC08438861	4.41	0.69	-54.71	0	8	-1	455.512	9	CCN1C(=O)/C(=C/c2ccc(c(c2)OC)OCC(=O)[O-])/SC1=Nc3ccc(cc3)OCC
ZINC00627061	5.33	-4.2	-65.69	1	6	-1	469.348	5	c1ccc(c(c1)C(=O)Nc2ccc(cc2)N3CCN(CC3)c4ccc(c(c4)Cl)Cl)C(=O)[O-]
ZINC00627058	5.3	-3.66	-66.39	1	6	-1	469.348	5	c1ccc(c(c1)C(=O)Nc2ccc(cc2)N3CCN(CC3)c4cccc(c4Cl)Cl)C(=O)[O-]
ZINC00627056	4.72	-4.07	-64.79	1	6	-1	434.903	5	c1ccc(c(c1)C(=O)Nc2ccc(cc2)N3CCN(CC3)c4ccc(cc4)Cl)C(=O)[O-]
ZINC02166692	6.83	1.07	-11.29	0	5	0	442.968	8	CCN\1C(=O)/C(=C/c2ccc(c(c2)Cl)OCC=C)/S/C1=N/c3ccc(cc3)OCC
ZINC00627054	4.7	-4.06	-66.44	1	6	-1	434.903	5	c1ccc(c(c1)C(=O)Nc2ccc(cc2)N3CCN(CC3)c4cccc(c4)Cl)C(=O)[O-]
ZINC01011938	5.77	0.92	-8.99	0	6	0	446.956	7	CCN\1C(=O)/C(=C/c2cc(c(cc2Cl)OC)OC)/S/C1=N/c3ccc(cc3)OCC
ZINC08438858	3.61	-1.34	-79.55	2	6	0	441.531	7	c1ccc(cc1)C[NH+]2CCN(CC2)c3ccc(cc3)NC(=O)/C=C(\c4ccccc4)/C(=O)[O-]
ZINC08438856	4.07	-2.94	-51.99	1	6	-1	444.486	6	c1ccc(cc1)/C(=C\C(=O)Nc2ccc(cc2)N3CCN(CC3)c4ccc(cc4)F)/C(=O)[O-]
ZINC08438854	5.17	-3.42	-53.1	1	6	-1	495.386	6	c1ccc(cc1)/C(=C\C(=O)Nc2ccc(cc2)N3CCN(CC3)c4cccc(c4Cl)Cl)/C(=O)[O-]
ZINC08438852	4.59	-3.82	-51.45	1	6	-1	460.941	6	c1ccc(cc1)/C(=C\C(=O)Nc2ccc(cc2)N3CCN(CC3)c4ccc(cc4)Cl)/C(=O)[O-]
ZINC08438850	4.56	-3.82	-51.88	1	6	-1	460.941	6	c1ccc(cc1)/C(=C\C(=O)Nc2ccc(cc2)N3CCN(CC3)c4cccc(c4)Cl)/C(=O)[O-]
ZINC01011918	5.15	1.51	-11.63	0	7	0	442.537	8	CCN\1C(=O)/C(=C/c2cc(c(c(c2)OC)OC)OC)/S/C1=N/c3ccc(cc3)OCC
ZINC00627001	4.33	13.39	-51.38	1	6	-1	440.523	6	Cc1cccc(c1)N2CCN(CC2)c3ccc(cc3)NC(=O)/C=C(\c4ccccc4)/C(=O)[O-]
ZINC01011914	5.71	0.13	-9.2	0	5	0	421.316	5	CCN\1C(=O)/C(=C/c2ccc(o2)Br)/S/C1=N/c3ccc(cc3)OCC
ZINC02165416	6.31	-2.02	-10.52	1	5	0	494.354	5	CCN\1C(=O)/C(=C/c2ccc(c(c2)I)O)/S/C1=N/c3ccc(cc3)OCC
ZINC01011902	6.31	0.03	-11.4	0	5	0	461.381	6	CCN\1C(=O)/C(=C/c2ccc(c(c2)Br)OC)/S/C1=N/c3ccc(cc3)OCC
ZINC02165373	6.15	0.93	-8.75	0	6	0	460.983	8	CCN\1C(=O)/C(=C/c2cc(c(cc2Cl)OCC)OC)/S/C1=N/c3ccc(cc3)OCC
ZINC13111165	5.03	13.26	-11.93	0	7	0	460.559	6	CCOC(=O)c1ccc(cc1)/N=C\2/N(C(=O)/C(=C/c3cc(n(c3C)c4cccnc4)C)/S2)C
ZINC13111165	5.03	12.86	-12.16	0	7	0	460.559	6	CCOC(=O)c1ccc(cc1)/N=C/2\N(C(=O)/C(=C/c3cc(n(c3C)c4cccnc4)C)/S2)C
ZINC02165312	6.05	14.88	-11.09	0	6	0	477.561	6	CCOC(=O)c1ccc(cc1)/N=C/2\N(C(=O)/C(=C/c3cc(n(c3C)c4ccc(cc4)F)C)/S2)C
ZINC02165312	6.05	15.28	-10.8	0	6	0	477.561	6	CCOC(=O)c1ccc(cc1)/N=C\2/N(C(=O)/C(=C/c3cc(n(c3C)c4ccc(cc4)F)C)/S2)C
ZINC02165306	6.52	15.48	-10.8	0	6	0	473.598	6	CCOC(=O)c1ccc(cc1)/N=C/2\N(C(=O)/C(=C/c3cc(n(c3C)c4cccc(c4)C)C)/S2)C
ZINC02165306	6.52	15.88	-10.76	0	6	0	473.598	6	CCOC(=O)c1ccc(cc1)/N=C\2/N(C(=O)/C(=C/c3cc(n(c3C)c4cccc(c4)C)C)/S2)C
ZINC08438840	7.03	1.48	-14	1	4	0	436.507	9	c1ccc(cc1)COc2ccc(cc2)/C=C/C(=O)c3ccc(cc3O)OCc4ccccc4
ZINC02165295	6.21	14.93	-12.77	0	6	0	477.561	6	CCOC(=O)c1ccc(cc1)/N=C/2\N(C(=O)/C(=C/c3cc(n(c3C)c4ccccc4F)C)/S2)C
ZINC02165295	6.21	15.33	-12.69	0	6	0	477.561	6	CCOC(=O)c1ccc(cc1)/N=C\2/N(C(=O)/C(=C/c3cc(n(c3C)c4ccccc4F)C)/S2)C
ZINC08438839	7.1	3.4	-14.62	0	4	0	450.534	10	COc1cc(ccc1C(=O)/C=C/c2ccc(cc2)OCc3ccccc3)OCc4ccccc4
ZINC08438837	3.8	-7.27	-10.59	1	6	0	424.979	5	c1cc(ccc1NC(=S)c2c(nc(s2)N3CCOCC3)N4CCOCC4)Cl
ZINC02165282	4.93	1.73	-12.81	0	5	0	451.931	6	COc1cc(ccc1OCc2ccccc2)/C=C\3/C(=O)N(C(=O)S3)c4cccc(c4)Cl
ZINC01011872	3.77	-1.06	-9.32	1	5	0	440.702	3	COc1cc(c(cc1O)Br)/C=C\2/C(=O)N(C(=O)S2)c3cccc(c3)Cl
ZINC08438833	3.02	2.58	-52.38	0	6	-1	345.286	3	c1ccc(c(c1)C(=O)c2ccc3c4c2cccc4C(=O)OC3=O)C(=O)[O-]
ZINC02165265	5.17	3.52	-9.15	0	4	0	436.964	3	Cc1ccc(c(c1)C)n2c(cc(c2C)/C=C\3/C(=O)N(C(=O)S3)c4ccc(cc4)Cl)C
ZINC02184622	7.01	0.86	-10.03	0	4	0	422.509	5	COC(=O)c1c(c(c2c(c1c3ccccc3)nsn2)c4ccccc4)c5ccccc5
ZINC02165261	4.94	1.81	-9.44	0	4	0	487.806	3	Cc1cc(c(n1c2ccc(cc2)Br)C)/C=C\3/C(=O)N(C(=O)S3)c4ccc(cc4)Cl
ZINC08438832	6.09	1.65	-13.66	0	4	0	426.541	4	C[C@H]1[C@@H]([C@]2(c3nc4ccccc4n3[C@@]1(S2)c5ccccc5)c6ccccc6)C(=O)OC
ZINC02165256	4.81	2.42	-9.5	0	4	0	443.355	3	Cc1cc(c(n1c2ccc(cc2)Cl)C)/C=C\3/C(=O)N(C(=O)S3)c4ccc(cc4)Cl
ZINC08438831	6.17	0.97	-10.8	1	4	0	426.541	5	CC1=C(S[C@]([C@H]1C(=O)OC)(c2ccccc2)c3[nH]c4ccccc4n3)c5ccccc5
ZINC08438830	5.36	3.78	-9.25	0	4	0	436.964	3	Cc1cccc(c1n2c(cc(c2C)/C=C\3/C(=O)N(C(=O)S3)c4ccc(cc4)Cl)C)C
ZINC00626914	5.36	1.73	-11.76	0	5	0	438.508	4	COC(=O)C1=C(S[C@]2([C@H]1C(=O)n3c2nc4c3cccc4)c5ccccc5)c6ccccc6
ZINC00626912	5.36	1.71	-12.85	0	5	0	438.508	4	COC(=O)C1=C(S[C@]2([C@@H]1C(=O)n3c2nc4c3cccc4)c5ccccc5)c6ccccc6
ZINC02165251	4.68	3.72	-12.37	0	7	0	467.934	4	Cc1cc(ccc1n2c(cc(c2C)/C=C\3/C(=O)N(C(=O)S3)c4ccc(cc4)Cl)C)[N+](=O)[O-]
ZINC02165246	4.46	3.41	-11.61	0	4	0	426.9	3	Cc1cc(c(n1c2ccccc2F)C)/C=C\3/C(=O)N(C(=O)S3)c4ccc(cc4)Cl
ZINC02184609	5.56	2.89	-25.73	1	2	1	428.555	3	c1ccc(cc1)C2=C(C(=O)[C@]3([C@H]2c4cccc5c4c3ccc5)[NH+]6CCCC6)c7ccccc7
ZINC02165240	4.28	3.1	-15.1	0	7	0	453.907	4	Cc1cc(c(n1c2cccc(c2)[N+](=O)[O-])C)/C=C\3/C(=O)N(C(=O)S3)c4ccc(cc4)Cl

Supplementary Table S2:
Detailed table of XP Docking using Schrodinger's Glide module

ZINC_ID	xLogP	ApolarDesolvation	PolarDesolvation	H Donors	H Acceptors	Charge	MolecularWeight	RotatableBonds	SMILES
ZINC02184607	5.56	2.24	-34.35	1	2	1	428.555	3	c1ccc(cc1)C2=C(C(=O)[C@@]3([C@H]2c4cccc5c4c3ccc5)[NH+]6CCCC6)c7ccccc7
ZINC01011866	3.01	2.64	-16.39	0	7	0	433.869	7	COc1cc(ccc1OCC(=O)OC)/C=C\2/C(=O)N(C(=O)S2)c3ccc(cc3)Cl
ZINC02165229	3.33	0.45	-32.13	0	8	-1	419.822	5	CCOc1cc(cc(c1[O-])/C=C\2/C(=O)N(C(=O)S2)c3ccc(cc3)Cl)[N+](=O)[O-]
ZINC00386658	2.09	8.09	-23.97	1	5	0	279.299	2	Cc1c(c(=O)n2ccccc2n1)NC(=O)c3ccccc3
ZINC01011863	4.3	0.56	-8.53	0	4	0	424.703	3	COc1ccc(cc1/C=C\2/C(=O)N(C(=O)S2)c3ccc(cc3)Cl)Br
ZINC02184604	8.67	2.35	-13.16	0	3	0	479.608	4	c1ccc(cc1)c2cc(c(nn2)c3ccccc3)c4c(cn5c4sc6c5cccc6)c7ccccc7
ZINC02165216	3.54	0.18	-46.16	0	6	-1	467.704	5	c1cc(ccc1N2C(=O)/C(=C/c3cc(ccc3OCC(=O)[O-])Br)/SC2=O)Cl
ZINC08438828	3.75	2.59	-10.72	0	5	0	328.279	0	c1ccc2c(c1)C(=O)c3cc4c5c(c3C2=O)cccc5C(=O)OC4=O
ZINC01011858	4.4	2.28	-14.77	0	6	0	452.315	7	CCOC(=O)COc1ccc(cc1Cl)/C=C\2/C(=O)N(C(=O)S2)c3ccc(cc3)Cl
ZINC02184593	7.46	3.03	-16.07	0	3	0	485.608	7	c1ccc(cc1)c2cn3c4ccccc4sc3c2[C@H](CC(=O)c5ccccc5)C(=O)c6ccccc6
ZINC02165211	4.67	0.53	-7.94	0	4	0	438.73	4	CCOc1ccc(cc1/C=C\2/C(=O)N(C(=O)S2)c3ccc(cc3)Cl)Br
ZINC02184592	7.46	3.06	-15.51	0	3	0	485.608	7	c1ccc(cc1)c2cn3c4ccccc4sc3c2[C@@H](CC(=O)c5ccccc5)C(=O)c6ccccc6
ZINC01011857	4.18	-1.1	-8.51	1	5	0	454.729	4	CCOc1cc(cc(c1O)/C=C\2/C(=O)N(C(=O)S2)c3ccc(cc3)Cl)Br
ZINC08438827	4.71	-0.94	-14.56	0	5	0	419.502	5	COc1ccc(cc1)N2C=C[C@@H]([C@H](S2(=O)=O)C(=O)c3ccccc3)c4ccccc4
ZINC01011854	3.39	2.61	-16.07	0	7	0	447.896	8	CCOC(=O)COc1ccc(cc1OC)/C=C\2/C(=O)N(C(=O)S2)c3ccc(cc3)Cl
ZINC08438826	5.42	2.07	-13.39	1	6	0	470.55	7	COC(=O)[C@@H]1C(=C(S[C@]1(c2ccccc2)c3[nH]c4ccccc4n3)c5ccccc5)C(=O)OC
ZINC02165195	4.58	2.89	-9.45	0	4	0	422.937	3	Cc1ccc(cc1)n2c(cc(c2C)/C=C\3/C(=O)N(C(=O)S3)c4ccc(cc4)Cl)C
ZINC02184584	8.1	1.47	-8.61	0	2	0	478.686	5	Cc1ccccc1C[C@]2(N(c3ccccc3S2)Cc4ccccc4C)c5nc6ccccc6s5
ZINC02165191	5.15	1.16	-8.8	0	4	0	428.724	4	CCOc1c(cc(cc1Cl)/C=C\2/C(=O)N(C(=O)S2)c3ccc(cc3)Cl)Cl
ZINC02184585	8.1	0.76	-8.72	0	2	0	478.686	5	Cc1ccccc1C[C@@]2(N(c3ccccc3S2)Cc4ccccc4C)c5nc6ccccc6s5
ZINC02165187	3.2	1.13	-54.57	0	7	-1	453.279	6	COc1cc(cc(c1OCC(=O)[O-])Cl)/C=C\2/C(=O)N(C(=O)S2)c3ccc(cc3)Cl
ZINC08438825	6.38	1.5	-7.59	0	2	0	453.335	3	c1ccc(cc1)[C@H]2[C@H]([C@@]23c4cccc5c4c(ccc5)C3=O)C(=O)c6ccc(cc6)Br
ZINC02165183	4.97	2.01	-9.21	0	5	0	450.343	7	CCOc1cc(cc(c1OCC=C)Cl)/C=C\2/C(=O)N(C(=O)S2)c3ccc(cc3)Cl
ZINC08438824	6.38	1.6	-9.51	0	2	0	453.335	3	c1ccc(cc1)[C@@H]2[C@H]([C@@]23c4cccc5c4c(ccc5)C3=O)C(=O)c6ccc(cc6)Br
ZINC02165177	4.86	1.19	-65.89	0	6	-1	459.286	4	c1cc(ccc1N2C(=O)/C(=C/c3ccc(o3)c4ccc(c(c4)C(=O)[O-])Cl)/SC2=O)Cl
ZINC08438823	6.38	2.35	-13.76	0	2	0	453.335	3	c1ccc(cc1)[C@H]2[C@@H]([C@@]23c4cccc5c4c(ccc5)C3=O)C(=O)c6ccc(cc6)Br
ZINC08438822	5.01	0.86	-10.99	0	4	0	413.907	4	c1ccc(cc1)Sc2ccc(o2)/C=C\3/C(=O)N(C(=O)S3)c4ccc(cc4)Cl
ZINC08438821	6.38	2.08	-14.42	0	2	0	453.335	3	c1ccc(cc1)[C@@H]2[C@@H]([C@@]23c4cccc5c4c(ccc5)C3=O)C(=O)c6ccc(cc6)Br
ZINC08438820	5.07	0.97	-56.79	0	6	-1	464.906	6	c1cc(ccc1COc2ccc(cc2)/C=C\3/C(=O)N(C(=O)S3)c4ccc(cc4)Cl)C(=O)[O-]
ZINC08438819	5.58	2.35	-14	0	6	0	470.55	6	COC(=O)[C@H]1[C@@H]([C@@]2(n3c4ccccc4nc3[C@]1(S2)c5ccccc5)c6ccccc6)C(=O)OC
ZINC02165159	5.47	2.69	-11.63	0	5	0	481.36	5	c1ccc(c(c1)COc2ccc(cc2Cl)/C=C\3/C(=O)N(C(=O)S3)c4ccc(cc4)Cl)C#N
ZINC02165155	5.26	2.5	-13.67	0	5	0	483.948	7	CCOc1cc(ccc1OCc2cccc(c2)F)/C=C\3/C(=O)N(C(=O)S3)c4ccc(cc4)Cl
ZINC02165148	5.42	1.61	-13.08	0	5	0	486.376	6	COc1cc(ccc1OCc2ccc(cc2)Cl)/C=C\3/C(=O)N(C(=O)S3)c4ccc(cc4)Cl
ZINC02184578	6.56	2.39	-14.77	0	2	0	424.499	3	c1ccc(cc1)C([C@@H]2c3cccc4c3c(ccc4)C2=O)[C@@H]5c6cccc7c6c(ccc7)C5=O
ZINC02165142	4.77	1.19	-6.37	0	4	0	414.697	3	COc1c(cc(cc1Cl)Cl)/C=C\2/C(=O)N(C(=O)S2)c3ccc(cc3)Cl
ZINC08438818	9.62	0.65	-14.17	0	2	0	556.734	4	c1ccc(cc1)C(=C2C=C[C@H]3[C@H]2[C@@]4(n5c6ccccc6nc5[C@]3(S4)c7ccccc7)c8ccccc8)c9ccccc9
ZINC02165138	4.93	2.25	-10.4	0	4	0	438.719	4	C#CCOc1c(cc(cc1Cl)/C=C\2/C(=O)N(C(=O)S2)c3ccc(cc3)Cl)Cl
ZINC08438817	9.62	0.96	-12.54	0	2	0	556.734	4	c1ccc(cc1)C(=C2C=C[C@@H]3[C@H]2[C@@]4(n5c6ccccc6nc5[C@]3(S4)c7ccccc7)c8ccccc8)c9ccccc9
ZINC02165122	4.76	-2.15	-7.71	1	4	0	489.572	2	c1cc(ccc1N2C(=O)/C(=C/c3cc(cc(c3O)Br)Br)/SC2=O)Cl
ZINC08438816	9.62	1.15	-12.55	0	2	0	556.734	4	c1ccc(cc1)C(=C2C=C[C@H]3[C@@H]2[C@@]4(n5c6ccccc6nc5[C@]3(S4)c7ccccc7)c8ccccc8)c9ccccc9
ZINC02165119	5.19	2.08	-12.72	0	5	0	465.958	6	Cc1ccc(cc1)COc2ccc(cc2OC)/C=C\3/C(=O)N(C(=O)S3)c4ccc(cc4)Cl
ZINC08438815	9.62	1.2	-12.38	0	2	0	556.734	4	c1ccc(cc1)C(=C2C=C[C@@H]3[C@@H]2[C@@]4(n5c6ccccc6nc5[C@]3(S4)c7ccccc7)c8ccccc8)c9ccccc9
ZINC02165111	5.57	2.06	-11.52	0	5	0	479.985	7	CCOc1cc(ccc1OCc2ccc(cc2)C)/C=C\3/C(=O)N(C(=O)S3)c4ccc(cc4)Cl
ZINC01592372	6.51	3.63	-2.62	0	0	0	240.39	2	CC(C)(C)c1ccc2cc(ccc2c1)C(C)(C)C
ZINC01011837	3.04	1.99	-9.52	0	8	0	420.83	5	COc1cc(c(cc1OC)[N+](=O)[O-])/C=C\2/C(=O)N(C(=O)S2)c3ccc(cc3)Cl
ZINC08438814	4.94	3	-34.5	0	8	0	598.703	6	CC1(N([C@H]([C@@H](N1C(=O)[C@H]2CC(=NO2)c3ccccc3)c4ccccc4)c5ccccc5)C(=O)[C@H]6CC(=NO6)c7ccccc7)C
ZINC02165104	4.26	0.85	-7.89	0	5	0	468.756	5	CCOc1cc(c(cc1OC)Br)/C=C\2/C(=O)N(C(=O)S2)c3ccc(cc3)Cl
ZINC02165100	4.9	0.57	-6.26	0	4	0	459.148	3	COc1c(cc(cc1Cl)Br)/C=C\2/C(=O)N(C(=O)S2)c3ccc(cc3)Cl
ZINC08438812	4.94	2.72	-30.41	0	8	0	598.703	6	CC1(N([C@H]([C@@H](N1C(=O)[C@H]2CC(=NO2)c3ccccc3)c4ccccc4)c5ccccc5)C(=O)[C@@H]6CC(=NO6)c7ccccc7)C
ZINC01011832	4.29	-1.49	-8.67	1	4	0	457.676	2	c1cc(ccc1N2C(=O)/C(=C/c3ccc(c(c3)I)O)/SC2=O)Cl
ZINC08438811	4.94	2.86	-23.08	0	8	0	598.703	6	CC1(N([C@H]([C@@H](N1C(=O)[C@@H]2CC(=NO2)c3ccccc3)c4ccccc4)c5ccccc5)C(=O)[C@@H]6CC(=NO6)c7ccccc7)C
ZINC02165086	4.84	0.98	-8.93	0	5	0	482.783	6	CCOc1cc(cc(c1OCC)Br)/C=C\2/C(=O)N(C(=O)S2)c3ccc(cc3)Cl
ZINC08438810	6.53	-0.77	-10.99	1	4	0	506.652	3	c1ccc(cc1)C(=O)C2=CN3c4ccccc4S[C@]([C@H]3[C@@H]5N2c6ccccc6S5)(c7ccccc7)O
ZINC02165080	4.46	1.02	-9.02	0	5	0	468.756	5	CCOc1cc(cc(c1OC)Br)/C=C\2/C(=O)N(C(=O)S2)c3ccc(cc3)Cl
ZINC08438809	6.53	-1.38	-12.59	1	4	0	506.652	3	c1ccc(cc1)C(=O)C2=CN3c4ccccc4S[C@@]([C@H]3[C@@H]5N2c6ccccc6S5)(c7ccccc7)O
ZINC02165076	4.26	0.85	-7.91	0	5	0	468.756	5	CCOc1cc(c(cc1OC)/C=C\2/C(=O)N(C(=O)S2)c3ccc(cc3)Cl)Br
ZINC08438808	6.53	-2.14	-13.94	1	4	0	506.652	3	c1ccc(cc1)C(=O)C2=CN3c4ccccc4S[C@]([C@@H]3[C@@H]5N2c6ccccc6S5)(c7ccccc7)O
ZINC08438807	6.53	13.87	-14.28	1	4	0	506.652	3	c1ccc(cc1)C(=O)C2=CN3c4ccccc4S[C@@]([C@@H]3[C@@H]5N2c6ccccc6S5)(c7ccccc7)O
ZINC02165071	4.83	3.1	-12.96	0	6	0	490.968	7	CCOc1cc(ccc1OCc2ccccc2C#N)/C=C\3/C(=O)N(C(=O)S3)c4ccc(cc4)Cl
ZINC08438806	8.02	0.41	-14.13	0	3	0	635.788	4	c1ccc(cc1)C(=O)[C@H]2[C@@]3(C(=C4c5ccccc5C(=O)c6c4cccc6)[C@@]3([C@@H]7N2c8ccccc8S7)c9ccccc9)c1ccccc1
ZINC01011831	4.28	0.63	-9.82	0	5	0	454.729	4	COc1cc(c(cc1/C=C\2/C(=O)N(C(=O)S2)c3ccc(cc3)Cl)Br)OC

Supplementary Table S2:
Detailed table of XP Docking using Schrodinger's Glide module

ZINC_ID	xLogP	ApolarDesolvation	PolarDesolvation	H Donors	H Acceptors	Charge	MolecularWeight	RotatableBonds	SMILES
ZINC08438805	8.02	0.79	-14.01	0	3	0	635.788	4	c1ccc(cc1)C(=O)[C@@H]2[C@@]3(C(=C4c5ccccc5C(=O)c6c4cccc6)[C@@]3([C@@H]7N2c8ccccc8S7)c9ccccc9)c1ccccc1
ZINC02165060	4.46	0.97	-9.18	0	5	0	468.756	5	CCOc1c(cc(cc1Br)/C=C\2/C(=O)N(C(=O)S2)c3ccc(cc3)Cl)OC
ZINC08438804	7.23	2.24	-17.71	0	4	0	541.672	5	CC1=C(C2=C([C@H]3N(c4ccccc4S3)[C@@H]([C@@]2(O1)c5ccccc5)C(=O)c6ccccc6)c7ccccc7)C(=O)C
ZINC02165054	4.45	3.08	-13.22	0	6	0	476.941	6	COc1cc(ccc1OCc2ccccc2C#N)/C=C\3/C(=O)N(C(=O)S3)c4ccc(cc4)Cl
ZINC02165050	3.13	0.44	-49.03	0	7	-1	497.73	6	COc1cc(c(cc1OCC(=O)[O-])Br)/C=C\2/C(=O)N(C(=O)S2)c3ccc(cc3)Cl
ZINC08438803	7.23	2.74	-13.66	0	4	0	541.672	5	CC1=C(C2=C([C@@H]3N(c4ccccc4S3)[C@@H]([C@@]2(O1)c5ccccc5)C(=O)c6ccccc6)c7ccccc7)C(=O)C
ZINC01011827	3.58	-1.11	-8.03	1	5	0	440.702	3	COc1cc(c(cc1O)Br)/C=C\2/C(=O)N(C(=O)S2)c3ccc(cc3)Cl
ZINC08438802	3.44	6.18	-29.81	1	9	1	506.531	9	COC(=O)C1=C(C(=C([C@]2([C@H]1c3cccc4c3c2ccc4)[NH+]5CCCC5)C(=O)OC)C(=O)OC)C(=O)OC
ZINC08438800	3.44	6.42	-36.34	1	9	1	506.531	9	COC(=O)C1=C(C(=C([C@@]2([C@H]1c3cccc4c3c2ccc4)[NH+]5CCCC5)C(=O)OC)C(=O)OC)C(=O)OC
ZINC08438798	3.44	6.73	-38.22	1	9	1	506.531	9	COC(=O)C1=C(C(=C([C@]2([C@@H]1c3cccc4c3c2ccc4)[NH+]5CCCC5)C(=O)OC)C(=O)OC)C(=O)OC
ZINC08438797	3.44	6.18	-30.23	1	9	1	506.531	9	COC(=O)C1=C(C(=C([C@@]2([C@@H]1c3cccc4c3c2ccc4)[NH+]5CCCC5)C(=O)OC)C(=O)OC)C(=O)OC
ZINC00088170	5.19	-0.33	-6.89	0	0	0	309.988	0	c1cc2cccc3c2c(c1)C(=C3Br)Br
ZINC00401042	2.31	3.54	-7.34	1	4	0	178.22	2	c1ccc(cc1)Nc2nnns2
ZINC02184550	1.84	6.81	-9.07	1	3	0	224.329	1	C/C=C\1/C(=O)N(C(=S)N1)C2CCCCC2
ZINC02184550	1.84	6.77	-9.3	1	3	0	224.329	1	C/C=C/1\C(=O)N(C(=S)N1)C2CCCCC2
ZINC04398550	5.12	11.48	-47.99	1	5	-1	455.559	6	c1ccc(cc1)[C@@H]2CC(=C(/C(=N/Cc3ccc4c(c3)OCO4)/C2)C(=S)Nc5ccccc5)[O-]
ZINC08438790	5.12	11.51	-48.57	1	5	-1	455.559	6	c1ccc(cc1)[C@H]2CC(=C(/C(=N/Cc3ccc4c(c3)OCO4)/C2)C(=S)Nc5ccccc5)[O-]
ZINC08438788	5.6	0.32	-46.33	2	3	1	428.681	0	C[C@H]1CC[C@@]2([C@H]3C[C@H]4[C@@H]([C@@H]3CC(=O)[C@H]2C1)CC[C@@H]5[C@@H]4C[NH+]6C[C@H](CC[C@@H]6[C@@]5(C)O)C)C
ZINC08438786	5.6	0.23	-45.19	2	3	1	428.681	0	C[C@H]1CC[C@@]2([C@@H]3C[C@H]4[C@@H]([C@@H]3CC(=O)[C@H]2C1)CC[C@@H]5[C@@H]4C[NH+]6C[C@H](CC[C@@H]6[C@@]5(C)O)C)C
ZINC08438784	5.6	0.32	-44.71	2	3	1	428.681	0	C[C@H]1CC[C@@]2([C@H]3C[C@@H]4[C@@H]([C@@H]3CC(=O)[C@H]2C1)CC[C@@H]5[C@@H]4C[NH+]6C[C@H](CC[C@@H]6[C@@]5(C)O)C)C
ZINC08438783	5.6	0.26	-46.22	2	3	1	428.681	0	C[C@H]1CC[C@@]2([C@@H]3C[C@@H]4[C@@H]([C@@H]3CC(=O)[C@H]2C1)CC[C@@H]5[C@@H]4C[NH+]6C[C@H](CC[C@@H]6[C@@]5(C)O)C)C
ZINC01011792	4.72	-2.43	-13.23	1	5	0	408.523	7	Cc1ccc(cc1)CN(CC(=O)Nc2ccc(cc2)C)S(=O)(=O)c3ccccc3
ZINC08438782	2.88	8.9	-50.43	2	6	1	427.908	6	c1ccc(cc1)C(=O)C2=C(C(=O)N([C@H]2c3ccccc3Cl)CC[NH+]4CCOCC4)O
ZINC08438782	2.3	9.62	-57.72	1	6	1	427.908	6	c1ccc(cc1)C(=O)[C@H]2[C@@H](N(C(=O)C2=O)CC[NH+]3CCOCC3)c4ccccc4Cl
ZINC08438781	2.88	8.76	-46.75	2	6	1	427.908	6	c1ccc(cc1)C(=O)C2=C(C(=O)N([C@@H]2c3ccccc3Cl)CC[NH+]4CCOCC4)O
ZINC08438781	2.3	9.48	-53.52	1	6	1	427.908	6	c1ccc(cc1)C(=O)[C@@H]2[C@H](N(C(=O)C2=O)CC[NH+]3CCOCC3)c4ccccc4Cl
ZINC08438780	6.73	1.31	-10.37	0	5	0	438.593	9	CCCN1C(=O)/C(=C\c2ccc(c(c2)OCC)OCC(C)C)/SC1=Nc3ccccc3
ZINC09087398	3.93	9.73	-57.73	0	5	-1	440.317	6	CN(C)c1ccc(cc1)[C@H]2C(=C(C(=O)N2CC=C)[O-])C(=O)c3ccc(cc3)Br
ZINC09088730	3.93	10.17	-56.58	0	5	-1	440.317	6	CN(C)c1ccc(cc1)[C@@H]2C(=C(C(=O)N2CC=C)[O-])C(=O)c3ccc(cc3)Br
ZINC01011789	3.58	2.65	-17.31	0	7	0	447.896	8	CCOc1cc(ccc1OCC(=O)OC)/C=C\2/C(=O)N(C(=O)S2)c3cccc(c3)Cl
ZINC09331361	4	9.42	-53.87	0	4	-1	415.238	5	C=CCN1[C@H](C(=C(C1=O)[O-])C(=O)c2ccc(cc2)Br)c3ccc(cc3)F
ZINC02164913	4.91	1.85	-9.93	0	5	0	430.957	7	CCN(CC)c1ccc(c(c1)OCC)/C=C\2/C(=O)N(C(=O)S2)c3cccc(c3)Cl
ZINC09374642	4	9.35	-52.4	0	4	-1	415.238	5	C=CCN1[C@@H](C(=C(C1=O)[O-])C(=O)c2ccc(cc2)Br)c3ccc(cc3)F
ZINC09374642	4.44	1.25	-17.24	1	4	0	416.246	4	C=CCN1[C@@H](/C(=C(\c2ccc(cc2)Br)/O)/C(=O)C1=O)c3ccc(cc3)F
ZINC09374642	3.41	0.98	-10.09	0	4	0	416.246	5	C=CCN1[C@@H]([C@@H](C(=O)C1=O)C(=O)c2ccc(cc2)Br)c3ccc(cc3)F
